# hSSB1 (NABP2/OBFC2B) modulates the DNA damage and androgen‐induced transcriptional response in prostate cancer

**DOI:** 10.1002/pros.24496

**Published:** 2023-02-22

**Authors:** Mark N. Adams, Laura V. Croft, Aaron Urquhart, Mohamed Ashick Mohamed Saleem, Anja Rockstroh, Pascal H. G. Duijf, Patrick B. Thomas, Genevieve P. Ferguson, Idris Mohd Najib, Esha T. Shah, Emma Bolderson, Shivashankar Nagaraj, Elizabeth D. Williams, Colleen C. Nelson, Kenneth J. O'Byrne, Derek J. Richard

**Affiliations:** ^1^ School of Biomedical Sciences, Faculty of Health, Translational Research Institute Queensland University of Technology Woolloongabba Queensland Australia; ^2^ LifeBytes India Private Limited Bengaluru India; ^3^ Centre for Data Science Queensland University of Technology Brisbane Queensland Australia; ^4^ Institute of Clinical Medicine University of Oslo Oslo Norway; ^5^ Department of Medical Genetics Oslo University Hospital Oslo Norway; ^6^ Diamantina Institute The University of Queensland Brisbane Queensland Australia; ^7^ Queensland Bladder Cancer Initiative Woolloongabba Queensland Australia; ^8^ Australian Prostate Cancer Research Centre – Queensland Brisbane Queensland Australia; ^9^ Cancer Services Princess Alexandra Hospital Woolloongabba Queensland Australia

**Keywords:** androgen receptor, DNA damage, hSSB1/NABP2, prostate cancer, radiation

## Abstract

**Background:**

Activation and regulation of androgen receptor (AR) signaling and the DNA damage response impact the prostate cancer (PCa) treatment modalities of androgen deprivation therapy (ADT) and radiotherapy. Here, we have evaluated a role for human single‐strand binding protein 1 (hSSB1/NABP2) in modulation of the cellular response to androgens and ionizing radiation (IR). hSSB1 has defined roles in transcription and maintenance of genome stability, yet little is known about this protein in PCa.

**Methods:**

We correlated hSSB1 with measures of genomic instability across available PCa cases from The Cancer Genome Atlas (TCGA). Microarray and subsequent pathway and transcription factor enrichment analysis were performed on LNCaP and DU145 prostate cancer cells.

**Results:**

Our data demonstrate that *hSSB1* expression in PCa correlates with measures of genomic instability including multigene signatures and genomic scars that are reflective of defects in the repair of DNA double‐strand breaks via homologous recombination. In response to IR‐induced DNA damage, we demonstrate that hSSB1 regulates cellular pathways that control cell cycle progression and the associated checkpoints. In keeping with a role for hSSB1 in transcription, our analysis revealed that hSSB1 negatively modulates p53 and RNA polymerase II transcription in PCa. Of relevance to PCa pathology, our findings highlight a transcriptional role for hSSB1 in regulating the androgen response. We identified that AR function is predicted to be impacted by hSSB1 depletion, whereby this protein is required to modulate AR gene activity in PCa.

**Conclusions:**

Our findings point to a key role for hSSB1 in mediating the cellular response to androgen and DNA damage via modulation of transcription. Exploiting hSSB1 in PCa might yield benefits as a strategy to ensure a durable response to ADT and/or radiotherapy and improved patient outcomes.

## 1 INTRODUCTION

Prostate cancer (PCa) is the second most commonly diagnosed cancer worldwide and the sixth leading cause of cancer‐related deaths.^1^, ^2^ Treatment for PCa varies for every individual and takes into account clinico‐pathological factors including age, the Gleason score, PSA levels, and clinical stage of the tumor.^3^ Metastatic PCa remains incurable, despite curative options for localized high‐risk disease (Gleason score > 7, PSA levels > 20 ng/mL) including surgery, radical prostatectomy, or radiotherapy,^4^, ^5^ metastatic PCa remains incurable. Although metastatic disease responds initially to first‐line androgen deprivation therapy (ADT), which blocks the androgen receptor (AR) signaling axis, development of castrate‐resistant prostate cancer (CRPC) is inevitable. Hence, further strategies are warranted to prevent disease recurrence.

While initially thought to be hormone refractory, it is clear that the AR axis is a driver of CRPC despite low/negligible circulating androgen levels.^6^, ^7^, ^8^, ^9^ AR overexpression, aberrant activation of AR transcription, and the expression of AR variants are some of the mechanisms for sustained AR signaling.^8^, ^9^, ^10^ Treatment options for CRPC include chemotherapy (cabazitaxel) and second‐generation anti‐androgen therapy (abiraterone and enzalutamide) and have demonstrated survival benefits.^11^, ^12^, ^13^, ^14^, ^15^ However, these options are not curative with the development of therapy resistance being the primary issue. As current therapies for metastatic CRPC only provide modest survival benefits, there is an urgent need to develop effective new treatment agents for this patient cohort.

An established component of the AR axis in CRPC is the crossover and regulation of DNA damage response (DDR) pathways. These pathways are central to repairing damaged DNA, such as modified bases, single and double‐stranded DNA breaks (DSBs), and hence preventing genomic instability, which is a key cancer hallmark.^16^ As such, CRPC is characterized by an accumulation of genomic scars including mutations, chromosomal translocations, and enrichment of DDR defects arising from aberrations in genes such as *BRCA1*, *BRCA2*, and ATM.^17^, ^18^, ^19^, ^20^ AR signaling is linked with genome instability in CRPC. Accumulating evidence has established this signaling axis as a regulator of key DDR genes, whose expression is necessary for repair of cytotoxic DSBs via pathways such as nonhomologous end joining (NHEJ) or homologous recombination (HR).^20^, ^21^, ^22^, ^23^ Indeed, clinical trials of ADT combined with radiotherapy, which causes DSB formation, markedly improved patient survival and reduced distant metastasis.^24^, ^25^, ^26^, ^27^, ^28^ Hence, blocking or exploiting DSB repair may yield improved outcomes for CRPC.

Single‐strand DNA binding proteins (SSBs) have an essential function in maintaining genomic stability. These proteins are characterized by an oligonucleotide/oligosaccharide binding domain (OB‐fold) which enables binding to ssDNA or ssRNA.^29^, ^30^ We and others have demonstrated that one of these proteins, hSSB1 (also termed nucleic acid binding protein 2 (NABP2)/OBFC2B/SOSS‐S1), functions in HR, replication fork stabilization, repair of oxidized DNA lesions, cell cycle and regulation of transcription.^31^, ^32^, ^33^, ^34^, ^35^, ^36^, ^37^, ^38^ Within these cellular pathways, hSSB1 is indicated to function as part of a heterotrimeric complex with integrator subunit 3 (INTS3)^39^, ^40^, ^41^ and INTS3‐NABP‐interacting protein (INIP). The best characterized function for hSSB1 is the repair of DSBs via HR, where this protein is an early responder to genotoxic stress. Consequently, depletion of hSSB1 yields enhanced sensitivity to ionizing radiation (IR), reduced HR capacity, and defective cell cycle checkpoints.^34^, ^37^, ^38^ Despite these established roles in preventing genome instability, little is known about hSSB1 in malignancies such as PCa.

In this study, we sought to evaluate a role for hSSB1 in PCa. Our data demonstrate that hSSB1 expression correlates with markers of genomic instability in PCa clinical samples. hSSB1 is also required to regulate pathways that modulate cell cycle checkpoints and transcription following induction of DSBs via IR in PCa cells. We also demonstrate that hSSB1 impacts androgen‐dependent transcription, where this protein is a novel modulator of *AR* gene activity.

## 2 MATERIALS AND METHODS

### 2.1 Ethical compliance

All human data were obtained from public resources with non‐identifiable patients providing informed consent according to TCGA Ethical Board regulations (https://www.cancer.gov/about-nci/organization/ccg/research/structural-genomics/tcga/history/policies). All methods were also performed in accordance with relevant guidelines and regulations and approved by Queensland University of Technology (approval number 1900000269).

### 2.2 Cell culture, transfections, and cell treatments

LNCaP and U2OS cell lines, originally sourced from American Type Culture Collection (ATCC), were cultured in RPMI‐1640 media with 10% FBS in an incubator at 37°C with 5% CO_2_. For IR treatment, cells were exposed to 6 Gy of gamma radiation generated from a Cesium‐source irradiator (Gammacell 40 Exactor) and were harvested at 24 h after treatment. For androgen stimulation, LNCaP cells were cultured in RPMI containing 10% charcoal‐stripped serum (CSS; Sigma‐Aldrich) for 48 h. Media were replaced and cells were treated with vehicle (ethanol) or dihydrotestosterone (DHT; 10 nM) and harvested after 48 h of treatment.

Stealth small interfering RNA (siRNA) HS128164 and HS187955 were used to deplete hSSB1 alongside Stealth RNAi™ siRNA negative control medium GC (Thermo Fisher Scientific). Transfection of siRNA was carried out using Lipofectamine RNAiMax (Thermo Fisher Scientific) at a final concentration of 40 nM. Subsequent treatments were performed at 72 h post‐siRNA transfection. Transient expression of FLAG‐tagged, wild‐type (WT), and F98A mutant (F98A3×FLAG) hSSB1 plasmids described in Paquet et al.,^32^ was performed using Fugene HD (Promega) transfection reagent as per manufacturer's instructions.

### 2.3 Real‐time quantitative reverse transcription polymerase chain reaction

RNA was isolated using the RNeasy kit (Qiagen) and 1 μg of RNA was used for cDNA synthesis using the SuperScript™ III First‐Strand Synthesis kit (Thermo Fisher Scientific). Real‐time quantitative reverse transcription polymerase chain reaction (qPCR) was performed using the SYBR™ Green PCR Master Mix (Thermo Fisher Scientific) and the ViiA7 system (ABI). Primers used in the study: hSSB1‐FWD 5′‐AGCCAAACCCAGAGTACAGC‐3′, hSSB1‐REV 5′‐ CTGGTTCTCAGAGGCTGGAG‐3′; HPRT‐FWD 5′‐TGCTGAGGATTTGGAAAGGG‐3′, HPRT‐REV 5′‐ ACAGAGGGCTACAATGTGATG‐3′; BIRC3‐FWD 5′‐AAGCTACCTCTCAGCCTACTTT‐3′, BIRC3‐REV 5′‐CCACTGTTTTCTGTACCCGGA‐3′; CXCR7‐FWD 5′‐TGGTGGACACGGTGATGTG‐3′, CXCR7‐REV 5′‐AAATGCTGCCGAAGAGGTT‐3′; EBP41L2‐FWD 5′‐CTCATTGGTCTGCACTTCCTT‐3′,

EPB41L2‐REV 5′‐TCAGTGAGCAAAGTGGAGATG‐3′; KLK‐FWD 5′‐AGTGCGAGAAGCATTCCCAAC‐3′, KLK‐REV 5′‐CCAGCAAGATCACGCTTTTGTT‐3′; TMPRSS2 FWD 5′‐CCATTTGCAGGATCTGTCTG‐3′, TMPRSS2 REV 5′‐GGATGTGTCTTGGGGAGCAA‐3′; FKBP5 FWD 5′‐AAAAGGCCACCTAGCTTTTTGC‐3′, FKBP REV 5′‐CCCCCTGGTGAACCATAATACA‐3′; AR FWD 5′‐CTGGACACGACAACAACCAG‐3′, AR REV 5′‐CAGATCAGGGGCGAAGTAGA‐3′, DDC FWD 5′‐ATTCATCTGCCCTGAGTTCCG‐3′, 5′‐CCAATAGCCATTTGTGGGGAT‐3′. Transcript levels were normalized to *HPRT* levels and analyzed using the comparative CT method.

### 2.4 Cell lysis, immunoprecipitation, and Western blot analyses

For whole‐cell lysate collection, cells were washed with phosphate‐buffered saline and lysed in lysis buffer (50 mM HEPES (pH 7.5), 150 mM KCl, 5 mM EDTA, 0.05% IGEPAL CA‐630 (v/v), 1× protease inhibitor cocktail (Roche) and 1× phosphatase inhibitor cocktail (Cell Signaling Technology). Following sonication and centrifugation, total protein yield was determined by Bicinchoninic Acid (BCA) Protein assay (Sigma‐Aldrich). Total protein (20 µg) samples were denatured in 1× Laemmli Buffer supplemented with 8% β‐mercaptoethanol for 5 min at 95°C.

For immunoprecipitation, protein samples were prepared at 1 µg/mL protein in lysis buffer. Lysates were incubated overnight with 3 µg of FLAG antibody (Cat#F1804, Sigma‐Aldrich), or INTS3 antibody (Cat# A302‐050 Bethyl) at 4°C. Following incubation, lysates were incubated for 1 h with protein A or G Dynabeads (Thermo Fisher Scientific) pre‐equilibrated with lysis buffer. The Dynabeads were denatured using 2× Laemmli sample buffer supplemented with 8% β‐mercaptoethanol for 5 min at 95°C.

Samples were separated on Bolt 4%–12% Bis‐Tris Plus pre‐cast gels (Thermo Fisher Scientific) and transferred onto nitrocellulose membrane (GE Healthcare Life Sciences) using the semi‐dry transfer Novex system (Thermo Fisher Scientific). Membranes were first blocked using Odyssey blocking buffer (Li‐Cor) and then incubated with primary antibody overnight at 4°C in a 1:1 solution of Odyssey blocking buffer and PBS‐T. RNA Pol II CTD (clone 4H8, cat# 2629) and Androgen Receptor (D6F11, cat# 5153) antibodies were from Cell Signaling Technology and hSSB1 antibody was raised in‐house as described previously.^34^ All primary antibodies were used at a dilution of 1:1000. Following incubation, membranes were washed with PBS‐T and incubated with appropriate secondary antibodies and imaged using the Li‐Cor Odyssey system (Li‐Cor).

### 2.5 Microarray gene expression profiling

Triplicate samples of hSSB1 siRNA knockdown and respective control siRNA transfected LNCaP cells were extracted for RNA and prepared for microarray profiling, which was performed on a custom Agilent 4× 180k oligo array (VPCv3 ID:032034, GEO GPL16604, Agilent Technologies). This microarray contains the Agilent 44k (ID:014850) probe set incorporating human gene expression protein‐coding probes as well as noncoding probes; with the probes targeting exonic regions, 3′UTRs, 5′UTRs, as well as intronic and intergenic regions.^42^ RNA was isolated with Trizol (Life Technologies), further purified using an RNeasy Mini Kit (Qiagen) with DNase treatment according to the manufacturer's protocol. RNA samples were analyzed by a Bioanalyzer (Agilent) to ensure the RNA was of high quality. RNA (100 ng) from each group was amplified and labeled using the Low Input Quick Amp Labeling Kit (Agilent Technologies) and the protocol for One‐Color Microarray‐Based Gene Expression Analysis. The input RNA was reverse‐transcribed into cDNA, using an oligo‐dT/T7‐promoter primer which introduces a T7 promoter region. The subsequent in vitro transcription uses a T7 RNA polymerase, which simultaneously amplifies target material into complementary RNA (cRNA) and incorporates cyanine three‐labeled CTP. cDNA synthesis and in vitro transcription were both performed at 40°C for 2 h. The labeled cRNA was then purified with Qiagen's RNeasy mini‐spin columns and quantified using a Nanodrop‐1000 (Thermo Fisher Scientific). cRNA (1650 ng) from each sample was loaded onto the 4× 180k custom microarray and allowed to hybridize at 65°C for 17 h. The arrays were scanned using an Agilent Microarray Scanner G2565CA.

### 2.6 Dual‐luciferase reporter assays

The dual‐luciferase reporter assay system (Promega) was used as per the manufacturer's recommendation.

### 2.7 Bioinformatics, data analysis, and statistical analysis

Correlations between hSSB1 transcript expression and measures of genomic instability were assessed using the log_2_‐transformed level 3 Illumina HiSeq RNASeq V2 mRNA levels (RSEM) from The Cancer Genome Atlas (TCGA) datasets. Homologous recombination deficiency (HRD) scores were generated from a 230 multi‐gene signature^43^ or unweighted sum of three genomic scars (loss of heterozygosity, telomeric allelic imbalances, and large‐scale transitions) (sum) as previously described.^44^ SNP6 array segmental copy number data was used to determine chromosome arm gains or losses per sample and the ploidy of tumors, as previously described.^45^ Whole‐genome doubling events were calculated using the ABSOLUTE method, as previously described.^46^ Chromosomal instability (CIN) was calculated using the CIN70 gene signature developed by Carter et al.^47^ Replication stress response defects were determined using the signature developed by McGrail et al.^48^ The correlation between relative hSSB1 transcript expression and each parameter was assessed by linear regression analysis with *p* values, *R*‐values and 95% confidence intervals reported according to Spearman's rank correlation, in the *R* statistical environment (*R* Core Team). Microarray data analysis was performed using “limma” (v3.48.0) R package.^49^ Transcription factor enrichment analysis was performed using the ChIP‐X Enrichment Analysis Version 3 (ChEA3) tool.^50^ Pathway analysis was performed using the Reactome tool.^51^ Statistically significant transcripts from the control siRNA‐treated cells versus hSSB1 siRNA‐treated cells at a *q*‐value threshold of ≤0.1 (65 proteins) were used for the Kyoto encyclopedia of genes and genomes (KEGG) database pathway overrepresentation analysis using GSEA (v4.1.0) tool.^52^

For in vitro experiments, data and statistical analyses were performed using GraphPad Prism V8 software. Results are shown as mean ± standard deviation (SD) unless otherwise stated. Data were analyzed using two‐tailed Student's *t* tests or Pearson correlation coefficients. *p* Values below 0.05 were considered significant and indicated using the following abbreviations: *p* < 0.05 (*), *p* < 0.0001 (****).

## 3 RESULTS

### 3.1 hSSB1 expression associated with genomic and chromosomal instability in PCa

**Figure 1:**
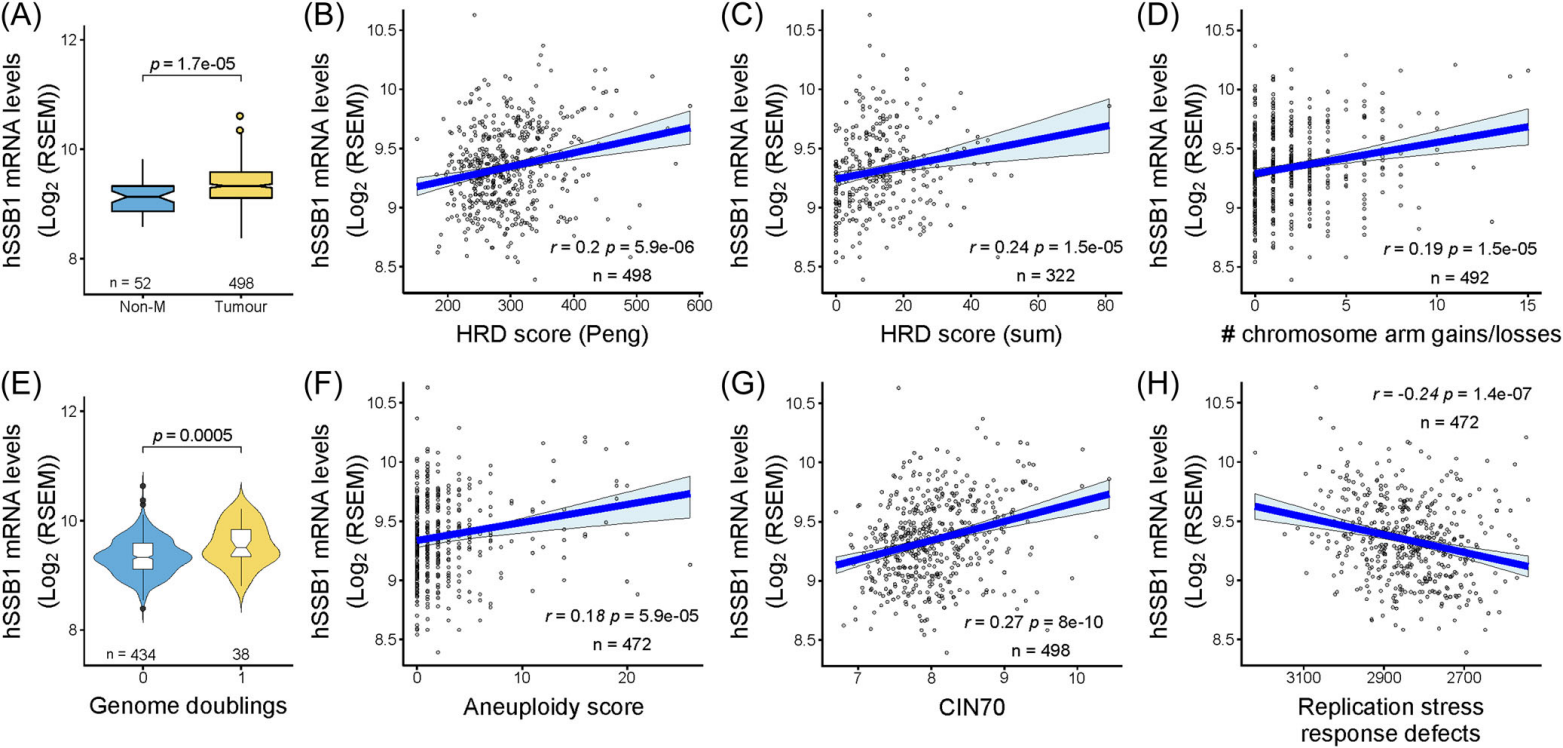
hSSB1 transcript is overexpressed in prostate cancer (PCa) and its expression correlates with genome instability. (A) hSSB1 mRNA levels are elevated in prostate tumors versus nonmalignant (non‐M) prostate tissue. (B–H) Scatter plots representing linear regression analysis of The Cancer Genome Atlas (TCGA) RNAseq data set assessing the correlation between hSSB1 levels and measures of genome instability (B–D, F, G), genome doublings (E) and DNA replication stress (H) in PCa. *R* and *p* values were determined according to Spearman's rank correlation. (B, C) Correlation with gene expression signature reflective of homologous recombination deficiency (HRD) score, (D) number of chromosome arm gains/losses, (E) genome doublings, (F) aneuploidy score, and (G) chromosomal instability multigene signature (CIN70). [Color figure can be viewed at wileyonlinelibrary.com]

Defective DNA repair yielding genomic instability is a key cancer hallmark. hSSB1 has defined roles in cellular pathways that are necessary for the maintenance of genomic stability. Therefore, we investigated whether hSSB1 expression associates with measures of genome instability in PCa. By evaluating available patient data from TCGA datasets, we first identified that *hSSB1* transcripts are markedly elevated in prostate tumors versus nonmalignant prostate tissue (Figure 1A). We next undertook bioinformatics analyses to correlate relative *hSSB1* transcript levels with gene expression signatures or DNA copy number‐based measures of genome instability, such as the homologous recombination deficiency (HRD) score in PCa. As shown in Figure 1, *hSSB1* expression correlated with an HRD score generated from a multigene signature reflective of defective HR^43^ (Figure 1B) and the unweighted sum of three genomic scars, namely loss of heterozygosity, telomeric allelic imbalance, and large‐scale state transitions^44^ (Figure 1C). In addition, *hSSB1* expression correlated with other features of genetic instability, such as an increase in the number of gains or losses of chromosomal arms (Figure 1D). *hSSB1* expression was markedly elevated in tumors exhibiting at least one whole genome duplication in PCa (Figure 1E). Consistently, we also identified that *hSSB1* levels were significantly higher in prostate tumors with features of abnormal chromosome count or aneuploidy (Figure 1F) and a multigene signature, termed CIN70, reflective of chromosomal instability (Figure 1G). Moreover, *hSSB1* expression negatively correlated with the level of genomic defects generated by replicative stress (Figure 1H). In keeping with the roles for hSSB1 in the maintenance of genomic stability by DNA repair and promoting replication fork stability, our data indicate that hSSB1 correlates with markers of genomic instability in PCa.

### 3.2 hSSB1 depletion and IR impact the transcriptional response in LNCAP PCa cells

**Figure 2:**
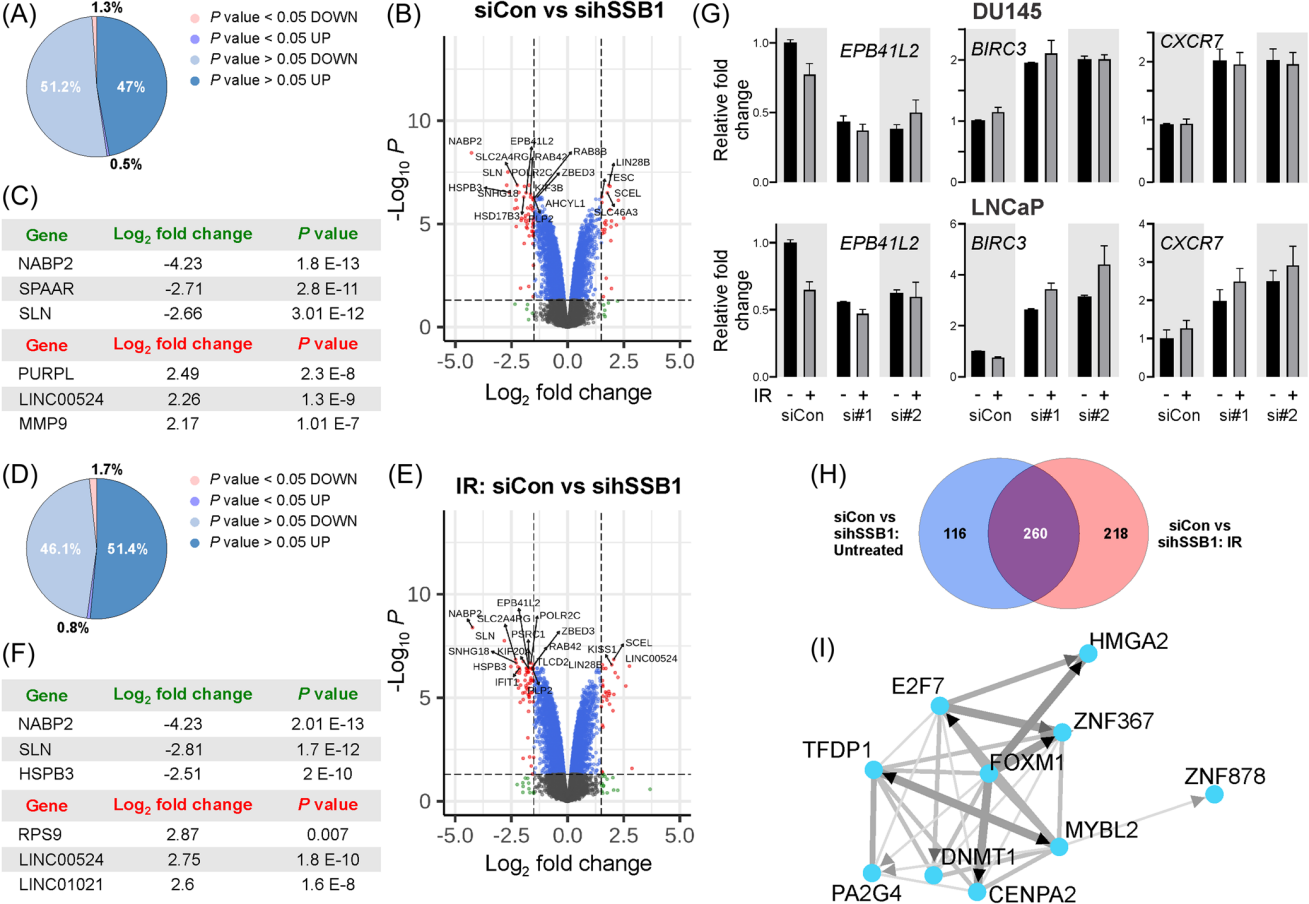
Identification of transcripts deregulated by hSSB1 depletion and by ionizing radiation (IR) in LNCaP prostate cancer (PCa) cells using microarrays. (A) Pie chart showing the proportion of identified transcripts up‐ or downregulated and considered significant (*p* value < 0.05) following siRNA‐mediated hSSB1 depletion. (b) Volcano scatter plot of log_2_ fold transcript changes (siControl‐treated vs. sihSSB1‐treated) ranked by significance (‐log_10_ *p* value). (C) List of top three hSSB1 depletion‐induced downregulated (green) and upregulated transcripts (red) ranked by *p* value. (D) Pie chart showing the proportion of identified transcripts up‐ or downregulated and considered significant (*p* value < 0.05) following IR treatment in sicontrol‐treated versus sihSSB1‐treated cells. (E) Volcano scatter plot of log_2_ fold transcript changes induced by IR treatment in sicontrol versus sihSSB1 cells ranked by significance (‐log_10_ *p* value). (F) List of top three IR treatment‐induced downregulated (green) and upregulated transcripts (red) ranked by *p* value. (G) qPCR validation of transcripts identified as downregulated (*EPB41L2*) or upregulated (*BIRC3* and *CXCR7*) following depletion of hSSB1 in DU145 (upper panel) and LNCaP (lower panel) PCa cell lines. (H) Venn diagram of significantly deregulated transcripts (siControl vs. sihSSB1) identified from the microarray analysis comparing untreated and irradiated LNCaP cells identified 116 transcripts uniquely deregulated transcripts following hSSB1 depletion, 218 uniquely deregulated transcripts following IR and hSSB1 depletion and 260 common deregulated transcripts irrespective of exposure to IR. (I) ChIP‐X enrichment analysis version 3 (ChEA3) tool identified an interlinked, coregulatory network of transcription factors predicted to be impacted by hSSB1 depletion and DNA damage in LNCaP cells. [Color figure can be viewed at wileyonlinelibrary.com]

In addition to maintenance of genome stability, hSSB1 also functions in regulation of transcription via the integrator complex.^35^ Given the correlations with several features of genome instability in the PCa clinical data, we sought to identify genes impacted by hSSB1 depletion and at 24 h following the induction of DNA damage by 6 Gy of IR, to examine the transcriptional impact following the response to DNA damage and cell cycle checkpoint recovery where hSSB1 is demonstrated to function. This level of IR induces complex DNA breakages and this timepoint is commonly employed for PCa in vitro analyses.^53^, ^54^, ^55^ We performed microarray analysis and utilized the well‐characterized LNCaP androgen‐sensitive PCa cell line, at 24 h following IR to examine the transcriptional response once cells had re‐entered the cell cycle following the repair of damaged DNA. Depletion of hSSB1 using siRNA induced the overall downregulation of 10,678 transcripts and upregulation of 9658 transcripts (Figure 2A, Supporting Information: Table 1). Of these, a total of 376 differentially regulated transcripts were considered statistically significant (Figure 2A). hSSB1 depletion yielded the downregulation of 272 of these transcripts and upregulation of 104 transcripts (Figure 2B). Based upon log_2_ fold change and statistical significance, the top three downregulated transcripts were *NABP2* (hSSB1), which confirms effective hSSB1 depletion, in addition to *SPAAR* and *SLN*, whereas the top three upregulated transcripts were *PURPL*, *LINC00524*, and *MMP9* (Figure 2C). Following induction of DNA damage, our microarray analysis indicated that hSSB1‐depleted LNCaP cells exposed to IR yielded the deregulation of 478 statistically significant transcripts (Figure 2D,E). The top three downregulated transcripts following IR treatment were *NABP2* (hSSB1), *SLN*, and *HSPB3*, whereas the top three upregulated transcripts were *RSP9*, *LINC00524*, and *LINC01021* (Figure 2F). Microarray analysis of control LNCaP cells exposed to IR did not identify any significantly deregulated transcripts at the cutoff selected (Supporting Information: Figure 1A). qPCR analysis was performed to validate the microarray analysis on a subset of transcripts that were identified to be significantly deregulated following hSSB1 depletion, including the transcripts *EBP4L2*, *BIRC3*, and *CXCR7* (see Supporting Information: Table 2). Validation of the microarray analysis with qRT‐PCR analysis confirmed the marked reduction in *NABP2*/*hSSB1* transcripts following hSSB1 depletion, irrespective of DNA damage in LNCaP and DU145 cell lines (Supporting Information: Figure 1B). Moreover, consistent with the microarray analysis, *EBP4L2* transcript levels were reduced in DU145 and LNCaP cells exposed to IR and hSSB1 depletion, whereas *BIRC3* and *CXCR7* transcripts were upregulated following hSSB1 depletion and IR in DU145 and LNCaP cell lines (Figure 2G). Venn diagram analysis of only the significantly deregulated transcripts comparing untreated and IR‐exposed microarray datasets indicated that in hSSB1‐depleted LNCaP cells, DNA damage uniquely impacted the differential regulation of 218 transcripts (Figure 2H). Of these transcripts, the top five significantly deregulated transcripts include *KIF20A* (*p* = 2.99E−10), *PLK1* (*p* = 9.77E−10), *PSRC1* (*p* = 1.04E−9), *INK2A* (*p* = 1.41E−9), and *PIF1* (*p* = 1.62E−9). To examine the regulatory networks controlling the expression of these significantly deregulated genes, we utilized the ChIP‐X Enrichment Analysis 3 (ChEA3) transcription factor (TF) enrichment analysis tool.^50^ The top 10 predicted TFs identified by ChEA3 analysis formed an inter‐linked coregulatory network (Figure 2I). Notably, the top‐ranked TF within this network predicted to be impacted by hSSB1 depletion and DNA damage was FOXM1, a TF linked with solid malignancies and the DNA damage response.^56^, ^57^, ^58^, ^59^ Indeed, of the top five deregulated transcripts, all genes, with the exception of INK2A, are regulated by FOXM1.^60^ Overall, these data suggest that hSSB1 participates in modulating the transcriptional response to DNA damage in PCa cells.

### 3.3 hSSB1 modulates cell cycle progression and transcription in PCa cells

**Figure 3:**
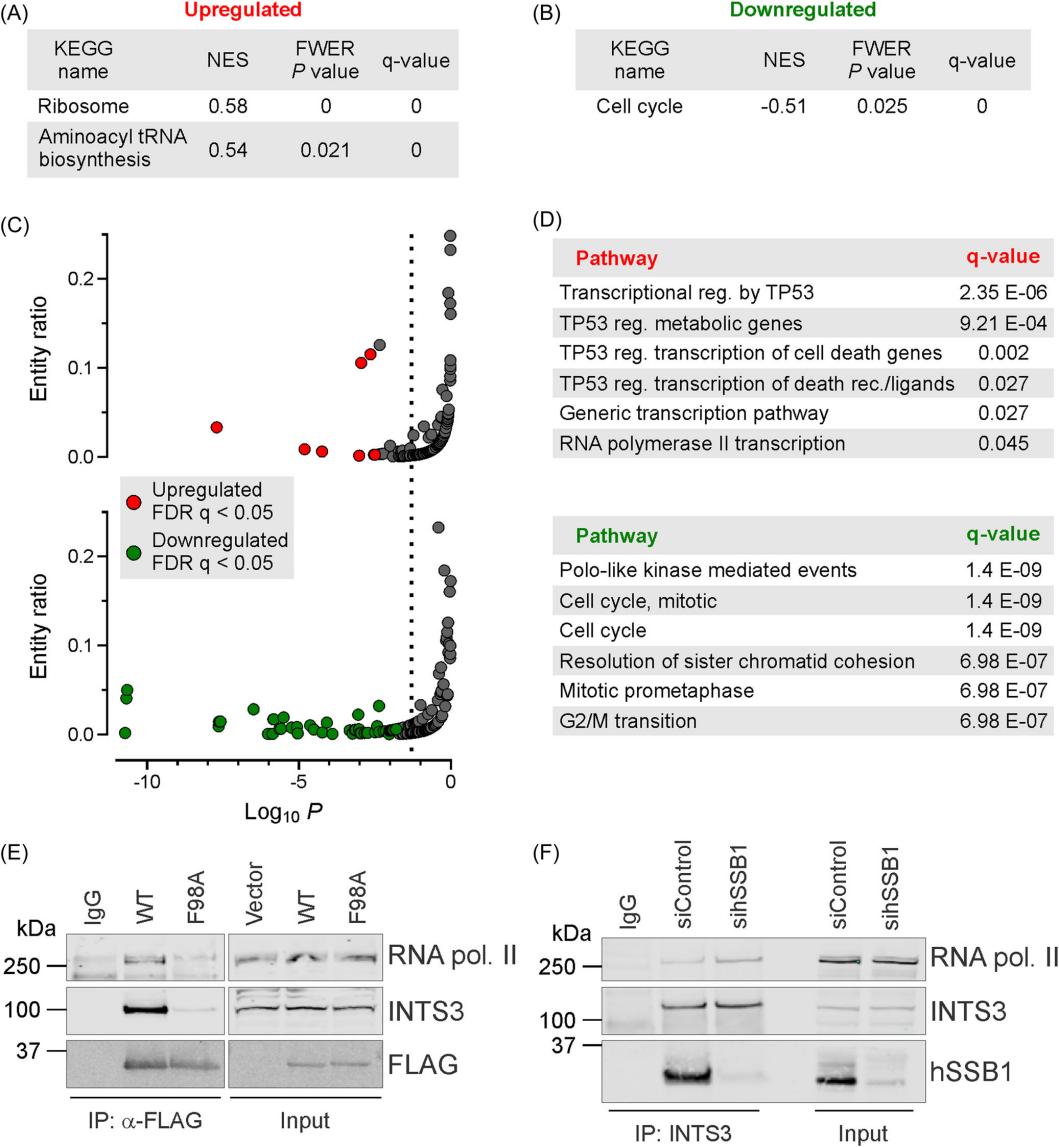
hSSB1 modulates cell cycle progression and transcription in prostate cancer cells. (A–D) Pathway representation analysis of differentially regulated transcripts in hSSB1‐depleted, IR‐treated LNCaP cells. (A, B) KEGG enrichment analysis identified two upregulated gene clusters involved in ribosome biogenesis and aminoacyl tRNA biosynthesis, and a single downregulated gene cluster involved in the cell cycle. (C, D) Reactome analysis identified the top‐ranked upregulated (red) and downregulated (green) pathways (D) (*q* value < 0.05). (E) Co‐immunoprecipitation of FLAG‐tagged wild‐type (WT) hSSB1 or FLAG‐tagged F98A mutated hSSB1 (F98A) identified that INTS3 mediates the interaction between hSSB1 and RNA polymerase II. (F) INTS3 and RNA polymerase II interaction remains unaffected by siRNA‐mediated depletion of hSSB1. [Color figure can be viewed at wileyonlinelibrary.com]

To examine the functional importance of hSSB1 depletion and the induction of DNA damage in PCa cells, the significant differentially regulated transcripts were classified using two approaches; first via overrepresentation analysis to enrich for clusters based on gene ontology, and second via Reactome pathway analysis. In the first approach, gene function was determined using KEGG enrichment analysis to identify and rank gene clusters by FDR (*q* value ≤ 0.05) and FWER significance (*p* value < 0.05). KEGG analysis identified upregulated gene clusters involved in the ribosome function and aminoacyl tRNA biosynthesis (Figure 3A), whereas the only significant downregulated cluster were genes involved in the cell cycle (Figure 3B).

To further evaluate the biological function of the differentially regulated transcripts, we also performed Reactome analysis as a complementary approach to identify top‐ranked pathways (*q* value ≤ 0.05).^51^ As shown in Figure 3C, out of the 121 upregulated pathways, 8 were identified as significant, whereas 56 pathways were considered significant from a total of 170 downregulated pathways. The top‐ranked downregulated pathways include cell cycle regulation, particularly modulation of the G2 and mitotic cell cycle phases and the associated DNA damage checkpoints (Figure 3D), which is consistent with the KEGG enrichment analysis. These findings are in keeping with the defined role for hSSB1 in modulating checkpoint activation following genotoxic stress.^34^, ^37^ The top‐ranked upregulated pathways were involved in modulation of transcription, notably via TP53 and RNA polymerase II (Figure 3D). These analyses are consistent with the KEGG enrichment analysis which point to a ribosomal contribution to transcription regulation or RNA processing. Taken together, these two gene classification approaches suggest that hSSB1 might impact gene transcription/translation and the cell cycle following the induction of IR‐induced DNA damage. Indeed, hSSB1 is reported to regulate the transcriptional activity of p53 by directly interacting with this protein.^37^ Moreover, hSSB1 plays a role in transcription as part of the integrator complex, where hSSB1 was found to co‐immunoprecipitate with RNA Polymerase II and the INTS3 subunit of the integrator complex.^35^

To further delineate the mechanism of hSSB1‐INTS3‐RNA polymerase II interaction, we investigated whether the integrator subunit INTS3 contributed to the association between hSSB1 and RNA polymerase II. Immunoprecipitation analysis was performed between endogenous RNA polymerase II and either hSSB1‐WT‐FLAG or the F98A mutant (hSSB1‐F98A‐FLAG). The F98 residue is essential for hSSB1 binding to INTS3.^61^ As shown in Figure 3E, WT hSSB1 is associated with INTS3 and RNA polymerase II. However, abrogation of hSSB1‐INTS3 binding via the F98A mutation markedly reduced the association between hSSB1 and RNA polymerase II, suggesting that INTS3 mediates the hSSB1‐RNA polymerase II interaction. We further investigated the associations between INTS3 and RNA polymerase II in cells depleted of hSSB1. siRNA‐mediated depletion of hSSB1 did not reduce the association between INTS3 and RNA polymerase II, demonstrating that hSSB1 is dispensable for the INTS3‐RNA polymerase II interaction (Figure 3F). Taken together, these data suggest that hSSB1 associates with RNA polymerase II via the INTS3 subunit of the integrator complex, where hSSB1 might be a necessary complex component required to regulate transcription following the induction of DNA damage in PCa cells.

### 3.4 hSSB1 modulates the androgen response in PCa

**Figure 4:**
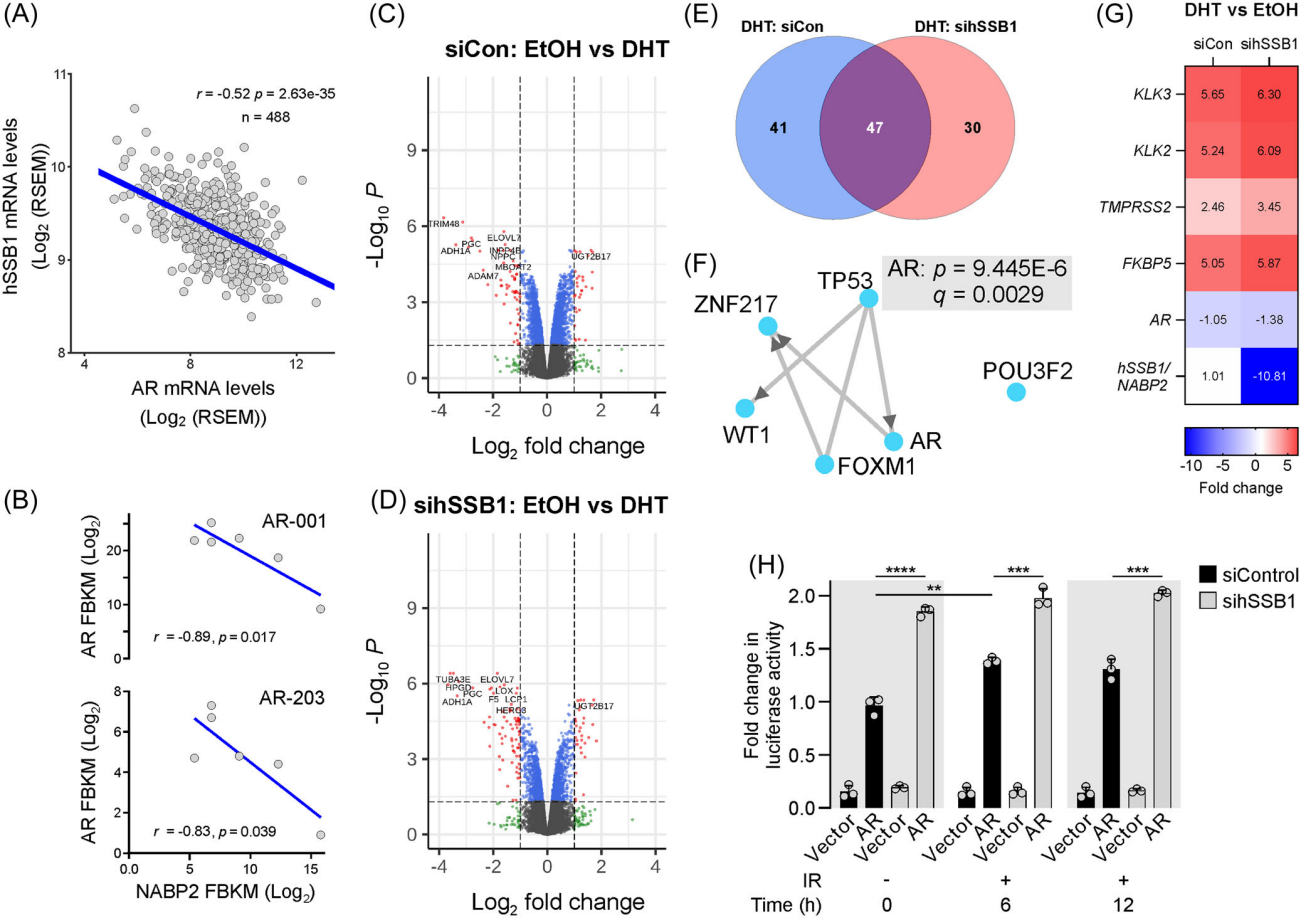
hSSB1 is androgen‐regulated and negatively impacts androgen receptor (AR) promoter activity. (A) Scatter plots representing linear regression analysis of The Cancer Genome Atlas (TCGA) RNAseq data set assessing the correlation between hSSB1 and AR levels. *R* and *p* values were determined according to Spearman's rank correlation. (B) RNAseq analysis of *hSSB1* and *AR* expression across a small panel of prostate cancer (PCa) patient‐derived xenografts (PDXs) revealed a strong correlation between *hSSB1* and *AR* variants 1 (*r* = −0.89) and 203 (*r* = −0.83) in six PCa PDXs. (C) Volcano scatter plot of log_2_ fold transcript changes in siControl LNCaP cells treated with vehicle EtOH or DHT, ranked by significance (‐log_10_ *p* value). (D) Volcano scatter plot of log_2_ fold transcript changes in sihSSB1 LNCaP cells treated with vehicle EtOH or DHT, ranked by significance (‐log_10_ *p* value). (E) Venn diagram analysis identified 41 unique transcripts impacted by DHT treatment, 30 unique transcripts impacted by hSSB1 depletion in DHT‐treated cells, and 47 deregulated transcripts common to both siControl and sihSSB1 DHT‐treated cells. (F) ChIP‐X enrichment analysis version 3 (ChEA3) tool identified AR as the top‐ranked deregulated transcription factor from a coregulatory network of transcription factors impacted by hSSB1 depletion in DHT‐treated LNCaP cells. (G) Heatmap representing fold change in AR‐dependent transcripts, *AR* and *hSSB1* as determined by qPCR analysis of DHT versus vehicle (ethanol (EtOH)) treated LNCaP either depleted of hSSB1 (sihSSB1) or transfected with control siRNA (siCon). (H) Luciferase gene reporter assay identified that IR treatment led to an enhanced AR promoter activity, and that AR promoter activity was significantly enhanced in both unirradiated and IR‐treated, sihSSB1depleted LNCaP cells. [Color figure can be viewed at wileyonlinelibrary.com]

Androgen‐dependent signaling is a key feature of PCa pathology, driven by the transcriptional activity of the AR.^62^, ^63^ Having observed that in PCa cells, hSSB1 modulates the transcriptional response following DNA damage, and that AR signaling controls the expression of DDR genes impacting radiosensitivity,^20^, ^21^, ^23^, ^64^ we sought to determine whether a correlation between hSSB1 and AR existed in prostate tumors. As shown in Figure 4A, expression of *hSSB1* and *AR* showed an inverse correlation in PCa samples (*n* = 488, *R* = −0.5210, *p* = 2.63 × 10^−35^). To confirm these analyses, we evaluated RNAseq analysis for levels of *hSSB1* and *AR* expression across a small panel of PCa, patient‐derived xenografts (PDXs). The six well‐characterized PDXs, bone marrow‐derived BM18^65^ and five LuCaP series PDXs (23.12, 35, 70, 96, and 105)^66^ have defined levels of AR and are each androgen‐dependent. Consistent with prior quantitative PCR analysis, varied expressions of *AR* variant 1 (full length) and variant *AR‐203* (N‐terminal domain truncation) were detected across the PDX panel (Figure 4B). *hSSB1* expression showed a strong negative correlation with both AR variant 1 (*r* = −0.89, *p* = 0.017) and variant 203 (*r* = −0.83, *p* = 0.039), whereby tumors with elevated *hSSB1* expression had lower levels of *AR* transcripts.

Given that hSSB1 modulates transcription pathways following DNA damage and levels are negatively correlated with AR expression, we next sought to determine whether hSSB1 might impact androgen‐dependent transcription. To explore this, hSSB1‐depleted LNCaP cells treated in the absence or presence of DHT were subjected to microarray analysis. After applying significance (*p* < 0.05) and fold change thresholds, our analysis identified that DHT induced the upregulation of 30 transcripts and downregulation of 58 transcripts (Figure 4C, Supporting Information: Table 2). Consistently, transcripts that are linked with or modulate androgen signaling were identified including *ERG1*,^67^ *SAMD14*,^68^ *TRIM48*, *HPDG*, and *PGC*.^69^ Furthermore, DHT impacted the expression of AR‐dependent transcripts with the upregulation of *KLK3*, *TMPRSS2*, and *FKBP5* and the downregulation of *DDC* (Supporting Information: Figure 2A). DHT treatment of hSSB1‐depleted cells induced the upregulation of 31 transcripts and the downregulation of 77 transcripts (Figure 4D). Of the classical AR‐dependent transcripts, *KLK3* and *TMPRSS2* upregulation was slightly increased in hSSB1‐depleted cells, while the downregulation of DDC was slightly reduced (Supporting Information: Figure 2A). Venn diagram analysis indicated that 30 of these transcripts were selectively impacted by hSSB1 depletion in DHT‐treated PCa cells (Figure 4E). The top significantly deregulated transcripts impacted by hSSB1 depletion and DHT treatment included *LOX* (*p* = 3.25E−10), *CADM2* (*p* = 1.82E−8), *CXCR4* (*p* = 1.31E−7), and *STEAP4* (*p* = 6.32E−06), each of which is regulated by AR^70^, ^71^, ^72^ or disrupted in PCa.^73^ We next performed ChEA3 enrichment analysis to identify the predicted transcription factors impacted by hSSB1 depletion. As shown in Figure 4F, a coregulatory network of six transcription factors was identified including p53 and FOXM1. However, the top‐ranked significantly deregulated transcription factor was AR (*q* = 0.0029).

To examine the impact of the androgen response and hSSB1 depletion, qPCR analysis was performed on well‐characterized AR‐dependent genes. These analyses indicated that, following DHT, the upregulation of *KLK3*, *KLK2*, *TMPRSS2*, and *FKBP5* transcripts was greater in hSSB1‐depleted cells versus control cells (Figure 4G), consistent with the microarray analysis. DHT treatment of hSSB1‐depleted cells resulted in a modest reduction in *AR* transcript. We next sought to determine whether the expression of hSSB1 might also be androgen‐regulated. qRT‐PCR analysis was performed on LNCaP cells treated with vehicle (ethanol), the androgen dihydrotestosterone (DHT), the potent AR antagonist enzalutamide or a combination of DHT and enzalutamide. While AR stimulation induced a robust increase in transcriptional expression (~3‐fold upregulation) of KLK3, hSSB1 expression was unaffected (Supporting Information: Figure 2B). Blockade of AR signaling with enzalutamide reduced the DHT‐mediated upregulation of *KLK3* transcript levels without impacting the expression of *hSSB1* transcripts. To further investigate whether AR signaling might impact hSSB1 expression, we analyzed previously performed RNAseq analysis of LNCaP cells depleted of AR and treated with DHT. Depletion of AR prevented the DHT‐induced upregulation of the AR‐dependent genes *KLK3*, *KLK2*, *TMPRSS2*, and *FKBP5* and the downregulation of *DDC* (Supporting Information: Figure 2C). However, *hSSB1* transcripts were minimally impacted by either AR depletion or DHT treatment, suggesting that unlike AR‐dependent genes, hSSB1 is not regulated by AR signaling.

Taken together, these data suggest that like the DDR, hSSB1 might also uniquely impact the androgen transcriptional response.

Both p53 and FOXM1 are identified as transcription factors that modulate AR gene activity in PCa.^74^, ^75^ Having observed that hSSB1 is predicted to impact these transcription factors in addition to binding RNA polymerase II, we next explored the possibility that hSSB1 might affect PCa cell transcriptional response by impacting AR gene transcription activity. Luciferase gene reporter assays utilizing the AR promoter were performed in irradiated control versus irradiated hSSB1‐depleted LNCaP cells. We focused on the impact of IR‐induced DNA damage as androgens are reported not to affect AR gene transcription activity in LNCaP cells.^76^ As shown in Figure 4H, AR promoter activity was readily detected in control LNCaP cells, which was elevated ~1.4‐fold at 6 and 12 h following exposure to IR. Depletion of hSSB1 markedly enhanced AR promoter activity ~1.9‐fold at all time points tested in both unirradiated and IR‐treated LNCaP cells, pointing to the possibility that this protein is a modulator of AR gene activity. Western blot analysis of LNCaP cells revealed that hSSB1 depletion induced a ~2‐fold increase in endogenous AR protein in both unirradiated and IR‐treated cells (Supporting Information: Figure 2D). Collectively, our findings highlight hSSB1 as a transcriptional regulator in PCa in response to IR‐induced DNA damage and androgen treatment.

## 4 DISCUSSION

hSSB1 has been primarily reported as a DNA damage repair protein, functioning in the repair of DSBs via HR, the recovery and repair of stalled and collapsed DNA replication forks and the removal of oxidative DNA bases by the base excision repair pathway. Despite these roles in maintaining genomic stability, little is known about hSSB1 in malignant disease, especially given that the loss of genomic stability is a key cancer hallmark. In this study, we report a role for hSSB1 in modulating transcription in PCa. We document that the expression of *hSSB1* correlates with measures of genomic instability including multigene signatures or genomic scars that are reflective of HR deficiencies (Figure 1). Moreover, in response to IR‐induced DNA damage, we demonstrate that hSSB1 is required to regulate cellular pathways controlling cell cycle progression and the associated checkpoints (Figures 2 and 3). In keeping with the role for hSSB1 in transcription, our analysis revealed that hSSB1 is required to negatively modulate p53 and RNA polymerase II transcription in PCa (Figure 3). Of relevance to PCa pathology, our findings highlight a transcriptional role for hSSB1 in regulating the androgen response (Figure 4). By using enrichment bioinformatics analysis, we identified that AR function is predicted to be impacted by hSSB1 depletion. Notably, we report that hSSB1 might also be required to regulate AR gene activity in PCa.

Our finding that hSSB1 regulates transcriptional pathways in PCa cells is in keeping with studies in other cell types.^35^ In these cell types, INTS3, in concert with hSSB1, functions to modulate transcription termination of snRNA genes and histone mRNAs associated with replication. To regulate these specific gene targets, the integrator complex containing the INTS3 and hSSB1 complex was reported to bind to RNA polymerase II. Consistently, we identified that in U2OS cancer cells, hSSB1 protein associates with RNA polymerase II via INTS3 (Figure 3). Interestingly, we identified that in the absence of depleted hSSB1 levels, INTS3 was still capable of associating with RNA polymerase II (Figure 3). These findings suggest that hSSB1 is an integral component of this protein complex required to modulate RNA polymerase II for efficient transcription. How hSSB1 functions within this complex, and whether this requires the ssDNA binding function of hSSB1, remains to be determined. Nonetheless, for this study, we focussed more specifically on exploring the impact of hSSB1 in PCa and not the other protein complex partners as depletion of INTS3 also results in loss of hSSB1.^61^ As such, while we were unable to identify defined PCa mechanistic roles in transcription, our study does suggest that hSSB1 participates in the DNA damage and androgen response by modulation of transcription.

Our findings have identified subsets of unique genes or transcripts that are impacted by hSSB1 in PCa. By examining these unique transcripts, our study has identified transcription factor networks predicted to be regulated by hSSB1. While we have previously identified proteins with roles in RNA metabolism and transcription regulation,^77^ none of the transcription factors identified in this study are known to associate with hSSB1. Thus, how hSSB1 mechanistically regulates these transcription factors requires further study. Nevertheless, our analysis identified one of the top‐ranked transcription factors impacted by hSSB1 depletion and IR as FOXM1 (Figure 2). Indeed, FOXM1 is linked with the DDR and activity of this transcription factor is associated with the chemotherapy response in solid malignancies.^56^, ^57^, ^58^, ^59^ In PCa, expression of FOXM1 has prognostic potential^78^ and deregulation of FOXM1 transcription factor activity contributes to driving PCa growth and metastasis.^79^, ^80^ Our finding that hSSB1 is a possible modulator of FOXM1 might be of therapeutic interest to impact PCa therapy response and disease progression.

Another key transcription factor identified by our study to be impacted by hSSB1 is AR (Figure 4). Indeed, like FOXM1,^75^ our findings point to the possibility that hSSB1 also participates in regulating the AR promoter. Control of AR gene transcription is tightly regulated, especially during development and in specific tissues such as prostate.^81^ The AR promoter is characterized as lacking classical CAAT and TATA sequence motifs but is located within a GC‐rich region.^82^ Sp1 is the predominant transcription factor driving the expression of the AR gene.^83^ Importantly, our AR reporter assay included each of these promoter features. How hSSB1 might then regulate the AR promoter remains an open question. While modulation of FOXM1 activity is one possible explanation, it is worth noting that the structurally related archaeal SSB from *Sulfolobus solfataricus*, functions to directly promote RNA polymerase II‐dependent transcription from promoters.^84^ Accordingly, it remains possible that the human SSB1 might modulate transcription initiation from a subset of human genes.

Our study identified that hSSB1 also modulates the transcriptional activity of factors involved in DDR such as p53. Indeed, p53 is reported to negatively regulate the AR promoter binding to a sequence −488 to −469 bp upstream of the AR transcription start site.^74^ In our study, hSSB1 depletion upregulated the transcriptional activity of p53 (Figure 3C,D) and was predicted to impact p53 in response to androgens (Figure 4F). Whether hSSB1 regulates gene promoters, such as AR, that contain repressive p53 binding motifs requires further investigation.

A more recently identified feature of the AR promoter that modulates its expression is G‐quadruplexes (GQ).^85^ These four‐stranded structures form intra‐ or intermolecular folding of single DNA strands. The AR promoter contains a guanine‐rich sequence that forms a GQ leading to reduced promoter activity.^85^ These structures can be resolved by the RecQ helicases such as Werner (WRN) which bind and unwind GQ.^86^ We have previously identified that hSSB1 associates with Bloom syndrome (BLM) helicase which like WRN, is a RecQ family member.^87^ BLM is also required for resolving genomic structures such as GQ, particularly at transcriptionally active sites.^88^ As such, a potential regulation of BLM activity by hSSB1 might result in modulation of gene promoters that themselves, are regulated by GQ structures.

## 5 CONCLUSIONS

We have identified that hSSB1 participates in the cellular response to DNA damage and androgens. Our exploratory findings in PCa point to a variety of mechanisms suggesting how hSSB1 might modulate these cellular responses via transcription and the activity of a subset of gene promoters. Indeed, given the clinical observations that combining ADT and radiotherapy markedly improves patient survival,^24^, ^25^, ^26^, ^27^, ^28^ strategies to target the function of hSSB1 in response to these therapies might have further benefits. As such, future work to either target hSSB1 directly or block its binding to INTS3 might yield viable therapeutic approaches.

## AUTHOR CONTRIBUTIONS

Mark N. Adams, Kenneth J. O'Byrne, and Derek J. Richard conceived and designed the study. Mark N. Adams, Laura V. Crof, Aaron Urquhart, Anja Rockstroh, Patrick B. Thomas, Idris Mohd Najib, Esha T. Shah, and Emma Bolderson performed the experiments. Mohamed Ashick Mohamed Saleem, Pascal H. G. Duijf, and Shivashankar Nagaraj performed the bioinformatics. Derek J. Richard, Kenneth J. O'Byrne, and Mark N. Adams wrote the manuscript. All authors contributed to drafting the manuscript.

## CONFLICT OF INTEREST STATEMENT

K.J.O. and D.J.R. are founders of Repluca. All other authors declare no conflict of interest.

## Supporting information

Supplemental Table 1. Microarray data for siControl LNCaP cells and hSSB1 depleted LNCaP cells treated with or without ionising radiation (IR).

Supplemental Table 2. Microarray data for siControl LNCaP cells and hSSB1 depleted LNCaP cells treated with or without dihydrotestosterone (DHT).

**Supplemental Fig. 1. a** Volcano scatter plot of log_2_ fold transcript changes (siControl‐treated vs siControl‐irradiated) ranked by significance (‐log_10_ *P* value). No significantly deregulated transcripts were identified. **b** qPCR analysis of *NABP2/hSSB1* transcripts to validate depletion of hSSB1 in LNCaP (left panel) and DU145 (right panel) prostate cancer cell lines.

**Supplemental Fig. 2. a** List of AR‐dependent transcripts from microarray analysis (see Figure 4) with DHT‐induced fold transcript changes and significance (adjusted *P* value) in siControl and sihSSB1 LNCaP cells. **b** Androgen stimulation (DHT) or AR antagonist enzalutamide (ENZ), normalised to vehicle (ethanol (EtOH)), does not impact *hSSB1* or *AR* transcript levels as determined by qPCR analysis of treated LNCaP cells. *KLK3* transcripts were evaluated as a known AR‐regulated gene (unpaired Student's *t* test, ****P = <0.0001). **c** Heatmap representing fold change in *hSSB1*, *AR* and AR‐dependent transcripts as determined by RNAseq analysis of AR depleted (shAR) versus non‐targeting control (shNC) LNCaP cells treated with vehicle (ethanol (EtOH)) or DHT. **d** Western blot analysis of control versus hSSB1 depleted LNCaP cells exposed to ionising radiation (6 Gy) assessing endogenous AR and hSSB1. Tubulin used as loading control. Densitometry quantification of AR levels relative to untreated control lysates indicated below AR blots.

## Data Availability

The data that supports the findings of this study are available in the Supporting Information of this article.
