# Supplementary figures and images for: hSSB1 (NABP2/OBFC2B) modulates the DNA damage and androgen‐induced transcriptional response in prostate cancer
Source: Prostate. 2023 Feb 22;83(7):628–40. doi: 10.1002/pros.24496 (PMC10953336; doi:10.1002/pros.24496)

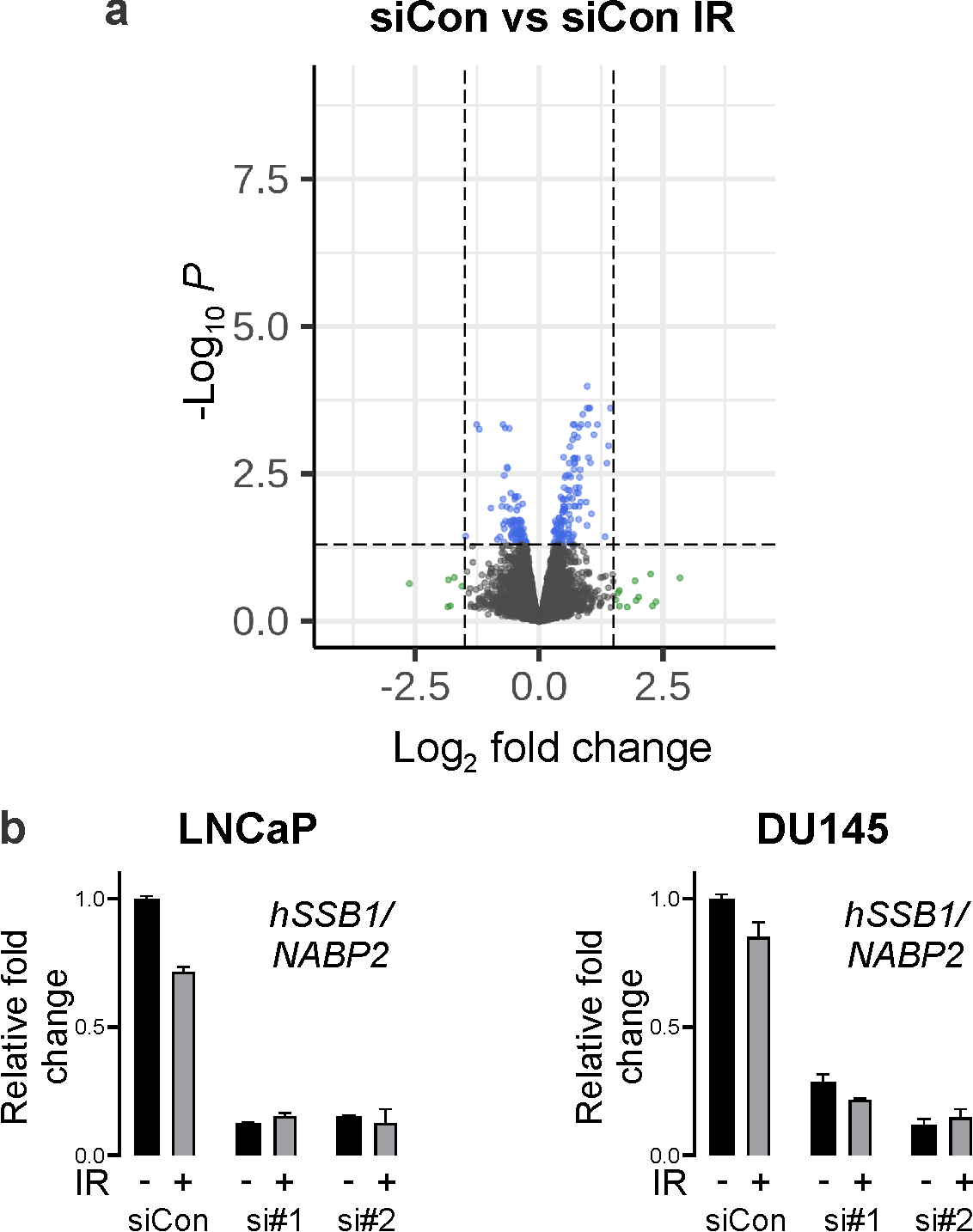

Supplement: Supplementary file 3 — Supplemental Fig. 1. a Volcano scatter plot of log2 fold transcript changes (siControl‐treated vs siControl‐irradiated) ranked by significance (‐log10 P value). No significantly deregulated transcripts were identified. b qPCR analysis of NABP2/hSSB1 transcripts to validate depletion of hSSB1 in LNCaP (left panel) and DU145 (right panel) prostate cancer cell lines. [file PROS-83-628-s001.tif]

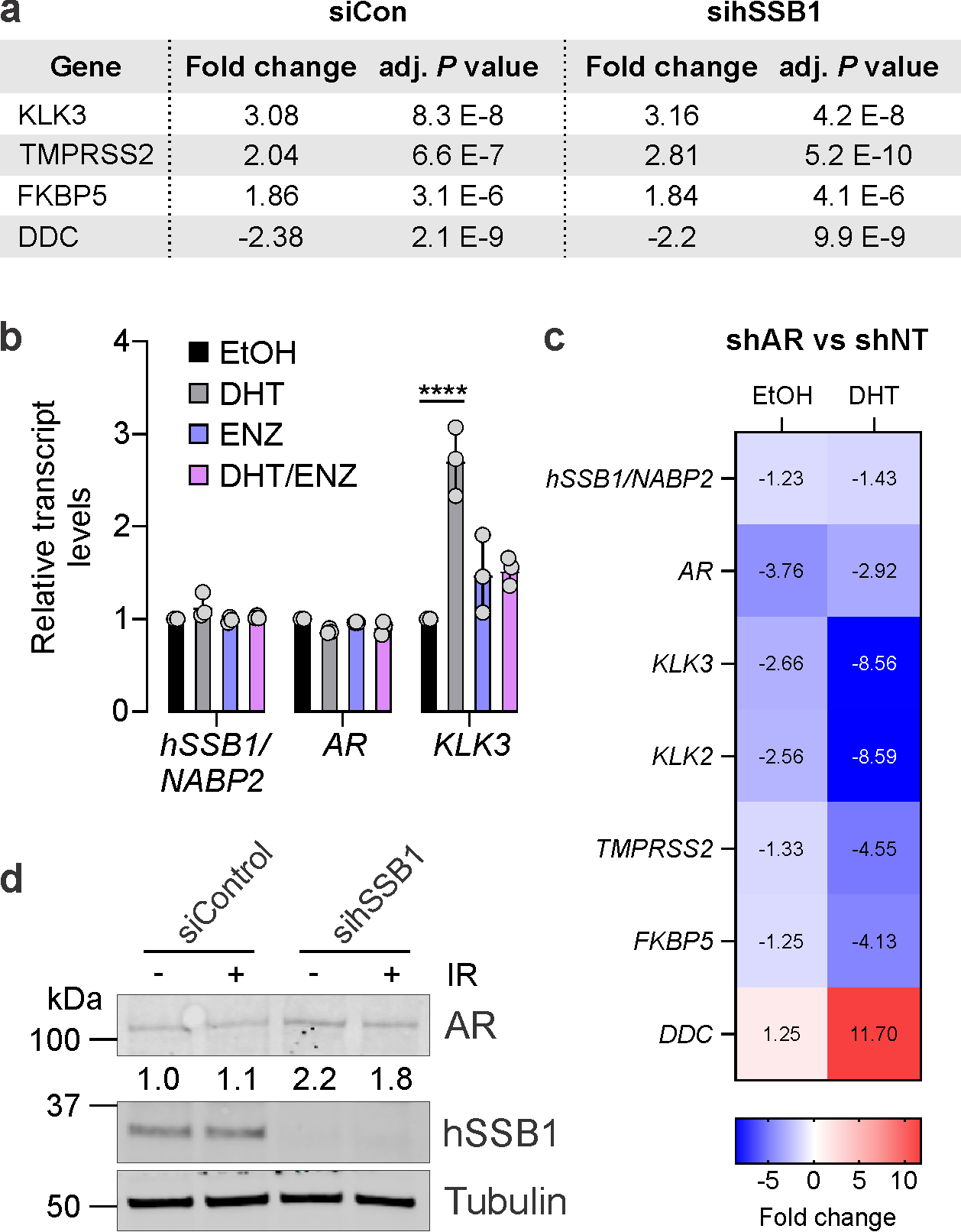

Supplement: Supplementary file 4 — Supplemental Fig. 2. a List of AR‐dependent transcripts from microarray analysis (see Figure 4) with DHT‐induced fold transcript changes and significance (adjusted P value) in siControl and sihSSB1 LNCaP cells. b Androgen stimulation (DHT) or AR antagonist enzalutamide (ENZ), normalised to vehicle (ethanol (EtOH)), does not impact hSSB1 or AR transcript levels as determined by qPCR analysis of treated LNCaP cells. KLK3 transcripts were evaluated as a known AR‐regulated gene (unpaired Student's t test, ****P = <0.0001). c Heatmap representing fold change in hSSB1, AR and AR‐dependent transcripts as determined by RNAseq analysis of AR depleted (shAR) versus non‐targeting control (shNC) LNCaP cells treated with vehicle (ethanol (EtOH)) or DHT. d Western blot analysis of control versus hSSB1 depleted LNCaP cells exposed to ionising radiation (6 Gy) assessing endogenous AR and hSSB1. Tubulin used as loading control. Densitometry quantification of AR levels relative to untreated control lysates indicated below AR blots. [file PROS-83-628-s004.tif]
