# Supplementary material for: hSSB1 (NABP2/OBFC2B) modulates the DNA damage and androgen‐induced transcriptional response in prostate cancer
Source: Prostate. 2023 Feb 22;83(7):628–40. doi: 10.1002/pros.24496 (PMC10953336; doi:10.1002/pros.24496)
Supplement: Supplementary file 2 — Supplemental Table 2. Microarray data for siControl LNCaP cells and hSSB1 depleted LNCaP cells treated with or without dihydrotestosterone (DHT). [file PROS-83-628-s003.docx]

coefficients.control.E.control.DHT	coefficients.hssb1.DHT.control.DHT	coefficients.hssb1.E.control.DHT	coefficients.hssb1.DHT.control.E	coefficients.hssb1.E.control.E	coefficients.hssb1.E.hssb1.DHT	df.residual	sigma	stdev.unscaled.control.E.control.DHT	stdev.unscaled.hssb1.DHT.control.DHT	stdev.unscaled.hssb1.E.control.DHT	stdev.unscaled.hssb1.DHT.control.E	stdev.unscaled.hssb1.E.control.E	stdev.unscaled.hssb1.E.hssb1.DHT	genes.Row	genes.Col	genes.ControlType	genes.ProbeName	genes.SystematicName	genes.probeID	genes.chr	genes.start	genes.end	genes.strand	genes.symbol	genes.geneDescription	genes.EnsemblGeneID	genes.geneBiotype	genes.blockType	genes.probeClassification	genes.refProbe	genes.FeatureNum	genes.query	genes.ensembl_gene_id	genes.hgnc_symbol	Amean	s2.post	t.control.E.control.DHT	t.hssb1.DHT.control.DHT	t.hssb1.E.control.DHT	t.hssb1.DHT.control.E	t.hssb1.E.control.E	t.hssb1.E.hssb1.DHT	df.total	p.value.control.E.control.DHT	p.value.hssb1.DHT.control.DHT	p.value.hssb1.E.control.DHT	p.value.hssb1.DHT.control.E	p.value.hssb1.E.control.E	p.value.hssb1.E.hssb1.DHT	lods.control.E.control.DHT	lods.hssb1.DHT.control.DHT	lods.hssb1.E.control.DHT	lods.hssb1.DHT.control.E	lods.hssb1.E.control.E	lods.hssb1.E.hssb1.DHT	F	F.p.value

ZNF384	-0.082493741	-0.047069696	-0.223528213	0.035424045	-0.141034472	-0.176458517	8	0.106750808	0.816496581	0.816496581	0.816496581	0.816496581	0.816496581	0.816496581	1	23	0	A_23_P204355	A_23_P204355	A_23_P204355	chr12	6667161	6667221	-	ZNF384	zinc finger protein 384 [Source:HGNC Symbol;Acc:HGNC:11955]	ENSG00000126746	protein_coding	3'UTR	3'UTR	1	23	12:6667161:6667221	ENSG00000126746	ZNF384	8.494248754	0.011358081	-0.948012698	-0.540921878	-2.56877166	0.40709082	-1.620758962	-2.027849782	9.961963646	0.365552336	0.600446827	0.028027648	0.692550527	0.136253162	0.07016333	-6.528977721	-6.030153577	-4.288276531	-7.059734809	-5.061815806	-5.193306927	2.449111614	0.124086162

GALNS	-0.061551766	-0.015611446	-0.09948705	0.04594032	-0.037935285	-0.083875605	8	0.106495494	0.816496581	0.816496581	0.816496581	0.816496581	0.816496581	0.816496581	1	24	0	P_119818	P_119818	P_119818	chr16	88844314	88844374	-	GALNS	galactosamine (N-acetyl)-6-sulfatase [Source:HGNC Symbol;Acc:HGNC:4122]	ENSG00000141012	protein_coding	exon	non-coding exon	1	24	16:88844314:88844374	ENSG00000141012	GALNS	7.665067848	0.011314359	-0.708714272	-0.179752022	-1.1455056	0.52896225	-0.436791328	-0.965753578	9.961963646	0.494752015	0.860950896	0.278772675	0.608415054	0.67157827	0.357026657	-6.731012768	-6.165082808	-6.374266867	-6.998721406	-6.196199774	-6.584471338	0.541672915	0.664631095

DRG1	-0.049226797	-0.048278193	-0.112279524	0.000948604	-0.063052727	-0.064001331	8	0.125658656	0.816496581	0.816496581	0.816496581	0.816496581	0.816496581	0.816496581	1	25	0	P_133104	P_133104	P_133104	chr22	31411021	31411081	+	DRG1	developmentally regulated GTP binding protein 1 [Source:HGNC Symbol;Acc:HGNC:3029]	ENSG00000185721	protein_coding	"3'UTR,exon;exon"	multiple Ref	1	25	22:31411021:31411081	ENSG00000185721	DRG1	9.232332738	0.014886993	-0.494132833	-0.484610858	-1.127048733	0.009521975	-0.6329159	-0.642437875	9.961963646	0.631925974	0.638430842	0.286136939	0.992590667	0.541043947	0.535093446	-6.866739934	-6.059779231	-6.394525291	-7.14956348	-6.087931752	-6.850482346	0.427108903	0.737971734

IL22RA2	0.198461534	0.010590027	-0.019746174	-0.187871508	-0.218207708	-0.030336201	8	0.186137766	0.816496581	0.816496581	0.816496581	0.816496581	0.816496581	0.816496581	1	27	0	A_23_P168288	A_23_P168288	A_23_P168288	chr6	137144090	137144150	-	IL22RA2	"interleukin 22 receptor, alpha 2 [Source:HGNC Symbol;Acc:HGNC:14901]"	ENSG00000164485	protein_coding	3'UTR	3'UTR	1	27	6:137144090:137144150	ENSG00000164485	IL22RA2	3.802492025	0.030030329	1.402626134	0.074844973	-0.139556009	-1.327781161	-1.542182143	-0.214400981	9.961963646	0.191115549	0.941819687	0.8917919	0.213870556	0.154172899	0.834559368	-6.019391885	-6.179135195	-7.036495086	-6.262365373	-5.166594545	-7.046324335	1.029955679	0.42055421

ZNF302	-0.018387078	-0.131790336	-0.163158558	-0.113403258	-0.14477148	-0.031368222	8	0.095558865	0.816496581	0.816496581	0.816496581	0.816496581	0.816496581	0.816496581	1	28	0	P_125417	P_125417	P_125417	chr19	34681084	34681144	+	ZNF302;CTD-2553L13.9	zinc finger protein 302 [Source:HGNC Symbol;Acc:HGNC:13848];	ENSG00000089335	protein_coding;TEC	intron;promoter	multiple non-Ref	0	28	19:34681084:34681144	ENSG00000089335	ZNF302	8.018639267	0.009539774	-0.230562843	-1.652571117	-2.045909646	-1.422008274	-1.815346803	-0.393338529	9.961963646	0.822320314	0.129541729	0.068078751	0.18556891	0.0996447	0.702355889	-6.969429497	-4.916701574	-5.14156381	-6.143422924	-4.790293266	-6.98751128	2.073700591	0.167693834

TNNI3	-0.178165225	0.078462129	0.050160496	0.256627354	0.228325721	-0.028301633	8	0.134654191	0.816496581	0.816496581	0.816496581	0.816496581	0.816496581	0.816496581	1	30	0	A_23_P67453	A_23_P67453	A_23_P67453	chr19	55154142	55154202	-	TNNI3	troponin I type 3 (cardiac) [Source:HGNC Symbol;Acc:HGNC:11947]	ENSG00000129991	protein_coding	exon	coding exon	1	30	19:55154142:55154202	ENSG00000129991	TNNI3	7.092436154	0.016767469	-1.685135455	0.742116291	0.474431698	2.427251746	2.159567153	-0.267684594	9.961963646	0.122980054	0.47515031	0.645420425	0.035703702	0.056251897	0.794402645	-5.636492861	-5.899819416	-6.925577394	-4.622606398	-4.276665577	-7.032374229	2.373739983	0.131656789

IP6K1	-0.133001029	0.045015388	-0.064562239	0.178016417	0.06843879	-0.109577627	8	0.090028551	0.816496581	0.816496581	0.816496581	0.816496581	0.816496581	0.816496581	1	32	0	A_24_P396105	A_24_P396105	A_24_P396105	chr3	49727109	49727169	-	IP6K1	inositol hexakisphosphate kinase 1 [Source:HGNC Symbol;Acc:HGNC:18360]	ENSG00000176095	protein_coding	"3'UTR;3'UTR,exon"	multiple Ref	1	32	3:49727109:49727169	ENSG00000176095	IP6K1	7.677735979	0.008715553	-1.744830115	0.590553363	-0.846986977	2.335383478	0.897843138	-1.43754034	9.961963646	0.111725896	0.56797441	0.416885718	0.041757984	0.390452578	0.181223828	-5.55044211	-6.001511046	-6.668911722	-4.774550973	-5.884931746	-6.046222095	2.072872144	0.167808232

UFSP2	0.055402748	0.203289324	0.164938262	0.147886576	0.109535514	-0.038351062	8	0.054111345	0.816496581	0.816496581	0.816496581	0.816496581	0.816496581	0.816496581	1	33	0	P_139046	P_139046	P_139046	chr4	185400018	185400078	-	UFSP2	UFM1-specific peptidase 2 [Source:HGNC Symbol;Acc:HGNC:25640]	ENSG00000109775	protein_coding	3'UTR	3'UTR	1	33	4:185400018:185400078	ENSG00000109775	UFSP2	9.110743809	0.004558058	1.005048493	3.687824817	2.992107036	2.682776324	1.987058543	-0.695717781	9.961963646	0.338661122	0.004218847	0.013579457	0.023054417	0.075096009	0.502512378	-6.473548242	-1.768168636	-3.569326977	-4.193130515	-4.538604599	-6.813221924	5.865432094	0.014209865

CFAP97D1	0.099039388	0.2698833	0.445808758	0.170843911	0.34676937	0.175925458	8	0.195681649	0.816496581	0.816496581	0.816496581	0.816496581	0.816496581	0.816496581	1	34	0	P_121673	P_121673	P_121673	chr17	43783180	43783240	+	C17orf105	chromosome 17 open reading frame 105 [Source:HGNC Symbol;Acc:HGNC:37241]	ENSG00000231256	protein_coding	exon	coding exon	1	34	17:43783180:43783240	ENSG00000231256	CFAP97D1	4.411854337	0.032956692	0.668161998	1.820747965	3.007616218	1.152585967	2.33945422	1.186868253	9.961963646	0.519210292	0.098770694	0.013224953	0.275987452	0.041469629	0.262811287	-6.760085602	-4.68337542	-3.542806552	-6.467917147	-3.996035788	-6.351944743	3.502912605	0.057577767

FBXO16	0.068159403	0.078136333	0.209081796	0.00997693	0.140922393	0.130945463	8	0.040900546	0.816496581	0.816496581	0.816496581	0.816496581	0.816496581	0.816496581	1	35	0	A_23_P168847	A_23_P168847	A_23_P168847	chr8	28428461	28428521	-	FBXO16	F-box protein 16 [Source:HGNC Symbol;Acc:HGNC:13618]	ENSG00000214050	protein_coding	3'UTR;exon	multiple Ref	1	35	8:28428461:28428521	ENSG00000214050	FBXO16	8.165566663	0.003550077	1.401046318	1.606126467	4.297767686	0.205080149	2.896721368	2.691641219	9.961963646	0.191573804	0.139443038	0.001580689	0.841639218	0.015982326	0.022706812	-6.021407425	-4.979003304	-1.373701215	-7.12666173	-3.099192951	-4.103916502	6.448560851	0.010592748

CR1L	-0.309473631	0.03154286	-0.133601447	0.34101649	0.175872184	-0.165144306	8	0.417961087	0.816496581	0.816496581	0.816496581	0.816496581	0.816496581	0.816496581	1	36	0	P_104681	P_104681	P_104681	chr1	207723622	207723682	+	CR1L	complement component (3b/4b) receptor 1-like [Source:HGNC Symbol;Acc:HGNC:2335]	ENSG00000197721	protein_coding	exon	coding exon	1	36	1:207723622:207723682	ENSG00000197721	CR1L	2.57385149	0.14249346	-1.004088085	0.102340899	-0.433470279	1.106428984	0.570617807	-0.535811177	9.961963646	0.339101503	0.920516816	0.673909138	0.294542852	0.580900659	0.60384521	-6.474503559	-6.176569029	-6.945471205	-6.518423954	-6.126362625	-6.916771433	0.50724107	0.686075245

DDX52	0.053299923	-0.022807068	0.09822166	-0.076106991	0.044921737	0.121028728	8	0.057988091	0.816496581	0.816496581	0.816496581	0.816496581	0.816496581	0.816496581	1	37	0	P_228541	P_228541	P_228541	chr17	37626787	37626847	-	DDX52	DEAD (Asp-Glu-Ala-Asp) box polypeptide 52 [Source:HGNC Symbol;Acc:HGNC:20038]	ENSG00000278053	protein_coding	3'UTR;exon	multiple Ref	1	37	17:37626787:37626847	ENSG00000278053	DDX52	9.393837419	0.00490705	0.93188423	-0.398753807	1.717286083	-1.330638038	0.785401853	2.116039891	9.961963646	0.373429799	0.698487835	0.116797246	0.212962087	0.45049347	0.060528501	-6.544164107	-6.098954306	-5.637878223	-6.258841472	-5.978669237	-5.054220074	1.806927131	0.209719334

RICTOR	-0.002826748	-0.157348204	-0.118388565	-0.154521456	-0.115561817	0.038959639	8	0.065547364	0.816496581	0.816496581	0.816496581	0.816496581	0.816496581	0.816496581	1	38	0	P_252599	P_252599	P_252599	chr5	38975536	38975596	-	RICTOR	"RPTOR independent companion of MTOR, complex 2 [Source:HGNC Symbol;Acc:HGNC:28611]"	ENSG00000164327	protein_coding	3'UTR;exon	multiple Ref	1	38	5:38975536:38975596	ENSG00000164327	RICTOR	6.102099978	0.005656973	-0.046029962	-2.562213766	-1.927805993	-2.516183804	-1.881776031	0.634407773	9.961963646	0.964196026	0.028344189	0.082845484	0.030668	0.08937525	0.54010912	-6.997204252	-3.556604466	-5.324554431	-4.474139995	-4.694130691	-6.855861977	3.426371546	0.060666051

DNMT1	-0.312292895	-0.132901471	-0.166348353	0.179391425	0.145944542	-0.033446883	8	0.064877692	0.816496581	0.816496581	0.816496581	0.816496581	0.816496581	0.816496581	1	39	0	P_232291	P_232291	P_232291	chr19	10173100	10173160	-	DNMT1	DNA (cytosine-5-)-methyltransferase 1 [Source:HGNC Symbol;Acc:HGNC:2976]	ENSG00000130816	protein_coding	3'UTR;5'UTR;exon	multiple Ref	1	39	19:10173100:10173160	ENSG00000130816	DNMT1	10.33521638	0.005586833	-5.11711172	-2.177672572	-2.725720374	2.939439148	2.391391346	-0.548047801	9.961963646	0.000458062	0.054559736	0.021418683	0.014857017	0.037957091	0.595725163	-0.050942842	-4.15610031	-4.023306399	-3.755337992	-3.913887395	-6.909734911	8.836008868	0.003701366

TRPC7	-0.008733259	-0.108966269	0.061391838	-0.10023301	0.070125097	0.170358107	8	0.123382067	0.816496581	0.816496581	0.816496581	0.816496581	0.816496581	0.816496581	1	40	0	P_254922	P_254922	P_254922	chr5	136231353	136231413	-	TRPC7	"transient receptor potential cation channel, subfamily C, member 7 [Source:HGNC Symbol;Acc:HGNC:20754]"	ENSG00000069018	protein_coding	3'UTR;exon	multiple Ref	1	40	5:136231353:136231413	ENSG00000069018	TRPC7	4.199003494	0.014431691	-0.089035533	-1.110910568	0.625889484	-1.021875035	0.714925017	1.736800052	9.961963646	0.930818207	0.292699763	0.545459335	0.331014349	0.491069786	0.113183298	-6.994036172	-5.570298509	-6.837318207	-6.606718051	-6.031797003	-5.633289518	1.034268009	0.418867483

RCAN2	0.414843121	0.244527077	0.362887784	-0.170316044	-0.051955337	0.118360706	8	0.151282528	0.816496581	0.816496581	0.816496581	0.816496581	0.816496581	0.816496581	1	41	0	P_142830	P_142830	P_142830	chr6	46223068	46223128	-	RCAN2	regulator of calcineurin 2 [Source:HGNC Symbol;Acc:HGNC:3041]	ENSG00000172348	protein_coding	3'UTR;exon	multiple Ref	1	41	6:46223068:46223128	ENSG00000172348	RCAN2	5.700676182	0.020585714	3.541168227	2.087322827	3.097669032	-1.4538454	-0.443499195	1.010346205	9.961963646	0.005377882	0.06351835	0.011344835	0.176756643	0.666881523	0.336239559	-2.58845645	-4.292947479	-3.388746856	-6.10201109	-6.193120959	-6.540659991	4.97058331	0.023113574

ATP6V0A4	0.022619072	-0.078926709	0.012493189	-0.101545781	-0.010125884	0.091419897	8	0.118636671	0.816496581	0.816496581	0.816496581	0.816496581	0.816496581	0.816496581	1	42	0	P_146032	P_146032	P_146032	chr7	138706487	138706547	+	ATP6V0A4	"ATPase, H+ transporting, lysosomal V0 subunit a4 [Source:HGNC Symbol;Acc:HGNC:866]"	ENSG00000105929	protein_coding	"3'ext,3'UTR;3'UTR"	multiple non-Ref	0	42	7:138706487:138706547	ENSG00000105929	ATP6V0A4	4.380235269	0.013509403	0.238342962	-0.831670966	0.131643931	-1.070013928	-0.106699031	0.963314897	9.961963646	0.816446149	0.425080916	0.897886696	0.309852981	0.917145881	0.358189961	-6.96744975	-5.830061957	-7.037664728	-6.557132348	-6.290633469	-6.586820299	0.475017361	0.706623069

PYROXD2	0.090998905	0.036862574	0.106048703	-0.054136332	0.015049797	0.069186129	8	0.078409844	0.816496581	0.816496581	0.816496581	0.816496581	0.816496581	0.816496581	1	43	0	P_106787	P_106787	P_106787	chr10	98385076	98385136	-	PYROXD2	pyridine nucleotide-disulphide oxidoreductase domain 2 [Source:HGNC Symbol;Acc:HGNC:23517]	ENSG00000119943	protein_coding	intron	intron sense	0	43	10:98385076:98385136	ENSG00000119943	PYROXD2	7.718408803	0.007143946	1.31859888	0.534148715	1.536674532	-0.784450165	0.218075652	1.002525817	9.961963646	0.216812353	0.604952824	0.155503466	0.45102645	0.831772407	0.339818765	-6.124442223	-6.033879519	-5.890832627	-6.823136977	-6.271452517	-6.54846029	1.008893923	0.428898018

TTC7B	-0.224641147	-0.001881885	-0.22598091	0.222759262	-0.001339763	-0.224099025	8	0.071664471	0.816496581	0.816496581	0.816496581	0.816496581	0.816496581	0.816496581	1	44	0	P_221872	P_221872	P_221872	chr14	90655036	90655096	-	TTC7B	tetratricopeptide repeat domain 7B [Source:HGNC Symbol;Acc:HGNC:19858]	ENSG00000165914	protein_coding	exon	coding exon	1	44	14:90655036:90655096	ENSG00000165914	TTC7B	8.020141274	0.006331009	-3.457788997	-0.028966914	-3.478411303	3.428822083	-0.020622306	-3.449444389	9.961963646	0.006179896	0.977463071	0.005970652	0.006486702	0.983954152	0.006266708	-2.729878018	-6.181645103	-2.738612279	-2.916662876	-6.296459062	-2.8098642	7.952066961	0.005328012

PYGM	-0.131517271	0.134910238	-0.230772723	0.266427509	-0.099255452	-0.36568296	8	0.166429326	0.816496581	0.816496581	0.816496581	0.816496581	0.816496581	0.816496581	1	45	0	P_212062	P_212062	P_212062	chr11	64753127	64753187	-	PYGM	"phosphorylase, glycogen, muscle [Source:HGNC Symbol;Acc:HGNC:9726]"	ENSG00000068976	protein_coding	exon	coding exon	1	45	11:64753127:64753187	ENSG00000068976	PYGM	4.974973305	0.024450267	-1.030117144	1.056692767	-1.807541598	2.086809911	-0.777424454	-2.864234365	9.961963646	0.327316152	0.315603223	0.100920306	0.063573011	0.45497386	0.016895572	-6.44835074	-5.625443179	-5.505756042	-5.176364153	-5.984904721	-3.810387024	3.101325075	0.076182423

METTL7A	-0.188738045	0.132890992	-0.151146187	0.321629037	0.037591857	-0.28403718	8	0.045547682	0.816496581	0.816496581	0.816496581	0.816496581	0.816496581	0.816496581	1	46	0	A_23_P415021	A_23_P415021	A_23_P415021	chr12	50932317	50932377	+	METTL7A	methyltransferase like 7A [Source:HGNC Symbol;Acc:HGNC:24550]	ENSG00000185432	protein_coding	3'UTR	3'UTR	1	46	12:50932317:50932377	ENSG00000185432	METTL7A	9.036653025	0.003872694	-3.714484008	2.615378726	-2.974652497	6.329862734	0.739831511	-5.590031223	9.961963646	0.004037774	0.025876949	0.013989997	8.72E-05	0.476475263	0.000234101	-2.296000398	-3.472229934	-3.599167295	1.564160947	-6.01352966	0.591009862	16.89151946	0.000310998

MRAP	-0.258117277	0.145883277	0.014710282	0.404000554	0.272827559	-0.131172995	8	0.166835115	0.816496581	0.816496581	0.816496581	0.816496581	0.816496581	0.816496581	1	47	0	P_241573	P_241573	P_241573	chr21	32306679	32306739	+	MRAP	melanocortin 2 receptor accessory protein [Source:HGNC Symbol;Acc:HGNC:1304]	ENSG00000170262	protein_coding	exon	coding exon	1	47	21:32306679:32306739	ENSG00000170262	MRAP	5.454828873	0.024558868	-2.017244458	1.140110554	0.114964154	3.157355011	2.132208611	-1.0251464	9.961963646	0.071415475	0.280909739	0.910757747	0.010251219	0.058904773	0.329542777	-5.139334225	-5.539719985	-7.039907616	-3.381621156	-4.31872842	-6.525763281	3.491412234	0.058029651

PRR23C	-0.188990278	0.354882291	-0.369872937	0.543872569	-0.180882659	-0.724755228	8	0.571324598	0.816496581	0.816496581	0.816496581	0.816496581	0.816496581	0.816496581	1	48	0	P_136153	P_136153	P_136153	chr3	139042128	139042188	-	PRR23C	proline rich 23C [Source:HGNC Symbol;Acc:HGNC:37173]	ENSG00000233701	protein_coding	3'UTR	3'UTR	1	48	3:139042128:139042188	ENSG00000233701	PRR23C	3.100069286	0.264333155	-0.450203921	0.845384223	-0.881094248	1.295588145	-0.430890327	-1.726478471	9.961963646	0.662202018	0.417738236	0.399025606	0.224332377	0.675722372	0.115081983	-6.888876661	-5.818770813	-6.638985326	-6.301706935	-6.198870954	-5.648267946	1.089771359	0.397799056

FAM47E	0.409441766	0.086111507	0.37839446	-0.323330259	-0.031047306	0.292282953	8	0.047071872	0.816496581	0.816496581	0.816496581	0.816496581	0.816496581	0.816496581	1	49	0	P_249482	P_249482	P_249482	chr4	76268708	76268768	+	FAM47E	"family with sequence similarity 47, member E [Source:HGNC Symbol;Acc:HGNC:34343]"	ENSG00000189157	protein_coding	exon	coding exon	1	49	4:76268708:76268768	ENSG00000189157	FAM47E	6.68318044	0.003986061	7.942656911	1.670455269	7.340378102	-6.272201642	-0.602278809	5.669922833	9.961963646	1.28E-05	0.125900157	2.53E-05	9.39E-05	0.560446508	0.000209658	3.644915675	-4.892451378	2.919073954	1.486327388	-6.107292473	0.705946097	31.93477474	2.01E-05

PLD3	-0.00438445	-0.157336076	-0.050131871	-0.152951627	-0.045747421	0.107204206	8	0.053708385	0.816496581	0.816496581	0.816496581	0.816496581	0.816496581	0.816496581	1	50	0	P_125821	P_125821	P_125821	chr19	40378223	40378283	+	PLD3	"phospholipase D family, member 3 [Source:HGNC Symbol;Acc:HGNC:17158]"	ENSG00000105223	protein_coding	3'UTR	3'UTR	1	50	19:40378223:40378283	ENSG00000105223	PLD3	8.940028763	0.004523168	-0.079843469	-2.865184605	-0.912931522	-2.785341136	-0.833088053	1.952253083	9.961963646	0.937942924	0.016868125	0.382840999	0.019338862	0.424318126	0.079561379	-6.994882663	-3.073440855	-6.610142219	-4.018710299	-5.940236567	-5.310585843	3.55209247	0.055691898

WASF1	0.223756574	-0.071704009	0.117158954	-0.295460583	-0.10659762	0.188862963	8	0.090669977	0.816496581	0.816496581	0.816496581	0.816496581	0.816496581	0.816496581	1	51	0	P_258686	P_258686	P_258686	chr6	110127488	110127548	-	WASF1	"WAS protein family, member 1 [Source:HGNC Symbol;Acc:HGNC:12732]"	ENSG00000112290	protein_coding	exon	coding exon	1	51	6:110127488:110127548	ENSG00000112290	WASF1	6.805525417	0.008808631	2.919894905	-0.93569617	1.52885713	-3.855591075	-1.391037775	2.464553299	9.961963646	0.015361553	0.371557079	0.157409327	0.00320554	0.1944987	0.033499306	-3.647503358	-5.740501302	-5.901403942	-2.193689644	-5.359072592	-4.485657607	5.768884882	0.014943139

DTWD2	0.070081397	0.138741676	0.191580329	0.06866028	0.121498932	0.052838653	8	0.089114047	0.816496581	0.816496581	0.816496581	0.816496581	0.816496581	0.816496581	1	52	0	P_140154	P_140154	P_140154	chr5	118840927	118840987	-	DTWD2	DTW domain containing 2 [Source:HGNC Symbol;Acc:HGNC:19334]	ENSG00000169570	protein_coding	3'UTR;exon	multiple Ref	1	52	5:118840927:118840987	ENSG00000169570	DTWD2	8.029091439	0.008583992	0.926411069	1.834036294	2.532514278	0.907625224	1.606103209	0.698477984	9.961963646	0.376130436	0.096650388	0.029822722	0.385505794	0.13944816	0.500858021	-6.549267966	-4.66446572	-4.349085079	-6.716879516	-5.081586651	-6.811218077	2.421129621	0.126835508

GOLGA5	0.113547526	0.116993766	0.157107291	0.00344624	0.043559765	0.040113525	8	0.03908101	0.816496581	0.816496581	0.816496581	0.816496581	0.816496581	0.816496581	1	53	0	A_23_P3193	A_23_P3193	A_23_P3193	chr14	92839627	92839687	+	GOLGA5	golgin A5 [Source:HGNC Symbol;Acc:HGNC:4428]	ENSG00000066455	protein_coding	3'UTR	3'UTR	1	53	14:92839627:92839687	ENSG00000066455	GOLGA5	8.9481227	0.00343321	2.373412093	2.445446669	3.283914301	0.072034576	0.910502207	0.838467632	9.961963646	0.039138776	0.034611167	0.008275424	0.944000098	0.384059365	0.421430836	-4.567180984	-3.740923361	-3.070201776	-7.146776127	-5.873709926	-6.700349728	3.989103121	0.041795362

GXYLT2	0.475878468	0.367216258	0.793380124	-0.108662209	0.317501656	0.426163866	8	0.074799481	0.816496581	0.816496581	0.816496581	0.816496581	0.816496581	0.816496581	1	54	0	P_244950	P_244950	P_244950	chr3	72908499	72908559	+	GXYLT2	glucoside xylosyltransferase 2 [Source:HGNC Symbol;Acc:HGNC:33383]	ENSG00000172986	protein_coding	exon	coding exon	1	54	3:72908499:72908559	ENSG00000172986	GXYLT2	7.60896972	0.006699744	7.12053433	5.494629726	11.87128814	-1.625904604	4.750753808	6.376658412	9.961963646	3.28E-05	0.000267364	3.35E-07	0.135146961	0.000787605	8.21E-05	2.679974895	0.803652715	7.302077373	-5.868382892	-0.208573001	1.682926292	47.94924118	3.16E-06

PCCB	-0.00790838	0.075671462	0.173449088	0.083579842	0.181357468	0.097777626	8	0.079306731	0.816496581	0.816496581	0.816496581	0.816496581	0.816496581	0.816496581	1	55	0	P_246316	P_246316	P_246316	chr3	136301051	136301111	+	PCCB	"propionyl CoA carboxylase, beta polypeptide [Source:HGNC Symbol;Acc:HGNC:8654]"	ENSG00000114054	protein_coding	exon	coding exon	1	55	3:136301051:136301111	ENSG00000114054	PCCB	9.874735396	0.007257542	-0.113694217	1.087884994	2.493577573	1.201579211	2.607271791	1.40569258	9.961963646	0.911738862	0.30226482	0.03187735	0.257313147	0.026238881	0.19022873	-6.991311105	-5.593980091	-4.414186227	-6.412592892	-3.568458267	-6.087287584	2.938663278	0.085696636

PPP3CB	0.14853334	-0.011135723	0.037134976	-0.159669063	-0.111398364	0.048270699	8	0.105761006	0.816496581	0.816496581	0.816496581	0.816496581	0.816496581	0.816496581	1	56	0	P_208701	P_208701	P_208701	chr10	73467552	73467612	-	PPP3CB	"protein phosphatase 3, catalytic subunit, beta isozyme [Source:HGNC Symbol;Acc:HGNC:9315]"	ENSG00000107758	protein_coding	exon	coding exon	1	56	10:73467552:73467612	ENSG00000107758	PPP3CB	7.664925707	0.011189162	1.719771671	-0.128933344	0.429961918	-1.848705015	-1.289809753	0.558895262	9.961963646	0.116331172	0.899976328	0.676375407	0.094358667	0.226254288	0.588575383	-5.58676064	-6.173331433	-6.947094892	-5.544279057	-5.48060755	-6.903372255	1.425464834	0.292818868

ZNRF2	0.033310502	0.109025197	0.127019519	0.075714695	0.093709016	0.017994322	8	0.086745033	0.816496581	0.816496581	0.816496581	0.816496581	0.816496581	0.816496581	1	57	0	P_144538	P_144538	P_144538	chr7	30355773	30355833	+	ZNRF2	zinc and ring finger 2 [Source:HGNC Symbol;Acc:HGNC:22316]	ENSG00000180233	protein_coding	exon	coding exon	1	57	7:30355773:30355833	ENSG00000180233	ZNRF2	6.465595139	0.008249429	0.449174267	1.470146338	1.712790117	1.02097207	1.26361585	0.242643779	9.961963646	0.662919672	0.17238565	0.117644589	0.331421398	0.235137202	0.813204021	-6.889371863	-5.155399632	-5.644366068	-6.607630603	-5.511014125	-7.039309702	1.332453791	0.31841356

MED13L	0.035527688	-0.118272227	-0.068234822	-0.153799915	-0.10376251	0.050037405	8	0.075685955	0.816496581	0.816496581	0.816496581	0.816496581	0.816496581	0.816496581	1	59	0	P_217651	P_217651	P_217651	chr12	115980830	115980890	-	MED13L	mediator complex subunit 13-like [Source:HGNC Symbol;Acc:HGNC:22962]	ENSG00000123066	protein_coding	exon	coding exon	1	59	12:115980830:115980890	ENSG00000123066	MED13L	6.431417135	0.006806872	0.527398349	-1.755717338	-1.012926391	-2.283115687	-1.54032474	0.742790947	9.961963646	0.609461039	0.109777232	0.33506484	0.045638569	0.154620503	0.474759522	-6.848673918	-4.774955922	-6.514015832	-4.860256808	-5.169034141	-6.778069764	2.087278033	0.165831861

KAT6B	0.196642302	-0.172237802	0.143465907	-0.368880104	-0.053176396	0.315703708	8	0.067117812	0.816496581	0.816496581	0.816496581	0.816496581	0.816496581	0.816496581	1	60	0	A_23_P388855	A_23_P388855	A_23_P388855	chr10	75030747	75030807	+	KAT6B	K(lysine) acetyltransferase 6B [Source:HGNC Symbol;Acc:HGNC:17582]	ENSG00000156650	protein_coding	exon	coding exon	1	60	10:75030747:75030807	ENSG00000156650	KAT6B	8.131563186	0.005824285	3.155740525	-2.764094012	2.302359003	-5.919834537	-0.853381521	5.066453015	9.961963646	0.010279334	0.020055703	0.044170687	0.000149338	0.413496226	0.000493143	-3.244750493	-3.235155862	-4.730272131	1.00059556	-5.923286245	-0.18508857	14.05690301	0.000651518

CAB39L	-0.568952727	0.05051813	-0.649525959	0.619470857	-0.080573231	-0.700044088	8	0.039580994	0.816496581	0.816496581	0.816496581	0.816496581	0.816496581	0.816496581	1	61	0	P_113810	P_113810	P_113810	chr13	49310656	49310716	-	CAB39L	calcium binding protein 39-like [Source:HGNC Symbol;Acc:HGNC:20290]	ENSG00000102547	protein_coding	3'UTR	3'UTR	1	61	13:49310656:49310716	ENSG00000102547	CAB39L	10.04869968	0.003464793	-11.83813092	1.051124641	-13.51460844	12.88925556	-1.67647752	-14.56573308	9.961963646	3.44E-07	0.318030656	9.87E-08	1.55E-07	0.124694775	4.84E-08	7.272321431	-5.630983877	8.488054663	8.066930168	-4.985738704	9.175779629	117.4991523	4.49E-08

CALCRL	-0.00791981	0.19925646	0.051189826	0.20717627	0.059109636	-0.148066634	8	0.10594017	0.816496581	0.816496581	0.816496581	0.816496581	0.816496581	0.816496581	1	62	0	P_238228	P_238228	P_238228	chr2	187380679	187380739	-	CALCRL	calcitonin receptor-like [Source:HGNC Symbol;Acc:HGNC:16709]	ENSG00000064989	protein_coding	exon	coding exon	1	62	2:187380679:187380739	ENSG00000064989	CALCRL	4.089258255	0.011219622	-0.09157381	2.30392817	0.591888878	2.395501981	0.683462688	-1.712039292	9.961963646	0.928851904	0.044053036	0.567114172	0.03769187	0.509897826	0.117786636	-6.993786213	-3.961901389	-6.85911208	-4.675294573	-6.054066635	-5.669141331	2.467238791	0.122342479

PRKAR2B	0.267238489	0.023668987	0.136960939	-0.243569502	-0.13027755	0.113291952	8	0.048516435	0.816496581	0.816496581	0.816496581	0.816496581	0.816496581	0.816496581	1	63	0	P_145627	P_145627	P_145627	chr7	107159522	107159582	+	PRKAR2B	"protein kinase, cAMP-dependent, regulatory, type II, beta [Source:HGNC Symbol;Acc:HGNC:9392]"	ENSG00000005249	protein_coding	exon	coding exon	1	63	7:107159522:107159582	ENSG00000005249	PRKAR2B	10.62508607	0.004096949	5.113453803	0.452892367	2.620668288	-4.660561437	-2.492785515	2.167775921	9.961963646	0.000460503	0.660329903	0.025643482	0.000902693	0.031920566	0.055478536	-0.056455395	-6.075102482	-4.200953079	-0.879855593	-3.752352365	-4.971592456	10.97574809	0.001686742

CLIC5	0.135204534	-0.117885867	0.071567024	-0.253090401	-0.06363751	0.189452891	8	0.106545623	0.816496581	0.816496581	0.816496581	0.816496581	0.816496581	0.816496581	1	64	0	A_23_P311895	A_23_P311895	A_23_P311895	chr6	45898902	45898962	-	CLIC5	chloride intracellular channel 5 [Source:HGNC Symbol;Acc:HGNC:13517]	ENSG00000112782	protein_coding	3'UTR	3'UTR	1	64	6:45898902:45898962	ENSG00000112782	CLIC5	3.805649844	0.011322935	1.556171233	-1.3568376	0.823719007	-2.913008833	-0.732452226	2.180556607	9.961963646	0.150838155	0.204780163	0.429378461	0.015543424	0.480770498	0.054294748	-5.816574286	-5.294853744	-6.688740912	-3.800592572	-6.019000257	-4.951070896	3.119687338	0.075189001

MIR4300HG	-0.222680708	-0.151723278	0.19808965	0.070957429	0.420770358	0.349812928	8	0.4276328	0.816496581	0.816496581	0.816496581	0.816496581	0.816496581	0.816496581	1	67	0	A_16_P19303238	A_16_P19303238	A_16_P19303238	chr11	81974592	81974652	+	RP11-179A16.1;RP11-876F14.1	NA	ENSG00000245832	lincRNA	intron	multiple non-Ref	0	67	11:81974592:81974652	ENSG00000245832	MIR4300HG	3.948412654	0.149061114	-0.706392437	-0.481299783	0.628384166	0.225092654	1.334776603	1.109683949	9.961963646	0.496132959	0.640700382	0.543889316	0.826457364	0.211651761	0.293203329	-6.732719225	-6.061425048	-6.835674803	-7.121975787	-5.427394674	-6.437372908	0.698200028	0.574234575

TBC1D25	-0.049376629	0.123367913	-0.100448343	0.172744542	-0.051071714	-0.223816256	8	0.091252206	0.816496581	0.816496581	0.816496581	0.816496581	0.816496581	0.816496581	1	68	0	P_150846	P_150846	P_150846	chrX	48562544	48562604	+	TBC1D25	"TBC1 domain family, member 25 [Source:HGNC Symbol;Acc:HGNC:8092]"	ENSG00000068354	protein_coding	3'UTR	3'UTR	1	68	X:48562544:48562604	ENSG00000068354	TBC1D25	8.576586208	0.008893691	-0.641248032	1.602163479	-1.304509916	2.24341151	-0.663261884	-2.906673395	9.961963646	0.535834916	0.140318318	0.221391466	0.048818352	0.522214011	0.015712678	-6.778516453	-4.98427308	-6.189665333	-4.924963361	-6.067885585	-3.737932493	3.100239121	0.07624166

TXNRD1	-0.107888015	-0.147871333	-0.185130444	-0.039983318	-0.077242429	-0.037259112	8	0.07113713	0.816496581	0.816496581	0.816496581	0.816496581	0.816496581	0.816496581	1	70	0	P_217168	P_217168	P_217168	chr12	104331578	104331638	+	TXNRD1	thioredoxin reductase 1 [Source:HGNC Symbol;Acc:HGNC:12437]	ENSG00000198431	protein_coding	exon	coding exon	1	70	12:104331578:104331638	ENSG00000198431	TXNRD1	10.85528715	0.006270534	-1.668655006	-2.287058851	-2.863328636	-0.618403845	-1.19467363	-0.576269785	9.961963646	0.126262518	0.045333973	0.016921776	0.550185966	0.259882493	0.577219756	-5.659960528	-3.988026469	-3.789249502	-6.944416497	-5.588887717	-6.892937514	3.059242309	0.078518417

REPS2	0.200835018	0.202016773	0.08671876	0.001181755	-0.114116258	-0.115298013	8	0.136832643	0.816496581	0.816496581	0.816496581	0.816496581	0.816496581	0.816496581	1	71	0	P_269620	P_269620	P_269620	chrX	17135299	17135359	+	REPS2	RALBP1 associated Eps domain containing 2 [Source:HGNC Symbol;Acc:HGNC:9963]	ENSG00000169891	protein_coding	exon	coding exon	1	71	X:17135299:17135359	ENSG00000169891	REPS2	8.513929793	0.017242412	1.873208231	1.884230576	0.80883452	0.011022345	-1.064373711	-1.075396056	9.961963646	0.090642338	0.089015292	0.437500777	0.991423232	0.312277825	0.307552522	-5.360206284	-4.592467808	-6.701173068	-7.149546624	-5.727041205	-6.473891703	1.667156131	0.236553809

HDAC8	-0.054502274	-0.01205159	0.045168046	0.042450685	0.09967032	0.057219636	8	0.072369347	0.816496581	0.816496581	0.816496581	0.816496581	0.816496581	0.816496581	1	72	0	P_151198	P_151198	P_151198	chrX	72488932	72488992	-	HDAC8	histone deacetylase 8 [Source:HGNC Symbol;Acc:HGNC:13315]	ENSG00000147099	protein_coding	3'UTR;exon	multiple Ref	1	72	X:72488932:72488992	ENSG00000147099	HDAC8	6.727703648	0.006412539	-0.833576058	-0.184321057	0.690815235	0.649255001	1.524391293	0.875136292	9.961963646	0.424055657	0.857459665	0.505458996	0.530856799	0.158507236	0.40210661	-6.631929192	-6.164208907	-6.792597592	-6.923863752	-5.189889078	-6.668396003	0.786202231	0.52860235

FER	0.096218313	-0.050950959	0.045089056	-0.147169272	-0.051129257	0.096040015	8	0.055266465	0.816496581	0.816496581	0.816496581	0.816496581	0.816496581	0.816496581	1	73	0	P_254235	P_254235	P_254235	chr5	109100459	109100519	+	FER	fer (fps/fes related) tyrosine kinase [Source:HGNC Symbol;Acc:HGNC:3655]	ENSG00000151422	protein_coding	3'UTR;exon	multiple Ref	1	73	5:109100459:109100519	ENSG00000151422	FER	6.97430329	0.004659519	1.726365731	-0.914170981	0.808995699	-2.640536712	-0.917370033	1.72316668	9.961963646	0.11510288	0.382220433	0.437412279	0.024785139	0.380622045	0.115697298	-5.577230677	-5.75976311	-6.701039506	-4.264683681	-5.867566565	-5.653063824	2.542304429	0.115421897

NEB	0.219830323	-0.047347201	0.071283048	-0.267177524	-0.148547276	0.118630248	8	0.043763376	0.816496581	0.816496581	0.816496581	0.816496581	0.816496581	0.816496581	1	74	0	P_236899	P_236899	P_236899	chr2	151710433	151710493	-	NEB	nebulin [Source:HGNC Symbol;Acc:HGNC:7720]	ENSG00000183091	protein_coding	exon	coding exon	1	74	2:151710433:151710493	ENSG00000183091	NEB	4.366712654	0.003744721	4.399704608	-0.947611295	1.426665566	-5.347315903	-2.973039041	2.374276862	9.961963646	0.001347951	0.365746924	0.184256781	0.000329076	0.014028578	0.039081123	-1.168181236	-5.729678879	-6.036447355	0.173728575	-2.974987924	-4.635343855	10.89344693	0.001734742

PVT1	-0.233570476	-0.269371624	-0.326165682	-0.035801149	-0.092595206	-0.056794057	8	0.105764519	0.816496581	0.816496581	0.816496581	0.816496581	0.816496581	0.816496581	1	75	0	P_147973	P_147973	P_147973	chr8	128040670	128040730	+	PVT1	Pvt1 oncogene (non-protein coding) [Source:HGNC Symbol;Acc:HGNC:9709]	ENSG00000249859	lincRNA	intron	intron sense	0	75	8:128040670:128040730	ENSG00000249859	PVT1	6.611922419	0.011189759	-2.704289652	-3.11879699	-3.776361184	-0.414507338	-1.072071531	-0.657564194	9.961963646	0.022219911	0.010944689	0.003648036	0.687287048	0.308971962	0.525719593	-4.013910614	-2.667464346	-2.235030072	-7.056458382	-5.719222616	-6.840179916	5.509087559	0.017152794

LONRF1	-0.067821188	0.176376226	-0.1706137	0.244197415	-0.102792511	-0.346989926	8	0.07742737	0.816496581	0.816496581	0.816496581	0.816496581	0.816496581	0.816496581	1	76	0	P_146547	P_146547	P_146547	chr8	12723153	12723213	+	LONRF1	LON peptidase N-terminal domain and ring finger 1 [Source:HGNC Symbol;Acc:HGNC:26302]	ENSG00000154359	protein_coding	3'UTR;exon	multiple non-Ref	0	76	8:12723153:12723213	ENSG00000154359	LONRF1	7.440012779	0.007020994	-0.991315099	2.57802055	-2.493792004	3.569335649	-1.502476905	-5.071812554	9.961963646	0.344998771	0.027587173	0.03186566	0.005131999	0.163992289	0.0004893	-6.48713792	-3.531544387	-4.413828305	-2.677205439	-5.21835671	-0.176943996	9.094795092	0.003342961

TP53BP1	0.001991938	-0.069977551	-0.01855517	-0.071969489	-0.020547108	0.051422381	8	0.049328509	0.816496581	0.816496581	0.816496581	0.816496581	0.816496581	0.816496581	1	77	0	P_223103	P_223103	P_223103	chr15	43415643	43415703	-	TP53BP1	tumor protein p53 binding protein 1 [Source:HGNC Symbol;Acc:HGNC:11999]	ENSG00000067369	protein_coding	3'UTR;exon	multiple Ref	1	77	15:43415643:43415703	ENSG00000067369	TP53BP1	7.531581282	0.004160758	0.037821196	-1.328673085	-0.352309489	-1.366494282	-0.390130686	0.976363596	9.961963646	0.970577425	0.213586583	0.731945571	0.201831749	0.704651426	0.351997483	-6.997579793	-5.328362135	-6.979758235	-6.214169215	-6.216363627	-6.574194567	0.810619839	0.516590265

PDCL3	-0.147215538	-0.269911806	-0.20868853	-0.122696268	-0.061472992	0.061223276	8	0.487250399	0.816496581	0.816496581	0.816496581	0.816496581	0.816496581	0.816496581	1	78	0	P_128539	P_128539	P_128539	chr2	100576679	100576739	+	PDCL3	phosducin-like 3 [Source:HGNC Symbol;Acc:HGNC:28860]	ENSG00000115539	protein_coding	"3'ext;3'ext,3'UTR"	multiple non-Ref	0	78	2:100576679:100576739	ENSG00000115539	PDCL3	7.399661219	0.192862229	-0.410559106	-0.752738136	-0.581996829	-0.34217903	-0.171437723	0.170741307	9.961963646	0.690086936	0.469021429	0.573502966	0.739325431	0.867311927	0.867845193	-6.907154897	-5.891911975	-6.86524003	-7.085958001	-6.281076415	-7.055475784	0.208253961	0.888374472

TTC37	0.050372559	-0.052711551	-0.006452965	-0.103084111	-0.056825524	0.046258587	8	0.060643151	0.816496581	0.816496581	0.816496581	0.816496581	0.816496581	0.816496581	1	80	0	P_254000	P_254000	P_254000	chr5	95536829	95536889	-	TTC37	tetratricopeptide repeat domain 37 [Source:HGNC Symbol;Acc:HGNC:23639]	ENSG00000198677	protein_coding	exon	coding exon	1	80	5:95536829:95536889	ENSG00000198677	TTC37	8.327184211	0.005159991	0.85884578	-0.898725299	-0.110022235	-1.757571079	-0.968868016	0.788703063	9.961963646	0.410614827	0.390004648	0.914576597	0.109448542	0.355545005	0.448647871	-6.610169674	-5.773352954	-7.04051404	-5.6795029	-5.820278575	-6.741809002	1.03454033	0.418761212

IL20RA	0.255928962	0.241157176	0.127931511	-0.014771786	-0.127997451	-0.113225665	8	0.172121164	0.816496581	0.816496581	0.816496581	0.816496581	0.816496581	0.816496581	1	81	0	P_259415	P_259415	P_259415	chr6	137009330	137009390	-	IL20RA	"interleukin 20 receptor, alpha [Source:HGNC Symbol;Acc:HGNC:6003]"	ENSG00000016402	protein_coding	exon	coding exon	1	81	6:137009330:137009390	ENSG00000016402	IL20RA	3.920747737	0.025997733	1.944004781	1.831800116	0.971751951	-0.112204664	-0.972252829	-0.860048165	9.961963646	0.080655536	0.097004232	0.354177014	0.912889845	0.353939816	0.409982643	-5.252550267	-4.667652386	-6.554597196	-7.142732449	-5.81709582	-6.681686671	1.629416068	0.244463684

MAPK1	-0.160085705	0.041094916	-0.075672931	0.201180621	0.084412774	-0.116767848	8	0.037541577	0.816496581	0.816496581	0.816496581	0.816496581	0.816496581	0.816496581	1	83	0	P_242228	P_242228	P_242228	chr22	21807663	21807723	-	MAPK1	mitogen-activated protein kinase 1 [Source:HGNC Symbol;Acc:HGNC:6871]	ENSG00000100030	protein_coding	exon	coding exon	1	83	22:21807663:21807723	ENSG00000100030	MAPK1	9.221753338	0.003338485	-3.393309485	0.871081957	-1.604026267	4.264391442	1.789283217	-2.475108224	9.961963646	0.006884441	0.404212617	0.139906289	0.001665827	0.103963109	0.032900259	-2.83950545	-5.79718565	-5.798410791	-1.516823594	-4.827571582	-4.468068022	7.059827137	0.007920411

XKR3	-0.041147297	-0.046911802	-0.053015605	-0.005764505	-0.011868308	-0.006103803	8	0.076715425	0.816496581	0.816496581	0.816496581	0.816496581	0.816496581	0.816496581	1	84	0	P_132546	P_132546	P_132546	chr22	16799774	16799834	-	XKR3	"XK, Kell blood group complex subunit-related family, member 3 [Source:HGNC Symbol;Acc:HGNC:28778]"	ENSG00000172967	protein_coding	exon	coding exon	1	84	22:16799774:16799834	ENSG00000172967	XKR3	3.421080415	0.006932866	-0.605244075	-0.690035366	-0.779817457	-0.084791292	-0.174573383	-0.089782091	9.961963646	0.558551651	0.505928695	0.453626824	0.934107138	0.86491173	0.930239826	-6.802079091	-5.937132425	-6.724836619	-7.145682759	-6.280501119	-7.066976662	0.249385119	0.859973234

PLP1	0.139445041	0.179172508	0.017724893	0.039727467	-0.121720148	-0.161447615	8	0.151174935	0.816496581	0.816496581	0.816496581	0.816496581	0.816496581	0.816496581	1	85	0	P_151399	P_151399	P_151399	chrX	103790692	103790752	+	PLP1	proteolipid protein 1 [Source:HGNC Symbol;Acc:HGNC:9086]	ENSG00000123560	protein_coding	3'UTR;exon	multiple Ref	1	85	X:103790692:103790752	ENSG00000123560	PLP1	3.4485645	0.02055958	1.191081779	1.530417345	0.151398695	0.339335566	-1.039683085	-1.37901865	9.961963646	0.261226998	0.157027326	0.882683224	0.741401885	0.32306294	0.198061099	-6.275004076	-5.078365892	-7.034617387	-7.087005469	-5.75181818	-6.121209057	1.1469261	0.377304055

TRAPPC11	0.156860819	0.036776811	0.035100529	-0.120084007	-0.12176029	-0.001676283	8	0.087192011	0.816496581	0.816496581	0.816496581	0.816496581	0.816496581	0.816496581	1	86	0	P_251802	P_251802	P_251802	chr4	183680170	183680230	+	TRAPPC11	trafficking protein particle complex 11 [Source:HGNC Symbol;Acc:HGNC:25751]	ENSG00000168538	protein_coding	exon	coding exon	1	86	4:183680170:183680230	ENSG00000168538	TRAPPC11	7.535386595	0.008311863	2.107225014	0.494049551	0.471530831	-1.613175463	-1.635694183	-0.02251872	9.961963646	0.06143166	0.631982726	0.647419014	0.137898158	0.133064519	0.982478848	-4.997911712	-6.055028688	-6.927042823	-5.886202858	-5.04156373	-7.071101506	1.697526862	0.23040164

LRSAM1	-0.3152074	-0.008219539	-0.26662672	0.30698786	0.04858068	-0.25840718	8	0.076716428	0.816496581	0.816496581	0.816496581	0.816496581	0.816496581	0.816496581	1	87	0	A_16_P18777817	A_16_P18777817	A_16_P18777817	chr9	127466341	127466401	+	LRSAM1;Y_RNA	leucine rich repeat and sterile alpha motif containing 1 [Source:HGNC Symbol;Acc:HGNC:25135];Y RNA [Source:RFAM;Acc:RF00019]	ENSG00000148356	protein_coding;misc_RNA	intron;promoter	multiple non-Ref	0	87	9:127466341:127466401	ENSG00000148356	LRSAM1	7.856197774	0.006932989	-4.636409293	-0.120901819	-3.921832423	4.515507473	0.71457687	-3.800930604	9.961963646	0.000936455	0.906172599	0.002878754	0.001126822	0.491275744	0.003504365	-0.791711143	-6.174387508	-1.991837544	-1.110724596	-6.03204837	-2.215312631	12.0398632	0.001189429

CDK13	-0.034493794	-0.261099754	-0.121574641	-0.22660596	-0.087080848	0.139525112	8	0.091309155	0.816496581	0.816496581	0.816496581	0.816496581	0.816496581	0.816496581	1	89	0	P_261049	P_261049	P_261049	chr7	40063033	40063093	+	CDK13	cyclin-dependent kinase 13 [Source:HGNC Symbol;Acc:HGNC:1733]	ENSG00000065883	protein_coding	exon	coding exon	1	89	7:40063033:40063093	ENSG00000065883	CDK13	6.950317924	0.00890204	-0.447756415	-3.389278984	-1.578133915	-2.941522568	-1.130377499	1.811145069	9.961963646	0.66390848	0.006931148	0.145730714	0.014804233	0.284797637	0.100329535	-6.890051982	-2.236902922	-5.834221477	-3.751769612	-5.658581941	-5.524034765	4.56479321	0.029288116

MPEG1	-0.074913417	-0.197576152	-0.125507358	-0.122662735	-0.050593941	0.072068794	8	0.34668704	0.816496581	0.816496581	0.816496581	0.816496581	0.816496581	0.816496581	1	91	0	P_108660	P_108660	P_108660	chr11	59210762	59210822	-	MPEG1	macrophage expressed 1 [Source:HGNC Symbol;Acc:HGNC:29619]	ENSG00000197629	protein_coding	exon	coding exon	1	91	11:59210762:59210822	ENSG00000197629	MPEG1	3.859068479	0.098727336	-0.292002472	-0.770125393	-0.48921088	-0.478122921	-0.197208408	0.280914513	9.961963646	0.776275253	0.459098608	0.635284304	0.642881586	0.847629966	0.78452388	-6.95203124	-5.878753139	-6.917978248	-7.026019881	-6.276041184	-7.02843915	0.210681916	0.886711524

SERPINB8	0.106815404	0.185166754	0.008129331	0.07835135	-0.098686073	-0.177037423	8	0.147462452	0.816496581	0.816496581	0.816496581	0.816496581	0.816496581	0.816496581	1	92	0	P_123743	P_123743	P_123743	chr18	63978346	63978406	+	SERPINB8	"serpin peptidase inhibitor, clade B (ovalbumin), member 8 [Source:HGNC Symbol;Acc:HGNC:8952]"	ENSG00000166401	protein_coding	exon	coding exon	1	92	18:63978346:63978406	ENSG00000166401	SERPINB8	5.056040977	0.019669245	0.932793847	1.617017787	0.070991539	0.68422394	-0.861802308	-1.546026248	9.961963646	0.372982312	0.137062492	0.94480945	0.509437155	0.40906156	0.153250142	-6.543313424	-4.964482673	-7.044369018	-6.899459762	-5.9161498	-5.901939932	1.181825577	0.365361328

SHPRH	0.028079211	-0.006754906	-0.044222257	-0.034834116	-0.072301468	-0.037467351	8	0.074510929	0.816496581	0.816496581	0.816496581	0.816496581	0.816496581	0.816496581	1	93	0	P_259645	P_259645	P_259645	chr6	145923642	145923702	-	SHPRH	"SNF2 histone linker PHD RING helicase, E3 ubiquitin protein ligase [Source:HGNC Symbol;Acc:HGNC:19336]"	ENSG00000146414	protein_coding	3'UTR;exon	multiple Ref	1	93	6:145923642:145923702	ENSG00000146414	SHPRH	6.583666638	0.006665145	0.42123623	-0.101335148	-0.663409559	-0.522571379	-1.084645789	-0.562074411	9.961963646	0.682526687	0.921294983	0.522123337	0.612695283	0.303629516	0.586488653	-6.902391907	-6.176676902	-6.811966923	-7.00229664	-5.706356072	-6.901485315	0.398880999	0.756848527

ARL6	-0.022461614	0.116626771	0.08124012	0.139088385	0.103701734	-0.035386651	8	0.091605093	0.816496581	0.816496581	0.816496581	0.816496581	0.816496581	0.816496581	1	95	0	P_135424	P_135424	P_135424	chr3	97784844	97784904	+	ARL6	ADP-ribosylation factor-like 6 [Source:HGNC Symbol;Acc:HGNC:13210]	ENSG00000113966	protein_coding	intron;promoter	multiple non-Ref	0	95	3:97784844:97784904	ENSG00000113966	ARL6	6.894021358	0.008945511	-0.29086001	1.510223822	1.051994871	1.801083832	1.342854881	-0.458228951	9.961963646	0.777123833	0.162034661	0.317650348	0.101987032	0.209113465	0.656621027	-6.952391518	-5.104386245	-6.474250629	-5.615369051	-5.417702057	-6.957869719	1.454867453	0.285218031

TYMS	-0.578649186	-0.083859917	-0.501090511	0.494789269	0.077558675	-0.417230594	8	0.070149108	0.816496581	0.816496581	0.816496581	0.816496581	0.816496581	0.816496581	1	96	0	P_123099	P_123099	P_123099	chr18	673016	673076	+	TYMS	thymidylate synthetase [Source:HGNC Symbol;Acc:HGNC:12441]	ENSG00000176890	protein_coding	3'UTR;exon	multiple Ref	1	96	18:673016:673076	ENSG00000176890	TYMS	12.79023804	0.006158433	-9.030792631	-1.30877488	-7.82035921	7.722017751	1.210433421	-6.51158433	9.961963646	4.12E-06	0.219996907	1.47E-05	1.64E-05	0.254048627	6.92E-05	4.801967636	-5.351745579	3.482213732	3.309965161	-5.57136743	1.861473413	41.32026052	6.26E-06

MANEA	0.876640158	0.015460267	0.717797707	-0.861179891	-0.158842451	0.70233744	8	0.084068558	0.816496581	0.816496581	0.816496581	0.816496581	0.816496581	0.816496581	1	97	0	P_258345	P_258345	P_258345	chr6	95606345	95606405	+	MANEA	"mannosidase, endo-alpha [Source:HGNC Symbol;Acc:HGNC:21072]"	ENSG00000172469	protein_coding	exon	coding exon	1	97	6:95606345:95606405	ENSG00000172469	MANEA	9.070391967	0.00788229	12.09318755	0.213273264	9.901967429	-11.87991429	-2.191220123	9.688694165	9.961963646	2.82E-07	0.835415141	1.79E-06	3.33E-07	0.05332569	2.18E-06	7.466217746	-6.158165624	5.630393469	7.303717881	-4.227779743	5.42280105	80.69070556	2.73E-07

COLEC11	-0.150216959	0.033265781	-0.250072478	0.18348274	-0.099855519	-0.283338259	8	0.17413028	0.816496581	0.816496581	0.816496581	0.816496581	0.816496581	0.816496581	1	98	0	P_127119	P_127119	P_127119	chr2	3643479	3643539	+	COLEC11	collectin sub-family member 11 [Source:HGNC Symbol;Acc:HGNC:17213]	ENSG00000118004	protein_coding	3'UTR;exon	multiple Ref	1	98	2:3643479:3643539	ENSG00000118004	COLEC11	3.896916421	0.026556385	-1.128964067	0.250010862	-1.879433874	1.378974929	-0.750469807	-2.129444736	9.961963646	0.28536572	0.807659049	0.089719986	0.198074158	0.470326012	0.059179347	-6.344206736	-6.149243582	-5.398101481	-6.198429469	-6.005556989	-5.032881127	1.978108109	0.181515612

SEMA4A	-0.276096772	-0.009592429	-0.289130618	0.266504343	-0.013033846	-0.279538189	8	0.137428754	0.816496581	0.816496581	0.816496581	0.816496581	0.816496581	0.816496581	1	101	0	P_204270	P_204270	P_204270	chr1	156158071	156158131	+	SEMA4A	"sema domain, immunoglobulin domain (Ig), transmembrane domain (TM) and short cytoplasmic domain, (semaphorin) 4A [Source:HGNC Symbol;Acc:HGNC:10729]"	ENSG00000196189	protein_coding	exon	coding exon	1	101	1:156158071:156158131	ENSG00000196189	SEMA4A	6.748489436	0.017373704	-2.565433448	-0.089130844	-2.686541214	2.476302604	-0.121107766	-2.59741037	9.961963646	0.028188338	0.930744364	0.022906145	0.032833141	0.906013627	0.026685942	-4.24785899	-6.177901149	-4.089683976	-4.540872184	-6.288889955	-4.263087878	4.450029848	0.03137956

TRIM45	0.665193563	0.019531325	0.49807745	-0.645662237	-0.167116113	0.478546125	8	0.075130406	0.816496581	0.816496581	0.816496581	0.816496581	0.816496581	0.816496581	1	102	0	A_23_P160518	A_23_P160518	A_23_P160518	chr1	117112213	117112273	-	TRIM45	tripartite motif containing 45 [Source:HGNC Symbol;Acc:HGNC:19018]	ENSG00000134253	protein_coding	3'UTR	3'UTR	1	102	1:117112213:117112273	ENSG00000134253	TRIM45	9.152977273	0.006739588	9.923777032	0.291380629	7.430633484	-9.632396404	-2.493143548	7.139252856	9.961963646	1.76E-06	0.776737098	2.28E-05	2.30E-06	0.031901024	3.21E-05	5.661187734	-6.137526728	3.027120558	5.340049338	-3.751779546	2.659853238	50.62472063	2.45E-06

KHNYN	0.040547476	-0.091414131	-0.08784237	-0.131961607	-0.128389846	0.003571761	8	0.069947757	0.816496581	0.816496581	0.816496581	0.816496581	0.816496581	0.816496581	1	103	0	P_114590	P_114590	P_114590	chr14	24437225	24437285	+	KHNYN	KH and NYN domain containing [Source:HGNC Symbol;Acc:HGNC:20166]	ENSG00000100441	protein_coding	exon	coding exon	1	103	14:24437225:24437285	ENSG00000100441	KHNYN	8.332782772	0.00613578	0.633978566	-1.429302263	-1.373456126	-2.063280829	-2.007434692	0.055846137	9.961963646	0.540377971	0.18351745	0.199728377	0.06612919	0.072592471	0.956568568	-6.783375159	-5.20650112	-6.104333822	-5.213551681	-4.508097957	-7.069674987	2.103542688	0.163633048

VIRMA	0.016224643	0.068718618	0.041556283	0.052493975	0.02533164	-0.027162335	8	0.054926375	0.816496581	0.816496581	0.816496581	0.816496581	0.816496581	0.816496581	1	104	0	P_147603	P_147603	P_147603	chr8	94510998	94511058	-	KIAA1429	KIAA1429 [Source:HGNC Symbol;Acc:HGNC:24500]	ENSG00000164944	protein_coding	3'UTR	3'UTR	1	104	8:94510998:94511058	ENSG00000164944	VIRMA	8.13585286	0.004629425	0.292050046	1.236962525	0.74802966	0.944912479	0.455979614	-0.488932865	9.961963646	0.776239923	0.244466679	0.471731997	0.367057174	0.65818311	0.635474259	-6.952016207	-5.434070991	-6.749880307	-6.682118656	-6.18727286	-6.942334981	0.585791707	0.637959503

UEVLD	0.181473524	0.041422971	0.21438054	-0.140050553	0.032907016	0.172957569	8	0.075231872	0.816496581	0.816496581	0.816496581	0.816496581	0.816496581	0.816496581	1	105	0	A_23_P203445	A_23_P203445	A_23_P203445	chr11	18532139	18532199	-	UEVLD	UEV and lactate/malate dehyrogenase domains [Source:HGNC Symbol;Acc:HGNC:30866]	ENSG00000151116	protein_coding	3'UTR;exon	multiple Ref	1	105	11:18532139:18532199	ENSG00000151116	UEVLD	8.324165607	0.00675184	2.704878978	0.617413059	3.195360975	-2.087465919	0.490481997	2.577947916	9.961963646	0.022197481	0.550813319	0.009611645	0.063503109	0.634416164	0.027590605	-4.012913564	-5.985036384	-3.221598461	-5.175324987	-6.17029907	-4.295839591	4.858633823	0.02464712

SSBP2	0.614959241	0.076067748	0.830020413	-0.538891493	0.215061171	0.753952664	8	0.080935119	0.816496581	0.816496581	0.816496581	0.816496581	0.816496581	0.816496581	1	106	0	P_139870	P_139870	P_139870	chr5	81420231	81420291	-	SSBP2	single-stranded DNA binding protein 2 [Source:HGNC Symbol;Acc:HGNC:15831]	ENSG00000145687	protein_coding	3'UTR;exon	multiple Ref	1	106	5:81420231:81420291	ENSG00000145687	SSBP2	7.032685708	0.007467087	8.715982413	1.078128615	11.76410212	-7.637853799	3.04811971	10.68597351	9.961963646	5.66E-06	0.306389568	3.65E-07	1.80E-05	0.012342887	8.91E-07	4.480327755	-5.603898155	7.21863648	3.211340807	-2.85278768	6.328826873	66.22378062	6.96E-07

SNORD114-3	0.394148033	0.368872223	0.261242559	-0.025275809	-0.132905474	-0.107629664	8	0.616768978	0.816496581	0.816496581	0.816496581	0.816496581	0.816496581	0.816496581	1	107	0	P_115796	P_115796	P_115796	chr14	100953354	100953414	+	SNORD114-3	"small nucleolar RNA, C/D box 114-3 [Source:HGNC Symbol;Acc:HGNC:32991]"	ENSG00000201839	snoRNA	exon	non-coding exon	1	107	14:100953354:100953414	ENSG00000201839	SNORD114-3	3.219253076	0.307691815	0.870255989	0.814448468	0.576808414	-0.055807521	-0.293447575	-0.237640054	9.961963646	0.404642596	0.434425348	0.576869633	0.956598566	0.775202324	0.816976376	-6.600161066	-5.84401603	-6.868415607	-7.147910127	-6.25108708	-7.040614972	0.316513082	0.813266381

TTBK2	0.274225078	-0.041615318	0.113636433	-0.315840396	-0.160588645	0.155251751	8	0.060161801	0.816496581	0.816496581	0.816496581	0.816496581	0.816496581	0.816496581	1	108	0	P_116325	P_116325	P_116325	chr15	42745567	42745627	-	TTBK2	tau tubulin kinase 2 [Source:HGNC Symbol;Acc:HGNC:19141]	ENSG00000128881	protein_coding	3'UTR	3'UTR	1	108	15:42745567:42745627	ENSG00000128881	TTBK2	5.779259343	0.005113293	4.696804004	-0.712768478	1.946313798	-5.409572482	-2.750490206	2.659082276	9.961963646	0.000854433	0.492346423	0.080347827	0.000301312	0.020528633	0.024009826	-0.696862337	-5.921145252	-5.296190228	0.265924255	-3.336809056	-4.159018569	11.70925559	0.001322364

PRPF18	0.020142026	0.115863492	0.042360667	0.095721467	0.022218641	-0.073502825	8	0.043627668	0.816496581	0.816496581	0.816496581	0.816496581	0.816496581	0.816496581	1	109	0	P_207454	P_207454	P_207454	chr10	13597475	13597535	+	PRPF18	pre-mRNA processing factor 18 [Source:HGNC Symbol;Acc:HGNC:17351]	ENSG00000165630	protein_coding	exon	coding exon	1	109	10:13597475:13597535	ENSG00000165630	PRPF18	9.173252591	0.003735197	0.403637994	2.321857215	0.848890517	1.918219221	0.445252523	-1.472966698	9.961963646	0.695006879	0.042730246	0.415874751	0.084167605	0.665656344	0.171638935	-6.91017922	-3.934080362	-6.667268339	-5.438915108	-6.19230878	-5.999839353	2.053667558	0.170485839

DICER1	-0.057667247	0.064708243	-0.033706602	0.12237549	0.023960645	-0.098414845	8	0.051763217	0.816496581	0.816496581	0.816496581	0.816496581	0.816496581	0.816496581	1	110	0	P_222096	P_222096	P_222096	chr14	95129471	95129531	-	DICER1	"dicer 1, ribonuclease type III [Source:HGNC Symbol;Acc:HGNC:17098]"	ENSG00000100697	protein_coding	exon	coding exon	1	110	14:95129471:95129531	ENSG00000100697	DICER1	7.95141347	0.004358413	-1.069819884	1.200441648	-0.625311499	2.270261532	0.444508384	-1.825753148	9.961963646	0.309936165	0.257734991	0.545823458	0.046645369	0.666176203	0.097967075	-6.407426054	-5.474655005	-6.8376981	-4.881244393	-6.192653857	-5.502294965	1.943606481	0.186826877

BUB3	-0.205932911	0.046919536	-0.187111788	0.252852447	0.018821122	-0.234031324	8	0.097190192	0.816496581	0.816496581	0.816496581	0.816496581	0.816496581	0.816496581	1	111	0	P_107295	P_107295	P_107295	chr10	123165256	123165316	-	BUB3	BUB3 mitotic checkpoint protein [Source:HGNC Symbol;Acc:HGNC:1151]	ENSG00000154473	protein_coding	3'ext;3'UTR	multiple non-Ref	0	111	10:123165256:123165316	ENSG00000154473	BUB3	10.50282223	0.009792284	-2.548762654	0.580707383	-2.315819927	3.129470037	0.232942727	-2.896527309	9.961963646	0.0290046	0.574338658	0.043171294	0.010748063	0.820522206	0.01598763	-4.275797176	-6.007379701	-4.708246288	-3.429464774	-6.267904782	-3.755261917	5.072358256	0.021817367

C1orf226	0.033841327	0.128824751	0.197642199	0.094983424	0.163800872	0.068817448	8	0.116657966	0.816496581	0.816496581	0.816496581	0.816496581	0.816496581	0.816496581	1	112	0	A_23_P103511	A_23_P103511	A_23_P103511	chr1	162386692	162386752	+	C1orf226	chromosome 1 open reading frame 226 [Source:HGNC Symbol;Acc:HGNC:34351]	ENSG00000239887	protein_coding	3'UTR	3'UTR	1	112	1:162386692:162386752	ENSG00000239887	C1orf226	5.871107215	0.013135518	0.361634125	1.376643021	2.112037874	1.015008896	1.750403749	0.735394853	9.961963646	0.725177975	0.198771713	0.060936958	0.334118938	0.110724388	0.479054775	-6.927463276	-5.271007629	-5.037182414	-6.61364068	-4.882682575	-6.78372971	1.853598528	0.201554744

ZFPM1	0.289551226	0.040754232	0.215713747	-0.248796994	-0.073837479	0.174959515	8	0.076947172	0.816496581	0.816496581	0.816496581	0.816496581	0.816496581	0.816496581	1	113	0	A_23_P54573	A_23_P54573	A_23_P54573	chr16	88534697	88534757	+	ZFPM1	"zinc finger protein, FOG family member 1 [Source:HGNC Symbol;Acc:HGNC:19762]"	ENSG00000179588	protein_coding	exon	coding exon	1	113	16:88534697:88534757	ENSG00000179588	ZFPM1	13.50506862	0.006961463	4.250311577	0.598229838	3.166453999	-3.652081739	-1.083857578	2.568224161	9.961963646	0.001703182	0.563039679	0.010094234	0.00447499	0.303962309	0.02805394	-1.409515716	-5.996871915	-3.271050187	-2.536776952	-5.707166054	-4.31218539	8.259613664	0.004679676

OTUD3	-0.131045244	-0.023765178	0.02953476	0.107280066	0.160580004	0.053299938	8	0.156764995	0.816496581	0.816496581	0.816496581	0.816496581	0.816496581	0.816496581	1	114	0	P_200568	P_200568	P_200568	chr1	19906556	19906616	+	OTUD3	OTU deubiquitinase 3 [Source:HGNC Symbol;Acc:HGNC:29038]	ENSG00000169914	protein_coding	exon	coding exon	1	114	1:19906556:19906616	ENSG00000169914	OTUD3	6.906423978	0.021941961	-1.083500652	-0.196493859	0.244197584	0.887006793	1.327698236	0.440691443	9.961963646	0.3041131	0.848174281	0.812033608	0.395984364	0.213896974	0.668845647	-6.393039818	-6.161774399	-7.014652509	-6.735592415	-5.43585462	-6.96630965	0.669331427	0.590015361

OTUB2	-0.031364607	0.118783009	0.061766404	0.150147617	0.093131012	-0.057016605	8	0.067140768	0.816496581	0.816496581	0.816496581	0.816496581	0.816496581	0.816496581	1	115	0	P_222046	P_222046	P_222046	chr14	94051243	94051303	+	OTUB2;DDX24	"OTU deubiquitinase, ubiquitin aldehyde binding 2 [Source:HGNC Symbol;Acc:HGNC:20351];DEAD (Asp-Glu-Ala-Asp) box helicase 24 [Source:HGNC Symbol;Acc:HGNC:13266]"	ENSG00000089723	protein_coding	"3'ext;exon;exon,intron"	multiple non-Ref	0	115	14:94051243:94051303	ENSG00000089723	OTUB2	7.066229838	0.00582676	-0.503236269	1.90583985	0.991024518	2.409076119	1.494260788	-0.914815331	9.961963646	0.625737682	0.085904009	0.345133807	0.036828901	0.166090938	0.381898104	-6.861906998	-4.56120404	-6.535773877	-4.652790281	-5.228964461	-6.632504706	2.290158669	0.140694935

CATSPER3	0.320024724	0.073226627	0.333413153	-0.246798097	0.013388428	0.260186526	8	0.057463434	0.816496581	0.816496581	0.816496581	0.816496581	0.816496581	0.816496581	1	116	0	P_254894	P_254894	P_254894	chr5	134996391	134996451	+	CATSPER3	"cation channel, sperm associated 3 [Source:HGNC Symbol;Acc:HGNC:20819]"	ENSG00000152705	protein_coding	exon	coding exon	1	116	5:134996391:134996451	ENSG00000152705	CATSPER3	6.099893139	0.004858407	5.623182846	1.286671564	5.85843211	-4.336511283	0.235249263	4.571760546	9.961963646	0.000223606	0.227303736	0.000162191	0.001487587	0.818780555	0.001033593	0.692962278	-5.377433157	0.991283225	-1.399386164	-6.267333643	-0.954235006	17.89310044	0.000245334

SSTR3	0.295857497	-0.114845636	0.247832189	-0.410703133	-0.048025308	0.362677825	8	0.316955896	0.816496581	0.816496581	0.816496581	0.816496581	0.816496581	0.816496581	1	117	0	P_133237	P_133237	P_133237	chr22	37206258	37206318	-	SSTR3	somatostatin receptor 3 [Source:HGNC Symbol;Acc:HGNC:11332]	ENSG00000278195	protein_coding	3'UTR	3'UTR	1	117	22:37206258:37206318	ENSG00000278195	SSTR3	6.894549037	0.082882377	1.258627188	-0.48857251	1.05431951	-1.747199698	-0.204307678	1.54289202	9.961963646	0.236860931	0.635720513	0.316636124	0.11129911	0.842226641	0.15400213	-6.196631634	-6.057795925	-6.471846518	-5.694669052	-6.274531371	-5.906199927	1.401566554	0.299166047

PCDH15	0.0504045	-0.064767362	0.02090156	-0.115171862	-0.02950294	0.085668922	8	0.07143919	0.816496581	0.816496581	0.816496581	0.816496581	0.816496581	0.816496581	1	118	0	P_208275	P_208275	P_208275	chr10	54079356	54079416	-	PCDH15	protocadherin-related 15 [Source:HGNC Symbol;Acc:HGNC:14674]	ENSG00000150275	protein_coding	"3'UTR,exon;exon"	multiple Ref	1	118	10:54079356:54079416	ENSG00000150275	PCDH15	3.954501388	0.006305119	0.777442525	-0.998976305	0.322387117	-1.77641883	-0.455055408	1.321363422	9.961963646	0.454963678	0.341452581	0.753823268	0.106157273	0.658825439	0.215923134	-6.678231865	-5.681739303	-6.990657703	-5.651822069	-6.187711258	-6.193009697	1.135840761	0.381186896

PTGR2	0.230170443	0.058454735	0.177112196	-0.171715708	-0.053058247	0.11865746	8	0.039788953	0.816496581	0.816496581	0.816496581	0.816496581	0.816496581	0.816496581	1	119	0	P_115311	P_115311	P_115311	chr14	73885339	73885399	+	PTGR2	prostaglandin reductase 2 [Source:HGNC Symbol;Acc:HGNC:20149]	ENSG00000140043	protein_coding	3'UTR	3'UTR	1	119	14:73885339:73885399	ENSG00000140043	PTGR2	9.27576212	0.003478048	4.779994228	1.213940825	3.678123316	-3.566053403	-1.101870912	2.464182491	9.961963646	0.000753727	0.2527647	0.004286823	0.00516004	0.296426607	0.033520545	-0.567037901	-5.459757266	-2.400331387	-2.682785421	-5.688540811	-4.486275233	9.642273336	0.00271275

GUK1	-0.048279934	0.150345797	0.104264603	0.198625732	0.152544537	-0.046081195	8	0.077580016	0.816496581	0.816496581	0.816496581	0.816496581	0.816496581	0.816496581	1	120	0	P_104994	P_104994	P_104994	chr1	228140303	228140363	+	GUK1	guanylate kinase 1 [Source:HGNC Symbol;Acc:HGNC:4693]	ENSG00000143774	protein_coding	5'UTR;exon	multiple Ref	1	120	1:228140303:228140363	ENSG00000143774	GUK1	10.12360835	0.007039995	-0.704735452	2.194576585	1.521935829	2.899312037	2.226671281	-0.672640756	9.961963646	0.497119926	0.053024128	0.15911375	0.015911684	0.050222403	0.516473903	-6.733933949	-4.130287827	-5.910735717	-3.824033519	-4.172761194	-6.82969301	3.574271329	0.054865486

GPR107	0.011769985	0.065438645	-0.026887043	0.053668661	-0.038657028	-0.092325689	8	0.038404615	0.816496581	0.816496581	0.816496581	0.816496581	0.816496581	0.816496581	1	121	0	P_149921	P_149921	P_149921	chr9	130135413	130135473	+	GPR107	G protein-coupled receptor 107 [Source:HGNC Symbol;Acc:HGNC:17830]	ENSG00000148358	protein_coding	3'UTR	3'UTR	1	121	9:130135413:130135473	ENSG00000148358	GPR107	7.58370495	0.003391121	0.247542575	1.376284779	-0.565479743	1.128742204	-0.813022318	-1.941764522	9.961963646	0.809515623	0.198879059	0.584257867	0.285454971	0.43520525	0.080955138	-6.965025017	-5.271441006	-6.87525685	-6.494206779	-5.956649126	-5.326704717	1.330119696	0.319087506

DYRK4	0.171027154	0.167341277	0.00463045	-0.003685878	-0.166396704	-0.162710827	8	0.159723332	0.816496581	0.816496581	0.816496581	0.816496581	0.816496581	0.816496581	1	122	0	P_214108	P_214108	P_214108	chr12	4599106	4599166	+	DYRK4	dual-specificity tyrosine-(Y)-phosphorylation regulated kinase 4 [Source:HGNC Symbol;Acc:HGNC:3095]	ENSG00000010219	protein_coding	exon	coding exon	1	122	12:4599106:4599166	ENSG00000010219	DYRK4	6.568892814	0.022693844	1.390454129	1.360487872	0.03764565	-0.029966257	-1.352808479	-1.322842222	9.961963646	0.194670428	0.203661419	0.970713918	0.976685811	0.206021007	0.215448723	-6.03488174	-5.290476144	-7.046346713	-7.149122012	-5.405704633	-6.191194634	1.227767905	0.350271947

RAET1E-AS1	0.270532834	0.085611246	0.342164584	-0.184921588	0.07163175	0.256553338	8	0.075787276	0.816496581	0.816496581	0.816496581	0.816496581	0.816496581	0.816496581	1	123	0	A_24_P135841	A_24_P135841	A_24_P135841	chr6	149919446	149919506	+	RAET1E-AS1	RAET1E antisense RNA 1 [Source:HGNC Symbol;Acc:HGNC:48994];RAET1E antisense RNA 1 [Source:EntrezGene;Acc:100652739]	ENSG00000223701	antisense	exon	non-coding exon	1	123	6:149919446:149919506	ENSG00000223701	RAET1E-AS1	10.14984343	0.006819197	4.012351595	1.269725434	5.074743036	-2.742626161	1.062391441	3.805017602	9.961963646	0.002487597	0.233040154	0.000487212	0.020807115	0.313133468	0.003481046	-1.799452622	-5.396919247	-0.153119899	-4.091456118	-5.729047387	-2.208456526	11.09883628	0.001617893

HERC4	-0.089380752	0.067863863	-0.144468296	0.157244615	-0.055087544	-0.212332158	8	0.03141311	0.816496581	0.816496581	0.816496581	0.816496581	0.816496581	0.816496581	1	124	0	P_208455	P_208455	P_208455	chr10	67990210	67990270	-	HERC4	HECT and RLD domain containing E3 ubiquitin protein ligase 4 [Source:HGNC Symbol;Acc:HGNC:24521]	ENSG00000148634	protein_coding	3'UTR;exon	multiple Ref	1	124	10:67990210:67990270	ENSG00000148634	HERC4	7.088365192	0.002999125	-1.998905932	1.517703469	-3.230880554	3.516609401	-1.231974621	-4.748584023	9.961963646	0.073630633	0.16016393	0.009050889	0.005602234	0.246245564	0.000790183	-5.167849553	-5.094772717	-3.160851616	-2.766925363	-5.547148218	-0.675411637	8.861831878	0.0036636

KDM5B	0.561664424	-0.020004461	0.532182724	-0.581668885	-0.0294817	0.552187185	8	0.13455383	0.816496581	0.816496581	0.816496581	0.816496581	0.816496581	0.816496581	1	125	0	P_205818	P_205818	P_205818	chr1	202766997	202767057	-	KDM5B	lysine (K)-specific demethylase 5B [Source:HGNC Symbol;Acc:HGNC:18039]	ENSG00000117139	protein_coding	exon	coding exon	1	125	1:202766997:202767057	ENSG00000117139	KDM5B	9.002415239	0.016745772	5.315816243	-0.189330202	5.03678967	-5.505146446	-0.279026573	5.226119872	9.961963646	0.000344156	0.853635849	0.000515008	0.000263461	0.785931194	0.000391293	0.245593453	-6.163225804	-0.210796386	0.406374895	-6.255441117	0.055780201	18.5599701	0.000210763

UGGT1	0.087725362	-0.016738032	0.003695967	-0.104463394	-0.084029395	0.020434	8	0.067625905	0.816496581	0.816496581	0.816496581	0.816496581	0.816496581	0.816496581	1	126	0	P_236487	P_236487	P_236487	chr2	128177860	128177920	+	UGGT1	UDP-glucose glycoprotein glucosyltransferase 1 [Source:HGNC Symbol;Acc:HGNC:15663]	ENSG00000136731	protein_coding	3'UTR;exon	multiple Ref	1	126	2:128177860:128177920	ENSG00000136731	UGGT1	7.469056992	0.005879264	1.401229698	-0.267355157	0.059035372	-1.668584855	-1.342194326	0.326390529	9.961963646	0.191520563	0.794649131	0.954091389	0.126276657	0.20932006	0.750882387	-6.021173545	-6.144546007	-7.045217648	-5.808049873	-5.418496113	-7.013491551	1.121766738	0.386179879

MSH3	0.053733306	-0.069506903	-0.029733354	-0.123240209	-0.08346666	0.039773548	8	0.072294752	0.816496581	0.816496581	0.816496581	0.816496581	0.816496581	0.816496581	1	127	0	A_16_P37245564	A_16_P37245564	A_16_P37245564	chr5	80735737	80735797	+	MSH3	mutS homolog 3 [Source:HGNC Symbol;Acc:HGNC:7326]	ENSG00000113318	protein_coding	intron	intron sense	0	127	5:80735737:80735797	ENSG00000113318	MSH3	8.404322881	0.006403873	0.822371065	-1.063780916	-0.455059477	-1.886151981	-1.277430543	0.608721439	9.961963646	0.430109826	0.312533517	0.65882261	0.08873446	0.230417341	0.556334148	-6.641397175	-5.618356555	-6.935200511	-5.487747145	-5.495032694	-6.872650111	1.262490502	0.339326568

EVC	0.167125266	-0.061255139	-0.06253942	-0.228380405	-0.229664686	-0.001284282	8	0.133882723	0.816496581	0.816496581	0.816496581	0.816496581	0.816496581	0.816496581	1	128	0	P_248145	P_248145	P_248145	chr4	5745281	5745341	+	EVC	Ellis van Creveld syndrome [Source:HGNC Symbol;Acc:HGNC:3497]	ENSG00000072840	protein_coding	exon	coding exon	1	128	4:5745281:5745341	ENSG00000072840	EVC	5.80989263	0.016601102	1.588617279	-0.582263678	-0.594471478	-2.170880957	-2.183088757	-0.012207801	9.961963646	0.143347121	0.573330104	0.565452706	0.055188677	0.054063113	0.990500847	-5.772056424	-6.006458189	-6.857496363	-5.04219986	-4.240360036	-7.07129711	2.115813645	0.161996651

ERAP2	-0.134650206	0.103602671	0.028941473	0.238252877	0.163591679	-0.074661198	8	0.081054753	0.816496581	0.816496581	0.816496581	0.816496581	0.816496581	0.816496581	1	129	0	P_254076	P_254076	P_254076	chr5	96896444	96896504	+	ERAP2	endoplasmic reticulum aminopeptidase 2 [Source:HGNC Symbol;Acc:HGNC:29499]	ENSG00000164308	protein_coding	exon	coding exon	1	129	5:96896444:96896504	ENSG00000164308	ERAP2	4.869620311	0.00748265	-1.906447853	1.466860653	0.409768463	3.373308506	2.316216317	-1.057092189	9.961963646	0.085817952	0.173259102	0.690648212	0.007119457	0.0431422	0.315429637	-5.309889057	-5.159544059	-6.956193007	-3.011548769	-4.032643593	-6.493015667	3.96927433	0.042329158

NOL10	-0.089672414	0.039330487	-0.02552377	0.1290029	0.064148643	-0.064854257	8	0.071366366	0.816496581	0.816496581	0.816496581	0.816496581	0.816496581	0.816496581	1	130	0	A_24_P34505	A_24_P34505	A_24_P34505	chr2	10570786	10570846	-	NOL10	nucleolar protein 10 [Source:HGNC Symbol;Acc:HGNC:25862]	ENSG00000115761	protein_coding	3'UTR	3'UTR	1	130	2:10570786:10570846	ENSG00000115761	NOL10	7.905776131	0.006296768	-1.384030475	0.607038325	-0.393941399	1.991068801	0.990089077	-1.000979724	9.961963646	0.19656897	0.557406843	0.701924824	0.074596939	0.345568781	0.340529708	-6.0430198	-5.99148024	-6.963028054	-5.326612934	-5.800174378	-6.549996564	1.397636245	0.300224732

MRPL47	0.107399949	0.097363649	0.12687362	-0.010036299	0.019473671	0.02950997	8	0.039958121	0.816496581	0.816496581	0.816496581	0.816496581	0.816496581	0.816496581	1	131	0	P_247355	P_247355	P_247355	chr3	179593764	179593824	-	MRPL47	mitochondrial ribosomal protein L47 [Source:HGNC Symbol;Acc:HGNC:16652]	ENSG00000136522	protein_coding	exon	coding exon	1	131	3:179593764:179593824	ENSG00000136522	MRPL47	11.89758576	0.003488882	2.226929941	2.018828021	2.630715059	-0.20810192	0.403785118	0.611887038	9.961963646	0.050200415	0.071227174	0.025205807	0.839342306	0.694902139	0.554319781	-4.806267125	-4.395314205	-4.184011983	-7.125981875	-6.210690521	-6.870615836	2.756049973	0.098103461

ZDHHC2	0.089584068	0.033619504	0.102579688	-0.055964564	0.01299562	0.068960184	8	0.079815988	0.816496581	0.816496581	0.816496581	0.816496581	0.816496581	0.816496581	1	132	0	P_263850	P_263850	P_263850	chr8	17208032	17208092	+	ZDHHC2	"zinc finger, DHHC-type containing 2 [Source:HGNC Symbol;Acc:HGNC:18469]"	ENSG00000104219	protein_coding	exon	coding exon	1	132	8:17208032:17208092	ENSG00000104219	ZDHHC2	8.754824782	0.007322617	1.28216305	0.481175808	1.468161572	-0.800987242	0.185998522	0.986985764	9.961963646	0.22881845	0.640785435	0.172912816	0.441823885	0.856178708	0.34701469	-6.168586409	-6.061486463	-5.982331175	-6.809654954	-6.278317337	-6.563813435	0.94688126	0.454509503

ZNF414	0.168407316	0.080772986	0.058886703	-0.08763433	-0.109520613	-0.021886283	8	0.138653659	0.816496581	0.816496581	0.816496581	0.816496581	0.816496581	0.816496581	1	133	0	P_124481	P_124481	P_124481	chr19	8511337	8511397	-	ZNF414	zinc finger protein 414 [Source:HGNC Symbol;Acc:HGNC:20630]	ENSG00000133250	protein_coding	3'UTR	3'UTR	1	133	19:8511337:8511397	ENSG00000133250	ZNF414	9.953116604	0.017645277	1.552717148	0.744727743	0.542935994	-0.807989404	-1.009781154	-0.201791749	9.961963646	0.151655594	0.473638785	0.599110282	0.437965	0.336497227	0.844140572	-5.821280924	-5.897884585	-6.888491632	-6.803871916	-5.781201951	-7.049178758	0.828925702	0.507766017

NGDN	-0.092179793	0.022970142	-0.046299775	0.115149936	0.045880019	-0.069269917	8	0.057734456	0.816496581	0.816496581	0.816496581	0.816496581	0.816496581	0.816496581	1	134	0	P_114451	P_114451	P_114451	chr14	23478092	23478152	+	NGDN	"neuroguidin, EIF4E binding protein [Source:HGNC Symbol;Acc:HGNC:20271]"	ENSG00000129460	protein_coding	3'UTR;exon	multiple Ref	1	134	14:23478092:23478152	ENSG00000129460	NGDN	10.85261806	0.004883479	-1.61553614	0.402572991	-0.811446377	2.01810913	0.804089762	-1.214019368	9.961963646	0.1373842	0.695765276	0.436068152	0.071312599	0.440111318	0.252736009	-5.734710679	-6.097367599	-6.699005872	-5.284470934	-5.963843322	-6.320865628	1.603939182	0.249975001

PLA2G2F	0.166885877	-0.090327311	0.154787557	-0.257213188	-0.01209832	0.245114868	8	0.198011123	0.816496581	0.816496581	0.816496581	0.816496581	0.816496581	0.816496581	1	136	0	P_308233	P_308233	P_308233	chr1	20143710	20143770	+	PLA2G2F	"phospholipase A2, group IIF [Source:HGNC Symbol;Acc:HGNC:30040]"	ENSG00000158786	protein_coding	exon	non-coding exon	1	136	1:20143710:20143770	ENSG00000158786	PLA2G2F	5.323448307	0.033693171	1.113510388	-0.602689699	1.032786929	-1.716200087	-0.080723458	1.635476629	9.961963646	0.291634674	0.560183727	0.326124902	0.117001413	0.93726059	0.133110484	-6.360980974	-5.994151161	-6.493952494	-5.739715474	-6.293220507	-5.778198336	1.382738653	0.30427621

YIPF4	-0.058034917	0.006623221	-0.106160342	0.064658138	-0.048125424	-0.112783562	8	0.060951423	0.816496581	0.816496581	0.816496581	0.816496581	0.816496581	0.816496581	1	137	0	A_16_P35628839	A_16_P35628839	A_16_P35628839	chr2	32295197	32295257	+	YIPF4	"Yip1 domain family, member 4 [Source:HGNC Symbol;Acc:HGNC:28145]"	ENSG00000119820	protein_coding	intron	intron sense	0	137	2:32295197:32295257	ENSG00000119820	YIPF4	8.511041081	0.005190093	-0.986614401	0.112597125	-1.804763871	1.099211526	-0.818149469	-1.917360995	9.961963646	0.347188014	0.912586569	0.101377882	0.29752997	0.432405783	0.08428692	-6.491754276	-6.17540831	-5.509873964	-6.526176666	-5.952488494	-5.364057388	1.632861073	0.24372919

CUTA	0.194986214	0.056109466	0.229139901	-0.138876748	0.034153687	0.173030435	8	0.077523906	0.816496581	0.816496581	0.816496581	0.816496581	0.816496581	0.816496581	1	138	0	A_23_P214678	A_23_P214678	A_23_P214678	chr6	33416584	33416644	-	CUTA	cutA divalent cation tolerance homolog (E. coli) [Source:HGNC Symbol;Acc:HGNC:21101]	ENSG00000112514	protein_coding	"3'UTR;3'UTR,exon;exon"	multiple Ref	1	138	6:33416584:33416644	ENSG00000112514	CUTA	13.10310421	0.007033006	2.847600306	0.819428862	3.346384546	-2.028171444	0.49878424	2.526955684	9.961963646	0.017383408	0.431709107	0.00744896	0.070125679	0.628760294	0.030107817	-3.770669504	-5.840006832	-2.963528141	-5.268728563	-6.16603882	-4.381421637	5.121058474	0.021227919

DHX35	-0.126775588	-0.034273236	-0.184982733	0.092502352	-0.058207145	-0.150709497	8	0.093883804	0.816496581	0.816496581	0.816496581	0.816496581	0.816496581	0.816496581	1	139	0	P_240847	P_240847	P_240847	chr20	39021853	39021913	+	DHX35	DEAH (Asp-Glu-Ala-His) box polypeptide 35 [Source:HGNC Symbol;Acc:HGNC:15861]	ENSG00000101452	protein_coding	3'UTR;exon	multiple Ref	1	139	20:39021853:39021913	ENSG00000101452	DHX35	5.712346339	0.009284942	-1.611356616	-0.435623346	-2.35118728	1.17573327	-0.739830664	-1.915563934	9.961963646	0.138295318	0.6723976	0.040649337	0.267034946	0.476475754	0.084537268	-5.740533166	-6.083027528	-4.650199167	-6.441995187	-6.01353029	-5.366799554	2.318900464	0.13750712

AGTPBP1	-0.073604118	0.069402709	-0.043430383	0.143006827	0.030173735	-0.112833093	8	0.034414684	0.816496581	0.816496581	0.816496581	0.816496581	0.816496581	0.816496581	1	140	0	P_148986	P_148986	P_148986	chr9	85547108	85547168	-	AGTPBP1	ATP/GTP binding protein 1 [Source:HGNC Symbol;Acc:HGNC:17258]	ENSG00000135049	protein_coding	exon	coding exon	1	140	9:85547108:85547168	ENSG00000135049	AGTPBP1	7.429935993	0.003157798	-1.604189118	1.512620143	-0.94655775	3.116809261	0.657631368	-2.459177893	9.961963646	0.13987032	0.161433254	0.366258009	0.010981712	0.525678182	0.033808507	-5.75049772	-5.101309413	-6.578742639	-3.451188181	-6.071669935	-4.494608877	3.658658148	0.05185151

MAP9	-0.843608159	0.19676288	-0.710291233	1.040371039	0.133316926	-0.907054113	8	0.104336387	0.816496581	0.816496581	0.816496581	0.816496581	0.816496581	0.816496581	1	141	0	P_251342	P_251342	P_251342	chr4	155373202	155373262	-	MAP9	microtubule-associated protein 9 [Source:HGNC Symbol;Acc:HGNC:26118]	ENSG00000164114	protein_coding	exon	coding exon	1	141	4:155373202:155373262	ENSG00000164114	MAP9	10.81979408	0.010948801	-9.874227221	2.303061393	-8.313785201	12.17728861	1.56044202	-10.61684659	9.961963646	1.84E-06	0.044117987	8.60E-06	2.64E-07	0.149832799	9.46E-07	5.615465317	-3.963244978	4.032784886	7.535410739	-5.142518001	6.268725205	72.56037471	4.52E-07

R3HDM2	0.167498779	-0.014324154	0.118881059	-0.181822933	-0.04861772	0.133205213	8	0.090988422	0.816496581	0.816496581	0.816496581	0.816496581	0.816496581	0.816496581	1	142	0	P_111997	P_111997	P_111997	chr12	57278689	57278749	+	R3HDM2;RP11-123K3.4	R3H domain containing 2 [Source:HGNC Symbol;Acc:HGNC:29167];Uncharacterized protein [Source:UniProtKB/TrEMBL;Acc:V9GYY9]	ENSG00000179912	protein_coding	intron	intron antisense	0	142	12:57278689:57278749	ENSG00000179912	R3HDM2	6.638352144	0.008855086	2.180021986	-0.186431036	1.547254989	-2.366453022	-0.632766997	1.733686025	9.961963646	0.054343776	0.855848499	0.152956214	0.039605763	0.541137303	0.113753112	-4.881806409	-6.163797989	-5.876472326	-4.723341003	-6.088027991	-5.637813529	2.697901934	0.102491665

TYMP	0.310934506	0.27398758	0.267521192	-0.036946927	-0.043413314	-0.006466387	8	0.296917286	0.816496581	0.816496581	0.816496581	0.816496581	0.816496581	0.816496581	1	143	0	P_133817	P_133817	P_133817	chr22	50528652	50528712	-	TYMP	thymidine phosphorylase [Source:HGNC Symbol;Acc:HGNC:3148]	ENSG00000025708	protein_coding	exon	non-coding exon	1	143	22:50528652:50528712	ENSG00000025708	TYMP	8.435377833	0.07300387	1.409423823	1.24194843	1.212637176	-0.167475393	-0.196786647	-0.029311255	9.961963646	0.189154375	0.242698939	0.253241305	0.870346923	0.847951237	0.97719525	-6.010702093	-5.428462083	-6.298486352	-7.134296229	-6.276129214	-7.070909189	0.844505413	0.500376873

CLCNKA	0.097546694	0.121969979	0.090302317	0.024423285	-0.007244377	-0.031667662	8	0.291789275	0.816496581	0.816496581	0.816496581	0.816496581	0.816496581	0.816496581	1	145	0	A_24_P2361	A_24_P2361	A_24_P2361	chr1	16032490	16033193	+	CLCNKA	"chloride channel, voltage-sensitive Ka [Source:HGNC Symbol;Acc:HGNC:2026]"	ENSG00000186510	protein_coding	"exon,intron"	multiple non-Ref	0	145	1:16032490:16033193	ENSG00000186510	CLCNKA	4.859557434	0.070579534	0.44969576	0.56228848	0.416298773	0.11259272	-0.033396987	-0.145989707	9.961963646	0.662556154	0.586348284	0.686018283	0.912589972	0.974017688	0.886841349	-6.889121192	-6.018110977	-6.953297	-7.142684804	-6.296092047	-7.059747687	0.121134126	0.945543397

KRBOX4	0.198365073	-0.009409238	0.039514333	-0.207774311	-0.15885074	0.048923571	8	0.161786637	0.816496581	0.816496581	0.816496581	0.816496581	0.816496581	0.816496581	1	146	0	P_150762	P_150762	P_150762	chrX	46474558	46474618	+	KRBOX4	KRAB box domain containing 4 [Source:HGNC Symbol;Acc:HGNC:26007]	ENSG00000147121	protein_coding	3'UTR	3'UTR	1	146	X:46474558:46474618	ENSG00000147121	KRBOX4	6.239865941	0.023226569	1.59411008	-0.075614926	0.317546813	-1.669725006	-1.276563266	0.39316174	9.961963646	0.142112064	0.941222415	0.757384445	0.126047033	0.230711345	0.702482318	-5.764465956	-6.179074159	-6.992331929	-5.806426051	-5.496039623	-6.987586072	1.203318677	0.358214285

DCTN5	-0.181633272	0.150407236	-0.100219712	0.332040508	0.08141356	-0.250626949	8	0.071699462	0.816496581	0.816496581	0.816496581	0.816496581	0.816496581	0.816496581	1	147	0	A_23_P309967	A_23_P309967	A_23_P309967	chr16	23668999	23669059	+	DCTN5	dynactin 5 (p25) [Source:HGNC Symbol;Acc:HGNC:24594]	ENSG00000166847	protein_coding	3'UTR;exon	multiple Ref	1	147	16:23668999:23669059	ENSG00000166847	DCTN5	7.948020844	0.006335037	-2.794901198	2.314407264	-1.542141433	5.109308462	1.252759765	-3.856548697	9.961963646	0.019024783	0.043275133	0.154182697	0.000463287	0.238901489	0.003200549	-3.860280293	-3.945647402	-5.883420206	-0.183862625	-5.52348589	-2.122142555	9.682260288	0.00267257

QSOX2	-0.329895566	0.041648568	-0.253356377	0.371544134	0.076539188	-0.295004946	8	0.08304266	0.816496581	0.816496581	0.816496581	0.816496581	0.816496581	0.816496581	2	22	0	A_24_P263682	A_24_P263682	A_24_P263682	chr9	136215175	136215235	-	QSOX2	quiescin Q6 sulfhydryl oxidase 2 [Source:HGNC Symbol;Acc:HGNC:30249]	ENSG00000165661	protein_coding	exon	coding exon	1	192	9:136215175:136215235	ENSG00000165661	QSOX2	6.658012091	0.007744615	-4.591157518	0.579623245	-3.525961418	5.170780762	1.0651961	-4.105584662	9.961963646	0.001003372	0.575041804	0.005515678	0.000423784	0.311923366	0.00214256	-0.863104343	-6.008020281	-2.657831306	-0.090727976	-5.726208031	-1.708747508	13.09575604	0.000860165

NDE1	-0.119978541	-0.115375033	-0.075166128	0.004603508	0.044812414	0.040208906	8	0.086504405	0.816496581	0.816496581	0.816496581	0.816496581	0.816496581	0.816496581	2	23	0	A_23_P206901	A_23_P206901	A_23_P206901	chr16	15724976	15725036	+	NDE1	nudE neurodevelopment protein 1 [Source:HGNC Symbol;Acc:HGNC:17619]	ENSG00000072864	protein_coding	3'UTR;exon	multiple Ref	1	193	16:15724976:15725036	ENSG00000072864	NDE1	7.670544363	0.008215951	-1.621139156	-1.558936975	-1.015637894	0.062202181	0.605501262	0.543299081	9.961963646	0.136171153	0.150186416	0.33383363	0.951632137	0.558387473	0.598869508	-5.726891404	-5.041265683	-6.511295231	-7.147497589	-6.105297898	-6.912483379	1.125928628	0.384695959

NGEF	-0.154382411	-0.035154408	0.050552657	0.119228003	0.204935068	0.085707066	8	0.138366852	0.816496581	0.816496581	0.816496581	0.816496581	0.816496581	0.816496581	2	24	0	P_239708	P_239708	P_239708	chr2	232885280	232885340	-	NGEF	neuronal guanine nucleotide exchange factor [Source:HGNC Symbol;Acc:HGNC:7807]	ENSG00000066248	protein_coding	exon	coding exon	1	194	2:232885280:232885340	ENSG00000066248	NGEF	6.518795517	0.017581473	-1.42598785	-0.324711597	0.466940984	1.101276254	1.892928834	0.791652581	9.961963646	0.184447224	0.752115214	0.65058726	0.296673052	0.087750523	0.447003097	-5.989411117	-6.126808005	-6.929343915	-6.523962864	-4.677830701	-6.739413777	1.296602603	0.328943881

KLHL23	-0.195963341	-0.059225574	-0.142078627	0.136737768	0.053884715	-0.082853053	8	0.101348947	0.816496581	0.816496581	0.816496581	0.816496581	0.816496581	0.816496581	2	25	0	P_237451	P_237451	P_237451	chr2	169741473	169741533	+	KLHL23	kelch-like family member 23 [Source:HGNC Symbol;Acc:HGNC:27506]	ENSG00000213160	protein_coding	5'UTR;exon	multiple Ref	1	195	2:169741473:169741533	ENSG00000213160	KLHL23	8.561532304	0.010455346	-2.347206277	-0.709391038	-1.701786887	1.637815239	0.645419391	-0.992395848	9.961963646	0.040925868	0.494349946	0.119741871	0.132617124	0.533238096	0.344496879	-4.610278221	-5.923549971	-5.660205235	-5.851635649	-6.079776325	-6.55849069	2.165424338	0.155572768

LRRK2	0.101625031	0.071285492	0.073361637	-0.03033954	-0.028263394	0.002076145	8	0.104340505	0.816496581	0.816496581	0.816496581	0.816496581	0.816496581	0.816496581	2	26	0	P_215066	P_215066	P_215066	chr12	40277911	40277971	+	LRRK2	leucine-rich repeat kinase 2 [Source:HGNC Symbol;Acc:HGNC:18618]	ENSG00000188906	protein_coding	exon	coding exon	1	196	12:40277911:40277971	ENSG00000188906	LRRK2	3.48521032	0.010949491	1.189458656	0.834352955	0.858652961	-0.355105701	-0.330805695	0.024300006	9.961963646	0.261836388	0.423638037	0.410716269	0.729913583	0.747643884	0.981093171	-6.276847911	-5.827866211	-6.658790108	-7.081088579	-6.238807551	-7.071055948	0.514060769	0.681785193

MCM7	-0.384459782	-0.027164779	-0.305498649	0.357295003	0.078961134	-0.278333869	8	0.080950292	0.816496581	0.816496581	0.816496581	0.816496581	0.816496581	0.816496581	2	27	0	P_145390	P_145390	P_145390	chr7	100100361	100100421	-	MCM7	minichromosome maintenance complex component 7 [Source:HGNC Symbol;Acc:HGNC:6950]	ENSG00000166508	protein_coding	5'UTR;exon	multiple Ref	1	197	7:100100361:100100421	ENSG00000166508	MCM7	11.00466871	0.00746906	-5.448332124	-0.384962866	-4.329342568	5.063369258	1.118989556	-3.944379702	9.961963646	0.000285302	0.70835607	0.001504367	0.00049537	0.289399842	0.002775646	0.440158834	-6.104561943	-1.322537035	-0.253814962	-5.670620622	-1.975612797	15.17061725	0.00048086

TRAPPC9	-0.111914835	-0.098806728	0.008915854	0.013108106	0.120830688	0.107722582	8	0.050895164	0.816496581	0.816496581	0.816496581	0.816496581	0.816496581	0.816496581	2	28	0	P_148051	P_148051	P_148051	chr8	139730891	139730951	-	TRAPPC9	trafficking protein particle complex 9 [Source:HGNC Symbol;Acc:HGNC:30832]	ENSG00000167632	protein_coding	3'UTR;exon	multiple Ref	1	198	8:139730891:139730951	ENSG00000167632	TRAPPC9	7.280551375	0.00428685	-2.093457381	-1.848259668	0.166778248	0.245197713	2.260235629	2.015037916	9.961963646	0.062868058	0.094427498	0.87088114	0.81128051	0.047445573	0.071678638	-5.01970596	-4.644154389	-7.031951879	-7.116833811	-4.120425547	-5.213314975	2.842601235	0.091975678

CSTL1	0.378149636	0.018722036	0.19477013	-0.3594276	-0.183379506	0.176048094	8	0.109349742	0.816496581	0.816496581	0.816496581	0.816496581	0.816496581	0.816496581	2	29	0	P_130809	P_130809	P_130809	chr20	23444789	23444849	+	CSTL1	cystatin-like 1 [Source:HGNC Symbol;Acc:HGNC:15958]	ENSG00000125823	protein_coding	exon	coding exon	1	199	20:23444789:23444849	ENSG00000125823	CSTL1	3.831337573	0.011809101	4.26187686	0.211003803	2.195126564	-4.050873057	-2.066750296	1.984122761	9.961963646	0.001672435	0.837138012	0.052974872	0.002338445	0.06574622	0.075463354	-1.390733763	-6.158670849	-4.904295448	-1.868098444	-4.418602793	-5.261375712	7.940745008	0.005353867

ADGRV1	0.105606424	-0.278247778	-0.025811782	-0.383854202	-0.131418207	0.252435996	8	0.088440718	0.816496581	0.816496581	0.816496581	0.816496581	0.816496581	0.816496581	2	30	0	P_253825	P_253825	P_253825	chr5	90679569	90679629	+	GPR98	G protein-coupled receptor 98 [Source:HGNC Symbol;Acc:HGNC:17416]	ENSG00000164199	protein_coding	exon	coding exon	1	200	5:90679569:90679629	ENSG00000164199	ADGRV1	5.979359191	0.008487984	1.403891956	-3.698920976	-0.343132095	-5.102812932	-1.747024051	3.355788881	9.961963646	0.190749047	0.004142473	0.738629941	0.000467686	0.111330694	0.007332115	-6.017775847	-1.750919713	-6.983201584	-0.19373525	-4.887444342	-2.969580744	9.353796754	0.00302512

SAMHD1	0.277843728	-0.02507107	0.308591801	-0.302914798	0.030748073	0.333662871	8	0.04390958	0.816496581	0.816496581	0.816496581	0.816496581	0.816496581	0.816496581	2	31	0	P_240721	P_240721	P_240721	chr20	36898439	36898499	-	SAMHD1	SAM domain and HD domain 1 [Source:HGNC Symbol;Acc:HGNC:15925]	ENSG00000101347	protein_coding	exon	coding exon	1	201	20:36898439:36898499	ENSG00000101347	SAMHD1	8.183957745	0.003755014	5.553163141	-0.501086509	6.167713866	-6.054249649	0.614550725	6.668800374	9.961963646	0.000246397	0.627196313	0.000107566	0.000124869	0.552627998	5.68E-05	0.592261268	-6.051430312	1.418551296	1.18794376	-6.099643942	2.066377867	25.10565212	5.82E-05

KAT2B	-0.197187415	0.089501885	-0.093736141	0.286689299	0.103451273	-0.183238026	8	0.098629896	0.816496581	0.816496581	0.816496581	0.816496581	0.816496581	0.816496581	2	32	0	P_243479	P_243479	P_243479	chr3	20127488	20127548	+	KAT2B	K(lysine) acetyltransferase 2B [Source:HGNC Symbol;Acc:HGNC:8638]	ENSG00000114166	protein_coding	exon	coding exon	1	202	3:20127488:20127548	ENSG00000114166	KAT2B	7.283936364	0.010018683	-2.41278989	1.095147191	-1.146957652	3.507937081	1.265832238	-2.242104843	9.961963646	0.036596197	0.299222365	0.278199682	0.005683758	0.234374673	0.048926566	-4.502182899	-5.58655245	-6.372662375	-2.781698611	-5.508458436	-4.851673389	4.545233705	0.02963253

LAMA1	-0.538175969	0.438049522	-0.38704789	0.976225491	0.151128079	-0.825097412	8	0.057765231	0.816496581	0.816496581	0.816496581	0.816496581	0.816496581	0.816496581	2	34	0	P_230536	P_230536	P_230536	chr18	6985312	6985372	-	LAMA1	"laminin, alpha 1 [Source:HGNC Symbol;Acc:HGNC:6481]"	ENSG00000101680	protein_coding	exon	coding exon	1	204	18:6985312:6985372	ENSG00000101680	LAMA1	5.112443732	0.004886334	-9.4292763	7.674980318	-6.78139067	17.10425662	2.64788563	-14.45637099	9.961963646	2.79E-06	1.73E-05	4.94E-05	1.03E-08	0.024474967	5.20E-08	5.194782651	3.203279721	2.226728927	10.6636874	-3.502918739	9.107631813	117.0595648	4.57E-08

PSMD9	-0.006711115	0.013326868	-0.005663261	0.020037982	0.001047854	-0.018990129	8	0.087363182	0.816496581	0.816496581	0.816496581	0.816496581	0.816496581	0.816496581	2	35	0	P_217845	P_217845	P_217845	chr12	121903047	121903107	+	PSMD9	"proteasome (prosome, macropain) 26S subunit, non-ATPase, 9 [Source:HGNC Symbol;Acc:HGNC:9567]"	ENSG00000110801	protein_coding	"3'UTR;3'UTR,exon;exon"	multiple Ref	1	205	12:121903047:121903107	ENSG00000110801	PSMD9	9.088688665	0.008335858	-0.090025416	0.178771618	-0.075969115	0.268797035	0.014056301	-0.254740733	9.961963646	0.930051324	0.861700442	0.940947675	0.793570488	0.989062586	0.804104885	-6.993939527	-6.165267487	-7.043969752	-7.110244405	-6.296580168	-7.036043119	0.030529573	0.992364787

ROBO3	0.115796027	0.044790063	0.039907046	-0.071005964	-0.075888981	-0.004883017	8	0.120901916	0.816496581	0.816496581	0.816496581	0.816496581	0.816496581	0.816496581	2	37	0	P_110409	P_110409	P_110409	chr11	124879010	124879070	+	ROBO3	"roundabout, axon guidance receptor, homolog 3 (Drosophila) [Source:HGNC Symbol;Acc:HGNC:13433]"	ENSG00000154134	protein_coding	exon	non-coding exon	1	207	11:124879010:124879070	ENSG00000154134	ROBO3	7.059971393	0.013945152	1.200957587	0.464532048	0.413888719	-0.736425539	-0.787068868	-0.050643329	9.961963646	0.257543596	0.652253015	0.687725415	0.47845474	0.449560876	0.960610782	-6.263744889	-6.069594396	-6.95437094	-6.860873294	-5.977359152	-7.069977576	0.498959624	0.691312705

0.006295032	-0.02984685	-0.01824157	-0.036141882	-0.024536602	0.011605281	8	0.021687432	0.816496581	0.816496581	0.816496581	0.816496581	0.816496581	0.816496581	2	38	0	P_123984	P_123984	P_123984	chr19	1457697	1457757	-	CTB-25B13.12	NA	ENSG00000267317	antisense	exon	non-coding exon	1	208	19:1457697:1457757	ENSG00000267317	5.934555228	0.002584396	0.15165754	-0.719059079	-0.439469025	-0.870716619	-0.591126565	0.279590054	9.961963646	0.88248433	0.488628242	0.669701593	0.404402764	0.567605108	0.785511078	-6.985823976	-5.916639031	-6.942665538	-6.750117561	-6.114119013	-7.028841512	0.319821273	0.810972152

NSDHL	-0.080782724	0.183978215	-0.085708687	0.264760939	-0.004925963	-0.269686902	8	0.070857202	0.816496581	0.816496581	0.816496581	0.816496581	0.816496581	0.816496581	2	39	0	P_151872	P_151872	P_151872	chrX	152869148	152869208	+	NSDHL	NAD(P) dependent steroid dehydrogenase-like [Source:HGNC Symbol;Acc:HGNC:13398]	ENSG00000147383	protein_coding	3'UTR	3'UTR	1	209	X:152869148:152869208	ENSG00000147383	NSDHL	8.213456412	0.006238614	-1.252622052	2.852777879	-1.329004349	4.105399931	-0.076382297	-4.181782227	9.961963646	0.238949554	0.017230057	0.213481195	0.002143191	0.940627185	0.001898035	-6.20372728	-3.093301869	-6.159699031	-1.777900269	-6.293583033	-1.583608431	7.636850272	0.006107507

TACC2	-0.020934335	-0.021622845	0.014230113	-0.00068851	0.035164448	0.035852958	8	0.069927095	0.816496581	0.816496581	0.816496581	0.816496581	0.816496581	0.816496581	2	40	0	A_23_P127186	A_23_P127186	A_23_P127186	chr10	122254244	122254304	+	TACC2	"transforming, acidic coiled-coil containing protein 2 [Source:HGNC Symbol;Acc:HGNC:11523]"	ENSG00000138162	protein_coding	"3'UTR;3'UTR,exon;exon"	multiple Ref	1	210	10:122254244:122254304	ENSG00000138162	TACC2	5.562244747	0.006133459	-0.327379946	-0.338147158	0.222536494	-0.010767213	0.54991644	0.560683652	9.961963646	0.750156211	0.742270372	0.828392484	0.991621749	0.594490243	0.587401031	-6.940189251	-6.122166444	-7.02014336	-7.149549664	-6.138311042	-6.902312016	0.147988967	0.928614791

WDR83	0.116215444	-0.049105246	0.011443445	-0.16532069	-0.104772	0.06054869	8	0.043541062	0.816496581	0.816496581	0.816496581	0.816496581	0.816496581	0.816496581	2	41	0	P_232394	P_232394	P_232394	chr19	12672854	12672914	+	WDR83	WD repeat domain 83 [Source:HGNC Symbol;Acc:HGNC:32672]	ENSG00000123154	protein_coding	"3'UTR;3'UTR,exon;exon"	multiple Ref	1	211	19:12672854:12672914	ENSG00000123154	WDR83	11.07627133	0.003729134	2.330802508	-0.984848703	0.229508305	-3.315651211	-2.101294203	1.214357008	9.961963646	0.042084821	0.348012983	0.823117411	0.007844369	0.062046548	0.252612699	-4.637187552	-5.695130718	-7.018431104	-3.110230868	-4.366035331	-6.320475855	3.889814098	0.044551353

VPS41	0.175230382	0.109673808	0.215904619	-0.065556574	0.040674237	0.106230811	8	0.055804528	0.816496581	0.816496581	0.816496581	0.816496581	0.816496581	0.816496581	2	42	0	P_261028	P_261028	P_261028	chr7	38752186	38752246	-	VPS41	vacuolar protein sorting 41 homolog (S. cerevisiae) [Source:HGNC Symbol;Acc:HGNC:12713]	ENSG00000006715	protein_coding	exon	coding exon	1	212	7:38752186:38752246	ENSG00000006715	VPS41	7.599873595	0.004707512	3.127946364	1.957730081	3.854001011	-1.170216283	0.726054646	1.89627093	9.961963646	0.010775911	0.07884255	0.003213845	0.26914752	0.484513992	0.087269028	-3.292236242	-4.485506707	-2.104985927	-6.448208944	-6.02370337	-5.3961646	5.660414043	0.015821664

CHD8	0.082080111	-0.110208871	0.005109125	-0.192288982	-0.076970986	0.115317996	8	0.073233593	0.816496581	0.816496581	0.816496581	0.816496581	0.816496581	0.816496581	2	43	0	P_220114	P_220114	P_220114	chr14	21397822	21397882	-	CHD8	chromodomain helicase DNA binding protein 8 [Source:HGNC Symbol;Acc:HGNC:20153]	ENSG00000100888	protein_coding	exon	coding exon	1	213	14:21397822:21397882	ENSG00000100888	CHD8	8.333657368	0.006513593	1.245584657	-1.672445088	0.077532151	-2.918029745	-1.168052507	1.749977239	9.961963646	0.241416283	0.125500731	0.939735345	0.0154106	0.269979688	0.110800736	-6.212011289	-4.889744533	-7.043838803	-3.791997736	-5.618095094	-5.614098387	2.882704922	0.089290321

CHD6	0.261358303	-0.122762594	0.127897487	-0.384120898	-0.133460817	0.250660081	8	0.062406957	0.816496581	0.816496581	0.816496581	0.816496581	0.816496581	0.816496581	2	44	0	P_131291	P_131291	P_131291	chr20	41404871	41404931	-	CHD6	chromodomain helicase DNA binding protein 6 [Source:HGNC Symbol;Acc:HGNC:19057]	ENSG00000124177	protein_coding	exon	coding exon	1	214	20:41404871:41404931	ENSG00000124177	CHD6	7.685085234	0.005334283	4.382721874	-2.058608052	2.144715148	-6.441329926	-2.238006726	4.2033232	9.961963646	0.00138406	0.066648337	0.057677501	7.56E-05	0.049267465	0.001834372	-1.195474002	-4.336015892	-4.985140205	1.713325278	-4.155112225	-1.5483538	15.36887541	0.000456409

TIGD6	-0.073367579	0.073240768	-0.068295497	0.146608346	0.005072082	-0.141536264	8	0.057887707	0.816496581	0.816496581	0.816496581	0.816496581	0.816496581	0.816496581	2	45	0	P_140853	P_140853	P_140853	chr5	150000110	150000170	-	TIGD6	tigger transposable element derived 6 [Source:HGNC Symbol;Acc:HGNC:18332]	ENSG00000164296	protein_coding	5'UTR;exon	multiple Ref	1	215	5:150000110:150000170	ENSG00000164296	TIGD6	7.276386784	0.004897709	-1.283965422	1.28174617	-1.195201722	2.565711592	0.0887637	-2.476947891	9.961963646	0.228211918	0.228958927	0.259685284	0.028174915	0.931028814	0.032796935	-6.166423374	-5.383115537	-6.318479295	-4.390951068	-6.292496237	-4.465000476	2.907662238	0.087665952

FRYL	-0.135208588	-0.057570215	-0.209432606	0.077638373	-0.074224018	-0.151862391	8	0.055007588	0.816496581	0.816496581	0.816496581	0.816496581	0.816496581	0.816496581	2	48	0	P_248893	P_248893	P_248893	chr4	48510834	48510894	-	FRYL	FRY-like [Source:HGNC Symbol;Acc:HGNC:29127]	ENSG00000075539	protein_coding	exon	coding exon	1	218	4:48510834:48510894	ENSG00000075539	FRYL	6.417063634	0.004636594	-2.431926033	-1.035485299	-3.766954546	1.396440734	-1.335028513	-2.731469247	9.961963646	0.035419834	0.32492421	0.003704658	0.192915069	0.211572221	0.02120872	-4.47049798	-5.646421977	-2.250822825	-6.176240554	-5.427093027	-4.036379298	5.394952115	0.018245966

RAP1GDS1	0.039130937	0.115505153	0.112535321	0.076374216	0.073404383	-0.002969833	8	0.060338255	0.816496581	0.816496581	0.816496581	0.816496581	0.816496581	0.816496581	2	49	0	P_138239	P_138239	P_138239	chr4	98442020	98442080	+	RAP1GDS1	"RAP1, GTP-GDP dissociation stimulator 1 [Source:HGNC Symbol;Acc:HGNC:9859]"	ENSG00000138698	protein_coding	exon	coding exon	1	219	4:98442020:98442080	ENSG00000138698	RAP1GDS1	7.786272728	0.005130369	0.669100872	1.975025491	1.924244248	1.30592462	1.255143376	-0.050781243	9.961963646	0.518635952	0.076612439	0.083334405	0.220928076	0.2380708	0.960503617	-6.759430141	-4.460088264	-5.329999017	-6.289149353	-5.520754132	-7.069969937	1.889090056	0.195591931

WWOX	-0.011587012	0.016200276	0.037485472	0.027787287	0.049072484	0.021285196	8	0.100139127	0.816496581	0.816496581	0.816496581	0.816496581	0.816496581	0.816496581	2	50	0	P_310261	P_310261	P_310261	chr16	78300680	78300740	-	WWOX	WW domain containing oxidoreductase [Source:HGNC Symbol;Acc:HGNC:12799]	ENSG00000186153	protein_coding	intron	intron antisense	0	220	16:78300680:78300740	ENSG00000186153	WWOX	5.621263894	0.01025959	-0.140104495	0.195885832	0.453256046	0.335990327	0.593360541	0.257370214	9.961963646	0.891369661	0.848637518	0.660076841	0.743847552	0.566167074	0.802131034	-6.987659403	-6.161899667	-6.936076805	-7.088226845	-6.112761039	-7.035312318	0.132441205	0.938518892

LRRC59	-0.236707962	0.287034516	-0.020196221	0.523742478	0.216511741	-0.307230736	8	0.086540704	0.816496581	0.816496581	0.816496581	0.816496581	0.816496581	0.816496581	2	51	0	P_122013	P_122013	P_122013	chr17	50392397	50392457	-	LRRC59	leucine rich repeat containing 59 [Source:HGNC Symbol;Acc:HGNC:28817]	ENSG00000108829	protein_coding	exon	coding exon	1	221	17:50392397:50392457	ENSG00000108829	LRRC59	9.226435333	0.008220995	-3.197395088	3.877194251	-0.272805766	7.074589339	2.924589322	-4.150000017	9.961963646	0.00957859	0.003094897	0.790574034	3.46E-05	0.015238803	0.00199625	-3.173598236	-1.475766695	-7.006629634	2.529042795	-3.053845329	-1.635723149	16.85932179	0.000313438

XPC	-0.190750652	0.034560695	0.060519779	0.225311347	0.251270431	0.025959084	8	0.097534706	0.816496581	0.816496581	0.816496581	0.816496581	0.816496581	0.816496581	2	52	0	P_243281	P_243281	P_243281	chr3	14156396	14156456	-	XPC	"xeroderma pigmentosum, complementation group C [Source:HGNC Symbol;Acc:HGNC:12816]"	ENSG00000154767	protein_coding	3'UTR;exon	multiple Ref	1	222	3:14156396:14156456	ENSG00000154767	XPC	9.457362799	0.009846157	-2.354389357	0.426574333	0.746981053	2.780963691	3.10137041	0.320406719	9.961963646	0.040428238	0.678760593	0.472337015	0.019484412	0.011273669	0.755279588	-4.598478229	-6.087062058	-6.750690627	-4.026171544	-2.766158839	-7.015583792	3.956335589	0.04268185

NIBAN2	-0.038609341	0.022094033	-0.161762015	0.060703374	-0.123152674	-0.183856048	8	0.101647585	0.816496581	0.816496581	0.816496581	0.816496581	0.816496581	0.816496581	2	53	0	P_149710	P_149710	P_149710	chr9	127507996	127508056	-	FAM129B;LRSAM1	"family with sequence similarity 129, member B [Source:HGNC Symbol;Acc:HGNC:25282];leucine rich repeat and sterile alpha motif containing 1 [Source:HGNC Symbol;Acc:HGNC:25135]"	ENSG00000136830	protein_coding	3'ext;intron	multiple non-Ref	0	223	9:127507996:127508056	ENSG00000136830	NIBAN2	8.254810438	0.010504029	-0.461381368	0.26402355	-1.933055016	0.725404918	-1.471673648	-2.197078566	9.961963646	0.654434709	0.79714318	0.082129836	0.484895196	0.171980933	0.052800408	-6.883431024	-6.145472441	-5.316522355	-6.869232314	-5.257939925	-4.924480264	1.92307879	0.190072641

AK9	0.208694999	0.072655666	0.195500548	-0.136039334	-0.013194452	0.122844882	8	0.118775185	0.816496581	0.816496581	0.816496581	0.816496581	0.816496581	0.816496581	2	54	0	P_258660	P_258660	P_258660	chr6	109662591	109662651	-	AK9	adenylate kinase 9 [Source:HGNC Symbol;Acc:HGNC:33814]	ENSG00000155085	protein_coding	exon	coding exon	1	224	6:109662591:109662651	ENSG00000155085	AK9	5.108271113	0.013535811	2.196926481	0.76484418	2.058028854	-1.432082301	-0.138897627	1.293184674	9.961963646	0.052813981	0.462098119	0.066712955	0.182740677	0.892298785	0.225130136	-4.854646487	-5.882778118	-5.122531451	-6.130384165	-6.286434061	-6.227322779	2.23157237	0.147465139

ZDHHC13	-0.005417017	0.05823493	0.04052394	0.063651947	0.045940957	-0.01771099	8	0.045984072	0.816496581	0.816496581	0.816496581	0.816496581	0.816496581	0.816496581	2	55	0	P_211236	P_211236	P_211236	chr11	19166307	19166367	+	ZDHHC13	"zinc finger, DHHC-type containing 13 [Source:HGNC Symbol;Acc:HGNC:18413]"	ENSG00000177054	protein_coding	exon	coding exon	1	225	11:19166307:19166367	ENSG00000177054	ZDHHC13	7.453119029	0.003904771	-0.106171523	1.141383039	0.794254207	1.247554562	0.90042573	-0.347128832	9.961963646	0.917553808	0.280404532	0.445555617	0.240723717	0.389142249	0.735716062	-6.992212567	-5.538373698	-6.713158463	-6.359122928	-5.882653128	-7.005947551	0.738754115	0.552746227

GPX5	-0.426011863	0.032234011	-0.14402046	0.458245874	0.281991403	-0.176254471	8	0.204074035	0.816496581	0.816496581	0.816496581	0.816496581	0.816496581	0.816496581	2	56	0	P_141941	P_141941	P_141941	chr6	28534171	28534231	+	GPX5	glutathione peroxidase 5 (epididymal androgen-related protein) [Source:HGNC Symbol;Acc:HGNC:4557]	ENSG00000224586	protein_coding	3'UTR;exon	multiple Ref	1	226	6:28534171:28534231	ENSG00000224586	GPX5	3.986422329	0.035650863	-2.763327122	0.209086	-0.934189111	2.972413122	1.829138011	-1.143275111	9.961963646	0.02008207	0.838594627	0.372296657	0.014043574	0.097427032	0.279654653	-3.913884255	-6.159093614	-6.590402947	-3.698845055	-4.770462887	-6.400734054	3.673411107	0.051345023

SNX9	0.051507979	0.127787088	0.091220787	0.076279109	0.039712807	-0.036566302	8	0.075074489	0.816496581	0.816496581	0.816496581	0.816496581	0.816496581	0.816496581	2	58	0	P_260016	P_260016	P_260016	chr6	157875075	157875135	+	SNX9	sorting nexin 9 [Source:HGNC Symbol;Acc:HGNC:14973]	ENSG00000130340	protein_coding	exon	coding exon	1	228	6:157875075:157875135	ENSG00000130340	SNX9	6.690408154	0.006732843	0.76881332	1.907363037	1.361570712	1.138549717	0.592757392	-0.545792325	9.961963646	0.459842632	0.085688569	0.203330537	0.281530403	0.566555128	0.597217532	-6.685097487	-4.558994439	-6.119259555	-6.483444302	-6.113128147	-6.911043145	1.338088087	0.316793295

ZNF519	-0.147319095	-0.368419953	-0.106837542	-0.221100858	0.040481553	0.261582411	8	0.210764021	0.816496581	0.816496581	0.816496581	0.816496581	0.816496581	0.816496581	2	59	0	P_123263	P_123263	P_123263	chr18	14104813	14104873	-	ZNF519	zinc finger protein 519 [Source:HGNC Symbol;Acc:HGNC:30574]	ENSG00000175322	protein_coding	3'UTR;exon	multiple Ref	1	229	18:14104813:14104873	ENSG00000175322	ZNF519	4.887969684	0.037879549	-0.927048115	-2.318389358	-0.672306205	-1.391341243	0.25474191	1.646083154	9.961963646	0.375815382	0.042983047	0.516678005	0.194409461	0.804104001	0.130885984	-6.548675203	-3.93946585	-6.805757469	-6.182738479	-6.262284961	-5.763257628	1.899442784	0.193891324

INO80C	0.086822148	0.07932186	-0.016028423	-0.007500288	-0.102850571	-0.095350283	8	0.097132808	0.816496581	0.816496581	0.816496581	0.816496581	0.816496581	0.816496581	2	60	0	P_123448	P_123448	P_123448	chr18	35497802	35497862	-	INO80C;RP11-322E11.6	INO80 complex subunit C [Source:HGNC Symbol;Acc:HGNC:26994];Uncharacterized protein [Source:UniProtKB/TrEMBL;Acc:K7ENP7]	ENSG00000153391	protein_coding	exon	coding exon	1	230	18:35497802:35497862	ENSG00000153391	INO80C	10.29460691	0.009783329	1.075060322	0.982189297	-0.198469191	-0.092871025	-1.273529512	-1.180658488	9.961963646	0.30769564	0.349258228	0.846669758	0.927847196	0.231742205	0.265160126	-6.401932448	-5.697634188	-7.025652041	-7.144898378	-5.499558072	-6.358978938	0.864049573	0.491263079

GALNT16	0.20800879	-0.006808708	0.377655771	-0.214817497	0.169646982	0.384464479	8	0.112731623	0.816496581	0.816496581	0.816496581	0.816496581	0.816496581	0.816496581	2	61	0	P_221370	P_221370	P_221370	chr14	69347107	69347167	+	GALNT16	polypeptide N-acetylgalactosaminyltransferase 16 [Source:HGNC Symbol;Acc:HGNC:23233]	ENSG00000100626	protein_coding	exon	coding exon	1	231	14:69347107:69347167	ENSG00000100626	GALNT16	4.868367717	0.012412237	2.286663616	-0.074848876	4.151611635	-2.361512492	1.86494802	4.226460512	9.961963646	0.045364414	0.941816659	0.001991143	0.039940601	0.091879705	0.001768491	-4.709322244	-6.179134887	-1.612109419	-4.731496746	-4.718642324	-1.510547628	8.23134214	0.004735171

UHMK1	0.034509847	-0.110197295	-0.049606284	-0.144707142	-0.084116131	0.060591011	8	0.101696679	0.816496581	0.816496581	0.816496581	0.816496581	0.816496581	0.816496581	2	62	0	P_204585	P_204585	P_204585	chr1	162500187	162500247	+	UHMK1	U2AF homology motif (UHM) kinase 1 [Source:HGNC Symbol;Acc:HGNC:19683]	ENSG00000152332	protein_coding	exon	coding exon	1	232	1:162500187:162500247	ENSG00000152332	UHMK1	9.205792343	0.010512046	0.412235161	-1.316354714	-0.592568679	-1.728589875	-1.00480384	0.723786035	9.961963646	0.688897759	0.217536428	0.566676573	0.114691246	0.338773264	0.485845831	-6.906415036	-5.342866763	-6.858687421	-5.721763198	-5.786025953	-6.792511009	1.129230856	0.383522995

GLOD4	-0.018837726	0.113792893	0.083496307	0.132630619	0.102334033	-0.030296586	8	0.036967931	0.816496581	0.816496581	0.816496581	0.816496581	0.816496581	0.816496581	2	63	0	P_119944	P_119944	P_119944	chr17	760172	760232	-	GLOD4	glyoxalase domain containing 4 [Source:HGNC Symbol;Acc:HGNC:14111]	ENSG00000167699	protein_coding	3'UTR;exon	multiple Ref	1	233	17:760172:760232	ENSG00000167699	GLOD4	9.636308635	0.003304161	-0.401368727	2.424544647	1.779026071	2.825913374	2.180394798	-0.645518577	9.961963646	0.696623265	0.035869124	0.105709182	0.018040849	0.054309583	0.533176439	-6.911159994	-3.773746031	-5.54787704	-3.949494415	-4.244524636	-6.848401961	3.726841578	0.049559268

UBE2J2	-0.059454542	-0.063241789	-0.030706714	-0.003787247	0.028747828	0.032535075	8	0.065645649	0.816496581	0.816496581	0.816496581	0.816496581	0.816496581	0.816496581	2	64	0	P_100044	P_100044	P_100044	chr1	1253980	1254040	-	UBE2J2	"ubiquitin-conjugating enzyme E2, J2 [Source:HGNC Symbol;Acc:HGNC:19268]"	ENSG00000160087	protein_coding	3'UTR	3'UTR	1	234	1:1253980:1254040	ENSG00000160087	UBE2J2	8.34449321	0.005667328	-0.967256139	-1.028870242	-0.499562472	-0.061614103	0.467693667	0.52930777	9.961963646	0.356311273	0.327873634	0.628231408	0.952088782	0.6500672	0.608184084	-6.510574872	-5.65289645	-6.912523468	-7.147537393	-6.181642534	-6.920450126	0.45773691	0.717824331

HHAT	0.058599238	0.37723208	0.280397004	0.318632842	0.221797766	-0.096835076	8	0.190318255	0.816496581	0.816496581	0.816496581	0.816496581	0.816496581	0.816496581	2	65	0	A_16_P00246127	A_16_P00246127	A_16_P00246127	chr1	210648559	210648619	+	HHAT	hedgehog acyltransferase [Source:HGNC Symbol;Acc:HGNC:18270]	ENSG00000054392	protein_coding	intron	intron sense	0	235	1:210648559:210648619	ENSG00000054392	HHAT	4.563924829	0.031294153	0.405700926	2.611696153	1.941276512	2.205995227	1.535575586	-0.670419641	9.961963646	0.693538855	0.026040736	0.08102054	0.052010477	0.155770159	0.517829858	-6.909282981	-3.478081974	-5.303922123	-4.985604449	-5.175263867	-6.831251608	3.071329058	0.077838908

BBS7	0.043137683	-0.093409995	0.017270018	-0.136547678	-0.025867665	0.110680013	8	0.169367167	0.816496581	0.816496581	0.816496581	0.816496581	0.816496581	0.816496581	2	67	0	P_250679	P_250679	P_250679	chr4	121833230	121833290	-	BBS7	Bardet-Biedl syndrome 7 [Source:HGNC Symbol;Acc:HGNC:18758]	ENSG00000138686	protein_coding	exon	coding exon	1	237	4:121833230:121833290	ENSG00000138686	BBS7	7.477220941	0.025242494	0.3325342	-0.720066906	0.133128885	-1.052601106	-0.199405315	0.853195791	9.961963646	0.746377431	0.488034176	0.896742268	0.317385615	0.845956979	0.413594407	-6.938353383	-5.915913779	-7.03745039	-6.575279191	-6.275579622	-6.687657036	0.420412212	0.742424436

COPB2	-0.111857865	0.017146142	0.056986372	0.129004007	0.168844237	0.03984023	8	0.06742834	0.816496581	0.816496581	0.816496581	0.816496581	0.816496581	0.816496581	2	68	0	P_246412	P_246412	P_246412	chr3	139366567	139366627	-	COPB2	"coatomer protein complex, subunit beta 2 (beta prime) [Source:HGNC Symbol;Acc:HGNC:2232]"	ENSG00000184432	protein_coding	exon	coding exon	1	238	3:139366567:139366627	ENSG00000184432	COPB2	9.911125429	0.005857836	-1.78996085	0.274374301	0.911901678	2.064335151	2.701862529	0.637527377	9.961963646	0.103848692	0.789402555	0.383357158	0.066012588	0.022312526	0.538157359	-5.484340831	-6.142556719	-6.611088782	-5.21188888	-3.415618402	-6.853779452	2.679797945	0.103904917

CCNL1	-0.032592835	-0.083739616	-0.010734814	-0.051146781	0.021858021	0.073004802	8	0.08523351	0.816496581	0.816496581	0.816496581	0.816496581	0.816496581	0.816496581	2	69	0	P_136396	P_136396	P_136396	chr3	157151878	157151938	-	CCNL1	cyclin L1 [Source:HGNC Symbol;Acc:HGNC:20569]	ENSG00000163660	protein_coding	exon	non-coding exon	1	239	3:157151878:157151938	ENSG00000163660	CCNL1	8.734856845	0.008040675	-0.445165507	-1.143747953	-0.146620226	-0.698582445	0.298545282	0.997127727	9.961963646	0.665717124	0.2794675	0.886356453	0.500795476	0.771421466	0.342305768	-6.891289462	-5.535868603	-7.035393346	-6.889101941	-6.24949641	-6.55381568	0.516539937	0.680230809

ACSL5	-0.111523557	-0.152151812	-0.194935033	-0.040628255	-0.083411476	-0.042783221	8	0.122106145	0.816496581	0.816496581	0.816496581	0.816496581	0.816496581	0.816496581	2	70	0	P_209913	P_209913	P_209913	chr10	112426799	112426859	+	ACSL5	acyl-CoA synthetase long-chain family member 5 [Source:HGNC Symbol;Acc:HGNC:16526]	ENSG00000197142	protein_coding	exon	coding exon	1	240	10:112426799:112426859	ENSG00000197142	ACSL5	4.464159905	0.014180155	-1.147022001	-1.564884413	-2.00491069	-0.417862412	-0.857888689	-0.440026276	9.961963646	0.278174311	0.148793314	0.072898257	0.684911688	0.41111852	0.66931134	-6.324380332	-5.033477956	-5.205609815	-7.054957426	-5.919473987	-6.966623533	1.481399115	0.278551082

C8orf37	0.083313913	0.036925727	-0.011448035	-0.046388186	-0.094761948	-0.048373762	8	0.046398454	0.816496581	0.816496581	0.816496581	0.816496581	0.816496581	0.816496581	2	73	0	P_265486	P_265486	P_265486	chr8	95259839	95259899	-	C8orf37	chromosome 8 open reading frame 37 [Source:HGNC Symbol;Acc:HGNC:27232]	ENSG00000156172	protein_coding	exon	coding exon	1	243	8:95259839:95259899	ENSG00000156172	C8orf37	6.641572433	0.003935513	1.626531521	0.720898309	-0.223499156	-0.905633212	-1.850030677	-0.944397465	9.961963646	0.135012736	0.487544444	0.827663566	0.386509549	0.094154051	0.367307592	-5.719351331	-5.915314801	-7.019910044	-6.718703345	-4.74028615	-6.604874113	1.391653771	0.301844332

RFX4	-0.137191914	-0.335661566	-0.167439688	-0.198469652	-0.030247774	0.168221879	8	0.117320472	0.816496581	0.816496581	0.816496581	0.816496581	0.816496581	0.816496581	2	74	0	P_217268	P_217268	P_217268	chr12	106639332	106639392	+	RFX4	"regulatory factor X, 4 (influences HLA class II expression) [Source:HGNC Symbol;Acc:HGNC:9985]"	ENSG00000111783	protein_coding	5'UTR;exon	multiple Ref	1	244	12:106639332:106639392	ENSG00000111783	RFX4	4.284700606	0.013260001	-1.459158142	-3.570059583	-1.780870142	-2.110901442	-0.321712001	1.789189441	9.961963646	0.175321689	0.005125835	0.105393299	0.061053425	0.754319613	0.103978952	-5.946286332	-1.952046773	-5.545163436	-5.138119481	-6.241929175	-5.556544211	4.30109479	0.034365691

RORB	0.123993723	0.06919276	0.145444068	-0.054800963	0.021450345	0.076251308	8	0.08920609	0.816496581	0.816496581	0.816496581	0.816496581	0.816496581	0.816496581	2	75	0	P_267554	P_267554	P_267554	chr9	74662546	74662606	+	RORB	RAR-related orphan receptor B [Source:HGNC Symbol;Acc:HGNC:10259]	ENSG00000198963	protein_coding	exon	coding exon	1	245	9:74662546:74662606	ENSG00000198963	RORB	3.987190074	0.008597172	1.637825074	0.913962696	1.9211613	-0.723862378	0.283336226	1.007198604	9.961963646	0.132615053	0.382324666	0.083759778	0.485800975	0.78271987	0.337676758	-5.703513096	-5.759947662	-5.33470802	-6.870393279	-6.254162531	-6.543805438	1.471227451	0.281085894

ECPAS	-0.157104791	-0.081241718	-0.176888424	0.075863073	-0.019783633	-0.095646706	8	0.044737917	0.816496581	0.816496581	0.816496581	0.816496581	0.816496581	0.816496581	2	76	0	P_268496	P_268496	P_268496	chr9	111370434	111370494	-	KIAA0368	KIAA0368 [Source:HGNC Symbol;Acc:HGNC:29020]	ENSG00000136813	protein_coding	exon	coding exon	1	246	9:111370434:111370494	ENSG00000136813	ECPAS	8.693747039	0.003813983	-3.115628803	-1.611147794	-3.507968571	1.504481008	-0.392339768	-1.896820777	9.961963646	0.01100376	0.138340981	0.00568346	0.163483892	0.703070268	0.087190047	-3.313281703	-4.97231561	-2.688382865	-6.034935779	-6.215458614	-5.395329604	5.1040177	0.021431985

TCP1	-0.150344722	-0.022629926	-0.039655023	0.127714796	0.110689699	-0.017025098	8	0.076913543	0.816496581	0.816496581	0.816496581	0.816496581	0.816496581	0.816496581	2	78	0	P_143963	P_143963	P_143963	chr6	159789404	159789464	-	TCP1	t-complex 1 [Source:HGNC Symbol;Acc:HGNC:11655]	ENSG00000120438	protein_coding	5'UTR;exon	multiple Ref	1	248	6:159789404:159789464	ENSG00000120438	TCP1	11.40583003	0.006957308	-2.207563461	-0.332283012	-0.582268397	1.875280449	1.625295064	-0.249985385	9.961963646	0.05187273	0.746561424	0.573327048	0.090334358	0.135277579	0.807678205	-4.837519868	-6.124214934	-6.865073085	-5.504214943	-5.055675929	-7.037345939	1.92392307	0.189937861

NME7	0.187172672	-0.027875228	0.136192055	-0.2150479	-0.050980616	0.164067283	8	0.090636806	0.816496581	0.816496581	0.816496581	0.816496581	0.816496581	0.816496581	2	79	0	P_204740	P_204740	P_204740	chr1	169235130	169235190	-	NME7	NME/NM23 family member 7 [Source:HGNC Symbol;Acc:HGNC:20461]	ENSG00000143156	protein_coding	3'UTR;exon	multiple Ref	1	249	1:169235130:169235190	ENSG00000143156	NME7	8.405237252	0.008803801	2.443165847	-0.363855492	1.777715601	-2.807021339	-0.665450247	2.141571093	9.961963646	0.034746297	0.72356939	0.105934188	0.018633966	0.520871292	0.057983703	-4.451858829	-6.112773652	-5.549804557	-3.981737454	-6.066406811	-5.013534726	3.695040417	0.050613056

CCDC136	-0.2878069	0.189531631	-0.309651184	0.477338531	-0.021844284	-0.499182815	8	0.134892646	0.816496581	0.816496581	0.816496581	0.816496581	0.816496581	0.816496581	2	80	0	P_262818	P_262818	P_262818	chr7	128805427	128805487	+	CCDC136	coiled-coil domain containing 136 [Source:HGNC Symbol;Acc:HGNC:22225]	ENSG00000128596	protein_coding	exon	coding exon	1	250	7:128805427:128805487	ENSG00000128596	CCDC136	6.396807404	0.016819085	-2.717976197	1.789889198	-2.924268135	4.507865395	-0.206291938	-4.714157333	9.961963646	0.021704817	0.103860785	0.01524717	0.001140159	0.840717923	0.000832303	-3.990747218	-4.727035118	-3.685255317	-1.122964766	-6.27409991	-0.729363244	10.28818804	0.002142945

UQCR10	-0.050822012	0.12821737	-0.280117756	0.179039382	-0.229295744	-0.408335126	8	0.324648216	0.816496581	0.816496581	0.816496581	0.816496581	0.816496581	0.816496581	2	81	0	P_132993	P_132993	P_132993	chr22	29767488	29767548	+	UQCR10	"ubiquinol-cytochrome c reductase, complex III subunit X [Source:HGNC Symbol;Acc:HGNC:30863]"	ENSG00000184076	protein_coding	exon	coding exon	1	251	22:29767488:29767548	ENSG00000184076	UQCR10	13.33133297	0.086845792	-0.211214192	0.532866115	-1.16415787	0.744080307	-0.952943678	-1.697023985	9.961963646	0.836978256	0.605808073	0.271482667	0.47401324	0.363168033	0.120660151	-6.974070995	-6.034580103	-6.353538623	-6.855000788	-5.835129594	-5.690746731	1.004244807	0.430763655

LMBR1	-0.017318657	-0.046812271	-0.12164915	-0.029493615	-0.104330493	-0.074836879	8	0.053663026	0.816496581	0.816496581	0.816496581	0.816496581	0.816496581	0.816496581	2	83	0	P_263519	P_263519	P_263519	chr7	156734186	156734246	-	LMBR1	limb development membrane protein 1 [Source:HGNC Symbol;Acc:HGNC:13243]	ENSG00000105983	protein_coding	3'UTR;exon	multiple Ref	1	253	7:156734186:156734246	ENSG00000105983	LMBR1	7.266196183	0.004519256	-0.315519594	-0.852848415	-2.216262578	-0.537328821	-1.900742983	-1.363414163	9.961963646	0.758877717	0.413778082	0.051115004	0.602835003	0.086628588	0.202768278	-6.944307082	-5.812558238	-4.870207819	-6.993978868	-4.666384543	-6.140849512	1.916527862	0.191122205

HDAC2	-0.038872986	-0.179377981	-0.054875257	-0.140504995	-0.016002271	0.124502724	8	0.124276697	0.816496581	0.816496581	0.816496581	0.816496581	0.816496581	0.816496581	2	84	0	P_258821	P_258821	P_258821	chr6	113959911	113959971	-	HDAC2	histone deacetylase 2 [Source:HGNC Symbol;Acc:HGNC:4853]	ENSG00000196591	protein_coding	exon	coding exon	1	254	6:113959911:113959971	ENSG00000196591	HDAC2	9.413745693	0.014609618	-0.393889226	-1.817587505	-0.556035811	-1.42369828	-0.162146586	1.261551694	9.961963646	0.701962125	0.099281253	0.590455653	0.185091854	0.874432066	0.23584918	-6.914354714	-4.687863294	-6.880862672	-6.141239791	-6.282720185	-6.265213737	1.235444978	0.347818535

SLC17A8	-0.003877365	0.08335609	0.122198585	0.087233456	0.126075951	0.038842495	8	0.171624564	0.816496581	0.816496581	0.816496581	0.816496581	0.816496581	0.816496581	2	85	0	P_216921	P_216921	P_216921	chr12	100402406	100402466	+	SLC17A8	"solute carrier family 17 (vesicular glutamate transporter), member 8 [Source:HGNC Symbol;Acc:HGNC:20151]"	ENSG00000179520	protein_coding	exon	coding exon	1	255	12:100402406:100402466	ENSG00000179520	SLC17A8	3.830368208	0.025860648	-0.029529944	0.634838514	0.930662271	0.664368458	0.960192215	0.295823757	9.961963646	0.977025159	0.539839386	0.374031548	0.521534791	0.359683571	0.773439188	-6.997884486	-5.973986771	-6.593704288	-6.913460025	-5.828394773	-7.023780821	0.453481742	0.720601344

PHKA2	0.04933703	-0.046530362	-0.077716968	-0.095867392	-0.127053998	-0.031186606	8	0.066266176	0.816496581	0.816496581	0.816496581	0.816496581	0.816496581	0.816496581	2	86	0	P_150583	P_150583	P_150583	chrX	18893136	18893196	+	PHKA2;PHKA2-AS1	"phosphorylase kinase, alpha 2 (liver) [Source:HGNC Symbol;Acc:HGNC:8926];PHKA2 antisense RNA 1 [Source:HGNC Symbol;Acc:HGNC:44110]"	ENSG00000044446	protein_coding;antisense	3'UTR;exon;intron	multiple non-Ref	0	256	X:18893136:18893196	ENSG00000044446	PHKA2	6.71943368	0.005733062	0.798041204	-0.752642516	-1.257095188	-1.55068372	-2.055136392	-0.504452672	9.961963646	0.443454153	0.469076376	0.237392349	0.152138643	0.067036543	0.624913091	-6.661569658	-5.891983604	-6.246544712	-5.972482566	-4.436204032	-6.934117561	1.611051153	0.248422292

TEX11	0.153772365	0.01408189	0.108195605	-0.139690475	-0.04557676	0.094113715	8	0.106196276	0.816496581	0.816496581	0.816496581	0.816496581	0.816496581	0.816496581	2	87	0	P_270552	P_270552	P_270552	chrX	70629618	70629678	-	TEX11	testis expressed 11 [Source:HGNC Symbol;Acc:HGNC:11733]	ENSG00000120498	protein_coding	exon	coding exon	1	257	X:70629618:70629678	ENSG00000120498	TEX11	3.502939863	0.011263251	1.774565525	0.162507981	1.248600106	-1.612057545	-0.525965419	1.086092125	9.961963646	0.106476845	0.874154891	0.24035679	0.138142144	0.610420237	0.303019582	-5.506987687	-6.168184621	-6.256575247	-5.887763981	-6.151617004	-6.46259631	1.464909854	0.282673454

TSNAX	0.270954006	0.078076148	0.203402454	-0.192877858	-0.067551552	0.125326307	8	0.033948218	0.816496581	0.816496581	0.816496581	0.816496581	0.816496581	0.816496581	2	89	0	P_105093	P_105093	P_105093	chr1	231565330	231565390	+	TSNAX	translin-associated factor X [Source:HGNC Symbol;Acc:HGNC:12380]	ENSG00000116918	protein_coding	3'UTR;exon	multiple Ref	1	259	1:231565330:231565390	ENSG00000116918	TSNAX	11.22058226	0.003132189	5.929488009	1.708598405	4.451207171	-4.220889603	-1.478280837	2.742608766	9.961963646	0.000147419	0.118439602	0.001244411	0.001784122	0.170239565	0.020807735	1.125111462	-4.840265186	-1.12624404	-1.587963901	-5.249492549	-4.017465625	14.23575133	0.000619721

ADCY10	-0.055322931	-0.008843836	-0.023712444	0.046479094	0.031610487	-0.014868608	8	0.107113922	0.816496581	0.816496581	0.816496581	0.816496581	0.816496581	0.816496581	2	90	0	P_204699	P_204699	P_204699	chr1	167904992	167905052	-	ADCY10	adenylate cyclase 10 (soluble) [Source:HGNC Symbol;Acc:HGNC:21285]	ENSG00000143199	protein_coding	5'UTR;exon	multiple Ref	1	260	1:167904992:167905052	ENSG00000143199	ADCY10	4.084182508	0.011420444	-0.634029292	-0.101354922	-0.271756827	0.53267437	0.362272465	-0.170401905	9.961963646	0.540346193	0.921279683	0.791357754	0.605935985	0.724715583	0.868105109	-6.783341432	-6.176674791	-7.006939258	-6.996625887	-6.227350721	-7.055538852	0.155022978	0.92405359

FXR1	0.047719848	-0.061355631	0.094784151	-0.109075479	0.047064303	0.156139782	8	0.036447166	0.816496581	0.816496581	0.816496581	0.816496581	0.816496581	0.816496581	2	91	0	A_24_P913005	A_24_P913005	A_24_P913005	chr3	180971321	180971381	+	FXR1	"fragile X mental retardation, autosomal homolog 1 [Source:HGNC Symbol;Acc:HGNC:4023]"	ENSG00000114416	protein_coding	exon	non-coding exon	1	261	3:180971321:180971381	ENSG00000114416	FXR1	8.301251218	0.003273458	1.021506831	-1.313398896	2.028980851	-2.334905727	1.00747402	3.342379747	9.961963646	0.331180287	0.218493181	0.07003102	0.041791954	0.337550821	0.007499299	-6.457061956	-5.346333342	-5.16807333	-4.775336928	-5.783440416	-2.992479005	4.087257864	0.03926815

NPAS3	0.008606586	0.18473923	0.107075051	0.176132643	0.098468465	-0.077664178	8	0.094097967	0.816496581	0.816496581	0.816496581	0.816496581	0.816496581	0.816496581	2	93	0	P_114659	P_114659	P_114659	chr14	33804089	33804149	+	NPAS3	neuronal PAS domain protein 3 [Source:HGNC Symbol;Acc:HGNC:19311]	ENSG00000151322	protein_coding	3'UTR	3'UTR	1	263	14:33804089:33804149	ENSG00000151322	NPAS3	4.568712398	0.009317272	0.109202397	2.344014914	1.358593501	2.234812517	1.249391104	-0.985421413	9.961963646	0.915210347	0.041148875	0.204241367	0.049534765	0.240079496	0.347745245	-6.991856772	-3.899622519	-6.122984471	-4.93892952	-5.527340215	-6.565348084	2.479754643	0.121155367

BTBD7	0.059836706	-0.097314393	-0.021086683	-0.157151099	-0.080923388	0.076227711	8	0.066488546	0.816496581	0.816496581	0.816496581	0.816496581	0.816496581	0.816496581	2	94	0	P_222003	P_222003	P_222003	chr14	93245975	93246035	-	BTBD7	BTB (POZ) domain containing 7 [Source:HGNC Symbol;Acc:HGNC:18269]	ENSG00000011114	protein_coding	3'UTR;exon	multiple Ref	1	264	14:93245975:93246035	ENSG00000011114	BTBD7	6.434496848	0.005756769	0.965881661	-1.570844963	-0.340380368	-2.536726625	-1.306262029	1.230464595	9.961963646	0.356965634	0.147408583	0.740638658	0.029608456	0.220817676	0.246786162	-6.511899471	-5.02565567	-6.984216738	-4.439676033	-5.461285248	-6.301788322	2.195280942	0.151850364

SNX12	0.053862848	0.119274508	0.045584536	0.06541166	-0.008278312	-0.073689972	8	0.072228966	0.816496581	0.816496581	0.816496581	0.816496581	0.816496581	0.816496581	2	95	0	P_151145	P_151145	P_151145	chrX	71060937	71060997	-	SNX12	sorting nexin 12 [Source:HGNC Symbol;Acc:HGNC:14976]	ENSG00000147164	protein_coding	3'UTR;exon	multiple Ref	1	265	X:71060937:71060997	ENSG00000147164	SNX12	7.92043966	0.006396238	0.824845526	1.826547385	0.698073005	1.001701859	-0.126772521	-1.128474379	9.961963646	0.428767876	0.09784011	0.50110054	0.340197511	0.901642715	0.28556274	-6.639315904	-4.675130622	-6.78734875	-6.626949186	-6.288144279	-6.416981571	1.132813954	0.3822547

LRRC7	0.045421259	-0.038560817	-0.053841324	-0.083982076	-0.099262583	-0.015280507	8	0.075481944	0.816496581	0.816496581	0.816496581	0.816496581	0.816496581	0.816496581	2	96	0	P_202213	P_202213	P_202213	chr1	69986326	69986386	+	LRRC7	leucine rich repeat containing 7 [Source:HGNC Symbol;Acc:HGNC:18531]	ENSG00000033122	protein_coding	5'UTR;exon	multiple Ref	1	266	1:69986326:69986386	ENSG00000033122	LRRC7	3.706056781	0.006782106	0.675495566	-0.573468491	-0.80071702	-1.248964056	-1.476212586	-0.22724853	9.961963646	0.514734218	0.579042536	0.441973254	0.24022917	0.170783019	0.824826223	-6.754943436	-6.011635758	-6.707869482	-6.357460445	-5.252139385	-7.043239766	0.869510804	0.488747099

ZNF699	0.087214576	-0.139610326	-0.103726695	-0.226824903	-0.190941271	0.035883632	8	0.106532323	0.816496581	0.816496581	0.816496581	0.816496581	0.816496581	0.816496581	2	97	0	P_124499	P_124499	P_124499	chr19	9295309	9295369	-	ZNF699	zinc finger protein 699 [Source:HGNC Symbol;Acc:HGNC:24750]	ENSG00000196110	protein_coding	3'UTR	3'UTR	1	267	19:9295309:9295369	ENSG00000196110	ZNF699	6.048601996	0.011320659	1.00391941	-1.607042339	-1.193988973	-2.610961749	-2.197908383	0.413053366	9.961963646	0.339178891	0.139241446	0.260138346	0.026073523	0.052726408	0.688317558	-6.474671264	-4.977784374	-6.319861903	-4.314669966	-4.217420981	-6.97896673	2.805764088	0.094526783

SH3GL2	0.127130867	0.114756478	0.045098536	-0.012374389	-0.082032331	-0.069657942	8	0.135763918	0.816496581	0.816496581	0.816496581	0.816496581	0.816496581	0.816496581	2	98	0	P_266944	P_266944	P_266944	chr9	17787453	17787513	+	SH3GL2	SH3-domain GRB2-like 2 [Source:HGNC Symbol;Acc:HGNC:10831]	ENSG00000107295	protein_coding	exon	coding exon	1	268	9:17787453:17787513	ENSG00000107295	SH3GL2	4.021610004	0.017008458	1.193889611	1.077681368	0.42352164	-0.116208243	-0.770367971	-0.654159728	9.961963646	0.260175494	0.306579681	0.680913176	0.909796753	0.458961137	0.527820864	-6.271809994	-5.604351141	-6.950042389	-7.142233014	-5.99037358	-6.842517858	0.633506038	0.610161719

GART	-0.28887642	0.015485969	-0.175961807	0.30436239	0.112914613	-0.191447776	8	0.038702121	0.816496581	0.816496581	0.816496581	0.816496581	0.816496581	0.816496581	2	99	0	P_241664	P_241664	P_241664	chr21	33532347	33532407	-	GART	"phosphoribosylglycinamide formyltransferase, phosphoribosylglycinamide synthetase, phosphoribosylaminoimidazole synthetase [Source:HGNC Symbol;Acc:HGNC:4163]"	ENSG00000159131	protein_coding	exon	coding exon	1	269	21:33532347:33532407	ENSG00000159131	GART	9.248855991	0.003409543	-6.059121567	0.324814921	-3.690761535	6.383936488	2.368360032	-4.015576455	9.961963646	0.000124068	0.752039323	0.004198492	8.13E-05	0.039477254	0.002474736	1.303926989	-6.126773011	-2.379020928	1.636735123	-3.950369983	-1.857407406	18.82146803	0.000198823

NSMF	-0.035582289	0.029416555	0.079322002	0.064998844	0.114904291	0.049905447	8	0.086323051	0.816496581	0.816496581	0.816496581	0.816496581	0.816496581	0.816496581	2	100	0	P_150329	P_150329	P_150329	chr9	137448232	137448292	+	NSMF	NMDA receptor synaptonuclear signaling and neuronal migration factor [Source:HGNC Symbol;Acc:HGNC:29843]	ENSG00000165802	protein_coding	"3'ext;3'UTR;3'UTR,intron"	multiple non-Ref	0	270	9:137448232:137448292	ENSG00000165802	NSMF	7.771761248	0.008190781	-0.481522813	0.398084063	1.073437211	0.879606876	1.554960024	0.675353149	9.961963646	0.640547386	0.698965736	0.308388266	0.399793221	0.151124355	0.514820921	-6.873295663	-6.099231048	-6.451915311	-6.742219091	-5.14976397	-6.827783294	0.865052233	0.490800155

SGCB	0.200036593	-0.186710645	-0.020126123	-0.386747237	-0.220162715	0.166584522	8	0.060608845	0.816496581	0.816496581	0.816496581	0.816496581	0.816496581	0.816496581	2	101	0	P_248965	P_248965	P_248965	chr4	52028729	52028789	-	SGCB	"sarcoglycan, beta (43kDa dystrophin-associated glycoprotein) [Source:HGNC Symbol;Acc:HGNC:10806]"	ENSG00000163069	protein_coding	exon	coding exon	1	271	4:52028729:52028789	ENSG00000163069	SGCB	9.483771598	0.00515665	3.411703166	-3.184423854	-0.343258977	-6.59612702	-3.754962143	2.841164877	9.961963646	0.006675381	0.009791393	0.738537369	6.22E-05	0.003778178	0.017575936	-2.808211761	-2.562643369	-6.983154582	1.917659549	-1.715489472	-3.849735414	14.59649179	0.00056107

SLC43A1	-0.272366601	0.304706695	-0.064550168	0.577073296	0.207816433	-0.369256863	8	0.064991645	0.816496581	0.816496581	0.816496581	0.816496581	0.816496581	0.816496581	2	102	0	P_108575	P_108575	P_108575	chr11	57487104	57487164	-	SLC43A1	"solute carrier family 43 (amino acid system L transporter), member 1 [Source:HGNC Symbol;Acc:HGNC:9225]"	ENSG00000149150	protein_coding	exon	coding exon	1	272	11:57487104:57487164	ENSG00000149150	SLC43A1	7.967813777	0.005598717	-4.458155547	4.987505221	-1.056571131	9.445660767	3.401584415	-6.044076352	9.961963646	0.0012311	0.000553654	0.3156561	2.75E-06	0.006789565	0.000126561	-1.074529605	0.134982667	-6.469513887	5.157853995	-2.279747481	1.232001557	30.53147409	2.46E-05

GDI2	0.008820903	-0.027067583	-0.047235404	-0.035888486	-0.056056307	-0.020167821	8	0.036886816	0.816496581	0.816496581	0.816496581	0.816496581	0.816496581	0.816496581	2	103	0	P_207270	P_207270	P_207270	chr10	5763625	5763685	-	GDI2;FAM208B	"GDP dissociation inhibitor 2 [Source:HGNC Symbol;Acc:HGNC:4227];family with sequence similarity 208, member B [Source:HGNC Symbol;Acc:HGNC:23484]"	ENSG00000057608	protein_coding	3'ext;3'UTR;exon	multiple non-Ref	0	273	10:5763625:5763685	ENSG00000057608	GDI2	9.22505862	0.00329935	0.188080818	-0.577139662	-1.007161421	-0.76522048	-1.19524224	-0.430021759	9.961963646	0.85458922	0.576654371	0.337693763	0.461883978	0.259670158	0.676333307	-6.979091257	-6.009483539	-6.51978051	-6.838501404	-5.588258536	-6.971289495	0.596987303	0.631338537

NCBP2AS2	-0.156830338	0.040988883	-0.11075469	0.197819221	0.046075647	-0.151743574	8	0.059160115	0.816496581	0.816496581	0.816496581	0.816496581	0.816496581	0.816496581	2	105	0	A_23_P6708	A_23_P6708	A_23_P6708	chr3	196943457	196943517	+	NCBP2-AS2	NCBP2 antisense RNA 2 (head to head) [Source:HGNC Symbol;Acc:HGNC:25121]	ENSG00000270170	lincRNA	exon	non-coding exon	1	275	3:196943457:196943517	ENSG00000270170	NCBP2AS2	13.0564294	0.00501731	-2.711691251	0.708722548	-1.915015487	3.4204138	0.796675765	-2.623738035	9.961963646	0.021939849	0.494747097	0.084613811	0.006578677	0.444211101	0.025508955	-4.001386007	-5.924024715	-5.344085107	-2.93102565	-5.969761719	-4.218712185	5.12346092	0.021199337

NAV2	-0.154209487	-0.067171121	0.079667149	0.087038366	0.233876636	0.14683827	8	0.181361278	0.816496581	0.816496581	0.816496581	0.816496581	0.816496581	0.816496581	2	106	0	A_24_P248147	A_24_P248147	A_24_P248147	chr11	19597023	19597083	+	NAV2	neuron navigator 2 [Source:HGNC Symbol;Acc:HGNC:15997]	ENSG00000166833	protein_coding	intron	intron sense	0	276	11:19597023:19597083	ENSG00000166833	NAV2	4.149423294	0.028620684	-1.116392009	-0.486282032	0.576746413	0.630109977	1.693138422	1.063028445	9.961963646	0.290457658	0.637286839	0.576909929	0.542804706	0.121413988	0.312858312	-6.357866695	-6.058944472	-6.868453394	-6.936726626	-4.962718176	-6.486842002	1.034870589	0.418632371

IL17RC	-0.026792454	-0.139750826	-0.057837139	-0.112958373	-0.031044686	0.081913687	8	0.112235415	0.816496581	0.816496581	0.816496581	0.816496581	0.816496581	0.816496581	2	107	0	P_133983	P_133983	P_133983	chr3	9924077	9924137	+	IL17RC	interleukin 17 receptor C [Source:HGNC Symbol;Acc:HGNC:18358]	ENSG00000163702	protein_coding	"3'UTR,exon;exon"	multiple Ref	1	277	3:9924077:9924137	ENSG00000163702	IL17RC	7.922764597	0.012322592	-0.295601806	-1.541874338	-0.638118595	-1.246272532	-0.342516789	0.903755743	9.961963646	0.773603819	0.154246996	0.537787929	0.241174261	0.739078926	0.387457272	-6.950887105	-5.063510798	-6.829204464	-6.360633883	-6.234662207	-6.642644304	0.89320661	0.477984072

FARSA	-0.095927664	0.028849058	-0.033182517	0.124776722	0.062745147	-0.062031575	8	0.085252135	0.816496581	0.816496581	0.816496581	0.816496581	0.816496581	0.816496581	2	108	0	P_124800	P_124800	P_124800	chr19	12924450	12924510	+	FARSA	"phenylalanyl-tRNA synthetase, alpha subunit [Source:HGNC Symbol;Acc:HGNC:3592]"	ENSG00000179115	protein_coding	3'UTR;exon;intron	multiple non-Ref	0	278	19:12924450:12924510	ENSG00000179115	FARSA	11.42534192	0.008043225	-1.310009312	0.393969099	-0.453147761	1.70397841	0.856861551	-0.84711686	9.961963646	0.219594633	0.701905021	0.660152185	0.119321475	0.411659548	0.416816684	-6.134927583	-6.100921498	-6.936129315	-5.757355726	-5.920344278	-6.692919008	1.072006676	0.404414468

MARCHF6	-0.010086733	-0.043113481	-0.078396275	-0.033026748	-0.068309542	-0.035282794	8	0.044901822	0.816496581	0.816496581	0.816496581	0.816496581	0.816496581	0.816496581	2	109	0	P_139201	P_139201	P_139201	chr5	10429968	10430028	+	Mar-06	"membrane-associated ring finger (C3HC4) 6, E3 ubiquitin protein ligase [Source:HGNC Symbol;Acc:HGNC:30550]"	ENSG00000145495	protein_coding	exon	coding exon	1	279	5:10429968:10430028	ENSG00000145495	MARCHF6	8.813983487	0.003825781	-0.19972668	-0.853686956	-1.552319047	-0.653960275	-1.352592366	-0.698632091	9.961963646	0.84571232	0.413334801	0.151750058	0.527944121	0.206087741	0.500765754	-6.97663607	-5.811857377	-5.869577906	-6.920648287	-5.40596576	-6.811105987	0.98727059	0.437649335

ZFP69	-0.068683197	-0.270691782	-0.300399952	-0.202008585	-0.231716755	-0.029708171	8	0.24592834	0.816496581	0.816496581	0.816496581	0.816496581	0.816496581	0.816496581	2	110	0	A_32_P24709	A_32_P24709	A_32_P24709	chr1	40495862	40495922	+	ZFP69	ZFP69 zinc finger protein [Source:HGNC Symbol;Acc:HGNC:24708]	ENSG00000187815	protein_coding	exon	coding exon	1	280	1:40495862:40495922	ENSG00000187815	ZFP69	3.834325115	0.050776023	-0.373307567	-1.471266549	-1.632736681	-1.097958982	-1.259429114	-0.161470132	9.961963646	0.716740609	0.172088729	0.133690598	0.298050742	0.236583147	0.874950925	-6.922844758	-5.153985318	-5.758305194	-6.527518062	-5.515833076	-7.057153709	1.297926848	0.328548046

GFRA4	-0.14272507	-0.13158788	-0.238721422	0.011137189	-0.095996353	-0.107133542	8	0.30878196	0.816496581	0.816496581	0.816496581	0.816496581	0.816496581	0.816496581	2	111	0	P_130565	P_130565	P_130565	chr20	3659469	3659529	-	GFRA4	GDNF family receptor alpha 4 [Source:HGNC Symbol;Acc:HGNC:13821]	ENSG00000125861	protein_coding	3'UTR	3'UTR	1	281	20:3659469:3659529	ENSG00000125861	GFRA4	7.105008775	0.078774961	-0.622804577	-0.574205599	-1.041700626	0.048598978	-0.418896048	-0.467495026	9.961963646	0.547404402	0.578562602	0.322171239	0.962199426	0.684180608	0.65020443	-6.790743879	-6.011204657	-6.484845785	-7.148321595	-6.204192523	-6.953282985	0.366520861	0.778808285

RAVER2	-0.044164453	-0.157518651	0.091539651	-0.113354199	0.135704104	0.249058303	8	0.103824037	0.816496581	0.816496581	0.816496581	0.816496581	0.816496581	0.816496581	2	112	0	P_202090	P_202090	P_202090	chr1	64778029	64778089	+	RAVER2	"ribonucleoprotein, PTB-binding 2 [Source:HGNC Symbol;Acc:HGNC:25577]"	ENSG00000162437	protein_coding	exon	coding exon	1	282	1:64778029:64778089	ENSG00000162437	RAVER2	6.810482477	0.010863155	-0.518967907	-1.850971081	1.075664667	-1.332003174	1.594632573	2.926635748	9.961963646	0.615115446	0.094009149	0.307438054	0.21252912	0.141995074	0.015185605	-6.853357862	-4.640274192	-6.449574614	-6.25715574	-5.096989048	-3.703824773	2.955793067	0.08463094

FKRP	0.141378815	0.064369566	0.114177463	-0.077009249	-0.027201352	0.049807897	8	0.125105782	0.816496581	0.816496581	0.816496581	0.816496581	0.816496581	0.816496581	2	113	0	A_23_P164946	A_23_P164946	A_23_P164946	chr19	46757719	46757779	+	FKRP	fukutin related protein [Source:HGNC Symbol;Acc:HGNC:17997]	ENSG00000181027	protein_coding	exon	coding exon	1	283	19:46757719:46757779	ENSG00000181027	FKRP	8.347492792	0.014775657	1.42448068	0.648563956	1.150409916	-0.775916724	-0.274070764	0.50184596	9.961963646	0.184871352	0.531285365	0.276841108	0.455823906	0.789629212	0.626680826	-5.991355181	-5.965084551	-6.368841448	-6.829996826	-6.256887564	-6.93551482	0.783705717	0.529846187

PLIN3	-0.090925684	0.079424104	0.05522404	0.170349788	0.146149724	-0.024200064	8	0.055333211	0.816496581	0.816496581	0.816496581	0.816496581	0.816496581	0.816496581	2	114	0	P_124273	P_124273	P_124273	chr19	4838341	4838401	-	PLIN3	perilipin 3 [Source:HGNC Symbol;Acc:HGNC:16893]	ENSG00000105355	protein_coding	3'UTR	3'UTR	1	284	19:4838341:4838401	ENSG00000105355	PLIN3	11.10739315	0.004665448	-1.630367643	1.424135432	0.990209619	3.054503075	2.620577262	-0.433925813	9.961963646	0.134194	0.184968625	0.345512707	0.012209442	0.025647481	0.673589207	-5.713978554	-5.212899973	-6.536575925	-3.558085164	-3.547002225	-6.969480989	3.675413551	0.051276731

SMARCA5	-0.019002132	-0.052058516	0.022518233	-0.033056384	0.041520365	0.074576749	8	0.045892545	0.816496581	0.816496581	0.816496581	0.816496581	0.816496581	0.816496581	2	116	0	P_251027	P_251027	P_251027	chr4	143524407	143524467	+	SMARCA5	"SWI/SNF related, matrix associated, actin dependent regulator of chromatin, subfamily a, member 5 [Source:HGNC Symbol;Acc:HGNC:11101]"	ENSG00000153147	protein_coding	exon	coding exon	1	286	4:143524407:143524467	ENSG00000153147	SMARCA5	9.414039383	0.003898018	-0.372757215	-1.021211052	0.441731157	-0.648453837	0.814488373	1.46294221	9.961963646	0.717137508	0.33131363	0.668118023	0.531353677	0.43440354	0.174305756	-6.923065706	-5.660351413	-6.941597889	-6.924409123	-5.955461762	-6.013038014	0.766838328	0.538326851

KIAA0100	-0.204779998	-0.025740564	-0.156045612	0.179039434	0.048734386	-0.130305048	8	0.050151101	0.816496581	0.816496581	0.816496581	0.816496581	0.816496581	0.816496581	2	117	0	P_120920	P_120920	P_120920	chr17	28614738	28614798	-	KIAA0100	KIAA0100 [Source:HGNC Symbol;Acc:HGNC:28960]	ENSG00000007202	protein_coding	3'UTR	3'UTR	1	287	17:28614738:28614798	ENSG00000007202	KIAA0100	9.34528235	0.004226473	-3.857839178	-0.484925076	-2.939734749	3.372914103	0.91810443	-2.454809673	9.961963646	0.003193835	0.638215668	0.014849516	0.007124174	0.380255778	0.034061843	-2.055960809	-6.059622487	-3.65883742	-3.012223373	-5.866907345	-4.501879555	7.000945277	0.008139538

ULK4	0.018545582	-0.022743288	-0.003747626	-0.04128887	-0.022293208	0.018995662	8	0.089326347	0.816496581	0.816496581	0.816496581	0.816496581	0.816496581	0.816496581	2	118	0	A_24_P481534	A_24_P481534	A_24_P481534	chr3	41858858	41858918	-	ULK4	unc-51 like kinase 4 [Source:HGNC Symbol;Acc:HGNC:15784]	ENSG00000168038	protein_coding	intron;promoter	multiple non-Ref	0	288	3:41858858:41858918	ENSG00000168038	ULK4	5.105813569	0.008614414	0.244722124	-0.300113838	-0.049452582	-0.544835962	-0.294174706	0.250661256	9.961963646	0.811638604	0.770259359	0.961536077	0.597850917	0.774662647	0.807170049	-6.965778044	-6.134828244	-7.045785223	-6.989663755	-6.250861834	-7.037162249	0.099769807	0.958312353

MOAP1	-0.030107331	-0.130967549	-0.191900783	-0.100860218	-0.161793452	-0.060933234	8	0.070074775	0.816496581	0.816496581	0.816496581	0.816496581	0.816496581	0.816496581	2	119	0	P_115637	P_115637	P_115637	chr14	93182246	93182306	-	MOAP1	modulator of apoptosis 1 [Source:HGNC Symbol;Acc:HGNC:16658]	ENSG00000165943	protein_coding	3'UTR	3'UTR	1	289	14:93182246:93182306	ENSG00000165943	MOAP1	11.47159336	0.006150062	-0.470195126	-2.045359091	-2.996971499	-1.575163965	-2.526776373	-0.951612408	9.961963646	0.648340256	0.068141422	0.013467239	0.14641233	0.030117059	0.363810638	-6.879046803	-4.355813803	-3.561009389	-5.938926044	-3.697902018	-6.598023676	3.859620328	0.045431993

CIDEB	-0.109660821	0.079462081	-0.221345033	0.189122902	-0.111684212	-0.300807115	8	0.171537705	0.816496581	0.816496581	0.816496581	0.816496581	0.816496581	0.816496581	2	120	0	A_23_P37359	A_23_P37359	A_23_P37359	chr14	24305439	24305499	-	CIDEB	cell death-inducing DFFA-like effector b [Source:HGNC Symbol;Acc:HGNC:1977]	ENSG00000136305	protein_coding	3'UTR	3'UTR	1	290	14:24305439:24305499	ENSG00000136305	CIDEB	6.554122592	0.025836711	-0.835561664	0.605462081	-1.686540578	1.441023745	-0.850978913	-2.292002658	9.961963646	0.422988849	0.558412483	0.122703779	0.180261405	0.414767535	0.044954871	-6.630239782	-5.992450422	-5.68206006	-6.118760762	-5.925311375	-4.77043858	1.99385958	0.179149324

LIMCH1	-0.581332911	-0.001422637	-0.451354118	0.579910274	0.129978793	-0.449931481	8	0.055266717	0.816496581	0.816496581	0.816496581	0.816496581	0.816496581	0.816496581	2	122	0	A_32_P117354	A_32_P117354	A_32_P117354	chr4	41698707	41698767	+	LIMCH1	LIM and calponin homology domains 1 [Source:HGNC Symbol;Acc:HGNC:29191]	ENSG00000064042	protein_coding	3'UTR	3'UTR	1	292	4:41698707:41698767	ENSG00000064042	LIMCH1	10.27247413	0.004659542	-10.43035127	-0.025525139	-8.09825474	10.40482613	2.332096534	-8.072729601	9.961963646	1.11E-06	0.980140169	1.08E-05	1.14E-06	0.041992245	1.11E-05	6.116265966	-6.181743966	3.795713471	6.060627899	-4.007636956	3.755056264	58.87377557	1.21E-06

SLC24A2	-0.026007161	-0.113450324	-0.064636172	-0.087443163	-0.038629011	0.048814152	8	0.152496477	0.816496581	0.816496581	0.816496581	0.816496581	0.816496581	0.816496581	2	123	0	P_148463	P_148463	P_148463	chr9	19516089	19516149	-	SLC24A2	"solute carrier family 24 (sodium/potassium/calcium exchanger), member 2 [Source:HGNC Symbol;Acc:HGNC:10976]"	ENSG00000155886	protein_coding	3'UTR	3'UTR	1	293	9:19516089:19516149	ENSG00000155886	SLC24A2	4.239334371	0.020881858	-0.220421532	-0.961538778	-0.547818497	-0.741117246	-0.327396965	0.413720281	9.961963646	0.82999451	0.359038943	0.595876796	0.475729372	0.750143721	0.687844795	-6.971912476	-5.716885284	-6.885668206	-6.857280406	-6.239987648	-6.978670622	0.350142599	0.790034064

USP19	0.037640242	0.118001793	0.024064169	0.080361551	-0.013576073	-0.093937623	8	0.06897676	0.816496581	0.816496581	0.816496581	0.816496581	0.816496581	0.816496581	2	124	0	P_134831	P_134831	P_134831	chr3	49117058	49117118	-	USP19	ubiquitin specific peptidase 19 [Source:HGNC Symbol;Acc:HGNC:12617]	ENSG00000172046	protein_coding	exon	coding exon	1	294	3:49117058:49117118	ENSG00000172046	USP19	7.826687347	0.006027451	0.593787674	1.861518574	0.37962049	1.2677309	-0.214167184	-1.481898084	9.961963646	0.565892355	0.092398018	0.712194255	0.233723065	0.834736767	0.169292694	-6.809311855	-4.625155303	-6.968987722	-6.335195611	-6.272346757	-5.988031424	1.301829679	0.327384536

DDX20	-0.225058169	-0.196538081	-0.240629683	0.028520088	-0.015571514	-0.044091602	8	0.080282157	0.816496581	0.816496581	0.816496581	0.816496581	0.816496581	0.816496581	2	125	0	P_102732	P_102732	P_102732	chr1	111766839	111766899	+	DDX20	DEAD (Asp-Glu-Ala-Asp) box polypeptide 20 [Source:HGNC Symbol;Acc:HGNC:2743]	ENSG00000064703	protein_coding	exon	coding exon	1	295	1:111766839:111766899	ENSG00000064703	DDX20	8.21873988	0.007382551	-3.20802087	-2.801490251	-3.429980116	0.406530618	-0.221959247	-0.628489865	9.961963646	0.009407802	0.018811305	0.006474139	0.692948804	0.828829651	0.543822852	-3.155451708	-3.175367843	-2.821020275	-7.059979969	-6.270548109	-6.859786649	5.085673615	0.021654282

SLU7	0.220942016	0.050125278	0.22661908	-0.170816738	0.005677064	0.176493802	8	0.051425241	0.816496581	0.816496581	0.816496581	0.816496581	0.816496581	0.816496581	2	126	0	P_255588	P_255588	P_255588	chr5	160413898	160413958	-	SLU7	SLU7 splicing factor homolog (S. cerevisiae) [Source:HGNC Symbol;Acc:HGNC:16939]	ENSG00000164609	protein_coding	exon	coding exon	1	296	5:160413898:160413958	ENSG00000164609	SLU7	10.58623041	0.004330406	4.112061726	0.932906477	4.217720386	-3.179155249	0.10565866	3.28481391	9.961963646	0.002120539	0.372926931	0.001793079	0.009879206	0.917950435	0.008262876	-1.635269964	-5.743018729	-1.503957427	-3.344220863	-6.290750842	-3.090853684	9.41278761	0.002957874

MOV10	-0.105190735	0.021372577	-0.127463501	0.126563312	-0.022272767	-0.148836078	8	0.072872463	0.816496581	0.816496581	0.816496581	0.816496581	0.816496581	0.816496581	2	127	0	P_308027	P_308027	P_308027	chr1	112691724	112691784	-	MOV10	Mov10 RISC complex RNA helicase [Source:HGNC Symbol;Acc:HGNC:7200]	ENSG00000155363	protein_coding	exon;promoter	multiple non-Ref	0	297	1:112691724:112691784	ENSG00000155363	MOV10	5.857822191	0.006471221	-1.601511237	0.325393885	-1.94061036	1.926905122	-0.339099123	-2.266004245	9.961963646	0.140462841	0.751614126	0.081109897	0.082968892	0.741574646	0.046983547	-5.754213954	-6.126576729	-5.304943954	-5.425623274	-6.235886501	-4.812834271	2.566569133	0.113284139

PROM2	0.106559118	-0.037483123	0.116088958	-0.144042242	0.00952984	0.153572082	8	0.046735957	0.816496581	0.816496581	0.816496581	0.816496581	0.816496581	0.816496581	2	128	0	P_311472	P_311472	P_311472	chr2	95280947	95281007	-	PROM2	prominin 2 [Source:HGNC Symbol;Acc:HGNC:20685]	ENSG00000155066	protein_coding	intron	intron antisense	0	298	2:95280947:95281007	ENSG00000155066	PROM2	7.499734489	0.003960756	2.073706205	-0.729444715	2.259162775	-2.80315092	0.18545657	2.98860749	9.961963646	0.064984749	0.482528048	0.047531986	0.018757882	0.856592509	0.013660775	-5.050876488	-5.909121725	-4.800690469	-3.988340281	-6.27842403	-3.597853378	4.459997901	0.031190988

WDR43	-0.039022442	-0.083448198	-0.156536119	-0.044425755	-0.117513677	-0.073087921	8	0.083711169	0.816496581	0.816496581	0.816496581	0.816496581	0.816496581	0.816496581	2	129	0	P_234193	P_234193	P_234193	chr2	28906521	28906581	+	WDR43	WD repeat domain 43 [Source:HGNC Symbol;Acc:HGNC:28945]	ENSG00000163811	protein_coding	5'UTR;exon	multiple Ref	1	299	2:28906521:28906581	ENSG00000163811	WDR43	10.15402839	0.007834137	-0.539963624	-1.154694291	-2.16603076	-0.614730667	-1.626067136	-1.011336469	9.961963646	0.60108326	0.275162345	0.055642091	0.552513818	0.135112151	0.335788345	-6.841560376	-5.524222404	-4.951036997	-6.94680194	-5.054629922	-6.539668755	1.726893075	0.224627498

MYH11	-0.022445814	-0.114304075	0.028220026	-0.091858261	0.05066584	0.142524102	8	0.084293208	0.816496581	0.816496581	0.816496581	0.816496581	0.816496581	0.816496581	2	132	0	P_225633	P_225633	P_225633	chr16	15714908	15714968	-	MYH11	"myosin, heavy chain 11, smooth muscle [Source:HGNC Symbol;Acc:HGNC:7569]"	ENSG00000133392	protein_coding	exon	coding exon	1	302	16:15714908:15714968	ENSG00000133392	MYH11	4.941297009	0.007912663	-0.309043521	-1.57378717	0.388545335	-1.264743649	0.697588856	1.962332505	9.961963646	0.763655009	0.146729264	0.705787057	0.234748939	0.501390564	0.078243221	-6.946492679	-5.0217881	-6.965298766	-6.338755721	-6.044179849	-5.295059687	1.443372834	0.28816254

CDCA7L	0.042831983	-0.190431991	-0.049350885	-0.233263975	-0.092182868	0.141081107	8	0.101004042	0.816496581	0.816496581	0.816496581	0.816496581	0.816496581	0.816496581	2	133	0	P_260674	P_260674	P_260674	chr7	21908129	21908189	-	CDCA7L	cell division cycle associated 7-like [Source:HGNC Symbol;Acc:HGNC:30777]	ENSG00000164649	protein_coding	exon	coding exon	1	303	7:21908129:21908189	ENSG00000164649	CDCA7L	9.622170447	0.010399299	0.514412823	-2.287091336	-0.592704934	-2.801504159	-1.107117757	1.694386403	9.961963646	0.618181673	0.045331472	0.566588886	0.018810857	0.294259005	0.121171405	-6.855858919	-3.987976208	-6.85860225	-3.991149309	-5.683070963	-5.694531193	2.965297839	0.084046385

SEMA5A	0.166677796	0.161736239	0.29648157	-0.004941557	0.129803774	0.134745331	8	0.126336818	0.816496581	0.816496581	0.816496581	0.816496581	0.816496581	0.816496581	2	135	0	P_313085	P_313085	P_313085	chr5	9109567	9109627	+	SEMA5A	"sema domain, seven thrombospondin repeats (type 1 and type 1-like), transmembrane domain (TM) and short cytoplasmic domain, (semaphorin) 5A [Source:HGNC Symbol;Acc:HGNC:10736]"	ENSG00000112902	protein_coding	intron	intron antisense	0	305	5:9109567:9109627	ENSG00000112902	SEMA5A	3.843079647	0.01502423	1.665433359	1.616057656	2.962423969	-0.049375703	1.29699061	1.346366313	9.961963646	0.126913318	0.137270887	0.01428511	0.96159582	0.223867951	0.208018098	-5.664533038	-4.965764973	-3.620068469	-7.148279989	-5.472195098	-6.162131804	2.943098541	0.085419189

BRWD1	0.032062673	-0.082553148	-0.073380534	-0.114615821	-0.105443207	0.009172614	8	0.041156935	0.816496581	0.816496581	0.816496581	0.816496581	0.816496581	0.816496581	2	136	0	P_132236	P_132236	P_132236	chr21	39296740	39296800	-	BRWD1	bromodomain and WD repeat domain containing 1 [Source:HGNC Symbol;Acc:HGNC:12760]	ENSG00000185658	protein_coding	3'UTR	3'UTR	1	306	21:39296740:39296800	ENSG00000185658	BRWD1	8.286969557	0.003566973	0.657499549	-1.692892482	-1.504792464	-2.350392031	-2.162292013	0.188100018	9.961963646	0.525759446	0.121461846	0.163405006	0.040704431	0.055994043	0.854574567	-6.767470367	-4.861829171	-5.933736234	-4.749836961	-4.272466371	-7.052083911	2.63297034	0.107668379

PTBP3	0.108543761	0.105520224	0.255545298	-0.003023537	0.147001538	0.150025075	8	0.028749568	0.816496581	0.816496581	0.816496581	0.816496581	0.816496581	0.816496581	2	138	0	A_24_P295452	A_24_P295452	A_24_P295452	chr9	112222439	112222499	-	PTBP3	polypyrimidine tract binding protein 3 [Source:HGNC Symbol;Acc:HGNC:10253]	ENSG00000119314	protein_coding	3'UTR	3'UTR	1	308	9:112222439:112222499	ENSG00000119314	PTBP3	7.68606108	0.002870439	2.481283283	2.412165978	5.841701751	-0.069117306	3.360418467	3.429535773	9.961963646	0.032554704	0.03663519	0.000165896	0.946263939	0.007275291	0.006478956	-4.388496875	-3.793156634	0.967780567	-7.147001202	-2.346156901	-2.843781127	11.52661598	0.001403438

ABCG2	0.323994741	0.080160382	0.115897534	-0.243834359	-0.208097206	0.035737153	8	0.08268632	0.816496581	0.816496581	0.816496581	0.816496581	0.816496581	0.816496581	2	139	0	P_249938	P_249938	P_249938	chr4	88094576	88094636	-	ABCG2	"ATP-binding cassette, sub-family G (WHITE), member 2 [Source:HGNC Symbol;Acc:HGNC:74]"	ENSG00000118777	protein_coding	exon	coding exon	1	309	4:88094576:88094636	ENSG00000118777	ABCG2	4.948185689	0.00769719	4.522905245	1.119023754	1.617907638	-3.403881491	-2.904997607	0.498883884	9.961963646	0.001114069	0.289385935	0.136869603	0.006763468	0.015757758	0.628692563	-0.971307213	-5.56186318	-5.779071406	-2.959275272	-3.085726795	-6.937094213	7.433469429	0.006683329

FOXP2	0.059279915	-0.111628711	0.089936701	-0.170908626	0.030656786	0.201565412	8	0.117691465	0.816496581	0.816496581	0.816496581	0.816496581	0.816496581	0.816496581	2	140	0	P_315041	P_315041	P_315041	chr7	114693658	114693718	+	FOXP2	forkhead box P2 [Source:HGNC Symbol;Acc:HGNC:13875]	ENSG00000128573	protein_coding	3'UTR	3'UTR	1	310	7:114693658:114693718	ENSG00000128573	FOXP2	3.805410846	0.013330018	0.628836638	-1.184148514	0.954041394	-1.812985152	0.325204756	2.138189908	9.961963646	0.543604833	0.263837976	0.362638782	0.100029091	0.751753015	0.058314737	-6.786781041	-5.492472679	-6.571625499	-5.597688924	-6.240740272	-5.018933102	1.77872793	0.214837768

MANCR	-0.05201634	0.285444619	-0.132437578	0.337460959	-0.080421238	-0.417882197	8	0.252643169	0.816496581	0.816496581	0.816496581	0.816496581	0.816496581	0.816496581	2	141	0	P_105388	P_105388	P_105388	chr10	4650188	4650248	-	LINC00704	long intergenic non-protein coding RNA 704 [Source:HGNC Symbol;Acc:HGNC:44678]	ENSG00000231298	lincRNA	exon	non-coding exon	1	311	10:4650188:4650248	ENSG00000231298	MANCR	3.672214205	0.053464507	-0.275519689	1.511940526	-0.701494188	1.787460215	-0.425974499	-2.213434714	9.961963646	0.788547455	0.16160362	0.499054049	0.104271494	0.679183323	0.051360142	-6.957094536	-5.10218233	-6.784857303	-5.635535435	-6.201069484	-4.898089356	1.854955146	0.201322984

ZNF354A	-0.057642457	0.007971575	-0.035574387	0.065614032	0.02206807	-0.043545962	8	0.07833173	0.816496581	0.816496581	0.816496581	0.816496581	0.816496581	0.816496581	2	142	0	P_141333	P_141333	P_141333	chr5	178711665	178711725	-	ZNF354A	zinc finger protein 354A [Source:HGNC Symbol;Acc:HGNC:11628]	ENSG00000169131	protein_coding	3'UTR	3'UTR	1	312	5:178711665:178711725	ENSG00000169131	ZNF354A	9.824502521	0.007134114	-0.835830256	0.115589863	-0.515837634	0.951420118	0.319992622	-0.631427496	9.961963646	0.422844682	0.910274401	0.61722174	0.363903524	0.755584229	0.541977532	-6.630010989	-6.175048792	-6.903727927	-6.675931971	-6.242509821	-6.857842695	0.397392983	0.757851192

ZAP70	-0.002321928	0.040239028	-0.002714103	0.042560956	-0.000392175	-0.042953131	8	0.064454935	0.816496581	0.816496581	0.816496581	0.816496581	0.816496581	0.816496581	2	143	0	P_128476	P_128476	P_128476	chr2	97738046	97738106	-	ZAP70	zeta-chain (TCR) associated protein kinase 70kDa [Source:HGNC Symbol;Acc:HGNC:12858]	ENSG00000115085	protein_coding	exon	exon antisense	0	313	2:97738046:97738106	ENSG00000115085	ZAP70	4.761911139	0.005542925	-0.038196621	0.661947609	-0.044648049	0.70014423	-0.006451427	-0.706595657	9.961963646	0.970285523	0.523021403	0.965270137	0.499860956	0.994979906	0.496011996	-6.997564221	-5.956236301	-7.04603207	-6.887963552	-6.296663119	-6.805283207	0.238524676	0.867510297

MED12	0.082377519	-0.091512645	-0.087981255	-0.173890164	-0.170358774	0.00353139	8	0.057145156	0.816496581	0.816496581	0.816496581	0.816496581	0.816496581	0.816496581	2	144	0	P_270575	P_270575	P_270575	chrX	71120975	71121035	+	MED12	mediator complex subunit 12 [Source:HGNC Symbol;Acc:HGNC:11957]	ENSG00000184634	protein_coding	exon	coding exon	1	314	X:71120975:71121035	ENSG00000184634	MED12	6.066212253	0.004829114	1.451846221	-1.612846433	-1.55060815	-3.064692653	-3.002454371	0.062238283	9.961963646	0.177299226	0.137969929	0.152156621	0.011999467	0.013341884	0.951604104	-5.955847899	-4.970050593	-5.871908724	-3.540605184	-2.92710813	-7.069262776	4.254077268	0.035377958

TEX2	-0.258801313	-0.0241321	-0.266941861	0.234669214	-0.008140547	-0.242809761	8	0.057134918	0.816496581	0.816496581	0.816496581	0.816496581	0.816496581	0.816496581	2	145	0	P_229658	P_229658	P_229658	chr17	64150840	64150900	-	TEX2	testis expressed 2 [Source:HGNC Symbol;Acc:HGNC:30884]	ENSG00000136478	protein_coding	exon	coding exon	1	315	17:64150840:64150900	ENSG00000136478	TEX2	6.775148115	0.004828174	-4.561636096	-0.425352777	-4.705121514	4.136283319	-0.143485418	-4.279768737	9.961963646	0.001049749	0.679621601	0.000843749	0.002040278	0.888767735	0.001626016	-0.909829129	-6.08760045	-0.723503218	-1.726959491	-6.285746416	-1.423686659	13.09557759	0.000860211

HERC2	-0.077069775	-0.119603431	-0.114901678	-0.042533657	-0.037831903	0.004701753	8	0.06420319	0.816496581	0.816496581	0.816496581	0.816496581	0.816496581	0.816496581	2	146	0	P_222516	P_222516	P_222516	chr15	28186585	28186645	-	HERC2	HECT and RLD domain containing E3 ubiquitin protein ligase 2 [Source:HGNC Symbol;Acc:HGNC:4868]	ENSG00000128731	protein_coding	exon	coding exon	1	316	15:28186585:28186645	ENSG00000128731	HERC2	6.939520554	0.005516915	-1.270812827	-1.97215543	-1.894627652	-0.701342603	-0.623814825	0.077527778	9.961963646	0.232668529	0.076978361	0.087505466	0.499144616	0.546766993	0.939738736	-6.182157884	-4.464312599	-5.375093379	-6.887088495	-6.093775614	-7.068095934	1.663502194	0.237306639

RASSF8	0.147207796	-0.128227834	0.087859029	-0.27543563	-0.059348768	0.216086863	8	0.096562721	0.816496581	0.816496581	0.816496581	0.816496581	0.816496581	0.816496581	2	148	0	A_16_P02590514	A_16_P02590514	A_16_P02590514	chr12	25971439	25971499	+	RASSF8	Ras association (RalGDS/AF-6) domain family (N-terminal) member 8 [Source:HGNC Symbol;Acc:HGNC:13232]	ENSG00000123094	protein_coding	intron;promoter	multiple non-Ref	0	318	12:25971439:25971499	ENSG00000123094	RASSF8	5.425478231	0.009694653	1.831092617	-1.595004112	1.092863439	-3.426096729	-0.738229177	2.687867552	9.961963646	0.097116433	0.141911936	0.300176574	0.006516367	0.477405858	0.022854137	-5.42335321	-4.993774426	-6.431372828	-2.921317874	-6.014721704	-4.110308199	4.433173646	0.031701543

DEF8	0.278318653	0.051533335	0.258525099	-0.226785318	-0.019793555	0.206991763	8	0.093960684	0.816496581	0.816496581	0.816496581	0.816496581	0.816496581	0.816496581	2	149	0	A_23_P88893	A_23_P88893	A_23_P88893	chr16	89959188	89959248	+	DEF8	differentially expressed in FDCP 8 homolog (mouse) [Source:HGNC Symbol;Acc:HGNC:25969]	ENSG00000140995	protein_coding	"3'UTR,exon;exon"	multiple Ref	1	319	16:89959188:89959248	ENSG00000140995	DEF8	8.991355421	0.009296539	3.535308265	0.654595817	3.283883087	-2.880712448	-0.251425178	2.629287269	9.961963646	0.005430554	0.527551429	0.00827586	0.016425944	0.806595805	0.02526755	-2.598381834	-5.961117188	-3.070255108	-3.855851866	-6.263172009	-4.209348855	6.497609785	0.010342034

SRRM4	0.102200046	0.305933651	-0.011040949	0.203733606	-0.113240995	-0.3169746	8	0.244291603	0.816496581	0.816496581	0.816496581	0.816496581	0.816496581	0.816496581	3	22	0	P_112941	P_112941	P_112941	chr12	119116948	119117008	+	SRRM4	serine/arginine repetitive matrix 4 [Source:HGNC Symbol;Acc:HGNC:29389]	ENSG00000139767	protein_coding	exon	coding exon	1	362	12:119116948:119117008	ENSG00000139767	SRRM4	4.281531714	0.050131683	0.559037031	1.673465399	-0.060394291	1.114428367	-0.619431323	-1.73385969	9.961963646	0.588482244	0.125296361	0.953036028	0.291259318	0.549535809	0.113721265	-6.830461101	-4.888355883	-7.045129049	-6.509785151	-6.09656244	-5.637561344	1.291428581	0.330495592

DPEP3	-0.148954626	-0.261922624	0.054843829	-0.112967997	0.203798456	0.316766453	8	0.354204317	0.816496581	0.816496581	0.816496581	0.816496581	0.816496581	0.816496581	3	23	0	A_23_P129413	A_23_P129413	A_23_P129413	chr16	67975677	67975737	-	DPEP3	dipeptidase 3 [Source:HGNC Symbol;Acc:HGNC:23029]	ENSG00000141096	protein_coding	3'UTR	3'UTR	1	363	16:67975677:67975737	ENSG00000141096	DPEP3	6.7774373	0.102958466	-0.568549911	-0.99974125	0.209335252	-0.431191339	0.777885163	1.209076502	9.961963646	0.582250565	0.341099992	0.838405277	0.675510703	0.454714322	0.254546748	-6.824790165	-5.681009807	-7.023242241	-7.048879216	-5.984546137	-6.326562509	0.603241684	0.627665979

CHKA	-0.158704828	0.097311263	-0.086626163	0.256016092	0.072078666	-0.183937426	8	0.042132517	0.816496581	0.816496581	0.816496581	0.816496581	0.816496581	0.816496581	3	24	0	P_109388	P_109388	P_109388	chr11	68082859	68082919	+	CHKA	choline kinase alpha [Source:HGNC Symbol;Acc:HGNC:1937]	ENSG00000110721	protein_coding	intron;promoter	multiple non-Ref	0	364	11:68082859:68082919	ENSG00000110721	CHKA	7.836030052	0.003632225	-3.225145963	1.977526653	-1.760387644	5.202672616	1.464758319	-3.737914297	9.961963646	0.009139097	0.076294884	0.108950851	0.000404722	0.17381998	0.003885321	-3.126210027	-4.456404871	-5.575222537	-0.04262227	-5.266755767	-2.321211726	10.09941089	0.002293213

CLEC2D	-0.044949328	-0.281279483	-0.200863192	-0.236330156	-0.155913865	0.080416291	8	0.238509536	0.816496581	0.816496581	0.816496581	0.816496581	0.816496581	0.816496581	3	25	0	P_214340	P_214340	P_214340	chr12	9692871	9692931	+	CLEC2D	"C-type lectin domain family 2, member D [Source:HGNC Symbol;Acc:HGNC:14351]"	ENSG00000069493	protein_coding	3'UTR;exon	multiple Ref	1	365	12:9692871:9692931	ENSG00000069493	CLEC2D	5.66682913	0.047889885	-0.251563096	-1.57420681	-1.124149552	-1.322643714	-0.872586456	0.450057258	9.961963646	0.806492144	0.1466326	0.287307403	0.215512356	0.403430222	0.662304218	-6.963936855	-5.021236135	-6.397684412	-6.268689019	-5.906922925	-6.96184168	1.08641139	0.399041194

NELL1	0.011868163	-0.167104655	0.011893375	-0.178972818	2.52E-05	0.17899803	8	0.177164407	0.816496581	0.816496581	0.816496581	0.816496581	0.816496581	0.816496581	3	26	0	P_211270	P_211270	P_211270	chr11	20678000	20678060	+	NELL1	NEL-like 1 (chicken) [Source:HGNC Symbol;Acc:HGNC:7750]	ENSG00000165973	protein_coding	exon	coding exon	1	366	11:20678000:20678060	ENSG00000165973	NELL1	3.929994379	0.027412339	0.087792229	-1.236121424	0.087978731	-1.323913653	0.000186501	1.324100154	9.961963646	0.931781524	0.244765916	0.931637015	0.215105542	0.999854875	0.215045851	-6.994156048	-5.435015588	-7.042895222	-6.267127454	-6.296685239	-6.189649546	0.841572087	0.501759642

ADGRB2	-0.034279529	-0.007696893	0.182998668	0.026582635	0.217278197	0.190695561	8	0.152162887	0.816496581	0.816496581	0.816496581	0.816496581	0.816496581	0.816496581	3	27	0	P_101114	P_101114	P_101114	chr1	31737471	31737531	-	BAI2	brain-specific angiogenesis inhibitor 2 [Source:HGNC Symbol;Acc:HGNC:944]	ENSG00000121753	protein_coding	exon	coding exon	1	367	1:31737471:31737531	ENSG00000121753	ADGRB2	6.881819438	0.020800242	-0.291102727	-0.065362237	1.554029863	0.225740491	1.84513259	1.619392099	9.961963646	0.776943526	0.94917866	0.151344471	0.825967118	0.09491212	0.136548361	-6.952315093	-6.179835733	-5.867245639	-7.121816881	-4.747375238	-5.800749635	1.430320942	0.291547843

SLC25A20	-0.176655956	0.43448895	0.050393557	0.611144907	0.227049513	-0.384095394	8	0.090912145	0.816496581	0.816496581	0.816496581	0.816496581	0.816496581	0.816496581	3	28	0	P_134789	P_134789	P_134789	chr3	48857078	48857138	+	SLC25A20	"solute carrier family 25 (carnitine/acylcarnitine translocase), member 20 [Source:HGNC Symbol;Acc:HGNC:1421]"	ENSG00000178537	protein_coding	3'ext;3'UTR	multiple non-Ref	0	368	3:48857078:48857138	ENSG00000178537	SLC25A20	8.183348979	0.008843944	-2.300651933	5.658500645	0.656292806	7.959152577	2.956944739	-5.002207838	9.961963646	0.044299025	0.000212978	0.526503727	1.26E-05	0.014419383	0.000541811	-4.686505725	1.010413003	-6.816879604	3.583361828	-3.001184722	-0.283037379	22.47601072	9.38E-05

PNKD	-0.301068738	0.166816527	-0.16327699	0.467885265	0.137791748	-0.330093517	8	0.081423551	0.816496581	0.816496581	0.816496581	0.816496581	0.816496581	0.816496581	3	29	0	A_24_P148521	A_24_P148521	A_24_P148521	chr2	218278553	218279085	-	PNKD;TMBIM1	paroxysmal nonkinesigenic dyskinesia [Source:HGNC Symbol;Acc:HGNC:9153];transmembrane BAX inhibitor motif containing 1 [Source:HGNC Symbol;Acc:HGNC:23410]	ENSG00000127838	protein_coding	"5'UTR,intron;exon,intron;intron"	multiple non-Ref	0	369	2:218278553:218279085	ENSG00000127838	PNKD	9.519210478	0.007530771	-4.249047448	2.3543173	-2.304363061	6.603364747	1.944684386	-4.658680361	9.961963646	0.001706579	0.0404332	0.044020484	6.16E-05	0.080564855	0.000905275	-1.411569642	-3.883573775	-4.726995335	1.927134054	-4.601635835	-0.816645323	16.33280489	0.00035679

ATP8A1	0.194493089	-0.066759613	0.14163768	-0.261252702	-0.052855409	0.208397293	8	0.065965907	0.816496581	0.816496581	0.816496581	0.816496581	0.816496581	0.816496581	3	30	0	P_248761	P_248761	P_248761	chr4	42586348	42586408	-	ATP8A1	"ATPase, aminophospholipid transporter (APLT), class I, type 8A, member 1 [Source:HGNC Symbol;Acc:HGNC:13531]"	ENSG00000124406	protein_coding	3'UTR;exon	multiple Ref	1	370	4:42586348:42586408	ENSG00000124406	ATP8A1	5.476034353	0.005701176	3.154768983	-1.082872189	2.297429501	-4.237641172	-0.857339482	3.38030169	9.961963646	0.010296291	0.304378747	0.044542276	0.001737553	0.411407745	0.007036358	-3.24641026	-5.599084669	-4.738328646	-1.560542714	-5.919939439	-2.927739638	7.753739171	0.005803463

MPZ	-0.013119024	0.005677621	-0.319382928	0.018796645	-0.306263904	-0.325060549	8	0.371431768	0.816496581	0.816496581	0.816496581	0.816496581	0.816496581	0.816496581	3	31	0	P_103838	P_103838	P_103838	chr1	161304765	161304825	-	MPZ	myelin protein zero [Source:HGNC Symbol;Acc:HGNC:7225]	ENSG00000158887	protein_coding	3'UTR	3'UTR	1	371	1:161304765:161304825	ENSG00000158887	MPZ	3.702472798	0.112997338	-0.047798368	0.020686066	-1.163652272	0.068484434	-1.115853904	-1.184338338	9.961963646	0.962821619	0.983904549	0.271678267	0.946755124	0.290677169	0.263766215	-6.997113728	-6.181861845	-6.35410385	-7.147048802	-5.673919019	-6.354813885	0.66821276	0.59063501

ASL	-0.480032149	0.11399279	-0.309322953	0.594024939	0.170709196	-0.423315743	8	0.147608467	0.816496581	0.816496581	0.816496581	0.816496581	0.816496581	0.816496581	3	32	0	P_261316	P_261316	P_261316	chr7	66087100	66087160	+	ASL	argininosuccinate lyase [Source:HGNC Symbol;Acc:HGNC:746]	ENSG00000126522	protein_coding	exon	non-coding exon	1	372	7:66087100:66087160	ENSG00000126522	ASL	9.112506333	0.019703844	-4.188325919	0.994597883	-2.698872033	5.182923802	1.489453886	-3.693469915	9.961963646	0.001878453	0.343475936	0.02242717	0.000416415	0.167329556	0.004179811	-1.5104555	-5.685906119	-4.068807829	-0.072394259	-5.23515391	-2.396103255	11.42301687	0.001452076

RABAC1	0.056796831	0.124283736	0.070803585	0.067486905	0.014006754	-0.053480151	8	0.096820308	0.816496581	0.816496581	0.816496581	0.816496581	0.816496581	0.816496581	3	34	0	P_125921	P_125921	P_125921	chr19	41958285	41958345	-	RABAC1	Rab acceptor 1 (prenylated) [Source:HGNC Symbol;Acc:HGNC:9794]	ENSG00000105404	protein_coding	exon	coding exon	1	374	19:41958285:41958345	ENSG00000105404	RABAC1	10.49639744	0.009734655	0.705033002	1.542764511	0.878902272	0.837731508	0.173869269	-0.663862239	9.961963646	0.496942604	0.154032792	0.400157216	0.421825135	0.86545057	0.521845445	-6.733716006	-5.062353838	-6.64093906	-6.77881866	-6.280631204	-6.83582558	0.803733458	0.519949876

ESCO1	0.033110016	0.016083295	-0.000722513	-0.01702672	-0.033832529	-0.016805809	8	0.051869967	0.816496581	0.816496581	0.816496581	0.816496581	0.816496581	0.816496581	3	35	0	P_230825	P_230825	P_230825	chr18	21564219	21564279	-	ESCO1	establishment of sister chromatid cohesion N-acetyltransferase 1 [Source:HGNC Symbol;Acc:HGNC:24645]	ENSG00000141446	protein_coding	exon	coding exon	1	375	18:21564219:21564279	ENSG00000141446	ESCO1	7.320134873	0.004367297	0.613618797	0.298067279	-0.013390142	-0.315551517	-0.627008939	-0.311457422	9.961963646	0.553219559	0.771775725	0.989580899	0.758854194	0.544754488	0.761873084	-6.796710715	-6.13546763	-7.047022688	-7.095432023	-6.091733533	-7.018642128	0.175878354	0.910287192

TRAPPC2L	0.039180901	0.039803044	0.232462999	0.000622143	0.193282098	0.192659955	8	0.078532287	0.816496581	0.816496581	0.816496581	0.816496581	0.816496581	0.816496581	3	36	0	A_24_P327050	A_24_P327050	A_24_P327050	chr16	88859892	88859952	+	TRAPPC2L	trafficking protein particle complex 2-like [Source:HGNC Symbol;Acc:HGNC:30887]	ENSG00000167515	protein_coding	3'UTR;exon	multiple Ref	1	376	16:88859892:88859952	ENSG00000167515	TRAPPC2L	8.585268269	0.007159378	0.567129697	0.576134998	3.364819779	0.009005301	2.797690082	2.788684781	9.961963646	0.58317865	0.577307389	0.00722169	0.992992694	0.01893413	0.019228424	-6.825642496	-6.010073795	-2.932075198	-7.149568715	-3.260196701	-3.939132211	4.596582246	0.028738492

PIP4K2A	-0.085628617	0.049903212	0.003780029	0.135531829	0.089408646	-0.046123183	8	0.11000642	0.816496581	0.816496581	0.816496581	0.816496581	0.816496581	0.816496581	3	37	0	P_105652	P_105652	P_105652	chr10	22539970	22540030	-	PIP4K2A	"phosphatidylinositol-5-phosphate 4-kinase, type II, alpha [Source:HGNC Symbol;Acc:HGNC:8997]"	ENSG00000150867	protein_coding	exon	coding exon	1	377	10:22539970:22540030	ENSG00000150867	PIP4K2A	7.400306422	0.011924778	-0.960371818	0.559691844	0.042395092	1.520063662	1.00276691	-0.517296752	9.961963646	0.359597543	0.58805215	0.967021432	0.159577549	0.339708003	0.616239475	-6.517193712	-6.019597826	-7.046139144	-6.014003048	-5.787994579	-6.92713254	0.803516212	0.520056221

TMC1	0.024933265	-0.042856313	0.085350413	-0.067789578	0.060417148	0.128206726	8	0.083649056	0.816496581	0.816496581	0.816496581	0.816496581	0.816496581	0.816496581	3	38	0	P_148893	P_148893	P_148893	chr9	72836000	72836060	+	TMC1	transmembrane channel-like 1 [Source:HGNC Symbol;Acc:HGNC:16513]	ENSG00000165091	protein_coding	3'UTR;exon	multiple Ref	1	378	9:72836000:72836060	ENSG00000165091	TMC1	3.938418992	0.007825789	0.345191996	-0.593330098	1.181645479	-0.938522094	0.836453482	1.774975576	9.961963646	0.737127594	0.566186657	0.264785684	0.370173119	0.422510293	0.106406063	-6.933725949	-5.999839403	-6.333874403	-6.688159373	-5.93744991	-5.577482856	1.0997501	0.394134815

C1orf127	0.357806911	-0.080576921	0.038840953	-0.438383833	-0.318965959	0.119417874	8	0.156369712	0.816496581	0.816496581	0.816496581	0.816496581	0.816496581	0.816496581	3	39	0	P_100396	P_100396	P_100396	chr1	10946475	10946535	-	C1orf127	chromosome 1 open reading frame 127 [Source:HGNC Symbol;Acc:HGNC:26730]	ENSG00000175262	protein_coding	3'UTR;exon	multiple Ref	1	379	1:10946475:10946535	ENSG00000175262	C1orf127	5.787471067	0.021842561	2.965122043	-0.667735581	0.321872387	-3.632857624	-2.643249656	0.989607967	9.961963646	0.014219458	0.519471271	0.754201687	0.004619398	0.024670181	0.34579265	-3.570346885	-5.952358671	-6.990836923	-2.569358864	-3.51040678	-6.561236542	5.084194759	0.021672323

ATF1	-0.00577656	0.066437467	-0.011157321	0.072214027	-0.005380761	-0.077594788	8	0.072225789	0.816496581	0.816496581	0.816496581	0.816496581	0.816496581	0.816496581	3	40	0	P_111541	P_111541	P_111541	chr12	50819950	50820010	+	ATF1	activating transcription factor 1 [Source:HGNC Symbol;Acc:HGNC:783]	ENSG00000123268	protein_coding	3'UTR	3'UTR	1	380	12:50819950:50820010	ENSG00000123268	ATF1	8.387791325	0.00639587	-0.088463708	1.017440193	-0.170866045	1.105903901	-0.082402337	-1.188306238	9.961963646	0.931261245	0.33301713	0.867749673	0.294759384	0.935958954	0.262269744	-6.994091515	-5.664005319	-7.031200293	-6.518989313	-6.293074941	-6.350311898	0.619015146	0.618487637

FBXL2	0.083429729	-0.187225343	0.232289897	-0.270655072	0.148860168	0.41951524	8	0.177668591	0.816496581	0.816496581	0.816496581	0.816496581	0.816496581	0.816496581	3	41	0	P_243669	P_243669	P_243669	chr3	33373100	33373160	+	FBXL2	F-box and leucine-rich repeat protein 2 [Source:HGNC Symbol;Acc:HGNC:13598]	ENSG00000153558	protein_coding	"3'UTR;3'UTR,exon;exon"	multiple Ref	1	381	3:33373100:33373160	ENSG00000153558	FBXL2	4.661355046	0.027556006	0.615542896	-1.381344897	1.713830288	-1.996887793	1.098287391	3.095175184	9.961963646	0.551998592	0.197367339	0.117448057	0.073878332	0.297914131	0.011393043	-6.795467668	-5.265312684	-5.64286588	-5.317564351	-5.69226507	-3.415433516	3.333021085	0.064702967

PLEKHO2	-0.0366204	-0.230410277	-0.148651802	-0.193789877	-0.112031402	0.081758475	8	0.169247597	0.816496581	0.816496581	0.816496581	0.816496581	0.816496581	0.816496581	3	42	0	A_23_P129246	A_23_P129246	A_23_P129246	chr15	64867756	64867816	+	PLEKHO2	"pleckstrin homology domain containing, family O member 2 [Source:HGNC Symbol;Acc:HGNC:30026]"	ENSG00000241839	protein_coding	3'UTR	3'UTR	1	382	15:64867756:64867816	ENSG00000241839	PLEKHO2	6.896809928	0.025209979	-0.282476598	-1.777302006	-1.146646539	-1.494825408	-0.864169941	0.630655467	9.961963646	0.78336008	0.106005292	0.278322372	0.165945974	0.407820598	0.542462144	-6.954992757	-4.744739753	-6.373006281	-6.047838619	-5.914132468	-6.858354389	1.32206879	0.321424445

NCAPG2	-0.327158443	-0.10579272	-0.275518087	0.221365724	0.051640356	-0.169725368	8	0.053079538	0.816496581	0.816496581	0.816496581	0.816496581	0.816496581	0.816496581	3	43	0	P_263574	P_263574	P_263574	chr7	158686171	158686231	-	NCAPG2	"non-SMC condensin II complex, subunit G2 [Source:HGNC Symbol;Acc:HGNC:21904]"	ENSG00000146918	protein_coding	exon	coding exon	1	383	7:158686171:158686231	ENSG00000146918	NCAPG2	8.158347922	0.00446924	-5.993588632	-1.938137483	-5.047530061	4.05545115	0.946058571	-3.109392578	9.961963646	0.000135331	0.081442412	0.000506974	0.002321355	0.366500344	0.011120989	1.213832552	-4.514189388	-0.194453641	-1.860512876	-5.84148764	-3.391088671	15.36117906	0.00045733

EPHA3	-0.033715293	-0.302538006	-0.137233987	-0.268822713	-0.103518694	0.16530402	8	0.055941252	0.816496581	0.816496581	0.816496581	0.816496581	0.816496581	0.816496581	3	45	0	P_245078	P_245078	P_245078	chr3	89449295	89449355	+	EPHA3	EPH receptor A3 [Source:HGNC Symbol;Acc:HGNC:3387]	ENSG00000044524	protein_coding	exon	coding exon	1	385	3:89449295:89449355	ENSG00000044524	EPHA3	8.678418177	0.004719782	-0.601051396	-5.393424553	-2.446506353	-4.792373157	-1.845454956	2.946918201	9.961963646	0.5612319	0.000308264	0.034548561	0.000739855	0.094862055	0.014668418	-6.804740959	0.67369167	-4.492585264	-0.672519318	-4.746908941	-3.669155046	11.74875931	0.001305574

KRT10	-0.047876042	-0.024231255	0.0047711	0.023644788	0.052647142	0.029002355	8	0.116755536	0.816496581	0.816496581	0.816496581	0.816496581	0.816496581	0.816496581	3	46	0	P_228688	P_228688	P_228688	chr17	40819520	40819580	-	KRT10	keratin 10 [Source:HGNC Symbol;Acc:HGNC:6413]	ENSG00000186395	protein_coding	exon	coding exon	1	386	17:40819520:40819580	ENSG00000186395	KRT10	7.610142924	0.013153807	-0.511255671	-0.258759199	0.050949325	0.252496472	0.562204996	0.309708525	9.961963646	0.620311418	0.801088965	0.960373011	0.805790711	0.586403024	0.763163963	-6.857580152	-6.146912979	-7.045703177	-7.11485964	-6.131268207	-7.019229876	0.134447136	0.937256128

TGOLN2	0.142246926	0.156151602	0.050259784	0.013904675	-0.091987143	-0.105891818	8	0.056289799	0.816496581	0.816496581	0.816496581	0.816496581	0.816496581	0.816496581	3	47	0	A_23_P209933	A_23_P209933	A_23_P209933	chr2	85324967	85326536	-	TGOLN2	trans-golgi network protein 2 [Source:HGNC Symbol;Acc:HGNC:15450]	ENSG00000152291	protein_coding	"exon,intron"	multiple non-Ref	0	387	2:85324967:85326536	ENSG00000152291	TGOLN2	8.229617154	0.004751196	2.527476172	2.774537645	0.893027419	0.247061473	-1.634448753	-1.881510226	9.961963646	0.030081008	0.019700075	0.392904199	0.809877639	0.133327846	0.089414311	-4.311417412	-3.218464016	-6.628276368	-7.116335138	-5.043256508	-5.418537916	3.52603929	0.056681643

PHKA1	0.011869005	0.105052439	-0.024863031	0.093183435	-0.036732036	-0.12991547	8	0.050011302	0.816496581	0.816496581	0.816496581	0.816496581	0.816496581	0.816496581	3	48	0	P_270637	P_270637	P_270637	chrX	72605540	72605600	-	PHKA1	"phosphorylase kinase, alpha 1 (muscle) [Source:HGNC Symbol;Acc:HGNC:8925]"	ENSG00000067177	protein_coding	exon	coding exon	1	388	X:72605540:72605600	ENSG00000067177	PHKA1	6.971112452	0.004215228	0.223897575	1.981715178	-0.469017628	1.757817603	-0.692915203	-2.450732806	9.961963646	0.827361938	0.07576587	0.6491529	0.109404898	0.504195548	0.034299968	-6.971073858	-4.450232332	-6.928305468	-5.679141833	-6.047471165	-4.508662518	2.2955747	0.140087659

SLC35F1	-0.097965963	0.189283746	0.153665005	0.287249709	0.251630968	-0.035618741	8	0.159091587	0.816496581	0.816496581	0.816496581	0.816496581	0.816496581	0.816496581	3	49	0	A_23_P413888	A_23_P413888	A_23_P413888	chr6	118317145	118317205	+	SLC35F1	"solute carrier family 35, member F1 [Source:HGNC Symbol;Acc:HGNC:21483]"	ENSG00000196376	protein_coding	3'UTR	3'UTR	1	389	6:118317145:118317205	ENSG00000196376	SLC35F1	4.235328734	0.022532101	-0.799318744	1.544394004	1.253775443	2.343712748	2.053094187	-0.290618561	9.961963646	0.442746704	0.15364136	0.238547236	0.041170051	0.067265907	0.777303212	-6.660523631	-5.060234955	-6.25047036	-4.760841009	-4.439295205	-7.025434067	2.398110082	0.129151062

CC2D2A	0.199058527	-0.129849547	-0.037310622	-0.328908074	-0.236369148	0.092538925	8	0.065376888	0.816496581	0.816496581	0.816496581	0.816496581	0.816496581	0.816496581	3	50	0	P_248267	P_248267	P_248267	chr4	15601365	15601425	+	CC2D2A	coiled-coil and C2 domain containing 2A [Source:HGNC Symbol;Acc:HGNC:29253]	ENSG00000048342	protein_coding	3'UTR;exon	multiple Ref	1	390	4:15601365:15601425	ENSG00000048342	CC2D2A	6.716612906	0.005639049	3.246560212	-2.117791086	-0.608520432	-5.364351298	-3.855080644	1.509270653	9.961963646	0.008814141	0.060350593	0.556462193	0.000321216	0.003208203	0.162274421	-3.089652975	-4.247018373	-6.84859257	0.199011641	-1.557929877	-5.951562157	10.21854982	0.002196962

VPS36	0.238284884	0.233686269	0.351426398	-0.004598616	0.113141514	0.11774013	8	0.071980389	0.816496581	0.816496581	0.816496581	0.816496581	0.816496581	0.816496581	3	51	0	P_219269	P_219269	P_219269	chr13	52436289	52436349	-	VPS36	vacuolar protein sorting 36 homolog (S. cerevisiae) [Source:HGNC Symbol;Acc:HGNC:20312]	ENSG00000136100	protein_coding	exon	coding exon	1	391	13:52436289:52436349	ENSG00000136100	VPS36	7.681786554	0.006367451	3.657289294	3.586707945	5.393829353	-0.070581349	1.736540059	1.807121408	9.961963646	0.004436686	0.004986195	0.000308087	0.945127758	0.113230773	0.100989402	-2.392261785	-1.925965187	0.323575692	-7.146889409	-4.902185578	-5.530007487	10.26997914	0.002156914

ADGRB3	0.056978771	-0.085495543	-0.039343988	-0.142474314	-0.09632276	0.046151554	8	0.188337127	0.816496581	0.816496581	0.816496581	0.816496581	0.816496581	0.816496581	3	52	0	P_257666	P_257666	P_257666	chr6	69361452	69361512	+	BAI3	brain-specific angiogenesis inhibitor 3 [Source:HGNC Symbol;Acc:HGNC:945]	ENSG00000135298	protein_coding	exon	coding exon	1	392	6:69361452:69361512	ENSG00000135298	ADGRB3	3.629221188	0.030691729	0.398334599	-0.597693348	-0.275051065	-0.996027947	-0.673385663	0.322642283	9.961963646	0.698786948	0.563383776	0.788897275	0.342814103	0.516019626	0.753635701	-6.912462977	-5.99719794	-7.005962922	-6.632580169	-6.06100727	-7.014806561	0.356863141	0.785419648

CREB3L4	-0.462487002	0.051914326	-0.650876662	0.514401328	-0.18838966	-0.702790988	8	0.09878358	0.816496581	0.816496581	0.816496581	0.816496581	0.816496581	0.816496581	3	53	0	A_14_P116859	A_14_P116859	A_14_P116859	chr1	153974287	153974347	+	CREB3L4	cAMP responsive element binding protein 3-like 4 [Source:HGNC Symbol;Acc:HGNC:18854]	ENSG00000143578	protein_coding	3'UTR	3'UTR	1	393	1:153974287:153974347	ENSG00000143578	CREB3L4	10.06911051	0.010043047	-5.652133464	0.634453941	-7.954476014	6.286587405	-2.30234255	-8.588929955	9.961963646	0.000214853	0.540080206	1.27E-05	9.22E-05	0.044171922	6.45E-06	0.734389811	-5.974233685	3.634632167	1.505789028	-4.054453598	4.317248336	35.70241889	1.22E-05

OSBP	0.065639126	0.001995757	0.076904462	-0.063643369	0.011265336	0.074908705	8	0.084455294	0.816496581	0.816496581	0.816496581	0.816496581	0.816496581	0.816496581	3	54	0	P_211865	P_211865	P_211865	chr11	59601736	59601796	-	OSBP	oxysterol binding protein [Source:HGNC Symbol;Acc:HGNC:8503]	ENSG00000110048	protein_coding	exon	coding exon	1	394	11:59601736:59601796	ENSG00000110048	OSBP	9.286720168	0.007934628	0.902495664	0.027440377	1.057386773	-0.875055287	0.154891109	1.029946396	9.961963646	0.388094263	0.978650415	0.315301659	0.402148613	0.88000043	0.327392451	-6.571272285	-6.181690493	-6.468667921	-6.746271493	-6.28394048	-6.520894385	0.630636796	0.611802194

FAM178B	-0.018898494	0.07015689	0.032799149	0.089055384	0.051697643	-0.037357741	8	0.198294789	0.816496581	0.816496581	0.816496581	0.816496581	0.816496581	0.816496581	3	55	0	A_24_P231546	A_24_P231546	A_24_P231546	chr2	96875889	96875949	-	FAM178B	"family with sequence similarity 178, member B [Source:HGNC Symbol;Acc:HGNC:28036]"	ENSG00000168754	protein_coding	3'UTR	3'UTR	1	395	2:96875889:96875949	ENSG00000168754	FAM178B	4.650697089	0.033783449	-0.125927571	0.467481	0.21855272	0.593408571	0.344480291	-0.24892828	9.961963646	0.902294461	0.650214121	0.831410765	0.566136179	0.737646531	0.808473183	-6.989713802	-6.068177689	-7.021098287	-6.960388329	-6.233953412	-7.037632261	0.135821204	0.936388424

CBLN1	0.086528756	0.24644913	0.12175125	0.159920374	0.035222494	-0.12469788	8	0.215911591	0.816496581	0.816496581	0.816496581	0.816496581	0.816496581	0.816496581	3	56	0	P_118955	P_118955	P_118955	chr16	49279024	49279084	-	CBLN1	cerebellin 1 precursor [Source:HGNC Symbol;Acc:HGNC:1543]	ENSG00000102924	protein_coding	3'UTR	3'UTR	1	396	16:49279024:49279084	ENSG00000102924	CBLN1	4.592671221	0.039643331	0.532256554	1.515960367	0.748917513	0.983703812	0.216660959	-0.767042854	9.961963646	0.606214754	0.160598204	0.471220116	0.348548668	0.832845058	0.460847829	-6.845942306	-5.097015686	-6.749193419	-6.644720793	-6.271778042	-6.759156453	0.790880036	0.526279594

RNH1	-0.189641517	0.04838064	-0.137396149	0.238022157	0.052245368	-0.185776789	8	0.152211319	0.816496581	0.816496581	0.816496581	0.816496581	0.816496581	0.816496581	3	57	0	P_107502	P_107502	P_107502	chr11	496103	496163	-	RNH1;PTDSS2	ribonuclease/angiogenin inhibitor 1 [Source:HGNC Symbol;Acc:HGNC:10074];phosphatidylserine synthase 2 [Source:HGNC Symbol;Acc:HGNC:15463]	ENSG00000023191	protein_coding	3'ext;intron	multiple non-Ref	0	397	11:496103:496163	ENSG00000023191	RNH1	7.832264047	0.020812081	-1.609983042	0.410732901	-1.166440096	2.020715942	0.443542946	-1.577172996	9.961963646	0.138595923	0.689963586	0.270601132	0.071003297	0.666850939	0.145950941	-5.742444773	-6.093928544	-6.350984921	-5.280395712	-6.19310073	-5.859321909	1.814804555	0.208314799

HMMR	-0.452395126	-0.022572639	-0.285799187	0.429822487	0.166595939	-0.263226548	8	0.124133843	0.816496581	0.816496581	0.816496581	0.816496581	0.816496581	0.816496581	3	58	0	P_255625	P_255625	P_255625	chr5	163469758	163469818	+	HMMR	hyaluronan-mediated motility receptor (RHAMM) [Source:HGNC Symbol;Acc:HGNC:5012]	ENSG00000072571	protein_coding	exon	coding exon	1	398	5:163469758:163469818	ENSG00000072571	HMMR	10.19156953	0.014581121	-4.588472111	-0.228945711	-2.898752711	4.3595264	1.689719399	-2.669807001	9.961963646	0.0010075	0.823542747	0.015926909	0.001435039	0.122080827	0.02357257	-0.867349795	-6.154530593	-3.728817396	-1.362043613	-4.967452237	-4.140879714	9.749625205	0.00260648

ABAT	-0.096345015	0.321054624	0.076096015	0.41739964	0.172441031	-0.244958609	8	0.088949878	0.816496581	0.816496581	0.816496581	0.816496581	0.816496581	0.816496581	3	60	0	P_225453	P_225453	P_225453	chr16	8750426	8750486	+	ABAT	4-aminobutyrate aminotransferase [Source:HGNC Symbol;Acc:HGNC:23]	ENSG00000183044	protein_coding	"3'UTR,exon;exon"	multiple Ref	1	400	16:8750426:8750486	ENSG00000183044	ABAT	8.792202848	0.008560516	-1.275336826	4.249859568	1.007297058	5.525196395	2.282633884	-3.24256251	9.961963646	0.231127631	0.001704396	0.337631735	0.000256186	0.045675923	0.008873892	-6.176758895	-0.914065056	-6.519645188	0.435672558	-4.085374928	-3.163119462	11.15914367	0.001585392

WARS2	-0.080291774	0.036321362	-0.023163679	0.116613136	0.057128095	-0.059485041	8	0.084286618	0.816496581	0.816496581	0.816496581	0.816496581	0.816496581	0.816496581	3	63	0	A_32_P92101	A_32_P92101	A_32_P92101	chr1	119140772	119141785	+	WARS2;RP11-418J17.1	"tryptophanyl tRNA synthetase 2, mitochondrial [Source:HGNC Symbol;Acc:HGNC:12730];"	ENSG00000116874	protein_coding;antisense	"exon,intron;promoter"	multiple non-Ref	0	403	1:119140772:119141785	ENSG00000116874	WARS2	8.341768669	0.007911771	-1.105553646	0.500116168	-0.318945368	1.605669814	0.786608278	-0.819061536	9.961963646	0.29490389	0.627855251	0.756354864	0.139543648	0.449818421	0.431909053	-6.369547628	-6.051929371	-6.991850728	-5.896672034	-5.977721368	-6.71678065	0.906980188	0.471841482

CASP10	-0.023968408	0.055603603	-0.130303983	0.079572011	-0.106335575	-0.185907586	8	0.144770325	0.816496581	0.816496581	0.816496581	0.816496581	0.816496581	0.816496581	3	64	0	P_129605	P_129605	P_129605	chr2	201229327	201229387	+	CASP10	"caspase 10, apoptosis-related cysteine peptidase [Source:HGNC Symbol;Acc:HGNC:1500]"	ENSG00000003400	protein_coding	3'UTR	3'UTR	1	404	2:201229327:201229387	ENSG00000003400	CASP10	4.258020438	0.01903746	-0.212755265	0.493564669	-1.15664164	0.706319934	-0.943886375	-1.650206309	9.961963646	0.835808301	0.632313196	0.274401966	0.496176119	0.367556224	0.130030259	-6.973716072	-6.055274854	-6.361922071	-6.883439532	-5.843485623	-5.757434781	0.956613495	0.450384765

BRWD3	0.107810641	-0.100714877	0.01507765	-0.208525518	-0.092732992	0.115792526	8	0.059784942	0.816496581	0.816496581	0.816496581	0.816496581	0.816496581	0.816496581	3	65	0	P_270773	P_270773	P_270773	chrX	80729915	80729975	-	BRWD3	bromodomain and WD repeat domain containing 3 [Source:HGNC Symbol;Acc:HGNC:17342]	ENSG00000165288	protein_coding	exon	coding exon	1	405	X:80729915:80729975	ENSG00000165288	BRWD3	5.956475422	0.005076993	1.853121851	-1.731155076	0.259164786	-3.584276927	-1.593957065	1.990319862	9.961963646	0.093678522	0.114218151	0.800784755	0.005006337	0.142146342	0.074689905	-5.390409531	-4.809114063	-7.010564366	-2.651813508	-5.097894119	-5.25176689	4.307873042	0.034222623

SAP30BP	0.016057838	0.106575485	0.076178591	0.090517648	0.060120753	-0.030396894	8	0.034197764	0.816496581	0.816496581	0.816496581	0.816496581	0.816496581	0.816496581	3	67	0	A_23_P54953	A_23_P54953	A_23_P54953	chr17	75702483	75702543	+	SAP30BP	SAP30 binding protein [Source:HGNC Symbol;Acc:HGNC:30785]	ENSG00000161526	protein_coding	3'UTR;exon	multiple Ref	1	407	17:75702483:75702543	ENSG00000161526	SAP30BP	9.184836871	0.003145846	0.350642027	2.327202778	1.663450356	1.976560751	1.312808329	-0.663752422	9.961963646	0.733158342	0.0423434	0.127315399	0.076417371	0.218684756	0.521912852	-6.93168156	-3.925774858	-5.714951219	-5.349123882	-5.453549522	-6.835901831	2.396119179	0.129353652

FAM102B	0.117542576	-0.105531861	0.058874201	-0.223074437	-0.058668374	0.164406063	8	0.0759662	0.816496581	0.816496581	0.816496581	0.816496581	0.816496581	0.816496581	3	68	0	P_203221	P_203221	P_203221	chr1	108624688	108624748	+	FAM102B	"family with sequence similarity 102, member B [Source:HGNC Symbol;Acc:HGNC:27637]"	ENSG00000162636	protein_coding	exon	coding exon	1	408	1:108624688:108624748	ENSG00000162636	FAM102B	5.680126	0.006841002	1.740527827	-1.562677527	0.871787821	-3.303205355	-0.868740007	2.434465348	9.961963646	0.112504595	0.14930891	0.403845411	0.008010587	0.405432604	0.035266554	-5.556697463	-5.036369736	-6.647251558	-3.131547348	-5.910225179	-4.535700721	3.97065142	0.042291825

HMGN3	-0.117191701	-0.132353927	-0.192043156	-0.015162227	-0.074851455	-0.059689228	8	0.136324178	0.816496581	0.816496581	0.816496581	0.816496581	0.816496581	0.816496581	3	71	0	P_143089	P_143089	P_143089	chr6	79202316	79202376	-	HMGN3	high mobility group nucleosomal binding domain 3 [Source:HGNC Symbol;Acc:HGNC:12312]	ENSG00000118418	protein_coding	exon	coding exon	1	411	6:79202316:79202376	ENSG00000118418	HMGN3	7.601963624	0.017130875	-1.096611276	-1.23849051	-1.79702734	-0.141879233	-0.700416064	-0.55853683	9.961963646	0.298611864	0.243923829	0.102662391	0.890003668	0.499698409	0.588810899	-6.379118256	-5.432353808	-5.521326395	-7.138615969	-6.042179148	-6.903584367	1.131399677	0.382754756

SLC9A4	-0.090878292	0.085350531	0.242600164	0.176228823	0.333478456	0.157249633	8	0.17671191	0.816496581	0.816496581	0.816496581	0.816496581	0.816496581	0.816496581	3	72	0	P_128591	P_128591	P_128591	chr2	102532469	102532529	+	SLC9A4	"solute carrier family 9, subfamily A (NHE4, cation proton antiporter 4), member 4 [Source:HGNC Symbol;Acc:HGNC:11077]"	ENSG00000180251	protein_coding	exon	coding exon	1	412	2:102532469:102532529	ENSG00000180251	SLC9A4	3.428560552	0.027283747	-0.67383533	0.632848632	1.79880758	1.306683963	2.47264291	1.165958947	9.961963646	0.515745516	0.54108612	0.102365498	0.220679683	0.033039226	0.270786792	-6.756112047	-5.975262882	-5.518693294	-6.288224062	-3.784552947	-6.375519082	2.211851059	0.149829465

SRD5A3	0.026899689	0.105661598	0.143511439	0.078761909	0.116611749	0.037849841	8	0.080014572	0.816496581	0.816496581	0.816496581	0.816496581	0.816496581	0.816496581	3	75	0	P_137725	P_137725	P_137725	chr4	55364201	55364261	+	SRD5A3	steroid 5 alpha-reductase 3 [Source:HGNC Symbol;Acc:HGNC:25812]	ENSG00000128039	protein_coding	exon	coding exon	1	415	4:55364201:55364261	ENSG00000128039	SRD5A3	11.15098293	0.007348106	0.384330808	1.509645966	2.050427679	1.125315158	1.666096871	0.540781713	9.961963646	0.708809722	0.162179978	0.06756651	0.286836374	0.126779037	0.600539897	-6.918352599	-5.105127758	-5.134473813	-6.497950585	-5.000019436	-6.913931245	1.827608772	0.20605498

SPC24	-0.416488425	0.041831514	-0.334908554	0.458319939	0.081579871	-0.376740068	8	0.073135004	0.816496581	0.816496581	0.816496581	0.816496581	0.816496581	0.816496581	3	76	0	P_124649	P_124649	P_124649	chr19	11147091	11147151	-	SPC24	"SPC24, NDC80 kinetochore complex component [Source:HGNC Symbol;Acc:HGNC:26913]"	ENSG00000161888	protein_coding	3'UTR	3'UTR	1	416	19:11147091:11147151	ENSG00000161888	SPC24	9.965471524	0.006502005	-6.325937854	0.63536833	-5.086841725	6.961306184	1.239096129	-5.722210055	9.961963646	8.76E-05	0.539507717	0.000478691	3.97E-05	0.24370894	0.000195153	1.66444165	-5.973646379	-0.134777314	2.386996507	-5.539073105	0.780670078	24.83932871	6.09E-05

UBR7	-0.294989407	0.123848251	-0.131267147	0.418837658	0.163722261	-0.255115398	8	0.045307334	0.816496581	0.816496581	0.816496581	0.816496581	0.816496581	0.816496581	3	77	0	A_23_P205393	A_23_P205393	A_23_P205393	chr14	93222351	93226979	+	UBR7;RP11-85G20.1	ubiquitin protein ligase E3 component n-recognin 7 (putative) [Source:HGNC Symbol;Acc:HGNC:20344];	ENSG00000012963	protein_coding;processed_pseudogene	"3'UTR,intron;exon,intron;exon,promoter"	multiple non-Ref	0	417	14:93222351:93226979	ENSG00000012963	UBR7	9.233221509	0.003855158	-5.818767051	2.442949154	-2.58928941	8.261716205	3.229477641	-5.032238564	9.961963646	0.000171123	0.034759163	0.027059782	9.09E-06	0.009072387	0.000518453	0.970469606	-3.744848258	-4.25379097	3.922844136	-2.558075432	-0.237178973	25.08989595	5.83E-05

TPK1	0.168477652	0.236786422	0.134326437	0.068308771	-0.034151214	-0.102459985	8	0.084998974	0.816496581	0.816496581	0.816496581	0.816496581	0.816496581	0.816496581	3	78	0	P_146197	P_146197	P_146197	chr7	144453375	144453435	-	TPK1	thiamin pyrophosphokinase 1 [Source:HGNC Symbol;Acc:HGNC:17358]	ENSG00000196511	protein_coding	3'UTR	3'UTR	1	418	7:144453375:144453435	ENSG00000196511	TPK1	4.986793721	0.008008613	2.305734406	3.240587671	1.838351169	0.934853265	-0.467383237	-1.402236502	9.961963646	0.043917989	0.008903562	0.095970985	0.371970598	0.650281665	0.191228483	-4.678205361	-2.473090306	-5.459870944	-6.691611957	-6.181793503	-6.091707247	3.709336591	0.050136063

EHMT1	0.011376843	0.028954044	-0.080828552	0.017577201	-0.092205395	-0.109782596	8	0.06143967	0.816496581	0.816496581	0.816496581	0.816496581	0.816496581	0.816496581	3	79	0	P_150361	P_150361	P_150361	chr9	137777165	137777225	-	EHMT1	euchromatic histone-lysine N-methyltransferase 1 [Source:HGNC Symbol;Acc:HGNC:24650]	ENSG00000181090	protein_coding	intron	intron antisense	0	419	9:137777165:137777225	ENSG00000181090	EHMT1	7.002190782	0.005238081	0.19252242	0.489969184	-1.367805449	0.297446764	-1.56032787	-1.857774633	9.961963646	0.851201082	0.634766332	0.201434187	0.772235686	0.149859593	0.092966954	-6.97817214	-6.057093051	-6.111440973	-7.101444158	-5.142669036	-5.454341286	1.353741776	0.31234018

FBXO46	-0.048241694	0.105055506	-0.142888973	0.1532972	-0.094647279	-0.247944479	8	0.142780639	0.816496581	0.816496581	0.816496581	0.816496581	0.816496581	0.816496581	3	80	0	P_126113	P_126113	P_126113	chr19	45711450	45711510	-	FBXO46	F-box protein 46 [Source:HGNC Symbol;Acc:HGNC:25069]	ENSG00000177051	protein_coding	3'UTR	3'UTR	1	420	19:45711450:45711510	ENSG00000177051	FBXO46	6.875791268	0.018578003	-0.433479597	0.94398465	-1.283940288	1.377464247	-0.850460691	-2.227924938	9.961963646	0.673902593	0.367508407	0.228220367	0.198525819	0.415042096	0.050115917	-6.896785124	-5.732984949	-6.21452565	-6.200339806	-5.925747535	-4.874654656	1.718642485	0.226232759

DGKI	-0.019051389	0.082419128	0.138070986	0.101470517	0.157122375	0.055651858	8	0.149113648	0.816496581	0.816496581	0.816496581	0.816496581	0.816496581	0.816496581	3	81	0	P_146017	P_146017	P_146017	chr7	137581849	137581909	-	DGKI	"diacylglycerol kinase, iota [Source:HGNC Symbol;Acc:HGNC:2855]"	ENSG00000157680	protein_coding	exon	coding exon	1	421	7:137581849:137581909	ENSG00000157680	DGKI	3.580354163	0.020062505	-0.164732657	0.712657831	1.193865697	0.877390489	1.358598354	0.481207865	9.961963646	0.872449053	0.49241198	0.260184435	0.400938974	0.20423988	0.640763441	-6.983572248	-5.921224194	-6.320002387	-6.744194647	-5.398698281	-6.946334021	0.80124965	0.521167037

HOOK1	-0.068235773	-0.128310104	-0.133774236	-0.060074331	-0.065538463	-0.005464132	8	0.078395983	0.816496581	0.816496581	0.816496581	0.816496581	0.816496581	0.816496581	3	82	0	P_201961	P_201961	P_201961	chr1	59875911	59875971	-	HOOK1	hook microtubule-tethering protein 1 [Source:HGNC Symbol;Acc:HGNC:19884]	ENSG00000134709	protein_coding	3'UTR	3'UTR antisense	0	422	1:59875911:59875971	ENSG00000134709	HOOK1	9.817132609	0.007142201	-0.988875719	-1.859475466	-1.938661898	-0.870599747	-0.949786179	-0.079186432	9.961963646	0.346133584	0.092708091	0.08137179	0.404463606	0.364693496	0.938452417	-6.489535776	-4.628086981	-5.307931861	-6.750220937	-5.838050123	-7.067954019	1.643373725	0.241503443

SLC36A3	-0.162512387	-0.292236599	-0.055903003	-0.129724212	0.106609384	0.236333596	8	0.17994579	0.816496581	0.816496581	0.816496581	0.816496581	0.816496581	0.816496581	3	83	0	A_32_P812442	A_32_P812442	A_32_P812442	chr5	151277513	151277573	-	SLC36A3	"solute carrier family 36, member 3 [Source:HGNC Symbol;Acc:HGNC:19659]"	ENSG00000186334	protein_coding	exon	coding exon	1	423	5:151277513:151277573	ENSG00000186334	SLC36A3	4.459078433	0.028209981	-1.185033709	-2.130977376	-0.407642421	-0.945943667	0.777391287	1.723334955	9.961963646	0.263503469	0.059026938	0.692158462	0.366556143	0.454992548	0.115665962	-6.28186499	-4.227069901	-6.957126297	-6.681140686	-5.984930529	-5.65282026	1.763428635	0.217675083

KDM5A	0.169799982	-0.112366324	0.016710222	-0.282166306	-0.15308976	0.129076546	8	0.052705973	0.816496581	0.816496581	0.816496581	0.816496581	0.816496581	0.816496581	3	84	0	P_214003	P_214003	P_214003	chr12	385897	385957	-	KDM5A	lysine (K)-specific demethylase 5A [Source:HGNC Symbol;Acc:HGNC:9886]	ENSG00000073614	protein_coding	exon	coding exon	1	424	12:385897:385957	ENSG00000073614	KDM5A	8.32952685	0.004437505	3.121863063	-2.065914679	0.307226335	-5.187777741	-2.814636728	2.373141013	9.961963646	0.01088783	0.065838265	0.7649974	0.000413508	0.018392552	0.039156865	-3.30262996	-4.325077455	-6.995818803	-0.065071615	-3.232666682	-4.63721798	9.095906089	0.003341515

GORAB	-0.007467429	0.103148239	0.099779557	0.110615668	0.107246987	-0.003368681	8	0.05274034	0.816496581	0.816496581	0.816496581	0.816496581	0.816496581	0.816496581	3	86	0	A_23_P368145	A_23_P368145	A_23_P368145	chr1	170552994	170553054	+	GORAB	"golgin, RAB6-interacting [Source:HGNC Symbol;Acc:HGNC:25676]"	ENSG00000120370	protein_coding	3'UTR;exon	multiple Ref	1	426	1:170552994:170553054	ENSG00000120370	GORAB	5.950080045	0.004440415	-0.137247665	1.895813719	1.833899022	2.033061384	1.971146687	-0.061914697	9.961963646	0.893569308	0.087334752	0.096672075	0.06955565	0.077107362	0.951855367	-6.988090942	-4.575729115	-5.466525206	-5.261066696	-4.5623394	-7.069284715	2.499787248	0.119283383

MAGED1	-0.019044404	-0.117873755	-0.171988384	-0.098829351	-0.15294398	-0.05411463	8	0.081652995	0.816496581	0.816496581	0.816496581	0.816496581	0.816496581	0.816496581	3	87	0	P_270288	P_270288	P_270288	chrX	51900236	51900296	+	MAGED1	"melanoma antigen family D, 1 [Source:HGNC Symbol;Acc:HGNC:6813]"	ENSG00000179222	protein_coding	exon	coding exon	1	427	X:51900236:51900296	ENSG00000179222	MAGED1	9.414775067	0.007560818	-0.268243132	-1.660268552	-2.422480784	-1.392025421	-2.154237653	-0.762212232	9.961963646	0.793984799	0.127962943	0.035995746	0.194208396	0.056759528	0.46359767	-6.959237652	-4.906281544	-4.532463363	-6.181867606	-4.284873636	-6.762966833	2.642716886	0.10687199

VCL	-0.179019555	-0.03539995	-0.267979624	0.143619605	-0.088960069	-0.232579674	8	0.082820618	0.816496581	0.816496581	0.816496581	0.816496581	0.816496581	0.816496581	3	88	0	P_106345	P_106345	P_106345	chr10	74122166	74122226	+	VCL;AP3M1	"vinculin [Source:HGNC Symbol;Acc:HGNC:12665];adaptor-related protein complex 3, mu 1 subunit [Source:HGNC Symbol;Acc:HGNC:569]"	ENSG00000035403	protein_coding	3'ext;3'UTR	multiple non-Ref	0	428	10:74122166:74122226	ENSG00000035403	VCL	8.257349056	0.00771504	-2.496186461	-0.493604605	-3.736614743	2.002581856	-1.240428282	-3.243010138	9.961963646	0.031735413	0.632285975	0.003893617	0.073181475	0.2432368	0.008867181	-4.363662702	-6.055254588	-2.30181244	-5.308699219	-5.537558833	-3.162353623	6.08383221	0.012703992

PPP1R12A	-0.039966532	-0.196494446	0.019330474	-0.156527914	0.059297005	0.215824919	8	0.087471979	0.816496581	0.816496581	0.816496581	0.816496581	0.816496581	0.816496581	3	89	0	P_216396	P_216396	P_216396	chr12	79832332	79832392	-	PPP1R12A	"protein phosphatase 1, regulatory subunit 12A [Source:HGNC Symbol;Acc:HGNC:7618]"	ENSG00000058272	protein_coding	exon	coding exon	1	429	12:79832332:79832392	ENSG00000058272	PPP1R12A	9.452035712	0.008351133	-0.535635548	-2.633438679	0.25906899	-2.097803131	0.794704538	2.892507669	9.961963646	0.603962173	0.025088444	0.800856604	0.062411234	0.445305378	0.016097902	-6.844028462	-3.443515311	-7.010591295	-5.158933486	-5.971327218	-3.7621262	3.447992559	0.059774161

FAM222B	0.168558104	-0.305426671	-0.002772687	-0.473984775	-0.171330791	0.302653984	8	0.126048799	0.816496581	0.816496581	0.816496581	0.816496581	0.816496581	0.816496581	3	90	0	P_120948	P_120948	P_120948	chr17	28755990	28756050	-	FAM222B	"family with sequence similarity 222, member B [Source:HGNC Symbol;Acc:HGNC:25563]"	ENSG00000173065	protein_coding	3'UTR	3'UTR	1	430	17:28755990:28756050	ENSG00000173065	FAM222B	8.313585667	0.014965855	1.687502787	-3.057748911	-0.027758482	-4.745251699	-1.715261269	3.02999043	9.961963646	0.122514914	0.012142152	0.978402988	0.000794159	0.117178171	0.012730045	-5.633111371	-2.76510469	-7.046699823	-0.746367628	-4.931963709	-3.527034317	7.80644025	0.005672329

CBY2	0.18659976	0.28469203	0.160241869	0.09809227	-0.02635789	-0.12445016	8	0.234164743	0.816496581	0.816496581	0.816496581	0.816496581	0.816496581	0.816496581	3	91	0	P_219030	P_219030	P_219030	chr13	45702405	45702465	+	SPERT	spermatid associated [Source:HGNC Symbol;Acc:HGNC:30720]	ENSG00000174015	protein_coding	exon	coding exon	1	431	13:45702405:45702465	ENSG00000174015	CBY2	3.512276814	0.046240675	1.062783075	1.621469776	0.912661125	0.558686701	-0.15012195	-0.708808651	9.961963646	0.312964279	0.136099873	0.382976475	0.588712417	0.883664381	0.494695932	-6.414769342	-4.958531244	-6.610390837	-6.981554518	-6.284712329	-6.803654522	0.90478326	0.472815693

TLCD2	0.293255082	0.019595862	0.153940053	-0.27365922	-0.139315029	0.134344191	8	0.087753871	0.816496581	0.816496581	0.816496581	0.816496581	0.816496581	0.816496581	3	92	0	A_24_P503710	A_24_P503710	A_24_P503710	chr17	1705056	1705116	-	TLCD2	TLC domain containing 2 [Source:HGNC Symbol;Acc:HGNC:33522]	ENSG00000185561	protein_coding	3'UTR	3'UTR	1	432	17:1705056:1705116	ENSG00000185561	TLCD2	8.908954819	0.0083908	3.920933732	0.262004244	2.05823798	-3.658929488	-1.862695753	1.796233736	9.961963646	0.002882946	0.798656007	0.066689617	0.004424692	0.092219799	0.102794996	-1.950923213	-6.146028385	-5.122202641	-2.525177697	-4.72191532	-5.546135631	6.62709454	0.009713535

BIRC6	0.006535532	-0.11692466	-0.034412335	-0.123460192	-0.040947866	0.082512325	8	0.055090289	0.816496581	0.816496581	0.816496581	0.816496581	0.816496581	0.816496581	3	93	0	P_234326	P_234326	P_234326	chr2	32518886	32518946	+	BIRC6	baculoviral IAP repeat containing 6 [Source:HGNC Symbol;Acc:HGNC:13516]	ENSG00000115760	protein_coding	exon	coding exon	1	433	2:32518886:32518946	ENSG00000115760	BIRC6	7.481723305	0.004643906	0.117458601	-2.101406277	-0.618469163	-2.218864877	-0.735927764	1.482937114	9.961963646	0.908831071	0.062034875	0.550144622	0.050890417	0.478744471	0.169021557	-6.990836914	-4.27174638	-6.842170717	-4.964784537	-6.016429786	-5.986654803	2.079377078	0.166912424

MYD88	-0.106078516	-0.079047076	-0.091767572	0.027031439	0.014310943	-0.012720496	8	0.080703691	0.816496581	0.816496581	0.816496581	0.816496581	0.816496581	0.816496581	3	94	0	P_134406	P_134406	P_134406	chr3	38142518	38142578	-	MYD88	myeloid differentiation primary response 88 [Source:HGNC Symbol;Acc:HGNC:7562]	ENSG00000172936	protein_coding	3'ext;3'UTR;exon	multiple non-Ref	0	434	3:38142518:38142578	ENSG00000172936	MYD88	8.735450639	0.007437047	-1.506512716	-1.122615877	-1.303270637	0.383896838	0.203242078	-0.18065476	9.961963646	0.162969894	0.287928091	0.221798055	0.709121269	0.843037139	0.860260856	-5.883622305	-5.558113403	-6.191171036	-7.069611546	-6.27476137	-7.053578634	0.908280265	0.471265962

MANSC1	-0.208915368	-0.013264442	-0.280501352	0.195650927	-0.071585984	-0.26723691	8	0.069905999	0.816496581	0.816496581	0.816496581	0.816496581	0.816496581	0.816496581	3	97	0	P_111003	P_111003	P_111003	chr12	12329945	12330005	-	MANSC1	MANSC domain containing 1 [Source:HGNC Symbol;Acc:HGNC:25505]	ENSG00000111261	protein_coding	3'UTR	3'UTR	1	437	12:12329945:12330005	ENSG00000111261	MANSC1	9.809931109	0.00613109	-3.267737599	-0.207474993	-4.38744561	3.060262606	-1.11970801	-4.179970617	9.961963646	0.008504442	0.839818717	0.001373915	0.0120903	0.289107791	0.001903495	-3.053511023	-6.159445791	-1.228712157	-3.548204966	-5.669863877	-1.586575851	9.676990246	0.002677824

HUWE1	0.020647245	-0.094534774	-0.084423129	-0.115182018	-0.105070374	0.010111645	8	0.051440957	0.816496581	0.816496581	0.816496581	0.816496581	0.816496581	0.816496581	3	98	0	P_270330	P_270330	P_270330	chrX	53544614	53544674	-	HUWE1	"HECT, UBA and WWE domain containing 1, E3 ubiquitin protein ligase [Source:HGNC Symbol;Acc:HGNC:30892]"	ENSG00000086758	protein_coding	exon	coding exon	1	438	X:53544614:53544674	ENSG00000086758	HUWE1	8.20965789	0.004331704	0.38421855	-1.75917	-1.571005356	-2.143388551	-1.955223907	0.188164644	9.961963646	0.708890306	0.109165756	0.147371479	0.05780651	0.079170713	0.854525247	-6.918398985	-4.770134939	-5.844019855	-5.086287261	-4.586011375	-7.052070674	2.360463626	0.13304587

CDKL5	0.26393908	-0.032382255	0.080170967	-0.296321335	-0.183768113	0.112553222	8	0.105328031	0.816496581	0.816496581	0.816496581	0.816496581	0.816496581	0.816496581	3	99	0	P_269655	P_269655	P_269655	chrX	18579908	18579968	+	CDKL5	cyclin-dependent kinase-like 5 [Source:HGNC Symbol;Acc:HGNC:11411]	ENSG00000008086	protein_coding	exon	coding exon	1	439	X:18579908:18579968	ENSG00000008086	CDKL5	5.198109375	0.011115766	3.066052777	-0.376169016	0.931307392	-3.442221793	-2.134745386	1.307476407	9.961963646	0.011971719	0.714678438	0.373713775	0.006342865	0.058653833	0.220420709	-3.397984897	-6.108037765	-6.593101197	-2.893780976	-4.314835872	-6.209984964	4.754173252	0.026188845

FPGS	-0.278375379	-0.006520236	-0.183756019	0.271855143	0.09461936	-0.177235783	8	0.107605974	0.816496581	0.816496581	0.816496581	0.816496581	0.816496581	0.816496581	3	100	0	P_149736	P_149736	P_149736	chr9	127813397	127813457	+	FPGS	folylpolyglutamate synthase [Source:HGNC Symbol;Acc:HGNC:3824]	ENSG00000136877	protein_coding	exon	coding exon	1	440	9:127813397:127813457	ENSG00000136877	FPGS	9.218610559	0.011505289	-3.178540025	-0.074449224	-2.098159195	3.1040908	1.080380829	-2.023709971	9.961963646	0.009889513	0.942126693	0.062373945	0.011221656	0.305433579	0.070649616	-3.205802856	-6.179166323	-5.059195502	-3.473010486	-5.710733334	-5.199777706	4.901489311	0.024046117

STK38L	0.184048142	0.014151434	0.128766132	-0.169896708	-0.05528201	0.114614698	8	0.068115786	0.816496581	0.816496581	0.816496581	0.816496581	0.816496581	0.816496581	3	101	0	P_214830	P_214830	P_214830	chr12	27315057	27315117	+	STK38L	serine/threonine kinase 38 like [Source:HGNC Symbol;Acc:HGNC:17848]	ENSG00000211455	protein_coding	3'UTR;exon	multiple Ref	1	441	12:27315057:27315117	ENSG00000211455	STK38L	6.657344103	0.005932665	2.926524559	0.225020041	2.04749281	-2.701504518	-0.879031749	1.822472769	9.961963646	0.01518849	0.826512319	0.067898839	0.022326219	0.400090311	0.098493081	-3.636197611	-6.155465039	-5.139080118	-4.16134907	-5.901361639	-5.507184381	4.033272882	0.040634848

LTBP1	0.320338822	0.147444617	0.534386516	-0.172894204	0.214047694	0.386941899	8	0.060874285	0.816496581	0.816496581	0.816496581	0.816496581	0.816496581	0.816496581	3	102	0	P_127587	P_127587	P_127587	chr2	33398485	33398545	+	LTBP1	latent transforming growth factor beta binding protein 1 [Source:HGNC Symbol;Acc:HGNC:6714]	ENSG00000049323	protein_coding	exon	coding exon	1	442	2:33398485:33398545	ENSG00000049323	LTBP1	5.714594756	0.005182546	5.449838374	2.508435689	9.091374333	-2.941402684	3.641535959	6.582938643	9.961963646	0.000284699	0.031077298	3.88E-06	0.014807265	0.004553617	6.32E-05	0.44235565	-3.641681593	4.846390716	-3.751974947	-1.89531978	1.954887708	30.64896504	2.42E-05

MECOM	0.446503475	-0.262781982	0.22043485	-0.709285457	-0.226068625	0.483216832	8	0.103834321	0.816496581	0.816496581	0.816496581	0.816496581	0.816496581	0.816496581	3	103	0	P_312108	P_312108	P_312108	chr3	169552731	169552791	+	MECOM	MDS1 and EVI1 complex locus [Source:HGNC Symbol;Acc:HGNC:3498]	ENSG00000085276	protein_coding	intron	intron antisense	0	443	3:169552731:169552791	ENSG00000085276	MECOM	5.399253642	0.010864869	5.246361544	-3.087656339	2.590082689	-8.334017882	-2.656278855	5.677739027	9.961963646	0.000380083	0.011539655	0.027023036	8.42E-06	0.024125453	0.000207418	0.142594985	-2.717257426	-4.252456618	4.002457564	-3.489357719	0.717141351	25.41914189	5.51E-05

CCRL2	-0.419850288	-0.117759186	-0.365084467	0.302091102	0.054765821	-0.247325281	8	0.469219404	0.816496581	0.816496581	0.816496581	0.816496581	0.816496581	0.816496581	3	104	0	A_24_P97405	A_24_P97405	A_24_P97405	chr3	46408710	46408770	+	CCRL2	chemokine (C-C motif) receptor-like 2 [Source:HGNC Symbol;Acc:HGNC:1612]	ENSG00000121797	protein_coding	exon	coding exon	1	444	3:46408710:46408770	ENSG00000121797	CCRL2	3.062169271	0.179012668	-1.215341145	-0.34087766	-1.056810457	0.874463485	0.158530688	-0.715932797	9.961963646	0.252253558	0.740275491	0.315552068	0.402455569	0.877206281	0.490473901	-6.24722293	-6.121200674	-6.469265717	-6.746797068	-6.283335286	-6.798380186	0.66874636	0.590339364

ADAM12	0.153369793	0.05709521	-0.077137158	-0.096274582	-0.23050695	-0.134232368	8	0.142550314	0.816496581	0.816496581	0.816496581	0.816496581	0.816496581	0.816496581	3	105	0	A_23_P202327	A_23_P202327	A_23_P202327	chr10	126015624	126015684	-	ADAM12	ADAM metallopeptidase domain 12 [Source:HGNC Symbol;Acc:HGNC:190]	ENSG00000148848	protein_coding	3'UTR	3'UTR	1	445	10:126015624:126015684	ENSG00000148848	ADAM12	3.93120188	0.018525227	1.380078173	0.513763839	-0.694108701	-0.866314334	-2.074186875	-1.20787254	9.961963646	0.197744861	0.618619162	0.503478337	0.406698884	0.064932441	0.254989376	-6.048014302	-6.044825987	-6.790221913	-6.754003258	-4.407313057	-6.32794752	1.527010621	0.267499797

NRAP	0.075426979	0.111225944	0.177214795	0.035798965	0.101787816	0.065988851	8	0.109474495	0.816496581	0.816496581	0.816496581	0.816496581	0.816496581	0.816496581	3	106	0	A_24_P942161	A_24_P942161	A_24_P942161	chr10	113620677	113621898	-	NRAP	nebulin-related anchoring protein [Source:HGNC Symbol;Acc:HGNC:7988]	ENSG00000197893	protein_coding	"exon,intron"	multiple non-Ref	0	446	10:113620677:113621898	ENSG00000197893	NRAP	4.127998206	0.011831024	0.849300122	1.252392826	1.995420581	0.403092704	1.146120459	0.743027755	9.961963646	0.41565743	0.239029577	0.074058905	0.69539514	0.278529933	0.474622401	-6.618455352	-5.416660151	-5.220357818	-7.061477342	-5.641786335	-6.777887706	1.383277977	0.304128466

DCP1B	0.0401741	-0.115245713	-0.038586405	-0.155419813	-0.078760504	0.076659309	8	0.105222076	0.816496581	0.816496581	0.816496581	0.816496581	0.816496581	0.816496581	3	107	0	P_309349	P_309349	P_309349	chr12	1977585	1977645	-	DCP1B;CACNA1C	"decapping mRNA 1B [Source:HGNC Symbol;Acc:HGNC:24451];calcium channel, voltage-dependent, L type, alpha 1C subunit [Source:HGNC Symbol;Acc:HGNC:1390]"	ENSG00000151065	protein_coding	intron	multiple non-Ref	0	447	12:1977585:1977645	ENSG00000151065	DCP1B	5.540657649	0.011097851	0.467059637	-1.339833907	-0.448601269	-1.806893544	-0.915660906	0.891232638	9.961963646	0.650505265	0.210059697	0.663319198	0.10102689	0.381475409	0.393820653	-6.880615584	-5.315140772	-6.938323147	-5.606745682	-5.869099023	-6.653999376	1.185356248	0.364176538

PLPP1	-0.156944506	0.140907467	0.125994548	0.297851973	0.282939054	-0.014912919	8	0.096517062	0.816496581	0.816496581	0.816496581	0.816496581	0.816496581	0.816496581	3	109	0	P_252876	P_252876	P_252876	chr5	55437535	55437595	-	PPAP2A	phosphatidic acid phosphatase type 2A [Source:HGNC Symbol;Acc:HGNC:9228]	ENSG00000067113	protein_coding	3'ext;intron	multiple non-Ref	0	449	5:55437535:55437595	ENSG00000067113	PLPP1	8.106659788	0.009687573	-1.952919085	1.753364228	1.567797204	3.706283312	3.520716288	-0.185567024	9.961963646	0.079473643	0.110195762	0.148115199	0.004092595	0.005564051	0.856508169	-5.238867295	-4.778238868	-5.848420869	-2.445064578	-2.088269333	-7.052599167	5.918763572	0.013823278

IPO5	-0.241929235	-0.065097944	-0.27092409	0.176831291	-0.028994855	-0.205826146	8	0.066973045	0.816496581	0.816496581	0.816496581	0.816496581	0.816496581	0.816496581	3	110	0	P_219674	P_219674	P_219674	chr13	98022909	98022969	-	IPO5	importin 5 [Source:HGNC Symbol;Acc:HGNC:6402]	ENSG00000065150	protein_coding	3'ext;3'UTR	multiple non-Ref	0	450	13:98022909:98022969	ENSG00000065150	IPO5	8.353764363	0.005808696	-3.887717214	-1.046100933	-4.353654278	2.841616281	-0.465937064	-3.307553345	9.961963646	0.003042458	0.320232859	0.001448257	0.017562361	0.651281216	0.00795211	-2.006171992	-5.635962959	-1.283226757	-3.922677706	-6.182495544	-3.051981138	9.065794572	0.003380988

FRY	-0.062314343	-0.083734611	-0.125940789	-0.021420268	-0.063626445	-0.042206178	8	0.044522683	0.816496581	0.816496581	0.816496581	0.816496581	0.816496581	0.816496581	3	111	0	P_309665	P_309665	P_309665	chr13	32267464	32267524	-	FRY	furry homolog (Drosophila) [Source:HGNC Symbol;Acc:HGNC:20367]	ENSG00000073910	protein_coding	intron	intron antisense	0	451	13:32267464:32267524	ENSG00000073910	FRY	6.376960559	0.003798554	-1.23829603	-1.663954569	-2.502665845	-0.425658539	-1.264369815	-0.838711276	9.961963646	0.243992867	0.127213054	0.031385597	0.679406041	0.234877579	0.421300385	-6.220555188	-4.901282452	-4.399010389	-7.0514246	-5.510145095	-6.700141312	2.174785171	0.154394326

ACVR1B	-0.132039724	-0.133861943	-0.068648091	-0.001822219	0.063391633	0.065213852	8	0.068871093	0.816496581	0.816496581	0.816496581	0.816496581	0.816496581	0.816496581	3	112	0	P_215504	P_215504	P_215504	chr12	51976434	51976494	+	ACVR1B	"activin A receptor, type IB [Source:HGNC Symbol;Acc:HGNC:172]"	ENSG00000135503	protein_coding	exon	coding exon	1	452	12:51976434:51976494	ENSG00000135503	ACVR1B	6.364378256	0.006015754	-2.084995846	-2.113769908	-1.083999421	-0.028774061	1.000996425	1.029770487	9.961963646	0.063766694	0.06075986	0.3039024	0.977613069	0.340522022	0.327471069	-5.033073572	-4.253093283	-6.440782169	-7.149160306	-5.789703065	-6.521073143	2.008922449	0.176920007

DNMBP	0.002025779	-0.061832874	-0.091309657	-0.063858653	-0.093335435	-0.029476783	8	0.04926296	0.816496581	0.816496581	0.816496581	0.816496581	0.816496581	0.816496581	3	113	0	P_209476	P_209476	P_209476	chr10	99886479	99886539	-	DNMBP	dynamin binding protein [Source:HGNC Symbol;Acc:HGNC:30373]	ENSG00000107554	protein_coding	exon	coding exon	1	453	10:99886479:99886539	ENSG00000107554	DNMBP	5.390587305	0.004155568	0.038487738	-1.174761902	-1.734790878	-1.21324964	-1.773278616	-0.560028976	9.961963646	0.970059176	0.267405945	0.113550645	0.253017303	0.10669927	0.58783078	-6.997552041	-5.502655615	-5.612531308	-6.399160433	-4.850331172	-6.902700507	1.553528403	0.261304916

ITGB1BP1	0.016216206	-0.080586528	0.053351503	-0.096802734	0.037135297	0.133938031	8	0.062548734	0.816496581	0.816496581	0.816496581	0.816496581	0.816496581	0.816496581	3	114	0	P_233539	P_233539	P_233539	chr2	9418625	9418685	-	ITGB1BP1	integrin beta 1 binding protein 1 [Source:HGNC Symbol;Acc:HGNC:23927]	ENSG00000119185	protein_coding	exon	coding exon	1	454	2:9418625:9418685	ENSG00000119185	ITGB1BP1	9.396279998	0.00534851	0.271567941	-1.349558395	0.893461612	-1.621126337	0.62189367	2.243020007	9.961963646	0.791498906	0.207026524	0.392682715	0.136173917	0.547979497	0.048850751	-6.958265428	-5.3035597	-6.627884405	-5.875081672	-6.094999227	-4.850188568	1.789878625	0.212796734

ATAD1	0.019668853	0.124241075	0.047692009	0.104572222	0.028023156	-0.076549066	8	0.036344362	0.816496581	0.816496581	0.816496581	0.816496581	0.816496581	0.816496581	3	115	0	P_106523	P_106523	P_106523	chr10	87754337	87754397	-	ATAD1	"ATPase family, AAA domain containing 1 [Source:HGNC Symbol;Acc:HGNC:25903]"	ENSG00000138138	protein_coding	3'UTR	3'UTR	1	455	10:87754337:87754397	ENSG00000138138	ATAD1	7.972388365	0.003267449	0.421424976	2.661990074	1.021849296	2.240565098	0.60042432	-1.640140778	9.961963646	0.682393368	0.023890478	0.331025947	0.04905438	0.561633388	0.132128143	-6.902306647	-3.398071078	-6.505040781	-4.929588322	-6.108435857	-5.77163505	2.729778206	0.100058046

KIAA1549L	0.035537346	0.114441503	-0.093731382	0.078904157	-0.129268729	-0.208172885	8	0.062158943	0.816496581	0.816496581	0.816496581	0.816496581	0.816496581	0.816496581	3	116	0	P_211475	P_211475	P_211475	chr11	33552181	33552241	+	KIAA1549L	KIAA1549-like [Source:HGNC Symbol;Acc:HGNC:24836]	ENSG00000110427	protein_coding	exon	coding exon	1	456	11:33552181:33552241	ENSG00000110427	KIAA1549L	3.502000103	0.005309473	0.597317042	1.923549938	-1.575451126	1.326232896	-2.172768168	-3.499001064	9.961963646	0.563625205	0.083430027	0.146346302	0.214364242	0.055013215	0.005769048	-6.807097361	-4.535465907	-5.837912146	-6.264272923	-4.256305641	-2.72552962	4.210283653	0.036352938

OPA1	-0.053400078	0.01491751	0.073715041	0.068317588	0.127115119	0.058797531	8	0.064221244	0.816496581	0.816496581	0.816496581	0.816496581	0.816496581	0.816496581	3	117	0	P_136891	P_136891	P_136891	chr3	193697438	193697498	+	OPA1	optic atrophy 1 (autosomal dominant) [Source:HGNC Symbol;Acc:HGNC:8140]	ENSG00000198836	protein_coding	3'UTR;exon	multiple Ref	1	457	3:193697438:193697498	ENSG00000198836	OPA1	9.104235862	0.005518777	-0.88037178	0.245935131	1.215291153	1.126306911	2.095662933	0.969356022	9.961963646	0.399398335	0.810725365	0.252271792	0.286436074	0.062635814	0.355313249	-6.591193222	-6.150302365	-6.295424245	-6.496868069	-4.374626285	-6.580992441	1.485415438	0.277557366

RSKR	-0.226806522	-0.236534513	-0.137748612	-0.00972799	0.089057911	0.098785901	8	0.177413565	0.816496581	0.816496581	0.816496581	0.816496581	0.816496581	0.816496581	3	118	0	P_120914	P_120914	P_120914	chr17	28610869	28610929	-	SGK494	uncharacterized serine/threonine-protein kinase SgK494 [Source:EntrezGene;Acc:124923]	ENSG00000167524	protein_coding	exon	non-coding exon	1	458	17:28610869:28610929	ENSG00000167524	RSKR	5.72345091	0.027483285	-1.675586474	-1.747454288	-1.017650234	-0.071867814	0.65793624	0.729804054	9.961963646	0.124872465	0.111253344	0.332922071	0.944129494	0.525490262	0.482317842	-5.650105932	-4.786474348	-6.509272326	-7.146789244	-6.071465781	-6.787974437	1.311242926	0.32459721

USP9X	0.027938292	-0.114938137	0.00436161	-0.142876429	-0.023576682	0.119299747	8	0.079216086	0.816496581	0.816496581	0.816496581	0.816496581	0.816496581	0.816496581	3	119	0	P_270083	P_270083	P_270083	chrX	41197444	41197504	+	USP9X	"ubiquitin specific peptidase 9, X-linked [Source:HGNC Symbol;Acc:HGNC:12632]"	ENSG00000124486	protein_coding	3'UTR;exon	multiple Ref	1	459	X:41197444:41197504	ENSG00000124486	USP9X	8.988138843	0.007246002	0.401972414	-1.653714545	0.062754264	-2.05568696	-0.33921815	1.71646881	9.961963646	0.696193106	0.129306106	0.951203459	0.066974835	0.741487675	0.116950863	-6.910899603	-4.915155385	-7.044970395	-5.225518212	-6.235844064	-5.662748029	1.697912453	0.230324723

NOP9	-0.246647626	0.099054964	-0.048938424	0.34570259	0.197709203	-0.147993388	8	0.07888282	0.816496581	0.816496581	0.816496581	0.816496581	0.816496581	0.816496581	3	121	0	P_220283	P_220283	P_220283	chr14	24300410	24300470	+	NOP9	NOP9 nucleolar protein [Source:HGNC Symbol;Acc:HGNC:19826]	ENSG00000196943	protein_coding	exon	coding exon	1	461	14:24300410:24300470	ENSG00000196943	NOP9	7.690154721	0.00720369	-3.559139864	1.429368998	-0.706184355	4.988508862	2.85295551	-2.135553353	9.961963646	0.005219626	0.18349877	0.496256835	0.000552837	0.01722482	0.058574124	-2.558030686	-5.206418374	-6.78142382	-0.368447311	-3.170380931	-5.023140453	8.799072097	0.0037562

MARCHF1	0.250685822	-0.026429106	0.165757176	-0.277114928	-0.084928646	0.192186281	8	0.105469476	0.816496581	0.816496581	0.816496581	0.816496581	0.816496581	0.816496581	3	123	0	A_24_P800978	A_24_P800978	A_24_P800978	chr4	163936691	163936751	-	Mar-01	"membrane-associated ring finger (C3HC4) 1, E3 ubiquitin protein ligase [Source:HGNC Symbol;Acc:HGNC:26077]"	ENSG00000145416	protein_coding	intron	intron sense	0	463	4:163936691:163936751	ENSG00000145416	MARCHF1	4.58979825	0.01113971	2.908964723	-0.306684021	1.923450527	-3.215648745	-0.985514196	2.230134548	9.961963646	0.01565125	0.765398176	0.083443726	0.009287128	0.347701883	0.049928763	-3.666139319	-6.13274658	-5.331211701	-3.281624964	-5.80453857	-4.871076681	4.756821327	0.026148364

STRA6	0.069021765	-0.031263253	0.209541144	-0.100285018	0.140519379	0.240804397	8	0.264409269	0.816496581	0.816496581	0.816496581	0.816496581	0.816496581	0.816496581	3	124	0	A_23_P65779	A_23_P65779	A_23_P65779	chr15	74179724	74179784	-	STRA6	stimulated by retinoic acid 6 [Source:HGNC Symbol;Acc:HGNC:30650]	ENSG00000137868	protein_coding	3'UTR;exon	multiple Ref	1	464	15:74179724:74179784	ENSG00000137868	STRA6	6.505968864	0.058350042	0.349954113	-0.158510928	1.06241539	-0.508465041	0.712461277	1.220926319	9.961963646	0.733658897	0.877221447	0.31312312	0.622196988	0.492528449	0.250223148	-6.93194133	-6.168859233	-6.463440875	-7.010042392	-6.033573485	-6.312876263	0.588853151	0.636143024

GBF1	-0.236967078	-0.119572417	-0.195687922	0.117394661	0.041279156	-0.076115505	8	0.096284784	0.816496581	0.816496581	0.816496581	0.816496581	0.816496581	0.816496581	3	126	0	P_209645	P_209645	P_209645	chr10	102369223	102369283	-	GBF1	golgi brefeldin A resistant guanine nucleotide exchange factor 1 [Source:HGNC Symbol;Acc:HGNC:4181]	ENSG00000107862	protein_coding	exon	exon antisense	0	466	10:102369223:102369283	ENSG00000107862	GBF1	7.502839753	0.009651609	-2.954158361	-1.490653715	-2.439550317	1.463504646	0.514608044	-0.948896602	9.961963646	0.014488156	0.167019642	0.034961569	0.17415519	0.618050103	0.365124107	-3.589056612	-5.129402164	-4.504140918	-6.08932926	-6.157731101	-6.600607374	3.367929654	0.063157083

PTPRG	0.063448815	-0.065467235	0.028889093	-0.128916049	-0.034559722	0.094356328	8	0.108067078	0.816496581	0.816496581	0.816496581	0.816496581	0.816496581	0.816496581	3	128	0	A_23_P41054	A_23_P41054	A_23_P41054	chr3	62293542	62293602	+	PTPRG	"protein tyrosine phosphatase, receptor type, G [Source:HGNC Symbol;Acc:HGNC:9671]"	ENSG00000144724	protein_coding	3'UTR	3'UTR	1	468	3:62293542:62293602	ENSG00000144724	PTPRG	6.176386461	0.011585151	0.721968575	-0.744935687	0.328721936	-1.466904262	-0.393246639	1.073657624	9.961963646	0.486914462	0.473518557	0.749171673	0.173247484	0.702421603	0.30829414	-6.721174702	-5.897730259	-6.988429075	-6.084852966	-6.215085656	-6.475719203	0.77390294	0.53475855

EFCAB1	0.000118836	0.088102088	0.047151329	0.087983252	0.047032493	-0.040950759	8	0.25160972	0.816496581	0.816496581	0.816496581	0.816496581	0.816496581	0.816496581	3	129	0	A_23_P146294	A_23_P146294	A_23_P146294	chr8	48723520	48723580	-	EFCAB1	EF-hand calcium binding domain 1 [Source:HGNC Symbol;Acc:HGNC:25678]	ENSG00000034239	protein_coding	3'UTR	3'UTR	1	469	8:48723520:48723580	ENSG00000034239	EFCAB1	3.069657226	0.053046019	0.000631929	0.46849551	0.250733966	0.467863581	0.250102037	-0.217761544	9.961963646	0.999508269	0.649513391	0.807115388	0.649949827	0.807590493	0.832010539	-6.9983601	-6.067688328	-7.012896685	-7.031210036	-6.263522677	-7.045534661	0.101870569	0.957089944

HNRNPA3	-0.052908871	-0.179311915	-0.205444908	-0.126403044	-0.152536037	-0.026132993	8	0.10911689	0.816496581	0.816496581	0.816496581	0.816496581	0.816496581	0.816496581	3	130	0	P_129316	P_129316	P_129316	chr2	177223849	177223909	+	HNRNPA3	heterogeneous nuclear ribonucleoprotein A3 [Source:HGNC Symbol;Acc:HGNC:24941]	ENSG00000170144	protein_coding	3'UTR	3'UTR	1	470	2:177223849:177223909	ENSG00000170144	HNRNPA3	7.865419372	0.011768249	-0.597335323	-2.024411754	-2.319450366	-1.427076432	-1.722115043	-0.295038611	9.961963646	0.563613475	0.070566957	0.042905547	0.184141407	0.115893311	0.77402162	-6.807085859	-4.38701728	-4.702299427	-6.136870806	-4.922393216	-7.024032042	2.487362931	0.120440354

CASP7	-0.014393084	0.192779875	0.194411333	0.207172959	0.208804417	0.001631457	8	0.033761857	0.816496581	0.816496581	0.816496581	0.816496581	0.816496581	0.816496581	3	132	0	P_107114	P_107114	P_107114	chr10	113729946	113730006	+	CASP7	"caspase 7, apoptosis-related cysteine peptidase [Source:HGNC Symbol;Acc:HGNC:1508]"	ENSG00000165806	protein_coding	3'UTR	3'UTR	1	472	10:113729946:113730006	ENSG00000165806	CASP7	7.271395584	0.003122056	-0.315485305	4.225586268	4.261346556	4.541071573	4.576831861	0.035760288	9.961963646	0.758902984	0.001770934	0.001673832	0.001083395	0.0010256	0.972179912	-6.944318772	-0.950047423	-1.432870756	-1.069834537	-0.461263406	-7.070679958	12.94736408	0.000899128

NUDT1	0.002054275	-0.010351925	-0.053467628	-0.0124062	-0.055521903	-0.043115703	8	0.129575046	0.816496581	0.816496581	0.816496581	0.816496581	0.816496581	0.816496581	3	133	0	P_144166	P_144166	P_144166	chr7	2251085	2251145	+	NUDT1	nudix (nucleoside diphosphate linked moiety X)-type motif 1 [Source:HGNC Symbol;Acc:HGNC:8048]	ENSG00000106268	protein_coding	3'UTR;exon	multiple Ref	1	473	7:2251085:2251145	ENSG00000106268	NUDT1	10.21300351	0.015689723	0.020086143	-0.101218318	-0.522791976	-0.121304461	-0.54287812	-0.421573659	9.961963646	0.984371268	0.921385383	0.612547283	0.905861802	0.599148665	0.682288355	-6.998140161	-6.176689364	-6.899888127	-7.14157202	-6.142279019	-6.975149046	0.128519304	0.940973807

METTL3	-0.2701158	0.005088244	-0.338978184	0.275204044	-0.068862384	-0.344066428	8	0.094834001	0.816496581	0.816496581	0.816496581	0.816496581	0.816496581	0.816496581	3	134	0	P_220141	P_220141	P_220141	chr14	21499031	21499091	-	METTL3	methyltransferase like 3 [Source:HGNC Symbol;Acc:HGNC:17563]	ENSG00000165819	protein_coding	3'UTR;exon	multiple Ref	1	474	14:21499031:21499091	ENSG00000165819	METTL3	8.84647482	0.009428945	-3.406936721	0.064177387	-4.275489339	3.471114108	-0.868552618	-4.339666726	9.961963646	0.006728919	0.950098517	0.001636993	0.006043842	0.40553033	0.001480264	-2.816319519	-6.17991661	-1.409875215	-2.844474213	-5.910385738	-1.326502195	10.25447829	0.002168892

AEBP2	0.075160638	-0.075949062	0.037847891	-0.1511097	-0.037312747	0.113796953	8	0.078355747	0.816496581	0.816496581	0.816496581	0.816496581	0.816496581	0.816496581	3	135	0	P_111100	P_111100	P_111100	chr12	19522010	19522070	+	AEBP2	AE binding protein 2 [Source:HGNC Symbol;Acc:HGNC:24051]	ENSG00000139154	protein_coding	3'UTR	3'UTR	1	475	12:19522010:19522070	ENSG00000139154	AEBP2	8.127918267	0.007137136	1.089617575	-1.101047506	0.548687826	-2.190665081	-0.540929749	1.649735332	9.961963646	0.301536811	0.296767895	0.59530204	0.053375716	0.6004416	0.130127752	-6.386560898	-5.580489528	-6.885163	-5.010351282	-6.143368953	-5.758100328	1.752312593	0.219763751

PROSER1	-0.145171462	-0.057230897	-0.212555789	0.087940565	-0.067384327	-0.155324892	8	0.095515592	0.816496581	0.816496581	0.816496581	0.816496581	0.816496581	0.816496581	3	136	0	P_218859	P_218859	P_218859	chr13	39024472	39024532	-	PROSER1	proline and serine rich 1 [Source:HGNC Symbol;Acc:HGNC:20291]	ENSG00000120685	protein_coding	exon	coding exon	1	476	13:39024472:39024532	ENSG00000120685	PROSER1	6.747653161	0.009533134	-1.820996187	-0.717890722	-2.666249115	1.103105466	-0.845252927	-1.948358393	9.961963646	0.098730697	0.489317497	0.023716739	0.295915474	0.417808126	0.080076274	-5.438387406	-5.917478661	-4.124007304	-6.52199887	-5.930117937	-5.316575714	2.777945525	0.096508835

NFATC1	0.22413425	0.054956028	0.237441683	-0.169178222	0.013307433	0.182485655	8	0.079478547	0.816496581	0.816496581	0.816496581	0.816496581	0.816496581	0.816496581	3	137	0	P_123825	P_123825	P_123825	chr18	79486877	79486937	+	NFATC1	"nuclear factor of activated T-cells, cytoplasmic, calcineurin-dependent 1 [Source:HGNC Symbol;Acc:HGNC:7775]"	ENSG00000131196	protein_coding	3'UTR;exon	multiple Ref	1	477	18:79486877:79486937	ENSG00000131196	NFATC1	5.780117557	0.00727945	3.217396292	0.788881306	3.408421476	-2.428514987	0.191025184	2.619540171	9.961963646	0.009259706	0.448548363	0.006712194	0.035626766	0.852342851	0.025693097	-3.139442162	-5.864261592	-2.857741805	-4.62050644	-6.277313024	-4.225793049	5.897912657	0.013972905

PPOX	-0.030738125	0.017932662	-0.036881793	0.048670787	-0.006143668	-0.054814454	8	0.082454353	0.816496581	0.816496581	0.816496581	0.816496581	0.816496581	0.816496581	3	138	0	P_103813	P_103813	P_103813	chr1	161172005	161172065	+	PPOX;B4GALT3	"protoporphyrinogen oxidase [Source:HGNC Symbol;Acc:HGNC:9280];UDP-Gal:betaGlcNAc beta 1,4- galactosyltransferase, polypeptide 3 [Source:HGNC Symbol;Acc:HGNC:926]"	ENSG00000143224	protein_coding	3'ext;exon;intron	multiple non-Ref	0	478	1:161172005:161172065	ENSG00000143224	PPOX	7.641939203	0.007666427	-0.429958454	0.250838316	-0.515894791	0.68079677	-0.085936337	-0.766733107	9.961963646	0.676377844	0.807036942	0.617183247	0.511513089	0.933219693	0.461023836	-6.898413466	-6.14902653	-6.903696566	-6.901903116	-6.292758749	-6.759401421	0.26211342	0.851120536

NCOA5	-0.006637004	-0.122956389	-0.056208862	-0.116319385	-0.049571858	0.066747526	8	0.0365839	0.816496581	0.816496581	0.816496581	0.816496581	0.816496581	0.816496581	3	139	0	A_23_P315843	A_23_P315843	A_23_P315843	chr20	46062272	46062332	-	NCOA5	nuclear receptor coactivator 5 [Source:HGNC Symbol;Acc:HGNC:15909]	ENSG00000124160	protein_coding	"3'UTR,exon"	multiple Ref	1	479	20:46062272:46062332	ENSG00000124160	NCOA5	7.846870968	0.003281477	-0.141900193	-2.628827048	-1.201754372	-2.486926855	-1.059854179	1.427072676	9.961963646	0.889987538	0.025287484	0.257248241	0.032242025	0.314231274	0.184142461	-6.987383622	-3.450849984	-6.310990754	-4.523118264	-5.73161095	-6.059786293	2.953285561	0.084785956

MYO3A	-0.101836901	0.203346009	-0.109441081	0.30518291	-0.00760418	-0.31278709	8	0.173427454	0.816496581	0.816496581	0.816496581	0.816496581	0.816496581	0.816496581	3	140	0	P_207787	P_207787	P_207787	chr10	26174497	26174557	+	MYO3A	myosin IIIA [Source:HGNC Symbol;Acc:HGNC:7601]	ENSG00000095777	protein_coding	exon	coding exon	1	480	10:26174497:26174557	ENSG00000095777	MYO3A	3.971923832	0.026360221	-0.768203505	1.533934309	-0.825565401	2.302137814	-0.057361896	-2.35949971	9.961963646	0.460188699	0.156169211	0.428378003	0.044187296	0.955391169	0.040077806	-6.685580102	-5.073812855	-6.687184939	-4.829132153	-6.294935443	-4.659706797	2.415836184	0.12736363

TDRD3	0.046881885	-0.011586296	-0.040258389	-0.058468181	-0.087140274	-0.028672094	8	0.038806431	0.816496581	0.816496581	0.816496581	0.816496581	0.816496581	0.816496581	3	141	0	P_219311	P_219311	P_219311	chr13	60509859	60509919	+	TDRD3	tudor domain containing 3 [Source:HGNC Symbol;Acc:HGNC:20612]	ENSG00000083544	protein_coding	3'UTR;exon	multiple Ref	1	481	13:60509859:60509919	ENSG00000083544	TDRD3	8.43870847	0.003416035	0.982402654	-0.242789032	-0.84360832	-1.225191686	-1.826010974	-0.600819288	9.961963646	0.349158205	0.813094588	0.41868424	0.248681432	0.097925843	0.561380489	-6.495875159	-6.151107899	-6.67182076	-6.38531592	-4.774965535	-6.877685435	1.155355175	0.374380712

GALNT17	0.11357523	-0.098694009	-0.014685551	-0.212269239	-0.128260781	0.084008458	8	0.247035039	0.816496581	0.816496581	0.816496581	0.816496581	0.816496581	0.816496581	3	142	0	A_16_P38058647	A_16_P38058647	A_16_P38058647	chr7	71434710	71434770	+	WBSCR17	Williams-Beuren syndrome chromosome region 17 [Source:HGNC Symbol;Acc:HGNC:16347]	ENSG00000185274	protein_coding	intron	intron sense	0	482	7:71434710:71434770	ENSG00000185274	GALNT17	4.831023553	0.051214139	0.614659102	-0.534123249	-0.079476902	-1.148782351	-0.694136003	0.454646348	9.961963646	0.552559227	0.604969798	0.938227172	0.277480954	0.503461938	0.659109831	-6.796039083	-6.033893444	-7.043672156	-6.472139153	-6.046613374	-6.95961953	0.44627317	0.725322364

GPA33	0.172113011	-0.074571333	-0.188164929	-0.246684345	-0.36027794	-0.113593595	8	0.180325805	0.816496581	0.816496581	0.816496581	0.816496581	0.816496581	0.816496581	3	143	0	P_204665	P_204665	P_204665	chr1	167069039	167069099	-	GPA33	glycoprotein A33 (transmembrane) [Source:HGNC Symbol;Acc:HGNC:4445]	ENSG00000143167	protein_coding	3'UTR;exon	multiple Ref	1	483	1:167069039:167069099	ENSG00000143167	GPA33	4.222251361	0.028319926	1.252602502	-0.542714569	-1.369425	-1.795317071	-2.622027502	-0.826710431	9.961963646	0.238956378	0.599257141	0.200944031	0.10294836	0.025583829	0.427758363	-6.20375034	-6.02916002	-6.109406017	-5.623914793	-3.54466268	-6.710344397	2.420086451	0.12693938

TSPOAP1	0.462938403	0.135429482	0.120657197	-0.327508921	-0.342281207	-0.014772286	8	0.176171862	0.816496581	0.816496581	0.816496581	0.816496581	0.816496581	0.816496581	3	144	0	A_24_P113131	A_24_P113131	A_24_P113131	chr17	58301238	58301298	-	BZRAP1	benzodiazepine receptor (peripheral) associated protein 1 [Source:HGNC Symbol;Acc:HGNC:16831]	ENSG00000005379	protein_coding	3'UTR;exon	multiple Ref	1	484	17:58301238:58301298	ENSG00000005379	TSPOAP1	5.512382945	0.027130706	3.44221737	1.006997289	0.897156717	-2.435220081	-2.545060654	-0.109840572	9.961963646	0.006342912	0.337768834	0.390801368	0.035221122	0.029189021	0.91471702	-2.756333422	-5.674067579	-6.624542142	-4.60935563	-3.668557747	-7.064791416	4.347914944	0.033391802

RPL35A	0.393546845	0.952726372	-0.376448189	0.559179528	-0.769995033	-1.329174561	8	1.183930289	0.816496581	0.816496581	0.816496581	0.816496581	0.816496581	0.816496581	3	147	0	P_137024	P_137024	P_137024	chr3	197950239	197950299	+	RPL35A	ribosomal protein L35a [Source:HGNC Symbol;Acc:HGNC:10345]	ENSG00000182899	protein_coding	5'UTR	5'UTR	1	487	3:197950239:197950299	ENSG00000182899	RPL35A	9.543818723	1.12784093	0.45385606	1.098727237	-0.434137115	0.644871178	-0.887993175	-1.532864352	9.961963646	0.659659427	0.29773124	0.673440829	0.533578958	0.395478577	0.156429837	-6.88711143	-5.582876748	-6.945161153	-6.926840354	-5.893571509	-5.919793815	0.859287623	0.493467808

EZH2	-0.246640808	-0.101659141	-0.141461382	0.144981667	0.105179426	-0.039802241	8	0.060329074	0.816496581	0.816496581	0.816496581	0.816496581	0.816496581	0.816496581	3	148	0	P_146212	P_146212	P_146212	chr7	148832633	148832693	-	EZH2	enhancer of zeste 2 polycomb repressive complex 2 subunit [Source:HGNC Symbol;Acc:HGNC:3527]	ENSG00000106462	protein_coding	3'UTR;exon	multiple Ref	1	488	7:148832633:148832693	ENSG00000106462	EZH2	8.158749413	0.005129479	-4.217683023	-1.738422918	-2.419061446	2.479260105	1.798621577	-0.680638527	9.961963646	0.001793185	0.112887372	0.036206497	0.032667525	0.102396481	0.511609064	-1.462591777	-4.799032328	-4.538130925	-4.535931726	-4.81424529	-6.824041895	6.084643608	0.012698764

FLYWCH2	0.185609601	0.101784787	0.156031858	-0.083824814	-0.029577743	0.054247071	8	0.093795088	0.816496581	0.816496581	0.816496581	0.816496581	0.816496581	0.816496581	3	149	0	A_24_P417596	A_24_P417596	A_24_P417596	chr16	2899285	2899345	+	FLYWCH2	FLYWCH family member 2 [Source:HGNC Symbol;Acc:HGNC:25178]	ENSG00000162076	protein_coding	3'UTR;exon	multiple Ref	1	489	16:2899285:2899345	ENSG00000162076	FLYWCH2	9.69931693	0.009271571	2.360855478	1.294648398	1.984642306	-1.06620708	-0.376213173	0.689993908	9.961963646	0.039985337	0.224644019	0.075398222	0.311488041	0.714646634	0.505953672	-4.587847516	-5.368197988	-5.237071716	-6.561119878	-6.221951357	-6.817354391	2.157163938	0.156621419

MYO3B	-0.005314531	0.021130071	0.008402832	0.026444602	0.013717363	-0.012727239	8	0.092854891	0.816496581	0.816496581	0.816496581	0.816496581	0.816496581	0.816496581	4	20	0	P_129196	P_129196	P_129196	chr2	170400254	170400314	+	MYO3B	myosin IIIB [Source:HGNC Symbol;Acc:HGNC:15576]	ENSG00000071909	protein_coding	exon	coding exon	1	530	2:170400254:170400314	ENSG00000071909	MYO3B	3.919903616	0.009130645	-0.068117678	0.270829439	0.107701214	0.338947117	0.175818892	-0.163128225	9.961963646	0.94703978	0.792050859	0.916370952	0.741685721	0.863958751	0.873679232	-6.99582889	-6.14356771	-7.040789681	-7.087147902	-6.280269731	-7.056860462	0.043666067	0.987135806

GRHL1	0.210912635	0.053778015	0.198191511	-0.157134621	-0.012721125	0.144413496	8	0.083707328	0.816496581	0.816496581	0.816496581	0.816496581	0.816496581	0.816496581	4	21	0	P_233595	P_233595	P_233595	chr2	9996338	9996398	+	GRHL1	grainyhead-like 1 (Drosophila) [Source:HGNC Symbol;Acc:HGNC:17923]	ENSG00000134317	protein_coding	"3'UTR;5'UTR,exon;exon"	multiple Ref	1	531	2:9996338:9996398	ENSG00000134317	GRHL1	6.015381334	0.00783362	2.918548888	0.744164827	2.742517594	-2.17438406	-0.176031294	1.998352767	9.961963646	0.015396933	0.473964345	0.020810986	0.054863405	0.863796258	0.073698448	-3.649798564	-5.898302163	-3.994808166	-5.036567895	-6.280230108	-5.239292655	4.224243082	0.03603872

DCAF8	-0.079514414	0.028066776	-0.047314219	0.10758119	0.032200194	-0.075380996	8	0.074664643	0.816496581	0.816496581	0.816496581	0.816496581	0.816496581	0.816496581	4	22	0	A_24_P76725	A_24_P76725	A_24_P76725	chr1	160239629	160239689	-	DCAF8	DDB1 and CUL4 associated factor 8 [Source:HGNC Symbol;Acc:HGNC:24891]	ENSG00000132716	protein_coding	exon	coding exon	1	532	1:160239629:160239689	ENSG00000132716	DCAF8	7.380973709	0.006683559	-1.19120795	0.420469263	-0.708815818	1.611677213	0.482392131	-1.129285082	9.961963646	0.261179676	0.683068546	0.494691673	0.138225239	0.639951213	0.285236621	-6.27486067	-6.089737964	-6.779488375	-5.888294953	-6.174384881	-6.416095842	1.033946841	0.418992853

GOLGA1	0.032733404	0.057300845	0.112324229	0.024567441	0.079590825	0.055023384	8	0.078446301	0.816496581	0.816496581	0.816496581	0.816496581	0.816496581	0.816496581	4	23	0	P_268929	P_268929	P_268929	chr9	124889456	124889516	-	GOLGA1	golgin A1 [Source:HGNC Symbol;Acc:HGNC:4424]	ENSG00000136935	protein_coding	3'UTR;exon	multiple Ref	1	533	9:124889456:124889516	ENSG00000136935	GOLGA1	7.040506043	0.007148539	0.474163546	0.830038087	1.62708574	0.355874541	1.152922193	0.797047652	9.961963646	0.645605047	0.42596101	0.134894176	0.729355255	0.275855737	0.444004856	-6.877046894	-5.831395804	-5.76623151	-7.080793413	-5.63447529	-6.73501222	0.942060589	0.456567389

ZNF280D	0.328489105	-0.043094271	0.259256268	-0.371583375	-0.069232837	0.302350538	8	0.062523888	0.816496581	0.816496581	0.816496581	0.816496581	0.816496581	0.816496581	4	25	0	P_223795	P_223795	P_223795	chr15	56704120	56704180	-	ZNF280D	zinc finger protein 280D [Source:HGNC Symbol;Acc:HGNC:25953]	ENSG00000137871	protein_coding	exon	coding exon	1	535	15:56704120:56704180	ENSG00000137871	ZNF280D	7.449926893	0.005346014	5.5023923	-0.721855247	4.342700172	-6.224247547	-1.159692128	5.064555419	9.961963646	0.000264477	0.486981145	0.00147326	9.99E-05	0.273214182	0.000494512	0.518796552	-5.914624615	-1.300929595	1.421245683	-5.627165985	-0.187973218	19.23205096	0.000181671

GC	0.912940495	0.947182755	2.177176495	0.03424226	1.264235999	1.22999374	8	0.173973236	0.816496581	0.816496581	0.816496581	0.816496581	0.816496581	0.816496581	4	26	0	P_249310	P_249310	P_249310	chr4	71765431	71765491	-	GC	group-specific component (vitamin D binding protein) [Source:HGNC Symbol;Acc:HGNC:4187]	ENSG00000145321	protein_coding	exon	coding exon	1	536	4:71765431:71765491	ENSG00000145321	GC	6.79646671	0.026512484	6.866934312	7.124496934	16.37623487	0.257562622	9.509300562	9.25173794	9.961963646	4.45E-05	3.27E-05	1.57E-08	0.801986659	2.59E-06	3.32E-06	2.365073397	2.665659128	10.20327634	-7.11345574	4.919502124	4.997784486	90.36368378	1.59E-07

WWP1	-0.065848486	-0.007062576	-0.182377521	0.05878591	-0.116529035	-0.175314945	8	0.10654496	0.816496581	0.816496581	0.816496581	0.816496581	0.816496581	0.816496581	4	27	0	P_147534	P_147534	P_147534	chr8	86435466	86435526	+	WWP1	WW domain containing E3 ubiquitin protein ligase 1 [Source:HGNC Symbol;Acc:HGNC:17004]	ENSG00000123124	protein_coding	exon	coding exon	1	537	8:86435466:86435526	ENSG00000123124	WWP1	6.957750663	0.011322821	-0.757903819	-0.081288935	-2.099131337	0.676614884	-1.341227518	-2.017842403	9.961963646	0.466059142	0.936822154	0.062272246	0.514053081	0.209622745	0.071344318	-6.693679991	-6.178605134	-5.057655369	-6.904869397	-5.419657838	-5.20893974	1.885960897	0.196109349

USP37	-0.169698602	-0.062191302	-0.10855367	0.1075073	0.061144932	-0.046362368	8	0.0858129	0.816496581	0.816496581	0.816496581	0.816496581	0.816496581	0.816496581	4	28	0	P_239328	P_239328	P_239328	chr2	218534608	218534668	-	USP37	ubiquitin specific peptidase 37 [Source:HGNC Symbol;Acc:HGNC:20063]	ENSG00000135913	protein_coding	exon	coding exon	1	538	2:218534608:218534668	ENSG00000135913	USP37	7.882606837	0.00812026	-2.306422796	-0.845259979	-1.475384332	1.461162818	0.831038464	-0.630124354	9.961963646	0.043866625	0.417804372	0.171001071	0.174782847	0.425421679	0.542795675	-4.67708071	-5.818873827	-5.972809638	-6.09240887	-5.941928915	-6.858706071	1.905581314	0.192891075

MIGA2	-0.156198192	0.008358708	-0.111294473	0.1645569	0.044903719	-0.119653181	8	0.083284623	0.816496581	0.816496581	0.816496581	0.816496581	0.816496581	0.816496581	4	29	0	A_23_P71864	A_23_P71864	A_23_P71864	chr9	129071267	129071327	+	FAM73B	"family with sequence similarity 73, member B [Source:HGNC Symbol;Acc:HGNC:23621]"	ENSG00000148343	protein_coding	3'UTR;exon	multiple Ref	1	539	9:129071267:129071327	ENSG00000148343	MIGA2	7.358721457	0.007776934	-2.169289064	0.116086191	-1.54566375	2.285375255	0.623625314	-1.661749941	9.961963646	0.0553371	0.909891026	0.15333695	0.045463783	0.546886531	0.127661092	-4.899012973	-6.174988258	-5.878635874	-4.856563735	-6.093896471	-5.741088723	2.580271488	0.112097551

CEP95	-0.034140629	-0.104716138	-0.118951488	-0.070575509	-0.084810858	-0.014235349	8	0.076448674	0.816496581	0.816496581	0.816496581	0.816496581	0.816496581	0.816496581	4	30	0	P_229692	P_229692	P_229692	chr17	64514298	64514358	+	CEP95	centrosomal protein 95kDa [Source:HGNC Symbol;Acc:HGNC:25141]	ENSG00000258890	protein_coding	3'UTR;5'UTR;exon	multiple Ref	1	540	17:64514298:64514358	ENSG00000258890	CEP95	7.696614536	0.006900056	-0.503374086	-1.543948995	-1.753836923	-1.04057491	-1.250462837	-0.209887928	9.961963646	0.62564423	0.153748171	0.110111571	0.322668547	0.239704203	0.837985466	-6.861833188	-5.060813746	-5.58479791	-6.587673468	-5.526114778	-7.04736568	1.400602386	0.299425368

HIPK2	-0.238841015	-0.078190254	-0.227613185	0.160650761	0.01122783	-0.149422931	8	0.089025806	0.816496581	0.816496581	0.816496581	0.816496581	0.816496581	0.816496581	4	31	0	A_24_P500621	A_24_P500621	A_24_P500621	chr7	139566001	139566061	-	HIPK2	homeodomain interacting protein kinase 2 [Source:HGNC Symbol;Acc:HGNC:14402]	ENSG00000064393	protein_coding	3'UTR	3'UTR	1	541	7:139566001:139566061	ENSG00000064393	HIPK2	7.433333534	0.008571368	-3.159580757	-1.034363468	-3.011050007	2.125217288	0.14853075	-1.976686538	9.961963646	0.010212589	0.325422986	0.013147746	0.05960169	0.884887481	0.076401409	-3.238189978	-5.647522296	-3.536934164	-5.115315088	-6.284964507	-5.272888884	4.760863344	0.026086719

IL1RAP	0.11990018	0.057573983	0.043636749	-0.062326197	-0.076263431	-0.013937234	8	0.082527029	0.816496581	0.816496581	0.816496581	0.816496581	0.816496581	0.816496581	4	32	0	P_247721	P_247721	P_247721	chr3	190623354	190623414	+	IL1RAP	interleukin 1 receptor accessory protein [Source:HGNC Symbol;Acc:HGNC:5995]	ENSG00000196083	protein_coding	exon	coding exon	1	542	3:190623354:190623414	ENSG00000196083	IL1RAP	6.521601724	0.007676056	1.676086398	0.804827564	0.609998762	-0.871258834	-1.066087636	-0.194828802	9.961963646	0.124772744	0.439704706	0.555520851	0.40412058	0.31153945	0.849442979	-5.649394302	-5.851700564	-6.847644423	-6.749637804	-5.72530423	-7.050681479	0.960450763	0.448769362

MROH7	0.002035595	0.130707247	0.367359347	0.128671652	0.365323752	0.2366521	8	0.255290408	0.816496581	0.816496581	0.816496581	0.816496581	0.816496581	0.816496581	4	35	0	P_201825	P_201825	P_201825	chr1	54653701	54653761	+	MROH7	maestro heat-like repeat family member 7 [Source:HGNC Symbol;Acc:HGNC:24802]	ENSG00000184313	protein_coding	exon	coding exon	1	545	1:54653701:54653761	ENSG00000184313	MROH7	4.64927869	0.054544311	0.010674863	0.685441768	1.926468858	0.674766905	1.915793995	1.24102709	9.961963646	0.991693605	0.508700713	0.083028717	0.515177919	0.08450518	0.243024813	-6.998298135	-5.940306016	-5.326598946	-6.906174891	-4.644279101	-6.289435956	1.641158647	0.241970474

SPAG1	0.080534249	0.126279146	0.231641887	0.045744897	0.151107639	0.105362741	8	0.032324673	0.816496581	0.816496581	0.816496581	0.816496581	0.816496581	0.816496581	4	36	0	P_265647	P_265647	P_265647	chr8	100240706	100240766	+	SPAG1	sperm associated antigen 1 [Source:HGNC Symbol;Acc:HGNC:11212]	ENSG00000104450	protein_coding	exon	coding exon	1	546	8:100240706:100240766	ENSG00000104450	SPAG1	6.79463955	0.003045783	1.787214782	2.802384818	5.140593121	1.015170037	3.353378339	2.338208302	9.961963646	0.104313077	0.018782508	0.000442714	0.334045829	0.007361884	0.041557678	-5.488387673	-3.173937049	-0.053540113	-6.613478647	-2.357525422	-4.69473569	9.202688255	0.003205914

HSPA8P8	-0.005686031	0.032274524	0.008397087	0.037960556	0.014083118	-0.023877438	8	0.213667088	0.816496581	0.816496581	0.816496581	0.816496581	0.816496581	0.816496581	4	39	0	A_24_P709536	A_24_P709536	A_24_P709536	chr7	10450724	10450784	-	HSPA8P8	heat shock 70kDa protein 8 pseudogene 8 [Source:HGNC Symbol;Acc:HGNC:44923]	ENSG00000229091	processed_pseudogene	promoter	promoter antisense	0	549	7:10450724:10450784	ENSG00000229091	HSPA8P8	3.410995567	0.038869034	-0.035322627	0.200495029	0.052164181	0.235817655	0.087486808	-0.148330847	9.961963646	0.972520238	0.845127438	0.959429061	0.818351525	0.932018184	0.885041162	-6.997679507	-6.160940467	-7.045634785	-7.119286911	-6.292615843	-7.059372081	0.021572166	0.995417689

CASZ1	0.123150669	-0.071742849	0.087775534	-0.194893517	-0.035375135	0.159518382	8	0.109194339	0.816496581	0.816496581	0.816496581	0.816496581	0.816496581	0.816496581	4	40	0	A_23_P201295	A_23_P201295	A_23_P201295	chr1	10648060	10648344	-	CASZ1;RP4-734G22.3	castor zinc finger 1 [Source:HGNC Symbol;Acc:HGNC:26002];	ENSG00000130940	protein_coding;antisense	"exon;exon,intron;promoter"	multiple non-Ref	0	550	1:10648060:10648344	ENSG00000130940	CASZ1	6.590388375	0.011781827	1.38955608	-0.809501992	0.990404906	-2.199058072	-0.399151175	1.799906897	9.961963646	0.194934913	0.437134366	0.345421877	0.05262405	0.698204356	0.102182557	-6.036020992	-5.847976879	-6.536383767	-4.9968101	-6.212636986	-5.540699989	1.966984059	0.18320866

GPATCH1	-0.067696822	-0.097936787	0.003481228	-0.030239965	0.07117805	0.101418015	8	0.063138971	0.816496581	0.816496581	0.816496581	0.816496581	0.816496581	0.816496581	4	41	0	P_232708	P_232708	P_232708	chr19	33097791	33097851	+	GPATCH1	G patch domain containing 1 [Source:HGNC Symbol;Acc:HGNC:24658]	ENSG00000076650	protein_coding	3'UTR;exon	multiple Ref	1	551	19:33097791:33097851	ENSG00000076650	GPATCH1	8.266896026	0.005408085	-1.127436706	-1.631059269	0.057977082	-0.503622562	1.185413788	1.689036351	9.961963646	0.285980588	0.13404686	0.954913341	0.625475758	0.263359944	0.122214442	-6.34587257	-4.945680908	-7.045285251	-7.012655015	-5.599102714	-5.702197635	1.407752423	0.297508331

CTSC	-0.184989225	0.197445958	-0.15354071	0.382435183	0.031448514	-0.350986669	8	0.085375463	0.816496581	0.816496581	0.816496581	0.816496581	0.816496581	0.816496581	4	42	0	P_109777	P_109777	P_109777	chr11	88320968	88321028	-	CTSC	cathepsin C [Source:HGNC Symbol;Acc:HGNC:2528]	ENSG00000109861	protein_coding	3'UTR	3'UTR	1	552	11:88320968:88321028	ENSG00000109861	CTSC	6.37940193	0.008060124	-2.523603948	2.693537429	-2.094586553	5.217141377	0.429017395	-4.788123982	9.961963646	0.030281027	0.022633143	0.062749054	0.000396378	0.677040067	0.000744586	-4.317890466	-3.347795629	-5.064853185	-0.02084555	-6.199711458	-0.613648578	11.38996082	0.001468019

MAPK10	0.062751903	-0.043173429	-0.091573845	-0.105925332	-0.154325749	-0.048400416	8	0.100368275	0.816496581	0.816496581	0.816496581	0.816496581	0.816496581	0.816496581	4	43	0	P_249834	P_249834	P_249834	chr4	86370713	86370773	-	MAPK10	mitogen-activated protein kinase 10 [Source:HGNC Symbol;Acc:HGNC:6872]	ENSG00000109339	protein_coding	5'UTR	5'UTR	1	553	4:86370713:86370773	ENSG00000109339	MAPK10	4.544301856	0.010296487	0.757404692	-0.521095872	-1.105280582	-1.278500564	-1.862685274	-0.58418471	9.961963646	0.466344843	0.613685674	0.295016587	0.23005503	0.092221384	0.572086525	-6.694070044	-6.040935074	-6.418090898	-6.322310576	-4.721930543	-6.888084963	1.25204664	0.342578135

UGP2	0.177420644	0.074952457	0.132818162	-0.102468187	-0.044602482	0.057865705	8	0.035895715	0.816496581	0.816496581	0.816496581	0.816496581	0.816496581	0.816496581	4	44	0	P_235273	P_235273	P_235273	chr2	63856373	63856433	+	UGP2	UDP-glucose pyrophosphorylase 2 [Source:HGNC Symbol;Acc:HGNC:12527]	ENSG00000169764	protein_coding	3'UTR;exon	multiple Ref	1	554	2:63856373:63856433	ENSG00000169764	UGP2	9.005794663	0.003241422	3.816647206	1.612366405	2.857165066	-2.204280802	-0.95948214	1.244798662	9.961963646	0.003415573	0.138074697	0.017101185	0.052161468	0.360023837	0.24169307	-2.124743333	-4.970690814	-3.799758111	-4.988374889	-5.829056384	-6.285006587	5.443149512	0.017774469

HNF4G	0.075556995	0.100263615	-0.042113505	0.02470662	-0.1176705	-0.14237712	8	0.10152778	0.816496581	0.816496581	0.816496581	0.816496581	0.816496581	0.816496581	4	46	0	P_147447	P_147447	P_147447	chr8	75564036	75564096	+	HNF4G	"hepatocyte nuclear factor 4, gamma [Source:HGNC Symbol;Acc:HGNC:5026]"	ENSG00000164749	protein_coding	exon	coding exon	1	556	8:75564036:75564096	ENSG00000164749	HNF4G	3.832830653	0.010484482	0.903746903	1.19926595	-0.503725038	0.295519048	-1.407471941	-1.702990989	9.961963646	0.387461739	0.25817156	0.625406284	0.773665208	0.189715744	0.119510725	-6.570133094	-5.47594672	-6.910299385	-7.102063764	-5.338761454	-5.68217327	1.24620388	0.344412342

DCTN1	0.1761627	0.070059551	0.242768706	-0.106103148	0.066606006	0.172709155	8	0.040976851	0.816496581	0.816496581	0.816496581	0.816496581	0.816496581	0.816496581	4	49	0	P_235561	P_235561	P_235561	chr2	74378012	74378072	-	DCTN1	dynactin 1 [Source:HGNC Symbol;Acc:HGNC:2711]	ENSG00000204843	protein_coding	exon	coding exon	1	559	2:74378012:74378072	ENSG00000204843	DCTN1	7.440914092	0.003555095	3.618545174	1.439088139	4.986694293	-2.179457035	1.368149119	3.547606154	9.961963646	0.004730053	0.180795647	0.000554315	0.054395632	0.201330092	0.005320626	-2.457614979	-5.19434109	-0.287239982	-5.028406401	-5.387096792	-2.642953326	9.873223037	0.002490245

URB1	-0.160743501	-0.021965424	-0.222123808	0.138778077	-0.061380307	-0.200158384	8	0.04563103	0.816496581	0.816496581	0.816496581	0.816496581	0.816496581	0.816496581	4	50	0	P_311761	P_311761	P_311761	chr21	32318325	32318385	-	URB1;MRAP	URB1 ribosome biogenesis 1 homolog (S. cerevisiae) [Source:HGNC Symbol;Acc:HGNC:17344];melanocortin 2 receptor accessory protein [Source:HGNC Symbol;Acc:HGNC:1304]	ENSG00000142207	protein_coding	3'ext;intron	multiple non-Ref	0	560	21:32318325:32318385	ENSG00000142207	URB1	5.716294048	0.003878797	-3.161043984	-0.431953218	-4.368096511	2.729090767	-1.207052527	-3.936143294	9.961963646	0.010187274	0.67497509	0.001415976	0.021295336	0.255291203	0.002812855	-3.235690288	-6.084673684	-1.25990985	-4.114477707	-5.575140235	-1.989321181	8.942864338	0.003548053

KRT13	-0.155901178	-0.014598643	0.06549828	0.141302535	0.221399457	0.080096922	8	0.221934745	0.816496581	0.816496581	0.816496581	0.816496581	0.816496581	0.816496581	4	51	0	P_121439	P_121439	P_121439	chr17	41501017	41501077	-	KRT13	keratin 13 [Source:HGNC Symbol;Acc:HGNC:6415]	ENSG00000171401	protein_coding	3'UTR;exon	multiple Ref	1	561	17:41501017:41501077	ENSG00000171401	KRT13	5.029888303	0.04176116	-0.934348194	-0.087492703	0.392544817	0.846855491	1.326893011	0.48003752	9.961963646	0.372218538	0.932013616	0.702923579	0.416955612	0.214153639	0.641566612	-6.541858173	-6.178053554	-6.96361866	-6.770975442	-5.436815054	-6.94693458	0.623833043	0.615708163

EIF4E3	0.587567932	0.276121635	0.533482811	-0.311446297	-0.054085121	0.257361176	8	0.103176125	0.816496581	0.816496581	0.816496581	0.816496581	0.816496581	0.816496581	4	52	0	A_24_P943113	A_24_P943113	A_24_P943113	chr3	71683017	71683077	-	EIF4E3	eukaryotic translation initiation factor 4E family member 3 [Source:HGNC Symbol;Acc:HGNC:31837]	ENSG00000163412	protein_coding	3'UTR	3'UTR	1	562	3:71683017:71683077	ENSG00000163412	EIF4E3	5.761637975	0.010755451	6.938880508	3.260857046	6.300162549	-3.678023462	-0.63871796	3.039305502	9.961963646	4.08E-05	0.008603818	9.06E-05	0.004287529	0.537413558	0.012529597	2.455269623	-2.440811563	1.597384336	-2.492853599	-6.08416557	-3.511089119	20.27441578	0.000145508

NARF	0.090690551	0.031626394	0.081157552	-0.059064156	-0.009532999	0.049531157	8	0.076362007	0.816496581	0.816496581	0.816496581	0.816496581	0.816496581	0.816496581	4	53	0	P_123055	P_123055	P_123055	chr17	82488191	82488251	+	NARF	nuclear prelamin A recognition factor [Source:HGNC Symbol;Acc:HGNC:29916]	ENSG00000141562	protein_coding	3'UTR;exon	multiple Ref	1	563	17:82488191:82488251	ENSG00000141562	NARF	10.09468607	0.00688942	1.338185529	0.46666365	1.197521249	-0.871521879	-0.14066428	0.730857599	9.961963646	0.210577511	0.650778931	0.258820507	0.403983732	0.890938761	0.481701875	-6.10035393	-6.068571211	-6.315831971	-6.749404966	-6.286171899	-6.787176772	0.792677045	0.525390015

C16orf70	-0.318579927	0.157470312	-0.121630587	0.476050239	0.196949341	-0.279100899	8	0.087291666	0.816496581	0.816496581	0.816496581	0.816496581	0.816496581	0.816496581	4	54	0	P_310233	P_310233	P_310233	chr16	67143753	67143813	+	C16orf70;B3GNT9	"chromosome 16 open reading frame 70 [Source:HGNC Symbol;Acc:HGNC:29564];UDP-GlcNAc:betaGal beta-1,3-N-acetylglucosaminyltransferase 9 [Source:HGNC Symbol;Acc:HGNC:28714]"	ENSG00000125149	protein_coding	3'ext;intron;promoter	multiple non-Ref	0	564	16:67143753:67143813	ENSG00000125149	C16orf70	7.071823119	0.008325827	-4.276124566	2.113638088	-1.632581013	6.389762654	2.643543554	-3.746219101	9.961963646	0.001635359	0.060773321	0.133723624	8.07E-05	0.02465776	0.003832736	-1.367618061	-4.25329236	-5.758523754	1.644530605	-3.509932126	-2.307235826	14.54492915	0.000569031

DOCK3	0.017531297	-0.095521868	-0.038276758	-0.113053165	-0.055808055	0.05724511	8	0.108307797	0.816496581	0.816496581	0.816496581	0.816496581	0.816496581	0.816496581	4	55	0	P_244368	P_244368	P_244368	chr3	50778698	50778758	+	DOCK3	dedicator of cytokinesis 3 [Source:HGNC Symbol;Acc:HGNC:2989]	ENSG00000088538	protein_coding	exon	coding exon	1	565	3:50778698:50778758	ENSG00000088538	DOCK3	4.731130644	0.011626978	0.19912521	-1.084963184	-0.434757756	-1.284088394	-0.633882965	0.650205429	9.961963646	0.84617024	0.303495587	0.673005095	0.228170584	0.540437865	0.530267702	-6.976766455	-5.596957624	-6.944872168	-6.315594428	-6.087306214	-6.845219411	0.646544664	0.602757296

GRM8	-0.124182096	-0.00302736	0.141625519	0.121154736	0.265807616	0.144652879	8	0.176765583	0.816496581	0.816496581	0.816496581	0.816496581	0.816496581	0.816496581	4	56	0	P_262774	P_262774	P_262774	chr7	127106643	127106703	-	GRM8	"glutamate receptor, metabotropic 8 [Source:HGNC Symbol;Acc:HGNC:4600]"	ENSG00000179603	protein_coding	exon	coding exon	1	566	7:127106643:127106703	ENSG00000179603	GRM8	4.021213619	0.027298983	-0.920516082	-0.022440701	1.049817747	0.898075381	1.97033383	1.072258448	9.961963646	0.379054774	0.982539541	0.318602442	0.390334618	0.077211461	0.308892023	-6.554736926	-6.181821955	-6.476498339	-6.725592177	-4.563549784	-6.477188367	1.298077286	0.328503112

EOGT	-0.091095035	0.164882384	0.0128465	0.255977419	0.103941535	-0.152035884	8	0.069714801	0.816496581	0.816496581	0.816496581	0.816496581	0.816496581	0.816496581	4	57	0	P_244875	P_244875	P_244875	chr3	69007733	69007793	-	EOGT	EGF domain-specific O-linked N-acetylglucosamine (GlcNAc) transferase [Source:HGNC Symbol;Acc:HGNC:28526]	ENSG00000163378	protein_coding	exon	coding exon	1	567	3:69007733:69007793	ENSG00000163378	EOGT	6.650370788	0.006109652	-1.427355307	2.583518902	0.201289999	4.010874209	1.628645306	-2.382228903	9.961963646	0.184063132	0.027328582	0.844522395	0.002493512	0.134561046	0.038554858	-5.987646096	-3.522822004	-7.025038701	-1.934469297	-5.051134977	-4.622216423	5.527840458	0.016980759

RFC4	-0.466570341	-0.067578832	-0.348202488	0.398991509	0.118367853	-0.280623656	8	0.079615569	0.816496581	0.816496581	0.816496581	0.816496581	0.816496581	0.816496581	4	58	0	P_247670	P_247670	P_247670	chr3	186790162	186790222	-	RFC4	"replication factor C (activator 1) 4, 37kDa [Source:HGNC Symbol;Acc:HGNC:9972]"	ENSG00000163918	protein_coding	exon	coding exon	1	568	3:186790162:186790222	ENSG00000163918	RFC4	10.93407907	0.007296957	-6.689472587	-0.968914445	-4.992368334	5.720558142	1.697104253	-4.023453888	9.961963646	5.54E-05	0.35552295	0.000549707	0.000195594	0.120644623	0.002443612	2.139642308	-5.710047921	-0.27856364	0.718118608	-4.957220592	-1.84436159	20.40078496	0.000141738

FBN1	-0.222645889	-0.172634728	-0.240613024	0.050011161	-0.017967134	-0.067978296	8	0.087445949	0.816496581	0.816496581	0.816496581	0.816496581	0.816496581	0.816496581	4	59	0	P_223299	P_223299	P_223299	chr15	48432866	48432926	-	FBN1	fibrillin 1 [Source:HGNC Symbol;Acc:HGNC:3603]	ENSG00000166147	protein_coding	3'UTR;exon	multiple Ref	1	569	15:48432866:48432926	ENSG00000166147	FBN1	3.966785128	0.008347477	-2.984576443	-2.314174961	-3.225426545	0.670401482	-0.240850103	-0.911251585	9.961963646	0.013755058	0.043292231	0.009134761	0.517840952	0.81455572	0.38368324	-3.537139578	-3.946007931	-3.170177755	-6.909245882	-6.265923628	-6.635783386	4.334050844	0.033676715

NIBAN1	-0.062401403	0.423309881	0.505809724	0.485711284	0.568211126	0.082499843	8	0.082583713	0.816496581	0.816496581	0.816496581	0.816496581	0.816496581	0.816496581	4	60	0	P_205329	P_205329	P_205329	chr1	184890107	184890167	-	FAM129A	"family with sequence similarity 129, member A [Source:HGNC Symbol;Acc:HGNC:16784]"	ENSG00000135842	protein_coding	exon	coding exon	1	570	1:184890107:184890167	ENSG00000135842	NIBAN1	5.269850544	0.007683572	-0.871883395	5.914560247	7.067262589	6.786443642	7.939145984	1.152702342	9.961963646	0.403795709	0.000150398	3.50E-05	4.91E-05	1.29E-05	0.275941857	-6.59872433	1.32446633	2.585855883	2.164459836	3.572076545	-6.390300765	32.6838305	1.81E-05

MAP3K2	0.060226227	-0.045319422	0.02990976	-0.105545649	-0.030316467	0.075229182	8	0.053736771	0.816496581	0.816496581	0.816496581	0.816496581	0.816496581	0.816496581	4	61	0	A_23_P313640	A_23_P313640	A_23_P313640	chr2	127306514	127306574	-	MAP3K2	mitogen-activated protein kinase kinase kinase 2 [Source:HGNC Symbol;Acc:HGNC:6854]	ENSG00000169967	protein_coding	3'UTR	3'UTR	1	571	2:127306514:127306574	ENSG00000169967	MAP3K2	7.421029305	0.004525617	1.096459	-0.825070591	0.544527311	-1.921529591	-0.551931689	1.369597903	9.961963646	0.298675316	0.428645959	0.598055406	0.083708856	0.593159952	0.200891762	-6.379280704	-5.835439644	-6.887574029	-5.433852543	-6.13716607	-6.133084584	1.342029464	0.315665375

SYNE2	0.268834971	-0.282848637	0.047201128	-0.551683608	-0.221633843	0.330049766	8	0.065997571	0.816496581	0.816496581	0.816496581	0.816496581	0.816496581	0.816496581	4	62	0	P_221203	P_221203	P_221203	chr14	64170232	64170292	+	SYNE2	"spectrin repeat containing, nuclear envelope 2 [Source:HGNC Symbol;Acc:HGNC:17084]"	ENSG00000054654	protein_coding	exon	coding exon	1	572	14:64170232:64170292	ENSG00000054654	SYNE2	7.631796363	0.005704532	4.359346425	-4.58658779	0.765399195	-8.945934215	-3.59394723	5.351986985	9.961963646	0.001435442	0.001010407	0.461782299	4.49E-06	0.004926713	0.000326901	-1.233100626	-0.424267138	-6.736310609	4.65392425	-1.971156983	0.243069895	27.03608085	4.21E-05

NUP205	-0.313012905	-0.09111203	-0.305091571	0.221900875	0.007921334	-0.213979541	8	0.043963625	0.816496581	0.816496581	0.816496581	0.816496581	0.816496581	0.816496581	4	63	0	P_263021	P_263021	P_263021	chr7	135584947	135585007	+	NUP205	nucleoporin 205kDa [Source:HGNC Symbol;Acc:HGNC:18658]	ENSG00000155561	protein_coding	exon	coding exon	1	573	7:135584947:135585007	ENSG00000155561	NUP205	8.674650936	0.003758828	-6.252902328	-1.820099482	-6.094661802	4.432802846	0.158240526	-4.27456232	9.961963646	9.63E-05	0.098875256	0.000118385	0.00128041	0.877428978	0.001639382	1.566757953	-4.684296571	1.318858582	-1.243592818	-6.283384048	-1.432154891	19.58385271	0.000168372

ACBD3	0.144299638	0.066913216	0.097882434	-0.077386422	-0.046417204	0.030969218	8	0.034885654	0.816496581	0.816496581	0.816496581	0.816496581	0.816496581	0.816496581	4	64	0	P_206485	P_206485	P_206485	chr1	226152377	226152437	-	ACBD3	acyl-CoA binding domain containing 3 [Source:HGNC Symbol;Acc:HGNC:15453]	ENSG00000182827	protein_coding	exon	coding exon	1	574	1:226152377:226152437	ENSG00000182827	ACBD3	9.703271248	0.003184008	3.132014512	1.452347115	2.12453204	-1.679667398	-1.007482473	0.672184925	9.961963646	0.010701722	0.177163147	0.05967042	0.124060524	0.337546957	0.516752007	-3.285285622	-5.17778102	-5.017318853	-5.792239717	-5.783432222	-6.830013258	3.453433251	0.059552185

ADCY3	-0.216940513	0.020186764	-0.168243098	0.237127277	0.048697415	-0.188429862	8	0.051845858	0.816496581	0.816496581	0.816496581	0.816496581	0.816496581	0.816496581	4	66	0	A_23_P67864	A_23_P67864	A_23_P67864	chr2	24819403	24819463	-	ADCY3	adenylate cyclase 3 [Source:HGNC Symbol;Acc:HGNC:234]	ENSG00000138031	protein_coding	3'UTR	3'UTR	1	576	2:24819403:24819463	ENSG00000138031	ADCY3	7.467135808	0.004365289	-4.021423362	0.374201781	-3.11872004	4.395625142	0.902703322	-3.492921821	9.961963646	0.002451595	0.716095916	0.01094612	0.001356533	0.387989238	0.005827829	-1.784469789	-6.108804657	-3.35272541	-1.303605682	-5.880639029	-2.735867761	9.729197341	0.002626312

ITGB7	0.322091109	0.06178357	0.467783209	-0.260307539	0.1456921	0.40599964	8	0.129935291	0.816496581	0.816496581	0.816496581	0.816496581	0.816496581	0.816496581	4	67	0	P_215545	P_215545	P_215545	chr12	53188536	53188596	-	ITGB7;ZNF740	"integrin, beta 7 [Source:HGNC Symbol;Acc:HGNC:6162];zinc finger protein 740 [Source:HGNC Symbol;Acc:HGNC:27465]"	ENSG00000139626	protein_coding	3'ext;3'UTR;exon	multiple non-Ref	0	577	12:53188536:53188596	ENSG00000139626	ITGB7	6.704071553	0.015764798	3.141811767	0.602662853	4.56295362	-2.539148914	1.421141853	3.960290767	9.961963646	0.010525193	0.560200894	0.001047632	0.029485935	0.185813891	0.00270522	-3.268546885	-5.994167594	-0.947938151	-4.435608378	-5.321747765	-1.949150567	9.200925627	0.003208098

FLT1	-0.009416917	-0.023835776	-0.037940286	-0.014418859	-0.028523369	-0.01410451	8	0.102137042	0.816496581	0.816496581	0.816496581	0.816496581	0.816496581	0.816496581	4	68	0	P_113503	P_113503	P_113503	chr13	28384884	28384944	-	FLT1	fms-related tyrosine kinase 1 [Source:HGNC Symbol;Acc:HGNC:3763]	ENSG00000102755	protein_coding	exon	coding exon	1	578	13:28384884:28384944	ENSG00000102755	FLT1	4.06928874	0.010584129	-0.112105474	-0.283757508	-0.451667323	-0.171652034	-0.339561849	-0.167909815	9.961963646	0.912966497	0.782406182	0.661182665	0.867147837	0.74123656	0.870014063	-6.991506613	-6.139818204	-6.936846006	-7.133523883	-6.235721442	-7.055998113	0.078341618	0.970284561

BTBD9	-0.107508129	-0.042092262	-0.12839005	0.065415866	-0.020881921	-0.086297788	8	0.057366079	0.816496581	0.816496581	0.816496581	0.816496581	0.816496581	0.816496581	4	69	0	A_32_P217401	A_32_P217401	A_32_P217401	chr6	38171962	38172022	-	BTBD9	BTB (POZ) domain containing 9 [Source:HGNC Symbol;Acc:HGNC:21228]	ENSG00000183826	protein_coding	3'UTR	3'UTR	1	579	6:38171962:38172022	ENSG00000183826	BTBD9	5.33374779	0.00484943	-1.890782617	-0.740291169	-2.258040188	1.150491448	-0.36725757	-1.517749019	9.961963646	0.08806103	0.476208517	0.047622567	0.276809086	0.72110854	0.160152595	-5.333654064	-5.90116806	-4.802514995	-6.470243377	-6.225442709	-5.940181435	2.163984365	0.155754979

PDCD6IP	0.032673164	-0.022630421	0.019452932	-0.055303584	-0.013220232	0.042083352	8	0.036019966	0.816496581	0.816496581	0.816496581	0.816496581	0.816496581	0.816496581	4	71	0	P_134346	P_134346	P_134346	chr3	33866386	33866446	+	PDCD6IP	programmed cell death 6 interacting protein [Source:HGNC Symbol;Acc:HGNC:8766]	ENSG00000170248	protein_coding	exon	coding exon	1	581	3:33866386:33866446	ENSG00000170248	PDCD6IP	8.985996992	0.003248597	0.702083666	-0.48628437	0.41800622	-1.188368036	-0.284077446	0.90429059	9.961963646	0.498701953	0.637285239	0.684809954	0.262246491	0.782167982	0.387187121	-6.735872585	-6.058943302	-6.952532504	-6.427681801	-6.253940683	-6.64215635	0.535797203	0.668252083

CA1	-0.08665241	-0.076856685	-0.201901139	0.009795726	-0.115248729	-0.125044454	8	0.260760154	0.816496581	0.816496581	0.816496581	0.816496581	0.816496581	0.816496581	4	72	0	P_147526	P_147526	P_147526	chr8	85328493	85328553	-	CA1	carbonic anhydrase I [Source:HGNC Symbol;Acc:HGNC:1368]	ENSG00000133742	protein_coding	3'UTR	3'UTR	1	582	8:85328493:85328553	ENSG00000133742	CA1	4.219514099	0.056811066	-0.445256028	-0.394921527	-1.037451804	0.050334501	-0.592195776	-0.642530277	9.961963646	0.665653896	0.701224248	0.324051272	0.960850758	0.566916593	0.535035889	-6.891246343	-6.100531747	-6.489194451	-7.14822772	-6.113469663	-6.850420081	0.366099458	0.779096255

ATP8B3	0.162689119	0.185511854	0.068790757	0.022822735	-0.093898362	-0.116721097	8	0.090043275	0.816496581	0.816496581	0.816496581	0.816496581	0.816496581	0.816496581	4	73	0	P_231965	P_231965	P_231965	chr19	1801967	1802027	-	ATP8B3	"ATPase, aminophospholipid transporter, class I, type 8B, member 3 [Source:HGNC Symbol;Acc:HGNC:13535]"	ENSG00000130270	protein_coding	3'UTR;exon	multiple Ref	1	583	19:1801967:1802027	ENSG00000130270	ATP8B3	5.411094924	0.008717683	2.134045193	2.433418309	0.902350361	0.299373116	-1.231694833	-1.531067948	9.961963646	0.058722994	0.035329677	0.388167764	0.770808069	0.246345657	0.156868272	-4.955302147	-3.759818695	-6.61982464	-7.100821038	-5.547464782	-5.922223338	2.540129202	0.115615848

HTT	-0.013322185	-0.039436034	-0.065661083	-0.026113849	-0.052338898	-0.026225049	8	0.071992184	0.816496581	0.816496581	0.816496581	0.816496581	0.816496581	0.816496581	4	74	0	P_248106	P_248106	P_248106	chr4	3206536	3206596	+	HTT	huntingtin [Source:HGNC Symbol;Acc:HGNC:4851]	ENSG00000197386	protein_coding	exon	coding exon	1	584	4:3206536:3206596	ENSG00000197386	HTT	7.009149831	0.006368815	-0.204452193	-0.605214808	-1.00768399	-0.400762615	-0.803231797	-0.402469182	9.961963646	0.842116737	0.558570335	0.337454833	0.697055266	0.440584467	0.695839218	-6.975598114	-5.992602407	-6.519259071	-7.062485209	-5.964530666	-6.983604255	0.398547687	0.757073059

CSTB	-0.116909668	-0.007530328	-0.05002999	0.109379339	0.066879677	-0.042499662	8	0.100489889	0.816496581	0.816496581	0.816496581	0.816496581	0.816496581	0.816496581	4	75	0	P_132371	P_132371	P_132371	chr21	43773887	43773947	-	CSTB	cystatin B (stefin B) [Source:HGNC Symbol;Acc:HGNC:2482]	ENSG00000160213	protein_coding	3'UTR	3'UTR	1	585	21:43773887:43773947	ENSG00000160213	CSTB	9.64560962	0.010316103	-1.409737364	-0.090803314	-0.603278995	1.31893405	0.806458369	-0.512475681	9.961963646	0.189064331	0.929448724	0.559806969	0.216704383	0.438806833	0.619487988	-6.010300606	-6.177742639	-6.851937029	-6.273244558	-5.961942427	-6.929773917	0.835357279	0.504702232

ARFGAP1	0.129638163	0.119654461	-0.011389331	-0.009983702	-0.141027494	-0.131043792	8	0.102912284	0.816496581	0.816496581	0.816496581	0.816496581	0.816496581	0.816496581	4	76	0	P_131803	P_131803	P_131803	chr20	63284379	63284439	+	ARFGAP1	ADP-ribosylation factor GTPase activating protein 1 [Source:HGNC Symbol;Acc:HGNC:15852]	ENSG00000101199	protein_coding	exon	non-coding exon	1	586	20:63284379:63284439	ENSG00000101199	ARFGAP1	6.729756296	0.010711785	1.534078483	1.415935934	-0.134776111	-0.118142549	-1.668854593	-1.550712044	9.961963646	0.156134122	0.187291689	0.895473072	0.908302907	0.126222298	0.152131905	-5.846568516	-5.223023908	-7.037209828	-7.14198546	-4.996230325	-5.895561249	1.596696186	0.251567756

SUPT3H	0.067893639	0.158341911	-0.037303108	0.090448271	-0.105196748	-0.195645019	8	0.042748277	0.816496581	0.816496581	0.816496581	0.816496581	0.816496581	0.816496581	4	77	0	A_16_P37603310	A_16_P37603310	A_16_P37603310	chr6	45244607	45244667	+	SUPT3H	suppressor of Ty 3 homolog (S. cerevisiae) [Source:HGNC Symbol;Acc:HGNC:11466]	ENSG00000196284	protein_coding	intron	intron antisense	0	587	6:45244607:45244667	ENSG00000196284	SUPT3H	6.095340794	0.003674198	1.37180835	3.199338803	-0.753718846	1.827530453	-2.125527196	-3.953057649	9.961963646	0.20022454	0.009547113	0.468458111	0.097683169	0.059570631	0.002737	-6.05843355	-2.53884636	-6.745466271	-5.576002175	-4.328973081	-1.961176999	6.02835287	0.013067652

NCOA3	0.194634497	0.004192288	0.113821706	-0.190442209	-0.08081279	0.109629418	8	0.072010646	0.816496581	0.816496581	0.816496581	0.816496581	0.816496581	0.816496581	4	78	0	P_241016	P_241016	P_241016	chr20	47647228	47647288	+	NCOA3	nuclear receptor coactivator 3 [Source:HGNC Symbol;Acc:HGNC:7670]	ENSG00000124151	protein_coding	exon	coding exon	1	588	20:47647228:47647288	ENSG00000124151	NCOA3	6.795856927	0.00637095	2.986505743	0.064327196	1.746500158	-2.922178547	-1.240005585	1.682172962	9.961963646	0.013709851	0.949982209	0.111424949	0.015301715	0.243386531	0.123564375	-3.533845885	-6.179906466	-5.59550005	-3.784895024	-5.538039445	-5.712013062	4.146677421	0.037826676

GDF2	-0.015787072	-0.092398067	0.029891117	-0.076610995	0.045678189	0.122289184	8	0.18491617	0.816496581	0.816496581	0.816496581	0.816496581	0.816496581	0.816496581	4	79	0	A_23_P46755	A_23_P46755	A_23_P46755	chr10	47326205	47326265	+	GDF2	growth differentiation factor 2 [Source:HGNC Symbol;Acc:HGNC:4217]	ENSG00000263761	protein_coding	3'UTR	3'UTR	1	589	10:47326205:47326265	ENSG00000263761	GDF2	3.897453908	0.029666322	-0.112257501	-0.65701708	0.212547457	-0.54475958	0.324804958	0.869564537	9.961963646	0.912849015	0.526056943	0.835966041	0.597901518	0.75204664	0.405002793	-6.991488024	-5.959515198	-7.022505483	-6.989707943	-6.240876996	-6.673327089	0.274642226	0.842395597

CNOT4	0.005956245	0.008152022	-0.085751943	0.002195778	-0.091708187	-0.093903965	8	0.071525172	0.816496581	0.816496581	0.816496581	0.816496581	0.816496581	0.816496581	4	80	0	P_263009	P_263009	P_263009	chr7	135410523	135410583	-	CNOT4	"CCR4-NOT transcription complex, subunit 4 [Source:HGNC Symbol;Acc:HGNC:7880]"	ENSG00000080802	protein_coding	5'UTR;exon	multiple Ref	1	590	7:135410523:135410583	ENSG00000080802	CNOT4	5.859405724	0.006314991	0.0917977	0.125639044	-1.321609763	0.033841345	-1.413407462	-1.447248807	9.961963646	0.928678489	0.902517032	0.215844046	0.973672129	0.188013053	0.178552416	-6.993763829	-6.173772792	-6.168786633	-7.148986808	-5.331387233	-6.033583838	0.977376559	0.441717061

NCK2	0.26292211	0.033449829	0.109498367	-0.229472281	-0.153423743	0.076048538	8	0.163043041	0.816496581	0.816496581	0.816496581	0.816496581	0.816496581	0.816496581	4	81	0	P_128614	P_128614	P_128614	chr2	105893292	105893352	+	NCK2	NCK adaptor protein 2 [Source:HGNC Symbol;Acc:HGNC:7665]	ENSG00000071051	protein_coding	3'UTR	3'UTR	1	591	2:105893292:105893352	ENSG00000071051	NCK2	7.132603194	0.023554309	2.098154932	0.266934275	0.873812164	-1.831220656	-1.224342768	0.606877888	9.961963646	0.062374391	0.794964071	0.402793585	0.097096118	0.24898766	0.557509155	-5.012275799	-6.144663675	-6.645459874	-5.570486521	-5.555764494	-6.873830301	1.7429568	0.221539615

C2orf80	-0.494915676	0.082949227	-0.386521827	0.577864904	0.10839385	-0.469471054	8	0.139640247	0.816496581	0.816496581	0.816496581	0.816496581	0.816496581	0.816496581	4	82	0	P_238978	P_238978	P_238978	chr2	208181222	208181282	-	C2orf80	chromosome 2 open reading frame 80 [Source:HGNC Symbol;Acc:HGNC:34352]	ENSG00000188674	protein_coding	"5'UTR,exon;exon"	multiple Ref	1	592	2:208181222:208181282	ENSG00000188674	C2orf80	4.037487641	0.017865764	-4.534882515	0.76005877	-3.541676202	5.294941285	0.993206313	-4.301734972	9.961963646	0.001093743	0.464826932	0.005373341	0.000354556	0.344120858	0.001570879	-0.952274127	-5.886404075	-2.631164239	0.095735685	-5.797191275	-1.387995092	13.53568414	0.000756004

TMX3	-0.089778905	-0.04166643	-0.146249957	0.048112475	-0.056471052	-0.104583527	8	0.072621989	0.816496581	0.816496581	0.816496581	0.816496581	0.816496581	0.816496581	4	83	0	P_123751	P_123751	P_123751	chr18	68676540	68676600	-	TMX3	thioredoxin-related transmembrane protein 3 [Source:HGNC Symbol;Acc:HGNC:24718]	ENSG00000166479	protein_coding	3'UTR	3'UTR	1	593	18:68676540:68676600	ENSG00000166479	TMX3	7.889782055	0.006441956	-1.369969992	-0.635803685	-2.231682955	0.734166307	-0.861712963	-1.59587927	9.961963646	0.200779316	0.53923527	0.049798015	0.479770619	0.409108439	0.141716275	-6.060743946	-5.973366481	-4.845269768	-6.86259611	-5.916225833	-5.83348231	1.848308826	0.202461375

PPP4R3A	0.012628097	-0.134415979	-0.097852046	-0.147044077	-0.110480144	0.036563933	8	0.044760474	0.816496581	0.816496581	0.816496581	0.816496581	0.816496581	0.816496581	4	84	0	P_221910	P_221910	P_221910	chr14	91470842	91470902	-	SMEK1	"SMEK homolog 1, suppressor of mek1 (Dictyostelium) [Source:HGNC Symbol;Acc:HGNC:20219]"	ENSG00000100796	protein_coding	exon	coding exon	1	594	14:91470842:91470902	ENSG00000100796	PPP4R3A	8.529432356	0.003815604	0.250381317	-2.665108513	-1.940143743	-2.91548983	-2.190525061	0.72496477	9.961963646	0.807380511	0.023763144	0.081172543	0.015477647	0.053388343	0.485153543	-6.964258502	-3.393104182	-5.305659618	-3.79634571	-4.228855679	-6.791625101	4.125617807	0.038330236

OBSCN	0.174108899	-0.037621319	0.006484195	-0.211730218	-0.167624704	0.044105514	8	0.080987757	0.816496581	0.816496581	0.816496581	0.816496581	0.816496581	0.816496581	4	85	0	A_24_P119685	A_24_P119685	A_24_P119685	chr1	228360874	228360934	+	OBSCN	"obscurin, cytoskeletal calmodulin and titin-interacting RhoGEF [Source:HGNC Symbol;Acc:HGNC:15719]"	ENSG00000154358	protein_coding	3'UTR	3'UTR	1	595	1:228360874:228360934	ENSG00000154358	OBSCN	5.657578306	0.007473932	2.466561938	-0.532972838	0.091860139	-2.999534776	-2.374701799	0.624832977	9.961963646	0.033384483	0.605736886	0.928630127	0.013408486	0.039052824	0.546125027	-4.412994976	-6.034521869	-7.042514334	-3.65235516	-3.940332765	-6.862194869	3.56720998	0.055127009

LINC00667	0.092333578	-0.021954657	-0.04885284	-0.114288235	-0.141186419	-0.026898183	8	0.084577561	0.816496581	0.816496581	0.816496581	0.816496581	0.816496581	0.816496581	4	86	0	A_32_P150876	A_32_P150876	A_32_P150876	chr18	5245815	5245875	+	LINC00667	long intergenic non-protein coding RNA 667 [Source:HGNC Symbol;Acc:HGNC:27906]	ENSG00000263753	lincRNA	exon	non-coding exon	1	596	18:5245815:5245875	ENSG00000263753	LINC00667	7.919411646	0.007951225	1.268201513	-0.301547174	-0.670993667	-1.569748686	-1.93919518	-0.369446493	9.961963646	0.233561784	0.769197955	0.51747922	0.147662409	0.081300033	0.719526973	-6.185267982	-6.134377885	-6.806678311	-5.946376339	-4.60975919	-6.997322949	1.418429645	0.294671388

PDE4B	-0.193228245	0.139568115	-0.21068685	0.33279636	-0.017458606	-0.350254965	8	0.092963004	0.816496581	0.816496581	0.816496581	0.816496581	0.816496581	0.816496581	4	87	0	P_102070	P_102070	P_102070	chr1	65993070	65993130	+	PDE4B	"phosphodiesterase 4B, cAMP-specific [Source:HGNC Symbol;Acc:HGNC:8781]"	ENSG00000184588	protein_coding	exon	coding exon	1	597	1:65993070:65993130	ENSG00000184588	PDE4B	3.858152222	0.009146778	-2.474469905	1.787301335	-2.698043814	4.26177124	-0.223573908	-4.485345149	9.961963646	0.032936185	0.104298411	0.022459024	0.001672713	0.827606972	0.001180444	-4.399839307	-4.73068009	-4.070210445	-1.521102118	-6.270167438	-1.092024564	9.154648049	0.003266087

SLC1A4	-0.275612078	0.247893525	0.11416495	0.523505603	0.389777028	-0.133728575	8	0.10805804	0.816496581	0.816496581	0.816496581	0.816496581	0.816496581	0.816496581	4	88	0	P_127986	P_127986	P_127986	chr2	65018095	65018155	+	SLC1A4	"solute carrier family 1 (glutamate/neutral amino acid transporter), member 4 [Source:HGNC Symbol;Acc:HGNC:10942]"	ENSG00000115902	protein_coding	exon	coding exon	1	598	2:65018095:65018155	ENSG00000115902	SLC1A4	7.29209608	0.011583582	-3.13633489	2.820910888	1.299143056	5.957245777	4.435477945	-1.521767832	9.961963646	0.010623506	0.018196025	0.223156714	0.000142048	0.00127511	0.159155321	-3.277904138	-3.144300625	-6.196178685	1.052996267	-0.670111464	-5.934773043	12.82665444	0.000932392

IQSEC2	0.093236423	-0.058419252	0.083690441	-0.151655675	-0.009545983	0.142109693	8	0.084755469	0.816496581	0.816496581	0.816496581	0.816496581	0.816496581	0.816496581	4	89	0	A_32_P159445	A_32_P159445	A_32_P159445	chrX	53234545	53234605	-	IQSEC2	IQ motif and Sec7 domain 2 [Source:HGNC Symbol;Acc:HGNC:29059]	ENSG00000124313	protein_coding	3'UTR;exon	multiple Ref	1	599	X:53234545:53234605	ENSG00000124313	IQSEC2	7.705134727	0.007975418	1.278658333	-0.801170405	1.147743291	-2.079828738	-0.130915042	1.948913696	9.961963646	0.230001649	0.441722658	0.277890049	0.064321458	0.898448527	0.08000267	-6.172786222	-5.854600765	-6.371793611	-5.187415017	-6.287577479	-5.315722005	1.955874455	0.184917869

SH3GLB2	-0.026485497	0.040297761	0.003199257	0.066783258	0.029684754	-0.037098505	8	0.21334234	0.816496581	0.816496581	0.816496581	0.816496581	0.816496581	0.816496581	4	90	0	P_149853	P_149853	P_149853	chr9	129010403	129010463	-	SH3GLB2	SH3-domain GRB2-like endophilin B2 [Source:HGNC Symbol;Acc:HGNC:10834]	ENSG00000148341	protein_coding	exon	non-coding exon	1	600	9:129010403:129010463	ENSG00000148341	SH3GLB2	8.229595053	0.038757674	-0.164768772	0.250696169	0.019902877	0.415464941	0.184671648	-0.230793293	9.961963646	0.872421367	0.807143802	0.984513844	0.686608709	0.857191908	0.822146152	-6.983565772	-6.149063867	-7.046904279	-7.056031172	-6.278578007	-7.04235744	0.05839562	0.980438122

OR10S1	-0.283151414	0.207146859	-0.411373405	0.490298273	-0.128221991	-0.618520264	8	0.301805876	0.816496581	0.816496581	0.816496581	0.816496581	0.816496581	0.816496581	4	91	0	P_110367	P_110367	P_110367	chr11	123976735	123976795	-	OR10S1	"olfactory receptor, family 10, subfamily S, member 1 [Source:HGNC Symbol;Acc:HGNC:14807]"	ENSG00000196248	protein_coding	exon	coding exon	1	601	11:123976735:123976795	ENSG00000196248	OR10S1	3.100146265	0.07535434	-1.263310856	0.92420826	-1.835387228	2.187519115	-0.572076372	-2.759595487	9.961963646	0.235242291	0.3772213	0.0964372	0.053660121	0.579949539	0.020210869	-6.191080404	-5.750827818	-5.464301784	-5.015422373	-6.125505195	-3.988605091	3.091106668	0.076741986

IBTK	0.035221923	0.083289831	0.064807779	0.048067909	0.029585856	-0.018482053	8	0.056678267	0.816496581	0.816496581	0.816496581	0.816496581	0.816496581	0.816496581	4	93	0	P_257999	P_257999	P_257999	chr6	82214231	82214291	-	IBTK	inhibitor of Bruton agammaglobulinemia tyrosine kinase [Source:HGNC Symbol;Acc:HGNC:17853]	ENSG00000005700	protein_coding	3'UTR;exon	multiple Ref	1	603	6:82214231:82214291	ENSG00000005700	IBTK	8.494184706	0.004786437	0.623523042	1.474454687	1.147272501	0.850931645	0.523749459	-0.327182186	9.961963646	0.546951048	0.171246104	0.278075563	0.414792573	0.611905118	0.750301335	-6.790273726	-5.149956477	-6.372314268	-6.767447724	-6.152819841	-7.01321191	0.830746357	0.506896799

BMP7	-0.104776953	-0.026772748	0.042824363	0.078004204	0.147601316	0.069597112	8	0.244936238	0.816496581	0.816496581	0.816496581	0.816496581	0.816496581	0.816496581	4	94	0	A_23_P68487	A_23_P68487	A_23_P68487	chr20	57170498	57170558	-	BMP7	bone morphogenetic protein 7 [Source:HGNC Symbol;Acc:HGNC:1074]	ENSG00000101144	protein_coding	3'UTR	3'UTR	1	604	20:57170498:57170558	ENSG00000101144	BMP7	3.740399009	0.050384945	-0.571690529	-0.146079136	0.233660957	0.425611393	0.805351486	0.379740093	9.961963646	0.580201062	0.886772571	0.819979765	0.679439277	0.439416117	0.712108234	-6.822898259	-6.170850582	-7.017386392	-7.051446154	-5.96283135	-6.993168894	0.229450872	0.87379046

LRRFIP1	-0.298605561	-0.075658901	-0.083854314	0.22294666	0.214751247	-0.008195413	8	0.095816669	0.816496581	0.816496581	0.816496581	0.816496581	0.816496581	0.816496581	4	95	0	P_239818	P_239818	P_239818	chr2	237727875	237727935	+	LRRFIP1	leucine rich repeat (in FLII) interacting protein 1 [Source:HGNC Symbol;Acc:HGNC:6702]	ENSG00000124831	protein_coding	exon	coding exon	1	605	2:237727875:237727935	ENSG00000124831	LRRFIP1	7.191169858	0.009579394	-3.736581811	-0.946752878	-1.049305662	2.789828933	2.687276149	-0.102552783	9.961963646	0.003893828	0.366163311	0.3188267	0.019190779	0.022877312	0.920352889	-2.258880608	-5.730462364	-6.477026487	-4.011059564	-3.439229969	-7.065636063	5.162423777	0.020742144

TACC1	-0.294545853	-0.046803262	-0.316020054	0.247742591	-0.021474201	-0.269216792	8	0.06173769	0.816496581	0.816496581	0.816496581	0.816496581	0.816496581	0.816496581	4	96	0	P_264448	P_264448	P_264448	chr8	38843301	38843361	+	TACC1	"transforming, acidic coiled-coil containing protein 1 [Source:HGNC Symbol;Acc:HGNC:11522]"	ENSG00000147526	protein_coding	exon	coding exon	1	606	8:38843301:38843361	ENSG00000147526	TACC1	9.161045728	0.00526756	-4.970427944	-0.789799747	-5.332802657	4.180628197	-0.362374713	-4.54300291	9.961963646	0.000567757	0.448035851	0.000335934	0.001901511	0.724641528	0.001080187	-0.273509348	-5.863544102	0.233545025	-1.654004074	-6.227311842	-0.999979599	15.33592746	0.000460367

HELZ	0.008213094	-0.083683037	-0.091684278	-0.09189613	-0.099897371	-0.008001241	8	0.048004076	0.816496581	0.816496581	0.816496581	0.816496581	0.816496581	0.816496581	4	97	0	P_229759	P_229759	P_229759	chr17	67107186	67107246	-	HELZ	helicase with zinc finger [Source:HGNC Symbol;Acc:HGNC:16878]	ENSG00000198265	protein_coding	3'UTR;exon	multiple Ref	1	607	17:67107186:67107246	ENSG00000198265	HELZ	8.030107774	0.004057236	0.15792005	-1.609044052	-1.762890637	-1.766964102	-1.920810687	-0.153846585	9.961963646	0.877674953	0.138801754	0.108510303	0.107796816	0.083808282	0.880802644	-6.984768585	-4.975118934	-5.571558929	-5.66572695	-4.636894101	-7.058463637	2.092848251	0.165074964

ARMC7	0.013138896	0.128627348	0.080940083	0.115488452	0.067801187	-0.047687265	8	0.109928241	0.816496581	0.816496581	0.816496581	0.816496581	0.816496581	0.816496581	4	98	0	P_122543	P_122543	P_122543	chr17	75128737	75128797	+	ARMC7	armadillo repeat containing 7 [Source:HGNC Symbol;Acc:HGNC:26168]	ENSG00000125449	protein_coding	exon	coding exon	1	608	17:75128737:75128797	ENSG00000125449	ARMC7	8.177715435	0.01191097	0.147445301	1.443462076	0.908313373	1.296016774	0.760868072	-0.535148703	9.961963646	0.88572201	0.179590336	0.385159472	0.224190352	0.464364712	0.604286457	-6.986509937	-5.188888851	-6.614379855	-6.301187606	-5.997666464	-6.917148106	0.912553317	0.46937953

TTLL4	-0.421282795	-0.003838473	-0.242945154	0.417444322	0.178337641	-0.239106682	8	0.054016751	0.816496581	0.816496581	0.816496581	0.816496581	0.816496581	0.816496581	4	99	0	A_23_P142697	A_23_P142697	A_23_P142697	chr2	218753608	218753668	+	TTLL4	"tubulin tyrosine ligase-like family, member 4 [Source:HGNC Symbol;Acc:HGNC:28976]"	ENSG00000135912	protein_coding	exon	coding exon	1	609	2:218753608:218753668	ENSG00000135912	TTLL4	6.680644085	0.004549844	-7.649289494	-0.069695678	-4.41118849	7.579593816	3.238101005	-4.341492811	9.961963646	1.78E-05	0.945815074	0.001324099	1.92E-05	0.008941066	0.001476043	3.310085836	-6.179527345	-1.190495176	3.142575129	-2.544091414	-1.323546354	27.45986192	3.93E-05

PLA2G15	-0.212575466	0.015653128	-0.244105013	0.228228595	-0.031529546	-0.259758141	8	0.072981352	0.816496581	0.816496581	0.816496581	0.816496581	0.816496581	0.816496581	4	100	0	P_119352	P_119352	P_119352	chr16	68260939	68260999	+	PLA2G15	"phospholipase A2, group XV [Source:HGNC Symbol;Acc:HGNC:17163]"	ENSG00000103066	protein_coding	3'UTR;exon	multiple Ref	1	610	16:68260939:68260999	ENSG00000103066	PLA2G15	7.710356832	0.006483975	-3.233241093	0.238081742	-3.712800785	3.471322835	-0.479559692	-3.950882527	9.961963646	0.009014833	0.816643184	0.004048963	0.006041735	0.64189467	0.002746633	-3.112389347	-6.15229403	-2.341889235	-2.844118164	-6.175800113	-1.9647946	8.697492171	0.003912097

RGS12	0.292208364	-0.007543044	0.235676679	-0.299751407	-0.056531685	0.243219722	8	0.059323949	0.816496581	0.816496581	0.816496581	0.816496581	0.816496581	0.816496581	4	101	0	P_137215	P_137215	P_137215	chr4	3432117	3432177	+	RGS12	regulator of G-protein signaling 12 [Source:HGNC Symbol;Acc:HGNC:9994]	ENSG00000159788	protein_coding	3'UTR	3'UTR	1	611	4:3432117:3432177	ENSG00000159788	RGS12	7.857315869	0.005032899	5.044628393	-0.130221637	4.068676368	-5.17485003	-0.975952024	4.198898006	9.961963646	0.000509131	0.898983062	0.002272716	0.0004213	0.352191599	0.001847264	-0.16053663	-6.173155736	-1.748501647	-0.084581796	-5.81360704	-1.555591756	14.56361005	0.000566131

ANP32A	-0.080614859	-0.18396031	-0.241513495	-0.103345451	-0.160898637	-0.057553185	8	0.103734922	0.816496581	0.816496581	0.816496581	0.816496581	0.816496581	0.816496581	4	102	0	A_23_P14649	A_23_P14649	A_23_P14649	chr15	68804523	68804583	-	ANP32A;MIR4312	"acidic (leucine-rich) nuclear phosphoprotein 32 family, member A [Source:HGNC Symbol;Acc:HGNC:13233];microRNA 4312 [Source:HGNC Symbol;Acc:HGNC:38376]"	ENSG00000140350	protein_coding;miRNA	intron;promoter	multiple non-Ref	0	612	15:68804523:68804583	ENSG00000140350	ANP32A	8.485155287	0.010848301	-0.947937856	-2.163161295	-2.839920445	-1.215223439	-1.891982588	-0.676759149	9.961963646	0.365588611	0.055912022	0.017613413	0.252296491	0.087887297	0.513965331	-6.529048693	-4.178208711	-3.829148751	-6.396879106	-4.679215333	-6.826790613	3.192895028	0.071377955

PPARGC1B	-0.498311279	0.030292937	-0.327482827	0.528604216	0.170828452	-0.357775764	8	0.105974319	0.816496581	0.816496581	0.816496581	0.816496581	0.816496581	0.816496581	4	103	0	A_32_P31832	A_32_P31832	A_32_P31832	chr5	149733743	149733803	+	PPARGC1B	"peroxisome proliferator-activated receptor gamma, coactivator 1 beta [Source:HGNC Symbol;Acc:HGNC:30022]"	ENSG00000155846	protein_coding	intron	intron sense	0	613	5:149733743:149733803	ENSG00000155846	PPARGC1B	7.384048889	0.011225433	-5.760295913	0.350175261	-3.785581558	6.110471175	1.974714355	-4.13575682	9.961963646	0.000185267	0.733497966	0.003593411	0.000115948	0.076652028	0.002041989	0.88808893	-6.117855322	-2.219557611	1.265549392	-4.557024528	-1.659116226	17.66268312	0.000258836

ARSB	0.035442559	0.362595669	0.09239429	0.327153111	0.056951731	-0.270201379	8	0.234734668	0.816496581	0.816496581	0.816496581	0.816496581	0.816496581	0.816496581	4	104	0	P_139814	P_139814	P_139814	chr5	78779961	78780021	-	ARSB	arylsulfatase B [Source:HGNC Symbol;Acc:HGNC:714]	ENSG00000113273	protein_coding	3'UTR	3'UTR	1	614	5:78779961:78780021	ENSG00000113273	ARSB	5.91937669	0.046455281	0.201397072	2.060395998	0.525016821	1.858998926	0.323619749	-1.535379177	9.961963646	0.844440911	0.06644924	0.611055652	0.092780552	0.752917342	0.155817866	-6.97627192	-4.333340549	-6.898649418	-5.528792916	-6.241281351	-5.91638973	1.746571847	0.220851472

TSC2	-0.150693074	-0.051256136	-0.217865483	0.099436938	-0.067172409	-0.166609347	8	0.100421338	0.816496581	0.816496581	0.816496581	0.816496581	0.816496581	0.816496581	4	105	0	P_117939	P_117939	P_117939	chr16	2082443	2082503	+	TSC2	tuberous sclerosis 2 [Source:HGNC Symbol;Acc:HGNC:12363]	ENSG00000103197	protein_coding	3'UTR;exon	multiple Ref	1	615	16:2082443:2082503	ENSG00000103197	TSC2	8.411444231	0.010305043	-1.818084277	-0.618395875	-2.628507057	1.199688402	-0.81042278	-2.010111181	9.961963646	0.099200841	0.55019101	0.025301353	0.258014624	0.436629231	0.072269535	-5.442715781	-5.984420717	-4.18773612	-6.414760085	-5.958749921	-5.220994969	2.788912925	0.095721608

TRIM27	-0.089265099	-0.014422915	-0.126113684	0.074842184	-0.036848585	-0.111690768	8	0.045944218	0.816496581	0.816496581	0.816496581	0.816496581	0.816496581	0.816496581	4	106	0	A_32_P137035	A_32_P137035	A_32_P137035	chr6	28904076	28904136	-	TRIM27	tripartite motif containing 27 [Source:HGNC Symbol;Acc:HGNC:9975]	ENSG00000204713	protein_coding	3'UTR;exon	multiple Ref	1	616	6:28904076:28904136	ENSG00000204713	TRIM27	8.781276246	0.003901829	-1.750222352	-0.282790349	-2.472713197	1.467432002	-0.722490845	-2.189922848	9.961963646	0.110756853	0.783126393	0.033035256	0.173106943	0.486607227	0.053442685	-5.542590536	-6.140104658	-4.448979267	-6.08415749	-6.026307229	-4.936005225	2.788111833	0.095778852

LMBR1L	0.042719069	0.036413643	0.060894699	-0.006305426	0.01817563	0.024481056	8	0.119846352	0.816496581	0.816496581	0.816496581	0.816496581	0.816496581	0.816496581	4	107	0	P_111460	P_111460	P_111460	chr12	49106422	49106482	-	LMBR1L	limb development membrane protein 1-like [Source:HGNC Symbol;Acc:HGNC:18268]	ENSG00000139636	protein_coding	exon	non-coding exon	1	617	12:49106422:49106482	ENSG00000139636	LMBR1L	6.26768963	0.013741075	0.446330999	0.380451591	0.636230909	-0.065879408	0.18989991	0.255779318	9.961963646	0.664903246	0.7115966	0.538967989	0.948777179	0.853201203	0.803325086	-6.890733649	-6.106354785	-6.830466347	-7.14724013	-6.27754016	-7.035755354	0.142428279	0.932186706

ACACB	-0.007309339	-0.16383356	-0.02219041	-0.15652422	-0.014881071	0.141643149	8	0.095247698	0.816496581	0.816496581	0.816496581	0.816496581	0.816496581	0.816496581	4	108	0	P_217346	P_217346	P_217346	chr12	109175976	109176036	+	ACACB	acetyl-CoA carboxylase beta [Source:HGNC Symbol;Acc:HGNC:85]	ENSG00000076555	protein_coding	5'UTR;exon	multiple Ref	1	618	12:109175976:109176036	ENSG00000076555	ACACB	5.922039851	0.009492094	-0.091884603	-2.059526935	-0.278952295	-1.967642333	-0.187067692	1.780574641	9.961963646	0.928611179	0.066545944	0.78598658	0.07755709	0.855362489	0.105443859	-6.993755125	-4.334641054	-7.004791706	-5.36292658	-6.278105946	-5.569245008	1.900827514	0.193665163

FREM2	0.15986609	0.007917481	0.083590784	-0.151948609	-0.076275306	0.075673303	8	0.141343182	0.816496581	0.816496581	0.816496581	0.816496581	0.816496581	0.816496581	4	109	0	P_218840	P_218840	P_218840	chr13	38850170	38850230	+	FREM2	FRAS1 related extracellular matrix protein 2 [Source:HGNC Symbol;Acc:HGNC:25396]	ENSG00000150893	protein_coding	exon	coding exon	1	619	13:38850170:38850230	ENSG00000150893	FREM2	3.600126268	0.018250023	1.449339981	0.071779583	0.757830914	-1.377560398	-0.691509067	0.686051331	9.961963646	0.177981451	0.944197955	0.466100866	0.198497046	0.505041338	0.508332342	-5.959118078	-6.179372005	-6.742257309	-6.20021826	-6.048457488	-6.820182782	0.921095042	0.465632277

TRIB2	0.302936536	-0.178577304	0.124343569	-0.48151384	-0.178592967	0.302920873	8	0.266445917	0.816496581	0.816496581	0.816496581	0.816496581	0.816496581	0.816496581	4	110	0	P_127220	P_127220	P_127220	chr2	12716950	12717010	+	TRIB2	tribbles pseudokinase 2 [Source:HGNC Symbol;Acc:HGNC:30809]	ENSG00000071575	protein_coding	5'UTR;exon	multiple Ref	1	620	2:12716950:12717010	ENSG00000071575	TRIB2	4.604396986	0.059218276	1.524647349	-0.898760569	0.625807951	-2.423407919	-0.898839398	1.524568521	9.961963646	0.158444105	0.389986746	0.545510691	0.035938811	0.389946739	0.158463538	-5.859292591	-5.773322143	-6.837371815	-4.628994536	-5.884053402	-5.930998728	2.088180512	0.165708955

KIAA0895L	0.001212827	-0.030043642	-0.002604155	-0.031256469	-0.003816982	0.027439487	8	0.068432019	0.816496581	0.816496581	0.816496581	0.816496581	0.816496581	0.816496581	4	111	0	P_119239	P_119239	P_119239	chr16	67179814	67179874	-	KIAA0895L	KIAA0895-like [Source:HGNC Symbol;Acc:HGNC:34408]	ENSG00000196123	protein_coding	exon	coding exon	1	621	16:67179814:67179874	ENSG00000196123	KIAA0895L	8.427373125	0.005967341	0.019228889	-0.476329823	-0.041287822	-0.495558712	-0.060516711	0.435042001	9.961963646	0.985038192	0.644114286	0.967882217	0.630954707	0.952940959	0.672805576	-6.998158552	-6.063875255	-7.046189737	-7.0169529	-6.294737713	-6.968961036	0.111244465	0.951543577

RNPEP	-0.019660254	0.016265004	-0.050387956	0.035925258	-0.030727703	-0.06665296	8	0.077887094	0.816496581	0.816496581	0.816496581	0.816496581	0.816496581	0.816496581	4	112	0	P_104430	P_104430	P_104430	chr1	201997494	201997554	+	RNPEP	arginyl aminopeptidase (aminopeptidase B) [Source:HGNC Symbol;Acc:HGNC:10078]	ENSG00000176393	protein_coding	3'UTR;exon	multiple Ref	1	622	1:201997494:201997554	ENSG00000176393	RNPEP	9.117243355	0.007078334	-0.286199743	0.236774149	-0.733511391	0.522973892	-0.447311648	-0.97028554	9.961963646	0.780588491	0.817629691	0.480152498	0.612425249	0.664218799	0.354872123	-6.95384676	-6.152619442	-6.761008958	-7.002072676	-6.191351023	-6.580093064	0.348509114	0.791157248

GON7	-0.232735595	0.133538724	-0.074639657	0.366274318	0.158095937	-0.208178381	8	0.081751451	0.816496581	0.816496581	0.816496581	0.816496581	0.816496581	0.816496581	4	113	0	P_115643	P_115643	P_115643	chr14	93203687	93203747	-	C14orf142	chromosome 14 open reading frame 142 [Source:HGNC Symbol;Acc:HGNC:20356]	ENSG00000170270	protein_coding	exon	coding exon	1	623	14:93203687:93203747	ENSG00000170270	GON7	8.710499722	0.007573738	-3.275316688	1.879306905	-1.05041309	5.154623593	2.224903597	-2.929719996	9.961963646	0.008396342	0.08973871	0.318341874	0.000433806	0.050372923	0.015105783	-3.040579214	-4.599569145	-6.475884064	-0.115154939	-4.175510991	-3.698553663	9.244410193	0.003154731

GPR156	-0.206250886	0.078609914	0.136433795	0.2848608	0.342684681	0.057823881	8	0.148955877	0.816496581	0.816496581	0.816496581	0.816496581	0.816496581	0.816496581	4	114	0	A_23_P92275	A_23_P92275	A_23_P92275	chr3	120167166	120167226	-	GPR156	G protein-coupled receptor 156 [Source:HGNC Symbol;Acc:HGNC:20844]	ENSG00000175697	protein_coding	3'UTR;exon	multiple Ref	1	624	3:120167166:120167226	ENSG00000175697	GPR156	4.44763672	0.02002474	-1.785081334	0.68036115	1.180821208	2.465442484	2.965902542	0.500460057	9.961963646	0.104675174	0.511777322	0.265098365	0.033448428	0.014200523	0.627621684	-5.49152952	-5.943793701	-6.334806232	-4.559002114	-2.986604143	-6.936254896	3.361531698	0.063437099

DHTKD1	-0.120883145	0.104778181	-0.103073464	0.225661326	0.017809681	-0.207851645	8	0.049839849	0.816496581	0.816496581	0.816496581	0.816496581	0.816496581	0.816496581	4	115	0	P_207395	P_207395	P_207395	chr10	12123071	12123131	-	DHTKD1	dehydrogenase E1 and transketolase domain containing 1 [Source:HGNC Symbol;Acc:HGNC:23537]	ENSG00000181192	protein_coding	3'UTR	3'UTR antisense	0	625	10:12123071:12123131	ENSG00000181192	DHTKD1	7.39049463	0.00420148	-2.284074352	1.979772743	-1.947562299	4.263847096	0.336512053	-3.927335043	9.961963646	0.045564333	0.07601077	0.080181905	0.001667255	0.743465926	0.002853226	-4.713541018	-4.45309551	-5.294272487	-1.517712388	-6.236805282	-2.003988907	7.774514604	0.005751339

HOXC5	0.106759285	-0.008562693	0.27201128	-0.115321979	0.165251995	0.280573974	8	0.127149883	0.816496581	0.816496581	0.816496581	0.816496581	0.816496581	0.816496581	4	116	0	P_111757	P_111757	P_111757	chr12	54033149	54033209	+	HOXC5	homeobox C5 [Source:HGNC Symbol;Acc:HGNC:5127]	ENSG00000172789	protein_coding	exon	coding exon	1	626	12:54033149:54033209	ENSG00000172789	HOXC5	6.282247475	0.015189741	1.060904041	-0.085090454	2.703070427	-1.145994495	1.642166385	2.78816088	9.961963646	0.313776672	0.933875269	0.022266386	0.278579649	0.131703542	0.019245686	-6.416723704	-6.178271943	-4.061696752	-6.475226891	-5.032755187	-3.940023753	3.370533736	0.063043532

DSTYK	0.012851616	-0.059917511	-0.087722494	-0.072769127	-0.10057411	-0.027804983	8	0.083068786	0.816496581	0.816496581	0.816496581	0.816496581	0.816496581	0.816496581	4	117	0	P_205879	P_205879	P_205879	chr1	205163722	205163782	-	DSTYK	dual serine/threonine and tyrosine protein kinase [Source:HGNC Symbol;Acc:HGNC:29043]	ENSG00000133059	protein_coding	exon	coding exon	1	627	1:205163722:205163782	ENSG00000133059	DSTYK	7.740597206	0.0077481	0.178815747	-0.833684607	-1.220559596	-1.012500353	-1.399375343	-0.38687499	9.961963646	0.861666701	0.42399729	0.250356059	0.335258597	0.192059517	0.706984393	-6.980939941	-5.828413959	-6.289330963	-6.616160419	-5.348787746	-6.990224508	0.891641899	0.478687139

RABGAP1	0.09239951	-0.063772511	0.04772844	-0.156172021	-0.04467107	0.111500951	8	0.038872803	0.816496581	0.816496581	0.816496581	0.816496581	0.816496581	0.816496581	4	118	0	P_268868	P_268868	P_268868	chr9	122989411	122989471	+	RABGAP1	RAB GTPase activating protein 1 [Source:HGNC Symbol;Acc:HGNC:17155]	ENSG00000011454	protein_coding	3'UTR;exon	multiple Ref	1	628	9:122989411:122989471	ENSG00000011454	RABGAP1	7.693653682	0.003420176	1.935045144	-1.335534007	0.999536527	-3.27057915	-0.935508617	2.335070534	9.961963646	0.081860019	0.211412687	0.341194329	0.008463746	0.371649062	0.041780232	-5.266274793	-5.320243459	-6.52736386	-3.187449056	-5.851155724	-4.699890427	3.925257135	0.043543403

SLC25A37	-0.31358713	-0.075398032	-0.326765743	0.238189097	-0.013178613	-0.25136771	8	0.038594653	0.816496581	0.816496581	0.816496581	0.816496581	0.816496581	0.816496581	4	119	0	A_24_P201089	A_24_P201089	A_24_P201089	chr8	23566255	23566315	+	SLC25A37	"solute carrier family 25 (mitochondrial iron transporter), member 37 [Source:HGNC Symbol;Acc:HGNC:29786]"	ENSG00000147454	protein_coding	exon	coding exon	1	629	8:23566255:23566315	ENSG00000147454	SLC25A37	7.935638768	0.003402872	-6.583867417	-1.583007089	-6.860556843	5.000860327	-0.276689426	-5.277549753	9.961963646	6.31E-05	0.144618378	4.49E-05	0.000542886	0.787674481	0.000363477	2.003471497	-5.009641216	2.327259962	-0.349479144	-6.256126437	0.132584229	24.30969237	6.69E-05

GIGYF2	0.059850234	-0.073078397	-0.001353678	-0.132928631	-0.061203912	0.071724719	8	0.087720883	0.816496581	0.816496581	0.816496581	0.816496581	0.816496581	0.816496581	4	120	0	P_239700	P_239700	P_239700	chr2	232796161	232796221	+	GIGYF2	GRB10 interacting GYF protein 2 [Source:HGNC Symbol;Acc:HGNC:11960]	ENSG00000204120	protein_coding	exon	coding exon	1	630	2:232796161:232796221	ENSG00000204120	GIGYF2	8.062937248	0.008386151	0.800442502	-0.977357169	-0.018104217	-1.777799671	-0.818546719	0.959252952	9.961963646	0.442125032	0.351529194	0.98591318	0.10591974	0.432189386	0.360133713	-6.659602303	-5.702168845	-7.046941611	-5.649788089	-5.95216518	-6.590721878	1.057836604	0.409776964

GPR143	0.677628348	0.208453628	0.755431709	-0.46917472	0.077803361	0.546978081	8	0.071321709	0.816496581	0.816496581	0.816496581	0.816496581	0.816496581	0.816496581	4	122	0	P_269447	P_269447	P_269447	chrX	9743564	9743624	-	GPR143	G protein-coupled receptor 143 [Source:HGNC Symbol;Acc:HGNC:20145]	ENSG00000101850	protein_coding	exon	coding exon	1	632	X:9743564:9743624	ENSG00000101850	GPR143	7.174973651	0.006291651	10.46296796	3.218642836	11.6642962	-7.244325122	1.201328245	8.445653367	9.961963646	1.08E-06	0.009240196	3.95E-07	2.83E-05	0.257406165	7.48E-06	6.144814987	-2.508062393	7.140218077	2.738805332	-5.581510371	4.164134551	63.52361049	8.48E-07

PPFIBP2	-0.299237824	0.133904514	-0.233336467	0.433142338	0.065901357	-0.367240981	8	0.077854971	0.816496581	0.816496581	0.816496581	0.816496581	0.816496581	0.816496581	4	123	0	A_23_P409623	A_23_P409623	A_23_P409623	chr11	7653441	7653501	+	PPFIBP2	"PTPRF interacting protein, binding protein 2 (liprin beta 2) [Source:HGNC Symbol;Acc:HGNC:9250]"	ENSG00000166387	protein_coding	3'UTR;exon	multiple Ref	1	633	11:7653441:7653501	ENSG00000166387	PPFIBP2	7.001078476	0.007074316	-4.357324378	1.949838412	-3.397707757	6.307162791	0.959616621	-5.347546169	9.961963646	0.001439981	0.079880242	0.006833844	8.98E-05	0.359959376	0.000328968	-1.236358704	-4.49707421	-2.875999025	1.533574427	-5.828931112	0.236500964	17.27166336	0.000283849

C12orf57	-0.024816183	0.01728615	-0.104454246	0.042102334	-0.079638063	-0.121740396	8	0.094734196	0.816496581	0.816496581	0.816496581	0.816496581	0.816496581	0.816496581	4	125	0	A_23_P2489	A_23_P2489	A_23_P2489	chr12	6943838	6943898	+	C12orf57	chromosome 12 open reading frame 57 [Source:HGNC Symbol;Acc:HGNC:29521]	ENSG00000111678	protein_coding	5'UTR	5'UTR	1	635	12:6943838:6943898	ENSG00000111678	C12orf57	11.2882286	0.009413751	-0.313255898	0.218203921	-1.318531065	0.53145982	-1.005275167	-1.536734986	9.961963646	0.76054641	0.831675168	0.216834204	0.606746524	0.338557246	0.155488807	-6.945076104	-6.157049519	-6.172559761	-6.997313033	-5.78556997	-5.914553077	0.923141416	0.464739203

PRMT7	-0.02619877	0.21603558	-0.043876909	0.24223435	-0.01767814	-0.25991249	8	0.140240585	0.816496581	0.816496581	0.816496581	0.816496581	0.816496581	0.816496581	4	127	0	P_119359	P_119359	P_119359	chr16	68351007	68351067	-	PRMT7	protein arginine methyltransferase 7 [Source:HGNC Symbol;Acc:HGNC:25557]	ENSG00000132600	protein_coding	intron;promoter	multiple non-Ref	0	637	16:68351007:68351067	ENSG00000132600	PRMT7	7.641357822	0.018000696	-0.239156331	1.972087886	-0.400531808	2.211244216	-0.161375477	-2.372619694	9.961963646	0.815832718	0.076986993	0.697219801	0.051550809	0.875023533	0.039191676	-6.967239028	-4.464411984	-6.960213593	-4.977117786	-6.282852516	-4.638078065	2.441953225	0.124782777

LMNTD1	0.139068103	-0.217953426	0.374090395	-0.357021529	0.235022292	0.592043821	8	0.15929976	0.816496581	0.816496581	0.816496581	0.816496581	0.816496581	0.816496581	4	128	0	P_214739	P_214739	P_214739	chr12	25519857	25519917	-	LMNTD1	lamin tail domain containing 1 [Source:HGNC Symbol;Acc:HGNC:26683]	ENSG00000152936	protein_coding	exon	coding exon	1	638	12:25519857:25519917	ENSG00000152936	LMNTD1	3.641164467	0.022585328	1.13333933	-1.776217438	3.048659962	-2.909556768	1.915320632	4.8248774	9.961963646	0.283610092	0.106191957	0.012331536	0.015635417	0.084571216	0.000704707	-6.339425186	-4.746262392	-3.472601453	-3.806501247	-4.644975497	-0.556432769	8.191191603	0.004815346

TIAM2	0.128025834	0.018041519	0.100422338	-0.109984315	-0.027603496	0.082380819	8	0.063888768	0.816496581	0.816496581	0.816496581	0.816496581	0.816496581	0.816496581	4	129	0	P_259980	P_259980	P_259980	chr6	155164432	155164492	+	TIAM2	T-cell lymphoma invasion and metastasis 2 [Source:HGNC Symbol;Acc:HGNC:11806]	ENSG00000146426	protein_coding	"5'UTR,exon;exon"	multiple Ref	1	639	6:155164432:155164492	ENSG00000146426	TIAM2	4.218656417	0.005484572	2.117248753	0.298364654	1.660751292	-1.818884099	-0.456497461	1.362386638	9.961963646	0.060405637	0.771555327	0.127864509	0.0990715	0.657823331	0.203081515	-4.982010278	-6.135374991	-5.718779388	-5.588904097	-6.187026846	-6.142137452	2.11711421	0.161824337

CPNE1	0.07321901	-0.100370545	-0.03244515	-0.173589555	-0.105664161	0.067925394	8	0.070380632	0.816496581	0.816496581	0.816496581	0.816496581	0.816496581	0.816496581	4	131	0	P_240676	P_240676	P_240676	chr20	35631267	35631327	-	CPNE1	copine I [Source:HGNC Symbol;Acc:HGNC:2314]	ENSG00000214078	protein_coding	exon	coding exon	1	641	20:35631267:35631327	ENSG00000214078	CPNE1	9.90107475	0.006184561	1.140289288	-1.56313854	-0.505290324	-2.703427828	-1.645579611	1.057848216	9.961963646	0.280838734	0.149201074	0.624345569	0.022252753	0.130990835	0.31510127	-6.331800628	-5.035765848	-6.909458508	-4.158083399	-5.02810206	-6.492230933	2.522412866	0.117209873

AEN	-0.249310161	0.070373679	-0.145093711	0.319683841	0.10421645	-0.215467391	8	0.080925671	0.816496581	0.816496581	0.816496581	0.816496581	0.816496581	0.816496581	4	132	0	P_117426	P_117426	P_117426	chr15	88630182	88630242	+	AEN	apoptosis enhancing nuclease [Source:HGNC Symbol;Acc:HGNC:25722]	ENSG00000181026	protein_coding	exon	coding exon	1	642	15:88630182:88630242	ENSG00000181026	AEN	8.729243617	0.007465859	-3.533830424	0.997507073	-2.056621234	4.531337497	1.477209191	-3.054128307	9.961963646	0.005443922	0.342130557	0.066870244	0.001099718	0.170520964	0.012217236	-2.600885291	-5.683139181	-5.12474432	-1.085394209	-5.250864274	-3.48571356	8.292589158	0.004615929

DLX6	0.03705192	-0.077450071	-0.090777039	-0.114501991	-0.127828958	-0.013326967	8	0.167568145	0.816496581	0.816496581	0.816496581	0.816496581	0.816496581	0.816496581	4	133	0	P_145271	P_145271	P_145271	chr7	97009813	97009873	+	DLX6	distal-less homeobox 6 [Source:HGNC Symbol;Acc:HGNC:2919]	ENSG00000006377	protein_coding	exon	coding exon	1	643	7:97009813:97009873	ENSG00000006377	DLX6	3.461235852	0.024755719	0.288415483	-0.602878341	-0.706616656	-0.891293824	-0.995032139	-0.103738315	9.961963646	0.778940574	0.560063106	0.495999498	0.393789385	0.343274862	0.919435757	-6.953157742	-5.994035666	-6.781106309	-6.731731696	-5.795440471	-7.0655026	0.456868027	0.718390788

FAM149A	0.122671228	0.34276665	0.354879176	0.220095422	0.232207948	0.012112526	8	0.10737244	0.816496581	0.816496581	0.816496581	0.816496581	0.816496581	0.816496581	4	135	0	A_23_P416965	A_23_P416965	A_23_P416965	chr4	186167078	186167225	+	FAM149A	"family with sequence similarity 149, member A [Source:HGNC Symbol;Acc:HGNC:24527]"	ENSG00000109794	protein_coding	"3'UTR,intron;exon,intron;exon,promoter"	multiple non-Ref	0	645	4:186167078:186167225	ENSG00000109794	FAM149A	6.286343928	0.011464972	1.403142717	3.920646572	4.059192528	2.517503854	2.656049811	0.138545956	9.961963646	0.190965907	0.002884287	0.002307485	0.030598801	0.024134925	0.892569556	-6.018732496	-1.409295596	-1.764147568	-4.471927184	-3.48972786	-7.0609024	7.871490707	0.005515328

PLCE1	0.017351453	-0.017568332	-0.046969033	-0.034919785	-0.064320487	-0.029400702	8	0.14571333	0.816496581	0.816496581	0.816496581	0.816496581	0.816496581	0.816496581	4	137	0	P_209215	P_209215	P_209215	chr10	94227363	94227423	+	PLCE1	"phospholipase C, epsilon 1 [Source:HGNC Symbol;Acc:HGNC:17175]"	ENSG00000138193	protein_coding	exon	coding exon	1	647	10:94227363:94227423	ENSG00000138193	PLCE1	3.962715785	0.019257439	0.153137738	-0.155051828	-0.414531932	-0.308189566	-0.567669669	-0.259480104	9.961963646	0.881347133	0.879877007	0.687269623	0.76428574	0.582825693	0.800548276	-6.98557836	-6.16942956	-6.954084908	-7.097918777	-6.128089466	-7.034720571	0.117314757	0.947877586

UTP20	-0.203295992	-0.051192681	-0.184546544	0.152103311	0.018749448	-0.133353863	8	0.040072273	0.816496581	0.816496581	0.816496581	0.816496581	0.816496581	0.816496581	4	138	0	P_216973	P_216973	P_216973	chr12	101293185	101293245	+	UTP20	"UTP20, small subunit (SSU) processome component, homolog (yeast) [Source:HGNC Symbol;Acc:HGNC:17897]"	ENSG00000120800	protein_coding	exon	coding exon	1	648	12:101293185:101293245	ENSG00000120800	UTP20	7.774507598	0.003496219	-4.21090224	-1.060362142	-3.822541937	3.150540098	0.388360302	-2.762179795	9.961963646	0.001812511	0.314011258	0.003382879	0.010370433	0.705919651	0.020121582	-1.473638004	-5.621779253	-2.1576121	-3.3933135	-6.21708534	-3.984212395	8.52904271	0.004188077

UBR4	-0.061003799	-0.02717101	-0.081756619	0.033832789	-0.02075282	-0.054585609	8	0.040771324	0.816496581	0.816496581	0.816496581	0.816496581	0.816496581	0.816496581	4	139	0	P_200486	P_200486	P_200486	chr1	19104160	19104220	-	UBR4	ubiquitin protein ligase E3 component n-recognin 4 [Source:HGNC Symbol;Acc:HGNC:30313]	ENSG00000127481	protein_coding	5'UTR;exon	multiple Ref	1	649	1:19104160:19104220	ENSG00000127481	UBR4	6.268164306	0.003541602	-1.255459262	-0.559179867	-1.682552661	0.696279394	-0.427093399	-1.123372793	9.961963646	0.23796089	0.588388412	0.123489344	0.502175499	0.678394878	0.287621632	-6.200377906	-6.019890227	-5.687758674	-6.890776438	-6.200571207	-6.422544015	1.108170623	0.391071401

AKIRIN2	-0.157174883	-0.081216736	-0.048998154	0.075958147	0.108176729	0.032218582	8	0.10793111	0.816496581	0.816496581	0.816496581	0.816496581	0.816496581	0.816496581	4	141	0	P_258181	P_258181	P_258181	chr6	87675859	87675919	-	AKIRIN2	akirin 2 [Source:HGNC Symbol;Acc:HGNC:21407]	ENSG00000135334	protein_coding	exon	coding exon	1	651	6:87675859:87675919	ENSG00000135334	AKIRIN2	7.406782798	0.011561566	-1.790277859	-0.925087532	-0.558106418	0.865190327	1.23217144	0.366981113	9.961963646	0.103795205	0.376785601	0.589093778	0.407286577	0.246175172	0.721308388	-5.483873457	-5.750041196	-6.879641165	-6.754992658	-5.546925497	-6.998301391	1.128973404	0.383614302

STARD5	-0.119279459	0.186171377	-0.070974514	0.305450837	0.048304945	-0.257145891	8	0.090469585	0.816496581	0.816496581	0.816496581	0.816496581	0.816496581	0.816496581	4	142	0	A_23_P158880	A_23_P158880	A_23_P158880	chr15	81312861	81312921	-	STARD5	StAR-related lipid transfer (START) domain containing 5 [Source:HGNC Symbol;Acc:HGNC:18065]	ENSG00000172345	protein_coding	3'UTR;exon	multiple Ref	1	652	15:81312861:81312921	ENSG00000172345	STARD5	6.71441743	0.008779481	-1.559110391	2.433459457	-0.927712978	3.992569847	0.631397413	-3.361172435	9.961963646	0.150145633	0.035327194	0.375486771	0.002568041	0.541996411	0.00726608	-5.812564323	-3.759754088	-6.596456965	-1.964898579	-6.0889122	-2.960389422	6.14165972	0.012337751

STIM1	-0.064642743	-0.102164922	0.050102461	-0.037522179	0.114745204	0.152267383	8	0.135906957	0.816496581	0.816496581	0.816496581	0.816496581	0.816496581	0.816496581	4	143	0	P_107703	P_107703	P_107703	chr11	4091923	4091983	+	STIM1	stromal interaction molecule 1 [Source:HGNC Symbol;Acc:HGNC:11386]	ENSG00000167323	protein_coding	3'UTR	3'UTR	1	653	11:4091923:4091983	ENSG00000167323	STIM1	7.008132249	0.017039664	-0.606505712	-0.958554756	0.470082598	-0.352049043	1.076588311	1.428637354	9.961963646	0.557746535	0.360468589	0.648417896	0.732134947	0.307044694	0.183703651	-6.801274733	-5.719639315	-6.92777121	-7.082255908	-5.714614374	-6.057762889	0.805273276	0.519196746

IDE	-0.045506554	0.068803867	-0.005639847	0.114310421	0.039866707	-0.074443713	8	0.068235951	0.816496581	0.816496581	0.816496581	0.816496581	0.816496581	0.816496581	4	144	0	P_209089	P_209089	P_209089	chr10	92463730	92463790	-	IDE	insulin-degrading enzyme [Source:HGNC Symbol;Acc:HGNC:5381]	ENSG00000119912	protein_coding	exon	coding exon	1	654	10:92463730:92463790	ENSG00000119912	IDE	9.009266218	0.005945822	-0.722792467	1.092829755	-0.089579157	1.815622222	0.63321331	-1.182408912	9.961963646	0.486429847	0.300190666	0.930397041	0.099599961	0.540857511	0.264496343	-6.720557745	-5.588926848	-7.042740159	-5.593763507	-6.087739469	-6.356998928	1.136710705	0.380880601

ORC2	-0.169721925	0.045662483	-0.0666722	0.215384408	0.103049725	-0.112334683	8	0.049919763	0.816496581	0.816496581	0.816496581	0.816496581	0.816496581	0.816496581	4	145	0	A_24_P921986	A_24_P921986	A_24_P921986	chr2	200954523	200954584	+	ORC2	"origin recognition complex, subunit 2 [Source:HGNC Symbol;Acc:HGNC:8488]"	ENSG00000115942	protein_coding	intron	intron antisense	0	655	2:200954523:200954584	ENSG00000115942	ORC2	7.959898479	0.004207882	-3.204437488	0.862131234	-1.258805527	4.066568721	1.945631961	-2.12093676	9.961963646	0.009465046	0.408889002	0.236799132	0.002280395	0.080438577	0.060032272	-3.161571135	-5.80476669	-6.244519278	-1.842101334	-4.600232552	-5.046430626	6.236186477	0.011765903

ACIN1	-0.163267938	-0.175501356	-0.099628227	-0.012233417	0.063639711	0.075873129	8	0.089234117	0.816496581	0.816496581	0.816496581	0.816496581	0.816496581	0.816496581	4	146	0	P_220197	P_220197	P_220197	chr14	23069515	23069575	-	ACIN1	apoptotic chromatin condensation inducer 1 [Source:HGNC Symbol;Acc:HGNC:17066]	ENSG00000100813	protein_coding	3'UTR;exon	multiple Ref	1	656	14:23069515:23069575	ENSG00000100813	ACIN1	8.017845783	0.008601189	-2.156092083	-2.317644771	-1.315675533	-0.161552688	0.84041655	1.001969238	9.961963646	0.056582398	0.043037516	0.21775596	0.874887599	0.420388121	0.340074572	-4.920128879	-3.940621907	-6.176053876	-7.135359022	-5.934155952	-6.549013557	2.247925677	0.145537678

MAP3K4	-0.149231124	-0.002663317	-0.122133286	0.146567808	0.027097838	-0.119469969	8	0.063926312	0.816496581	0.816496581	0.816496581	0.816496581	0.816496581	0.816496581	4	147	0	P_260184	P_260184	P_260184	chr6	161086607	161086667	+	MAP3K4	mitogen-activated protein kinase kinase kinase 4 [Source:HGNC Symbol;Acc:HGNC:6856]	ENSG00000085511	protein_coding	3'UTR;exon	multiple Ref	1	657	6:161086607:161086667	ENSG00000085511	MAP3K4	6.483817485	0.005488425	-2.467068257	-0.044029583	-2.019090551	2.423038674	0.447977706	-1.975060968	9.961963646	0.0333556	0.965750871	0.071196003	0.035961476	0.663754107	0.076607926	-4.412152956	-6.181065551	-5.183519298	-4.629608057	-6.191040314	-5.275403203	3.356292886	0.063667482

NISCH	0.058475973	0.021151389	-0.013020344	-0.037324585	-0.071496317	-0.034171732	8	0.104724981	0.816496581	0.816496581	0.816496581	0.816496581	0.816496581	0.816496581	4	148	0	P_135074	P_135074	P_135074	chr3	52451394	52451454	+	NISCH;TNNC1	nischarin [Source:HGNC Symbol;Acc:HGNC:18006];troponin C type 1 (slow) [Source:HGNC Symbol;Acc:HGNC:11943]	ENSG00000010322	protein_coding	exon;promoter	multiple non-Ref	0	658	3:52451394:52451454	ENSG00000010322	NISCH	6.98851951	0.011014041	0.682416843	0.246837514	-0.151947908	-0.435579329	-0.83436475	-0.398785421	9.961963646	0.510531129	0.810046178	0.882261226	0.672428486	0.423631698	0.69846528	-6.750043442	-6.150069427	-7.034526615	-7.046837997	-5.939180575	-6.985190992	0.265772229	0.84857329

GYPC	-0.204209291	0.009203236	0.130641661	0.213412527	0.334850952	0.121438425	8	0.410578366	0.816496581	0.816496581	0.816496581	0.816496581	0.816496581	0.816496581	4	149	0	A_24_P481530	A_24_P481530	A_24_P481530	chr2	126677835	126677895	+	GYPC;AC013474.4	glycophorin C (Gerbich blood group) [Source:HGNC Symbol;Acc:HGNC:4704];	ENSG00000136732	protein_coding;processed_pseudogene	intron;promoter	multiple non-Ref	0	659	2:126677835:126677895	ENSG00000136732	GYPC	4.340952925	0.137581275	-0.674281837	0.030388309	0.431367734	0.704670146	1.105649571	0.400979425	9.961963646	0.515473418	0.976357559	0.675386678	0.49715885	0.294864308	0.696900723	-6.755798016	-6.181600631	-6.946445799	-6.884651605	-5.684603575	-6.984247651	0.420243843	0.742536597

CRB2	-0.240701771	-0.093131804	0.015928208	0.147569967	0.256629979	0.109060012	8	0.245383342	0.816496581	0.816496581	0.816496581	0.816496581	0.816496581	0.816496581	5	20	0	P_149619	P_149619	P_149619	chr9	123375283	123375343	+	CRB2	crumbs family member 2 [Source:HGNC Symbol;Acc:HGNC:18688]	ENSG00000148204	protein_coding	exon	coding exon	1	700	9:123375283:123375343	ENSG00000148204	CRB2	4.175230112	0.050560994	-1.311043541	-0.507266104	0.086757045	0.803777437	1.397800586	0.594023149	9.961963646	0.219258071	0.623007967	0.932583678	0.440283519	0.192518218	0.565740936	-6.133667638	-6.048230547	-7.043011712	-6.807355843	-5.3507334	-6.881967063	0.8227415	0.510729829

NOD1	-0.086295021	0.100911785	0.026260605	0.187206805	0.112555626	-0.074651179	8	0.066026478	0.816496581	0.816496581	0.816496581	0.816496581	0.816496581	0.816496581	5	21	0	P_260854	P_260854	P_260854	chr7	30450421	30450481	+	NOD1	nucleotide-binding oligomerization domain containing 1 [Source:HGNC Symbol;Acc:HGNC:16390]	ENSG00000106100	protein_coding	intron;promoter	multiple non-Ref	0	701	7:30450421:30450481	ENSG00000106100	NOD1	5.167062147	0.005707597	-1.398958158	1.635915524	0.425719669	3.034873683	1.824677828	-1.210195855	9.961963646	0.192180945	0.133017769	0.679362948	0.01262456	0.098139216	0.25413578	-6.024069185	-4.939157228	-6.949041245	-3.591755209	-4.776884028	-6.325273912	3.136503703	0.074292654

NEBL	0.036786317	0.134643416	0.146524994	0.097857099	0.109738677	0.011881578	8	0.077251829	0.816496581	0.816496581	0.816496581	0.816496581	0.816496581	0.816496581	5	22	0	P_207645	P_207645	P_207645	chr10	20809805	20809865	-	NEBL	nebulette [Source:HGNC Symbol;Acc:HGNC:16932]	ENSG00000078114	protein_coding	exon	coding exon	1	702	10:20809805:20809865	ENSG00000078114	NEBL	5.139408822	0.006999189	0.538527736	1.971091972	2.145030543	1.432564236	1.606502807	0.173938571	9.961963646	0.602037577	0.077114365	0.05764687	0.182606305	0.13936017	0.865397532	-6.842381267	-4.465877228	-4.984636481	-6.129758883	-5.081048942	-7.054875542	2.239952984	0.146473659

CYP7B1	0.070412377	0.234570413	0.157366778	0.164158036	0.086954401	-0.077203635	8	0.174260879	0.816496581	0.816496581	0.816496581	0.816496581	0.816496581	0.816496581	5	23	0	P_147300	P_147300	P_147300	chr8	64596069	64596129	-	CYP7B1	"cytochrome P450, family 7, subfamily B, polypeptide 1 [Source:HGNC Symbol;Acc:HGNC:2652]"	ENSG00000172817	protein_coding	3'ext;intron	multiple non-Ref	0	703	8:64596069:64596129	ENSG00000172817	CYP7B1	3.445119105	0.026592923	0.528824531	1.761715681	1.181886145	1.23289115	0.653061614	-0.579829536	9.961963646	0.608507128	0.108716902	0.264694444	0.245917909	0.528499686	0.574907972	-6.847874476	-4.766577348	-6.333602241	-6.376336944	-6.07471965	-6.890762723	1.177144123	0.366938859

TRMT2B	-0.154476194	-0.016922594	-0.121740853	0.1375536	0.032735342	-0.104818258	8	0.060790393	0.816496581	0.816496581	0.816496581	0.816496581	0.816496581	0.816496581	5	24	0	A_32_P55045	A_32_P55045	A_32_P55045	chrX	101007203	101007263	-	TRMT2B	tRNA methyltransferase 2 homolog B (S. cerevisiae) [Source:HGNC Symbol;Acc:HGNC:25748]	ENSG00000188917	protein_coding	3'ext	3'extended sense	0	704	X:101007203:101007263	ENSG00000188917	TRMT2B	7.687796443	0.005174349	-2.630142721	-0.288127494	-2.072784217	2.342015227	0.557358504	-1.784656723	9.961963646	0.025230539	0.779154696	0.065085196	0.041289214	0.589585501	0.104747379	-4.1390931	-6.138511908	-5.099299124	-4.76363625	-6.134063263	-5.5632307	3.379631006	0.062648742

EDC3	0.056524168	0.032071169	0.03600991	-0.024452999	-0.020514258	0.00393874	8	0.079209026	0.816496581	0.816496581	0.816496581	0.816496581	0.816496581	0.816496581	5	26	0	P_310096	P_310096	P_310096	chr15	74634359	74634419	+	EDC3;CLK3	enhancer of mRNA decapping 3 [Source:HGNC Symbol;Acc:HGNC:26114];CDC-like kinase 3 [Source:HGNC Symbol;Acc:HGNC:2071]	ENSG00000179151	protein_coding	3'ext;intron	multiple non-Ref	0	706	15:74634359:74634419	ENSG00000179151	EDC3	6.414380178	0.007245104	0.813312583	0.4614643	0.518137882	-0.351848283	-0.295174701	0.056673582	9.961963646	0.435046441	0.654377238	0.615673595	0.732280936	0.773920656	0.955925818	-6.648969709	-6.07105908	-6.902463248	-7.082332234	-6.250551167	-7.06962414	0.226171789	0.876055265

SYNE1	0.102680867	0.124275245	0.100205637	0.021594378	-0.00247523	-0.024069608	8	0.053314545	0.816496581	0.816496581	0.816496581	0.816496581	0.816496581	0.816496581	5	28	0	P_143869	P_143869	P_143869	chr6	152180135	152180195	-	SYNE1	"spectrin repeat containing, nuclear envelope 1 [Source:HGNC Symbol;Acc:HGNC:17089]"	ENSG00000131018	protein_coding	exon	coding exon	1	708	6:152180135:152180195	ENSG00000131018	SYNE1	3.766938312	0.004489319	1.876916401	2.271642731	1.831671361	0.39472633	-0.045245039	-0.43997137	9.961963646	0.090091904	0.046536162	0.097024642	0.701363747	0.964806109	0.669349788	-5.354613517	-4.011855866	-5.469851741	-7.065069852	-6.29559654	-6.966649423	2.064937774	0.168908495

ZNF711	0.027831471	-0.025076691	0.050897156	-0.052908161	0.023065685	0.075973846	8	0.193940308	0.816496581	0.816496581	0.816496581	0.816496581	0.816496581	0.816496581	5	29	0	P_270814	P_270814	P_270814	chrX	85255738	85255798	+	ZNF711	zinc finger protein 711 [Source:HGNC Symbol;Acc:HGNC:13128]	ENSG00000147180	protein_coding	exon	coding exon	1	709	X:85255738:85255798	ENSG00000147180	ZNF711	3.772416943	0.032411848	0.189334558	-0.170594079	0.34624798	-0.359928638	0.156913422	0.516842059	9.961963646	0.853632526	0.867957939	0.736357884	0.726413944	0.87844766	0.616545485	-6.978833974	-6.166768797	-6.982042451	-7.079226711	-6.283605934	-6.927382658	0.10102229	0.957584495

SRGAP3-AS4	0.346931346	0.356141634	0.192065734	0.009210288	-0.154865612	-0.164075899	8	0.189843796	0.816496581	0.816496581	0.816496581	0.816496581	0.816496581	0.816496581	5	31	0	A_32_P210982	A_32_P210982	A_32_P210982	chr3	9256833	9256893	+	SRGAP3-AS4	SRGAP3 antisense RNA 4 [Source:HGNC Symbol;Acc:HGNC:42434]	ENSG00000235830	lincRNA	exon	non-coding exon	1	711	3:9256833:9256893	ENSG00000235830	SRGAP3-AS4	5.847333274	0.031149305	2.40749278	2.471406581	1.332819516	0.063913801	-1.074673264	-1.138587065	9.961963646	0.036928554	0.033109134	0.21227056	0.950303162	0.3078607	0.281515539	-4.510942539	-3.700080065	-6.154996563	-7.147379589	-5.716570056	-6.405897964	2.68927799	0.103162029

LDHAL6A	-0.058219949	-0.036655359	-0.026098265	0.02156459	0.032121684	0.010557094	8	0.199042433	0.816496581	0.816496581	0.816496581	0.816496581	0.816496581	0.816496581	5	32	0	P_211202	P_211202	P_211202	chr11	18475553	18475613	+	LDHAL6A	lactate dehydrogenase A-like 6A [Source:HGNC Symbol;Acc:HGNC:28335]	ENSG00000166800	protein_coding	exon	coding exon	1	712	11:18475553:18475613	ENSG00000166800	LDHAL6A	4.46087663	0.03402201	-0.386578295	-0.243390223	-0.173291511	0.143188072	0.213286784	0.070098712	9.961963646	0.707197157	0.812641697	0.865892769	0.888996514	0.835404879	0.945502298	-6.917421135	-6.150954761	-7.030745792	-7.138412347	-6.272545978	-7.068694749	0.051603239	0.983623231

GOLGA4	-0.127399117	-0.093104527	-0.135518867	0.034294591	-0.00811975	-0.042414341	8	0.051757929	0.816496581	0.816496581	0.816496581	0.816496581	0.816496581	0.816496581	5	33	0	P_134376	P_134376	P_134376	chr3	37288043	37288103	+	GOLGA4	golgin A4 [Source:HGNC Symbol;Acc:HGNC:4427]	ENSG00000144674	protein_coding	intron	intron sense	0	713	3:37288043:37288103	ENSG00000144674	GOLGA4	8.597712411	0.004357973	-2.363577062	-1.72732534	-2.51421904	0.636251722	-0.150641978	-0.7868937	9.961963646	0.039800342	0.114925116	0.030771282	0.53895497	0.883264723	0.449658812	-4.583370674	-4.814417851	-4.379701183	-6.932638829	-6.284629334	-6.743274437	2.656359616	0.10576888

NBEA	0.467664518	-0.144543352	0.265451011	-0.61220787	-0.202213508	0.409994363	8	0.053216618	0.816496581	0.816496581	0.816496581	0.816496581	0.816496581	0.816496581	5	34	0	P_218712	P_218712	P_218712	chr13	35044987	35045047	+	NBEA	neurobeachin [Source:HGNC Symbol;Acc:HGNC:7648]	ENSG00000172915	protein_coding	exon	coding exon	1	714	13:35044987:35045047	ENSG00000172915	NBEA	5.536212264	0.004480941	8.556485758	-2.644594751	4.856746033	-11.20108051	-3.699739725	7.501340783	9.961963646	6.67E-06	0.024613382	0.000671967	5.76E-07	0.004136895	2.10E-05	4.313409076	-3.425765426	-0.487197513	6.751863346	-1.802872755	3.097248658	49.86961735	2.63E-06

HEY1	0.065495547	0.082046012	0.161673592	0.016550465	0.096178045	0.07962758	8	0.089427674	0.816496581	0.816496581	0.816496581	0.816496581	0.816496581	0.816496581	5	35	0	P_147461	P_147461	P_147461	chr8	79764840	79764900	-	HEY1	hes-related family bHLH transcription factor with YRPW motif 1 [Source:HGNC Symbol;Acc:HGNC:4880]	ENSG00000164683	protein_coding	3'UTR;exon	multiple Ref	1	715	8:79764840:79764900	ENSG00000164683	HEY1	5.503149853	0.008628959	0.863531531	1.08174252	2.131599017	0.21821099	1.268067486	1.049856497	9.961963646	0.408154956	0.304856697	0.058965227	0.83166981	0.233607706	0.318585478	-6.606073245	-5.600232482	-5.006064392	-7.123635856	-5.505877769	-6.500503457	1.536229634	0.265327306

SPTAN1	0.364892453	-0.150829969	0.198106369	-0.515722423	-0.166786085	0.348936338	8	0.069209824	0.816496581	0.816496581	0.816496581	0.816496581	0.816496581	0.816496581	5	36	0	P_269106	P_269106	P_269106	chr9	128612176	128612236	+	SPTAN1	"spectrin, alpha, non-erythrocytic 1 [Source:HGNC Symbol;Acc:HGNC:11273]"	ENSG00000197694	protein_coding	exon	coding exon	1	716	9:128612176:128612236	ENSG00000197694	SPTAN1	7.801440951	0.006053315	5.743992656	-2.374305713	3.11851209	-8.118298368	-2.625480565	5.492817803	9.961963646	0.000189431	0.039079201	0.010949988	1.06E-05	0.025432904	0.000268043	0.86503072	-3.852388754	-3.353081243	3.763210659	-3.539091437	0.449908123	25.22114348	5.70E-05

EEA1	-0.141294799	0.053761583	-0.025215322	0.195056382	0.116079477	-0.078976905	8	0.061134938	0.816496581	0.816496581	0.816496581	0.816496581	0.816496581	0.816496581	5	37	0	P_216648	P_216648	P_216648	chr12	92819327	92819387	-	EEA1	early endosome antigen 1 [Source:HGNC Symbol;Acc:HGNC:3185]	ENSG00000102189	protein_coding	3'UTR;5'UTR;exon	multiple Ref	1	717	12:92819327:92819387	ENSG00000102189	EEA1	8.7915362	0.005208085	-2.397909482	0.912386089	-0.427928414	3.310295571	1.969981068	-1.340314503	9.961963646	0.037537381	0.38311431	0.677806741	0.007915455	0.077256678	0.209908925	-4.526777579	-5.76134348	-6.948030188	-3.119403075	-4.564075	-6.169642431	3.900144356	0.044254734

ZNF436	0.026678701	-0.006072865	-0.129962569	-0.032751566	-0.15664127	-0.123889704	8	0.106483613	0.816496581	0.816496581	0.816496581	0.816496581	0.816496581	0.816496581	5	38	0	P_100763	P_100763	P_100763	chr1	23359775	23359835	+	ZNF436	zinc finger protein 436 [Source:HGNC Symbol;Acc:HGNC:20814]	ENSG00000125945	protein_coding	3'ext;3'UTR	multiple non-Ref	0	718	1:23359775:23359835	ENSG00000125945	ZNF436	5.313304381	0.011312326	0.307209289	-0.069929964	-1.496538713	-0.377139253	-1.803748002	-1.426608749	9.961963646	0.765009996	0.945633254	0.16550676	0.713979759	0.101545702	0.18427274	-6.947103631	-6.179510108	-5.944751779	-7.072383414	-4.806915225	-6.060385952	1.294974877	0.329431162

NRXN3	-0.132842952	0.046287225	-0.090915256	0.179130177	0.041927696	-0.137202481	8	0.128754663	0.816496581	0.816496581	0.816496581	0.816496581	0.816496581	0.816496581	5	39	0	P_115492	P_115492	P_115492	chr14	79692202	79692262	+	NRXN3	neurexin 3 [Source:HGNC Symbol;Acc:HGNC:8010]	ENSG00000021645	protein_coding	exon	coding exon	1	719	14:79692202:79692262	ENSG00000021645	NRXN3	4.460914148	0.015519532	-1.306005005	0.455058751	-0.893805638	1.761063756	0.412199367	-1.348864389	9.961963646	0.22090177	0.658823114	0.392507288	0.108831689	0.688923146	0.207241769	-6.139799208	-6.074087447	-6.627573725	-5.674384955	-6.207100662	-6.15902475	1.300384178	0.327814929

BMP6	-0.072029694	0.25583502	-0.204948735	0.327864714	-0.132919041	-0.460783755	8	0.134666799	0.816496581	0.816496581	0.816496581	0.816496581	0.816496581	0.816496581	5	40	0	P_141588	P_141588	P_141588	chr6	7880349	7880409	+	BMP6	bone morphogenetic protein 6 [Source:HGNC Symbol;Acc:HGNC:1073]	ENSG00000153162	protein_coding	3'UTR	3'UTR	1	720	6:7880349:7880409	ENSG00000153162	BMP6	5.778795543	0.016770196	-0.681221174	2.419560901	-1.938303629	3.100782075	-1.257082455	-4.35786453	9.961963646	0.511255739	0.036175637	0.081420031	0.011284951	0.237396769	0.001438767	-6.750893179	-3.781563419	-5.30848111	-3.47868753	-5.51852909	-1.297064465	6.710241207	0.009334016

ELAPOR1	0.300397106	-0.107111382	0.188288416	-0.407508488	-0.11210869	0.295399798	8	0.067516436	0.816496581	0.816496581	0.816496581	0.816496581	0.816496581	0.816496581	5	41	0	P_102596	P_102596	P_102596	chr1	109200825	109200885	-	KIAA1324	KIAA1324 [Source:HGNC Symbol;Acc:HGNC:29618]	ENSG00000116299	protein_coding	3'UTR;exon	multiple non-Ref	0	721	1:109200825:109200885	ENSG00000116299	ELAPOR1	5.768703019	0.005867383	4.803072532	-1.712612162	3.010558028	-6.515684695	-1.792514505	4.72317019	9.961963646	0.000728084	0.117678242	0.01315878	6.88E-05	0.103418549	0.00082105	-0.531193324	-4.834737632	-3.53777555	1.811880616	-4.822964229	-0.715223045	17.17359962	0.00029057

PDE11A	0.057893993	-0.106984338	0.014025942	-0.164878331	-0.043868051	0.12101028	8	0.122790968	0.816496581	0.816496581	0.816496581	0.816496581	0.816496581	0.816496581	5	43	0	P_237711	P_237711	P_237711	chr2	177820219	177820279	-	PDE11A	phosphodiesterase 11A [Source:HGNC Symbol;Acc:HGNC:8773]	ENSG00000128655	protein_coding	exon	coding exon	1	723	2:177820219:177820279	ENSG00000128655	PDE11A	4.43156932	0.014314837	0.592633144	-1.095147556	0.143576867	-1.6877807	-0.449056276	1.238724424	9.961963646	0.566635086	0.299222212	0.888697375	0.122460413	0.663001933	0.243840812	-6.81003361	-5.586552076	-7.035874631	-5.780628602	-6.19053623	-6.292135405	1.025978326	0.422116403

PRELID2	-0.014706652	0.280964667	0.104470342	0.295671319	0.119176994	-0.176494325	8	0.185863426	0.816496581	0.816496581	0.816496581	0.816496581	0.816496581	0.816496581	5	44	0	A_16_P01357890	A_16_P01357890	A_16_P01357890	chr5	145838185	145838245	+	PRELID2	PRELI domain containing 2 [Source:HGNC Symbol;Acc:HGNC:28306]	ENSG00000186314	protein_coding	promoter	promoter antisense	0	724	5:145838185:145838245	ENSG00000186314	PRELID2	3.430042155	0.029948374	-0.104081328	1.98843187	0.739353311	2.092513198	0.843434639	-1.249078559	9.961963646	0.919170424	0.074924748	0.476752869	0.062967731	0.418776836	0.240189031	-6.992452114	-4.440323146	-6.756554436	-5.167325507	-5.931638415	-6.279968381	1.86025471	0.20042058

WDR24	-0.180638517	0.024080674	-0.131808118	0.204719191	0.048830398	-0.155888793	8	0.146654744	0.816496581	0.816496581	0.816496581	0.816496581	0.816496581	0.816496581	5	45	0	P_117779	P_117779	P_117779	chr16	689159	689219	-	WDR24	WD repeat domain 24 [Source:HGNC Symbol;Acc:HGNC:20852]	ENSG00000127580	protein_coding	exon	coding exon	1	725	16:689159:689219	ENSG00000127580	WDR24	7.827834986	0.019478471	-1.585179913	0.211318173	-1.156672371	1.796498087	0.428507542	-1.367990545	9.961963646	0.144124841	0.836899302	0.27438998	0.102750808	0.67739897	0.201378117	-5.776798613	-6.158601184	-6.361887878	-5.62216578	-6.199939651	-6.135105247	1.529627321	0.266881107

JPT2	-0.285248429	0.453850338	-0.172970819	0.739098768	0.11227761	-0.626821158	8	0.345642125	0.816496581	0.816496581	0.816496581	0.816496581	0.816496581	0.816496581	5	46	0	P_225260	P_225260	P_225260	chr16	1699381	1699441	-	HN1L	hematological and neurological expressed 1-like [Source:HGNC Symbol;Acc:HGNC:14137]	ENSG00000206053	protein_coding	3'ext;3'UTR;intron	multiple non-Ref	0	726	16:1699381:1699441	ENSG00000206053	JPT2	9.221321834	0.098146386	-1.115146009	1.774275829	-0.676209574	2.889421838	0.438936435	-2.450485403	9.961963646	0.29096614	0.106526878	0.514299661	0.016183078	0.670074672	0.034314471	-6.359214061	-4.748987097	-6.803009269	-3.840954684	-6.195220104	-4.509074053	3.232527865	0.069410423

DDX24	-0.058174133	0.005051275	0.061258454	0.063225408	0.119432587	0.056207179	8	0.092579734	0.816496581	0.816496581	0.816496581	0.816496581	0.816496581	0.816496581	5	48	0	A_24_P238878	A_24_P238878	A_24_P238878	chr14	94079373	94079433	-	DDX24	DEAD (Asp-Glu-Ala-Asp) box helicase 24 [Source:HGNC Symbol;Acc:HGNC:13266]	ENSG00000089737	protein_coding	exon	coding exon	1	728	14:94079373:94079433	ENSG00000089737	DDX24	9.203595347	0.00908967	-0.747311193	0.064889226	0.786932726	0.812200419	1.53424392	0.722043501	9.961963646	0.472146479	0.949545872	0.449636991	0.435655137	0.156093866	0.486870378	-6.701908673	-6.179868198	-6.719104558	-6.800372832	-5.177008611	-6.793818281	0.786144748	0.528630957

CHTF18	0.081351199	-0.000864165	-0.085292168	-0.082215364	-0.166643367	-0.084428003	8	0.201154974	0.816496581	0.816496581	0.816496581	0.816496581	0.816496581	0.816496581	5	49	0	A_32_P44274	A_32_P44274	A_32_P44274	chr16	797944	798004	+	CHTF18	"CTF18, chromosome transmission fidelity factor 18 homolog (S. cerevisiae) [Source:HGNC Symbol;Acc:HGNC:18435]"	ENSG00000127586	protein_coding	"3'UTR,exon;exon"	multiple Ref	1	729	16:797944:798004	ENSG00000127586	CHTF18	7.444418428	0.034700939	0.534858587	-0.005681616	-0.560769223	-0.540540203	-1.095627811	-0.555087607	9.961963646	0.604479743	0.995578917	0.587344872	0.600700277	0.299021847	0.591079862	-6.84446954	-6.182070407	-6.87806404	-6.992139869	-5.695023015	-6.90561901	0.400212396	0.755952015

C11orf1	0.148531463	0.087880558	0.105947721	-0.060650905	-0.042583742	0.018067163	8	0.10671346	0.816496581	0.816496581	0.816496581	0.816496581	0.816496581	0.816496581	5	51	0	A_24_P406939	A_24_P406939	A_24_P406939	chr11	111884803	111884863	+	C11orf1	chromosome 11 open reading frame 1 [Source:HGNC Symbol;Acc:HGNC:1163]	ENSG00000137720	protein_coding	3'UTR	3'UTR	1	731	11:111884803:111884863	ENSG00000137720	C11orf1	9.247531552	0.011351678	1.70739518	1.010202407	1.217887608	-0.697192773	-0.489507573	0.207685201	9.961963646	0.118668706	0.336305118	0.251326187	0.50162791	0.635081619	0.83965897	-5.60459451	-5.670988179	-6.292423717	-6.89011294	-6.170794631	-7.047865965	1.031297667	0.420028521

EPN2	-0.059303865	-0.119857363	-0.089782476	-0.060553498	-0.030478611	0.030074886	8	0.08806206	0.816496581	0.816496581	0.816496581	0.816496581	0.816496581	0.816496581	5	53	0	P_310359	P_310359	P_310359	chr17	19328721	19328781	+	EPN2	epsin 2 [Source:HGNC Symbol;Acc:HGNC:18639]	ENSG00000072134	protein_coding	exon	coding exon	1	733	17:19328721:19328781	ENSG00000072134	EPN2	7.820367984	0.008434313	-0.790867592	-1.598400098	-1.197325854	-0.807532507	-0.406458262	0.401074245	9.961963646	0.447440452	0.141154025	0.258893266	0.438216111	0.693000252	0.696833141	-6.667415947	-4.989270568	-6.316055127	-6.80425061	-6.209557802	-6.984206769	0.932020207	0.460885144

PDHX	-0.217354003	0.208899976	-0.015025724	0.426253979	0.202328279	-0.2239257	8	0.057033113	0.816496581	0.816496581	0.816496581	0.816496581	0.816496581	0.816496581	5	54	0	P_211532	P_211532	P_211532	chr11	34947546	34947606	+	PDHX	"pyruvate dehydrogenase complex, component X [Source:HGNC Symbol;Acc:HGNC:21350]"	ENSG00000110435	protein_coding	exon	coding exon	1	734	11:34947546:34947606	ENSG00000110435	PDHX	10.28627824	0.00481884	-3.834793647	3.68563858	-0.265100026	7.520432227	3.569693621	-3.950738606	9.961963646	0.00331597	0.004234067	0.796337064	2.06E-05	0.00512895	0.002747272	-2.09442262	-1.771568742	-7.008876798	3.072326552	-2.009890115	-1.965033984	18.87796684	0.000196351

CDYL2	-0.32010437	0.027141936	-0.363866864	0.347246307	-0.043762494	-0.391008801	8	0.155237877	0.816496581	0.816496581	0.816496581	0.816496581	0.816496581	0.816496581	5	55	0	A_32_P217261	A_32_P217261	A_32_P217261	chr16	80597960	80598020	-	CDYL2	"chromodomain protein, Y-like 2 [Source:HGNC Symbol;Acc:HGNC:23030]"	ENSG00000166446	protein_coding	3'UTR	3'UTR	1	735	16:80597960:80598020	ENSG00000166446	CDYL2	6.468951019	0.021559333	-2.670051093	0.226396023	-3.035082331	2.896447116	-0.365031238	-3.261478354	9.961963646	0.023562711	0.825471128	0.012620073	0.015989823	0.722718554	0.008594795	-4.071782627	-6.155139327	-3.495828767	-3.828935571	-6.226297956	-3.13076068	5.925341252	0.013776475

GAS5-AS1	0.070153626	0.201342087	0.230856823	0.131188461	0.160703197	0.029514736	8	0.268759075	0.816496581	0.816496581	0.816496581	0.816496581	0.816496581	0.816496581	5	56	0	A_32_P307211	A_32_P307211	A_32_P307211	chr1	173863834	173863894	+	GAS5-AS1	GAS5 antisense RNA 1 [Source:HGNC Symbol;Acc:HGNC:44119]	ENSG00000270084	antisense	exon	non-coding exon	1	736	1:173863834:173863894	ENSG00000270084	GAS5-AS1	5.006639437	0.060212469	0.350148713	1.004932697	1.152245779	0.654783984	0.802097066	0.147313082	9.961963646	0.733517285	0.338714196	0.276120769	0.527435196	0.441210764	0.885823674	-6.931867896	-5.676047009	-6.366805965	-6.920083194	-5.96543875	-7.0595361	0.592327849	0.634086787

NUCB1	0.050543648	0.046701253	-0.069787056	-0.003842395	-0.120330704	-0.116488309	8	0.05560269	0.816496581	0.816496581	0.816496581	0.816496581	0.816496581	0.816496581	5	57	0	P_126325	P_126325	P_126325	chr19	48922879	48922939	+	NUCB1	nucleobindin 1 [Source:HGNC Symbol;Acc:HGNC:8043]	ENSG00000104805	protein_coding	3'UTR;exon	multiple Ref	1	737	19:48922879:48922939	ENSG00000104805	NUCB1	7.380235031	0.004689455	0.903963786	0.835243259	-1.248128577	-0.068720527	-2.152092363	-2.083371836	9.961963646	0.387352174	0.423159796	0.240522214	0.946571884	0.056965104	0.063940562	-6.569935497	-5.827135968	-6.257130555	-7.147031096	-4.288175726	-5.106010699	2.008211505	0.177024498

MRGPRD	-0.058851394	0.196559221	0.127729708	0.255410615	0.186581102	-0.068829513	8	0.329626066	0.816496581	0.816496581	0.816496581	0.816496581	0.816496581	0.816496581	5	58	0	A_23_P127352	A_23_P127352	A_23_P127352	chr11	68980918	68980978	-	MRGPRD	"MAS-related GPR, member D [Source:HGNC Symbol;Acc:HGNC:29626]"	ENSG00000172938	protein_coding	exon	coding exon	1	738	11:68980918:68980978	ENSG00000172938	MRGPRD	3.854344134	0.089461243	-0.240982174	0.804862303	0.523022153	1.045844478	0.764004328	-0.28184015	9.961963646	0.81445617	0.439685567	0.612392876	0.320345588	0.462576284	0.783834185	-6.966763417	-5.85167296	-6.899760204	-6.582256653	-5.995267679	-7.028156837	0.456059177	0.718918381

PPM1F	-0.452666203	0.046464999	-0.327295782	0.499131202	0.125370421	-0.373760781	8	0.075043606	0.816496581	0.816496581	0.816496581	0.816496581	0.816496581	0.816496581	5	59	0	A_24_P125894	A_24_P125894	A_24_P125894	chr22	21920801	21920861	-	PPM1F	"protein phosphatase, Mg2+/Mn2+ dependent, 1F [Source:HGNC Symbol;Acc:HGNC:19388]"	ENSG00000100034	protein_coding	3'UTR	3'UTR	1	739	22:21920801:21920861	ENSG00000100034	PPM1F	7.383112544	0.00672912	-6.758410475	0.69373312	-4.886601263	7.452143595	1.871809212	-5.580334383	9.961963646	5.08E-05	0.503703968	0.000642761	2.23E-05	0.090850815	0.00023727	2.227717159	-5.934563817	-0.441046717	2.990711851	-4.708660457	0.576996671	26.7024159	4.44E-05

TCEA3	-0.079024582	0.196545779	-0.009975177	0.275570361	0.069049405	-0.206520956	8	0.11910073	0.816496581	0.816496581	0.816496581	0.816496581	0.816496581	0.816496581	5	60	0	P_308344	P_308344	P_308344	chr1	23383967	23384027	-	TCEA3	"transcription elongation factor A (SII), 3 [Source:HGNC Symbol;Acc:HGNC:11615]"	ENSG00000204219	protein_coding	3'UTR	3'UTR	1	740	1:23383967:23384027	ENSG00000204219	TCEA3	7.163466442	0.013598	-0.829985161	2.064295375	-0.104768017	2.894280536	0.725217144	-2.169063392	9.961963646	0.425989557	0.066016983	0.918639276	0.016049172	0.4850054	0.055358172	-6.63497556	-4.327502869	-7.041129637	-3.832642477	-6.024316316	-4.969527119	3.094800471	0.076539152

NLGN3	-0.094140473	0.047724338	-0.233978531	0.14186481	-0.139838058	-0.281702868	8	0.069094071	0.816496581	0.816496581	0.816496581	0.816496581	0.816496581	0.816496581	5	61	0	P_151155	P_151155	P_151155	chrX	71170236	71170296	+	NLGN3	neuroligin 3 [Source:HGNC Symbol;Acc:HGNC:14289]	ENSG00000196338	protein_coding	3'UTR;exon	multiple Ref	1	741	X:71170236:71170296	ENSG00000196338	NLGN3	4.76390411	0.006040459	-1.483498259	0.752056682	-3.687114929	2.23555494	-2.203616671	-4.439171611	9.961963646	0.168875281	0.46941311	0.004223783	0.049472512	0.052220073	0.00126783	-5.914235838	-5.892422281	-2.3851685	-4.937724392	-4.208572134	-1.166049193	7.653508292	0.006063031

CYP2R1	-0.230981893	-0.029290141	-0.25058903	0.201691751	-0.019607138	-0.221298889	8	0.042659275	0.816496581	0.816496581	0.816496581	0.816496581	0.816496581	0.816496581	5	62	0	P_211084	P_211084	P_211084	chr11	14885940	14886000	-	CYP2R1	"cytochrome P450, family 2, subfamily R, polypeptide 1 [Source:HGNC Symbol;Acc:HGNC:20580]"	ENSG00000186104	protein_coding	5'UTR	5'UTR	1	742	11:14885940:14886000	ENSG00000186104	CYP2R1	8.293330512	0.003668094	-4.670929987	-0.592307033	-5.067426725	4.078622954	-0.396496738	-4.475119692	9.961963646	0.000888597	0.566844976	0.000492443	0.002236841	0.700098934	0.001199231	-0.737435772	-6.000456153	-0.164222201	-1.822153849	-6.213742178	-1.108393982	14.11104988	0.000641689

GPR22	-0.159793117	0.070142002	-0.016097025	0.22993512	0.143696093	-0.086239027	8	0.404786567	0.816496581	0.816496581	0.816496581	0.816496581	0.816496581	0.816496581	5	63	0	A_23_P20172	A_23_P20172	A_23_P20172	chr7	107475297	107475357	+	GPR22	G protein-coupled receptor 22 [Source:HGNC Symbol;Acc:HGNC:4477]	ENSG00000172209	protein_coding	exon	coding exon	1	743	7:107475297:107475357	ENSG00000172209	GPR22	3.642638555	0.133788907	-0.53504911	0.23486253	-0.05389906	0.769911641	0.48115005	-0.28876159	9.961963646	0.604352805	0.819072503	0.958081165	0.459219765	0.640803107	0.778683266	-6.844361437	-6.153091986	-7.045534326	-6.834784354	-6.175006467	-7.026016886	0.208665938	0.888092459

GRIA2	0.140310814	0.048617107	0.284221525	-0.091693708	0.143910711	0.235604418	8	0.083141437	0.816496581	0.816496581	0.816496581	0.816496581	0.816496581	0.816496581	5	64	0	P_251399	P_251399	P_251399	chr4	157332847	157332907	+	GRIA2	"glutamate receptor, ionotropic, AMPA 2 [Source:HGNC Symbol;Acc:HGNC:4572]"	ENSG00000120251	protein_coding	exon	coding exon	1	744	4:157332847:157332907	ENSG00000120251	GRIA2	3.578636853	0.007757797	1.951046241	0.676029311	3.952149673	-1.27501693	2.001103432	3.276120362	9.961963646	0.079720596	0.514409351	0.002741017	0.231236314	0.073361811	0.008384962	-5.241744301	-5.946748776	-1.941413748	-6.326487025	-4.517590325	-3.105719122	6.041021973	0.012983522

ESRP2	-0.202289154	-0.147718972	-0.300860142	0.054570182	-0.098570988	-0.15314117	8	0.141108068	0.816496581	0.816496581	0.816496581	0.816496581	0.816496581	0.816496581	5	65	0	A_23_P100220	A_23_P100220	A_23_P100220	chr16	68229150	68229210	-	ESRP2	epithelial splicing regulatory protein 2 [Source:HGNC Symbol;Acc:HGNC:26152]	ENSG00000103067	protein_coding	3'UTR;exon	multiple Ref	1	745	16:68229150:68229210	ENSG00000103067	ESRP2	8.519866271	0.018196694	-1.836631316	-1.341175661	-2.731580751	0.495455655	-0.894949436	-1.390405091	9.961963646	0.096241256	0.209638991	0.021204668	0.631024882	0.391924434	0.194684862	-5.415088414	-5.313546231	-4.013366281	-7.0170074	-5.887478284	-6.106782224	2.602189543	0.110229808

NDEL1	-0.090124271	0.033712174	-0.120043874	0.123836444	-0.029919604	-0.153756048	8	0.064469774	0.816496581	0.816496581	0.816496581	0.816496581	0.816496581	0.816496581	5	66	0	P_120531	P_120531	P_120531	chr17	8466864	8466924	+	NDEL1	nudE neurodevelopment protein 1-like 1 [Source:HGNC Symbol;Acc:HGNC:17620]	ENSG00000166579	protein_coding	exon	non-coding exon	1	746	17:8466864:8466924	ENSG00000166579	NDEL1	7.51904195	0.005544461	-1.482373772	0.554501487	-1.974494657	2.036875259	-0.492120884	-2.528996143	9.961963646	0.169168516	0.591465885	0.076679995	0.069114109	0.633297705	0.030002852	-5.915723981	-6.022550533	-5.252772291	-5.255085644	-6.169463469	-4.378003745	2.865066388	0.090459825

SPOCK2	0.002416441	-0.046318126	-0.057710841	-0.048734567	-0.060127282	-0.011392715	8	0.120838881	0.816496581	0.816496581	0.816496581	0.816496581	0.816496581	0.816496581	5	67	0	P_208606	P_208606	P_208606	chr10	72059382	72059442	+	SPOCK2	"sparc/osteonectin, cwcv and kazal-like domains proteoglycan (testican) 2 [Source:HGNC Symbol;Acc:HGNC:13564]"	ENSG00000107742	protein_coding	3'ext;3'UTR	multiple non-Ref	0	747	10:72059382:72059442	ENSG00000107742	SPOCK2	5.952759782	0.013932914	0.025072685	-0.480590988	-0.598800349	-0.505663673	-0.623873035	-0.118209361	9.961963646	0.980492118	0.641186724	0.562673889	0.624092701	0.546730279	0.908251315	-6.998017285	-6.061775968	-6.854773466	-7.011556685	-6.093738485	-7.063750166	0.208174161	0.888429091

STK17A	-0.055574011	0.036986283	0.078937266	0.092560294	0.134511277	0.041950983	8	0.136810297	0.816496581	0.816496581	0.816496581	0.816496581	0.816496581	0.816496581	5	68	0	A_24_P337796	A_24_P337796	A_24_P337796	chr7	43624773	43624833	+	STK17A	serine/threonine kinase 17a [Source:HGNC Symbol;Acc:HGNC:11395]	ENSG00000164543	protein_coding	exon	coding exon	1	748	7:43624773:43624833	ENSG00000164543	STK17A	6.026640347	0.017237502	-0.518418166	0.345023879	0.736360611	0.863442045	1.254778777	0.391336731	9.961963646	0.61548509	0.737250164	0.478492525	0.408201838	0.23819771	0.703788012	-6.853660816	-6.119719678	-6.758840355	-6.756529377	-5.521172226	-6.988356243	0.567195367	0.649089572

KRI1	-0.272600177	-0.078896055	-0.1672156	0.193704122	0.105384577	-0.088319545	8	0.076533858	0.816496581	0.816496581	0.816496581	0.816496581	0.816496581	0.816496581	5	69	0	A_23_P101332	A_23_P101332	A_23_P101332	chr19	10553837	10553897	-	KRI1	KRI1 homolog (S. cerevisiae) [Source:HGNC Symbol;Acc:HGNC:25769]	ENSG00000129347	protein_coding	3'UTR;exon	multiple Ref	1	749	19:10553837:10553897	ENSG00000129347	KRI1	11.11327066	0.006910521	-4.016209636	-1.162373036	-2.463582051	2.8538366	1.552627586	-1.301209014	9.961963646	0.002472218	0.272173661	0.033554966	0.017198868	0.151676842	0.222475813	-1.793079636	-5.516002898	-4.464185113	-3.901798623	-5.152842344	-6.217604755	5.96641135	0.01348849

MYLK	0.676058316	-0.085904144	0.490956236	-0.76196246	-0.185102079	0.57686038	8	0.120489826	0.816496581	0.816496581	0.816496581	0.816496581	0.816496581	0.816496581	5	70	0	P_135793	P_135793	P_135793	chr3	123614236	123614296	+	MYLK;MYLK-AS1	myosin light chain kinase [Source:HGNC Symbol;Acc:HGNC:7590];MYLK antisense RNA 1 [Source:HGNC Symbol;Acc:HGNC:42440]	ENSG00000065534	protein_coding;antisense	3'UTR;exon;intron	multiple non-Ref	0	750	3:123614236:123614296	ENSG00000065534	MYLK	6.720671232	0.013865267	7.031786289	-0.893502184	5.106511156	-7.925288473	-1.925275133	6.00001334	9.961963646	3.65E-05	0.392662023	0.000465176	1.31E-05	0.083192619	0.00013418	2.570732339	-5.777904342	-0.105001725	3.544719198	-4.63031499	1.171089628	29.80631042	2.74E-05

NDUFS2	-0.022905455	0.113720584	0.112314977	0.136626038	0.135220432	-0.001405607	8	0.0742617	0.816496581	0.816496581	0.816496581	0.816496581	0.816496581	0.816496581	5	71	0	P_103824	P_103824	P_103824	chr1	161214312	161214372	+	NDUFS2	"NADH dehydrogenase (ubiquinone) Fe-S protein 2, 49kDa (NADH-coenzyme Q reductase) [Source:HGNC Symbol;Acc:HGNC:7708]"	ENSG00000158864	protein_coding	3'UTR;exon	multiple Ref	1	751	1:161214312:161214372	ENSG00000158864	NDUFS2	9.803427468	0.006635369	-0.344391164	1.70982698	1.688693216	2.054218144	2.03308438	-0.021133764	9.961963646	0.737711527	0.118206084	0.122281615	0.067139581	0.06955298	0.98355626	-6.93402372	-4.838573971	-5.678980912	-5.227830763	-4.469521019	-7.071134532	2.374581545	0.131569313

NME5	0.232312216	0.195057505	0.420796206	-0.037254712	0.18848399	0.225738702	8	0.059123814	0.816496581	0.816496581	0.816496581	0.816496581	0.816496581	0.816496581	5	72	0	P_140452	P_140452	P_140452	chr5	138115684	138115744	-	NME5	NME/NM23 family member 5 [Source:HGNC Symbol;Acc:HGNC:7853]	ENSG00000112981	protein_coding	exon	coding exon	1	752	5:138115684:138115744	ENSG00000112981	NME5	9.211612572	0.005013862	4.018199549	3.373821618	7.278322049	-0.644377931	3.2601225	3.904500432	9.961963646	0.002464326	0.007113326	2.72E-05	0.533885752	0.008614497	0.002960737	-1.789793185	-2.261385475	2.844193591	-6.927174107	-2.5083964	-2.042049842	17.79855284	0.000250769

SNRNP70	-0.084451337	0.026784429	-0.056843726	0.111235767	0.027607612	-0.083628155	8	0.092868762	0.816496581	0.816496581	0.816496581	0.816496581	0.816496581	0.816496581	5	73	0	A_16_P03459189	A_16_P03459189	A_16_P03459189	chr19	49102152	49102210	+	SNRNP70	small nuclear ribonucleoprotein 70kDa (U1) [Source:HGNC Symbol;Acc:HGNC:11150]	ENSG00000104852	protein_coding	3'UTR	3'UTR	1	753	19:49102152:49102210	ENSG00000104852	SNRNP70	7.723248382	0.009132714	-1.082311425	0.343263883	-0.728497805	1.425575308	0.35381362	-1.071761688	9.961963646	0.304615928	0.73853379	0.483082244	0.184563233	0.730852258	0.309104507	-6.394296091	-6.120350468	-6.764806594	-6.138813061	-6.230530198	-6.477709611	0.85434322	0.495767796

PAN2	-0.01894105	-0.099442564	-0.092159754	-0.080501514	-0.073218704	0.00728281	8	0.055374232	0.816496581	0.816496581	0.816496581	0.816496581	0.816496581	0.816496581	5	74	0	P_215708	P_215708	P_215708	chr12	56323061	56323121	-	PAN2	PAN2 poly(A) specific ribonuclease subunit [Source:HGNC Symbol;Acc:HGNC:20074]	ENSG00000135473	protein_coding	exon	coding exon	1	754	12:56323061:56323121	ENSG00000135473	PAN2	6.629063913	0.004669095	-0.339494976	-1.782385377	-1.651849985	-1.442890401	-1.312355009	0.130535392	9.961963646	0.741285417	0.105134391	0.129690528	0.179747477	0.218831905	0.898741187	-6.935829563	-4.737597056	-5.731379572	-6.116328247	-5.454086074	-7.062077801	1.640335118	0.242144373

IQGAP1	0.080512541	-0.044896214	0.070281876	-0.125408755	-0.010230665	0.11517809	8	0.040135425	0.816496581	0.816496581	0.816496581	0.816496581	0.816496581	0.816496581	5	75	0	P_224989	P_224989	P_224989	chr15	90474073	90474133	+	IQGAP1	IQ motif containing GTPase activating protein 1 [Source:HGNC Symbol;Acc:HGNC:6110]	ENSG00000140575	protein_coding	exon	coding exon	1	755	15:90474073:90474133	ENSG00000140575	IQGAP1	8.543422592	0.003500286	1.6666998	-0.929401922	1.454913571	-2.596101721	-0.211786229	2.384315493	9.961963646	0.126657127	0.374652928	0.176467324	0.026745835	0.836543928	0.038417921	-5.662736158	-5.746172382	-5.999717535	-4.339747913	-6.272883658	-4.618769888	3.038001263	0.079729677

STYXL2	-0.117485428	0.081864408	0.003060092	0.199349836	0.120545521	-0.078804315	8	0.187183911	0.816496581	0.816496581	0.816496581	0.816496581	0.816496581	0.816496581	5	76	0	P_204666	P_204666	P_204666	chr1	167113722	167113782	+	DUSP27	dual specificity phosphatase 27 (putative) [Source:HGNC Symbol;Acc:HGNC:25034]	ENSG00000198842	protein_coding	exon	coding exon	1	756	1:167113722:167113782	ENSG00000198842	STYXL2	4.454497743	0.030343961	-0.826025581	0.57557857	0.021515133	1.40160415	0.847540713	-0.554063437	9.961963646	0.428128901	0.577669234	0.983259575	0.191411888	0.416591457	0.591754475	-6.638321442	-6.010400293	-7.046867813	-6.16964495	-5.928200852	-6.906220872	0.667312933	0.591133881

HCN1	0.141517776	0.004237545	0.079747831	-0.137280231	-0.061769945	0.075510286	8	0.182559308	0.816496581	0.816496581	0.816496581	0.816496581	0.816496581	0.816496581	5	78	0	A_23_P113019	A_23_P113019	A_23_P113019	chr5	45261896	45261956	-	HCN1	hyperpolarization activated cyclic nucleotide-gated potassium channel 1 [Source:HGNC Symbol;Acc:HGNC:4845]	ENSG00000164588	protein_coding	"3'UTR,exon"	multiple Ref	1	758	5:45261896:45261956	ENSG00000164588	HCN1	3.613480822	0.028970806	1.018301392	0.030491559	0.573831287	-0.987809833	-0.444470105	0.543339728	9.961963646	0.332627505	0.976277256	0.578806291	0.346630302	0.66620295	0.598842557	-6.46028986	-6.181597318	-6.870225758	-6.640689682	-6.192671593	-6.912459949	0.472614968	0.708172872

AFF1	0.019874843	-0.189810144	0.001746231	-0.209684987	-0.018128612	0.191556375	8	0.046890574	0.816496581	0.816496581	0.816496581	0.816496581	0.816496581	0.816496581	5	80	0	P_314970	P_314970	P_314970	chr4	87140596	87140656	+	AFF1	"AF4/FMR2 family, member 1 [Source:HGNC Symbol;Acc:HGNC:7135]"	ENSG00000172493	protein_coding	3'UTR	3'UTR	1	760	4:87140596:87140656	ENSG00000172493	AFF1	6.492023049	0.003972381	0.386210332	-3.688413563	0.033932969	-4.074623895	-0.352277364	3.722346531	9.961963646	0.707461066	0.004214758	0.973600878	0.002251192	0.731968929	0.003985931	-6.917573997	-1.76725309	-7.046491822	-1.828769775	-6.231099789	-2.347425861	7.389537901	0.006816121

TJP2	-0.060391207	-0.032639506	0.014949321	0.0277517	0.075340528	0.047588827	8	0.067466522	0.816496581	0.816496581	0.816496581	0.816496581	0.816496581	0.816496581	5	81	0	P_267435	P_267435	P_267435	chr9	69237913	69237973	+	TJP2	tight junction protein 2 [Source:HGNC Symbol;Acc:HGNC:11828]	ENSG00000119139	protein_coding	exon	coding exon	1	761	9:69237913:69237973	ENSG00000119139	TJP2	7.087634768	0.005861973	-0.966045194	-0.522116379	0.239136134	0.443928815	1.205181328	0.761252513	9.961963646	0.356887734	0.613000601	0.815847949	0.666581221	0.255981018	0.464145248	-6.511741955	-6.040389388	-7.015980651	-7.042899056	-5.577225008	-6.763721309	0.582012523	0.640208031

DDX3X	0.13335821	-0.060329659	0.024841812	-0.193687869	-0.108516398	0.085171471	8	0.069425954	0.816496581	0.816496581	0.816496581	0.816496581	0.816496581	0.816496581	5	83	0	P_150736	P_150736	P_150736	chrX	41338506	41338566	+	DDX3X	"DEAD (Asp-Glu-Ala-Asp) box helicase 3, X-linked [Source:HGNC Symbol;Acc:HGNC:2745]"	ENSG00000215301	protein_coding	intron	intron sense	0	763	X:41338506:41338566	ENSG00000215301	DDX3X	9.564220145	0.006077377	2.095112227	-0.947803708	0.39027507	-3.042915935	-1.704837157	1.338078778	9.961963646	0.062693726	0.365653638	0.704548038	0.012452772	0.119157108	0.210611082	-5.017089199	-5.729503181	-6.964574185	-3.577961093	-4.946481295	-6.172411168	3.232733946	0.069400362

FAM24B	-0.223155895	-0.071073638	-0.356182311	0.152082257	-0.133026415	-0.285108672	8	0.087504271	0.816496581	0.816496581	0.816496581	0.816496581	0.816496581	0.816496581	5	84	0	P_210305	P_210305	P_210305	chr10	122850423	122850483	-	FAM24B	"family with sequence similarity 24, member B [Source:HGNC Symbol;Acc:HGNC:23475]"	ENSG00000213185	protein_coding	exon	coding exon	1	764	10:122850423:122850483	ENSG00000213185	FAM24B	8.933787118	0.00835567	-2.989945975	-0.952277504	-4.772295462	2.037668471	-1.782349487	-3.820017958	9.961963646	0.013629615	0.363489493	0.000762494	0.069022611	0.105140517	0.003396837	-3.527972519	-5.72540972	-0.618419085	-5.253841128	-4.837444245	-2.183305808	9.090468842	0.003348601

UPF3B	-0.018079064	0.003615886	0.07672516	0.02169495	0.094804223	0.073109274	8	0.061593964	0.816496581	0.816496581	0.816496581	0.816496581	0.816496581	0.816496581	5	85	0	P_271295	P_271295	P_271295	chrX	119851494	119851554	-	UPF3B	UPF3 regulator of nonsense transcripts homolog B (yeast) [Source:HGNC Symbol;Acc:HGNC:20439]	ENSG00000125351	protein_coding	exon	coding exon	1	765	X:119851494:119851554	ENSG00000125351	UPF3B	10.1159371	0.005253325	-0.305495217	0.061100276	1.296481365	0.366595493	1.601976582	1.235381088	9.961963646	0.766276971	0.952487785	0.224036495	0.721587185	0.140359717	0.245029551	-6.947671336	-6.180119748	-6.199401929	-7.076612731	-5.087135046	-6.296048319	1.000802026	0.432150808

RAX2	0.200296287	0.327147016	0.092265304	0.126850729	-0.108030983	-0.234881712	8	0.199073214	0.816496581	0.816496581	0.816496581	0.816496581	0.816496581	0.816496581	5	88	0	P_124190	P_124190	P_124190	chr19	3771536	3771596	-	RAX2	retina and anterior neural fold homeobox 2 [Source:HGNC Symbol;Acc:HGNC:18286]	ENSG00000173976	protein_coding	exon	coding exon	1	768	19:3771536:3771596	ENSG00000173976	RAX2	4.518590023	0.034031851	1.329767586	2.171929917	0.612549601	0.842162331	-0.717217985	-1.559380315	9.961963646	0.213238544	0.055091084	0.553898695	0.419455578	0.489714642	0.150082174	-6.110736874	-4.164855067	-6.846003391	-6.775018999	-6.030138705	-5.883730433	1.752680734	0.219694209

LRRC37A16P	-0.14005234	-0.230825496	-0.271734037	-0.090773156	-0.131681697	-0.040908542	8	0.182445679	0.816496581	0.816496581	0.816496581	0.816496581	0.816496581	0.816496581	5	89	0	P_229810	P_229810	P_229810	chr17	68126665	68126725	-	LRRC37A16P	"leucine rich repeat containing 37, member A16, pseudogene [Source:HGNC Symbol;Acc:HGNC:43820]"	ENSG00000267023	transcribed_unprocessed_pseudogene	exon	non-coding exon	1	769	17:68126665:68126725	ENSG00000267023	LRRC37A16P	5.860330998	0.028937499	-1.008336537	-1.661877131	-1.956406856	-0.653540595	-0.948070319	-0.294529725	9.961963646	0.337156651	0.127635205	0.079015656	0.528203531	0.365524409	0.774399198	-6.470272001	-4.904100679	-5.280671404	-6.920935951	-5.839633842	-7.024194518	1.503134392	0.273221234

FSD1L	0.326434006	0.105474016	0.472339736	-0.22095999	0.14590573	0.36686572	8	0.117159417	0.816496581	0.816496581	0.816496581	0.816496581	0.816496581	0.816496581	5	90	0	P_268355	P_268355	P_268355	chr9	105546422	105546482	+	FSD1L	fibronectin type III and SPRY domain containing 1-like [Source:HGNC Symbol;Acc:HGNC:13753]	ENSG00000106701	protein_coding	exon	coding exon	1	770	9:105546422:105546482	ENSG00000106701	FSD1L	6.821587503	0.013229675	3.475893346	1.123095094	5.029508313	-2.352798253	1.553614967	3.906413219	9.961963646	0.005995803	0.287734037	0.000520532	0.040537954	0.151442744	0.002951571	-2.699136213	-5.55761246	-0.221885196	-4.745870586	-5.151539525	-2.038859603	10.30809572	0.002127796

DOCK9	0.101131453	-0.001988172	-0.005764941	-0.103119626	-0.106896394	-0.003776768	8	0.053908815	0.816496581	0.816496581	0.816496581	0.816496581	0.816496581	0.816496581	5	91	0	P_219744	P_219744	P_219744	chr13	98920953	98921013	-	DOCK9	dedicator of cytokinesis 9 [Source:HGNC Symbol;Acc:HGNC:14132]	ENSG00000088387	protein_coding	exon	coding exon	1	771	13:98920953:98921013	ENSG00000088387	DOCK9	6.000813818	0.004540489	1.838148327	-0.036136688	-0.104782596	-1.874285014	-1.942930923	-0.068645909	9.961963646	0.096002825	0.971887227	0.918628	0.090482181	0.080799019	0.946629798	-5.412822614	-6.181399059	-7.04112797	-5.505720542	-4.604231809	-7.068804813	1.780618612	0.214490108

SPINK5	0.436443183	0.08782249	0.20439453	-0.348620693	-0.232048652	0.116572041	8	0.096234507	0.816496581	0.816496581	0.816496581	0.816496581	0.816496581	0.816496581	5	92	0	P_255305	P_255305	P_255305	chr5	148111782	148111842	+	SPINK5	"serine peptidase inhibitor, Kazal type 5 [Source:HGNC Symbol;Acc:HGNC:15464]"	ENSG00000133710	protein_coding	exon	coding exon	1	772	5:148111782:148111842	ENSG00000133710	SPINK5	4.063864894	0.009643837	5.443126451	1.09528327	2.549118234	-4.347843181	-2.894008216	1.453834964	9.961963646	0.000287399	0.299165581	0.028986948	0.001461464	0.016056647	0.176759471	0.432563982	-5.586412905	-4.3212595	-1.380991689	-3.10360723	-6.024978681	11.11965579	0.001606583

CD55	0.031494449	0.272476755	0.318969498	0.240982306	0.287475049	0.046492743	8	0.129040309	0.816496581	0.816496581	0.816496581	0.816496581	0.816496581	0.816496581	5	93	0	A_24_P188377	A_24_P188377	A_24_P188377	chr1	207336725	207336785	+	CD55	"CD55 molecule, decay accelerating factor for complement (Cromer blood group) [Source:HGNC Symbol;Acc:HGNC:2665]"	ENSG00000196352	protein_coding	exon	coding exon	1	773	1:207336725:207336785	ENSG00000196352	CD55	4.604519326	0.015578667	0.309039861	2.673683147	3.129894042	2.364643286	2.820854181	0.456210895	9.961963646	0.763657712	0.023416501	0.010740327	0.039728096	0.018197792	0.658022414	-6.946493901	-3.379443669	-3.333605141	-4.726329028	-3.2225637	-6.958856995	5.132868079	0.021087859

HID1	-0.047273947	0.083800377	0.086011759	0.131074323	0.133285706	0.002211383	8	0.085699041	0.816496581	0.816496581	0.816496581	0.816496581	0.816496581	0.816496581	5	94	0	P_229991	P_229991	P_229991	chr17	74958140	74958200	-	HID1	HID1 domain containing [Source:HGNC Symbol;Acc:HGNC:15736]	ENSG00000167861	protein_coding	3'UTR;exon	multiple Ref	1	774	17:74958140:74958200	ENSG00000167861	HID1	7.425502117	0.008104578	-0.643135212	1.140056562	1.170141167	1.783191774	1.813276379	0.030084605	9.961963646	0.534659167	0.280931191	0.269176375	0.10499684	0.099981616	0.976593765	-6.77724678	-5.539777083	-6.346835546	-5.641837747	-4.793264113	-7.070884097	1.591871904	0.252635078

UNC79	0.018434059	0.041873433	0.056433293	0.023439374	0.037999234	0.01455986	8	0.083650555	0.816496581	0.816496581	0.816496581	0.816496581	0.816496581	0.816496581	5	95	0	P_222021	P_222021	P_222021	chr14	93580306	93580366	+	UNC79	unc-79 homolog (C. elegans) [Source:HGNC Symbol;Acc:HGNC:19966]	ENSG00000133958	protein_coding	exon	coding exon	1	775	14:93580306:93580366	ENSG00000133958	UNC79	4.10931604	0.00782599	0.255209572	0.579715015	0.781288394	0.324505443	0.526078822	0.201573379	9.961963646	0.803752839	0.574982266	0.452800118	0.752266641	0.610344297	0.844306743	-6.962934962	-6.0079661	-6.723655403	-7.092330011	-6.151555319	-7.049226681	0.239051253	0.867145318

TTC7A	-0.168106259	0.072599582	-0.147342081	0.240705841	0.020764178	-0.219941663	8	0.091035645	0.816496581	0.816496581	0.816496581	0.816496581	0.816496581	0.816496581	5	96	0	P_127773	P_127773	P_127773	chr2	46916189	46916249	+	TTC7A	tetratricopeptide repeat domain 7A [Source:HGNC Symbol;Acc:HGNC:19750]	ENSG00000068724	protein_coding	5'UTR	5'UTR	1	776	2:46916189:46916249	ENSG00000068724	TTC7A	6.191512422	0.008861989	-2.187076139	0.944526483	-1.916932492	3.131602622	0.270143647	-2.861458976	9.961963646	0.053700284	0.367244848	0.084346552	0.01070921	0.792563524	0.016975996	-4.870481473	-5.732491678	-5.341161669	-3.425805694	-6.258015643	-3.815122343	4.569653756	0.029203272

WBP4	-0.034016409	0.049689028	0.005869139	0.083705436	0.039885547	-0.043819889	8	0.05325825	0.816496581	0.816496581	0.816496581	0.816496581	0.816496581	0.816496581	5	98	0	P_218897	P_218897	P_218897	chr13	41072795	41072855	+	WBP4	WW domain binding protein 4 [Source:HGNC Symbol;Acc:HGNC:12739]	ENSG00000120688	protein_coding	exon	coding exon	1	778	13:41072795:41072855	ENSG00000120688	WBP4	9.390350993	0.004484501	-0.622124103	0.908759713	0.107340336	1.530883817	0.729464439	-0.801419378	9.961963646	0.547833982	0.384934961	0.916649987	0.156913273	0.482516508	0.441585085	-6.791188709	-5.764546346	-7.040832012	-5.999389238	-6.021201294	-6.731426284	0.790400202	0.526517384

DOCK8	-0.180586295	-0.057761638	-0.122869501	0.122824657	0.057716794	-0.065107863	8	0.084219057	0.816496581	0.816496581	0.816496581	0.816496581	0.816496581	0.816496581	5	99	0	P_266575	P_266575	P_266575	chr9	449803	449863	+	DOCK8	dedicator of cytokinesis 8 [Source:HGNC Symbol;Acc:HGNC:19191]	ENSG00000107099	protein_coding	exon	coding exon	1	779	9:449803:449863	ENSG00000107099	DOCK8	4.011349328	0.007902629	-2.487967045	-0.795791578	-1.692793297	1.692175468	0.795173749	-0.897001719	9.961963646	0.032184719	0.444701715	0.121481151	0.121601467	0.445044746	0.390880157	-4.377363376	-5.858845306	-5.673110151	-5.774326265	-5.970954891	-6.648785095	2.331530764	0.13613311

MFSD1	-0.016558982	0.060322	-0.035428965	0.076880982	-0.018869984	-0.095750966	8	0.054198018	0.816496581	0.816496581	0.816496581	0.816496581	0.816496581	0.816496581	5	100	0	A_23_P166677	A_23_P166677	A_23_P166677	chr3	158829521	158829581	+	MFSD1	major facilitator superfamily domain containing 1 [Source:HGNC Symbol;Acc:HGNC:25874]	ENSG00000118855	protein_coding	3'UTR;exon	multiple Ref	1	780	3:158829521:158829581	ENSG00000118855	MFSD1	8.628755359	0.004565597	-0.300144572	1.093383707	-0.642177869	1.393528279	-0.342033297	-1.735561575	9.961963646	0.770236595	0.299958987	0.535255421	0.193767358	0.7394318	0.113409607	-6.949423423	-5.588359635	-6.826479258	-6.179953646	-6.234836134	-5.635089274	1.128174822	0.383897675

CCSER2	0.101481894	0.076767413	0.069166495	-0.024714481	-0.032315398	-0.007600918	8	0.133202945	0.816496581	0.816496581	0.816496581	0.816496581	0.816496581	0.816496581	5	101	0	P_106481	P_106481	P_106481	chr10	84424969	84425029	+	CCSER2	coiled-coil serine-rich protein 2 [Source:HGNC Symbol;Acc:HGNC:29197]	ENSG00000107771	protein_coding	5'UTR;exon	multiple Ref	1	781	10:84424969:84425029	ENSG00000107771	CCSER2	7.293201325	0.0164553	0.968905205	0.73294204	0.660371765	-0.235963165	-0.30853344	-0.072570275	9.961963646	0.355527339	0.48048464	0.523990458	0.818241702	0.764031733	0.943584445	-6.508983594	-5.906568599	-6.81406984	-7.119249579	-6.246301809	-7.068502213	0.344701691	0.793777575

TMEM51	0.125345377	0.004790758	0.29370761	-0.12055462	0.168362233	0.288916852	8	0.157961684	0.816496581	0.816496581	0.816496581	0.816496581	0.816496581	0.816496581	5	102	0	A_23_P12044	A_23_P12044	A_23_P12044	chr1	15220302	15220362	+	TMEM51	transmembrane protein 51 [Source:HGNC Symbol;Acc:HGNC:25488]	ENSG00000171729	protein_coding	3'UTR	3'UTR	1	782	1:15220302:15220362	ENSG00000171729	TMEM51	6.854389935	0.022244415	1.029303513	0.03934045	2.411850211	-0.989963063	1.382546698	2.372509761	9.961963646	0.327679841	0.969396196	0.036654941	0.345627407	0.197009729	0.039199021	-6.44917643	-6.181271602	-4.550076953	-6.638570228	-5.369504948	-4.638259428	2.566383273	0.113300335

SLC5A1	0.340249446	-0.215237806	0.126847024	-0.555487252	-0.213402422	0.34208483	8	0.12328982	0.816496581	0.816496581	0.816496581	0.816496581	0.816496581	0.816496581	5	103	0	P_133129	P_133129	P_133129	chr22	32102140	32102200	+	SLC5A1	"solute carrier family 5 (sodium/glucose cotransporter), member 1 [Source:HGNC Symbol;Acc:HGNC:11036]"	ENSG00000100170	protein_coding	exon	coding exon	1	783	22:32102140:32102200	ENSG00000100170	SLC5A1	4.501331791	0.014413418	3.471039624	-2.195738925	1.294024284	-5.666778549	-2.17701534	3.489763209	9.961963646	0.006044594	0.052920082	0.224851187	0.000210566	0.054620297	0.005858614	-2.707376212	-4.128510653	-6.202373235	0.640912684	-4.249746602	-2.741240024	11.26235109	0.001531569

CUBN	-0.005113479	-0.141419176	-0.09648097	-0.136305697	-0.091367491	0.044938206	8	0.095550976	0.816496581	0.816496581	0.816496581	0.816496581	0.816496581	0.816496581	5	104	0	P_207565	P_207565	P_207565	chr10	17088163	17088223	-	CUBN	cubilin (intrinsic factor-cobalamin receptor) [Source:HGNC Symbol;Acc:HGNC:2548]	ENSG00000107611	protein_coding	exon	coding exon	1	784	10:17088163:17088223	ENSG00000107611	CUBN	3.790123952	0.009538563	-0.064124002	-1.773423485	-1.209889797	-1.709299483	-1.145765795	0.563533688	9.961963646	0.950139964	0.10667421	0.254248096	0.118306295	0.278669932	0.585532141	-6.996116959	-4.750182746	-6.301650999	-5.749683925	-5.642166657	-6.900615819	1.527505044	0.267382771

HNRNPU	-0.077096376	-0.209361236	-0.127177542	-0.13226486	-0.050081166	0.082183694	8	0.134759083	0.816496581	0.816496581	0.816496581	0.816496581	0.816496581	0.816496581	5	105	0	P_207100	P_207100	P_207100	chr1	244856727	244856787	-	HNRNPU	heterogeneous nuclear ribonucleoprotein U (scaffold attachment factor A) [Source:HGNC Symbol;Acc:HGNC:5048]	ENSG00000153187	protein_coding	exon	coding exon	1	785	1:244856727:244856787	ENSG00000153187	HNRNPU	11.07490968	0.016790163	-0.728705661	-1.978857185	-1.20206681	-1.250151523	-0.473361148	0.776790375	9.961963646	0.482960558	0.076126461	0.257132499	0.239813168	0.64615765	0.455331223	-6.716111201	-4.454444666	-6.31063293	-6.356058781	-6.178869491	-6.751402648	1.380367535	0.304926725

CCR2	0.03709294	-0.005472965	0.271966548	-0.042565905	0.234873608	0.277439513	8	0.274200024	0.816496581	0.816496581	0.816496581	0.816496581	0.816496581	0.816496581	5	106	0	P_134613	P_134613	P_134613	chr3	46358246	46358306	+	CCR2	chemokine (C-C motif) receptor 2 [Source:HGNC Symbol;Acc:HGNC:1603]	ENSG00000121807	protein_coding	exon	coding exon	1	786	3:46358246:46358306	ENSG00000121807	CCR2	3.685011868	0.062584863	0.18159431	-0.026793759	1.331454914	-0.208388069	1.149860604	1.358248673	9.961963646	0.859542808	0.979153373	0.212702918	0.83912488	0.277056929	0.204347083	-6.980395263	-6.181708979	-6.15667961	-7.125916985	-5.637770182	-6.147317422	0.83615184	0.504325042

TRIP12	0.078070525	-0.122232748	-0.034304848	-0.200303273	-0.112375373	0.0879279	8	0.058186042	0.816496581	0.816496581	0.816496581	0.816496581	0.816496581	0.816496581	5	107	0	P_239585	P_239585	P_239585	chr2	229803998	229804058	-	TRIP12	thyroid hormone receptor interactor 12 [Source:HGNC Symbol;Acc:HGNC:12306]	ENSG00000153827	protein_coding	exon	coding exon	1	787	2:229803998:229804058	ENSG00000153827	TRIP12	8.228406196	0.004925518	1.36240673	-2.133080549	-0.598653024	-3.495487279	-1.961059754	1.534427525	9.961963646	0.203075386	0.058818404	0.562768335	0.005802948	0.07840853	0.156049199	-6.070227037	-4.223884315	-6.854866437	-2.802914426	-4.577344671	-5.917678297	4.197204113	0.036650317

CACNA2D4	-0.03427617	0.463353311	0.236616666	0.497629481	0.270892836	-0.226736645	8	0.346825666	0.816496581	0.816496581	0.816496581	0.816496581	0.816496581	0.816496581	5	108	0	P_110619	P_110619	P_110619	chr12	1792288	1792348	-	CACNA2D4	"calcium channel, voltage-dependent, alpha 2/delta subunit 4 [Source:HGNC Symbol;Acc:HGNC:20202]"	ENSG00000151062	protein_coding	3'UTR;exon	multiple Ref	1	788	12:1792288:1792348	ENSG00000151062	CACNA2D4	3.988672092	0.098804541	-0.133551715	1.805383408	0.921939679	1.938935124	1.055491394	-0.88344373	9.961963646	0.896416446	0.10127566	0.37834709	0.081335018	0.316125771	0.397815159	-6.988636092	-4.705158324	-6.601824133	-5.407169279	-5.736007629	-6.66099377	1.630203734	0.244295525

USP15	0.061273704	-0.021009822	0.142302159	-0.082283527	0.081028455	0.163311981	8	0.057324713	0.816496581	0.816496581	0.816496581	0.816496581	0.816496581	0.816496581	5	109	0	A_23_P325802	A_23_P325802	A_23_P325802	chr12	62336266	62336326	+	USP15	ubiquitin specific peptidase 15 [Source:HGNC Symbol;Acc:HGNC:12613]	ENSG00000135655	protein_coding	3'UTR	3'UTR	1	789	12:62336266:62336326	ENSG00000135655	USP15	8.997983476	0.00484562	1.078065386	-0.369652242	2.503700954	-1.447717628	1.425635568	2.873353197	9.961963646	0.306416439	0.719378384	0.031330066	0.178424274	0.184546284	0.016634031	-6.398772638	-6.110563251	-4.397281167	-6.110028184	-5.316131499	-3.794825917	3.325311321	0.065050415

KIF9	0.058055618	0.121583495	0.2282809	0.063527877	0.170225281	0.106697404	8	0.053670466	0.816496581	0.816496581	0.816496581	0.816496581	0.816496581	0.816496581	5	111	0	A_24_P916686	A_24_P916686	A_24_P916686	chr3	47283074	47287259	+	KIF9;KLHL18	kinesin family member 9 [Source:HGNC Symbol;Acc:HGNC:16666];kelch-like family member 18 [Source:HGNC Symbol;Acc:HGNC:29120]	ENSG00000088727	protein_coding	"5'UTR,intron,promoter;exon,intron;promoter"	multiple non-Ref	0	791	3:47283074:47287259	ENSG00000088727	KIF9	7.352238541	0.004519898	1.057610074	2.214909309	4.158635909	1.157299235	3.101025835	1.9437266	9.961963646	0.315204675	0.051232173	0.001969045	0.274145571	0.011280275	0.080692682	-6.420143085	-4.099158476	-1.600593631	-6.462670663	-2.766719213	-5.323692317	6.342065132	0.011162601

DNAJB9	0.188788704	0.1690434	0.361507201	-0.019745304	0.172718497	0.192463801	8	0.056280705	0.816496581	0.816496581	0.816496581	0.816496581	0.816496581	0.816496581	5	112	0	A_23_P258944	A_23_P258944	A_23_P258944	chr7	108574070	108574130	+	DNAJB9	"DnaJ (Hsp40) homolog, subfamily B, member 9 [Source:HGNC Symbol;Acc:HGNC:6968]"	ENSG00000128590	protein_coding	3'UTR	3'UTR	1	792	7:108574070:108574130	ENSG00000128590	DNAJB9	9.696698178	0.004750373	3.354731421	3.003861955	6.423899005	-0.350869466	3.069167584	3.42003705	9.961963646	0.007345159	0.013309894	7.73E-05	0.732992876	0.01190842	0.006582829	-2.905188523	-2.851363867	1.762240225	-7.082703754	-2.818540596	-2.859970404	13.79724007	0.000701242

H4C2	-0.243691734	-0.526178859	-0.530504465	-0.282487125	-0.286812732	-0.004325606	8	0.178714867	0.816496581	0.816496581	0.816496581	0.816496581	0.816496581	0.816496581	5	113	0	A_24_P166407	A_24_P166407	A_24_P166407	chr6	26026920	26026980	-	HIST1H4B	"histone cluster 1, H4b [Source:HGNC Symbol;Acc:HGNC:4789]"	ENSG00000278705	protein_coding	"3'UTR,exon"	multiple Ref	1	793	6:26026920:26026980	ENSG00000278705	H4C2	10.21474259	0.027855446	-1.788262923	-3.861214867	-3.892957106	-2.072951943	-2.104694183	-0.03174224	9.961963646	0.104135602	0.003176344	0.003016693	0.065066912	0.061693334	0.975304566	-5.486843415	-1.500271824	-2.039948916	-5.198286548	-4.360844407	-7.070828114	6.998309083	0.008149517

IGSF1	0.001363651	-0.049437583	0.025668399	-0.050801233	0.024304749	0.075105982	8	0.125173756	0.816496581	0.816496581	0.816496581	0.816496581	0.816496581	0.816496581	5	115	0	P_271526	P_271526	P_271526	chrX	131277907	131277967	-	IGSF1	"immunoglobulin superfamily, member 1 [Source:HGNC Symbol;Acc:HGNC:5948]"	ENSG00000147255	protein_coding	exon	coding exon	1	795	X:131277907:131277967	ENSG00000147255	IGSF1	3.859292472	0.014789319	0.013733293	-0.497884684	0.258505821	-0.511617976	0.244772529	0.756390505	9.961963646	0.989313906	0.629371906	0.801279028	0.620066828	0.81160065	0.466925713	-6.998257399	-6.053073568	-7.010749405	-7.008328562	-6.26491664	-6.767530544	0.20144936	0.893022584

KIF16B	0.084375456	-0.062216396	0.068724013	-0.146591852	-0.015651443	0.130940409	8	0.0697809	0.816496581	0.816496581	0.816496581	0.816496581	0.816496581	0.816496581	5	116	0	P_240263	P_240263	P_240263	chr20	16477736	16477796	-	KIF16B	kinesin family member 16B [Source:HGNC Symbol;Acc:HGNC:15869]	ENSG00000089177	protein_coding	intron	intron sense	0	796	20:16477736:16477796	ENSG00000089177	KIF16B	5.728849132	0.006117057	1.321266757	-0.974269762	1.076174971	-2.295536519	-0.245091787	2.050444733	9.961963646	0.215954175	0.352985841	0.307220679	0.044685777	0.811360264	0.067564583	-6.121175754	-5.70505662	-6.449037827	-4.839942266	-6.264833971	-5.157894944	2.231163579	0.147513702

KDM1A	0.065083783	-0.068926073	-0.007246054	-0.134009856	-0.072329838	0.061680019	8	0.059088579	0.816496581	0.816496581	0.816496581	0.816496581	0.816496581	0.816496581	5	117	0	P_100750	P_100750	P_100750	chr1	23083110	23083170	+	KDM1A	lysine (K)-specific demethylase 1A [Source:HGNC Symbol;Acc:HGNC:29079]	ENSG00000004487	protein_coding	exon	non-coding exon	1	797	1:23083110:23083170	ENSG00000004487	KDM1A	8.487546135	0.005010517	1.126100508	-1.192581038	-0.125373559	-2.318681546	-1.251474067	1.067207479	9.961963646	0.286519348	0.260665119	0.902721835	0.042961691	0.239350535	0.311057729	-6.347328484	-5.483273571	-7.038543368	-4.802000034	-5.524957828	-6.482479362	1.797912279	0.211340132

ATF7	-0.077006814	-0.08185148	-0.23925239	-0.004844666	-0.162245576	-0.15740091	8	0.057304415	0.816496581	0.816496581	0.816496581	0.816496581	0.816496581	0.816496581	5	118	0	P_111736	P_111736	P_111736	chr12	53537414	53537474	-	ATF7;RP11-793H13.10	activating transcription factor 7 [Source:HGNC Symbol;Acc:HGNC:792];Uncharacterized protein [Source:UniProtKB/TrEMBL;Acc:K7ELQ4]	ENSG00000170653	protein_coding	exon	coding exon	1	798	12:53537414:53537474	ENSG00000170653	ATF7	6.904809501	0.004843751	-1.355139083	-1.440393827	-4.210280194	-0.085254745	-2.855141111	-2.769886367	9.961963646	0.205302486	0.180435118	0.001814295	0.933747936	0.017160517	0.019857671	-6.079305675	-5.192714616	-1.516103622	-7.145639692	-3.16682699	-3.971110139	6.244827541	0.011715227

PRKG2	0.109924436	-0.141157588	-0.019391641	-0.251082024	-0.129316077	0.121765947	8	0.096550316	0.816496581	0.816496581	0.816496581	0.816496581	0.816496581	0.816496581	5	119	0	P_249650	P_249650	P_249650	chr4	81204586	81204646	-	PRKG2	"protein kinase, cGMP-dependent, type II [Source:HGNC Symbol;Acc:HGNC:9416]"	ENSG00000138669	protein_coding	exon	coding exon	1	799	4:81204586:81204646	ENSG00000138669	PRKG2	5.131023596	0.009692729	1.36746696	-1.756009349	-0.241233242	-3.123476309	-1.608700201	1.514776107	9.961963646	0.201536758	0.109725395	0.814266936	0.010858035	0.138877195	0.160893834	-6.063886297	-4.774548368	-7.015433698	-3.439748821	-5.07809072	-5.944176567	3.27504924	0.067370589

FIP1L1	0.113702721	-0.161826652	0.085132021	-0.275529372	-0.0285707	0.246958673	8	0.049236342	0.816496581	0.816496581	0.816496581	0.816496581	0.816496581	0.816496581	5	120	0	P_248990	P_248990	P_248990	chr4	53399776	53399836	+	FIP1L1	factor interacting with PAPOLA and CPSF1 [Source:HGNC Symbol;Acc:HGNC:19124]	ENSG00000145216	protein_coding	3'UTR;exon	multiple Ref	1	800	4:53399776:53399836	ENSG00000145216	FIP1L1	7.745409629	0.004153463	2.16078387	-3.075321467	1.617831983	-5.236105337	-0.542951887	4.693153451	9.961963646	0.056136618	0.011784362	0.136885993	0.000385719	0.599099742	0.000859167	-4.912626979	-2.736988506	-5.779177071	0.007651441	-6.142237681	-0.762359277	11.08020909	0.001628092

PARP11	0.138344146	0.109877876	0.05266902	-0.028466271	-0.085675126	-0.057208856	8	0.053438825	0.816496581	0.816496581	0.816496581	0.816496581	0.816496581	0.816496581	5	121	0	P_214088	P_214088	P_214088	chr12	3829920	3829980	-	PARP11	"poly (ADP-ribose) polymerase family, member 11 [Source:HGNC Symbol;Acc:HGNC:1186]"	ENSG00000111224	protein_coding	"5'UTR;5'UTR,exon;exon"	multiple Ref	1	801	12:3829920:3829980	ENSG00000111224	PARP11	6.194336351	0.004499973	2.525814501	2.006092338	0.961603206	-0.519722164	-1.564211296	-1.044489132	9.961963646	0.03016668	0.072754948	0.359008121	0.61460847	0.148950408	0.320941849	-4.314195419	-4.414205074	-6.564386732	-7.003877318	-5.137527121	-6.506031265	2.52277529	0.117177005

EPN1	0.030934982	-0.083021414	0.11534016	-0.113956396	0.084405178	0.198361574	8	0.054136804	0.816496581	0.816496581	0.816496581	0.816496581	0.816496581	0.816496581	5	122	0	P_126867	P_126867	P_126867	chr19	55695705	55695765	+	EPN1	epsin 1 [Source:HGNC Symbol;Acc:HGNC:21604]	ENSG00000063245	protein_coding	3'UTR	3'UTR	1	802	19:55695705:55695765	ENSG00000063245	EPN1	7.433543555	0.004560271	0.561048194	-1.505706859	2.091851513	-2.066755053	1.530803319	3.597558372	9.961963646	0.587161808	0.163173598	0.063037672	0.065745697	0.156932949	0.004897318	-6.829269663	-5.110177866	-5.069181942	-5.208071117	-5.181512247	-2.558246316	4.419172077	0.031971997

SPRYD7	-0.219356135	0.226271612	-0.094875081	0.445627747	0.124481054	-0.321146693	8	0.034305992	0.816496581	0.816496581	0.816496581	0.816496581	0.816496581	0.816496581	5	123	0	P_219186	P_219186	P_219186	chr13	49915072	49915132	-	SPRYD7	SPRY domain containing 7 [Source:HGNC Symbol;Acc:HGNC:14297]	ENSG00000123178	protein_coding	exon	coding exon	1	803	13:49915072:49915132	ENSG00000123178	SPRYD7	8.911700578	0.0031518	-4.785376448	4.936241432	-2.069752813	9.721617881	2.715623636	-7.005994245	9.961963646	0.000747662	0.000597154	0.065416496	2.12E-06	0.021792497	3.76E-05	-0.558671724	0.064966593	-5.104077399	5.425968484	-3.393330262	2.494690425	33.75310087	1.57E-05

SLCO5A1	0.275826301	0.095022521	0.283096892	-0.180803781	0.00727059	0.188074371	8	0.061383528	0.816496581	0.816496581	0.816496581	0.816496581	0.816496581	0.816496581	5	124	0	P_147381	P_147381	P_147381	chr8	69672585	69672645	-	SLCO5A1	"solute carrier organic anion transporter family, member 5A1 [Source:HGNC Symbol;Acc:HGNC:19046]"	ENSG00000137571	protein_coding	3'UTR	3'UTR	1	804	8:69672585:69672645	ENSG00000137571	SLCO5A1	7.060408903	0.005232543	4.670086149	1.608850773	4.793186382	-3.061235375	0.123100233	3.184335609	9.961963646	0.000889736	0.138844155	0.000738953	0.012070294	0.90447586	0.009792857	-0.738760557	-4.975376382	-0.585865582	-3.546536191	-6.288631552	-3.262771233	11.14984168	0.001590354

BMI1	0.002185851	-0.18095234	-0.177533955	-0.183138191	-0.179719806	0.003418384	8	0.070256813	0.816496581	0.816496581	0.816496581	0.816496581	0.816496581	0.816496581	5	125	0	P_207697	P_207697	P_207697	chr10	22329064	22329124	+	BMI1	"BMI1 proto-oncogene, polycomb ring finger [Source:HGNC Symbol;Acc:HGNC:1066]"	ENSG00000168283	protein_coding	exon	coding exon	1	805	10:22329064:22329124	ENSG00000168283	BMI1	7.777984272	0.006170577	0.03408029	-2.821284906	-2.767987799	-2.855365196	-2.802068089	0.053297107	9.961963646	0.973486314	0.018184377	0.019922363	0.017153937	0.018792699	0.95854883	-6.997726552	-3.143702204	-3.951552905	-3.899186356	-3.253085637	-7.069826928	5.271683832	0.019521541

F5	-1.792116177	0.245809985	-1.742670493	2.037926162	0.049445684	-1.988480478	8	0.119640214	0.816496581	0.816496581	0.816496581	0.816496581	0.816496581	0.816496581	5	126	0	P_204765	P_204765	P_204765	chr1	169530844	169530904	-	F5	"coagulation factor V (proaccelerin, labile factor) [Source:HGNC Symbol;Acc:HGNC:3542]"	ENSG00000198734	protein_coding	exon	coding exon	1	806	1:169530844:169530904	ENSG00000198734	F5	5.831582238	0.01370143	-18.75119005	2.571948068	-18.23383217	21.32313811	0.517357875	-20.80578024	9.961963646	4.24E-09	0.027875591	5.56E-09	1.21E-09	0.616198345	1.54E-09	11.29786212	-3.541174405	11.12858151	12.57606816	-6.156262344	12.25832987	263.0865084	8.79E-10

S100PBP	-0.140828985	-0.033616238	-0.018105719	0.107212748	0.122723266	0.015510518	8	0.082395296	0.816496581	0.816496581	0.816496581	0.816496581	0.816496581	0.816496581	5	127	0	A_23_P200425	A_23_P200425	A_23_P200425	chr1	32856012	32856072	+	S100PBP	S100P binding protein [Source:HGNC Symbol;Acc:HGNC:25768]	ENSG00000116497	protein_coding	"3'UTR,exon;exon"	multiple Ref	1	807	1:32856012:32856072	ENSG00000116497	S100PBP	8.743881429	0.007658609	-1.970891567	-0.470456839	-0.253388245	1.500434729	1.717503322	0.217068594	9.961963646	0.07714002	0.648159703	0.80512071	0.164511758	0.116756444	0.832535943	-5.211197173	-6.066739385	-7.01217061	-6.040349207	-4.928835142	-7.045698488	1.56969827	0.25760791

MCM10	-0.543928133	-0.05364	-0.236852174	0.490288133	0.307075958	-0.183212174	8	0.064619918	0.816496581	0.816496581	0.816496581	0.816496581	0.816496581	0.816496581	5	129	0	P_105512	P_105512	P_105512	chr10	13211026	13211086	+	MCM10	minichromosome maintenance complex component 10 [Source:HGNC Symbol;Acc:HGNC:18043]	ENSG00000065328	protein_coding	3'UTR	3'UTR	1	809	10:13211026:13211086	ENSG00000065328	MCM10	9.252773591	0.005560026	-8.934057473	-0.881040736	-3.890313461	8.053016737	5.043744012	-3.009272726	9.961963646	4.54E-06	0.399053205	0.003029663	1.14E-05	0.000509791	0.01318765	4.704219988	-5.788672472	-2.044357812	3.689784558	0.206139506	-3.56249299	32.51238485	1.86E-05

WDR53	-0.116774687	0.015886725	0.055681146	0.132661412	0.172455832	0.039794421	8	0.085568839	0.816496581	0.816496581	0.816496581	0.816496581	0.816496581	0.816496581	5	130	0	A_23_P110076	A_23_P110076	A_23_P110076	chr3	196554475	196554535	-	WDR53	WD repeat domain 53 [Source:HGNC Symbol;Acc:HGNC:28786]	ENSG00000185798	protein_coding	exon	coding exon	1	810	3:196554475:196554535	ENSG00000185798	WDR53	9.619313011	0.00808667	-1.590411369	0.216369052	0.758348659	1.806780421	2.348760028	0.541979606	9.961963646	0.142942678	0.833066433	0.465804609	0.101045506	0.040817723	0.599744736	-5.769578883	-6.157467812	-6.741852172	-5.606913724	-3.981349627	-6.913243066	2.037697938	0.172750404

ACAD10	0.089542986	-0.050547058	0.089019598	-0.140090045	-0.000523388	0.139566657	8	0.052748369	0.816496581	0.816496581	0.816496581	0.816496581	0.816496581	0.816496581	5	131	0	A_24_P8284	A_24_P8284	A_24_P8284	chr12	111712514	111712574	+	ACAD10	"acyl-CoA dehydrogenase family, member 10 [Source:HGNC Symbol;Acc:HGNC:21597]"	ENSG00000111271	protein_coding	exon	coding exon	1	811	12:111712514:111712574	ENSG00000111271	ACAD10	6.869911645	0.004441095	1.645629875	-0.928958849	1.636010993	-2.574588724	-0.009618882	2.564969842	9.961963646	0.130980365	0.374871548	0.132997609	0.027749804	0.992515262	0.028210727	-5.69253096	-5.746570387	-5.753705251	-4.376006266	-6.296636046	-4.317653289	3.242544018	0.068923407

ARHGEF10	0.036587379	-0.224965828	-0.020575472	-0.261553207	-0.057162851	0.204390356	8	0.065759441	0.816496581	0.816496581	0.816496581	0.816496581	0.816496581	0.816496581	5	132	0	P_263645	P_263645	P_263645	chr8	1925290	1925350	+	ARHGEF10	Rho guanine nucleotide exchange factor (GEF) 10 [Source:HGNC Symbol;Acc:HGNC:14103]	ENSG00000104728	protein_coding	exon	coding exon	1	812	8:1925290:1925350	ENSG00000104728	ARHGEF10	7.057163114	0.005679336	0.594604446	-3.656060759	-0.334384894	-4.250665204	-0.92898934	3.321675864	9.961963646	0.565367237	0.004445691	0.74502233	0.001702233	0.3748565	0.007765187	-6.80880046	-1.817620584	-6.986400901	-1.539246291	-5.857084918	-3.027847264	7.299475733	0.007098369

COCH	-0.138281315	0.028603493	-0.215036292	0.166884808	-0.076754977	-0.243639786	8	0.207649063	0.816496581	0.816496581	0.816496581	0.816496581	0.816496581	0.816496581	5	133	0	P_220348	P_220348	P_220348	chr14	30880663	30880723	+	COCH	cochlin [Source:HGNC Symbol;Acc:HGNC:2180]	ENSG00000100473	protein_coding	3'UTR;exon	multiple Ref	1	813	14:30880663:30880723	ENSG00000100473	COCH	3.462801716	0.036832896	-0.882452213	0.182535261	-1.372269651	1.064987474	-0.489817437	-1.554804911	9.961963646	0.398325673	0.858823825	0.200085533	0.312013257	0.634869969	0.151161042	-6.589337894	-6.164553062	-6.105827766	-6.562394985	-6.170637145	-5.889980114	1.08111713	0.401006991

PWWP3A	0.09739845	-0.076156084	0.042374637	-0.173554534	-0.055023813	0.118530721	8	0.097390347	0.816496581	0.816496581	0.816496581	0.816496581	0.816496581	0.816496581	5	135	0	P_123971	P_123971	P_123971	chr19	1373973	1374033	+	MUM1	melanoma associated antigen (mutated) 1 [Source:HGNC Symbol;Acc:HGNC:29641]	ENSG00000160953	protein_coding	exon	non-coding exon	1	815	19:1373973:1374033	ENSG00000160953	PWWP3A	7.688603965	0.00982356	1.203547504	-0.941056701	0.523621151	-2.144604205	-0.679926353	1.464677852	9.961963646	0.256584549	0.36893498	0.611991151	0.05768828	0.512041138	0.173841478	-6.260780716	-5.735646421	-6.899427056	-5.084342084	-6.056512982	-6.010756936	1.635866945	0.243090389

CCDC89	0.184553314	-0.167075765	0.227516968	-0.351629079	0.042963654	0.394592733	8	0.135103478	0.816496581	0.816496581	0.816496581	0.816496581	0.816496581	0.816496581	5	137	0	P_109743	P_109743	P_109743	chr11	85684884	85684944	-	CCDC89	coiled-coil domain containing 89 [Source:HGNC Symbol;Acc:HGNC:26762]	ENSG00000179071	protein_coding	3'UTR	3'UTR	1	817	11:85684884:85684944	ENSG00000179071	CCDC89	4.047122866	0.016864798	1.740511543	-1.575681799	2.145699258	-3.316193342	0.405187714	3.721381057	9.961963646	0.112507551	0.146293282	0.057581977	0.00783721	0.693903943	0.00399226	-5.556721124	-5.019295358	-4.983568375	-3.109302452	-6.210097085	-2.349052262	5.854361867	0.014291724

NMD3	-0.071733983	0.066614978	-0.192938646	0.13834896	-0.121204663	-0.259553624	8	0.09238125	0.816496581	0.816496581	0.816496581	0.816496581	0.816496581	0.816496581	5	138	0	P_312092	P_312092	P_312092	chr3	161118144	161118204	-	NMD3	NMD3 ribosome export adaptor [Source:HGNC Symbol;Acc:HGNC:24250]	ENSG00000169251	protein_coding	intron	intron antisense	0	818	3:161118144:161118204	ENSG00000169251	NMD3	6.102421096	0.009060189	-0.923000539	0.85713435	-2.482539901	1.780134889	-1.559539362	-3.339674251	9.961963646	0.377820337	0.411515808	0.032484824	0.105519141	0.150044794	0.007533504	-6.552435599	-5.808969809	-4.432600683	-5.646346463	-5.143712151	-2.997099899	4.0840135	0.039348723

KBTBD12	-0.675854161	-0.657784093	-0.613551192	0.018070067	0.062302969	0.044232901	8	0.582743723	0.816496581	0.816496581	0.816496581	0.816496581	0.816496581	0.816496581	5	139	0	P_135904	P_135904	P_135904	chr3	127984660	127984720	+	KBTBD12	kelch repeat and BTB (POZ) domain containing 12 [Source:HGNC Symbol;Acc:HGNC:25731]	ENSG00000187715	protein_coding	3'UTR	3'UTR	1	819	3:127984660:127984720	ENSG00000187715	KBTBD12	3.880498836	0.274916168	-1.578695896	-1.536486877	-1.433165327	0.042209019	0.14553057	0.103321551	9.961963646	0.145602053	0.155548978	0.18243883	0.967166081	0.887194476	0.919758154	-5.785727357	-5.070504402	-6.028037641	-7.148638853	-6.285432707	-7.065549693	1.1567786	0.373889508

USP54	0.481939163	-0.04893448	0.336628101	-0.530873642	-0.145311062	0.385562581	8	0.098832586	0.816496581	0.816496581	0.816496581	0.816496581	0.816496581	0.816496581	5	140	0	A_24_P914155	A_24_P914155	A_24_P914155	chr10	73530245	73530305	-	USP54	ubiquitin specific peptidase 54 [Source:HGNC Symbol;Acc:HGNC:23513]	ENSG00000166348	protein_coding	exon	coding exon	1	820	10:73530245:73530305	ENSG00000166348	USP54	7.52057001	0.010050824	5.887582522	-0.597805301	4.112398156	-6.485387823	-1.775184366	4.710203457	9.961963646	0.000155947	0.563311961	0.002119402	7.15E-05	0.106370038	0.000837291	1.066790922	-5.997129929	-1.676501357	1.771813813	-4.847626402	-0.735569965	19.88839485	0.000157793

CCNH	-0.364896037	-0.063736006	-0.328120642	0.301160031	0.036775395	-0.264384636	8	0.06386969	0.816496581	0.816496581	0.816496581	0.816496581	0.816496581	0.816496581	5	141	0	P_139902	P_139902	P_139902	chr5	87394287	87394347	-	CCNH	cyclin H [Source:HGNC Symbol;Acc:HGNC:1594]	ENSG00000134480	protein_coding	3'UTR;exon	multiple Ref	1	821	5:87394287:87394347	ENSG00000134480	CCNH	10.8953235	0.005482614	-6.035606679	-1.054233053	-5.427318843	4.981373625	0.608287835	-4.37308579	9.961963646	0.000127989	0.3166738	0.000293866	0.000558675	0.556610384	0.001405002	1.271669499	-5.627893628	0.372751952	-0.379414716	-6.103565166	-1.272473671	18.55062029	0.000211206

MACO1	0.242205988	0.138583671	0.257016624	-0.103622316	0.014810637	0.118432953	8	0.048147033	0.816496581	0.816496581	0.816496581	0.816496581	0.816496581	0.816496581	5	142	0	P_100855	P_100855	P_100855	chr1	25498487	25498547	+	TMEM57	transmembrane protein 57 [Source:HGNC Symbol;Acc:HGNC:25572]	ENSG00000204178	protein_coding	3'UTR	3'UTR	1	822	1:25498487:25498547	ENSG00000204178	MACO1	8.188276919	0.004068274	4.650775705	2.661047224	4.935165655	-1.989728481	0.284389949	2.27411843	9.961963646	0.000916214	0.023929112	0.000598105	0.074763391	0.78193534	0.046341036	-0.769103668	-3.399572675	-0.36624352	-5.328695551	-6.25384698	-4.79961844	10.37970979	0.002074355

IFI27L1	-0.120402173	0.099895512	-0.181345238	0.220297685	-0.060943064	-0.28124075	8	0.081235494	0.816496581	0.816496581	0.816496581	0.816496581	0.816496581	0.816496581	5	143	0	P_115673	P_115673	P_115673	chr14	94102615	94102675	+	IFI27L1	"interferon, alpha-inducible protein 27-like 1 [Source:HGNC Symbol;Acc:HGNC:19754]"	ENSG00000165948	protein_coding	3'UTR	3'UTR	1	823	14:94102615:94102675	ENSG00000165948	IFI27L1	10.49053477	0.007506206	-1.702039833	1.412151764	-2.563548562	3.114191597	-0.861508728	-3.975700326	9.961963646	0.119693281	0.188372179	0.028279474	0.011030664	0.409215616	0.002638798	-5.612289641	-5.227683453	-4.297046944	-3.455679581	-5.916399613	-1.923547233	6.284912183	0.011483537

OCRL	0.221617779	0.084927211	0.114609381	-0.136690567	-0.107008398	0.029682169	8	0.042149968	0.816496581	0.816496581	0.816496581	0.816496581	0.816496581	0.816496581	5	144	0	P_271470	P_271470	P_271470	chrX	129576492	129576552	+	OCRL	oculocerebrorenal syndrome of Lowe [Source:HGNC Symbol;Acc:HGNC:8108]	ENSG00000122126	protein_coding	exon	coding exon	1	824	X:129576492:129576552	ENSG00000122126	OCRL	7.008976383	0.003633407	4.502909617	1.725581582	2.328674555	-2.777328035	-2.174235062	0.603092973	9.961963646	0.001148898	0.115248327	0.042237491	0.019606134	0.054877202	0.559925884	-1.003124727	-4.816830774	-4.687177559	-4.032367388	-4.254040742	-6.876242884	6.913520374	0.008478436

SOX5-AS1	0.096881617	0.393774026	0.754462492	0.296892409	0.657580875	0.360688466	8	0.340075445	0.816496581	0.816496581	0.816496581	0.816496581	0.816496581	0.816496581	5	145	0	A_32_P212178	A_32_P212178	A_32_P212178	chr12	24227435	24227495	+	RP11-778H2.1	NA	ENSG00000256120	lincRNA	exon	non-coding exon	1	825	12:24227435:24227495	ENSG00000256120	SOX5-AS1	3.393778429	0.09508099	0.384804515	1.564032766	2.996652851	1.17922825	2.611848336	1.432620086	9.961963646	0.708469713	0.148992099	0.013474561	0.265703474	0.026033947	0.182590739	-6.918156711	-5.034594191	-3.561554252	-6.438047396	-3.561080005	-6.052605948	3.639822103	0.052506836

TPX2	-0.403649182	-0.087105081	-0.178702954	0.316544101	0.224946228	-0.091597873	8	0.132083612	0.816496581	0.816496581	0.816496581	0.816496581	0.816496581	0.816496581	5	146	0	P_240520	P_240520	P_240520	chr20	31793953	31794013	+	TPX2	"TPX2, microtubule-associated [Source:HGNC Symbol;Acc:HGNC:1249]"	ENSG00000088325	protein_coding	exon	coding exon	1	826	20:31793953:31794013	ENSG00000088325	TPX2	9.964688937	0.016216838	-3.882099101	-0.837733787	-1.718677029	3.044365314	2.163422072	-0.880943242	9.961963646	0.003070338	0.421823914	0.116536221	0.012422067	0.055887439	0.399103492	-2.01552738	-5.825089624	-5.63586918	-3.575475009	-4.270724354	-6.663228085	5.575159713	0.016555749

GPRC5C	0.057850226	0.048563238	0.232765302	-0.009286987	0.174915076	0.184202063	8	0.074837337	0.816496581	0.816496581	0.816496581	0.816496581	0.816496581	0.816496581	5	148	0	A_23_P346673	A_23_P346673	A_23_P346673	chr17	74450239	74450299	+	GPRC5C	"G protein-coupled receptor, class C, group 5, member C [Source:HGNC Symbol;Acc:HGNC:13309]"	ENSG00000170412	protein_coding	exon	non-coding exon	1	828	17:74450239:74450299	ENSG00000170412	GPRC5C	5.376007033	0.006704293	0.865314936	0.726401581	3.481668212	-0.138913355	2.616353276	2.755266631	9.961963646	0.407221396	0.484310518	0.005938283	0.892286676	0.025833776	0.02036132	-6.604509103	-5.911334394	-2.733075048	-7.139070493	-3.553815382	-3.995961962	4.642423055	0.027967425

ABCB1	0.051666421	-0.011979342	0.274438649	-0.063645763	0.222772228	0.286417991	8	0.093337071	0.816496581	0.816496581	0.816496581	0.816496581	0.816496581	0.816496581	5	149	0	P_261623	P_261623	P_261623	chr7	87549850	87549910	-	ABCB1	"ATP-binding cassette, sub-family B (MDR/TAP), member 1 [Source:HGNC Symbol;Acc:HGNC:40]"	ENSG00000085563	protein_coding	exon	coding exon	1	829	7:87549850:87549910	ENSG00000085563	ABCB1	3.850458812	0.009202741	0.659622406	-0.152939606	3.503743403	-0.812562012	2.844120997	3.656683009	9.961963646	0.524451646	0.881499336	0.005723621	0.435457172	0.017487232	0.004441128	-6.766008753	-6.169771661	-2.695559962	-6.800071629	-3.184745117	-2.458211284	5.80922033	0.014631416

MTDH	-0.208773834	0.077662515	-0.092327119	0.286436349	0.116446715	-0.169989634	8	0.068399565	0.816496581	0.816496581	0.816496581	0.816496581	0.816496581	0.816496581	5	151	0	A_23_P250196	NM_178812	A_23_P250196	chr8	97724916	97724976	+	MTDH	metadherin [Source:HGNC Symbol;Acc:HGNC:29608]	ENSG00000147649	protein_coding	3'UTR	3'UTR	1	831	8:97724916:97724976	ENSG00000147649	MTDH	10.56166581	0.005963775	-3.311014415	1.231675928	-1.464246818	4.542690343	1.846767597	-2.695922746	9.961963646	0.007905876	0.246352422	0.173956679	0.001080706	0.094658447	0.022540811	-2.979693044	-5.440000385	-5.987479355	-1.06724818	-4.74500984	-4.09666301	7.656407725	0.006055329

DPY19L3	0.006561222	0.054398774	-0.060708226	0.047837552	-0.067269448	-0.115107001	8	0.073968419	0.816496581	0.816496581	0.816496581	0.816496581	0.816496581	0.816496581	6	19	0	A_24_P254101	A_24_P254101	A_24_P254101	chr19	32485023	32485083	+	DPY19L3	dpy-19-like 3 (C. elegans) [Source:HGNC Symbol;Acc:HGNC:27120]	ENSG00000178904	protein_coding	3'UTR;exon	multiple Ref	1	869	19:32485023:32485083	ENSG00000178904	DPY19L3	6.893448267	0.006600458	0.098910716	0.820063932	-0.915179195	0.721153216	-1.014089911	-1.735243127	9.961963646	0.923171166	0.431363568	0.381716171	0.487394354	0.334536108	0.113467864	-6.993024272	-5.839494078	-6.608073177	-6.8724269	-5.777010375	-5.635551934	1.013225027	0.427167792

TRIM37	-0.108018655	0.066833711	-0.080566412	0.174852366	0.027452243	-0.147400123	8	0.034805751	0.816496581	0.816496581	0.816496581	0.816496581	0.816496581	0.816496581	6	20	0	P_229359	P_229359	P_229359	chr17	59079753	59079813	-	TRIM37	tripartite motif containing 37 [Source:HGNC Symbol;Acc:HGNC:7523]	ENSG00000108395	protein_coding	3'UTR;exon	multiple Ref	1	870	17:59079753:59079813	ENSG00000108395	TRIM37	8.447417627	0.003179537	-2.346186229	1.451641232	-1.749918161	3.797827461	0.596268068	-3.201559393	9.961963646	0.040997019	0.177354941	0.110811315	0.00352218	0.564298502	0.009511281	-4.611953091	-5.178665075	-5.59051714	-2.290708823	-6.110986504	-3.233287906	5.950512874	0.013599108

TLE4	0.013838132	0.007945757	-0.10173395	-0.005892375	-0.115572081	-0.109679706	8	0.201197226	0.816496581	0.816496581	0.816496581	0.816496581	0.816496581	0.816496581	6	21	0	A_23_P83094	A_23_P83094	A_23_P83094	chr9	79726141	79726201	+	TLE4;RP11-79D8.2	transducin-like enhancer of split 4 [Source:HGNC Symbol;Acc:HGNC:11840];	ENSG00000106829	protein_coding;processed_pseudogene	3'UTR;exon	multiple Ref	1	871	9:79726141:79726201	ENSG00000106829	TLE4	4.336138548	0.034714591	0.090963478	0.052230582	-0.668737238	-0.038732896	-0.759700717	-0.72096782	9.961963646	0.929324658	0.959377469	0.518858353	0.969868564	0.465031524	0.487503512	-6.993846956	-6.180649487	-6.808257522	-7.148792696	-5.998557089	-6.794623854	0.259441983	0.852979811

CMC2	-0.508754077	-0.02877989	-0.389499077	0.479974187	0.119255	-0.360719187	8	0.095270083	0.816496581	0.816496581	0.816496581	0.816496581	0.816496581	0.816496581	6	23	0	P_227088	P_227088	P_227088	chr16	80997313	80997373	-	CMC2	C-x(9)-C motif containing 2 [Source:HGNC Symbol;Acc:HGNC:24447]	ENSG00000103121	protein_coding	exon	coding exon	1	873	16:80997313:80997373	ENSG00000103121	CMC2	8.862822402	0.009495519	-6.394317246	-0.361722406	-4.895451021	6.032594839	1.498866225	-4.533728615	9.961963646	8.03E-05	0.725114021	0.000634366	0.000128501	0.164911711	0.001095684	1.755220635	-6.113578482	-0.427390709	1.157933754	-5.223022847	-1.014755555	20.69617874	0.000133373

WDR26	0.191248666	0.024569779	0.05539131	-0.166678887	-0.135857356	0.030821531	8	0.044941205	0.816496581	0.816496581	0.816496581	0.816496581	0.816496581	0.816496581	6	24	0	A_23_P316215	A_23_P316215	A_23_P316215	chr1	224429796	224429856	-	WDR26	WD repeat domain 26 [Source:HGNC Symbol;Acc:HGNC:21208]	ENSG00000162923	protein_coding	exon	non-coding exon	1	874	1:224429796:224429856	ENSG00000162923	WDR26	8.274441728	0.003828623	3.785495666	0.486323863	1.096392293	-3.299171803	-2.689103373	0.61006843	9.961963646	0.003593916	0.637258216	0.298703115	0.008065231	0.022805786	0.555476512	-2.176864935	-6.058923542	-6.42761015	-3.138456787	-3.436272939	-6.871785705	5.70942657	0.015417261

SLC22A18	0.136977013	0.306221249	0.309682363	0.169244236	0.17270535	0.003461114	8	0.130652788	0.816496581	0.816496581	0.816496581	0.816496581	0.816496581	0.816496581	6	26	0	P_107652	P_107652	P_107652	chr11	2920325	2920385	+	SLC22A18	"solute carrier family 22, member 18 [Source:HGNC Symbol;Acc:HGNC:10964]"	ENSG00000110628	protein_coding	exon	non-coding exon	1	876	11:2920325:2920385	ENSG00000110628	SLC22A18	9.515784649	0.015914946	1.329813501	2.972886781	3.006488304	1.643073279	1.676674802	0.033601523	9.961963646	0.213223954	0.014032224	0.013250414	0.131513838	0.124655465	0.973858628	-6.110680364	-2.900965622	-3.544735435	-5.844220174	-4.985466836	-7.070761749	4.192914822	0.03674847

SAR1B	-0.178751905	0.013090162	-0.066210043	0.191842067	0.112541862	-0.079300205	8	0.098747842	0.816496581	0.816496581	0.816496581	0.816496581	0.816496581	0.816496581	6	28	0	P_254843	P_254843	P_254843	chr5	134614477	134614537	-	SAR1B	"secretion associated, Ras related GTPase 1B [Source:HGNC Symbol;Acc:HGNC:10535]"	ENSG00000152700	protein_coding	exon	non-coding exon	1	878	5:134614477:134614537	ENSG00000152700	SAR1B	9.211093807	0.010037378	-2.185174702	0.160022304	-0.80939283	2.345197005	1.375781872	-0.969415134	9.961963646	0.053873011	0.876061659	0.437194277	0.041066135	0.199029836	0.355285184	-4.873535335	-6.168606112	-6.700710323	-4.758396473	-5.377785953	-6.580935268	2.298033803	0.139812945

MTARC2	0.031778814	0.167034916	0.15873781	0.135256101	0.126958995	-0.008297106	8	0.072961763	0.816496581	0.816496581	0.816496581	0.816496581	0.816496581	0.816496581	6	32	0	A_14_P131287	A_14_P131287	A_14_P131287	chr1	220784677	220784737	+	Mar-02	mitochondrial amidoxime reducing component 2 [Source:HGNC Symbol;Acc:HGNC:26064]	ENSG00000117791	protein_coding	3'UTR	3'UTR	1	882	1:220784677:220784737	ENSG00000117791	MTARC2	6.837736334	0.00648168	0.483436615	2.541026013	2.414805923	2.057589398	1.931369308	-0.12622009	9.961963646	0.639235267	0.029391336	0.036470478	0.066762022	0.082359032	0.90206882	-6.87231111	-3.590158515	-4.545181653	-5.22252197	-4.621323534	-7.062682086	3.416934142	0.061060303

AKR1D1	-1.010078847	0.117959058	-0.69950435	1.128037904	0.310574497	-0.817463408	8	0.17494451	0.816496581	0.816496581	0.816496581	0.816496581	0.816496581	0.816496581	6	33	0	P_146023	P_146023	P_146023	chr7	138116779	138116839	+	AKR1D1	"aldo-keto reductase family 1, member D1 [Source:HGNC Symbol;Acc:HGNC:388]"	ENSG00000122787	protein_coding	3'UTR;exon	multiple Ref	1	883	7:138116779:138116839	ENSG00000122787	AKR1D1	4.350141571	0.026784635	-7.558890273	0.882742546	-5.234716721	8.441632819	2.324173552	-6.117459267	9.961963646	1.97E-05	0.398176139	0.000386489	7.52E-06	0.042562197	0.000114888	3.204823916	-5.78720947	0.087710884	4.1198993	-4.020118887	1.332833565	33.23409516	1.68E-05

DYRK1A	0.117903947	-0.145489415	-0.004079304	-0.263393362	-0.121983251	0.141410111	8	0.06575305	0.816496581	0.816496581	0.816496581	0.816496581	0.816496581	0.816496581	6	36	0	P_241798	P_241798	P_241798	chr21	37472820	37472880	+	DYRK1A	dual-specificity tyrosine-(Y)-phosphorylation regulated kinase 1A [Source:HGNC Symbol;Acc:HGNC:3091]	ENSG00000157540	protein_coding	exon	coding exon	1	886	21:37472820:37472880	ENSG00000157540	DYRK1A	6.606770331	0.005678661	1.916244861	-2.364580234	-0.066299262	-4.280825095	-1.982544123	2.298280972	9.961963646	0.084442327	0.039732364	0.948451256	0.001623318	0.075661583	0.044477875	-5.294980675	-3.867569753	-7.044720658	-1.490007959	-4.545346431	-4.760178468	6.134278294	0.01238379

OSGIN1	-0.00755137	0.038243112	0.06878373	0.045794482	0.0763351	0.030540618	8	0.157183345	0.816496581	0.816496581	0.816496581	0.816496581	0.816496581	0.816496581	6	37	0	P_119675	P_119675	P_119675	chr16	83966133	83966193	+	OSGIN1	oxidative stress induced growth inhibitor 1 [Source:HGNC Symbol;Acc:HGNC:30093]	ENSG00000140961	protein_coding	3'UTR	3'UTR	1	887	16:83966133:83966193	ENSG00000140961	OSGIN1	6.866793181	0.022047434	-0.062286273	0.315442228	0.567351657	0.377728501	0.62963793	0.251909429	9.961963646	0.951566841	0.758934726	0.583033551	0.71355557	0.543101246	0.806231853	-6.996243677	-6.129902966	-6.874135189	-7.072143616	-6.090045541	-7.036821729	0.171308731	0.913331382

ZNF589	-0.358103355	-0.090534917	-0.444632213	0.267568439	-0.086528858	-0.354097296	8	0.077518244	0.816496581	0.816496581	0.816496581	0.816496581	0.816496581	0.816496581	6	38	0	A_23_P110031	A_23_P110031	A_23_P110031	chr3	48270678	48270738	+	ZNF589	zinc finger protein 589 [Source:HGNC Symbol;Acc:HGNC:16747]	ENSG00000164048	protein_coding	3'UTR	3'UTR	1	888	3:48270678:48270738	ENSG00000164048	ZNF589	6.164206228	0.007032301	-5.230043283	-1.322248244	-6.493783662	3.907795039	-1.263740379	-5.171535419	9.961963646	0.000389093	0.215639172	7.07E-05	0.002944967	0.235094305	0.000423322	0.118293855	-5.33593883	1.854412105	-2.106276765	-5.510870616	-0.026152057	19.14726664	0.000185063

AFF4	0.291460336	-0.011989117	0.139099654	-0.303449453	-0.152360682	0.151088771	8	0.105163586	0.816496581	0.816496581	0.816496581	0.816496581	0.816496581	0.816496581	6	39	0	P_254786	P_254786	P_254786	chr5	132881090	132881150	-	AFF4	"AF4/FMR2 family, member 4 [Source:HGNC Symbol;Acc:HGNC:17869]"	ENSG00000072364	protein_coding	exon	coding exon	1	889	5:132881090:132881150	ENSG00000072364	AFF4	8.33039991	0.011087969	3.38999524	-0.139446251	1.617877655	-3.529441491	-1.772117585	1.757323906	9.961963646	0.006922824	0.891876398	0.136876098	0.005483824	0.106900303	0.109492317	-2.845145734	-6.171846845	-5.779113283	-2.745074185	-4.851978275	-5.60336567	5.46909773	0.017526662

TTC13	-0.242015245	0.070522142	-0.106372525	0.312537387	0.13564272	-0.176894667	8	0.090997442	0.816496581	0.816496581	0.816496581	0.816496581	0.816496581	0.816496581	6	40	0	P_206639	P_206639	P_206639	chr1	230958223	230958283	-	TTC13	tetratricopeptide repeat domain 13 [Source:HGNC Symbol;Acc:HGNC:26204]	ENSG00000143643	protein_coding	exon	coding exon	1	890	1:230958223:230958283	ENSG00000143643	TTC13	6.835823393	0.008856405	-3.14963065	0.917788048	-1.384351492	4.067418698	1.765279157	-2.30213954	9.961963646	0.010386449	0.380413537	0.196473721	0.002277295	0.108091421	0.044187166	-3.255188643	-5.756552568	-6.090574124	-1.840694263	-4.861668681	-4.753868566	6.273148169	0.01155096

PPRC1	-0.257888029	0.08684232	-0.152642011	0.344730349	0.105246018	-0.239484331	8	0.04097332	0.816496581	0.816496581	0.816496581	0.816496581	0.816496581	0.816496581	6	41	0	P_209620	P_209620	P_209620	chr10	102149177	102149237	-	PPRC1;NOLC1	"peroxisome proliferator-activated receptor gamma, coactivator-related 1 [Source:HGNC Symbol;Acc:HGNC:30025];nucleolar and coiled-body phosphoprotein 1 [Source:HGNC Symbol;Acc:HGNC:15608]"	ENSG00000148840	protein_coding	exon;promoter	multiple non-Ref	0	891	10:102149177:102149237	ENSG00000148840	PPRC1	8.63112755	0.003554862	-5.297432384	1.783880071	-3.135510931	7.081312455	2.161921454	-4.919391002	9.961963646	0.000353297	0.104879565	0.010638378	3.44E-05	0.056029042	0.000612236	0.218399288	-4.735494942	-3.323994005	2.53742106	-4.273037532	-0.410166474	20.01595751	0.0001536

CRMP1	0.069337597	-0.08776087	0.013922392	-0.157098466	-0.055415205	0.101683262	8	0.102160474	0.816496581	0.816496581	0.816496581	0.816496581	0.816496581	0.816496581	6	42	0	P_248147	P_248147	P_248147	chr4	5836017	5836077	-	CRMP1	collapsin response mediator protein 1 [Source:HGNC Symbol;Acc:HGNC:2365]	ENSG00000072832	protein_coding	exon	coding exon	1	892	4:5836017:5836077	ENSG00000072832	CRMP1	4.493754294	0.010587973	0.825292685	-1.044576208	0.165711661	-1.869868893	-0.659581024	1.210287869	9.961963646	0.428525674	0.320903516	0.87169859	0.091140692	0.524477121	0.254102021	-6.638939218	-5.637470399	-7.032145006	-5.512395363	-6.070362879	-6.325167947	1.199326893	0.359529879

GMDS	-0.273398501	0.065936991	-0.185784998	0.339335492	0.087613503	-0.251721989	8	0.051237553	0.816496581	0.816496581	0.816496581	0.816496581	0.816496581	0.816496581	6	43	0	P_141469	P_141469	P_141469	chr6	1679334	1679394	-	GMDS	"GDP-mannose 4,6-dehydratase [Source:HGNC Symbol;Acc:HGNC:4369]"	ENSG00000112699	protein_coding	exon	non-coding exon	1	893	6:1679334:1679394	ENSG00000112699	GMDS	6.755936353	0.004314932	-5.097470954	1.229384554	-3.463931319	6.326855508	1.633539635	-4.693315873	9.961963646	0.000471336	0.247173412	0.006116791	8.75E-05	0.133520359	0.000858956	-0.080564546	-5.442564663	-2.763237542	1.56011302	-5.044491718	-0.762103888	17.36986382	0.000277304

MEMO1	-0.000122756	0.087464935	-0.080598235	0.08758769	-0.080475479	-0.16806317	8	0.318760788	0.816496581	0.816496581	0.816496581	0.816496581	0.816496581	0.816496581	6	44	0	P_127564	P_127564	P_127564	chr2	31867440	31867500	+	MEMO1	mediator of cell motility 1 [Source:HGNC Symbol;Acc:HGNC:14014]	ENSG00000162959	protein_coding	3'ext;3'UTR	multiple non-Ref	0	894	2:31867440:31867500	ENSG00000162959	MEMO1	4.150359281	0.083803802	-0.000519344	0.370039211	-0.340988161	0.370558555	-0.340468817	-0.711027372	9.961963646	0.999595876	0.719098953	0.740194802	0.718724003	0.74057406	0.493378631	-6.998360171	-6.110414484	-6.9839932	-7.075036746	-6.235397281	-6.802017004	0.168665799	0.915085378

ATP8A2	0.101457728	-0.027303923	-0.036344585	-0.128761651	-0.137802313	-0.009040662	8	0.113775144	0.816496581	0.816496581	0.816496581	0.816496581	0.816496581	0.816496581	6	46	0	P_218415	P_218415	P_218415	chr13	25581890	25581950	+	ATP8A2	"ATPase, aminophospholipid transporter, class I, type 8A, member 2 [Source:HGNC Symbol;Acc:HGNC:13533]"	ENSG00000132932	protein_coding	exon	coding exon	1	896	13:25581890:25581950	ENSG00000132932	ATP8A2	3.785750936	0.012602051	1.106904488	-0.297885978	-0.396519659	-1.404790466	-1.503424147	-0.098633681	9.961963646	0.294346872	0.771910106	0.700082565	0.190489258	0.163751823	0.923385585	-6.368096609	-6.135524066	-6.961932383	-6.165566724	-5.21713143	-7.066066367	0.952441744	0.452147973

ILRUN	-0.011627524	-0.044116175	-0.121618563	-0.032488651	-0.109991039	-0.077502388	8	0.06997661	0.816496581	0.816496581	0.816496581	0.816496581	0.816496581	0.816496581	6	47	0	P_142414	P_142414	P_142414	chr6	34603131	34603191	+	C6orf106	chromosome 6 open reading frame 106 [Source:HGNC Symbol;Acc:HGNC:21215]	ENSG00000196821	protein_coding	intron	intron antisense	0	897	6:34603131:34603191	ENSG00000196821	ILRUN	7.86361171	0.006139022	-0.181753708	-0.689594654	-1.901060357	-0.507840947	-1.71930665	-1.211465703	9.961963646	0.859421003	0.506194245	0.086583303	0.622619069	0.11641824	0.253670204	-6.980363763	-5.937437733	-5.365326282	-7.010380437	-4.926317223	-6.323810996	1.467362102	0.282056018

HPS5	-0.115330666	-0.08879468	-0.079345136	0.026535986	0.03598553	0.009449544	8	0.045744882	0.816496581	0.816496581	0.816496581	0.816496581	0.816496581	0.816496581	6	48	0	P_211170	P_211170	P_211170	chr11	18285345	18285405	-	HPS5	Hermansky-Pudlak syndrome 5 [Source:HGNC Symbol;Acc:HGNC:17022]	ENSG00000110756	protein_coding	exon	coding exon	1	898	11:18285345:18285405	ENSG00000110756	HPS5	7.744608935	0.003887151	-2.265555509	-1.744282623	-1.558655788	0.521272886	0.70689972	0.185626835	9.961963646	0.04701933	0.111824717	0.150252563	0.613566816	0.495831042	0.8564625	-4.743671638	-4.790888259	-5.860931564	-7.003018028	-6.037563353	-7.05258708	1.901760242	0.193512998

FRMD1	-0.021515361	0.178112686	0.123790536	0.199628047	0.145305897	-0.05432215	8	0.213068111	0.816496581	0.816496581	0.816496581	0.816496581	0.816496581	0.816496581	6	49	0	P_144054	P_144054	P_144054	chr6	168056877	168056937	-	FRMD1	FERM domain containing 1 [Source:HGNC Symbol;Acc:HGNC:21240]	ENSG00000153303	protein_coding	3'UTR;exon	multiple Ref	1	899	6:168056877:168056937	ENSG00000153303	FRMD1	5.106548248	0.038663769	-0.13401153	1.109400574	0.771047221	1.243412104	0.905058751	-0.338353353	9.961963646	0.896062147	0.293319759	0.458576343	0.242181973	0.386799356	0.742119658	-6.988569077	-5.571863252	-6.731838544	-6.364000999	-5.878551585	-7.009195228	0.720488422	0.562326678

HSPB7	0.105721891	0.004634898	0.078442417	-0.101086993	-0.027279475	0.073807518	8	0.226294654	0.816496581	0.816496581	0.816496581	0.816496581	0.816496581	0.816496581	6	50	0	P_100541	P_100541	P_100541	chr1	16014027	16014087	-	HSPB7	"heat shock 27kDa protein family, member 7 (cardiovascular) [Source:HGNC Symbol;Acc:HGNC:5249]"	ENSG00000173641	protein_coding	3'UTR;exon	multiple Ref	1	900	1:16014027:16014087	ENSG00000173641	HSPB7	4.589494861	0.04333052	0.622033252	0.027270236	0.461529687	-0.594763016	-0.160503565	0.434259452	9.961963646	0.547891351	0.978782755	0.654331928	0.565265321	0.875692418	0.67335493	-6.791248065	-6.1816954	-6.932029164	-6.959538544	-6.28300139	-6.969325706	0.194794066	0.897549115

ERAP1	0.499322698	0.187154187	0.581627882	-0.312168511	0.082305184	0.394473694	8	0.079144173	0.816496581	0.816496581	0.816496581	0.816496581	0.816496581	0.816496581	6	51	0	P_254053	P_254053	P_254053	chr5	96780422	96780482	-	ERAP1	endoplasmic reticulum aminopeptidase 1 [Source:HGNC Symbol;Acc:HGNC:18173]	ENSG00000164307	protein_coding	exon	coding exon	1	901	5:96780422:96780482	ENSG00000164307	ERAP1	5.831026705	0.007236857	7.188726205	2.694450334	8.373670195	-4.49427587	1.184943991	5.679219861	9.961963646	3.03E-05	0.022597761	8.07E-06	0.001164291	0.263537357	0.000206996	2.763221839	-3.346339877	4.097735829	-1.144749626	-5.599619398	0.719261378	30.48539095	2.47E-05

MIA3	0.341526065	-0.093073638	0.174649795	-0.434599703	-0.16687627	0.267723433	8	0.054593371	0.816496581	0.816496581	0.816496581	0.816496581	0.816496581	0.816496581	6	53	0	P_104854	P_104854	P_104854	chr1	222662184	222662244	-	MIA3;RP11-378J18.8	"melanoma inhibitory activity family, member 3 [Source:HGNC Symbol;Acc:HGNC:24008];"	ENSG00000154305	protein_coding;antisense	intron;promoter	multiple non-Ref	0	903	1:222662184:222662244	ENSG00000154305	MIA3	9.292868252	0.004600137	6.167144375	-1.680687418	3.153757823	-7.847831793	-3.013386552	4.834445241	9.961963646	0.000107646	0.123858324	0.010313969	1.42E-05	0.013095472	0.000694703	1.451098765	-4.878513673	-3.292772601	3.455834545	-2.909314394	-0.541568929	24.14089859	6.90E-05

RAD54B	-0.33653373	-0.004796533	-0.258140769	0.331737197	0.078392962	-0.253344236	8	0.076296697	0.816496581	0.816496581	0.816496581	0.816496581	0.816496581	0.816496581	6	55	0	P_314157	P_314157	P_314157	chr8	94375988	94376048	-	RAD54B;FSBP	RAD54 homolog B (S. cerevisiae) [Source:HGNC Symbol;Acc:HGNC:17228];fibrinogen silencer binding protein [Source:HGNC Symbol;Acc:HGNC:43653]	ENSG00000197275	protein_coding	3'ext;intron;promoter	multiple non-Ref	0	905	8:94375988:94376048	ENSG00000197275	RAD54B	8.588039305	0.006881414	-4.968615542	-0.070816457	-3.811214508	4.897799085	1.157401034	-3.740398051	9.961963646	0.000569276	0.944945313	0.003445996	0.000632159	0.274105897	0.003869516	-0.27627866	-6.179444367	-2.176583649	-0.508408222	-5.629643227	-2.317031281	13.08935033	0.000861805

ZNF774	0.277214249	0.084640466	0.240762329	-0.192573782	-0.03645192	0.156121863	8	0.073954958	0.816496581	0.816496581	0.816496581	0.816496581	0.816496581	0.816496581	6	56	0	A_23_P65823	A_23_P65823	A_23_P65823	chr15	90348004	90348064	-	ZNF774;GABARAPL3	"zinc finger protein 774 [Source:HGNC Symbol;Acc:HGNC:33108];GABA(A) receptors associated protein like 3, pseudogene [Source:EntrezGene;Acc:23766]"	ENSG00000196391	protein_coding;TEC	exon;promoter	multiple non-Ref	0	906	15:90348004:90348064	ENSG00000196391	ZNF774	6.424371002	0.006598859	4.179523398	1.276113371	3.629942516	-2.903410027	-0.549580882	2.353829146	9.961963646	0.001904845	0.23086398	0.004641715	0.015800587	0.594711903	0.040466835	-1.524827059	-5.389595245	-2.481687075	-3.817020983	-6.138501309	-4.669044722	7.757617404	0.00579369

GFM2	0.038728313	0.055622831	0.021552796	0.016894519	-0.017175516	-0.034070035	8	0.034049625	0.816496581	0.816496581	0.816496581	0.816496581	0.816496581	0.816496581	6	57	0	P_253381	P_253381	P_253381	chr5	74733021	74733081	-	GFM2	"G elongation factor, mitochondrial 2 [Source:HGNC Symbol;Acc:HGNC:29682]"	ENSG00000164347	protein_coding	exon	coding exon	1	907	5:74733021:74733081	ENSG00000164347	GFM2	8.302010653	0.003137727	0.846772284	1.216161216	0.471239499	0.369388933	-0.375532785	-0.744921717	9.961963646	0.416999846	0.251954604	0.647619894	0.719568545	0.715136746	0.473526633	-6.620636137	-5.457295172	-6.92718952	-7.075503583	-6.222219475	-6.776429766	0.541753248	0.664581699

LRRC45	-0.432317446	0.057055481	-0.226382922	0.489372928	0.205934524	-0.283438403	8	0.095656724	0.816496581	0.816496581	0.816496581	0.816496581	0.816496581	0.816496581	6	58	0	P_122982	P_122982	P_122982	chr17	82031090	82031150	+	LRRC45	leucine rich repeat containing 45 [Source:HGNC Symbol;Acc:HGNC:28302]	ENSG00000169683	protein_coding	3'UTR	3'UTR	1	908	17:82031090:82031150	ENSG00000169683	LRRC45	10.34410809	0.009554801	-5.41673483	0.714878419	-2.836471832	6.131613249	2.580262998	-3.551350251	9.961963646	0.000298283	0.491097349	0.017717693	0.000112773	0.027481417	0.005287618	0.393998916	-5.919637775	-3.835024388	1.29461808	-3.611962611	-2.636598506	15.79402777	0.000408824

EFNA5	-0.438705697	-0.125138375	-0.35460543	0.313567322	0.084100267	-0.229467054	8	0.132534185	0.816496581	0.816496581	0.816496581	0.816496581	0.816496581	0.816496581	6	59	0	A_16_P17246700	A_16_P17246700	A_16_P17246700	chr5	107440772	107440832	+	EFNA5	ephrin-A5 [Source:HGNC Symbol;Acc:HGNC:3225]	ENSG00000184349	protein_coding	intron	intron antisense	0	909	5:107440772:107440832	ENSG00000184349	EFNA5	7.520416219	0.016312586	-4.20685456	-1.199982012	-3.400396847	3.006872548	0.806457714	-2.200414835	9.961963646	0.001824152	0.257905596	0.006803098	0.013241735	0.438807194	0.052503501	-1.480234496	-5.475160109	-2.871416057	-3.639774242	-5.961942953	-4.91910251	7.538960474	0.006376907

TOP2A	-0.274797689	-0.176537348	-0.274088349	0.09826034	0.00070934	-0.097551001	8	0.063241956	0.816496581	0.816496581	0.816496581	0.816496581	0.816496581	0.816496581	6	61	0	P_228664	P_228664	P_228664	chr17	40411353	40411413	-	TOP2A	topoisomerase (DNA) II alpha 170kDa [Source:HGNC Symbol;Acc:HGNC:11989]	ENSG00000131747	protein_coding	exon	coding exon	1	911	17:40411353:40411413	ENSG00000131747	TOP2A	10.34166403	0.005418537	-4.572120803	-2.93725208	-4.560318714	1.634868724	0.01180209	-1.623066634	9.961963646	0.001033023	0.014912631	0.001051871	0.133238998	0.990816523	0.135756068	-0.893221119	-2.958036007	-0.952123484	-5.855785206	-6.296611171	-5.795609092	9.272587587	0.003120718

XRCC5	-0.002745718	-0.043859275	-0.050889034	-0.041113557	-0.048143316	-0.007029759	8	0.028543324	0.816496581	0.816496581	0.816496581	0.816496581	0.816496581	0.816496581	6	62	0	P_239261	P_239261	P_239261	chr2	216132327	216132387	+	XRCC5	X-ray repair complementing defective repair in Chinese hamster cells 5 (double-strand-break rejoining) [Source:HGNC Symbol;Acc:HGNC:12833]	ENSG00000079246	protein_coding	exon	coding exon	1	912	2:216132327:216132387	ENSG00000079246	XRCC5	11.41585053	0.00286095	-0.062870438	-1.004273429	-1.16523824	-0.94140299	-1.102367802	-0.160964811	9.961963646	0.951113255	0.339016483	0.271065079	0.368766051	0.296220801	0.875338561	-6.996203796	-5.676678392	-6.352330213	-6.685440353	-5.688023665	-7.057242487	0.749610333	0.547127763

GH2	-0.135708963	0.546935736	0.150678655	0.682644699	0.286387617	-0.396257081	8	0.315915084	0.816496581	0.816496581	0.816496581	0.816496581	0.816496581	0.816496581	6	63	0	A_24_P216014	A_24_P216014	A_24_P216014	chr17	63881028	63881087	-	GH2	growth hormone 2 [Source:HGNC Symbol;Acc:HGNC:4262]	ENSG00000136487	protein_coding	exon	coding exon	1	913	17:63881028:63881087	ENSG00000136487	GH2	4.005219404	0.082353405	-0.579179764	2.334216581	0.643067533	2.913396346	1.222247297	-1.691149048	9.961963646	0.575329571	0.041841002	0.534701306	0.015533131	0.249744858	0.12180159	-6.818347412	-3.914870114	-6.825879855	-3.799929262	-5.558123437	-5.699171804	3.173217321	0.072379372

DGKA	0.049803518	0.101977729	-0.050372012	0.052174212	-0.10017553	-0.152349742	8	0.149846772	0.816496581	0.816496581	0.816496581	0.816496581	0.816496581	0.816496581	6	64	0	P_111855	P_111855	P_111855	chr12	55942023	55942083	+	DGKA	"diacylglycerol kinase, alpha 80kDa [Source:HGNC Symbol;Acc:HGNC:2849]"	ENSG00000065357	protein_coding	3'UTR;exon	multiple Ref	1	914	12:55942023:55942083	ENSG00000065357	DGKA	5.950950103	0.020238515	0.428762044	0.87793356	-0.433656255	0.449171516	-0.862418299	-1.311589815	9.961963646	0.677219807	0.400658025	0.673778515	0.662921589	0.408738447	0.219080474	-6.898963831	-5.791337446	-6.94538478	-7.04038904	-5.915625404	-6.204969932	0.634741698	0.609456468

GLS2	-0.007907519	-0.134915991	-0.098175922	-0.127008472	-0.090268403	0.036740069	8	0.077483834	0.816496581	0.816496581	0.816496581	0.816496581	0.816496581	0.816496581	6	65	0	P_111939	P_111939	P_111939	chr12	56471028	56471088	-	GLS2	"glutaminase 2 (liver, mitochondrial) [Source:HGNC Symbol;Acc:HGNC:29570]"	ENSG00000135423	protein_coding	3'UTR;exon	multiple Ref	1	915	12:56471028:56471088	ENSG00000135423	GLS2	7.421025399	0.007028018	-0.115523258	-1.971027201	-1.434280785	-1.855503942	-1.318757526	0.536746416	9.961963646	0.910325849	0.077122656	0.182128391	0.093313592	0.216761242	0.603222572	-6.991082629	-4.465972513	-6.026591887	-5.534055416	-5.44649614	-6.916238923	1.904261371	0.193105648

HORMAD1	-0.023913111	-0.107882271	0.001328598	-0.08396916	0.025241709	0.109210869	8	0.26329404	0.816496581	0.816496581	0.816496581	0.816496581	0.816496581	0.816496581	6	66	0	P_103054	P_103054	P_103054	chr1	150704117	150704177	-	HORMAD1	HORMA domain containing 1 [Source:HGNC Symbol;Acc:HGNC:25245]	ENSG00000143452	protein_coding	exon	coding exon	1	916	1:150704117:150704177	ENSG00000143452	HORMAD1	3.368358258	0.057877436	-0.121738254	-0.549213327	0.006763704	-0.427475072	0.128501958	0.555977031	9.961963646	0.905526978	0.594954748	0.994736916	0.678126021	0.900308964	0.590494338	-6.990279107	-6.025532344	-7.047095619	-7.050592391	-6.287909857	-6.905095486	0.137476563	0.935340229

CNNM1	-0.334511964	-0.016875206	-0.215817267	0.317636757	0.118694697	-0.19894206	8	0.092600321	0.816496581	0.816496581	0.816496581	0.816496581	0.816496581	0.816496581	6	67	0	A_23_P46947	A_23_P46947	A_23_P46947	chr10	99393794	99393854	+	CNNM1	cyclin and CBS domain divalent metal cation transport mediator 1 [Source:HGNC Symbol;Acc:HGNC:102]	ENSG00000119946	protein_coding	3'UTR	3'UTR	1	917	10:99393794:99393854	ENSG00000119946	CNNM1	5.390947848	0.009092732	-4.296453325	-0.216744226	-2.771945144	4.0797091	1.524508181	-2.555200919	9.961963646	0.001583954	0.832781913	0.019787759	0.00223296	0.158478414	0.028686617	-1.334679609	-6.157382568	-3.944827904	-1.820357265	-5.189736563	-4.334058836	8.614562065	0.004045149

SACS	-0.298419352	-0.033306813	-0.352688247	0.265112539	-0.054268895	-0.319381434	8	0.062196491	0.816496581	0.816496581	0.816496581	0.816496581	0.816496581	0.816496581	6	68	0	P_218277	P_218277	P_218277	chr13	23358334	23358394	-	SACS	sacsin molecular chaperone [Source:HGNC Symbol;Acc:HGNC:10519]	ENSG00000151835	protein_coding	5'UTR;exon	multiple Ref	1	918	13:23358334:23358394	ENSG00000151835	SACS	8.204241443	0.005313223	-5.014106962	-0.559628326	-5.925944755	4.454478636	-0.911837793	-5.366316429	9.961963646	0.000532425	0.588093862	0.000148121	0.001238125	0.383389194	0.000320323	-0.206910939	-6.019634116	1.085715992	-1.208684613	-5.872518264	0.264246791	18.34040897	0.000221458

CNGB3	-0.38962229	-0.113496033	-0.677375466	0.276126257	-0.287753176	-0.563879432	8	0.106661898	0.816496581	0.816496581	0.816496581	0.816496581	0.816496581	0.816496581	6	71	0	P_265281	P_265281	P_265281	chr8	86670965	86671025	-	CNGB3	cyclic nucleotide gated channel beta 3 [Source:HGNC Symbol;Acc:HGNC:2153]	ENSG00000170289	protein_coding	exon	coding exon	1	921	8:86670965:86671025	ENSG00000170289	CNGB3	4.554303488	0.011342843	-4.480520449	-1.305164799	-7.789581614	3.175355651	-3.309061165	-6.484416816	9.961963646	0.001189269	0.221176858	1.52E-05	0.009943036	0.007931934	7.16E-05	-1.038813616	-5.355961856	3.446937905	-3.350739111	-2.429160372	1.825723934	24.25608852	6.76E-05

ASPM	-0.494324013	-0.104868393	-0.415758262	0.38945562	0.078565751	-0.310889869	8	0.060302551	0.816496581	0.816496581	0.816496581	0.816496581	0.816496581	0.816496581	6	73	0	P_205632	P_205632	P_205632	chr1	197089929	197089989	-	ASPM	"asp (abnormal spindle) homolog, microcephaly associated (Drosophila) [Source:HGNC Symbol;Acc:HGNC:19048]"	ENSG00000066279	protein_coding	exon	coding exon	1	923	1:197089929:197089989	ENSG00000066279	ASPM	8.163039906	0.00512691	-8.455309475	-1.793752066	-7.111458635	6.661557409	1.343850841	-5.317706568	9.961963646	7.41E-06	0.103210679	3.32E-05	5.73E-05	0.208802293	0.00034323	4.206107445	-4.721589547	2.640424652	2.003054864	-5.416504305	0.192287711	33.29048901	1.67E-05

CDH26	0.224683786	-0.109557537	0.211099852	-0.334241323	-0.013583934	0.320657389	8	0.037019917	0.816496581	0.816496581	0.816496581	0.816496581	0.816496581	0.816496581	6	74	0	P_241262	P_241262	P_241262	chr20	59995890	59995950	+	CDH26	cadherin 26 [Source:HGNC Symbol;Acc:HGNC:15902]	ENSG00000124215	protein_coding	exon	coding exon	1	924	20:59995890:59995950	ENSG00000124215	CDH26	8.343089769	0.00330725	4.785021358	-2.333213101	4.495728499	-7.118234459	-0.289292859	6.828941601	9.961963646	0.000748061	0.041912524	0.001161686	3.29E-05	0.778288358	4.66E-05	-0.559223555	-3.916430781	-1.055007792	2.583329802	-6.252363598	2.271671316	24.32321385	6.68E-05

FHOD3	0.036615339	-0.166768316	0.142372995	-0.203383655	0.105757656	0.309141311	8	0.086905785	0.816496581	0.816496581	0.816496581	0.816496581	0.816496581	0.816496581	6	75	0	P_231231	P_231231	P_231231	chr18	36512483	36512543	+	FHOD3	formin homology 2 domain containing 3 [Source:HGNC Symbol;Acc:HGNC:26178]	ENSG00000134775	protein_coding	exon	coding exon	1	925	18:36512483:36512543	ENSG00000134775	FHOD3	5.099841142	0.008271846	0.493068744	-2.24573213	1.917220354	-2.738800874	1.42415161	4.162952484	9.961963646	0.63265128	0.048626738	0.084306488	0.020943943	0.184964066	0.001955593	-6.867299371	-4.051806266	-5.340722564	-4.097963864	-5.317987428	-1.614470257	5.970262481	0.013461856

RYR2	0.04231307	-0.0398496	-0.033010622	-0.08216267	-0.075323692	0.006838978	8	0.04234235	0.816496581	0.816496581	0.816496581	0.816496581	0.816496581	0.816496581	6	76	0	P_206955	P_206955	P_206955	chr1	237643326	237643386	+	RYR2	ryanodine receptor 2 (cardiac) [Source:HGNC Symbol;Acc:HGNC:10484]	ENSG00000198626	protein_coding	exon	coding exon	1	926	1:237643326:237643386	ENSG00000198626	RYR2	5.243029868	0.00364646	0.858191946	-0.808227961	-0.669520088	-1.666419907	-1.527712034	0.138707873	9.961963646	0.410958877	0.437833927	0.518379625	0.126713707	0.157690208	0.892444886	-6.610739751	-5.848993645	-6.807710177	-5.81113152	-5.185553434	-7.060877923	1.161347041	0.372317791

EYS	-0.099781906	-0.03909804	-0.009118654	0.060683866	0.090663252	0.029979386	8	0.127945454	0.816496581	0.816496581	0.816496581	0.816496581	0.816496581	0.816496581	6	77	0	P_257631	P_257631	P_257631	chr6	64822650	64822710	-	EYS	eyes shut homolog (Drosophila) [Source:HGNC Symbol;Acc:HGNC:21555]	ENSG00000188107	protein_coding	exon	coding exon	1	927	6:64822650:64822710	ENSG00000188107	EYS	3.965825649	0.015352718	-0.986290353	-0.38646305	-0.090132978	0.599827303	0.896157374	0.296330072	9.961963646	0.347339309	0.707279808	0.929967998	0.56201578	0.391309554	0.773063674	-6.492071848	-6.103961209	-7.04268585	-6.956345218	-5.88641611	-7.023618469	0.396824774	0.758234257

PHF11	0.126930097	0.240106889	0.127303331	0.113176792	0.000373234	-0.112803558	8	0.215442143	0.816496581	0.816496581	0.816496581	0.816496581	0.816496581	0.816496581	6	78	0	P_219175	P_219175	P_219175	chr13	49522047	49522107	+	PHF11	PHD finger protein 11 [Source:HGNC Symbol;Acc:HGNC:17024]	ENSG00000136147	protein_coding	exon	coding exon	1	928	13:49522047:49522107	ENSG00000136147	PHF11	3.91794267	0.039480714	0.782379999	1.479986482	0.784680561	0.697606483	0.002300563	-0.695305921	9.961963646	0.452187243	0.169792516	0.450897382	0.501380003	0.998209835	0.502759517	-6.674272993	-5.142953072	-6.720923874	-6.889812153	-6.296682442	-6.813520309	0.731385409	0.55659189

UIMC1	0.015933231	-0.032958049	-0.121533322	-0.04889128	-0.137466552	-0.088575273	8	0.055348336	0.816496581	0.816496581	0.816496581	0.816496581	0.816496581	0.816496581	6	79	0	P_255809	P_255809	P_255809	chr5	176943337	176943397	-	UIMC1	ubiquitin interaction motif containing 1 [Source:HGNC Symbol;Acc:HGNC:30298]	ENSG00000087206	protein_coding	3'UTR;exon	multiple Ref	1	929	5:176943337:176943397	ENSG00000087206	UIMC1	8.841476504	0.004666792	0.285653963	-0.590878112	-2.17887229	-0.876532075	-2.464526253	-1.587994178	9.961963646	0.780994581	0.567765164	0.054449354	0.40138334	0.033500854	0.143487824	-6.954015677	-6.001315918	-4.930434143	-6.744958641	-3.797513853	-5.844396269	2.423690537	0.126580931

LINC01524	-0.207325072	-0.188174962	-0.532667218	0.01915011	-0.325342146	-0.344492256	8	0.478455186	0.816496581	0.816496581	0.816496581	0.816496581	0.816496581	0.816496581	6	80	0	A_32_P82701	A_32_P82701	A_32_P82701	chr20	52609102	52609162	+	LINC01524	long intergenic non-protein coding RNA 1524 [Source:HGNC Symbol;Acc:HGNC:51228]	ENSG00000234948	lincRNA	intron	intron sense	0	930	20:52609102:52609162	ENSG00000234948	LINC01524	3.196185964	0.186041416	-0.588698101	-0.534321497	-1.512504867	0.054376604	-0.923806766	-0.97818337	9.961963646	0.569170627	0.604837658	0.16146214	0.957710166	0.377420368	0.351140137	-6.812483773	-6.03378502	-5.923408917	-7.147996322	-5.861773696	-6.572422647	0.788825725	0.527298397

GNAS	0.049972914	-0.09792351	-0.071909333	-0.147896425	-0.121882248	0.026014177	8	0.046652503	0.816496581	0.816496581	0.816496581	0.816496581	0.816496581	0.816496581	6	81	0	P_241205	P_241205	P_241205	chr20	58894343	58894403	+	GNAS	GNAS complex locus [Source:HGNC Symbol;Acc:HGNC:4392]	ENSG00000087460	protein_coding	intron;promoter	multiple non-Ref	0	931	20:58894343:58894403	ENSG00000087460	GNAS	8.506839213	0.003954497	0.973273031	-1.907159366	-1.400506966	-2.880432398	-2.373779998	0.5066524	9.961963646	0.353457042	0.085717347	0.191730466	0.016433814	0.039114238	0.623423291	-6.504758016	-4.559289937	-6.070036852	-3.856330825	-3.941792116	-6.932933105	3.455725989	0.059458936

MYO1A	0.052534585	0.024560634	-0.011904555	-0.027973952	-0.06443914	-0.036465188	8	0.159146376	0.816496581	0.816496581	0.816496581	0.816496581	0.816496581	0.816496581	6	82	0	P_215785	P_215785	P_215785	chr12	57046577	57046637	-	MYO1A	myosin IA [Source:HGNC Symbol;Acc:HGNC:7595]	ENSG00000166866	protein_coding	exon	coding exon	1	932	12:57046577:57046637	ENSG00000166866	MYO1A	4.129037146	0.022546103	0.428504312	0.200331596	-0.097100853	-0.228172716	-0.525605165	-0.297432449	9.961963646	0.677401244	0.845251838	0.924572078	0.824127249	0.610661512	0.772246298	-6.899082198	-6.160974856	-7.041974014	-7.121216254	-6.151812879	-7.023264044	0.108327818	0.953284803

CCNA2	-0.648116487	0.008852668	-0.447487292	0.656969155	0.200629195	-0.45633996	8	0.063544857	0.816496581	0.816496581	0.816496581	0.816496581	0.816496581	0.816496581	6	83	0	A_23_P58321	NM_001237	A_23_P58321	chr4	121818839	121818899	-	CCNA2	cyclin A2 [Source:HGNC Symbol;Acc:HGNC:1578]	ENSG00000145386	protein_coding	exon	coding exon	1	933	4:121818839:121818899	ENSG00000145386	CCNA2	10.79824285	0.005449377	-10.75289163	0.146874488	-7.424255443	10.89976612	3.328636185	-7.571129931	9.961963646	8.41E-07	0.886160929	2.30E-05	7.42E-07	0.007674732	1.94E-05	6.394727973	-6.170728026	3.019518569	6.496106479	-2.397504313	3.179682525	59.66209134	1.14E-06

LYPLAL1	-0.327546112	0.072139488	-0.230652536	0.399685601	0.096893576	-0.302792025	8	0.056262337	0.816496581	0.816496581	0.816496581	0.816496581	0.816496581	0.816496581	6	84	0	P_104817	P_104817	P_104817	chr1	219212465	219212525	+	LYPLAL1	lysophospholipase-like 1 [Source:HGNC Symbol;Acc:HGNC:20440]	ENSG00000143353	protein_coding	3'UTR;exon	multiple Ref	1	934	1:219212465:219212525	ENSG00000143353	LYPLAL1	9.282457873	0.004748713	-5.821435627	1.282126005	-4.099358357	7.103561632	1.72207727	-5.381484362	9.961963646	0.000170506	0.228830931	0.002163955	3.35E-05	0.115900357	0.000313514	0.97421758	-5.38267787	-1.69795263	2.565106485	-4.922446017	0.286630474	22.45403512	9.42E-05

POLE2	-0.413334726	0.102420819	-0.277679798	0.515755545	0.135654928	-0.380100617	8	0.056833603	0.816496581	0.816496581	0.816496581	0.816496581	0.816496581	0.816496581	6	85	0	P_220647	P_220647	P_220647	chr14	49663314	49663374	-	POLE2	"polymerase (DNA directed), epsilon 2, accessory subunit [Source:HGNC Symbol;Acc:HGNC:9178]"	ENSG00000100479	protein_coding	exon	coding exon	1	935	14:49663314:49663374	ENSG00000100479	POLE2	8.735327356	0.004800597	-7.306340354	1.810448807	-4.908426474	9.116789161	2.39791388	-6.718875281	9.961963646	2.64E-05	0.100443435	0.000622268	3.79E-06	0.037537099	5.34E-05	2.90540324	-4.697986649	-0.407388348	4.828958056	-3.903540347	2.130940226	35.79368422	1.20E-05

DNTTIP1	0.098843861	0.061751649	0.100040556	-0.037092212	0.001196695	0.038288907	8	0.063689155	0.816496581	0.816496581	0.816496581	0.816496581	0.816496581	0.816496581	6	86	0	P_240964	P_240964	P_240964	chr20	45795373	45795433	+	DNTTIP1	"deoxynucleotidyltransferase, terminal, interacting protein 1 [Source:HGNC Symbol;Acc:HGNC:16160]"	ENSG00000101457	protein_coding	exon	coding exon	1	936	20:45795373:45795433	ENSG00000101457	DNTTIP1	10.13724759	0.005464121	1.637703138	1.023137583	1.657530689	-0.614565555	0.019827552	0.634393106	9.961963646	0.132640736	0.330445831	0.128522505	0.552618588	0.984572445	0.540118306	-5.703684436	-5.65848045	-5.723342698	-6.946908858	-6.29647616	-6.855871745	1.209471439	0.356196919

MYO9A	0.049335518	-0.053826795	0.022988746	-0.103162313	-0.026346772	0.076815541	8	0.066735568	0.816496581	0.816496581	0.816496581	0.816496581	0.816496581	0.816496581	6	87	0	P_224401	P_224401	P_224401	chr15	71904925	71904985	-	MYO9A	myosin IXA [Source:HGNC Symbol;Acc:HGNC:7608]	ENSG00000066933	protein_coding	exon	coding exon	1	937	15:71904925:71904985	ENSG00000066933	MYO9A	7.011595398	0.005783197	0.794550205	-0.86688238	0.370234537	-1.661432585	-0.424315668	1.237116917	9.961963646	0.445391126	0.406402101	0.718957925	0.127725703	0.680352983	0.244411783	-6.664420523	-5.800750909	-6.972777417	-5.818222065	-6.201805889	-6.294017736	0.998454831	0.433099273

PMFBP1	-0.006117265	-0.021261312	-0.136828519	-0.015144048	-0.130711254	-0.115567207	8	0.141472912	0.816496581	0.816496581	0.816496581	0.816496581	0.816496581	0.816496581	6	88	0	P_226979	P_226979	P_226979	chr16	72128656	72128716	-	PMFBP1	polyamine modulated factor 1 binding protein 1 [Source:HGNC Symbol;Acc:HGNC:17728]	ENSG00000118557	protein_coding	3'UTR;exon	multiple Ref	1	938	16:72128656:72128716	ENSG00000118557	PMFBP1	4.632028724	0.018279487	-0.055414177	-0.19259885	-1.239482084	-0.137184673	-1.184067907	-1.046883234	9.961963646	0.956904123	0.851142808	0.243572074	0.89361782	0.263868454	0.319889173	-6.996684904	-6.162570188	-6.267286235	-7.139331003	-5.60058251	-6.503568399	0.682213638	0.582923466

MLH3	-0.028695314	0.019495244	-0.051243395	0.048190558	-0.022548081	-0.070738639	8	0.069371633	0.816496581	0.816496581	0.816496581	0.816496581	0.816496581	0.816496581	6	90	0	P_115387	P_115387	P_115387	chr14	75017017	75017077	-	MLH3	mutL homolog 3 [Source:HGNC Symbol;Acc:HGNC:7128]	ENSG00000119684	protein_coding	3'UTR;exon	multiple Ref	1	940	14:75017017:75017077	ENSG00000119684	MLH3	7.974374431	0.006071322	-0.451039905	0.306430969	-0.805456108	0.757470874	-0.354416203	-1.111887078	9.961963646	0.661619613	0.765585209	0.439358504	0.466306954	0.730414433	0.292299355	-6.888473803	-6.132827574	-6.703967235	-6.844597581	-6.230306122	-6.434995829	0.48029372	0.703227816

SP140L	-0.071495953	-0.073466352	0.032033584	-0.001970399	0.103529536	0.105499936	8	0.078266978	0.816496581	0.816496581	0.816496581	0.816496581	0.816496581	0.816496581	6	91	0	P_130099	P_130099	P_130099	chr2	230402924	230402984	+	SP140L	SP140 nuclear body protein-like [Source:HGNC Symbol;Acc:HGNC:25105]	ENSG00000185404	protein_coding	3'UTR;exon	multiple Ref	1	941	2:230402924:230402984	ENSG00000185404	SP140L	4.133787128	0.007125971	-1.037301633	-1.065889244	0.464760412	-0.028587611	1.502062045	1.530649655	9.961963646	0.324117871	0.311624851	0.652095017	0.977758089	0.164097701	0.156970516	-6.441036994	-5.616241488	-6.930429585	-7.149166154	-5.218893192	-5.922788801	1.171335467	0.368906688

CILK1	0.115857193	0.133799506	0.238113247	0.017942314	0.122256054	0.104313741	8	0.067808949	0.816496581	0.816496581	0.816496581	0.816496581	0.816496581	0.816496581	6	92	0	P_257392	P_257392	P_257392	chr6	53016122	53016182	-	ICK	intestinal cell (MAK-like) kinase [Source:HGNC Symbol;Acc:HGNC:21219]	ENSG00000112144	protein_coding	exon	coding exon	1	942	6:53016122:53016182	ENSG00000112144	CILK1	6.45841665	0.005899172	1.847451781	2.133558827	3.796939416	0.286107046	1.949487635	1.663380589	9.961963646	0.094552482	0.058771081	0.003527296	0.780657458	0.079926663	0.127329566	-5.398907126	-4.223159741	-2.200508711	-7.105032097	-4.594519617	-5.738774515	4.838515757	0.024935433

ZNF652	-0.084411324	-0.191367618	-0.162721693	-0.106956294	-0.078310369	0.028645925	8	0.100015159	0.816496581	0.816496581	0.816496581	0.816496581	0.816496581	0.816496581	6	94	0	P_229123	P_229123	P_229123	chr17	49311941	49312001	-	ZNF652	zinc finger protein 652 [Source:HGNC Symbol;Acc:HGNC:29147]	ENSG00000198740	protein_coding	exon	coding exon	1	944	17:49311941:49312001	ENSG00000198740	ZNF652	8.571650685	0.010239664	-1.021653172	-2.31617423	-1.969464827	-1.294521058	-0.947811655	0.346709403	9.961963646	0.331114328	0.043145288	0.077322895	0.224686275	0.365649786	0.736021648	-6.456914394	-3.942904811	-5.260541638	-6.302999291	-5.83987238	-7.006104623	2.163594848	0.155804311

JMY	0.065178949	-0.141907982	-0.241398414	-0.20708693	-0.306577363	-0.099490432	8	0.069350339	0.816496581	0.816496581	0.816496581	0.816496581	0.816496581	0.816496581	6	95	0	A_32_P176550	A_32_P176550	A_32_P176550	chr5	79321985	79322045	+	JMY	"junction mediating and regulatory protein, p53 cofactor [Source:HGNC Symbol;Acc:HGNC:28916]"	ENSG00000152409	protein_coding	3'UTR	3'UTR	1	945	5:79321985:79322045	ENSG00000152409	JMY	8.931925545	0.00606895	1.024698714	-2.230979929	-3.79510025	-3.255678643	-4.819798964	-1.564120321	9.961963646	0.329743872	0.049857338	0.003537915	0.008679396	0.000710079	0.148971651	-6.453839554	-4.074493561	-2.203592494	-3.212988885	-0.109592085	-5.877244225	9.451073779	0.002915188

FAM177A1	-0.060958358	0.097023884	-0.085454191	0.157982243	-0.024495833	-0.182478076	8	0.040201586	0.816496581	0.816496581	0.816496581	0.816496581	0.816496581	0.816496581	6	97	0	A_32_P18357	A_32_P18357	A_32_P18357	chr14	35110568	35110628	+	FAM177A1	"family with sequence similarity 177, member A1 [Source:HGNC Symbol;Acc:HGNC:19829]"	ENSG00000151327	protein_coding	3'UTR;exon	multiple Ref	1	947	14:35110568:35110628	ENSG00000151327	FAM177A1	10.00256348	0.003504555	-1.261137604	2.0072796	-1.767919894	3.268417203	-0.50678229	-3.775199493	9.961963646	0.235992222	0.072611226	0.107630027	0.008494691	0.623335376	0.003654979	-6.193658062	-4.412445974	-5.564189483	-3.191154348	-6.161870473	-2.258511919	5.656115119	0.015857736

SPAG17	-0.302450206	0.033460185	-0.260443985	0.335910391	0.042006221	-0.29390417	8	0.087441723	0.816496581	0.816496581	0.816496581	0.816496581	0.816496581	0.816496581	6	98	0	P_203695	P_203695	P_203695	chr1	117991424	117991484	-	SPAG17	sperm associated antigen 17 [Source:HGNC Symbol;Acc:HGNC:26620]	ENSG00000155761	protein_coding	exon	coding exon	1	948	1:117991424:117991484	ENSG00000155761	SPAG17	4.616411018	0.008346883	-4.054500438	0.448550976	-3.491385461	4.503051414	0.563114977	-3.939936437	9.961963646	0.002324893	0.66335427	0.005842782	0.001148647	0.585806508	0.002795654	-1.729915923	-6.077122622	-2.716557861	-1.130679133	-6.130740879	-1.983007155	10.82460231	0.001776156

ACTRT1	-0.767413108	-0.492411825	-0.22411589	0.275001283	0.543297218	0.268295935	8	0.600864214	0.816496581	0.816496581	0.816496581	0.816496581	0.816496581	0.816496581	6	99	0	P_151633	P_151633	P_151633	chrX	128050965	128051025	-	ACTRT1	actin-related protein T1 [Source:HGNC Symbol;Acc:HGNC:24027]	ENSG00000123165	protein_coding	3'UTR	3'UTR	1	949	X:128050965:128051025	ENSG00000123165	ACTRT1	3.242197156	0.292139726	-1.738919725	-1.115780571	-0.507835399	0.623139154	1.231084326	0.607945172	9.961963646	0.112796921	0.290707094	0.622622822	0.547193256	0.246564179	0.556828739	-5.559033484	-5.56524079	-6.908085997	-6.941321881	-5.548155355	-6.87314746	1.133362207	0.382061043

FANCI	-0.502933278	-0.086755025	-0.328862116	0.416178252	0.174071162	-0.24210709	8	0.040109855	0.816496581	0.816496581	0.816496581	0.816496581	0.816496581	0.816496581	6	100	0	P_224902	P_224902	P_224902	chr15	89292804	89292864	+	FANCI	"Fanconi anemia, complementation group I [Source:HGNC Symbol;Acc:HGNC:25568]"	ENSG00000140525	protein_coding	exon	coding exon	1	950	15:89292804:89292864	ENSG00000140525	FANCI	9.100426466	0.003498638	-10.4137337	-1.796349082	-6.809417169	8.617384617	3.604316529	-5.013068087	9.961963646	1.13E-06	0.102775713	4.77E-05	6.26E-06	0.004842795	0.000533237	6.101686562	-4.717925275	2.26241437	4.309026029	-1.954613956	-0.266438415	45.07035811	4.20E-06

RNF121	-0.112638034	0.130975502	0.016614428	0.243613536	0.129252462	-0.114361074	8	0.072454005	0.816496581	0.816496581	0.816496581	0.816496581	0.816496581	0.816496581	6	101	0	P_109491	P_109491	P_109491	chr11	71928820	71928880	+	RNF121	ring finger protein 121 [Source:HGNC Symbol;Acc:HGNC:21070]	ENSG00000137522	protein_coding	5'UTR	5'UTR	1	951	11:71928820:71928880	ENSG00000137522	RNF121	7.738232476	0.006422385	-1.721402838	2.001647168	0.25391178	3.723050005	1.975314617	-1.747735388	9.961963646	0.116026229	0.073295438	0.804727449	0.003981327	0.076575667	0.111202832	-5.584405043	-4.420787614	-7.012026507	-2.416741256	-4.556129909	-5.617368797	4.641973079	0.027974873

ZSWIM5	0.319991424	0.020704693	0.129108629	-0.299286731	-0.190882795	0.108403936	8	0.122893666	0.816496581	0.816496581	0.816496581	0.816496581	0.816496581	0.816496581	6	102	0	P_201477	P_201477	P_201477	chr1	45020689	45020749	-	ZSWIM5	"zinc finger, SWIM-type containing 5 [Source:HGNC Symbol;Acc:HGNC:29299]"	ENSG00000162415	protein_coding	exon	coding exon	1	952	1:45020689:45020749	ENSG00000162415	ZSWIM5	5.329721526	0.014335099	3.273283549	0.211794213	1.32068899	-3.061489336	-1.952594559	1.108894777	9.961963646	0.008425201	0.836537866	0.216139786	0.012065077	0.079516383	0.293527664	-3.044048096	-6.158495497	-6.16991574	-3.546100521	-4.589912624	-6.438223497	4.486408581	0.030697859

ZPLD1	-0.12782464	-0.056265546	-0.118991555	0.071559094	0.008833085	-0.062726009	8	0.126648456	0.816496581	0.816496581	0.816496581	0.816496581	0.816496581	0.816496581	6	103	0	P_135502	P_135502	P_135502	chr3	102479763	102479823	+	ZPLD1	zona pellucida-like domain containing 1 [Source:HGNC Symbol;Acc:HGNC:27022]	ENSG00000170044	protein_coding	3'UTR	3'UTR	1	953	3:102479763:102479823	ENSG00000170044	ZPLD1	5.52969172	0.015087543	-1.274532596	-0.561020726	-1.18645838	0.713511871	0.088074216	-0.625437655	9.961963646	0.231400943	0.587179831	0.262965808	0.491906114	0.931563029	0.54574397	-6.177719678	-6.018837705	-6.328423723	-6.878125828	-6.292561035	-6.861797558	0.709148266	0.568355362

TFAP2A	0.141368406	0.370695793	0.062230214	0.229327387	-0.079138192	-0.308465579	8	0.123567373	0.816496581	0.816496581	0.816496581	0.816496581	0.816496581	0.816496581	6	104	0	P_256135	P_256135	P_256135	chr6	10414986	10415046	-	TFAP2A	transcription factor AP-2 alpha (activating enhancer binding protein 2 alpha) [Source:HGNC Symbol;Acc:HGNC:11742]	ENSG00000137203	protein_coding	"5'UTR;5'UTR,exon"	multiple Ref	1	954	6:10414986:10415046	ENSG00000137203	TFAP2A	5.889453926	0.01446844	1.439418707	3.774439231	0.6336305	2.335020525	-0.805788207	-3.140808731	9.961963646	0.180704312	0.003659531	0.540596053	0.041783789	0.439175657	0.010543128	-5.972027468	-1.633899028	-6.832199031	-4.775148077	-5.962480752	-3.337294117	5.447683384	0.01773087

ZBTB25	-0.173087113	-0.118725141	-0.215703554	0.054361972	-0.042616441	-0.096978413	8	0.131644493	0.816496581	0.816496581	0.816496581	0.816496581	0.816496581	0.816496581	6	105	0	P_115077	P_115077	P_115077	chr14	64457849	64457909	+	ZBTB25;MTHFD1	"zinc finger and BTB domain containing 25 [Source:HGNC Symbol;Acc:HGNC:13112];methylenetetrahydrofolate dehydrogenase (NADP+ dependent) 1, methenyltetrahydrofolate cyclohydrolase, formyltetrahydrofolate synthetase [Source:HGNC Symbol;Acc:HGNC:7432]"	ENSG00000089775	protein_coding	3'ext;intron;promoter	multiple non-Ref	0	955	14:64457849:64457909	ENSG00000089775	ZBTB25	6.618554143	0.016123838	-1.669460524	-1.14512821	-2.080504791	0.524332315	-0.411044267	-0.935376582	9.961963646	0.126100266	0.278921749	0.064248614	0.611514376	0.689742619	0.371713826	-5.658816478	-5.534404716	-5.08711719	-7.001315625	-6.207597709	-6.613377996	1.62428806	0.245561713

CDKN2B	0.171604285	-0.054544021	0.316303119	-0.226148306	0.144698833	0.370847139	8	0.167122058	0.816496581	0.816496581	0.816496581	0.816496581	0.816496581	0.816496581	6	106	0	A_24_P360674	A_24_P360674	A_24_P360674	chr9	22004945	22005005	-	CDKN2B	"cyclin-dependent kinase inhibitor 2B (p15, inhibits CDK4) [Source:HGNC Symbol;Acc:HGNC:1788]"	ENSG00000147883	protein_coding	3'UTR	3'UTR	1	956	9:22004945:22005005	ENSG00000147883	CDKN2B	4.780702413	0.024635822	1.339029762	-0.425607479	2.468116044	-1.764637241	1.129086282	2.893723523	9.961963646	0.210312174	0.679442036	0.033295909	0.10820385	0.285316565	0.016064466	-6.099310122	-6.087488315	-4.45663641	-5.66914312	-5.659951633	-3.760049979	3.471359248	0.058827713

OGFOD2	-0.05798013	0.00949801	0.02311299	0.06747814	0.08109312	0.01361498	8	0.126382762	0.816496581	0.816496581	0.816496581	0.816496581	0.816496581	0.816496581	6	107	0	P_217963	P_217963	P_217963	chr12	122979109	122979169	+	OGFOD2	2-oxoglutarate and iron-dependent oxygenase domain containing 2 [Source:HGNC Symbol;Acc:HGNC:25823]	ENSG00000111325	protein_coding	3'UTR;exon	multiple Ref	1	957	12:122979109:122979169	ENSG00000111325	OGFOD2	7.42886683	0.015033555	-0.579153888	0.094874042	0.230871816	0.674027929	0.810025703	0.135997774	9.961963646	0.575346364	0.926296098	0.82208681	0.515628137	0.436847015	0.894531973	-6.818363231	-6.17734454	-7.018090119	-6.906696025	-5.959070299	-7.061283861	0.254445465	0.856455671

ABL2	-0.053426341	0.047706689	-0.055808238	0.10113303	-0.002381897	-0.103514927	8	0.069295675	0.816496581	0.816496581	0.816496581	0.816496581	0.816496581	0.816496581	6	108	0	P_104154	P_104154	P_104154	chr1	179229240	179229300	-	ABL2	"ABL proto-oncogene 2, non-receptor tyrosine kinase [Source:HGNC Symbol;Acc:HGNC:77]"	ENSG00000143322	protein_coding	exon	coding exon	1	958	1:179229240:179229300	ENSG00000143322	ABL2	6.785513806	0.006062864	-0.840353767	0.7503882	-0.877819108	1.590741967	-0.037465342	-1.628207309	9.961963646	0.420421684	0.470372989	0.400717223	0.142868261	0.970854115	0.134654529	-6.626148167	-5.893669989	-6.64190296	-5.917408708	-6.295938728	-5.78840618	1.203794332	0.358057877

CAND2	0.310293997	0.352850002	0.181750272	0.042556006	-0.128543725	-0.171099731	8	0.204521641	0.816496581	0.816496581	0.816496581	0.816496581	0.816496581	0.816496581	6	109	0	P_134102	P_134102	P_134102	chr3	12833758	12833818	+	CAND2	cullin-associated and neddylation-dissociated 2 (putative) [Source:HGNC Symbol;Acc:HGNC:30689]	ENSG00000144712	protein_coding	exon	coding exon	1	959	3:12833758:12833818	ENSG00000144712	CAND2	4.604775168	0.035797733	2.008589728	2.284062528	1.176502712	0.2754728	-0.832087016	-1.107559817	9.961963646	0.072452942	0.045565248	0.266741359	0.788582454	0.424856869	0.294076942	-5.152805353	-3.992661439	-6.339680235	-7.108272163	-5.941063575	-6.439661279	2.105079296	0.163427079

SLMAP	0.066015046	0.016427488	0.072669166	-0.049587559	0.00665412	0.056241679	8	0.055313236	0.816496581	0.816496581	0.816496581	0.816496581	0.816496581	0.816496581	6	110	0	P_135198	P_135198	P_135198	chr3	57925873	57925933	+	SLMAP	sarcolemma associated protein [Source:HGNC Symbol;Acc:HGNC:16643]	ENSG00000163681	protein_coding	"3'UTR,exon;exon"	multiple Ref	1	960	3:57925873:57925933	ENSG00000163681	SLMAP	8.511370197	0.004663673	1.183925926	0.294613571	1.303262129	-0.889312355	0.119336204	1.008648558	9.961963646	0.263922144	0.774336982	0.221800849	0.394802846	0.907381245	0.337014141	-6.283118816	-6.136536899	-6.19118137	-6.733518094	-6.28911618	-6.542357467	0.834909907	0.504914734

SSNA1	-0.05149201	0.030171595	-0.016600431	0.081663604	0.034891579	-0.046772025	8	0.0922171	0.816496581	0.816496581	0.816496581	0.816496581	0.816496581	0.816496581	6	111	0	A_24_P303915	A_24_P303915	A_24_P303915	chr9	137189831	137189891	+	SSNA1	Sjogren syndrome nuclear autoantigen 1 [Source:HGNC Symbol;Acc:HGNC:11321]	ENSG00000176101	protein_coding	exon	coding exon	1	961	9:137189831:137189891	ENSG00000176101	SSNA1	12.96467721	0.009035854	-0.663438787	0.388740044	-0.213885023	1.052178831	0.449553764	-0.602625067	9.961963646	0.522105392	0.705647541	0.834950876	0.317569998	0.662655126	0.560225057	-6.763370283	-6.103045084	-7.022195417	-6.57571632	-6.190303331	-6.876540162	0.384892083	0.766302665

SMC1B	-0.188889288	-0.013033339	-0.002533869	0.175855949	0.186355419	0.01049947	8	0.131884165	0.816496581	0.816496581	0.816496581	0.816496581	0.816496581	0.816496581	6	112	0	P_311926	P_311926	P_311926	chr22	45354690	45354750	-	SMC1B	structural maintenance of chromosomes 1B [Source:HGNC Symbol;Acc:HGNC:11112]	ENSG00000077935	protein_coding	intron	intron sense	0	962	22:45354690:45354750	ENSG00000077935	SMC1B	3.740009702	0.016174559	-1.819016905	-0.125511959	-0.024401332	1.693504947	1.794615573	0.101110627	9.961963646	0.099050039	0.902615069	0.981014351	0.121342698	0.103065866	0.921468712	-5.44132986	-6.17378959	-7.046795444	-5.772417943	-4.819966217	-7.065796353	1.570666433	0.257388458

ECI2	-0.267161975	0.091266368	-0.205512361	0.358428343	0.061649614	-0.296778729	8	0.058297364	0.816496581	0.816496581	0.816496581	0.816496581	0.816496581	0.816496581	6	113	0	P_256059	P_256059	P_256059	chr6	4130787	4130847	-	ECI2	enoyl-CoA delta isomerase 2 [Source:HGNC Symbol;Acc:HGNC:14601]	ENSG00000198721	protein_coding	exon	coding exon	1	963	6:4130787:4130847	ENSG00000198721	ECI2	10.95063825	0.004935931	-4.657316239	1.591006119	-3.582605858	6.248322358	1.074710381	-5.173611977	9.961963646	0.000907153	0.142808826	0.005020231	9.69E-05	0.307844869	0.000422054	-0.758820602	-4.999069656	-2.561784116	1.453959155	-5.716532177	-0.023027254	17.33662589	0.000279499

PRPF38B	-0.163461554	-0.066348188	-0.190321168	0.097113365	-0.026859615	-0.12397298	8	0.052667985	0.816496581	0.816496581	0.816496581	0.816496581	0.816496581	0.816496581	6	114	0	P_102562	P_102562	P_102562	chr1	108697957	108698017	+	PRPF38B	pre-mRNA processing factor 38B [Source:HGNC Symbol;Acc:HGNC:25512]	ENSG00000134186	protein_coding	exon	non-coding exon	1	964	1:108697957:108698017	ENSG00000134186	PRPF38B	8.969451989	0.00443429	-3.006416819	-1.220288835	-3.500424097	1.786127983	-0.494007278	-2.280135262	9.961963646	0.01325203	0.250454227	0.005755378	0.104497389	0.632011534	0.045870115	-3.499848979	-5.452709527	-2.701199055	-5.637503289	-6.168498394	-4.789809204	5.235654827	0.019914313

IFRD1	-0.01239733	0.130140549	0.173156443	0.14253788	0.185553773	0.043015894	8	0.065255928	0.816496581	0.816496581	0.816496581	0.816496581	0.816496581	0.816496581	6	115	0	P_145684	P_145684	P_145684	chr7	112476105	112476165	+	IFRD1	interferon-related developmental regulator 1 [Source:HGNC Symbol;Acc:HGNC:5456]	ENSG00000006652	protein_coding	3'UTR;exon	multiple Ref	1	965	7:112476105:112476165	ENSG00000006652	IFRD1	8.536241719	0.00562636	-0.202423087	2.12492938	2.82729107	2.327352467	3.029714157	0.70236169	9.961963646	0.843660197	0.059630558	0.017998348	0.042332616	0.01273604	0.498535941	-6.976046755	-4.236224639	-3.850662964	-4.787756777	-2.882740402	-6.808386423	4.606487349	0.028569756

RECK	0.105475546	0.107828537	0.080708573	0.002352991	-0.024766973	-0.027119964	8	0.085126763	0.816496581	0.816496581	0.816496581	0.816496581	0.816496581	0.816496581	6	116	0	P_267336	P_267336	P_267336	chr9	36083499	36083559	+	RECK	reversion-inducing-cysteine-rich protein with kazal motifs [Source:HGNC Symbol;Acc:HGNC:11345]	ENSG00000122707	protein_coding	exon	coding exon	1	966	9:36083499:36083559	ENSG00000122707	RECK	5.668034057	0.008026071	1.44193572	1.474103007	1.103351241	0.032167287	-0.338584479	-0.370751766	9.961963646	0.180010163	0.171338877	0.295813799	0.974974005	0.741950734	0.718584531	-5.968757836	-5.15040116	-6.420162299	-7.149047231	-6.23606982	-6.996802342	0.953778886	0.451582029

GABRD	0.192585842	0.046592988	0.434397082	-0.145992855	0.24181124	0.387804095	8	0.108061227	0.816496581	0.816496581	0.816496581	0.816496581	0.816496581	0.816496581	6	117	0	P_100140	P_100140	P_100140	chr1	2030582	2030642	+	GABRD	"gamma-aminobutyric acid (GABA) A receptor, delta [Source:HGNC Symbol;Acc:HGNC:4084]"	ENSG00000187730	protein_coding	3'UTR	3'UTR	1	967	1:2030582:2030642	ENSG00000187730	GABRD	5.61844044	0.011584135	2.191483343	0.53019347	4.943115019	-1.661289873	2.751631676	4.412921549	9.961963646	0.053301981	0.607592228	0.000591116	0.127754768	0.020488524	0.001320539	-4.863399645	-6.03603486	-0.354030987	-5.818424784	-3.334957534	-1.208255519	9.887221329	0.002477477

HARS1	-0.102040397	-0.02696194	-0.096791985	0.075078457	0.005248413	-0.069830044	8	0.090806643	0.816496581	0.816496581	0.816496581	0.816496581	0.816496581	0.816496581	6	118	0	P_140592	P_140592	P_140592	chr5	140675245	140675305	-	HARS	histidyl-tRNA synthetase [Source:HGNC Symbol;Acc:HGNC:4816]	ENSG00000170445	protein_coding	exon	non-coding exon	1	968	5:140675245:140675305	ENSG00000170445	HARS1	9.292103408	0.008828548	-1.330065786	-0.351440757	-1.261654312	0.978625029	0.068411474	-0.910213554	9.961963646	0.213143801	0.732577317	0.235813743	0.350932291	0.94681175	0.384204315	-6.110369843	-6.117393201	-6.241141244	-6.649687822	-6.294196563	-6.636736351	0.879205499	0.484313536

TEX10	-0.083144553	0.001634148	-0.12422898	0.084778702	-0.041084426	-0.125863128	8	0.05440089	0.816496581	0.816496581	0.816496581	0.816496581	0.816496581	0.816496581	6	119	0	P_268236	P_268236	P_268236	chr9	100308499	100308559	-	TEX10	testis expressed 10 [Source:HGNC Symbol;Acc:HGNC:25988]	ENSG00000136891	protein_coding	exon	coding exon	1	969	9:100308499:100308559	ENSG00000136891	TEX10	7.803040507	0.004583289	-1.50414881	0.029562998	-2.247397628	1.533711808	-0.743248818	-2.276960626	9.961963646	0.163568068	0.976999451	0.048489666	0.156223378	0.47449442	0.046118003	-5.886780383	-6.181626713	-4.819797926	-5.995559183	-6.010979679	-4.794985805	2.567229021	0.113226656

TBX21	0.004553277	0.054525335	-0.160105811	0.049972058	-0.164659088	-0.214631147	8	0.254872991	0.816496581	0.816496581	0.816496581	0.816496581	0.816496581	0.816496581	6	120	0	P_229063	P_229063	P_229063	chr17	47744483	47744543	+	TBX21	T-box 21 [Source:HGNC Symbol;Acc:HGNC:11599]	ENSG00000073861	protein_coding	exon	coding exon	1	970	17:47744483:47744543	ENSG00000073861	TBX21	4.18213282	0.0543733	0.023915356	0.286385568	-0.840930065	0.262470211	-0.864845421	-1.127315633	9.961963646	0.981392388	0.780450243	0.420113671	0.798306835	0.40746703	0.286029373	-6.998048221	-6.139034964	-6.674119597	-7.112069543	-5.913556059	-6.418246703	0.47957346	0.703690595

LINC00305	0.055209915	0.030416113	-0.007583522	-0.024793802	-0.062793437	-0.037999635	8	0.32086658	0.816496581	0.816496581	0.816496581	0.816496581	0.816496581	0.816496581	6	121	0	P_231743	P_231743	P_231743	chr18	64098576	64098636	-	LINC00305	long intergenic non-protein coding RNA 305 [Source:HGNC Symbol;Acc:HGNC:28597]	ENSG00000179676	lincRNA	exon	non-coding exon	1	971	18:64098576:64098636	ENSG00000179676	LINC00305	3.356296918	0.084885454	0.232084467	0.127859414	-0.031878651	-0.104225053	-0.263963118	-0.159738065	9.961963646	0.821170534	0.90080446	0.975198478	0.919059251	0.797188442	0.876279753	-6.969047412	-6.173476565	-7.046565647	-7.143675784	-6.259758537	-7.057456857	0.029547186	0.992722079

RARA	0.116326853	0.014422634	0.236117237	-0.101904218	0.119790384	0.221694602	8	0.112690246	0.816496581	0.816496581	0.816496581	0.816496581	0.816496581	0.816496581	6	122	0	P_121374	P_121374	P_121374	chr17	40341401	40341461	+	RARA	"retinoic acid receptor, alpha [Source:HGNC Symbol;Acc:HGNC:9864]"	ENSG00000131759	protein_coding	exon	coding exon	1	972	17:40341401:40341461	ENSG00000131759	RARA	8.083150437	0.012404747	1.279179978	0.158597475	2.59644643	-1.120582503	1.317266452	2.437848955	9.961963646	0.229825223	0.877155025	0.026730046	0.288752621	0.217242016	0.035063323	-6.172161639	-6.168844803	-4.241749624	-6.503106205	-5.448266034	-4.530080421	2.889498703	0.088844642

ARHGAP36	-0.339665397	-0.217573531	-0.192968542	0.122091866	0.146696855	0.024604989	8	0.209509285	0.816496581	0.816496581	0.816496581	0.816496581	0.816496581	0.816496581	6	123	0	P_151673	P_151673	P_151673	chrX	131089099	131089159	+	ARHGAP36	Rho GTPase activating protein 36 [Source:HGNC Symbol;Acc:HGNC:26388]	ENSG00000147256	protein_coding	3'UTR	3'UTR	1	973	X:131089099:131089159	ENSG00000147256	ARHGAP36	3.473016771	0.037456072	-2.149491928	-1.376862502	-1.221155665	0.772629426	0.928336263	0.155706838	9.961963646	0.057215252	0.19870597	0.250140055	0.457680838	0.375178897	0.879374033	-4.930672306	-5.270742079	-6.288640348	-6.832621694	-5.857676976	-7.05814981	1.58171602	0.254898953

MCF2	0.121515533	0.14062955	0.220987434	0.019114017	0.099471901	0.080357884	8	0.132244792	0.816496581	0.816496581	0.816496581	0.816496581	0.816496581	0.816496581	6	125	0	P_271701	P_271701	P_271701	chrX	139602405	139602465	-	MCF2	MCF.2 cell line derived transforming sequence [Source:HGNC Symbol;Acc:HGNC:6940]	ENSG00000101977	protein_coding	exon	coding exon	1	975	X:139602405:139602465	ENSG00000101977	MCF2	3.663996153	0.016251052	1.167445701	1.351081296	2.123109895	0.183635595	0.955664195	0.772028599	9.961963646	0.270213426	0.206554855	0.059813304	0.857983227	0.361857392	0.458020763	-6.301667308	-5.301740918	-5.019582012	-7.131202943	-5.832606825	-6.755201311	1.539831667	0.26448395

TIMM8A	0.138087316	0.045662525	0.036976496	-0.092424791	-0.10111082	-0.008686029	8	0.148526069	0.816496581	0.816496581	0.816496581	0.816496581	0.816496581	0.816496581	6	126	0	P_151358	P_151358	P_151358	chrX	101348038	101348098	-	TIMM8A	translocase of inner mitochondrial membrane 8 homolog A (yeast) [Source:HGNC Symbol;Acc:HGNC:11817]	ENSG00000126953	protein_coding	exon	non-coding exon	1	976	X:101348038:101348098	ENSG00000126953	TIMM8A	9.588819093	0.019922061	1.198208181	0.396221841	0.320851625	-0.80198634	-0.877356557	-0.075370217	9.961963646	0.258564845	0.700295263	0.754952331	0.44127191	0.400956533	0.941412238	-6.266886393	-6.099998164	-6.991191505	-6.808832508	-5.902810422	-7.068276046	0.519042941	0.678664295

SMARCA2	0.050832956	0.030639425	0.112510462	-0.02019353	0.061677507	0.081871037	8	0.081569219	0.816496581	0.816496581	0.816496581	0.816496581	0.816496581	0.816496581	6	127	0	P_266582	P_266582	P_266582	chr9	2039628	2039688	+	SMARCA2	"SWI/SNF related, matrix associated, actin dependent regulator of chromatin, subfamily a, member 2 [Source:HGNC Symbol;Acc:HGNC:11098]"	ENSG00000080503	protein_coding	exon	coding exon	1	977	9:2039628:2039688	ENSG00000080503	SMARCA2	7.095596359	0.007549837	0.716509896	0.431874387	1.58587788	-0.284635508	0.869367984	1.154003493	9.961963646	0.490132874	0.6750305	0.143966622	0.781752551	0.405105223	0.275432479	-6.725246329	-6.084708894	-5.823547265	-7.105487647	-5.909686898	-6.388855622	0.897242115	0.476175768

NFXL1	0.074746157	-0.127449093	-0.008256375	-0.20219525	-0.083002531	0.119192718	8	0.024561355	0.816496581	0.816496581	0.816496581	0.816496581	0.816496581	0.816496581	6	128	0	P_248839	P_248839	P_248839	chr4	47862845	47862905	-	NFXL1	"nuclear transcription factor, X-box binding-like 1 [Source:HGNC Symbol;Acc:HGNC:18726]"	ENSG00000170448	protein_coding	3'UTR;exon	multiple Ref	1	978	4:47862845:47862905	ENSG00000170448	NFXL1	8.331333528	0.002691135	1.764683292	-3.008947809	-0.194924888	-4.773631101	-1.959608179	2.814022922	9.961963646	0.108195781	0.013194958	0.849369754	0.000760965	0.078597465	0.018411893	-5.521471625	-2.843220684	-7.026410519	-0.701855154	-4.579501387	-3.895991575	7.792035476	0.005707816

MAP1LC3A	0.390063868	0.102778801	0.197447576	-0.287285067	-0.192616292	0.094668775	8	0.154174283	0.816496581	0.816496581	0.816496581	0.816496581	0.816496581	0.816496581	6	129	0	A_23_P154786	A_23_P154786	A_23_P154786	chr20	34559850	34559910	+	MAP1LC3A	microtubule-associated protein 1 light chain 3 alpha [Source:HGNC Symbol;Acc:HGNC:6838]	ENSG00000101460	protein_coding	"3'UTR,exon;exon"	multiple Ref	1	979	20:34559850:34559910	ENSG00000101460	MAP1LC3A	8.546827132	0.021295057	3.273723165	0.862600635	1.65713555	-2.41112253	-1.616587615	0.794534915	9.961963646	0.008418952	0.408642837	0.128603444	0.036700495	0.137155825	0.445399622	-3.043298026	-5.804370781	-5.723902232	-4.649394731	-5.067453402	-6.737065504	3.877599092	0.04490515

ARHGEF6	0.105424142	-0.031745085	0.072618733	-0.137169227	-0.032805409	0.104363818	8	0.122459459	0.816496581	0.816496581	0.816496581	0.816496581	0.816496581	0.816496581	6	130	0	P_271657	P_271657	P_271657	chrX	136672019	136672079	-	ARHGEF6	Rac/Cdc42 guanine nucleotide exchange factor (GEF) 6 [Source:HGNC Symbol;Acc:HGNC:685]	ENSG00000129675	protein_coding	exon	coding exon	1	980	X:136672019:136672079	ENSG00000129675	ARHGEF6	4.496948204	0.014249546	1.08164611	-0.325702889	0.745064353	-1.407348998	-0.336581757	1.070767241	9.961963646	0.304897514	0.751387227	0.473444176	0.18975115	0.743414945	0.309530207	-6.394998441	-6.12647183	-6.752169146	-6.162287541	-6.236780617	-6.478752496	0.845270422	0.500016889

SCD5	0.461747996	0.119036261	0.421670521	-0.342711735	-0.040077475	0.30263426	8	0.15856365	0.816496581	0.816496581	0.816496581	0.816496581	0.816496581	0.816496581	6	132	0	P_249677	P_249677	P_249677	chr4	82636694	82636754	-	SCD5	stearoyl-CoA desaturase 5 [Source:HGNC Symbol;Acc:HGNC:21088]	ENSG00000145284	protein_coding	exon	coding exon	1	982	4:82636694:82636754	ENSG00000145284	SCD5	5.936810044	0.022397427	3.77877979	0.974150019	3.450800126	-2.804629771	-0.327979664	2.476650108	9.961963646	0.003633624	0.353042425	0.006252518	0.018710437	0.749716175	0.032813638	-2.188113204	-5.705168469	-2.785578838	-3.985817518	-6.239786766	-4.46549705	6.873913507	0.008637518

BICRA	0.034795531	-0.089679001	-0.037445385	-0.124474532	-0.072240916	0.052233616	8	0.196276317	0.816496581	0.816496581	0.816496581	0.816496581	0.816496581	0.816496581	6	133	0	P_233067	P_233067	P_233067	chr19	47699323	47699383	+	GLTSCR1	glioma tumor suppressor candidate region gene 1 [Source:HGNC Symbol;Acc:HGNC:4332]	ENSG00000063169	protein_coding	exon	coding exon	1	983	19:47699323:47699383	ENSG00000063169	BICRA	7.528936131	0.033143872	0.234081707	-0.603302007	-0.251908201	-0.837383714	-0.485989908	0.351393806	9.961963646	0.819662041	0.559792259	0.806232776	0.422011515	0.637486741	0.732611466	-6.968542126	-5.993776158	-7.012576405	-6.779116176	-6.172575801	-7.004339842	0.25718343	0.854551275

SYTL2	0.879795482	-0.045298813	1.143068599	-0.925094295	0.263273116	1.188367411	8	0.125592524	0.816496581	0.816496581	0.816496581	0.816496581	0.816496581	0.816496581	6	134	0	P_212639	P_212639	P_212639	chr11	85757664	85757724	-	SYTL2	synaptotagmin-like 2 [Source:HGNC Symbol;Acc:HGNC:15585]	ENSG00000137501	protein_coding	exon	coding exon	1	984	11:85757664:85757724	ENSG00000137501	SYTL2	7.578449384	0.01487365	8.83524458	-0.454908099	11.47913446	-9.290152679	2.643889881	11.93404256	9.961963646	5.01E-06	0.658927846	4.58E-07	3.20E-06	0.02464313	3.19E-07	4.603383351	-6.074158183	6.992858072	5.003629441	-3.509372787	7.351244158	74.29291309	4.04E-07

CHD7	-0.129675985	0.049835361	-0.080287409	0.179511346	0.049388576	-0.13012277	8	0.078896938	0.816496581	0.816496581	0.816496581	0.816496581	0.816496581	0.816496581	6	135	0	P_147272	P_147272	P_147272	chr8	60865980	60866040	+	CHD7	chromodomain helicase DNA binding protein 7 [Source:HGNC Symbol;Acc:HGNC:20626]	ENSG00000171316	protein_coding	3'UTR	3'UTR	1	985	8:60865980:60866040	ENSG00000171316	CHD7	6.320822027	0.007205479	-1.87099986	0.719037941	-1.158408251	2.590037801	0.712591609	-1.877446192	9.961963646	0.090971625	0.488640707	0.273713596	0.027025114	0.492451218	0.090013517	-5.363534525	-5.916654232	-6.35995534	-4.349973889	-6.033479648	-5.424683499	2.683408754	0.10362122

MC1R	-0.200403865	-0.164026653	-0.246320476	0.036377212	-0.04591661	-0.082293823	8	0.126647869	0.816496581	0.816496581	0.816496581	0.816496581	0.816496581	0.816496581	6	136	0	A_23_P329271	A_23_P329271	A_23_P329271	chr16	89920867	89920927	+	MC1R	melanocortin 1 receptor (alpha melanocyte stimulating hormone receptor) [Source:HGNC Symbol;Acc:HGNC:6929]	ENSG00000258839	protein_coding	3'UTR;exon	multiple Ref	1	986	16:89920867:89920927	ENSG00000258839	MC1R	9.88218106	0.015087424	-1.998224037	-1.635507379	-2.456057894	0.362716658	-0.457833856	-0.820550514	9.961963646	0.073714238	0.133103986	0.033989262	0.724393892	0.656895281	0.431098946	-5.168907756	-4.939705926	-4.476704994	-7.07813925	-6.186390728	-6.715531822	2.285747071	0.14119187

TCEA2	0.075764663	-0.080790017	0.005127021	-0.15655468	-0.070637641	0.085917038	8	0.100124581	0.816496581	0.816496581	0.816496581	0.816496581	0.816496581	0.816496581	6	137	0	P_131893	P_131893	P_131893	chr20	64064049	64064109	+	TCEA2	"transcription elongation factor A (SII), 2 [Source:HGNC Symbol;Acc:HGNC:11614]"	ENSG00000171703	protein_coding	exon	non-coding exon	1	987	20:64064049:64064109	ENSG00000171703	TCEA2	9.725450908	0.010257251	0.916213815	-0.97698488	0.062000509	-1.893198695	-0.854213307	1.038985389	9.961963646	0.381199194	0.351704607	0.951788734	0.087711552	0.41305672	0.323371736	-6.558709715	-5.702517457	-7.045021728	-5.477049173	-5.922584001	-6.511675946	1.198527264	0.35979406

ZCCHC2	-0.270723103	0.144499877	-0.223712982	0.415222981	0.047010121	-0.368212859	8	0.101200673	0.816496581	0.816496581	0.816496581	0.816496581	0.816496581	0.816496581	6	138	0	A_23_P304054	A_23_P304054	A_23_P304054	chr18	62586249	62586309	+	ZCCHC2	"zinc finger, CCHC domain containing 2 [Source:HGNC Symbol;Acc:HGNC:22916]"	ENSG00000141664	protein_coding	3'UTR;exon	multiple Ref	1	988	18:62586249:62586309	ENSG00000141664	ZCCHC2	7.312938262	0.010431228	-3.246408958	1.732787822	-2.682681381	4.979196781	0.563727578	-4.415469203	9.961963646	0.008816394	0.113917951	0.023058168	0.000560468	0.585405116	0.001315323	-3.089911148	-4.806851047	-4.096215786	-0.382762122	-6.13038543	-1.204155389	10.89084363	0.001736287

TERB1	0.010223028	0.153423558	-0.102650876	0.14320053	-0.112873904	-0.256074434	8	0.18451545	0.816496581	0.816496581	0.816496581	0.816496581	0.816496581	0.816496581	6	139	0	P_226606	P_226606	P_226606	chr16	66767414	66767474	-	CCDC79	coiled-coil domain containing 79 [Source:HGNC Symbol;Acc:HGNC:26675]	ENSG00000249961	protein_coding	3'UTR;exon	multiple Ref	1	989	16:66767414:66767474	ENSG00000249961	TERB1	3.603646223	0.029547439	0.072839214	1.093144959	-0.731388902	1.020305746	-0.804228115	-1.824533861	9.961963646	0.94337578	0.300058822	0.48139143	0.331722011	0.440035051	0.098162283	-6.995465897	-5.588604125	-6.762619531	-6.608303583	-5.963732423	-5.504112824	1.125321944	0.384911882

POLD3	-0.338030688	0.026711654	-0.141387138	0.364742342	0.19664355	-0.168098791	8	0.156137346	0.816496581	0.816496581	0.816496581	0.816496581	0.816496581	0.816496581	6	141	0	P_212456	P_212456	P_212456	chr11	74636215	74636275	+	POLD3	"polymerase (DNA-directed), delta 3, accessory subunit [Source:HGNC Symbol;Acc:HGNC:20932]"	ENSG00000077514	protein_coding	exon	coding exon	1	991	11:74636215:74636275	ENSG00000077514	POLD3	8.683452496	0.021784247	-2.804984638	0.221653774	-1.173232972	3.026638412	1.631751666	-1.394886746	9.961963646	0.018699071	0.82906102	0.267990726	0.012802972	0.133899701	0.193369425	-3.843147245	-6.156253592	-6.343361618	-3.605879327	-5.046919893	-6.101082	3.843734572	0.045903635

NIPBL	0.132437586	-0.141124835	-0.015274583	-0.273562421	-0.147712169	0.125850252	8	0.053616066	0.816496581	0.816496581	0.816496581	0.816496581	0.816496581	0.816496581	6	142	0	P_252427	P_252427	P_252427	chr5	37002705	37002765	+	NIPBL	Nipped-B homolog (Drosophila) [Source:HGNC Symbol;Acc:HGNC:28862]	ENSG00000164190	protein_coding	exon	coding exon	1	992	5:37002705:37002765	ENSG00000164190	NIPBL	7.490218902	0.004515211	2.413892193	-2.572231565	-0.278404332	-4.986123758	-2.692296525	2.293827233	9.961963646	0.036527406	0.027862059	0.786395206	0.000554781	0.022681326	0.044815738	-4.500359448	-3.540724897	-7.004957194	-0.3721126	-3.431104867	-4.767457704	8.3153823	0.004572474

ANKRD1	-0.085562677	-0.173624439	0.068912171	-0.088061762	0.154474848	0.242536611	8	0.155384987	0.816496581	0.816496581	0.816496581	0.816496581	0.816496581	0.816496581	6	143	0	P_208996	P_208996	P_208996	chr10	90918864	90918924	-	ANKRD1	ankyrin repeat domain 1 (cardiac muscle) [Source:HGNC Symbol;Acc:HGNC:15819]	ENSG00000148677	protein_coding	exon	coding exon	1	993	10:90918864:90918924	ENSG00000148677	ANKRD1	3.905938672	0.021596029	-0.713087852	-1.447003329	0.574320886	-0.733915478	1.287408737	2.021324215	9.961963646	0.492157229	0.178619544	0.578487559	0.479916854	0.227056857	0.070931307	-6.727784629	-5.184466861	-6.869928674	-6.862787092	-5.483413047	-5.203504318	1.535660361	0.265460872

PDPR	-0.008595081	-0.093767466	0.033474268	-0.085172384	0.04206935	0.127241734	8	0.064640158	0.816496581	0.816496581	0.816496581	0.816496581	0.816496581	0.816496581	6	144	0	P_226800	P_226800	P_226800	chr16	70162492	70162552	+	PDPR;RP11-296I10.3	pyruvate dehydrogenase phosphatase regulatory subunit [Source:HGNC Symbol;Acc:HGNC:30264];	ENSG00000090857	protein_coding;antisense	"3'ext;3'ext,3'UTR;intron"	multiple non-Ref	0	994	16:70162492:70162552	ENSG00000090857	PDPR	6.550415859	0.005562127	-0.141148147	-1.539846257	0.549713337	-1.39869811	0.690861483	2.089559594	9.961963646	0.890566331	0.154735994	0.594624402	0.19225667	0.50543115	0.063280503	-6.987499546	-5.066145215	-6.884566061	-6.173359049	-6.04891112	-5.096224504	1.582189889	0.254792807

OR56A1	0.1134889	0.261027382	0.087721482	0.147538482	-0.025767419	-0.173305901	8	0.427110027	0.816496581	0.816496581	0.816496581	0.816496581	0.816496581	0.816496581	6	145	0	P_107782	P_107782	P_107782	chr11	6026756	6026816	-	OR56A1	"olfactory receptor, family 56, subfamily A, member 1 [Source:HGNC Symbol;Acc:HGNC:14781]"	ENSG00000180934	protein_coding	exon	coding exon	1	995	11:6026756:6026816	ENSG00000180934	OR56A1	3.347727964	0.14870228	0.360445994	0.829035034	0.278607481	0.46858904	-0.081838514	-0.550427553	9.961963646	0.726038928	0.426502248	0.786243706	0.649448807	0.936396065	0.594152701	-6.927925343	-5.832214042	-7.004895879	-7.030846567	-6.293124161	-6.90834909	0.237347709	0.868325878

AHI1	-0.174900716	-0.072170717	-0.175473096	0.102729999	-0.00057238	-0.103302379	8	0.070057574	0.816496581	0.816496581	0.816496581	0.816496581	0.816496581	0.816496581	6	146	0	P_143649	P_143649	P_143649	chr6	135302730	135302790	-	AHI1	Abelson helper integration site 1 [Source:HGNC Symbol;Acc:HGNC:21575]	ENSG00000135541	protein_coding	3'UTR;exon	multiple Ref	1	996	6:135302730:135302790	ENSG00000135541	AHI1	7.227101683	0.006148127	-2.731906364	-1.127288934	-2.740846799	1.60461743	-0.008940435	-1.613557865	9.961963646	0.02119284	0.286040132	0.020870649	0.139775755	0.993043167	0.137814787	-3.967155178	-5.553221504	-3.997643868	-5.898137658	-6.296642743	-5.808897635	3.570796651	0.054993989

COMMD8	-0.064397301	0.17970055	0.018875051	0.244097851	0.083272352	-0.160825499	8	0.083612408	0.816496581	0.816496581	0.816496581	0.816496581	0.816496581	0.816496581	6	149	0	P_248804	P_248804	P_248804	chr4	47456576	47456636	-	COMMD8	COMM domain containing 8 [Source:HGNC Symbol;Acc:HGNC:26036]	ENSG00000169019	protein_coding	exon	coding exon	1	999	4:47456576:47456636	ENSG00000169019	COMMD8	10.02600441	0.007820866	-0.891837794	2.488671711	0.261400449	3.380509505	1.153238242	-2.227271262	9.961963646	0.39351148	0.032145954	0.79910853	0.007033904	0.275731971	0.050171413	-6.580921432	-3.672870986	-7.009933124	-2.99923298	-5.634134779	-4.875712916	4.12928866	0.03824189

ADK	-0.110464785	-0.03908873	0.029409394	0.071376055	0.139874179	0.068498124	8	0.056185792	0.816496581	0.816496581	0.816496581	0.816496581	0.816496581	0.816496581	6	150	0	P_208780	P_208780	P_208780	chr10	74200778	74200838	+	ADK	adenosine kinase [Source:HGNC Symbol;Acc:HGNC:257]	ENSG00000156110	protein_coding	exon	coding exon	1	1000	10:74200778:74200838	ENSG00000156110	ADK	10.29878275	0.004741801	-1.964707074	-0.69522522	0.523070267	1.269481854	2.487777341	1.218295488	9.961963646	0.07793568	0.502807951	0.612360603	0.233123466	0.032195162	0.251177901	-5.220731177	-5.933523831	-6.899733458	-6.33310607	-3.760363328	-6.315923329	2.316979779	0.137717487

ECM2	0.153243721	0.279511588	0.265613839	0.126267867	0.112370118	-0.013897749	8	0.271939597	0.816496581	0.816496581	0.816496581	0.816496581	0.816496581	0.816496581	6	151	0	P_267953	P_267953	P_267953	chr9	92500726	92500786	-	ECM2	"extracellular matrix protein 2, female organ and adipocyte specific [Source:HGNC Symbol;Acc:HGNC:3154]"	ENSG00000106823	protein_coding	exon	coding exon	1	1001	9:92500726:92500786	ENSG00000106823	ECM2	3.667421528	0.061593485	0.756242235	1.379361367	1.310777384	0.623119132	0.554535149	-0.068583983	9.961963646	0.467010673	0.197958761	0.219344644	0.54720589	0.591443712	0.94667786	-6.694977585	-5.26771672	-6.182035016	-6.941335012	-6.135681083	-7.068809454	0.815527983	0.514209165

RAD51	-0.40806843	0.080592759	-0.311004734	0.488661189	0.097063696	-0.391597493	8	0.076060898	0.816496581	0.816496581	0.816496581	0.816496581	0.816496581	0.816496581	7	20	0	P_116212	P_116212	P_116212	chr15	40729914	40729974	+	RAD51	RAD51 recombinase [Source:HGNC Symbol;Acc:HGNC:9817]	ENSG00000051180	protein_coding	3'UTR;exon	multiple Ref	1	1040	15:40729914:40729974	ENSG00000051180	RAD51	8.095743447	0.006852563	-6.037429838	1.192381207	-4.601358802	7.229811045	1.436071036	-5.793740009	9.961963646	0.00012768	0.260739954	0.000987856	2.88E-05	0.181631099	0.00017703	1.274173318	-5.483492127	-0.887040079	2.721011065	-5.303045011	0.882255625	24.49078765	6.48E-05

MRFAP1L1	-0.024623448	-0.033175588	0.083854353	-0.00855214	0.108477801	0.117029941	8	0.127582812	0.816496581	0.816496581	0.816496581	0.816496581	0.816496581	0.816496581	7	21	0	A_23_P133058	A_23_P133058	A_23_P133058	chr4	6708067	6708127	-	MRFAP1L1	Morf4 family associated protein 1-like 1 [Source:HGNC Symbol;Acc:HGNC:28796]	ENSG00000178988	protein_coding	3'UTR;exon	multiple Ref	1	1041	4:6708067:6708127	ENSG00000178988	MRFAP1L1	10.26175877	0.015278303	-0.243981528	-0.328720434	0.830871161	-0.084738906	1.074852689	1.159591595	9.961963646	0.812196324	0.749172775	0.425511844	0.93414774	0.307784176	0.273253262	-6.965974361	-6.12544233	-6.682696851	-7.145687612	-5.71638694	-6.382634991	0.560845441	0.652927657

GABRA3	0.410004773	0.074842022	0.424844063	-0.335162751	0.014839289	0.350002041	8	0.124687904	0.816496581	0.816496581	0.816496581	0.816496581	0.816496581	0.816496581	7	23	0	P_271835	P_271835	P_271835	chrX	152255886	152255946	-	GABRA3	"gamma-aminobutyric acid (GABA) A receptor, alpha 3 [Source:HGNC Symbol;Acc:HGNC:4077]"	ENSG00000011677	protein_coding	exon	coding exon	1	1043	X:152255886:152255946	ENSG00000011677	GABRA3	6.359723085	0.014691832	4.142824827	0.756228728	4.292765952	-3.3865961	0.149941124	3.536537224	9.961963646	0.002019155	0.467018413	0.00159315	0.006962419	0.883803359	0.005419464	-1.584841561	-5.889291618	-1.381817372	-2.988824825	-6.284741122	-2.661745751	10.02688833	0.002354282

CREB3	0.213086607	-0.014927948	0.13330509	-0.228014555	-0.079781517	0.148233037	8	0.056627746	0.816496581	0.816496581	0.816496581	0.816496581	0.816496581	0.816496581	7	24	0	P_267290	P_267290	P_267290	chr9	35735314	35735374	+	CREB3	cAMP responsive element binding protein 3 [Source:HGNC Symbol;Acc:HGNC:2347]	ENSG00000107175	protein_coding	exon	coding exon	1	1044	9:35735314:35735374	ENSG00000107175	CREB3	9.934112739	0.00478184	3.774020497	-0.264391939	2.360993718	-4.038412437	-1.413026779	2.625385658	9.961963646	0.00366204	0.796867285	0.03997592	0.002385636	0.188121866	0.02543704	-2.196086875	-6.145370564	-4.634060501	-1.888756243	-5.331860798	-4.215932475	7.514249114	0.006447151

MOB3A	-0.024336351	0.18930934	0.031068212	0.213645691	0.055404564	-0.158241128	8	0.084175959	0.816496581	0.816496581	0.816496581	0.816496581	0.816496581	0.816496581	7	25	0	P_124034	P_124034	P_124034	chr19	2073141	2073201	-	MOB3A	MOB kinase activator 3A [Source:HGNC Symbol;Acc:HGNC:29802]	ENSG00000172081	protein_coding	3'UTR;exon	multiple Ref	1	1045	19:2073141:2073201	ENSG00000172081	MOB3A	7.421888636	0.007896801	-0.335409615	2.609108144	0.428189787	2.944517759	0.763599401	-2.180918358	9.961963646	0.74427241	0.026156459	0.677622692	0.014728684	0.462806941	0.054261598	-6.937317018	-3.482193974	-6.947910211	-3.746639332	-5.995577881	-4.950489432	3.518827528	0.056959298

ATP6V1B2	0.013227769	0.020877904	0.07739399	0.007650135	0.064166221	0.056516086	8	0.072432635	0.816496581	0.816496581	0.816496581	0.816496581	0.816496581	0.816496581	7	27	0	P_146641	P_146641	P_146641	chr8	20213817	20213877	+	ATP6V1B2	"ATPase, H+ transporting, lysosomal 56/58kDa, V1 subunit B2 [Source:HGNC Symbol;Acc:HGNC:854]"	ENSG00000147416	protein_coding	exon	non-coding exon	1	1047	8:20213817:20213877	ENSG00000147416	ATP6V1B2	8.359398106	0.006419899	0.202193947	0.319130595	1.183010982	0.116936647	0.980817035	0.863880388	9.961963646	0.843834538	0.756218542	0.264268337	0.90923417	0.349902056	0.407972224	-6.976097139	-6.128681962	-6.332329642	-7.142140271	-5.809002245	-6.678329793	0.544034546	0.663180218

INPP5A	-0.038179105	0.035926803	-0.022965652	0.074105908	0.015213454	-0.058892455	8	0.045137928	0.816496581	0.816496581	0.816496581	0.816496581	0.816496581	0.816496581	7	28	0	P_107408	P_107408	P_107408	chr10	132782190	132782250	+	INPP5A	"inositol polyphosphate-5-phosphatase, 40kDa [Source:HGNC Symbol;Acc:HGNC:6076]"	ENSG00000068383	protein_coding	3'UTR	3'UTR	1	1048	10:132782190:132782250	ENSG00000068383	INPP5A	8.420828431	0.003842853	-0.754300627	0.709802119	-0.453730001	1.464102746	0.300570627	-1.16353212	9.961963646	0.468124141	0.494105821	0.659747113	0.173995199	0.769921047	0.271724766	-6.696490646	-5.923257833	-6.935846832	-6.088542222	-6.248856963	-6.378234699	0.811067658	0.516372552

CHID1	0.04517472	0.047060302	0.0684443	0.001885582	0.02326958	0.021383998	8	0.084212288	0.816496581	0.816496581	0.816496581	0.816496581	0.816496581	0.816496581	7	30	0	P_107570	P_107570	P_107570	chr11	866882	866942	-	CHID1;TSPAN4	chitinase domain containing 1 [Source:HGNC Symbol;Acc:HGNC:28474];tetraspanin 4 [Source:HGNC Symbol;Acc:HGNC:11859]	ENSG00000177830	protein_coding	3'ext;3'UTR;exon	multiple non-Ref	0	1050	11:866882:866942	ENSG00000177830	CHID1	11.14320034	0.007901713	0.622415589	0.648395068	0.943022984	0.025979478	0.320607395	0.294627917	9.961963646	0.547649944	0.531390136	0.367976517	0.979786757	0.755131972	0.774326337	-6.790998218	-5.965195151	-6.582088047	-7.149243978	-6.242302558	-7.024163189	0.314563221	0.814619335

MROH9	-0.085819169	-0.041734474	0.115657073	0.044084695	0.201476242	0.157391547	8	0.15503887	0.816496581	0.816496581	0.816496581	0.816496581	0.816496581	0.816496581	7	31	0	P_204850	P_204850	P_204850	chr1	170995471	170995531	+	MROH9	maestro heat-like repeat family member 9 [Source:HGNC Symbol;Acc:HGNC:26287]	ENSG00000117501	protein_coding	exon	coding exon	1	1051	1:170995471:170995531	ENSG00000117501	MROH9	3.64591795	0.021509747	-0.716658553	-0.348516168	0.96582886	0.368142385	1.682487413	1.314345028	9.961963646	0.490045052	0.73470563	0.356990789	0.720469066	0.123502234	0.218186553	-6.725135815	-6.118458712	-6.560320973	-7.075999542	-4.977448903	-6.201604606	1.043613992	0.415236881

DGLUCY	-0.051062318	0.030182016	0.019490339	0.081244334	0.070552657	-0.010691677	8	0.080054057	0.816496581	0.816496581	0.816496581	0.816496581	0.816496581	0.816496581	7	33	0	P_309943	P_309943	P_309943	chr14	91160234	91160294	-	C14orf159	chromosome 14 open reading frame 159 [Source:HGNC Symbol;Acc:HGNC:20498]	ENSG00000133943	protein_coding	"5'UTR,intron;exon,intron;promoter"	multiple non-Ref	0	1053	14:91160234:91160294	ENSG00000133943	DGLUCY	6.042822029	0.007353181	-0.72930388	0.431078392	0.278373185	1.160382272	1.007677066	-0.152705206	9.961963646	0.482610449	0.675590123	0.786418434	0.272946022	0.337457998	0.881679407	-6.715659546	-6.085064091	-7.004966591	-6.459230023	-5.78324358	-7.058654332	0.530070554	0.671796543

PRDM6	0.165352357	-0.131118748	0.297354464	-0.296471105	0.132002107	0.428473212	8	0.206933781	0.816496581	0.816496581	0.816496581	0.816496581	0.816496581	0.816496581	7	35	0	P_254522	P_254522	P_254522	chr5	123159578	123159638	+	PRDM6	PR domain containing 6 [Source:HGNC Symbol;Acc:HGNC:9350]	ENSG00000061455	protein_coding	exon	coding exon	1	1055	5:123159578:123159638	ENSG00000061455	PRDM6	4.370434061	0.036594755	1.058635844	-0.839461914	1.903753292	-1.898097758	0.845117448	2.743215206	9.961963646	0.314759454	0.420898654	0.086199952	0.087006878	0.417880251	0.020786125	-6.419079167	-5.823666621	-5.361232917	-5.469600695	-5.930231324	-4.01643567	2.88198517	0.089337692

KIAA0586	-0.126892848	-0.014465935	-0.095948704	0.112426913	0.030944145	-0.081482768	8	0.052335302	0.816496581	0.816496581	0.816496581	0.816496581	0.816496581	0.816496581	7	36	0	P_221022	P_221022	P_221022	chr14	58487987	58488047	+	KIAA0586	KIAA0586 [Source:HGNC Symbol;Acc:HGNC:19960]	ENSG00000100578	protein_coding	exon	coding exon	1	1056	14:58487987:58488047	ENSG00000100578	KIAA0586	8.221362216	0.004406237	-2.341255624	-0.266905921	-1.770316016	2.074349703	0.570939608	-1.503410095	9.961963646	0.041342645	0.794985289	0.10721293	0.06491473	0.580690742	0.163755388	-4.62004603	-6.144671595	-5.560674584	-5.196077968	-6.126173627	-5.959405651	2.595979351	0.11075526

C17orf75	0.065722499	-0.02069354	0.073050551	-0.086416039	0.007328051	0.093744091	8	0.050127102	0.816496581	0.816496581	0.816496581	0.816496581	0.816496581	0.816496581	7	37	0	P_121066	P_121066	P_121066	chr17	32331370	32331430	-	C17orf75	chromosome 17 open reading frame 75 [Source:HGNC Symbol;Acc:HGNC:30173]	ENSG00000108666	protein_coding	3'UTR	3'UTR	1	1057	17:32331370:32331430	ENSG00000108666	C17orf75	9.869932543	0.00422454	1.238425748	-0.389933634	1.376510083	-1.628359383	0.138084335	1.766443717	9.961963646	0.243946817	0.704792538	0.198811541	0.134622065	0.892925006	0.107887724	-6.220403449	-6.102562772	-6.100484422	-5.864937046	-6.286553639	-5.590009954	1.561911925	0.259380629

CLN3	0.075632783	0.151698165	0.169605269	0.076065381	0.093972486	0.017907104	8	0.052047097	0.816496581	0.816496581	0.816496581	0.816496581	0.816496581	0.816496581	7	38	0	P_226021	P_226021	P_226021	chr16	28477804	28477864	+	CLN3	"ceroid-lipofuscinosis, neuronal 3 [Source:HGNC Symbol;Acc:HGNC:2074];battenin isoform d [Source:RefSeq peptide;Acc:NP_001273038]"	ENSG00000188603	protein_coding	3'ext;3'UTR;exon;intron	multiple non-Ref	0	1058	16:28477804:28477864	ENSG00000188603	CLN3	8.900946056	0.004382079	1.39931545	2.806634591	3.13794182	1.407319141	1.73862637	0.331307229	9.961963646	0.192076945	0.018646311	0.010594564	0.18975975	0.112850323	0.747276335	-6.023613936	-3.167139516	-3.319834517	-6.162325831	-4.89925571	-7.011744074	4.132515603	0.038164427

TTC41P	0.031536192	0.037981027	0.052238401	0.006444835	0.020702209	0.014257374	8	0.228818238	0.816496581	0.816496581	0.816496581	0.816496581	0.816496581	0.816496581	7	40	0	P_217135	P_217135	P_217135	chr12	103896970	103897030	-	GNN	Grp94 neighboring nucleotidase pseudogene [Source:EntrezGene;Acc:253724]	ENSG00000214198	unitary_pseudogene	exon	non-coding exon	1	1060	12:103896970:103897030	ENSG00000214198	TTC41P	4.076695728	0.044252841	0.183604914	0.221126988	0.304133965	0.037522074	0.120529051	0.083006977	9.961963646	0.858006664	0.829460055	0.767283671	0.970810003	0.906460351	0.935490224	-6.979995925	-6.156375928	-6.996841588	-7.148843181	-6.288964221	-7.067615718	0.032988449	0.991448357

VWF	-0.251795169	-0.183305728	-0.499233974	0.068489441	-0.247438805	-0.315928246	8	0.120040782	0.816496581	0.816496581	0.816496581	0.816496581	0.816496581	0.816496581	7	44	0	A_32_P150382	A_32_P150382	A_32_P150382	chr12	6124510	6124570	-	VWF	von Willebrand factor [Source:HGNC Symbol;Acc:HGNC:12726]	ENSG00000110799	protein_coding	5'UTR;exon	multiple Ref	1	1064	12:6124510:6124570	ENSG00000110799	VWF	5.280670424	0.013778531	-2.627190529	-1.912582656	-5.208927456	0.714607874	-2.581736927	-3.296344801	9.961963646	0.025358494	0.084954121	0.000401092	0.4912574	0.027412125	0.008103758	-4.144065703	-4.551416822	0.049136234	-6.877311789	-3.609590257	-3.071139923	9.289157793	0.003100921

COPRS	-0.295148368	0.216512113	-0.098763324	0.511660481	0.196385044	-0.315275436	8	0.061694407	0.816496581	0.816496581	0.816496581	0.816496581	0.816496581	0.816496581	7	45	0	A_24_P130296	A_24_P130296	A_24_P130296	chr17	31852239	31852299	-	COPRS	"coordinator of PRMT5, differentiation stimulator [Source:HGNC Symbol;Acc:HGNC:28848]"	ENSG00000172301	protein_coding	3'UTR;exon	multiple Ref	1	1065	17:31852239:31852299	ENSG00000172301	COPRS	12.06402375	0.00526327	-4.982624887	3.655106234	-1.667299055	8.63773112	3.315325831	-5.322405289	9.961963646	0.000557646	0.004452701	0.126536065	6.13E-06	0.007848669	0.000340941	-0.254884897	-1.819108333	-5.709486454	4.330709623	-2.419026912	0.199258337	25.81600347	5.15E-05

CEP192	-0.209609936	-0.081182535	-0.247799901	0.128427401	-0.038189965	-0.166617366	8	0.061997782	0.816496581	0.816496581	0.816496581	0.816496581	0.816496581	0.816496581	7	46	0	P_230738	P_230738	P_230738	chr18	13068362	13068422	+	CEP192	centrosomal protein 192kDa [Source:HGNC Symbol;Acc:HGNC:25515]	ENSG00000101639	protein_coding	3'UTR;exon	multiple Ref	1	1066	18:13068362:13068422	ENSG00000101639	CEP192	7.472164053	0.005293405	-3.528498559	-1.366597702	-4.171374743	2.161900856	-0.642876185	-2.804777041	9.961963646	0.005492436	0.201800366	0.001929624	0.056030988	0.534820457	0.018705719	-2.609918611	-5.283131266	-1.579723857	-5.056622585	-6.081447002	-3.911738429	7.445356327	0.006647932

KIAA1841	0.200020679	0.096405858	0.183113033	-0.10361482	-0.016907646	0.086707174	8	0.068119074	0.816496581	0.816496581	0.816496581	0.816496581	0.816496581	0.816496581	7	47	0	P_127916	P_127916	P_127916	chr2	61124154	61124214	+	KIAA1841	KIAA1841 [Source:HGNC Symbol;Acc:HGNC:29387]	ENSG00000162929	protein_coding	3'UTR	3'UTR	1	1067	2:61124154:61124214	ENSG00000162929	KIAA1841	5.805981702	0.005933024	3.180405291	1.532890022	2.911567256	-1.647515269	-0.268838035	1.378677234	9.961963646	0.009858298	0.15642358	0.015581773	0.130588191	0.793539823	0.198163093	-3.202616746	-5.075165435	-3.706942572	-5.837945025	-6.258387133	-6.1216404	4.27154748	0.034997735

DLEU2	-0.071035595	0.175997058	-0.030504706	0.247032653	0.040530889	-0.206501764	8	0.078034131	0.816496581	0.816496581	0.816496581	0.816496581	0.816496581	0.816496581	7	48	0	P_219189	P_219189	P_219189	chr13	50027206	50027266	-	DLEU2	deleted in lymphocytic leukemia 2 (non-protein coding) [Source:HGNC Symbol;Acc:HGNC:13748]	ENSG00000231607	antisense	exon	non-coding exon	1	1068	13:50027206:50027266	ENSG00000231607	DLEU2	6.75984009	0.007096744	-1.032742545	2.558712289	-0.443489042	3.591454834	0.589253503	-3.002201331	9.961963646	0.32614468	0.028514652	0.666888621	0.004947109	0.568812374	0.013347643	-6.445682848	-3.562152572	-6.94076459	-2.639620345	-6.115253925	-3.574593932	5.011538102	0.022581202

MYH6	0.167732856	0.281241881	0.232554416	0.113509025	0.06482156	-0.048687464	8	0.111364734	0.816496581	0.816496581	0.816496581	0.816496581	0.816496581	0.816496581	7	49	0	P_220210	P_220210	P_220210	chr14	23393809	23393869	-	MYH6	"myosin, heavy chain 6, cardiac muscle, alpha [Source:HGNC Symbol;Acc:HGNC:7576]"	ENSG00000197616	protein_coding	exon	coding exon	1	1069	14:23393809:23393869	ENSG00000197616	MYH6	4.121176626	0.01216625	1.862453376	3.122822223	2.582211786	1.260368847	0.719758411	-0.540610436	9.961963646	0.092256467	0.010870105	0.027389837	0.236257967	0.488215972	0.600653635	-5.376397359	-2.661030404	-4.265692664	-6.343958526	-6.028295872	-6.914029525	3.714568395	0.049962841

ATR	-0.153053954	0.011964005	-0.113066505	0.165017958	0.039987448	-0.12503051	8	0.057128682	0.816496581	0.816496581	0.816496581	0.816496581	0.816496581	0.816496581	7	50	0	P_246545	P_246545	P_246545	chr3	142513500	142513560	-	ATR	ATR serine/threonine kinase [Source:HGNC Symbol;Acc:HGNC:882]	ENSG00000175054	protein_coding	exon	coding exon	1	1070	3:142513500:142513560	ENSG00000175054	ATR	7.667351026	0.004827602	-2.697891313	0.210890239	-1.993030138	2.908781551	0.704861175	-2.203920376	9.961963646	0.022464895	0.837224249	0.074353994	0.015656151	0.497044998	0.052193265	-4.024733655	-6.15869599	-5.224068002	-3.807828067	-6.039018753	-4.913448925	4.185060962	0.036929006

MT3	0.426747619	-0.134679016	0.253807936	-0.561426636	-0.172939683	0.388486953	8	0.248074308	0.816496581	0.816496581	0.816496581	0.816496581	0.816496581	0.816496581	7	52	0	P_119050	P_119050	P_119050	chr16	56591024	56591084	+	MT3	metallothionein 3 [Source:HGNC Symbol;Acc:HGNC:7408]	ENSG00000087250	protein_coding	3'UTR;exon	multiple Ref	1	1072	16:56591024:56591084	ENSG00000087250	MT3	10.71316445	0.051627352	2.300259383	-0.725948213	1.368078134	-3.026207596	-0.932181249	2.094026347	9.961963646	0.044328588	0.484576426	0.201351589	0.012812376	0.373283638	0.062808067	-4.687146585	-5.911663331	-6.111098461	-3.606618177	-5.854186239	-5.089153217	3.683612076	0.05099825

USP38	-0.031504188	0.034102449	0.010417205	0.065606636	0.041921392	-0.023685244	8	0.062605969	0.816496581	0.816496581	0.816496581	0.816496581	0.816496581	0.816496581	7	53	0	P_251012	P_251012	P_251012	chr4	143206166	143206226	+	USP38	ubiquitin specific peptidase 38 [Source:HGNC Symbol;Acc:HGNC:20067]	ENSG00000170185	protein_coding	3'UTR;exon	multiple Ref	1	1073	4:143206166:143206226	ENSG00000170185	USP38	7.000639976	0.005354262	-0.527307688	0.570796602	0.174360064	1.09810429	0.701667752	-0.396436538	9.961963646	0.609521704	0.580784022	0.865074969	0.297990291	0.498950363	0.700141926	-6.848724669	-6.013194088	-7.030543539	-6.527362507	-6.041291044	-6.986195369	0.414932698	0.746079909

KRT75	-0.039656665	0.115866036	0.118432631	0.155522701	0.158089296	0.002566595	8	0.113960781	0.816496581	0.816496581	0.816496581	0.816496581	0.816496581	0.816496581	7	54	0	P_215515	P_215515	P_215515	chr12	52432005	52432065	-	KRT75	keratin 75 [Source:HGNC Symbol;Acc:HGNC:24431]	ENSG00000170454	protein_coding	exon	coding exon	1	1074	12:52432005:52432065	ENSG00000170454	KRT75	3.955661509	0.012636001	-0.432072864	1.262399912	1.290363837	1.694472776	1.7224367	0.027963925	9.961963646	0.674890994	0.235556396	0.226069412	0.121154632	0.115833326	0.978243192	-6.897437197	-5.405286164	-6.206792329	-5.771028212	-4.92194357	-7.070951343	1.547364581	0.262730117

PTPN13	0.047357831	-0.136807417	-0.010234848	-0.184165248	-0.057592679	0.126572569	8	0.074578224	0.816496581	0.816496581	0.816496581	0.816496581	0.816496581	0.816496581	7	55	0	P_249860	P_249860	P_249860	chr4	86771472	86771532	+	PTPN13	"protein tyrosine phosphatase, non-receptor type 13 (APO-1/CD95 (Fas)-associated phosphatase) [Source:HGNC Symbol;Acc:HGNC:9646]"	ENSG00000163629	protein_coding	exon	coding exon	1	1075	4:86771472:86771532	ENSG00000163629	PTPN13	8.602969835	0.006673202	0.710019529	-2.051106131	-0.15344752	-2.76112566	-0.863467049	1.897658611	9.961963646	0.493976739	0.067489905	0.881109171	0.020157954	0.408188738	0.087069828	-6.730051235	-4.347231834	-7.034277099	-4.059967502	-5.914731859	-5.394057053	2.78420145	0.096058859

PDCD4	0.019416284	-0.026372724	-0.043623347	-0.045789007	-0.063039631	-0.017250623	8	0.040421089	0.816496581	0.816496581	0.816496581	0.816496581	0.816496581	0.816496581	7	56	0	P_209863	P_209863	P_209863	chr10	110881343	110881403	+	PDCD4	programmed cell death 4 (neoplastic transformation inhibitor) [Source:HGNC Symbol;Acc:HGNC:8763]	ENSG00000150593	protein_coding	exon	coding exon	1	1076	10:110881343:110881403	ENSG00000150593	PDCD4	9.51278734	0.003518766	0.400881979	-0.544509434	-0.900677698	-0.945391412	-1.301559677	-0.356168264	9.961963646	0.696970181	0.598067252	0.389014574	0.36682441	0.222360412	0.729141999	-6.911369669	-6.028162163	-6.621346479	-6.68166455	-5.466825373	-7.002517368	0.663849258	0.593057841

BNIP3	0.085958851	0.105729131	0.014367777	0.01977028	-0.071591075	-0.091361354	8	0.183064238	0.816496581	0.816496581	0.816496581	0.816496581	0.816496581	0.816496581	7	60	0	P_308686	P_308686	P_308686	chr10	131969733	131969793	+	BNIP3;RP11-45A17.2	BCL2/adenovirus E1B 19kDa interacting protein 3 [Source:HGNC Symbol;Acc:HGNC:1084];	ENSG00000176171	protein_coding;antisense	3'UTR;intron;promoter	multiple non-Ref	0	1080	10:131969733:131969793	ENSG00000176171	BNIP3	7.601027186	0.029119061	0.616946563	0.758842435	0.103120858	0.141895871	-0.513825705	-0.655721576	9.961963646	0.551108839	0.46552218	0.919913408	0.889990864	0.61857745	0.526856261	-6.794558567	-5.887322495	-7.041316435	-7.138613392	-6.158147615	-6.841446674	0.280203482	0.83852165

TRIML1	0.052912483	-0.065261753	0.038198684	-0.118174236	-0.014713799	0.103460437	8	0.228063094	0.816496581	0.816496581	0.816496581	0.816496581	0.816496581	0.816496581	7	63	0	P_139082	P_139082	P_139082	chr4	188147202	188147262	+	TRIML1	tripartite motif family-like 1 [Source:HGNC Symbol;Acc:HGNC:26698]	ENSG00000184108	protein_coding	exon	coding exon	1	1083	4:188147202:188147262	ENSG00000184108	TRIML1	3.404254605	0.043975778	0.309027412	-0.38115147	0.223093679	-0.690178882	-0.085933734	0.604245148	9.961963646	0.763666905	0.711093467	0.827970569	0.505842239	0.93322171	0.55918958	-6.94649806	-6.106077987	-7.020008439	-6.895187739	-6.292758987	-6.875509947	0.189898145	0.900865278

POP1	-0.319006227	0.046304169	-0.180867988	0.365310396	0.138138239	-0.227172157	8	0.048132272	0.816496581	0.816496581	0.816496581	0.816496581	0.816496581	0.816496581	7	64	0	P_265530	P_265530	P_265530	chr8	98123419	98123479	+	POP1	"processing of precursor 1, ribonuclease P/MRP subunit (S. cerevisiae) [Source:HGNC Symbol;Acc:HGNC:30129]"	ENSG00000104356	protein_coding	exon	coding exon	1	1084	8:98123419:98123479	ENSG00000104356	POP1	8.530355042	0.004067133	-6.126333119	0.889245223	-3.473466815	7.015578342	2.652866304	-4.362712038	9.961963646	0.000113557	0.394837214	0.006020144	3.72E-05	0.024266955	0.001427921	1.395692069	-5.781597247	-2.747019777	2.45525508	-3.494872067	-1.289229882	20.94616362	0.000126753

GABRQ	-0.110950455	-0.392100531	-0.173209791	-0.281150076	-0.062259336	0.21889074	8	0.166006049	0.816496581	0.816496581	0.816496581	0.816496581	0.816496581	0.816496581	7	65	0	A_23_P125475	A_23_P125475	A_23_P125475	chrX	152653005	152653065	+	GABRQ	"gamma-aminobutyric acid (GABA) A receptor, theta [Source:HGNC Symbol;Acc:HGNC:14454]"	ENSG00000268089	protein_coding	exon	coding exon	1	1085	X:152653005:152653065	ENSG00000268089	GABRQ	3.895501095	0.024337267	-0.871041429	-3.078273148	-1.359822306	-2.207231719	-0.488780877	1.718450842	9.961963646	0.404233707	0.011725327	0.203865019	0.051901839	0.635578117	0.116578631	-6.599467937	-2.732266569	-6.121447736	-4.983605878	-6.171163595	-5.659884396	3.357907892	0.063596355

GLIS3	0.012520731	0.118004509	-0.037908543	0.105483778	-0.050429274	-0.155913052	8	0.135708125	0.816496581	0.816496581	0.816496581	0.816496581	0.816496581	0.816496581	7	66	0	P_314314	P_314314	P_314314	chr9	3857635	3857695	+	GLIS3	GLIS family zinc finger 3 [Source:HGNC Symbol;Acc:HGNC:28510]	ENSG00000107249	protein_coding	intron	intron antisense	0	1086	9:3857635:3857695	ENSG00000107249	GLIS3	3.880889035	0.016996294	0.117624604	1.108580177	-0.356127576	0.990955572	-0.47375218	-1.464707752	9.961963646	0.908702874	0.293657037	0.729171539	0.345165853	0.645888322	0.17383349	-6.990815648	-5.572712708	-6.978299586	-6.637592014	-6.178676987	-6.010717624	0.786902547	0.528253957

SLC13A1	-0.241852277	0.031403777	-0.065850685	0.273256055	0.176001592	-0.097254463	8	0.220460559	0.816496581	0.816496581	0.816496581	0.816496581	0.816496581	0.816496581	7	67	0	P_262733	P_262733	P_262733	chr7	123199863	123199923	-	SLC13A1	"solute carrier family 13 (sodium/sulfate symporter), member 1 [Source:HGNC Symbol;Acc:HGNC:10916]"	ENSG00000081800	protein_coding	exon	coding exon	1	1087	7:123199863:123199923	ENSG00000081800	SLC13A1	3.717029869	0.041237429	-1.458646381	0.189400764	-0.397155093	1.648047145	1.061491288	-0.586555857	9.961963646	0.175459476	0.853582013	0.699628838	0.130477748	0.313522606	0.57055361	-5.946956554	-6.163211768	-6.961661283	-5.837193004	-5.729957425	-6.886619211	1.0846875	0.399680133

PLB1	-0.110608804	0.071424977	-0.000763735	0.182033781	0.109845069	-0.072188712	8	0.103765095	0.816496581	0.816496581	0.816496581	0.816496581	0.816496581	0.816496581	7	68	0	P_234170	P_234170	P_234170	chr2	28589490	28589550	+	PLB1	phospholipase B1 [Source:HGNC Symbol;Acc:HGNC:30041]	ENSG00000163803	protein_coding	exon	coding exon	1	1088	2:28589490:28589550	ENSG00000163803	PLB1	3.836697624	0.010853329	-1.300330795	0.839680872	-0.008978568	2.140011667	1.291352227	-0.84865944	9.961963646	0.222765047	0.420781519	0.993013495	0.058136154	0.225739933	0.415997387	-6.146684414	-5.823486142	-7.047076578	-5.091688532	-5.478803289	-6.691586816	1.560578019	0.259685717

ZNF160	0.090029706	-0.076021814	0.036693942	-0.16605152	-0.053335764	0.112715756	8	0.169501029	0.816496581	0.816496581	0.816496581	0.816496581	0.816496581	0.816496581	7	69	0	P_126680	P_126680	P_126680	chr19	53108312	53108372	+	ZNF160;ZNF415	zinc finger protein 160 [Source:HGNC Symbol;Acc:HGNC:12948];zinc finger protein 415 [Source:HGNC Symbol;Acc:HGNC:20636]	ENSG00000170949	protein_coding	3'ext;3'UTR;promoter	multiple non-Ref	0	1089	19:53108312:53108372	ENSG00000170949	ZNF160	7.198568292	0.025278921	0.693509143	-0.585604747	0.282657642	-1.27911389	-0.410851501	0.868262389	9.961963646	0.503838553	0.571168218	0.783225233	0.229847569	0.689879415	0.405681721	-6.742095835	-6.004472124	-7.003664264	-6.321574429	-6.207680526	-6.674475617	0.577099011	0.643141656

AGPAT2	0.28604115	0.064676658	0.319444822	-0.221364492	0.033403672	0.254768164	8	0.05220507	0.816496581	0.816496581	0.816496581	0.816496581	0.816496581	0.816496581	7	70	0	P_269359	P_269359	P_269359	chr9	136670888	136670948	+	AGPAT2;EGFL7	"1-acylglycerol-3-phosphate O-acyltransferase 2 [Source:HGNC Symbol;Acc:HGNC:325];EGF-like-domain, multiple 7 [Source:HGNC Symbol;Acc:HGNC:20594]"	ENSG00000169692	protein_coding	3'ext;intron	multiple non-Ref	0	1090	9:136670888:136670948	ENSG00000169692	AGPAT2	9.878096562	0.004395304	5.284205234	1.194809678	5.901290774	-4.089395555	0.61708554	4.706481095	9.961963646	0.000360035	0.259831676	0.0001531	0.002198658	0.55102079	0.000842016	0.198802693	-5.480834274	1.051307233	-1.804340727	-6.098046326	-0.741415219	17.2384238	0.000286106

MYO1E	-0.116488559	0.04763474	-0.09231131	0.164123299	0.024177249	-0.139946049	8	0.079625787	0.816496581	0.816496581	0.816496581	0.816496581	0.816496581	0.816496581	7	71	0	P_223906	P_223906	P_223906	chr15	59261419	59261479	-	MYO1E	myosin IE [Source:HGNC Symbol;Acc:HGNC:7599]	ENSG00000157483	protein_coding	exon	coding exon	1	1091	15:59261419:59261479	ENSG00000157483	MYO1E	5.916068243	0.007298263	-1.67001026	0.682904013	-1.323398932	2.352914273	0.346611329	-2.006302944	9.961963646	0.125989642	0.510236068	0.215270351	0.040529944	0.736093109	0.072729434	-5.658035529	-5.942051047	-6.166591073	-4.745679312	-6.233179666	-5.226926029	2.448045564	0.124189614

ARL15	0.274344609	-0.016624263	0.253293907	-0.290968872	-0.021050702	0.26991817	8	0.062653478	0.816496581	0.816496581	0.816496581	0.816496581	0.816496581	0.816496581	7	75	0	A_16_P17119490	A_16_P17119490	A_16_P17119490	chr5	54047481	54047541	+	ARL15	ADP-ribosylation factor-like 15 [Source:HGNC Symbol;Acc:HGNC:25945]	ENSG00000185305	protein_coding	intron	intron antisense	0	1095	5:54047481:54047541	ENSG00000185305	ARL15	6.469159153	0.005359041	4.589850251	-0.278127863	4.237667028	-4.867978114	-0.352183223	4.515794891	9.961963646	0.001005379	0.786601399	0.001737482	0.000660817	0.73203738	0.001126324	-0.865170924	-6.141472103	-1.471426947	-0.554672303	-6.231134614	-1.043360062	13.88592495	0.000683764

RMDN2	0.099455003	0.024350086	-0.043892345	-0.075104917	-0.143347348	-0.06824243	8	0.094342913	0.816496581	0.816496581	0.816496581	0.816496581	0.816496581	0.816496581	7	76	0	P_127642	P_127642	P_127642	chr2	38067073	38067133	+	RMDN2	regulator of microtubule dynamics 2 [Source:HGNC Symbol;Acc:HGNC:26567]	ENSG00000115841	protein_coding	3'UTR;exon	multiple Ref	1	1096	2:38067073:38067133	ENSG00000115841	RMDN2	5.607944771	0.009354339	1.259405852	0.308346886	-0.555811915	-0.951058967	-1.815217767	-0.8641588	9.961963646	0.236591201	0.76416953	0.590603014	0.364078026	0.099665668	0.407826431	-6.195709769	-6.132212716	-6.880994499	-6.676276237	-4.790478468	-6.678085415	1.15606782	0.374134699

H2AC14	-0.094982216	-0.17042997	-0.188473898	-0.075447755	-0.093491682	-0.018043927	8	0.17429313	0.816496581	0.816496581	0.816496581	0.816496581	0.816496581	0.816496581	7	78	0	P_141897	P_141897	P_141897	chr6	27815429	27815489	+	HIST1H2AJ;HIST1H3H	"histone cluster 1, H2aj [Source:HGNC Symbol;Acc:HGNC:4727];histone cluster 1, H3h [Source:HGNC Symbol;Acc:HGNC:4775]"	ENSG00000276368	protein_coding	3'ext;promoter	multiple non-Ref	0	1098	6:27815429:27815489	ENSG00000276368	H2AC14	11.19389647	0.026601951	-0.713232597	-1.279778633	-1.415272598	-0.566546036	-0.702040001	-0.135493965	9.961963646	0.492071498	0.229622888	0.187480708	0.583560294	0.498728028	0.894920058	-6.727677487	-5.385381183	-6.051127359	-6.976868103	-6.041026649	-7.061358445	0.8302873	0.507115819

C5orf63	-0.10069713	0.070950629	-0.127710166	0.171647759	-0.027013036	-0.198660795	8	0.116894921	0.816496581	0.816496581	0.816496581	0.816496581	0.816496581	0.816496581	7	80	0	P_140212	P_140212	P_140212	chr5	127058881	127058941	-	C5orf63	chromosome 5 open reading frame 63 [Source:HGNC Symbol;Acc:HGNC:40051]	ENSG00000164241	protein_coding	exon	coding exon	1	1100	5:127058881:127058941	ENSG00000164241	C5orf63	7.845536423	0.01317996	-1.07425076	0.75691102	-1.362429526	1.83116178	-0.288178766	-2.119340546	9.961963646	0.308040954	0.466627533	0.203068433	0.097105459	0.779116573	0.060193597	-6.402782507	-5.888778171	-6.11818402	-5.570574565	-6.252702857	-5.04897046	1.918491336	0.19080692

ARID1A	-0.045544387	-0.094746209	-0.116233199	-0.049201822	-0.070688811	-0.021486989	8	0.102932298	0.816496581	0.816496581	0.816496581	0.816496581	0.816496581	0.816496581	7	81	0	P_200782	P_200782	P_200782	chr1	26775647	26775707	+	ARID1A	AT rich interactive domain 1A (SWI-like) [Source:HGNC Symbol;Acc:HGNC:11110]	ENSG00000117713	protein_coding	3'UTR;exon	multiple Ref	1	1101	1:26775647:26775707	ENSG00000117713	ARID1A	7.332213009	0.010715093	-0.53886815	-1.121010023	-1.375237927	-0.582141873	-0.836369777	-0.254227903	9.961963646	0.60181126	0.288579109	0.199193026	0.573409004	0.422555194	0.804490016	-6.842186841	-5.559790874	-6.102088633	-6.967387087	-5.937519338	-7.036184784	0.75689285	0.543390295

GCNT7	-0.028813044	0.245320554	-0.113614828	0.274133598	-0.084801784	-0.358935382	8	0.367886086	0.816496581	0.816496581	0.816496581	0.816496581	0.816496581	0.816496581	7	82	0	P_241148	P_241148	P_241148	chr20	56496680	56496740	-	GCNT7	glucosaminyl (N-acetyl) transferase family member 7 [Source:HGNC Symbol;Acc:HGNC:16099]	ENSG00000124091	protein_coding	exon	coding exon	1	1102	20:56496680:56496740	ENSG00000124091	GCNT7	3.525606995	0.110892222	-0.105970317	0.902254431	-0.417859329	1.008224749	-0.311889012	-1.32011376	9.961963646	0.917709409	0.388216294	0.684913869	0.337207719	0.761554641	0.216324713	-6.992235833	-5.770265029	-6.952598392	-6.62044343	-6.245205436	-6.19454241	0.642731904	0.604913959

ITFG2	-0.056166274	-0.148989133	-0.134965357	-0.092822859	-0.078799083	0.014023776	8	0.068973235	0.816496581	0.816496581	0.816496581	0.816496581	0.816496581	0.816496581	7	83	0	P_110641	P_110641	P_110641	chr12	2812625	2812685	+	ITFG2	integrin alpha FG-GAP repeat containing 2 [Source:HGNC Symbol;Acc:HGNC:30879]	ENSG00000111203	protein_coding	5'UTR	5'UTR	1	1103	12:2812625:2812685	ENSG00000111203	ITFG2	6.441418501	0.006027061	-0.886070852	-2.350430571	-2.129193554	-1.464359719	-1.243122701	0.221237017	9.961963646	0.396464704	0.040701759	0.059204361	0.173926498	0.242284118	0.829376705	-6.586101892	-3.8896304	-5.009896739	-6.08820401	-5.534492469	-7.044705191	2.430293311	0.125927325

IMP3	-0.045172804	0.05902392	-0.148068844	0.104196724	-0.10289604	-0.207092764	8	0.076665455	0.816496581	0.816496581	0.816496581	0.816496581	0.816496581	0.816496581	7	84	0	A_23_P33809	A_23_P33809	A_23_P33809	chr15	75639365	75639425	-	IMP3	"IMP3, U3 small nucleolar ribonucleoprotein [Source:HGNC Symbol;Acc:HGNC:14497]"	ENSG00000177971	protein_coding	3'UTR	3'UTR	1	1104	15:75639365:75639425	ENSG00000177971	IMP3	10.82468652	0.006926711	-0.664751228	0.868580647	-2.178942581	1.533331875	-1.514191353	-3.047523228	9.961963646	0.521299967	0.405515711	0.054442893	0.156315909	0.161039984	0.012355433	-6.762459704	-5.799310923	-4.930321252	-5.996073994	-5.203170514	-3.497021309	3.312566204	0.065629654

MARK1	0.185319857	0.032960866	0.310306135	-0.152358992	0.124986278	0.277345269	8	0.052420036	0.816496581	0.816496581	0.816496581	0.816496581	0.816496581	0.816496581	7	85	0	P_104832	P_104832	P_104832	chr1	220662258	220662318	+	MARK1	MAP/microtubule affinity-regulating kinase 1 [Source:HGNC Symbol;Acc:HGNC:6896]	ENSG00000116141	protein_coding	3'UTR	3'UTR	1	1105	1:220662258:220662318	ENSG00000116141	MARK1	6.162443456	0.004413366	3.416509525	0.607658095	5.720724595	-2.80885143	2.30421507	5.1130665	9.961963646	0.006621841	0.557011711	0.000195549	0.018575661	0.044031559	0.000460763	-2.800037219	-5.991098131	0.796633024	-3.97861503	-4.051511991	-0.114390464	14.01849634	0.0006586

APPL1	0.25273321	-0.049309407	0.102345058	-0.302042618	-0.150388153	0.151654465	8	0.065669809	0.816496581	0.816496581	0.816496581	0.816496581	0.816496581	0.816496581	7	86	0	A_23_P166663	A_23_P166663	A_23_P166663	chr3	57272376	57272436	+	APPL1	"adaptor protein, phosphotyrosine interaction, PH domain and leucine zipper containing 1 [Source:HGNC Symbol;Acc:HGNC:24035]"	ENSG00000157500	protein_coding	3'UTR	3'UTR	1	1106	3:57272376:57272436	ENSG00000157500	APPL1	9.438161954	0.005669876	4.110751032	-0.802026363	1.664660736	-4.912777395	-2.446090296	2.466687099	9.961963646	0.002124976	0.441249807	0.127069842	0.000618266	0.034573128	0.033377341	-1.637420929	-5.853923007	-5.713233364	-0.485217823	-3.826920512	-4.482103077	9.419316736	0.002950542

GBP7	0.100338637	-0.132250034	0.324163039	-0.232588671	0.223824402	0.456413073	8	0.325804533	0.816496581	0.816496581	0.816496581	0.816496581	0.816496581	0.816496581	7	87	0	P_102340	P_102340	P_102340	chr1	89132167	89132227	-	GBP7	guanylate binding protein 7 [Source:HGNC Symbol;Acc:HGNC:29606]	ENSG00000213512	protein_coding	exon	coding exon	1	1107	1:89132167:89132227	ENSG00000213512	GBP7	3.29547636	0.087449793	0.415560669	-0.547724326	1.342547724	-0.963284995	0.926987055	1.89027205	9.961963646	0.686540914	0.595939075	0.20920951	0.358204242	0.375845571	0.088135048	-6.904938436	-6.026367102	-6.142963549	-6.664561452	-5.858899023	-5.405267167	1.272579734	0.336218073

SPIN1	0.001557366	-0.131665008	-0.130064018	-0.133222374	-0.131621384	0.00160099	8	0.13515768	0.816496581	0.816496581	0.816496581	0.816496581	0.816496581	0.816496581	7	88	0	P_267855	P_267855	P_267855	chr9	88462689	88462749	+	SPIN1	spindlin 1 [Source:HGNC Symbol;Acc:HGNC:11243]	ENSG00000106723	protein_coding	exon	coding exon	1	1108	9:88462689:88462749	ENSG00000106723	SPIN1	7.208774107	0.016876562	0.014682305	-1.24129217	-1.226198589	-1.255974475	-1.240880894	0.015093581	9.961963646	0.988575521	0.242931019	0.24831861	0.237781716	0.243076555	0.988255529	-6.998242684	-5.429201273	-6.282787591	-6.349171478	-5.537044077	-7.07125407	1.027011649	0.421709943

ACTR3	-0.270089387	0.139197571	-0.128145052	0.409286958	0.141944336	-0.267342623	8	0.059723176	0.816496581	0.816496581	0.816496581	0.816496581	0.816496581	0.816496581	7	89	0	P_236281	P_236281	P_236281	chr2	113909896	113909956	+	ACTR3	ARP3 actin-related protein 3 homolog (yeast) [Source:HGNC Symbol;Acc:HGNC:170]	ENSG00000115091	protein_coding	intron	intron sense	0	1109	2:113909896:113909956	ENSG00000115091	ACTR3	8.002016168	0.005071065	-4.645190706	2.394019511	-2.20393037	7.039210217	2.441260337	-4.59794988	9.961963646	0.000924027	0.03778731	0.052192383	3.61E-05	0.03485959	0.000993012	-0.777889064	-3.821572705	-4.890110263	2.484858262	-3.834617018	-0.912671704	18.1363018	0.00023199

HNF4A	-0.124749257	-0.065205872	0.091829729	0.059543385	0.216578986	0.157035601	8	0.146098783	0.816496581	0.816496581	0.816496581	0.816496581	0.816496581	0.816496581	7	90	0	P_131330	P_131330	P_131330	chr20	44429858	44429918	+	HNF4A	"hepatocyte nuclear factor 4, alpha [Source:HGNC Symbol;Acc:HGNC:5024]"	ENSG00000101076	protein_coding	3'UTR	3'UTR	1	1110	20:44429858:44429918	ENSG00000101076	HNF4A	4.666170425	0.019347766	-1.098419372	-0.574138835	0.808562355	0.524280537	1.906981727	1.38270119	9.961963646	0.297859243	0.578606064	0.437650242	0.611549082	0.085742454	0.196963798	-6.377188029	-6.011243726	-6.701398546	-7.001344515	-4.657231019	-6.116551969	1.335540928	0.317524636

SIRT3	0.13956863	0.114173255	0.071031523	-0.025395375	-0.068537107	-0.043141733	8	0.042987971	0.816496581	0.816496581	0.816496581	0.816496581	0.816496581	0.816496581	7	91	0	P_210528	P_210528	P_210528	chr11	214772	214832	-	SIRT3;RIC8A	sirtuin 3 [Source:HGNC Symbol;Acc:HGNC:14931];RIC8 guanine nucleotide exchange factor A [Source:HGNC Symbol;Acc:HGNC:29550]	ENSG00000142082	protein_coding	3'ext;3'UTR	multiple non-Ref	0	1111	11:214772:214832	ENSG00000142082	SIRT3	9.049098539	0.003690701	2.813707865	2.301736335	1.431997681	-0.51197153	-1.381710184	-0.869738654	9.961963646	0.018421828	0.044217457	0.182764279	0.619828192	0.197258585	0.40491207	-3.828320162	-3.96529869	-6.029550247	-7.008135755	-5.370530413	-6.673173402	3.032207112	0.080063918

PKN2-AS1	0.616412128	0.369069217	0.340732529	-0.247342911	-0.275679599	-0.028336688	8	0.23309365	0.816496581	0.816496581	0.816496581	0.816496581	0.816496581	0.816496581	7	92	0	A_24_P618247	A_24_P618247	A_24_P618247	chr1	88540472	88540532	-	PKN2-AS1	PKN2 antisense RNA 1 [Source:HGNC Symbol;Acc:HGNC:50597]	ENSG00000237505	antisense	exon	non-coding exon	1	1112	1:88540472:88540532	ENSG00000237505	PKN2-AS1	3.974144579	0.045838764	3.526146513	2.111237067	1.949138837	-1.414909446	-1.577007676	-0.162098231	9.961963646	0.005513979	0.061019006	0.079972846	0.187584258	0.14598886	0.874469154	-2.61390405	-4.25691768	-5.291850095	-6.152574393	-5.120530729	-7.057042975	4.200895196	0.036566103

ALS2	0.028030582	0.019834291	0.047187835	-0.008196291	0.019157253	0.027353544	8	0.052710859	0.816496581	0.816496581	0.816496581	0.816496581	0.816496581	0.816496581	7	94	0	P_238730	P_238730	P_238730	chr2	201746565	201746625	-	ALS2	amyotrophic lateral sclerosis 2 (juvenile) [Source:HGNC Symbol;Acc:HGNC:443]	ENSG00000003393	protein_coding	exon	coding exon	1	1114	2:201746565:201746625	ENSG00000003393	ALS2	6.324225447	0.004437919	0.515333157	0.364647007	0.867533028	-0.15068615	0.352199871	0.502886021	9.961963646	0.617561533	0.722996561	0.406062345	0.883230777	0.732025275	0.625975213	-6.855355276	-6.112473836	-6.651005727	-7.137209954	-6.231128456	-6.934958148	0.258465779	0.853659092

AZI2	0.100124229	0.075966215	0.03450684	-0.024158013	-0.065617389	-0.041459375	8	0.058822127	0.816496581	0.816496581	0.816496581	0.816496581	0.816496581	0.816496581	7	95	0	P_134272	P_134272	P_134272	chr3	28324044	28324104	-	AZI2	5-azacytidine induced 2 [Source:HGNC Symbol;Acc:HGNC:24002]	ENSG00000163512	protein_coding	exon	coding exon	1	1115	3:28324044:28324104	ENSG00000163512	AZI2	9.350366306	0.004985287	1.736759701	1.317713634	0.598557312	-0.419046066	-1.138202389	-0.719156323	9.961963646	0.113190666	0.217097736	0.562829699	0.68407453	0.281668664	0.488570902	-5.562169458	-5.341271222	-6.854926826	-7.054425109	-5.650255947	-6.795978036	1.183210739	0.364896001

MRPL39	-0.191709333	0.109597842	0.06230889	0.301307175	0.254018223	-0.047288953	8	0.060727919	0.816496581	0.816496581	0.816496581	0.816496581	0.816496581	0.816496581	7	96	0	P_241443	P_241443	P_241443	chr21	25603825	25603885	-	MRPL39	mitochondrial ribosomal protein L39 [Source:HGNC Symbol;Acc:HGNC:14027]	ENSG00000154719	protein_coding	exon	coding exon	1	1116	21:25603825:25603885	ENSG00000154719	MRPL39	10.85567288	0.005168253	-3.266006227	1.86713516	1.061509208	5.133141387	4.327515435	-0.805625952	9.961963646	0.008529337	0.091550559	0.313514856	0.000447524	0.001508675	0.439264985	-3.056465397	-4.617088466	-6.464384272	-0.147690182	-0.831665184	-6.727959563	10.16755928	0.002237552

FMO2	-0.068208935	-0.183473727	-0.157363772	-0.115264792	-0.089154836	0.026109955	8	0.103877852	0.816496581	0.816496581	0.816496581	0.816496581	0.816496581	0.816496581	7	97	0	P_204868	P_204868	P_204868	chr1	171193419	171193479	+	FMO2	flavin containing monooxygenase 2 (non-functional) [Source:HGNC Symbol;Acc:HGNC:3770]	ENSG00000094963	protein_coding	exon	coding exon	1	1117	1:171193419:171193479	ENSG00000094963	FMO2	3.606693662	0.010872131	-0.801179018	-2.155073963	-1.848387628	-1.353894945	-1.04720861	0.306686335	9.961963646	0.441717898	0.056679581	0.094407716	0.205685789	0.319746308	0.765396466	-6.658997845	-4.190509379	-5.44484144	-6.229959499	-5.744315222	-7.020237884	1.954417055	0.185143463

DLG3	0.207735622	-0.03662707	0.243984058	-0.244362692	0.036248436	0.280611128	8	0.04595596	0.816496581	0.816496581	0.816496581	0.816496581	0.816496581	0.816496581	7	98	0	P_151141	P_151141	P_151141	chrX	70504017	70504077	-	DLG3	"discs, large homolog 3 (Drosophila) [Source:HGNC Symbol;Acc:HGNC:2902]"	ENSG00000082458	protein_coding	3'UTR	3'UTR antisense	0	1118	X:70504017:70504077	ENSG00000082458	DLG3	9.038330966	0.003902695	4.072623749	-0.71806787	4.783268551	-4.790691619	0.710644802	5.501336421	9.961963646	0.002258407	0.489212952	0.000750031	0.000741723	0.493605616	0.000264868	-1.700076349	-5.917351434	-0.601312584	-0.675149355	-6.034879722	0.462327287	15.61699806	0.000427877

DNAJC3-DT	0.39503554	0.011410532	0.240423218	-0.383625008	-0.154612323	0.229012686	8	0.079397756	0.816496581	0.816496581	0.816496581	0.816496581	0.816496581	0.816496581	7	99	0	A_32_P64716	A_32_P64716	A_32_P64716	chr13	95672868	95672928	-	DNAJC3-AS1	DNAJC3 antisense RNA 1 (head to head) [Source:HGNC Symbol;Acc:HGNC:39808]	ENSG00000247400	lincRNA	exon	non-coding exon	1	1119	13:95672868:95672928	ENSG00000247400	DNAJC3-DT	7.515566544	0.007269143	5.67466469	0.16391169	3.453666834	-5.510752999	-2.220997855	3.289755144	9.961963646	0.000208296	0.873078475	0.006222624	0.000261405	0.050707046	0.0081943	0.766546961	-6.167943743	-2.780700663	0.414573204	-4.181584383	-3.082405433	15.04670333	0.000496941

TRIM47	-0.303735332	0.089314267	-0.180851469	0.393049598	0.122883863	-0.270165735	8	0.061261066	0.816496581	0.816496581	0.816496581	0.816496581	0.816496581	0.816496581	7	100	0	A_23_P26697	A_23_P26697	A_23_P26697	chr17	75874473	75874533	-	TRIM47	tripartite motif containing 47 [Source:HGNC Symbol;Acc:HGNC:19020]	ENSG00000132481	protein_coding	"3'UTR,exon;exon"	multiple Ref	1	1120	17:75874473:75874533	ENSG00000132481	TRIM47	9.752321016	0.005220482	-5.148558411	1.513948724	-3.065578001	6.662507136	2.08298041	-4.579526726	9.961963646	0.000437633	0.161100658	0.011981397	5.72E-05	0.063982536	0.001021379	-0.003631873	-5.099602262	-3.443656713	2.004290153	-4.393944034	-0.94190027	17.98288945	0.000240303

EIF3A	-0.217397758	-0.140248235	0.009726414	0.077149523	0.227124171	0.149974649	8	0.133018425	0.816496581	0.816496581	0.816496581	0.816496581	0.816496581	0.816496581	7	101	0	P_210143	P_210143	P_210143	chr10	119069445	119069505	-	EIF3A	"eukaryotic translation initiation factor 3, subunit A [Source:HGNC Symbol;Acc:HGNC:3271]"	ENSG00000107581	protein_coding	exon	coding exon	1	1121	10:119069445:119069505	ENSG00000107581	EIF3A	11.00503435	0.016415852	-2.078112146	-1.340637381	0.0929751	0.737474765	2.171087246	1.433612481	9.961963646	0.064506774	0.209807683	0.927766594	0.477844399	0.055169471	0.18231433	-5.043933111	-5.314186053	-7.042401886	-6.860071576	-4.258900388	-6.051319504	2.239539442	0.1465224

HMGB4	0.134220891	0.149470259	0.047218021	0.015249368	-0.08700287	-0.102252239	8	0.26914971	0.816496581	0.816496581	0.816496581	0.816496581	0.816496581	0.816496581	7	102	0	A_23_P11697	A_23_P11697	A_23_P11697	chr1	33864363	33864423	+	HMGB4	high mobility group box 4 [Source:HGNC Symbol;Acc:HGNC:24954]	ENSG00000176256	protein_coding	exon	coding exon	1	1122	1:33864363:33864423	ENSG00000176256	HMGB4	4.441084711	0.060381212	0.66898262	0.744988391	0.235343656	0.076005771	-0.433638964	-0.509644735	9.961963646	0.518708269	0.473488088	0.818709302	0.940919242	0.673790659	0.621399543	-6.759512743	-5.897691139	-7.016957789	-7.146454804	-6.197631088	-6.931314012	0.251914911	0.858215097

EMC7	-0.053734287	-0.113252767	0.022016006	-0.05951848	0.075750293	0.135268773	8	0.082564319	0.816496581	0.816496581	0.816496581	0.816496581	0.816496581	0.816496581	7	103	0	P_222654	P_222654	P_222654	chr15	34090316	34090376	-	EMC7	ER membrane protein complex subunit 7 [Source:HGNC Symbol;Acc:HGNC:24301]	ENSG00000134153	protein_coding	exon	coding exon	1	1123	15:34090316:34090376	ENSG00000134153	EMC7	10.45621325	0.007681	-0.750910627	-1.582652541	0.307663014	-0.831741914	1.058573641	1.890315555	9.961963646	0.470072302	0.144699052	0.764674742	0.425042704	0.314786439	0.088128739	-6.699124105	-5.010109079	-6.995673556	-6.783927392	-5.732902922	-5.405201202	1.424829671	0.292985578

TMEM135	0.028969574	0.099996103	0.227085547	0.071026529	0.198115973	0.127089444	8	0.065187475	0.816496581	0.816496581	0.816496581	0.816496581	0.816496581	0.816496581	7	106	0	P_314718	P_314718	P_314718	chr11	87328248	87328308	+	TMEM135;RP11-777F6.3	transmembrane protein 135 [Source:HGNC Symbol;Acc:HGNC:26167];	ENSG00000166575	protein_coding;processed_pseudogene	3'ext;promoter	multiple non-Ref	0	1126	11:87328248:87328308	ENSG00000166575	TMEM135	7.257744537	0.005619189	0.473315684	1.633773575	3.710208253	1.160457891	3.236892569	2.076434678	9.961963646	0.646188967	0.133470797	0.004066261	0.272916652	0.008959351	0.064688357	-6.877475529	-4.942035955	-2.346255076	-6.459145545	-2.546050858	-5.116968735	5.465767684	0.017558232

AQR	0.033819427	-0.001879562	0.025422867	-0.03569899	-0.008396561	0.027302429	8	0.037455657	0.816496581	0.816496581	0.816496581	0.816496581	0.816496581	0.816496581	7	107	0	P_222689	P_222689	P_222689	chr15	34874716	34874776	-	AQR	aquarius intron-binding spliceosomal factor [Source:HGNC Symbol;Acc:HGNC:29513]	ENSG00000021776	protein_coding	3'UTR;exon	multiple Ref	1	1127	15:34874716:34874776	ENSG00000021776	AQR	8.145272202	0.00333331	0.717420879	-0.039871672	0.53930231	-0.757292551	-0.178118569	0.579173982	9.961963646	0.489594844	0.968983187	0.601522683	0.466409049	0.862199796	0.575333323	-6.724568762	-6.181249423	-6.89057744	-6.844737208	-6.279838208	-6.891164167	0.291298364	0.830794668

SNX31	0.013226398	-0.142953483	0.010346729	-0.156179881	-0.002879669	0.153300212	8	0.121828068	0.816496581	0.816496581	0.816496581	0.816496581	0.816496581	0.816496581	7	108	0	P_313920	P_313920	P_313920	chr8	100629747	100629807	-	SNX31	sorting nexin 31 [Source:HGNC Symbol;Acc:HGNC:28605]	ENSG00000174226	protein_coding	intron	intron sense	0	1128	8:100629747:100629807	ENSG00000174226	SNX31	3.552978965	0.014125682	0.13629581	-1.473111629	0.106621302	-1.609407439	-0.029674508	1.579732931	9.961963646	0.89430241	0.171600638	0.917205988	0.138722065	0.976912722	0.145364897	-6.988232757	-5.151654359	-7.040915931	-5.891462212	-6.296216912	-5.855796481	1.214438589	0.354577477

FAM110B	-0.123240484	-0.163993624	-0.122413333	-0.04075314	0.000827151	0.041580291	8	0.146384067	0.816496581	0.816496581	0.816496581	0.816496581	0.816496581	0.816496581	7	109	0	A_24_P418908	A_24_P418908	A_24_P418908	chr8	58099085	58099145	+	FAM110B;RP11-648L3.2	"family with sequence similarity 110, member B [Source:HGNC Symbol;Acc:HGNC:28587];"	ENSG00000169122	protein_coding;lincRNA	"exon,intron;intron"	multiple non-Ref	0	1129	8:58099085:58099145	ENSG00000169122	FAM110B	6.211337946	0.019414773	-1.083260382	-1.441472722	-1.075989887	-0.35821234	0.007270496	0.365482835	9.961963646	0.304214639	0.180137678	0.307299506	0.727658579	0.994342571	0.722391858	-6.393293722	-5.191369946	-6.449232539	-7.079892083	-6.296657142	-6.998892903	0.778499591	0.532449449

KLF8	0.188197526	0.087238266	0.27270267	-0.10095926	0.084505144	0.185464404	8	0.152229856	0.816496581	0.816496581	0.816496581	0.816496581	0.816496581	0.816496581	7	110	0	P_151036	P_151036	P_151036	chrX	56269397	56269457	+	KLF8	Kruppel-like factor 8 [Source:HGNC Symbol;Acc:HGNC:6351]	ENSG00000102349	protein_coding	3'UTR;exon	multiple Ref	1	1130	X:56269397:56269457	ENSG00000102349	KLF8	3.76674873	0.020816613	1.597550183	0.740538468	2.314888033	-0.857011715	0.71733785	1.574349565	9.961963646	0.14134337	0.476065046	0.043239766	0.411580421	0.489643866	0.146599729	-5.759704235	-5.900985496	-4.709772343	-6.762158545	-6.030051886	-5.863206255	2.031148273	0.173689302

RBM22	0.430507161	-0.070220835	0.030387984	-0.500727996	-0.400119177	0.100608819	8	0.258782727	0.816496581	0.816496581	0.816496581	0.816496581	0.816496581	0.816496581	7	111	0	P_255392	P_255392	P_255392	chr5	150696790	150696850	-	RBM22	RNA binding motif protein 22 [Source:HGNC Symbol;Acc:HGNC:25503]	ENSG00000086589	protein_coding	exon	coding exon	1	1131	5:150696790:150696850	ENSG00000086589	RBM22	8.373167388	0.055986041	2.228363985	-0.363472651	0.157292365	-2.591836637	-2.07107162	0.520765017	9.961963646	0.050078676	0.723846521	0.87815676	0.026941966	0.065272168	0.613907863	-4.803949355	-6.112918439	-7.033626225	-4.346940862	-4.412044263	-6.925217851	2.733435053	0.099783245

TCTN2	0.026919124	0.031832452	-0.098590209	0.004913328	-0.125509333	-0.130422661	8	0.075510523	0.816496581	0.816496581	0.816496581	0.816496581	0.816496581	0.816496581	7	112	0	P_113192	P_113192	P_113192	chr12	123690539	123690599	-	TCTN2	tectonic family member 2 [Source:HGNC Symbol;Acc:HGNC:25774]	ENSG00000168778	protein_coding	exon;promoter	multiple non-Ref	0	1132	12:123690539:123690599	ENSG00000168778	TCTN2	6.212781138	0.006785572	0.400233401	0.473284731	-1.465838742	0.07305133	-1.866072143	-1.939123473	9.961963646	0.697432551	0.646210289	0.17353154	0.943211205	0.091710397	0.081309678	-6.911648672	-6.065364514	-5.985386926	-7.146695498	-4.717008066	-5.330757408	1.630105002	0.244316596

AREL1	0.106036424	0.059848084	0.031254081	-0.04618834	-0.074782343	-0.028594003	8	0.052019785	0.816496581	0.816496581	0.816496581	0.816496581	0.816496581	0.816496581	7	113	0	P_221552	P_221552	P_221552	chr14	74673077	74673137	-	AREL1	apoptosis resistant E3 ubiquitin protein ligase 1 [Source:HGNC Symbol;Acc:HGNC:20363]	ENSG00000119682	protein_coding	exon	coding exon	1	1133	14:74673077:74673137	ENSG00000119682	AREL1	6.494667272	0.004379796	1.962337727	1.107564249	0.57839617	-0.854773478	-1.383941557	-0.529168079	9.961963646	0.078242543	0.294075117	0.575838224	0.412760912	0.19659536	0.608277458	-5.224380307	-5.573763949	-6.86744664	-6.764109412	-5.3677941	-6.920528678	1.389660139	0.302386247

PDLIM1	-0.003323675	0.064865609	-0.051085942	0.068189283	-0.047762267	-0.11595155	8	0.121806488	0.816496581	0.816496581	0.816496581	0.816496581	0.816496581	0.816496581	7	115	0	P_106674	P_106674	P_106674	chr10	95273641	95273701	-	PDLIM1	PDZ and LIM domain 1 [Source:HGNC Symbol;Acc:HGNC:2067]	ENSG00000107438	protein_coding	intron;promoter	multiple non-Ref	0	1135	10:95273641:95273701	ENSG00000107438	PDLIM1	6.269945323	0.01412146	-0.034255028	0.66852912	-0.526510742	0.702784148	-0.492255714	-1.195039862	9.961963646	0.973350429	0.518985666	0.610055108	0.498283752	0.633205734	0.259745715	-6.997720037	-5.951824646	-6.897814868	-6.886034076	-6.169394607	-6.342646107	0.481609945	0.702382693

ATXN3	0.100720807	-0.035264289	-0.037705707	-0.135985095	-0.138426514	-0.002441419	8	0.051694323	0.816496581	0.816496581	0.816496581	0.816496581	0.816496581	0.816496581	7	117	0	P_115606	P_115606	P_115606	chr14	92058606	92058666	-	ATXN3	ataxin 3 [Source:HGNC Symbol;Acc:HGNC:7106]	ENSG00000066427	protein_coding	3'UTR;exon	multiple Ref	1	1137	14:92058606:92058666	ENSG00000066427	ATXN3	9.038977789	0.004352689	1.869760597	-0.654639093	-0.69996109	-2.52439969	-2.569721687	-0.045321997	9.961963646	0.091156896	0.527524696	0.499970486	0.030239816	0.027982083	0.964746292	-5.365401406	-5.961088603	-6.785975133	-4.460363609	-3.628923135	-7.070256489	2.898769998	0.088240672

TRIM66	-0.043657864	-0.172505521	-0.104830518	-0.128847657	-0.061172653	0.067675004	8	0.095921476	0.816496581	0.816496581	0.816496581	0.816496581	0.816496581	0.816496581	7	118	0	P_107895	P_107895	P_107895	chr11	8612079	8612139	+	TRIM66	tripartite motif containing 66 [Source:HGNC Symbol;Acc:HGNC:29005]	ENSG00000166436	protein_coding	3'UTR	3'UTR antisense	0	1138	11:8612079:8612139	ENSG00000166436	TRIM66	5.232284629	0.009595532	-0.545850338	-2.156820968	-1.310686504	-1.61097063	-0.764836166	0.846134464	9.961963646	0.597179122	0.056512923	0.219374211	0.138379732	0.46210268	0.417339029	-6.838173435	-4.187853486	-6.182145842	-5.889281203	-5.994629977	-6.693766325	1.760645442	0.218195878

DISC1	0.132372439	0.007302879	0.05483673	-0.12506956	-0.077535709	0.047533851	8	0.136245269	0.816496581	0.816496581	0.816496581	0.816496581	0.816496581	0.816496581	7	119	0	P_105104	P_105104	P_105104	chr1	231771460	231771520	+	DISC1	disrupted in schizophrenia 1 [Source:HGNC Symbol;Acc:HGNC:2888]	ENSG00000162946	protein_coding	intron	intron sense	0	1139	1:231771460:231771520	ENSG00000162946	DISC1	4.195191313	0.017113603	1.23928865	0.068370543	0.513388871	-1.170918107	-0.725899779	0.445018328	9.961963646	0.243640661	0.946843519	0.618872004	0.268878042	0.484604838	0.665819934	-6.219393768	-6.179623746	-6.9050684	-6.44741971	-6.023816761	-6.964256724	0.650016673	0.60079952

FCER1G	-0.156224615	-0.010986884	-0.158990224	0.145237731	-0.002765609	-0.148003339	8	0.189372182	0.816496581	0.816496581	0.816496581	0.816496581	0.816496581	0.816496581	7	120	0	A_23_P160849	A_23_P160849	A_23_P160849	chr1	161219084	161219144	+	FCER1G	"Fc fragment of IgE, high affinity I, receptor for; gamma polypeptide [Source:HGNC Symbol;Acc:HGNC:3611]"	ENSG00000158869	protein_coding	3'UTR;exon	multiple Ref	1	1140	1:161219084:161219144	ENSG00000158869	FCER1G	7.071057314	0.031005684	-1.086611852	-0.07641868	-1.105847894	1.010193172	-0.019236042	-1.029429214	9.961963646	0.302800638	0.940598965	0.294782488	0.336309329	0.985032627	0.327623633	-6.389748082	-6.179009779	-6.417481277	-6.618473426	-6.296488449	-6.521419873	0.748341579	0.547781485

S100A13	0.152420281	0.095298308	0.141366312	-0.057121973	-0.011053969	0.046068004	8	0.105440765	0.816496581	0.816496581	0.816496581	0.816496581	0.816496581	0.816496581	7	121	0	A_23_P372874	A_23_P372874	A_23_P372874	chr1	153618808	153618868	-	S100A13	S100 calcium binding protein A13 [Source:HGNC Symbol;Acc:HGNC:10490]	ENSG00000189171	protein_coding	3'UTR;exon	multiple Ref	1	1141	1:153618808:153618868	ENSG00000189171	S100A13	8.963302594	0.011134847	1.769075037	1.106085464	1.640776484	-0.662989573	-0.128298553	0.53469102	9.961963646	0.107428764	0.294684498	0.131994757	0.522381238	0.900465814	0.604591397	-5.515040004	-5.575292808	-5.747000995	-6.914418303	-6.287937593	-6.91740808	1.297851532	0.328570545

VPS13C	0.042361813	-0.028530023	0.048478149	-0.070891837	0.006116336	0.077008173	8	0.070640134	0.816496581	0.816496581	0.816496581	0.816496581	0.816496581	0.816496581	7	123	0	P_223956	P_223956	P_223956	chr15	61920548	61920608	-	VPS13C	vacuolar protein sorting 13 homolog C (S. cerevisiae) [Source:HGNC Symbol;Acc:HGNC:23594]	ENSG00000129003	protein_coding	exon	coding exon	1	1143	15:61920548:61920608	ENSG00000129003	VPS13C	7.841321493	0.006213949	0.658167347	-0.443265483	0.753195682	-1.10143283	0.095028335	1.196461165	9.961963646	0.525347837	0.667044913	0.468758561	0.296608146	0.92617663	0.259215446	-6.767011044	-6.079556885	-6.74587342	-6.52379485	-6.291884364	-6.341023908	0.641779377	0.605453852

CAST	0.07483398	0.218514518	0.281957835	0.143680537	0.207123855	0.063443318	8	0.103760428	0.816496581	0.816496581	0.816496581	0.816496581	0.816496581	0.816496581	7	124	0	P_139987	P_139987	P_139987	chr5	96762292	96762352	+	CAST	calpastatin [Source:HGNC Symbol;Acc:HGNC:1515]	ENSG00000153113	protein_coding	exon	coding exon	1	1144	5:96762292:96762352	ENSG00000153113	CAST	8.91425497	0.010852551	0.879789062	2.568975776	3.314850001	1.689186713	2.435060939	0.745874226	9.961963646	0.399699141	0.028017852	0.007854961	0.122185017	0.035230697	0.47297616	-6.591712218	-3.545886781	-3.017359854	-5.778613284	-3.844490973	-6.775695288	4.616849625	0.028394495

BLOC1S6	0.441930892	-0.184774954	0.183196119	-0.626705846	-0.258734773	0.367971073	8	0.045609902	0.816496581	0.816496581	0.816496581	0.816496581	0.816496581	0.816496581	7	126	0	P_116446	P_116446	P_116446	chr15	45609630	45609690	+	BLOC1S6	"biogenesis of lysosomal organelles complex-1, subunit 6, pallidin [Source:HGNC Symbol;Acc:HGNC:8549]"	ENSG00000104164	protein_coding	3'UTR	3'UTR	1	1146	15:45609630:45609690	ENSG00000104164	BLOC1S6	9.920048876	0.003877249	8.692369111	-3.634351274	3.603297072	-12.32672038	-5.089072039	7.237648346	9.961963646	5.80E-06	0.004608006	0.004850979	2.35E-07	0.000477138	2.86E-05	4.455786226	-1.851481656	-2.526753502	7.64963218	0.269049646	2.780344739	55.32996383	1.62E-06

PAPSS1	0.220156583	0.176572245	0.516936532	-0.043584338	0.29677995	0.340364288	8	0.072070626	0.816496581	0.816496581	0.816496581	0.816496581	0.816496581	0.816496581	7	127	0	P_250358	P_250358	P_250358	chr4	107654830	107654890	-	PAPSS1	3'-phosphoadenosine 5'-phosphosulfate synthase 1 [Source:HGNC Symbol;Acc:HGNC:8603]	ENSG00000138801	protein_coding	exon	coding exon	1	1147	4:107654830:107654890	ENSG00000138801	PAPSS1	6.686481969	0.00637789	3.376282672	2.707880922	7.92764783	-0.668401751	4.551365157	5.219766908	9.961963646	0.007083992	0.02208358	1.30E-05	0.519063591	0.001066413	0.000394884	-2.868487427	-3.324917515	3.604311184	-6.910646576	-0.498662585	0.046266364	21.66452603	0.000109813

AMPD1	0.037845056	0.160875259	0.011425189	0.123030203	-0.026419867	-0.14945007	8	0.16619956	0.816496581	0.816496581	0.816496581	0.816496581	0.816496581	0.816496581	7	129	0	P_203556	P_203556	P_203556	chr1	114686762	114686822	-	AMPD1	adenosine monophosphate deaminase 1 [Source:HGNC Symbol;Acc:HGNC:468]	ENSG00000116748	protein_coding	exon	coding exon	1	1149	1:114686762:114686822	ENSG00000116748	AMPD1	3.82629559	0.024388892	0.296796481	1.261649885	0.089601029	0.964853403	-0.207195452	-1.172048856	9.961963646	0.772717811	0.235815272	0.930380097	0.357455739	0.840031166	0.268444321	-6.950504305	-5.406140875	-7.04273802	-6.663049648	-6.273902079	-6.368685618	0.672574582	0.588222356

EMC3	-0.038929875	0.087605988	0.137019427	0.126535863	0.175949301	0.049413439	8	0.078363485	0.816496581	0.816496581	0.816496581	0.816496581	0.816496581	0.816496581	7	130	0	P_243180	P_243180	P_243180	chr3	9969718	9969778	-	EMC3	ER membrane protein complex subunit 3 [Source:HGNC Symbol;Acc:HGNC:23999]	ENSG00000125037	protein_coding	exon	coding exon	1	1150	3:9969718:9969778	ENSG00000125037	EMC3	9.512598009	0.00713811	-0.564335042	1.269953457	1.986260283	1.834288499	2.550595325	0.716306826	9.961963646	0.585007235	0.232962185	0.075195726	0.096610555	0.028913731	0.490252858	-6.827313854	-5.396658258	-5.234565174	-5.565896925	-3.659668068	-6.798101961	2.709955336	0.101563313

SPAG9	0.150071938	-0.001706043	0.133938872	-0.151777981	-0.016133067	0.135644914	8	0.071344243	0.816496581	0.816496581	0.816496581	0.816496581	0.816496581	0.816496581	7	131	0	P_229187	P_229187	P_229187	chr17	50993763	50993823	-	SPAG9	sperm associated antigen 9 [Source:HGNC Symbol;Acc:HGNC:14524]	ENSG00000008294	protein_coding	exon	coding exon	1	1151	17:50993763:50993823	ENSG00000008294	SPAG9	8.210472773	0.006294232	2.316721019	-0.026336868	2.067668365	-2.343057887	-0.249052654	2.094005233	9.961963646	0.043105185	0.979508761	0.065645234	0.041215981	0.808379637	0.062810293	-4.660244326	-6.181721775	-5.107361434	-4.761919411	-6.263799492	-5.089186657	3.263324629	0.067925857

ECRG4	0.09900498	-0.090326055	-0.04104243	-0.189331035	-0.140047409	0.049283625	8	0.122797764	0.816496581	0.816496581	0.816496581	0.816496581	0.816496581	0.816496581	7	132	0	P_128620	P_128620	P_128620	chr2	106077973	106078033	+	C2orf40	chromosome 2 open reading frame 40 [Source:HGNC Symbol;Acc:HGNC:24642]	ENSG00000119147	protein_coding	3'UTR;exon	multiple Ref	1	1152	2:106077973:106078033	ENSG00000119147	ECRG4	3.752280832	0.014316177	1.013419235	-0.924581386	-0.420112076	-1.938000622	-1.433531312	0.50446931	9.961963646	0.334840803	0.377036364	0.683320962	0.081460852	0.182336924	0.624901816	-6.465190438	-5.750494084	-6.951585464	-5.408604651	-5.306235593	-6.93410862	1.35395186	0.312280897

ANAPC5	-0.141761554	0.03834473	-0.181079472	0.180106284	-0.039317918	-0.219424202	8	0.109360427	0.816496581	0.816496581	0.816496581	0.816496581	0.816496581	0.816496581	7	133	0	A_32_P202042	A_32_P202042	A_32_P202042	chr12	121346948	121347008	+	ANAPC5	anaphase promoting complex subunit 5 [Source:HGNC Symbol;Acc:HGNC:15713]	ENSG00000089053	protein_coding	"5'UTR,exon,intron;exon;exon,intron;promoter"	multiple non-Ref	0	1153	12:121346948:121347008	ENSG00000089053	ANAPC5	8.815189606	0.011810978	-1.597574688	0.432124002	-2.04066598	2.02969869	-0.443091292	-2.472789982	9.961963646	0.141337908	0.674855052	0.06867788	0.069947172	0.667166703	0.03303092	-5.759670293	-6.084597379	-5.149784626	-5.26633639	-6.193309468	-4.47193282	2.88899844	0.08887737

LSM6	-0.510904267	-0.062660329	-0.446525666	0.448243938	0.064378601	-0.383865337	8	0.052869549	0.816496581	0.816496581	0.816496581	0.816496581	0.816496581	0.816496581	7	134	0	A_23_P259451	A_23_P259451	A_23_P259451	chr4	146189818	146189878	+	LSM6	"LSM6 homolog, U6 small nuclear RNA associated (S. cerevisiae) [Source:HGNC Symbol;Acc:HGNC:17017]"	ENSG00000164167	protein_coding	3'UTR	3'UTR	1	1154	4:146189818:146189878	ENSG00000164167	LSM6	10.87107707	0.004451373	-9.378602933	-1.150247471	-8.19681336	8.228355462	1.181789574	-7.046565888	9.961963646	2.93E-06	0.276904918	9.74E-06	9.42E-06	0.264731052	3.58E-05	5.145691457	-5.52896363	3.904769954	3.885915564	-5.603084684	2.545218422	45.87092708	3.87E-06

DPYD	-0.041983257	0.117168904	-0.084031148	0.159152161	-0.042047891	-0.201200052	8	0.09777827	0.816496581	0.816496581	0.816496581	0.816496581	0.816496581	0.816496581	7	135	0	P_308586	P_308586	P_308586	chr1	97721315	97721375	+	DPYD	dihydropyrimidine dehydrogenase [Source:HGNC Symbol;Acc:HGNC:3012]	ENSG00000188641	protein_coding	intron	intron antisense	0	1155	1:97721315:97721375	ENSG00000188641	DPYD	3.670303936	0.009884359	-0.51718688	1.443390163	-1.035169973	1.960577043	-0.517983093	-2.478560137	9.961963646	0.616313412	0.179610096	0.325064349	0.078471311	0.615777711	0.032706647	-6.854338256	-5.188978577	-6.49152401	-5.37384195	-6.155927365	-4.462311715	2.279643914	0.141882744

RORA	0.271310967	0.062032906	0.326053178	-0.209278061	0.054742211	0.264020271	8	0.093886354	0.816496581	0.816496581	0.816496581	0.816496581	0.816496581	0.816496581	7	136	0	P_223920	P_223920	P_223920	chr15	60499891	60499951	-	RORA	RAR-related orphan receptor A [Source:HGNC Symbol;Acc:HGNC:10258]	ENSG00000069667	protein_coding	exon	coding exon	1	1156	15:60499891:60499951	ENSG00000069667	RORA	5.06646183	0.009285327	3.448374212	0.788440942	4.144150094	-2.65993327	0.695775882	3.355709152	9.961963646	0.006277932	0.448794233	0.002014904	0.023974836	0.50247752	0.007333098	-2.745871719	-5.864605346	-1.624348325	-4.231848882	-6.045458971	-2.969716875	8.084506136	0.005036415

PCLO	-0.125695175	-0.015232422	-0.154687528	0.110462754	-0.028992352	-0.139455106	8	0.067421304	0.816496581	0.816496581	0.816496581	0.816496581	0.816496581	0.816496581	7	137	0	P_261516	P_261516	P_261516	chr7	82838217	82838277	-	PCLO	piccolo presynaptic cytomatrix protein [Source:HGNC Symbol;Acc:HGNC:13406]	ENSG00000186472	protein_coding	3'UTR;exon	multiple Ref	1	1157	7:82838217:82838277	ENSG00000186472	PCLO	5.524508787	0.005857075	-2.011517723	-0.243766602	-2.475486449	1.767751121	-0.463968726	-2.231719846	9.961963646	0.072100371	0.812358199	0.03287899	0.107659461	0.652642836	0.049794904	-5.148250608	-6.150858697	-4.444358476	-5.664570963	-6.183447728	-4.868508928	3.092407223	0.076670497

USP9Y	0.080640735	-0.146481584	-0.032062575	-0.227122319	-0.11270331	0.114419008	8	0.081786071	0.816496581	0.816496581	0.816496581	0.816496581	0.816496581	0.816496581	7	138	0	P_271986	P_271986	P_271986	chrY	12847084	12847144	+	USP9Y	"ubiquitin specific peptidase 9, Y-linked [Source:HGNC Symbol;Acc:HGNC:12633]"	ENSG00000114374	protein_coding	exon	coding exon	1	1158	Y:12847084:12847144	ENSG00000114374	USP9Y	8.168928433	0.007578285	1.134526512	-2.060834886	-0.451085195	-3.195361398	-1.585611708	1.60974969	9.961963646	0.283135182	0.066400454	0.661588067	0.009611638	0.144026941	0.138647049	-6.338125307	-4.332683701	-6.937127202	-3.316420865	-5.10905724	-5.814206839	3.508910284	0.057343759

TMC5	0.015764951	0.022569438	0.081523257	0.006804488	0.065758307	0.058953819	8	0.103109503	0.816496581	0.816496581	0.816496581	0.816496581	0.816496581	0.816496581	7	139	0	P_225725	P_225725	P_225725	chr16	19469765	19469825	+	TMC5	transmembrane channel-like 5 [Source:HGNC Symbol;Acc:HGNC:22999]	ENSG00000103534	protein_coding	exon	coding exon	1	1159	16:19469765:19469825	ENSG00000103534	TMC5	4.535945063	0.010744414	0.186271701	0.266670524	0.963242831	0.080398823	0.77697113	0.696572307	9.961963646	0.855970142	0.795161452	0.35822438	0.937512302	0.455229332	0.501999852	-6.97945952	-6.14473732	-6.562810902	-7.146079287	-5.985257375	-6.812602325	0.354834692	0.786811262

CREBBP	0.208679963	-0.223894331	0.059116757	-0.432574294	-0.149563206	0.283011088	8	0.073065749	0.816496581	0.816496581	0.816496581	0.816496581	0.816496581	0.816496581	7	140	0	P_225402	P_225402	P_225402	chr16	3738558	3738618	-	CREBBP	CREB binding protein [Source:HGNC Symbol;Acc:HGNC:2348]	ENSG00000005339	protein_coding	exon	coding exon	1	1160	16:3738558:3738618	ENSG00000005339	CREBBP	7.917950973	0.006493874	3.171571143	-3.402802989	0.898471502	-6.574374132	-2.273099642	4.30127449	9.961963646	0.010007032	0.006775708	0.39013348	6.39E-05	0.046421237	0.001572014	-3.217706965	-2.215497491	-6.623350078	1.889141543	-4.100306864	-1.388742693	14.8892537	0.000518315

PARD3B	0.130957802	-0.038462373	0.123337946	-0.169420175	-0.007619856	0.161800319	8	0.091016872	0.816496581	0.816496581	0.816496581	0.816496581	0.816496581	0.816496581	7	143	0	P_238880	P_238880	P_238880	chr2	205172257	205172317	+	PARD3B	par-3 family cell polarity regulator beta [Source:HGNC Symbol;Acc:HGNC:14446]	ENSG00000116117	protein_coding	exon	coding exon	1	1163	2:205172257:205172317	ENSG00000116117	PARD3B	5.658643926	0.008859245	1.704035583	-0.500476118	1.604885278	-2.204511701	-0.099150306	2.105361396	9.961963646	0.119310526	0.627610776	0.139716648	0.052141108	0.922985734	0.061624245	-5.609423472	-6.051744351	-5.797216851	-4.988001807	-6.291459059	-5.071182949	2.505353266	0.118769312

FADS6	0.172876682	0.047330736	0.170360734	-0.125545946	-0.002515948	0.123029998	8	0.187069945	0.816496581	0.816496581	0.816496581	0.816496581	0.816496581	0.816496581	7	144	0	P_122511	P_122511	P_122511	chr17	74878232	74878292	-	FADS6	fatty acid desaturase 6 [Source:HGNC Symbol;Acc:HGNC:30459]	ENSG00000172782	protein_coding	3'UTR;exon	multiple Ref	1	1164	17:74878232:74878292	ENSG00000172782	FADS6	6.759834626	0.030309709	1.216161253	0.332964554	1.198461942	-0.883196699	-0.017699311	0.865497388	9.961963646	0.251954591	0.74606224	0.258470451	0.397942308	0.986228199	0.40712597	-6.246276507	-6.123978652	-6.314757247	-6.7390103	-6.296518638	-6.676909515	0.759276669	0.54217237

ZNF618	0.118022868	0.021380084	0.084840317	-0.096642784	-0.033182551	0.063460233	8	0.042211594	0.816496581	0.816496581	0.816496581	0.816496581	0.816496581	0.816496581	7	145	0	P_149473	P_149473	P_149473	chr9	114047934	114047994	+	ZNF618	zinc finger protein 618 [Source:HGNC Symbol;Acc:HGNC:29416]	ENSG00000157657	protein_coding	exon	coding exon	1	1165	9:114047934:114047994	ENSG00000157657	ZNF618	6.499964155	0.003637582	2.396654467	0.434158863	1.722826507	-1.962495604	-0.673827959	1.288667644	9.961963646	0.037617838	0.673425558	0.115760669	0.078222061	0.515750008	0.22663576	-4.528850108	-6.083685995	-5.629870594	-5.370879609	-6.060704602	-6.232774431	2.47777919	0.121341833

NEK1	0.172935504	-0.165676183	0.076355503	-0.338611687	-0.096580001	0.242031686	8	0.071258073	0.816496581	0.816496581	0.816496581	0.816496581	0.816496581	0.816496581	7	146	0	A_23_P259801	A_23_P259801	A_23_P259801	chr4	169394432	169394493	-	NEK1	NIMA-related kinase 1 [Source:HGNC Symbol;Acc:HGNC:7744]	ENSG00000137601	protein_coding	3'UTR	3'UTR	0	1166	4:169394432:169394493	ENSG00000137601	NEK1	8.371242966	0.006284364	2.671770271	-2.559617258	1.179655756	-5.231387529	-1.492114515	3.739273014	9.961963646	0.023493392	0.028470498	0.265540971	0.000388342	0.166642992	0.003876666	-4.068879407	-3.560718755	-6.336122925	0.000566842	-5.231729567	-2.318924783	9.776261412	0.00258089

ARMC8	-0.095459153	0.111146766	-0.068843201	0.20660592	0.026615952	-0.179989967	8	0.073691678	0.816496581	0.816496581	0.816496581	0.816496581	0.816496581	0.816496581	7	148	0	P_246368	P_246368	P_246368	chr3	138239468	138239528	+	ARMC8	armadillo repeat containing 8 [Source:HGNC Symbol;Acc:HGNC:24999]	ENSG00000114098	protein_coding	"3'UTR,exon;exon"	multiple Ref	1	1168	3:138239468:138239528	ENSG00000114098	ARMC8	7.910994383	0.006567642	-1.442641641	1.679723189	-1.040403827	3.12236483	0.402237814	-2.720127017	9.961963646	0.179815893	0.124049456	0.322744177	0.010878554	0.696004031	0.021624966	-5.967840168	-4.879829043	-6.486174569	-3.441655902	-6.211342875	-4.055626611	3.8825289	0.044761961

PHIP	0.014418266	-0.119724033	0.025663675	-0.1341423	0.011245409	0.145387708	8	0.083192845	0.816496581	0.816496581	0.816496581	0.816496581	0.816496581	0.816496581	7	150	0	P_143086	P_143086	P_143086	chr6	78990871	78990931	-	PHIP	pleckstrin homology domain interacting protein [Source:HGNC Symbol;Acc:HGNC:15673]	ENSG00000146247	protein_coding	exon	coding exon	1	1170	6:78990871:78990931	ENSG00000146247	PHIP	8.822651409	0.007764664	0.200399826	-1.664047173	0.356700025	-1.864446999	0.156300199	2.020747198	9.961963646	0.845199903	0.127194266	0.728755978	0.091955259	0.878918447	0.070999596	-6.976489684	-4.901156783	-6.978079568	-5.520580112	-6.283707834	-5.20440536	1.753409933	0.219556539

DAB1	0.585341177	-0.013733466	0.395330597	-0.599074644	-0.19001058	0.409064063	8	0.145861051	0.816496581	0.816496581	0.816496581	0.816496581	0.816496581	0.816496581	7	152	0	A_23_P23850	A_23_P23850	A_23_P23850	chr1	57014965	57015025	-	DAB1	"Dab, reelin signal transducer, homolog 1 (Drosophila) [Source:HGNC Symbol;Acc:HGNC:2661]"	ENSG00000173406	protein_coding	exon	coding exon	1	1172	1:57014965:57015025	ENSG00000173406	DAB1	4.679242907	0.019292027	5.16137925	-0.12109797	3.485917646	-5.28247722	-1.675461603	3.607015617	9.961963646	0.000429585	0.906021189	0.005896322	0.000360925	0.124897385	0.004821195	0.015615767	-6.174362522	-2.725851281	0.077116985	-4.987138433	-2.542228285	13.75473686	0.000709809

CSMD1	0.472848208	0.010880913	0.368229236	-0.461967295	-0.104618971	0.357348324	8	0.045261718	0.816496581	0.816496581	0.816496581	0.816496581	0.816496581	0.816496581	7	153	0	P_263673	P_263673	P_263673	chr8	3029388	3029448	-	CSMD1	CUB and Sushi multiple domains 1 [Source:HGNC Symbol;Acc:HGNC:14026]	ENSG00000183117	protein_coding	exon	coding exon	1	1173	8:3029388:3029448	ENSG00000183117	CSMD1	5.565185789	0.00385184	9.331108798	0.214722142	7.266575215	-9.116386656	-2.064533584	7.051853073	9.961963646	3.07E-06	0.834315695	2.76E-05	3.79E-06	0.065990664	3.56E-05	5.099455461	-6.157840277	2.829964557	4.828549104	-4.421965151	2.55178738	46.16970812	3.76E-06

SNX16	0.02068941	0.167625908	0.102813313	0.146936498	0.082123903	-0.064812595	8	0.047060435	0.816496581	0.816496581	0.816496581	0.816496581	0.816496581	0.816496581	8	20	0	P_265218	P_265218	P_265218	chr8	81839614	81839674	-	SNX16	sorting nexin 16 [Source:HGNC Symbol;Acc:HGNC:14980]	ENSG00000104497	protein_coding	exon	coding exon	1	1210	8:81839614:81839674	ENSG00000104497	SNX16	7.935676075	0.003985196	0.401392149	3.252085139	1.994665688	2.85069299	1.593273539	-1.257419451	9.961963646	0.696606574	0.008732244	0.074151973	0.017291643	0.14229955	0.237279788	-6.911149898	-2.454777891	-5.221529683	-3.907170443	-5.098809707	-6.270113586	4.493656564	0.030564158

ANKHD1	-0.176337417	-0.033247018	-0.151482164	0.143090398	0.024855253	-0.118235145	8	0.0601018	0.816496581	0.816496581	0.816496581	0.816496581	0.816496581	0.816496581	8	21	0	P_255086	P_255086	P_255086	chr5	140445915	140445975	+	ANKHD1	ankyrin repeat and KH domain containing 1 [Source:HGNC Symbol;Acc:HGNC:24714]	ENSG00000131503	protein_coding	5'UTR;exon	multiple Ref	1	1211	5:140445915:140445975	ENSG00000131503	ANKHD1	8.411342165	0.005107499	-3.021940939	-0.569762947	-2.595989898	2.452177992	0.425951041	-2.026226951	9.961963646	0.012905888	0.581458499	0.026750959	0.034215369	0.679199857	0.070353578	-3.473337149	-6.013795125	-4.2425179	-4.581120452	-6.201079917	-5.195844159	4.416021209	0.032033238

CXorf58	0.025539979	-0.127507648	0.178959558	-0.153047627	0.153419579	0.306467206	8	0.153480197	0.816496581	0.816496581	0.816496581	0.816496581	0.816496581	0.816496581	8	23	0	P_269821	P_269821	P_269821	chrX	23910358	23910418	+	CXorf58	chromosome X open reading frame 58 [Source:HGNC Symbol;Acc:HGNC:26356]	ENSG00000165182	protein_coding	exon	coding exon	1	1213	X:23910358:23910418	ENSG00000165182	CXorf58	4.283300128	0.021123574	0.215219925	-1.074479586	1.50805379	-1.289699511	1.292833865	2.582533376	9.961963646	0.833938051	0.307943319	0.16258096	0.226291087	0.225246772	0.027374754	-6.973143124	-5.607589725	-5.929373171	-6.308829261	-5.477068763	-4.288127265	2.246545849	0.145699165

RRBP1	-0.159469472	0.094788675	0.06156029	0.254258148	0.221029762	-0.033228385	8	0.060786489	0.816496581	0.816496581	0.816496581	0.816496581	0.816496581	0.816496581	8	24	0	P_240282	P_240282	P_240282	chr20	17613700	17613760	+	RRBP1;DSTN	ribosome binding protein 1 [Source:HGNC Symbol;Acc:HGNC:10448];destrin (actin depolymerizing factor) [Source:HGNC Symbol;Acc:HGNC:15750]	ENSG00000125844	protein_coding	3'ext;3'UTR;exon	multiple non-Ref	0	1214	20:17613700:17613760	ENSG00000125844	RRBP1	10.36152051	0.005173968	-2.715259271	1.613950472	1.048176475	4.329209743	3.763435746	-0.565773997	9.961963646	0.021806108	0.137729237	0.319321627	0.00150468	0.003726074	0.58406532	-3.995346688	-4.968577711	-6.478190364	-1.41124678	-1.702110287	-6.899276839	7.383624797	0.006834235

ERCC8	0.04947299	0.165894694	0.19209161	0.116421704	0.142618621	0.026196916	8	0.09745635	0.816496581	0.816496581	0.816496581	0.816496581	0.816496581	0.816496581	8	25	0	P_252998	P_252998	P_252998	chr5	60874614	60874674	-	ERCC8	excision repair cross-complementation group 8 [Source:HGNC Symbol;Acc:HGNC:3439]	ENSG00000049167	protein_coding	3'UTR;exon	multiple Ref	1	1215	5:60874614:60874674	ENSG00000049167	ERCC8	7.431119717	0.009833887	0.611014018	2.04887524	2.372419123	1.437861222	1.761405106	0.323543883	9.961963646	0.554874898	0.067742107	0.039205078	0.181134988	0.108771572	0.752973088	-6.798387764	-4.350564256	-4.615234296	-6.122877283	-4.867150054	-7.014491608	2.579045644	0.112203107

DGCR8	-0.215218001	-0.060187492	-0.11541846	0.155030509	0.099799541	-0.055230968	8	0.095650314	0.816496581	0.816496581	0.816496581	0.816496581	0.816496581	0.816496581	8	26	0	P_242179	P_242179	P_242179	chr22	20094735	20094795	+	DGCR8	DGCR8 microprocessor complex subunit [Source:HGNC Symbol;Acc:HGNC:2847]	ENSG00000128191	protein_coding	exon	coding exon	1	1216	22:20094735:20094795	ENSG00000128191	DGCR8	6.982011064	0.009553816	-2.696719601	-0.754159914	-1.44621371	1.942559687	1.250505891	-0.692053797	9.961963646	0.02251005	0.468204903	0.178835617	0.080848677	0.239689137	0.504713581	-4.026715254	-5.890845957	-6.011079596	-5.401599115	-5.526065534	-6.815870781	2.624804909	0.108340966

TET2	0.224266058	-0.076777905	0.139332209	-0.301043962	-0.084933848	0.216110114	8	0.128455322	0.816496581	0.816496581	0.816496581	0.816496581	0.816496581	0.816496581	8	27	0	A_24_P932233	A_24_P932233	A_24_P932233	chr4	105235705	105235765	+	TET2	tet methylcytosine dioxygenase 2 [Source:HGNC Symbol;Acc:HGNC:25941]	ENSG00000168769	protein_coding	exon	coding exon	1	1217	4:105235705:105235765	ENSG00000168769	TET2	6.390562189	0.015457702	2.209208556	-0.756326684	1.372538995	-2.96553524	-0.836669562	2.128865678	9.961963646	0.05172861	0.466962282	0.200004407	0.014209431	0.422394398	0.059237028	-4.834868615	-5.889217928	-6.1054887	-3.710631442	-5.937270659	-5.033803949	3.56049668	0.055377017

IK	0.05557258	-0.022294493	0.117091134	-0.077867073	0.061518553	0.139385626	8	0.045745961	0.816496581	0.816496581	0.816496581	0.816496581	0.816496581	0.816496581	8	28	0	P_140582	P_140582	P_140582	chr5	140657602	140657662	+	IK	"IK cytokine, down-regulator of HLA II [Source:HGNC Symbol;Acc:HGNC:5958]"	ENSG00000113141	protein_coding	3'UTR;exon	multiple Ref	1	1218	5:140657602:140657662	ENSG00000113141	IK	9.859403411	0.003887231	1.091656594	-0.437948531	2.300114688	-1.529605125	1.208458095	2.738063219	9.961963646	0.300681772	0.670766948	0.04433949	0.157226087	0.254774024	0.020970432	-6.384394847	-6.081977691	-4.733940758	-6.001119589	-5.573572676	-4.025184679	2.995182268	0.08223934

FBLN2	0.615519053	0.403987824	0.610765562	-0.211531228	-0.00475349	0.206777738	8	0.181576147	0.816496581	0.816496581	0.816496581	0.816496581	0.816496581	0.816496581	8	29	0	P_243278	P_243278	P_243278	chr3	13636507	13636567	+	FBLN2	fibulin 2 [Source:HGNC Symbol;Acc:HGNC:3601]	ENSG00000163520	protein_coding	exon	coding exon	1	1219	3:13636507:13636567	ENSG00000163520	FBLN2	7.275985335	0.028683309	4.451152811	2.921455528	4.41677774	-1.529697283	-0.034375071	1.495322211	9.961963646	0.001244516	0.015320635	0.001312653	0.157203524	0.973257079	0.165818512	-1.085726188	-2.983334958	-1.181509095	-6.000994908	-6.296056794	-5.970198628	8.738788881	0.0038478

UBQLNL	-0.145746676	0.005451654	0.263235046	0.15119833	0.408981722	0.257783392	8	0.365161854	0.816496581	0.816496581	0.816496581	0.816496581	0.816496581	0.816496581	8	30	0	P_107749	P_107749	P_107749	chr11	5515093	5515153	-	UBQLNL	ubiquilin-like [Source:HGNC Symbol;Acc:HGNC:28294]	ENSG00000175518	protein_coding	exon	coding exon	1	1220	11:5515093:5515153	ENSG00000175518	UBQLNL	3.636105731	0.109288528	-0.539954296	0.020196989	0.975218767	0.560151285	1.515173063	0.955021778	9.961963646	0.601089456	0.984285033	0.352537629	0.58775048	0.160794688	0.362166574	-6.841565715	-6.181872386	-6.55123364	-6.980685863	-5.201894582	-6.594771456	0.794099279	0.524687032

UTP11	-0.072506525	0.027161415	-0.042272199	0.09966794	0.030234326	-0.069433614	8	0.037094111	0.816496581	0.816496581	0.816496581	0.816496581	0.816496581	0.816496581	8	32	0	P_101379	P_101379	P_101379	chr1	38023740	38023800	+	UTP11L	"UTP11-like, U3 small nucleolar ribonucleoprotein (yeast) [Source:HGNC Symbol;Acc:HGNC:24329]"	ENSG00000183520	protein_coding	3'UTR;exon	multiple Ref	1	1222	1:38023740:38023800	ENSG00000183520	UTP11	10.95706664	0.003311665	-1.543119281	0.578062505	-0.899657593	2.121181785	0.643461688	-1.477720097	9.961963646	0.153947495	0.57605489	0.389531655	0.060007545	0.534455919	0.170386757	-5.834325887	-6.00894051	-6.622273425	-5.121749252	-6.081062911	-5.993560684	1.770311493	0.216393308

B4GALT1	-0.033879618	0.12348783	0.141449471	0.157367448	0.175329089	0.017961642	8	0.069619924	0.816496581	0.816496581	0.816496581	0.816496581	0.816496581	0.816496581	8	33	0	A_24_P892137	A_24_P892137	A_24_P892137	chr9	33165551	33165611	+	B4GALT1;B4GALT1-AS1	"UDP-Gal:betaGlcNAc beta 1,4- galactosyltransferase, polypeptide 1 [Source:HGNC Symbol;Acc:HGNC:924];B4GALT1 antisense RNA 1 [Source:HGNC Symbol;Acc:HGNC:49910]"	ENSG00000086062	protein_coding;antisense	intron;promoter	multiple non-Ref	0	1223	9:33165551:33165611	ENSG00000086062	B4GALT1	7.463405293	0.006099036	-0.531316779	1.936596698	2.21827997	2.467913478	2.749596749	0.281683271	9.961963646	0.60684202	0.081650239	0.050940814	0.033307441	0.020560083	0.783951062	-6.846472557	-4.516439898	-4.866948509	-4.55487866	-3.338258242	-7.028204748	3.780615833	0.047836121

TGM4	0.018281969	0.095638927	0.050413111	0.077356957	0.032131141	-0.045225816	8	0.121885064	0.816496581	0.816496581	0.816496581	0.816496581	0.816496581	0.816496581	8	35	0	P_134569	P_134569	P_134569	chr3	44909920	44909980	+	TGM4	transglutaminase 4 [Source:HGNC Symbol;Acc:HGNC:11780]	ENSG00000163810	protein_coding	intron	intron sense	0	1225	3:44909920:44909980	ENSG00000163810	TGM4	4.383242549	0.014136837	0.188318282	0.985154165	0.519293637	0.796835884	0.330975356	-0.465860528	9.961963646	0.854407999	0.347870162	0.614896479	0.444122292	0.74751954	0.651334135	-6.979042657	-5.694842814	-6.9018258	-6.813062659	-6.238748502	-6.954098452	0.372862755	0.774480349

SLC26A2	-0.266289915	-0.161706355	-0.256441756	0.10458356	0.009848159	-0.094735401	8	0.150676801	0.816496581	0.816496581	0.816496581	0.816496581	0.816496581	0.816496581	8	36	0	P_140844	P_140844	P_140844	chr5	149981564	149981624	-	SLC26A2	"solute carrier family 26 (anion exchanger), member 2 [Source:HGNC Symbol;Acc:HGNC:10994]"	ENSG00000155850	protein_coding	exon;intron	multiple non-Ref	0	1226	5:149981564:149981624	ENSG00000155850	SLC26A2	6.103314875	0.020438831	-2.281247059	-1.385302729	-2.196880049	0.89594433	0.08436701	-0.81157732	9.961963646	0.045783607	0.19619171	0.052818125	0.391417951	0.934435993	0.435996412	-4.718145962	-5.260508959	-4.901471672	-6.727525735	-6.292900795	-6.723027758	2.23632092	0.146902394

GLB1L2	-0.195782213	0.119924408	-0.078844631	0.315706621	0.116937582	-0.198769038	8	0.122719451	0.816496581	0.816496581	0.816496581	0.816496581	0.816496581	0.816496581	8	37	0	P_213962	P_213962	P_213962	chr11	134368720	134368780	+	GLB1L2	"galactosidase, beta 1-like 2 [Source:HGNC Symbol;Acc:HGNC:25129]"	ENSG00000149328	protein_coding	exon	coding exon	1	1227	11:134368720:134368780	ENSG00000149328	GLB1L2	6.38077374	0.014300736	-2.005116742	1.228213912	-0.807492605	3.233330653	1.197624136	-2.035706517	9.961963646	0.072873248	0.247593703	0.438238045	0.009013468	0.258782201	0.069249134	-5.15820429	-5.443873396	-6.702284146	-3.251303308	-5.585620503	-5.1810115	3.702313096	0.050369731

C22orf31	0.267351327	0.301243142	0.525902412	0.033891815	0.258551085	0.22465927	8	0.23313607	0.816496581	0.816496581	0.816496581	0.816496581	0.816496581	0.816496581	8	38	0	A_23_P132237	A_23_P132237	A_23_P132237	chr22	29060516	29060576	-	C22orf31	chromosome 22 open reading frame 31 [Source:HGNC Symbol;Acc:HGNC:26931]	ENSG00000100249	protein_coding	exon	coding exon	1	1228	22:29060516:29060576	ENSG00000100249	C22orf31	3.852300977	0.045854647	1.529101441	1.722943845	3.00787038	0.193842404	1.478768938	1.284926534	9.961963646	0.157349456	0.115738807	0.013219223	0.850194783	0.17011153	0.227889027	-5.853289346	-4.820478353	-3.542371898	-7.129103509	-5.248867565	-6.237279333	3.038223264	0.079716903

TPH2	-0.114619243	-0.10825707	-0.014550799	0.006362173	0.100068444	0.093706271	8	0.184536281	0.816496581	0.816496581	0.816496581	0.816496581	0.816496581	0.816496581	8	41	0	P_216272	P_216272	P_216272	chr12	71994501	71994561	+	TPH2	tryptophan hydroxylase 2 [Source:HGNC Symbol;Acc:HGNC:20692]	ENSG00000139287	protein_coding	exon	coding exon	1	1231	12:71994501:71994561	ENSG00000139287	TPH2	3.697913628	0.029553613	-0.816578379	-0.771252542	-0.103663815	0.045325837	0.712914564	0.667588727	9.961963646	0.433262328	0.45846007	0.919493387	0.964743308	0.492259878	0.519561168	-6.646248075	-5.877890936	-7.041255186	-7.14848968	-6.033247056	-6.83323129	0.371392206	0.775482914

ICE1	0.032398449	-0.024726158	0.014944535	-0.057124606	-0.017453914	0.039670693	8	0.09192503	0.816496581	0.816496581	0.816496581	0.816496581	0.816496581	0.816496581	8	42	0	P_252022	P_252022	P_252022	chr5	5441163	5441223	+	ICE1	interactor of little elongator complex ELL subunit 1 [Source:HGNC Symbol;Acc:HGNC:29154]	ENSG00000164151	protein_coding	exon	coding exon	1	1232	5:5441163:5441223	ENSG00000164151	ICE1	7.346158029	0.008992664	0.418432736	-0.319343494	0.193011795	-0.73777623	-0.225420941	0.512355289	9.961963646	0.684508262	0.756061865	0.850827971	0.477669126	0.826208926	0.619569221	-6.903653955	-6.12861106	-7.026814283	-6.859841035	-6.269728634	-6.929839578	0.195326014	0.897188083

SLC2A9	0.133658597	-0.016588772	0.341853786	-0.150247369	0.208195189	0.358442558	8	0.110232048	0.816496581	0.816496581	0.816496581	0.816496581	0.816496581	0.816496581	8	43	0	P_137337	P_137337	P_137337	chr4	10025903	10025963	-	SLC2A9	"solute carrier family 2 (facilitated glucose transporter), member 9 [Source:HGNC Symbol;Acc:HGNC:13446]"	ENSG00000109667	protein_coding	exon	coding exon	1	1233	4:10025903:10025963	ENSG00000109667	SLC2A9	4.370766393	0.011964683	1.49655239	-0.185741633	3.827678208	-1.682294023	2.331125818	4.013419841	9.961963646	0.165503258	0.856374847	0.003354658	0.123540448	0.042061673	0.002483329	-5.896907818	-6.163932761	-2.149013602	-5.788484091	-4.009166821	-1.860980132	6.882848174	0.008601322

LAPTM5	0.097142142	0.228507525	0.286403067	0.131365383	0.189260925	0.057895541	8	0.15478527	0.816496581	0.816496581	0.816496581	0.816496581	0.816496581	0.816496581	8	44	0	P_101081	P_101081	P_101081	chr1	30732741	30732801	+	LAPTM5	lysosomal protein transmembrane 5 [Source:HGNC Symbol;Acc:HGNC:29612]	ENSG00000162511	protein_coding	3'UTR	3'UTR antisense	0	1234	1:30732741:30732801	ENSG00000162511	LAPTM5	3.909355669	0.02144665	0.81240685	1.911025171	2.395209825	1.098618322	1.582802975	0.484184653	9.961963646	0.435542113	0.085172657	0.03771066	0.29777652	0.144664817	0.638722761	-6.649722836	-4.55367885	-4.577608359	-6.526812099	-5.112806682	-6.944800204	2.332619075	0.136015472

CLSTN1	0.119224983	-0.037934538	0.081498589	-0.157159521	-0.037726394	0.119433127	8	0.055596517	0.816496581	0.816496581	0.816496581	0.816496581	0.816496581	0.816496581	8	45	0	P_200204	P_200204	P_200204	chr1	9744394	9744454	-	CLSTN1	calsyntenin 1 [Source:HGNC Symbol;Acc:HGNC:17447]	ENSG00000171603	protein_coding	exon	coding exon	1	1235	1:9744394:9744454	ENSG00000171603	CLSTN1	8.593122384	0.004688904	2.132442085	-0.678492076	1.45767285	-2.810934161	-0.674769236	2.136164925	9.961963646	0.058881634	0.512911976	0.175721847	0.01850953	0.5151765	0.058513858	-4.957854607	-5.94507084	-5.996104696	-3.975061297	-6.060059874	-5.022164689	3.342055975	0.064298601

PLXDC2	0.148541554	-0.301377583	-0.093883456	-0.449919137	-0.24242501	0.207494127	8	0.103941587	0.816496581	0.816496581	0.816496581	0.816496581	0.816496581	0.816496581	8	46	0	P_207634	P_207634	P_207634	chr10	20068176	20068236	+	PLXDC2	plexin domain containing 2 [Source:HGNC Symbol;Acc:HGNC:21013]	ENSG00000120594	protein_coding	exon	coding exon	1	1236	10:20068176:20068236	ENSG00000120594	PLXDC2	5.917137921	0.010882767	1.743909396	-3.538236846	-1.102211716	-5.282146241	-2.846121112	2.436025129	9.961963646	0.111892129	0.005404164	0.296285438	0.000361096	0.017427471	0.035172724	-5.551781484	-2.00197625	-6.421384402	0.076622269	-3.18149329	-4.533110103	9.785150559	0.002572417

PTPRJ	-0.248952866	0.311579845	-0.10973543	0.560532712	0.139217436	-0.421315276	8	0.069934769	0.816496581	0.816496581	0.816496581	0.816496581	0.816496581	0.816496581	8	47	0	P_211781	P_211781	P_211781	chr11	48136141	48136201	+	PTPRJ	"protein tyrosine phosphatase, receptor type, J [Source:HGNC Symbol;Acc:HGNC:9673]"	ENSG00000149177	protein_coding	exon	coding exon	1	1237	11:48136141:48136201	ENSG00000149177	PTPRJ	5.730582035	0.006134321	-3.892956224	4.872274478	-1.715968299	8.765230702	2.176987925	-6.588242777	9.961963646	0.003016697	0.000656604	0.117045031	5.38E-06	0.054622824	6.28E-05	-1.997450695	-0.023018427	-5.639780785	4.465604103	-4.249788953	1.96180399	27.80203375	3.72E-05

STT3B	0.030343489	0.03766871	0.035620348	0.007325221	0.005276859	-0.002048362	8	0.03580312	0.816496581	0.816496581	0.816496581	0.816496581	0.816496581	0.816496581	8	48	0	A_32_P13612	A_32_P13612	A_32_P13612	chr3	31530949	31531009	-	STT3B	"STT3B, subunit of the oligosaccharyltransferase complex (catalytic) [Source:HGNC Symbol;Acc:HGNC:30611]"	ENSG00000163527	protein_coding	promoter	promoter antisense	0	1238	3:31530949:31531009	ENSG00000163527	STT3B	8.750860744	0.00323609	0.653282212	0.810991064	0.766890707	0.157708851	0.113608495	-0.044100357	9.961963646	0.528363277	0.43631767	0.460934277	0.877837062	0.911805095	0.965695858	-6.770361245	-5.846786727	-6.735132476	-7.136028408	-6.289824846	-7.070316156	0.285388221	0.834910265

GBA2	-0.125932075	-0.107910676	-0.207233484	0.018021399	-0.081301409	-0.099322808	8	0.10408467	0.816496581	0.816496581	0.816496581	0.816496581	0.816496581	0.816496581	8	49	0	P_267295	P_267295	P_267295	chr9	35741720	35741780	-	GBA2	"glucosidase, beta (bile acid) 2 [Source:HGNC Symbol;Acc:HGNC:18986]"	ENSG00000070610	protein_coding	exon	coding exon	1	1239	9:35741720:35741780	ENSG00000070610	GBA2	8.602859247	0.01090667	-1.476848324	-1.265505247	-2.430297638	0.211343077	-0.953449314	-1.164792391	9.961963646	0.170615813	0.234487044	0.035518473	0.836880393	0.362924177	0.271237348	-5.923025922	-5.40174357	-4.519499418	-7.125241705	-5.834661161	-6.376824992	1.999900652	0.178251335

KIAA1755	-0.07722188	0.106596147	0.111657426	0.183818027	0.188879306	0.005061278	8	0.189518856	0.816496581	0.816496581	0.816496581	0.816496581	0.816496581	0.816496581	8	51	0	A_32_P113971	A_32_P113971	A_32_P113971	chr20	38213059	38213119	-	KIAA1755	KIAA1755 [Source:HGNC Symbol;Acc:HGNC:29372]	ENSG00000149633	protein_coding	exon	coding exon	1	1241	20:38213059:38213119	ENSG00000149633	KIAA1755	4.053629497	0.031050312	-0.536726473	0.740890718	0.776068858	1.277617192	1.312795332	0.03517814	9.961963646	0.603235845	0.475860733	0.455738087	0.230354108	0.218688974	0.972632593	-6.843408128	-5.90072536	-6.727837979	-6.323370404	-5.453564908	-7.070702516	0.799375335	0.522087424

TTF2	-0.342801774	-0.04051563	-0.265322298	0.302286144	0.077479476	-0.224806667	8	0.043991333	0.816496581	0.816496581	0.816496581	0.816496581	0.816496581	0.816496581	8	52	0	P_203643	P_203643	P_203643	chr1	117096173	117096233	+	TTF2	"transcription termination factor, RNA polymerase II [Source:HGNC Symbol;Acc:HGNC:12398]"	ENSG00000116830	protein_coding	exon	coding exon	1	1242	1:117096173:117096233	ENSG00000116830	TTF2	7.881030752	0.003760785	-6.846197613	-0.809149875	-5.298831625	6.037047738	1.547365988	-4.48968175	9.961963646	4.56E-05	0.437327637	0.000352592	0.000127745	0.152929686	0.001172571	2.338949015	-5.848258033	0.183194661	1.164110139	-5.15977655	-1.085086436	22.43338183	9.46E-05

TRNAU1AP	0.020861305	-0.081446879	-0.055651169	-0.102308184	-0.076512474	0.02579571	8	0.043163415	0.816496581	0.816496581	0.816496581	0.816496581	0.816496581	0.816496581	8	53	0	A_23_P51781	A_23_P51781	A_23_P51781	chr1	28571178	28571238	+	TRNAU1AP	tRNA selenocysteine 1 associated protein 1 [Source:HGNC Symbol;Acc:HGNC:30813]	ENSG00000180098	protein_coding	exon	coding exon	1	1243	1:28571178:28571238	ENSG00000180098	TRNAU1AP	8.474249186	0.003702839	0.419874685	-1.639278161	-1.12008891	-2.059152846	-1.539963595	0.519189251	9.961963646	0.683488744	0.132309333	0.288953049	0.066587611	0.154707665	0.614966647	-6.903005849	-4.934633713	-6.40209855	-5.220058794	-5.16950828	-6.92608927	1.833213767	0.205074691

SCOC	-0.22161539	0.155345226	-0.128231904	0.376960617	0.093383486	-0.283577131	8	0.066887587	0.816496581	0.816496581	0.816496581	0.816496581	0.816496581	0.816496581	8	54	0	P_138632	P_138632	P_138632	chr4	140387793	140387853	-	SCOC;CLGN	short coiled-coil protein [Source:HGNC Symbol;Acc:HGNC:20335];calmegin [Source:HGNC Symbol;Acc:HGNC:2060]	ENSG00000153130	protein_coding	3'ext	multiple non-Ref	0	1244	4:140387793:140387853	ENSG00000153130	SCOC	8.299144432	0.005799509	-3.564100372	2.498319174	-2.062272737	6.062419546	1.501827634	-4.560591912	9.961963646	0.005176801	0.031619847	0.066240864	0.000123528	0.164157289	0.001051431	-2.549636369	-3.657651894	-5.115856163	1.199248725	-5.219196283	-0.971987828	13.83413243	0.000693907

HSPA4	0.066435362	-0.037011411	0.105299093	-0.103446773	0.038863731	0.142310504	8	0.04156828	0.816496581	0.816496581	0.816496581	0.816496581	0.816496581	0.816496581	8	55	0	P_254803	P_254803	P_254803	chr5	133086798	133086858	+	HSPA4	heat shock 70kDa protein 4 [Source:HGNC Symbol;Acc:HGNC:5237]	ENSG00000170606	protein_coding	exon	coding exon	1	1245	5:133086798:133086858	ENSG00000170606	HSPA4	10.59803417	0.003594299	1.357181108	-0.756091122	2.151112521	-2.11327223	0.793931414	2.907203643	9.961963646	0.204674664	0.467097272	0.057059236	0.060810696	0.445735043	0.015698441	-6.076758166	-5.889395121	-4.974917612	-5.134346928	-5.971940286	-3.737026714	3.431496509	0.06045322

GLMN	0.084606343	-0.218846884	0.01388542	-0.303453227	-0.070720923	0.232732304	8	0.075318345	0.816496581	0.816496581	0.816496581	0.816496581	0.816496581	0.816496581	8	56	0	P_202828	P_202828	P_202828	chr1	92247061	92247121	-	GLMN	"glomulin, FKBP associated protein [Source:HGNC Symbol;Acc:HGNC:14373]"	ENSG00000174842	protein_coding	3'UTR;exon	multiple Ref	1	1246	1:92247061:92247121	ENSG00000174842	GLMN	9.294976671	0.006762294	1.260089862	-3.259409768	0.206803364	-4.519499629	-1.053286497	3.466213132	9.961963646	0.236354467	0.008624872	0.840329173	0.001119921	0.317086517	0.006093519	-6.194899626	-2.443115568	-7.023815124	-1.104333465	-5.738224197	-2.781312405	7.634044824	0.006115035

METAP1	-0.349658562	0.051488291	-0.305584446	0.401146853	0.044074116	-0.357072737	8	0.07645393	0.816496581	0.816496581	0.816496581	0.816496581	0.816496581	0.816496581	8	58	0	P_138248	P_138248	P_138248	chr4	99057758	99057818	+	METAP1	methionyl aminopeptidase 1 [Source:HGNC Symbol;Acc:HGNC:15789]	ENSG00000164024	protein_coding	exon	coding exon	1	1248	4:99057758:99057818	ENSG00000164024	METAP1	7.496171279	0.006900701	-5.155172353	0.759114863	-4.505367978	5.914287216	0.649804375	-5.264482842	9.961963646	0.000433461	0.465366404	0.001144554	0.000150453	0.530516238	0.000370338	0.006300464	-5.887116905	-1.039618311	0.992808702	-6.076881554	0.113106636	18.42770276	0.00021713

ERBB4	0.15299545	0.087631412	0.30985275	-0.065364038	0.1568573	0.222221338	8	0.068016123	0.816496581	0.816496581	0.816496581	0.816496581	0.816496581	0.816496581	8	59	0	P_239127	P_239127	P_239127	chr2	211623921	211623981	-	ERBB4	v-erb-b2 avian erythroblastic leukemia viral oncogene homolog 4 [Source:HGNC Symbol;Acc:HGNC:3432]	ENSG00000178568	protein_coding	exon	coding exon	1	1249	2:211623921:211623981	ENSG00000178568	ERBB4	4.153377939	0.005921769	2.43499687	1.394696469	4.931456957	-1.040300401	2.496460087	3.536760488	9.961963646	0.035234552	0.193425121	0.000601396	0.322789905	0.031720563	0.005417451	-4.465407644	-5.249071239	-0.371944222	-6.587955046	-3.746472644	-2.661366625	8.669478058	0.003956448

CENPJ	-0.510572106	-0.069674182	-0.265055016	0.440897924	0.245517089	-0.195380834	8	0.081311091	0.816496581	0.816496581	0.816496581	0.816496581	0.816496581	0.816496581	8	60	0	P_113434	P_113434	P_113434	chr13	24883033	24883093	-	CENPJ	centromere protein J [Source:HGNC Symbol;Acc:HGNC:17272]	ENSG00000151849	protein_coding	3'UTR	3'UTR	1	1250	13:24883033:24883093	ENSG00000151849	CENPJ	7.465789571	0.007516074	-7.212854918	-0.984287547	-3.744433658	6.228567371	3.46842126	-2.760146111	9.961963646	2.94E-05	0.348275468	0.003843978	9.94E-05	0.006071085	0.020191812	2.792534447	-5.695659429	-2.288664284	1.427121506	-2.172184496	-3.987669206	20.90971426	0.000127693

RPA2	-0.375502397	-0.07277571	-0.319030353	0.302726686	0.056472044	-0.246254643	8	0.050255657	0.816496581	0.816496581	0.816496581	0.816496581	0.816496581	0.816496581	8	61	0	P_200808	P_200808	P_200808	chr1	27897004	27897064	-	RPA2	"replication protein A2, 32kDa [Source:HGNC Symbol;Acc:HGNC:10290]"	ENSG00000117748	protein_coding	exon	coding exon	1	1251	1:27897004:27897064	ENSG00000117748	RPA2	10.91966606	0.004234903	-7.067024306	-1.369652282	-6.004210037	5.697372023	1.062814269	-4.634557755	9.961963646	3.50E-05	0.200875325	0.000133433	0.000201904	0.312950806	0.000939098	2.614230408	-5.279450977	1.194371951	0.684883474	-5.728619706	-0.854728109	23.82301097	7.30E-05

GAD1	0.27942819	0.239443701	0.388415025	-0.039984488	0.108986836	0.148971324	8	0.158851734	0.816496581	0.816496581	0.816496581	0.816496581	0.816496581	0.816496581	8	62	0	A_23_P209578	A_23_P209578	A_23_P209578	chr2	170842648	170842708	+	GAD1	"glutamate decarboxylase 1 (brain, 67kDa) [Source:HGNC Symbol;Acc:HGNC:4092]"	ENSG00000128683	protein_coding	3'UTR	3'UTR	1	1252	2:170842648:170842708	ENSG00000128683	GAD1	4.503289594	0.02247086	2.283000456	1.956316863	3.173451042	-0.326683593	0.890450586	1.217134179	9.961963646	0.0456475	0.079027442	0.00997519	0.750667268	0.394220452	0.251600284	-4.715290328	-4.487579604	-3.259079305	-7.091562559	-5.89142358	-6.317266829	3.581849716	0.054586453

PHACTR1	0.216982825	0.079168287	0.321368064	-0.137814538	0.104385239	0.242199777	8	0.122995258	0.816496581	0.816496581	0.816496581	0.816496581	0.816496581	0.816496581	8	63	0	P_313221	P_313221	P_313221	chr6	12896317	12896377	-	PHACTR1	phosphatase and actin regulator 1 [Source:HGNC Symbol;Acc:HGNC:20990]	ENSG00000112137	protein_coding	intron	intron antisense	0	1253	6:12896317:12896377	ENSG00000112137	PHACTR1	3.741546154	0.014355159	2.218027792	0.809268941	3.285067831	-1.408758851	1.067040039	2.47579889	9.961963646	0.050962556	0.437262277	0.008259337	0.189345464	0.31112972	0.032861431	-4.820644227	-5.848162975	-3.068230882	-6.160478882	-5.724338066	-4.466916435	4.269831708	0.03503486

GAB1	0.216939961	-0.057895648	0.02492345	-0.274835609	-0.192016511	0.082819098	8	0.134071396	0.816496581	0.816496581	0.816496581	0.816496581	0.816496581	0.816496581	8	64	0	A_23_P18505	A_23_P18505	A_23_P18505	chr4	143469398	143469458	+	GAB1	GRB2-associated binding protein 1 [Source:HGNC Symbol;Acc:HGNC:4066]	ENSG00000109458	protein_coding	3'UTR;exon	multiple Ref	1	1254	4:143469398:143469458	ENSG00000109458	GAB1	4.895408915	0.016641701	2.059616349	-0.549658174	0.2366219	-2.609274522	-1.822994449	0.786280073	9.961963646	0.066535988	0.594660842	0.817744577	0.026149004	0.098409256	0.450002001	-5.073042104	-6.025282543	-7.016630173	-4.317518654	-4.779305575	-6.74377075	2.55833203	0.11400457

BUB1B	-0.459378468	-0.056727148	-0.284789787	0.402651321	0.174588681	-0.22806264	8	0.057613185	0.816496581	0.816496581	0.816496581	0.816496581	0.816496581	0.816496581	8	65	0	P_222808	P_222808	P_222808	chr15	40209712	40209772	+	BUB1B	BUB1 mitotic checkpoint serine/threonine kinase B [Source:HGNC Symbol;Acc:HGNC:1149]	ENSG00000156970	protein_coding	exon	coding exon	1	1255	15:40209712:40209772	ENSG00000156970	BUB1B	9.214011336	0.004872246	-8.060308399	-0.995341174	-4.996954959	7.064967225	3.06335344	-4.001613785	9.961963646	1.13E-05	0.343131823	0.000546011	3.50E-05	0.012026852	0.002530929	3.776348481	-5.685199795	-0.27155345	2.517041759	-2.828000113	-1.880547245	27.70660724	3.78E-05

RUNDC3B	-0.086061406	0.08385336	0.085967158	0.169914766	0.172028564	0.002113798	8	0.157818738	0.816496581	0.816496581	0.816496581	0.816496581	0.816496581	0.816496581	8	66	0	P_261634	P_261634	P_261634	chr7	87710595	87710655	+	RUNDC3B	RUN domain containing 3B [Source:HGNC Symbol;Acc:HGNC:30286]	ENSG00000105784	protein_coding	exon	coding exon	1	1256	7:87710595:87710655	ENSG00000105784	RUNDC3B	3.602313062	0.022208166	-0.707290336	0.689143642	0.70651576	1.396433978	1.413806097	0.017372118	9.961963646	0.495598639	0.506466088	0.496059551	0.192917042	0.187899167	0.986482759	-6.732059904	-5.937749993	-6.781180429	-6.176249173	-5.330891248	-7.071213658	0.903920071	0.473199042

ANKRD13C	0.133268729	0.109036667	0.132245554	-0.024232061	-0.001023175	0.023208887	8	0.091073231	0.816496581	0.816496581	0.816496581	0.816496581	0.816496581	0.816496581	8	67	0	A_23_P114616	A_23_P114616	A_23_P114616	chr1	70261342	70261402	-	ANKRD13C	ankyrin repeat domain 13C [Source:HGNC Symbol;Acc:HGNC:25374]	ENSG00000118454	protein_coding	3'UTR	3'UTR	1	1257	1:70261342:70261402	ENSG00000118454	ANKRD13C	7.248559011	0.008867486	1.733299576	1.418136207	1.719992118	-0.315163369	-0.013307458	0.301855911	9.961963646	0.113824007	0.186665881	0.116289918	0.759140224	0.989645233	0.768969397	-5.56718864	-5.220310938	-5.633968897	-7.095564571	-6.296591067	-7.021828861	1.360738773	0.310372511

ICAM1	-0.226762592	0.003079183	0.072939823	0.229841775	0.299702415	0.069860639	8	0.119017907	0.816496581	0.816496581	0.816496581	0.816496581	0.816496581	0.816496581	8	68	0	P_124564	P_124564	P_124564	chr19	10285520	10285580	+	ICAM1	intercellular adhesion molecule 1 [Source:HGNC Symbol;Acc:HGNC:5344]	ENSG00000090339	protein_coding	3'UTR	3'UTR	1	1258	19:10285520:10285580	ENSG00000090339	ICAM1	4.505669572	0.013582162	-2.383046904	0.03235912	0.766524216	2.415406024	3.14957112	0.734165096	9.961963646	0.038501118	0.974824817	0.461142558	0.036433138	0.010387498	0.479771325	-4.551303409	-6.181535446	-6.735422153	-4.64228487	-2.687801855	-6.784665876	3.760083654	0.048485511

DNA2	-0.385791435	0.067255136	-0.211564665	0.453046571	0.17422677	-0.278819801	8	0.082771965	0.816496581	0.816496581	0.816496581	0.816496581	0.816496581	0.816496581	8	70	0	P_106112	P_106112	P_106112	chr10	68414552	68414612	-	DNA2	DNA replication helicase/nuclease 2 [Source:HGNC Symbol;Acc:HGNC:2939]	ENSG00000138346	protein_coding	3'UTR	3'UTR	1	1260	10:68414552:68414612	ENSG00000138346	DNA2	8.702983398	0.00770857	-5.381598675	0.938175704	-2.951221867	6.319774379	2.430376808	-3.889397571	9.961963646	0.000313464	0.370342559	0.014560995	8.83E-05	0.035513671	0.00303417	0.342498099	-5.73825852	-3.639211461	1.550576413	-3.851947942	-2.06725175	16.58753026	0.000334975

GRIPAP1	-0.096293423	-0.215636638	-0.159671514	-0.119343215	-0.063378091	0.055965124	8	0.087728721	0.816496581	0.816496581	0.816496581	0.816496581	0.816496581	0.816496581	8	71	0	P_150901	P_150901	P_150901	chrX	48989648	48989708	-	GRIPAP1	GRIP1 associated protein 1 [Source:HGNC Symbol;Acc:HGNC:18706]	ENSG00000068400	protein_coding	3'UTR;exon	multiple Ref	1	1261	X:48989648:48989708	ENSG00000068400	GRIPAP1	6.909314849	0.008387255	-1.287752262	-2.883754257	-2.135320846	-1.596001995	-0.847568584	0.748433411	9.961963646	0.226941886	0.016340705	0.058597051	0.141688857	0.41657665	0.471499175	-6.161871714	-3.043709411	-5.000131707	-5.910114882	-5.92817747	-6.773717751	3.059947844	0.07847856

COG3	0.086714912	0.038706945	-0.006841597	-0.048007967	-0.093556509	-0.045548543	8	0.035070771	0.816496581	0.816496581	0.816496581	0.816496581	0.816496581	0.816496581	8	72	0	P_219003	P_219003	P_219003	chr13	45476287	45476347	+	COG3	component of oligomeric golgi complex 3 [Source:HGNC Symbol;Acc:HGNC:18619]	ENSG00000136152	protein_coding	exon	coding exon	1	1262	13:45476287:45476347	ENSG00000136152	COG3	7.614691752	0.003194408	1.879075457	0.838763127	-0.148254517	-1.04031233	-2.027329975	-0.987017644	9.961963646	0.08977285	0.421272626	0.885099844	0.32278463	0.070224215	0.346999813	-5.351354762	-5.824242332	-7.035130746	-6.587942811	-4.478192245	-6.563782139	1.737157993	0.222648622

SMC2	-0.39186842	-0.047454287	-0.251069105	0.344414134	0.140799315	-0.203614818	8	0.057945914	0.816496581	0.816496581	0.816496581	0.816496581	0.816496581	0.816496581	8	73	0	P_268288	P_268288	P_268288	chr9	104098508	104098568	+	SMC2	structural maintenance of chromosomes 2 [Source:HGNC Symbol;Acc:HGNC:14011]	ENSG00000136824	protein_coding	"5'UTR,exon;exon"	multiple Ref	1	1263	9:104098508:104098568	ENSG00000136824	SMC2	10.18314693	0.004903123	-6.854084927	-0.830012559	-4.391394866	6.024072369	2.462690062	-3.561382307	9.961963646	4.52E-05	0.42597478	0.001365493	0.000129961	0.033606165	0.005200221	2.348892311	-5.83141664	-1.222350377	1.146104877	-3.800444741	-2.619575672	20.33158068	0.000143788

CAMKK2	-0.181148208	0.096842676	-0.227481012	0.277990884	-0.046332803	-0.324323687	8	0.048625352	0.816496581	0.816496581	0.816496581	0.816496581	0.816496581	0.816496581	8	74	0	P_113039	P_113039	P_113039	chr12	121240796	121240856	-	CAMKK2	"calcium/calmodulin-dependent protein kinase kinase 2, beta [Source:HGNC Symbol;Acc:HGNC:1470]"	ENSG00000110931	protein_coding	exon	coding exon	1	1264	12:121240796:121240856	ENSG00000110931	CAMKK2	8.174437341	0.004105446	-3.462577448	1.851110026	-4.34821094	5.313687474	-0.885633492	-6.199320966	9.961963646	0.006130643	0.093987757	0.001460625	0.000345201	0.396689305	0.000103219	-2.721745303	-4.640075283	-1.292021808	0.123696566	-5.89562928	1.444418533	16.96236516	0.00030571

AAK1	-0.233239081	-0.078399553	-0.237794848	0.154839528	-0.004555767	-0.159395295	8	0.067047615	0.816496581	0.816496581	0.816496581	0.816496581	0.816496581	0.816496581	8	75	0	A_24_P356	A_24_P356	A_24_P356	chr2	69481289	69481349	-	AAK1	AP2 associated kinase 1 [Source:HGNC Symbol;Acc:HGNC:19679]	ENSG00000115977	protein_coding	3'UTR	3'UTR	1	1265	2:69481289:69481349	ENSG00000115977	AAK1	6.947828477	0.005816721	-3.745482911	-1.258983627	-3.818641951	2.486499284	-0.07315904	-2.559658324	9.961963646	0.003837367	0.236737429	0.003404472	0.032265611	0.943127638	0.028468496	-2.243940291	-5.409176342	-2.164142594	-4.523833101	-6.293839259	-4.326574776	7.155931672	0.007577736

AP1G1	-0.028485719	-0.081445731	-0.073523394	-0.052960012	-0.045037675	0.007922337	8	0.070705808	0.816496581	0.816496581	0.816496581	0.816496581	0.816496581	0.816496581	8	76	0	P_226927	P_226927	P_226927	chr16	71756018	71756078	-	AP1G1	"adaptor-related protein complex 1, gamma 1 subunit [Source:HGNC Symbol;Acc:HGNC:555]"	ENSG00000166747	protein_coding	3'UTR;exon	multiple Ref	1	1266	16:71756018:71756078	ENSG00000166747	AP1G1	7.930837823	0.006221403	-0.442311905	-1.26464832	-1.141634262	-0.822336415	-0.699322356	0.123014059	9.961963646	0.667711754	0.234781737	0.280304875	0.430128637	0.500352605	0.90454236	-6.892644426	-5.402721776	-6.378537019	-6.791885293	-6.042953988	-7.063117927	0.713120897	0.566236368

NFRKB	-0.025213149	0.02072184	0.053750915	0.04593499	0.078964064	0.033029075	8	0.077522436	0.816496581	0.816496581	0.816496581	0.816496581	0.816496581	0.816496581	8	77	0	P_213864	P_213864	P_213864	chr11	129878470	129878530	-	NFRKB	nuclear factor related to kappaB binding protein [Source:HGNC Symbol;Acc:HGNC:7802]	ENSG00000170322	protein_coding	exon	coding exon	1	1267	11:129878470:129878530	ENSG00000170322	NFRKB	7.095812339	0.007032823	-0.368220423	0.30262799	0.784994541	0.670848413	1.153214964	0.48236655	9.961963646	0.720412677	0.768397927	0.45072153	0.517567933	0.275741085	0.639968752	-6.924875002	-6.134036896	-6.720670515	-6.908932311	-5.634159862	-6.945738071	0.476001041	0.705989192

CEP112	-0.1053993	0.022400113	-0.031718132	0.127799413	0.073681168	-0.054118245	8	0.101223468	0.816496581	0.816496581	0.816496581	0.816496581	0.816496581	0.816496581	8	78	0	P_229743	P_229743	P_229743	chr17	66096250	66096310	-	CEP112	centrosomal protein 112kDa [Source:HGNC Symbol;Acc:HGNC:28514]	ENSG00000154240	protein_coding	exon	coding exon	1	1268	17:66096250:66096310	ENSG00000154240	CEP112	6.675509124	0.010434934	-1.263684069	0.268565981	-0.38028429	1.53225005	0.883399779	-0.648850271	9.961963646	0.235113701	0.793743305	0.711716891	0.156579645	0.397837779	0.531107776	-6.190637419	-6.144206472	-6.968716214	-5.99753944	-5.897572914	-6.846141788	0.893805553	0.477715235

ZNF331	0.066518576	0.142941751	0.127146192	0.076423175	0.060627616	-0.015795559	8	0.20312759	0.816496581	0.816496581	0.816496581	0.816496581	0.816496581	0.816496581	8	79	0	A_23_P50376	A_23_P50376	A_23_P50376	chr19	53578062	53578122	+	ZNF331	zinc finger protein 331 [Source:HGNC Symbol;Acc:HGNC:15489]	ENSG00000130844	protein_coding	3'UTR	3'UTR	1	1269	19:53578062:53578122	ENSG00000130844	ZNF331	6.892395103	0.035341371	0.433358059	0.931243022	0.828337441	0.497884963	0.394979382	-0.102905581	9.961963646	0.673987962	0.373745473	0.426878936	0.629371716	0.701182904	0.92007995	-6.896841541	-5.744516858	-6.684843219	-7.015719821	-6.214370732	-7.065596507	0.3592739	0.783767078

HERPUD1	0.090708041	0.041336982	0.182195661	-0.049371059	0.09148762	0.140858679	8	0.111818247	0.816496581	0.816496581	0.816496581	0.816496581	0.816496581	0.816496581	8	80	0	P_226464	P_226464	P_226464	chr16	56936757	56936817	+	HERPUD1	"homocysteine-inducible, endoplasmic reticulum stress-inducible, ubiquitin-like domain member 1 [Source:HGNC Symbol;Acc:HGNC:13744]"	ENSG00000051108	protein_coding	3'UTR;exon	multiple Ref	1	1270	16:56936757:56936817	ENSG00000051108	HERPUD1	9.52840881	0.012247532	1.003846073	0.45746735	2.016319581	-0.546378723	1.012473508	1.558852231	9.961963646	0.339212541	0.657149738	0.071525669	0.596829344	0.335270809	0.150206348	-6.474744171	-6.07295348	-5.18784122	-6.988769988	-5.778584506	-5.884452327	1.506030093	0.272519963

GSTM1	0.094199304	0.306789277	0.283718271	0.212589974	0.189518967	-0.023071007	8	0.264124196	0.816496581	0.816496581	0.816496581	0.816496581	0.816496581	0.816496581	8	83	0	P_102639	P_102639	P_102639	chr1	109689265	109689325	+	GSTM1	glutathione S-transferase mu 1 [Source:HGNC Symbol;Acc:HGNC:4632]	ENSG00000134184	protein_coding	"3'UTR,exon;exon"	multiple Ref	1	1273	1:109689265:109689325	ENSG00000134184	GSTM1	4.05330624	0.058229045	0.478105222	1.557098088	1.440002012	1.078992866	0.961896789	-0.117096076	9.961963646	0.642893748	0.150619467	0.180543242	0.306022452	0.358867696	0.909111042	-6.875044568	-5.043670043	-6.019164999	-6.547682704	-5.826804891	-7.063893076	1.13832123	0.380314271

NUP155	-0.25347727	-0.053391927	-0.245888139	0.200085343	0.00758913	-0.192496213	8	0.055951494	0.816496581	0.816496581	0.816496581	0.816496581	0.816496581	0.816496581	8	84	0	P_252507	P_252507	P_252507	chr5	37305056	37305116	-	NUP155	nucleoporin 155kDa [Source:HGNC Symbol;Acc:HGNC:8063]	ENSG00000113569	protein_coding	exon	coding exon	1	1274	5:37305056:37305116	ENSG00000113569	NUP155	8.653957599	0.004720702	-4.518365423	-0.95173913	-4.383085187	3.566626292	0.135280236	-3.431346057	9.961963646	0.001121877	0.363749433	0.001383277	0.005155134	0.895084702	0.006459353	-0.978526433	-5.725903168	-1.23573858	-2.681811431	-6.286960564	-2.840696248	10.84102147	0.001766172

CLCN5	-0.05324093	0.180251765	0.15596473	0.233492696	0.20920566	-0.024287035	8	0.074468764	0.816496581	0.816496581	0.816496581	0.816496581	0.816496581	0.816496581	8	85	0	P_150942	P_150942	P_150942	chrX	50092159	50092219	+	CLCN5	"chloride channel, voltage-sensitive 5 [Source:HGNC Symbol;Acc:HGNC:2023]"	ENSG00000171365	protein_coding	exon	coding exon	1	1275	X:50092159:50092219	ENSG00000171365	CLCN5	5.74704078	0.006660101	-0.799007525	2.705108945	2.34062388	3.50411647	3.139631405	-0.364485065	9.961963646	0.442918976	0.022188735	0.041387135	0.005720063	0.01056422	0.723113746	-6.660778587	-3.32933976	-4.667562646	-2.788208398	-2.703954814	-6.99928551	5.950585755	0.013598599

BTF3L4	0.152317852	0.002568676	0.046576276	-0.149749175	-0.105741575	0.0440076	8	0.131838835	0.816496581	0.816496581	0.816496581	0.816496581	0.816496581	0.816496581	8	86	0	P_101843	P_101843	P_101843	chr1	52088226	52088286	+	BTF3L4	basic transcription factor 3-like 4 [Source:HGNC Symbol;Acc:HGNC:30547]	ENSG00000134717	protein_coding	3'UTR	3'UTR	1	1276	1:52088226:52088286	ENSG00000134717	BTF3L4	6.915476412	0.016164959	1.46726694	0.024743876	0.44866593	-1.442523065	-1.018601011	0.423922054	9.961963646	0.17315089	0.980747892	0.663274107	0.179848513	0.332492031	0.680630655	-5.935646561	-6.181764669	-6.938292095	-6.1168071	-5.772606552	-6.974083581	0.942152728	0.456527964

IRAK3	-0.076275761	-0.037587746	-0.01043895	0.038688015	0.065836811	0.027148796	8	0.152855312	0.816496581	0.816496581	0.816496581	0.816496581	0.816496581	0.816496581	8	88	0	P_216024	P_216024	P_216024	chr12	66245150	66245210	+	IRAK3	interleukin-1 receptor-associated kinase 3 [Source:HGNC Symbol;Acc:HGNC:17020]	ENSG00000090376	protein_coding	exon	coding exon	1	1278	12:66245150:66245210	ENSG00000090376	IRAK3	4.470245133	0.020969849	-0.645111115	-0.317902728	-0.08828863	0.327208387	0.556822485	0.229614098	9.961963646	0.533429756	0.757122382	0.931396897	0.750282107	0.589938046	0.823037435	-6.775913739	-6.129089981	-7.042865414	-7.091376902	-6.134371001	-7.042652453	0.16581077	0.916974371

NDUFAF4	0.132744623	0.070215124	-0.03948134	-0.062529499	-0.172225962	-0.109696464	8	0.09675065	0.816496581	0.816496581	0.816496581	0.816496581	0.816496581	0.816496581	8	89	0	A_24_P171983	A_24_P171983	A_24_P171983	chr6	96891308	96891368	-	NDUFAF4	"NADH dehydrogenase (ubiquinone) complex I, assembly factor 4 [Source:HGNC Symbol;Acc:HGNC:21034]"	ENSG00000123545	protein_coding	exon	coding exon	1	1279	6:96891308:96891368	ENSG00000123545	NDUFAF4	8.655463324	0.009723827	1.64870879	0.872082724	-0.490364356	-0.776626066	-2.139073146	-1.362447081	9.961963646	0.130340475	0.403692064	0.634496486	0.455423856	0.058228089	0.203063078	-5.688190457	-5.796333875	-6.917375807	-6.829429131	-4.308191497	-6.14206171	1.792378362	0.21234226

WDR36	-0.237103532	-0.057868555	-0.223509777	0.179234977	0.013593756	-0.165641221	8	0.055400581	0.816496581	0.816496581	0.816496581	0.816496581	0.816496581	0.816496581	8	90	0	P_312767	P_312767	P_312767	chr5	111128381	111128441	+	WDR36	WD repeat domain 36 [Source:HGNC Symbol;Acc:HGNC:30696]	ENSG00000134987	protein_coding	3'UTR	3'UTR	1	1280	5:111128381:111128441	ENSG00000134987	WDR36	8.550291727	0.004671438	-4.248722211	-1.036962268	-4.00513203	3.211759943	0.243590181	-2.968169762	9.961963646	0.001707454	0.324268414	0.002516645	0.00934845	0.81249108	0.014145667	-1.41209811	-5.64497188	-1.853520948	-3.288294367	-6.265221871	-3.632814396	9.058797266	0.003390241

BRI3BP	-0.308030207	0.002410103	-0.150295682	0.31044031	0.157734526	-0.152705784	8	0.060285322	0.816496581	0.816496581	0.816496581	0.816496581	0.816496581	0.816496581	8	91	0	P_113231	P_113231	P_113231	chr12	125025980	125026040	+	BRI3BP	BRI3 binding protein [Source:HGNC Symbol;Acc:HGNC:14251]	ENSG00000184992	protein_coding	3'UTR	3'UTR	1	1281	12:125025980:125026040	ENSG00000184992	BRI3BP	10.5897462	0.005125241	-5.269650193	0.041231018	-2.571194801	5.310881211	2.698455392	-2.612425819	9.961963646	0.000367608	0.967926378	0.027911576	0.000346585	0.022443189	0.026008202	0.177209339	-6.181191314	-4.284206518	0.119514094	-3.421134919	-4.237788847	12.78230754	0.000944983

HECW2	-0.079573324	0.012878042	0.038362334	0.092451366	0.117935658	0.025484292	8	0.060539408	0.816496581	0.816496581	0.816496581	0.816496581	0.816496581	0.816496581	8	92	0	P_238420	P_238420	P_238420	chr2	196271192	196271252	-	HECW2	"HECT, C2 and WW domain containing E3 ubiquitin protein ligase 2 [Source:HGNC Symbol;Acc:HGNC:29853]"	ENSG00000138411	protein_coding	exon	coding exon	1	1282	2:196271192:196271252	ENSG00000138411	HECW2	3.858468255	0.005149895	-1.358044341	0.219784108	0.654713769	1.577828449	2.01275811	0.434929661	9.961963646	0.204409748	0.830477502	0.527478567	0.14580069	0.071951501	0.672884427	-6.075680457	-6.156686483	-6.817963029	-5.935254634	-4.500107415	-6.969013426	1.50852354	0.271917751

NUP58	-0.278013325	-0.014693296	-0.251457767	0.263320029	0.026555558	-0.236764471	8	0.071800282	0.816496581	0.816496581	0.816496581	0.816496581	0.816496581	0.816496581	8	93	0	P_218394	P_218394	P_218394	chr13	25315407	25315467	+	NUPL1	nucleoporin like 1 [Source:HGNC Symbol;Acc:HGNC:20261]	ENSG00000139496	protein_coding	exon	coding exon	1	1283	13:25315407:25315467	ENSG00000139496	NUP58	8.116042771	0.006346655	-4.27404198	-0.225887602	-3.865789721	4.048154378	0.408252259	-3.639902119	9.961963646	0.001640724	0.825855803	0.0031528	0.002348657	0.691725113	0.004565926	-1.370995327	-6.155259906	-2.085289303	-1.87260417	-6.208793564	-2.486577335	10.51098357	0.00198051

SEPTIN3	0.390779255	-0.263766	0.134106694	-0.654545254	-0.25667256	0.397872694	8	0.068808905	0.816496581	0.816496581	0.816496581	0.816496581	0.816496581	0.816496581	8	94	0	A_23_P503127	A_23_P503127	A_23_P503127	chr22	41997415	41997475	+	Sep-03	septin 3 [Source:HGNC Symbol;Acc:HGNC:10750]	ENSG00000100167	protein_coding	3'UTR	3'UTR	1	1284	22:41997415:41997475	ENSG00000100167	SEPTIN3	8.040720143	0.006008878	6.174196091	-4.167424411	2.118845916	-10.3416205	-4.055350175	6.286270328	9.961963646	0.000106659	0.001941759	0.060243672	1.20E-06	0.00232173	9.22E-05	1.460648459	-1.036620743	-5.026364212	6.003586724	-1.246404834	1.561908034	37.14830098	1.02E-05

SP6	0.126981285	0.047056769	0.060754192	-0.079924516	-0.066227093	0.013697423	8	0.138692162	0.816496581	0.816496581	0.816496581	0.816496581	0.816496581	0.816496581	8	95	0	P_121860	P_121860	P_121860	chr17	47844916	47844976	-	SP6	Sp6 transcription factor [Source:HGNC Symbol;Acc:HGNC:14530]	ENSG00000189120	protein_coding	3'UTR	3'UTR	1	1285	17:47844916:47844976	ENSG00000189120	SP6	8.891043828	0.017653852	1.170484339	0.433758495	0.560018196	-0.736725844	-0.610466144	0.1262597	9.961963646	0.26904457	0.67370671	0.587837859	0.47828	0.555223428	0.902038266	-6.29826208	-6.083865639	-6.878509572	-6.860643934	-6.102205532	-7.062676631	0.467195966	0.711677595

DNAI4	0.201169534	0.118140413	0.241483286	-0.08302912	0.040313753	0.123342873	8	0.082205775	0.816496581	0.816496581	0.816496581	0.816496581	0.816496581	0.816496581	8	97	0	P_102083	P_102083	P_102083	chr1	66822360	66822420	-	WDR78	WD repeat domain 78 [Source:HGNC Symbol;Acc:HGNC:26252]	ENSG00000152763	protein_coding	3'UTR;exon	multiple Ref	1	1287	1:66822360:66822420	ENSG00000152763	DNAI4	4.440576525	0.007633557	2.819969114	1.65607739	3.385082207	-1.163891724	0.565113093	1.729004816	9.961963646	0.018225389	0.12882042	0.006980129	0.271585617	0.584497835	0.114614599	-3.81767488	-4.911958402	-2.897520876	-6.455305041	-6.129580181	-5.644606178	4.469509013	0.03101232

LRBA	0.309586621	-0.22445566	0.069077473	-0.53404228	-0.240509147	0.293533133	8	0.073236669	0.816496581	0.816496581	0.816496581	0.816496581	0.816496581	0.816496581	8	98	0	P_251161	P_251161	P_251161	chr4	150588059	150588119	-	LRBA	"LPS-responsive vesicle trafficking, beach and anchor containing [Source:HGNC Symbol;Acc:HGNC:1742]"	ENSG00000198589	protein_coding	exon	coding exon	1	1288	4:150588059:150588119	ENSG00000198589	LRBA	8.445230628	0.006513955	4.697918019	-3.406071896	1.048237505	-8.103989915	-3.649680514	4.454309401	9.961963646	0.000852994	0.00673868	0.319294862	1.08E-05	0.004492768	0.00123845	-0.695117503	-2.210325682	-6.478127485	3.747158247	-1.882362836	-1.14175011	22.267709	9.77E-05

USP31	-0.26349263	0.09094421	-0.214561858	0.35443684	0.048930772	-0.305506068	8	0.07411326	0.816496581	0.816496581	0.816496581	0.816496581	0.816496581	0.816496581	8	99	0	P_225886	P_225886	P_225886	chr16	23106221	23106281	-	USP31	ubiquitin specific peptidase 31 [Source:HGNC Symbol;Acc:HGNC:20060]	ENSG00000103404	protein_coding	exon	coding exon	1	1289	16:23106221:23106281	ENSG00000103404	USP31	7.092275035	0.006617682	-3.966990413	1.369202659	-3.230317428	5.336193073	0.736672985	-4.599520088	9.961963646	0.002676128	0.201011263	0.009059512	0.000334318	0.478310755	0.000990634	-1.874502592	-5.279993047	-3.16181451	0.157198131	-6.015877213	-0.910182652	13.03665136	0.000875437

SIAE	-0.057222697	0.009870669	-0.000544878	0.067093366	0.056677819	-0.010415547	8	0.098589882	0.816496581	0.816496581	0.816496581	0.816496581	0.816496581	0.816496581	8	102	0	P_213726	P_213726	P_213726	chr11	124639713	124639773	-	SIAE	sialic acid acetylesterase [Source:HGNC Symbol;Acc:HGNC:18187]	ENSG00000110013	protein_coding	exon	coding exon	1	1292	11:124639713:124639773	ENSG00000110013	SIAE	7.743146997	0.010012346	-0.700399829	0.120815957	-0.006669252	0.821215787	0.693730577	-0.12748521	9.961963646	0.499708116	0.906238877	0.994810411	0.430737321	0.503705497	0.901093046	-6.737100151	-6.174398433	-7.047096312	-6.792828188	-6.046898397	-7.062507025	0.279518476	0.838998819

DMD	0.058648006	0.035188557	0.036966626	-0.023459449	-0.02168138	0.001778069	8	0.053101334	0.816496581	0.816496581	0.816496581	0.816496581	0.816496581	0.816496581	8	104	0	P_269902	P_269902	P_269902	chrX	31679374	31679434	-	DMD	dystrophin [Source:HGNC Symbol;Acc:HGNC:2928]	ENSG00000198947	protein_coding	exon	coding exon	1	1294	X:31679374:31679434	ENSG00000198947	DMD	4.02045159	0.004471098	1.074216377	0.644525309	0.677093015	-0.429691068	-0.397123362	0.032567706	9.961963646	0.308055626	0.533794074	0.51376229	0.676565974	0.699651492	0.974662601	-6.402818598	-5.967722114	-6.802385262	-7.049572551	-6.213481922	-7.070799116	0.395201781	0.759328999

TRIM23	0.063818507	0.088452491	0.166477714	0.024633984	0.102659207	0.078025223	8	0.046266342	0.816496581	0.816496581	0.816496581	0.816496581	0.816496581	0.816496581	8	105	0	P_253089	P_253089	P_253089	chr5	65610860	65610920	-	TRIM23	tripartite motif containing 23 [Source:HGNC Symbol;Acc:HGNC:660]	ENSG00000113595	protein_coding	3'UTR;exon	multiple Ref	1	1295	5:65610860:65610920	ENSG00000113595	TRIM23	8.221294104	0.003925682	1.247483226	1.729012549	3.254199543	0.481529323	2.006716317	1.525186994	9.961963646	0.240748769	0.114613171	0.008701108	0.64054292	0.07267938	0.158311127	-6.209779747	-4.812081985	-3.120984274	-7.024272822	-4.509175607	-5.93016467	3.620081615	0.053204226

IRF1-AS1	-0.078092668	-0.148189021	-0.199896024	-0.070096353	-0.121803356	-0.051707003	8	0.127447533	0.816496581	0.816496581	0.816496581	0.816496581	0.816496581	0.816496581	8	106	0	A_24_P127641	A_24_P127641	A_24_P127641	chr5	132475922	132475982	+	C5orf56	chromosome 5 open reading frame 56 [Source:HGNC Symbol;Acc:HGNC:33838]	ENSG00000197536	protein_coding	"3'UTR,exon;exon"	multiple Ref	1	1296	5:132475922:132475982	ENSG00000197536	IRF1-AS1	5.483641037	0.015250597	-0.774484022	-1.469664594	-1.98246879	-0.695180572	-1.207984768	-0.512804196	9.961963646	0.456632609	0.172513476	0.075671055	0.502834748	0.254948091	0.619266356	-6.680593373	-5.156007647	-5.240437515	-6.8915736	-5.574100703	-6.929594674	1.482565565	0.278262063

MTMR14	-0.110341047	0.051083142	-0.017815067	0.161424189	0.09252598	-0.06889821	8	0.08410661	0.816496581	0.816496581	0.816496581	0.816496581	0.816496581	0.816496581	8	107	0	P_243148	P_243148	P_243148	chr3	9683184	9683244	+	MTMR14	myotubularin related protein 14 [Source:HGNC Symbol;Acc:HGNC:26190]	ENSG00000163719	protein_coding	"3'UTR;3'UTR,exon;exon"	multiple Ref	1	1297	3:9683184:9683244	ENSG00000163719	MTMR14	7.168637969	0.007887429	-1.521650801	0.704458649	-0.245677487	2.22610945	1.275973314	-0.950136136	9.961963646	0.159184285	0.49728492	0.810919313	0.050270196	0.230911514	0.364524196	-5.863325263	-5.927043339	-7.014258984	-4.953046878	-5.496724295	-6.599428899	1.726411739	0.224720787

LRRN4CL	-0.250885261	-0.122005992	-0.241867107	0.128879269	0.009018154	-0.119861115	8	0.107570991	0.816496581	0.816496581	0.816496581	0.816496581	0.816496581	0.816496581	8	108	0	P_108865	P_108865	P_108865	chr11	62688394	62688454	-	LRRN4CL	LRRN4 C-terminal like [Source:HGNC Symbol;Acc:HGNC:33724]	ENSG00000177363	protein_coding	exon	coding exon	1	1298	11:62688394:62688454	ENSG00000177363	LRRN4CL	5.452912107	0.011499244	-2.865405787	-1.393452425	-2.762407822	1.471953363	0.102997965	-1.368955398	9.961963646	0.016861743	0.193789598	0.020113723	0.171906902	0.92000848	0.201086052	-3.740357436	-5.250588897	-3.961033408	-6.078192491	-6.291045797	-6.133892492	3.639075188	0.052533025

SPATA6L	-0.21026288	-0.002338264	-0.105258856	0.207924616	0.105004024	-0.102920593	8	0.13137181	0.816496581	0.816496581	0.816496581	0.816496581	0.816496581	0.816496581	8	109	0	P_148336	P_148336	P_148336	chr9	4573946	4574006	-	SPATA6L;SLC1A1	"spermatogenesis associated 6-like [Source:HGNC Symbol;Acc:HGNC:25472];solute carrier family 1 (neuronal/epithelial high affinity glutamate transporter, system Xag), member 1 [Source:HGNC Symbol;Acc:HGNC:10939]"	ENSG00000106686	protein_coding	exon;intron	multiple non-Ref	0	1299	9:4573946:4574006	ENSG00000106686	SPATA6L	4.52985258	0.016066243	-2.031660215	-0.022593417	-1.017061264	2.009066798	1.014598951	-0.994467847	9.961963646	0.069718537	0.98242074	0.333188673	0.072395385	0.334304983	0.343536163	-5.116843084	-6.18181833	-6.509864724	-5.298589829	-5.776514228	-6.556445832	1.869549006	0.198849254

IVNS1ABP	-0.307819175	0.002750238	-0.432617064	0.310569413	-0.124797889	-0.435367302	8	0.072349503	0.816496581	0.816496581	0.816496581	0.816496581	0.816496581	0.816496581	8	110	0	P_205354	P_205354	P_205354	chr1	185307031	185307091	-	IVNS1ABP	influenza virus NS1A binding protein [Source:HGNC Symbol;Acc:HGNC:16951]	ENSG00000116679	protein_coding	exon	coding exon	1	1300	1:185307031:185307091	ENSG00000116679	IVNS1ABP	7.895607157	0.006410233	-4.708736457	0.042070626	-6.617780526	4.750807084	-1.909044069	-6.659851153	9.961963646	0.00083915	0.967273666	6.05E-05	0.000787542	0.085451389	5.74E-05	-0.678181947	-6.18115445	2.016305236	-0.737645436	-4.654202224	2.054803956	22.75620377	8.90E-05

STAG1	-0.233423096	-0.121710282	-0.348444479	0.111712813	-0.115021384	-0.226734197	8	0.055504818	0.816496581	0.816496581	0.816496581	0.816496581	0.816496581	0.816496581	8	111	0	P_246351	P_246351	P_246351	chr3	136542132	136542192	-	STAG1	stromal antigen 1 [Source:HGNC Symbol;Acc:HGNC:11354]	ENSG00000118007	protein_coding	3'UTR;5'UTR;exon	multiple Ref	1	1301	3:136542132:136542192	ENSG00000118007	STAG1	6.561010735	0.004680722	-4.178621365	-2.178795479	-6.237675588	1.999825887	-2.059054223	-4.058880109	9.961963646	0.001907571	0.054456415	9.82E-05	0.073517981	0.0665986	0.00230864	-1.526300293	-4.154387569	1.513320874	-5.312991341	-4.430270587	-1.785775016	14.3050231	0.000607908

SPPL2B	-0.091927328	-0.171687412	-0.124558961	-0.079760084	-0.032631633	0.047128451	8	0.145527837	0.816496581	0.816496581	0.816496581	0.816496581	0.816496581	0.816496581	8	112	0	P_124077	P_124077	P_124077	chr19	2355042	2355102	+	SPPL2B	signal peptide peptidase like 2B [Source:HGNC Symbol;Acc:HGNC:30627]	ENSG00000005206	protein_coding	3'ext	3'extended sense	0	1302	19:2355042:2355102	ENSG00000005206	SPPL2B	9.461764447	0.019214055	-0.812233223	-1.516961533	-1.100553324	-0.70472831	-0.288320101	0.416408209	9.961963646	0.435637174	0.160348649	0.29697287	0.497124183	0.779011489	0.685940809	-6.649867124	-5.095727607	-6.423161211	-6.884608916	-6.252659889	-6.977472509	0.820879832	0.511625483

HERPUD2	0.272393107	-0.087411889	0.033734711	-0.359804996	-0.238658396	0.1211466	8	0.140608486	0.816496581	0.816496581	0.816496581	0.816496581	0.816496581	0.816496581	8	113	0	P_313809	P_313809	P_313809	chr7	35634834	35634894	+	HERPUD2	HERPUD family member 2 [Source:HGNC Symbol;Acc:HGNC:21915]	ENSG00000122557	protein_coding	intron	intron antisense	0	1303	7:35634834:35634894	ENSG00000122557	HERPUD2	6.358655328	0.018083671	2.480838205	-0.796109548	0.30724111	-3.276947753	-2.173597095	1.103350658	9.961963646	0.032579491	0.44452524	0.764986482	0.008373264	0.054936316	0.29581404	-4.389238026	-5.858595085	-6.995813892	-3.176534807	-4.25502583	-6.444185793	3.927173216	0.043489691

ADAM17	-0.108351984	-0.0068842	-0.184806021	0.101467784	-0.076454038	-0.177921822	8	0.042988432	0.816496581	0.816496581	0.816496581	0.816496581	0.816496581	0.816496581	8	114	0	P_233560	P_233560	P_233560	chr2	9492899	9492959	-	ADAM17	ADAM metallopeptidase domain 17 [Source:HGNC Symbol;Acc:HGNC:195]	ENSG00000151694	protein_coding	exon	coding exon	1	1304	2:9492899:9492959	ENSG00000151694	ADAM17	6.739919406	0.003690733	-2.184369898	-0.138785076	-3.725679002	2.045584823	-1.541309103	-3.586893926	9.961963646	0.05394628	0.892385443	0.003964168	0.06811572	0.154383145	0.004984658	-4.874827639	-6.171943628	-2.320210456	-5.241409946	-5.167741454	-2.57631651	6.349546038	0.011121391

EP400P1	-0.050958307	-0.012681198	0.00537741	0.038277109	0.056335717	0.018058608	8	0.075209993	0.816496581	0.816496581	0.816496581	0.816496581	0.816496581	0.816496581	8	115	0	P_218111	P_218111	P_218111	chr12	132088449	132088509	+	EP400NL	EP400 N-terminal like [Source:HGNC Symbol;Acc:HGNC:26602]	ENSG00000185684	protein_coding	exon	non-coding exon	1	1305	12:132088449:132088509	ENSG00000185684	EP400P1	6.437193174	0.006749197	-0.75968681	-0.189051389	0.080166464	0.570635421	0.839853274	0.269217853	9.961963646	0.465039471	0.853848582	0.937692471	0.580889168	0.420689306	0.793255767	-6.692284766	-6.163281212	-7.043612077	-6.974405392	-5.934624946	-7.031927759	0.287124616	0.833700956

RASAL2	-0.033802497	-0.041536888	-0.076429132	-0.007734391	-0.042626635	-0.034892244	8	0.049153934	0.816496581	0.816496581	0.816496581	0.816496581	0.816496581	0.816496581	8	116	0	P_205072	P_205072	P_205072	chr1	178451655	178451715	+	RASAL2	RAS protein activator like 2 [Source:HGNC Symbol;Acc:HGNC:9874]	ENSG00000075391	protein_coding	exon	coding exon	1	1306	1:178451655:178451715	ENSG00000075391	RASAL2	7.151281035	0.004146951	-0.642880039	-0.789978205	-1.453583835	-0.147098166	-0.810703796	-0.66360563	9.961963646	0.534818056	0.447936312	0.176827551	0.88598893	0.436475146	0.522002963	-6.777418658	-5.863404604	-6.001457034	-7.137792946	-5.958523103	-6.836003737	0.711586203	0.567054068

EWSR1	-0.160874496	-0.214105558	-0.191338167	-0.053231063	-0.030463672	0.022767391	8	0.098350921	0.816496581	0.816496581	0.816496581	0.816496581	0.816496581	0.816496581	8	117	0	P_132957	P_132957	P_132957	chr22	29292052	29292112	+	EWSR1	EWS RNA-binding protein 1 [Source:HGNC Symbol;Acc:HGNC:3508]	ENSG00000182944	protein_coding	exon	non-coding exon	1	1307	22:29292052:29292112	ENSG00000182944	EWSR1	7.655240069	0.009974553	-1.972813838	-2.625589635	-2.346391716	-0.652775796	-0.373577878	0.279197918	9.961963646	0.076894272	0.025428151	0.040982676	0.528676454	0.716545701	0.785803436	-5.20823116	-3.455998047	-4.658084539	-6.921459736	-6.222987215	-7.028960281	2.822482934	0.093358715

SPG7	-0.142379234	-0.089352592	-0.084589625	0.053026642	0.057789609	0.004762967	8	0.050451975	0.816496581	0.816496581	0.816496581	0.816496581	0.816496581	0.816496581	8	118	0	P_227242	P_227242	P_227242	chr16	89510532	89510592	+	SPG7	spastic paraplegia 7 (pure and complicated autosomal recessive) [Source:HGNC Symbol;Acc:HGNC:11237]	ENSG00000197912	protein_coding	5'UTR;exon	multiple Ref	1	1308	16:89510532:89510592	ENSG00000197912	SPG7	6.920469006	0.00425078	-2.67459451	-1.67848882	-1.58901646	0.99610569	1.085578051	0.08947236	9.961963646	0.023379957	0.124294524	0.143257046	0.342778151	0.303236264	0.930479778	-4.064109421	-4.881512341	-5.819212245	-6.63250319	-5.705397469	-7.067006969	2.445744229	0.124413286

CCDC157	-0.008763395	0.098663498	-0.078739817	0.107426893	-0.069976422	-0.177403315	8	0.141366701	0.816496581	0.816496581	0.816496581	0.816496581	0.816496581	0.816496581	8	120	0	P_133021	P_133021	P_133021	chr22	30375531	30375591	+	CCDC157	coiled-coil domain containing 157 [Source:HGNC Symbol;Acc:HGNC:33854]	ENSG00000187860	protein_coding	exon	coding exon	1	1310	22:30375531:30375591	ENSG00000187860	CCDC157	5.706174677	0.018255363	-0.079436993	0.894348757	-0.713747825	0.97378575	-0.634310832	-1.608096582	9.961963646	0.938258119	0.392230451	0.49176641	0.353214599	0.540169836	0.139009719	-6.99491797	-5.777168165	-6.775843372	-6.65440091	-6.087029172	-5.816509259	0.875364904	0.486064909

LTBP3	0.203860905	-0.07815418	0.054384755	-0.282015085	-0.14947615	0.132538935	8	0.09078607	0.816496581	0.816496581	0.816496581	0.816496581	0.816496581	0.816496581	8	121	0	P_212117	P_212117	P_212117	chr11	65539079	65539139	-	LTBP3	latent transforming growth factor beta binding protein 3 [Source:HGNC Symbol;Acc:HGNC:6716]	ENSG00000168056	protein_coding	3'UTR;exon	multiple Ref	1	1311	11:65539079:65539139	ENSG00000168056	LTBP3	9.267608094	0.008825548	2.657717	-1.018889291	0.709009348	-3.676606291	-1.948707652	1.72789864	9.961963646	0.024066068	0.332361722	0.494576684	0.004297556	0.080029973	0.114819033	-4.092602992	-5.662602448	-6.779345777	-2.495251761	-4.595675701	-5.64620986	4.817469723	0.025241365

TRA2B	-0.243888302	-0.063055257	-0.134925673	0.180833045	0.108962629	-0.071870416	8	0.068490994	0.816496581	0.816496581	0.816496581	0.816496581	0.816496581	0.816496581	8	122	0	A_24_P145911	A_24_P145911	A_24_P145911	chr3	185931772	185931832	-	TRA2B	transformer 2 beta homolog (Drosophila) [Source:HGNC Symbol;Acc:HGNC:10781]	ENSG00000136527	protein_coding	exon	coding exon	1	1312	3:185931772:185931832	ENSG00000136527	TRA2B	9.506295173	0.005973826	-3.864651369	-0.99917291	-2.138030741	2.865478459	1.726620628	-1.138857831	9.961963646	0.003158641	0.341361934	0.058330365	0.016859646	0.115055637	0.281407797	-2.044601335	-5.681551852	-4.995809692	-3.881900333	-4.916090851	-6.405600156	5.499039152	0.017245833

RPL34	-0.084406062	-0.125450198	-0.340243864	-0.041044137	-0.255837802	-0.214793666	8	0.218456409	0.816496581	0.816496581	0.816496581	0.816496581	0.816496581	0.816496581	8	123	0	P_138397	P_138397	P_138397	chr4	108630271	108630331	+	RPL34	ribosomal protein L34 [Source:HGNC Symbol;Acc:HGNC:10340]	ENSG00000109475	protein_coding	3'UTR	3'UTR	1	1313	4:108630271:108630331	ENSG00000109475	RPL34	6.080946078	0.040531018	-0.51348234	-0.763173404	-2.069865743	-0.249691064	-1.556383403	-1.306692339	9.961963646	0.618808972	0.46304968	0.065404126	0.807899522	0.150788069	0.220676945	-6.856367249	-5.884046371	-5.103899441	-7.115625213	-5.147884035	-6.210939635	1.55376029	0.261251472

CD79A	0.120123292	0.072165696	-0.028734685	-0.047957597	-0.148857977	-0.100900381	8	0.136625786	0.816496581	0.816496581	0.816496581	0.816496581	0.816496581	0.816496581	8	124	0	P_125911	P_125911	P_125911	chr19	41879181	41879241	+	CD79A	"CD79a molecule, immunoglobulin-associated alpha [Source:HGNC Symbol;Acc:HGNC:1698]"	ENSG00000105369	protein_coding	exon	coding exon	1	1314	19:41879181:41879241	ENSG00000105369	CD79A	6.166410798	0.017196986	1.121880734	0.673985052	-0.268365017	-0.447895682	-1.390245751	-0.94235007	9.961963646	0.288225978	0.515654267	0.793893625	0.663811326	0.19473177	0.368304326	-6.351917564	-5.948137363	-7.007932401	-7.041002469	-5.360047484	-6.606810151	0.801051576	0.521264225

RALGAPA2	0.067352396	0.034428681	-0.024603747	-0.032923716	-0.091956143	-0.059032427	8	0.05794067	0.816496581	0.816496581	0.816496581	0.816496581	0.816496581	0.816496581	8	126	0	P_130777	P_130777	P_130777	chr20	20393059	20393119	-	RALGAPA2	"Ral GTPase activating protein, alpha subunit 2 (catalytic) [Source:HGNC Symbol;Acc:HGNC:16207]"	ENSG00000188559	protein_coding	"3'UTR;3'UTR,exon"	multiple Ref	1	1316	20:20393059:20393119	ENSG00000188559	RALGAPA2	5.837326126	0.004902635	1.178104688	0.602214507	-0.430360176	-0.575890181	-1.608464864	-1.032574683	9.961963646	0.266130936	0.560487638	0.676095242	0.577466578	0.13892885	0.326219486	-6.289693	-5.994441945	-6.946911216	-6.97121653	-5.078407651	-6.518220604	0.986803674	0.437840396

CDC14A	-0.091796757	0.024572051	0.041953508	0.116368808	0.133750265	0.017381457	8	0.089520532	0.816496581	0.816496581	0.816496581	0.816496581	0.816496581	0.816496581	8	127	0	P_203148	P_203148	P_203148	chr1	100455429	100455489	+	CDC14A	cell division cycle 14A [Source:HGNC Symbol;Acc:HGNC:1718]	ENSG00000079335	protein_coding	exon	coding exon	1	1317	1:100455429:100455489	ENSG00000079335	CDC14A	5.918322217	0.008642303	-1.209367288	0.323722055	0.552712339	1.533089343	1.762079627	0.228990284	9.961963646	0.254439936	0.752842169	0.592645058	0.156375001	0.108652869	0.823509046	-6.254102636	-6.127142586	-6.882814359	-5.996402585	-4.866196114	-7.042807915	1.230096705	0.349525691

DIS3L2	-0.229842462	-0.026759291	-0.224295158	0.203083171	0.005547304	-0.197535867	8	0.047253197	0.816496581	0.816496581	0.816496581	0.816496581	0.816496581	0.816496581	8	128	0	P_239684	P_239684	P_239684	chr2	232015611	232015671	+	DIS3L2	DIS3 like 3'-5' exoribonuclease 2 [Source:HGNC Symbol;Acc:HGNC:28648]	ENSG00000144535	protein_coding	exon	coding exon	1	1318	2:232015611:232015671	ENSG00000144535	DIS3L2	6.397955305	0.003999796	-4.450993636	-0.518204661	-4.343567826	3.932788974	0.10742581	-3.825363164	9.961963646	0.001244823	0.61562868	0.001471263	0.002828158	0.916583896	0.003367347	-1.085980765	-6.042475586	-1.299526838	-2.064519417	-6.290550794	-2.174348608	11.50970577	0.001411242

FAM13B	0.125244044	-0.354260476	-0.194036077	-0.47950452	-0.319280121	0.160224399	8	0.091799109	0.816496581	0.816496581	0.816496581	0.816496581	0.816496581	0.816496581	8	129	0	P_254958	P_254958	P_254958	chr5	137962405	137962465	-	FAM13B	"family with sequence similarity 13, member B [Source:HGNC Symbol;Acc:HGNC:1335]"	ENSG00000031003	protein_coding	exon	coding exon	1	1319	5:137962405:137962465	ENSG00000031003	FAM13B	7.384362698	0.008974086	1.619226494	-4.580081644	-2.50860916	-6.199308138	-4.127835655	2.071472484	9.961963646	0.136584165	0.001020512	0.031068075	0.000103221	0.002067901	0.065228358	-5.729562377	-0.433560809	-4.38907955	1.387272294	-1.13493796	-5.124798348	14.94226818	0.000510997

SPART	0.054681588	0.092119312	0.144521303	0.037437724	0.089839715	0.052401991	8	0.12913394	0.816496581	0.816496581	0.816496581	0.816496581	0.816496581	0.816496581	8	130	0	P_218779	P_218779	P_218779	chr13	36331404	36331464	-	SPG20	spastic paraplegia 20 (Troyer syndrome) [Source:HGNC Symbol;Acc:HGNC:18514]	ENSG00000133104	protein_coding	exon	coding exon	1	1320	13:36331404:36331464	ENSG00000133104	SPART	8.988145683	0.01559808	0.53623009	0.903359778	1.417235207	0.367129689	0.881005117	0.513875429	9.961963646	0.603566276	0.387657359	0.18692193	0.721200982	0.399071577	0.618543927	-6.843690535	-5.769295775	-6.048604052	-7.07640126	-5.899651964	-6.929009436	0.714529902	0.565486635

NPLOC4	-0.091590328	-0.028192018	-0.12552265	0.06339831	-0.033932322	-0.097330632	8	0.074734044	0.816496581	0.816496581	0.816496581	0.816496581	0.816496581	0.816496581	8	131	0	P_122910	P_122910	P_122910	chr17	81567422	81567482	-	NPLOC4	nuclear protein localization 4 homolog (S. cerevisiae) [Source:HGNC Symbol;Acc:HGNC:18261]	ENSG00000182446	protein_coding	3'UTR;exon	multiple Ref	1	1321	17:81567422:81567482	ENSG00000182446	NPLOC4	8.287590945	0.006691886	-1.371263707	-0.422082675	-1.879288542	0.949181032	-0.508024834	-1.457205866	9.961963646	0.200388766	0.681928896	0.089741418	0.364986386	0.622494689	0.175847822	-6.059118261	-6.089034404	-5.398321138	-6.678064619	-6.161217253	-6.020564642	1.478787694	0.279199368

MYO1H	0.040315263	0.163645614	0.075987233	0.12333035	0.03567197	-0.08765838	8	0.096340926	0.816496581	0.816496581	0.816496581	0.816496581	0.816496581	0.816496581	8	132	0	P_217362	P_217362	P_217362	chr12	109397752	109397812	+	MYO1H	myosin IH [Source:HGNC Symbol;Acc:HGNC:13879]	ENSG00000174527	protein_coding	exon	coding exon	1	1322	12:109397752:109397812	ENSG00000174527	MYO1H	3.814412141	0.009660294	0.502365677	2.039176537	0.946871593	1.53681086	0.444505916	-1.092304944	9.961963646	0.626328183	0.06884897	0.366105707	0.15547041	0.666177927	0.300410284	-6.862372814	-4.365036055	-6.578445104	-5.99135703	-6.192655001	-6.455995005	1.509946876	0.27157467

ULK1	0.223014759	-0.040112837	0.223339376	-0.263127596	0.000324617	0.263452212	8	0.113651526	0.816496581	0.816496581	0.816496581	0.816496581	0.816496581	0.816496581	8	133	0	A_24_P73370	A_24_P73370	A_24_P73370	chr12	131922796	131922856	+	ULK1	unc-51 like autophagy activating kinase 1 [Source:HGNC Symbol;Acc:HGNC:12558]	ENSG00000177169	protein_coding	3'UTR	3'UTR	1	1323	12:131922796:131922856	ENSG00000177169	ULK1	9.061662841	0.012579474	2.435274922	-0.43802386	2.438819669	-2.873298783	0.003544747	2.876843529	9.961963646	0.035217823	0.670714149	0.035005231	0.01663558	0.997241687	0.01653501	-4.464946656	-6.081943589	-4.505354238	-3.868529873	-6.296678574	-3.788868652	4.767059563	0.025992555

BECN1	-0.08209083	0.00075471	-0.042308911	0.08284554	0.039781919	-0.043063621	8	0.039240114	0.816496581	0.816496581	0.816496581	0.816496581	0.816496581	0.816496581	8	134	0	P_228834	P_228834	P_228834	chr17	42813947	42814007	-	BECN1	"beclin 1, autophagy related [Source:HGNC Symbol;Acc:HGNC:1034]"	ENSG00000126581	protein_coding	exon	coding exon	1	1324	17:42813947:42814007	ENSG00000126581	BECN1	8.845202493	0.003443217	-1.713397427	0.015752283	-0.883070365	1.72914971	0.830327062	-0.898822648	9.961963646	0.117529806	0.987743031	0.398007344	0.114587845	0.425805168	0.38995524	-5.595954275	-6.181956616	-6.637220427	-5.720950386	-5.942515476	-6.647133347	1.367179341	0.308573652

PRPS1	-0.407006095	0.25593569	-0.226103596	0.662941785	0.180902499	-0.482039286	8	0.253373877	0.816496581	0.816496581	0.816496581	0.816496581	0.816496581	0.816496581	8	135	0	P_151435	P_151435	P_151435	chrX	107650190	107650250	+	PRPS1	phosphoribosyl pyrophosphate synthetase 1 [Source:HGNC Symbol;Acc:HGNC:9462]	ENSG00000147224	protein_coding	3'UTR	3'UTR	1	1325	X:107650190:107650250	ENSG00000147224	PRPS1	10.84165857	0.053761437	-2.149864602	1.351889044	-1.19431164	3.501753645	0.955552962	-2.546200683	9.961963646	0.057179338	0.206305047	0.260017742	0.005742636	0.361910912	0.029132105	-4.930077286	-5.300775673	-6.319494146	-2.792234742	-5.832710089	-4.349162446	4.589066677	0.028867315

MYO6	0.097677312	0.006916145	0.136923633	-0.090761168	0.03924632	0.130007488	8	0.073239273	0.816496581	0.816496581	0.816496581	0.816496581	0.816496581	0.816496581	8	136	0	P_143073	P_143073	P_143073	chr6	75915071	75915131	+	MYO6	myosin VI [Source:HGNC Symbol;Acc:HGNC:7605]	ENSG00000196586	protein_coding	3'UTR	3'UTR	1	1326	6:75915071:75915131	ENSG00000196586	MYO6	10.64068329	0.006514261	1.48219977	0.104948712	2.07774121	-1.377251058	0.59554144	1.972792498	9.961963646	0.16921393	0.918499517	0.064546885	0.198589628	0.56476516	0.076896996	-5.915954191	-6.176284375	-5.09147974	-6.200609279	-6.111430738	-5.278910422	2.111389876	0.162584371

SNX27	-0.011709333	-0.092539336	-0.036819759	-0.080830004	-0.025110427	0.055719577	8	0.047797164	0.816496581	0.816496581	0.816496581	0.816496581	0.816496581	0.816496581	8	137	0	P_204028	P_204028	P_204028	chr1	151668575	151668635	+	SNX27	sorting nexin family member 27 [Source:HGNC Symbol;Acc:HGNC:20073]	ENSG00000143376	protein_coding	3'UTR;exon	multiple Ref	1	1327	1:151668575:151668635	ENSG00000143376	SNX27	8.41018073	0.004041317	-0.225588156	-1.782832452	-0.709357389	-1.557244296	-0.483769233	1.073475063	9.961963646	0.826082389	0.105058111	0.494369933	0.150584996	0.639007355	0.3083721	-6.9706613	-4.736968388	-6.779089242	-5.963520749	-6.173693942	-6.475910982	1.257326775	0.34092991

BOK	0.103481361	0.143435444	0.102840657	0.039954084	-0.000640703	-0.040594787	8	0.063967271	0.816496581	0.816496581	0.816496581	0.816496581	0.816496581	0.816496581	8	138	0	P_130395	P_130395	P_130395	chr2	241573722	241573782	-	BOK	BCL2-related ovarian killer [Source:HGNC Symbol;Acc:HGNC:1087]	ENSG00000176720	protein_coding	3'UTR	3'UTR antisense	0	1328	2:241573722:241573782	ENSG00000176720	BOK	7.842128195	0.005492632	1.710084261	2.370346646	1.699496298	0.660262385	-0.010587963	-0.670850348	9.961963646	0.118157235	0.039343814	0.120182702	0.524057761	0.991761221	0.517566752	-5.600725607	-3.858570416	-5.663495552	-6.91630824	-6.29662563	-6.83094974	2.052886296	0.170595817

DYNC2H1	-0.163861514	0.039708415	-0.10033069	0.203569929	0.063530824	-0.140039106	8	0.087500803	0.816496581	0.816496581	0.816496581	0.816496581	0.816496581	0.816496581	8	139	0	P_213068	P_213068	P_213068	chr11	103286326	103286386	+	DYNC2H1	"dynein, cytoplasmic 2, heavy chain 1 [Source:HGNC Symbol;Acc:HGNC:2962]"	ENSG00000187240	protein_coding	exon	coding exon	1	1329	11:103286326:103286386	ENSG00000187240	DYNC2H1	7.144175856	0.008355183	-2.195556452	0.532047246	-1.34431624	2.727603699	0.851240213	-1.876363486	9.961963646	0.052936403	0.60635443	0.208657019	0.02134967	0.414629143	0.090173781	-4.856850338	-6.035026557	-6.140769535	-4.117006046	-5.925091367	-5.426319695	3.099316722	0.076292019

MROH8	0.033401001	-0.002851527	0.054447572	-0.036252528	0.021046572	0.057299099	8	0.106920702	0.816496581	0.816496581	0.816496581	0.816496581	0.816496581	0.816496581	8	140	0	P_240751	P_240751	P_240751	chr20	37112323	37112383	-	MROH8	maestro heat-like repeat family member 8 [Source:HGNC Symbol;Acc:HGNC:16125]	ENSG00000101353	protein_coding	exon	coding exon	1	1330	20:37112323:37112383	ENSG00000101353	MROH8	4.572417654	0.011387233	0.383350478	-0.032727592	0.624906515	-0.41607807	0.241556037	0.657634107	9.961963646	0.70951358	0.97453826	0.546078677	0.686174538	0.814023658	0.525676493	-6.918757242	-6.181522805	-6.837964092	-7.05575714	-6.265743582	-6.840131788	0.200414956	0.893727455

ATP6V1G1	0.040121725	-0.020796689	0.015450471	-0.060918414	-0.024671254	0.03624716	8	0.042957665	0.816496581	0.816496581	0.816496581	0.816496581	0.816496581	0.816496581	8	141	0	P_268668	P_268668	P_268668	chr9	114597662	114597722	-	ATP6V1G1	"ATPase, H+ transporting, lysosomal 13kDa, V1 subunit G1 [Source:HGNC Symbol;Acc:HGNC:864]"	ENSG00000136888	protein_coding	exon	exon antisense	0	1331	9:114597662:114597722	ENSG00000136888	ATP6V1G1	8.618005646	0.003688609	0.809084507	-0.419380749	0.311570263	-1.228465256	-0.497514244	0.730951012	9.961963646	0.437363522	0.683837902	0.761789822	0.247503415	0.629623855	0.481647284	-6.652479111	-6.090211166	-6.994364931	-6.381503393	-6.166694913	-6.787105997	0.536418445	0.667868479

VAV2	-0.087460644	0.00537134	-0.027530182	0.092831984	0.059930462	-0.032901522	8	0.080805608	0.816496581	0.816496581	0.816496581	0.816496581	0.816496581	0.816496581	8	142	0	P_269311	P_269311	P_269311	chr9	133939112	133939172	-	VAV2	vav 2 guanine nucleotide exchange factor [Source:HGNC Symbol;Acc:HGNC:12658]	ENSG00000160293	protein_coding	exon	coding exon	1	1332	9:133939112:133939172	ENSG00000160293	VAV2	6.771519715	0.007450266	-1.241001819	0.07621534	-0.390632908	1.317217159	0.850368911	-0.466848248	9.961963646	0.243033757	0.940756686	0.704291829	0.217257925	0.415090735	0.650651349	-6.217387668	-6.179026131	-6.964423898	-6.2753499	-5.925824758	-6.953605996	0.729103996	0.557787816

LCN15	0.832126839	0.093760518	0.461301913	-0.738366321	-0.370824926	0.367541395	8	0.247810282	0.816496581	0.816496581	0.816496581	0.816496581	0.816496581	0.816496581	8	143	0	P_150191	P_150191	P_150191	chr9	136763384	136763444	-	LCN15	lipocalin 15 [Source:HGNC Symbol;Acc:HGNC:33777]	ENSG00000177984	protein_coding	exon	coding exon	1	1333	9:136763384:136763444	ENSG00000177984	LCN15	6.471024675	0.051522211	4.489912874	0.505904312	2.489050108	-3.984008562	-2.000862766	1.983145796	9.961963646	0.001172153	0.623929745	0.032125157	0.002603698	0.073391206	0.075585973	-1.023833812	-6.04893887	-4.421741739	-1.979142914	-4.517950915	-5.262889361	8.403211752	0.004409546

HRCT1	0.018383998	0.434964594	0.010085285	0.416580596	-0.008298713	-0.424879309	8	0.488561875	0.816496581	0.816496581	0.816496581	0.816496581	0.816496581	0.816496581	8	145	0	P_148755	P_148755	P_148755	chr9	35905326	35905386	+	HRCT1;LINC00961	histidine rich carboxyl terminus 1 [Source:HGNC Symbol;Acc:HGNC:33872];long intergenic non-protein coding RNA 961 [Source:HGNC Symbol;Acc:HGNC:27244]	ENSG00000196196	protein_coding;lincRNA	promoter	promoter sense	0	1335	9:35905326:35905386	ENSG00000196196	HRCT1	4.362623019	0.193889941	0.051133787	1.209823168	0.028051504	1.158689381	-0.023082282	-1.181771664	9.961963646	0.96022968	0.254272552	0.978175072	0.273604178	0.982040442	0.264737842	-6.996933705	-5.464314454	-7.046690893	-6.461120135	-6.29640188	-6.357720013	0.701125298	0.572658031

DENND3	0.111151406	-0.069272494	-0.075836198	-0.1804239	-0.186987604	-0.006563704	8	0.092638809	0.816496581	0.816496581	0.816496581	0.816496581	0.816496581	0.816496581	8	147	0	P_266462	P_266462	P_266462	chr8	141144179	141144239	+	DENND3	DENN/MADD domain containing 3 [Source:HGNC Symbol;Acc:HGNC:29134]	ENSG00000105339	protein_coding	exon	coding exon	1	1337	8:141144179:141144239	ENSG00000105339	DENND3	4.86352918	0.009098457	1.427173321	-0.889452127	-0.973729454	-2.316625448	-2.400902775	-0.084277327	9.961963646	0.184114208	0.394731297	0.353241213	0.043112192	0.037346167	0.934505507	-5.987881055	-5.781418103	-6.552679804	-4.8053752	-3.898796729	-7.067499705	2.485714682	0.120594831

ZRANB3	0.041921115	-0.04078466	0.096225812	-0.082705775	0.054304697	0.137010472	8	0.089676225	0.816496581	0.816496581	0.816496581	0.816496581	0.816496581	0.816496581	8	148	0	P_236577	P_236577	P_236577	chr2	135349994	135350054	-	ZRANB3	"zinc finger, RAN-binding domain containing 3 [Source:HGNC Symbol;Acc:HGNC:25249]"	ENSG00000121988	protein_coding	5'UTR;exon	multiple Ref	1	1338	2:135349994:135350054	ENSG00000121988	ZRANB3	7.319576418	0.008664708	0.551571075	-0.53661833	1.266077366	-1.088189405	0.714506291	1.802695696	9.961963646	0.593397881	0.603307825	0.234290463	0.302136812	0.491317504	0.101719811	-6.834848876	-6.032526093	-6.235885552	-6.537939498	-6.032099315	-5.53656931	1.189921496	0.362650863

CYB5D2	0.042394515	-0.061003147	0.002407335	-0.103397662	-0.039987179	0.063410482	8	0.081617366	0.816496581	0.816496581	0.816496581	0.816496581	0.816496581	0.816496581	8	149	0	P_120128	P_120128	P_120128	chr17	4157188	4157248	-	CYB5D2	cytochrome b5 domain containing 2 [Source:HGNC Symbol;Acc:HGNC:28471]	ENSG00000167740	protein_coding	3'ext;3'UTR;exon;intron	multiple non-Ref	0	1339	17:4157188:4157248	ENSG00000167740	CYB5D2	8.213508825	0.007556147	0.597317311	-0.859503547	0.033918139	-1.456820858	-0.563399171	0.893421686	9.961963646	0.563625032	0.410268907	0.97361241	0.175951741	0.585620278	0.392703078	-6.807097191	-5.80697957	-7.046492372	-6.098110328	-6.130576026	-6.652024212	0.722438784	0.561296063

PELI3	-0.036794741	0.055637207	0.010821018	0.092431948	0.047615759	-0.044816189	8	0.085284668	0.816496581	0.816496581	0.816496581	0.816496581	0.816496581	0.816496581	8	150	0	P_109229	P_109229	P_109229	chr11	66476554	66476614	+	PELI3	pellino E3 ubiquitin protein ligase family member 3 [Source:HGNC Symbol;Acc:HGNC:30010]	ENSG00000174516	protein_coding	3'UTR	3'UTR	1	1340	11:66476554:66476614	ENSG00000174516	PELI3	8.159916809	0.008047681	-0.502337979	0.75958361	0.147733292	1.26192159	0.650071271	-0.611850318	9.961963646	0.626346974	0.465098451	0.885500579	0.235721464	0.530350833	0.554343123	-6.862387622	-5.88676301	-7.035214812	-6.342113392	-6.07670478	-6.870639489	0.540089233	0.665605486

FANCA	-0.531203124	-0.027091789	-0.33550973	0.504111336	0.195693395	-0.308417941	8	0.053819217	0.816496581	0.816496581	0.816496581	0.816496581	0.816496581	0.816496581	8	151	0	P_119882	P_119882	P_119882	chr16	89737991	89738051	-	FANCA	"Fanconi anemia, complementation group A [Source:HGNC Symbol;Acc:HGNC:3582]"	ENSG00000187741	protein_coding	3'UTR	3'UTR	1	1341	16:89737991:89738051	ENSG00000187741	FANCA	7.222784168	0.004532738	-9.663310756	-0.49283666	-6.103380477	9.170474096	3.559930279	-5.610543817	9.961963646	2.24E-06	0.632809528	0.000117034	3.59E-06	0.005212777	0.000227546	5.418345273	-6.05564402	1.33079648	4.883360496	-2.025497484	0.620608397	43.18710278	5.11E-06

BACH2	-0.014876357	0.019061864	-0.099762492	0.033938221	-0.084886135	-0.118824356	8	0.109833577	0.816496581	0.816496581	0.816496581	0.816496581	0.816496581	0.816496581	8	152	0	P_258317	P_258317	P_258317	chr6	89941410	89941470	+	BACH2	"BTB and CNC homology 1, basic leucine zipper transcription factor 2 [Source:HGNC Symbol;Acc:HGNC:14078]"	ENSG00000112182	protein_coding	intron	intron antisense	0	1342	6:89941410:89941470	ENSG00000112182	BACH2	3.91527069	0.011894264	-0.167060368	0.214063304	-1.120325279	0.381123672	-0.953264912	-1.334388584	9.961963646	0.870664946	0.834815591	0.288857055	0.711113448	0.363013096	0.211774327	-6.983151971	-6.15798849	-6.401841942	-7.070754844	-5.834832019	-6.176974121	0.693903856	0.576557458

PCGF3	0.007569631	-0.06314661	-0.138710089	-0.070716241	-0.14627972	-0.075563479	8	0.116245117	0.816496581	0.816496581	0.816496581	0.816496581	0.816496581	0.816496581	8	153	0	A_23_P21346	A_23_P21346	A_23_P21346	chr4	766157	766217	+	PCGF3	polycomb group ring finger 3 [Source:HGNC Symbol;Acc:HGNC:10066]	ENSG00000185619	protein_coding	3'UTR	3'UTR	1	1343	4:766157:766217	ENSG00000185619	PCGF3	7.291677324	0.013058301	0.081129164	-0.676787496	-1.486655482	-0.75791666	-1.567784646	-0.809867986	9.961963646	0.936946028	0.51394809	0.16805432	0.466051793	0.148118117	0.436933539	-6.994769799	-5.946232798	-5.957891789	-6.844248403	-5.132789113	-6.72444751	1.060506716	0.408760626

DIP2A	-0.165391411	0.067824544	-0.1326368	0.233215955	0.032754611	-0.200461344	8	0.077384973	0.816496581	0.816496581	0.816496581	0.816496581	0.816496581	0.816496581	9	18	0	P_132527	P_132527	P_132527	chr21	46546218	46546278	+	DIP2A	DIP2 disco-interacting protein 2 homolog A (Drosophila) [Source:HGNC Symbol;Acc:HGNC:17217]	ENSG00000160305	protein_coding	exon	non-coding exon	1	1378	21:46546218:46546278	ENSG00000160305	DIP2A	6.725884867	0.007015723	-2.418367863	0.9917365	-1.939427042	3.410104363	0.47894082	-2.931163543	9.961963646	0.036249393	0.344803012	0.081268853	0.00669329	0.642319682	0.015068569	-4.492953552	-5.688621191	-5.306758695	-2.948640453	-6.176108276	-3.696086461	5.173889574	0.020609853

PMPCB	0.112006881	0.086657803	0.132760449	-0.025349078	0.020753569	0.046102647	8	0.047594502	0.816496581	0.816496581	0.816496581	0.816496581	0.816496581	0.816496581	9	19	0	P_145569	P_145569	P_145569	chr7	103311037	103311097	-	PMPCB;DNAJC2	"peptidase (mitochondrial processing) beta [Source:HGNC Symbol;Acc:HGNC:9119];DnaJ (Hsp40) homolog, subfamily C, member 2 [Source:HGNC Symbol;Acc:HGNC:13192]"	ENSG00000105819	protein_coding	3'ext;exon;intron	multiple non-Ref	0	1379	7:103311037:103311097	ENSG00000105819	PMPCB	9.200148086	0.004025792	2.162044559	1.672736795	2.562646201	-0.489307764	0.400601642	0.889909406	9.961963646	0.056017413	0.125442271	0.028323207	0.635218115	0.697170017	0.394497281	-4.910610223	-4.889347563	-4.2985618	-7.020239111	-6.212030061	-6.655191319	2.538580521	0.115754168

ANKRD44	0.122494979	-0.058528247	0.058683248	-0.181023226	-0.063811731	0.117211495	8	0.086917104	0.816496581	0.816496581	0.816496581	0.816496581	0.816496581	0.816496581	9	20	0	P_238500	P_238500	P_238500	chr2	197089949	197090009	-	ANKRD44	ankyrin repeat domain 44 [Source:HGNC Symbol;Acc:HGNC:25259]	ENSG00000065413	protein_coding	exon	coding exon	1	1380	2:197089949:197090009	ENSG00000065413	ANKRD44	5.664481654	0.008273426	1.649381884	-0.788076635	0.790163704	-2.437458519	-0.85921818	1.578240339	9.961963646	0.13020096	0.448997705	0.447832861	0.035086715	0.410418957	0.145706342	-5.68724095	-5.864889603	-6.71648652	-4.605631339	-5.918346185	-5.857852426	2.189364423	0.152579662

TTN	-0.530311396	-0.142375343	-0.495147843	0.387936053	0.035163553	-0.3527725	8	0.085232446	0.816496581	0.816496581	0.816496581	0.816496581	0.816496581	0.816496581	9	23	0	P_237814	P_237814	P_237814	chr2	178579576	178579636	-	TTN	titin [Source:HGNC Symbol;Acc:HGNC:12403]	ENSG00000155657	protein_coding	exon	coding exon	1	1383	2:178579576:178579636	ENSG00000155657	TTN	4.16494745	0.00804053	-7.243263114	-1.944634937	-6.762981402	5.298628177	0.480281712	-4.818346465	9.961963646	2.84E-05	0.08057145	5.05E-05	0.000352695	0.641398991	0.000711624	2.829369595	-4.504690758	2.203231812	0.101238854	-6.175440109	-0.566586047	25.58449613	5.36E-05

PRKAG1	-0.374782075	0.16236192	0.111121236	0.537143995	0.485903312	-0.051240683	8	0.330264641	0.816496581	0.816496581	0.816496581	0.816496581	0.816496581	0.816496581	9	24	0	P_111448	P_111448	P_111448	chr12	49002642	49002702	-	PRKAG1	"protein kinase, AMP-activated, gamma 1 non-catalytic subunit [Source:HGNC Symbol;Acc:HGNC:9385]"	ENSG00000181929	protein_coding	3'UTR	3'UTR	1	1384	12:49002642:49002702	ENSG00000181929	PRKAG1	9.711470875	0.089799642	-1.531747351	0.663578803	0.454156351	2.195326155	1.985903702	-0.209422452	9.961963646	0.156702329	0.522019431	0.659450568	0.052957008	0.075240309	0.838339037	-5.84971807	-5.955146602	-6.935639761	-5.002833332	-4.540329831	-7.047471835	1.966662162	0.183257925

TFE3	0.0721163	0.083064989	0.11246971	0.01094869	0.04035341	0.02940472	8	0.096597195	0.816496581	0.816496581	0.816496581	0.816496581	0.816496581	0.816496581	9	26	0	P_150902	P_150902	P_150902	chrX	49028993	49029053	+	TFE3	transcription factor binding to IGHM enhancer 3 [Source:HGNC Symbol;Acc:HGNC:11752]	ENSG00000068323	protein_coding	3'UTR	3'UTR antisense	0	1386	X:49028993:49029053	ENSG00000068323	TFE3	6.089138515	0.0097	0.896795023	1.032946359	1.398605816	0.136151336	0.501810793	0.365659457	9.961963646	0.390985243	0.326053868	0.192283551	0.89441369	0.626704691	0.722264101	-6.576445493	-5.648910873	-6.072461935	-7.139485173	-6.16446887	-6.998823296	0.705229304	0.570453193

PDK4	0.016178359	-0.00604178	0.042545454	-0.02222014	0.026367095	0.048587234	8	0.186975017	0.816496581	0.816496581	0.816496581	0.816496581	0.816496581	0.816496581	9	27	0	P_261912	P_261912	P_261912	chr7	95587760	95587820	+	PDK4	"pyruvate dehydrogenase kinase, isozyme 4 [Source:HGNC Symbol;Acc:HGNC:8812]"	ENSG00000004799	protein_coding	exon;promoter	multiple non-Ref	0	1387	7:95587760:95587820	ENSG00000004799	PDK4	4.118555842	0.030281195	0.113865878	-0.042523015	0.299441707	-0.156388894	0.185575828	0.341964722	9.961963646	0.911606233	0.966921989	0.770757253	0.878850351	0.856501447	0.739481854	-6.991289817	-6.181134279	-6.998374128	-7.136254573	-6.278400578	-7.007868546	0.046712454	0.985816076

AP3D1	-0.143775247	0.074865855	0.050843136	0.218641102	0.194618383	-0.024022719	8	0.060229155	0.816496581	0.816496581	0.816496581	0.816496581	0.816496581	0.816496581	9	29	0	P_231982	P_231982	P_231982	chr19	2116206	2116266	-	AP3D1	"adaptor-related protein complex 3, delta 1 subunit [Source:HGNC Symbol;Acc:HGNC:568]"	ENSG00000065000	protein_coding	exon	coding exon	1	1389	19:2116206:2116266	ENSG00000065000	AP3D1	9.756124459	0.005119805	-2.460951349	1.281453032	0.870264431	3.742404381	3.33121578	-0.411188601	9.961963646	0.033706186	0.229057749	0.4046382	0.003856797	0.007641485	0.689640199	-4.422322817	-5.383453245	-6.648597514	-2.384075281	-2.393334384	-6.979792233	5.621238131	0.01615405

CEP57	-0.131865526	-0.260750185	-0.364485482	-0.128884659	-0.232619956	-0.103735297	8	0.040206189	0.816496581	0.816496581	0.816496581	0.816496581	0.816496581	0.816496581	9	32	0	P_212878	P_212878	P_212878	chr11	95822253	95822313	-	CEP57	centrosomal protein 57kDa [Source:HGNC Symbol;Acc:HGNC:30794]	ENSG00000166037	protein_coding	3'UTR;intron;promoter	multiple non-Ref	0	1392	11:95822253:95822313	ENSG00000166037	CEP57	9.294330096	0.003504852	-2.727985562	-5.394304058	-7.540341787	-2.666318496	-4.812356224	-2.146037728	9.961963646	0.021335705	0.000307881	2.01E-05	0.023713919	0.000718032	0.057549158	-3.973797049	0.674828549	3.157095659	-4.22103134	-0.120224927	-5.006398582	21.37844674	0.000116212

ACOT11	0.08264532	-0.137768082	0.032579768	-0.220413402	-0.050065552	0.17034785	8	0.133340772	0.816496581	0.816496581	0.816496581	0.816496581	0.816496581	0.816496581	9	33	0	P_101941	P_101941	P_101941	chr1	54609187	54609247	+	ACOT11	acyl-CoA thioesterase 11 [Source:HGNC Symbol;Acc:HGNC:18156]	ENSG00000162390	protein_coding	3'UTR;exon	multiple Ref	1	1393	1:54609187:54609247	ENSG00000162390	ACOT11	8.032898398	0.016484802	0.788355367	-1.314172493	0.310779061	-2.10252786	-0.477576305	1.624951555	9.961963646	0.448842023	0.218242442	0.762373699	0.061918165	0.643257247	0.135351239	-6.669452347	-5.345426587	-6.99463123	-5.151431276	-6.176786385	-5.792969541	1.622384539	0.245970741

TESMIN	0.107040463	0.084639902	0.143814875	-0.022400561	0.036774412	0.059174973	8	0.066642304	0.816496581	0.816496581	0.816496581	0.816496581	0.816496581	0.816496581	9	35	0	P_212275	P_212275	P_212275	chr11	68715856	68715916	-	MTL5	"metallothionein-like 5, testis-specific (tesmin) [Source:HGNC Symbol;Acc:HGNC:7446]"	ENSG00000132749	protein_coding	exon	coding exon	1	1395	11:68715856:68715916	ENSG00000132749	TESMIN	7.400940705	0.005773207	1.72538113	1.364307344	2.318146464	-0.361073786	0.592765335	0.953839121	9.961963646	0.115285534	0.202496325	0.043000808	0.725583963	0.566550017	0.362736264	-5.578654898	-5.285887183	-4.704435602	-7.078781037	-6.113123315	-6.595900696	1.933938615	0.188347434

DHX36	0.008481262	-0.15024633	0.096332723	-0.158727593	0.08785146	0.246579053	8	0.065362436	0.816496581	0.816496581	0.816496581	0.816496581	0.816496581	0.816496581	9	36	0	P_136348	P_136348	P_136348	chr3	154278893	154278953	+	DHX36	DEAH (Asp-Glu-Ala-His) box polypeptide 36 [Source:HGNC Symbol;Acc:HGNC:14410]	ENSG00000174953	protein_coding	exon;intron;promoter	multiple non-Ref	0	1396	3:154278893:154278953	ENSG00000174953	DHX36	11.99194255	0.005637532	0.13834441	-2.450783731	1.571357314	-2.589128142	1.433012903	4.022141045	9.961963646	0.892724744	0.034296983	0.147290089	0.027067259	0.182481286	0.00244877	-6.98792632	-3.732533366	-5.843536704	-4.35150752	-5.306886407	-1.846535328	5.571562163	0.016587611

AGPS	0.077618802	-0.065480038	0.097551498	-0.14309884	0.019932697	0.163031536	8	0.045814834	0.816496581	0.816496581	0.816496581	0.816496581	0.816496581	0.816496581	9	37	0	P_237687	P_237687	P_237687	chr2	177436775	177436835	+	AGPS	alkylglycerone phosphate synthase [Source:HGNC Symbol;Acc:HGNC:327]	ENSG00000018510	protein_coding	exon	coding exon	1	1397	2:177436775:177436835	ENSG00000018510	AGPS	7.413061689	0.003892295	1.523735934	-1.285439665	1.915035017	-2.809175599	0.391299083	3.200474682	9.961963646	0.158668915	0.227716792	0.084611084	0.018565352	0.703814957	0.009528767	-5.860519668	-5.378855821	-5.344055331	-3.978061924	-6.215885577	-3.235144567	4.321517692	0.033936774

PRKAR2A	-0.206670648	0.130592516	0.008326292	0.337263164	0.21499694	-0.122266224	8	0.059136533	0.816496581	0.816496581	0.816496581	0.816496581	0.816496581	0.816496581	9	38	0	P_134786	P_134786	P_134786	chr3	48751245	48751305	-	PRKAR2A	"protein kinase, cAMP-dependent, regulatory, type II, alpha [Source:HGNC Symbol;Acc:HGNC:9391]"	ENSG00000114302	protein_coding	3'UTR	3'UTR	1	1398	3:48751245:48751305	ENSG00000114302	PRKAR2A	9.425095667	0.00501507	-3.574258382	2.258527752	0.143998766	5.832786133	3.718257148	-2.114528986	9.961963646	0.005090239	0.047583205	0.888372787	0.000167907	0.004012808	0.0606824	-2.53245186	-4.032093347	-7.035808511	0.877899951	-1.77353454	-5.056622113	11.70251156	0.001325256

MTFR1	0.102062443	0.082159992	0.230098546	-0.019902451	0.128036103	0.147938554	8	0.05506591	0.816496581	0.816496581	0.816496581	0.816496581	0.816496581	0.816496581	9	39	0	P_147319	P_147319	P_147319	chr8	65733394	65733454	-	MTFR1;PDE7A	mitochondrial fission regulator 1 [Source:HGNC Symbol;Acc:HGNC:29510];phosphodiesterase 7A [Source:HGNC Symbol;Acc:HGNC:8791]	ENSG00000066855	protein_coding	intron	multiple non-Ref	0	1399	8:65733394:65733454	ENSG00000066855	MTFR1	7.758912576	0.00464175	1.834723969	1.476947855	4.136363039	-0.357776114	2.30163907	2.659415184	9.961963646	0.096541813	0.170589648	0.00204002	0.727975057	0.044224768	0.023996132	-5.417935918	-5.146802078	-1.637128	-7.080060705	-4.055558553	-4.158455691	5.859186913	0.014255976

CLEC2A	0.152426507	-0.114361768	0.269821994	-0.266788275	0.117395487	0.384183762	8	0.175863282	0.816496581	0.816496581	0.816496581	0.816496581	0.816496581	0.816496581	9	40	0	P_110932	P_110932	P_110932	chr12	9916699	9916759	-	CLEC2A	"C-type lectin domain family 2, member A [Source:HGNC Symbol;Acc:HGNC:24191]"	ENSG00000188393	protein_coding	exon	coding exon	1	1400	12:9916699:9916759	ENSG00000188393	CLEC2A	4.93532966	0.027043469	1.135206647	-0.85171695	2.009517422	-1.986923597	0.874310775	2.861234371	9.961963646	0.282863387	0.414376729	0.072341059	0.075112857	0.402534803	0.016982521	-6.337380136	-5.813502991	-5.198440193	-5.333051901	-5.905438523	-3.815505541	3.158537167	0.073137318

ZNF12	0.159171228	-0.028645117	0.086301633	-0.187816345	-0.072869595	0.11494675	8	0.07471054	0.816496581	0.816496581	0.816496581	0.816496581	0.816496581	0.816496581	9	41	0	P_260440	P_260440	P_260440	chr7	6697684	6697744	-	ZNF12	zinc finger protein 12 [Source:HGNC Symbol;Acc:HGNC:12902]	ENSG00000164631	protein_coding	"5'UTR,exon;exon"	multiple Ref	1	1401	7:6697684:6697744	ENSG00000164631	ZNF12	9.268471086	0.006689065	2.383567662	-0.428956763	1.29235531	-2.812524425	-1.091212352	1.721312073	9.961963646	0.038466944	0.677082743	0.225405961	0.018459196	0.3008679	0.116043178	-4.550444746	-6.086007745	-6.204389216	-3.972347666	-5.699590299	-5.655747361	3.266589041	0.067770713

SNW1	0.045110726	-0.020671051	0.050114131	-0.065781777	0.005003404	0.070785182	8	0.053341944	0.816496581	0.816496581	0.816496581	0.816496581	0.816496581	0.816496581	9	42	0	A_23_P37347	A_23_P37347	A_23_P37347	chr14	77717788	77717848	-	SNW1	SNW domain containing 1 [Source:HGNC Symbol;Acc:HGNC:16696]	ENSG00000100603	protein_coding	3'UTR	3'UTR	1	1402	14:77717788:77717848	ENSG00000100603	SNW1	9.865534501	0.004491666	0.824369167	-0.377750004	0.915803124	-1.202119171	0.091433957	1.293553128	9.961963646	0.429025996	0.713540093	0.381404348	0.257113106	0.928960229	0.225007687	-6.639716993	-6.107418617	-6.607498074	-6.411973539	-6.292240522	-6.226877491	0.797950886	0.522787997

PSMG1	-0.391518768	0.024785729	-0.213563834	0.416304498	0.177954935	-0.238349563	8	0.049420288	0.816496581	0.816496581	0.816496581	0.816496581	0.816496581	0.816496581	9	44	0	P_241815	P_241815	P_241815	chr21	39180284	39180344	-	PSMG1	"proteasome (prosome, macropain) assembly chaperone 1 [Source:HGNC Symbol;Acc:HGNC:3043]"	ENSG00000183527	protein_coding	3'UTR;exon	multiple Ref	1	1404	21:39180284:39180344	ENSG00000183527	PSMG1	12.73756502	0.004168036	-7.427325825	0.470198886	-4.051423091	7.897524711	3.375902735	-4.521621977	9.961963646	2.29E-05	0.648337663	0.002336385	1.35E-05	0.007088513	0.00111627	3.049834321	-6.066864405	-1.776972538	3.512939085	-2.321164044	-1.034061141	27.66882769	3.80E-05

SPOCK1	-0.576338564	0.081010905	-0.528949223	0.657349469	0.04738934	-0.609960129	8	0.17183984	0.816496581	0.816496581	0.816496581	0.816496581	0.816496581	0.816496581	9	45	0	P_140436	P_140436	P_140436	chr5	137192365	137192425	-	SPOCK1	"sparc/osteonectin, cwcv and kazal-like domains proteoglycan (testican) 1 [Source:HGNC Symbol;Acc:HGNC:11251]"	ENSG00000152377	protein_coding	intron	intron sense	0	1405	5:137192365:137192425	ENSG00000152377	SPOCK1	4.387944963	0.025920025	-4.384353854	0.616270536	-4.023851104	5.00062439	0.36050275	-4.64012164	9.961963646	0.001380546	0.551537256	0.002442053	0.000543074	0.725997792	0.000931179	-1.192849663	-5.98575096	-1.822538631	-0.349841272	-6.228021964	-0.845937314	13.74344353	0.000712106

FGD4	-0.060557801	-0.015009905	-0.022058751	0.045547896	0.03849905	-0.007048846	8	0.070378978	0.816496581	0.816496581	0.816496581	0.816496581	0.816496581	0.816496581	9	47	0	P_214989	P_214989	P_214989	chr12	32640473	32640533	+	FGD4	"FYVE, RhoGEF and PH domain containing 4 [Source:HGNC Symbol;Acc:HGNC:19125]"	ENSG00000139132	protein_coding	3'UTR;exon	multiple Ref	1	1407	12:32640473:32640533	ENSG00000139132	FGD4	6.97622094	0.006184374	-0.94312194	-0.233762954	-0.343541072	0.709358986	0.599580868	-0.109778118	9.961963646	0.367928328	0.81990274	0.73833157	0.494368984	0.562173666	0.914765297	-6.533605794	-6.153362081	-6.983050024	-6.881200085	-6.108954804	-7.0647989	0.322813867	0.808898204

NUDT5	0.022088757	0.122072769	0.135925493	0.099984012	0.113836736	0.013852724	8	0.051676375	0.816496581	0.816496581	0.816496581	0.816496581	0.816496581	0.816496581	9	48	0	A_23_P1199	A_23_P1199	A_23_P1199	chr10	12172785	12172845	-	NUDT5	nudix (nucleoside diphosphate linked moiety X)-type motif 5 [Source:HGNC Symbol;Acc:HGNC:8052]	ENSG00000165609	protein_coding	exon	coding exon	1	1408	10:12172785:12172845	ENSG00000165609	NUDT5	11.88250637	0.004351199	0.41012139	2.266522045	2.523725221	1.856400656	2.113603832	0.257203176	9.961963646	0.690397646	0.046942289	0.030274743	0.09317656	0.060776819	0.802256381	-6.907347639	-4.019761373	-4.363798933	-5.532705663	-4.347227492	-7.035358963	3.275701459	0.067339859

NAA35	-0.060105471	0.061072624	0.037493239	0.121178096	0.09759871	-0.023579385	8	0.051893572	0.816496581	0.816496581	0.816496581	0.816496581	0.816496581	0.816496581	9	49	0	P_267815	P_267815	P_267815	chr9	86018307	86018367	+	NAA35	"N(alpha)-acetyltransferase 35, NatC auxiliary subunit [Source:HGNC Symbol;Acc:HGNC:24340]"	ENSG00000135040	protein_coding	exon	coding exon	1	1409	9:86018307:86018367	ENSG00000135040	NAA35	9.709087001	0.004369264	-1.113667374	1.131587319	0.694695444	2.245254693	1.808362817	-0.436891875	9.961963646	0.291570457	0.284312091	0.503125974	0.0486661	0.10078539	0.671507758	-6.360811474	-5.548708136	-6.789797602	-4.921967477	-4.800308021	-6.96809648	1.917594311	0.190950885

CDK19	0.119679027	-0.155715522	-0.050283115	-0.275394549	-0.169962142	0.105432407	8	0.117611875	0.816496581	0.816496581	0.816496581	0.816496581	0.816496581	0.816496581	9	51	0	P_258709	P_258709	P_258709	chr6	110670432	110670492	-	CDK19	cyclin-dependent kinase 19 [Source:HGNC Symbol;Acc:HGNC:19338]	ENSG00000155111	protein_coding	exon	coding exon	1	1411	6:110670432:110670492	ENSG00000155111	CDK19	8.009018774	0.013314978	1.270262404	-1.652750521	-0.533700456	-2.923012925	-1.803962859	1.119050066	9.961963646	0.23285658	0.129504734	0.605251656	0.015279911	0.101510187	0.289375236	-6.182813826	-4.916459015	-6.893767136	-3.783466494	-4.806607788	-6.427241981	2.946757774	0.085191086

DLEC1	0.061106734	-0.041579075	-0.057332326	-0.102685809	-0.11843906	-0.015753251	8	0.140247783	0.816496581	0.816496581	0.816496581	0.816496581	0.816496581	0.816496581	9	52	0	P_243808	P_243808	P_243808	chr3	38086316	38086376	+	DLEC1	deleted in lung and esophageal cancer 1 [Source:HGNC Symbol;Acc:HGNC:2899]	ENSG00000008226	protein_coding	exon	coding exon	1	1412	3:38086316:38086376	ENSG00000008226	DLEC1	4.413522948	0.018002317	0.557789703	-0.379538857	-0.523336453	-0.93732856	-1.081126155	-0.143797595	9.961963646	0.589301979	0.712252969	0.612182071	0.370757185	0.305117717	0.888527555	-6.831198017	-6.106715024	-6.899585443	-6.689283801	-5.709969355	-7.060093989	0.466192747	0.712327765

CAPS2	0.269636676	0.100814147	0.259732667	-0.168822529	-0.009904009	0.15891852	8	0.131799191	0.816496581	0.816496581	0.816496581	0.816496581	0.816496581	0.816496581	9	53	0	P_216305	P_216305	P_216305	chr12	75316311	75316371	-	CAPS2	calcyphosine 2 [Source:HGNC Symbol;Acc:HGNC:16471]	ENSG00000180881	protein_coding	3'UTR;5'UTR;exon	multiple Ref	1	1413	12:75316311:75316371	ENSG00000180881	CAPS2	5.053799016	0.016156566	2.598065348	0.971387668	2.502635962	-1.626677679	-0.095429386	1.531248293	9.961963646	0.026656015	0.354349599	0.031387202	0.134981461	0.925866107	0.156824208	-4.193071398	-5.707745609	-4.399060309	-5.867298027	-6.291843775	-5.921979512	3.159438781	0.073090497

LINC02004	-0.17363051	-0.518811863	-0.321861576	-0.345181352	-0.148231066	0.196950286	8	0.482710617	0.816496581	0.816496581	0.816496581	0.816496581	0.816496581	0.816496581	9	55	0	A_32_P139634	A_32_P139634	A_32_P139634	chr3	134320972	134321032	+	RP11-200A1.1	NA	ENSG00000240006	lincRNA	exon	non-coding exon	1	1415	3:134320972:134321032	ENSG00000240006	LINC02004	10.53252109	0.189326049	-0.488727172	-1.46032776	-0.905961155	-0.971600588	-0.417233983	0.554366604	9.961963646	0.635614817	0.175007131	0.386344175	0.354248718	0.685356336	0.591554739	-6.869570017	-5.167767087	-6.616531275	-6.656522775	-6.204918518	-6.906042822	0.769598543	0.536929874

REXO2	-0.099697903	-0.038416181	0.002444273	0.061281722	0.102142176	0.040860454	8	0.067787549	0.816496581	0.816496581	0.816496581	0.816496581	0.816496581	0.816496581	9	56	0	P_213436	P_213436	P_213436	chr11	114446022	114446082	+	REXO2	RNA exonuclease 2 [Source:HGNC Symbol;Acc:HGNC:17851]	ENSG00000076043	protein_coding	"3'UTR,exon;exon"	multiple Ref	1	1416	11:114446022:114446082	ENSG00000076043	REXO2	11.24749515	0.005896841	-1.590090806	-0.612703119	0.03898393	0.977387687	1.629074736	0.651687049	9.961963646	0.143014868	0.553801154	0.969673386	0.351514817	0.134469446	0.529350126	-5.770021685	-5.987974287	-7.046290717	-6.65089473	-5.050552544	-6.844208938	1.156530137	0.373975198

GABPB1	-0.027941859	-0.013070192	-0.046884785	0.014871666	-0.018942927	-0.033814593	8	0.076741539	0.816496581	0.816496581	0.816496581	0.816496581	0.816496581	0.816496581	9	57	0	P_223425	P_223425	P_223425	chr15	50289492	50289552	-	GABPB1	"GA binding protein transcription factor, beta subunit 1 [Source:HGNC Symbol;Acc:HGNC:4074]"	ENSG00000104064	protein_coding	exon	coding exon	1	1417	15:50289492:50289552	ENSG00000104064	GABPB1	6.982909277	0.006936084	-0.410907197	-0.192207546	-0.689477964	0.218699651	-0.278570767	-0.497270418	9.961963646	0.689839889	0.851441171	0.50626457	0.831299393	0.786271085	0.629789717	-6.907001479	-6.162649261	-6.793559278	-7.123519659	-6.255575218	-6.937950779	0.175646234	0.910442166

G3BP2	-0.040394251	-0.004087529	-0.107711363	0.036306722	-0.067317113	-0.103623835	8	0.0685846	0.816496581	0.816496581	0.816496581	0.816496581	0.816496581	0.816496581	9	60	0	P_249426	P_249426	P_249426	chr4	75661930	75661990	-	G3BP2	GTPase activating protein (SH3 domain) binding protein 2 [Source:HGNC Symbol;Acc:HGNC:30291]	ENSG00000138757	protein_coding	exon	coding exon	1	1420	4:75661930:75661990	ENSG00000138757	G3BP2	9.303490024	0.00598413	-0.639535529	-0.064715143	-1.70532298	0.574820385	-1.065787451	-1.640607837	9.961963646	0.53690314	0.949681021	0.11906421	0.578162479	0.311668676	0.132030131	-6.779665634	-6.179880086	-5.655121071	-6.971867955	-5.72560861	-5.770977227	1.246539275	0.344306756

TSKU	-0.09710682	0.161990877	-0.091846023	0.259097697	0.005260796	-0.253836901	8	0.237011441	0.816496581	0.816496581	0.816496581	0.816496581	0.816496581	0.816496581	9	62	0	A_23_P162142	A_23_P162142	A_23_P162142	chr11	76797818	76797878	+	TSKU	"tsukushi, small leucine rich proteoglycan [Source:HGNC Symbol;Acc:HGNC:28850]"	ENSG00000182704	protein_coding	exon	coding exon	1	1422	11:76797818:76797878	ENSG00000182704	TSKU	6.11300032	0.047317809	-0.546742769	0.912060978	-0.517122786	1.458803748	0.029619984	-1.429183764	9.961963646	0.596588417	0.383277285	0.616356546	0.175417097	0.976955129	0.183550623	-6.837656954	-5.761631059	-6.903022006	-6.09550794	-6.296218631	-6.057055941	0.928291801	0.46249944

LPO	0.161902777	0.086018099	0.220060746	-0.075884678	0.058157969	0.134042647	8	0.251740752	0.816496581	0.816496581	0.816496581	0.816496581	0.816496581	0.816496581	9	63	0	P_122144	P_122144	P_122144	chr17	58238642	58238702	+	LPO	lactoperoxidase [Source:HGNC Symbol;Acc:HGNC:6678]	ENSG00000167419	protein_coding	5'UTR	5'UTR	1	1423	17:58238642:58238702	ENSG00000167419	LPO	4.401283901	0.053098985	0.860511775	0.457185407	1.169620848	-0.403326368	0.309109073	0.712435442	9.961963646	0.409739068	0.657345516	0.269376315	0.695228754	0.7636066	0.492543759	-6.608715407	-6.073086515	-6.347419491	-7.06137596	-6.246114558	-6.800975388	0.513882796	0.681896885

SNAI3	0.171397878	0.103835822	0.187700979	-0.067562056	0.016303101	0.083865157	8	0.170719655	0.816496581	0.816496581	0.816496581	0.816496581	0.816496581	0.816496581	9	65	0	P_119794	P_119794	P_119794	chr16	88678502	88678562	-	SNAI3	snail family zinc finger 3 [Source:HGNC Symbol;Acc:HGNC:18411]	ENSG00000185669	protein_coding	exon	coding exon	1	1425	16:88678502:88678562	ENSG00000185669	SNAI3	5.2071081	0.025611869	1.311687662	0.794643247	1.436453363	-0.517044415	0.124765701	0.641810116	9.961963646	0.219048676	0.44533943	0.181525047	0.616409289	0.903190786	0.535484569	-6.132882588	-5.859748236	-6.023773876	-7.00535541	-6.288412331	-6.850905147	0.851700385	0.497001683

CUL5	0.031402777	-0.124897304	-0.052879498	-0.156300081	-0.084282275	0.072017806	8	0.081597673	0.816496581	0.816496581	0.816496581	0.816496581	0.816496581	0.816496581	9	67	0	P_213162	P_213162	P_213162	chr11	108054895	108054955	+	CUL5	cullin 5 [Source:HGNC Symbol;Acc:HGNC:2556]	ENSG00000166266	protein_coding	3'UTR;exon	multiple Ref	1	1427	11:108054895:108054955	ENSG00000166266	CUL5	9.754267373	0.007553566	0.442524865	-1.760040597	-0.745172713	-2.202565462	-1.187697578	1.014867884	9.961963646	0.667562804	0.109012063	0.473381538	0.052312963	0.262498856	0.334182925	-6.892543597	-4.768918569	-6.752085649	-4.991146076	-5.59658883	-6.536127455	1.856788418	0.201010285

FRS2	0.206798148	-0.109204128	0.079733356	-0.316002275	-0.127064791	0.188937484	8	0.063576907	0.816496581	0.816496581	0.816496581	0.816496581	0.816496581	0.816496581	9	69	0	P_216129	P_216129	P_216129	chr12	69569036	69569096	+	FRS2	fibroblast growth factor receptor substrate 2 [Source:HGNC Symbol;Acc:HGNC:16971]	ENSG00000166225	protein_coding	exon	coding exon	1	1429	12:69569036:69569096	ENSG00000166225	FRS2	7.582856803	0.005452649	3.429955667	-1.811260501	1.322458062	-5.241216168	-2.107497605	3.133718563	9.961963646	0.006474404	0.100310663	0.215571881	0.000382899	0.061403539	0.010670802	-2.77717437	-4.696836547	-6.16774591	0.015322632	-4.356562037	-3.349434539	9.754373485	0.002601896

ARR3	-0.251272458	0.091673242	-0.082398684	0.3429457	0.168873774	-0.174071926	8	0.248110664	0.816496581	0.816496581	0.816496581	0.816496581	0.816496581	0.816496581	9	70	0	P_151126	P_151126	P_151126	chrX	70278078	70278138	+	ARR3	"arrestin 3, retinal (X-arrestin) [Source:HGNC Symbol;Acc:HGNC:710]"	ENSG00000120500	protein_coding	exon	coding exon	1	1430	X:70278078:70278138	ENSG00000120500	ARR3	3.968089474	0.051641839	-1.354221391	0.494068734	-0.444083931	1.848290125	0.91013746	-0.938152665	9.961963646	0.205585157	0.631969654	0.666472811	0.094422789	0.384242532	0.370353831	-6.080449677	-6.055018944	-6.940481871	-5.544902344	-5.874035112	-6.610768283	1.233314506	0.348497463

CERS6	-0.064000414	0.024661834	-0.032296292	0.088662248	0.031704122	-0.056958126	8	0.07177152	0.816496581	0.816496581	0.816496581	0.816496581	0.816496581	0.816496581	9	73	0	P_237400	P_237400	P_237400	chr2	168694991	168695051	+	CERS6	ceramide synthase 6 [Source:HGNC Symbol;Acc:HGNC:23826]	ENSG00000172292	protein_coding	exon	coding exon	1	1433	2:168694991:168695051	ENSG00000172292	CERS6	8.811600233	0.006343339	-0.984168463	0.37923817	-0.496637291	1.363406633	0.487531172	-0.875875461	9.961963646	0.348331189	0.712469255	0.630220501	0.202770572	0.636432392	0.401723473	-6.494149208	-6.106833516	-6.914075911	-6.218048105	-6.171796887	-6.667739801	0.703796644	0.571221951

CES2	-0.181989356	0.003760457	-0.197723248	0.185749813	-0.015733892	-0.201483705	8	0.064481489	0.816496581	0.816496581	0.816496581	0.816496581	0.816496581	0.816496581	9	75	0	P_119212	P_119212	P_119212	chr16	66944328	66944388	+	CES2	carboxylesterase 2 [Source:HGNC Symbol;Acc:HGNC:1864]	ENSG00000172831	protein_coding	3'UTR	3'UTR	1	1435	16:66944328:66944388	ENSG00000172831	CES2	10.15955263	0.005545674	-2.993053249	0.061845635	-3.251817699	3.054898884	-0.258764449	-3.313663334	9.961963646	0.013557554	0.951908994	0.00873619	0.012201216	0.801085027	0.007870677	-3.522667369	-6.180071458	-3.125055826	-3.557406185	-6.26119376	-3.04153901	6.652706984	0.009594691

RCOR2	0.210980261	0.064552072	0.08198812	-0.146428189	-0.128992141	0.017436048	8	0.157655976	0.816496581	0.816496581	0.816496581	0.816496581	0.816496581	0.816496581	9	76	0	A_23_P24397	A_23_P24397	A_23_P24397	chr11	63912677	63912735	-	RCOR2	REST corepressor 2 [Source:HGNC Symbol;Acc:HGNC:27455]	ENSG00000167771	protein_coding	exon	coding exon	1	1436	11:63912677:63912735	ENSG00000167771	RCOR2	10.76533332	0.022166931	1.735540169	0.531010404	0.674440705	-1.204529765	-1.061099464	0.1434303	9.961963646	0.113413522	0.607046586	0.515376626	0.256221567	0.313692107	0.888810142	-5.563939091	-6.035590923	-6.804256454	-6.409205973	-5.730353361	-7.060151503	1.057723045	0.40982025

ELOVL4	-0.122442681	-0.083631804	-0.154779208	0.038810876	-0.032336527	-0.071147404	8	0.060441993	0.816496581	0.816496581	0.816496581	0.816496581	0.816496581	0.816496581	9	78	0	P_143095	P_143095	P_143095	chr6	79916544	79916604	-	ELOVL4	ELOVL fatty acid elongase 4 [Source:HGNC Symbol;Acc:HGNC:14415]	ENSG00000118402	protein_coding	3'UTR	3'UTR	1	1438	6:79916544:79916604	ENSG00000118402	ELOVL4	5.280745588	0.00514043	-2.091600389	-1.428622056	-2.643982067	0.662978333	-0.552381678	-1.215360011	9.961963646	0.063064235	0.183707937	0.024639237	0.522388141	0.592863123	0.252246677	-5.02264143	-5.207344373	-4.161624048	-6.914426107	-6.136909869	-6.319317508	2.604693347	0.11001879

ACAT2	-0.571577772	0.141008577	-0.446491208	0.712586349	0.125086564	-0.587499785	8	0.075422166	0.816496581	0.816496581	0.816496581	0.816496581	0.816496581	0.816496581	9	79	0	P_260108	P_260108	P_260108	chr6	159776196	159776256	+	ACAT2	acetyl-CoA acetyltransferase 2 [Source:HGNC Symbol;Acc:HGNC:94]	ENSG00000120437	protein_coding	exon	coding exon	1	1439	6:159776196:159776256	ENSG00000120437	ACAT2	10.6475301	0.006774862	-8.50492975	2.098171238	-6.643673955	10.60310099	1.861255795	-8.741845194	9.961963646	7.04E-06	0.062372684	5.86E-05	9.57E-07	0.092437845	5.51E-06	4.258870563	-4.276620814	2.049848036	6.237437375	-4.724006914	4.47825354	52.19740622	2.13E-06

RTTN	-0.16529525	-0.050243284	-0.182893545	0.115051965	-0.017598295	-0.13265026	8	0.034376804	0.816496581	0.816496581	0.816496581	0.816496581	0.816496581	0.816496581	9	81	0	P_231825	P_231825	P_231825	chr18	70204096	70204156	-	RTTN	rotatin [Source:HGNC Symbol;Acc:HGNC:18654]	ENSG00000176225	protein_coding	exon	coding exon	1	1441	18:70204096:70204156	ENSG00000176225	RTTN	6.216249668	0.003155705	-3.603775892	-1.095406778	-3.987454855	2.508369114	-0.383678963	-2.892048076	9.961963646	0.004847133	0.29911405	0.002589282	0.031080838	0.709277702	0.016110559	-2.482556959	-5.586286239	-1.882813041	-4.48723468	-6.2189784	-3.762910995	7.481663357	0.0065412

TSEN2	-0.03698104	0.082634224	0.02155467	0.119615265	0.05853571	-0.061079555	8	0.09870058	0.816496581	0.816496581	0.816496581	0.816496581	0.816496581	0.816496581	9	82	0	P_134079	P_134079	P_134079	chr3	12519059	12519119	-	TSEN2;MKRN2OS	TSEN2 tRNA splicing endonuclease subunit [Source:HGNC Symbol;Acc:HGNC:28422];MKRN2 opposite strand [Source:HGNC Symbol;Acc:HGNC:40375]	ENSG00000154743	protein_coding	exon;intron	multiple non-Ref	0	1442	3:12519059:12519119	ENSG00000154743	TSEN2	7.53722055	0.010029884	-0.452248146	1.010549579	0.26359614	1.462797725	0.715844286	-0.746953439	9.961963646	0.660778287	0.336146855	0.797463315	0.174344454	0.490526219	0.472352954	-6.887890288	-5.670654151	-7.009307962	-6.090259228	-6.031132752	-6.774862095	0.750895238	0.546466502

C12orf40	0.198811823	0.0803748	0.23391695	-0.118437022	0.035105127	0.15354215	8	0.130532934	0.816496581	0.816496581	0.816496581	0.816496581	0.816496581	0.816496581	9	85	0	A_14_P135131	A_14_P135131	A_14_P135131	chr12	39688336	39688396	+	C12orf40	chromosome 12 open reading frame 40 [Source:HGNC Symbol;Acc:HGNC:26846]	ENSG00000180116	protein_coding	intron	intron sense	0	1445	12:39688336:39688396	ENSG00000180116	C12orf40	4.075592727	0.015889807	1.931650383	0.780919423	2.272730867	-1.15073096	0.341080483	1.491811443	9.961963646	0.082320774	0.453007397	0.0464503	0.276715033	0.74012739	0.166721075	-5.271467541	-5.870450721	-4.778615997	-6.469977532	-6.235178188	-5.974872201	2.195405494	0.151835055

CTNND1	0.084347899	-0.123746384	-0.068017004	-0.208094283	-0.152364904	0.05572938	8	0.056388603	0.816496581	0.816496581	0.816496581	0.816496581	0.816496581	0.816496581	9	87	0	P_211830	P_211830	P_211830	chr11	57810163	57810223	+	CTNND1	"catenin (cadherin-associated protein), delta 1 [Source:HGNC Symbol;Acc:HGNC:2515]"	ENSG00000198561	protein_coding	exon	coding exon	1	1447	11:57810163:57810223	ENSG00000198561	CTNND1	8.48955976	0.004760136	1.497304811	-2.196688447	-1.207406334	-3.693993258	-2.704711145	0.989282113	9.961963646	0.165310695	0.052835231	0.25516094	0.004176211	0.022203867	0.345944337	-5.89590614	-4.127058654	-6.304507065	-2.465840028	-3.41100562	-6.561557071	5.077486591	0.021754382

GPLD1	-0.125195819	0.047721507	0.133553123	0.172917327	0.258748942	0.085831615	8	0.114911575	0.816496581	0.816496581	0.816496581	0.816496581	0.816496581	0.816496581	9	88	0	P_256370	P_256370	P_256370	chr6	24460278	24460338	-	GPLD1	glycosylphosphatidylinositol specific phospholipase D1 [Source:HGNC Symbol;Acc:HGNC:4459]	ENSG00000112293	protein_coding	exon	coding exon	1	1448	6:24460278:24460338	ENSG00000112293	GPLD1	4.887570164	0.012810754	-1.354715533	0.516383595	1.445148017	1.871099128	2.79986355	0.928764422	9.961963646	0.205432909	0.616854111	0.179127591	0.0909568	0.018863783	0.374967511	-6.079833742	-6.043441738	-6.012468356	-5.510536656	-3.256666498	-6.619567785	2.732201697	0.099875828

MYO1C	-0.191735126	0.085794722	-0.201504192	0.277529848	-0.009769065	-0.287298914	8	0.098179404	0.816496581	0.816496581	0.816496581	0.816496581	0.816496581	0.816496581	9	89	0	P_227320	P_227320	P_227320	chr17	1477890	1477950	-	MYO1C	myosin IC [Source:HGNC Symbol;Acc:HGNC:7597]	ENSG00000197879	protein_coding	exon	coding exon	1	1449	17:1477890:1477950	ENSG00000197879	MYO1C	8.569422984	0.009947484	-2.354456656	1.053536499	-2.474418197	3.407993155	-0.119961541	-3.527954695	9.961963646	0.040423603	0.316977472	0.032939097	0.006717014	0.906898459	0.005497409	-4.598367627	-5.62858674	-4.446138538	-2.952248287	-6.289036705	-2.67632216	6.14190386	0.012336232

TRIM7	0.13198707	0.400283979	0.290491319	0.268296908	0.158504248	-0.10979266	8	0.061378413	0.816496581	0.816496581	0.816496581	0.816496581	0.816496581	0.816496581	9	90	0	A_24_P286190	A_24_P286190	A_24_P286190	chr5	181204898	181204958	-	TRIM7	tripartite motif containing 7 [Source:HGNC Symbol;Acc:HGNC:16278]	ENSG00000146054	protein_coding	exon	coding exon	1	1450	5:181204898:181204958	ENSG00000146054	TRIM7	6.383386589	0.005232039	2.234814767	6.777637721	4.918620342	4.542822955	2.683805575	-1.85901738	9.961963646	0.049534576	4.96E-05	0.000612936	0.001080486	0.023013787	0.092777745	-4.793517293	2.304401071	-0.391690634	-1.067036318	-3.444845838	-5.452472081	17.73659909	0.000254409

PIK3R1	0.164600836	-0.06170381	-0.111972424	-0.226304646	-0.276573259	-0.050268614	8	0.0513576	0.816496581	0.816496581	0.816496581	0.816496581	0.816496581	0.816496581	9	91	0	A_16_P01251876	A_16_P01251876	A_16_P01251876	chr5	68290748	68290808	+	PIK3R1	"phosphoinositide-3-kinase, regulatory subunit 1 (alpha) [Source:HGNC Symbol;Acc:HGNC:8979]"	ENSG00000145675	protein_coding	exon	coding exon	1	1451	5:68290748:68290808	ENSG00000145675	PIK3R1	7.904532621	0.004324823	3.065444752	-1.149141309	-2.085319171	-4.214586061	-5.150763923	-0.936177862	9.961963646	0.011984115	0.277339736	0.063732133	0.001801985	0.000436237	0.371320914	-3.39902367	-5.530140884	-5.079511912	-1.598290962	0.354128888	-6.612625401	10.03926116	0.002343726

DNAAF6	-0.351574868	0.017926422	-0.222397755	0.36950129	0.129177113	-0.240324177	8	0.147981023	0.816496581	0.816496581	0.816496581	0.816496581	0.816496581	0.816496581	9	92	0	P_271066	P_271066	P_271066	chrX	107212968	107213028	+	PIH1D3	PIH1 domain containing 3 [Source:HGNC Symbol;Acc:HGNC:28570]	ENSG00000080572	protein_coding	exon	coding exon	1	1452	X:107212968:107213028	ENSG00000080572	DNAAF6	4.093031964	0.01979228	-3.06066319	0.156059904	-1.936101481	3.216723094	1.12456171	-2.092161384	9.961963646	0.012082058	0.879102941	0.081717141	0.00927026	0.287140779	0.063004909	-3.407192565	-6.169264644	-5.311856145	-3.27978247	-5.664741731	-5.092106331	4.854931427	0.024699879

MAFG-DT	0.345356381	-0.341987024	0.132385702	-0.687343406	-0.21297068	0.474372726	8	0.236563031	0.816496581	0.816496581	0.816496581	0.816496581	0.816496581	0.816496581	9	94	0	A_24_P136629	A_24_P136629	A_24_P136629	chr17	81929917	81929977	+	MAFG-AS1	MAFG antisense RNA 1 (head to head) [Source:HGNC Symbol;Acc:HGNC:43649]	ENSG00000265688	antisense	exon	non-coding exon	1	1454	17:81929917:81929977	ENSG00000265688	MAFG-DT	11.61995449	0.047147276	1.947981431	-1.928976584	0.746721077	-3.876958015	-1.201260355	2.675697661	9.961963646	0.080126275	0.082685383	0.472487091	0.003096085	0.257431333	0.023335797	-5.246449742	-4.527558903	-6.750891368	-2.157880271	-5.581585787	-4.130911919	5.284393758	0.019385197

PKHD1L1	0.000635179	-0.067654119	0.029140162	-0.068289299	0.028504983	0.096794282	8	0.091359937	0.816496581	0.816496581	0.816496581	0.816496581	0.816496581	0.816496581	9	95	0	A_23_P392486	A_23_P392486	A_23_P392486	chr8	109526791	109526851	+	PKHD1L1	polycystic kidney and hepatic disease 1 (autosomal recessive)-like 1 [Source:HGNC Symbol;Acc:HGNC:20313]	ENSG00000205038	protein_coding	exon	coding exon	1	1455	8:109526791:109526851	ENSG00000205038	PKHD1L1	3.802183882	0.008909489	0.008241675	-0.877836156	0.378103926	-0.886077831	0.369862251	1.255940082	9.961963646	0.993586879	0.400708405	0.713285362	0.396461121	0.719226731	0.237793673	-6.998323252	-5.791420859	-6.969606298	-6.736426907	-6.224435701	-6.271864934	0.568824053	0.64810822

PSPH	-0.035234175	0.00788876	0.156064397	0.043122934	0.191298572	0.148175638	8	0.051090441	0.816496581	0.816496581	0.816496581	0.816496581	0.816496581	0.816496581	9	97	0	P_144901	P_144901	P_144901	chr7	56015022	56015082	-	PSPH	phosphoserine phosphatase [Source:HGNC Symbol;Acc:HGNC:9577]	ENSG00000146733	protein_coding	exon	coding exon	1	1457	7:56015022:56015082	ENSG00000146733	PSPH	10.28491665	0.004302844	-0.657857687	0.147291122	2.913880185	0.805148809	3.571737872	2.766589063	9.961963646	0.525538678	0.88584056	0.01552029	0.439527742	0.005111576	0.019970159	-6.767224087	-6.170663562	-3.702993619	-6.806223266	-2.00662334	-3.976716558	5.000793369	0.022719443

ELP1	-0.027283112	0.003517944	-0.019331777	0.030801057	0.007951335	-0.022849722	8	0.043585818	0.816496581	0.816496581	0.816496581	0.816496581	0.816496581	0.816496581	9	98	0	P_268391	P_268391	P_268391	chr9	108903562	108903622	-	IKBKAP	"inhibitor of kappa light polypeptide gene enhancer in B-cells, kinase complex-associated protein [Source:HGNC Symbol;Acc:HGNC:5959]"	ENSG00000070061	protein_coding	exon	coding exon	1	1458	9:108903562:108903622	ENSG00000070061	ELP1	6.527559168	0.003732266	-0.546957105	0.070525852	-0.38755303	0.617482957	0.159404075	-0.458078882	9.961963646	0.596446593	0.945170824	0.706498259	0.550769047	0.876536034	0.656725191	-6.837532791	-6.179466009	-6.965713031	-6.945015778	-6.283187978	-6.95794328	0.178477408	0.90854954

SEC23IP	-0.000892015	-0.120526775	-0.105318371	-0.11963476	-0.104426356	0.015208404	8	0.07330105	0.816496581	0.816496581	0.816496581	0.816496581	0.816496581	0.816496581	9	99	0	P_210208	P_210208	P_210208	chr10	119920924	119920984	+	SEC23IP	SEC23 interacting protein [Source:HGNC Symbol;Acc:HGNC:17018]	ENSG00000107651	protein_coding	exon	coding exon	1	1459	10:119920924:119920984	ENSG00000107651	SEC23IP	8.385915095	0.006521531	-0.01352829	-1.827908178	-1.597257638	-1.814379888	-1.583729348	0.23065054	9.961963646	0.989473411	0.097622929	0.141408596	0.099801914	0.144454158	0.822254036	-6.998260449	-4.673194249	-5.807805397	-5.595613142	-5.111570473	-7.042393233	1.957672663	0.184639962

LHX8	0.127249901	0.073390063	0.231530244	-0.053859838	0.104280343	0.158140181	8	0.233722917	0.816496581	0.816496581	0.816496581	0.816496581	0.816496581	0.816496581	9	100	0	P_202313	P_202313	P_202313	chr1	75141044	75141104	+	LHX8	LIM homeobox 8 [Source:HGNC Symbol;Acc:HGNC:28838]	ENSG00000162624	protein_coding	exon	coding exon	1	1460	1:75141044:75141104	ENSG00000162624	LHX8	4.578102649	0.046074663	0.726059138	0.418747092	1.321059179	-0.307312046	0.595000041	0.902312087	9.961963646	0.484511358	0.684285942	0.216020846	0.764934065	0.565112999	0.388187126	-6.718105332	-6.090486086	-6.169461858	-7.098211349	-6.111761403	-6.643960166	0.6183902	0.618848997

SH3YL1	0.204006652	0.074380289	0.137147384	-0.129626363	-0.066859268	0.062767095	8	0.049943189	0.816496581	0.816496581	0.816496581	0.816496581	0.816496581	0.816496581	9	101	0	P_233442	P_233442	P_233442	chr2	253011	253071	-	SH3YL1	SH3 and SYLF domain containing 1 [Source:HGNC Symbol;Acc:HGNC:29546]	ENSG00000035115	protein_coding	5'UTR;exon	multiple Ref	1	1461	2:253011:253071	ENSG00000035115	SH3YL1	6.979922546	0.004209761	3.850891246	1.404024821	2.58883549	-2.446866425	-1.262055756	1.184810669	9.961963646	0.003230154	0.190710613	0.027080831	0.034527313	0.235675154	0.263587722	-2.067551226	-5.23766293	-4.254554462	-4.589969408	-5.512811134	-6.35427857	5.414405772	0.01805388

ABCC4	-0.94335967	0.058886206	-1.004057152	1.002245876	-0.060697482	-1.062943359	8	0.090882157	0.816496581	0.816496581	0.816496581	0.816496581	0.816496581	0.816496581	9	102	0	P_114013	P_114013	P_114013	chr13	95163121	95163181	-	ABCC4	"ATP-binding cassette, sub-family C (CFTR/MRP), member 4 [Source:HGNC Symbol;Acc:HGNC:55]"	ENSG00000125257	protein_coding	exon	coding exon	1	1462	13:95163121:95163181	ENSG00000125257	ABCC4	7.557723868	0.008839566	-12.28874291	0.767085423	-13.07942304	13.05582833	-0.790680136	-13.84650846	9.961963646	2.42E-07	0.460823643	1.35E-07	1.37E-07	0.447544934	7.84E-08	7.611897359	-5.881072984	8.19013618	8.186784669	-5.974512737	8.716305663	114.2464257	5.14E-08

CCN4	0.009828983	-0.174816906	-0.223950555	-0.184645889	-0.233779538	-0.049133649	8	0.124914078	0.816496581	0.816496581	0.816496581	0.816496581	0.816496581	0.816496581	9	103	0	A_24_P372189	A_24_P372189	A_24_P372189	chr8	133231137	133231197	+	WISP1	WNT1 inducible signaling pathway protein 1 [Source:HGNC Symbol;Acc:HGNC:12769]	ENSG00000104415	protein_coding	3'ext	3'extended sense	0	1463	8:133231137:133231197	ENSG00000104415	CCN4	4.010147005	0.014737167	0.099162438	-1.763689215	-2.259387765	-1.862851653	-2.358550203	-0.49569855	9.961963646	0.922976344	0.108370089	0.047513851	0.09219622	0.040142691	0.630859494	-6.992997091	-4.763817565	-4.800324761	-5.52298624	-3.965883283	-6.938782724	2.917326384	0.087046357

XPO6	-0.180188556	0.021076669	-0.182497634	0.201265225	-0.002309078	-0.203574303	8	0.08169589	0.816496581	0.816496581	0.816496581	0.816496581	0.816496581	0.816496581	9	104	0	P_226014	P_226014	P_226014	chr16	28106382	28106442	-	XPO6	exportin 6 [Source:HGNC Symbol;Acc:HGNC:19733]	ENSG00000169180	protein_coding	exon	coding exon	1	1464	16:28106382:28106442	ENSG00000169180	XPO6	7.939158329	0.007566445	-2.537037487	0.296757459	-2.569549086	2.833794947	-0.032511599	-2.866306546	9.961963646	0.029592704	0.772746746	0.027990356	0.017799066	0.974706235	0.016835777	-4.295425307	-6.135874591	-4.286970813	-3.936036439	-6.296123082	-3.806851254	4.895728454	0.024125881

CUL9	0.073738685	-0.082014505	-0.104755346	-0.15575319	-0.178494031	-0.022740842	8	0.124137612	0.816496581	0.816496581	0.816496581	0.816496581	0.816496581	0.816496581	9	105	0	P_257115	P_257115	P_257115	chr6	43187832	43187892	+	CUL9	cullin 9 [Source:HGNC Symbol;Acc:HGNC:15982]	ENSG00000112659	protein_coding	exon	coding exon	1	1465	6:43187832:43187892	ENSG00000112659	CUL9	6.074643405	0.014581872	0.747884226	-0.831820566	-1.06246635	-1.579704793	-1.810350576	-0.230645783	9.961963646	0.471815879	0.425000346	0.313101102	0.145371327	0.100459514	0.822257631	-6.701466167	-5.82993964	-6.463387804	-5.932666985	-4.797459553	-7.042394426	1.367687489	0.308432226

SMIM22	0.107779989	-0.207512518	0.231184956	-0.315292507	0.123404967	0.438697474	8	0.209491317	0.816496581	0.816496581	0.816496581	0.816496581	0.816496581	0.816496581	9	106	0	A_24_P229884	A_24_P229884	A_24_P229884	chr16	4795786	4795846	+	SMIM22	small integral membrane protein 22 [Source:HGNC Symbol;Acc:HGNC:48329]	ENSG00000267795	protein_coding	exon	coding exon	1	1466	16:4795786:4795846	ENSG00000267795	SMIM22	6.859594959	0.037450026	0.68211516	-1.313299769	1.463117268	-1.995414929	0.781002108	2.776417037	9.961963646	0.510713899	0.218525327	0.17425888	0.074059601	0.452960941	0.019636754	-6.75025797	-5.346449505	-5.988963152	-5.319855727	-5.982115202	-3.960003679	2.771814352	0.096952254

LYST	0.226873823	0.004250115	0.190153958	-0.222623708	-0.036719865	0.185903843	8	0.073444518	0.816496581	0.816496581	0.816496581	0.816496581	0.816496581	0.816496581	9	108	0	P_206830	P_206830	P_206830	chr1	235812970	235813030	-	LYST	lysosomal trafficking regulator [Source:HGNC Symbol;Acc:HGNC:1968]	ENSG00000143669	protein_coding	exon	coding exon	1	1468	1:235812970:235813030	ENSG00000143669	LYST	5.265338193	0.006538438	3.436315313	0.064373824	2.880142581	-3.371941489	-0.556172731	2.815768757	9.961963646	0.006405856	0.949946008	0.016441963	0.007135819	0.590365546	0.018356936	-2.766364055	-6.179903304	-3.760572862	-3.013887002	-6.134743665	-3.893017601	6.619249902	0.009750286

FHDC1	-0.133013356	0.075117883	-0.215941654	0.20813124	-0.082928298	-0.291059538	8	0.086953931	0.816496581	0.816496581	0.816496581	0.816496581	0.816496581	0.816496581	9	109	0	P_251238	P_251238	P_251238	chr4	152960570	152960630	+	FHDC1	FH2 domain containing 1 [Source:HGNC Symbol;Acc:HGNC:29363]	ENSG00000137460	protein_coding	exon	coding exon	1	1469	4:152960570:152960630	ENSG00000137460	FHDC1	6.14102455	0.008278568	-1.790454419	1.011140161	-2.906728316	2.801594579	-1.116273897	-3.917868477	9.961963646	0.103765427	0.335877757	0.015711202	0.018807944	0.290505828	0.002897292	-5.483613131	-5.670085717	-3.715203585	-3.990995076	-5.67347764	-2.019761561	6.186982327	0.012059514

SELENBP1	0.485043549	-0.221466006	0.232481535	-0.706509555	-0.252562014	0.453947541	8	0.098872483	0.816496581	0.816496581	0.816496581	0.816496581	0.816496581	0.816496581	9	110	0	P_103127	P_103127	P_103127	chr1	151365767	151365827	-	SELENBP1	selenium binding protein 1 [Source:HGNC Symbol;Acc:HGNC:10719]	ENSG00000143416	protein_coding	3'UTR;exon	multiple Ref	1	1470	1:151365767:151365827	ENSG00000143416	SELENBP1	10.10851538	0.010057159	5.92364073	-2.70467478	2.839202982	-8.62831551	-3.084437748	5.543877762	9.961963646	0.000148579	0.022205251	0.017635057	6.19E-06	0.011603003	0.000249602	1.116988896	-3.330032363	-3.830371197	4.320680662	-2.793699201	0.524190377	27.52700402	3.89E-05

PSMB7	-0.043446886	0.005342011	0.016477279	0.048788897	0.059924165	0.011135268	8	0.09397703	0.816496581	0.816496581	0.816496581	0.816496581	0.816496581	0.816496581	9	111	0	P_268917	P_268917	P_268917	chr9	124405316	124405376	-	PSMB7	"proteasome (prosome, macropain) subunit, beta type, 7 [Source:HGNC Symbol;Acc:HGNC:9544]"	ENSG00000136930	protein_coding	exon	coding exon	1	1471	9:124405316:124405376	ENSG00000136930	PSMB7	8.96022603	0.009299006	-0.551805481	0.067847228	0.209272831	0.619652709	0.761078313	0.141425603	9.961963646	0.593243216	0.947249695	0.838452696	0.54939578	0.464244685	0.890352786	-6.834711957	-6.179661307	-7.023256448	-6.943602461	-5.997505933	-7.060462831	0.222683089	0.878461719

IL13RA1	0.106907004	0.058995441	0.195513113	-0.047911563	0.088606109	0.136517672	8	0.053529894	0.816496581	0.816496581	0.816496581	0.816496581	0.816496581	0.816496581	9	112	0	P_151544	P_151544	P_151544	chrX	118794249	118794309	-	IL13RA1	"interleukin 13 receptor, alpha 1 [Source:HGNC Symbol;Acc:HGNC:5974]"	ENSG00000131724	protein_coding	3'UTR	3'UTR antisense	0	1472	X:118794249:118794309	ENSG00000131724	IL13RA1	7.746321027	0.004507796	1.950157331	1.076172628	3.566476625	-0.873984703	1.616319294	2.490303997	9.961963646	0.07983806	0.307221677	0.005156415	0.402704023	0.137214071	0.032056336	-5.243109392	-5.605878163	-2.589111611	-6.747222048	-5.067815757	-4.442714361	4.543161329	0.029669304

PLA2G4A	-0.128594498	0.132874482	-0.233588621	0.26146898	-0.104994123	-0.366463104	8	0.082514269	0.816496581	0.816496581	0.816496581	0.816496581	0.816496581	0.816496581	9	114	0	P_205534	P_205534	P_205534	chr1	186946708	186946768	+	PLA2G4A	"phospholipase A2, group IVA (cytosolic, calcium-dependent) [Source:HGNC Symbol;Acc:HGNC:9035]"	ENSG00000116711	protein_coding	exon	coding exon	1	1474	1:186946708:186946768	ENSG00000116711	PLA2G4A	4.601492675	0.007674365	-1.797822449	1.857659007	-3.265698566	3.655481456	-1.467876117	-5.123357573	9.961963646	0.102529692	0.092984577	0.008533768	0.004449944	0.172988749	0.000453925	-5.47273789	-4.630692204	-3.101329686	-2.531017805	-5.262784354	-0.098824082	9.852307909	0.002509471

GALK2	-0.021645192	0.014020206	0.035122911	0.035665398	0.056768104	0.021102706	8	0.053527186	0.816496581	0.816496581	0.816496581	0.816496581	0.816496581	0.816496581	9	115	0	P_116492	P_116492	P_116492	chr15	49328459	49328519	+	GALK2	galactokinase 2 [Source:HGNC Symbol;Acc:HGNC:4119]	ENSG00000156958	protein_coding	3'UTR	3'UTR	1	1475	15:49328459:49328519	ENSG00000156958	GALK2	8.295109883	0.004507563	-0.39485365	0.255757919	0.640715478	0.650611569	1.035569128	0.384957559	9.961963646	0.701272755	0.803341151	0.536166979	0.530016084	0.324886962	0.708359878	-6.913946048	-6.147721445	-6.827462871	-6.922938894	-5.755901566	-6.991021009	0.379288166	0.770106983

KLHL5	0.160402743	0.055271268	0.263250509	-0.105131475	0.102847766	0.207979241	8	0.077498461	0.816496581	0.816496581	0.816496581	0.816496581	0.816496581	0.816496581	9	118	0	A_24_P848714	A_24_P848714	A_24_P848714	chr4	39126129	39126189	+	KLHL5;RP11-360F5.1	kelch-like family member 5 [Source:HGNC Symbol;Acc:HGNC:6356];	ENSG00000109790	protein_coding;antisense	3'ext;intron	multiple non-Ref	0	1478	4:39126129:39126189	ENSG00000109790	KLHL5	7.000528735	0.007029839	2.343067193	0.807369578	3.845405756	-1.535697615	1.502338562	3.038036178	9.961963646	0.041215328	0.43830568	0.003259133	0.155740525	0.164027434	0.012556721	-4.617073133	-5.849677909	-2.119355241	-5.992867132	-5.21853562	-3.513261976	5.795667845	0.014735278

RITA1	-0.185115342	0.019959811	-0.057483495	0.205075153	0.127631847	-0.077443306	8	0.059585305	0.816496581	0.816496581	0.816496581	0.816496581	0.816496581	0.816496581	9	119	0	P_112865	P_112865	P_112865	chr12	113191742	113191802	+	RITA1	RBPJ interacting and tubulin associated 1 [Source:HGNC Symbol;Acc:HGNC:25925]	ENSG00000139405	protein_coding	exon	coding exon	1	1479	12:113191742:113191802	ENSG00000139405	RITA1	9.806243217	0.005057856	-3.187900997	0.343731104	-0.989932495	3.531632101	2.197968502	-1.333663599	9.961963646	0.009733876	0.738192947	0.345641629	0.00546387	0.052721051	0.21200349	-3.189813599	-6.120183321	-6.536848556	-2.741344892	-4.217327823	-6.177869542	5.057163268	0.022005269

MACF1	0.067909416	-0.182980917	-0.041687974	-0.250890332	-0.10959739	0.141292943	8	0.062145877	0.816496581	0.816496581	0.816496581	0.816496581	0.816496581	0.816496581	9	120	0	P_201214	P_201214	P_201214	chr1	39315631	39315691	+	MACF1	microtubule-actin crosslinking factor 1 [Source:HGNC Symbol;Acc:HGNC:13664]	ENSG00000127603	protein_coding	3'UTR;exon	multiple Ref	1	1480	1:39315631:39315691	ENSG00000127603	MACF1	7.777843656	0.005308169	1.141571863	-3.075948516	-0.700783798	-4.21752038	-1.842355662	2.375164718	9.961963646	0.280329625	0.011771795	0.499478568	0.001793646	0.095344403	0.039022018	-6.330389649	-2.735985368	-6.785375545	-1.5934831	-4.751390429	-4.633878743	6.346483917	0.011138237

MTHFD1L	-0.267273876	0.160538417	0.069622254	0.427812293	0.33689613	-0.090916163	8	0.074964762	0.816496581	0.816496581	0.816496581	0.816496581	0.816496581	0.816496581	9	122	0	A_23_P214908	A_23_P214908	A_23_P214908	chr6	150898938	150898998	+	MTHFD1L	methylenetetrahydrofolate dehydrogenase (NADP+ dependent) 1-like [Source:HGNC Symbol;Acc:HGNC:21055]	ENSG00000120254	protein_coding	3'UTR	3'UTR	1	1482	6:150898938:150898998	ENSG00000120254	MTHFD1L	7.969097907	0.006719622	-3.993279619	2.398568829	1.040210633	6.391848448	5.033490252	-1.358358196	9.961963646	0.002565107	0.037495179	0.322829598	8.05E-05	0.000517503	0.204313501	-1.830980536	-3.814453221	-6.486372416	1.647320289	0.191861743	-6.147180458	15.13639007	0.000485238

TCTN3	0.015780345	0.090026602	-0.027125018	0.074246257	-0.042905363	-0.117151619	8	0.087508001	0.816496581	0.816496581	0.816496581	0.816496581	0.816496581	0.816496581	9	123	0	P_209321	P_209321	P_209321	chr10	95683521	95683581	-	TCTN3	tectonic family member 3 [Source:HGNC Symbol;Acc:HGNC:24519]	ENSG00000119977	protein_coding	exon	coding exon	1	1483	10:95683521:95683581	ENSG00000119977	TCTN3	9.237534823	0.008356195	0.211425735	1.206180242	-0.363422144	0.994754507	-0.574847878	-1.569602386	9.961963646	0.836817632	0.255612584	0.723883085	0.343403404	0.578144589	0.147696312	-6.974022427	-5.46833679	-6.975470203	-6.633840381	-6.123870308	-5.869727813	0.902547589	0.473809242

RGL2	-0.044544107	0.006309191	-0.071750937	0.050853299	-0.02720683	-0.078060128	8	0.076178653	0.816496581	0.816496581	0.816496581	0.816496581	0.816496581	0.816496581	9	124	0	P_256783	P_256783	P_256783	chr6	33292441	33292501	-	RGL2	ral guanine nucleotide dissociation stimulator-like 2 [Source:HGNC Symbol;Acc:HGNC:9769]	ENSG00000237441	protein_coding	exon	coding exon	1	1484	6:33292441:33292501	ENSG00000237441	RGL2	7.544888802	0.00686696	-0.658345146	0.093247475	-1.060451856	0.751592621	-0.40210671	-1.153699331	9.961963646	0.525238279	0.927555656	0.313972412	0.469679958	0.696097429	0.275551486	-6.766888679	-6.177505701	-6.465484238	-6.849184812	-6.211398038	-6.389193555	0.60404583	0.627195152

MYCBP2	-0.039624141	-0.127573397	0.156729242	-0.087949256	0.196353383	0.284302638	8	0.109710872	0.816496581	0.816496581	0.816496581	0.816496581	0.816496581	0.816496581	9	125	0	P_309734	P_309734	P_309734	chr13	77048960	77049020	-	MYCBP2	"MYC binding protein 2, E3 ubiquitin protein ligase [Source:HGNC Symbol;Acc:HGNC:23386]"	ENSG00000005810	protein_coding	intron	intron sense	0	1485	13:77048960:77049020	ENSG00000005810	MYCBP2	9.773591743	0.01187263	-0.445381347	-1.4339443	1.761660414	-0.988562953	2.207041762	3.195604715	9.961963646	0.665566366	0.182221989	0.108726629	0.346279283	0.051918515	0.009607678	-6.891186635	-5.200739503	-5.573359933	-6.639948806	-4.203259195	-3.243480533	3.56969796	0.055034696

FANCD2	-0.25464125	-0.096747044	-0.254962319	0.157894206	-0.000321069	-0.158215274	8	0.042636891	0.816496581	0.816496581	0.816496581	0.816496581	0.816496581	0.816496581	9	127	0	P_243201	P_243201	P_243201	chr3	10088865	10088925	+	FANCD2	"Fanconi anemia, complementation group D2 [Source:HGNC Symbol;Acc:HGNC:3585]"	ENSG00000144554	protein_coding	exon	coding exon	1	1487	3:10088865:10088925	ENSG00000144554	FANCD2	8.384366035	0.00366656	-5.150447519	-1.956833682	-5.156941551	3.193613836	-0.006494032	-3.200107868	9.961963646	0.000436437	0.078959779	0.000432353	0.00964013	0.994946755	0.009534687	-0.000794302	-4.486821609	-0.028914358	-3.319418475	-6.296662826	-3.235772434	12.89837596	0.000912453

RPS6KA6	0.10150547	0.084366591	0.163300637	-0.017138879	0.061795167	0.078934046	8	0.06660279	0.816496581	0.816496581	0.816496581	0.816496581	0.816496581	0.816496581	9	128	0	P_270798	P_270798	P_270798	chrX	84145477	84145537	-	RPS6KA6	"ribosomal protein S6 kinase, 90kDa, polypeptide 6 [Source:HGNC Symbol;Acc:HGNC:10435]"	ENSG00000072133	protein_coding	exon	coding exon	1	1488	X:84145477:84145537	ENSG00000072133	RPS6KA6	7.219047952	0.005768979	1.636762238	1.360400094	2.63320111	-0.276362143	0.996438873	1.272801016	9.961963646	0.132839065	0.203688261	0.02509866	0.7879187	0.342624102	0.231990307	-5.705006307	-5.290581504	-4.179818238	-7.108005825	-5.794089758	-6.251816044	2.358786003	0.133222619

BRCA2	-0.39735929	-0.215305272	-0.305749578	0.182054018	0.091609712	-0.090444306	8	0.07273823	0.816496581	0.816496581	0.816496581	0.816496581	0.816496581	0.816496581	9	129	0	P_218650	P_218650	P_218650	chr13	32379441	32379501	+	BRCA2	"breast cancer 2, early onset [Source:HGNC Symbol;Acc:HGNC:1101]"	ENSG00000139618	protein_coding	exon	coding exon	1	1489	13:32379441:32379501	ENSG00000139618	BRCA2	9.386754013	0.006455525	-6.057078675	-3.281969253	-4.660641631	2.775109422	1.396437044	-1.378672378	9.961963646	0.000124403	0.008302622	0.000902583	0.019680789	0.192916147	0.198164544	1.301127704	-2.407215822	-0.793427065	-4.036147853	-5.352416927	-6.121646534	13.45551849	0.000773806

SLC35C2	-0.202229108	0.082164039	0.065753843	0.284393147	0.267982951	-0.016410196	8	0.074962768	0.816496581	0.816496581	0.816496581	0.816496581	0.816496581	0.816496581	9	130	0	P_131460	P_131460	P_131460	chr20	46349947	46350007	-	SLC35C2	"solute carrier family 35 (GDP-fucose transporter), member C2 [Source:HGNC Symbol;Acc:HGNC:17117]"	ENSG00000080189	protein_coding	3'UTR;exon	multiple Ref	1	1490	20:46349947:46350007	ENSG00000080189	SLC35C2	7.928751115	0.006719382	-3.021514163	1.227616582	0.982431114	4.249130745	4.003945276	-0.245185468	9.961963646	0.01291528	0.247808383	0.349144864	0.001706355	0.002521454	0.81128973	-3.474066046	-5.44454084	-6.544204509	-1.541754332	-1.325879211	-7.038636384	7.624761162	0.006140029

PDE1B	0.054090775	0.032428734	0.049150774	-0.021662041	-0.004940001	0.01672204	8	0.110254516	0.816496581	0.816496581	0.816496581	0.816496581	0.816496581	0.816496581	9	131	0	P_215617	P_215617	P_215617	chr12	54573420	54573480	+	PDE1B	"phosphodiesterase 1B, calmodulin-dependent [Source:HGNC Symbol;Acc:HGNC:8775]"	ENSG00000123360	protein_coding	3'UTR;exon	multiple Ref	1	1491	12:54573420:54573480	ENSG00000123360	PDE1B	5.053265748	0.011968662	0.605544469	0.363038624	0.550241317	-0.242505846	-0.055303152	0.187202693	9.961963646	0.558359894	0.724160755	0.594275681	0.813307944	0.956990371	0.855259439	-6.801887715	-6.113082406	-6.88425832	-7.11754747	-6.295058772	-7.052267236	0.149693171	0.927513958

PEBP4	0.467849141	-0.171620704	-0.048551927	-0.639469844	-0.516401068	0.123068776	8	0.108414871	0.816496581	0.816496581	0.816496581	0.816496581	0.816496581	0.816496581	9	134	0	P_264041	P_264041	P_264041	chr8	22713440	22713500	-	PEBP4	phosphatidylethanolamine-binding protein 4 [Source:HGNC Symbol;Acc:HGNC:28319]	ENSG00000134020	protein_coding	exon	coding exon	1	1494	8:22713440:22713500	ENSG00000134020	PEBP4	6.55061713	0.011645614	5.309703563	-1.947754058	-0.551024505	-7.257457621	-5.860728068	1.396729553	9.961963646	0.000347167	0.080156449	0.5937586	2.79E-05	0.00016169	0.192830723	0.236556786	-4.500126187	-6.883801317	2.754882983	1.288144276	-6.098734563	20.21000064	0.000147476

PCDH7	0.056849436	-0.303477564	-0.339636567	-0.360327001	-0.396486003	-0.036159002	8	0.107510852	0.816496581	0.816496581	0.816496581	0.816496581	0.816496581	0.816496581	9	135	0	P_137495	P_137495	P_137495	chr4	30724536	30724596	+	PCDH7	protocadherin 7 [Source:HGNC Symbol;Acc:HGNC:8659]	ENSG00000169851	protein_coding	exon	coding exon	1	1495	4:30724536:30724596	ENSG00000169851	PCDH7	4.847273828	0.011488857	0.649581108	-3.467638488	-3.880803618	-4.117219595	-4.530384726	-0.41316513	9.961963646	0.530654627	0.006079028	0.003076805	0.002103174	0.00110133	0.68823832	-6.772884201	-2.113081523	-2.060223346	-1.758391597	-0.529549014	-6.978917139	10.85894957	0.001755348

TUB	-0.006498138	-0.049250615	-0.065388401	-0.042752477	-0.058890263	-0.016137786	8	0.092693577	0.816496581	0.816496581	0.816496581	0.816496581	0.816496581	0.816496581	9	136	0	P_107877	P_107877	P_107877	chr11	8093944	8094004	+	TUB	tubby bipartite transcription factor [Source:HGNC Symbol;Acc:HGNC:12406]	ENSG00000166402	protein_coding	intron	intron sense	0	1496	11:8093944:8094004	ENSG00000166402	TUB	5.46144907	0.009106608	-0.083398118	-0.632090064	-0.839204921	-0.548691947	-0.755806803	-0.207114856	9.961963646	0.935187018	0.541561828	0.421036164	0.595299317	0.467260236	0.840092421	-6.994566229	-5.975748382	-6.675596992	-6.987425412	-6.001519151	-7.047994649	0.337660222	0.798631872

ZCCHC4	-0.195674686	-0.007845894	-0.15445881	0.187828792	0.041215876	-0.146612915	8	0.07181021	0.816496581	0.816496581	0.816496581	0.816496581	0.816496581	0.816496581	9	137	0	A_23_P155807	A_23_P155807	A_23_P155807	chr4	25333201	25333261	+	ZCCHC4	"zinc finger, CCHC domain containing 4 [Source:HGNC Symbol;Acc:HGNC:22917]"	ENSG00000168228	protein_coding	exon	coding exon	1	1497	4:25333201:25333261	ENSG00000168228	ZCCHC4	6.916745756	0.0063478	-3.007936393	-0.120608092	-2.374360649	2.887328301	0.633575744	-2.253752557	9.961963646	0.013217735	0.906399335	0.039075542	0.016241124	0.540630365	0.047970078	-3.497254068	-6.174424848	-4.612032703	-3.84453595	-6.087505033	-4.832760918	4.752883279	0.026208592

SRCAP	0.103292728	-0.128806564	-0.014417773	-0.232099292	-0.117710502	0.114388791	8	0.048905777	0.816496581	0.816496581	0.816496581	0.816496581	0.816496581	0.816496581	9	138	0	P_226105	P_226105	P_226105	chr16	30713641	30713701	+	SRCAP	Snf2-related CREBBP activator protein [Source:HGNC Symbol;Acc:HGNC:16974]	ENSG00000080603	protein_coding	exon	coding exon	1	1498	16:30713641:30713701	ENSG00000080603	SRCAP	6.717704921	0.00412741	1.969139961	-2.455527668	-0.274855878	-4.424667629	-2.243995839	2.18067179	9.961963646	0.077364593	0.034020075	0.789042993	0.001296672	0.048770035	0.054284191	-5.213898765	-3.725072732	-7.006021094	-1.256709823	-4.145776549	-4.950885758	6.55853408	0.010040437

FRMD5	0.206279765	-0.088843429	0.161360218	-0.295123194	-0.044919547	0.250203647	8	0.102012631	0.816496581	0.816496581	0.816496581	0.816496581	0.816496581	0.816496581	9	139	0	A_23_P26037	A_23_P26037	A_23_P26037	chr15	43873631	43873691	-	FRMD5	FERM domain containing 5 [Source:HGNC Symbol;Acc:HGNC:28214]	ENSG00000171877	protein_coding	3'UTR	3'UTR	1	1499	15:43873631:43873691	ENSG00000171877	FRMD5	5.842579046	0.010563733	2.458066086	-1.058673983	1.922796835	-3.516740069	-0.535269251	2.981470818	9.961963646	0.03387281	0.314742909	0.083533861	0.005601015	0.604206152	0.013828146	-4.427117756	-5.623466149	-5.332210271	-2.766702805	-6.14651455	-3.610062681	5.400528214	0.018190658

CCNI	0.187496866	-0.093058788	0.106438448	-0.280555654	-0.081058418	0.199497236	8	0.041104711	0.816496581	0.816496581	0.816496581	0.816496581	0.816496581	0.816496581	9	143	0	P_249525	P_249525	P_249525	chr4	77045574	77045634	-	CCNI	cyclin I [Source:HGNC Symbol;Acc:HGNC:1595]	ENSG00000118816	protein_coding	3'ext	3'extended sense	0	1503	4:77045574:77045634	ENSG00000118816	CCNI	12.60055797	0.003563523	3.846802395	-1.909252014	2.183757448	-5.756054409	-1.663044947	4.093009462	9.961963646	0.003251729	0.085422092	0.054002102	0.000186341	0.127397742	0.002186003	-2.074374331	-4.556253141	-4.922585299	0.768850064	-5.004208792	-1.729463267	12.64375597	0.000985654

STRIP1	-0.088603499	-0.037466375	-0.086314068	0.051137123	0.00228943	-0.048847693	8	0.07571935	0.816496581	0.816496581	0.816496581	0.816496581	0.816496581	0.816496581	9	144	0	P_203356	P_203356	P_203356	chr1	110049500	110049560	+	STRIP1	striatin interacting protein 1 [Source:HGNC Symbol;Acc:HGNC:25916]	ENSG00000143093	protein_coding	3'UTR;exon	multiple Ref	1	1504	1:110049500:110049560	ENSG00000143093	STRIP1	7.569827164	0.006810933	-1.314901445	-0.556011804	-1.280925639	0.758889641	0.033975806	-0.724913835	9.961963646	0.218006393	0.590471453	0.22923563	0.465495185	0.973567565	0.485183445	-6.128961607	-6.021693998	-6.218145497	-6.843485646	-6.296071308	-6.791663409	0.796908889	0.52330107

KAZALD1	0.042973137	0.12642713	-0.001637124	0.083453992	-0.044610261	-0.128064254	8	0.104044136	0.816496581	0.816496581	0.816496581	0.816496581	0.816496581	0.816496581	9	145	0	P_106890	P_106890	P_106890	chr10	101065055	101065115	+	KAZALD1	Kazal-type serine peptidase inhibitor domain 1 [Source:HGNC Symbol;Acc:HGNC:25460]	ENSG00000107821	protein_coding	3'UTR	3'UTR	1	1505	10:101065055:101065115	ENSG00000107821	KAZALD1	7.424161164	0.010899896	0.504117209	1.483114701	-0.019205073	0.978997492	-0.523322282	-1.502319774	9.961963646	0.625140445	0.168975253	0.985056721	0.350757078	0.612191574	0.164032207	-6.861434865	-5.138985448	-7.046919182	-6.649324274	-6.153051163	-5.960862777	0.990566536	0.436303166

MMP17	0.157789587	0.205932814	0.094297234	0.048143227	-0.063492353	-0.111635579	8	0.366730492	0.816496581	0.816496581	0.816496581	0.816496581	0.816496581	0.816496581	9	146	0	P_113276	P_113276	P_113276	chr12	131851711	131851771	+	MMP17	matrix metallopeptidase 17 (membrane-inserted) [Source:HGNC Symbol;Acc:HGNC:7163]	ENSG00000198598	protein_coding	3'UTR	3'UTR	1	1506	12:131851711:131851771	ENSG00000198598	MMP17	7.14098978	0.110210494	0.582119963	0.759730755	0.347882922	0.177610792	-0.234237041	-0.411847833	9.961963646	0.573423197	0.465014357	0.735166773	0.862588112	0.819544751	0.689172493	-6.816545666	-5.886651878	-6.98143015	-7.132389255	-6.267584972	-6.979500814	0.215518037	0.883392805

PRKAA1	0.073115075	-0.008299776	0.035165724	-0.081414851	-0.037949351	0.043465501	8	0.093646332	0.816496581	0.816496581	0.816496581	0.816496581	0.816496581	0.816496581	9	147	0	P_252631	P_252631	P_252631	chr5	40764764	40764824	-	PRKAA1	"protein kinase, AMP-activated, alpha 1 catalytic subunit [Source:HGNC Symbol;Acc:HGNC:9376]"	ENSG00000132356	protein_coding	exon	coding exon	1	1507	5:40764764:40764824	ENSG00000132356	PRKAA1	8.298528796	0.009249179	0.93110993	-0.105696454	0.447830425	-1.036806383	-0.483279505	0.553526879	9.961963646	0.37381102	0.917921206	0.663856849	0.324337582	0.639342935	0.592108065	-6.544887694	-6.176201437	-6.938692999	-6.591533827	-6.17393987	-6.906535768	0.448934682	0.723576888

SCEL	0.386597761	0.055233104	0.247711297	-0.331364657	-0.138886464	0.192478193	8	0.109739906	0.816496581	0.816496581	0.816496581	0.816496581	0.816496581	0.816496581	9	148	0	A_23_P500000	A_23_P500000	A_23_P500000	chr13	77644428	77644488	+	SCEL	sciellin [Source:HGNC Symbol;Acc:HGNC:10573]	ENSG00000136155	protein_coding	3'UTR	3'UTR	1	1508	13:77644428:77644488	ENSG00000136155	SCEL	5.186320331	0.011877747	4.344481313	0.620694716	2.783712712	-3.723786597	-1.560768601	2.163017996	9.961963646	0.001469163	0.548736974	0.019392879	0.003976512	0.149756164	0.055925535	-1.257064517	-5.982977122	-3.924823814	-2.415497485	-5.142085858	-4.979221279	7.998344728	0.005223877

PCGF5	0.075781892	0.058323261	0.194191043	-0.017458631	0.118409151	0.135867782	8	0.106726688	0.816496581	0.816496581	0.816496581	0.816496581	0.816496581	0.816496581	9	151	0	P_209000	P_209000	P_209000	chr10	91251380	91251440	+	PCGF5	polycomb group ring finger 5 [Source:HGNC Symbol;Acc:HGNC:28264]	ENSG00000180628	protein_coding	exon	coding exon	1	1511	10:91251380:91251440	ENSG00000180628	PCGF5	7.795528704	0.011353946	0.871039141	0.670369159	2.232037155	-0.200669982	1.360998014	1.561667996	9.961963646	0.404234898	0.517860701	0.049768153	0.844994273	0.203505479	0.149545293	-6.599469957	-5.950584139	-4.844696284	-7.127636256	-5.39578852	-5.880601461	1.753580804	0.219524293

SDCCAG8	-0.016010525	-0.039411234	-0.035415583	-0.023400709	-0.019405058	0.003995651	8	0.051802991	0.816496581	0.816496581	0.816496581	0.816496581	0.816496581	0.816496581	9	153	0	P_308353	P_308353	P_308353	chr1	243287303	243287363	-	SDCCAG8	serologically defined colon cancer antigen 8 [Source:HGNC Symbol;Acc:HGNC:10671]	ENSG00000054282	protein_coding	intron	intron antisense	0	1513	1:243287303:243287363	ENSG00000054282	SDCCAG8	7.290842732	0.004361721	-0.296908248	-0.73086426	-0.65676665	-0.433956012	-0.359858402	0.07409761	9.961963646	0.77263494	0.481697982	0.526211401	0.673568	0.726464861	0.942399471	-6.950468415	-5.908086741	-6.816554018	-7.047595456	-6.228265567	-7.068379902	0.229494309	0.873760441

ELSPBP1	-0.016375698	0.172279724	-0.047630178	0.188655422	-0.03125448	-0.219909902	8	0.122983334	0.816496581	0.816496581	0.816496581	0.816496581	0.816496581	0.816496581	9	154	0	P_126241	P_126241	P_126241	chr19	48024978	48025038	+	ELSPBP1	epididymal sperm binding protein 1 [Source:HGNC Symbol;Acc:HGNC:14417]	ENSG00000169393	protein_coding	3'UTR	3'UTR	1	1514	19:48024978:48025038	ENSG00000169393	ELSPBP1	4.210134632	0.014352804	-0.167408332	1.761211146	-0.486922071	1.928619478	-0.319513739	-2.248133217	9.961963646	0.870398309	0.108805727	0.636848964	0.082734193	0.755936586	0.048429245	-6.983088641	-4.76728264	-6.919169653	-5.422996619	-6.242671001	-4.841888989	2.040458073	0.172356509

HAO1	-0.203796949	0.173939576	-0.029690411	0.377736525	0.174106538	-0.203629987	8	0.211554842	0.816496581	0.816496581	0.816496581	0.816496581	0.816496581	0.816496581	10	21	0	P_240122	P_240122	P_240122	chr20	7940285	7940345	-	HAO1	hydroxyacid oxidase (glycolate oxidase) 1 [Source:HGNC Symbol;Acc:HGNC:4809]	ENSG00000101323	protein_coding	exon	coding exon	1	1551	20:7940285:7940345	ENSG00000101323	HAO1	3.90063799	0.038147752	-1.277935205	1.090710674	-0.186177576	2.368645878	1.091757628	-1.27688825	9.961963646	0.230246403	0.3010782	0.856042003	0.039458027	0.300639452	0.230601141	-6.173651746	-5.591094599	-7.02822438	-4.719719578	-5.69902705	-6.246927078	1.881715312	0.196813912

FGF9	0.175489914	-0.07302999	-0.022695815	-0.248519904	-0.198185729	0.050334175	8	0.193026248	0.816496581	0.816496581	0.816496581	0.816496581	0.816496581	0.816496581	10	22	0	P_218270	P_218270	P_218270	chr13	21672121	21672181	+	FGF9	fibroblast growth factor 9 [Source:HGNC Symbol;Acc:HGNC:3687]	ENSG00000102678	protein_coding	exon	coding exon	1	1552	13:21672121:21672181	ENSG00000102678	FGF9	3.880084983	0.032127799	1.19910525	-0.499006709	-0.155078263	-1.698111959	-1.354183514	0.343928446	9.961963646	0.258231278	0.628609081	0.879856706	0.120449832	0.205596832	0.738049002	-6.265861981	-6.052498855	-7.034002996	-5.765798715	-5.404042531	-7.007141374	1.091099059	0.397309383

CDKAL1	0.002064997	-0.073776646	0.008603252	-0.075841643	0.006538255	0.082379898	8	0.084741278	0.816496581	0.816496581	0.816496581	0.816496581	0.816496581	0.816496581	10	23	0	A_32_P128310	A_32_P128310	A_32_P128310	chr6	21000286	21000346	+	CDKAL1	CDK5 regulatory subunit associated protein 1-like 1 [Source:HGNC Symbol;Acc:HGNC:21050]	ENSG00000145996	protein_coding	exon	coding exon	1	1553	6:21000286:21000346	ENSG00000145996	CDKAL1	5.677809147	0.007973486	0.028323113	-1.011906573	0.118000585	-1.040229686	0.089677472	1.129907159	9.961963646	0.977963814	0.335528782	0.908412531	0.322821172	0.930320875	0.284986577	-6.997922582	-5.669347646	-7.039521837	-6.588027572	-6.292409582	-6.415415862	0.567581881	0.648856567

DOCK7	-0.027708963	0.040427114	-0.090780866	0.068136077	-0.063071903	-0.13120798	8	0.044307754	0.816496581	0.816496581	0.816496581	0.816496581	0.816496581	0.816496581	10	26	0	A_32_P230547	A_32_P230547	A_32_P230547	chr1	62473985	62474045	-	DOCK7	dedicator of cytokinesis 7 [Source:HGNC Symbol;Acc:HGNC:19190]	ENSG00000116641	protein_coding	exon	coding exon	1	1556	1:62473985:62474045	ENSG00000116641	DOCK7	7.754805553	0.003783222	-0.551740635	0.804984368	-1.807627845	1.356725003	-1.25588721	-2.612612213	9.961963646	0.593286001	0.439618322	0.100906129	0.204814754	0.237812056	0.025999897	-6.83474984	-5.851575959	-5.505628133	-6.226421418	-5.519900893	-4.237474634	2.410605663	0.127888016

LBX1	-0.139570703	0.00032418	-0.118036177	0.139894883	0.021534526	-0.118360357	8	0.179132241	0.816496581	0.816496581	0.816496581	0.816496581	0.816496581	0.816496581	10	27	0	A_23_P86489	A_23_P86489	A_23_P86489	chr10	101226977	101227200	-	LBX1;LBX1-AS1	ladybird homeobox 1 [Source:HGNC Symbol;Acc:HGNC:16960];LBX1 antisense RNA 1 (head to head) [Source:HGNC Symbol;Acc:HGNC:48678]	ENSG00000138136	protein_coding;processed_transcript	3'UTR;promoter	multiple non-Ref	0	1557	10:101226977:101227200	ENSG00000138136	LBX1	7.811584004	0.027975387	-1.022002239	0.002373797	-0.864316324	1.024376036	0.157685916	-0.86669012	9.961963646	0.330957037	0.998152849	0.407743958	0.329888872	0.877854667	0.406502533	-6.456562344	-6.182084454	-6.653833484	-6.60418709	-6.283477002	-6.675860427	0.602816633	0.627914971

FAM133B	-0.226631857	-0.221510838	-0.323250796	0.005121019	-0.096618939	-0.101739958	8	0.103492986	0.816496581	0.816496581	0.816496581	0.816496581	0.816496581	0.816496581	10	29	0	P_145217	P_145217	P_145217	chr7	92560969	92561029	-	FAM133B	"family with sequence similarity 133, member B [Source:HGNC Symbol;Acc:HGNC:28629]"	ENSG00000234545	protein_coding	3'UTR	3'UTR	1	1559	7:92560969:92561029	ENSG00000234545	FAM133B	7.163410392	0.010808039	-2.669888566	-2.609559232	-3.808130138	0.060329334	-1.138241572	-1.198570906	9.961963646	0.023569275	0.026136252	0.003463395	0.953086473	0.281653063	0.258429927	-4.072057075	-3.481477294	-2.181751486	-7.147623038	-5.650214144	-6.338613308	5.195960869	0.020358024

CSNK1D	0.078124485	-0.083227061	-0.024222828	-0.161351546	-0.102347313	0.059004233	8	0.061514749	0.816496581	0.816496581	0.816496581	0.816496581	0.816496581	0.816496581	10	30	0	P_230301	P_230301	P_230301	chr17	82253170	82253230	-	CSNK1D	"casein kinase 1, delta [Source:HGNC Symbol;Acc:HGNC:2452]"	ENSG00000141551	protein_coding	exon	coding exon	1	1560	17:82253170:82253230	ENSG00000141551	CSNK1D	9.476002107	0.005245494	1.321111889	-1.407398199	-0.409616351	-2.728510088	-1.73072824	0.997781848	9.961963646	0.216003915	0.18973698	0.690756219	0.021316536	0.114296748	0.342003687	-6.121365496	-5.233525148	-6.956259937	-4.11546501	-4.910337714	-6.553167991	2.554941337	0.114302693

STRBP	0.140945479	-0.146006676	0.209437645	-0.286952155	0.068492166	0.355444321	8	0.070619159	0.816496581	0.816496581	0.816496581	0.816496581	0.816496581	0.816496581	10	32	0	P_314223	P_314223	P_314223	chr9	123162728	123162788	+	STRBP	spermatid perinuclear RNA binding protein [Source:HGNC Symbol;Acc:HGNC:16462]	ENSG00000165209	protein_coding	intron	intron antisense	0	1562	9:123162728:123162788	ENSG00000165209	STRBP	9.480403014	0.00621157	2.190262158	-2.268912063	3.254615558	-4.459174221	1.0643534	5.523527622	9.961963646	0.05341206	0.046752308	0.008694996	0.001229161	0.312286584	0.000256784	-4.865362426	-4.016072187	-3.120273146	-1.201130447	-5.727061776	0.494630152	12.0106955	0.001200491

GNE	-0.114974829	0.151723977	-0.145003505	0.266698806	-0.030028676	-0.296727482	8	0.229455588	0.816496581	0.816496581	0.816496581	0.816496581	0.816496581	0.816496581	10	33	0	A_23_P216489	A_23_P216489	A_23_P216489	chr9	36214644	36214704	-	GNE	glucosamine (UDP-N-acetyl)-2-epimerase/N-acetylmannosamine kinase [Source:HGNC Symbol;Acc:HGNC:23657]	ENSG00000159921	protein_coding	3'UTR	3'UTR	1	1563	9:36214644:36214704	ENSG00000159921	GNE	3.824632291	0.044487398	-0.667620864	0.881011029	-0.84198747	1.548631894	-0.174366606	-1.7229985	9.961963646	0.519541494	0.399068527	0.419548919	0.152627432	0.865069963	0.115728625	-6.760463005	-5.788697989	-6.673212746	-5.975280661	-6.280539375	-5.653307237	1.22137154	0.352330763

MLLT3	0.052378491	0.017059024	0.077874821	-0.035319467	0.02549633	0.060815797	8	0.056912157	0.816496581	0.816496581	0.816496581	0.816496581	0.816496581	0.816496581	10	34	0	P_314293	P_314293	P_314293	chr9	20620855	20620915	+	MLLT3	"myeloid/lymphoid or mixed-lineage leukemia (trithorax homolog, Drosophila); translocated to, 3 [Source:HGNC Symbol;Acc:HGNC:7136]"	ENSG00000171843	protein_coding	intron	intron antisense	0	1564	9:20620855:20620915	ENSG00000171843	MLLT3	6.54617129	0.004807772	0.925180896	0.301319932	1.375532128	-0.623860964	0.450351232	1.074212196	9.961963646	0.376739359	0.769366198	0.199104748	0.546737892	0.662099374	0.30805741	-6.550411659	-6.134449425	-6.10171773	-6.940848247	-6.189929481	-6.475136472	0.7641321	0.539700004

BORCS7	0.275008312	-0.108784246	0.07677881	-0.383792558	-0.198229502	0.185563057	8	0.072063583	0.816496581	0.816496581	0.816496581	0.816496581	0.816496581	0.816496581	10	35	0	A_24_P201353	A_24_P201353	A_24_P201353	chr10	102864029	102864089	+	C10orf32	chromosome 10 open reading frame 32 [Source:HGNC Symbol;Acc:HGNC:23516]	ENSG00000166275	protein_coding	3'UTR	3'UTR	1	1565	10:102864029:102864089	ENSG00000166275	BORCS7	10.80173741	0.006377075	4.217748701	-1.668402714	1.177541596	-5.886151415	-3.040207105	2.84594431	9.961963646	0.001792999	0.126313374	0.266345372	0.000156247	0.012510367	0.017432746	-1.46248481	-4.895241743	-6.338508943	0.953246723	-2.865663957	-3.841585747	12.32476878	0.001087559

NHSL1	0.150514781	0.027819895	0.004219823	-0.122694886	-0.146294957	-0.023600072	8	0.095348134	0.816496581	0.816496581	0.816496581	0.816496581	0.816496581	0.816496581	10	36	0	P_143709	P_143709	P_143709	chr6	138571784	138571844	-	NHSL1	NHS-like 1 [Source:HGNC Symbol;Acc:HGNC:21021]	ENSG00000135540	protein_coding	exon	coding exon	1	1566	6:138571784:138571844	ENSG00000135540	NHSL1	5.504866198	0.009507467	1.890568289	0.349436854	0.05300386	-1.541131435	-1.837564429	-0.296432994	9.961963646	0.088092095	0.734035363	0.958776664	0.154425963	0.096094532	0.772987349	-5.333978581	-6.118124216	-7.045586573	-5.985489873	-4.758311443	-7.023585434	1.589794271	0.25309633

MTMR9	-0.397411204	0.248195192	-0.349695947	0.645606396	0.047715256	-0.59789114	8	0.099978762	0.816496581	0.816496581	0.816496581	0.816496581	0.816496581	0.816496581	10	37	0	A_24_P261052	A_24_P261052	A_24_P261052	chr8	11327335	11327395	+	MTMR9	myotubularin related protein 9 [Source:HGNC Symbol;Acc:HGNC:14596]	ENSG00000104643	protein_coding	3'UTR	3'UTR	1	1567	8:11327335:11327395	ENSG00000104643	MTMR9	7.831511786	0.010233819	-4.8113492	3.004831592	-4.233673587	7.816180792	0.577675613	-7.238505179	9.961963646	0.000719115	0.013287903	0.001748466	1.48E-05	0.576306174	2.85E-05	-0.518356413	-2.849811312	-1.477935931	3.419313	-6.122194651	2.781388582	27.3207391	4.02E-05

SMTN	-0.091205028	-0.086355296	-0.041508387	0.004849732	0.049696641	0.044846909	8	0.067037154	0.816496581	0.816496581	0.816496581	0.816496581	0.816496581	0.816496581	10	38	0	P_311871	P_311871	P_311871	chr22	31095611	31095671	-	SMTN	smoothelin [Source:HGNC Symbol;Acc:HGNC:11126]	ENSG00000183963	protein_coding	exon;intron;promoter	multiple non-Ref	0	1568	22:31095611:31095671	ENSG00000183963	SMTN	6.933052556	0.005815595	-1.464762907	-1.386875676	-0.666629324	0.077887231	0.798133583	0.720246352	9.961963646	0.173818754	0.195726132	0.520148696	0.939459958	0.443402972	0.487928449	-5.938936212	-5.258597309	-6.809728402	-7.146296557	-5.968601771	-6.795163554	0.945864602	0.454942684

CHEK2	-0.444330343	0.070048009	-0.272408413	0.514378352	0.17192193	-0.342456422	8	0.030621248	0.816496581	0.816496581	0.816496581	0.816496581	0.816496581	0.816496581	10	39	0	P_242327	P_242327	P_242327	chr22	28719394	28719454	-	CHEK2	checkpoint kinase 2 [Source:HGNC Symbol;Acc:HGNC:16627]	ENSG00000183765	protein_coding	"3'UTR;5'UTR,exon;exon"	multiple Ref	1	1569	22:28719394:28719454	ENSG00000183765	CHEK2	9.023161551	0.002959677	-10.0029814	1.576954951	-6.132591055	11.57993635	3.870390345	-7.709546006	9.961963646	1.63E-06	0.146000955	0.000112628	4.23E-07	0.003129306	1.66E-05	5.733817867	-5.017619308	1.370714097	7.063889544	-1.533939365	3.341430784	58.11117397	1.29E-06

MAMDC4	-0.241313841	0.105503044	0.002193253	0.346816885	0.243507094	-0.103309791	8	0.182062191	0.816496581	0.816496581	0.816496581	0.816496581	0.816496581	0.816496581	10	40	0	P_150212	P_150212	P_150212	chr9	136857452	136857512	+	MAMDC4	MAM domain containing 4 [Source:HGNC Symbol;Acc:HGNC:24083]	ENSG00000177943	protein_coding	exon	coding exon	1	1570	9:136857452:136857512	ENSG00000177943	MAMDC4	8.765264413	0.028825245	-1.74076987	0.761069152	0.015821511	2.501839022	1.756591381	-0.745247642	9.961963646	0.112460655	0.464249914	0.98768917	0.031430023	0.109622142	0.47333823	-5.556345759	-5.885640175	-7.046983902	-4.498170009	-4.873952492	-6.776178541	2.251660356	0.145101644

RAPGEF6	-0.046573447	-0.13791258	-0.176761784	-0.091339133	-0.130188337	-0.038849205	8	0.075433697	0.816496581	0.816496581	0.816496581	0.816496581	0.816496581	0.816496581	10	42	0	P_254682	P_254682	P_254682	chr5	131433547	131433607	-	RAPGEF6;CTC-432M15.3	Rap guanine nucleotide exchange factor (GEF) 6 [Source:HGNC Symbol;Acc:HGNC:20655];Uncharacterized protein [Source:UniProtKB/TrEMBL;Acc:E9PCH4]	ENSG00000158987	protein_coding	exon	coding exon	1	1572	5:131433547:131433607	ENSG00000158987	RAPGEF6	7.364124973	0.006776259	-0.692929434	-2.051892057	-2.629898607	-1.358962623	-1.936969173	-0.57800655	9.961963646	0.504186992	0.067401269	0.025241095	0.204128254	0.081599952	0.576091228	-6.742514077	-4.346057526	-4.185389117	-6.223620404	-4.613050614	-6.89187802	2.92324991	0.086669143

DIS3	0.080049231	0.029449465	-0.011748943	-0.050599767	-0.091798174	-0.041198407	8	0.069230896	0.816496581	0.816496581	0.816496581	0.816496581	0.816496581	0.816496581	10	43	0	P_219339	P_219339	P_219339	chr13	72761362	72761422	-	DIS3	DIS3 exosome endoribonuclease and 3'-5' exoribonuclease [Source:HGNC Symbol;Acc:HGNC:20604]	ENSG00000083520	protein_coding	3'UTR;exon	multiple Ref	1	1573	13:72761362:72761422	ENSG00000083520	DIS3	7.073647366	0.006055658	1.259859566	0.463492142	-0.184911432	-0.796367424	-1.444770998	-0.648403574	9.961963646	0.236434151	0.65297272	0.857008789	0.444382151	0.179230984	0.531384859	-6.195172425	-6.070091935	-7.028480081	-6.813446224	-5.292088023	-6.846445441	0.829709139	0.507391802

INPP5B	-0.002578743	0.061410549	0.059084072	0.063989292	0.061662814	-0.002326478	8	0.071975956	0.816496581	0.816496581	0.816496581	0.816496581	0.816496581	0.816496581	10	44	0	P_201172	P_201172	P_201172	chr1	37886910	37886970	-	INPP5B	"inositol polyphosphate-5-phosphatase, 75kDa [Source:HGNC Symbol;Acc:HGNC:6077]"	ENSG00000204084	protein_coding	exon	coding exon	1	1574	1:37886910:37886970	ENSG00000204084	INPP5B	6.095823997	0.006366939	-0.03958114	0.942590974	0.906881853	0.982172115	0.946462993	-0.035709122	9.961963646	0.969209065	0.368186946	0.385880156	0.349266284	0.366304001	0.972219699	-6.997505468	-5.734252639	-6.615689736	-6.646221014	-5.841115233	-7.070681956	0.595701418	0.632095939

KRTAP4-7	-0.150007158	0.277918804	-0.163198728	0.427925962	-0.01319157	-0.441117532	8	0.644968849	0.816496581	0.816496581	0.816496581	0.816496581	0.816496581	0.816496581	10	45	0	A_23_P66844	A_23_P66844	A_23_P66844	chr17	41084877	41084937	+	KRTAP4-7	keratin associated protein 4-7 [Source:HGNC Symbol;Acc:HGNC:18898]	ENSG00000240871	protein_coding	exon	coding exon	1	1575	17:41084877:41084937	ENSG00000240871	KRTAP4-7	3.253446225	0.336265174	-0.316822974	0.586979071	-0.344684262	0.903802045	-0.027861288	-0.931663332	9.961963646	0.757917513	0.570280247	0.737497793	0.38743388	0.978323024	0.373538528	-6.943861837	-6.003652101	-6.982625445	-6.720376915	-6.296272396	-6.616858582	0.374893237	0.773097047

SKAP2	0.24061059	-0.142340671	0.079519713	-0.382951262	-0.161090878	0.221860384	8	0.053616905	0.816496581	0.816496581	0.816496581	0.816496581	0.816496581	0.816496581	10	46	0	P_144465	P_144465	P_144465	chr7	26667104	26667164	-	SKAP2	src kinase associated phosphoprotein 2 [Source:HGNC Symbol;Acc:HGNC:15687]	ENSG00000005020	protein_coding	3'UTR	3'UTR	1	1576	7:26667104:26667164	ENSG00000005020	SKAP2	10.36359472	0.004515283	4.385487516	-2.594371413	1.449365576	-6.979858929	-2.93612194	4.043736989	9.961963646	0.00137811	0.026825232	0.177974472	3.88E-05	0.014941452	0.00236535	-1.191026854	-3.505598229	-6.006968348	2.41037333	-3.035076222	-1.810801477	16.95916265	0.000305947

HESX1	0.150972088	0.031092046	0.036715381	-0.119880042	-0.114256708	0.005623334	8	0.136860775	0.816496581	0.816496581	0.816496581	0.816496581	0.816496581	0.816496581	10	47	0	P_244632	P_244632	P_244632	chr3	57199788	57199848	-	HESX1	HESX homeobox 1 [Source:HGNC Symbol;Acc:HGNC:4877]	ENSG00000163666	protein_coding	exon	coding exon	1	1577	3:57199788:57199848	ENSG00000163666	HESX1	5.819041969	0.017248595	1.407879306	0.289946631	0.34238663	-1.117932675	-1.065492677	0.052439999	9.961963646	0.189598467	0.77780248	0.739173916	0.28982988	0.311795613	0.959214758	-6.012678972	-6.137962339	-6.983477385	-6.505985501	-5.72590744	-7.069876425	0.761869976	0.540850478

PATZ1	0.246572878	-0.118060379	0.137296145	-0.364633257	-0.109276733	0.255356524	8	0.07868853	0.816496581	0.816496581	0.816496581	0.816496581	0.816496581	0.816496581	10	48	0	P_133103	P_133103	P_133103	chr22	31343294	31343354	-	PATZ1	POZ (BTB) and AT hook containing zinc finger 1 [Source:HGNC Symbol;Acc:HGNC:13071]	ENSG00000100105	protein_coding	exon	non-coding exon	1	1578	22:31343294:31343354	ENSG00000100105	PATZ1	8.296779001	0.007179105	3.564148392	-1.706532834	1.984580938	-5.270681226	-1.579567454	3.691113772	9.961963646	0.005176388	0.11883315	0.075405913	0.000367066	0.145402716	0.004196058	-2.549555115	-4.843107152	-5.237166769	0.059475713	-5.117121061	-2.400077987	10.5755674	0.001936213

VIT	-0.093547341	-0.018671843	0.041657485	0.074875498	0.135204826	0.060329327	8	0.238356112	0.816496581	0.816496581	0.816496581	0.816496581	0.816496581	0.816496581	10	49	0	P_127602	P_127602	P_127602	chr2	36814272	36814332	-	VIT	vitrin [Source:HGNC Symbol;Acc:HGNC:12697]	ENSG00000205221	protein_coding	exon	exon antisense	0	1579	2:36814272:36814332	ENSG00000205221	VIT	3.703753933	0.047831131	-0.523867855	-0.104562867	0.233283136	0.419304988	0.757150991	0.337846003	9.961963646	0.611825736	0.918797954	0.8202651	0.683891464	0.466490106	0.742490516	-6.850644146	-6.176326942	-7.017482209	-7.054308472	-6.000498167	-7.009380507	0.20050436	0.893666551

SERINC2	-0.015532238	0.066504912	0.082680996	0.082037151	0.098213235	0.016176084	8	0.105207951	0.816496581	0.816496581	0.816496581	0.816496581	0.816496581	0.816496581	10	50	0	P_200900	P_200900	P_200900	chr1	31426674	31426734	+	SERINC2	serine incorporator 2 [Source:HGNC Symbol;Acc:HGNC:23231]	ENSG00000168528	protein_coding	exon	coding exon	1	1580	1:31426674:31426734	ENSG00000168528	SERINC2	6.82952607	0.011095464	-0.180595506	0.773261916	0.961343505	0.953857422	1.141939012	0.188081589	9.961963646	0.860306145	0.457323175	0.359132372	0.362727443	0.280184021	0.854588631	-6.980592018	-5.876351125	-6.564636124	-6.673605775	-5.646264604	-7.052087685	0.633995573	0.609882229

TOP3B	-0.110650501	-0.051711355	-0.131153647	0.058939146	-0.020503146	-0.079442292	8	0.080242994	0.816496581	0.816496581	0.816496581	0.816496581	0.816496581	0.816496581	10	51	0	P_132753	P_132753	P_132753	chr22	21959516	21959576	-	TOP3B	topoisomerase (DNA) III beta [Source:HGNC Symbol;Acc:HGNC:11993]	ENSG00000100038	protein_coding	3'UTR	3'UTR	1	1581	22:21959516:21959576	ENSG00000100038	TOP3B	6.521658166	0.007377502	-1.577772326	-0.737355408	-1.870127952	0.840416918	-0.292355626	-1.132772543	9.961963646	0.14581355	0.477913805	0.09110194	0.420387924	0.776013005	0.283837045	-5.786997377	-5.903331182	-5.412150436	-6.776517856	-6.251424309	-6.41228004	1.434230518	0.2905291

LINC01013	0.062209099	-0.13619942	0.14170089	-0.198408518	0.079491791	0.277900309	8	0.367524514	0.816496581	0.816496581	0.816496581	0.816496581	0.816496581	0.816496581	10	52	0	A_24_P469141	A_24_P469141	A_24_P469141	chr6	132146566	132146626	+	LINC01013	long intergenic non-protein coding RNA 1013 [Source:HGNC Symbol;Acc:HGNC:48987]	ENSG00000228495	lincRNA	exon	non-coding exon	1	1582	6:132146566:132146626	ENSG00000228495	LINC01013	3.412654574	0.110678687	0.229016904	-0.501405262	0.521658404	-0.730422166	0.2926415	1.023063666	9.961963646	0.82348892	0.626979929	0.613307995	0.481956396	0.77580074	0.330479095	-6.96981514	-6.051266173	-6.900517343	-6.865440815	-6.251336142	-6.527870196	0.373633054	0.773955431

TH	-0.045469254	0.094168385	0.189052515	0.139637639	0.234521769	0.09488413	8	0.276813091	0.816496581	0.816496581	0.816496581	0.816496581	0.816496581	0.816496581	10	53	0	A_24_P924602	A_24_P924602	A_24_P924602	chr11	2170691	2170751	-	TH	tyrosine hydroxylase [Source:HGNC Symbol;Acc:HGNC:11782]	ENSG00000180176	protein_coding	exon	coding exon	1	1583	11:2170691:2170751	ENSG00000180176	TH	5.662344562	0.063741128	-0.220573628	0.456815548	0.917103211	0.677389176	1.137676839	0.460287663	9.961963646	0.829879274	0.657602382	0.380755179	0.51358222	0.28187797	0.655192847	-6.971876053	-6.073260914	-6.606298662	-6.904321435	-5.650816523	-6.95685825	0.510573482	0.683976329

CNST	0.260465382	0.030203305	0.215441361	-0.230262077	-0.045024022	0.185238056	8	0.046298055	0.816496581	0.816496581	0.816496581	0.816496581	0.816496581	0.816496581	10	54	0	P_207122	P_207122	P_207122	chr1	246591881	246591941	+	CNST	"consortin, connexin sorting protein [Source:HGNC Symbol;Acc:HGNC:26486]"	ENSG00000162852	protein_coding	exon	coding exon	1	1584	1:246591881:246591941	ENSG00000162852	CNST	7.012710281	0.003928039	5.089882171	0.590217643	4.210045613	-4.499664528	-0.879836558	3.61982797	9.961963646	0.000476575	0.568190769	0.001814968	0.001154658	0.399674618	0.004720024	-0.092024675	-6.001712662	-1.516486685	-1.136108397	-5.900664767	-2.520537731	13.01733154	0.000880499

RAB2A	-0.032725779	0.128114489	0.057850754	0.160840268	0.090576533	-0.070263735	8	0.087233648	0.816496581	0.816496581	0.816496581	0.816496581	0.816496581	0.816496581	10	55	0	P_264821	P_264821	P_264821	chr8	60584741	60584801	-	RAB2A	"RAB2A, member RAS oncogene family [Source:HGNC Symbol;Acc:HGNC:9763]"	ENSG00000104388	protein_coding	3'UTR;exon	multiple non-Ref	0	1585	8:60584741:60584801	ENSG00000104388	RAB2A	8.990606188	0.008317696	-0.439474935	1.720451236	0.776878576	2.15992617	1.216353511	-0.943572659	9.961963646	0.669697454	0.116204041	0.455281502	0.056217858	0.251884545	0.367708899	-6.893983191	-4.823922596	-6.727190752	-5.059791235	-5.564742452	-6.60565448	1.798626214	0.211211245

CCDC138	-0.090163082	0.004281035	-0.055453947	0.094444117	0.034709135	-0.059734982	8	0.09583379	0.816496581	0.816496581	0.816496581	0.816496581	0.816496581	0.816496581	10	56	0	P_236132	P_236132	P_236132	chr2	108794661	108794721	+	CCDC138	coiled-coil domain containing 138 [Source:HGNC Symbol;Acc:HGNC:26531]	ENSG00000163006	protein_coding	3'UTR;exon	multiple Ref	1	1586	2:108794661:108794721	ENSG00000163006	CCDC138	7.271800863	0.009582029	-1.128094885	0.053563097	-0.693824041	1.181657982	0.434270844	-0.747387138	9.961963646	0.285715499	0.958342177	0.503649342	0.264780943	0.673346931	0.472102656	-6.345154933	-6.180575193	-6.790427652	-6.435297666	-6.197344985	-6.77452696	0.65005886	0.600775768

E4F1	-0.044637908	0.023734915	-0.024355223	0.068372823	0.020282685	-0.048090138	8	0.157173736	0.816496581	0.816496581	0.816496581	0.816496581	0.816496581	0.816496581	10	57	0	A_23_P15045	A_23_P15045	A_23_P15045	chr16	2235438	2235498	+	E4F1	E4F transcription factor 1 [Source:HGNC Symbol;Acc:HGNC:3121]	ENSG00000167967	protein_coding	3'UTR;exon	multiple Ref	1	1587	16:2235438:2235498	ENSG00000167967	E4F1	9.6622417	0.022045008	-0.368208935	0.195784441	-0.200901235	0.563993376	0.167307701	-0.396685676	9.961963646	0.720420978	0.84871477	0.844818266	0.585231003	0.870475418	0.69996401	-6.924879556	-6.161920518	-7.025123742	-6.978396952	-6.28181833	-6.986089111	0.119618432	0.946472166

ECT2L	0.007791409	0.080178657	0.233805756	0.072387249	0.226014348	0.153627099	8	0.16072513	0.816496581	0.816496581	0.816496581	0.816496581	0.816496581	0.816496581	10	58	0	P_259470	P_259470	P_259470	chr6	138876498	138876558	+	ECT2L	epithelial cell transforming 2 like [Source:HGNC Symbol;Acc:HGNC:21118]	ENSG00000203734	protein_coding	exon	coding exon	1	1588	6:138876498:138876558	ENSG00000203734	ECT2L	3.946187269	0.022951644	0.062987549	0.648182809	1.890139803	0.58519526	1.827152254	1.241956993	9.961963646	0.951022324	0.531521829	0.088154229	0.571432941	0.097743519	0.242695911	-6.996195756	-5.965334116	-5.381898391	-6.965502716	-4.773322621	-6.288344779	1.536688814	0.265219626

DOP1B	0.199345197	0.048877221	0.269784296	-0.150467976	0.070439099	0.220907075	8	0.085530482	0.816496581	0.816496581	0.816496581	0.816496581	0.816496581	0.816496581	10	59	0	P_241743	P_241743	P_241743	chr21	36246617	36246677	+	DOPEY2	dopey family member 2 [Source:HGNC Symbol;Acc:HGNC:1291]	ENSG00000142197	protein_coding	exon	coding exon	1	1589	21:36246617:36246677	ENSG00000142197	DOP1B	7.55121859	0.0080814	2.715864545	0.665899724	3.675521729	-2.049964821	0.959657184	3.009622004	9.961963646	0.021783502	0.52059576	0.004305245	0.067618818	0.359939935	0.013179798	-3.994322077	-5.953591927	-2.404719731	-5.234523603	-5.828893325	-3.56189526	5.918320822	0.013826436

TGDS	-0.172357236	0.101722387	-0.047369596	0.274079623	0.124987641	-0.149091983	8	0.153677695	0.816496581	0.816496581	0.816496581	0.816496581	0.816496581	0.816496581	10	61	0	P_219564	P_219564	P_219564	chr13	94578004	94578064	-	TGDS	"TDP-glucose 4,6-dehydratase [Source:HGNC Symbol;Acc:HGNC:20324]"	ENSG00000088451	protein_coding	exon	coding exon	1	1591	13:94578004:94578064	ENSG00000088451	TGDS	6.887230176	0.021172289	-1.450745524	0.856205989	-0.398713917	2.306951513	1.052031606	-1.254919907	9.961963646	0.17759857	0.41200511	0.698516295	0.043827216	0.317634302	0.238148579	-5.957284555	-5.809748393	-6.960994447	-4.821243509	-5.73948409	-6.273071797	1.833390636	0.205043846

COL4A5	-0.071510206	0.056937937	-0.164442499	0.128448144	-0.092932293	-0.221380437	8	0.090637566	0.816496581	0.816496581	0.816496581	0.816496581	0.816496581	0.816496581	10	62	0	P_314449	P_314449	P_314449	chrX	108689421	108689481	-	COL4A5	"collagen, type IV, alpha 5 [Source:HGNC Symbol;Acc:HGNC:2207]"	ENSG00000188153	protein_coding	intron;promoter	multiple non-Ref	0	1592	X:108689421:108689481	ENSG00000188153	COL4A5	5.953932796	0.008803912	-0.933417228	0.743206521	-2.146455305	1.676623749	-1.213038077	-2.889661826	9.961963646	0.372675861	0.474518906	0.057508693	0.124665636	0.253094662	0.016176437	-6.54273003	-5.899012398	-4.982360633	-5.796587539	-5.568455563	-3.766985574	3.110594678	0.075678994

SERINC3	0.208609565	-0.056617197	0.1451204	-0.265226762	-0.063489165	0.201737597	8	0.077488264	0.816496581	0.816496581	0.816496581	0.816496581	0.816496581	0.816496581	10	63	0	P_131338	P_131338	P_131338	chr20	44500116	44500176	-	SERINC3	serine incorporator 3 [Source:HGNC Symbol;Acc:HGNC:11699]	ENSG00000132824	protein_coding	3'UTR	3'UTR	1	1593	20:44500116:44500176	ENSG00000132824	SERINC3	10.69419829	0.00702857	3.047518668	-0.827104764	2.120023254	-3.874623431	-0.927495414	2.947128017	9.961963646	0.012355529	0.427545108	0.060124547	0.003107852	0.37559428	0.014663162	-3.429647791	-5.833786246	-5.024492069	-2.161790752	-5.858438751	-3.668796323	6.504081491	0.010309487

RAB1AP1	-0.07555289	0.135983572	0.088838126	0.211536462	0.164391016	-0.047145446	8	0.093865084	0.816496581	0.816496581	0.816496581	0.816496581	0.816496581	0.816496581	10	65	0	A_24_P251351	A_24_P251351	A_24_P251351	chr2	190993364	190993424	-	AC067945.3	NA	ENSG00000229023	processed_pseudogene	exon	non-coding exon	1	1595	2:190993364:190993424	ENSG00000229023	RAB1AP1	8.943715104	0.00928212	-0.960446398	1.728655677	1.129331496	2.689102075	2.089777894	-0.599324181	9.961963646	0.359561823	0.114679088	0.285217959	0.022805836	0.063257336	0.562338145	-6.517122217	-4.812576156	-6.392033438	-4.182399511	-4.383595527	-6.878631252	2.857288075	0.090981306

DYNC1H1	0.024190697	-0.135861039	-0.089473798	-0.160051736	-0.113664495	0.046387241	8	0.067111642	0.816496581	0.816496581	0.816496581	0.816496581	0.816496581	0.816496581	10	68	0	P_222291	P_222291	P_222291	chr14	102050070	102050130	-	DYNC1H1;RP11-1017G21.4	"dynein, cytoplasmic 1, heavy chain 1 [Source:HGNC Symbol;Acc:HGNC:2961];"	ENSG00000197102	protein_coding;antisense	exon;intron	multiple non-Ref	0	1598	14:102050070:102050130	ENSG00000197102	DYNC1H1	9.217593976	0.00582362	0.388237526	-2.180439623	-1.435968809	-2.568677149	-1.824206335	0.744470814	9.961963646	0.706007638	0.054305473	0.181659464	0.028032185	0.098214782	0.473787362	-6.916730092	-4.151879294	-6.024402624	-4.385959622	-4.777562379	-6.776777165	2.907853264	0.087653655

TREH	-0.040146324	0.071660846	0.01481561	0.111807171	0.054961934	-0.056845237	8	0.181830226	0.816496581	0.816496581	0.816496581	0.816496581	0.816496581	0.816496581	10	69	0	P_213569	P_213569	P_213569	chr11	118661170	118661230	-	TREH	trehalase (brush-border membrane glycoprotein) [Source:HGNC Symbol;Acc:HGNC:12266]	ENSG00000118094	protein_coding	3'UTR;exon	multiple Ref	1	1599	11:118661170:118661230	ENSG00000118094	TREH	4.361230817	0.028757458	-0.289945364	0.517550001	0.10700151	0.807495366	0.396946874	-0.410548491	9.961963646	0.777803421	0.616069071	0.916911982	0.438236528	0.699777501	0.69009447	-6.952678954	-6.042823281	-7.040871627	-6.804281386	-6.213555263	-6.980074768	0.223590215	0.877836322

LRRC1	-0.14787694	-0.015196321	-0.041419609	0.132680619	0.106457332	-0.026223287	8	0.07605574	0.816496581	0.816496581	0.816496581	0.816496581	0.816496581	0.816496581	10	72	0	P_313539	P_313539	P_313539	chr6	53924141	53924201	-	LRRC1;RP3-523E19.2	leucine rich repeat containing 1 [Source:HGNC Symbol;Acc:HGNC:14307];	ENSG00000137269	protein_coding;antisense	3'ext;promoter	multiple non-Ref	0	1602	6:53924141:53924201	ENSG00000137269	LRRC1	6.067884153	0.006851933	-2.187960741	-0.224842052	-0.612837115	1.963118689	1.575123625	-0.387995063	9.961963646	0.053620109	0.826647027	0.553716024	0.078141273	0.146421607	0.70618141	-4.869060421	-6.155507028	-6.845818026	-5.369917266	-5.123038253	-6.989757453	1.949780845	0.185863241

FANCM	-0.322985475	-0.005119744	-0.196476334	0.317865731	0.126509141	-0.19135659	8	0.059768863	0.816496581	0.816496581	0.816496581	0.816496581	0.816496581	0.816496581	10	73	0	P_220607	P_220607	P_220607	chr14	45185246	45185306	+	FANCM	"Fanconi anemia, complementation group M [Source:HGNC Symbol;Acc:HGNC:23168]"	ENSG00000187790	protein_coding	exon	coding exon	1	1603	14:45185246:45185306	ENSG00000187790	FANCM	7.984545804	0.005075449	-5.552535859	-0.088014986	-3.377680964	5.464520872	2.174854895	-3.289665977	9.961963646	0.000246613	0.931608923	0.007067382	0.000278888	0.054819827	0.008195533	0.591355893	-6.17800527	-2.910140491	0.346834167	-4.253083564	-3.082557879	14.61000237	0.000559006

OVCH1	-0.192608209	-0.062204419	0.013956644	0.13040379	0.206564853	0.076161063	8	0.098563526	0.816496581	0.816496581	0.816496581	0.816496581	0.816496581	0.816496581	10	74	0	P_214892	P_214892	P_214892	chr12	29439371	29439431	-	OVCH1	ovochymase 1 [Source:HGNC Symbol;Acc:HGNC:23080]	ENSG00000187950	protein_coding	exon	coding exon	1	1604	12:29439371:29439431	ENSG00000187950	OVCH1	3.534811618	0.010008173	-2.357995766	-0.761534295	0.170863473	1.596461472	2.528859239	0.932397768	9.961963646	0.040180626	0.463984431	0.867751642	0.141586244	0.030009883	0.373177117	-4.592550051	-5.885288202	-7.031200771	-5.909477074	-3.694561108	-6.616171086	2.663742787	0.1051775

NOL7	-0.101464241	-0.017349891	-0.10051024	0.08411435	0.000954001	-0.083160349	8	0.064804076	0.816496581	0.816496581	0.816496581	0.816496581	0.816496581	0.816496581	10	76	0	P_256218	P_256218	P_256218	chr6	13620269	13620329	+	NOL7	"nucleolar protein 7, 27kDa [Source:HGNC Symbol;Acc:HGNC:21040]"	ENSG00000225921	protein_coding	exon	coding exon	1	1606	6:13620269:13620329	ENSG00000225921	NOL7	12.65497203	0.005579166	-1.663696055	-0.284483922	-1.648053428	1.379212133	0.015642627	-1.363569506	9.961963646	0.127265518	0.781865387	0.130476444	0.198003318	0.987828347	0.202720957	-5.666996755	-6.139602421	-5.736742109	-6.198129383	-6.29655511	-6.140654741	1.554448061	0.261093031

SLC44A3	0.347803575	0.125864781	0.473253325	-0.221938794	0.125449749	0.347388544	8	0.110115085	0.816496581	0.816496581	0.816496581	0.816496581	0.816496581	0.816496581	10	77	0	P_202988	P_202988	P_202988	chr1	94845327	94845387	+	SLC44A3	"solute carrier family 44, member 3 [Source:HGNC Symbol;Acc:HGNC:28689]"	ENSG00000143036	protein_coding	3'UTR;exon	multiple Ref	1	1607	1:94845327:94845387	ENSG00000143036	SLC44A3	6.898011294	0.011943987	3.897669543	1.410506842	5.303525327	-2.487162701	1.405855784	3.893018484	9.961963646	0.002993717	0.188843505	0.000350239	0.032229023	0.19018163	0.003016393	-1.989606827	-5.229706361	0.190161418	-4.522723951	-5.340765829	-2.061207552	11.43779	0.001445018

RUNDC3A	0.255659384	0.226377562	0.189440068	-0.029281822	-0.066219316	-0.036937494	8	0.108640756	0.816496581	0.816496581	0.816496581	0.816496581	0.816496581	0.816496581	10	78	0	P_121719	P_121719	P_121719	chr17	44317554	44317614	+	RUNDC3A	RUN domain containing 3A [Source:HGNC Symbol;Acc:HGNC:16984]	ENSG00000108309	protein_coding	3'UTR	3'UTR	1	1608	17:44317554:44317614	ENSG00000108309	RUNDC3A	5.979952905	0.011684987	2.896631495	2.564867229	2.146363879	-0.331764266	-0.750267616	-0.41850335	9.961963646	0.015984782	0.028215685	0.05751755	0.746941454	0.47044241	0.684458319	-3.687161774	-3.552399273	-4.982506688	-7.089752906	-6.005709462	-6.97653343	3.404001651	0.061605518

C11orf42	-0.064439289	-0.1564396	-0.061101864	-0.092000311	0.003337426	0.095337737	8	0.206912837	0.816496581	0.816496581	0.816496581	0.816496581	0.816496581	0.816496581	10	79	0	A_32_P511713	A_32_P511713	A_32_P511713	chr11	6210330	6210390	+	C11orf42	chromosome 11 open reading frame 42 [Source:HGNC Symbol;Acc:HGNC:28541]	ENSG00000180878	protein_coding	exon	coding exon	1	1609	11:6210330:6210390	ENSG00000180878	C11orf42	6.310217067	0.036587795	-0.41259908	-1.001668947	-0.391229838	-0.589069867	0.021369242	0.610439109	9.961963646	0.688639673	0.340212646	0.70386452	0.568930812	0.983373069	0.55524063	-6.906254006	-5.679169438	-6.964172898	-6.963098298	-6.29644238	-6.871547517	0.341122554	0.796243686

ZNF569	0.221099882	-0.044620346	0.143879198	-0.265720227	-0.077220684	0.188499543	8	0.081202945	0.816496581	0.816496581	0.816496581	0.816496581	0.816496581	0.816496581	10	82	0	P_232823	P_232823	P_232823	chr19	37425884	37425944	-	ZNF569	zinc finger protein 569 [Source:HGNC Symbol;Acc:HGNC:24737]	ENSG00000196437	protein_coding	exon	coding exon	1	1612	19:37425884:37425944	ENSG00000196437	ZNF569	7.933972149	0.00750196	3.126416032	-0.630944543	2.034493312	-3.757360575	-1.091922721	2.665437855	9.961963646	0.010803954	0.542280658	0.06938956	0.003763353	0.300570312	0.023749735	-3.294850903	-5.976480526	-5.159450954	-2.358853905	-5.698856473	-4.148270692	6.121057323	0.012466766

CDC42BPB	0.014340901	-0.122324446	-0.071241421	-0.136665347	-0.085582322	0.051083025	8	0.04191304	0.816496581	0.816496581	0.816496581	0.816496581	0.816496581	0.816496581	10	83	0	P_115841	P_115841	P_115841	chr14	102949827	102949887	+	CDC42BPB	CDC42 binding protein kinase beta (DMPK-like) [Source:HGNC Symbol;Acc:HGNC:1738]	ENSG00000198752	protein_coding	exon;promoter	multiple non-Ref	0	1613	14:102949827:102949887	ENSG00000198752	CDC42BPB	7.792687588	0.003617412	0.292027032	-2.490920625	-1.450705329	-2.782947657	-1.742732361	1.040215296	9.961963646	0.776257014	0.032022546	0.177609509	0.019418309	0.112104967	0.322827535	-6.952023479	-3.669324225	-6.005219034	-4.022790092	-4.893484264	-6.510416619	3.376412151	0.062788092

FZR1	-0.157028822	0.169728658	0.050239922	0.32675748	0.207268744	-0.119488736	8	0.070626844	0.816496581	0.816496581	0.816496581	0.816496581	0.816496581	0.816496581	10	84	0	A_23_P142325	A_23_P142325	A_23_P142325	chr19	3534458	3534518	+	FZR1	fizzy/cell division cycle 20 related 1 (Drosophila) [Source:HGNC Symbol;Acc:HGNC:24824]	ENSG00000105325	protein_coding	exon	coding exon	1	1614	19:3534458:3534518	ENSG00000105325	FZR1	6.850047325	0.006212441	-2.440022632	2.637361471	0.780662715	5.077384103	3.220685347	-1.856698756	9.961963646	0.034933373	0.024920365	0.453151647	0.000485338	0.00920832	0.093131046	-4.45707337	-3.437274977	-6.724158087	-0.232442543	-2.572336866	-5.45595902	8.853132327	0.003676271

ABL1	-0.110778257	-0.037803714	-0.007311299	0.072974543	0.103466958	0.030492415	8	0.086141309	0.816496581	0.816496581	0.816496581	0.816496581	0.816496581	0.816496581	10	86	0	A_24_P282416	A_24_P282416	A_24_P282416	chr9	130714192	130714252	+	ABL1	"ABL proto-oncogene 1, non-receptor tyrosine kinase [Source:HGNC Symbol;Acc:HGNC:76]"	ENSG00000097007	protein_coding	5'UTR	5'UTR	1	1616	9:130714192:130714252	ENSG00000097007	ABL1	7.481039259	0.00816561	-1.501432635	-0.512372479	-0.099093663	0.989060157	1.402338972	0.413278816	9.961963646	0.164257742	0.619557622	0.923029572	0.346047687	0.191198777	0.688157725	-5.890405243	-6.045558457	-7.041760704	-6.639459432	-5.345122192	-6.978866683	0.940373173	0.45729005

HABP2	-0.063966339	-0.173682012	0.15811696	-0.109715673	0.222083299	0.331798972	8	0.129902745	0.816496581	0.816496581	0.816496581	0.816496581	0.816496581	0.816496581	10	87	0	P_209926	P_209926	P_209926	chr10	113574325	113574385	+	HABP2	hyaluronan binding protein 2 [Source:HGNC Symbol;Acc:HGNC:4798]	ENSG00000148702	protein_coding	exon	coding exon	1	1617	10:113574325:113574385	ENSG00000148702	HABP2	4.124477733	0.015758007	-0.624088939	-1.69453222	1.542671457	-1.070443281	2.166760395	3.237203676	9.961963646	0.546594116	0.12114309	0.154055171	0.309668984	0.055573654	0.00895464	-6.789903059	-4.859582745	-5.882700706	-6.55668191	-4.265576489	-3.172288144	3.660158124	0.051799743

ZSCAN32	0.136315556	0.057485696	0.093071164	-0.07882986	-0.043244392	0.035585468	8	0.146992521	0.816496581	0.816496581	0.816496581	0.816496581	0.816496581	0.816496581	10	90	0	P_225375	P_225375	P_225375	chr16	3384649	3384709	-	ZSCAN32	zinc finger and SCAN domain containing 32 [Source:HGNC Symbol;Acc:HGNC:20812]	ENSG00000140987	protein_coding	3'UTR;exon	multiple Ref	1	1620	16:3384649:3384709	ENSG00000140987	ZSCAN32	7.051174271	0.019558124	1.193788682	0.503433179	0.815074269	-0.690355503	-0.378714413	0.31164109	9.961963646	0.260213232	0.625604161	0.434083419	0.505735851	0.712846059	0.761737561	-6.271924905	-6.050219597	-6.695985479	-6.895060519	-6.220961652	-7.018580215	0.510009657	0.684331104

KCNAB1	0.036510403	0.016219566	0.128801149	-0.020290836	0.092290746	0.112581583	8	0.139775042	0.816496581	0.816496581	0.816496581	0.816496581	0.816496581	0.816496581	10	91	0	A_23_P44494	A_23_P44494	A_23_P44494	chr3	156536809	156536869	+	KCNAB1	"potassium voltage-gated channel, shaker-related subfamily, beta member 1 [Source:HGNC Symbol;Acc:HGNC:6228]"	ENSG00000169282	protein_coding	3'UTR;exon	multiple Ref	1	1621	3:156536809:156536869	ENSG00000169282	KCNAB1	3.771508609	0.01789601	0.334259795	0.148493266	1.17919942	-0.18576653	0.844939625	1.030706155	9.961963646	0.7451139	0.884916298	0.265714435	0.856355837	0.417974932	0.327053057	-6.937732485	-6.17047654	-6.336638213	-7.130773943	-5.930380126	-6.52012204	0.555846414	0.655962599

CATSPER1	0.258010238	0.001998973	0.163040401	-0.256011265	-0.094969837	0.161041428	8	0.353255885	0.816496581	0.816496581	0.816496581	0.816496581	0.816496581	0.816496581	10	92	0	P_109179	P_109179	P_109179	chr11	66016754	66016814	-	CATSPER1	"cation channel, sperm associated 1 [Source:HGNC Symbol;Acc:HGNC:17116]"	ENSG00000175294	protein_coding	3'UTR;exon	multiple Ref	1	1622	11:66016754:66016814	ENSG00000175294	CATSPER1	3.927945177	0.102419634	0.987395084	0.007649992	0.623949233	-0.979745092	-0.363445851	0.616299241	9.961963646	0.346823723	0.994047277	0.546682221	0.350405588	0.723865923	0.551519061	-6.490988847	-6.182056573	-6.838592197	-6.648594223	-6.226903899	-6.867764157	0.472689717	0.708124613

B4GALT1-AS1	0.052805525	0.227942436	-0.123202242	0.175136911	-0.176007767	-0.351144678	8	0.152389456	0.816496581	0.816496581	0.816496581	0.816496581	0.816496581	0.816496581	10	93	0	A_32_P171903	A_32_P171903	A_32_P171903	chr9	33178920	33179347	+	B4GALT1-AS1	B4GALT1 antisense RNA 1 [Source:HGNC Symbol;Acc:HGNC:49910]	ENSG00000233554	antisense	"exon,intron"	multiple non-Ref	0	1623	9:33178920:33179347	ENSG00000233554	B4GALT1-AS1	5.908468108	0.020855655	0.447829891	1.93312037	-1.044846092	1.485290479	-1.492675983	-2.977966462	9.961963646	0.663857221	0.082120963	0.320784729	0.168408831	0.16649842	0.013911093	-6.890016788	-4.521514738	-6.481617223	-6.060528712	-5.231006449	-3.616057407	3.055277164	0.078742864

NFAT5	0.097110292	-0.373683165	-0.096140025	-0.470793457	-0.193250317	0.27754314	8	0.135404514	0.816496581	0.816496581	0.816496581	0.816496581	0.816496581	0.816496581	10	94	0	P_119420	P_119420	P_119420	chr16	69694179	69694239	+	NFAT5	"nuclear factor of activated T-cells 5, tonicity-responsive [Source:HGNC Symbol;Acc:HGNC:7774]"	ENSG00000102908	protein_coding	3'UTR;exon	multiple Ref	1	1624	16:69694179:69694239	ENSG00000102908	NFAT5	7.732805986	0.016930193	0.914071018	-3.517371263	-0.904938173	-4.431442282	-1.819009191	2.61243309	9.961963646	0.382270455	0.005595131	0.386860205	0.001283115	0.099051286	0.026007878	-6.560682558	-2.034766442	-6.617465457	-1.245785968	-4.785034124	-4.237776589	7.299603552	0.007097959

DLAT	-0.204159906	0.057256894	-0.175535442	0.2614168	0.028624463	-0.232792336	8	0.031059669	0.816496581	0.816496581	0.816496581	0.816496581	0.816496581	0.816496581	10	96	0	P_213341	P_213341	P_213341	chr11	112045902	112045962	+	DLAT	dihydrolipoamide S-acetyltransferase [Source:HGNC Symbol;Acc:HGNC:2896]	ENSG00000150768	protein_coding	3'UTR;exon	multiple Ref	1	1626	11:112045902:112045962	ENSG00000150768	DLAT	9.417196629	0.002981393	-4.579377696	1.284292052	-3.937321024	5.863669748	0.642056672	-5.221613077	9.961963646	0.001021612	0.228102143	0.002807502	0.00016105	0.535330933	0.000393837	-0.881734693	-5.380180287	-1.966065168	0.921554153	-6.081984065	0.049031681	16.69711764	0.000326084

AP5B1	0.119018766	0.110525316	0.206364785	-0.00849345	0.087346019	0.095839469	8	0.143050254	0.816496581	0.816496581	0.816496581	0.816496581	0.816496581	0.816496581	10	97	0	A_24_P9883	A_24_P9883	A_24_P9883	chr11	65776568	65776628	-	AP5B1	"adaptor-related protein complex 5, beta 1 subunit [Source:HGNC Symbol;Acc:HGNC:25104]"	ENSG00000254470	protein_coding	3'UTR	3'UTR	1	1627	11:65776568:65776628	ENSG00000254470	AP5B1	7.565116666	0.01863989	1.0676758	0.991484027	1.851226446	-0.076191774	0.783550646	0.85974242	9.961963646	0.310856441	0.344920287	0.093969836	0.940774966	0.451530593	0.410143333	-6.409667591	-5.68886045	-5.440583138	-7.146439332	-5.980121214	-6.681953939	1.151487562	0.37571896

SLC7A9	-0.207374167	-0.133410722	-0.373664817	0.073963446	-0.166290649	-0.240254095	8	0.213329046	0.816496581	0.816496581	0.816496581	0.816496581	0.816496581	0.816496581	10	98	0	P_232684	P_232684	P_232684	chr19	32858462	32858522	-	SLC7A9	"solute carrier family 7 (amino acid transporter light chain, bo,+ system), member 9 [Source:HGNC Symbol;Acc:HGNC:11067]"	ENSG00000021488	protein_coding	3'UTR;exon	multiple Ref	1	1628	19:32858462:32858522	ENSG00000021488	SLC7A9	4.581973065	0.038753119	-1.290170048	-0.830009444	-2.324740639	0.460160604	-1.034570591	-1.494731195	9.961963646	0.226134057	0.42597646	0.042521153	0.655280949	0.325330854	0.165970155	-6.158960622	-5.831419182	-4.693628826	-7.035036129	-5.756890734	-5.970985881	1.879029818	0.197261091

LAMA3	0.283257014	-0.152229497	0.302529654	-0.435486511	0.01927264	0.454759151	8	0.055664048	0.816496581	0.816496581	0.816496581	0.816496581	0.816496581	0.816496581	10	99	0	P_230944	P_230944	P_230944	chr18	23916631	23916691	+	LAMA3	"laminin, alpha 3 [Source:HGNC Symbol;Acc:HGNC:6483]"	ENSG00000053747	protein_coding	exon	coding exon	1	1629	18:23916631:23916691	ENSG00000053747	LAMA3	6.730584906	0.004694937	5.063040463	-2.721006246	5.407526742	-7.784046709	0.344486279	8.128532989	9.961963646	0.000495608	0.021592408	0.000302184	1.53E-05	0.737642164	1.05E-05	-0.132626079	-3.30397258	0.343708734	3.382114159	-6.233951244	3.817259518	31.51045028	2.14E-05

ARIH2	-0.332218146	-0.023637144	-0.318098534	0.308581002	0.014119611	-0.294461391	8	0.084383779	0.816496581	0.816496581	0.816496581	0.816496581	0.816496581	0.816496581	10	100	0	P_134794	P_134794	P_134794	chr3	48979526	48979586	+	ARIH2	ariadne RBR E3 ubiquitin protein ligase 2 [Source:HGNC Symbol;Acc:HGNC:690]	ENSG00000177479	protein_coding	3'UTR;exon	multiple Ref	1	1630	3:48979526:48979586	ENSG00000177479	ARIH2	9.033434766	0.007924932	-4.570578927	-0.32519425	-4.376324642	4.245384678	0.194254286	-4.051130392	9.961963646	0.001035465	0.75176073	0.001397926	0.001716461	0.849880839	0.002337481	-0.895662559	-6.126644449	-1.246637353	-1.547878378	-6.276653838	-1.798579553	12.43680727	0.001050387

EEF1AKNMT	-0.300194128	-0.006060151	-0.298095767	0.294133977	0.002098361	-0.292035616	8	0.068510901	0.816496581	0.816496581	0.816496581	0.816496581	0.816496581	0.816496581	10	106	0	P_104038	P_104038	P_104038	chr1	171784439	171784499	+	METTL13	methyltransferase like 13 [Source:HGNC Symbol;Acc:HGNC:24248]	ENSG00000010165	protein_coding	exon	coding exon	1	1636	1:171784439:171784499	ENSG00000010165	EEF1AKNMT	10.47244352	0.005976016	-4.75600112	-0.096011491	-4.722756614	4.659989629	0.033244506	-4.626745123	9.961963646	0.000781408	0.925415423	0.000821563	0.000903477	0.974136267	0.000950337	-0.604382224	-6.177230188	-0.695855339	-0.880760173	-6.296097451	-0.867078927	14.67609565	0.000549039

FSIP1	0.146942237	0.170649376	0.240571862	0.023707139	0.093629625	0.069922486	8	0.081108108	0.816496581	0.816496581	0.816496581	0.816496581	0.816496581	0.816496581	10	108	0	P_222767	P_222767	P_222767	chr15	39776416	39776476	-	FSIP1	fibrous sheath interacting protein 1 [Source:HGNC Symbol;Acc:HGNC:21674]	ENSG00000150667	protein_coding	exon	coding exon	1	1638	15:39776416:39776476	ENSG00000150667	FSIP1	5.966231412	0.007489598	2.079519564	2.415021873	3.404561584	0.335502309	1.32504202	0.989539711	9.961963646	0.064354798	0.036457037	0.006755762	0.744204587	0.214744616	0.34582442	-5.041713936	-3.788680271	-2.864318825	-7.088404036	-5.439021307	-6.56130369	4.099209806	0.038973018

KRT18P2	-0.270982825	0.011509607	-0.2391104	0.282492432	0.031872425	-0.250620008	8	0.090477022	0.816496581	0.816496581	0.816496581	0.816496581	0.816496581	0.816496581	10	109	0	A_24_P16230	A_24_P16230	A_24_P16230	chr21	20425774	20425834	-	KRT18P2	keratin 18 pseudogene 2 [Source:HGNC Symbol;Acc:HGNC:6435]	ENSG00000234439	processed_pseudogene	exon	non-coding exon	1	1639	21:20425774:20425834	ENSG00000234439	KRT18P2	12.25884926	0.008780562	-3.541818029	0.150433647	-3.125236908	3.692251676	0.416581121	-3.275670555	9.961963646	0.005372074	0.883424828	0.010825613	0.004188203	0.685818406	0.00839133	-2.587355976	-6.170171477	-3.341574108	-2.468785015	-6.205202929	-3.106488321	7.811748469	0.005659319

SLC45A3	-0.590422304	0.11622367	-0.561861503	0.706645974	0.028560801	-0.678085173	8	0.089540772	0.816496581	0.816496581	0.816496581	0.816496581	0.816496581	0.816496581	10	111	0	P_205886	P_205886	P_205886	chr1	205658275	205658335	+	SLC45A3	"solute carrier family 45, member 3 [Source:HGNC Symbol;Acc:HGNC:8642]"	ENSG00000158715	protein_coding	3'UTR;exon	multiple non-Ref	0	1641	1:205658275:205658335	ENSG00000158715	SLC45A3	7.957300642	0.008645214	-7.777150802	1.530919479	-7.400942697	9.308070281	0.376208105	-8.931862177	9.961963646	1.54E-05	0.156904556	2.36E-05	3.14E-06	0.714650284	4.55E-06	3.457279049	-5.077716215	2.991689125	5.021516551	-6.221953356	4.674932329	47.36026812	3.34E-06

CASR	-0.009217731	-0.149325953	0.177120332	-0.140108222	0.186338063	0.326446285	8	0.118664489	0.816496581	0.816496581	0.816496581	0.816496581	0.816496581	0.816496581	10	112	0	P_245849	P_245849	P_245849	chr3	122262290	122262350	+	CASR	calcium-sensing receptor [Source:HGNC Symbol;Acc:HGNC:1514]	ENSG00000036828	protein_coding	exon	coding exon	1	1642	3:122262290:122262350	ENSG00000036828	CASR	4.224177329	0.013514704	-0.097110544	-1.573177215	1.865996271	-1.47606667	1.963106815	3.439173485	9.961963646	0.924564576	0.146869868	0.091721815	0.170821416	0.078142812	0.006375294	-6.993216644	-5.02259024	-5.418377334	-6.072755634	-4.574302064	-2.827359517	3.971122422	0.042279065

DYNC2LI1	0.301897371	0.237540144	0.398024705	-0.064357226	0.096127334	0.16048456	8	0.05789305	0.816496581	0.816496581	0.816496581	0.816496581	0.816496581	0.816496581	10	113	0	P_234701	P_234701	P_234701	chr2	43789650	43789710	+	DYNC2LI1	"dynein, cytoplasmic 2, light intermediate chain 1 [Source:HGNC Symbol;Acc:HGNC:24595]"	ENSG00000138036	protein_coding	"3'UTR;3'UTR,exon;exon"	multiple Ref	1	1643	2:43789650:43789710	ENSG00000138036	DYNC2LI1	6.940617396	0.004898206	5.283071024	4.156847907	6.965257029	-1.126223117	1.682186005	2.808409122	9.961963646	0.000360619	0.001974645	3.95E-05	0.286469879	0.123561797	0.018589736	0.197121128	-1.05241704	2.458942607	-6.49695956	-4.977865034	-3.905553204	17.61505325	0.000261737

COL6A3	-0.056016979	0.19539328	0.125609207	0.251410259	0.181626185	-0.069784073	8	0.076235594	0.816496581	0.816496581	0.816496581	0.816496581	0.816496581	0.816496581	10	115	0	P_239802	P_239802	P_239802	chr2	237333449	237333509	-	COL6A3	"collagen, type VI, alpha 3 [Source:HGNC Symbol;Acc:HGNC:2213]"	ENSG00000163359	protein_coding	exon	coding exon	1	1645	2:237333449:237333509	ENSG00000163359	COL6A3	3.89696414	0.006873929	-0.827490061	2.886374838	1.855515468	3.713864899	2.683005529	-1.03085937	9.961963646	0.427336807	0.016267629	0.09331183	0.004041886	0.023045362	0.326984646	-6.637085564	-3.039513279	-5.434143512	-2.43225448	-3.446140307	-6.519966231	5.752136559	0.015074904

ZNF850	-0.39305935	-0.242036132	-0.564087183	0.151023218	-0.171027832	-0.32205105	8	0.18418222	0.816496581	0.816496581	0.816496581	0.816496581	0.816496581	0.816496581	10	116	0	A_24_P153207	A_24_P153207	A_24_P153207	chr19	36749136	36749196	-	ZNF850	zinc finger protein 850 [Source:HGNC Symbol;Acc:HGNC:27994]	ENSG00000267041	protein_coding	exon	coding exon	1	1646	19:36749136:36749196	ENSG00000267041	ZNF850	8.128452331	0.029448775	-2.805240775	-1.727397216	-4.025856057	1.077843559	-1.220615282	-2.298458841	9.961963646	0.01869087	0.114911811	0.002434203	0.306510728	0.250335873	0.044464434	-3.84271195	-4.814318365	-1.819222423	-6.548895744	-5.55995861	-4.759887669	5.832559231	0.014454598

CUL4A	-0.136138613	-0.027521067	-0.088142808	0.108617546	0.047995805	-0.060621741	8	0.017465128	0.816496581	0.816496581	0.816496581	0.816496581	0.816496581	0.816496581	10	118	0	P_114208	P_114208	P_114208	chr13	113230156	113230216	+	CUL4A	cullin 4A [Source:HGNC Symbol;Acc:HGNC:2554]	ENSG00000139842	protein_coding	"3'ext,3'UTR;intron"	multiple non-Ref	0	1648	13:113230156:113230216	ENSG00000139842	CUL4A	8.956838506	0.00245164	-3.367430002	-0.680741956	-2.180239161	2.686688046	1.187190841	-1.499497205	9.961963646	0.007190094	0.511546333	0.054323855	0.022900381	0.262689724	0.164750715	-2.883560891	-5.943533106	-4.928238644	-4.18649512	-5.597146932	-5.964631825	4.572107308	0.029160554

PAK5	0.215187823	-0.088241637	0.019070063	-0.30342946	-0.19611776	0.1073117	8	0.217994217	0.816496581	0.816496581	0.816496581	0.816496581	0.816496581	0.816496581	10	119	0	P_240175	P_240175	P_240175	chr20	9557612	9557672	-	PAK7	p21 protein (Cdc42/Rac)-activated kinase 7 [Source:HGNC Symbol;Acc:HGNC:15916]	ENSG00000101349	protein_coding	exon	coding exon	1	1649	20:9557612:9557672	ENSG00000101349	PAK5	4.408709461	0.040369022	1.311714154	-0.53789198	0.116244826	-1.849606134	-1.195469328	0.654136806	9.961963646	0.219040067	0.602460364	0.909768497	0.094219534	0.259585396	0.527835028	-6.132850294	-6.031825797	-7.039746129	-5.542925076	-5.588007195	-6.842533561	1.216919899	0.353771554

DTL	-0.419026794	-0.086361027	-0.313628614	0.332665767	0.10539818	-0.227267587	8	0.045670799	0.816496581	0.816496581	0.816496581	0.816496581	0.816496581	0.816496581	10	121	0	P_206073	P_206073	P_206073	chr1	212068643	212068703	+	DTL	denticleless E3 ubiquitin protein ligase homolog (Drosophila) [Source:HGNC Symbol;Acc:HGNC:30288]	ENSG00000143476	protein_coding	exon	coding exon	1	1651	1:212068643:212068703	ENSG00000143476	DTL	9.632402233	0.003881713	-8.237126385	-1.697663984	-6.165234698	6.539462401	2.071891687	-4.467570714	9.961963646	9.33E-06	0.120536391	0.000107915	6.68E-05	0.065182574	0.001213304	3.970890265	-4.855289063	1.415180388	1.843238678	-4.410799063	-1.120487493	29.29315412	2.95E-05

MTG1	-0.246617525	-0.144535301	-0.284346022	0.102082225	-0.037728497	-0.139810721	8	0.086913986	0.816496581	0.816496581	0.816496581	0.816496581	0.816496581	0.816496581	10	122	0	P_107448	P_107448	P_107448	chr10	133420422	133420482	+	MTG1;RP11-108K14.8	mitochondrial ribosome-associated GTPase 1 [Source:HGNC Symbol;Acc:HGNC:32159];Mitochondrial GTPase 1 [Source:UniProtKB/TrEMBL;Acc:E9PI62]	ENSG00000148824	protein_coding	3'UTR;exon	multiple Ref	1	1652	10:133420422:133420482	ENSG00000148824	MTG1	9.478097653	0.008272991	-3.320766178	-1.946203693	-3.828789747	1.374562485	-0.508023569	-1.882586054	9.961963646	0.00777709	0.080362475	0.003348584	0.199395823	0.622495545	0.089256311	-2.963067605	-4.502395399	-2.147153125	-6.204005252	-6.161217919	-5.416910014	5.861077996	0.014241995

RGS11	0.228843399	-0.074625877	-0.000509563	-0.303469276	-0.229352963	0.074116313	8	0.124301546	0.816496581	0.816496581	0.816496581	0.816496581	0.816496581	0.816496581	10	125	0	P_117702	P_117702	P_117702	chr16	270522	270582	-	RGS11	regulator of G-protein signaling 11 [Source:HGNC Symbol;Acc:HGNC:9993]	ENSG00000076344	protein_coding	3'UTR;exon	multiple Ref	1	1655	16:270522:270582	ENSG00000076344	RGS11	6.060825018	0.014614578	2.318413374	-0.756035048	-0.005162389	-3.074448421	-2.323575763	0.750872659	9.961963646	0.042981292	0.467129409	0.995982943	0.011801881	0.042605505	0.470094151	-4.65747543	-5.889437294	-7.047106048	-3.523868407	-4.021060201	-6.771827265	3.560283897	0.055384963

SULT4A1	0.284613682	-0.037000877	-0.072068189	-0.321614558	-0.356681871	-0.035067312	8	0.205989316	0.816496581	0.816496581	0.816496581	0.816496581	0.816496581	0.816496581	10	126	0	P_133622	P_133622	P_133622	chr22	43824506	43824566	-	SULT4A1	"sulfotransferase family 4A, member 1 [Source:HGNC Symbol;Acc:HGNC:14903]"	ENSG00000130540	protein_coding	3'UTR	3'UTR	1	1656	22:43824506:43824566	ENSG00000130540	SULT4A1	4.271022287	0.036281571	1.830030638	-0.237911044	-0.463389507	-2.067941682	-2.293420146	-0.225478463	9.961963646	0.097285074	0.816771948	0.653043773	0.065615199	0.044846744	0.826165396	-5.424936486	-6.152336612	-6.931109645	-5.206198383	-4.068461185	-7.043675294	2.201223823	0.15112192

ZC3H8	0.14530297	0.00330925	-0.138288779	-0.141993721	-0.283591749	-0.141598029	8	0.113243646	0.816496581	0.816496581	0.816496581	0.816496581	0.816496581	0.816496581	10	128	0	A_23_P108871	A_23_P108871	A_23_P108871	chr2	112216055	112216115	-	ZC3H8	zinc finger CCCH-type containing 8 [Source:HGNC Symbol;Acc:HGNC:30941]	ENSG00000144161	protein_coding	3'UTR;exon	multiple Ref	1	1658	2:112216055:112216115	ENSG00000144161	ZC3H8	8.036687103	0.012505154	1.591386226	0.036243541	-1.514565448	-1.555142684	-3.105951673	-1.550808989	9.961963646	0.142723339	0.97180414	0.160946472	0.151081164	0.011186217	0.152108846	-5.768231967	-6.181394982	-5.920644118	-5.966394071	-2.758708698	-5.895429156	3.216357549	0.070205315

LRRC40	0.012988096	0.028950682	-0.00722405	0.015962586	-0.020212146	-0.036174732	8	0.032613394	0.816496581	0.816496581	0.816496581	0.816496581	0.816496581	0.816496581	10	129	0	P_202234	P_202234	P_202234	chr1	70173632	70173692	-	LRRC40	leucine rich repeat containing 40 [Source:HGNC Symbol;Acc:HGNC:26004]	ENSG00000066557	protein_coding	exon	coding exon	1	1659	1:70173632:70173692	ENSG00000066557	LRRC40	7.760754323	0.00306084	0.287521824	0.64089095	-0.159921219	0.353369126	-0.447443043	-0.800812169	9.961963646	0.779605079	0.536057554	0.876139219	0.731175283	0.664127116	0.441920655	-6.953436268	-5.970082734	-7.033171975	-7.08175298	-6.191289764	-6.731925381	0.247559992	0.86124113

WHAMM	-0.231251095	-0.023693531	-0.418499822	0.207557564	-0.187248727	-0.394806291	8	0.131429259	0.816496581	0.816496581	0.816496581	0.816496581	0.816496581	0.816496581	10	130	0	A_24_P767222	A_24_P767222	A_24_P767222	chr15	82808587	82808647	-	WHAMM;FSD2	"WAS protein homolog associated with actin, golgi membranes and microtubules [Source:HGNC Symbol;Acc:HGNC:30493];fibronectin type III and SPRY domain containing 2 [Source:HGNC Symbol;Acc:HGNC:18024]"	ENSG00000156232	protein_coding	promoter	multiple non-Ref	0	1660	15:82808587:82808647	ENSG00000156232	WHAMM	6.004054605	0.016078367	-2.233615729	-0.228851861	-4.042219932	2.004763868	-1.808604203	-3.813368071	9.961963646	0.049635276	0.823613706	0.002371111	0.072916082	0.100745765	0.003433902	-4.795457092	-6.154553119	-1.792172698	-5.305299208	-4.799962194	-2.194452976	7.202142958	0.007419296

CLEC4M	0.089629858	0.038575713	0.00657949	-0.051054146	-0.083050368	-0.031996223	8	0.25099424	0.816496581	0.816496581	0.816496581	0.816496581	0.816496581	0.816496581	10	131	0	P_124417	P_124417	P_124417	chr19	7765208	7765268	+	CLEC4M	"C-type lectin domain family 4, member M [Source:HGNC Symbol;Acc:HGNC:13523]"	ENSG00000104938	protein_coding	exon	coding exon	1	1661	19:7765208:7765268	ENSG00000104938	CLEC4M	5.234431133	0.0527976	0.477739607	0.205613912	0.035069598	-0.272125695	-0.442670009	-0.170544315	9.961963646	0.643145007	0.841233379	0.972716997	0.791082125	0.667461295	0.867996049	-6.875230963	-6.159849276	-7.046448996	-7.109266967	-6.193503984	-7.055512404	0.095139431	0.960978232

CCT6B	0.196674131	0.052615709	0.117211804	-0.144058422	-0.079462327	0.064596095	8	0.058167095	0.816496581	0.816496581	0.816496581	0.816496581	0.816496581	0.816496581	10	132	0	P_228396	P_228396	P_228396	chr17	34939616	34939676	-	CCT6B	"chaperonin containing TCP1, subunit 6B (zeta 2) [Source:HGNC Symbol;Acc:HGNC:1621]"	ENSG00000132141	protein_coding	exon	coding exon	1	1662	17:34939616:34939676	ENSG00000132141	CCT6B	9.065292952	0.004923747	3.432772194	0.918360447	2.045827883	-2.514411746	-1.386944311	1.127467436	9.961963646	0.006443953	0.380128153	0.068088055	0.030761137	0.195705838	0.285968207	-2.7723865	-5.756043528	-5.14169206	-4.477110116	-5.364107219	-6.41808102	4.388297723	0.032578152

DRC1	-0.071615715	-0.043403881	0.193756922	0.028211834	0.265372636	0.237160802	8	0.187999436	0.816496581	0.816496581	0.816496581	0.816496581	0.816496581	0.816496581	10	133	0	P_234019	P_234019	P_234019	chr2	26431928	26431988	+	DRC1	dynein regulatory complex subunit 1 [Source:HGNC Symbol;Acc:HGNC:24245]	ENSG00000157856	protein_coding	3'UTR;exon	multiple Ref	1	1663	2:26431928:26431988	ENSG00000157856	DRC1	4.010056448	0.030589673	-0.501494928	-0.30393924	1.356798764	0.197555687	1.858293692	1.660738004	9.961963646	0.626919067	0.767427714	0.204792093	0.847365453	0.092887884	0.127867217	-6.862837948	-6.1336216	-6.125227248	-7.128311771	-4.728307135	-5.742524217	1.405852254	0.298016439

SNORD116-29	-0.235658299	0.261233526	0.255764392	0.496891825	0.491422691	-0.005469134	8	0.287330278	0.816496581	0.816496581	0.816496581	0.816496581	0.816496581	0.816496581	10	134	0	P_116030	P_116030	P_116030	chr15	25106544	25106604	+	SNORD116-29	"small nucleolar RNA, C/D box 116-29 [Source:HGNC Symbol;Acc:HGNC:33348]"	ENSG00000207245	snoRNA	exon	non-coding exon	1	1664	15:25106544:25106604	ENSG00000207245	SNORD116-29	5.414097304	0.068505813	-1.10271849	1.222392936	1.196801156	2.325111426	2.299519646	-0.02559178	9.961963646	0.296075617	0.249692171	0.259088728	0.042494338	0.04438435	0.980088331	-6.372588541	-5.450367593	-6.316654238	-4.791439494	-4.058886973	-7.071020741	2.472859041	0.121807721

TNFRSF10B	-0.050268288	0.12814808	0.161703311	0.178416368	0.211971599	0.033555231	8	0.082918516	0.816496581	0.816496581	0.816496581	0.816496581	0.816496581	0.816496581	10	135	0	P_264049	P_264049	P_264049	chr8	23028330	23028390	-	TNFRSF10B	"tumor necrosis factor receptor superfamily, member 10b [Source:HGNC Symbol;Acc:HGNC:11905]"	ENSG00000120889	protein_coding	3'UTR;exon	multiple Ref	1	1665	8:23028330:23028390	ENSG00000120889	TNFRSF10B	7.937536184	0.00772807	-0.700332387	1.785345288	2.252833155	2.485677675	2.953165542	0.467487867	9.961963646	0.499748441	0.104630313	0.048044915	0.032310979	0.014512741	0.650209376	-6.737149263	-4.733433448	-4.810974169	-4.525206635	-3.007336004	-6.953286563	3.978584267	0.042077527

ARID1B	-0.034684686	-0.231404148	-0.126856894	-0.196719462	-0.092172208	0.104547254	8	0.093858652	0.816496581	0.816496581	0.816496581	0.816496581	0.816496581	0.816496581	10	136	0	P_313337	P_313337	P_313337	chr6	157144963	157145023	+	ARID1B	AT rich interactive domain 1B (SWI1-like) [Source:HGNC Symbol;Acc:HGNC:18040]	ENSG00000049618	protein_coding	intron;promoter	multiple non-Ref	0	1666	6:157144963:157145023	ENSG00000049618	ARID1B	6.961472441	0.00928115	-0.440943052	-2.941818508	-1.612719401	-2.500875456	-1.171776349	1.329099107	9.961963646	0.66866953	0.014796751	0.137997647	0.031481876	0.268548796	0.213451057	-6.893291421	-2.950722565	-5.786310965	-4.499783072	-5.614039043	-6.183499329	3.473922178	0.058724991

LGR4	-0.01541646	-0.034016764	-0.169477036	-0.018600303	-0.154060576	-0.135460273	8	0.063183569	0.816496581	0.816496581	0.816496581	0.816496581	0.816496581	0.816496581	10	138	0	P_211357	P_211357	P_211357	chr11	27372282	27372342	-	LGR4	leucine-rich repeat containing G protein-coupled receptor 4 [Source:HGNC Symbol;Acc:HGNC:13299]	ENSG00000205213	protein_coding	exon	coding exon	1	1668	11:27372282:27372342	ENSG00000205213	LGR4	6.624626064	0.005412609	-0.256641553	-0.566285314	-2.821325331	-0.309643761	-2.564683778	-2.255040017	9.961963646	0.802677867	0.583730818	0.018183118	0.76321178	0.028224551	0.047865473	-6.962537625	-6.015809783	-3.860822397	-7.097432135	-3.637025018	-4.830668544	3.350851364	0.063907831

ATF7IP	-0.041035687	-0.116134205	-0.116578194	-0.075098517	-0.075542507	-0.000443989	8	0.082847481	0.816496581	0.816496581	0.816496581	0.816496581	0.816496581	0.816496581	10	140	0	P_111051	P_111051	P_111051	chr12	14502367	14502427	+	ATF7IP	activating transcription factor 7 interacting protein [Source:HGNC Symbol;Acc:HGNC:20092]	ENSG00000171681	protein_coding	3'UTR	3'UTR	1	1670	12:14502367:14502427	ENSG00000171681	ATF7IP	7.853266348	0.007718613	-0.57205488	-1.618960057	-1.625149458	-1.046905177	-1.053094578	-0.006189401	9.961963646	0.579963547	0.136641787	0.135308798	0.319879536	0.317170247	0.995183796	-6.822678143	-4.961887372	-5.768943803	-6.581163664	-5.738416958	-7.071357597	1.29907436	0.32820547

SKA2	-0.173392354	-0.034455906	-0.1301603	0.138936448	0.043232053	-0.095704395	8	0.035140243	0.816496581	0.816496581	0.816496581	0.816496581	0.816496581	0.816496581	10	141	0	P_122180	P_122180	P_122180	chr17	59110075	59110135	-	SKA2	spindle and kinetochore associated complex subunit 2 [Source:HGNC Symbol;Acc:HGNC:28006]	ENSG00000182628	protein_coding	3'UTR	3'UTR	1	1671	17:59110075:59110135	ENSG00000182628	SKA2	9.988213961	0.003198325	-3.75503738	-0.746187537	-2.81879093	3.008849843	0.93624645	-2.072603393	9.961963646	0.003777712	0.472795181	0.018262191	0.013197163	0.371287296	0.065104913	-2.227910904	-5.896800385	-3.865137741	-3.636383886	-5.850482499	-5.123014583	6.138017249	0.012360444

LIPI	-0.069418589	0.078269498	0.143941514	0.147688087	0.213360103	0.065672016	8	0.108514554	0.816496581	0.816496581	0.816496581	0.816496581	0.816496581	0.816496581	10	142	0	P_311720	P_311720	P_311720	chr21	14109154	14109214	-	LIPI;AP001347.6	"lipase, member I [Source:HGNC Symbol;Acc:HGNC:18821];"	ENSG00000188992	protein_coding;antisense	intron	multiple non-Ref	0	1672	21:14109154:14109214	ENSG00000188992	LIPI	4.306483965	0.011662979	-0.787257245	0.887632979	1.632401367	1.674890224	2.419658612	0.744768387	9.961963646	0.44945557	0.395663222	0.133761747	0.125011468	0.036169603	0.473615284	-6.670340686	-5.782991968	-5.758775965	-5.799061899	-3.868999153	-6.776547921	2.214514251	0.149507605

NDST4	-0.007464802	0.048993075	0.110106862	0.056457877	0.117571663	0.061113787	8	0.139361	0.816496581	0.816496581	0.816496581	0.816496581	0.816496581	0.816496581	10	144	0	P_250564	P_250564	P_250564	chr4	114935205	114935265	-	NDST4	N-deacetylase/N-sulfotransferase (heparan glucosaminyl) 4 [Source:HGNC Symbol;Acc:HGNC:20779]	ENSG00000138653	protein_coding	exon	coding exon	1	1674	4:114935205:114935265	ENSG00000138653	NDST4	3.613758188	0.017803198	-0.068519611	0.449708729	1.010673785	0.51822834	1.079193396	0.560965056	9.961963646	0.946727821	0.662547115	0.336090247	0.615612755	0.305937319	0.58721636	-6.995798936	-6.076585719	-6.516271506	-7.004702787	-5.711949632	-6.902144898	0.496049195	0.693160552

RAB3GAP2	0.25577869	0.025773147	0.149080184	-0.230005543	-0.106698506	0.123307037	8	0.059228842	0.816496581	0.816496581	0.816496581	0.816496581	0.816496581	0.816496581	10	145	0	P_206314	P_206314	P_206314	chr1	220157801	220157861	-	RAB3GAP2	RAB3 GTPase activating protein subunit 2 (non-catalytic) [Source:HGNC Symbol;Acc:HGNC:17168]	ENSG00000118873	protein_coding	exon	coding exon	1	1675	1:220157801:220157861	ENSG00000118873	RAB3GAP2	7.663557044	0.005023844	4.419691111	0.445343397	2.576009614	-3.974347715	-1.843681497	2.130666218	9.961963646	0.001306728	0.665592872	0.027682354	0.002644559	0.095137784	0.059057849	-1.136108589	-6.078603192	-4.276117161	-1.995225821	-4.749473743	-5.030934197	8.350360924	0.004506737

MYO5A	0.170490195	-0.097640445	0.129526924	-0.26813064	-0.040963271	0.227167369	8	0.074127413	0.816496581	0.816496581	0.816496581	0.816496581	0.816496581	0.816496581	10	146	0	P_223642	P_223642	P_223642	chr15	52405286	52405346	-	MYO5A	"myosin VA (heavy chain 12, myoxin) [Source:HGNC Symbol;Acc:HGNC:7602]"	ENSG00000197535	protein_coding	3'UTR;exon	multiple Ref	1	1676	15:52405286:52405346	ENSG00000197535	MYO5A	8.200390837	0.006619367	2.566473654	-1.469830157	1.949833178	-4.036303811	-0.616640476	3.419663335	9.961963646	0.028138168	0.172469536	0.079880935	0.00239372	0.551302792	0.006586951	-4.246114554	-5.155798701	-5.290782945	-1.892253672	-6.098327276	-2.86060744	6.819188065	0.008863247

DCHS2	-0.049601816	0.059333223	0.14054859	0.108935039	0.190150406	0.081215367	8	0.107977148	0.816496581	0.816496581	0.816496581	0.816496581	0.816496581	0.816496581	10	147	0	P_251288	P_251288	P_251288	chr4	154255518	154255578	-	DCHS2	dachsous cadherin-related 2 [Source:HGNC Symbol;Acc:HGNC:23111]	ENSG00000197410	protein_coding	exon	coding exon	1	1677	4:154255518:154255578	ENSG00000197410	DCHS2	3.756629438	0.011569548	-0.564787407	0.675593349	1.600346105	1.240380756	2.165133512	0.924752756	9.961963646	0.584711037	0.514674692	0.140721339	0.243253631	0.05572636	0.376951448	-6.82704384	-5.947045228	-5.803521613	-6.367563149	-4.268085553	-6.623305227	1.736339008	0.222805767

GNPTAB	0.059658974	0.00783502	0.085111634	-0.051823954	0.02545266	0.077276614	8	0.047550879	0.816496581	0.816496581	0.816496581	0.816496581	0.816496581	0.816496581	10	149	0	P_309221	P_309221	P_309221	chr12	101783204	101783264	-	GNPTAB	"N-acetylglucosamine-1-phosphate transferase, alpha and beta subunits [Source:HGNC Symbol;Acc:HGNC:29670]"	ENSG00000111670	protein_coding	intron	intron sense	0	1679	12:101783204:101783264	ENSG00000111670	GNPTAB	8.1648624	0.004022459	1.152061261	0.151300338	1.64357195	-1.000760923	0.491510689	1.492271612	9.961963646	0.276193102	0.882758802	0.13140963	0.340630409	0.63371402	0.16660253	-6.318804587	-6.170033943	-5.743063061	-6.627884809	-6.169774892	-5.974260014	1.253574241	0.342100374

MRPL30	0.131494884	-0.018427651	0.079201204	-0.149922535	-0.05229368	0.097628855	8	0.05294827	0.816496581	0.816496581	0.816496581	0.816496581	0.816496581	0.816496581	10	150	0	P_128509	P_128509	P_128509	chr2	99188196	99188256	+	MRPL30;RP11-38C17.1	mitochondrial ribosomal protein L30 [Source:HGNC Symbol;Acc:HGNC:14036];	ENSG00000185414	protein_coding	exon	coding exon	1	1680	2:99188196:99188256	ENSG00000185414	MRPL30	10.63704561	0.004458063	2.412022714	-0.338020089	1.452794949	-2.750042802	-0.959227764	1.790815038	9.961963646	0.03664415	0.742363257	0.17704156	0.020544376	0.36014579	0.103704628	-4.503451854	-6.122211203	-6.002488531	-4.078835243	-5.8292933	-5.554144089	3.288766022	0.066727794

C16orf87	0.239254725	0.070800387	0.056732149	-0.168454338	-0.182522576	-0.014068238	8	0.060322752	0.816496581	0.816496581	0.816496581	0.816496581	0.816496581	0.816496581	10	151	0	P_118918	P_118918	P_118918	chr16	46802961	46803021	-	C16orf87	chromosome 16 open reading frame 87 [Source:HGNC Symbol;Acc:HGNC:33754]	ENSG00000155330	protein_coding	3'UTR;exon	multiple Ref	1	1681	16:46802961:46803021	ENSG00000155330	C16orf87	8.801985832	0.005128866	4.091621582	1.210794861	0.970206488	-2.880826721	-3.121415094	-0.240588373	9.961963646	0.002190854	0.253916076	0.354909624	0.016422734	0.010896119	0.814753011	-1.668836277	-5.46324006	-6.556093442	-3.855656428	-2.733565642	-7.039849128	6.20816159	0.011932071

MGAT5	-0.007114628	-0.106696713	-0.11643305	-0.099582085	-0.109318422	-0.009736337	8	0.068379615	0.816496581	0.816496581	0.816496581	0.816496581	0.816496581	0.816496581	10	152	0	P_236518	P_236518	P_236518	chr2	134349869	134349929	+	MGAT5	"mannosyl (alpha-1,6-)-glycoprotein beta-1,6-N-acetyl-glucosaminyltransferase [Source:HGNC Symbol;Acc:HGNC:7049]"	ENSG00000152127	protein_coding	exon	coding exon	1	1682	2:134349869:134349929	ENSG00000152127	MGAT5	6.73923607	0.005961584	-0.11285402	-1.692450011	-1.846890233	-1.579595991	-1.734036213	-0.154440222	9.961963646	0.912388059	0.121547989	0.094639445	0.145396193	0.113688903	0.880346701	-6.99141484	-4.862435156	-5.447086296	-5.932817083	-4.905699409	-7.058363898	1.968997245	0.182900902

PLK2	0.176429253	-0.112960154	-0.211191083	-0.289389407	-0.387620336	-0.098230929	8	0.141013097	0.816496581	0.816496581	0.816496581	0.816496581	0.816496581	0.816496581	10	153	0	P_252956	P_252956	P_252956	chr5	58458103	58458163	-	PLK2	polo-like kinase 2 [Source:HGNC Symbol;Acc:HGNC:19699]	ENSG00000145632	protein_coding	exon	coding exon	1	1683	5:58458103:58458163	ENSG00000145632	PLK2	7.248965707	0.018175177	1.602791013	-1.026198982	-1.918588699	-2.628989995	-3.521379711	-0.892389716	9.961963646	0.140179394	0.329070333	0.084116287	0.025280424	0.005557909	0.393229652	-5.752438397	-5.655501544	-5.338634877	-4.284211031	-2.087206242	-6.652955865	4.568511474	0.029223185

EPS15L1	-0.118868561	-0.048413568	-0.099699342	0.070454992	0.019169219	-0.051285774	8	0.085187815	0.816496581	0.816496581	0.816496581	0.816496581	0.816496581	0.816496581	10	154	0	P_310809	P_310809	P_310809	chr19	16361422	16361482	+	EPS15L1;CTD-2013N17.6	epidermal growth factor receptor pathway substrate 15-like 1 [Source:HGNC Symbol;Acc:HGNC:24634];	ENSG00000127527	protein_coding;TEC	3'ext;intron;promoter	multiple non-Ref	0	1684	19:16361422:16361482	ENSG00000127527	EPS15L1	6.905855557	0.008034422	-1.624184358	-0.661508478	-1.362261905	0.962675879	0.261922453	-0.700753427	9.961963646	0.135515882	0.523291337	0.203119567	0.358495242	0.798717302	0.499496723	-5.722635114	-5.956529239	-6.118393976	-6.665148034	-6.260325269	-6.809560751	1.069621563	0.405311731

INAFM2	0.077048026	0.05117658	0.108656671	-0.025871446	0.031608645	0.057480091	8	0.04646812	0.816496581	0.816496581	0.816496581	0.816496581	0.816496581	0.816496581	11	17	0	P_116182	P_116182	P_116182	chr15	40326232	40326292	-	INAFM2	InaF-motif containing 2 [Source:HGNC Symbol;Acc:HGNC:35165]	ENSG00000259330	antisense	intron	intron antisense	0	1717	15:40326232:40326292	ENSG00000259330	INAFM2	10.41615993	0.003940708	1.503211151	0.998457841	2.119897534	-0.504753309	0.616686383	1.121439692	9.961963646	0.163805867	0.341691712	0.060137257	0.624709375	0.5512737	0.288404808	-5.888032197	-5.682233484	-5.024692001	-7.012047077	-6.098298305	-6.424646654	1.607205393	0.249260534

DST	0.089985997	-0.084795695	-0.00837251	-0.174781692	-0.098358507	0.076423186	8	0.073613413	0.816496581	0.816496581	0.816496581	0.816496581	0.816496581	0.816496581	11	19	0	P_257515	P_257515	P_257515	chr6	56572132	56572192	-	DST	dystonin [Source:HGNC Symbol;Acc:HGNC:1090]	ENSG00000151914	protein_coding	3'UTR;exon	multiple Ref	1	1719	6:56572132:56572192	ENSG00000151914	DST	5.228256737	0.006558384	1.360887249	-1.282392649	-0.126620166	-2.643279897	-1.487507415	1.155772483	9.961963646	0.203539329	0.228741112	0.901760227	0.024668903	0.167833389	0.27474114	-6.072127897	-5.382370573	-7.03837209	-4.260042388	-5.237656699	-6.386888872	2.341332438	0.135077911

BPIFB2	-0.096039177	-0.112296577	0.077736256	-0.0162574	0.173775433	0.190032833	8	0.19232852	0.816496581	0.816496581	0.816496581	0.816496581	0.816496581	0.816496581	11	20	0	A_23_P109229	A_23_P109229	A_23_P109229	chr20	33002615	33002874	-	BPIFB2;SUN5	"BPI fold containing family B, member 2 [Source:HGNC Symbol;Acc:HGNC:16177];Sad1 and UNC84 domain containing 5 [Source:HGNC Symbol;Acc:HGNC:16252]"	ENSG00000078898	protein_coding	"exon,intron;promoter"	multiple non-Ref	0	1720	20:33002615:33002874	ENSG00000078898	BPIFB2	5.706107004	0.031911879	-0.658442521	-0.769902904	0.532958092	-0.111460382	1.191400614	1.302860996	9.961963646	0.525178284	0.459224718	0.605746721	0.913465034	0.261107427	0.221932589	-6.766821651	-5.878923198	-6.894187479	-7.142823371	-5.592505079	-6.215598813	0.739941163	0.552129148

PIAS4	-0.082451232	-0.15224944	-0.106804206	-0.069798209	-0.024352975	0.045445234	8	0.09203954	0.816496581	0.816496581	0.816496581	0.816496581	0.816496581	0.816496581	11	23	0	P_124211	P_124211	P_124211	chr19	4037941	4038001	+	PIAS4	"protein inhibitor of activated STAT, 4 [Source:HGNC Symbol;Acc:HGNC:17002]"	ENSG00000105229	protein_coding	3'UTR	3'UTR	1	1723	19:4037941:4038001	ENSG00000105229	PIAS4	7.890336714	0.009009581	-1.06387468	-1.964486416	-1.378103016	-0.900611736	-0.314228336	0.5863834	9.961963646	0.312493063	0.07796421	0.198334735	0.389047995	0.759829415	0.570665025	-6.413632714	-4.475588052	-6.09847429	-6.723285793	-6.244434229	-6.886726004	1.357315095	0.311333555

SORBS1	0.37311314	-0.046009217	0.297663085	-0.419122356	-0.075450054	0.343672302	8	0.105672158	0.816496581	0.816496581	0.816496581	0.816496581	0.816496581	0.816496581	11	24	0	P_209306	P_209306	P_209306	chr10	95395006	95395066	-	SORBS1	sorbin and SH3 domain containing 1 [Source:HGNC Symbol;Acc:HGNC:14565]	ENSG00000095637	protein_coding	exon	coding exon	1	1724	10:95395006:95395066	ENSG00000095637	SORBS1	5.054415727	0.011174077	4.322951353	-0.533070494	3.448774384	-4.856021847	-0.87417697	3.981844877	9.961963646	0.001519495	0.60567175	0.006273733	0.000672693	0.402604238	0.002612791	-1.291822096	-6.034468573	-2.789026233	-0.573255993	-5.9055538	-1.913344792	11.84438991	0.001265977

ZNF599	0.080178586	-0.008370355	0.025421345	-0.088548941	-0.054757241	0.0337917	8	0.103119797	0.816496581	0.816496581	0.816496581	0.816496581	0.816496581	0.816496581	11	25	0	P_125422	P_125422	P_125422	chr19	34759033	34759093	-	ZNF599	zinc finger protein 599 [Source:HGNC Symbol;Acc:HGNC:26408]	ENSG00000153896	protein_coding	exon	coding exon	1	1725	19:34759033:34759093	ENSG00000153896	ZNF599	6.509544877	0.010746119	0.947279631	-0.09889258	0.300343566	-1.046172211	-0.646936065	0.399236146	9.961963646	0.365907762	0.923185203	0.770089209	0.320201533	0.532295739	0.698143745	-6.529672686	-6.176934456	-6.998081388	-6.581919038	-6.078777116	-6.984997606	0.444952794	0.72618933

CDC42BPA	-0.131528134	-0.060124635	-0.071789875	0.071403499	0.059738259	-0.01166524	8	0.045700931	0.816496581	0.816496581	0.816496581	0.816496581	0.816496581	0.816496581	11	26	0	P_206520	P_206520	P_206520	chr1	227069776	227069836	-	CDC42BPA	CDC42 binding protein kinase alpha (DMPK-like) [Source:HGNC Symbol;Acc:HGNC:1737]	ENSG00000143776	protein_coding	exon	coding exon	1	1726	1:227069776:227069836	ENSG00000143776	CDC42BPA	8.488202235	0.003883924	-2.584811903	-1.181578936	-1.410826097	1.403232967	1.173985806	-0.229247161	9.961963646	0.027268122	0.264810915	0.188751949	0.190939774	0.267702657	0.823314835	-4.215338987	-5.495266235	-6.056835606	-6.167560969	-5.611627909	-7.042743949	2.244611888	0.145925858

TUSC3	-0.185508866	-0.08671558	-0.299105546	0.098793287	-0.11359668	-0.212389966	8	0.073228684	0.816496581	0.816496581	0.816496581	0.816496581	0.816496581	0.816496581	11	28	0	A_24_P95154	A_24_P95154	A_24_P95154	chr8	15743551	15743611	+	TUSC3	tumor suppressor candidate 3 [Source:HGNC Symbol;Acc:HGNC:30242]	ENSG00000104723	protein_coding	"3'UTR,exon;exon"	multiple Ref	1	1728	8:15743551:15743611	ENSG00000104723	TUSC3	8.110093028	0.006513016	-2.815264683	-1.315987281	-4.539196952	1.499277402	-1.723932269	-3.22320967	9.961963646	0.018372787	0.217655171	0.001086519	0.164806783	0.115554791	0.00916908	-3.825673506	-5.34329798	-0.98570708	-6.041895884	-4.919852332	-3.196233953	7.645117083	0.006085386

SPATA4	0.511444839	0.288941443	0.433315182	-0.222503396	-0.078129657	0.144373739	8	0.103839845	0.816496581	0.816496581	0.816496581	0.816496581	0.816496581	0.816496581	11	30	0	P_138973	P_138973	P_138973	chr4	176184686	176184746	-	SPATA4	spermatogenesis associated 4 [Source:HGNC Symbol;Acc:HGNC:17333]	ENSG00000150628	protein_coding	3'UTR	3'UTR	1	1730	4:176184686:176184746	ENSG00000150628	SPATA4	5.014671496	0.010865791	6.009159887	3.39488288	5.091184837	-2.614277007	-0.917975049	1.696301958	9.961963646	0.000132559	0.006866297	0.000475671	0.025925841	0.380320286	0.120799911	1.235295546	-2.228031515	-0.128197929	-4.309071577	-5.867023513	-5.691783032	14.01832636	0.000658631

IFT88	0.13874784	0.033294078	0.11451068	-0.105453762	-0.02423716	0.081216602	8	0.038622894	0.816496581	0.816496581	0.816496581	0.816496581	0.816496581	0.816496581	11	31	0	P_218219	P_218219	P_218219	chr13	20599505	20599565	+	IFT88	intraflagellar transport 88 [Source:HGNC Symbol;Acc:HGNC:20606]	ENSG00000032742	protein_coding	exon	coding exon	1	1731	13:20599505:20599565	ENSG00000032742	IFT88	8.16318955	0.003404623	2.912308356	0.698840589	2.403571909	-2.213467767	-0.508736447	1.70473132	9.961963646	0.015562046	0.500640938	0.037176467	0.05135727	0.622013479	0.119177354	-3.660438914	-5.93099557	-4.563779478	-4.973520666	-6.160842467	-5.67966965	3.801906209	0.047173649

OR13C9	-0.088669867	0.624785477	0.247860199	0.713455344	0.336530066	-0.376925279	8	0.69222354	0.816496581	0.816496581	0.816496581	0.816496581	0.816496581	0.816496581	11	32	0	P_149292	P_149292	P_149292	chr9	104617487	104617547	-	OR13C9	"olfactory receptor, family 13, subfamily C, member 9 [Source:HGNC Symbol;Acc:HGNC:15104]"	ENSG00000136839	protein_coding	exon	coding exon	1	1732	9:104617487:104617547	ENSG00000136839	OR13C9	3.070858605	0.387009076	-0.174566583	1.230030784	0.487968568	1.404597367	0.662535151	-0.742062216	9.961963646	0.864916933	0.246941647	0.636133332	0.190545064	0.52266037	0.475181641	-6.98175676	-5.441841817	-6.918625575	-6.165814051	-6.068375689	-6.778629684	0.790677578	0.526379912

SEC31A	0.125339176	-0.047490452	0.090231003	-0.172829628	-0.035108173	0.137721455	8	0.048119198	0.816496581	0.816496581	0.816496581	0.816496581	0.816496581	0.816496581	11	34	0	P_138080	P_138080	P_138080	chr4	82900076	82900136	-	SEC31A;THAP9-AS1	SEC31 homolog A (S. cerevisiae) [Source:HGNC Symbol;Acc:HGNC:17052];THAP9 antisense RNA 1 [Source:HGNC Symbol;Acc:HGNC:44172]	ENSG00000138674	protein_coding;antisense	5'UTR;exon	multiple Ref	1	1734	4:82900076:82900136	ENSG00000138674	SEC31A	8.991794169	0.004066122	2.407366654	-0.91214044	1.733050385	-3.319507094	-0.674316269	2.645190825	9.961963646	0.036936503	0.383237447	0.113869743	0.007793596	0.515452439	0.024588253	-4.511151049	-5.761560779	-5.615057684	-3.103627755	-6.060370236	-4.182495926	4.683623718	0.027295393

CC2D2B	0.085509691	0.064677007	0.236872339	-0.020832683	0.151362648	0.172195331	8	0.134553328	0.816496581	0.816496581	0.816496581	0.816496581	0.816496581	0.816496581	11	36	0	P_209341	P_209341	P_209341	chr10	96012668	96012728	+	CC2D2B	coiled-coil and C2 domain containing 2B [Source:HGNC Symbol;Acc:HGNC:31666]	ENSG00000188649	protein_coding	exon	coding exon	1	1736	10:96012668:96012728	ENSG00000188649	CC2D2B	4.064420319	0.016745663	0.809300456	0.612130989	2.241861597	-0.197169467	1.432561141	1.629730608	9.961963646	0.437244978	0.554164719	0.048946736	0.847659628	0.182607168	0.134329654	-6.652300256	-5.988329746	-4.828777829	-7.128394815	-5.30745343	-5.786269272	1.800457345	0.210881087

CLDN3	-0.085024604	0.019487034	-0.068056336	0.104511639	0.016968268	-0.08754337	8	0.101910403	0.816496581	0.816496581	0.816496581	0.816496581	0.816496581	0.816496581	11	37	0	A_23_P71017	A_23_P71017	A_23_P71017	chr7	73769032	73769092	-	CLDN3	claudin 3 [Source:HGNC Symbol;Acc:HGNC:2045]	ENSG00000165215	protein_coding	3'UTR	3'UTR	1	1737	7:73769032:73769092	ENSG00000165215	CLDN3	10.01559813	0.010546992	-1.013971964	0.232395159	-0.811614675	1.246367123	0.202357289	-1.044009834	9.961963646	0.334589677	0.820935822	0.435975947	0.241140995	0.843710259	0.321152908	-6.464636583	-6.153696311	-6.698866017	-6.36052244	-6.274951438	-6.506523787	0.737533508	0.553381454

VEGFA	0.040596246	0.068926313	0.082862309	0.028330067	0.042266063	0.013935996	8	0.097300081	0.816496581	0.816496581	0.816496581	0.816496581	0.816496581	0.816496581	11	38	0	P_142768	P_142768	P_142768	chr6	43781974	43782034	-	VEGFA	vascular endothelial growth factor A [Source:HGNC Symbol;Acc:HGNC:12680]	ENSG00000112715	protein_coding	"exon;exon,intron;intron"	multiple non-Ref	0	1738	6:43781974:43782034	ENSG00000112715	VEGFA	5.55601224	0.009809447	0.502006395	0.852331266	1.024661463	0.350324872	0.522655068	0.172330196	9.961963646	0.626571955	0.414051627	0.329760609	0.733389101	0.612639133	0.866628636	-6.862564827	-5.812990181	-6.502198981	-7.082910023	-6.153412113	-7.055178875	0.409000609	0.750049278

LMLN	-0.139917187	-0.029226127	-0.228228447	0.11069106	-0.088311261	-0.19900232	8	0.051396085	0.816496581	0.816496581	0.816496581	0.816496581	0.816496581	0.816496581	11	39	0	P_247921	P_247921	P_247921	chr3	197999274	197999334	+	LMLN	leishmanolysin-like (metallopeptidase M8 family) [Source:HGNC Symbol;Acc:HGNC:15991]	ENSG00000185621	protein_coding	exon	coding exon	1	1739	3:197999274:197999334	ENSG00000185621	LMLN	6.707609201	0.004327999	-2.604792503	-0.544093247	-4.24885436	2.060699256	-1.644061857	-3.704761113	9.961963646	0.026350572	0.598343048	0.001707098	0.066415527	0.131307326	0.004102856	-4.181760942	-6.028393818	-1.453202728	-5.217621729	-5.030171822	-2.377061582	7.634736756	0.006113178

ZNF260	0.31123208	0.011115775	0.114512776	-0.300116305	-0.196719304	0.103397	8	0.080270349	0.816496581	0.816496581	0.816496581	0.816496581	0.816496581	0.816496581	11	41	0	P_125589	P_125589	P_125589	chr19	36514239	36514299	-	ZNF260	zinc finger protein 260 [Source:HGNC Symbol;Acc:HGNC:13499]	ENSG00000254004	protein_coding	exon	coding exon	1	1741	19:36514239:36514299	ENSG00000254004	ZNF260	8.241786052	0.007381028	4.436817283	0.158462661	1.632454669	-4.278354622	-2.804362613	1.473992008	9.961963646	0.001272465	0.877258491	0.133750435	0.001629635	0.018719	0.171368168	-1.108667003	-6.168867277	-5.758701133	-1.494037075	-3.249358409	-5.998486133	8.452797774	0.004320635

ADCYAP1	0.142268256	-0.108104074	0.185030239	-0.25037233	0.042761983	0.293134312	8	0.243161152	0.816496581	0.816496581	0.816496581	0.816496581	0.816496581	0.816496581	11	43	0	P_230391	P_230391	P_230391	chr18	909575	909635	+	ADCYAP1	adenylate cyclase activating polypeptide 1 (pituitary) [Source:HGNC Symbol;Acc:HGNC:241]	ENSG00000141433	protein_coding	exon	coding exon	1	1743	18:909575:909635	ENSG00000141433	ADCYAP1	5.424034101	0.049689167	0.781668804	-0.593959499	1.016617267	-1.375628303	0.234948463	1.610576766	9.961963646	0.452586478	0.565781863	0.333389757	0.199075897	0.819007629	0.138465916	-6.674844595	-5.999459479	-6.510311122	-6.202659541	-6.267408442	-5.813054384	1.0898027	0.397787489

LEMD2	-0.15147783	-0.038944165	-0.066890156	0.112533664	0.084587674	-0.027945991	8	0.157032169	0.816496581	0.816496581	0.816496581	0.816496581	0.816496581	0.816496581	11	44	0	P_142392	P_142392	P_142392	chr6	33784355	33784415	-	LEMD2	LEM domain containing 2 [Source:HGNC Symbol;Acc:HGNC:21244]	ENSG00000161904	protein_coding	3'UTR;5'UTR;exon	multiple Ref	1	1744	6:33784355:33784415	ENSG00000161904	LEMD2	7.135540695	0.022009288	-1.250523078	-0.321503004	-0.552210733	0.929020074	0.698312345	-0.230707729	9.961963646	0.239683123	0.754473291	0.592975875	0.374841333	0.500957204	0.822210816	-6.206201617	-6.127889253	-6.883107968	-6.697077994	-6.043668561	-7.042378896	0.562675555	0.651819528

KIF21A	-0.183007997	-0.01152652	-0.127865283	0.171481476	0.055142714	-0.116338762	8	0.049374794	0.816496581	0.816496581	0.816496581	0.816496581	0.816496581	0.816496581	11	45	0	P_111284	P_111284	P_111284	chr12	39294284	39294344	-	KIF21A	kinesin family member 21A [Source:HGNC Symbol;Acc:HGNC:19349]	ENSG00000139116	protein_coding	3'UTR;exon	multiple Ref	1	1745	12:39294284:39294344	ENSG00000139116	KIF21A	9.137455591	0.004164427	-3.473266287	-0.21875915	-2.426725517	3.254507137	1.04654077	-2.207966367	9.961963646	0.00602216	0.831254294	0.035735799	0.008696588	0.320039592	0.051837397	-2.703595919	-6.156922255	-4.525424927	-3.214997099	-5.744982799	-4.906919953	5.760435127	0.015009444

C12orf65	-0.202348382	0.005217312	-0.122767443	0.207565694	0.079580939	-0.127984755	8	0.092019038	0.816496581	0.816496581	0.816496581	0.816496581	0.816496581	0.816496581	11	46	0	P_113170	P_113170	P_113170	chr12	123254583	123254643	+	C12orf65	chromosome 12 open reading frame 65 [Source:HGNC Symbol;Acc:HGNC:26784]	ENSG00000130921	protein_coding	exon	non-coding exon	1	1746	12:123254583:123254643	ENSG00000130921	C12orf65	9.013520468	0.009006551	-2.611356194	0.067330703	-1.584344385	2.678686897	1.027011809	-1.651675088	9.961963646	0.026055908	0.947650616	0.144314447	0.023216557	0.328705848	0.12972664	-4.170720291	-6.179698098	-5.825663486	-4.200065772	-5.76435383	-5.755358531	3.382001511	0.062546353

SRPK1	-0.196946962	0.097420258	-0.048623136	0.29436722	0.148323826	-0.146043395	8	0.049020523	0.816496581	0.816496581	0.816496581	0.816496581	0.816496581	0.816496581	11	47	0	P_256879	P_256879	P_256879	chr6	35890894	35890954	-	SRPK1	SRSF protein kinase 1 [Source:HGNC Symbol;Acc:HGNC:11305]	ENSG00000096063	protein_coding	exon	coding exon	1	1747	6:35890894:35890954	ENSG00000096063	SRPK1	9.53784844	0.004136433	-3.750437187	1.8551622	-0.925924508	5.605599387	2.82451268	-2.781086708	9.961963646	0.003806312	0.093365865	0.376371195	0.000229108	0.018084163	0.019480307	-2.235627591	-4.634271305	-6.598122633	0.552579535	-3.216618233	-3.952060277	10.91663362	0.001721055

EPRS1	0.005742677	0.060436918	0.248201589	0.054694241	0.242458912	0.18776467	8	0.062248556	0.816496581	0.816496581	0.816496581	0.816496581	0.816496581	0.816496581	11	50	0	P_206256	P_206256	P_206256	chr1	219978545	219978605	-	EPRS	glutamyl-prolyl-tRNA synthetase [Source:HGNC Symbol;Acc:HGNC:3418]	ENSG00000136628	protein_coding	exon	coding exon	1	1750	1:219978545:219978605	ENSG00000136628	EPRS1	11.76300569	0.005318426	0.0964425	1.014977408	4.168296661	0.918534909	4.071854161	3.153319252	9.961963646	0.92508174	0.334133225	0.001939073	0.380041201	0.002261189	0.010321647	-6.993287137	-5.66638586	-1.584764886	-6.706830888	-1.220963021	-3.315873121	7.630217056	0.006125326

RASA3	0.008455936	0.078028881	0.091583891	0.069572945	0.083127955	0.01355501	8	0.091660237	0.816496581	0.816496581	0.816496581	0.816496581	0.816496581	0.816496581	11	51	0	P_114242	P_114242	P_114242	chr13	113979068	113979128	-	RASA3	RAS p21 protein activator 3 [Source:HGNC Symbol;Acc:HGNC:20331]	ENSG00000185989	protein_coding	3'UTR	3'UTR	1	1751	13:113979068:113979128	ENSG00000185989	RASA3	5.391968478	0.008953626	0.109448001	1.009953853	1.185400873	0.900505852	1.075952872	0.17544702	9.961963646	0.915020484	0.336418459	0.26336482	0.389101647	0.307315272	0.864243257	-6.991827503	-5.671227266	-6.329622791	-6.723382187	-5.715263599	-7.054588509	0.739421318	0.552399302

VTA1	0.492972172	-0.009485532	0.509876504	-0.502457704	0.016904331	0.519362036	8	0.031624569	0.816496581	0.816496581	0.816496581	0.816496581	0.816496581	0.816496581	11	52	0	P_259504	P_259504	P_259504	chr6	142166243	142166303	+	VTA1	vesicle (multivesicular body) trafficking 1 [Source:HGNC Symbol;Acc:HGNC:20954]	ENSG00000009844	protein_coding	exon	coding exon	1	1752	6:142166243:142166303	ENSG00000009844	VTA1	9.574117489	0.00300983	11.00517832	-0.211756315	11.38255293	-11.21693463	0.37737461	11.59430924	9.961963646	6.78E-07	0.836566639	4.96E-07	5.68E-07	0.713810318	4.18E-07	6.606700727	-6.158503919	6.91501044	6.765131141	-6.221492585	7.084216184	85.18522379	2.11E-07

CFAP65	0.049809312	-0.21624017	-0.058340402	-0.266049481	-0.108149714	0.157899768	8	0.160821424	0.816496581	0.816496581	0.816496581	0.816496581	0.816496581	0.816496581	11	53	0	P_129915	P_129915	P_129915	chr2	219035164	219035224	-	CCDC108	coiled-coil domain containing 108 [Source:HGNC Symbol;Acc:HGNC:25325]	ENSG00000181378	protein_coding	3'UTR	3'UTR	1	1753	2:219035164:219035224	ENSG00000181378	CFAP65	4.825711054	0.022976509	0.402452054	-1.747189377	-0.471382033	-2.149641431	-0.873834087	1.275807344	9.961963646	0.695851418	0.111300965	0.647521611	0.057200842	0.402782204	0.230967852	-6.910692449	-4.786843173	-6.92711776	-5.076277728	-5.905849136	-6.248221102	1.741511344	0.221815458

RPGRIP1	-0.141904933	0.06788628	-0.167053132	0.209791212	-0.025148199	-0.234939411	8	0.127295955	0.816496581	0.816496581	0.816496581	0.816496581	0.816496581	0.816496581	11	54	0	P_220085	P_220085	P_220085	chr14	21302524	21302584	+	RPGRIP1	retinitis pigmentosa GTPase regulator interacting protein 1 [Source:HGNC Symbol;Acc:HGNC:13436]	ENSG00000092200	protein_coding	exon	coding exon	1	1754	14:21302524:21302584	ENSG00000092200	RPGRIP1	4.709196175	0.015219588	-1.408775104	0.673947685	-1.65843631	2.082722789	-0.249661207	-2.332383996	9.961963646	0.189340792	0.515677039	0.128337172	0.064010176	0.807921974	0.041971706	-6.011532586	-5.94816271	-5.722060018	-5.182835615	-6.263639103	-4.704302314	2.504890635	0.11881194

LRRC52-AS1	0.039951841	0.290733634	0.345444877	0.250781793	0.305493037	0.054711243	8	0.159288704	0.816496581	0.816496581	0.816496581	0.816496581	0.816496581	0.816496581	11	56	0	P_204630	P_204630	P_204630	chr1	165482464	165482524	-	RP11-280O1.2	NA	ENSG00000237463	antisense	exon	non-coding exon	1	1756	1:165482464:165482524	ENSG00000237463	LRRC52-AS1	6.558376188	0.022582499	0.325609016	2.369490137	2.815388845	2.043881122	2.489779829	0.445898708	9.961963646	0.751456155	0.039401291	0.018368881	0.068309935	0.032085088	0.665205067	-6.940813552	-3.859907448	-3.870929883	-5.244086944	-3.757160565	-6.963836661	4.037033064	0.040537837

NUP214	-0.062531275	-0.021209562	-0.119439706	0.041321713	-0.056908431	-0.098230144	8	0.040012228	0.816496581	0.816496581	0.816496581	0.816496581	0.816496581	0.816496581	11	57	0	P_269201	P_269201	P_269201	chr9	131128398	131128458	+	NUP214	nucleoporin 214kDa [Source:HGNC Symbol;Acc:HGNC:8064]	ENSG00000126883	protein_coding	exon	coding exon	1	1757	9:131128398:131128458	ENSG00000126883	NUP214	7.398730251	0.003492357	-1.295936139	-0.43955985	-2.47534105	0.856376289	-1.179404912	-2.035781201	9.961963646	0.224217065	0.66963798	0.032887165	0.411915322	0.265636312	0.069240498	-6.152002413	-6.081247021	-4.444600768	-6.762712835	-5.605699789	-5.18089453	2.37812634	0.1312016

C9	0.357784128	-0.076111871	-0.081195219	-0.433895999	-0.438979347	-0.005083349	8	0.238842799	0.816496581	0.816496581	0.816496581	0.816496581	0.816496581	0.816496581	11	58	0	P_252617	P_252617	P_252617	chr5	39341207	39341267	-	C9	complement component 9 [Source:HGNC Symbol;Acc:HGNC:1358]	ENSG00000113600	protein_coding	exon	coding exon	1	1758	5:39341207:39341267	ENSG00000113600	C9	3.62046067	0.048017638	1.999706706	-0.42540014	-0.453811703	-2.425106846	-2.453518409	-0.028411563	9.961963646	0.073532566	0.679588209	0.659690281	0.035834708	0.034137087	0.977895017	-5.166606673	-6.087579603	-6.935807165	-4.626171365	-3.815077636	-7.070937558	2.714573378	0.101210263

P4HA1	0.050311329	0.319221857	0.073571042	0.268910528	0.023259713	-0.245650815	8	0.177340304	0.816496581	0.816496581	0.816496581	0.816496581	0.816496581	0.816496581	11	59	0	P_208640	P_208640	P_208640	chr10	73073735	73073795	-	P4HA1	"prolyl 4-hydroxylase, alpha polypeptide I [Source:HGNC Symbol;Acc:HGNC:8546]"	ENSG00000122884	protein_coding	exon	coding exon	1	1759	10:73073735:73073795	ENSG00000122884	P4HA1	8.541849894	0.027462414	0.371827975	2.359222447	0.543729852	1.987394472	0.171901876	-1.815492596	9.961963646	0.717807852	0.040096743	0.598583916	0.075054083	0.86695655	0.099621015	-6.923438046	-3.875926748	-6.888034185	-5.332320754	-6.280991911	-5.517573839	2.212521526	0.14974836

MED14	0.05654182	-0.102061636	0.017396617	-0.158603456	-0.039145203	0.119458253	8	0.04436589	0.816496581	0.816496581	0.816496581	0.816496581	0.816496581	0.816496581	11	60	0	P_270037	P_270037	P_270037	chrX	40675235	40675295	-	MED14	mediator complex subunit 14 [Source:HGNC Symbol;Acc:HGNC:2370]	ENSG00000180182	protein_coding	exon	coding exon	1	1760	X:40675235:40675295	ENSG00000180182	MED14	6.766422198	0.003787362	1.125244755	-2.031139465	0.346211915	-3.15638422	-0.77903284	2.37735138	9.961963646	0.286864807	0.069779166	0.736384167	0.010268115	0.454068203	0.038876823	-6.348260206	-4.37700886	-6.982055926	-3.383286724	-5.983652049	-4.630269756	3.622169845	0.053129935

HEATR5B	-0.005236288	-0.048918807	-0.149196383	-0.043682519	-0.143960095	-0.100277576	8	0.044879124	0.816496581	0.816496581	0.816496581	0.816496581	0.816496581	0.816496581	11	62	0	P_234444	P_234444	P_234444	chr2	37058450	37058510	-	HEATR5B	HEAT repeat containing 5B [Source:HGNC Symbol;Acc:HGNC:29273]	ENSG00000008869	protein_coding	exon	coding exon	1	1762	2:37058450:37058510	ENSG00000008869	HEATR5B	6.355784598	0.003824145	-0.103705549	-0.968845048	-2.954858989	-0.865139499	-2.85115344	-1.986013941	9.961963646	0.919461103	0.355555915	0.014470832	0.407313166	0.017278022	0.075226523	-6.992494676	-5.710112453	-3.632996491	-6.755037373	-3.173311124	-5.258444727	3.750400147	0.048795417

DKK3	-0.114551253	-0.201311343	0.053731717	-0.086760089	0.168282971	0.25504306	8	0.141864534	0.816496581	0.816496581	0.816496581	0.816496581	0.816496581	0.816496581	11	63	0	A_23_P363149	A_23_P363149	A_23_P363149	chr11	11963999	11964059	+	DKK3;USP47	dickkopf WNT signaling pathway inhibitor 3 [Source:HGNC Symbol;Acc:HGNC:2893];ubiquitin specific peptidase 47 [Source:HGNC Symbol;Acc:HGNC:20076]	ENSG00000050165	protein_coding	3'ext;3'UTR;exon	multiple non-Ref	0	1763	11:11963999:11964059	ENSG00000050165	DKK3	4.229818051	0.018368595	-1.03516006	-1.819180981	0.485554947	-0.784020921	1.520715007	2.304735928	9.961963646	0.325068755	0.099023531	0.637784443	0.451266978	0.159416055	0.043992593	-6.443221373	-4.68560101	-6.919878747	-6.823483621	-5.194682254	-4.749620928	2.142635005	0.158485974

PLA2R1	0.031119472	-0.042754475	0.029430566	-0.073873948	-0.001688906	0.072185041	8	0.10006056	0.816496581	0.816496581	0.816496581	0.816496581	0.816496581	0.816496581	11	65	0	P_311096	P_311096	P_311096	chr2	159950782	159950842	+	PLA2R1	"phospholipase A2 receptor 1, 180kDa [Source:HGNC Symbol;Acc:HGNC:9042]"	ENSG00000153246	protein_coding	intron	intron antisense	0	1765	2:159950782:159950842	ENSG00000153246	PLA2R1	4.038624462	0.010246959	0.376513331	-0.517284798	0.356079319	-0.89379813	-0.020434012	0.873364117	9.961963646	0.714430461	0.616247519	0.729206575	0.392511116	0.984100637	0.403026221	-6.92155145	-6.042964014	-6.978318117	-6.729469092	-6.296463173	-6.669967331	0.34969931	0.790338809

EIF2B4	-0.107985839	0.071454375	-0.13626345	0.179440214	-0.028277611	-0.207717825	8	0.089403141	0.816496581	0.816496581	0.816496581	0.816496581	0.816496581	0.816496581	11	66	0	P_234072	P_234072	P_234072	chr2	27367073	27367133	-	EIF2B4	"eukaryotic translation initiation factor 2B, subunit 4 delta, 67kDa [Source:HGNC Symbol;Acc:HGNC:3260]"	ENSG00000115211	protein_coding	3'UTR;exon	multiple Ref	1	1766	2:27367073:27367133	ENSG00000115211	EIF2B4	9.197603213	0.008625436	-1.424039088	0.942288589	-1.796943755	2.366327677	-0.372904666	-2.739232343	9.961963646	0.184995777	0.368334287	0.10267635	0.039614224	0.717031162	0.020928464	-5.991924524	-5.734527485	-5.521449991	-4.723547978	-6.223250701	-4.023199481	3.231126727	0.069478875

CALCOCO2	0.168205057	0.124187633	0.267937313	-0.044017424	0.099732256	0.143749679	8	0.101187377	0.816496581	0.816496581	0.816496581	0.816496581	0.816496581	0.816496581	11	67	0	P_229107	P_229107	P_229107	chr17	48852944	48853004	+	CALCOCO2	calcium binding and coiled-coil domain 2 [Source:HGNC Symbol;Acc:HGNC:29912]	ENSG00000136436	protein_coding	exon	coding exon	1	1767	17:48852944:48853004	ENSG00000136436	CALCOCO2	9.215404504	0.010429067	2.017260331	1.489365366	3.213335684	-0.527894965	1.196075353	1.723970317	9.961963646	0.071413585	0.167352441	0.009323552	0.609128787	0.259359302	0.115547712	-5.139309496	-5.131041986	-3.190854695	-6.999321334	-5.587336276	-5.651900512	3.549069609	0.055805675

NSD3	-0.092004939	-0.150870925	-0.04184032	-0.058865987	0.050164618	0.109030605	8	0.085750087	0.816496581	0.816496581	0.816496581	0.816496581	0.816496581	0.816496581	11	68	0	P_264425	P_264425	P_264425	chr8	38305309	38305369	-	WHSC1L1	Wolf-Hirschhorn syndrome candidate 1-like 1 [Source:HGNC Symbol;Acc:HGNC:12767]	ENSG00000147548	protein_coding	exon	coding exon	1	1768	8:38305309:38305369	ENSG00000147548	NSD3	8.488116024	0.008111606	-1.251132608	-2.051623937	-0.568967165	-0.800491328	0.682165443	1.482656772	9.961963646	0.23946991	0.067431495	0.581978048	0.442098034	0.510683433	0.169094676	-6.205483394	-4.346458163	-6.873164376	-6.810062851	-6.054965374	-5.987026292	1.567104063	0.258196991

CYBC1	-0.019534256	-0.034957839	0.041252368	-0.015423582	0.060786625	0.076210207	8	0.060041813	0.816496581	0.816496581	0.816496581	0.816496581	0.816496581	0.816496581	11	71	0	P_123045	P_123045	P_123045	chr17	82443643	82443703	-	C17orf62	chromosome 17 open reading frame 62 [Source:HGNC Symbol;Acc:HGNC:28672]	ENSG00000178927	protein_coding	"3'UTR;3'UTR,exon;exon"	multiple Ref	1	1771	17:82443643:82443703	ENSG00000178927	CYBC1	7.15221291	0.005101711	-0.334953551	-0.599421448	0.707353636	-0.264467897	1.042307187	1.306775084	9.961963646	0.744606136	0.562275815	0.495560984	0.796810402	0.321903517	0.220649892	-6.937481975	-5.996146804	-6.780564615	-7.111497888	-5.749206837	-6.210838905	0.63930963	0.606855759

ANKRD6	0.207653037	0.07873755	0.256218224	-0.128915487	0.048565187	0.177480674	8	0.084870943	0.816496581	0.816496581	0.816496581	0.816496581	0.816496581	0.816496581	11	72	0	P_258228	P_258228	P_258228	chr6	89629149	89629209	+	ANKRD6	ankyrin repeat domain 6 [Source:HGNC Symbol;Acc:HGNC:17280]	ENSG00000135299	protein_coding	exon	coding exon	1	1772	6:89629149:89629209	ENSG00000135299	ANKRD6	5.382179463	0.007991148	2.844980814	1.07875532	3.510355262	-1.766225494	0.665374448	2.431599941	9.961963646	0.017461517	0.306123323	0.005660903	0.107925868	0.520917766	0.035439565	-3.775127533	-5.603263159	-2.684329175	-5.666811588	-6.066458106	-4.54045877	5.17586281	0.020587188

PHF20L1	0.144054986	-0.02103831	0.084377994	-0.165093296	-0.059676992	0.105416304	8	0.055101148	0.816496581	0.816496581	0.816496581	0.816496581	0.816496581	0.816496581	11	73	0	P_266370	P_266370	P_266370	chr8	132814829	132814889	+	PHF20L1	PHD finger protein 20-like 1 [Source:HGNC Symbol;Acc:HGNC:24280]	ENSG00000129292	protein_coding	3'UTR;exon	multiple Ref	1	1773	8:132814829:132814889	ENSG00000129292	PHF20L1	8.209490032	0.004644867	2.588733105	-0.378067915	1.516310635	-2.96680102	-1.07242247	1.89437855	9.961963646	0.027085581	0.713311279	0.160510856	0.014178758	0.308821889	0.08754136	-4.208753019	-6.107293811	-5.918300668	-3.708462423	-5.718865116	-5.399037505	3.780723452	0.047832744

TSPAN14	-0.019574079	0.079858102	0.038692432	0.099432181	0.058266511	-0.04116567	8	0.07692887	0.816496581	0.816496581	0.816496581	0.816496581	0.816496581	0.816496581	11	74	0	P_308830	P_308830	P_308830	chr10	80511273	80511333	+	TSPAN14	tetraspanin 14 [Source:HGNC Symbol;Acc:HGNC:23303]	ENSG00000108219	protein_coding	intron;promoter	multiple non-Ref	0	1774	10:80511273:80511333	ENSG00000108219	TSPAN14	6.464918349	0.006959202	-0.287373854	1.172424544	0.568057039	1.459798397	0.855430893	-0.604367505	9.961963646	0.779715125	0.268300342	0.582572558	0.175149438	0.412413938	0.55911142	-6.953482304	-5.505181882	-6.873711619	-6.09420168	-5.92155498	-6.875432036	0.834647549	0.505039396

UGT2A1	0.436999343	0.250536701	0.474328892	-0.186462642	0.037329549	0.223792191	8	0.158154582	0.816496581	0.816496581	0.816496581	0.816496581	0.816496581	0.816496581	11	75	0	P_137822	P_137822	P_137822	chr4	69589193	69589253	-	UGT2A1	"UDP glucuronosyltransferase 2 family, polypeptide A1, complex locus [Source:HGNC Symbol;Acc:HGNC:12542]"	ENSG00000173610	protein_coding	3'UTR	3'UTR	1	1775	4:69589193:69589253	ENSG00000173610	UGT2A1	6.307369234	0.022293384	3.584581153	2.055081206	3.890784816	-1.529499947	0.306203663	1.83570361	9.961963646	0.005003812	0.067042731	0.003027346	0.157251842	0.765753228	0.096387335	-2.514995848	-4.34129068	-2.043571668	-6.001261877	-6.247056183	-5.487437279	6.335620968	0.011198245

MIR205HG	0.132851045	0.1447784	0.230866347	0.011927355	0.098015302	0.086087947	8	0.262474183	0.816496581	0.816496581	0.816496581	0.816496581	0.816496581	0.816496581	11	76	0	P_205987	P_205987	P_205987	chr1	209431741	209431801	-	MIR205HG;MIR205	MIR205 host gene (non-protein coding) [Source:HGNC Symbol;Acc:HGNC:43562];microRNA 205 [Source:HGNC Symbol;Acc:HGNC:31583]	ENSG00000230937	processed_transcript;miRNA	exon;intron;promoter	multiple non-Ref	0	1776	1:209431741:209431801	ENSG00000230937	MIR205HG	6.627337161	0.057531276	0.678357454	0.739260323	1.178838353	0.060902869	0.500480899	0.43957803	9.961963646	0.512993758	0.476806862	0.265851749	0.952641082	0.627607529	0.669625247	-6.752922848	-5.901928479	-6.337045819	-7.147585028	-6.16515983	-6.966834798	0.473958943	0.707305563

MCL1	0.127878594	0.034134666	0.094082599	-0.093743929	-0.033795996	0.059947933	8	0.066618723	0.816496581	0.816496581	0.816496581	0.816496581	0.816496581	0.816496581	11	77	0	P_103041	P_103041	P_103041	chr1	150574781	150574841	+	MCL1;ADAMTSL4-AS1	myeloid cell leukemia 1 [Source:HGNC Symbol;Acc:HGNC:6943];ADAMTSL4 antisense RNA 1 [Source:HGNC Symbol;Acc:HGNC:32041]	ENSG00000143384	protein_coding;processed_transcript	3'ext;3'UTR;promoter	multiple non-Ref	0	1777	1:150574781:150574841	ENSG00000143384	MCL1	10.17280821	0.005770684	2.061720808	0.550335658	1.516845351	-1.51138515	-0.544875458	0.966509693	9.961963646	0.06630208	0.594213382	0.160377592	0.161742958	0.597824752	0.356666532	-5.069735229	-6.024901742	-5.917582312	-6.025677806	-6.141157867	-6.583742043	1.728282862	0.224358391

SFPQ	0.07713448	-0.165111761	-0.013427146	-0.24224624	-0.090561625	0.151684615	8	0.064126357	0.816496581	0.816496581	0.816496581	0.816496581	0.816496581	0.816496581	11	78	0	P_200994	P_200994	P_200994	chr1	35189018	35189078	-	SFPQ	splicing factor proline/glutamine-rich [Source:HGNC Symbol;Acc:HGNC:10774]	ENSG00000116560	protein_coding	exon	coding exon	1	1778	1:35189018:35189078	ENSG00000116560	SFPQ	10.36014132	0.005508997	1.272793458	-2.724503622	-0.221560879	-3.99729708	-1.494354337	2.502942743	9.961963646	0.231992882	0.021463388	0.829131383	0.002548572	0.166066912	0.031370733	-6.179795872	-3.298390083	-7.020378798	-1.957036639	-5.228843882	-4.421600569	5.594702267	0.016383941

FDPS	-0.195692786	0.064852365	-0.123986927	0.260545151	0.071705859	-0.188839292	8	0.11663858	0.816496581	0.816496581	0.816496581	0.816496581	0.816496581	0.816496581	11	79	0	P_103463	P_103463	P_103463	chr1	155319628	155319688	+	FDPS	farnesyl diphosphate synthase [Source:HGNC Symbol;Acc:HGNC:3631]	ENSG00000160752	protein_coding	exon	coding exon	1	1779	1:155319628:155319688	ENSG00000160752	FDPS	7.141867075	0.013131886	-2.091495299	0.693119139	-1.325128435	2.784614439	0.766366864	-2.018247575	9.961963646	0.063075355	0.504072952	0.214716995	0.019362949	0.461232002	0.07129614	-5.022807523	-5.934991172	-6.16446676	-4.019949031	-5.99345491	-5.208307429	3.170908519	0.072497958

ABHD4	0.116415121	0.084383158	-0.163495883	-0.032031963	-0.279911004	-0.247879041	8	0.1578385	0.816496581	0.816496581	0.816496581	0.816496581	0.816496581	0.816496581	11	80	0	P_114364	P_114364	P_114364	chr14	22611180	22611240	+	ABHD4	abhydrolase domain containing 4 [Source:HGNC Symbol;Acc:HGNC:20154]	ENSG00000100439	protein_coding	3'UTR	3'UTR	1	1780	14:22611180:22611240	ENSG00000100439	ABHD4	6.287353561	0.022213175	0.956642575	0.693419554	-1.343529273	-0.263223021	-2.300171848	-2.036948827	9.961963646	0.361386884	0.503892391	0.20890272	0.797742817	0.044335183	0.069105618	-6.520762776	-5.934782114	-6.14174609	-7.111854622	-4.05786282	-5.179065569	2.118384002	0.161656306

KIAA0930	-0.028873366	0.110974076	0.125183595	0.139847442	0.154056961	0.014209519	8	0.098147	0.816496581	0.816496581	0.816496581	0.816496581	0.816496581	0.816496581	11	81	0	A_23_P315589	A_23_P315589	A_23_P315589	chr22	45196677	45196737	-	KIAA0930	KIAA0930 [Source:HGNC Symbol;Acc:HGNC:1314]	ENSG00000100364	protein_coding	3'UTR;exon	multiple Ref	1	1781	22:45196677:45196737	ENSG00000100364	KIAA0930	9.357365877	0.009942375	-0.354648377	1.363082384	1.537616344	1.717730761	1.892264721	0.17453396	9.961963646	0.730245768	0.202869375	0.155275226	0.11671374	0.087846495	0.864941897	-6.930158797	-5.287359868	-5.8895568	-5.737501356	-4.678802524	-7.054762543	1.818293321	0.207696235

PATL2	-0.033020497	-0.120679741	0.141605675	-0.087659244	0.174626172	0.262285416	8	0.136163697	0.816496581	0.816496581	0.816496581	0.816496581	0.816496581	0.816496581	11	82	0	P_223198	P_223198	P_223198	chr15	44670026	44670086	-	PATL2	protein associated with topoisomerase II homolog 2 (yeast) [Source:HGNC Symbol;Acc:HGNC:33630]	ENSG00000229474	protein_coding	3'UTR;exon	multiple Ref	1	1782	15:44670026:44670086	ENSG00000229474	PATL2	3.959383332	0.017095758	-0.309303656	-1.130409542	1.326423177	-0.821105886	1.635726833	2.456832719	9.961963646	0.763462909	0.284784768	0.214303517	0.430797046	0.133057622	0.033944284	-6.946405743	-5.549946121	-6.162875189	-6.792920597	-5.041519343	-4.498512679	2.086456181	0.16594388

ELOF1	-0.148312227	0.211476192	0.161725742	0.359788419	0.310037969	-0.049750451	8	0.114792402	0.816496581	0.816496581	0.816496581	0.816496581	0.816496581	0.816496581	11	84	0	P_124703	P_124703	P_124703	chr19	11553055	11553115	-	ELOF1	elongation factor 1 homolog (S. cerevisiae) [Source:HGNC Symbol;Acc:HGNC:28691]	ENSG00000130165	protein_coding	3'UTR	3'UTR	1	1784	19:11553055:11553115	ENSG00000130165	ELOF1	9.352197082	0.012788771	-1.606231667	2.290301775	1.751500958	3.896533442	3.357732625	-0.538800817	9.961963646	0.139419869	0.04508495	0.1105282	0.00299924	0.007308202	0.601856021	-5.747660726	-3.983008196	-5.588207937	-2.12511156	-2.350493669	-6.915066103	6.273477593	0.011549065

ACYP1	-0.249428024	0.083546096	-0.117361039	0.332974121	0.132066985	-0.200907136	8	0.045280228	0.816496581	0.816496581	0.816496581	0.816496581	0.816496581	0.816496581	11	86	0	P_115391	P_115391	P_115391	chr14	75053244	75053304	-	ACYP1	"acylphosphatase 1, erythrocyte (common) type [Source:HGNC Symbol;Acc:HGNC:179]"	ENSG00000119640	protein_coding	3'UTR	3'UTR	1	1786	14:75053244:75053304	ENSG00000119640	ACYP1	8.72738798	0.003853186	-4.921311966	1.648396986	-2.315578968	6.569708952	2.605732998	-3.963975954	9.961963646	0.000610497	0.130405149	0.043188988	6.43E-05	0.026308148	0.002689177	-0.348723294	-4.922341028	-4.708640895	1.883017182	-3.570938675	-1.943025311	16.32707593	0.0003573

ARID4A	0.062888076	-0.02584309	0.079518548	-0.088731166	0.016630471	0.105361638	8	0.078510263	0.816496581	0.816496581	0.816496581	0.816496581	0.816496581	0.816496581	11	87	0	P_309893	P_309893	P_309893	chr14	58347217	58347277	+	ARID4A	AT rich interactive domain 4A (RBP1-like) [Source:HGNC Symbol;Acc:HGNC:9885]	ENSG00000032219	protein_coding	intron;promoter	multiple non-Ref	0	1787	14:58347217:58347277	ENSG00000032219	ARID4A	8.106191013	0.007156601	0.910459318	-0.374142178	1.151226227	-1.284601496	0.240766908	1.525368405	9.961963646	0.3840809	0.716138879	0.276520629	0.227998183	0.81461843	0.158266446	-6.563998971	-6.108827831	-6.367936683	-6.314976669	-6.265944812	-5.929919985	1.054859807	0.410913266

USP13	-0.121447691	-0.014682158	-0.091408951	0.106765533	0.03003874	-0.076726793	8	0.040495103	0.816496581	0.816496581	0.816496581	0.816496581	0.816496581	0.816496581	11	88	0	P_136608	P_136608	P_136608	chr3	179784274	179784334	+	USP13	ubiquitin specific peptidase 13 (isopeptidase T-3) [Source:HGNC Symbol;Acc:HGNC:12611]	ENSG00000058056	protein_coding	3'UTR	3'UTR	1	1788	3:179784274:179784334	ENSG00000058056	USP13	8.293644167	0.003523576	-2.505780883	-0.302931005	-1.886003756	2.202849878	0.619777127	-1.583072751	9.961963646	0.031218778	0.768173685	0.088756096	0.052287815	0.549317094	0.144603442	-4.347657514	-6.133941082	-5.388163282	-4.990686644	-6.096343253	-5.851191908	2.945084301	0.085295315

GPR155	0.249149134	0.117421252	0.192483266	-0.131727882	-0.056665868	0.075062014	8	0.065372424	0.816496581	0.816496581	0.816496581	0.816496581	0.816496581	0.816496581	11	89	0	A_32_P132317	A_32_P132317	A_32_P132317	chr2	174432373	174432433	-	GPR155	G protein-coupled receptor 155 [Source:HGNC Symbol;Acc:HGNC:22951]	ENSG00000163328	protein_coding	3'UTR	3'UTR	1	1789	2:174432373:174432433	ENSG00000163328	GPR155	6.523461392	0.005638581	4.063685687	1.915170464	3.139450982	-2.148515223	-0.924234705	1.224280518	9.961963646	0.002290943	0.084592175	0.010567456	0.057309478	0.377208191	0.249010127	-1.714788081	-4.547656631	-3.317252208	-5.078081325	-5.861387361	-6.308984353	6.167793664	0.012176388

FAM86C1P	-0.211896436	0.165430015	-0.06018516	0.377326451	0.151711277	-0.225615174	8	0.112126744	0.816496581	0.816496581	0.816496581	0.816496581	0.816496581	0.816496581	11	90	0	P_109490	P_109490	P_109490	chr11	71796106	71796166	+	FAM86C1	"family with sequence similarity 86, member C1 [Source:HGNC Symbol;Acc:HGNC:25561]"	ENSG00000158483	protein_coding	3'UTR;exon	multiple Ref	1	1790	11:71796106:71796166	ENSG00000158483	FAM86C1P	11.2124421	0.012303012	-2.33971822	1.826645251	-0.664552537	4.16636347	1.675165683	-2.491197788	9.961963646	0.041450995	0.097824476	0.521421853	0.001945032	0.124956457	0.032007369	-4.6225685	-4.674991384	-6.811173409	-1.677447529	-4.987546066	-4.44122201	5.937229224	0.013692366

POLA1	-0.372181607	-0.153013568	-0.368615708	0.21916804	0.003565899	-0.21560214	8	0.055635989	0.816496581	0.816496581	0.816496581	0.816496581	0.816496581	0.816496581	11	91	0	P_269859	P_269859	P_269859	chrX	24727011	24727071	+	POLA1	"polymerase (DNA directed), alpha 1, catalytic subunit [Source:HGNC Symbol;Acc:HGNC:9173]"	ENSG00000101868	protein_coding	exon	coding exon	1	1791	X:24727011:24727071	ENSG00000101868	POLA1	7.507649687	0.004692429	-6.654289005	-2.735751797	-6.590533773	3.918537208	0.063755232	-3.854781976	9.961963646	5.78E-05	0.021053653	6.26E-05	0.002894156	0.950426275	0.003209763	2.094444025	-3.280431687	1.98091104	-2.08832227	-6.294523737	-2.125097688	20.90289636	0.00012787

CNOT1	-0.025961337	-0.050915545	-0.112223267	-0.024954208	-0.086261931	-0.061307722	8	0.061433851	0.816496581	0.816496581	0.816496581	0.816496581	0.816496581	0.816496581	11	94	0	A_24_P942579	A_24_P942579	A_24_P942579	chr16	58528545	58528604	+	CNOT1	"CCR4-NOT transcription complex, subunit 1 [Source:HGNC Symbol;Acc:HGNC:7877]"	ENSG00000125107	protein_coding	exon;promoter	multiple non-Ref	0	1794	16:58528545:58528604	ENSG00000125107	CNOT1	8.352735402	0.005237507	-0.439349755	-0.861655642	-1.89918053	-0.422305887	-1.459830775	-1.037524888	9.961963646	0.669785134	0.409138518	0.086851846	0.681771294	0.175140731	0.324018864	-6.894042072	-5.805167633	-5.368182104	-7.05295158	-5.273021529	-6.513169828	1.321378564	0.32162569

PANX3	-0.436335166	-0.175669194	-0.412521296	0.260665972	0.02381387	-0.236852102	8	0.110629221	0.816496581	0.816496581	0.816496581	0.816496581	0.816496581	0.816496581	11	96	0	P_110381	P_110381	P_110381	chr11	124619852	124619912	+	PANX3	pannexin 3 [Source:HGNC Symbol;Acc:HGNC:20573]	ENSG00000154143	protein_coding	exon	coding exon	1	1796	11:124619852:124619912	ENSG00000154143	PANX3	6.687067613	0.012035127	-4.871250912	-1.961172943	-4.60539259	2.910077969	0.265858322	-2.644219647	9.961963646	0.000657605	0.078393815	0.000981791	0.015621492	0.795769367	0.024629208	-0.425737574	-4.480454159	-0.880655298	-3.805609174	-6.259228319	-4.184136522	10.71934294	0.001841793

WTAP	-0.115431698	-0.10471088	-0.231885827	0.010720819	-0.116454129	-0.127174947	8	0.076437588	0.816496581	0.816496581	0.816496581	0.816496581	0.816496581	0.816496581	11	98	0	A_23_P371155	A_23_P371155	A_23_P371155	chr6	159756194	159756254	+	WTAP	Wilms tumor 1 associated protein [Source:HGNC Symbol;Acc:HGNC:16846]	ENSG00000146457	protein_coding	3'UTR	3'UTR	1	1798	6:159756194:159756254	ENSG00000146457	WTAP	9.019639385	0.006898695	-1.702108569	-1.544023766	-3.419293475	0.158084803	-1.717184906	-1.875269709	9.961963646	0.11968008	0.15373022	0.006591033	0.877548499	0.116816254	0.090335952	-5.612190958	-5.060716504	-2.839220367	-7.135963645	-4.929279591	-5.42797217	3.910517021	0.043959259

DDR2	-0.075894717	-0.068243169	-0.276852631	0.007651548	-0.200957914	-0.208609462	8	0.132805056	0.816496581	0.816496581	0.816496581	0.816496581	0.816496581	0.816496581	11	100	0	P_204599	P_204599	P_204599	chr1	162759919	162759979	+	DDR2	discoidin domain receptor tyrosine kinase 2 [Source:HGNC Symbol;Acc:HGNC:2731]	ENSG00000162733	protein_coding	exon	coding exon	1	1800	1:162759919:162759979	ENSG00000162733	DDR2	4.886666804	0.016370304	-0.726488613	-0.653245535	-2.650122318	0.073243078	-1.923633705	-1.996876783	9.961963646	0.484259484	0.528385956	0.024381335	0.943062436	0.083418485	0.073879685	-6.71778217	-5.962008233	-4.151256128	-7.146680166	-4.63273468	-5.241586294	2.611818632	0.109420877

MAT2A	-0.185557507	0.031360067	-0.002483413	0.216917574	0.183074094	-0.033843479	8	0.035694827	0.816496581	0.816496581	0.816496581	0.816496581	0.816496581	0.816496581	11	101	0	P_128294	P_128294	P_128294	chr2	85542973	85543033	+	MAT2A	"methionine adenosyltransferase II, alpha [Source:HGNC Symbol;Acc:HGNC:6904]"	ENSG00000168906	protein_coding	exon	coding exon	1	1801	2:85542973:85543033	ENSG00000168906	MAT2A	9.009116371	0.003229872	-3.998816532	0.675818268	-0.05351824	4.6746348	3.945298292	-0.729336508	9.961963646	0.002542347	0.514537789	0.958377027	0.000883618	0.002771528	0.482591358	-1.82182345	-5.946892306	-7.045556659	-0.857605899	-1.416968122	-6.788328097	9.066608176	0.003379915

IQCF3	-0.162543154	0.135098342	-0.07391921	0.297641496	0.088623944	-0.209017552	8	0.281190012	0.816496581	0.816496581	0.816496581	0.816496581	0.816496581	0.816496581	11	102	0	A_32_P152204	A_32_P152204	A_32_P152204	chr3	51830784	51830844	+	IQCF3	IQ motif containing F3 [Source:HGNC Symbol;Acc:HGNC:31816]	ENSG00000229972	protein_coding	"3'UTR;3'UTR,exon;exon"	multiple Ref	1	1802	3:51830784:51830844	ENSG00000229972	IQCF3	4.027868078	0.065702457	-0.776646708	0.645512779	-0.353193042	1.422159487	0.423453666	-0.998705821	9.961963646	0.455412218	0.533180043	0.731303263	0.185526183	0.680961142	0.341577321	-6.678867878	-5.96707861	-6.979422048	-6.143227658	-6.202187466	-6.552252511	0.723979352	0.560483285

TRRAP	-0.006994217	-0.097592481	-0.119745389	-0.090598265	-0.112751172	-0.022152907	8	0.049756625	0.816496581	0.816496581	0.816496581	0.816496581	0.816496581	0.816496581	11	104	0	A_23_P94053	A_23_P94053	A_23_P94053	chr7	99012730	99012790	+	TRRAP	transformation/transcription domain-associated protein [Source:HGNC Symbol;Acc:HGNC:12347]	ENSG00000196367	protein_coding	3'UTR	3'UTR	1	1804	7:99012730:99012790	ENSG00000196367	TRRAP	6.816871724	0.004194824	-0.132259803	-1.845462144	-2.264370967	-1.713202341	-2.132111165	-0.418908823	9.961963646	0.897412024	0.094860939	0.047113916	0.117566667	0.058914433	0.684171575	-6.988823151	-4.648155066	-4.792221935	-5.744048618	-4.318877916	-6.976351164	2.70117399	0.102238665

SGMS1	0.219749116	-0.034881392	0.33117361	-0.254630508	0.111424494	0.366055003	8	0.062711406	0.816496581	0.816496581	0.816496581	0.816496581	0.816496581	0.816496581	11	105	0	A_32_P163125	A_32_P163125	A_32_P163125	chr10	50305799	50305859	-	SGMS1	sphingomyelin synthase 1 [Source:HGNC Symbol;Acc:HGNC:29799]	ENSG00000198964	protein_coding	3'UTR	3'UTR	1	1805	10:50305799:50305859	ENSG00000198964	SGMS1	8.214323391	0.005364873	3.674455939	-0.583256679	5.537600615	-4.257712618	1.863144677	6.120857294	9.961963646	0.004312816	0.572687101	0.000251794	0.001683438	0.092151918	0.000114376	-2.363342048	-6.005869015	0.53353977	-1.527731064	-4.721263089	1.337484178	17.26185904	0.000284512

BTNL8	0.274266253	-0.054391232	0.081224432	-0.328657486	-0.193041822	0.135615664	8	0.328869031	0.816496581	0.816496581	0.816496581	0.816496581	0.816496581	0.816496581	11	107	0	P_141427	P_141427	P_141427	chr5	180947680	180947740	+	BTNL8	butyrophilin-like 8 [Source:HGNC Symbol;Acc:HGNC:26131]	ENSG00000113303	protein_coding	3'UTR;exon	multiple Ref	1	1807	5:180947680:180947740	ENSG00000113303	BTNL8	4.267254428	0.089060917	1.125574955	-0.223218891	0.333340996	-1.348793846	-0.792233959	0.556559887	9.961963646	0.28673147	0.827875763	0.745786573	0.207263658	0.446679364	0.590110801	-6.347900757	-6.155888424	-6.986777317	-6.236323958	-5.973284488	-6.904751983	0.697416711	0.574657438

FAM166B	0.035539909	0.258795854	0.28432519	0.223255946	0.248785281	0.025529336	8	0.391987145	0.816496581	0.816496581	0.816496581	0.816496581	0.816496581	0.816496581	11	108	0	P_148695	P_148695	P_148695	chr9	35562947	35563007	-	FAM166B	"family with sequence similarity 166, member B [Source:HGNC Symbol;Acc:HGNC:34242]"	ENSG00000215187	protein_coding	exon	coding exon	1	1808	9:35562947:35563007	ENSG00000215187	FAM166B	4.04649061	0.125599162	0.122819851	0.894354248	0.98257927	0.771534398	0.859759419	0.088225021	9.961963646	0.904692232	0.392227652	0.349075422	0.458300487	0.410134398	0.931446182	-6.990134983	-5.777163388	-6.54405967	-6.833493869	-5.917886626	-7.06712796	0.520441916	0.677789978

STS	-0.452879396	0.12079565	-0.406334249	0.573675046	0.046545146	-0.527129899	8	0.054355086	0.816496581	0.816496581	0.816496581	0.816496581	0.816496581	0.816496581	11	109	0	P_269430	P_269430	P_269430	chrX	7257528	7257588	+	STS	"steroid sulfatase (microsomal), isozyme S [Source:HGNC Symbol;Acc:HGNC:11425]"	ENSG00000101846	protein_coding	exon	coding exon	1	1809	X:7257528:7257588	ENSG00000101846	STS	5.708471538	0.004579289	-8.196513788	2.186240355	-7.354108643	10.38275414	0.842405144	-9.540348998	9.961963646	9.74E-06	0.053776141	2.49E-05	1.16E-06	0.419325986	2.51E-06	3.926520709	-4.143025066	2.935576656	6.040746216	-5.932498042	5.280524974	54.26248135	1.78E-06

RNF220	-0.015940845	-0.003538999	0.13119753	0.012401845	0.147138375	0.13473653	8	0.050762308	0.816496581	0.816496581	0.816496581	0.816496581	0.816496581	0.816496581	11	110	0	P_101627	P_101627	P_101627	chr1	44632026	44632086	+	RNF220	ring finger protein 220 [Source:HGNC Symbol;Acc:HGNC:25552]	ENSG00000187147	protein_coding	5'UTR;exon	multiple Ref	1	1810	1:44632026:44632086	ENSG00000187147	RNF220	9.127551458	0.004276004	-0.298564302	-0.066283747	2.457266233	0.232280555	2.755830535	2.52354998	9.961963646	0.771407371	0.9484633	0.033919145	0.821022397	0.020341658	0.030283824	-6.949935074	-6.179771809	-4.474694977	-7.120187382	-3.328146199	-4.387125103	3.358092035	0.063588251

NEK9	-0.038782619	-0.018715143	-0.153719625	0.020067476	-0.114937006	-0.135004482	8	0.060813981	0.816496581	0.816496581	0.816496581	0.816496581	0.816496581	0.816496581	11	111	0	A_23_P3131	A_23_P3131	A_23_P3131	chr14	75084350	75084410	-	NEK9	NIMA-related kinase 9 [Source:HGNC Symbol;Acc:HGNC:18591]	ENSG00000119638	protein_coding	3'UTR;exon	multiple Ref	1	1811	14:75084350:75084410	ENSG00000119638	NEK9	6.566368415	0.005176653	-0.660173737	-0.318576888	-2.616678856	0.341596849	-1.956505118	-2.298101968	9.961963646	0.52411231	0.756626081	0.025819369	0.739750393	0.079002789	0.044491407	-6.765628449	-6.128866149	-4.20767704	-7.086173158	-4.5841096	-4.760471115	2.768367029	0.097202629

ACTN4	0.086618283	-0.093728485	-0.001238203	-0.180346768	-0.087856486	0.092490282	8	0.095483365	0.816496581	0.816496581	0.816496581	0.816496581	0.816496581	0.816496581	11	112	0	P_125692	P_125692	P_125692	chr19	38730464	38730524	+	ACTN4	"actinin, alpha 4 [Source:HGNC Symbol;Acc:HGNC:166]"	ENSG00000130402	protein_coding	3'UTR	3'UTR	1	1812	19:38730464:38730524	ENSG00000130402	ACTN4	8.861972692	0.009528191	1.086800875	-1.176012677	-0.015535746	-2.262813552	-1.102336621	1.160476931	9.961963646	0.30272104	0.266928306	0.987911505	0.047238557	0.296233712	0.272909257	-6.389547851	-5.50130221	-7.046988795	-4.893388168	-5.688056122	-6.381647345	1.707760204	0.228370308

FFAR1	-0.072043531	-0.042148779	0.241453046	0.029894752	0.313496577	0.283601824	8	0.341145143	0.816496581	0.816496581	0.816496581	0.816496581	0.816496581	0.816496581	11	113	0	A_23_P39356	A_23_P39356	A_23_P39356	chr19	35352375	35352435	+	FFAR1	free fatty acid receptor 1 [Source:HGNC Symbol;Acc:HGNC:4498]	ENSG00000126266	protein_coding	exon	coding exon	1	1813	19:35352375:35352435	ENSG00000126266	FFAR1	5.703682557	0.095666177	-0.285273487	-0.166898109	0.956090732	0.118375378	1.24136422	1.122988841	9.961963646	0.781277716	0.870789287	0.361652213	0.90812312	0.242905531	0.287777054	-6.954133244	-6.167424417	-6.569668396	-7.141955387	-5.536494241	-6.422961863	0.639907871	0.606515907

PARP2	-0.292285307	0.001172939	-0.129358582	0.293458246	0.162926725	-0.130531521	8	0.052398087	0.816496581	0.816496581	0.816496581	0.816496581	0.816496581	0.816496581	11	114	0	P_114274	P_114274	P_114274	chr14	20357815	20357875	+	PARP2	poly (ADP-ribose) polymerase 2 [Source:HGNC Symbol;Acc:HGNC:272]	ENSG00000129484	protein_coding	3'UTR	3'UTR	1	1814	14:20357815:20357875	ENSG00000129484	PARP2	10.26859949	0.004411518	-5.389625441	0.021628536	-2.385321085	5.411253977	3.004304356	-2.406949621	9.961963646	0.000309924	0.983171353	0.038352097	0.000300598	0.013299856	0.0369628	0.354279201	-6.181840823	-4.593945715	0.268406649	-2.92409696	-4.581333062	13.13986794	0.000848976

FBN2	0.145790303	0.117110083	0.146729194	-0.02868022	0.000938891	0.029619111	8	0.066722949	0.816496581	0.816496581	0.816496581	0.816496581	0.816496581	0.816496581	11	115	0	P_254657	P_254657	P_254657	chr5	128392017	128392077	-	FBN2	fibrillin 2 [Source:HGNC Symbol;Acc:HGNC:3604]	ENSG00000138829	protein_coding	exon	coding exon	1	1815	5:128392017:128392077	ENSG00000138829	FBN2	3.616676642	0.005781844	2.348232413	1.886282471	2.363355052	-0.461949942	0.015122639	0.477072581	9.961963646	0.040854415	0.088715418	0.039815401	0.654040745	0.98823292	0.643603525	-4.608593151	-4.589505947	-4.630171644	-7.034152783	-6.296563619	-6.94844978	2.516488137	0.117748712

KIAA1958	0.158832717	-0.043699277	-0.007341946	-0.202531994	-0.166174663	0.036357331	8	0.086281924	0.816496581	0.816496581	0.816496581	0.816496581	0.816496581	0.816496581	11	116	0	P_149411	P_149411	P_149411	chr9	112574306	112574366	+	KIAA1958	KIAA1958 [Source:HGNC Symbol;Acc:HGNC:23427]	ENSG00000165185	protein_coding	exon	coding exon	1	1816	9:112574306:112574366	ENSG00000165185	KIAA1958	6.851591071	0.00818508	2.150176615	-0.591573101	-0.099390607	-2.741749716	-2.249567222	0.492182494	9.961963646	0.057149287	0.567317506	0.922799756	0.020838385	0.048311667	0.633255678	-4.92957909	-6.000897992	-7.041728549	-4.09294727	-4.137085406	-6.940634596	2.96718075	0.083931152

STK3	0.343248286	-0.006991906	0.306413427	-0.350240191	-0.036834859	0.313405333	8	0.073324639	0.816496581	0.816496581	0.816496581	0.816496581	0.816496581	0.816496581	11	117	0	A_16_P18411211	A_16_P18411211	A_16_P18411211	chr8	98450781	98450841	+	STK3	serine/threonine kinase 3 [Source:HGNC Symbol;Acc:HGNC:11406]	ENSG00000104375	protein_coding	3'ext;intron	multiple non-Ref	0	1817	8:98450781:98450841	ENSG00000104375	STK3	6.332449576	0.006524309	5.204592723	-0.106016617	4.646074456	-5.31060934	-0.558518267	4.752091073	9.961963646	0.000403604	0.917673604	0.000922786	0.000346719	0.588823098	0.000786021	0.080315684	-6.176165746	-0.816385827	0.119108837	-6.133396471	-0.669924742	16.63031943	0.000331469

COBL	0.481075499	0.019428484	0.287992165	-0.461647015	-0.193083335	0.268563681	8	0.079191128	0.816496581	0.816496581	0.816496581	0.816496581	0.816496581	0.816496581	11	118	0	P_144851	P_144851	P_144851	chr7	51017064	51017124	-	COBL	cordon-bleu WH2 repeat protein [Source:HGNC Symbol;Acc:HGNC:22199]	ENSG00000106078	protein_coding	3'UTR	3'UTR	1	1818	7:51017064:51017124	ENSG00000106078	COBL	7.856692088	0.007242827	6.923166876	0.279595694	4.144500849	-6.643571182	-2.778666027	3.864905155	9.961963646	4.16E-05	0.785506873	0.00201378	5.86E-05	0.019561249	0.003157338	2.435628523	-6.14104402	-1.62377285	1.979637747	-3.291087719	-2.10816901	21.99680257	0.000102904

MINDY1	-0.0585434	-0.058254966	-0.111603161	0.000288434	-0.053059761	-0.053348195	8	0.062079668	0.816496581	0.816496581	0.816496581	0.816496581	0.816496581	0.816496581	11	119	0	A_24_P942163	A_24_P942163	A_24_P942163	chr1	150993245	150993305	-	FAM63A;ANXA9	"family with sequence similarity 63, member A [Source:HGNC Symbol;Acc:HGNC:25648];annexin A9 [Source:HGNC Symbol;Acc:HGNC:547]"	ENSG00000143409	protein_coding	3'ext;intron	multiple non-Ref	0	1819	1:150993245:150993305	ENSG00000143409	MINDY1	6.882826574	0.005301564	-0.984739972	-0.979888314	-1.877241395	0.004851658	-0.892501423	-0.897353081	9.961963646	0.348063832	0.35033828	0.090043811	0.996224734	0.393172629	0.390701568	-6.493590054	-5.699795796	-5.401414388	-7.149600191	-5.889627173	-6.6484666	1.175959009	0.367339403

SERPINB9	0.135181945	-0.127189647	0.201921534	-0.262371592	0.066739589	0.329111182	8	0.132190832	0.816496581	0.816496581	0.816496581	0.816496581	0.816496581	0.816496581	11	120	0	P_256011	P_256011	P_256011	chr6	2893410	2893470	-	SERPINB9	"serpin peptidase inhibitor, clade B (ovalbumin), member 9 [Source:HGNC Symbol;Acc:HGNC:8955]"	ENSG00000170542	protein_coding	exon	coding exon	1	1820	6:2893410:2893470	ENSG00000170542	SERPINB9	4.387771362	0.016239593	1.299202209	-1.222390096	1.940620868	-2.521592305	0.641418659	3.163010965	9.961963646	0.223137194	0.249693199	0.081108487	0.030385458	0.53572855	0.010153346	-6.148051338	-5.450370756	-5.304927836	-4.465072105	-6.082401748	-3.299279344	3.953776216	0.042752029

NCAM2	0.35001683	-0.200288384	0.246475289	-0.550305214	-0.103541542	0.446763673	8	0.085251949	0.816496581	0.816496581	0.816496581	0.816496581	0.816496581	0.816496581	11	121	0	A_14_P113154	A_14_P113154	A_14_P113154	chr21	21157832	21157892	+	NCAM2	neural cell adhesion molecule 2 [Source:HGNC Symbol;Acc:HGNC:7657]	ENSG00000154654	protein_coding	intron	intron sense	0	1821	21:21157832:21157892	ENSG00000154654	NCAM2	8.809613604	0.0080432	4.779914523	-2.735186631	3.36592618	-7.515101154	-1.413988343	6.101112811	9.961963646	0.000753817	0.021074052	0.007208279	2.07E-05	0.187847121	0.000117384	-0.567161826	-3.281334155	-2.930187965	3.065975596	-5.330664465	1.310438648	22.89299562	8.67E-05

BARD1	0.300160782	0.032789366	0.375923963	-0.267371415	0.075763182	0.343134597	8	0.067882761	0.816496581	0.816496581	0.816496581	0.816496581	0.816496581	0.816496581	11	122	0	P_239158	P_239158	P_239158	chr2	214745066	214745126	-	BARD1	BRCA1 associated RING domain 1 [Source:HGNC Symbol;Acc:HGNC:952]	ENSG00000138376	protein_coding	3'UTR;exon	multiple Ref	1	1822	2:214745066:214745126	ENSG00000138376	BARD1	10.97573408	0.005907215	4.783086029	0.522501174	5.990378382	-4.260584856	1.207292352	5.467877208	9.961963646	0.000750237	0.612742387	0.00013591	0.00167584	0.255202899	0.000277577	-0.562231479	-6.040183369	1.175233079	-1.523039634	-5.574872865	0.413487178	18.09056198	0.000234432

TP63	0.023254595	0.131318097	0.102222777	0.108063502	0.078968182	-0.02909532	8	0.064334677	0.816496581	0.816496581	0.816496581	0.816496581	0.816496581	0.816496581	11	124	0	P_136851	P_136851	P_136851	chr3	189873512	189873572	+	TP63	tumor protein p63 [Source:HGNC Symbol;Acc:HGNC:15979]	ENSG00000073282	protein_coding	exon	non-coding exon	1	1824	3:189873512:189873572	ENSG00000073282	TP63	4.002059353	0.005530487	0.382977018	2.162661351	1.683494161	1.779684334	1.300517143	-0.47916719	9.961963646	0.709781792	0.05595918	0.123303474	0.105596324	0.222703649	0.642164206	-6.918911129	-4.178969548	-5.68641397	-5.647010669	-5.468051756	-6.947380282	2.124358412	0.160868446

SLC8A1	0.063006264	-0.017242178	-0.039436109	-0.080248442	-0.102442373	-0.022193931	8	0.111636084	0.816496581	0.816496581	0.816496581	0.816496581	0.816496581	0.816496581	11	125	0	A_24_P67948	A_24_P67948	A_24_P67948	chr2	40362196	40362256	-	SLC8A1	"solute carrier family 8 (sodium/calcium exchanger), member 1 [Source:HGNC Symbol;Acc:HGNC:11068]"	ENSG00000183023	protein_coding	intron	intron sense	0	1825	2:40362196:40362256	ENSG00000183023	SLC8A1	3.730689015	0.012214844	0.698208934	-0.191070573	-0.437014384	-0.889279508	-1.135223318	-0.24594381	9.961963646	0.501019132	0.852308233	0.67142185	0.394819662	0.282856728	0.810718831	-6.738693382	-6.162878111	-6.94381808	-6.733547679	-5.65343096	-7.03843415	0.475837272	0.706094695

ITGAX	0.243709968	0.226665339	0.161356532	-0.017044629	-0.082353437	-0.065308807	8	0.147601737	0.816496581	0.816496581	0.816496581	0.816496581	0.816496581	0.816496581	11	127	0	P_118872	P_118872	P_118872	chr16	31381811	31381871	+	ITGAX	"integrin, alpha X (complement component 3 receptor 4 subunit) [Source:HGNC Symbol;Acc:HGNC:6152]"	ENSG00000140678	protein_coding	exon	coding exon	1	1827	16:31381811:31381871	ENSG00000140678	ITGAX	3.697379011	0.019702249	2.126478629	1.977756606	1.40790801	-0.148722023	-0.718570619	-0.569848596	9.961963646	0.059475375	0.076265751	0.189590206	0.884740439	0.488916328	0.581402596	-4.967343415	-4.456066132	-6.060575251	-7.137530827	-6.029158228	-6.896828787	1.879804485	0.197131976

ZCCHC8	0.044072653	0.116537181	0.088787904	0.072464529	0.044715251	-0.027749277	8	0.052715933	0.816496581	0.816496581	0.816496581	0.816496581	0.816496581	0.816496581	11	128	0	P_113120	P_113120	P_113120	chr12	122473499	122473559	-	ZCCHC8	"zinc finger, CCHC domain containing 8 [Source:HGNC Symbol;Acc:HGNC:25265]"	ENSG00000033030	protein_coding	exon	coding exon	1	1828	12:122473499:122473559	ENSG00000033030	ZCCHC8	6.712648222	0.004438348	0.810222208	2.142394581	1.632257808	1.332172373	0.8220356	-0.510136773	9.961963646	0.43673923	0.057903352	0.133792219	0.212475508	0.430291972	0.621067088	-6.651536367	-4.209764131	-5.7589775	-6.256946722	-5.949319787	-6.931046914	1.770207567	0.216412597

ZFYVE9	0.081823323	-0.005633326	0.02674579	-0.087456649	-0.055077533	0.032379116	8	0.042872729	0.816496581	0.816496581	0.816496581	0.816496581	0.816496581	0.816496581	11	129	0	A_23_P251974	A_23_P251974	A_23_P251974	chr1	52263834	52263894	+	ZFYVE9	"zinc finger, FYVE domain containing 9 [Source:HGNC Symbol;Acc:HGNC:6775]"	ENSG00000157077	protein_coding	exon	non-coding exon	1	1829	1:52263834:52263894	ENSG00000157077	ZFYVE9	5.819433642	0.003682755	1.651339317	-0.11369048	0.539777326	-1.765029798	-1.111561991	0.653467807	9.961963646	0.129795993	0.91174175	0.601207032	0.108135083	0.292432607	0.52824853	-5.684478427	-6.175278055	-6.890305522	-5.668566954	-5.678422137	-6.842991659	1.301521175	0.327476339

SBNO2	0.039767474	0.044054348	0.039121547	0.004286875	-0.000645927	-0.004932801	8	0.089052441	0.816496581	0.816496581	0.816496581	0.816496581	0.816496581	0.816496581	11	130	0	P_123939	P_123939	P_123939	chr19	1132089	1132149	-	SBNO2	strawberry notch homolog 2 (Drosophila) [Source:HGNC Symbol;Acc:HGNC:29158]	ENSG00000064932	protein_coding	exon	coding exon	1	1830	19:1132089:1132149	ENSG00000064932	SBNO2	8.947800957	0.008575177	0.525959229	0.582656854	0.51741629	0.056697625	-0.008542939	-0.065240564	9.961963646	0.610424382	0.573075461	0.616159038	0.955907143	0.993352462	0.949273117	-6.849478544	-6.006225019	-6.902860554	-7.14785538	-6.296646439	-7.069053777	0.148564279	0.928243481

CPN2	-0.174969464	-0.072954608	0.066877404	0.102014857	0.241846868	0.139832012	8	0.267948013	0.816496581	0.816496581	0.816496581	0.816496581	0.816496581	0.816496581	11	132	0	A_24_P137461	A_24_P137461	A_24_P137461	chr3	194340606	194340666	-	CPN2	"carboxypeptidase N, polypeptide 2 [Source:HGNC Symbol;Acc:HGNC:2313]"	ENSG00000178772	protein_coding	3'UTR	3'UTR	1	1832	3:194340606:194340666	ENSG00000178772	CPN2	4.325355058	0.059862897	-0.875848571	-0.365190515	0.334769721	0.510658056	1.210618292	0.699960236	9.961963646	0.401737407	0.722603323	0.74474067	0.620714972	0.253980822	0.499970996	-6.595214097	-6.112267595	-6.986261847	-7.008851406	-5.571160905	-6.810139028	0.538143038	0.666804514

NUP133	-0.04871292	-0.02380401	0.005612754	0.024908911	0.054325675	0.029416764	8	0.115876148	0.816496581	0.816496581	0.816496581	0.816496581	0.816496581	0.816496581	11	136	0	P_206577	P_206577	P_206577	chr1	229475637	229475697	-	NUP133	nucleoporin 133kDa [Source:HGNC Symbol;Acc:HGNC:18016]	ENSG00000069248	protein_coding	exon	coding exon	1	1836	1:229475637:229475697	ENSG00000069248	NUP133	7.697877702	0.012989523	-0.523471674	-0.255799175	0.060314963	0.2676725	0.583786637	0.316114137	9.961963646	0.612091387	0.803310179	0.953097634	0.794411694	0.572344096	0.758439662	-6.850864457	-6.147710395	-7.045134276	-7.110571938	-6.118547197	-7.017061335	0.142579543	0.93208996

BRD8	0.086519684	-0.025895644	-0.042631201	-0.112415328	-0.129150885	-0.016735557	8	0.06623415	0.816496581	0.816496581	0.816496581	0.816496581	0.816496581	0.816496581	11	138	0	P_254974	P_254974	P_254974	chr5	138160906	138160966	-	BRD8	bromodomain containing 8 [Source:HGNC Symbol;Acc:HGNC:19874]	ENSG00000112983	protein_coding	3'UTR;exon	multiple Ref	1	1838	5:138160906:138160966	ENSG00000112983	BRD8	7.633530981	0.005729654	1.399897869	-0.418994323	-0.689777447	-1.818892192	-2.089675316	-0.270783124	9.961963646	0.191907518	0.684111117	0.506084094	0.099070193	0.063268221	0.792085479	-6.022871671	-6.09037887	-6.793344055	-5.588892035	-4.383751783	-7.031469375	1.726583074	0.224687575

LPCAT3	-0.099290175	-0.01685041	0.037971237	0.082439765	0.137261412	0.054821647	8	0.114057548	0.816496581	0.816496581	0.816496581	0.816496581	0.816496581	0.816496581	11	139	0	P_110840	P_110840	P_110840	chr12	6977147	6977207	-	LPCAT3	lysophosphatidylcholine acyltransferase 3 [Source:HGNC Symbol;Acc:HGNC:30244]	ENSG00000111684	protein_coding	3'UTR;exon	multiple Ref	1	1839	12:6977147:6977207	ENSG00000111684	LPCAT3	9.00288939	0.01265372	-1.081042569	-0.183462368	0.413419787	0.897580201	1.494462356	0.596882155	9.961963646	0.305153128	0.858115551	0.688057792	0.390586158	0.166039174	0.563904288	-6.395635283	-6.164374807	-6.9545792	-6.726041815	-5.228704648	-6.880171386	0.794760609	0.524360471

LIN7C	0.130943309	0.058611435	0.108069046	-0.072331874	-0.022874263	0.049457611	8	0.102855509	0.816496581	0.816496581	0.816496581	0.816496581	0.816496581	0.816496581	11	140	0	P_211371	P_211371	P_211371	chr11	27498435	27498495	+	LIN7C	lin-7 homolog C (C. elegans) [Source:HGNC Symbol;Acc:HGNC:17789]	ENSG00000148943	protein_coding	3'UTR	3'UTR antisense	0	1840	11:27498435:27498495	ENSG00000148943	LIN7C	7.151072314	0.010702403	1.550201977	0.69388473	1.279399843	-0.856317247	-0.270802134	0.585515113	9.961963646	0.152253281	0.503612881	0.229750896	0.411946449	0.792071269	0.571226159	-5.824704178	-5.934458238	-6.219975282	-6.76276432	-6.257827609	-6.887263241	0.945151186	0.455246921

CXorf21	0.187953308	0.278994822	0.235507056	0.091041514	0.047553748	-0.043487766	8	0.261005855	0.816496581	0.816496581	0.816496581	0.816496581	0.816496581	0.816496581	11	143	0	P_150675	P_150675	P_150675	chrX	30559459	30559519	-	CXorf21	chromosome X open reading frame 21 [Source:HGNC Symbol;Acc:HGNC:25667]	ENSG00000120280	protein_coding	exon	coding exon	1	1843	X:30559459:30559519	ENSG00000120280	CXorf21	3.386484051	0.056914016	0.964908179	1.432293948	1.209037963	0.46738577	0.244129784	-0.223255985	9.961963646	0.357429619	0.182681656	0.254560907	0.650279915	0.812084668	0.827847677	-6.512836685	-5.202789265	-6.302631127	-7.031449135	-6.26508275	-7.044217365	0.795363098	0.524063143

DET1	0.100231527	0.045561468	0.036310098	-0.054670059	-0.06392143	-0.009251371	8	0.123211011	0.816496581	0.816496581	0.816496581	0.816496581	0.816496581	0.816496581	11	144	0	P_117424	P_117424	P_117424	chr15	88546539	88546599	-	DET1	de-etiolated homolog 1 (Arabidopsis) [Source:HGNC Symbol;Acc:HGNC:25477]	ENSG00000140543	protein_coding	5'UTR	5'UTR	1	1844	15:88546539:88546599	ENSG00000140543	DET1	6.794335375	0.014397817	1.023061281	0.465045035	0.370616471	-0.558016246	-0.65244481	-0.094428564	9.961963646	0.330480168	0.651898121	0.718682195	0.589153051	0.528881202	0.92664104	-6.455493658	-6.069348568	-6.972625006	-6.981951457	-6.075129797	-7.066509466	0.357710159	0.784838853

BBS1	0.051501191	-0.0615191	-0.000323436	-0.113020291	-0.051824627	0.061195664	8	0.04729866	0.816496581	0.816496581	0.816496581	0.816496581	0.816496581	0.816496581	11	146	0	P_212167	P_212167	P_212167	chr11	66514582	66514642	+	BBS1;CTD-3074O7.11	Bardet-Biedl syndrome 1 [Source:HGNC Symbol;Acc:HGNC:966];Bardet-Biedl syndrome 1 protein [Source:UniProtKB/TrEMBL;Acc:E9PPR3]	ENSG00000174483	protein_coding	3'UTR;exon	multiple Ref	1	1846	11:66514582:66514642	ENSG00000174483	BBS1	7.602666853	0.004003248	0.996911608	-1.190828861	-0.006260762	-2.187740469	-1.003172371	1.184568099	9.961963646	0.342405617	0.26132188	0.995128267	0.053640062	0.339521786	0.263679377	-6.481618412	-5.485189008	-7.047099196	-5.015065647	-5.787602972	-6.354553506	1.601285012	0.250557315

MYO10	0.046970591	0.089256064	0.211681893	0.042285474	0.164711303	0.122425829	8	0.053718153	0.816496581	0.816496581	0.816496581	0.816496581	0.816496581	0.816496581	11	147	0	A_24_P924183	A_24_P924183	A_24_P924183	chr5	16814820	16814880	-	MYO10	myosin X [Source:HGNC Symbol;Acc:HGNC:7593]	ENSG00000145555	protein_coding	3'UTR	3'UTR	1	1847	5:16814820:16814880	ENSG00000145555	MYO10	6.461101203	0.00452401	0.855283041	1.625255234	3.854495579	0.769972193	2.999212538	2.229240345	9.961963646	0.412491954	0.135286118	0.00321126	0.459185442	0.013415858	0.05000442	-6.613271488	-4.953463623	-2.104159335	-6.834736244	-2.932384809	-4.872524786	5.464624227	0.017569089

TMEM8B	0.135722916	0.142925999	0.099552898	0.007203083	-0.036170017	-0.043373101	8	0.109916879	0.816496581	0.816496581	0.816496581	0.816496581	0.816496581	0.816496581	11	148	0	A_24_P912730	A_24_P912730	A_24_P912730	chr9	35855889	35855949	+	TMEM8B	transmembrane protein 8B [Source:HGNC Symbol;Acc:HGNC:21427]	ENSG00000137103	protein_coding	3'ext	3'extended sense	0	1848	9:35855889:35855949	ENSG00000137103	TMEM8B	7.594221641	0.011908964	1.523217135	1.604057275	1.117281335	0.080840141	-0.4059358	-0.486775941	9.961963646	0.158797007	0.13989944	0.290095154	0.937170119	0.693371798	0.636948924	-5.861217947	-4.981755731	-6.405143302	-7.146040399	-6.209779758	-6.943457677	1.091629597	0.397113898

PHRF1	-0.041764275	0.0169814	-0.026880025	0.058745676	0.014884251	-0.043861425	8	0.074278426	0.816496581	0.816496581	0.816496581	0.816496581	0.816496581	0.816496581	11	149	0	P_210553	P_210553	P_210553	chr11	587337	587397	+	PHRF1	PHD and ring finger domains 1 [Source:HGNC Symbol;Acc:HGNC:24351]	ENSG00000070047	protein_coding	exon	coding exon	1	1849	11:587337:587397	ENSG00000070047	PHRF1	8.599468574	0.006637364	-0.62784545	0.255282649	-0.404089402	0.883128099	0.223756048	-0.659372051	9.961963646	0.544228135	0.803697971	0.694685535	0.397977622	0.82746908	0.524605778	-6.787434636	-6.147848618	-6.958675522	-6.739071723	-6.270124325	-6.83893389	0.314566816	0.81461684

MKLN1	0.071750851	-0.032985979	0.191921229	-0.10473683	0.120170378	0.224907208	8	0.069198178	0.816496581	0.816496581	0.816496581	0.816496581	0.816496581	0.816496581	11	150	0	P_262934	P_262934	P_262934	chr7	131487662	131487722	+	MKLN1	"muskelin 1, intracellular mediator containing kelch motifs [Source:HGNC Symbol;Acc:HGNC:7109]"	ENSG00000128585	protein_coding	3'UTR;exon	multiple Ref	1	1850	7:131487662:131487722	ENSG00000128585	MKLN1	7.976102331	0.006052021	1.129594289	-0.519307762	3.02147114	-1.64890205	1.891876851	3.540778901	9.961963646	0.285112313	0.614886985	0.012916227	0.130300403	0.087902593	0.005381365	-6.343518871	-6.041888781	-3.519110832	-5.835983962	-4.679370037	-2.654543467	4.918357146	0.023814374

CCDC183	0.331665876	0.123904996	0.204182173	-0.207760879	-0.127483703	0.080277177	8	0.056332896	0.816496581	0.816496581	0.816496581	0.816496581	0.816496581	0.816496581	11	151	0	P_150196	P_150196	P_150196	chr9	136799589	136799649	+	CCDC183;RP11-216L13.19	coiled-coil domain containing 183 [Source:HGNC Symbol;Acc:HGNC:28236];	ENSG00000213213	protein_coding;processed_transcript	exon	non-coding exon	1	1851	9:136799589:136799649	ENSG00000213213	CCDC183	7.880669383	0.004755093	5.89069988	2.200670015	3.626468657	-3.690029865	-2.264231223	1.425798642	9.961963646	0.000155295	0.052480858	0.004668457	0.004203554	0.047125087	0.184500423	1.071138113	-4.120967984	-2.487559618	-2.472542409	-4.114180133	-6.061432767	12.24508895	0.001114957

TXNL4B	-0.107565805	0.146802241	-0.12349487	0.254368046	-0.015929066	-0.270297111	8	0.118887653	0.816496581	0.816496581	0.816496581	0.816496581	0.816496581	0.816496581	11	152	0	P_226966	P_226966	P_226966	chr16	72086636	72086696	-	TXNL4B	thioredoxin-like 4B [Source:HGNC Symbol;Acc:HGNC:26041]	ENSG00000140830	protein_coding	exon	coding exon	1	1852	16:72086636:72086696	ENSG00000140830	TXNL4B	6.416178158	0.013557277	-1.131445477	1.544159244	-1.298997509	2.67560472	-0.167552032	-2.843156753	9.961963646	0.284368984	0.153697699	0.223204748	0.023339515	0.870288199	0.017516116	-6.341496651	-5.060540303	-6.19635506	-4.205291756	-6.281774939	-3.846339114	3.437077632	0.060222452

SOS2	0.361085561	-0.09921752	0.30492117	-0.460303081	-0.056164391	0.40413869	8	0.056124958	0.816496581	0.816496581	0.816496581	0.816496581	0.816496581	0.816496581	11	154	0	P_220700	P_220700	P_220700	chr14	50180571	50180631	-	SOS2	son of sevenless homolog 2 (Drosophila) [Source:HGNC Symbol;Acc:HGNC:11188]	ENSG00000100485	protein_coding	exon	coding exon	1	1854	14:50180571:50180631	ENSG00000100485	SOS2	7.109212459	0.004736315	6.425922525	-1.765687046	5.426414202	-8.19160957	-0.999508323	7.192101248	9.961963646	7.71E-05	0.108020036	0.00029424	9.80E-06	0.341207328	3.01E-05	1.796958866	-4.761022239	0.371425708	3.845097054	-5.791137166	2.724722661	32.28065112	1.92E-05

CAMK4	0.026167335	0.090838824	0.136621126	0.064671489	0.110453791	0.045782302	8	0.208303696	0.816496581	0.816496581	0.816496581	0.816496581	0.816496581	0.816496581	12	16	0	P_254305	P_254305	P_254305	chr5	111482876	111482936	+	CAMK4	calcium/calmodulin-dependent protein kinase IV [Source:HGNC Symbol;Acc:HGNC:1464]	ENSG00000152495	protein_coding	3'UTR;exon	multiple Ref	1	1886	5:111482876:111482936	ENSG00000152495	CAMK4	3.934975747	0.037051565	0.16649525	0.577981391	0.869281047	0.411486141	0.702785797	0.291299656	9.961963646	0.871098019	0.576107568	0.405150535	0.689429086	0.498282768	0.776797243	-6.983254544	-6.008988272	-6.649465289	-7.05779997	-6.040496554	-7.025219381	0.310919484	0.81714897

CSE1L	-0.208025967	-0.040192697	-0.194698506	0.16783327	0.013327461	-0.154505809	8	0.053008015	0.816496581	0.816496581	0.816496581	0.816496581	0.816496581	0.816496581	12	18	0	P_241051	P_241051	P_241051	chr20	49072637	49072697	+	CSE1L	CSE1 chromosome segregation 1-like (yeast) [Source:HGNC Symbol;Acc:HGNC:2431]	ENSG00000124207	protein_coding	exon	coding exon	1	1888	20:49072637:49072697	ENSG00000124207	CSE1L	10.43444621	0.004463146	-3.8136656	-0.736838323	-3.569338028	3.076827277	0.244327571	-2.832499705	9.961963646	0.003432235	0.478214562	0.005131978	0.011754207	0.811935716	0.017838574	-2.129728201	-5.903711386	-2.584262302	-3.519787169	-6.26503168	-3.864507577	7.562794435	0.006310025

EXOC6B	0.092021723	-0.107845296	0.070896358	-0.19986702	-0.021125365	0.178741655	8	0.079458049	0.816496581	0.816496581	0.816496581	0.816496581	0.816496581	0.816496581	12	21	0	P_235488	P_235488	P_235488	chr2	72515042	72515102	-	EXOC6B	exocyst complex component 6B [Source:HGNC Symbol;Acc:HGNC:17085]	ENSG00000144036	protein_coding	3'UTR;exon	multiple Ref	1	1891	2:72515042:72515102	ENSG00000144036	EXOC6B	6.230350192	0.007276834	1.321188392	-1.548373022	1.017884063	-2.869561414	-0.303304329	2.566257085	9.961963646	0.215979343	0.152689199	0.332816271	0.016742284	0.767897442	0.028148606	-6.121271768	-5.055055329	-6.509037059	-3.874920064	-6.247987145	-4.315490627	3.348522901	0.064011007

SMPDL3B	0.107363535	0.049031916	0.047464106	-0.058331618	-0.059899429	-0.001567811	8	0.067053247	0.816496581	0.816496581	0.816496581	0.816496581	0.816496581	0.816496581	12	22	0	P_101008	P_101008	P_101008	chr1	27956111	27956171	+	SMPDL3B	"sphingomyelin phosphodiesterase, acid-like 3B [Source:HGNC Symbol;Acc:HGNC:21416]"	ENSG00000130768	protein_coding	exon	coding exon	1	1892	1:27956111:27956171	ENSG00000130768	SMPDL3B	9.889744884	0.005817328	1.724013484	0.787340742	0.762165278	-0.936672742	-0.961848206	-0.025175464	9.961963646	0.115539682	0.449408899	0.46362445	0.371078396	0.358890932	0.98041217	-5.580632469	-5.865463443	-6.738858055	-6.689901137	-5.826850238	-7.071032287	0.996027574	0.434082434

SNAP91	0.176150866	0.331819333	0.398659403	0.155668467	0.222508537	0.06684007	8	0.1557737	0.816496581	0.816496581	0.816496581	0.816496581	0.816496581	0.816496581	12	24	0	P_143119	P_143119	P_143119	chr6	83556152	83556212	-	SNAP91	"synaptosomal-associated protein, 91kDa [Source:HGNC Symbol;Acc:HGNC:14986]"	ENSG00000065609	protein_coding	3'UTR;exon	multiple Ref	1	1894	6:83556152:83556212	ENSG00000065609	SNAP91	4.610948072	0.02169316	1.464768535	2.759217312	3.315020603	1.294448777	1.850252068	0.555803291	9.961963646	0.17381725	0.020223968	0.007852705	0.224710264	0.094119919	0.59060869	-5.938928822	-3.242948704	-3.017068531	-6.303086804	-4.73996552	-6.905197813	4.359356046	0.033158846

SPEG	0.329538153	-0.138868183	0.277119659	-0.468406336	-0.052418494	0.415987842	8	0.153522287	0.816496581	0.816496581	0.816496581	0.816496581	0.816496581	0.816496581	12	26	0	A_14_P120831	A_14_P120831	A_14_P120831	chr2	219466795	219466855	+	SPEG	SPEG complex locus [Source:HGNC Symbol;Acc:HGNC:16901]	ENSG00000072195	protein_coding	3'UTR;exon	multiple Ref	1	1896	2:219466795:219466855	ENSG00000072195	SPEG	6.009362531	0.02113395	2.776265469	-1.169925055	2.334654521	-3.946190523	-0.441610948	3.504579575	9.961963646	0.019641853	0.269259405	0.041809827	0.002767535	0.66820213	0.00571565	-3.891926979	-5.507879229	-4.677364969	-2.042155057	-6.193992195	-2.716044901	7.096084032	0.007788994

EXO1	-0.447067221	0.042897071	-0.238506166	0.489964293	0.208561056	-0.281403237	8	0.068375613	0.816496581	0.816496581	0.816496581	0.816496581	0.816496581	0.816496581	12	27	0	A_23_P23303	A_23_P23303	A_23_P23303	chr1	241885414	241885474	+	EXO1	exonuclease 1 [Source:HGNC Symbol;Acc:HGNC:3511]	ENSG00000174371	protein_coding	exon	coding exon	1	1897	1:241885414:241885474	ENSG00000174371	EXO1	9.48090411	0.005961144	-7.091753758	0.680469182	-3.783384057	7.77222294	3.308369701	-4.463853239	9.961963646	3.39E-05	0.511711786	0.003606352	1.55E-05	0.00794118	0.001220298	2.644660127	-5.943719786	-2.223244494	3.36839631	-2.430278994	-1.126445631	26.05812497	4.95E-05

PRKCQ	-0.071763796	-0.057334362	0.02042826	0.014429434	0.092192055	0.077762621	8	0.155593102	0.816496581	0.816496581	0.816496581	0.816496581	0.816496581	0.816496581	12	28	0	A_32_P173939	A_32_P173939	A_32_P173939	chr10	6500538	6500598	+	PRKCQ	"protein kinase C, theta [Source:HGNC Symbol;Acc:HGNC:9410]"	ENSG00000065675	protein_coding	intron	intron antisense	0	1898	10:6500538:6500598	ENSG00000065675	PRKCQ	4.688951449	0.021648002	-0.597368222	-0.477256328	0.170046651	0.120111894	0.767414873	0.647302979	9.961963646	0.563592365	0.6434772	0.868377181	0.906782385	0.460636494	0.53206791	-6.807065158	-6.063420323	-7.031352397	-7.141729234	-5.99264919	-6.847192777	0.272648643	0.843784263

NCAM1	-0.111392061	-0.158787278	0.012669912	-0.047395217	0.124061973	0.17145719	8	0.134059573	0.816496581	0.816496581	0.816496581	0.816496581	0.816496581	0.816496581	12	29	0	P_110068	P_110068	P_110068	chr11	113275388	113275448	+	NCAM1	neural cell adhesion molecule 1 [Source:HGNC Symbol;Acc:HGNC:7656]	ENSG00000149294	protein_coding	3'UTR;exon	multiple Ref	1	1899	11:113275388:113275448	ENSG00000149294	NCAM1	3.687630393	0.016639155	-1.057631147	-1.507633217	0.120296667	-0.45000207	1.177927814	1.627929884	9.961963646	0.315195523	0.162687023	0.906639743	0.662342677	0.266198279	0.134713772	-6.420121236	-5.107709196	-7.039223461	-7.039988804	-5.60731765	-5.788795232	1.274364059	0.335671636

PPP2CB	-0.016532093	-0.01909626	-0.06723488	-0.002564167	-0.050702787	-0.04813862	8	0.076384225	0.816496581	0.816496581	0.816496581	0.816496581	0.816496581	0.816496581	12	30	0	P_146929	P_146929	P_146929	chr8	30807738	30807798	-	PPP2CB	"protein phosphatase 2, catalytic subunit, beta isozyme [Source:HGNC Symbol;Acc:HGNC:9300]"	ENSG00000104695	protein_coding	intron	intron sense	0	1900	8:30807738:30807798	ENSG00000104695	PPP2CB	7.807678388	0.006892146	-0.243891267	-0.28171938	-0.991888925	-0.037828113	-0.747997658	-0.710169545	9.961963646	0.812264304	0.78392416	0.344732223	0.970572047	0.471750454	0.493887683	-6.965998247	-6.140420737	-6.534922513	-7.148830573	-6.007418733	-6.802650668	0.364660774	0.78007975

ATP11C	0.381863711	-0.028735191	0.346006317	-0.410598902	-0.035857394	0.374741508	8	0.047568753	0.816496581	0.816496581	0.816496581	0.816496581	0.816496581	0.816496581	12	32	0	P_271745	P_271745	P_271745	chrX	139789338	139789398	-	ATP11C	"ATPase, class VI, type 11C [Source:HGNC Symbol;Acc:HGNC:13554]"	ENSG00000101974	protein_coding	exon	coding exon	1	1902	X:139789338:139789398	ENSG00000101974	ATP11C	8.128177563	0.004023825	7.372834584	-0.554804766	6.680517861	-7.92763935	-0.692316723	7.235322627	9.961963646	2.44E-05	0.591266127	5.60E-05	1.30E-05	0.504555427	2.86E-05	2.985010795	-6.022378712	2.097419844	3.54740607	-6.047891185	2.777510948	35.82874644	1.20E-05

DNAJB14	-0.004434688	0.015299764	-8.45E-05	0.019734452	0.004350195	-0.015384257	8	0.085784251	0.816496581	0.816496581	0.816496581	0.816496581	0.816496581	0.816496581	12	34	0	P_250167	P_250167	P_250167	chr4	99930449	99930509	-	DNAJB14	"DnaJ (Hsp40) homolog, subfamily B, member 14 [Source:HGNC Symbol;Acc:HGNC:25881]"	ENSG00000164031	protein_coding	exon	coding exon	1	1904	4:99930449:99930509	ENSG00000164031	DNAJB14	9.568740187	0.008116312	-0.06028779	0.207994081	-0.001148652	0.268281871	0.059139137	-0.209142733	9.961963646	0.953118736	0.83942425	0.999106185	0.793955821	0.9540108	0.838551527	-6.9963773	-6.15933261	-7.047119882	-7.11039462	-6.294825354	-7.047535514	0.027685126	0.993384874

ARFGAP3	-0.011479132	0.076130146	0.018729611	0.087609278	0.030208743	-0.057400536	8	0.067908572	0.816496581	0.816496581	0.816496581	0.816496581	0.816496581	0.816496581	12	35	0	P_242771	P_242771	P_242771	chr22	42822269	42822329	-	ARFGAP3	ADP-ribosylation factor GTPase activating protein 3 [Source:HGNC Symbol;Acc:HGNC:661]	ENSG00000242247	protein_coding	exon	coding exon	1	1905	22:42822269:42822329	ENSG00000242247	ARFGAP3	9.250348755	0.005910029	-0.182877327	1.212851055	0.298386764	1.395728382	0.481264092	-0.91446429	9.961963646	0.858562484	0.253163063	0.771538941	0.193123234	0.640724867	0.382073684	-6.980140939	-5.460964462	-6.998715461	-6.177149213	-6.174949459	-6.632828146	0.76823404	0.537620014

FBXO28	-0.299854416	0.015853415	-0.321102269	0.315707831	-0.021247853	-0.336955684	8	0.078982052	0.816496581	0.816496581	0.816496581	0.816496581	0.816496581	0.816496581	12	38	0	P_206439	P_206439	P_206439	chr1	224130521	224130581	+	FBXO28	F-box protein 28 [Source:HGNC Symbol;Acc:HGNC:29046]	ENSG00000143756	protein_coding	exon	coding exon	1	1908	1:224130521:224130581	ENSG00000143756	FBXO28	8.242291608	0.00721627	-4.323143816	0.228566235	-4.629484221	4.55171005	-0.306340405	-4.858050455	9.961963646	0.001519037	0.823829675	0.000946381	0.001065849	0.76565215	0.000670662	-1.291511131	-6.154621619	-0.842568151	-1.052843434	-6.247012061	-0.504952467	14.09775036	0.000644087

C5	0.034167375	-0.003038801	0.015960575	-0.037206176	-0.0182068	0.018999376	8	0.079372783	0.816496581	0.816496581	0.816496581	0.816496581	0.816496581	0.816496581	12	39	0	P_268774	P_268774	P_268774	chr9	121037880	121037940	-	C5	complement component 5 [Source:HGNC Symbol;Acc:HGNC:1331]	ENSG00000106804	protein_coding	exon	coding exon	1	1909	9:121037880:121037940	ENSG00000106804	C5	4.010163081	0.007265959	0.490920071	-0.043661775	0.229323051	-0.534581846	-0.261597019	0.272984826	9.961963646	0.634117106	0.966036776	0.823257461	0.604664149	0.798961198	0.790440273	-6.868425519	-6.18108255	-7.018477277	-6.995543721	-6.260415247	-7.030820184	0.120704899	0.945806738

USP28	-0.083481635	0.143646353	0.068321944	0.227127988	0.151803579	-0.075324409	8	0.01386373	0.816496581	0.816496581	0.816496581	0.816496581	0.816496581	0.816496581	12	40	0	P_213406	P_213406	P_213406	chr11	113827232	113827292	-	USP28	ubiquitin specific peptidase 28 [Source:HGNC Symbol;Acc:HGNC:12625]	ENSG00000048028	protein_coding	3'UTR;exon	multiple Ref	1	1910	11:113827232:113827292	ENSG00000048028	USP28	7.427083554	0.002361033	-2.104192656	3.62067182	1.722085757	5.724864476	3.826278413	-1.898586063	9.961963646	0.061745315	0.004713438	0.115898774	0.000194446	0.003362325	0.08693693	-5.002716593	-1.872843557	-5.63094201	0.724282858	-1.603138568	-5.392648084	11.92026322	0.001235583

ABR	0.059295143	0.025569535	0.106300679	-0.033725608	0.047005536	0.080731144	8	0.041464118	0.816496581	0.816496581	0.816496581	0.816496581	0.816496581	0.816496581	12	42	0	P_227310	P_227310	P_227310	chr17	1004285	1004345	+	ABR;TIMM22	active BCR-related [Source:HGNC Symbol;Acc:HGNC:81];translocase of inner mitochondrial membrane 22 homolog (yeast) [Source:HGNC Symbol;Acc:HGNC:17317]	ENSG00000159842	protein_coding	3'ext;3'UTR;exon	multiple non-Ref	0	1912	17:1004285:1004345	ENSG00000159842	ABR	6.561909002	0.003587354	1.212488516	0.522855097	2.173674693	-0.689633418	0.961186177	1.650819595	9.961963646	0.2532957	0.612504939	0.054929122	0.506170884	0.359207659	0.129903406	-6.25051125	-6.039993751	-4.938778261	-6.895580449	-5.827467972	-5.756567974	1.765507663	0.21728699

RER1	-0.128074507	0.050790352	-0.04935171	0.178864859	0.078722797	-0.100142061	8	0.067516699	0.816496581	0.816496581	0.816496581	0.816496581	0.816496581	0.816496581	12	43	0	P_100162	P_100162	P_100162	chr1	2403147	2403207	+	RER1	retention in endoplasmic reticulum sorting receptor 1 [Source:HGNC Symbol;Acc:HGNC:30309]	ENSG00000157916	protein_coding	"3'UTR;3'UTR,exon;exon"	multiple Ref	1	1913	1:2403147:2403207	ENSG00000157916	RER1	10.85532103	0.005867412	-2.047788213	0.812088881	-0.789086385	2.859877093	1.258701828	-1.601175265	9.961963646	0.067865319	0.435716214	0.448433891	0.017022008	0.236835064	0.140537337	-5.091603442	-5.84590807	-6.717360515	-3.891475094	-5.51666902	-5.826134111	2.967095291	0.083936378

EDRF1	-0.021462642	-0.028421195	-0.017198281	-0.006958554	0.004264361	0.011222914	8	0.042792623	0.816496581	0.816496581	0.816496581	0.816496581	0.816496581	0.816496581	12	44	0	P_210377	P_210377	P_210377	chr10	125749505	125749565	+	EDRF1	erythroid differentiation regulatory factor 1 [Source:HGNC Symbol;Acc:HGNC:24640]	ENSG00000107938	protein_coding	3'UTR;exon	multiple Ref	1	1914	10:125749505:125749565	ENSG00000107938	EDRF1	8.026756307	0.003677244	-0.433478498	-0.574019607	-0.347351697	-0.140541109	0.086126801	0.22666791	9.961963646	0.673903364	0.578683682	0.735553708	0.89103357	0.933072086	0.825265436	-6.896785634	-6.011313484	-6.981629407	-7.138822222	-6.292741332	-7.043383	0.119433882	0.946585034

CRYBG2	0.261335059	0.231999098	0.431695008	-0.029335961	0.170359949	0.19969591	8	0.106324115	0.816496581	0.816496581	0.816496581	0.816496581	0.816496581	0.816496581	12	45	0	A_23_P360329	A_23_P360329	A_23_P360329	chr1	26321956	26322016	-	AIM1L	absent in melanoma 1-like [Source:HGNC Symbol;Acc:HGNC:17295]	ENSG00000176092	protein_coding	"3'UTR,exon;exon"	multiple Ref	1	1915	1:26321956:26322016	ENSG00000176092	CRYBG2	7.708804117	0.011285069	3.012944979	2.674729215	4.977033358	-0.338215763	1.964088379	2.302304142	9.961963646	0.013105335	0.02337456	0.000562257	0.742220225	0.0780157	0.044174806	-3.48870082	-3.377776823	-0.302023349	-7.087415636	-4.572842666	-4.753599323	8.379252049	0.004453293

PRKD1	0.354886509	-0.097040301	0.324827614	-0.451926809	-0.030058895	0.421867915	8	0.108896752	0.816496581	0.816496581	0.816496581	0.816496581	0.816496581	0.816496581	12	46	0	P_220330	P_220330	P_220330	chr14	29905716	29905776	-	PRKD1	protein kinase D1 [Source:HGNC Symbol;Acc:HGNC:9407]	ENSG00000184304	protein_coding	intron	intron sense	0	1916	14:29905716:29905776	ENSG00000184304	PRKD1	8.96164788	0.011729708	4.013206585	-1.097372721	3.673287905	-5.110579306	-0.33991868	4.770660626	9.961963646	0.00248418	0.298294733	0.004321129	0.000462432	0.740975884	0.00076437	-1.79804015	-5.584268548	-2.408488156	-0.181931758	-6.235594007	-0.640900387	13.29931106	0.000809961

WDFY4	-0.076806164	-0.053031666	0.090701954	0.023774498	0.167508118	0.14373362	8	0.113971177	0.816496581	0.816496581	0.816496581	0.816496581	0.816496581	0.816496581	12	47	0	P_208203	P_208203	P_208203	chr10	48900246	48900306	+	WDFY4	WDFY family member 4 [Source:HGNC Symbol;Acc:HGNC:29323]	ENSG00000128815	protein_coding	exon	coding exon	1	1917	10:48900246:48900306	ENSG00000128815	WDFY4	3.868238634	0.012637904	-0.836766306	-0.577754559	0.988154266	0.259011747	1.824920572	1.565908825	9.961963646	0.422342516	0.576254894	0.346469734	0.800899538	0.098100332	0.148554513	-6.629213139	-6.009121804	-6.538596461	-7.113049106	-4.77653475	-5.874793759	1.309986611	0.324967671

TUBGCP2	-0.026645302	0.062996767	-0.099422301	0.08964207	-0.072776999	-0.162419068	8	0.118326362	0.816496581	0.816496581	0.816496581	0.816496581	0.816496581	0.816496581	12	48	0	P_308688	P_308688	P_308688	chr10	133311993	133312053	-	TUBGCP2	"tubulin, gamma complex associated protein 2 [Source:HGNC Symbol;Acc:HGNC:18599]"	ENSG00000130640	protein_coding	5'UTR	5'UTR	1	1918	10:133311993:133312053	ENSG00000130640	TUBGCP2	8.232749603	0.013450353	-0.281384023	0.66526864	-1.049935439	0.946652663	-0.768551416	-1.715204079	9.961963646	0.784174022	0.520982643	0.318550919	0.366211944	0.459991241	0.117188946	-6.955326254	-5.95401515	-6.476376927	-6.680467764	-5.991774303	-5.664574376	1.008811889	0.428930862

ACAD11	0.11643667	-0.080345529	-0.081995102	-0.196782199	-0.198431772	-0.001649573	8	0.036519557	0.816496581	0.816496581	0.816496581	0.816496581	0.816496581	0.816496581	12	49	0	P_246204	P_246204	P_246204	chr3	132576943	132577003	-	ACAD11	"acyl-CoA dehydrogenase family, member 11 [Source:HGNC Symbol;Acc:HGNC:30211]"	ENSG00000240303	protein_coding	3'UTR;exon	multiple Ref	1	1919	3:132576943:132577003	ENSG00000240303	ACAD11	6.038511847	0.0032777	2.490868454	-1.718789652	-1.754078108	-4.209658106	-4.244946562	-0.035288456	9.961963646	0.032025403	0.116515109	0.110068636	0.001816081	0.001717647	0.97254681	-4.372528258	-4.826217109	-5.584445694	-1.606367664	-0.95637998	-7.07069827	7.996248582	0.00522854

CDS1	-0.098120464	0.064397762	0.097827542	0.162518226	0.195948006	0.03342978	8	0.059942738	0.816496581	0.816496581	0.816496581	0.816496581	0.816496581	0.816496581	12	50	0	P_249756	P_249756	P_249756	chr4	84635263	84635323	+	CDS1	CDP-diacylglycerol synthase (phosphatidate cytidylyltransferase) 1 [Source:HGNC Symbol;Acc:HGNC:1800]	ENSG00000163624	protein_coding	exon	coding exon	1	1920	4:84635263:84635323	ENSG00000163624	CDS1	8.199212853	0.005092165	-1.68404619	1.105261845	1.67901875	2.789308035	3.36306494	0.573756905	9.961963646	0.12319461	0.295024321	0.12418926	0.019207909	0.007243012	0.578854723	-5.638047884	-5.576143634	-5.692802288	-4.011947655	-2.341884163	-6.894465191	4.382726894	0.032688978

ERG	0.01573092	-0.051316653	0.136686382	-0.067047573	0.120955461	0.188003034	8	0.098906877	0.816496581	0.816496581	0.816496581	0.816496581	0.816496581	0.816496581	12	51	0	P_132219	P_132219	P_132219	chr21	38445403	38445463	-	ERG	v-ets avian erythroblastosis virus E26 oncogene homolog [Source:HGNC Symbol;Acc:HGNC:3446]	ENSG00000157554	protein_coding	exon	coding exon	1	1921	21:38445403:38445463	ENSG00000157554	ERG	5.037354854	0.010062621	0.192063214	-0.626539396	1.668842349	-0.81860261	1.476779135	2.295381744	9.961963646	0.851551228	0.545050065	0.126224765	0.432158946	0.170634003	0.04469753	-6.978268153	-5.979284626	-5.707293149	-6.79502255	-5.251414577	-4.764917477	1.889039812	0.195600226

SGF29	0.011777605	-0.045168884	0.131613946	-0.056946489	0.119836341	0.17678283	8	0.129167457	0.816496581	0.816496581	0.816496581	0.816496581	0.816496581	0.816496581	12	52	0	P_118614	P_118614	P_118614	chr16	28591945	28592005	+	CCDC101;SULT1A2	"coiled-coil domain containing 101 [Source:HGNC Symbol;Acc:HGNC:25156];sulfotransferase family, cytosolic, 1A, phenol-preferring, member 2 [Source:HGNC Symbol;Acc:HGNC:11454]"	ENSG00000176476	protein_coding	3'ext;3'UTR	multiple non-Ref	0	1922	16:28591945:28592005	ENSG00000176476	SGF29	8.860341011	0.015605032	0.115470299	-0.442845929	1.290372822	-0.558316228	1.174902524	1.733218751	9.961963646	0.910366758	0.667338272	0.226066414	0.588955877	0.267352211	0.113838839	-6.991089295	-6.079748927	-6.206781492	-6.98177391	-5.610626518	-5.638492004	1.095111353	0.395833587

BBOF1	0.092904534	0.161363309	0.163739186	0.068458775	0.070834652	0.002375877	8	0.039878241	0.816496581	0.816496581	0.816496581	0.816496581	0.816496581	0.816496581	12	53	0	P_221529	P_221529	P_221529	chr14	74056920	74056980	+	CCDC176	coiled-coil domain containing 176 [Source:HGNC Symbol;Acc:HGNC:19855]	ENSG00000119636	protein_coding	exon	coding exon	1	1923	14:74056920:74056980	ENSG00000119636	BBOF1	6.194972347	0.003483761	1.927784009	3.34831459	3.397614411	1.420530581	1.469830402	0.049299821	9.961963646	0.082848493	0.007424824	0.006834914	0.185986895	0.172469472	0.961654788	-5.277376698	-2.301823925	-2.876158119	-6.145330391	-5.260292298	-7.070050913	5.108680746	0.021375912

RBKS	0.625585442	0.195040037	0.691501986	-0.430545406	0.065916543	0.496461949	8	0.05468001	0.816496581	0.816496581	0.816496581	0.816496581	0.816496581	0.816496581	12	57	0	P_127510	P_127510	P_127510	chr2	27781695	27781755	+	RBKS;MRPL33	ribokinase [Source:HGNC Symbol;Acc:HGNC:30325];mitochondrial ribosomal protein L33 [Source:HGNC Symbol;Acc:HGNC:14487]	ENSG00000171174	protein_coding	3'ext;3'UTR;exon;intron	multiple non-Ref	0	1927	2:27781695:27781755	ENSG00000171174	RBKS	8.395761088	0.00460774	11.28725412	3.519050011	12.47656692	-7.768204111	1.189312802	8.957516913	9.961963646	5.36E-07	0.005579511	2.10E-07	1.56E-05	0.261891204	4.43E-06	6.837859256	-2.032126794	7.758685494	3.363729952	-5.594808702	4.701203313	72.90785169	4.42E-07

YME1L1	-0.005979116	-0.074950844	-0.019460081	-0.068971729	-0.013480965	0.055490764	8	0.0438379	0.816496581	0.816496581	0.816496581	0.816496581	0.816496581	0.816496581	12	59	0	P_105699	P_105699	P_105699	chr10	27114521	27114581	-	YME1L1	YME1-like 1 ATPase [Source:HGNC Symbol;Acc:HGNC:12843]	ENSG00000136758	protein_coding	exon	coding exon	1	1929	10:27114521:27114581	ENSG00000136758	YME1L1	9.829806306	0.003749963	-0.119582903	-1.499024234	-0.389203524	-1.379441331	-0.26962062	1.10982071	9.961963646	0.907190778	0.164871382	0.705315486	0.197934889	0.792954583	0.293147151	-6.990562522	-5.118726838	-6.965023421	-6.197839393	-6.258164674	-6.437225455	0.936684797	0.458873858

PKLR	0.183802789	0.098301358	-0.022074511	-0.085501431	-0.2058773	-0.120375869	8	0.32623774	0.816496581	0.816496581	0.816496581	0.816496581	0.816496581	0.816496581	12	60	0	A_23_P201022	A_23_P201022	A_23_P201022	chr1	155290302	155290362	-	PKLR	"pyruvate kinase, liver and RBC [Source:HGNC Symbol;Acc:HGNC:9020]"	ENSG00000143627	protein_coding	3'UTR	3'UTR	1	1930	1:155290302:155290362	ENSG00000143627	PKLR	4.468228147	0.087676631	0.760248899	0.40659611	-0.091305049	-0.353652789	-0.851553948	-0.497901159	9.961963646	0.464718317	0.692902237	0.929060078	0.730969132	0.41446302	0.629360702	-6.691844317	-6.095680356	-7.042569811	-7.081644669	-5.924827132	-6.937616242	0.308292564	0.818973707

NDC1	-0.482800981	0.010839281	-0.354427265	0.493640263	0.128373716	-0.365266547	8	0.056042339	0.816496581	0.816496581	0.816496581	0.816496581	0.816496581	0.816496581	12	61	0	A_24_P222192	A_24_P222192	A_24_P222192	chr1	53766878	53766938	-	NDC1	NDC1 transmembrane nucleoporin [Source:HGNC Symbol;Acc:HGNC:25525]	ENSG00000058804	protein_coding	3'UTR	3'UTR	1	1931	1:53766878:53766938	ENSG00000058804	NDC1	9.130493869	0.004728872	-8.598743074	0.193048895	-6.312391874	8.791791969	2.2863512	-6.505440769	9.961963646	6.38E-06	0.850799687	8.92E-05	5.24E-06	0.045388491	6.97E-05	4.357897305	-6.162479047	1.61377251	4.493494005	-4.079548366	1.853398002	39.77761816	7.45E-06

RCVRN	0.200604918	0.492365656	0.278763395	0.291760738	0.078158477	-0.213602261	8	0.273414016	0.816496581	0.816496581	0.816496581	0.816496581	0.816496581	0.816496581	12	62	0	P_227767	P_227767	P_227767	chr17	9900989	9901049	-	RCVRN	recoverin [Source:HGNC Symbol;Acc:HGNC:9937]	ENSG00000109047	protein_coding	exon	coding exon	1	1932	17:9900989:9901049	ENSG00000109047	RCVRN	3.459373723	0.062239205	0.984816204	2.417137535	1.368514348	1.432321331	0.383698145	-1.048623187	9.961963646	0.348028181	0.036325609	0.201219515	0.182674021	0.709263929	0.319125762	-6.49351545	-3.785363427	-6.110550447	-6.130074055	-6.218970688	-6.501775639	1.997271265	0.17864154

GALNT18	-0.124953245	0.121921412	-0.006916535	0.246874657	0.11803671	-0.128837946	8	0.114998615	0.816496581	0.816496581	0.816496581	0.816496581	0.816496581	0.816496581	12	65	0	P_107974	P_107974	P_107974	chr11	11584392	11584452	-	GALNT18	polypeptide N-acetylgalactosaminyltransferase 18 [Source:HGNC Symbol;Acc:HGNC:30488]	ENSG00000110328	protein_coding	intron	intron sense	0	1935	11:11584392:11584452	ENSG00000110328	GALNT18	6.773230196	0.012826824	-1.351243433	1.31845721	-0.074795354	2.669700643	1.276448079	-1.393252563	9.961963646	0.206504691	0.216858003	0.941858179	0.023576867	0.230750417	0.193848208	-6.084158357	-5.340397692	-7.044066337	-4.215299802	-5.496173321	-6.103161946	2.377926083	0.131222341

RAD52	-0.053454107	-0.036029315	-0.056244281	0.017424792	-0.002790174	-0.020214965	8	0.06759342	0.816496581	0.816496581	0.816496581	0.816496581	0.816496581	0.816496581	12	66	0	P_214039	P_214039	P_214039	chr12	927187	927247	-	RAD52	RAD52 homolog (S. cerevisiae) [Source:HGNC Symbol;Acc:HGNC:9824]	ENSG00000002016	protein_coding	"3'UTR;3'UTR,exon;exon"	multiple Ref	1	1936	12:927187:927247	ENSG00000002016	RAD52	5.968035449	0.005875736	-0.854074137	-0.575665894	-0.898654717	0.278408243	-0.04458058	-0.322988823	9.961963646	0.413130233	0.577612439	0.390040474	0.786392289	0.965322579	0.753380994	-6.614321466	-6.010349072	-6.623183846	-7.107389855	-6.29562828	-7.014685607	0.342039085	0.795611913

COL6A1	0.247767665	0.015156469	0.19203457	-0.232611196	-0.055733095	0.1768781	8	0.144099662	0.816496581	0.816496581	0.816496581	0.816496581	0.816496581	0.816496581	12	67	0	P_132479	P_132479	P_132479	chr21	46002541	46002601	+	COL6A1	"collagen, type VI, alpha 1 [Source:HGNC Symbol;Acc:HGNC:2211]"	ENSG00000142156	protein_coding	exon	coding exon	1	1937	21:46002541:46002601	ENSG00000142156	COL6A1	7.296361685	0.018881881	2.208348621	0.13508933	1.711600573	-2.073259291	-0.496748048	1.576511243	9.961963646	0.051803897	0.89523177	0.117869709	0.06503342	0.63014513	0.146102776	-4.836254579	-6.172476171	-5.646081087	-5.197800962	-6.167089969	-5.860232692	2.475863281	0.121523

SORCS2	-0.121916706	-0.075316169	-0.113042179	0.046600538	0.008874527	-0.03772601	8	0.142809462	0.816496581	0.816496581	0.816496581	0.816496581	0.816496581	0.816496581	12	68	0	A_24_P561377	A_24_P561377	A_24_P561377	chr4	7560806	7560866	-	SORCS2	sortilin-related VPS10 domain containing receptor 2 [Source:HGNC Symbol;Acc:HGNC:16698]	ENSG00000184985	protein_coding	intron	intron antisense	0	1938	4:7560806:7560866	ENSG00000184985	SORCS2	3.734783616	0.018584614	-1.095297468	-0.676639088	-1.015568876	0.41865838	0.079728592	-0.338929788	9.961963646	0.299159657	0.514038358	0.333864927	0.684348678	0.938032002	0.741698384	-6.380519246	-5.946333837	-6.511364553	-7.054599621	-6.293305355	-7.00898439	0.497567005	0.692196421

CIZ1	-0.000485922	0.003882564	0.015162644	0.004368486	0.015648566	0.01128008	8	0.072376717	0.816496581	0.816496581	0.816496581	0.816496581	0.816496581	0.816496581	12	69	0	P_149784	P_149784	P_149784	chr9	128167687	128167747	-	CIZ1;C9orf16	CDKN1A interacting zinc finger protein 1 [Source:HGNC Symbol;Acc:HGNC:16744];chromosome 9 open reading frame 16 [Source:HGNC Symbol;Acc:HGNC:17823]	ENSG00000148337	protein_coding	3'ext;intron;promoter	multiple non-Ref	0	1939	9:128167687:128167747	ENSG00000148337	CIZ1	7.320212751	0.006413396	-0.007431359	0.059377274	0.231887077	0.066808633	0.239318436	0.172509803	9.961963646	0.9942174	0.953825853	0.821319663	0.948055858	0.815710476	0.866491141	-6.998330182	-6.180229138	-7.017834927	-7.147172733	-6.266312483	-7.055145141	0.02480814	0.994370029

CAMK1D	0.88011005	0.115733504	0.915707303	-0.764376546	0.035597253	0.799973799	8	0.093382991	0.816496581	0.816496581	0.816496581	0.816496581	0.816496581	0.816496581	12	70	0	P_207419	P_207419	P_207419	chr10	12666750	12666810	+	CAMK1D	calcium/calmodulin-dependent protein kinase ID [Source:HGNC Symbol;Acc:HGNC:19341]	ENSG00000183049	protein_coding	exon	coding exon	1	1940	10:12666750:12666810	ENSG00000183049	CAMK1D	5.559531596	0.009209627	11.23211625	1.477010943	11.68641453	-9.755105306	0.454298282	10.20940359	9.961963646	5.61E-07	0.170573065	3.88E-07	2.05E-06	0.659351863	1.36E-06	6.79314933	-5.146722215	7.157657225	5.458029988	-6.188069767	5.906497058	77.41911156	3.32E-07

KIAA0232	0.063922935	0.003675822	0.037021659	-0.060247113	-0.026901276	0.033345837	8	0.083066089	0.816496581	0.816496581	0.816496581	0.816496581	0.816496581	0.816496581	12	72	0	A_24_P179679	A_24_P179679	A_24_P179679	chr4	6849971	6850031	-	KIAA0232	KIAA0232 [Source:HGNC Symbol;Acc:HGNC:28992]	ENSG00000170871	protein_coding	intron	intron antisense	0	1942	4:6849971:6850031	ENSG00000170871	KIAA0232	6.336977441	0.00774774	0.889436227	0.051146109	0.515126612	-0.838290118	-0.374309615	0.463980503	9.961963646	0.394739436	0.960220105	0.61770068	0.421525897	0.716018187	0.652634685	-6.583082244	-6.180708572	-6.904117769	-6.778340581	-6.2227003	-6.955033034	0.352864012	0.788164202

GML	0.272365644	-0.336619345	0.232189247	-0.608984989	-0.040176397	0.568808592	8	0.594967812	0.816496581	0.816496581	0.816496581	0.816496581	0.816496581	0.816496581	12	73	0	A_23_P59976	A_23_P59976	A_23_P59976	chr8	142846767	142846827	+	GML	glycosylphosphatidylinositol anchored molecule like [Source:HGNC Symbol;Acc:HGNC:4375]	ENSG00000104499	protein_coding	3'UTR	3'UTR	1	1943	8:142846767:142846827	ENSG00000104499	GML	4.542063762	0.286477304	0.62323645	-0.77026398	0.531303433	-1.39350043	-0.091933017	1.301567413	9.961963646	0.547131863	0.459020066	0.60685093	0.193775523	0.928573681	0.222357867	-6.790461328	-5.878647188	-6.895122396	-6.179989126	-6.292191891	-6.217169717	0.818064412	0.512983022

SETX	0.144164495	-0.068015364	0.004521146	-0.212179859	-0.139643349	0.07253651	8	0.063736094	0.816496581	0.816496581	0.816496581	0.816496581	0.816496581	0.816496581	12	74	0	P_269237	P_269237	P_269237	chr9	132277059	132277119	-	SETX	senataxin [Source:HGNC Symbol;Acc:HGNC:445]	ENSG00000107290	protein_coding	exon	coding exon	1	1944	9:132277059:132277119	ENSG00000107290	SETX	8.380704489	0.005468924	2.387552865	-1.126423508	0.074876102	-3.513976372	-2.312676763	1.201299609	9.961963646	0.038206404	0.286389041	0.941795539	0.005626857	0.043402666	0.25741678	-4.543872015	-5.55412863	-7.044059741	-2.771410244	-4.03821121	-6.335490783	4.35241159	0.033300012

PAN3	0.116701957	-0.079183857	-0.021778632	-0.195885814	-0.138480588	0.057405226	8	0.056616381	0.816496581	0.816496581	0.816496581	0.816496581	0.816496581	0.816496581	12	77	0	P_113496	P_113496	P_113496	chr13	28292727	28292787	+	PAN3	PAN3 poly(A) specific ribonuclease subunit [Source:HGNC Symbol;Acc:HGNC:29991]	ENSG00000152520	protein_coding	3'UTR	3'UTR	1	1947	13:28292727:28292787	ENSG00000152520	PAN3	5.918090835	0.004780807	2.067155628	-1.402593076	-0.385767493	-3.469748705	-2.452923121	1.016825583	9.961963646	0.065701616	0.191125129	0.707778731	0.006057639	0.034171831	0.3332954	-5.061188984	-5.239417147	-6.966455864	-2.846803384	-3.816026999	-6.534159961	4.246523078	0.035543892

PKD1L1	0.036961003	-0.022168643	0.011899318	-0.059129645	-0.025061685	0.03406796	8	0.096345539	0.816496581	0.816496581	0.816496581	0.816496581	0.816496581	0.816496581	12	80	0	P_261168	P_261168	P_261168	chr7	47854881	47854941	-	PKD1L1	polycystic kidney disease 1 like 1 [Source:HGNC Symbol;Acc:HGNC:18053]	ENSG00000158683	protein_coding	exon	coding exon	1	1950	7:47854881:47854941	ENSG00000158683	PKD1L1	3.834990125	0.009661008	0.46055145	-0.276231699	0.148271087	-0.736783149	-0.312280363	0.424502786	9.961963646	0.655009956	0.788016045	0.885087105	0.478246661	0.761265928	0.680220999	-6.883839758	-6.142021825	-7.035128069	-6.860600157	-6.245076813	-6.973819223	0.188494492	0.901813709

PTCD1	-0.077702935	-0.076847137	-0.131401459	0.000855797	-0.053698524	-0.054554321	8	0.060563733	0.816496581	0.816496581	0.816496581	0.816496581	0.816496581	0.816496581	12	82	0	P_145321	P_145321	P_145321	chr7	99417456	99417516	-	PTCD1	pentatricopeptide repeat domain 1 [Source:HGNC Symbol;Acc:HGNC:22198]	ENSG00000106246	protein_coding	3'UTR	3'UTR	1	1952	7:99417456:99417516	ENSG00000106246	PTCD1	8.81225857	0.005152261	-1.325818711	-1.311216532	-2.242058339	0.014602179	-0.916239628	-0.930841807	9.961963646	0.214496472	0.219201818	0.048930421	0.988637863	0.381186302	0.373943093	-6.115591833	-5.348889361	-4.828458819	-7.14949646	-5.868580392	-6.617627065	1.701680732	0.229574593

CLEC12B	-0.043717756	0.069131836	0.065404896	0.112849593	0.109122652	-0.00372694	8	0.179091329	0.816496581	0.816496581	0.816496581	0.816496581	0.816496581	0.816496581	12	84	0	P_110941	P_110941	P_110941	chr12	10017651	10017711	+	CLEC12B	"C-type lectin domain family 12, member B [Source:HGNC Symbol;Acc:HGNC:31966]"	ENSG00000256660	protein_coding	3'UTR	3'UTR	1	1954	12:10017651:10017711	ENSG00000256660	CLEC12B	4.100783813	0.027963617	-0.320189304	0.506322293	0.479026142	0.826511597	0.799215446	-0.027296151	9.961963646	0.75543953	0.623646747	0.642261079	0.42786592	0.442803879	0.978762597	-6.942703551	-6.048721653	-6.923238855	-6.788362439	-5.967739761	-7.070971501	0.318493555	0.811892733

SMCHD1	-0.46266597	-0.094913533	-0.468970286	0.367752436	-0.006304316	-0.374056752	8	0.06990907	0.816496581	0.816496581	0.816496581	0.816496581	0.816496581	0.816496581	12	85	0	P_230428	P_230428	P_230428	chr18	2722603	2722663	+	SMCHD1	structural maintenance of chromosomes flexible hinge domain containing 1 [Source:HGNC Symbol;Acc:HGNC:29090]	ENSG00000101596	protein_coding	exon	coding exon	1	1955	18:2722603:2722663	ENSG00000101596	SMCHD1	9.938450322	0.006131435	-7.236559395	-1.484542773	-7.335165219	5.752016622	-0.098605823	-5.850622445	9.961963646	2.86E-05	0.168603296	2.55E-05	0.000187369	0.923407147	0.000163909	2.821259147	-5.13717245	2.912802415	0.763088325	-6.291516272	0.962513742	29.28358487	2.96E-05

THSD4	0.003648879	0.064888813	-0.035815098	0.061239934	-0.039463977	-0.100703911	8	0.12378586	0.816496581	0.816496581	0.816496581	0.816496581	0.816496581	0.816496581	12	86	0	A_23_P148249	A_23_P148249	A_23_P148249	chr15	71780413	71780473	+	THSD4	"thrombospondin, type I, domain containing 4 [Source:HGNC Symbol;Acc:HGNC:25835]"	ENSG00000187720	protein_coding	3'UTR	3'UTR	1	1956	15:71780413:71780473	ENSG00000187720	THSD4	4.265880372	0.01451184	0.037097428	0.659711753	-0.364125034	0.622614325	-0.401222462	-1.023836787	9.961963646	0.971140185	0.524396645	0.7233743	0.547524488	0.696727504	0.330131297	-6.997609378	-5.957725961	-6.975194618	-6.941665943	-6.211769638	-6.527088503	0.361008781	0.78257873

ZZEF1	-0.114394531	-0.04957767	-0.187233607	0.064816861	-0.072839076	-0.137655937	8	0.062979136	0.816496581	0.816496581	0.816496581	0.816496581	0.816496581	0.816496581	12	90	0	P_227454	P_227454	P_227454	chr17	4017370	4017430	-	ZZEF1	"zinc finger, ZZ-type with EF-hand domain 1 [Source:HGNC Symbol;Acc:HGNC:29027]"	ENSG00000074755	protein_coding	exon	coding exon	1	1960	17:4017370:4017430	ENSG00000074755	ZZEF1	6.339368596	0.005391897	-1.908007655	-0.826915175	-3.12290416	1.08109248	-1.214896505	-2.295988985	9.961963646	0.085597546	0.427647628	0.010868592	0.305131983	0.25241577	0.044651436	-5.307517805	-5.833940495	-3.345565766	-6.545464024	-5.566375135	-4.763925044	3.6655187	0.051615243

KIF6	0.175403747	0.134787012	0.301373118	-0.040616735	0.125969371	0.166586106	8	0.141798376	0.816496581	0.816496581	0.816496581	0.816496581	0.816496581	0.816496581	12	91	0	P_256987	P_256987	P_256987	chr6	39596053	39596113	-	KIF6	kinesin family member 6 [Source:HGNC Symbol;Acc:HGNC:21202]	ENSG00000164627	protein_coding	5'UTR;exon	multiple Ref	1	1961	6:39596053:39596113	ENSG00000164627	KIF6	4.482406335	0.018353524	1.585713639	1.218523588	2.724522544	-0.367190051	1.138808905	1.505998956	9.961963646	0.144003839	0.251095005	0.021462692	0.721157347	0.281427263	0.163099736	-5.776062684	-5.454672042	-4.025337754	-7.076377345	-5.649608762	-5.955943197	2.520342947	0.117397796

ADAMTS9-AS2	0.09575925	-0.121760245	0.045701379	-0.217519495	-0.050057871	0.167461624	8	0.161856015	0.816496581	0.816496581	0.816496581	0.816496581	0.816496581	0.816496581	12	93	0	P_312333	P_312333	P_312333	chr3	64973089	64973149	-	ADAMTS9-AS2	ADAMTS9 antisense RNA 2 [Source:HGNC Symbol;Acc:HGNC:42435]	ENSG00000241684	antisense	intron	intron antisense	0	1963	3:64973089:64973149	ENSG00000241684	ADAMTS9-AS2	3.888367789	0.0232446	0.769246136	-0.978115409	0.367124946	-1.747361544	-0.40212119	1.345240355	9.961963646	0.459597113	0.351172128	0.72120441	0.111270013	0.696087113	0.208368808	-6.684754746	-5.701458491	-6.974012605	-5.694432745	-6.211391946	-6.163530948	1.117979254	0.387535754

PIGG	-0.105885898	0.039742055	-0.061128341	0.145627952	0.044757557	-0.100870396	8	0.075080421	0.816496581	0.816496581	0.816496581	0.816496581	0.816496581	0.816496581	12	94	0	P_137045	P_137045	P_137045	chr4	539440	539500	+	PIGG	"phosphatidylinositol glycan anchor biosynthesis, class G [Source:HGNC Symbol;Acc:HGNC:25985]"	ENSG00000174227	protein_coding	3'UTR;exon	multiple Ref	1	1964	4:539440:539500	ENSG00000174227	PIGG	6.638484226	0.006733558	-1.580379703	0.593162428	-0.912359359	2.173542131	0.668020343	-1.505521787	9.961963646	0.145217161	0.56629452	0.383127707	0.054941411	0.51929698	0.163220411	-5.783410782	-5.999940549	-6.610668224	-5.037921763	-6.064664411	-5.956581664	1.853162283	0.201629336

ANO4	0.130027877	0.146533458	0.1372659	0.016505581	0.007238024	-0.009267558	8	0.186045635	0.816496581	0.816496581	0.816496581	0.816496581	0.816496581	0.816496581	12	95	0	P_216944	P_216944	P_216944	chr12	101043575	101043635	+	ANO4	anoctamin 4 [Source:HGNC Symbol;Acc:HGNC:23837]	ENSG00000151572	protein_coding	exon	coding exon	1	1965	12:101043575:101043635	ENSG00000151572	ANO4	3.759291213	0.030002793	0.919393137	1.03609979	0.97057131	0.116706652	0.051178173	-0.065528479	9.961963646	0.379613665	0.324651246	0.354736584	0.909411801	0.96019519	0.949049604	-6.555775385	-5.645818866	-6.555740415	-7.142169617	-6.295292326	-7.069033217	0.480221469	0.703274229

AIFM1	-0.083176254	-0.094823404	0.033016287	-0.01164715	0.116192541	0.127839691	8	0.107184439	0.816496581	0.816496581	0.816496581	0.816496581	0.816496581	0.816496581	12	96	0	P_271488	P_271488	P_271488	chrX	130137112	130137172	+	AIFM1	"apoptosis-inducing factor, mitochondrion-associated, 1 [Source:HGNC Symbol;Acc:HGNC:8768]"	ENSG00000156709	protein_coding	"3'UTR;5'UTR,exon;exon"	multiple non-Ref	0	1966	X:130137112:130137172	ENSG00000156709	AIFM1	9.286063695	0.011432579	-0.95273667	-1.086148148	0.378182787	-0.133411479	1.330919457	1.464330936	9.961963646	0.363267902	0.302995976	0.713228608	0.896524506	0.212872772	0.173934192	-6.52448855	-5.595750812	-6.969574191	-7.13988833	-5.432008498	-6.011213012	1.027309582	0.421592828

WDR1	-0.281517888	0.061101574	-0.133438517	0.342619462	0.14807937	-0.194540091	8	0.082729613	0.816496581	0.816496581	0.816496581	0.816496581	0.816496581	0.816496581	12	97	0	P_248207	P_248207	P_248207	chr4	10084440	10084500	-	WDR1	WD repeat domain 1 [Source:HGNC Symbol;Acc:HGNC:12754]	ENSG00000071127	protein_coding	exon	coding exon	1	1967	4:10084440:10084500	ENSG00000071127	WDR1	10.14143218	0.007702941	-3.928469116	0.852647935	-1.862080946	4.781117051	2.06638817	-2.714728882	9.961963646	0.002847994	0.41388411	0.092312838	0.000752458	0.065786094	0.021825937	-1.938405085	-5.812725713	-5.424272097	-0.690132038	-4.419152166	-4.064783187	9.021002796	0.003440746

UNC5D	-0.121150939	-0.116750103	-0.077711807	0.004400835	0.043439132	0.039038297	8	0.086333106	0.816496581	0.816496581	0.816496581	0.816496581	0.816496581	0.816496581	12	98	0	A_24_P668349	A_24_P668349	A_24_P668349	chr8	35672881	35672941	-	UNC5D	unc-5 homolog D (C. elegans) [Source:HGNC Symbol;Acc:HGNC:18634]	ENSG00000156687	protein_coding	intron	intron antisense	0	1968	8:35672881:35672941	ENSG00000156687	UNC5D	3.757434402	0.008192175	-1.639354245	-1.579804332	-1.051557519	0.059549913	0.587796726	0.528246813	9.961963646	0.132293343	0.145348581	0.317841437	0.953691776	0.569752308	0.608893445	-5.701363743	-5.01386538	-6.47470246	-7.147674117	-6.116134288	-6.921046237	1.152855527	0.375245024

THADA	-0.071367437	0.016921324	-0.145119937	0.088288761	-0.0737525	-0.162041261	8	0.067987363	0.816496581	0.816496581	0.816496581	0.816496581	0.816496581	0.816496581	12	99	0	P_234670	P_234670	P_234670	chr2	43592316	43592376	-	THADA	thyroid adenoma associated [Source:HGNC Symbol;Acc:HGNC:19217]	ENSG00000115970	protein_coding	exon	coding exon	1	1969	2:43592316:43592376	ENSG00000115970	THADA	6.515226887	0.005918628	-1.136148775	0.269382548	-2.310267064	1.405531323	-1.174118289	-2.579649612	9.961963646	0.282487233	0.793132606	0.043580864	0.190275279	0.267651989	0.027510305	-6.336347354	-6.143976641	-4.717336911	-6.164617602	-5.611483226	-4.292977795	2.784899847	0.096008779

SHCBP1	-0.636675881	0.030779623	-0.226799456	0.667455504	0.409876425	-0.257579079	8	0.059619899	0.816496581	0.816496581	0.816496581	0.816496581	0.816496581	0.816496581	12	101	0	P_226186	P_226186	P_226186	chr16	46618214	46618274	-	SHCBP1	SHC SH2-domain binding protein 1 [Source:HGNC Symbol;Acc:HGNC:29547]	ENSG00000171241	protein_coding	exon	coding exon	1	1971	16:46618214:46618274	ENSG00000171241	SHCBP1	9.27569012	0.005061167	-10.96070986	0.529887384	-3.904471817	11.49059725	7.056238048	-4.434359201	9.961963646	7.04E-07	0.607796732	0.002960875	4.54E-07	3.54E-05	0.001277323	6.569701419	-6.036201027	-2.020753464	6.991243891	2.679062463	-1.173780232	56.19178393	1.51E-06

MS4A2	0.351183532	0.218300934	0.302498761	-0.132882598	-0.048684771	0.084197827	8	0.488528111	0.816496581	0.816496581	0.816496581	0.816496581	0.816496581	0.816496581	12	104	0	A_23_P1904	A_23_P1904	A_23_P1904	chr11	60098282	60098342	+	MS4A2	"membrane-spanning 4-domains, subfamily A, member 2 [Source:HGNC Symbol;Acc:HGNC:7316]"	ENSG00000149534	protein_coding	3'UTR	3'UTR	1	1974	11:60098282:60098342	ENSG00000149534	MS4A2	4.127634725	0.193863449	0.976858826	0.607230051	0.841436336	-0.369628775	-0.135422491	0.234206284	9.961963646	0.351764015	0.557284592	0.419843211	0.719395331	0.894975117	0.819567974	-6.501277286	-5.991362073	-6.673685533	-7.07540797	-6.28694012	-7.041495147	0.373468979	0.774067225

KLKB1	-0.150337929	0.055310433	-0.091167722	0.205648362	0.059170207	-0.146478155	8	0.072468733	0.816496581	0.816496581	0.816496581	0.816496581	0.816496581	0.816496581	12	105	0	P_251930	P_251930	P_251930	chr4	186251268	186251328	+	KLKB1	"kallikrein B, plasma (Fletcher factor) 1 [Source:HGNC Symbol;Acc:HGNC:6371]"	ENSG00000164344	protein_coding	exon	coding exon	1	1975	4:186251268:186251328	ENSG00000164344	KLKB1	4.375957673	0.006424099	-2.297248984	0.845174849	-1.393094596	3.142423833	0.904154388	-2.238269445	9.961963646	0.044555941	0.417849691	0.193894542	0.010514264	0.387255904	0.049245542	-4.692060155	-5.818944402	-6.079479511	-3.407238903	-5.879353598	-4.857894057	3.939093131	0.043157311

SIRT5	-0.026488798	0.003563088	-0.080918295	0.030051886	-0.054429497	-0.084481383	8	0.063020606	0.816496581	0.816496581	0.816496581	0.816496581	0.816496581	0.816496581	12	106	0	P_141678	P_141678	P_141678	chr6	13611805	13611865	+	SIRT5	sirtuin 5 [Source:HGNC Symbol;Acc:HGNC:14933]	ENSG00000124523	protein_coding	3'UTR;exon	multiple Ref	1	1976	6:13611805:13611865	ENSG00000124523	SIRT5	7.984709363	0.005396093	-0.441639774	0.059406299	-1.349126431	0.501046073	-0.907486657	-1.40853273	9.961963646	0.668181961	0.953803312	0.207160475	0.627223766	0.385575557	0.189410481	-6.892962353	-6.180227322	-6.134791957	-7.014035406	-5.876395113	-6.08365015	0.846210096	0.49957508

PPP1R9A	0.122879308	0.008543942	0.228900976	-0.114335366	0.106021668	0.220357034	8	0.062163846	0.816496581	0.816496581	0.816496581	0.816496581	0.816496581	0.816496581	12	107	0	P_145246	P_145246	P_145246	chr7	95284270	95284330	+	PPP1R9A	"protein phosphatase 1, regulatory subunit 9A [Source:HGNC Symbol;Acc:HGNC:14946]"	ENSG00000158528	protein_coding	exon	coding exon	1	1977	7:95284270:95284330	ENSG00000158528	PPP1R9A	7.7624014	0.005309963	2.065278619	0.14360123	3.847224566	-1.921677389	1.781945947	3.703623336	9.961963646	0.06590841	0.888678631	0.003249494	0.083688429	0.105209416	0.004110543	-5.064141579	-6.171228158	-2.11631402	-5.433626423	-4.838018246	-2.378979883	6.612022518	0.009784293

NUDT13	0.027241414	0.215374137	-0.016341625	0.188132723	-0.043583039	-0.231715762	8	0.065995822	0.816496581	0.816496581	0.816496581	0.816496581	0.816496581	0.816496581	12	108	0	P_106259	P_106259	P_106259	chr10	73125183	73125243	+	NUDT13	nudix (nucleoside diphosphate linked moiety X)-type motif 13 [Source:HGNC Symbol;Acc:HGNC:18827]	ENSG00000166321	protein_coding	5'UTR;exon	multiple Ref	1	1978	10:73125183:73125243	ENSG00000166321	NUDT13	5.846213095	0.005704347	0.441745692	3.492498472	-0.26499514	3.05075278	-0.706740833	-3.757493612	9.961963646	0.668107853	0.005831945	0.796415596	0.012287663	0.495925594	0.003762533	-6.892912283	-2.073906418	-7.008906947	-3.564518402	-6.037676926	-2.288271695	6.064706772	0.012827973

LVRN	0.026757765	0.107246354	0.078458469	0.080488589	0.051700705	-0.028787884	8	0.137595157	0.816496581	0.816496581	0.816496581	0.816496581	0.816496581	0.816496581	12	109	0	P_254413	P_254413	P_254413	chr5	116002851	116002911	+	AQPEP	Aminopeptidase Q [Source:UniProtKB/Swiss-Prot;Acc:Q6Q4G3]	ENSG00000172901	protein_coding	exon	coding exon	1	1979	5:116002851:116002911	ENSG00000172901	LVRN	3.726006146	0.017410455	0.248365	0.995458365	0.728249838	0.747093365	0.479884837	-0.267208527	9.961963646	0.80889688	0.343077592	0.483227438	0.47227219	0.641671429	0.794758847	-6.964803835	-5.685088394	-6.764993815	-6.852674369	-6.175638058	-7.032512343	0.407134784	0.751300293

ALK	0.103954661	0.167181234	0.163774642	0.063226573	0.059819982	-0.003406591	8	0.191772201	0.816496581	0.816496581	0.816496581	0.816496581	0.816496581	0.816496581	12	110	0	A_23_P324304	A_23_P324304	A_23_P324304	chr2	29193074	29193134	-	ALK	anaplastic lymphoma receptor tyrosine kinase [Source:HGNC Symbol;Acc:HGNC:427]	ENSG00000171094	protein_coding	3'UTR	3'UTR	1	1980	2:29193074:29193134	ENSG00000171094	ALK	4.355358699	0.031740281	0.714634886	1.149285094	1.125866529	0.434650208	0.411231643	-0.023418565	9.961963646	0.491241419	0.277283186	0.286613771	0.673080592	0.689609658	0.981778844	-6.726638474	-5.529987906	-6.39581425	-7.047271869	-6.207517171	-7.071078925	0.576285898	0.643628237

HEXB	-0.053774511	0.048958653	0.067717703	0.102733164	0.121492213	0.018759049	8	0.05714361	0.816496581	0.816496581	0.816496581	0.816496581	0.816496581	0.816496581	12	113	0	P_253372	P_253372	P_253372	chr5	74718911	74718971	+	HEXB	hexosaminidase B (beta polypeptide) [Source:HGNC Symbol;Acc:HGNC:4879]	ENSG00000049860	protein_coding	exon	coding exon	1	1983	5:74718911:74718971	ENSG00000049860	HEXB	9.625019599	0.004828972	-0.947752097	0.86287473	1.193494723	1.810626827	2.14124682	0.330619993	9.961963646	0.365678659	0.408499143	0.260323171	0.100414302	0.058015372	0.747779992	-6.529224828	-5.804139514	-6.320425078	-5.601196999	-4.304852544	-7.011989866	1.839972254	0.203899835

NUP160	-0.260323387	-0.146555172	-0.191584775	0.113768216	0.068738612	-0.045029603	8	0.144514929	0.816496581	0.816496581	0.816496581	0.816496581	0.816496581	0.816496581	12	114	0	A_23_P43726	A_23_P43726	A_23_P43726	chr11	47778733	47778793	-	NUP160	nucleoporin 160kDa [Source:HGNC Symbol;Acc:HGNC:18017]	ENSG00000030066	protein_coding	3'UTR	3'UTR	1	1984	11:47778733:47778793	ENSG00000030066	NUP160	8.123472615	0.018978128	-2.314366468	-1.302927018	-1.703256028	1.01143945	0.61111044	-0.40032901	9.961963646	0.043278135	0.221910901	0.119459901	0.335741448	0.554813571	0.697364383	-4.664095675	-5.358571342	-5.658093614	-6.617224534	-6.10180252	-6.984527828	1.918620185	0.190786251

RAB27A	-0.561531474	0.071312501	-0.568596132	0.632843975	-0.007064659	-0.639908634	8	0.085358999	0.816496581	0.816496581	0.816496581	0.816496581	0.816496581	0.816496581	12	117	0	P_116588	P_116588	P_116588	chr15	55202965	55203025	-	RAB27A	"RAB27A, member RAS oncogene family [Source:HGNC Symbol;Acc:HGNC:9766]"	ENSG00000069974	protein_coding	3'UTR	3'UTR	1	1987	15:55202965:55203025	ENSG00000069974	RAB27A	7.689717974	0.008057867	-7.66142744	0.972974055	-7.757816286	8.634401495	-0.096388846	-8.730790341	9.961963646	1.75E-05	0.353598472	1.57E-05	6.15E-06	0.925123277	5.57E-06	3.324143667	-5.70626632	3.410412225	4.327164162	-6.291745979	4.466696181	45.1027904	4.19E-06

GPI	-0.120548514	0.059074257	-0.069217135	0.179622771	0.051331379	-0.128291391	8	0.121060047	0.816496581	0.816496581	0.816496581	0.816496581	0.816496581	0.816496581	12	118	0	A_24_P928119	A_24_P928119	A_24_P928119	chr19	34387425	34387485	-	GPI	glucose-6-phosphate isomerase [Source:HGNC Symbol;Acc:HGNC:4458]	ENSG00000105220	protein_coding	intron;promoter	multiple non-Ref	0	1988	19:34387425:34387485	ENSG00000105220	GPI	8.609085694	0.013975878	-1.248872049	0.612004125	-0.717083455	1.860876173	0.531788593	-1.32908758	9.961963646	0.240261428	0.554245354	0.489794085	0.092495408	0.606527059	0.213454723	-6.208145785	-5.988408524	-6.773365251	-5.525964325	-6.148433308	-6.183513529	1.326762027	0.320059811

SLCO1C1	0.069580822	-0.053154771	-0.037810586	-0.122735593	-0.107391408	0.015344185	8	0.100036772	0.816496581	0.816496581	0.816496581	0.816496581	0.816496581	0.816496581	12	119	0	P_214575	P_214575	P_214575	chr12	20717170	20717230	+	SLCO1C1	"solute carrier organic anion transporter family, member 1C1 [Source:HGNC Symbol;Acc:HGNC:13819]"	ENSG00000139155	protein_coding	3'UTR;exon	multiple Ref	1	1989	12:20717170:20717230	ENSG00000139155	SLCO1C1	3.505067933	0.010243136	0.842012827	-0.643237573	-0.457554216	-1.485250399	-1.299567043	0.185683356	9.961963646	0.419535383	0.534595437	0.657089424	0.16841925	0.223016835	0.856419343	-6.624726896	-5.968559935	-6.933982808	-6.060581946	-5.469168808	-7.052575655	0.876902932	0.485362757

ANKRD37	0.133311004	0.238329639	-0.162051633	0.105018635	-0.295362637	-0.400381272	8	0.093648404	0.816496581	0.816496581	0.816496581	0.816496581	0.816496581	0.816496581	12	120	0	P_251875	P_251875	P_251875	chr4	185398968	185399028	+	ANKRD37	ankyrin repeat domain 37 [Source:HGNC Symbol;Acc:HGNC:29593]	ENSG00000186352	protein_coding	exon	coding exon	1	1990	4:185398968:185399028	ENSG00000186352	ANKRD37	9.532935194	0.009249491	1.697667796	3.035042423	-2.063669386	1.337374628	-3.761337181	-5.098711809	9.961963646	0.120535654	0.012620931	0.066086195	0.210832635	0.003738908	0.000470486	-5.618562082	-2.801445541	-5.113658158	-6.250511173	-1.705423023	-0.136128884	9.714230031	0.002640958

NCDN	-0.046149082	0.152785435	0.137245394	0.198934517	0.183394476	-0.015540041	8	0.130430497	0.816496581	0.816496581	0.816496581	0.816496581	0.816496581	0.816496581	12	121	0	A_23_P97736	A_23_P97736	A_23_P97736	chr1	35566287	35566347	+	NCDN	neurochondrin [Source:HGNC Symbol;Acc:HGNC:17597]	ENSG00000020129	protein_coding	3'UTR	3'UTR	1	1991	1:35566287:35566347	ENSG00000020129	NCDN	8.08430476	0.01586834	-0.448686451	1.485463011	1.334374287	1.934149462	1.783060738	-0.151088724	9.961963646	0.663259797	0.168363986	0.211778844	0.081981351	0.10501918	0.882921412	-6.889606093	-5.136003596	-6.15307751	-5.41451666	-4.836432395	-7.058921984	1.855257028	0.201271454

CEBPA	0.19931986	-0.016384172	0.11639024	-0.215704033	-0.08292962	0.132774413	8	0.070745734	0.816496581	0.816496581	0.816496581	0.816496581	0.816496581	0.816496581	12	122	0	A_23_P375494	A_23_P375494	A_23_P375494	chr19	33300241	33300301	-	CEBPA	"CCAAT/enhancer binding protein (C/EBP), alpha [Source:HGNC Symbol;Acc:HGNC:1833]"	ENSG00000245848	protein_coding	3'UTR	3'UTR	1	1992	19:33300241:33300301	ENSG00000245848	CEBPA	11.78636435	0.006225939	3.093810996	-0.254312505	1.806590695	-3.348123501	-1.287220301	2.0609032	9.961963646	0.011419501	0.804426477	0.101076734	0.00742721	0.227119943	0.066392863	-3.350559399	-6.148107483	-5.507166094	-3.054638625	-5.483633072	-5.141450576	5.002383391	0.022698922

RPS6KA5	-0.123946819	0.017831488	-0.034328109	0.141778307	0.08961871	-0.052159597	8	0.054830086	0.816496581	0.816496581	0.816496581	0.816496581	0.816496581	0.816496581	12	123	0	P_115574	P_115574	P_115574	chr14	90871553	90871613	+	RPS6KA5	"ribosomal protein S6 kinase, 90kDa, polypeptide 5 [Source:HGNC Symbol;Acc:HGNC:10434]"	ENSG00000100784	protein_coding	3'ext;3'UTR	multiple non-Ref	0	1993	14:90871553:90871613	ENSG00000100784	RPS6KA5	6.583851817	0.004620938	-2.233140066	0.321268508	-0.618486834	2.554408574	1.614653232	-0.939755342	9.961963646	0.049675279	0.754645733	0.550133436	0.028725563	0.137576223	0.369570313	-4.796226524	-6.127967863	-6.842159224	-4.40996518	-5.070064934	-6.60925871	2.58131705	0.112007611

ASCC3	-0.133575852	-0.01117852	-0.083174816	0.122397332	0.050401037	-0.071996296	8	0.061963888	0.816496581	0.816496581	0.816496581	0.816496581	0.816496581	0.816496581	12	124	0	P_258435	P_258435	P_258435	chr6	100509931	100509991	-	ASCC3	activating signal cointegrator 1 complex subunit 3 [Source:HGNC Symbol;Acc:HGNC:18697]	ENSG00000112249	protein_coding	exon	coding exon	1	1994	6:100509931:100509991	ENSG00000112249	ASCC3	7.727008995	0.005290031	-2.249285022	-0.188235201	-1.400581496	2.061049821	0.848703526	-1.212346296	9.961963646	0.048334783	0.854471401	0.191708812	0.066376575	0.415973988	0.253347748	-4.770080957	-6.163442945	-6.069941738	-5.217069154	-5.927224746	-6.322795849	2.249058619	0.145405242

NR6A1	-0.106367826	0.124376844	0.017919694	0.230744669	0.12428752	-0.10645715	8	0.076501764	0.816496581	0.816496581	0.816496581	0.816496581	0.816496581	0.816496581	12	125	0	P_268920	P_268920	P_268920	chr9	124536004	124536064	-	NR6A1	"nuclear receptor subfamily 6, group A, member 1 [Source:HGNC Symbol;Acc:HGNC:7985]"	ENSG00000148200	protein_coding	exon	coding exon	1	1995	9:124536004:124536064	ENSG00000148200	NR6A1	4.858006719	0.006906577	-1.567561169	1.832963205	0.264085651	3.400524375	1.831646821	-1.568877554	9.961963646	0.148170048	0.096820041	0.797096669	0.006801643	0.097028533	0.147864381	-5.801009328	-4.665995153	-7.009167885	-2.96501327	-4.766847785	-5.870722529	3.877769373	0.044900195

VSTM1	-0.039416386	-0.216792401	0.012925102	-0.177376015	0.052341488	0.229717503	8	0.312228416	0.816496581	0.816496581	0.816496581	0.816496581	0.816496581	0.816496581	12	126	0	P_126708	P_126708	P_126708	chr19	54040833	54040893	-	VSTM1	V-set and transmembrane domain containing 1 [Source:HGNC Symbol;Acc:HGNC:29455]	ENSG00000189068	protein_coding	3'UTR	3'UTR	1	1996	19:54040833:54040893	ENSG00000189068	VSTM1	5.131406894	0.080493726	-0.170153705	-0.935855222	0.055795426	-0.765701517	0.225949131	0.991650648	9.961963646	0.868295191	0.371479087	0.956607961	0.461610328	0.825809247	0.344842887	-6.982584383	-5.740357585	-7.045420758	-6.838121182	-6.269602494	-6.559225275	0.42143554	0.741742954

SPATS2	0.131071644	-0.05513627	0.108073261	-0.186207914	-0.022998383	0.163209532	8	0.081550778	0.816496581	0.816496581	0.816496581	0.816496581	0.816496581	0.816496581	12	127	0	P_111476	P_111476	P_111476	chr12	49527285	49527345	+	SPATS2	"spermatogenesis associated, serine-rich 2 [Source:HGNC Symbol;Acc:HGNC:18650]"	ENSG00000123352	protein_coding	3'UTR	3'UTR	1	1997	12:49527285:49527345	ENSG00000123352	SPATS2	8.330248894	0.007547422	1.847800439	-0.777291118	1.523577593	-2.625091557	-0.324222846	2.300868711	9.961963646	0.094498524	0.455048992	0.158708	0.025449862	0.752474237	0.044282707	-5.398384964	-5.873252853	-5.908524546	-4.290800598	-6.241075775	-4.755947098	3.10500993	0.075981826

CROCC	0.110579041	-0.037186406	-0.041022962	-0.147765447	-0.151602003	-0.003836555	8	0.156312645	0.816496581	0.816496581	0.816496581	0.816496581	0.816496581	0.816496581	12	128	0	P_100577	P_100577	P_100577	chr1	16972841	16972901	-	CROCC;MFAP2	"ciliary rootlet coiled-coil, rootletin [Source:HGNC Symbol;Acc:HGNC:21299];microfibrillar-associated protein 2 [Source:HGNC Symbol;Acc:HGNC:7033]"	ENSG00000058453	protein_coding	3'ext;3'UTR	multiple non-Ref	0	1998	1:16972841:16972901	ENSG00000058453	CROCC	6.118096542	0.021828232	0.91666188	-0.308262406	-0.340066118	-1.224924286	-1.256727998	-0.031803712	9.961963646	0.380975459	0.764231934	0.740868187	0.248777858	0.237519863	0.975256759	-6.558296696	-6.132239907	-6.984332162	-6.385627013	-5.518936004	-7.07082598	0.688625987	0.579423365

HACL1	0.121735274	-0.014579024	0.113312795	-0.136314298	-0.008422479	0.127891818	8	0.048236615	0.816496581	0.816496581	0.816496581	0.816496581	0.816496581	0.816496581	12	129	0	P_134182	P_134182	P_134182	chr3	15563484	15563544	+	HACL1	2-hydroxyacyl-CoA lyase 1 [Source:HGNC Symbol;Acc:HGNC:17856]	ENSG00000131373	protein_coding	3'UTR;exon	multiple non-Ref	0	1999	3:15563484:15563544	ENSG00000131373	HACL1	8.609005061	0.004075208	2.335539279	-0.279704324	2.173950693	-2.615243602	-0.161588585	2.453655017	9.961963646	0.041746912	0.78542589	0.054903544	0.02588294	0.874860064	0.034129119	-4.629422661	-6.141012251	-4.93833535	-4.307439074	-6.282816007	-4.503800908	3.857512127	0.045494251

MRPS22	-0.10037623	0.039736578	-0.003742295	0.140112807	0.096633934	-0.043478873	8	0.108988147	0.816496581	0.816496581	0.816496581	0.816496581	0.816496581	0.816496581	12	130	0	P_136157	P_136157	P_136157	chr3	139357790	139357850	+	MRPS22;COPB2	"mitochondrial ribosomal protein S22 [Source:HGNC Symbol;Acc:HGNC:14508];coatomer protein complex, subunit beta 2 (beta prime) [Source:HGNC Symbol;Acc:HGNC:2232]"	ENSG00000175110	protein_coding	"3'ext;3'ext,3'UTR;3'UTR;intron"	multiple non-Ref	0	2000	3:139357790:139357850	ENSG00000175110	MRPS22	8.118990395	0.0117457	-1.134323833	0.449052006	-0.04229064	1.583375839	1.092033194	-0.491342645	9.961963646	0.283216216	0.66300491	0.967102631	0.144534515	0.300524053	0.633828694	-6.3383473	-6.076890435	-7.046143974	-5.927598978	-5.698742319	-6.94107508	0.905193317	0.472633697

WAS	0.147033384	-0.249151287	-0.176107864	-0.39618467	-0.323141247	0.073043423	8	0.262689255	0.816496581	0.816496581	0.816496581	0.816496581	0.816496581	0.816496581	12	131	0	A_23_P96331	A_23_P96331	A_23_P96331	chrX	48691134	48691194	+	WAS	Wiskott-Aldrich syndrome [Source:HGNC Symbol;Acc:HGNC:12731]	ENSG00000015285	protein_coding	"3'UTR,exon"	multiple Ref	1	2001	X:48691134:48691194	ENSG00000015285	WAS	5.841717253	0.057621979	0.75018348	-1.271202324	-0.898525264	-2.021385804	-1.648708743	0.37267706	9.961963646	0.470490852	0.232535534	0.390106187	0.070924022	0.130340485	0.717195321	-6.699687598	-5.395228248	-6.623301303	-5.279348167	-5.023831522	-6.99603118	1.654867922	0.239096546

TM9SF1	0.045052805	0.161005771	0.106855981	0.115952966	0.061803175	-0.05414979	8	0.059807043	0.816496581	0.816496581	0.816496581	0.816496581	0.816496581	0.816496581	12	134	0	A_23_P151586	A_23_P151586	A_23_P151586	chr14	24189509	24189569	-	TM9SF1	transmembrane 9 superfamily member 1 [Source:HGNC Symbol;Acc:HGNC:11864]	ENSG00000100926	protein_coding	3'UTR;exon	multiple Ref	1	2004	14:24189509:24189569	ENSG00000100926	TM9SF1	10.61950133	0.005079116	0.774236106	2.766897215	1.836328681	1.992661109	1.062092575	-0.930568533	9.961963646	0.456772642	0.019959619	0.096288887	0.074399648	0.313262628	0.374077738	-6.6807909	-3.23067598	-5.46289477	-5.324137996	-5.729349602	-6.617882569	2.931993583	0.086115868

SLC44A2	-0.079015692	-0.099902749	-0.126670089	-0.020887057	-0.047654397	-0.026767341	8	0.07371092	0.816496581	0.816496581	0.816496581	0.816496581	0.816496581	0.816496581	12	135	0	P_124608	P_124608	P_124608	chr19	10635364	10635424	+	SLC44A2	"solute carrier family 44 (choline transporter), member 2 [Source:HGNC Symbol;Acc:HGNC:17292]"	ENSG00000129353	protein_coding	exon	non-coding exon	1	2005	19:10635364:10635424	ENSG00000129353	SLC44A2	9.323852901	0.00656992	-1.193930202	-1.509534447	-1.913990015	-0.315604244	-0.720059813	-0.404455569	9.961963646	0.260160318	0.162208035	0.084757101	0.758815342	0.488038356	0.694424918	-6.271763778	-5.105270841	-5.345648406	-7.095414005	-6.028076846	-6.982742782	1.358199639	0.311084938

ERP44	-0.067554475	0.016633515	-0.027667387	0.08418799	0.039887088	-0.044300902	8	0.087554685	0.816496581	0.816496581	0.816496581	0.816496581	0.816496581	0.816496581	12	136	0	P_149247	P_149247	P_149247	chr9	99974436	99974496	+	ERP44;STX17	endoplasmic reticulum protein 44 [Source:HGNC Symbol;Acc:HGNC:18311];syntaxin 17 [Source:HGNC Symbol;Acc:HGNC:11432]	ENSG00000023318	protein_coding	3'ext	multiple non-Ref	0	2006	9:99974436:99974496	ENSG00000023318	ERP44	10.11021092	0.008362758	-0.904742499	0.222769081	-0.370543341	1.12751158	0.534199158	-0.593312422	9.961963646	0.386958966	0.828216355	0.718734987	0.285950422	0.6049192	0.566198028	-6.569225698	-6.155993633	-6.972654201	-6.495552161	-6.147105709	-6.88241221	0.485693025	0.699765745

CATSPERD	0.209820285	0.386561184	-0.059691339	0.176740899	-0.269511624	-0.446252523	8	0.152110751	0.816496581	0.816496581	0.816496581	0.816496581	0.816496581	0.816496581	12	137	0	P_232117	P_232117	P_232117	chr19	5751763	5751823	+	CATSPERD	catsper channel auxiliary subunit delta [Source:HGNC Symbol;Acc:HGNC:28598]	ENSG00000174898	protein_coding	exon	coding exon	1	2007	19:5751763:5751823	ENSG00000174898	CATSPERD	4.031896293	0.020787503	1.782345679	3.283694219	-0.507055841	1.50134854	-2.28940152	-3.79075006	9.961963646	0.105141167	0.008278497	0.623150247	0.164279135	0.045153946	0.003563166	-5.495555412	-2.404472072	-6.908507093	-6.039127439	-4.074765296	-2.232397435	6.013617042	0.013166328

DNAJB6	0.436848771	0.091069363	0.506159535	-0.345779408	0.069310764	0.415090172	8	0.103454984	0.816496581	0.816496581	0.816496581	0.816496581	0.816496581	0.816496581	12	139	0	A_16_P01844000	A_16_P01844000	A_16_P01844000	chr7	157335489	157335549	+	DNAJB6	"DnaJ (Hsp40) homolog, subfamily B, member 6 [Source:HGNC Symbol;Acc:HGNC:14888]"	ENSG00000105993	protein_coding	intron;promoter	multiple non-Ref	0	2009	7:157335489:157335549	ENSG00000105993	DNAJB6	6.608526956	0.010801723	5.147901443	1.073177116	5.964671468	-4.074724327	0.816770026	4.891494352	9.961963646	0.000438049	0.308499366	0.000140647	0.002250831	0.433157783	0.000638105	-0.004618806	-5.608905008	1.139589852	-1.828603602	-5.953610137	-0.453207938	17.40451879	0.000275037

ZC3H14	-0.126498594	-0.065561016	-0.162798367	0.060937579	-0.036299772	-0.097237351	8	0.046037868	0.816496581	0.816496581	0.816496581	0.816496581	0.816496581	0.816496581	12	140	0	P_221797	P_221797	P_221797	chr14	88575880	88575940	+	ZC3H14	zinc finger CCCH-type containing 14 [Source:HGNC Symbol;Acc:HGNC:20509]	ENSG00000100722	protein_coding	exon	coding exon	1	2010	14:88575880:88575940	ENSG00000100722	ZC3H14	9.485360799	0.003908746	-2.47806441	-1.28431798	-3.189164594	1.19374643	-0.711100184	-1.904846614	9.961963646	0.032734383	0.228093431	0.009713061	0.260229032	0.493335438	0.086044764	-4.393856312	-5.380150373	-3.232197838	-6.421554018	-6.034552536	-5.383128828	3.920030222	0.04369033

LUC7L	0.115686924	-0.135219496	-0.034786611	-0.25090642	-0.150473535	0.100432885	8	0.085229365	0.816496581	0.816496581	0.816496581	0.816496581	0.816496581	0.816496581	12	141	0	P_117691	P_117691	P_117691	chr16	189125	189185	-	LUC7L	LUC7-like (S. cerevisiae) [Source:HGNC Symbol;Acc:HGNC:6723]	ENSG00000007392	protein_coding	3'UTR;exon	multiple Ref	1	2011	16:189125:189185	ENSG00000007392	LUC7L	8.874258485	0.008040108	1.580152354	-1.846945171	-0.475145709	-3.427097524	-2.055298062	1.371799462	9.961963646	0.145269077	0.094630934	0.64492895	0.006505457	0.067018417	0.200227219	-5.783723653	-4.646034571	-6.92521538	-2.91960844	-4.43595927	-6.13031431	3.997488783	0.041572033

ENPP3	-0.07102121	-0.081061173	-0.034438614	-0.010039963	0.036582596	0.04662256	8	0.073978686	0.816496581	0.816496581	0.816496581	0.816496581	0.816496581	0.816496581	12	142	0	P_259226	P_259226	P_259226	chr6	131652607	131652667	+	ENPP3	ectonucleotide pyrophosphatase/phosphodiesterase 3 [Source:HGNC Symbol;Acc:HGNC:3358]	ENSG00000154269	protein_coding	3'UTR;exon	multiple Ref	1	2012	6:131652607:131652667	ENSG00000154269	ENPP3	4.641243198	0.006601678	-1.070548972	-1.221887879	-0.519115663	-0.151338907	0.551433309	0.702772215	9.961963646	0.309623703	0.24987492	0.615016114	0.882729165	0.593488792	0.498290874	-6.406663015	-5.450930006	-6.901924048	-7.137102388	-6.137449592	-6.808086274	0.621572851	0.617010682

SLC35D1	0.024496988	-0.047489165	-0.14773039	-0.071986153	-0.172227378	-0.100241225	8	0.239047691	0.816496581	0.816496581	0.816496581	0.816496581	0.816496581	0.816496581	12	143	0	P_202175	P_202175	P_202175	chr1	67052011	67052071	-	SLC35D1	"solute carrier family 35 (UDP-GlcA/UDP-GalNAc transporter), member D1 [Source:HGNC Symbol;Acc:HGNC:20800]"	ENSG00000116704	protein_coding	exon	coding exon	1	2013	1:67052011:67052071	ENSG00000116704	SLC35D1	3.909014653	0.04809627	0.136805203	-0.265206685	-0.825011069	-0.402011888	-0.961816272	-0.559804384	9.961963646	0.893910071	0.796257207	0.428678199	0.696164983	0.358906205	0.587978248	-6.988156986	-6.145144752	-6.687652398	-7.061945546	-5.826880043	-6.902833683	0.36162976	0.782153562

ARPC5L	-0.070486196	0.007607967	0.02158567	0.078094163	0.092071866	0.013977703	8	0.065620066	0.816496581	0.816496581	0.816496581	0.816496581	0.816496581	0.816496581	12	144	0	P_149651	P_149651	P_149651	chr9	124868609	124868669	+	ARPC5L	"actin related protein 2/3 complex, subunit 5-like [Source:HGNC Symbol;Acc:HGNC:23366]"	ENSG00000136950	protein_coding	5'UTR	5'UTR	1	2014	9:124868609:124868669	ENSG00000136950	ARPC5L	10.60698647	0.005664631	-1.147001229	0.123802213	0.351257289	1.270803442	1.498258518	0.227455076	9.961963646	0.278182501	0.903934177	0.732710763	0.232671735	0.165066897	0.824669996	-6.324403276	-6.174013939	-6.980157519	-6.331527548	-5.223807514	-7.043188726	0.894296046	0.477495193

MDM2	0.195947922	0.084072068	0.279571842	-0.111875854	0.08362392	0.195499774	8	0.084632889	0.816496581	0.816496581	0.816496581	0.816496581	0.816496581	0.816496581	12	145	0	P_112187	P_112187	P_112187	chr12	68824591	68824651	+	MDM2	"MDM2 proto-oncogene, E3 ubiquitin protein ligase [Source:HGNC Symbol;Acc:HGNC:6973]"	ENSG00000135679	protein_coding	3'UTR;exon	multiple Ref	1	2015	12:68824591:68824651	ENSG00000135679	MDM2	9.105962773	0.007958743	2.690072844	1.154184158	3.838104592	-1.535888686	1.148031748	2.683920434	9.961963646	0.022767926	0.27536181	0.003298126	0.155694135	0.277776431	0.023009257	-4.037953885	-5.524767011	-2.131566599	-5.992607995	-5.639735238	-4.116992246	5.69667328	0.015521287

VWA3B	0.076213298	0.083382544	0.021668205	0.007169246	-0.054545093	-0.061714339	8	0.141246292	0.816496581	0.816496581	0.816496581	0.816496581	0.816496581	0.816496581	12	146	0	P_235851	P_235851	P_235851	chr2	98133879	98133939	+	VWA3B	von Willebrand factor A domain containing 3B [Source:HGNC Symbol;Acc:HGNC:28385]	ENSG00000168658	protein_coding	3'UTR;exon	multiple Ref	1	2016	2:98133879:98133939	ENSG00000168658	VWA3B	3.83162389	0.018228035	0.691363504	0.756398811	0.196561578	0.065035306	-0.494801926	-0.559837232	9.961963646	0.505128943	0.466920954	0.848122692	0.949432464	0.631470116	0.587956679	-6.743642258	-5.889163664	-7.026061953	-7.147300535	-6.168090803	-6.902814209	0.275205896	0.842002953

SENP2	-0.076167568	-0.053924641	-0.123913849	0.022242927	-0.04774628	-0.069989208	8	0.053165364	0.816496581	0.816496581	0.816496581	0.816496581	0.816496581	0.816496581	12	147	0	P_136754	P_136754	P_136754	chr3	185626318	185626378	+	SENP2	SUMO1/sentrin/SMT3 specific peptidase 2 [Source:HGNC Symbol;Acc:HGNC:23116]	ENSG00000163904	protein_coding	3'UTR;exon	multiple Ref	1	2017	3:185626318:185626378	ENSG00000163904	SENP2	8.361812724	0.004476563	-1.394258795	-0.987098664	-2.268261634	0.407160131	-0.874002839	-1.28116297	9.961963646	0.193553283	0.346962009	0.046803936	0.692501257	0.40269461	0.229155568	-6.030049712	-5.693008381	-4.785891722	-7.059704454	-5.905703796	-6.241801842	1.772395182	0.216006989

SYTL4	0.091402476	0.123588369	0.183261665	0.032185893	0.091859189	0.059673296	8	0.063956037	0.816496581	0.816496581	0.816496581	0.816496581	0.816496581	0.816496581	12	148	0	P_270897	P_270897	P_270897	chrX	100701927	100701987	-	SYTL4	synaptotagmin-like 4 [Source:HGNC Symbol;Acc:HGNC:15588]	ENSG00000102362	protein_coding	exon	coding exon	1	2018	X:100701927:100701987	ENSG00000102362	SYTL4	6.880620759	0.005491478	1.510633015	2.042577816	3.028814233	0.5319448	1.518181218	0.986236418	9.961963646	0.161931827	0.068458862	0.012755586	0.606422801	0.160045083	0.347364495	-5.878110353	-4.35996381	-3.506550765	-6.997038827	-5.197981736	-6.564548803	3.198058942	0.071117885

PSMB6	-0.0117179	0.067678899	0.017686052	0.079396799	0.029403952	-0.049992847	8	0.063013985	0.816496581	0.816496581	0.816496581	0.816496581	0.816496581	0.816496581	12	149	0	P_120179	P_120179	P_120179	chr17	4798008	4798068	-	PSMB6	"proteasome (prosome, macropain) subunit, beta type, 6 [Source:HGNC Symbol;Acc:HGNC:9543]"	ENSG00000142507	protein_coding	3'UTR;exon	multiple non-Ref	0	2019	17:4798008:4798068	ENSG00000142507	PSMB6	12.41250913	0.005395423	-0.195381157	1.128460025	0.294892542	1.323841182	0.490273698	-0.833567483	9.961963646	0.84902206	0.285568517	0.774129992	0.21512874	0.634558387	0.424060268	-6.97756931	-5.551993139	-6.999837589	-6.267216595	-6.170405083	-6.704530193	0.68105266	0.583559304

SGCD	0.034626426	-0.011875349	0.118572598	-0.046501775	0.083946171	0.130447947	8	0.169686782	0.816496581	0.816496581	0.816496581	0.816496581	0.816496581	0.816496581	12	150	0	P_255516	P_255516	P_255516	chr5	156589258	156589318	+	SGCD	"sarcoglycan, delta (35kDa dystrophin-associated glycoprotein) [Source:HGNC Symbol;Acc:HGNC:10807]"	ENSG00000170624	protein_coding	exon	coding exon	1	2020	5:156589258:156589318	ENSG00000170624	SGCD	3.928148569	0.025329518	0.266464795	-0.091385765	0.9124656	-0.357850559	0.646000806	1.003851365	9.961963646	0.795315422	0.928997558	0.383074459	0.727921044	0.53287673	0.339210113	-6.959752818	-6.177686746	-6.610570575	-7.080031942	-6.079393534	-6.547141621	0.410839991	0.748817169

NEUROG3	0.206311875	-0.021565748	0.17193469	-0.227877623	-0.034377184	0.193500438	8	0.20082356	0.816496581	0.816496581	0.816496581	0.816496581	0.816496581	0.816496581	12	151	0	P_106158	P_106158	P_106158	chr10	69572398	69572458	-	NEUROG3	neurogenin 3 [Source:HGNC Symbol;Acc:HGNC:13806]	ENSG00000122859	protein_coding	exon	coding exon	1	2021	10:69572398:69572458	ENSG00000122859	NEUROG3	9.449313651	0.034593955	1.358531604	-0.142007097	1.132163193	-1.500538701	-0.226368411	1.27417029	9.961963646	0.20426034	0.889905268	0.284081199	0.164485276	0.825492018	0.231524156	-6.075071923	-6.171467667	-6.38893697	-6.040210219	-6.269502155	-6.250179432	1.178989435	0.366316139

ABHD17A	-0.014696047	0.056504553	0.028298081	0.0712006	0.042994128	-0.028206472	8	0.097429755	0.816496581	0.816496581	0.816496581	0.816496581	0.816496581	0.816496581	12	152	0	P_310819	P_310819	P_310819	chr19	1881210	1881270	-	ABHD17A	abhydrolase domain containing 17A [Source:HGNC Symbol;Acc:HGNC:28756]	ENSG00000129968	protein_coding	"exon,intron;promoter"	multiple non-Ref	0	2022	19:1881210:1881270	ENSG00000129968	ABHD17A	7.373857738	0.009829725	-0.181541313	0.698004773	0.349568209	0.879546086	0.531109522	-0.348436564	9.961963646	0.859583307	0.501141408	0.733939755	0.399824615	0.606980401	0.734763593	-6.98040573	-5.931581117	-6.980796048	-6.742273333	-6.148806278	-7.00545663	0.303242087	0.822484076

STAM	0.014835249	0.030382811	0.000169878	0.015547562	-0.014665371	-0.030212933	8	0.069946561	0.816496581	0.816496581	0.816496581	0.816496581	0.816496581	0.816496581	12	153	0	P_207593	P_207593	P_207593	chr10	17660481	17660541	+	STAM	signal transducing adaptor molecule (SH3 domain and ITAM motif) 1 [Source:HGNC Symbol;Acc:HGNC:11357]	ENSG00000136738	protein_coding	exon	coding exon	1	2023	10:17660481:17660541	ENSG00000136738	STAM	8.266653333	0.006135645	0.231958525	0.475054514	0.002656149	0.243095989	-0.229302377	-0.472398365	9.961963646	0.821265682	0.644991712	0.99793314	0.812863342	0.823273091	0.646821006	-6.969079131	-6.064500077	-7.04711675	-7.11739168	-6.268794873	-6.950820275	0.102387343	0.956788041

THRAP3	-0.151823105	-0.029802062	-0.165786413	0.122021043	-0.013963308	-0.135984351	8	0.087351756	0.816496581	0.816496581	0.816496581	0.816496581	0.816496581	0.816496581	12	154	0	P_101302	P_101302	P_101302	chr1	36264202	36264262	-	THRAP3	thyroid hormone receptor associated protein 3 [Source:HGNC Symbol;Acc:HGNC:22964]	ENSG00000054118	protein_coding	intron	intron antisense	0	2024	1:36264202:36264262	ENSG00000054118	THRAP3	7.553953206	0.008334254	-2.036808129	-0.39981452	-2.224135209	1.636993608	-0.187327081	-1.824320689	9.961963646	0.069121857	0.697731238	0.050438487	0.132790271	0.855164495	0.098196449	-5.108795586	-6.098515092	-4.857483198	-5.852793176	-6.278054479	-5.504430585	2.549700319	0.114765313

ISYNA1	0.130575365	0.062937522	0.114552846	-0.067637843	-0.016022519	0.051615323	8	0.093524538	0.816496581	0.816496581	0.816496581	0.816496581	0.816496581	0.816496581	12	155	0	P_125145	P_125145	P_125145	chr19	18436513	18436573	-	ISYNA1	inositol-3-phosphate synthase 1 [Source:HGNC Symbol;Acc:HGNC:29821]	ENSG00000105655	protein_coding	exon	non-coding exon	1	2025	19:18436513:18436573	ENSG00000105655	ISYNA1	8.84382183	0.009230873	1.664506508	0.802294639	1.460259805	-0.862211869	-0.204246703	0.657965166	9.961963646	0.127101107	0.441101674	0.175025393	0.408846708	0.842273014	0.525472435	-5.66584761	-5.853710452	-5.992713478	-6.757609085	-6.274544564	-6.839903826	1.127443581	0.384157355

CATSPERE	0.050959625	-0.034145338	-0.046814548	-0.085104963	-0.097774173	-0.01266921	8	0.116061362	0.816496581	0.816496581	0.816496581	0.816496581	0.816496581	0.816496581	13	16	0	P_207087	P_207087	P_207087	chr1	244477915	244477975	+	C1orf101	chromosome 1 open reading frame 101 [Source:HGNC Symbol;Acc:HGNC:28491]	ENSG00000179397	protein_coding	5'UTR;exon	multiple Ref	1	2056	1:244477915:244477975	ENSG00000179397	CATSPERE	3.899140431	0.013024021	0.546889151	-0.366441373	-0.502404961	-0.913330525	-1.049294113	-0.135963588	9.961963646	0.596491555	0.721698623	0.626301531	0.38264115	0.318831759	0.894558306	-6.837572161	-6.111791807	-6.911006609	-6.711637159	-5.742228363	-7.06128893	0.439909108	0.729507321

UHRF2	-0.216139697	0.018947988	-0.300459377	0.235087684	-0.08431968	-0.319407364	8	0.04554431	0.816496581	0.816496581	0.816496581	0.816496581	0.816496581	0.816496581	13	18	0	P_266743	P_266743	P_266743	chr9	6475440	6475500	+	UHRF2	"ubiquitin-like with PHD and ring finger domains 2, E3 ubiquitin protein ligase [Source:HGNC Symbol;Acc:HGNC:12557]"	ENSG00000147854	protein_coding	exon	coding exon	1	2058	9:6475440:6475500	ENSG00000147854	UHRF2	7.297572889	0.003872447	-4.253901299	0.372920245	-5.913418737	4.626821544	-1.659517439	-6.286338982	9.961963646	0.001693575	0.717019927	0.000150629	0.000950227	0.128116237	9.22E-05	-1.403684283	-6.10930214	1.068244724	-0.933306254	-5.009045741	1.562000386	19.48046674	0.000172155

CACNG6	-0.226002994	0.033404295	-0.091689652	0.259407289	0.134313342	-0.125093947	8	0.174512177	0.816496581	0.816496581	0.816496581	0.816496581	0.816496581	0.816496581	13	19	0	A_23_P501933	A_23_P501933	A_23_P501933	chr19	54012540	54012600	+	CACNG6	"calcium channel, voltage-dependent, gamma subunit 6 [Source:HGNC Symbol;Acc:HGNC:13625]"	ENSG00000130433	protein_coding	3'UTR	3'UTR	1	2059	19:54012540:54012600	ENSG00000130433	CACNG6	5.234702753	0.026663308	-1.695129211	0.25054799	-0.687715703	1.945677202	1.007413508	-0.938263693	9.961963646	0.121027226	0.807255202	0.507327352	0.080432552	0.337578488	0.370299513	-5.622200073	-6.149102767	-6.794824008	-5.396804465	-5.783499071	-6.610663774	1.515011789	0.270357815

SPATA6	0.687505997	0.161661094	0.79770187	-0.525844903	0.110195873	0.636040776	8	0.068628212	0.816496581	0.816496581	0.816496581	0.816496581	0.816496581	0.816496581	13	20	0	A_23_P327483	A_23_P327483	A_23_P327483	chr1	48298811	48298871	-	SPATA6	spermatogenesis associated 6 [Source:HGNC Symbol;Acc:HGNC:18309]	ENSG00000132122	protein_coding	3'UTR;exon	multiple Ref	1	2060	1:48298811:48298871	ENSG00000132122	SPATA6	7.731061719	0.005988935	10.88046119	2.55844642	12.62441968	-8.322014767	1.743958493	10.06597326	9.961963646	7.54E-07	0.028527637	1.88E-07	8.53E-06	0.111883259	1.54E-06	6.502540402	-3.562573796	7.866589074	3.989280284	-4.89175944	5.775645217	76.32119911	3.55E-07

CEMIP2	-0.078323658	0.063276006	-0.166781392	0.141599664	-0.088457734	-0.230057398	8	0.077300788	0.816496581	0.816496581	0.816496581	0.816496581	0.816496581	0.816496581	13	21	0	P_148877	P_148877	P_148877	chr9	71766596	71766656	+	TMEM2	transmembrane protein 2 [Source:HGNC Symbol;Acc:HGNC:11869]	ENSG00000135048	protein_coding	intron	intron antisense	0	2061	9:71766596:71766656	ENSG00000135048	CEMIP2	6.05985555	0.007005265	-1.146109981	0.925917704	-2.440511877	2.072027685	-1.294401897	-3.366429582	9.961963646	0.278534069	0.376374562	0.034904189	0.065167727	0.224725824	0.007202187	-6.32538745	-5.749297928	-4.502544009	-5.199746635	-5.475231579	-2.951415032	4.238102172	0.035729958

PARP15	0.191746632	0.000318847	0.071561873	-0.191427785	-0.120184759	0.071243026	8	0.160199106	0.816496581	0.816496581	0.816496581	0.816496581	0.816496581	0.816496581	13	22	0	P_245867	P_245867	P_245867	chr3	122615792	122615852	+	PARP15	"poly (ADP-ribose) polymerase family, member 15 [Source:HGNC Symbol;Acc:HGNC:26876]"	ENSG00000173200	protein_coding	exon	coding exon	1	2062	3:122615792:122615852	ENSG00000173200	PARP15	3.26921764	0.022816077	1.554722495	0.002585278	0.580238895	-1.552137217	-0.9744836	0.577653617	9.961963646	0.151180538	0.997988287	0.57464245	0.151793222	0.352884809	0.576320462	-5.81854915	-6.182083901	-6.866318952	-5.970499054	-5.814993238	-6.892093564	1.074379637	0.403523917

FAT2	-0.097327148	-0.023217096	-0.124005956	0.074110053	-0.026678807	-0.10078886	8	0.171404328	0.816496581	0.816496581	0.816496581	0.816496581	0.816496581	0.816496581	13	24	0	P_255427	P_255427	P_255427	chr5	151542369	151542429	-	FAT2	FAT atypical cadherin 2 [Source:HGNC Symbol;Acc:HGNC:3596]	ENSG00000086570	protein_coding	exon	coding exon	1	2064	5:151542369:151542429	ENSG00000086570	FAT2	4.641116468	0.025799979	-0.742112849	-0.177028766	-0.945536928	0.565084083	-0.203424079	-0.768508162	9.961963646	0.475152304	0.863033256	0.366753709	0.584516824	0.842898695	0.460015786	-6.705909083	-6.165593311	-6.579709847	-6.977744494	-6.274722172	-6.757996397	0.404569487	0.753022241

C10orf55	0.202859675	0.275186325	0.313729377	0.07232665	0.110869701	0.038543051	8	0.298153162	0.816496581	0.816496581	0.816496581	0.816496581	0.816496581	0.816496581	13	25	0	P_106331	P_106331	P_106331	chr10	73910091	73910151	-	C10orf55	chromosome 10 open reading frame 55 [Source:HGNC Symbol;Acc:HGNC:31008]	ENSG00000222047	protein_coding	3'UTR;exon	multiple Ref	1	2065	10:73910091:73910151	ENSG00000222047	C10orf55	5.422770295	0.073594464	0.915838262	1.242366997	1.416374972	0.326528735	0.500536709	0.174007975	9.961963646	0.381386792	0.24255101	0.187166674	0.750780937	0.627569628	0.865344417	-6.559055763	-5.427990477	-6.049710333	-7.091617288	-6.165130869	-7.054862389	0.795965054	0.523766247

DHX15	-0.15801818	0.036689803	-0.147457307	0.194707983	0.010560874	-0.18414711	8	0.076705766	0.816496581	0.816496581	0.816496581	0.816496581	0.816496581	0.816496581	13	26	0	P_248383	P_248383	P_248383	chr4	24537050	24537110	-	DHX15	DEAH (Asp-Glu-Ala-His) box helicase 15 [Source:HGNC Symbol;Acc:HGNC:2738]	ENSG00000109606	protein_coding	exon	coding exon	1	2066	4:24537050:24537110	ENSG00000109606	DHX15	8.802899183	0.006931676	-2.324521482	0.539724197	-2.169166082	2.864245679	0.155355399	-2.708890279	9.961963646	0.042537011	0.601242332	0.055348582	0.016895245	0.879643891	0.022045414	-4.647476962	-6.030815641	-4.946010649	-3.884007741	-6.283864056	-4.074684053	4.327684829	0.033808509

TNFAIP3	-0.009997378	0.063139915	0.009173816	0.073137293	0.019171194	-0.053966099	8	0.11300216	0.816496581	0.816496581	0.816496581	0.816496581	0.816496581	0.816496581	13	28	0	P_259426	P_259426	P_259426	chr6	137876085	137876145	+	TNFAIP3	"tumor necrosis factor, alpha-induced protein 3 [Source:HGNC Symbol;Acc:HGNC:11896]"	ENSG00000118503	protein_coding	3'UTR;exon	multiple Ref	1	2068	6:137876085:137876145	ENSG00000118503	TNFAIP3	4.548357595	0.012461279	-0.109685804	0.692736876	0.100650135	0.80242268	0.210335939	-0.592086742	9.961963646	0.914836657	0.504302763	0.921825041	0.441030986	0.837645197	0.566986784	-6.991799101	-5.935257069	-7.041591092	-6.808473035	-6.273207781	-6.883178717	0.256789313	0.854825449

IQCB2P	0.025658629	0.043119851	-0.034360743	0.017461222	-0.060019372	-0.077480594	8	0.109445193	0.816496581	0.816496581	0.816496581	0.816496581	0.816496581	0.816496581	13	29	0	A_24_P455100	A_24_P455100	A_24_P455100	chr6	28011546	28011606	+	IQCB2P	IQ motif containing B2 pseudogene [Source:HGNC Symbol;Acc:HGNC:17727]	ENSG00000217539	processed_pseudogene	exon	non-coding exon	1	2069	6:28011546:28011606	ENSG00000217539	IQCB2P	6.547580184	0.011825872	0.288976477	0.485630879	-0.386982735	0.196654402	-0.675959213	-0.872613615	9.961963646	0.778523526	0.637732467	0.706907133	0.848051979	0.51445201	0.403416108	-6.952982464	-6.059270051	-6.965950651	-7.128505311	-6.059243626	-6.670631954	0.287691454	0.833306207

DAW1	0.031826454	0.019644729	0.176995288	-0.012181725	0.145168834	0.157350559	8	0.201838241	0.816496581	0.816496581	0.816496581	0.816496581	0.816496581	0.816496581	13	30	0	P_239557	P_239557	P_239557	chr2	227904958	227905018	+	DAW1	dynein assembly factor with WDR repeat domains 1 [Source:HGNC Symbol;Acc:HGNC:26383]	ENSG00000123977	protein_coding	exon	coding exon	1	2070	2:227904958:227905018	ENSG00000123977	DAW1	3.513476328	0.034922062	0.208585419	0.128748366	1.159998413	-0.079837053	0.951412994	1.031250047	9.961963646	0.838974935	0.900118959	0.273095147	0.937947899	0.363906966	0.326810256	-6.974670525	-6.173356518	-6.358183094	-7.146128482	-5.836546401	-6.519568855	0.56345294	0.651349302

ANO7	-0.073221362	-0.025201728	0.004537708	0.048019634	0.07775907	0.029739436	8	0.147911644	0.816496581	0.816496581	0.816496581	0.816496581	0.816496581	0.816496581	13	31	0	P_130360	P_130360	P_130360	chr2	241223383	241223443	+	ANO7	anoctamin 7 [Source:HGNC Symbol;Acc:HGNC:31677]	ENSG00000146205	protein_coding	exon	non-coding exon	1	2071	2:241223383:241223443	ENSG00000146205	ANO7	5.628716005	0.019775794	-0.637700073	-0.219487091	0.039519845	0.418212983	0.677219918	0.259006935	9.961963646	0.538049432	0.830702585	0.969256721	0.684663695	0.513685127	0.800903147	-6.780894181	-6.156754918	-7.046267745	-7.054799918	-6.058377437	-7.034853692	0.192835216	0.898877373

GANC	-0.071890248	0.16444954	0.020422611	0.236339788	0.092312859	-0.144026929	8	0.058362685	0.816496581	0.816496581	0.816496581	0.816496581	0.816496581	0.816496581	13	32	0	P_222976	P_222976	P_222976	chr15	42352051	42352111	+	GANC	"glucosidase, alpha; neutral C [Source:HGNC Symbol;Acc:HGNC:4139]"	ENSG00000214013	protein_coding	exon	coding exon	1	2072	15:42352051:42352111	ENSG00000214013	GANC	6.036477301	0.004942051	-1.252454659	2.865000443	0.355797828	4.117455102	1.608252487	-2.509202614	9.961963646	0.239007989	0.016873441	0.729410956	0.002102385	0.13897548	0.031036545	-6.203924719	-3.073735687	-6.978426166	-1.758003047	-5.078693639	-4.411134384	5.956578773	0.013556775

GCH1	-0.030744267	0.258488392	0.195080004	0.289232659	0.225824272	-0.063408387	8	0.131182752	0.816496581	0.816496581	0.816496581	0.816496581	0.816496581	0.816496581	13	33	0	A_23_P163079	A_23_P163079	A_23_P163079	chr14	54843553	54843613	-	GCH1	GTP cyclohydrolase 1 [Source:HGNC Symbol;Acc:HGNC:4193]	ENSG00000131979	protein_coding	3'UTR;exon	multiple Ref	1	2073	14:54843553:54843613	ENSG00000131979	GCH1	7.445198716	0.016026381	-0.297434985	2.50074234	1.887298783	2.798177325	2.184733768	-0.613443557	9.961963646	0.772244418	0.031489046	0.088567237	0.018918337	0.053913142	0.553330836	-6.950299093	-3.653827636	-5.38620239	-3.99682364	-4.237816196	-6.869611999	3.813874582	0.046806035

SLC6A11	-0.112329248	-0.050877081	-0.009829155	0.061452167	0.102500093	0.041047926	8	0.147606952	0.816496581	0.816496581	0.816496581	0.816496581	0.816496581	0.816496581	13	34	0	A_24_P589044	A_24_P589044	A_24_P589044	chr3	10832808	10832868	+	SLC6A11	"solute carrier family 6 (neurotransmitter transporter), member 11 [Source:HGNC Symbol;Acc:HGNC:11044]"	ENSG00000132164	protein_coding	intron	intron sense	0	2074	3:10832808:10832868	ENSG00000132164	SLC6A11	5.883904016	0.019703485	-0.980092255	-0.443911393	-0.085761092	0.536180862	0.894331163	0.358150301	9.961963646	0.350242454	0.666593398	0.933355507	0.603599051	0.392239417	0.727703585	-6.498129561	-6.079260892	-7.043105474	-6.994633739	-5.88802148	-7.001753757	0.396763817	0.758275359

PCDH1	-0.082536127	-0.022916846	0.084830368	0.059619281	0.167366495	0.107747214	8	0.149833789	0.816496581	0.816496581	0.816496581	0.816496581	0.816496581	0.816496581	13	35	0	A_23_P213359	A_23_P213359	A_23_P213359	chr5	141863474	141863534	-	PCDH1	protocadherin 1 [Source:HGNC Symbol;Acc:HGNC:8655]	ENSG00000156453	protein_coding	3'UTR;exon	multiple Ref	1	2075	5:141863474:141863534	ENSG00000156453	PCDH1	7.604139591	0.02023539	-0.71061427	-0.197307996	0.730367077	0.513306274	1.440981347	0.927675073	9.961963646	0.493623734	0.847554111	0.481988603	0.618927706	0.180273093	0.3755055	-6.729612581	-6.161606066	-6.763393412	-7.007406728	-5.296866038	-6.620584013	0.712971795	0.566315761

BRAP	0.032983061	0.017177743	0.007278493	-0.015805319	-0.025704568	-0.00989925	8	0.041949276	0.816496581	0.816496581	0.816496581	0.816496581	0.816496581	0.816496581	13	36	0	P_217512	P_217512	P_217512	chr12	111658735	111658795	-	BRAP	BRCA1 associated protein [Source:HGNC Symbol;Acc:HGNC:1099]	ENSG00000089234	protein_coding	exon	coding exon	1	2076	12:111658735:111658795	ENSG00000089234	BRAP	8.095934178	0.003619853	0.671415172	0.349676367	0.148163646	-0.321738804	-0.523251526	-0.201512722	9.961963646	0.517221839	0.733861034	0.885169706	0.754299905	0.612239029	0.844352902	-6.757810857	-6.118037057	-7.035145424	-7.093297564	-6.153089462	-7.049239983	0.168823293	0.914980999

HEXD	-0.042687169	0.074842404	0.016305555	0.117529573	0.058992723	-0.05853685	8	0.055692351	0.816496581	0.816496581	0.816496581	0.816496581	0.816496581	0.816496581	13	37	0	A_23_P300174	A_23_P300174	A_23_P300174	chr17	82441203	82441263	+	HEXDC	"hexosaminidase (glycosyl hydrolase family 20, catalytic domain) containing [Source:HGNC Symbol;Acc:HGNC:26307]"	ENSG00000169660	protein_coding	3'UTR;exon	multiple Ref	1	2077	17:82441203:82441263	ENSG00000169660	HEXD	6.737169228	0.004697468	-0.762800635	1.337400334	0.291372979	2.100200968	1.054173613	-1.046027355	9.961963646	0.463262156	0.210824544	0.77674278	0.062160531	0.316699705	0.320265199	-6.689841148	-5.318030062	-7.000954723	-5.155126859	-5.737332826	-6.504449379	1.511950336	0.27109259

GCC2	0.130885411	0.029821178	0.077538086	-0.101064233	-0.053347325	0.047716908	8	0.041551553	0.816496581	0.816496581	0.816496581	0.816496581	0.816496581	0.816496581	13	38	0	P_236111	P_236111	P_236111	chr2	108482373	108482433	+	GCC2	GRIP and coiled-coil domain containing 2 [Source:HGNC Symbol;Acc:HGNC:23218]	ENSG00000135968	protein_coding	3'UTR;exon	multiple Ref	1	2078	2:108482373:108482433	ENSG00000135968	GCC2	8.664175964	0.003593183	2.674220455	0.609299414	1.584240248	-2.064921042	-1.089980208	0.974940834	9.961963646	0.023394949	0.555966057	0.144338094	0.065947875	0.301384609	0.352668852	-4.064741224	-5.99008446	-5.825807152	-5.210964711	-5.700862249	-6.575578021	2.739163895	0.099354529

NR5A2	0.184337482	0.210573822	0.210043943	0.02623634	0.025706462	-0.000529879	8	0.1569443	0.816496581	0.816496581	0.816496581	0.816496581	0.816496581	0.816496581	13	40	0	P_205697	P_205697	P_205697	chr1	200027851	200027911	+	NR5A2	"nuclear receptor subfamily 5, group A, member 2 [Source:HGNC Symbol;Acc:HGNC:7984]"	ENSG00000116833	protein_coding	exon	coding exon	1	2080	1:200027851:200027911	ENSG00000116833	NR5A2	3.863960323	0.021987132	1.522561999	1.739264832	1.734888224	0.216702833	0.212326225	-0.004376608	9.961963646	0.15895889	0.112734128	0.113532822	0.832813303	0.836133979	0.996594385	-5.862099517	-4.797863057	-5.612389968	-7.12399285	-6.272762411	-7.071368061	1.407522674	0.297569714

HTR3B	0.04650637	0.009407787	0.033613894	-0.037098583	-0.012892476	0.024206107	8	0.258963018	0.816496581	0.816496581	0.816496581	0.816496581	0.816496581	0.816496581	13	41	0	P_213420	P_213420	P_213420	chr11	113944598	113944658	+	HTR3B	"5-hydroxytryptamine (serotonin) receptor 3B, ionotropic [Source:HGNC Symbol;Acc:HGNC:5298]"	ENSG00000149305	protein_coding	"3'UTR,exon;exon"	multiple Ref	1	2081	11:113944598:113944658	ENSG00000149305	HTR3B	4.264352469	0.056061002	0.240562344	0.048663427	0.173873755	-0.191898917	-0.066688589	0.125210328	9.961963646	0.814772632	0.96214934	0.865447137	0.851676513	0.94814904	0.902847762	-6.966873094	-6.180839168	-7.03063574	-7.129511951	-6.294320307	-7.062820563	0.024570107	0.994449333

SLC25A26	0.011426233	0.151406581	0.109333956	0.139980347	0.097907723	-0.042072625	8	0.084749417	0.816496581	0.816496581	0.816496581	0.816496581	0.816496581	0.816496581	13	43	0	A_16_P16260500	A_16_P16260500	A_16_P16260500	chr3	66385769	66385829	+	SLC25A26;LRIG1	"solute carrier family 25 (S-adenosylmethionine carrier), member 26 [Source:HGNC Symbol;Acc:HGNC:20661];leucine-rich repeats and immunoglobulin-like domains 1 [Source:HGNC Symbol;Acc:HGNC:17360]"	ENSG00000144741	protein_coding	intron	multiple non-Ref	0	2083	3:66385769:66385829	ENSG00000144741	SLC25A26	7.140128509	0.007974594	0.156709177	2.076519869	1.499499766	1.919810692	1.342790588	-0.577020103	9.961963646	0.878604458	0.064679124	0.164750063	0.083946768	0.209133566	0.576732061	-6.984975961	-4.309175789	-5.940804304	-5.436481761	-5.417779355	-6.892480157	2.067783989	0.168512844

KLHL21	0.006755056	-0.020491654	-0.133630619	-0.02724671	-0.140385675	-0.113138965	8	0.068004668	0.816496581	0.816496581	0.816496581	0.816496581	0.816496581	0.816496581	13	44	0	P_200126	P_200126	P_200126	chr1	6593727	6593787	-	KLHL21	kelch-like family member 21 [Source:HGNC Symbol;Acc:HGNC:29041]	ENSG00000162413	protein_coding	3'UTR;exon	multiple Ref	1	2084	1:6593727:6593787	ENSG00000162413	KLHL21	6.952258681	0.005920518	0.107521349	-0.326169086	-2.127020918	-0.433690434	-2.234542267	-1.800851833	9.961963646	0.916510024	0.751044949	0.059421147	0.67375451	0.049557445	0.102025547	-6.992055345	-6.126313383	-5.013356779	-7.047719118	-4.160509143	-5.539300743	2.177749826	0.154023282

EREG	-0.341462575	-0.142775321	-0.09059321	0.198687253	0.250869364	0.052182111	8	0.174278527	0.816496581	0.816496581	0.816496581	0.816496581	0.816496581	0.816496581	13	46	0	P_137929	P_137929	P_137929	chr4	74384744	74384804	+	EREG	epiregulin [Source:HGNC Symbol;Acc:HGNC:3443]	ENSG00000124882	protein_coding	exon	coding exon	1	2086	4:74384744:74384804	ENSG00000124882	EREG	3.163561705	0.026597863	-2.564279531	-1.072199009	-0.680327309	1.492080522	1.883952221	0.391871699	9.961963646	0.028244097	0.308917442	0.511797852	0.166651748	0.089056045	0.703405167	-4.24979396	-5.609891917	-6.800094428	-6.051497067	-4.690953563	-6.988130841	2.35285486	0.133849717

WDR75	-0.10679065	-0.01065075	-0.152594989	0.0961399	-0.045804339	-0.141944239	8	0.062128381	0.816496581	0.816496581	0.816496581	0.816496581	0.816496581	0.816496581	13	47	0	P_129456	P_129456	P_129456	chr2	189475425	189475485	+	WDR75	WD repeat domain 75 [Source:HGNC Symbol;Acc:HGNC:25725]	ENSG00000115368	protein_coding	3'UTR	3'UTR	1	2087	2:189475425:189475485	ENSG00000115368	WDR75	8.882161209	0.005306423	-1.795469094	-0.179070855	-2.565576532	1.616398238	-0.770107439	-2.386505677	9.961963646	0.102922911	0.861471652	0.028181432	0.137196932	0.459108784	0.038274698	-5.476213908	-6.165211226	-4.293642071	-5.88169887	-5.990574653	-4.615151379	3.12319611	0.075000923

NUP98	-0.259567772	-0.100747736	-0.312473867	0.158820036	-0.052906095	-0.211726131	8	0.055653527	0.816496581	0.816496581	0.816496581	0.816496581	0.816496581	0.816496581	13	48	0	A_23_P203586	A_23_P203586	A_23_P203586	chr11	3675516	3675576	-	NUP98	nucleoporin 98kDa [Source:HGNC Symbol;Acc:HGNC:8068]	ENSG00000110713	protein_coding	3'UTR	3'UTR	1	2088	11:3675516:3675576	ENSG00000110713	NUP98	8.415977612	0.004693997	-4.640075031	-1.800982651	-5.585832844	2.83909238	-0.945757813	-3.784850193	9.961963646	0.000931245	0.102003828	0.000235468	0.017638396	0.366646409	0.003597713	-0.785939936	-4.711381155	0.603306219	-3.926988881	-5.841764505	-2.24230272	13.20922629	0.000831733

PLEKHH2	0.079075233	0.073107204	0.149534609	-0.005968029	0.070459375	0.076427405	8	0.085008875	0.816496581	0.816496581	0.816496581	0.816496581	0.816496581	0.816496581	13	49	0	P_234684	P_234684	P_234684	chr2	43726391	43726451	+	PLEKHH2	"pleckstrin homology domain containing, family H (with MyTH4 domain) member 2 [Source:HGNC Symbol;Acc:HGNC:30506]"	ENSG00000152527	protein_coding	exon	coding exon	1	2089	2:43726391:43726451	ENSG00000152527	PLEKHH2	4.065610236	0.008009965	1.082108509	1.00043875	2.046312933	-0.081669759	0.964204424	1.045874183	9.961963646	0.304701788	0.340778725	0.068032878	0.936526898	0.357765318	0.320332529	-6.394510338	-5.680344236	-5.1409312	-7.145966719	-5.824648826	-6.504606982	1.398241011	0.300061554

ASPN	0.007038297	0.23095216	0.272236183	0.223913863	0.265197886	0.041284023	8	0.226642802	0.816496581	0.816496581	0.816496581	0.816496581	0.816496581	0.816496581	13	51	0	P_267950	P_267950	P_267950	chr9	92466425	92466485	-	ASPN	asporin [Source:HGNC Symbol;Acc:HGNC:14872]	ENSG00000106819	protein_coding	exon	coding exon	1	2091	9:92466425:92466485	ENSG00000106819	ASPN	3.745084748	0.043457153	0.041350674	1.356866189	1.5994138	1.315515516	1.558063126	0.242547611	9.961963646	0.967833356	0.204771381	0.140928486	0.217807709	0.150392067	0.813276477	-6.997427331	-5.29481949	-5.804815257	-6.277434646	-5.14566422	-7.039335042	1.436315226	0.289987524

SPATA3	0.155607469	0.157645741	0.024360043	0.002038272	-0.131247426	-0.133285698	8	0.215668932	0.816496581	0.816496581	0.816496581	0.816496581	0.816496581	0.816496581	13	52	0	P_130116	P_130116	P_130116	chr2	231002690	231002750	+	SPATA3	spermatogenesis associated 3 [Source:HGNC Symbol;Acc:HGNC:17884]	ENSG00000173699	protein_coding	3'UTR;exon	multiple Ref	1	2092	2:231002690:231002750	ENSG00000173699	SPATA3	4.774076115	0.03955923	0.958191144	0.970742304	0.150002937	0.012551159	-0.808188208	-0.820739367	9.961963646	0.360643077	0.354655501	0.883755851	0.990233687	0.437855768	0.43099627	-6.51928212	-5.70834684	-7.0348466	-7.149526915	-5.960551082	-6.715373286	0.534985098	0.668753809

KIF5B	0.472374093	-0.099033558	0.547223348	-0.571407652	0.074849255	0.646256906	8	0.06641507	0.816496581	0.816496581	0.816496581	0.816496581	0.816496581	0.816496581	13	53	0	P_208005	P_208005	P_208005	chr10	32048463	32048523	-	KIF5B	kinesin family member 5B [Source:HGNC Symbol;Acc:HGNC:6324]	ENSG00000170759	protein_coding	exon	coding exon	1	2093	10:32048463:32048523	ENSG00000170759	KIF5B	11.85747988	0.005748927	7.630242142	-1.599685592	8.839279524	-9.229927735	1.209037381	10.43896512	9.961963646	1.82E-05	0.140868069	4.99E-06	3.39E-06	0.254561121	1.10E-06	3.287989685	-4.987564281	4.589538027	4.943280676	-5.572926237	6.112280444	55.75629696	1.56E-06

CCDC66	0.010639234	-0.102137286	-0.045256965	-0.11277652	-0.055896198	0.056880322	8	0.075735566	0.816496581	0.816496581	0.816496581	0.816496581	0.816496581	0.816496581	13	55	0	P_244591	P_244591	P_244591	chr3	56566680	56566740	+	CCDC66	coiled-coil domain containing 66 [Source:HGNC Symbol;Acc:HGNC:27709]	ENSG00000180376	protein_coding	3'UTR;exon	multiple Ref	1	2095	3:56566680:56566740	ENSG00000180376	CCDC66	7.74714023	0.006812905	0.157866436	-1.515527335	-0.671529166	-1.673393771	-0.829395602	0.843998169	9.961963646	0.877716105	0.160706248	0.517152244	0.125310699	0.426307636	0.418476447	-6.984777801	-5.097572655	-6.806302817	-5.801196728	-5.943282821	-6.695605946	1.162195504	0.372026689

NPHP3	0.006732995	-0.069811153	-0.141895231	-0.076544148	-0.148628226	-0.072084078	8	0.068346216	0.816496581	0.816496581	0.816496581	0.816496581	0.816496581	0.816496581	13	56	0	P_246230	P_246230	P_246230	chr3	132683398	132683458	-	NPHP3	nephronophthisis 3 (adolescent) [Source:HGNC Symbol;Acc:HGNC:7907]	ENSG00000113971	protein_coding	3'UTR;exon	multiple Ref	1	2096	3:132683398:132683458	ENSG00000113971	NPHP3	6.84866237	0.005957916	0.106833317	-1.107702745	-2.251470297	-1.214536062	-2.358303614	-1.143767552	9.961963646	0.91704204	0.294018095	0.048156055	0.252547326	0.040159559	0.279459744	-6.992135732	-5.573620682	-4.8131872	-6.397673891	-3.966273036	-6.400190683	2.442072537	0.124771129

DRAIC	0.33400421	-0.149982669	0.212702537	-0.483986879	-0.121301673	0.362685206	8	0.085146109	0.816496581	0.816496581	0.816496581	0.816496581	0.816496581	0.816496581	13	57	0	P_224344	P_224344	P_224344	chr15	69561933	69561993	+	RP11-279F6.1	NA	ENSG00000245750	lincRNA	exon	non-coding exon	1	2097	15:69561933:69561993	ENSG00000245750	DRAIC	8.602233056	0.008028717	4.565354442	-2.050046144	2.907336029	-6.615400586	-1.658018413	4.957382173	9.961963646	0.001043785	0.067609625	0.015694888	6.07E-05	0.128422664	0.000578789	-0.903937498	-4.348815371	-3.714166152	1.942873881	-5.011099512	-0.351726959	17.43099019	0.000273321

MIATNB	-0.003241471	-0.170897821	-0.008162876	-0.16765635	-0.004921405	0.162734945	8	0.223435926	0.816496581	0.816496581	0.816496581	0.816496581	0.816496581	0.816496581	13	58	0	P_311859	P_311859	P_311859	chr22	26776162	26776222	-	MIATNB	MIAT neighbor (non-protein coding) [Source:HGNC Symbol;Acc:HGNC:50731]	ENSG00000244625	lincRNA	intron	intron antisense	0	2098	22:26776162:26776222	ENSG00000244625	MIATNB	4.418709923	0.042298067	-0.019303118	-1.017704962	-0.048610327	-0.998401844	-0.029307209	0.969094635	9.961963646	0.984980443	0.332897306	0.962190606	0.341717547	0.977198397	0.355437369	-6.998156991	-5.663749116	-7.045830318	-6.630227411	-6.296228433	-6.581245224	0.495878088	0.693269304

GFAP	-0.028297428	-0.311340113	0.079640696	-0.283042685	0.107938124	0.390980809	8	0.23720143	0.816496581	0.816496581	0.816496581	0.816496581	0.816496581	0.816496581	13	60	0	A_24_P59786	A_24_P59786	A_24_P59786	chr17	44909917	44909977	-	GFAP	glial fibrillary acidic protein [Source:HGNC Symbol;Acc:HGNC:4235]	ENSG00000131095	protein_coding	3'UTR	3'UTR	1	2100	17:44909917:44909977	ENSG00000131095	GFAP	4.11708502	0.04739016	-0.159201993	-1.75160679	0.448060426	-1.592404796	0.60726242	2.199667216	9.961963646	0.876691105	0.110509294	0.663696406	0.142494484	0.557263954	0.052569894	-6.984547306	-4.780689315	-6.938582708	-5.915104481	-6.104203641	-4.920307842	1.839547718	0.203973404

CCDC116	0.10338399	0.248827399	0.284155923	0.145443409	0.180771933	0.035328524	8	0.123605252	0.816496581	0.816496581	0.816496581	0.816496581	0.816496581	0.816496581	13	61	0	P_132732	P_132732	P_132732	chr22	21637262	21637322	+	CCDC116	coiled-coil domain containing 116 [Source:HGNC Symbol;Acc:HGNC:26688]	ENSG00000161180	protein_coding	3'UTR	3'UTR	1	2101	22:21637262:21637322	ENSG00000161180	CCDC116	4.301351748	0.014475958	1.052386466	2.532912372	2.892535372	1.480525906	1.840148907	0.359623	9.961963646	0.317479326	0.029802406	0.016097139	0.169651345	0.095689224	0.726635527	-6.425548202	-3.602995961	-3.73942809	-6.066850602	-4.754579175	-7.001183702	3.599559483	0.053940937

LINC00469	0.144956329	-0.067061579	-0.160537216	-0.212017908	-0.305493545	-0.093475637	8	0.496756214	0.816496581	0.816496581	0.816496581	0.816496581	0.816496581	0.816496581	13	62	0	A_24_P85348	A_24_P85348	A_24_P85348	chr17	73789952	73790012	+	LINC00469;AC125421.1	long intergenic non-protein coding RNA 469 [Source:HGNC Symbol;Acc:HGNC:26863];	ENSG00000177338	lincRNA	intron	multiple non-Ref	0	2102	17:73789952:73790012	ENSG00000177338	LINC00469	3.957255682	0.200373829	0.39660877	-0.1834843	-0.439238964	-0.58009307	-0.835847734	-0.255754665	9.961963646	0.700018929	0.858098798	0.669862739	0.574737029	0.422835302	0.803343594	-6.913199849	-6.164370579	-6.942773823	-6.968646327	-5.937952201	-7.035762199	0.247409288	0.861345802

SPIRE2	0.087256365	0.031109466	0.02993596	-0.056146899	-0.057320405	-0.001173507	8	0.085271768	0.816496581	0.816496581	0.816496581	0.816496581	0.816496581	0.816496581	13	63	0	P_119892	P_119892	P_119892	chr16	89870320	89870380	+	SPIRE2	spire-type actin nucleation factor 2 [Source:HGNC Symbol;Acc:HGNC:30623]	ENSG00000204991	protein_coding	3'UTR;exon	multiple Ref	1	2103	16:89870320:89870380	ENSG00000204991	SPIRE2	7.652775454	0.008045914	1.191393042	0.42476674	0.408743754	-0.766626301	-0.782649288	-0.016022987	9.961963646	0.261110267	0.680034839	0.691375945	0.461084536	0.452036136	0.987532414	-6.274650273	-6.087858213	-6.95664342	-6.837389615	-5.980827094	-7.071238271	0.494571358	0.694100262

KDR	0.049245813	0.023119881	0.19804061	-0.026125932	0.148794797	0.174920729	8	0.14669829	0.816496581	0.816496581	0.816496581	0.816496581	0.816496581	0.816496581	13	64	0	P_249014	P_249014	P_249014	chr4	55081955	55082015	-	KDR	kinase insert domain receptor (a type III receptor tyrosine kinase) [Source:HGNC Symbol;Acc:HGNC:6307]	ENSG00000128052	protein_coding	exon	coding exon	1	2104	4:55081955:55082015	ENSG00000128052	KDR	3.820924488	0.019488729	0.43203923	0.202833397	1.737433236	-0.229205834	1.305394006	1.534599839	9.961963646	0.674914633	0.843348035	0.113067755	0.82334608	0.221101786	0.15600729	-6.897452763	-6.160445381	-5.608693275	-7.120959203	-5.462308967	-5.917445014	1.226343171	0.350729371

STAT3	-0.047101258	-0.107012383	-0.144511653	-0.059911125	-0.097410395	-0.03749927	8	0.067918765	0.816496581	0.816496581	0.816496581	0.816496581	0.816496581	0.816496581	13	67	0	P_228767	P_228767	P_228767	chr17	42323003	42323063	-	STAT3	signal transducer and activator of transcription 3 (acute-phase response factor) [Source:HGNC Symbol;Acc:HGNC:11364]	ENSG00000168610	protein_coding	exon	coding exon	1	2107	17:42323003:42323063	ENSG00000168610	STAT3	7.452723761	0.005911141	-0.75031299	-1.704684435	-2.302039804	-0.954371445	-1.551726813	-0.597355369	9.961963646	0.470416287	0.119186325	0.044194657	0.362479762	0.151890683	0.563600612	-6.699587271	-4.84564879	-4.730793969	-6.673114553	-5.154030457	-6.879873397	2.073970043	0.167656646

PPWD1	-0.008353329	-0.087726569	-0.074386809	-0.07937324	-0.06603348	0.01333976	8	0.043486454	0.816496581	0.816496581	0.816496581	0.816496581	0.816496581	0.816496581	13	69	0	P_253082	P_253082	P_253082	chr5	65585029	65585089	+	PPWD1	peptidylprolyl isomerase domain and WD repeat containing 1 [Source:HGNC Symbol;Acc:HGNC:28954]	ENSG00000113593	protein_coding	3'UTR;exon	multiple Ref	1	2109	5:65585029:65585089	ENSG00000113593	PPWD1	8.463411769	0.003725318	-0.167619125	-1.760334171	-1.492656604	-1.592715046	-1.325037479	0.267677567	9.961963646	0.870236791	0.10896028	0.166503408	0.142424841	0.214746067	0.794407902	-6.983050212	-4.768508329	-5.949919699	-5.914674403	-5.439026717	-7.032376269	1.61983552	0.246519698

ZNF653	0.025376916	0.00680991	0.012403588	-0.018567006	-0.012973328	0.005593678	8	0.059783312	0.816496581	0.816496581	0.816496581	0.816496581	0.816496581	0.816496581	13	70	0	P_124696	P_124696	P_124696	chr19	11483442	11483502	-	ZNF653	zinc finger protein 653 [Source:HGNC Symbol;Acc:HGNC:25196]	ENSG00000161914	protein_coding	"3'UTR;3'UTR,exon"	multiple Ref	1	2110	19:11483442:11483502	ENSG00000161914	ZNF653	10.56128776	0.005076836	0.436202225	0.11705512	0.213204502	-0.319147105	-0.222997724	0.096149381	9.961963646	0.671991465	0.909142672	0.835467328	0.756206392	0.828043224	0.925308668	-6.895517288	-6.174869341	-7.022353412	-7.094196577	-6.270303592	-7.06633047	0.068376334	0.975494642

SAA4	-0.025993715	0.165687942	0.206239287	0.191681656	0.232233002	0.040551345	8	0.113691773	0.816496581	0.816496581	0.816496581	0.816496581	0.816496581	0.816496581	13	71	0	A_23_P87238	NM_006512	A_23_P87238	chr11	18232436	18232496	-	SAA4	"serum amyloid A4, constitutive [Source:HGNC Symbol;Acc:HGNC:10516]"	ENSG00000148965	protein_coding	exon	coding exon	1	2111	11:18232436:18232496	ENSG00000148965	SAA4	3.866348533	0.012586822	-0.283763113	1.80874979	2.251432805	2.092512903	2.535195918	0.442683015	9.961963646	0.782402008	0.100721873	0.048159116	0.062967762	0.02968614	0.667452199	-6.954598433	-4.700393218	-4.813248075	-5.167325975	-3.684394129	-6.965367127	3.237140637	0.06918563

ASS1	0.260013279	0.169840887	0.46190568	-0.090172392	0.201892401	0.292064793	8	0.051677128	0.816496581	0.816496581	0.816496581	0.816496581	0.816496581	0.816496581	13	72	0	A_23_P31921	A_23_P31921	A_23_P31921	chr9	130466732	130466792	+	ASS1	argininosuccinate synthase 1 [Source:HGNC Symbol;Acc:HGNC:758]	ENSG00000130707	protein_coding	exon	coding exon	1	2112	9:130466732:130466792	ENSG00000130707	ASS1	10.62479172	0.004351262	4.827625353	3.153408834	8.576129577	-1.674216519	3.748504224	5.422720743	9.961963646	0.000701818	0.010320078	6.53E-06	0.125146102	0.003818397	0.000295776	-0.49314055	-2.612159953	4.314424167	-5.800023134	-1.725691359	0.347315601	25.51002291	5.43E-05

EXPH5	0.154887888	-0.057062431	0.127520916	-0.211950319	-0.027366973	0.184583346	8	0.046968009	0.816496581	0.816496581	0.816496581	0.816496581	0.816496581	0.816496581	13	73	0	A_23_P403335	A_23_P403335	A_23_P403335	chr11	108509072	108509132	-	EXPH5	exophilin 5 [Source:HGNC Symbol;Acc:HGNC:30578]	ENSG00000110723	protein_coding	3'UTR	3'UTR	1	2113	11:108509072:108509132	ENSG00000110723	EXPH5	9.385406817	0.003978217	3.007591413	-1.108030321	2.476183357	-4.115621734	-0.531408056	3.584213677	9.961963646	0.013225513	0.293883259	0.032839836	0.002108537	0.606781081	0.005006862	-3.497843179	-5.573281769	-4.443197101	-1.761027996	-6.148642368	-2.580859247	7.745357629	0.005824652

ITGB3BP	-0.003662239	-0.025711027	-0.072829535	-0.022048788	-0.069167296	-0.047118508	8	0.054121484	0.816496581	0.816496581	0.816496581	0.816496581	0.816496581	0.816496581	13	74	0	A_23_P23765	NM_014288	A_23_P23765	chr1	63454422	63454898	-	ITGB3BP	integrin beta 3 binding protein (beta3-endonexin) [Source:HGNC Symbol;Acc:HGNC:6157]	ENSG00000142856	protein_coding	"exon,intron"	multiple non-Ref	0	2114	1:63454422:63454898	ENSG00000142856	ITGB3BP	10.63265124	0.004558939	-0.066429421	-0.46637274	-1.321056129	-0.399943319	-1.254626708	-0.854683389	9.961963646	0.948350219	0.650980013	0.216021825	0.697639391	0.238250658	0.412808475	-6.995952788	-6.068711113	-6.169465598	-7.062838246	-5.52134658	-6.686364414	0.738605371	0.552823598

JAM3	0.104804768	-0.044830107	-0.042973899	-0.149634876	-0.147778667	0.001856208	8	0.095621571	0.816496581	0.816496581	0.816496581	0.816496581	0.816496581	0.816496581	13	76	0	P_213905	P_213905	P_213905	chr11	134139856	134139916	+	JAM3	junctional adhesion molecule 3 [Source:HGNC Symbol;Acc:HGNC:15532]	ENSG00000166086	protein_coding	3'UTR;exon	multiple Ref	1	2116	11:134139856:134139916	ENSG00000166086	JAM3	5.712069187	0.009549401	1.313525809	-0.561859006	-0.538595012	-1.875384815	-1.852120821	0.023263994	9.961963646	0.218452029	0.586629915	0.601992847	0.090318873	0.093832271	0.981899086	-6.130640778	-6.018357341	-6.890981909	-5.504057068	-4.73725838	-7.071082866	1.546513522	0.262927596

POLR3GL	0.506157648	0.073767937	0.407239491	-0.432389711	-0.098918157	0.333471554	8	0.050241672	0.816496581	0.816496581	0.816496581	0.816496581	0.816496581	0.816496581	13	77	0	P_203796	P_203796	P_203796	chr1	145977092	145977152	+	POLR3GL	polymerase (RNA) III (DNA directed) polypeptide G (32kD)-like [Source:HGNC Symbol;Acc:HGNC:28466]	ENSG00000121851	protein_coding	exon	coding exon	1	2117	1:145977092:145977152	ENSG00000121851	POLR3GL	10.89791262	0.004233775	9.527249927	1.388511222	7.66534384	-8.138738705	-1.861906087	6.276832618	9.961963646	2.54E-06	0.195243018	1.75E-05	1.04E-05	0.092339315	9.33E-05	5.289001339	-5.256608062	3.303396361	3.786102406	-4.723062433	1.549206568	43.42639008	4.99E-06

BMP10	-0.021816149	0.168244953	0.143864961	0.190061102	0.16568111	-0.024379993	8	0.26175199	0.816496581	0.816496581	0.816496581	0.816496581	0.816496581	0.816496581	13	78	0	A_23_P131588	A_23_P131588	A_23_P131588	chr2	68865940	68866000	-	BMP10	bone morphogenetic protein 10 [Source:HGNC Symbol;Acc:HGNC:20869]	ENSG00000163217	protein_coding	exon	coding exon	1	2118	2:68865940:68866000	ENSG00000163217	BMP10	4.801966156	0.057227245	-0.11169206	0.861363082	0.736544921	0.973055142	0.848236982	-0.124818161	9.961963646	0.913285985	0.409292059	0.478385269	0.35356011	0.416221654	0.903150313	-6.991557038	-5.805414179	-6.758699813	-6.655110792	-5.927616518	-7.062874045	0.496473626	0.692890846

EMSY	0.024853711	0.045889116	0.03896129	0.021035405	0.014107579	-0.006927826	8	0.043055958	0.816496581	0.816496581	0.816496581	0.816496581	0.816496581	0.816496581	13	79	0	P_212505	P_212505	P_212505	chr11	76537890	76537950	+	C11orf30	chromosome 11 open reading frame 30 [Source:HGNC Symbol;Acc:HGNC:18071]	ENSG00000158636	protein_coding	exon	coding exon	1	2119	11:76537890:76537950	ENSG00000158636	EMSY	7.618800958	0.003695399	0.500732985	0.924537762	0.784961381	0.423804778	0.284228396	-0.139576381	9.961963646	0.627436344	0.377057982	0.450740099	0.680713397	0.782055605	0.891776216	-6.863244331	-5.750533108	-6.720697277	-7.052270343	-6.253895434	-7.060746144	0.333590967	0.801441758

TROAP	-0.214989498	-0.025345958	-0.200393327	0.18964354	0.014596171	-0.175047369	8	0.098936991	0.816496581	0.816496581	0.816496581	0.816496581	0.816496581	0.816496581	13	82	0	P_111468	P_111468	P_111468	chr12	49324164	49324224	+	TROAP	trophinin associated protein [Source:HGNC Symbol;Acc:HGNC:12327]	ENSG00000135451	protein_coding	3'UTR;exon	multiple Ref	1	2122	12:49324164:49324224	ENSG00000135451	TROAP	8.246994973	0.010067406	-2.624243239	-0.309382364	-2.446076847	2.314860875	0.178166392	-2.136694483	9.961963646	0.025486882	0.76340479	0.034573922	0.043241763	0.862163226	0.058461723	-4.149029104	-6.131878846	-4.493299007	-4.808271117	-6.279829175	-5.021319697	3.820241568	0.04661186

TSN	0.014994207	0.033487851	-0.018846616	0.018493644	-0.033840823	-0.052334467	8	0.04045999	0.816496581	0.816496581	0.816496581	0.816496581	0.816496581	0.816496581	13	83	0	P_128809	P_128809	P_128809	chr2	121767790	121767850	+	TSN	translin [Source:HGNC Symbol;Acc:HGNC:12379]	ENSG00000211460	protein_coding	3'UTR	3'UTR	1	2123	2:121767790:121767850	ENSG00000211460	TSN	9.017756863	0.003521293	0.309469645	0.691165159	-0.388980586	0.381695514	-0.698450231	-1.080145745	9.961963646	0.763340342	0.50524833	0.705475199	0.710702462	0.500874638	0.305533257	-6.946350233	-5.936348943	-6.965116735	-7.070519749	-6.043571063	-6.46888676	0.420837609	0.742141098

NCLN	-0.330700912	0.201625368	-0.016665444	0.53232628	0.314035468	-0.218290812	8	0.058726306	0.816496581	0.816496581	0.816496581	0.816496581	0.816496581	0.816496581	13	84	0	P_124135	P_124135	P_124135	chr19	3207804	3207864	+	NCLN	nicalin [Source:HGNC Symbol;Acc:HGNC:26923]	ENSG00000125912	protein_coding	3'UTR	3'UTR	1	2124	19:3207804:3207864	ENSG00000125912	NCLN	10.74716076	0.004976242	-5.741565097	3.500580522	-0.289342208	9.242145619	5.452222888	-3.789922731	9.961963646	0.00019006	0.005753877	0.778251678	3.35E-06	0.000283746	0.003567989	0.861594048	-2.061182297	-7.001593306	4.955551969	0.760852158	-2.233786251	29.13514284	3.03E-05

PALMD	0.01260064	0.003701537	-0.04829959	-0.008899103	-0.060900229	-0.052001127	8	0.077285245	0.816496581	0.816496581	0.816496581	0.816496581	0.816496581	0.816496581	13	85	0	P_102477	P_102477	P_102477	chr1	99694189	99694249	+	PALMD	palmdelphin [Source:HGNC Symbol;Acc:HGNC:15846]	ENSG00000099260	protein_coding	3'UTR;exon	multiple Ref	1	2125	1:99694189:99694249	ENSG00000099260	PALMD	6.314914261	0.007003336	0.18441054	0.054172047	-0.706865184	-0.130238493	-0.891275724	-0.761037231	9.961963646	0.857391322	0.957869083	0.495851593	0.898970067	0.393798634	0.464268137	-6.979834689	-6.180540618	-6.780923693	-7.140345013	-5.890701231	-6.763890436	0.321185399	0.810026604

MSH6	-0.323146929	0.019867398	-0.174746253	0.343014327	0.148400676	-0.194613651	8	0.070080355	0.816496581	0.816496581	0.816496581	0.816496581	0.816496581	0.816496581	13	90	0	P_127784	P_127784	P_127784	chr2	47760350	47760410	+	MSH6	mutS homolog 6 [Source:HGNC Symbol;Acc:HGNC:7329]	ENSG00000116062	protein_coding	intron	intron sense	0	2130	2:47760350:47760410	ENSG00000116062	MSH6	10.84657206	0.00615069	-5.046423888	0.310259219	-2.72892479	5.356683106	2.317499098	-3.039184009	9.961963646	0.000507795	0.762757408	0.021301394	0.000324729	0.04304818	0.012532191	-0.157812722	-6.131595262	-4.017871561	0.187636064	-4.030625258	-3.511297095	12.71853013	0.000963449

ABHD12	-0.336173558	0.099057291	-0.160945761	0.435230849	0.175227797	-0.260003052	8	0.05316208	0.816496581	0.816496581	0.816496581	0.816496581	0.816496581	0.816496581	13	92	0	A_23_P149494	A_23_P149494	A_23_P149494	chr20	25307969	25308029	-	ABHD12	abhydrolase domain containing 12 [Source:HGNC Symbol;Acc:HGNC:15868]	ENSG00000100997	protein_coding	exon	coding exon	1	2132	20:25307969:25308029	ENSG00000100997	ABHD12	10.33456177	0.004476282	-6.153900299	1.813315403	-2.946228644	7.967215701	3.207671654	-4.759544047	9.961963646	0.000109527	0.099975256	0.014685704	1.25E-05	0.009413365	0.000777254	1.433144089	-4.693923823	-3.647743004	3.5925433	-2.593451764	-0.65826996	24.37630131	6.61E-05

MIR155HG	-0.237199019	-0.069128859	-0.054459646	0.16807016	0.182739373	0.014669213	8	0.146716324	0.816496581	0.816496581	0.816496581	0.816496581	0.816496581	0.816496581	13	93	0	P_131960	P_131960	P_131960	chr21	25574926	25574986	-	MIR155HG	MIR155 host gene (non-protein coding) [Source:HGNC Symbol;Acc:HGNC:35460]	ENSG00000234883	lincRNA	exon	exon antisense	0	2133	21:25574926:25574986	ENSG00000234883	MIR155HG	4.173188421	0.019492978	-2.08074767	-0.606409391	-0.47772871	1.474338279	1.60301896	0.128680681	9.961963646	0.064222463	0.557807979	0.643152497	0.171276808	0.140128961	0.900171151	-5.039777021	-5.991867631	-6.923901419	-6.075041356	-5.085734303	-7.062340003	1.6142283	0.247732221

SLAMF1	-0.140033479	-0.072141674	0.040080975	0.067891805	0.180114453	0.112222648	8	0.191748003	0.816496581	0.816496581	0.816496581	0.816496581	0.816496581	0.816496581	13	94	0	P_103761	P_103761	P_103761	chr1	160624095	160624155	-	SLAMF1	signaling lymphocytic activation molecule family member 1 [Source:HGNC Symbol;Acc:HGNC:10903]	ENSG00000117090	protein_coding	exon	coding exon	1	2134	1:160624095:160624155	ENSG00000117090	SLAMF1	3.631469587	0.031732828	-0.962771262	-0.495995178	0.275568464	0.466776084	1.238339726	0.771563642	9.961963646	0.358449663	0.630657547	0.788511048	0.650701223	0.243977354	0.458283931	-6.514891242	-6.054038617	-7.005808524	-7.031753885	-5.539932393	-6.755571118	0.59925889	0.630002489

CNTN2	0.799016411	-0.039253459	0.480193932	-0.83826987	-0.318822479	0.519447391	8	0.201356609	0.816496581	0.816496581	0.816496581	0.816496581	0.816496581	0.816496581	13	97	0	A_24_P114142	A_24_P114142	A_24_P114142	chr1	205077914	205077974	+	CNTN2	contactin 2 (axonal) [Source:HGNC Symbol;Acc:HGNC:2172]	ENSG00000184144	protein_coding	3'UTR	3'UTR	1	2137	1:205077914:205077974	ENSG00000184144	CNTN2	5.017128014	0.034766115	5.248355521	-0.257837144	3.154163594	-5.506192665	-2.094191927	3.412000738	9.961963646	0.000378997	0.80178068	0.010306871	0.000263076	0.062790619	0.006672054	0.145561758	-6.147162346	-3.292078331	0.407905097	-4.37686906	-2.873670597	13.98433503	0.000664976

DLGAP5	-0.290529414	-0.061317299	-0.210763874	0.229212114	0.07976554	-0.149446574	8	0.068544309	0.816496581	0.816496581	0.816496581	0.816496581	0.816496581	0.816496581	13	98	0	P_220896	P_220896	P_220896	chr14	55179630	55179690	-	DLGAP5	"discs, large (Drosophila) homolog-associated protein 5 [Source:HGNC Symbol;Acc:HGNC:16864]"	ENSG00000126787	protein_coding	exon	coding exon	1	2138	14:55179630:55179690	ENSG00000126787	DLGAP5	9.777112779	0.005979693	-4.601466811	-0.971156462	-3.338123178	3.63031035	1.263343633	-2.366966717	9.961963646	0.000987693	0.354459169	0.007553187	0.004638893	0.235230995	0.039571106	-0.846815075	-5.707961042	-2.977627394	-2.57367813	-5.511327795	-4.647401192	8.939571642	0.003552662

GLRA2	0.197024424	0.304818684	0.123972418	0.10779426	-0.073052006	-0.180846266	8	0.214600221	0.816496581	0.816496581	0.816496581	0.816496581	0.816496581	0.816496581	13	100	0	P_315132	P_315132	P_315132	chrX	14731749	14731809	+	GLRA2	"glycine receptor, alpha 2 [Source:HGNC Symbol;Acc:HGNC:4327]"	ENSG00000101958	protein_coding	3'UTR	3'UTR	1	2140	X:14731749:14731809	ENSG00000101958	GLRA2	3.869299212	0.039189959	1.218928671	1.885818145	0.76697869	0.666889474	-0.451949981	-1.118839455	9.961963646	0.250947845	0.088783195	0.460884285	0.519989343	0.66098586	0.289460887	-6.243079364	-4.590176316	-6.735062915	-6.911703298	-6.18917808	-6.427470516	1.255192596	0.34159504

TATDN3	-0.143191805	0.088133267	-0.091154747	0.231325072	0.052037058	-0.179288014	8	0.054247527	0.816496581	0.816496581	0.816496581	0.816496581	0.816496581	0.816496581	13	101	0	P_206091	P_206091	P_206091	chr1	212804369	212804429	+	TATDN3	TatD DNase domain containing 3 [Source:HGNC Symbol;Acc:HGNC:27010]	ENSG00000203705	protein_coding	3'UTR;exon	multiple Ref	1	2141	1:212804369:212804429	ENSG00000203705	TATDN3	8.900602091	0.004569908	-2.594239443	1.596731031	-1.651471886	4.190970474	0.942767557	-3.248202917	9.961963646	0.026831297	0.141526075	0.129768607	0.0018706	0.368100923	0.008789709	-4.199501581	-4.991484818	-5.73191394	-1.637021727	-5.844513223	-3.153469709	6.835142339	0.008796718

BRK1	0.159813551	-0.008715157	0.020978223	-0.168528708	-0.138835327	0.029693381	8	0.094930668	0.816496581	0.816496581	0.816496581	0.816496581	0.816496581	0.816496581	13	102	0	P_243206	P_243206	P_243206	chr3	10126468	10126528	-	BRK1	"BRICK1, SCAR/WAVE actin-nucleating complex subunit [Source:HGNC Symbol;Acc:HGNC:23057]"	ENSG00000254999	protein_coding	3'UTR	3'UTR antisense	0	2142	3:10126468:10126528	ENSG00000254999	BRK1	9.66685509	0.009443676	2.014135507	-0.109837418	0.264389249	-2.123972925	-1.749746258	0.374226667	9.961963646	0.071786527	0.914719458	0.7968693	0.059726556	0.110842103	0.716077977	-5.144176087	-6.175731498	-7.009080879	-5.117299571	-4.883609303	-6.995407676	1.975271055	0.181945668

PSMB1	-0.082111624	-0.157066765	-0.082412974	-0.074955141	-0.00030135	0.07465379	8	0.068618371	0.816496581	0.816496581	0.816496581	0.816496581	0.816496581	0.816496581	13	103	0	P_260310	P_260310	P_260310	chr6	170546102	170546162	-	PSMB1	"proteasome (prosome, macropain) subunit, beta type, 1 [Source:HGNC Symbol;Acc:HGNC:9537]"	ENSG00000008018	protein_coding	exon	coding exon	1	2143	6:170546102:170546162	ENSG00000008018	PSMB1	10.61762722	0.005987851	-1.299615214	-2.485961756	-1.304384813	-1.186346542	-0.004769599	1.181576943	9.961963646	0.223000948	0.032295285	0.221432482	0.263007983	0.996288586	0.264811671	-6.147551211	-3.677144076	-6.189817377	-6.429979591	-6.296673157	-6.357940292	2.062331707	0.169271714

SLC39A13	0.299795856	0.183499655	0.289690296	-0.116296201	-0.01010556	0.106190641	8	0.074489793	0.816496581	0.816496581	0.816496581	0.816496581	0.816496581	0.816496581	13	104	0	P_108464	P_108464	P_108464	chr11	47415162	47415222	+	SLC39A13	"solute carrier family 39 (zinc transporter), member 13 [Source:HGNC Symbol;Acc:HGNC:20859]"	ENSG00000165915	protein_coding	3'UTR;exon	multiple Ref	1	2144	11:47415162:47415222	ENSG00000165915	SLC39A13	9.080402994	0.006662616	4.49830458	2.753331379	4.346675109	-1.744973201	-0.151629471	1.59334373	9.961963646	0.001157081	0.020428945	0.001464134	0.111700083	0.882505898	0.14228381	-1.010459868	-3.252352956	-1.294503992	-5.697918716	-6.284470946	-5.836995298	8.719304916	0.003877976

TMTC3	0.077450722	0.080405142	0.136985217	0.00295442	0.059534495	0.056580075	8	0.099262282	0.816496581	0.816496581	0.816496581	0.816496581	0.816496581	0.816496581	13	105	0	P_216585	P_216585	P_216585	chr12	88190505	88190565	+	TMTC3	transmembrane and tetratricopeptide repeat containing 3 [Source:HGNC Symbol;Acc:HGNC:26899]	ENSG00000139324	protein_coding	exon	coding exon	1	2145	12:88190505:88190565	ENSG00000139324	TMTC3	7.592878948	0.010119181	0.942971193	0.978941589	1.667810325	0.035970397	0.724839132	0.688868736	9.961963646	0.36800174	0.350783371	0.12643286	0.972016533	0.485227302	0.50663183	-6.533748128	-5.700683974	-5.708759971	-7.148905536	-6.024592765	-6.818163087	0.938389726	0.458141034

GALC	0.087505777	0.008211377	-0.013120283	-0.0792944	-0.10062606	-0.02133166	8	0.168449206	0.816496581	0.816496581	0.816496581	0.816496581	0.816496581	0.816496581	13	106	0	P_221769	P_221769	P_221769	chr14	87965559	87965619	-	GALC	galactosylceramidase [Source:HGNC Symbol;Acc:HGNC:4115]	ENSG00000054983	protein_coding	3'UTR;exon	multiple Ref	1	2146	14:87965559:87965619	ENSG00000054983	GALC	3.536069885	0.024993464	0.677905447	0.063613369	-0.101642561	-0.614292078	-0.779548007	-0.16525593	9.961963646	0.513268411	0.950536419	0.921057124	0.552792144	0.453778369	0.872047918	-6.753242497	-6.179954591	-7.041481568	-6.94708589	-5.983250334	-7.056479795	0.247715507	0.861133113

HOMER1	-0.026191369	-0.003720037	0.008093799	0.022471333	0.034285168	0.011813836	8	0.054994173	0.816496581	0.816496581	0.816496581	0.816496581	0.816496581	0.816496581	13	107	0	P_139826	P_139826	P_139826	chr5	79374052	79374112	-	HOMER1	homer homolog 1 (Drosophila) [Source:HGNC Symbol;Acc:HGNC:17512]	ENSG00000152413	protein_coding	3'UTR	3'UTR	1	2147	5:79374052:79374112	ENSG00000152413	HOMER1	9.864737498	0.004635409	-0.471150651	-0.066918904	0.14559753	0.404231747	0.61674818	0.212516433	9.961963646	0.647681163	0.947970262	0.887142975	0.694584217	0.55123454	0.835989591	-6.87856673	-6.179727228	-7.035556199	-7.060982598	-6.098259304	-7.046761846	0.139434096	0.934096745

AMPD3	0.226712648	0.022597106	0.14537051	-0.204115542	-0.081342138	0.122773404	8	0.189696779	0.816496581	0.816496581	0.816496581	0.816496581	0.816496581	0.816496581	13	108	0	A_23_P116286	A_23_P116286	A_23_P116286	chr11	10507033	10507093	+	AMPD3	adenosine monophosphate deaminase 3 [Source:HGNC Symbol;Acc:HGNC:470]	ENSG00000133805	protein_coding	3'UTR	3'UTR	1	2148	11:10507033:10507093	ENSG00000133805	AMPD3	3.812702512	0.031104495	1.574380863	0.156923097	1.009509397	-1.417457767	-0.564871466	0.8525863	9.961963646	0.146592523	0.878440233	0.336621202	0.186858655	0.584656006	0.413916712	-5.791657241	-6.169122585	-6.517435881	-6.149292707	-6.129720745	-6.688186082	1.096263146	0.395411041

STX16	0.059320799	-0.006370634	-0.027487248	-0.065691433	-0.086808047	-0.021116615	8	0.049984785	0.816496581	0.816496581	0.816496581	0.816496581	0.816496581	0.816496581	13	109	0	P_131666	P_131666	P_131666	chr20	58676231	58676291	+	STX16	syntaxin 16 [Source:HGNC Symbol;Acc:HGNC:11431]	ENSG00000124222	protein_coding	3'UTR;exon	multiple Ref	1	2149	20:58676231:58676291	ENSG00000124222	STX16	8.470148728	0.004213099	1.119313676	-0.120206366	-0.518652035	-1.239520042	-1.637965711	-0.398445669	9.961963646	0.289268059	0.906709454	0.615327823	0.243558617	0.132585436	0.698707692	-6.354702766	-6.17447577	-6.902179837	-6.368573421	-5.038474434	-6.985336624	0.985735527	0.438277816

MTFR2	-0.455399325	0.102266559	-0.286571966	0.557665884	0.168827359	-0.388838525	8	0.078495466	0.816496581	0.816496581	0.816496581	0.816496581	0.816496581	0.816496581	13	110	0	P_259378	P_259378	P_259378	chr6	136249036	136249096	-	MTFR2	mitochondrial fission regulator 2 [Source:HGNC Symbol;Acc:HGNC:21115]	ENSG00000146410	protein_coding	exon	coding exon	1	2150	6:136249036:136249096	ENSG00000146410	MTFR2	8.261040708	0.007154735	-6.593882923	1.480752559	-4.14937372	8.074635483	2.444509203	-5.63012628	9.961963646	6.24E-05	0.169592059	0.001998239	1.11E-05	0.034666646	0.000221472	2.016451097	-5.141981912	-1.615779518	3.714153761	-3.829440326	0.648808029	27.62715129	3.83E-05

ATG5	-0.089257914	0.04336342	-0.055289699	0.132621335	0.033968215	-0.098653119	8	0.072283138	0.816496581	0.816496581	0.816496581	0.816496581	0.816496581	0.816496581	13	111	0	A_23_P111381	A_23_P111381	A_23_P111381	chr6	106185990	106186050	-	ATG5	autophagy related 5 [Source:HGNC Symbol;Acc:HGNC:589]	ENSG00000057663	protein_coding	3'UTR;exon	multiple Ref	1	2151	6:106185990:106186050	ENSG00000057663	ATG5	7.862593922	0.006402525	-1.366207679	0.663733161	-0.846280264	2.02994084	0.519927415	-1.510013425	9.961963646	0.201918738	0.521924675	0.417261478	0.069918909	0.614470546	0.162087557	-6.065465741	-5.955043359	-6.669521037	-5.265957035	-6.154883194	-5.950566705	1.615730048	0.247406809

ANKRD31	0.326300603	-0.008455431	0.136971999	-0.334756034	-0.189328604	0.14542743	8	0.104655674	0.816496581	0.816496581	0.816496581	0.816496581	0.816496581	0.816496581	13	112	0	P_253411	P_253411	P_253411	chr5	75137862	75137922	-	ANKRD31	ankyrin repeat domain 31 [Source:HGNC Symbol;Acc:HGNC:26853]	ENSG00000145700	protein_coding	exon	coding exon	1	2152	5:75137862:75137922	ENSG00000145700	ANKRD31	5.177977984	0.011002388	3.809956661	-0.098727444	1.599314791	-3.908684105	-2.21064187	1.698042235	9.961963646	0.003453081	0.923313014	0.1409505	0.002940726	0.051603358	0.120463301	-2.13593022	-6.176951642	-5.804952613	-2.104790348	-4.197672008	-5.689284861	6.688570168	0.009431195

MAPK8	0.066222187	-0.115158026	-0.079873573	-0.181380213	-0.14609576	0.035284453	8	0.046489426	0.816496581	0.816496581	0.816496581	0.816496581	0.816496581	0.816496581	13	115	0	P_208182	P_208182	P_208182	chr10	48427083	48427143	+	MAPK8	mitogen-activated protein kinase 8 [Source:HGNC Symbol;Acc:HGNC:6881]	ENSG00000107643	protein_coding	exon	coding exon	1	2155	10:48427083:48427143	ENSG00000107643	MAPK8	6.410009459	0.003942299	1.291737793	-2.246286064	-1.558023359	-3.538023857	-2.849761152	0.688262705	9.961963646	0.225611512	0.048581107	0.150401432	0.005406079	0.01731924	0.506997322	-6.157070932	-4.050953536	-5.861795457	-2.73046525	-3.175574945	-6.818598163	5.04238023	0.022189935

C1orf74	0.038572827	0.049040272	0.02981106	0.010467445	-0.008761767	-0.019229212	8	0.107157805	0.816496581	0.816496581	0.816496581	0.816496581	0.816496581	0.816496581	13	116	0	A_32_P141135	A_32_P141135	A_32_P141135	chr1	209779558	209779618	-	C1orf74	chromosome 1 open reading frame 74 [Source:HGNC Symbol;Acc:HGNC:26319]	ENSG00000162757	protein_coding	3'UTR	3'UTR	1	2156	1:209779558:209779618	ENSG00000162757	C1orf74	8.047087777	0.011427995	0.441918401	0.561841075	0.341537221	0.119922674	-0.10038118	-0.220303854	9.961963646	0.667987022	0.586641675	0.739793924	0.906928464	0.922033166	0.830083672	-6.892830615	-6.018367623	-6.983790929	-7.141754037	-6.291328563	-7.044929181	0.116766093	0.94821119

USP8	0.025913644	0.007101225	0.011534556	-0.018812419	-0.014379088	0.004433331	8	0.057139347	0.816496581	0.816496581	0.816496581	0.816496581	0.816496581	0.816496581	13	118	0	P_223440	P_223440	P_223440	chr15	50494213	50494273	+	USP8	ubiquitin specific peptidase 8 [Source:HGNC Symbol;Acc:HGNC:12631]	ENSG00000138592	protein_coding	exon	coding exon	1	2158	15:50494213:50494273	ENSG00000138592	USP8	8.248728885	0.004828581	0.456735082	0.125161035	0.20329971	-0.331574047	-0.253435372	0.078138675	9.961963646	0.657658272	0.902885789	0.842993299	0.747080825	0.805085308	0.939264952	-6.885710237	-6.173835888	-7.024596485	-7.089821151	-6.262635759	-7.068044015	0.074313247	0.972421251

ANXA4	-0.205391199	0.162929221	-0.143325053	0.36832042	0.062066146	-0.306254275	8	0.087689475	0.816496581	0.816496581	0.816496581	0.816496581	0.816496581	0.816496581	13	119	0	P_128040	P_128040	P_128040	chr2	69825455	69825515	+	ANXA4	annexin A4 [Source:HGNC Symbol;Acc:HGNC:542]	ENSG00000196975	protein_coding	exon	coding exon	1	2159	2:69825455:69825515	ENSG00000196975	ANXA4	9.051900279	0.008381727	-2.747645558	2.179605327	-1.917348204	4.927250886	0.830297354	-4.096953531	9.961963646	0.020628933	0.054382016	0.084288699	0.000605151	0.425821188	0.002172278	-3.940480416	-4.153152154	-5.340527531	-0.462838857	-5.942539961	-1.722964095	9.621447143	0.002733964

NOL9	-0.198733809	-0.128374033	-0.24105392	0.070359776	-0.042320112	-0.112679888	8	0.05814628	0.816496581	0.816496581	0.816496581	0.816496581	0.816496581	0.816496581	13	120	0	P_200116	P_200116	P_200116	chr1	6532015	6532075	-	NOL9	nucleolar protein 9 [Source:HGNC Symbol;Acc:HGNC:26265]	ENSG00000162408	protein_coding	exon	coding exon	1	2160	1:6532015:6532075	ENSG00000162408	NOL9	7.993908534	0.004921803	-3.469407091	-2.241097187	-4.208212915	1.228309904	-0.738805824	-1.967115728	9.961963646	0.006061097	0.049010175	0.001820237	0.247559218	0.477070823	0.077624884	-2.710148005	-4.058938965	-1.519479647	-6.381684491	-6.014292977	-5.287679464	6.782080276	0.00902033

EFS	0.017964721	-0.134447664	-0.175037761	-0.152412384	-0.193002482	-0.040590097	8	0.057152339	0.816496581	0.816496581	0.816496581	0.816496581	0.816496581	0.816496581	13	122	0	P_309802	P_309802	P_309802	chr14	23365719	23365779	-	EFS	embryonal Fyn-associated substrate [Source:HGNC Symbol;Acc:HGNC:16898]	ENSG00000100842	protein_coding	"5'UTR,promoter;promoter"	multiple non-Ref	0	2162	14:23365719:23365779	ENSG00000100842	EFS	8.860426829	0.004829773	0.316594034	-2.369384385	-3.084707658	-2.68597842	-3.401301692	-0.715323273	9.961963646	0.758086142	0.039408394	0.011597677	0.022928248	0.006792784	0.49083425	-6.943940175	-3.860072521	-3.410925274	-4.187698958	-2.280203176	-6.798833307	5.754109356	0.015059312

RAB38	-0.02655667	-0.259886495	-0.039372176	-0.233329825	-0.012815506	0.220514319	8	0.330541726	0.816496581	0.816496581	0.816496581	0.816496581	0.816496581	0.816496581	13	123	0	P_109774	P_109774	P_109774	chr11	88113314	88113374	-	RAB38	"RAB38, member RAS oncogene family [Source:HGNC Symbol;Acc:HGNC:9776]"	ENSG00000123892	protein_coding	3'UTR	3'UTR	1	2163	11:88113314:88113374	ENSG00000123892	RAB38	4.006069406	0.089946681	-0.108449282	-1.061296592	-0.160783869	-0.952847311	-0.052334588	0.900512723	9.961963646	0.915792575	0.313606822	0.875477372	0.363214522	0.95929666	0.389098165	-6.991946113	-5.620844592	-7.033021282	-6.67457044	-6.295228675	-6.64559775	0.480923871	0.702823116

ORC5	-0.686009182	0.033998021	-0.642800605	0.720007202	0.043208576	-0.676798626	8	0.08117221	0.816496581	0.816496581	0.816496581	0.816496581	0.816496581	0.816496581	13	125	0	P_262238	P_262238	P_262238	chr7	104166771	104166831	-	ORC5	"origin recognition complex, subunit 5 [Source:HGNC Symbol;Acc:HGNC:8491]"	ENSG00000164815	protein_coding	3'UTR;exon	multiple Ref	1	2165	7:104166771:104166831	ENSG00000164815	ORC5	8.902364937	0.007497952	-9.702959635	0.480870277	-9.09181465	10.18382991	0.611144984	-9.572684927	9.961963646	2.16E-06	0.640995068	3.88E-06	1.39E-06	0.554791602	2.44E-06	5.455712178	-6.061637753	4.846833738	5.859727498	-6.101780901	5.311712027	62.12665701	9.41E-07

RHOXF1	0.666456493	0.324917204	0.561825679	-0.341539289	-0.104630814	0.236908475	8	0.187051407	0.816496581	0.816496581	0.816496581	0.816496581	0.816496581	0.816496581	13	126	0	P_151586	P_151586	P_151586	chrX	120109118	120109178	-	RHOXF1	"Rhox homeobox family, member 1 [Source:HGNC Symbol;Acc:HGNC:29993]"	ENSG00000101883	protein_coding	3'UTR	3'UTR	1	2166	X:120109118:120109178	ENSG00000101883	RHOXF1	4.194531904	0.03030414	4.688851242	2.285953322	3.952721684	-2.40289792	-0.736129557	1.666768362	9.961963646	0.000864783	0.045419172	0.002738486	0.037219247	0.478627003	0.126643271	-0.70932338	-3.989736787	-1.940463275	-4.663037082	-6.016280208	-5.733962534	8.654806531	0.003979915

KLK6	-0.189461162	0.04426218	0.185686704	0.233723342	0.375147866	0.141424524	8	0.274564347	0.816496581	0.816496581	0.816496581	0.816496581	0.816496581	0.816496581	13	127	0	A_24_P236935	A_24_P236935	A_24_P236935	chr19	50963468	50963528	-	KLK6	kallikrein-related peptidase 6 [Source:HGNC Symbol;Acc:HGNC:6367]	ENSG00000167755	protein_coding	5'UTR;exon	multiple Ref	1	2167	19:50963468:50963528	ENSG00000167755	KLK6	5.298819886	0.062745416	-0.926349402	0.216415035	0.907894606	1.142764437	1.834244008	0.691479572	9.961963646	0.376160944	0.833031559	0.385370197	0.279856892	0.096617581	0.505059089	-6.549325329	-6.157457372	-6.614763219	-6.478797213	-4.76310286	-6.816284763	1.146289489	0.37752586

COMMD10	0.026293067	-0.077844908	-0.012504973	-0.104137975	-0.03879804	0.065339935	8	0.075640634	0.816496581	0.816496581	0.816496581	0.816496581	0.816496581	0.816496581	13	128	0	A_23_P252403	A_23_P252403	A_23_P252403	chr5	116292834	116292894	+	COMMD10	COMM domain containing 10 [Source:HGNC Symbol;Acc:HGNC:30201]	ENSG00000145781	protein_coding	3'UTR	3'UTR	1	2168	5:116292834:116292894	ENSG00000145781	COMMD10	8.291748356	0.006801365	0.390471052	-1.156053145	-0.185707888	-1.546524197	-0.57617894	0.970345257	9.961963646	0.704407712	0.27463158	0.856400612	0.153130968	0.577278819	0.354843797	-6.915795331	-5.522770837	-7.028319437	-5.978152597	-6.123082504	-6.580035259	0.864783853	0.490924021

PPP3CA	-0.001816248	-0.163410749	-0.009597438	-0.161594501	-0.00778119	0.153813311	8	0.066373497	0.816496581	0.816496581	0.816496581	0.816496581	0.816496581	0.816496581	13	129	0	A_23_P92623	A_23_P92623	A_23_P92623	chr4	101025521	101025581	-	PPP3CA	"protein phosphatase 3, catalytic subunit, alpha isozyme [Source:HGNC Symbol;Acc:HGNC:9314]"	ENSG00000138814	protein_coding	3'UTR	3'UTR	1	2169	4:101025521:101025581	ENSG00000138814	PPP3CA	9.218591293	0.005744493	-0.029349111	-2.640586399	-0.155086885	-2.611237287	-0.125737773	2.485499514	9.961963646	0.977165806	0.024783029	0.879850086	0.026061217	0.90244087	0.032320825	-6.997890295	-3.432143957	-7.034001539	-4.31420472	-6.288283023	-4.450734286	3.334947972	0.064616475

APLP2	-0.045027539	-0.063740003	-0.148452962	-0.018712465	-0.103425424	-0.084712959	8	0.058199664	0.816496581	0.816496581	0.816496581	0.816496581	0.816496581	0.816496581	13	133	0	P_213881	P_213881	P_213881	chr11	130121749	130121809	-	APLP2;AP003041.1	amyloid beta (A4) precursor-like protein 2 [Source:HGNC Symbol;Acc:HGNC:598];	ENSG00000084234	protein_coding;miRNA	"3'ext;3'UTR;exon;exon,promoter;intron"	multiple non-Ref	0	2173	11:130121749:130121809	ENSG00000084234	APLP2	9.941778015	0.004926791	-0.785672891	-1.112181438	-2.590314102	-0.326508547	-1.80464121	-1.478132664	9.961963646	0.450341754	0.292178739	0.027012326	0.750795755	0.101398132	0.170278449	-6.671620444	-5.568980293	-4.252067351	-7.091624421	-4.805637025	-5.993015125	2.352028415	0.133937371

DMTN	0.202368448	-0.117483378	0.11308567	-0.319851827	-0.089282779	0.230569048	8	0.079105133	0.816496581	0.816496581	0.816496581	0.816496581	0.816496581	0.816496581	13	134	0	P_263994	P_263994	P_263994	chr8	22073734	22073794	+	DMTN	dematin actin binding protein [Source:HGNC Symbol;Acc:HGNC:3382]	ENSG00000158856	protein_coding	exon	coding exon	1	2174	8:22073734:22073794	ENSG00000158856	DMTN	7.570602359	0.007231896	2.914488547	-1.691982934	1.628647608	-4.60647148	-1.285840939	3.320630542	9.961963646	0.015504159	0.121638983	0.134560554	0.000980175	0.227582177	0.007778866	-3.656721773	-4.863074748	-5.76404233	-0.965618534	-5.485242975	-3.029633379	7.984849397	0.00525399

MRPL10	0.053564438	0.030504107	0.077851908	-0.023060331	0.02428747	0.0473478	8	0.070960954	0.816496581	0.816496581	0.816496581	0.816496581	0.816496581	0.816496581	13	135	0	A_23_P218423	A_23_P218423	A_23_P218423	chr17	47823708	47823768	-	MRPL10	mitochondrial ribosomal protein L10 [Source:HGNC Symbol;Acc:HGNC:14055]	ENSG00000159111	protein_coding	3'UTR	3'UTR	1	2175	17:47823708:47823768	ENSG00000159111	MRPL10	10.93116937	0.006250431	0.829788127	0.472551326	1.206035031	-0.357236801	0.376246904	0.733483704	9.961963646	0.426095843	0.646715595	0.255666116	0.728366398	0.71462234	0.480168646	-6.635142384	-6.065721837	-6.306082244	-7.080268898	-6.221938052	-6.785183988	0.528925299	0.672507198

MFSD14A	-0.232110546	0.027838767	-0.115112075	0.259949313	0.116998471	-0.142950842	8	0.070631129	0.816496581	0.816496581	0.816496581	0.816496581	0.816496581	0.816496581	13	136	0	P_203100	P_203100	P_203100	chr1	100068091	100068151	+	HIAT1	hippocampus abundant transcript 1 [Source:HGNC Symbol;Acc:HGNC:23363]	ENSG00000156875	protein_coding	exon	coding exon	1	2176	1:100068091:100068151	ENSG00000156875	MFSD14A	7.986883682	0.006212927	-3.606553367	0.432561127	-1.788621196	4.039114495	1.817932172	-2.221182323	9.961963646	0.004824887	0.674547856	0.104075002	0.002382951	0.099225456	0.050691217	-2.477865226	-6.084401944	-5.533741825	-1.887591895	-4.786581239	-4.88556562	6.824412716	0.008841394

SNX30	0.030838473	-0.08566095	-0.059497345	-0.116499424	-0.090335818	0.026163606	8	0.069701036	0.816496581	0.816496581	0.816496581	0.816496581	0.816496581	0.816496581	13	137	0	P_149419	P_149419	P_149419	chr9	112868988	112869048	+	SNX30	sorting nexin family member 30 [Source:HGNC Symbol;Acc:HGNC:23685]	ENSG00000148158	protein_coding	3'UTR	3'UTR	1	2177	9:112868988:112869048	ENSG00000148158	SNX30	6.378931481	0.006108111	0.483264658	-1.342378702	-0.932373128	-1.82564336	-1.415637787	0.410005573	9.961963646	0.639353109	0.209262377	0.373189238	0.097984637	0.187376627	0.690479867	-6.872399725	-5.312115615	-6.592104126	-5.578820644	-5.328611067	-6.98031407	1.401658925	0.299141216

LAMP5	0.154545385	-0.071417085	0.031641761	-0.22596247	-0.122903624	0.103058846	8	0.244033164	0.816496581	0.816496581	0.816496581	0.816496581	0.816496581	0.816496581	13	138	0	P_240174	P_240174	P_240174	chr20	9518168	9518228	+	LAMP5	"lysosomal-associated membrane protein family, member 5 [Source:HGNC Symbol;Acc:HGNC:16097]"	ENSG00000125869	protein_coding	exon	coding exon	1	2178	20:9518168:9518228	ENSG00000125869	LAMP5	3.98662195	0.050030336	0.846223269	-0.391048874	0.173256515	-1.237272143	-0.672966754	0.564305389	9.961963646	0.417291792	0.70399405	0.865919555	0.244356601	0.516275066	0.585026654	-6.621109033	-6.102110827	-7.030752395	-6.371209494	-6.061293768	-6.900155154	0.533533007	0.669651683

CCNF	-0.421600095	-0.058106044	-0.248815766	0.363494051	0.172784329	-0.190709722	8	0.047873595	0.816496581	0.816496581	0.816496581	0.816496581	0.816496581	0.816496581	13	140	0	P_117977	P_117977	P_117977	chr16	2431129	2431189	+	CCNF	cyclin F [Source:HGNC Symbol;Acc:HGNC:1591]	ENSG00000162063	protein_coding	exon	coding exon	1	2180	16:2431129:2431189	ENSG00000162063	CCNF	9.020653174	0.00404719	-8.116514326	-1.118639554	-4.790123996	6.997874772	3.32639033	-3.671484442	9.961963646	1.06E-05	0.2895422	0.000742355	3.80E-05	0.0077038	0.004333997	3.838574449	-5.562263698	-0.590633781	2.433030998	-2.401135078	-2.433209058	27.26489485	4.06E-05

REEP5	-0.104252896	0.012794539	-0.106845925	0.117047435	-0.002593029	-0.119640464	8	0.043166828	0.816496581	0.816496581	0.816496581	0.816496581	0.816496581	0.816496581	13	141	0	P_140093	P_140093	P_140093	chr5	112887015	112887075	-	REEP5	receptor accessory protein 5 [Source:HGNC Symbol;Acc:HGNC:30077]	ENSG00000129625	protein_coding	3'UTR;exon	multiple Ref	1	2181	5:112887015:112887075	ENSG00000129625	REEP5	9.792502409	0.003703076	-2.098226951	0.257506964	-2.150415069	2.355733915	-0.052188118	-2.407922033	9.961963646	0.062366852	0.802028421	0.057126331	0.040335748	0.959410462	0.036901511	-5.012161837	-6.147251429	-4.976032629	-4.741029958	-6.295236815	-4.579722641	3.407240923	0.061468414

KMT2E	0.179164472	-0.158452072	0.139001817	-0.337616544	-0.040162654	0.297453889	8	0.070019113	0.816496581	0.816496581	0.816496581	0.816496581	0.816496581	0.816496581	13	143	0	P_262267	P_262267	P_262267	chr7	105110308	105110368	+	KMT2E	lysine (K)-specific methyltransferase 2E [Source:HGNC Symbol;Acc:HGNC:18541]	ENSG00000005483	protein_coding	3'UTR;exon	multiple Ref	1	2183	7:105110308:105110368	ENSG00000005483	KMT2E	8.648129317	0.0061438	2.79949038	-2.47585387	2.171938703	-5.27534425	-0.627551676	4.647792574	9.961963646	0.018875842	0.032858342	0.055090268	0.000364625	0.544412951	0.000920378	-3.852483504	-3.693074718	-4.941563633	0.066451795	-6.091385592	-0.833824305	11.4182284	0.001454373

KRT76	-0.525833998	0.026931459	-0.125440785	0.552765457	0.400393213	-0.152372244	8	0.316740451	0.816496581	0.816496581	0.816496581	0.816496581	0.816496581	0.816496581	13	144	0	A_23_P61498	A_23_P61498	A_23_P61498	chr12	52768245	52768305	-	KRT76	keratin 76 [Source:HGNC Symbol;Acc:HGNC:24430]	ENSG00000185069	protein_coding	3'UTR	3'UTR	1	2184	12:52768245:52768305	ENSG00000185069	KRT76	3.761177107	0.082772738	-2.238466659	0.114646777	-0.533999352	2.353113436	1.704467307	-0.648646129	9.961963646	0.049229091	0.911002928	0.605052388	0.040516197	0.119227874	0.531234394	-4.787607199	-6.175163099	-6.893597738	-4.745350959	-4.946995524	-6.846280582	2.362020937	0.132882044

DTNA	0.110928991	0.003929246	0.197783665	-0.106999745	0.086854674	0.193854419	8	0.16428313	0.816496581	0.816496581	0.816496581	0.816496581	0.816496581	0.816496581	13	146	0	P_123420	P_123420	P_123420	chr18	34868680	34868740	+	DTNA	"dystrobrevin, alpha [Source:HGNC Symbol;Acc:HGNC:3057]"	ENSG00000134769	protein_coding	3'UTR	3'UTR	1	2186	18:34868680:34868740	ENSG00000134769	DTNA	3.490763797	0.02388028	0.879166204	0.031141188	1.567531738	-0.848025016	0.688365535	1.536390551	9.961963646	0.400020843	0.975772016	0.148176888	0.41633421	0.506935295	0.155572345	-6.592266641	-6.181576213	-5.848784798	-6.769964771	-6.050655931	-5.915019763	1.130758033	0.382981863

NDFIP1	-0.0596425	0.087444192	-0.02008588	0.147086693	0.039556621	-0.107530072	8	0.05751232	0.816496581	0.816496581	0.816496581	0.816496581	0.816496581	0.816496581	13	148	0	P_140712	P_140712	P_140712	chr5	142154216	142154276	+	NDFIP1	Nedd4 family interacting protein 1 [Source:HGNC Symbol;Acc:HGNC:17592]	ENSG00000131507	protein_coding	3'UTR	3'UTR	1	2188	5:142154216:142154276	ENSG00000131507	NDFIP1	9.872126442	0.004862921	-1.047497195	1.535776423	-0.352766941	2.583273618	0.694730254	-1.888543364	9.961963646	0.319619637	0.15572139	0.731612995	0.027340066	0.503105074	0.088386087	-6.430588004	-5.071425571	-6.97958428	-4.361375475	-6.046195332	-5.407887739	2.383808809	0.130614654

RIMS2	0.077261008	-0.000191327	0.066751257	-0.077452335	-0.010509752	0.066942584	8	0.174061722	0.816496581	0.816496581	0.816496581	0.816496581	0.816496581	0.816496581	13	149	0	P_313952	P_313952	P_313952	chr8	104254408	104254468	+	RIMS2	regulating synaptic membrane exocytosis 2 [Source:HGNC Symbol;Acc:HGNC:17283]	ENSG00000176406	protein_coding	3'UTR	3'UTR	1	2189	8:104254408:104254468	ENSG00000176406	RIMS2	3.661875783	0.026537215	0.580869176	-0.001438448	0.501854019	-0.582307624	-0.079015158	0.503292467	9.961963646	0.574233762	0.998880682	0.626675357	0.573301639	0.938585236	0.625699574	-6.817313186	-6.182086333	-6.911301252	-6.967285031	-6.293365553	-6.934740307	0.197982899	0.895382828

TRPM6	0.053723037	-0.024711418	0.142982666	-0.078434455	0.08925963	0.167694084	8	0.047960119	0.816496581	0.816496581	0.816496581	0.816496581	0.816496581	0.816496581	13	150	0	P_267583	P_267583	P_267583	chr9	74827777	74827837	-	TRPM6	"transient receptor potential cation channel, subfamily M, member 6 [Source:HGNC Symbol;Acc:HGNC:17995]"	ENSG00000119121	protein_coding	exon	coding exon	1	2190	9:74827777:74827837	ENSG00000119121	TRPM6	3.839887638	0.004053848	1.033409471	-0.475345682	2.750396299	-1.508755154	1.716986828	3.225741981	9.961963646	0.325847594	0.644791335	0.020531937	0.162404218	0.116853474	0.009129889	-6.445004257	-6.064357564	-3.981433321	-6.029207574	-4.92955605	-3.191900508	4.081394278	0.039413914

KAT6A	-0.009766509	-0.155252905	-0.210112274	-0.145486396	-0.200345765	-0.054859369	8	0.060982968	0.816496581	0.816496581	0.816496581	0.816496581	0.816496581	0.816496581	13	152	0	P_264551	P_264551	P_264551	chr8	41977013	41977073	-	KAT6A	K(lysine) acetyltransferase 6A [Source:HGNC Symbol;Acc:HGNC:13013]	ENSG00000083168	protein_coding	exon	coding exon	1	2192	8:41977013:41977073	ENSG00000083168	KAT6A	7.549261777	0.005193181	-0.165984771	-2.638569978	-3.570921513	-2.472585206	-3.404936742	-0.932351535	9.961963646	0.871489258	0.024868811	0.005118507	0.033042486	0.006751514	0.37319986	-6.983346903	-3.435352272	-2.581578957	-4.547080109	-2.274344611	-6.616214376	6.386272354	0.010921703

AKAP8	-0.171146011	-0.108576124	-0.193915681	0.062569887	-0.02276967	-0.085339557	8	0.056160609	0.816496581	0.816496581	0.816496581	0.816496581	0.816496581	0.816496581	13	154	0	P_124925	P_124925	P_124925	chr19	15355282	15355342	-	AKAP8	A kinase (PRKA) anchor protein 8 [Source:HGNC Symbol;Acc:HGNC:378]	ENSG00000105127	protein_coding	3'UTR;exon	multiple Ref	1	2194	19:15355282:15355342	ENSG00000105127	AKAP8	8.54357418	0.004739529	-3.044702119	-1.9315785	-3.449776486	1.113123619	-0.405074368	-1.518197987	9.961963646	0.012414943	0.082330557	0.006263229	0.291792934	0.693984586	0.160040913	-3.434459159	-4.523764368	-2.787320835	-6.511197496	-6.210145116	-5.939577668	4.76836981	0.025972695

C4orf19	0.071230298	0.021468716	0.236921243	-0.049761582	0.165690945	0.215452526	8	0.109408119	0.816496581	0.816496581	0.816496581	0.816496581	0.816496581	0.816496581	13	155	0	P_137511	P_137511	P_137511	chr4	37591157	37591217	+	C4orf19	chromosome 4 open reading frame 19 [Source:HGNC Symbol;Acc:HGNC:25618]	ENSG00000154274	protein_coding	3'UTR	3'UTR	1	2195	4:37591157:37591217	ENSG00000154274	C4orf19	5.484147776	0.011819356	0.802441681	0.241854847	2.669025481	-0.560586834	1.866583799	2.427170633	9.961963646	0.441020497	0.813798474	0.023604163	0.587464575	0.091633431	0.035708648	-6.657960441	-6.151345117	-4.119313549	-6.980427124	-4.716264068	-4.547810976	2.919275567	0.086922017

USP6NL	0.116004725	-0.029135926	0.049351611	-0.145140651	-0.066653114	0.078487537	8	0.064216686	0.816496581	0.816496581	0.816496581	0.816496581	0.816496581	0.816496581	13	156	0	P_308627	P_308627	P_308627	chr10	11464087	11464147	+	USP6NL	USP6 N-terminal like [Source:HGNC Symbol;Acc:HGNC:16858]	ENSG00000148429	protein_coding	intron	intron antisense	0	2196	10:11464087:11464147	ENSG00000148429	USP6NL	8.054852727	0.005518306	1.912574691	-0.480365213	0.813662055	-2.392939904	-1.098912636	1.294027267	9.961963646	0.084955238	0.641341678	0.43485529	0.037856963	0.297654176	0.224850196	-5.300569769	-6.061887645	-6.697162615	-4.679538467	-5.691615947	-6.226304344	2.19316926	0.152110191

LINC00844	-0.524995537	-0.267861564	-0.624166555	0.257133973	-0.099171018	-0.356304991	8	0.282648203	0.816496581	0.816496581	0.816496581	0.816496581	0.816496581	0.816496581	14	15	0	A_32_P160294	A_32_P160294	A_32_P160294	chr10	59001303	59001363	+	LINC00844	long intergenic non-protein coding RNA 844 [Source:HGNC Symbol;Acc:HGNC:45031]	ENSG00000237949	lincRNA	exon	non-coding exon	1	2225	10:59001303:59001363	ENSG00000237949	LINC00844	4.171279537	0.066362715	-2.495968883	-1.273485357	-2.967454368	1.222483527	-0.471485484	-1.693969011	9.961963646	0.031747227	0.231757236	0.014162953	0.249659404	0.64745028	0.121252489	-4.364025503	-5.392611504	-3.611470943	-6.388464285	-6.179790766	-5.695129779	3.540617765	0.056125263

EEPD1	0.089122259	-0.182188633	0.031747453	-0.271310892	-0.057374806	0.213936086	8	0.16475671	0.816496581	0.816496581	0.816496581	0.816496581	0.816496581	0.816496581	14	17	0	P_144609	P_144609	P_144609	chr7	36299307	36299367	+	EEPD1	endonuclease/exonuclease/phosphatase family domain containing 1 [Source:HGNC Symbol;Acc:HGNC:22223]	ENSG00000122547	protein_coding	3'UTR	3'UTR	1	2227	7:36299307:36299367	ENSG00000122547	EEPD1	8.17813464	0.024005417	0.704493816	-1.440165082	0.25095733	-2.144658898	-0.453536486	1.691122412	9.961963646	0.497263956	0.180498235	0.806947475	0.057682966	0.659881731	0.121806787	-6.734110875	-5.192999629	-7.012835875	-5.08425456	-6.18842991	-5.699209966	1.716419787	0.226667472

C1RL	0.087822023	0.026929761	-0.060000318	-0.060892262	-0.147822341	-0.086930078	8	0.112606176	0.816496581	0.816496581	0.816496581	0.816496581	0.816496581	0.816496581	14	18	0	P_110852	P_110852	P_110852	chr12	7094669	7094729	+	C1RL;C1R	"complement component 1, r subcomponent-like [Source:HGNC Symbol;Acc:HGNC:21265];complement component 1, r subcomponent [Source:HGNC Symbol;Acc:HGNC:1246]"	ENSG00000139178	protein_coding	3'ext;3'UTR;intron;promoter	multiple non-Ref	0	2228	12:7094669:7094729	ENSG00000139178	C1RL	5.656778281	0.012389537	0.96632126	0.296312921	-0.660194116	-0.670008339	-1.626515376	-0.956507037	9.961963646	0.356756255	0.773076393	0.52409977	0.518081184	0.135016192	0.361452039	-6.511475995	-6.136012309	-6.814192545	-6.909521558	-5.054022516	-6.593351636	0.911133925	0.470005274

CCDC25	0.204543908	0.080139337	0.151847289	-0.124404571	-0.052696619	0.071707953	8	0.125461782	0.816496581	0.816496581	0.816496581	0.816496581	0.816496581	0.816496581	14	19	0	A_24_P158142	A_24_P158142	A_24_P158142	chr8	27748538	27748598	-	CCDC25	coiled-coil domain containing 25 [Source:HGNC Symbol;Acc:HGNC:25591]	ENSG00000147419	protein_coding	3'UTR;exon	multiple Ref	1	2229	8:27748538:27748598	ENSG00000147419	CCDC25	7.228620315	0.014847291	2.055931145	0.805504107	1.526261888	-1.250427038	-0.529669257	0.720757781	9.961963646	0.066947483	0.439332074	0.158046545	0.239716731	0.60794249	0.487627199	-5.078829681	-5.851162789	-5.904906091	-6.35573343	-6.149595837	-6.794781026	1.594795695	0.251987602

PDILT	0.087064271	0.096358413	0.18703352	0.009294143	0.099969249	0.090675107	8	0.137003586	0.816496581	0.816496581	0.816496581	0.816496581	0.816496581	0.816496581	14	20	0	A_14_P103345	A_14_P103345	A_14_P103345	chr16	20395175	20395235	+	PDILT	"protein disulfide isomerase-like, testis expressed [Source:HGNC Symbol;Acc:HGNC:27338]"	ENSG00000169340	protein_coding	intron	intron antisense	0	2230	16:20395175:20395235	ENSG00000169340	PDILT	3.588886191	0.017280003	0.811173367	0.897766421	1.742581761	0.086593054	0.931408394	0.84481534	9.961963646	0.436217754	0.390491549	0.112132226	0.932710761	0.373664041	0.418041115	-6.650747309	-5.774190192	-5.601206139	-7.145514015	-5.854888856	-6.694902733	1.014885147	0.426506581

CXCR1	-0.083384847	-0.186007943	-0.142990955	-0.102623095	-0.059606108	0.043016988	8	0.274944296	0.816496581	0.816496581	0.816496581	0.816496581	0.816496581	0.816496581	14	21	0	P_129843	P_129843	P_129843	chr2	218163833	218163893	-	CXCR1	chemokine (C-X-C motif) receptor 1 [Source:HGNC Symbol;Acc:HGNC:6026]	ENSG00000163464	protein_coding	3'UTR	3'UTR	1	2231	2:218163833:218163893	ENSG00000163464	CXCR1	3.930090674	0.062913082	-0.407157358	-0.908252579	-0.698206226	-0.501095221	-0.291048868	0.210046353	9.961963646	0.692503228	0.385190059	0.501020754	0.627190399	0.776983535	0.837865137	-6.908647574	-5.764993401	-6.787251939	-7.014009138	-6.251826254	-7.047329497	0.309686188	0.818005552

VWDE	0.106222928	-0.060073382	-0.067620258	-0.16629631	-0.173843186	-0.007546876	8	0.039959374	0.816496581	0.816496581	0.816496581	0.816496581	0.816496581	0.816496581	14	22	0	P_260499	P_260499	P_260499	chr7	12340321	12340381	-	VWDE	von Willebrand factor D and EGF domains [Source:HGNC Symbol;Acc:HGNC:21897]	ENSG00000146530	protein_coding	3'UTR;exon	multiple Ref	1	2232	7:12340321:12340381	ENSG00000146530	VWDE	6.058220908	0.003488963	2.202499129	-1.245602738	-1.402084854	-3.448101867	-3.604583983	-0.156482116	9.961963646	0.05231883	0.241409919	0.191272452	0.006280792	0.00484065	0.878778779	-4.845677436	-5.42434086	-6.068022447	-2.883742733	-1.9541874	-7.058017911	5.54588156	0.016817197

NPHP1	0.129171042	0.175989386	0.263662996	0.046818345	0.134491954	0.087673609	8	0.0385608	0.816496581	0.816496581	0.816496581	0.816496581	0.816496581	0.816496581	14	24	0	P_236154	P_236154	P_236154	chr2	110160126	110160186	-	NPHP1	nephronophthisis 1 (juvenile) [Source:HGNC Symbol;Acc:HGNC:7905]	ENSG00000144061	protein_coding	exon	coding exon	1	2234	2:110160126:110160186	ENSG00000144061	NPHP1	6.270509129	0.003400774	2.71282575	3.696095756	5.537400294	0.983270005	2.824574543	1.841304538	9.961963646	0.021897236	0.004161781	0.000251864	0.348751801	0.018082248	0.095508505	-3.999465845	-1.755310204	0.533249307	-6.64514588	-3.216517694	-5.479056768	10.66979884	0.001873693

ARHGEF37	-0.064622305	-0.19071703	0.012818044	-0.126094725	0.077440349	0.203535074	8	0.048952899	0.816496581	0.816496581	0.816496581	0.816496581	0.816496581	0.816496581	14	25	0	A_16_P01363492	A_16_P01363492	A_16_P01363492	chr5	149633626	149633686	+	ARHGEF37	Rho guanine nucleotide exchange factor (GEF) 37 [Source:HGNC Symbol;Acc:HGNC:34430]	ENSG00000183111	protein_coding	3'UTR	3'UTR	1	2235	5:149633626:149633686	ENSG00000183111	ARHGEF37	5.798561015	0.004131113	-1.231386946	-3.634139343	0.244249596	-2.402752397	1.475636542	3.87838894	9.961963646	0.246455841	0.004609621	0.811994438	0.03722849	0.170934647	0.003088896	-6.228620327	-1.851812458	-7.014638718	-4.663278356	-5.252876161	-2.085635708	6.29592725	0.011420833

AIF1	0.205529874	-0.338308096	-0.203642547	-0.54383797	-0.409172421	0.13466555	8	0.432272399	0.816496581	0.816496581	0.816496581	0.816496581	0.816496581	0.816496581	14	26	0	P_142126	P_142126	P_142126	chr6	31616373	31616433	+	AIF1	allograft inflammatory factor 1 [Source:HGNC Symbol;Acc:HGNC:352]	ENSG00000204472	protein_coding	exon	coding exon	1	2236	6:31616373:31616433	ENSG00000204472	AIF1	4.823485457	0.152264993	0.645090361	-1.061837327	-0.639166664	-1.706927688	-1.284257025	0.422670663	9.961963646	0.533442661	0.313372969	0.537133392	0.118757828	0.228113913	0.681513774	-6.77592776	-5.620303439	-6.828502363	-5.753105132	-5.487090118	-6.974652047	1.115623806	0.388381604

CABLES1	0.115810293	-0.035022829	0.113527477	-0.150833122	-0.002282817	0.148550305	8	0.077608041	0.816496581	0.816496581	0.816496581	0.816496581	0.816496581	0.816496581	14	28	0	P_123292	P_123292	P_123292	chr18	23235904	23235964	+	CABLES1	Cdk5 and Abl enzyme substrate 1 [Source:HGNC Symbol;Acc:HGNC:25097]	ENSG00000134508	protein_coding	exon	coding exon	1	2238	18:23235904:23235964	ENSG00000134508	CABLES1	7.040601431	0.007043488	1.690047486	-0.511096569	1.656733793	-2.201144055	-0.033313693	2.167830363	9.961963646	0.122016694	0.620418843	0.128685785	0.05243882	0.974082463	0.055473441	-5.629473618	-6.046228496	-5.72447106	-4.993441851	-6.296095002	-4.971505131	2.567980084	0.113161273

BLMH	-0.196614537	-0.016198127	-0.12855695	0.18041641	0.068057587	-0.112358823	8	0.037975172	0.816496581	0.816496581	0.816496581	0.816496581	0.816496581	0.816496581	14	29	0	P_121012	P_121012	P_121012	chr17	30266893	30266953	-	BLMH	bleomycin hydrolase [Source:HGNC Symbol;Acc:HGNC:1059]	ENSG00000108578	protein_coding	3'UTR;exon	multiple Ref	1	2239	17:30266893:30266953	ENSG00000108578	BLMH	9.970730892	0.00336478	-4.151288562	-0.342004711	-2.714331322	3.809283851	1.43695724	-2.372326611	9.961963646	0.001992166	0.739452665	0.021840812	0.003456876	0.181385362	0.03921126	-1.570986557	-6.12079983	-4.042615742	-2.27144272	-5.301930844	-4.638561577	7.820196932	0.005638685

EEF1A2	0.150933328	-0.095625833	0.054783319	-0.246559161	-0.096150009	0.150409152	8	0.090978987	0.816496581	0.816496581	0.816496581	0.816496581	0.816496581	0.816496581	14	31	0	P_241326	P_241326	P_241326	chr20	63495903	63495963	-	EEF1A2	eukaryotic translation elongation factor 1 alpha 2 [Source:HGNC Symbol;Acc:HGNC:3192]	ENSG00000101210	protein_coding	exon	coding exon	1	2241	20:63495903:63495963	ENSG00000101210	EEF1A2	10.13339866	0.008853708	1.964573087	-1.24468161	0.713068714	-3.209254697	-1.251504374	1.957750324	9.961963646	0.077953003	0.241734312	0.492168565	0.009388174	0.239339942	0.078839904	-5.220937587	-5.425380502	-6.776346624	-3.292591062	-5.524923144	-5.302122346	3.602601993	0.053830954

YKT6	-0.149942293	0.042849209	-0.080431595	0.192791502	0.069510697	-0.123280804	8	0.113649983	0.816496581	0.816496581	0.816496581	0.816496581	0.816496581	0.816496581	14	32	0	P_261111	P_261111	P_261111	chr7	44208137	44208197	+	YKT6	YKT6 v-SNARE homolog (S. cerevisiae) [Source:HGNC Symbol;Acc:HGNC:16959]	ENSG00000106636	protein_coding	exon	coding exon	1	2242	7:44208137:44208197	ENSG00000106636	YKT6	9.779590849	0.012579192	-1.637357076	0.467909716	-0.878306177	2.105266792	0.7590509	-1.346215893	9.961963646	0.13271365	0.64991796	0.400465336	0.061634036	0.465402976	0.208064922	-5.704170668	-6.067971016	-6.641469647	-5.147079297	-5.999052337	-6.162318767	1.748650534	0.220456899

FAM135A	-0.091370615	0.061457906	-0.038907043	0.152828521	0.052463572	-0.100364949	8	0.077886668	0.816496581	0.816496581	0.816496581	0.816496581	0.816496581	0.816496581	14	33	0	P_257703	P_257703	P_257703	chr6	70538341	70538401	+	FAM135A	"family with sequence similarity 135, member A [Source:HGNC Symbol;Acc:HGNC:21084]"	ENSG00000082269	protein_coding	3'UTR;exon	multiple Ref	1	2243	6:70538341:70538401	ENSG00000082269	FAM135A	6.331015646	0.00707828	-1.330112277	0.894663077	-0.566382701	2.224775354	0.763729576	-1.461045778	9.961963646	0.213129034	0.392070299	0.583667119	0.050383859	0.462732781	0.174814267	-6.110312615	-5.776894684	-6.874716226	-4.955209331	-5.995478174	-6.015528421	1.759662177	0.218380211

WARS2-AS1	-0.289940946	-0.159078616	-0.015354851	0.13086233	0.274586095	0.143723765	8	0.19475313	0.816496581	0.816496581	0.816496581	0.816496581	0.816496581	0.816496581	14	34	0	A_32_P214807	A_32_P214807	A_32_P214807	chr1	119163052	119163112	-	RP11-418J17.1	NA	ENSG00000231365	antisense	intron	intron antisense	0	2244	1:119163052:119163112	ENSG00000231365	WARS2-AS1	4.767203748	0.032665564	-1.964762685	-1.077984097	-0.104050974	0.886778588	1.860711711	0.973933123	9.961963646	0.077928491	0.306450989	0.919193904	0.396101445	0.092520357	0.353144934	-5.220645505	-5.604044543	-7.041211317	-6.735797482	-4.724797022	-6.576556884	1.705056465	0.228904992

MYH2	-0.073401978	0.008539581	0.207188367	0.081941559	0.280590344	0.198648786	8	0.156587429	0.816496581	0.816496581	0.816496581	0.816496581	0.816496581	0.816496581	14	35	0	P_227827	P_227827	P_227827	chr17	10542885	10542945	-	MYH2	"myosin, heavy chain 2, skeletal muscle, adult [Source:HGNC Symbol;Acc:HGNC:7572]"	ENSG00000125414	protein_coding	exon	coding exon	1	2245	17:10542885:10542945	ENSG00000125414	MYH2	3.700172849	0.021897278	-0.607516843	0.070678467	1.714809688	0.67819531	2.322326531	1.644131221	9.961963646	0.557101752	0.945052394	0.117263279	0.513092272	0.042696146	0.131292847	-6.800628964	-6.179454654	-5.641452889	-6.903750333	-4.023027108	-5.76601132	1.978493549	0.181457276

TLK1	-0.071936661	-0.103172685	-0.072423169	-0.031236024	-0.000486508	0.030749516	8	0.086566641	0.816496581	0.816496581	0.816496581	0.816496581	0.816496581	0.816496581	14	36	0	P_237521	P_237521	P_237521	chr2	171006973	171007033	-	TLK1	tousled-like kinase 1 [Source:HGNC Symbol;Acc:HGNC:11841]	ENSG00000198586	protein_coding	3'UTR;exon	multiple Ref	1	2246	2:171006973:171007033	ENSG00000198586	TLK1	6.598069026	0.008224601	-0.971490361	-1.393326681	-0.978060552	-0.42183632	-0.006570191	0.415266129	9.961963646	0.35430094	0.193826471	0.351197952	0.682102857	0.994887493	0.686749517	-6.506484525	-5.250742247	-6.548469135	-7.053164516	-6.296662297	-6.977982493	0.698698369	0.573965706

EZR	-0.012760145	-0.04983041	0.081136745	-0.037070265	0.09389689	0.130967155	8	0.093930913	0.816496581	0.816496581	0.816496581	0.816496581	0.816496581	0.816496581	14	37	0	P_143936	P_143936	P_143936	chr6	158766087	158766147	-	EZR	ezrin [Source:HGNC Symbol;Acc:HGNC:12691]	ENSG00000092820	protein_coding	3'UTR	3'UTR	1	2247	6:158766087:158766147	ENSG00000092820	EZR	7.162771265	0.009292047	-0.162123341	-0.633117601	1.030878554	-0.47099426	1.193001895	1.663996155	9.961963646	0.874449894	0.540917504	0.326976081	0.647789015	0.260507568	0.127204617	-6.984036398	-5.975090608	-6.495894011	-7.029637649	-5.590736249	-5.737900576	0.983967464	0.439002886

CAB39	-0.000333293	-0.020515062	0.024958596	-0.020181769	0.025291888	0.045473657	8	0.06247702	0.816496581	0.816496581	0.816496581	0.816496581	0.816496581	0.816496581	14	38	0	P_130110	P_130110	P_130110	chr2	230820739	230820799	+	CAB39	calcium binding protein 39 [Source:HGNC Symbol;Acc:HGNC:20292]	ENSG00000135932	protein_coding	3'UTR	3'UTR	1	2248	2:230820739:230820799	ENSG00000135932	CAB39	8.02027314	0.005341309	-0.005585313	-0.343791068	0.418255742	-0.338205755	0.423841055	0.76204681	9.961963646	0.995653853	0.738149207	0.68463345	0.742227541	0.680687802	0.463692024	-6.998343295	-6.120161853	-6.95242053	-7.087419296	-6.202016076	-6.763096939	0.194650179	0.897646747

GRM5	0.113026585	0.020734407	0.131908663	-0.092292178	0.018882078	0.111174256	8	0.15570024	0.816496581	0.816496581	0.816496581	0.816496581	0.816496581	0.816496581	14	39	0	P_212702	P_212702	P_212702	chr11	88597183	88597243	-	GRM5	"glutamate receptor, metabotropic 5 [Source:HGNC Symbol;Acc:HGNC:4597]"	ENSG00000168959	protein_coding	exon	coding exon	1	2249	11:88597183:88597243	ENSG00000168959	GRM5	3.59097677	0.021674785	0.940261888	0.17248838	1.097340848	-0.767773508	0.15707896	0.924852468	9.961963646	0.36932292	0.866507541	0.298308003	0.460432821	0.878320581	0.376902046	-6.536302974	-6.166427238	-6.426597123	-6.836481008	-6.283578358	-6.623212495	0.597917274	0.630791269

XRCC6P5	-0.166900784	-0.186771479	-0.264455983	-0.019870695	-0.097555199	-0.077684504	8	0.068505766	0.816496581	0.816496581	0.816496581	0.816496581	0.816496581	0.816496581	14	40	0	P_270873	P_270873	P_270873	chrX	99719481	99719541	-	XRCC6P5	X-ray repair complementing defective repair in Chinese hamster cells 6 pseudogene 5 [Source:HGNC Symbol;Acc:HGNC:45187]	ENSG00000215070	processed_pseudogene	exon	non-coding exon	1	2250	X:99719481:99719541	ENSG00000215070	XRCC6P5	11.02512296	0.005975451	-2.644348323	-2.959176315	-4.189996705	-0.314827991	-1.545648382	-1.23082039	9.961963646	0.024623778	0.014364542	0.001873488	0.759387397	0.153340632	0.246658699	-4.115152439	-2.922923029	-1.549249998	-7.095678969	-5.162037127	-6.301373495	6.218073221	0.011872983

NSMCE1	0.041911333	-0.011061045	0.059210605	-0.052972378	0.017299272	0.07027165	8	0.09350095	0.816496581	0.816496581	0.816496581	0.816496581	0.816496581	0.816496581	14	41	0	A_32_P171124	A_32_P171124	A_32_P171124	chr16	27231062	27231122	+	NSMCE1	non-SMC element 1 homolog (S. cerevisiae) [Source:HGNC Symbol;Acc:HGNC:29897]	ENSG00000169189	protein_coding	intron;promoter	multiple non-Ref	0	2251	16:27231062:27231122	ENSG00000169189	NSMCE1	12.08689667	0.00922733	0.534366319	-0.141027483	0.754930727	-0.675393801	0.220564409	0.89595821	9.961963646	0.604807785	0.890659203	0.467762607	0.514796171	0.829886259	0.391410888	-6.844748687	-6.171613528	-6.744522164	-6.905732389	-6.270874757	-6.649730371	0.363817179	0.780656694

DMGDH	0.149658406	0.052577072	0.222059671	-0.097081334	0.072401264	0.169482599	8	0.208723199	0.816496581	0.816496581	0.816496581	0.816496581	0.816496581	0.816496581	14	42	0	P_253584	P_253584	P_253584	chr5	79030832	79030892	-	DMGDH	dimethylglycine dehydrogenase [Source:HGNC Symbol;Acc:HGNC:24475]	ENSG00000132837	protein_coding	3'UTR;exon	multiple Ref	1	2252	5:79030832:79030892	ENSG00000132837	DMGDH	4.307580253	0.037192055	0.950433337	0.333900402	1.410230932	-0.616532935	0.459797595	1.07633053	9.961963646	0.364380462	0.745376993	0.188922661	0.551370944	0.655532688	0.307154438	-6.526679718	-6.123653435	-6.057598751	-6.945633152	-6.185452769	-6.472908386	0.792263064	0.525594815

MED8	-0.055767511	0.129142848	-0.05030484	0.184910359	0.005462671	-0.179447688	8	0.083800466	0.816496581	0.816496581	0.816496581	0.816496581	0.816496581	0.816496581	14	43	0	A_24_P233915	A_24_P233915	A_24_P233915	chr1	43386612	43386672	-	MED8	mediator complex subunit 8 [Source:HGNC Symbol;Acc:HGNC:19971]	ENSG00000159479	protein_coding	exon	coding exon	1	2253	1:43386612:43386672	ENSG00000159479	MED8	9.937319973	0.007846149	-0.771078535	1.785614548	-0.695548031	2.556693083	0.075530504	-2.481162579	9.961963646	0.458558609	0.104584567	0.502614229	0.028613415	0.941287902	0.032561425	-6.683301795	-4.733054521	-6.789180461	-4.406123489	-6.293651817	-4.457970748	2.827553435	0.093007839

SLC5A7	-0.025242689	-0.053106336	0.149004367	-0.027863647	0.174247056	0.202110703	8	0.186182628	0.816496581	0.816496581	0.816496581	0.816496581	0.816496581	0.816496581	14	44	0	P_236096	P_236096	P_236096	chr2	108001980	108002040	+	SLC5A7	"solute carrier family 5 (sodium/choline cotransporter), member 7 [Source:HGNC Symbol;Acc:HGNC:14025]"	ENSG00000115665	protein_coding	exon	coding exon	1	2254	2:108001980:108002040	ENSG00000115665	SLC5A7	3.407854191	0.030043743	-0.178362781	-0.375245033	1.052852681	-0.196882252	1.231215462	1.428097714	9.961963646	0.862013052	0.715344066	0.317275805	0.847878409	0.246517228	0.183854891	-6.981027944	-6.10839845	-6.473363986	-7.128456466	-5.548007042	-6.058460903	0.812539683	0.51565756

FAM13A-AS1	-1.505846632	-0.033564888	-1.338829078	1.472281744	0.167017554	-1.30526419	8	0.236220063	0.816496581	0.816496581	0.816496581	0.816496581	0.816496581	0.816496581	14	45	0	P_138182	P_138182	P_138182	chr4	88722087	88722147	+	FAM13A-AS1	FAM13A antisense RNA 1 [Source:HGNC Symbol;Acc:HGNC:19370]	ENSG00000248019	antisense	exon	non-coding exon	1	2255	4:88722087:88722147	ENSG00000248019	FAM13A-AS1	6.527713536	0.047017061	-8.505476189	-0.189584616	-7.562110642	8.315891573	0.943365546	-7.372526027	9.961963646	7.03E-06	0.853441744	1.96E-05	8.58E-06	0.367809719	2.44E-05	4.259450121	-6.163175174	3.182710252	3.982552037	-5.843964114	2.943522304	42.32711935	5.61E-06

GDI1	0.04943685	0.006094782	0.134334598	-0.043342068	0.084897747	0.128239816	8	0.117703083	0.816496581	0.816496581	0.816496581	0.816496581	0.816496581	0.816496581	14	47	0	P_271873	P_271873	P_271873	chrX	154441101	154441161	+	GDI1	GDP dissociation inhibitor 1 [Source:HGNC Symbol;Acc:HGNC:4226]	ENSG00000203879	protein_coding	exon	coding exon	1	2257	X:154441101:154441161	ENSG00000203879	GDI1	9.459090464	0.013332214	0.524378992	0.06464764	1.424893383	-0.459731353	0.900514391	1.360245743	9.961963646	0.611483089	0.949733427	0.184755132	0.65557863	0.38909732	0.203735468	-6.850359675	-6.179884688	-6.038735935	-7.03524755	-5.882574805	-6.144818804	0.863670227	0.491438339

MFAP4	0.024860965	-0.109780908	0.230697496	-0.134641873	0.205836531	0.340478404	8	0.290028194	0.816496581	0.816496581	0.816496581	0.816496581	0.816496581	0.816496581	14	48	0	P_120795	P_120795	P_120795	chr17	19385392	19385452	-	MFAP4	microfibrillar-associated protein 4 [Source:HGNC Symbol;Acc:HGNC:7035]	ENSG00000166482	protein_coding	exon	coding exon	1	2258	17:19385392:19385452	ENSG00000166482	MFAP4	3.877978727	0.069756702	0.115284426	-0.509072314	1.069782624	-0.62435674	0.954498199	1.578854938	9.961963646	0.910510339	0.621786422	0.30995214	0.546425251	0.362418704	0.14556566	-6.991112669	-6.047288281	-6.455747375	-6.940522638	-5.833688795	-5.857005997	0.868425175	0.489246184

ING3	-0.01935834	-0.035338459	0.004918791	-0.01598012	0.024277131	0.040257251	8	0.082088964	0.816496581	0.816496581	0.816496581	0.816496581	0.816496581	0.816496581	14	49	0	P_262649	P_262649	P_262649	chr7	120967978	120968038	+	ING3	"inhibitor of growth family, member 3 [Source:HGNC Symbol;Acc:HGNC:14587]"	ENSG00000071243	protein_coding	3'UTR;exon	multiple Ref	1	2259	7:120967978:120968038	ENSG00000071243	ING3	8.238138124	0.007618146	-0.271637111	-0.495870883	0.069020706	-0.224233772	0.340657817	0.564891589	9.961963646	0.791447216	0.630742164	0.946338911	0.827107438	0.740436032	0.584642832	-6.958245077	-6.054101978	-7.044519637	-7.122185762	-6.235329624	-6.899804828	0.134978258	0.93692099

MBOAT2	-1.252303922	0.065205048	-1.272449422	1.31750897	-0.0201455	-1.33765447	8	0.098748212	0.816496581	0.816496581	0.816496581	0.816496581	0.816496581	0.816496581	14	50	0	P_233515	P_233515	P_233515	chr2	8858542	8858602	+	MBOAT2	membrane bound O-acyltransferase domain containing 2 [Source:HGNC Symbol;Acc:HGNC:25193]	ENSG00000143797	protein_coding	3'UTR	3'UTR antisense	0	2260	2:8858542:8858602	ENSG00000143797	MBOAT2	10.01802055	0.010037437	-15.30889903	0.797104822	-15.55516946	16.10600385	-0.246270432	-16.35227429	9.961963646	3.00E-08	0.443973155	2.58E-08	1.84E-08	0.810472978	1.59E-08	9.574966193	-5.857811306	9.75055621	10.12191529	-6.26452785	10.20947742	167.3036577	8.07E-09

MMP24OS	0.2307644	0.362403264	0.559810282	0.131638864	0.329045882	0.197407018	8	0.189768004	0.816496581	0.816496581	0.816496581	0.816496581	0.816496581	0.816496581	14	51	0	P_131062	P_131062	P_131062	chr20	35272008	35272068	-	MMP24-AS1;RP4-614O4.11	MMP24 antisense RNA 1 [Source:HGNC Symbol;Acc:HGNC:44421];	ENSG00000126005	antisense	exon	non-coding exon	1	2261	20:35272008:35272068	ENSG00000126005	MMP24OS	8.895729525	0.0311262	1.601958979	2.515791706	3.886184819	0.913832726	2.28422584	1.370393114	9.961963646	0.140363616	0.030688584	0.003050036	0.382389718	0.045552613	0.200651512	-5.753592852	-3.630062049	-2.051244656	-6.711174379	-4.082880001	-6.1320843	5.321444693	0.018994183

GALNT3	0.687502666	0.197559787	1.466240383	-0.489942879	0.778737717	1.268680596	8	0.157653082	0.816496581	0.816496581	0.816496581	0.816496581	0.816496581	0.816496581	14	52	0	P_237311	P_237311	P_237311	chr2	165754626	165754686	-	GALNT3	polypeptide N-acetylgalactosaminyltransferase 3 [Source:HGNC Symbol;Acc:HGNC:4125]	ENSG00000115339	protein_coding	exon	coding exon	1	2262	2:165754626:165754686	ENSG00000115339	GALNT3	10.05141844	0.022166198	5.655544346	1.625169172	12.06160778	-4.030375174	6.406063438	10.43643861	9.961963646	0.000213846	0.135304571	2.89E-07	0.002416605	7.91E-05	1.11E-06	0.739262655	-4.953578909	7.44828734	-1.902089637	1.950040115	6.110039663	57.71826046	1.33E-06

USP42	-0.002464427	-0.002181692	-0.003462667	0.000282735	-0.00099824	-0.001280975	8	0.042662184	0.816496581	0.816496581	0.816496581	0.816496581	0.816496581	0.816496581	14	54	0	P_260404	P_260404	P_260404	chr7	6135891	6135951	+	USP42	ubiquitin specific peptidase 42 [Source:HGNC Symbol;Acc:HGNC:20068]	ENSG00000106346	protein_coding	3'UTR;exon	multiple Ref	1	2264	7:6135891:6135951	ENSG00000106346	USP42	6.923121531	0.003668293	-0.049834439	-0.044117117	-0.070020362	0.005717322	-0.020185923	-0.025903245	9.961963646	0.961239341	0.96568283	0.945563101	0.995551133	0.984293642	0.979846055	-6.997005277	-6.181061484	-7.044443769	-7.149595188	-6.296468533	-7.07101198	0.00174063	0.999892517

STRN	0.172135191	0.051354731	0.170902379	-0.120780461	-0.001232812	0.119547648	8	0.069996283	0.816496581	0.816496581	0.816496581	0.816496581	0.816496581	0.816496581	14	55	0	P_234416	P_234416	P_234416	chr2	36855222	36855282	-	STRN	"striatin, calmodulin binding protein [Source:HGNC Symbol;Acc:HGNC:11424]"	ENSG00000115808	protein_coding	exon	coding exon	1	2265	2:36855222:36855282	ENSG00000115808	STRN	7.667227136	0.006141233	2.690218145	0.802598395	2.670951114	-1.88761975	-0.019267031	1.868352719	9.961963646	0.022762258	0.440933991	0.023526396	0.088520487	0.985008518	0.0913678	-4.037708247	-5.853469713	-4.116057606	-5.485520392	-6.296487815	-5.438411748	3.678273187	0.051179392

MTUS2	0.101216333	0.107011099	0.110532607	0.005794766	0.009316274	0.003521509	8	0.084983599	0.816496581	0.816496581	0.816496581	0.816496581	0.816496581	0.816496581	14	56	0	A_16_P02745845	A_16_P02745845	A_16_P02745845	chr13	29498451	29498511	+	MTUS2	microtubule associated tumor suppressor candidate 2 [Source:HGNC Symbol;Acc:HGNC:20595]	ENSG00000132938	protein_coding	exon	coding exon	1	2266	13:29498451:29498511	ENSG00000132938	MTUS2	4.310227428	0.008006514	1.385397798	1.464713608	1.512914233	0.07931581	0.127516435	0.048200625	9.961963646	0.196163544	0.173831925	0.16135958	0.93835209	0.901068965	0.962509002	-6.041289679	-5.162249112	-5.922859834	-7.14617382	-6.288043831	-7.070109447	1.063082871	0.40778264

RMC1	-0.202076331	0.054488033	-0.077721422	0.256564363	0.124354909	-0.132209454	8	0.063461323	0.816496581	0.816496581	0.816496581	0.816496581	0.816496581	0.816496581	14	57	0	P_230888	P_230888	P_230888	chr18	23519093	23519153	+	C18orf8	chromosome 18 open reading frame 8 [Source:HGNC Symbol;Acc:HGNC:24326]	ENSG00000141452	protein_coding	3'UTR;exon	multiple Ref	1	2267	18:23519093:23519153	ENSG00000141452	RMC1	7.702658291	0.005440857	-3.355269543	0.904717719	-1.290484234	4.259987262	2.064785309	-2.195201953	9.961963646	0.007338518	0.386971474	0.226029256	0.001677418	0.065962862	0.052968124	-2.904271886	-5.76810366	-6.20664712	-1.524015645	-4.421583397	-4.927503969	6.828573145	0.008824038

CD28	0.115916508	-0.069280293	0.137665897	-0.185196801	0.021749389	0.206946189	8	0.193475471	0.816496581	0.816496581	0.816496581	0.816496581	0.816496581	0.816496581	14	59	0	P_129666	P_129666	P_129666	chr2	203729712	203729772	+	CD28	CD28 molecule [Source:HGNC Symbol;Acc:HGNC:1653]	ENSG00000178562	protein_coding	exon	coding exon	1	2269	2:203729712:203729772	ENSG00000178562	CD28	4.342157674	0.03226723	0.790332933	-0.472361514	0.938622926	-1.262694447	0.148289993	1.41098444	9.961963646	0.447738497	0.646846403	0.370123806	0.235454799	0.88507257	0.188706554	-6.667849817	-6.06581423	-6.58623779	-6.341194389	-6.28500243	-6.080506274	0.889338137	0.479724243

DCAF17	-0.058198047	0.060911493	0.031274101	0.11910954	0.089472148	-0.029637392	8	0.045582342	0.816496581	0.816496581	0.816496581	0.816496581	0.816496581	0.816496581	14	60	0	P_129217	P_129217	P_129217	chr2	171458417	171458477	+	DCAF17	DDB1 and CUL4 associated factor 17 [Source:HGNC Symbol;Acc:HGNC:25784]	ENSG00000115827	protein_coding	exon	coding exon	1	2270	2:171458417:171458477	ENSG00000115827	DCAF17	6.887283102	0.00387523	-1.144999436	1.198384295	0.615292609	2.343383731	1.760292045	-0.583091686	9.961963646	0.27897263	0.258499331	0.552157329	0.041193121	0.108967709	0.572793912	-6.326612967	-5.476914766	-6.84423165	-4.761382833	-4.868723794	-6.888758762	2.009300821	0.176864425

CDK20	0.05597125	0.116546252	0.172801933	0.060575003	0.116830684	0.056255681	8	0.072503309	0.816496581	0.816496581	0.816496581	0.816496581	0.816496581	0.816496581	14	61	0	P_149013	P_149013	P_149013	chr9	87967071	87967131	+	CDK20	cyclin-dependent kinase 20 [Source:HGNC Symbol;Acc:HGNC:21420]	ENSG00000156345	protein_coding	3'UTR;exon	multiple non-Ref	0	2271	9:87967071:87967131	ENSG00000156345	CDK20	8.459020313	0.006428125	0.855004678	1.780335287	2.639684871	0.925330609	1.784680194	0.859349585	9.961963646	0.412638865	0.105484829	0.024821345	0.376665215	0.104743386	0.410349858	-6.61351337	-4.740478864	-4.16887758	-6.700520407	-4.834127758	-6.682297216	2.608060798	0.109735734

CLASP2	0.018278175	-0.104824264	-0.022557676	-0.12310244	-0.040835852	0.082266588	8	0.055475081	0.816496581	0.816496581	0.816496581	0.816496581	0.816496581	0.816496581	14	62	0	A_24_P932319	A_24_P932319	A_24_P932319	chr3	33645277	33645337	-	CLASP2	cytoplasmic linker associated protein 2 [Source:HGNC Symbol;Acc:HGNC:17078]	ENSG00000163539	protein_coding	"5'UTR,exon,intron;intron;promoter"	multiple non-Ref	0	2272	3:33645277:33645337	ENSG00000163539	CLASP2	7.506670342	0.004678072	0.32729926	-1.877042056	-0.403930402	-2.204341316	-0.731229662	1.473111654	9.961963646	0.75021542	0.090073307	0.694798715	0.052156132	0.481484462	0.171600632	-6.940217767	-4.602832913	-6.958744544	-4.988277112	-6.019901866	-5.999648074	1.89290781	0.194962795

PKD1L3	-0.012763883	-0.051286307	-0.097007621	-0.038522424	-0.084243738	-0.045721314	8	0.123691785	0.816496581	0.816496581	0.816496581	0.816496581	0.816496581	0.816496581	14	64	0	P_226943	P_226943	P_226943	chr16	71937291	71937351	-	PKD1L3	polycystic kidney disease 1-like 3 [Source:HGNC Symbol;Acc:HGNC:21716]	ENSG00000277481	protein_coding	exon	coding exon	1	2274	16:71937291:71937351	ENSG00000277481	PKD1L3	3.685802546	0.014493143	-0.129851551	-0.52175397	-0.986893269	-0.391902419	-0.857041718	-0.465139299	9.961963646	0.899268377	0.613243844	0.347057853	0.703383185	0.411564613	0.651832917	-6.989167011	-6.040583291	-6.539834393	-7.066265956	-5.920191687	-6.954457411	0.394584923	0.759745305

SPEN	-0.111398563	-0.179681606	-0.156734036	-0.068283043	-0.045335473	0.02294757	8	0.057828296	0.816496581	0.816496581	0.816496581	0.816496581	0.816496581	0.816496581	14	66	0	P_200439	P_200439	P_200439	chr1	15920910	15920970	+	SPEN	spen family transcriptional repressor [Source:HGNC Symbol;Acc:HGNC:17575]	ENSG00000065526	protein_coding	exon	coding exon	1	2276	1:15920910:15920970	ENSG00000065526	SPEN	7.190592992	0.004892188	-1.950624371	-3.146282226	-2.744462959	-1.195657854	-0.793838587	0.401819267	9.961963646	0.079776323	0.010445635	0.020741732	0.259515044	0.44578665	0.696302221	-5.242392197	-2.623543478	-3.99150618	-6.419371282	-5.97201386	-6.983885224	3.909556724	0.043986515

SLC28A1	-0.10526839	-0.18565816	-0.066359315	-0.08038977	0.038909075	0.119298845	8	0.1492974	0.816496581	0.816496581	0.816496581	0.816496581	0.816496581	0.816496581	14	67	0	P_117395	P_117395	P_117395	chr15	84895384	84895444	+	SLC28A1	"solute carrier family 28 (concentrative nucleoside transporter), member 1 [Source:HGNC Symbol;Acc:HGNC:11001]"	ENSG00000156222	protein_coding	exon	coding exon	1	2277	15:84895384:84895444	ENSG00000156222	SLC28A1	4.568374948	0.020106539	-0.909232488	-1.603581385	-0.573163939	-0.694348896	0.33606855	1.030417446	9.961963646	0.384697254	0.140004595	0.579240894	0.503334072	0.74379033	0.327181996	-6.565122973	-4.982388472	-6.870630316	-6.892176197	-6.236962099	-6.520415585	0.897252741	0.476171016

OR4K2	0.087252948	0.344112168	0.315471906	0.25685922	0.228218957	-0.028640263	8	0.471442778	0.816496581	0.816496581	0.816496581	0.816496581	0.816496581	0.816496581	14	71	0	P_114254	P_114254	P_114254	chr14	19877112	19877172	+	OR4K2	"olfactory receptor, family 4, subfamily K, member 2 [Source:HGNC Symbol;Acc:HGNC:14728]"	ENSG00000165762	protein_coding	exon	coding exon	1	2281	14:19877112:19877172	ENSG00000165762	OR4K2	3.273204262	0.180692212	0.251394645	0.991461701	0.9089429	0.740067056	0.657548255	-0.082518801	9.961963646	0.806618754	0.344930659	0.384842844	0.476338561	0.525729419	0.935868667	-6.963982794	-5.688881605	-6.613803268	-6.858086411	-6.071725576	-7.067659832	0.476541846	0.705640875

CCDC191	0.381563139	0.277838004	0.535125581	-0.103725134	0.153562442	0.257287577	8	0.106587531	0.816496581	0.816496581	0.816496581	0.816496581	0.816496581	0.816496581	14	72	0	P_245634	P_245634	P_245634	chr3	113978220	113978280	-	KIAA1407	KIAA1407 [Source:HGNC Symbol;Acc:HGNC:29272]	ENSG00000163617	protein_coding	exon	coding exon	1	2282	3:113978220:113978280	ENSG00000163617	CCDC191	6.495907162	0.011330108	4.390308355	3.196835301	6.157215072	-1.193473054	1.766906717	2.960379771	9.961963646	0.001367805	0.009587675	0.000109053	0.260331276	0.107806837	0.014335055	-1.183277287	-2.542840004	1.404270217	-6.421865984	-4.859364049	-3.646137041	13.45267442	0.000774447

MCTP2	-0.405375612	0.229061688	-0.074295093	0.6344373	0.331080519	-0.303356781	8	0.072413373	0.816496581	0.816496581	0.816496581	0.816496581	0.816496581	0.816496581	14	74	0	A_14_P132246	A_14_P132246	A_14_P132246	chr15	94440192	94440252	+	MCTP2	"multiple C2 domains, transmembrane 2 [Source:HGNC Symbol;Acc:HGNC:25636]"	ENSG00000140563	protein_coding	3'UTR;exon	multiple Ref	1	2284	15:94440192:94440252	ENSG00000140563	MCTP2	7.129612872	0.006417658	-6.197477433	3.501948807	-1.135840812	9.699426239	5.06163662	-4.637789619	9.961963646	0.000103467	0.005740768	0.282610147	2.16E-06	0.000496625	0.000934489	1.492126895	-2.059028697	-6.384906573	5.404665883	0.231012663	-0.849621338	32.19510559	1.94E-05

CIP2A	-0.451221236	-0.111809239	-0.405589827	0.339411997	0.045631409	-0.293780588	8	0.05078758	0.816496581	0.816496581	0.816496581	0.816496581	0.816496581	0.816496581	14	75	0	P_245426	P_245426	P_245426	chr3	108576270	108576330	-	KIAA1524	KIAA1524 [Source:HGNC Symbol;Acc:HGNC:29302]	ENSG00000163507	protein_coding	3'UTR;exon	multiple Ref	1	2285	3:108576270:108576330	ENSG00000163507	CIP2A	7.702224503	0.004278065	-8.44911944	-2.093628447	-7.594671131	6.355490993	0.854448309	-5.501042684	9.961963646	7.46E-06	0.062850015	1.89E-05	8.44E-05	0.412932605	0.000264977	4.199506517	-4.283461269	3.220915093	1.598607684	-5.922385491	0.461899226	34.13895787	1.49E-05

AK3	0.273048932	-0.034368353	0.219101909	-0.307417285	-0.053947023	0.253470262	8	0.255464469	0.816496581	0.816496581	0.816496581	0.816496581	0.816496581	0.816496581	14	78	0	P_314321	P_314321	P_314321	chr9	4730804	4730864	+	AK3	adenylate kinase 3 [Source:HGNC Symbol;Acc:HGNC:17376]	ENSG00000147853	protein_coding	intron	intron antisense	0	2288	9:4730804:4730864	ENSG00000147853	AK3	5.509865001	0.054615705	1.430959494	-0.180113217	1.14824092	-1.611072711	-0.282718574	1.328354137	9.961963646	0.183054062	0.860674789	0.277694065	0.138357402	0.78317985	0.213688095	-5.982988733	-6.165014517	-6.371243094	-5.889138734	-6.254346959	-6.18441689	1.306425473	0.326020354

TMEM130	0.119326813	0.108604453	0.052177812	-0.01072236	-0.067149001	-0.056426641	8	0.208427737	0.816496581	0.816496581	0.816496581	0.816496581	0.816496581	0.816496581	14	80	0	P_145299	P_145299	P_145299	chr7	98847901	98847961	-	TMEM130	transmembrane protein 130 [Source:HGNC Symbol;Acc:HGNC:25429]	ENSG00000166448	protein_coding	3'UTR	3'UTR	1	2290	7:98847901:98847961	ENSG00000166448	TMEM130	4.16477861	0.037093076	0.758817335	0.690632221	0.331806636	-0.068185114	-0.427010699	-0.358825585	9.961963646	0.465536534	0.505569198	0.746910411	0.94698744	0.67845314	0.727213767	-6.692965512	-5.936718666	-6.987328499	-7.147071163	-6.200608079	-7.00149265	0.2464359	0.862021792

CADPS	0.157901101	0.057505124	0.09783854	-0.100395977	-0.060062561	0.040333416	8	0.112335949	0.816496581	0.816496581	0.816496581	0.816496581	0.816496581	0.816496581	14	81	0	P_135250	P_135250	P_135250	chr3	62395023	62395083	+	CADPS	Ca++-dependent secretion activator [Source:HGNC Symbol;Acc:HGNC:1426]	ENSG00000163618	protein_coding	3'ext	3'extended antisense	0	2291	3:62395023:62395083	ENSG00000163618	CADPS	3.817047405	0.012340722	1.740846568	0.63398923	1.078661805	-1.106857338	-0.662184763	0.444672576	9.961963646	0.112446735	0.54037129	0.30616304	0.2943663	0.522875659	0.666061482	-5.556234307	-5.974531867	-6.446419056	-6.517962588	-6.068611812	-6.964421477	1.076225989	0.402832471

SLC9A2	-0.639566144	-0.102617635	-0.407452984	0.53694851	0.232113161	-0.304835349	8	0.109847928	0.816496581	0.816496581	0.816496581	0.816496581	0.816496581	0.816496581	14	84	0	P_236059	P_236059	P_236059	chr2	102709027	102709087	-	SLC9A2	"solute carrier family 9, subfamily A (NHE2, cation proton antiporter 2), member 2 [Source:HGNC Symbol;Acc:HGNC:11072]"	ENSG00000115616	protein_coding	3'UTR	3'UTR antisense	0	2294	2:102709027:102709087	ENSG00000115616	SLC9A2	7.328389935	0.011896795	-7.181515463	-1.152265697	-4.575179483	6.029249766	2.60633598	-3.422913786	9.961963646	3.05E-05	0.276112962	0.001028197	0.000129071	0.026280984	0.00655119	2.754447432	-5.526813494	-0.928530494	1.153292137	-3.569966742	-2.855066938	21.44922111	0.000114588

ZNF555	0.374508729	0.270249059	0.157935265	-0.104259671	-0.216573465	-0.112313794	8	0.205260482	0.816496581	0.816496581	0.816496581	0.816496581	0.816496581	0.816496581	14	85	0	P_124107	P_124107	P_124107	chr19	2853892	2853952	+	ZNF555	zinc finger protein 555 [Source:HGNC Symbol;Acc:HGNC:28382]	ENSG00000186300	protein_coding	exon	coding exon	1	2295	19:2853892:2853952	ENSG00000186300	ZNF555	4.12039126	0.036040869	2.416072416	1.743460821	1.018889565	-0.672611596	-1.397182852	-0.724571256	9.961963646	0.036391718	0.1119732	0.332361599	0.516491691	0.192698408	0.48538459	-4.496752261	-4.792031274	-6.508024876	-6.907693383	-5.351496234	-6.791921	2.14078788	0.158724877

BCAP29	-0.229485271	0.071700643	-0.166627289	0.301185914	0.062857981	-0.238327933	8	0.059426629	0.816496581	0.816496581	0.816496581	0.816496581	0.816496581	0.816496581	14	86	0	A_32_P150735	A_32_P150735	A_32_P150735	chr7	107630574	107630634	+	BCAP29	B-cell receptor-associated protein 29 [Source:HGNC Symbol;Acc:HGNC:24131]	ENSG00000075790	protein_coding	3'ext	3'extended sense	0	2296	7:107630574:107630634	ENSG00000075790	BCAP29	7.64020008	0.005042691	-3.957940747	1.236623584	-2.873826874	5.194564331	1.084113873	-4.110450458	9.961963646	0.002715502	0.244587228	0.016620558	0.00040948	0.303854067	0.002125995	-1.889501187	-5.434451694	-3.771346082	-0.054839027	-5.70690273	-1.70073661	11.75133637	0.001304488

NOLC1	-0.274004799	0.029846937	-0.186691058	0.303851736	0.087313741	-0.216537995	8	0.064469011	0.816496581	0.816496581	0.816496581	0.816496581	0.816496581	0.816496581	14	87	0	P_209623	P_209623	P_209623	chr10	102159465	102159525	-	NOLC1	nucleolar and coiled-body phosphoprotein 1 [Source:HGNC Symbol;Acc:HGNC:15608]	ENSG00000166197	protein_coding	3'UTR;exon;promoter	multiple non-Ref	0	2297	10:102159465:102159525	ENSG00000166197	NOLC1	11.19583414	0.005544382	-4.506892745	0.490929149	-3.070736638	4.997821894	1.436156107	-3.561665787	9.961963646	0.001141869	0.634110909	0.011876663	0.000545316	0.181607497	0.005197773	-0.996782454	-6.056608844	-3.434830376	-0.354143274	-5.302938076	-2.619094746	11.61812473	0.001362094

CELF5	0.147443163	0.006734165	-0.16365425	-0.140708998	-0.311097413	-0.170388415	8	0.175409444	0.816496581	0.816496581	0.816496581	0.816496581	0.816496581	0.816496581	14	89	0	P_314836	P_314836	P_314836	chr19	3296955	3297015	+	CELF5	"CUGBP, Elav-like family member 5 [Source:HGNC Symbol;Acc:HGNC:14058]"	ENSG00000161082	protein_coding	3'UTR	3'UTR	1	2299	19:3296955:3297015	ENSG00000161082	CELF5	4.39613601	0.026915446	1.100701303	0.050272281	-1.22172126	-1.050429022	-2.322422562	-1.27199354	9.961963646	0.29691148	0.960899107	0.249935233	0.318334903	0.042689171	0.232265562	-6.374748413	-6.180755278	-6.28798481	-6.577526185	-4.022875917	-6.25278058	1.803614792	0.21031319

CS	-0.185207084	-0.15674793	-0.064934558	0.028459155	0.120272526	0.091813371	8	0.079899455	0.816496581	0.816496581	0.816496581	0.816496581	0.816496581	0.816496581	14	91	0	P_111915	P_111915	P_111915	chr12	56273257	56273317	-	CS	citrate synthase [Source:HGNC Symbol;Acc:HGNC:2422]	ENSG00000062485	protein_coding	exon	non-coding exon	1	2301	12:56273257:56273317	ENSG00000062485	CS	7.968936714	0.007333322	-2.648822411	-2.24180101	-0.928690789	0.407021401	1.720131622	1.313110221	9.961963646	0.024435708	0.048951761	0.375003858	0.692599876	0.116263818	0.218586807	-4.107607873	-4.057856126	-6.595545153	-7.059765206	-4.925164883	-6.203113461	2.958862698	0.084441629

FCMR	-0.038254213	0.068957868	-0.035954508	0.107212082	0.002299705	-0.104912376	8	0.110074054	0.816496581	0.816496581	0.816496581	0.816496581	0.816496581	0.816496581	14	93	0	A_23_P138125	A_23_P138125	A_23_P138125	chr1	206905018	206905078	-	FAIM3	Fas apoptotic inhibitory molecule 3 [Source:HGNC Symbol;Acc:HGNC:14315]	ENSG00000162894	protein_coding	3'UTR;exon	multiple Ref	1	2303	1:206905018:206905078	ENSG00000162894	FCMR	5.222581661	0.011936731	-0.428827072	0.773012907	-0.403047536	1.201839979	0.025779536	-1.176060442	9.961963646	0.677174032	0.457463964	0.695427306	0.257216524	0.979942283	0.266910079	-6.898933955	-5.876542136	-6.95912732	-6.412293809	-6.296331786	-6.364169537	0.628681835	0.61292222

MAPK8IP1	0.017746144	-0.162172561	-0.10248563	-0.179918705	-0.120231774	0.059686931	8	0.070566657	0.816496581	0.816496581	0.816496581	0.816496581	0.816496581	0.816496581	14	94	0	P_108384	P_108384	P_108384	chr11	45906405	45906465	+	MAPK8IP1	mitogen-activated protein kinase 8 interacting protein 1 [Source:HGNC Symbol;Acc:HGNC:6882]	ENSG00000121653	protein_coding	3'UTR	3'UTR	1	2304	11:45906405:45906465	ENSG00000121653	MAPK8IP1	8.775148366	0.006205617	0.275903462	-2.521334831	-1.593368113	-2.797238293	-1.869271575	0.927966718	9.961963646	0.788261011	0.03039885	0.142278343	0.018948786	0.091230102	0.375361414	-6.956979937	-3.621302292	-5.813193338	-3.998425142	-4.712354243	-6.620312042	3.525319084	0.056709299

WIF1	-0.07791377	-0.079134593	0.027574909	-0.001220822	0.10548868	0.106709502	8	0.194242641	0.816496581	0.816496581	0.816496581	0.816496581	0.816496581	0.816496581	14	95	0	P_215999	P_215999	P_215999	chr12	65055117	65055177	-	WIF1	WNT inhibitory factor 1 [Source:HGNC Symbol;Acc:HGNC:18081]	ENSG00000156076	protein_coding	exon	coding exon	1	2305	12:65055117:65055177	ENSG00000156076	WIF1	5.81839992	0.032506095	-0.529270212	-0.537563287	0.187317056	-0.008293075	0.716587268	0.724880343	9.961963646	0.608209188	0.602679011	0.855172146	0.993546884	0.490087163	0.485203107	-6.847624232	-6.032006673	-7.027992778	-7.149575453	-6.030595321	-6.791688597	0.273867754	0.842935077

CAPN13	-0.461981469	0.539330087	-0.59381983	1.001311556	-0.131838362	-1.133149918	8	0.440101474	0.816496581	0.816496581	0.816496581	0.816496581	0.816496581	0.816496581	14	96	0	P_234233	P_234233	P_234233	chr2	30751101	30751161	-	CAPN13	calpain 13 [Source:HGNC Symbol;Acc:HGNC:16663]	ENSG00000162949	protein_coding	exon	coding exon	1	2306	2:30751101:30751161	ENSG00000162949	CAPN13	6.319041694	0.157749759	-1.424576686	1.663090666	-1.831116491	3.087667352	-0.406539805	-3.494207157	9.961963646	0.184844311	0.127388453	0.097112645	0.011539439	0.692942271	0.005815349	-5.991231386	-4.902454645	-5.470680012	-3.501189079	-6.209523136	-2.733681787	5.009454141	0.022607936

TMEM187	-0.145971295	0.243509198	-0.070005026	0.389480493	0.075966269	-0.313514224	8	0.08173916	0.816496581	0.816496581	0.816496581	0.816496581	0.816496581	0.816496581	14	97	0	P_151944	P_151944	P_151944	chrX	153982749	153982809	-	TMEM187	transmembrane protein 187 [Source:HGNC Symbol;Acc:HGNC:13705]	ENSG00000177854	protein_coding	exon	exon antisense	0	2307	X:153982749:153982809	ENSG00000177854	TMEM187	7.909478174	0.007572124	-2.054490901	3.427300068	-0.98529433	5.481790969	1.069196571	-4.412594398	9.961963646	0.067108959	0.006503251	0.347804642	0.000272214	0.310203487	0.00132121	-5.081090453	-2.176760739	-6.541402221	0.372174182	-5.722147867	-1.208782089	11.2670544	0.001529169

CCDC125	-0.250815615	0.041825836	-0.134786676	0.292641451	0.116028939	-0.176612512	8	0.123528833	0.816496581	0.816496581	0.816496581	0.816496581	0.816496581	0.816496581	14	98	0	A_23_P432591	A_23_P432591	A_23_P432591	chr5	69282938	69282998	-	CCDC125	coiled-coil domain containing 125 [Source:HGNC Symbol;Acc:HGNC:28924]	ENSG00000183323	protein_coding	3'UTR;exon	multiple Ref	1	2308	5:69282938:69282998	ENSG00000183323	CCDC125	9.931236197	0.014460792	-2.554489704	0.425984914	-1.372766109	2.980474618	1.181723595	-1.798751023	9.961963646	0.028721573	0.679175982	0.199936022	0.013851675	0.264756066	0.102374918	-4.266203398	-6.087322026	-6.10520276	-3.685028644	-5.603157092	-5.542411088	3.684428738	0.050970609

RBBP5	0.074103586	0.00706694	-0.012202982	-0.067036645	-0.086306568	-0.019269922	8	0.089295818	0.816496581	0.816496581	0.816496581	0.816496581	0.816496581	0.816496581	14	100	0	P_104560	P_104560	P_104560	chr1	205086851	205086911	-	RBBP5	retinoblastoma binding protein 5 [Source:HGNC Symbol;Acc:HGNC:9888]	ENSG00000117222	protein_coding	3'UTR	3'UTR	1	2310	1:205086851:205086911	ENSG00000117222	RBBP5	7.380336029	0.008610034	0.978098082	0.093277009	-0.16106796	-0.884821073	-1.139166042	-0.254344969	9.961963646	0.351180285	0.927532783	0.875259432	0.397106746	0.281285194	0.804402095	-6.500071889	-6.1775028	-7.032971478	-6.737554674	-5.649227555	-7.036152471	0.522769719	0.676337141

INO80	-0.272947501	-0.189378192	-0.334137487	0.083569309	-0.061189986	-0.144759295	8	0.064062807	0.816496581	0.816496581	0.816496581	0.816496581	0.816496581	0.816496581	14	102	0	P_222858	P_222858	P_222858	chr15	41049971	41050031	-	INO80	INO80 complex subunit [Source:HGNC Symbol;Acc:HGNC:26956]	ENSG00000128908	protein_coding	3'UTR;5'UTR;exon	multiple Ref	1	2312	15:41049971:41050031	ENSG00000128908	INO80	7.388611698	0.005502455	-4.506574146	-3.126780279	-5.516868098	1.379793867	-1.010293953	-2.39008782	9.961963646	0.001142429	0.010797273	0.000259181	0.197829676	0.336263381	0.038041577	-0.997289678	-2.654704389	0.503447081	-6.197393287	-5.780703854	-4.609231259	11.52647397	0.001403503

SVBP	0.352450576	0.184313065	0.306650032	-0.168137511	-0.045800544	0.122336967	8	0.086206416	0.816496581	0.816496581	0.816496581	0.816496581	0.816496581	0.816496581	14	103	0	P_101523	P_101523	P_101523	chr1	42807306	42807366	+	CCDC23	coiled-coil domain containing 23 [Source:HGNC Symbol;Acc:HGNC:29204]	ENSG00000177868	protein_coding	3'UTR	3'UTR antisense	0	2313	1:42807306:42807366	ENSG00000177868	SVBP	7.806663951	0.008174621	4.774303773	2.496709104	4.153888529	-2.277594669	-0.620415244	1.657179425	9.961963646	0.000760197	0.031707054	0.001983952	0.04606839	0.548913625	0.128594455	-0.575887604	-3.660192552	-1.608375991	-4.869275722	-6.095938487	-5.747568371	9.100153163	0.003335992

NEDD1	-0.133037375	0.081678237	-0.009666389	0.214715613	0.123370987	-0.091344626	8	0.044954616	0.816496581	0.816496581	0.816496581	0.816496581	0.816496581	0.816496581	14	104	0	A_24_P198355	A_24_P198355	A_24_P198355	chr12	96951459	96951969	+	NEDD1	"neural precursor cell expressed, developmentally down-regulated 1 [Source:HGNC Symbol;Acc:HGNC:7723]"	ENSG00000139350	protein_coding	"exon,intron"	multiple non-Ref	0	2314	12:96951459:96951969	ENSG00000139350	NEDD1	7.285758054	0.003829591	-2.632952996	1.616500314	-0.19130825	4.249453311	2.441644746	-1.807808565	9.961963646	0.025109333	0.137174774	0.852126962	0.001705488	0.034836706	0.100876429	-4.134358659	-4.965173835	-7.027170495	-1.541227081	-3.834004582	-5.528987942	6.144961318	0.012317225

USH2A	-0.033497044	-0.011651249	0.126716995	0.021845795	0.160214039	0.138368244	8	0.04923413	0.816496581	0.816496581	0.816496581	0.816496581	0.816496581	0.816496581	14	105	0	P_206222	P_206222	P_206222	chr1	216323473	216323533	-	USH2A	"Usher syndrome 2A (autosomal recessive, mild) [Source:HGNC Symbol;Acc:HGNC:12601]"	ENSG00000042781	protein_coding	exon	coding exon	1	2315	1:216323473:216323533	ENSG00000042781	USH2A	3.625401415	0.004153288	-0.636584558	-0.221422682	2.408155259	0.415161876	3.044739817	2.629577941	9.961963646	0.538746796	0.829236064	0.036886826	0.68682336	0.012414146	0.025254968	-6.781639254	-6.156307295	-4.55619445	-7.056166479	-2.858287721	-4.208858305	3.76849329	0.048218271

CHMP4B	0.209292638	-0.040605882	0.013283168	-0.24989852	-0.19600947	0.05388905	8	0.134804216	0.816496581	0.816496581	0.816496581	0.816496581	0.816496581	0.816496581	14	106	0	P_131004	P_131004	P_131004	chr20	33853818	33853878	+	CHMP4B	charged multivesicular body protein 4B [Source:HGNC Symbol;Acc:HGNC:16171]	ENSG00000101421	protein_coding	3'UTR	3'UTR	1	2316	20:33853818:33853878	ENSG00000101421	CHMP4B	11.51167336	0.016799933	1.977633503	-0.383690292	0.125514387	-2.361323795	-1.852119116	0.509204679	9.961963646	0.076281346	0.709269568	0.902613195	0.039953444	0.093832533	0.62169695	-5.200789065	-6.105069756	-7.038524103	-4.731808151	-4.73726085	-6.931552685	2.223248511	0.148457693

WDR3	-0.179181835	-0.036969733	-0.123553711	0.142212103	0.055628124	-0.086583978	8	0.068660661	0.816496581	0.816496581	0.816496581	0.816496581	0.816496581	0.816496581	14	107	0	P_203683	P_203683	P_203683	chr1	117952601	117952661	+	WDR3	WD repeat domain 3 [Source:HGNC Symbol;Acc:HGNC:12755]	ENSG00000065183	protein_coding	exon	coding exon	1	2317	1:117952601:117952661	ENSG00000065183	WDR3	9.465690642	0.005992513	-2.834882873	-0.58490785	-1.954775711	2.249975023	0.880107163	-1.36986786	9.961963646	0.017765949	0.571618785	0.079229536	0.048278281	0.399534914	0.200810175	-3.792309387	-6.00488726	-5.283181807	-4.914291511	-5.900430333	-6.132745053	3.318890059	0.065341486

AGAP2	-0.10376328	-0.00788545	0.120311313	0.095877829	0.224074592	0.128196763	8	0.147809577	0.816496581	0.816496581	0.816496581	0.816496581	0.816496581	0.816496581	14	111	0	P_112054	P_112054	P_112054	chr12	57725276	57725336	-	AGAP2	"ArfGAP with GTPase domain, ankyrin repeat and PH domain 2 [Source:HGNC Symbol;Acc:HGNC:16921]"	ENSG00000135439	protein_coding	3'UTR	3'UTR	1	2321	12:57725276:57725336	ENSG00000135439	AGAP2	4.372307819	0.019751555	-0.904250322	-0.068718153	1.048458989	0.835532168	1.952709311	1.117177143	9.961963646	0.387207456	0.946573726	0.319197745	0.423004683	0.079501268	0.290137606	-6.569674379	-6.179598637	-6.477899265	-6.780698252	-4.589742409	-6.429273128	1.280154918	0.333905052

ITGA3	0.263528014	0.13344858	0.255935946	-0.130079433	-0.007592068	0.122487366	8	0.086619744	0.816496581	0.816496581	0.816496581	0.816496581	0.816496581	0.816496581	14	112	0	P_121979	P_121979	P_121979	chr17	50070870	50070930	+	ITGA3	"integrin, alpha 3 (antigen CD49C, alpha 3 subunit of VLA-3 receptor) [Source:HGNC Symbol;Acc:HGNC:6139]"	ENSG00000005884	protein_coding	3'UTR;exon	multiple Ref	1	2322	17:50070870:50070930	ENSG00000005884	ITGA3	6.681867726	0.008231986	3.557296819	1.801388033	3.454813453	-1.755908786	-0.102483366	1.65342542	9.961963646	0.00523563	0.101936551	0.006210708	0.109743244	0.920406594	0.129365649	-2.56114996	-4.710808236	-2.778749624	-5.681936851	-6.291101977	-5.752882915	5.610438271	0.016247145

NEAT1	-0.001565075	0.026600649	0.0462535	0.028165724	0.047818575	0.019652851	8	0.129015181	0.816496581	0.816496581	0.816496581	0.816496581	0.816496581	0.816496581	14	113	0	P_109111	P_109111	P_109111	chr11	65424350	65424410	-	NEAT1	nuclear paraspeckle assembly transcript 1 (non-protein coding) [Source:HGNC Symbol;Acc:HGNC:30815]	ENSG00000245532	lincRNA	exon;promoter	multiple non-Ref	0	2323	11:65424350:65424410	ENSG00000245532	NEAT1	7.006223918	0.01557346	-0.015359891	0.261062994	0.453939191	0.276422885	0.469299082	0.192876197	9.961963646	0.988048328	0.799361474	0.659601604	0.787873373	0.648958612	0.85093135	-6.998231576	-6.146286088	-6.935745255	-7.107987604	-6.180860259	-7.051093485	0.101383845	0.957373862

FAM167A-AS1	0.140591725	0.054775487	-0.140207415	-0.085816238	-0.280799141	-0.194982902	8	0.224872741	0.816496581	0.816496581	0.816496581	0.816496581	0.816496581	0.816496581	14	115	0	P_263777	P_263777	P_263777	chr8	11433985	11434045	+	C8orf12	chromosome 8 open reading frame 12 [Source:HGNC Symbol;Acc:HGNC:15548]	ENSG00000184608	processed_transcript	exon	non-coding exon	1	2325	8:11433985:11434045	ENSG00000184608	FAM167A-AS1	4.412513225	0.042815344	0.832157766	0.324214294	-0.829883047	-0.507943472	-1.662040813	-1.154097341	9.961963646	0.424818777	0.75248052	0.426044638	0.62254972	0.127601898	0.275395767	-6.633133777	-6.126976276	-6.683534582	-7.010324931	-5.005586242	-6.388751339	0.973105706	0.443485366

CLOCK	0.214424476	-0.071028607	0.056404121	-0.285453082	-0.158020355	0.127432728	8	0.087998873	0.816496581	0.816496581	0.816496581	0.816496581	0.816496581	0.816496581	14	116	0	A_24_P166168	A_24_P166168	A_24_P166168	chr4	55429438	55429498	-	CLOCK	clock circadian regulator [Source:HGNC Symbol;Acc:HGNC:2082]	ENSG00000134852	protein_coding	3'UTR	3'UTR	1	2326	4:55429438:55429498	ENSG00000134852	CLOCK	7.274616901	0.008425379	2.861048805	-0.947729076	0.752595721	-3.808777882	-2.108453084	1.700324798	9.961963646	0.016987914	0.36568982	0.469103267	0.003459734	0.061305066	0.120023085	-3.747776261	-5.729571333	-6.746340026	-2.272293356	-4.355102032	-5.686006116	5.248943127	0.019768361

CCDC28A	0.047451564	-0.003767276	-0.253782214	-0.05121884	-0.301233777	-0.250014937	8	0.09865661	0.816496581	0.816496581	0.816496581	0.816496581	0.816496581	0.816496581	14	117	0	P_143711	P_143711	P_143711	chr6	138785321	138785381	+	CCDC28A	coiled-coil domain containing 28A [Source:HGNC Symbol;Acc:HGNC:21098]	ENSG00000024862	protein_coding	exon	coding exon	1	2327	6:138785321:138785381	ENSG00000024862	CCDC28A	7.310877761	0.010022915	0.58049586	-0.04608675	-3.104629479	-0.62658261	-3.685125339	-3.058542729	9.961963646	0.574475809	0.964151888	0.011211385	0.545022858	0.004237648	0.012125753	-6.817541966	-6.180967842	-3.376836521	-6.939057813	-1.82605261	-3.478155892	5.550876966	0.016772241

DDX27	-0.027588657	-0.064771781	-0.010910363	-0.037183124	0.016678294	0.053861418	8	0.038251651	0.816496581	0.816496581	0.816496581	0.816496581	0.816496581	0.816496581	14	118	0	P_131516	P_131516	P_131516	chr20	49234847	49234907	+	DDX27	DEAD (Asp-Glu-Ala-Asp) box polypeptide 27 [Source:HGNC Symbol;Acc:HGNC:15837]	ENSG00000124228	protein_coding	exon	non-coding exon	1	2328	20:49234847:49234907	ENSG00000124228	DDX27	9.889492686	0.003381704	-0.581043125	-1.364154771	-0.229782529	-0.783111646	0.351260596	1.134372242	9.961963646	0.574120999	0.202542758	0.822910112	0.451776767	0.732708358	0.28319686	-6.817206539	-5.286070659	-7.018362686	-6.824217368	-6.231475442	-6.410526701	0.712462934	0.566586798

ZNF304	0.067416171	0.061255617	-0.001454423	-0.006160553	-0.068870594	-0.062710041	8	0.093035794	0.816496581	0.816496581	0.816496581	0.816496581	0.816496581	0.816496581	14	121	0	A_24_P228266	A_24_P228266	A_24_P228266	chr19	57359335	57359395	+	ZNF304	zinc finger protein 304 [Source:HGNC Symbol;Acc:HGNC:13505]	ENSG00000131845	protein_coding	3'UTR	3'UTR	1	2331	19:57359335:57359395	ENSG00000131845	ZNF304	7.210030106	0.00915765	0.862815006	0.783970155	-0.018614203	-0.078844851	-0.881429209	-0.802584358	9.961963646	0.40853045	0.45129543	0.985516411	0.938717305	0.398852879	0.440941739	-6.606700895	-5.868085768	-7.046931385	-7.146214529	-5.899284116	-6.730467795	0.46444684	0.713460253

FOXN1	-0.041996338	0.199311724	0.125708036	0.241308062	0.167704374	-0.073603688	8	0.223842605	0.816496581	0.816496581	0.816496581	0.816496581	0.816496581	0.816496581	14	122	0	P_120897	P_120897	P_120897	chr17	28534463	28534523	+	FOXN1	forkhead box N1 [Source:HGNC Symbol;Acc:HGNC:12765]	ENSG00000109101	protein_coding	exon	coding exon	1	2332	17:28534463:28534523	ENSG00000109101	FOXN1	4.879473004	0.042444142	-0.249659542	1.184866977	0.747308276	1.434526519	0.996967817	-0.437558702	9.961963646	0.807923226	0.26356645	0.472148163	0.182060062	0.342379646	0.67104021	-6.964454214	-5.491690785	-6.750437872	-6.127211516	-5.793581477	-6.967783967	0.877996347	0.484864223

WDR90	-0.203871016	-0.157666162	-0.1302636	0.046204855	0.073607417	0.027402562	8	0.108426945	0.816496581	0.816496581	0.816496581	0.816496581	0.816496581	0.816496581	14	124	0	A_24_P275723	A_24_P275723	A_24_P275723	chr16	663046	663106	+	WDR90	WD repeat domain 90 [Source:HGNC Symbol;Acc:HGNC:26960]	ENSG00000161996	protein_coding	exon	non-coding exon	1	2334	16:663046:663106	ENSG00000161996	WDR90	10.08007004	0.011647716	-2.313559787	-1.789219957	-1.478251452	0.524339829	0.835308334	0.310968505	9.961963646	0.043337544	0.103973796	0.170247276	0.611509339	0.423124855	0.762233882	-4.665414904	-4.727977981	-5.969021733	-7.001311432	-5.938399211	-7.018806764	1.968077997	0.183041354

HSD11B1	0.069365618	0.093472561	0.128414058	0.024106942	0.05904844	0.034941497	8	0.150852845	0.816496581	0.816496581	0.816496581	0.816496581	0.816496581	0.816496581	14	125	0	A_23_P63209	A_23_P63209	A_23_P63209	chr1	209734555	209734615	+	HSD11B1	hydroxysteroid (11-beta) dehydrogenase 1 [Source:HGNC Symbol;Acc:HGNC:5208]	ENSG00000117594	protein_coding	3'UTR	3'UTR	1	2335	1:209734555:209734615	ENSG00000117594	HSD11B1	4.221746372	0.020481459	0.593621254	0.799925094	1.098949539	0.20630384	0.505328285	0.299024445	9.961963646	0.565999383	0.442411195	0.297638839	0.840708876	0.624319855	0.771066402	-6.809415973	-5.855585689	-6.424877525	-7.126387606	-6.162632936	-7.022749921	0.431215001	0.735249742

EFCAB13	0.069583725	-0.053944852	0.024324936	-0.123528577	-0.045258789	0.078269788	8	0.06619748	0.816496581	0.816496581	0.816496581	0.816496581	0.816496581	0.816496581	14	126	0	P_229034	P_229034	P_229034	chr17	47403941	47404001	+	EFCAB13	EF-hand calcium binding domain 13 [Source:HGNC Symbol;Acc:HGNC:26864]	ENSG00000178852	protein_coding	exon	coding exon	1	2336	17:47403941:47404001	ENSG00000178852	EFCAB13	4.329571463	0.005725754	1.126255563	-0.873130745	0.393714108	-1.999386308	-0.732541455	1.266844853	9.961963646	0.286456789	0.40314743	0.702087329	0.073571789	0.480718416	0.234026966	-6.347159605	-5.795440989	-6.963124312	-5.313675706	-6.0189344	-6.258920372	1.387479694	0.3029802

MAX	-0.003027505	0.043945642	0.030580411	0.046973147	0.033607915	-0.013365231	8	0.065845186	0.816496581	0.816496581	0.816496581	0.816496581	0.816496581	0.816496581	14	127	0	A_16_P20074925	A_16_P20074925	A_16_P20074925	chr14	65053660	65053720	+	MAX;FNTB	"MYC associated factor X [Source:HGNC Symbol;Acc:HGNC:6913];farnesyltransferase, CAAX box, beta [Source:HGNC Symbol;Acc:HGNC:3785]"	ENSG00000125952	protein_coding	intron	multiple non-Ref	0	2337	14:65053660:65053720	ENSG00000125952	MAX	7.870311303	0.005688398	-0.049162674	0.71361908	0.496585409	0.762781755	0.545748084	-0.217033671	9.961963646	0.961761366	0.491842634	0.630255808	0.463272919	0.597246824	0.832562424	-6.997041558	-5.920538009	-6.914103367	-6.840425761	-6.140666827	-7.045706731	0.280841137	0.83807747

C19orf12	-0.079683253	0.039579842	-0.16165244	0.119263095	-0.081969187	-0.201232282	8	0.050968635	0.816496581	0.816496581	0.816496581	0.816496581	0.816496581	0.816496581	14	128	0	P_125331	P_125331	P_125331	chr19	29702868	29702928	-	C19orf12	chromosome 19 open reading frame 12 [Source:HGNC Symbol;Acc:HGNC:25443]	ENSG00000131943	protein_coding	3'UTR;exon	multiple Ref	1	2338	19:29702868:29702928	ENSG00000131943	C19orf12	9.40307838	0.00429286	-1.489495852	0.739854468	-3.021722006	2.22935032	-1.532226154	-3.761576474	9.961963646	0.167318708	0.476461939	0.012910705	0.04999511	0.156585475	0.003737442	-5.906286562	-5.901490315	-3.518681749	-4.947791932	-5.179650541	-2.28140687	5.560660635	0.016684611

GREB1L	0.051430494	-0.03010967	0.08574335	-0.081540164	0.034312856	0.11585302	8	0.062334058	0.816496581	0.816496581	0.816496581	0.816496581	0.816496581	0.816496581	14	129	0	P_230819	P_230819	P_230819	chr18	21516694	21516754	+	GREB1L	growth regulation by estrogen in breast cancer-like [Source:HGNC Symbol;Acc:HGNC:31042]	ENSG00000141449	protein_coding	exon	coding exon	1	2339	18:21516694:21516754	ENSG00000141449	GREB1L	5.348159583	0.00532698	0.863029775	-0.505255531	1.438816903	-1.368285306	0.575787128	1.944072434	9.961963646	0.408417876	0.624369136	0.180870619	0.201288854	0.577533594	0.080646505	-6.606512812	-6.049275693	-6.020705008	-6.211916481	-6.123314579	-5.323161215	1.508908458	0.271824922

PKD2L2	0.137605353	0.021936827	-0.030077486	-0.115668526	-0.167682839	-0.052014313	8	0.053777966	0.816496581	0.816496581	0.816496581	0.816496581	0.816496581	0.816496581	14	130	0	A_32_P235321	A_32_P235321	A_32_P235321	chr5	137894518	137894578	+	PKD2L2	polycystic kidney disease 2-like 2 [Source:HGNC Symbol;Acc:HGNC:9012]	ENSG00000078795	protein_coding	3'UTR;exon	multiple Ref	1	2340	5:137894518:137894578	ENSG00000078795	PKD2L2	3.83963695	0.004529174	2.504214226	0.399217865	-0.547365822	-2.10499636	-3.051580048	-0.946583688	9.961963646	0.031302567	0.698156784	0.596176197	0.061662034	0.012270365	0.36624542	-4.350271888	-6.09876229	-6.885930977	-5.147509091	-2.84715689	-6.602802929	3.561540713	0.055338048

FAM83D	-0.500240336	-0.128861287	-0.441630084	0.371379049	0.058610253	-0.312768797	8	0.053156203	0.816496581	0.816496581	0.816496581	0.816496581	0.816496581	0.816496581	14	131	0	P_311605	P_311605	P_311605	chr20	38926703	38926763	+	FAM83D	"family with sequence similarity 83, member D [Source:HGNC Symbol;Acc:HGNC:16122]"	ENSG00000101447	protein_coding	exon	coding exon	1	2341	20:38926703:38926763	ENSG00000101447	FAM83D	9.239011823	0.00447578	-9.157774746	-2.359031362	-8.084811509	6.798743384	1.072963237	-5.725780148	9.961963646	3.64E-06	0.040109798	1.10E-05	4.84E-05	0.308590749	0.000194202	4.928845037	-3.876224717	3.780752925	2.180244249	-5.718314059	0.785757735	39.15879407	8.01E-06

HSDL1	0.132768126	0.187426069	0.111033827	0.054657943	-0.021734299	-0.076392242	8	0.047794911	0.816496581	0.816496581	0.816496581	0.816496581	0.816496581	0.816496581	14	132	0	A_23_P206324	A_23_P206324	A_23_P206324	chr16	84122936	84122996	-	HSDL1	hydroxysteroid dehydrogenase like 1 [Source:HGNC Symbol;Acc:HGNC:16475]	ENSG00000103160	protein_coding	3'UTR;exon	multiple Ref	1	2342	16:84122936:84122996	ENSG00000103160	HSDL1	7.430668262	0.004041144	2.557921846	3.610966362	2.139187025	1.053044516	-0.41873482	-1.471779337	9.961963646	0.028553273	0.004789761	0.058216926	0.31719209	0.68429462	0.171952958	-4.260451939	-1.888011453	-4.993964924	-6.574820033	-6.20426307	-6.001405696	4.601423231	0.028655876

TSPAN16	0.045334123	-0.386663041	-0.016892035	-0.431997165	-0.062226159	0.369771006	8	0.316479193	0.816496581	0.816496581	0.816496581	0.816496581	0.816496581	0.816496581	14	135	0	P_232353	P_232353	P_232353	chr19	11298886	11298946	+	TSPAN16	tetraspanin 16 [Source:HGNC Symbol;Acc:HGNC:30725]	ENSG00000130167	protein_coding	exon	coding exon	1	2345	19:11298886:11298946	ENSG00000130167	TSPAN16	4.396150326	0.082639886	0.193141676	-1.647340731	-0.071966893	-1.840482408	-0.26510857	1.575373838	9.961963646	0.850728953	0.130624452	0.944052615	0.095637039	0.796330667	0.14636407	-6.978042304	-4.923766839	-7.04429291	-5.556619152	-6.259438523	-5.861797588	1.449279201	0.286645256

ITFG1	0.03224412	-0.044181187	-0.031130838	-0.076425307	-0.063374958	0.013050349	8	0.049164277	0.816496581	0.816496581	0.816496581	0.816496581	0.816496581	0.816496581	14	136	0	P_226216	P_226216	P_226216	chr16	47218867	47218927	-	ITFG1	integrin alpha FG-GAP repeat containing 1 [Source:HGNC Symbol;Acc:HGNC:30697]	ENSG00000129636	protein_coding	exon	coding exon	1	2346	16:47218867:47218927	ENSG00000129636	ITFG1	9.286587065	0.004147768	0.613181335	-0.840186641	-0.592010224	-1.453367976	-1.205191559	0.248176417	9.961963646	0.553497371	0.420511036	0.567036046	0.176886087	0.255977242	0.809038747	-6.796992831	-5.823069101	-6.859036311	-6.102636503	-5.577213616	-7.037835177	0.843122958	0.501028075

GFOD1	-0.322859454	0.3332858	-0.031360787	0.656145254	0.291498667	-0.364646587	8	0.158237958	0.816496581	0.816496581	0.816496581	0.816496581	0.816496581	0.816496581	14	137	0	A_32_P15035	A_32_P15035	A_32_P15035	chr6	13358825	13358885	-	GFOD1	glucose-fructose oxidoreductase domain containing 1 [Source:HGNC Symbol;Acc:HGNC:21096]	ENSG00000145990	protein_coding	3'UTR	3'UTR	1	2347	6:13358825:13358885	ENSG00000145990	GFOD1	6.347232475	0.022314568	-2.647066757	2.732550495	-0.257121466	5.379617252	2.389945291	-2.989671961	9.961963646	0.024509336	0.021169461	0.802317698	0.000314344	0.038050826	0.013635988	-4.110568646	-3.285543385	-7.011136622	0.221633153	-3.916180462	-3.596032192	9.688360795	0.002666503

ZNF570	0.175235284	0.075280593	0.047314392	-0.099954692	-0.127920893	-0.027966201	8	0.097963365	0.816496581	0.816496581	0.816496581	0.816496581	0.816496581	0.816496581	14	138	0	A_32_P155043	A_32_P155043	A_32_P155043	chr19	37487829	37487889	+	ZNF570	zinc finger protein 570 [Source:HGNC Symbol;Acc:HGNC:26416]	ENSG00000171827	protein_coding	3'UTR	3'UTR	1	2348	19:37487829:37487889	ENSG00000171827	ZNF570	6.396101617	0.009913454	2.15553301	0.926011009	0.582004551	-1.229522001	-1.57352846	-0.344006458	9.961963646	0.056635743	0.376328385	0.573497964	0.247124103	0.146788886	0.737992101	-4.921022418	-5.749214356	-6.865235284	-6.38027107	-5.125159924	-7.007112402	1.658100809	0.238424549

SLF2	-0.106365309	-0.130051993	-0.100207928	-0.023686684	0.006157381	0.029844065	8	0.086154659	0.816496581	0.816496581	0.816496581	0.816496581	0.816496581	0.816496581	14	139	0	P_209558	P_209558	P_209558	chr10	100956473	100956533	+	FAM178A	"family with sequence similarity 178, member A [Source:HGNC Symbol;Acc:HGNC:17814]"	ENSG00000119906	protein_coding	exon	coding exon	1	2349	10:100956473:100956533	ENSG00000119906	SLF2	6.271126516	0.008167457	-1.441458725	-1.762459793	-1.358014129	-0.321001068	0.083444595	0.404445664	9.961963646	0.180141534	0.108586019	0.204419015	0.754842417	0.93515099	0.694431967	-5.969377745	-4.765536965	-6.1237087	-7.093554196	-6.292983067	-6.982747088	1.216975189	0.353753619

ARHGEF12	0.103655844	-0.049283596	0.136150544	-0.15293944	0.0324947	0.18543414	8	0.065158252	0.816496581	0.816496581	0.816496581	0.816496581	0.816496581	0.816496581	14	142	0	P_213650	P_213650	P_213650	chr11	120488920	120488980	-	ARHGEF12	Rho guanine nucleotide exchange factor (GEF) 12 [Source:HGNC Symbol;Acc:HGNC:14193]	ENSG00000196914	protein_coding	3'ext;3'UTR	multiple non-Ref	0	2352	11:120488920:120488980	ENSG00000196914	ARHGEF12	7.04720945	0.005616131	1.694028939	-0.805432998	2.225084012	-2.499461937	0.531055073	3.030517009	9.961963646	0.121240845	0.43937123	0.05035754	0.031558094	0.607016759	0.012718627	-5.623775924	-5.851219332	-4.855948633	-4.502149131	-6.148836165	-3.526132972	4.030469671	0.040707349

SLC2A13	-0.066433987	0.243841271	0.206876194	0.310275258	0.273310181	-0.036965078	8	0.074624983	0.816496581	0.816496581	0.816496581	0.816496581	0.816496581	0.816496581	14	143	0	A_23_P10211	A_23_P10211	A_23_P10211	chr12	39759101	39759161	-	SLC2A13	"solute carrier family 2 (facilitated glucose transporter), member 13 [Source:HGNC Symbol;Acc:HGNC:15956]"	ENSG00000151229	protein_coding	3'UTR	3'UTR	1	2353	12:39759101:39759161	ENSG00000151229	SLC2A13	5.728933692	0.006678805	-0.995603861	3.654293881	3.100321798	4.649897741	4.095925659	-0.553972082	9.961963646	0.343010272	0.004458676	0.011293784	0.000917437	0.002175846	0.59181467	-6.482910441	-1.820374567	-3.3842076	-0.896732456	-1.183920994	-6.906274507	10.44368786	0.002027967

SEC24A	-0.26207395	0.09921162	-0.174255105	0.36128557	0.087818845	-0.273466726	8	0.068701164	0.816496581	0.816496581	0.816496581	0.816496581	0.816496581	0.816496581	14	145	0	A_23_P394605	A_23_P394605	A_23_P394605	chr5	134727532	134727592	+	SEC24A	SEC24 family member A [Source:HGNC Symbol;Acc:HGNC:10703]	ENSG00000113615	protein_coding	3'UTR	3'UTR	1	2355	5:134727532:134727592	ENSG00000113615	SEC24A	7.843115303	0.005996981	-4.144795955	1.56906828	-2.755908612	5.713864236	1.388887344	-4.324976892	9.961963646	0.002012836	0.14782014	0.020338937	0.000197394	0.195132061	0.001514683	-1.581614111	-5.027989103	-3.972072782	0.708531427	-5.361718673	-1.350295392	13.41983646	0.000781891

RYR1	-0.014000761	-0.203274303	-0.069529936	-0.189273542	-0.055529175	0.133744367	8	0.083647632	0.816496581	0.816496581	0.816496581	0.816496581	0.816496581	0.816496581	14	146	0	P_232863	P_232863	P_232863	chr19	38490655	38490715	+	RYR1	ryanodine receptor 1 (skeletal) [Source:HGNC Symbol;Acc:HGNC:10483]	ENSG00000196218	protein_coding	exon	coding exon	1	2356	19:38490655:38490715	ENSG00000196218	RYR1	4.352418624	0.007825597	-0.193837827	-2.814293342	-0.962628497	-2.620455516	-0.768790671	1.851664845	9.961963646	0.850198272	0.01840337	0.358517886	0.025652832	0.459855482	0.093902382	-6.977895852	-3.154887941	-6.563401593	-4.298634726	-5.991589983	-5.463522221	3.295160441	0.066430628

STK39	-0.816729465	0.218955426	-0.775628473	1.03568489	0.041100992	-0.994583899	8	0.071416333	0.816496581	0.816496581	0.816496581	0.816496581	0.816496581	0.816496581	14	147	0	P_237397	P_237397	P_237397	chr2	168167298	168167358	-	STK39	serine threonine kinase 39 [Source:HGNC Symbol;Acc:HGNC:17717]	ENSG00000198648	protein_coding	exon	coding exon	1	2357	2:168167298:168167358	ENSG00000198648	STK39	7.707618205	0.006302497	-12.59991245	3.377886204	-11.96583603	15.97779866	0.63407642	-15.34372224	9.961963646	1.91E-07	0.007064947	3.11E-07	1.99E-08	0.540316669	2.94E-08	7.838532254	-2.254945937	7.375021114	10.04940096	-6.087180974	9.643764768	134.0785093	2.37E-08

NADK	-0.16089714	-0.024189038	-0.251296463	0.136708103	-0.090399323	-0.227107426	8	0.114203907	0.816496581	0.816496581	0.816496581	0.816496581	0.816496581	0.816496581	14	150	0	P_100126	P_100126	P_100126	chr1	1758539	1758599	-	NADK	NAD kinase [Source:HGNC Symbol;Acc:HGNC:29831]	ENSG00000008130	protein_coding	"5'UTR,promoter;intron;promoter"	multiple non-Ref	0	2360	1:1758539:1758599	ENSG00000008130	NADK	6.293810226	0.012680549	-1.749947153	-0.263084461	-2.733146963	1.486862692	-0.98319981	-2.470062501	9.961963646	0.110806124	0.797846619	0.021147835	0.168000561	0.348784679	0.033185301	-5.542991557	-6.145731509	-4.01070926	-6.058439787	-5.80674009	-4.476478798	3.313378681	0.065592546

C9orf72	-0.190694999	0.163979056	-0.242474838	0.354674055	-0.051779839	-0.406453894	8	0.080225969	0.816496581	0.816496581	0.816496581	0.816496581	0.816496581	0.816496581	14	152	0	A_23_P324633	A_23_P324633	A_23_P324633	chr9	27547617	27547677	-	C9orf72	chromosome 9 open reading frame 72 [Source:HGNC Symbol;Acc:HGNC:28337]	ENSG00000147894	protein_coding	3'UTR;exon	multiple Ref	1	2362	9:27547617:27547677	ENSG00000147894	C9orf72	6.638403741	0.007375308	-2.719536176	2.338535249	-3.457977916	5.058071425	-0.73844174	-5.796513165	9.961963646	0.021646872	0.041534555	0.006177945	0.000499219	0.47728234	0.000176364	-3.988106055	-3.908151592	-2.773365469	-0.261901336	-6.014563702	0.886179182	14.09186383	0.000645152

PMPCA	0.048631568	0.065986481	0.089715435	0.017354913	0.041083867	0.023728954	8	0.111440692	0.816496581	0.816496581	0.816496581	0.816496581	0.816496581	0.816496581	14	153	0	P_150151	P_150151	P_150151	chr9	136423288	136423348	+	PMPCA	peptidase (mitochondrial processing) alpha [Source:HGNC Symbol;Acc:HGNC:18667]	ENSG00000165688	protein_coding	3'UTR	3'UTR	1	2363	9:136423288:136423348	ENSG00000165688	PMPCA	7.899026023	0.012179841	0.539688424	0.732284428	0.995616293	0.192596005	0.455927869	0.263331865	9.961963646	0.601266102	0.480868451	0.34300452	0.851144978	0.658219065	0.797661279	-6.841717866	-5.907049504	-6.531244427	-7.129365922	-6.187297427	-7.033628003	0.355510499	0.786347513

GOLM2	0.336521198	-0.070218924	0.124761033	-0.406740122	-0.211760164	0.194979958	8	0.111319802	0.816496581	0.816496581	0.816496581	0.816496581	0.816496581	0.816496581	14	155	0	P_116390	P_116390	P_116390	chr15	44380916	44380976	+	CASC4	cancer susceptibility candidate 4 [Source:HGNC Symbol;Acc:HGNC:24892]	ENSG00000166734	protein_coding	exon	coding exon	1	2365	15:44380916:44380976	ENSG00000166734	GOLM2	9.695000929	0.012158215	3.73786105	-0.779946651	1.385765326	-4.517807701	-2.352095724	2.165711977	9.961963646	0.00388566	0.453554175	0.196054688	0.00112284	0.040586491	0.055672017	-2.256733013	-5.871203118	-6.08878322	-1.107041856	-3.976081754	-4.974902497	7.855586106	0.005553228

TM2D1	0.007330149	0.050729329	-0.010269438	0.04339918	-0.017599587	-0.060998767	8	0.086913244	0.816496581	0.816496581	0.816496581	0.816496581	0.816496581	0.816496581	14	156	0	P_308495	P_308495	P_308495	chr1	61681706	61681766	+	TM2D1	TM2 domain containing 1 [Source:HGNC Symbol;Acc:HGNC:24142]	ENSG00000162604	protein_coding	3'ext;intron	multiple non-Ref	0	2366	1:61681706:61681766	ENSG00000162604	TM2D1	7.880834046	0.008272887	0.098702887	0.683087273	-0.1382814	0.584384386	-0.236984287	-0.821368673	9.961963646	0.923332021	0.510125101	0.892773262	0.57195735	0.817471131	0.430654245	-6.993046661	-5.941925229	-7.036688105	-6.966004037	-6.266900355	-6.714844777	0.261297565	0.851688414

DDAH1	-0.039125753	0.054369809	-0.089721707	0.093495562	-0.050595954	-0.144091516	8	0.045223896	0.816496581	0.816496581	0.816496581	0.816496581	0.816496581	0.816496581	15	15	0	P_102282	P_102282	P_102282	chr1	85321302	85321362	-	DDAH1	dimethylarginine dimethylaminohydrolase 1 [Source:HGNC Symbol;Acc:HGNC:2715]	ENSG00000153904	protein_coding	3'UTR;exon	multiple Ref	1	2395	1:85321302:85321362	ENSG00000153904	DDAH1	7.829061634	0.003849092	-0.772376777	1.073307854	-1.771185415	1.845684632	-0.998808638	-2.84449327	9.961963646	0.457823757	0.308443517	0.107061958	0.0948264	0.341529901	0.017476093	-6.682270536	-5.60877304	-5.559398645	-5.548815013	-5.791810861	-3.844060127	2.896827638	0.088366802

MCF2L2	0.053528951	-0.178684771	-0.024552975	-0.232213723	-0.078081926	0.154131797	8	0.062337614	0.816496581	0.816496581	0.816496581	0.816496581	0.816496581	0.816496581	15	16	0	P_247470	P_247470	P_247470	chr3	183229676	183229736	-	MCF2L2	MCF.2 cell line derived transforming sequence-like 2 [Source:HGNC Symbol;Acc:HGNC:30319]	ENSG00000053524	protein_coding	exon	coding exon	1	2396	3:183229676:183229736	ENSG00000053524	MCF2L2	4.705317889	0.005327336	0.898212937	-2.998320902	-0.4119976	-3.896533839	-1.310210538	2.586323301	9.961963646	0.390264763	0.013436277	0.689066257	0.002999238	0.219529116	0.027197619	-6.575161366	-2.860236256	-6.955209421	-2.125110895	-5.456622533	-4.281751005	5.592525428	0.016402972

KALRN	0.36275021	-0.200345031	0.245308892	-0.563095241	-0.117441318	0.445653923	8	0.052959017	0.816496581	0.816496581	0.816496581	0.816496581	0.816496581	0.816496581	15	17	0	P_245944	P_245944	P_245944	chr3	124482840	124482900	+	KALRN	"kalirin, RhoGEF kinase [Source:HGNC Symbol;Acc:HGNC:4814]"	ENSG00000160145	protein_coding	exon	coding exon	1	2397	3:124482840:124482900	ENSG00000160145	KALRN	4.937255117	0.004458977	6.653278335	-3.674570589	4.499262282	-10.32784892	-2.154016053	8.173832871	9.961963646	5.79E-05	0.004312001	0.001155374	1.22E-06	0.05678073	9.98E-06	2.093143193	-1.788790116	-1.049364672	5.991114252	-4.285214779	3.867495421	42.68795584	5.39E-06

CUL2	0.042130369	-0.040923197	0.017842138	-0.083053566	-0.024288231	0.058765335	8	0.049919607	0.816496581	0.816496581	0.816496581	0.816496581	0.816496581	0.816496581	15	19	0	P_208107	P_208107	P_208107	chr10	35074195	35074255	-	CUL2	cullin 2 [Source:HGNC Symbol;Acc:HGNC:2552]	ENSG00000108094	protein_coding	5'UTR;exon	multiple Ref	1	2399	10:35074195:35074255	ENSG00000108094	CUL2	8.239732176	0.00420787	0.795444271	-0.77265222	0.336869264	-1.56809649	-0.458575007	1.109521483	9.961963646	0.444894525	0.457667945	0.74320468	0.148045678	0.656380858	0.293270077	-6.663691447	-5.876818717	-6.985500443	-5.948646351	-6.186037177	-6.437548056	0.873909917	0.486730119

PNPT1	-0.073617104	0.112619657	0.086781669	0.186236761	0.160398773	-0.025837988	8	0.043924855	0.816496581	0.816496581	0.816496581	0.816496581	0.816496581	0.816496581	15	20	0	P_127876	P_127876	P_127876	chr2	55634062	55634122	-	PNPT1	polyribonucleotide nucleotidyltransferase 1 [Source:HGNC Symbol;Acc:HGNC:23166]	ENSG00000138035	protein_coding	3'UTR	3'UTR	1	2400	2:55634062:55634122	ENSG00000138035	PNPT1	9.020591785	0.003756092	-1.471147663	2.250565925	1.734225379	3.721713588	3.205373042	-0.516340546	9.961963646	0.172120219	0.048229944	0.113654233	0.003990079	0.009450066	0.616883094	-5.930541184	-4.044363063	-5.613352294	-2.418997976	-2.59718204	-6.927658291	5.771506005	0.014922642

OSBPL1A	0.06468718	0.005939466	0.128019458	-0.058747714	0.063332277	0.122079992	8	0.034838173	0.816496581	0.816496581	0.816496581	0.816496581	0.816496581	0.816496581	15	23	0	P_230976	P_230976	P_230976	chr18	24317148	24317208	-	OSBPL1A	oxysterol binding protein-like 1A [Source:HGNC Symbol;Acc:HGNC:16398]	ENSG00000141447	protein_coding	3'UTR;exon	multiple Ref	1	2403	18:24317148:24317208	ENSG00000141447	OSBPL1A	9.041823629	0.00318135	1.404617693	0.128969591	2.779815013	-1.275648102	1.37519732	2.650845422	9.961963646	0.190539189	0.899948379	0.019522787	0.231021918	0.199205213	0.024351141	-6.016848883	-6.173326514	-3.931450728	-6.325730934	-5.378500245	-4.172941741	3.377063787	0.062759852

KLRK1	-0.024843311	0.07641646	-0.065410227	0.101259771	-0.040566917	-0.141826688	8	0.225911181	0.816496581	0.816496581	0.816496581	0.816496581	0.816496581	0.816496581	15	24	0	P_214374	P_214374	P_214374	chr12	10378150	10378210	-	KLRK1	"killer cell lectin-like receptor subfamily K, member 1 [Source:HGNC Symbol;Acc:HGNC:18788]"	ENSG00000213809	protein_coding	3'UTR;exon	multiple Ref	1	2404	12:10378150:10378210	ENSG00000213809	KLRK1	4.227696104	0.043191264	-0.146405416	0.450333847	-0.38547244	0.596739264	-0.239067024	-0.835806288	9.961963646	0.886521645	0.662111487	0.707990415	0.563996003	0.815900065	0.422857546	-6.986676321	-6.076295272	-6.966578293	-6.958295403	-6.266376077	-6.702622839	0.247441177	0.861323653

AOX1	0.070322542	0.060271953	0.126608147	-0.010050589	0.056285605	0.066336194	8	0.103702323	0.816496581	0.816496581	0.816496581	0.816496581	0.816496581	0.816496581	15	25	0	P_238594	P_238594	P_238594	chr2	200613906	200613966	+	AOX1	aldehyde oxidase 1 [Source:HGNC Symbol;Acc:HGNC:553]	ENSG00000138356	protein_coding	exon	coding exon	1	2405	2:200613906:200613966	ENSG00000138356	AOX1	4.132204255	0.01084287	0.827119114	0.708906175	1.489138693	-0.118212939	0.662019579	0.780232518	9.961963646	0.427537348	0.494637984	0.167411053	0.908248552	0.52297717	0.453393449	-6.637398788	-5.923894348	-5.954595472	-7.141976374	-6.068723089	-6.748643839	0.744202508	0.549919464

KIF21B	-0.140349893	-0.084624654	-0.250670473	0.05572524	-0.11032058	-0.166045819	8	0.102826832	0.816496581	0.816496581	0.816496581	0.816496581	0.816496581	0.816496581	15	26	0	P_104375	P_104375	P_104375	chr1	200973614	200973674	-	KIF21B	kinesin family member 21B [Source:HGNC Symbol;Acc:HGNC:29442]	ENSG00000116852	protein_coding	3'UTR	3'UTR	1	2406	1:200973614:200973674	ENSG00000116852	KIF21B	5.82309929	0.010697666	-1.661931767	-1.002069877	-2.968276016	0.659861891	-1.306344249	-1.966206139	9.961963646	0.127624086	0.340028307	0.014143101	0.524304231	0.22079078	0.077742114	-5.669497263	-5.67878631	-3.610066585	-6.916585181	-5.461188257	-5.289083508	3.097457223	0.076393657

POGZ	0.073602275	-0.134438531	-0.074453407	-0.208040805	-0.148055682	0.059985124	8	0.041030655	0.816496581	0.816496581	0.816496581	0.816496581	0.816496581	0.816496581	15	27	0	A_23_P500734	A_23_P500734	A_23_P500734	chr1	151402965	151403025	-	POGZ	pogo transposable element with ZNF domain [Source:HGNC Symbol;Acc:HGNC:18801]	ENSG00000143442	protein_coding	3'UTR;exon	multiple Ref	1	2407	1:151402965:151403025	ENSG00000143442	POGZ	7.550724071	0.003558638	1.511106094	-2.760116907	-1.528580427	-4.271223001	-3.039686521	1.23153648	9.961963646	0.161813009	0.020192822	0.15747716	0.001648015	0.012521467	0.246402322	-5.877476888	-3.241511248	-5.901777514	-1.505672233	-2.866511138	-6.300538328	6.872994543	0.008641252

CPS1	0.19155402	-0.053145674	0.044507287	-0.244699694	-0.147046733	0.097652961	8	0.057560634	0.816496581	0.816496581	0.816496581	0.816496581	0.816496581	0.816496581	15	28	0	A_23_P5300	NM_001875	A_23_P5300	chr2	210677888	210677948	+	CPS1	"carbamoyl-phosphate synthase 1, mitochondrial [Source:HGNC Symbol;Acc:HGNC:2323]"	ENSG00000021826	protein_coding	exon	coding exon	1	2408	2:210677888:210677948	ENSG00000021826	CPS1	6.600133027	0.004867386	3.362706955	-0.93296569	0.781319878	-4.295672646	-2.581387077	1.714285569	9.961963646	0.00724737	0.372897817	0.452782434	0.001585896	0.027428556	0.117362129	-2.891604188	-5.742965359	-6.723630099	-1.465808543	-3.610153375	-5.665900305	6.807303588	0.008913196

ARHGAP29	0.456794056	-0.153941578	0.507734714	-0.610735634	0.050940659	0.661676292	8	0.076372414	0.816496581	0.816496581	0.816496581	0.816496581	0.816496581	0.816496581	15	29	0	P_202948	P_202948	P_202948	chr1	94185341	94185401	-	ARHGAP29	Rho GTPase activating protein 29 [Source:HGNC Symbol;Acc:HGNC:30207]	ENSG00000137962	protein_coding	exon	coding exon	1	2409	1:94185341:94185401	ENSG00000137962	ARHGAP29	8.705319957	0.006890697	6.739605911	-2.271276421	7.491191793	-9.010882332	0.751585883	9.762468215	9.961963646	5.20E-05	0.0465651	2.13E-05	4.20E-06	0.469683834	2.04E-06	2.203756096	-4.012421552	3.099049268	4.720803198	-6.004714697	5.492803028	47.29426771	3.36E-06

PIK3C2G	0.05243317	0.090830309	-0.067227931	0.03839714	-0.119661101	-0.158058241	8	0.318316588	0.816496581	0.816496581	0.816496581	0.816496581	0.816496581	0.816496581	15	30	0	P_309343	P_309343	P_309343	chr12	18314003	18314063	+	PIK3C2G	"phosphatidylinositol-4-phosphate 3-kinase, catalytic subunit type 2 gamma [Source:HGNC Symbol;Acc:HGNC:8973]"	ENSG00000139144	protein_coding	3'UTR;exon	multiple Ref	1	2410	12:18314003:18314063	ENSG00000139144	PIK3C2G	3.613595329	0.083576545	0.222131185	0.384799247	-0.284808646	0.162668063	-0.506939831	-0.669607894	9.961963646	0.828699431	0.708473494	0.781623678	0.874032121	0.623228755	0.518325947	-6.971501635	-6.104627325	-7.003003052	-7.135161784	-6.161787736	-6.831820052	0.168392052	0.915266763

RPL15	0.139137314	0.006899673	0.107582045	-0.132237641	-0.031555268	0.100682372	8	0.049022689	0.816496581	0.816496581	0.816496581	0.816496581	0.816496581	0.816496581	15	31	0	P_134238	P_134238	P_134238	chr3	23920486	23920546	+	RPL15	ribosomal protein L15 [Source:HGNC Symbol;Acc:HGNC:10306]	ENSG00000174748	protein_coding	3'UTR	3'UTR	1	2411	3:23920486:23920546	ENSG00000174748	RPL15	11.95052658	0.004136604	2.649520448	0.131386927	2.048629669	-2.518133521	-0.600890779	1.917242741	9.961963646	0.024406495	0.898084788	0.067769923	0.030565847	0.56133472	0.084303372	-4.1064306	-6.172995315	-5.137296121	-4.470871582	-6.10814857	-5.364237869	3.601998423	0.053852751

GATA2-AS1	-0.13268959	-0.032250039	-0.131303925	0.100439551	0.001385665	-0.099053886	8	0.122383848	0.816496581	0.816496581	0.816496581	0.816496581	0.816496581	0.816496581	15	32	0	P_246055	P_246055	P_246055	chr3	128490009	128490069	+	GATA2-AS1	GATA2 antisense RNA 1 [Source:HGNC Symbol;Acc:HGNC:51108]	ENSG00000244300	antisense	exon	non-coding exon	1	2412	3:128490009:128490069	ENSG00000244300	GATA2-AS1	9.252014287	0.014234679	-1.362098902	-0.331056437	-1.34787463	1.031042466	0.014224273	-1.016818193	9.961963646	0.203169303	0.74746012	0.207549066	0.326902907	0.988931894	0.333298748	-6.070612244	-6.124638979	-6.136348974	-6.597416404	-6.296577642	-6.534167395	0.979807989	0.440713707

ZNF185	-0.206370026	0.184150153	-0.157238884	0.390520179	0.049131141	-0.341389037	8	0.138982887	0.816496581	0.816496581	0.816496581	0.816496581	0.816496581	0.816496581	15	33	0	P_151874	P_151874	P_151874	chrX	152971693	152971753	+	ZNF185	zinc finger protein 185 (LIM domain) [Source:HGNC Symbol;Acc:HGNC:12976]	ENSG00000147394	protein_coding	3'UTR	3'UTR	1	2413	X:152971693:152971753	ENSG00000147394	ZNF185	4.694528793	0.01771868	-1.898788367	1.694345714	-1.446738031	3.593134081	0.452050336	-3.141083745	9.961963646	0.086907967	0.121179308	0.178692115	0.004933358	0.660915992	0.010538207	-5.321520245	-4.859838314	-6.010396083	-2.6367683	-6.189130829	-3.336823218	5.258437452	0.019664862

GRPEL2	-0.136541749	0.132190024	-0.002379025	0.268731773	0.134162724	-0.134569049	8	0.051891525	0.816496581	0.816496581	0.816496581	0.816496581	0.816496581	0.816496581	15	34	0	P_140824	P_140824	P_140824	chr5	149351594	149351654	+	GRPEL2	"GrpE-like 2, mitochondrial (E. coli) [Source:HGNC Symbol;Acc:HGNC:21060]"	ENSG00000164284	protein_coding	3'UTR;exon	multiple Ref	1	2414	5:149351594:149351654	ENSG00000164284	GRPEL2	9.961679395	0.004369093	-2.529970359	2.449337629	-0.044080746	4.979307988	2.485889613	-2.493418375	9.961963646	0.029952864	0.034381835	0.965711101	0.000560377	0.03229927	0.031886031	-4.307246877	-3.734807036	-7.046059553	-0.382591098	-3.76338209	-4.437513807	8.265373058	0.004668467

AGTR2	0.602178344	0.770758199	1.074905584	0.168579856	0.47272724	0.304147384	8	1.598535938	0.816496581	0.816496581	0.816496581	0.816496581	0.816496581	0.816496581	15	35	0	A_23_P62309	A_23_P62309	A_23_P62309	chrX	116174310	116174370	+	AGTR2	"angiotensin II receptor, type 2 [Source:HGNC Symbol;Acc:HGNC:338]"	ENSG00000180772	protein_coding	exon	coding exon	1	2415	X:116174310:116174370	ENSG00000180772	AGTR2	2.685535183	2.054265683	0.514567623	0.658620853	0.918517939	0.14405323	0.403950316	0.259897086	9.961963646	0.618077344	0.525068419	0.380049658	0.888330887	0.694784539	0.800235584	-6.855774266	-5.958451114	-6.604991857	-7.138276728	-6.21062073	-7.034603059	0.298951693	0.825468105

LPP	0.056093328	-0.084207324	0.039245012	-0.140300652	-0.016848316	0.123452336	8	0.039525438	0.816496581	0.816496581	0.816496581	0.816496581	0.816496581	0.816496581	15	36	0	A_24_P114551	A_24_P114551	A_24_P114551	chr3	188877953	188878013	+	LPP	LIM domain containing preferred translocation partner in lipoma [Source:HGNC Symbol;Acc:HGNC:6679]	ENSG00000145012	protein_coding	3'UTR	3'UTR	1	2416	3:188877953:188878013	ENSG00000145012	LPP	7.735880244	0.003461264	1.167722011	-1.752984705	0.816982451	-2.920706716	-0.35073956	2.569967155	9.961963646	0.270106973	0.110263401	0.433041924	0.015340254	0.733087383	0.027970322	-6.301357939	-4.778768152	-6.694392101	-3.787414864	-6.231667533	-4.309256261	3.393711256	0.062043479

IDI1	-0.415979365	-0.004200546	-0.282345132	0.411778819	0.133634233	-0.278144586	8	0.047455451	0.816496581	0.816496581	0.816496581	0.816496581	0.816496581	0.816496581	15	37	0	P_207199	P_207199	P_207199	chr10	1042696	1042756	-	IDI1	isopentenyl-diphosphate delta isomerase 1 [Source:HGNC Symbol;Acc:HGNC:5387]	ENSG00000067064	protein_coding	exon	coding exon	1	2417	10:1042696:1042756	ENSG00000067064	IDI1	9.052232853	0.004015179	-8.04016529	-0.081189332	-5.457245529	7.958975958	2.582919761	-5.376056197	9.961963646	1.15E-05	0.936899378	0.000281751	1.26E-05	0.027356643	0.000315933	3.75395956	-6.17861366	0.416558801	3.583160628	-3.60768628	0.278623828	32.22518856	1.93E-05

EIF4G1	-0.224352186	-0.245337928	-0.260665327	-0.020985742	-0.036313141	-0.015327399	8	0.056235306	0.816496581	0.816496581	0.816496581	0.816496581	0.816496581	0.816496581	15	38	0	P_136724	P_136724	P_136724	chr3	184331749	184331809	+	EIF4G1	"eukaryotic translation initiation factor 4 gamma, 1 [Source:HGNC Symbol;Acc:HGNC:3296]"	ENSG00000114867	protein_coding	3'UTR;exon	multiple Ref	1	2418	3:184331749:184331809	ENSG00000114867	EIF4G1	10.83393484	0.004746271	-3.988408665	-4.361481532	-4.633963533	-0.373072867	-0.645554868	-0.272482001	9.961963646	0.002585307	0.001430666	0.000939948	0.716909858	0.53315388	0.790815911	-1.839038941	-0.7497444	-0.835495328	-7.074028338	-6.079687158	-7.030968903	9.505618958	0.002855643

PRKACB	0.182538244	-0.166061386	-0.06065409	-0.34859963	-0.243192334	0.105407296	8	0.086232271	0.816496581	0.816496581	0.816496581	0.816496581	0.816496581	0.816496581	15	39	0	P_202497	P_202497	P_202497	chr1	84196682	84196742	+	PRKACB	"protein kinase, cAMP-dependent, catalytic, beta [Source:HGNC Symbol;Acc:HGNC:9381]"	ENSG00000142875	protein_coding	exon	coding exon	1	2419	1:84196682:84196742	ENSG00000142875	PRKACB	9.87409373	0.008178201	2.472126017	-2.24897897	-0.821441851	-4.721104987	-3.293567868	1.427537119	9.961963646	0.033068436	0.048359866	0.430614485	0.000823614	0.008141787	0.184012116	-4.403739602	-4.046807201	-6.69065571	-0.784328024	-2.454231304	-6.059185839	7.836393998	0.005599379

CRNKL1	-0.070946816	0.170194841	0.008863135	0.241141656	0.079809951	-0.161331705	8	0.032814809	0.816496581	0.816496581	0.816496581	0.816496581	0.816496581	0.816496581	15	40	0	P_240356	P_240356	P_240356	chr20	20050477	20050537	-	CRNKL1	crooked neck pre-mRNA splicing factor 1 [Source:HGNC Symbol;Acc:HGNC:15762]	ENSG00000101343	protein_coding	3'UTR;exon	multiple Ref	1	2420	20:20050477:20050537	ENSG00000101343	CRNKL1	8.2891875	0.003071422	-1.567865338	3.761163745	0.195867881	5.329029083	1.763733219	-3.565295864	9.961963646	0.14809937	0.00373997	0.848651195	0.000337742	0.108362368	0.005166534	-5.800592729	-1.654422365	-7.026210043	0.146541795	-4.863856747	-2.612936765	10.14392652	0.002256669

ASF1B	-0.553429976	-0.043916422	-0.422066064	0.509513554	0.131363913	-0.378149641	8	0.130033377	0.816496581	0.816496581	0.816496581	0.816496581	0.816496581	0.816496581	15	41	0	P_124862	P_124862	P_124862	chr19	14137523	14137583	+	ASF1B;CTB-55O6.12	anti-silencing function 1B histone chaperone [Source:HGNC Symbol;Acc:HGNC:20996];	ENSG00000105011	protein_coding;antisense	intron;promoter	multiple non-Ref	0	2421	19:14137523:14137583	ENSG00000105011	ASF1B	8.683580588	0.015785275	-5.394885473	-0.428101258	-4.11433817	4.966784215	1.280547303	-3.686236912	9.961963646	0.000307628	0.677685028	0.002112856	0.000570816	0.229363306	0.004229896	0.361994396	-6.086386988	-1.673311558	-0.401862092	-5.49141007	-2.408306485	14.35215465	0.000600025

RAD21	-0.171699505	-0.155581951	-0.185438372	0.016117554	-0.013738867	-0.029856421	8	0.080335104	0.816496581	0.816496581	0.816496581	0.816496581	0.816496581	0.816496581	15	42	0	P_266018	P_266018	P_266018	chr8	116861840	116861900	-	RAD21	RAD21 homolog (S. pombe) [Source:HGNC Symbol;Acc:HGNC:9811]	ENSG00000164754	protein_coding	exon	coding exon	1	2422	8:116861840:116861900	ENSG00000164754	RAD21	11.15159095	0.00738938	-2.446305353	-2.216668942	-2.642051194	0.229636411	-0.19574584	-0.425382252	9.961963646	0.034560427	0.051079871	0.024720899	0.823020567	0.848744181	0.679600821	-4.446648827	-4.096460406	-4.164883544	-7.120851732	-6.276345652	-6.973418212	3.025077494	0.080477476

FAF1	-0.282674765	-0.083276409	-0.26883163	0.199398356	0.013843135	-0.185555221	8	0.071715545	0.816496581	0.816496581	0.816496581	0.816496581	0.816496581	0.816496581	15	43	0	P_201632	P_201632	P_201632	chr1	50857928	50857988	-	FAF1	Fas (TNFRSF6) associated factor 1 [Source:HGNC Symbol;Acc:HGNC:3578]	ENSG00000185104	protein_coding	exon	coding exon	1	2423	1:50857928:50857988	ENSG00000185104	FAF1	8.727073801	0.006336889	-4.349052087	-1.28123726	-4.136070513	3.067814827	0.212981573	-2.854833254	9.961963646	0.001458706	0.229130511	0.002040969	0.011935868	0.835636528	0.017169559	-1.249693048	-5.383701789	-1.637608213	-3.535248949	-6.272614853	-3.826425416	9.211637355	0.003194852

TOPBP1	-0.416041769	-0.079610364	-0.302114115	0.336431405	0.113927654	-0.222503751	8	0.074255699	0.816496581	0.816496581	0.816496581	0.816496581	0.816496581	0.816496581	15	44	0	P_246271	P_246271	P_246271	chr3	133652462	133652522	-	TOPBP1	topoisomerase (DNA) II binding protein 1 [Source:HGNC Symbol;Acc:HGNC:17008]	ENSG00000163781	protein_coding	"3'UTR,exon;exon"	multiple Ref	1	2424	3:133652462:133652522	ENSG00000163781	TOPBP1	8.361921507	0.006634653	-6.255664395	-1.197032983	-4.542631664	5.058631413	1.713032732	-3.345598681	9.961963646	9.59E-05	0.259002353	0.001080803	0.000498811	0.117598722	0.00745881	1.570465813	-5.478397472	-0.980241851	-0.261046408	-4.935071291	-2.98698153	16.81983181	0.000316462

SFI1	-0.164439544	-0.082326735	0.027604207	0.082112809	0.192043751	0.109930942	8	0.107776959	0.816496581	0.816496581	0.816496581	0.816496581	0.816496581	0.816496581	15	45	0	P_311880	P_311880	P_311880	chr22	31533718	31533778	-	SFI1	"Sfi1 homolog, spindle assembly associated (yeast) [Source:HGNC Symbol;Acc:HGNC:29064]"	ENSG00000198089	protein_coding	3'ext;intron	multiple non-Ref	0	2425	22:31533718:31533778	ENSG00000198089	SFI1	6.772160656	0.011534863	-1.87519167	-0.938815593	0.314785474	0.936376077	2.189977145	1.253601068	9.961963646	0.090347533	0.370029594	0.759418734	0.371223764	0.053437783	0.238608025	-5.357215454	-5.737678929	-6.99327594	-6.690180275	-4.229703761	-6.274630924	1.956854017	0.184766421

MLH1	-0.092684952	-0.014716805	-0.180704161	0.077968147	-0.088019209	-0.165987356	8	0.038262088	0.816496581	0.816496581	0.816496581	0.816496581	0.816496581	0.816496581	15	46	0	P_243748	P_243748	P_243748	chr3	37014484	37014544	+	MLH1	mutL homolog 1 [Source:HGNC Symbol;Acc:HGNC:7127]	ENSG00000076242	protein_coding	exon	coding exon	1	2426	3:37014484:37014544	ENSG00000076242	MLH1	10.0044479	0.003382346	-1.951847418	-0.309920408	-3.80543921	1.64192701	-1.853591792	-3.495518802	9.961963646	0.079614865	0.763007529	0.003478649	0.131753655	0.093606424	0.005802643	-5.240513706	-6.13170493	-2.186260823	-5.845837928	-4.735126587	-2.731451167	6.12291752	0.012455051

SLC12A1	-0.09190264	-0.102637362	-0.031485282	-0.010734722	0.060417357	0.071152079	8	0.125743554	0.816496581	0.816496581	0.816496581	0.816496581	0.816496581	0.816496581	15	47	0	P_223289	P_223289	P_223289	chr15	48288105	48288165	+	SLC12A1	"solute carrier family 12 (sodium/potassium/chloride transporter), member 1 [Source:HGNC Symbol;Acc:HGNC:10910]"	ENSG00000074803	protein_coding	exon	coding exon	1	2427	15:48288105:48288165	ENSG00000074803	SLC12A1	3.541435362	0.014904133	-0.921977345	-1.029669253	-0.3158638	-0.107691907	0.606113545	0.713805452	9.961963646	0.378328378	0.32751632	0.758624097	0.916378148	0.557996727	0.491732294	-6.553384005	-5.652116179	-6.992908258	-7.143274464	-6.104917803	-6.799960141	0.483086723	0.701435363

CLEC4D	-0.014393225	0.008753398	0.106900142	0.023146623	0.121293367	0.098146744	8	0.211146617	0.816496581	0.816496581	0.816496581	0.816496581	0.816496581	0.816496581	15	48	0	P_214280	P_214280	P_214280	chr12	8519061	8519121	+	CLEC4D	"C-type lectin domain family 4, member D [Source:HGNC Symbol;Acc:HGNC:14554]"	ENSG00000166527	protein_coding	exon	coding exon	1	2428	12:8519061:8519121	ENSG00000166527	CLEC4D	3.418433906	0.038009179	-0.090418958	0.054989283	0.671552033	0.145408241	0.761970991	0.61656275	9.961963646	0.92974646	0.957234198	0.517138285	0.887288564	0.463735273	0.551352049	-6.993900808	-6.180493603	-6.806286777	-7.138062687	-5.996823885	-6.867593246	0.240679086	0.866016716

ABCA3	-0.166811609	0.06699642	-0.078370424	0.23380803	0.088441185	-0.145366844	8	0.054815869	0.816496581	0.816496581	0.816496581	0.816496581	0.816496581	0.816496581	15	49	0	P_117975	P_117975	P_117975	chr16	2292177	2292237	-	ABCA3	"ATP-binding cassette, sub-family A (ABC1), member 3 [Source:HGNC Symbol;Acc:HGNC:33]"	ENSG00000167972	protein_coding	exon	coding exon	1	2429	16:2292177:2292237	ENSG00000167972	ABCA3	7.236341803	0.004619686	-3.005838786	1.207232757	-1.412185049	4.213071544	1.593653737	-2.619417806	9.961963646	0.013265099	0.25522484	0.188362652	0.001806305	0.142214313	0.025698484	-3.500836048	-5.467175568	-6.0550923	-1.600772885	-5.098300457	-4.225999421	6.606299677	0.009811322

FAM234B	0.257232807	0.135602718	0.23610621	-0.121630089	-0.021126597	0.100503492	8	0.065729535	0.816496581	0.816496581	0.816496581	0.816496581	0.816496581	0.816496581	15	51	0	A_23_P394567	A_23_P394567	A_23_P394567	chr12	13083190	13083250	+	KIAA1467	KIAA1467 [Source:HGNC Symbol;Acc:HGNC:29288]	ENSG00000084444	protein_coding	3'UTR	3'UTR	1	2431	12:13083190:13083250	ENSG00000084444	FAM234B	7.120020459	0.005676178	4.181614365	2.204377739	3.83817729	-1.977236626	-0.343437075	1.633799551	9.961963646	0.001898541	0.05215292	0.003297736	0.076331643	0.738407437	0.133465295	-1.521412384	-4.115293187	-2.131444985	-5.348076754	-6.23433049	-5.780555665	7.295583249	0.00711088

SRSF9	0.082136857	0.038596474	-0.020455489	-0.043540383	-0.102592346	-0.059051963	8	0.13241394	0.816496581	0.816496581	0.816496581	0.816496581	0.816496581	0.816496581	15	52	0	A_24_P390070	A_24_P390070	A_24_P390070	chr12	120465696	120465756	-	SRSF9	serine/arginine-rich splicing factor 9 [Source:HGNC Symbol;Acc:HGNC:10791]	ENSG00000111786	protein_coding	exon	coding exon	1	2432	12:120465696:120465756	ENSG00000111786	SRSF9	10.40133682	0.016287001	0.788248475	0.370401462	-0.196306612	-0.417847013	-0.984555087	-0.566708074	9.961963646	0.448901722	0.718837412	0.848316932	0.684922582	0.348150306	0.583454327	-6.669538866	-6.110275083	-7.026116444	-7.054964342	-5.805451418	-6.898717092	0.377028679	0.771643511

ATP10D	0.127695864	0.247887705	0.340427587	0.120191841	0.212731723	0.092539881	8	0.142931925	0.816496581	0.816496581	0.816496581	0.816496581	0.816496581	0.816496581	15	54	0	P_248822	P_248822	P_248822	chr4	47580418	47580478	+	ATP10D	"ATPase, class V, type 10D [Source:HGNC Symbol;Acc:HGNC:13549]"	ENSG00000145246	protein_coding	3'UTR;exon	multiple Ref	1	2434	4:47580418:47580478	ENSG00000145246	ATP10D	4.033767458	0.018612715	1.14635096	2.225336834	3.056085607	1.078985874	1.909734647	0.830748773	9.961963646	0.278438976	0.050335992	0.012176587	0.306025421	0.085354131	0.425577812	-6.325121403	-4.083160497	-3.459897423	-6.547690087	-4.653187706	-6.706925277	3.517890708	0.056995484

SALL3	0.132793857	0.346181574	0.264472294	0.213387717	0.131678437	-0.08170928	8	0.204875397	0.816496581	0.816496581	0.816496581	0.816496581	0.816496581	0.816496581	15	55	0	A_23_P393670	A_23_P393670	A_23_P393670	chr18	78997655	78997715	+	SALL3	spalt-like transcription factor 3 [Source:HGNC Symbol;Acc:HGNC:10527]	ENSG00000256463	protein_coding	3'UTR	3'UTR	1	2435	18:78997655:78997715	ENSG00000256463	SALL3	3.604457834	0.035914037	0.858205912	2.237265186	1.709203207	1.379059273	0.850997295	-0.528061978	9.961963646	0.410951526	0.049329394	0.118324594	0.198048966	0.414757799	0.60901707	-6.610727578	-4.06483281	-5.649535515	-6.19832277	-5.9252959	-6.921149972	1.928016472	0.189285968

PLPP5	-0.576269878	-0.04624532	-0.621494867	0.530024558	-0.045224989	-0.575249547	8	0.09560784	0.816496581	0.816496581	0.816496581	0.816496581	0.816496581	0.816496581	15	56	0	P_264400	P_264400	P_264400	chr8	38267896	38267956	-	PPAPDC1B	phosphatidic acid phosphatase type 2 domain containing 1B [Source:HGNC Symbol;Acc:HGNC:25026]	ENSG00000147535	protein_coding	"3'UTR,exon;5'UTR;exon"	multiple Ref	1	2436	8:38267896:38267956	ENSG00000147535	PLPP5	11.51365871	0.009547292	-7.223229967	-0.579659973	-7.79010065	6.643569995	-0.566870683	-7.210440677	9.961963646	2.91E-05	0.575017975	1.52E-05	5.86E-05	0.583347997	2.95E-05	2.805115597	-6.007998598	3.44753373	1.979636199	-6.128556023	2.747150432	34.94092406	1.34E-05

MTM1	-0.06557329	0.057402229	-0.086529918	0.122975519	-0.020956628	-0.143932147	8	0.06968416	0.816496581	0.816496581	0.816496581	0.816496581	0.816496581	0.816496581	15	57	0	A_23_P62128	A_23_P62128	A_23_P62128	chrX	150672486	150672546	+	MTM1	myotubularin 1 [Source:HGNC Symbol;Acc:HGNC:7448]	ENSG00000171100	protein_coding	3'UTR;exon	multiple Ref	1	2437	X:150672486:150672546	ENSG00000171100	MTM1	6.051747031	0.006106222	-1.027747205	0.899680046	-1.356205879	1.927427251	-0.328458673	-2.255885925	9.961963646	0.328376345	0.389520268	0.204974298	0.082897346	0.749364776	0.047796863	-6.450754337	-5.772518575	-6.125967695	-5.424823404	-6.239621368	-4.829293573	2.102810851	0.16373125

RGS22	0.161117435	0.047711979	0.041566454	-0.113405456	-0.119550981	-0.006145525	8	0.116499609	0.816496581	0.816496581	0.816496581	0.816496581	0.816496581	0.816496581	15	59	0	P_265622	P_265622	P_265622	chr8	100047463	100047523	-	RGS22	regulator of G-protein signaling 22 [Source:HGNC Symbol;Acc:HGNC:24499]	ENSG00000132554	protein_coding	3'UTR;exon	multiple Ref	1	2439	8:100047463:100047523	ENSG00000132554	RGS22	3.834103315	0.013105868	1.723674595	0.51043468	0.444688317	-1.213239915	-1.278986279	-0.065746364	9.961963646	0.115602736	0.620865846	0.666050484	0.253020858	0.229890721	0.94888046	-5.58112236	-6.046575462	-6.940194266	-6.399171666	-5.493225236	-7.069017598	1.090237491	0.397627066

SLC5A12	0.104529502	0.034847873	0.020267932	-0.069681628	-0.08426157	-0.014579941	8	0.109034578	0.816496581	0.816496581	0.816496581	0.816496581	0.816496581	0.816496581	15	61	0	P_211336	P_211336	P_211336	chr11	26671101	26671161	-	SLC5A12	"solute carrier family 5 (sodium/monocarboxylate cotransporter), member 12 [Source:HGNC Symbol;Acc:HGNC:28750]"	ENSG00000148942	protein_coding	3'UTR;exon	multiple Ref	1	2441	11:26671101:26671161	ENSG00000148942	SLC5A12	4.159434513	0.011753829	1.18085024	0.393669912	0.22896304	-0.787180328	-0.9518872	-0.164706872	9.961963646	0.265087346	0.702118929	0.823529645	0.449498566	0.363677928	0.872468821	-6.286595363	-6.101043742	-7.018566903	-6.820928268	-5.836107676	-7.056578496	0.525779638	0.674462236

RAB33A	0.691194111	0.361533568	0.765158842	-0.329660543	0.073964731	0.403625274	8	0.156932632	0.816496581	0.816496581	0.816496581	0.816496581	0.816496581	0.816496581	15	63	0	P_151663	P_151663	P_151663	chrX	130184680	130184740	+	RAB33A	"RAB33A, member RAS oncogene family [Source:HGNC Symbol;Acc:HGNC:9773]"	ENSG00000134594	protein_coding	exon	coding exon	1	2443	X:130184680:130184740	ENSG00000134594	RAB33A	6.027263146	0.021984191	5.709399231	2.986338343	6.320362448	-2.723060888	0.610963217	3.334024105	9.961963646	0.000198604	0.013713768	8.82E-05	0.021516517	0.554907209	0.007605458	0.815976364	-2.879424009	1.624442419	-4.124728649	-6.101894643	-3.006751024	16.72774852	0.000323649

PFKL	-0.13568855	-0.088292339	-0.119345011	0.047396211	0.01634354	-0.031052672	8	0.174739678	0.816496581	0.816496581	0.816496581	0.816496581	0.816496581	0.816496581	15	64	0	A_23_P29079	A_23_P29079	A_23_P29079	chr21	44324606	44324866	+	PFKL;C21orf2	"phosphofructokinase, liver [Source:HGNC Symbol;Acc:HGNC:8876];chromosome 21 open reading frame 2 [Source:HGNC Symbol;Acc:HGNC:1260]"	ENSG00000141959	protein_coding	"3'ext;3'UTR,intron;exon,intron"	multiple non-Ref	0	2444	21:44324606:44324866	ENSG00000141959	PFKL	8.022579284	0.026727115	-1.016512662	-0.661443285	-0.894074808	0.355069377	0.122437854	-0.232631523	9.961963646	0.333437145	0.523331418	0.39237007	0.729939965	0.904987033	0.820757272	-6.462087555	-5.956572713	-6.627330574	-7.081102509	-6.288717922	-7.041894566	0.410892949	0.748781712

BORCS5	0.119607703	0.213467831	0.194417891	0.093860128	0.074810188	-0.01904994	8	0.038429805	0.816496581	0.816496581	0.816496581	0.816496581	0.816496581	0.816496581	15	65	0	A_23_P257593	A_23_P257593	A_23_P257593	chr12	12465982	12466042	+	LOH12CR1	"loss of heterozygosity, 12, chromosomal region 1 [Source:HGNC Symbol;Acc:HGNC:17950]"	ENSG00000165714	protein_coding	3'UTR	3'UTR	1	2445	12:12465982:12466042	ENSG00000165714	BORCS5	8.938765515	0.003392675	2.514974889	4.488559012	4.087998523	1.973584124	1.573023634	-0.40056049	9.961963646	0.030731508	0.001174604	0.002203571	0.076796003	0.146905289	0.697199354	-4.332307832	-0.565017294	-1.716655862	-5.353733704	-5.125831111	-6.984428165	8.285646496	0.004629264

N4BP2L2	0.075915496	-0.078567257	0.119292372	-0.154482753	0.043376876	0.197859629	8	0.105919407	0.816496581	0.816496581	0.816496581	0.816496581	0.816496581	0.816496581	15	67	0	P_113559	P_113559	P_113559	chr13	32442415	32442475	+	N4BP2L2	NEDD4 binding protein 2-like 2 [Source:HGNC Symbol;Acc:HGNC:26916]	ENSG00000244754	protein_coding	exon	exon antisense	0	2447	13:32442415:32442475	ENSG00000244754	N4BP2L2	8.048254766	0.011216089	0.877920803	-0.908586956	1.379550425	-1.786507759	0.501629622	2.288137381	9.961963646	0.400664623	0.385021847	0.197902326	0.104432948	0.626827646	0.045251005	-6.593374199	-5.76469866	-6.09664643	-5.636942394	-6.164563102	-4.776751057	2.02970825	0.173896527

FKBP15	-0.137909427	0.099517097	0.102900545	0.237426524	0.240809972	0.003383447	8	0.09917738	0.816496581	0.816496581	0.816496581	0.816496581	0.816496581	0.816496581	15	68	0	P_268624	P_268624	P_268624	chr9	113176579	113176639	-	FKBP15	"FK506 binding protein 15, 133kDa [Source:HGNC Symbol;Acc:HGNC:23397]"	ENSG00000119321	protein_coding	exon	coding exon	1	2448	9:113176579:113176639	ENSG00000119321	FKBP15	6.938491707	0.010105651	-1.680186291	1.212442588	1.253664002	2.892628879	2.933850293	0.041221414	9.961963646	0.123957626	0.253312507	0.238586084	0.016094565	0.014999554	0.967933844	-5.643553759	-5.461416738	-6.250602009	-3.835468234	-3.038773387	-7.070450323	3.806865835	0.047020897

MMUT	0.238862887	-0.004239958	0.236278934	-0.243102845	-0.002583953	0.240518892	8	0.054307119	0.816496581	0.816496581	0.816496581	0.816496581	0.816496581	0.816496581	15	70	0	P_257279	P_257279	P_257279	chr6	49447669	49447729	-	MUT	methylmalonyl CoA mutase [Source:HGNC Symbol;Acc:HGNC:7526]	ENSG00000146085	protein_coding	exon	coding exon	1	2450	6:49447669:49447729	ENSG00000146085	MMUT	9.37380242	0.004575103	4.325077211	-0.07677268	4.278289722	-4.401849891	-0.046787489	4.355062401	9.961963646	0.001514445	0.94032439	0.001629801	0.001343461	0.96360726	0.001445076	-1.288387562	-6.178981208	-1.40532481	-1.29354539	-6.295521053	-1.301594636	12.56016499	0.001011208

MLLT6	-0.026287955	-0.065287945	-0.066965717	-0.03899999	-0.040677763	-0.001677773	8	0.107130775	0.816496581	0.816496581	0.816496581	0.816496581	0.816496581	0.816496581	15	71	0	A_24_P265111	A_24_P265111	A_24_P265111	chr17	38717858	38717918	+	MLLT6	"myeloid/lymphoid or mixed-lineage leukemia (trithorax homolog, Drosophila); translocated to, 6 [Source:HGNC Symbol;Acc:HGNC:7138]"	ENSG00000275023	protein_coding	exon	coding exon	1	2451	17:38717858:38717918	ENSG00000275023	MLLT6	6.633835336	0.011423343	-0.301235266	-0.74813852	-0.767364219	-0.446903255	-0.466128953	-0.019225698	9.961963646	0.769428885	0.471669216	0.460665265	0.664503799	0.651148546	0.985040674	-6.949068738	-5.895348426	-6.734758027	-7.041478475	-6.182402512	-7.0711766	0.276111687	0.841371985

BCORL1	0.077885612	-0.010617523	0.106757104	-0.088503135	0.028871492	0.117374627	8	0.134395315	0.816496581	0.816496581	0.816496581	0.816496581	0.816496581	0.816496581	15	72	0	P_271484	P_271484	P_271484	chrX	130028789	130028849	+	BCORL1	BCL6 corepressor-like 1 [Source:HGNC Symbol;Acc:HGNC:25657]	ENSG00000085185	protein_coding	exon	coding exon	1	2452	X:130028789:130028849	ENSG00000085185	BCORL1	6.917310174	0.016711536	0.737895202	-0.10059136	1.011426281	-0.838486562	0.273531079	1.112017641	9.961963646	0.477599966	0.921870522	0.335747445	0.421420699	0.790032258	0.292245851	-6.709136438	-6.176755994	-6.515518439	-6.77817239	-6.257043541	-6.434854842	0.598675546	0.63034535

MYPN	0.047619127	-0.039452565	0.293781603	-0.087071691	0.246162476	0.333234167	8	0.229802039	0.816496581	0.816496581	0.816496581	0.816496581	0.816496581	0.816496581	15	73	0	A_23_P104438	A_23_P104438	A_23_P104438	chr10	68211235	68211295	+	MYPN	myopalladin [Source:HGNC Symbol;Acc:HGNC:23246]	ENSG00000138347	protein_coding	3'UTR	3'UTR	1	2453	10:68211235:68211295	ENSG00000138347	MYPN	3.621763942	0.044615172	0.27611231	-0.228759735	1.703448227	-0.504872045	1.427335917	1.932207962	9.961963646	0.788105143	0.823683363	0.119423057	0.624628927	0.184068573	0.08224493	-6.956917507	-6.154575222	-5.65781729	-7.011983165	-5.3140034	-5.341357568	1.50931935	0.271725868

SMG6	-0.002468948	-0.135232672	-0.097233892	-0.132763724	-0.094764944	0.03799878	8	0.141087322	0.816496581	0.816496581	0.816496581	0.816496581	0.816496581	0.816496581	15	76	0	P_120033	P_120033	P_120033	chr17	2094959	2095019	-	SMG6	SMG6 nonsense mediated mRNA decay factor [Source:HGNC Symbol;Acc:HGNC:17809]	ENSG00000070366	protein_coding	exon	non-coding exon	1	2456	17:2094959:2095019	ENSG00000070366	SMG6	5.949772117	0.018191992	-0.022419064	-1.227968218	-0.882923687	-1.205549154	-0.860504623	0.345044531	9.961963646	0.982556374	0.247681987	0.398082863	0.255845301	0.409742824	0.737235107	-6.998086052	-5.444147958	-6.637351543	-6.408034387	-5.917253476	-7.006726272	0.766805916	0.538343277

SORL1	0.359685551	-0.252745741	0.041625118	-0.612431292	-0.318060433	0.294370859	8	0.051038619	0.816496581	0.816496581	0.816496581	0.816496581	0.816496581	0.816496581	15	77	0	P_110333	P_110333	P_110333	chr11	121629618	121629678	+	SORL1	"sortilin-related receptor, L(DLR class) A repeats containing [Source:HGNC Symbol;Acc:HGNC:11185]"	ENSG00000137642	protein_coding	3'UTR;exon	multiple Ref	1	2457	11:121629618:121629678	ENSG00000137642	SORL1	7.302447324	0.004298593	6.719012376	-4.721351065	0.777567185	-11.44036344	-5.941445191	5.49891825	9.961963646	5.34E-05	0.000823308	0.454893443	4.73E-07	0.000145079	0.000265765	2.177460823	-0.233314972	-6.726639861	6.950145895	1.389112206	0.45880294	44.07694708	4.66E-06

HOXB13	0.12566386	-0.027494261	-0.012543586	-0.153158121	-0.138207446	0.014950675	8	0.05753457	0.816496581	0.816496581	0.816496581	0.816496581	0.816496581	0.816496581	15	78	0	A_23_P4212	A_23_P4212	A_23_P4212	chr17	48726536	48726596	-	HOXB13	homeobox B13 [Source:HGNC Symbol;Acc:HGNC:5112]	ENSG00000159184	protein_coding	3'UTR	3'UTR	1	2458	17:48726536:48726596	ENSG00000159184	HOXB13	11.97157777	0.004864977	2.206559536	-0.482777812	-0.220255597	-2.689337348	-2.426815134	0.262522215	9.961963646	0.05196087	0.639686801	0.830120236	0.022796643	0.035730331	0.798267869	-4.839137478	-6.060691701	-7.020692191	-4.18200032	-3.857615917	-7.033858981	3.056896152	0.07865113

FST	0.227251115	-0.046015505	-0.267879519	-0.27326662	-0.495130634	-0.221864014	8	0.234019937	0.816496581	0.816496581	0.816496581	0.816496581	0.816496581	0.816496581	15	79	0	P_139485	P_139485	P_139485	chr5	53485879	53485939	+	FST	follistatin [Source:HGNC Symbol;Acc:HGNC:3971]	ENSG00000134363	protein_coding	3'UTR	3'UTR	1	2459	5:53485879:53485939	ENSG00000134363	FST	3.679002697	0.046186231	1.295076408	-0.262236756	-1.526612729	-1.557313164	-2.821689137	-1.264375973	9.961963646	0.224502036	0.798481769	0.15796027	0.150568761	0.018171796	0.23487546	-6.153041286	-6.145964586	-5.904432863	-5.963426552	-3.221206843	-6.261858141	2.67705632	0.104120936

BCO2	0.010269027	0.109195074	0.276823945	0.098926047	0.266554918	0.167628871	8	0.13507173	0.816496581	0.816496581	0.816496581	0.816496581	0.816496581	0.816496581	15	80	0	P_213351	P_213351	P_213351	chr11	112179413	112179473	+	BCO2	beta-carotene oxygenase 2 [Source:HGNC Symbol;Acc:HGNC:18503]	ENSG00000197580	protein_coding	exon	coding exon	1	2460	11:112179413:112179473	ENSG00000197580	BCO2	4.018035877	0.01685791	0.096866383	1.030022749	2.611243807	0.933156366	2.514377424	1.581221058	9.961963646	0.924753588	0.327358331	0.026060926	0.372804078	0.030762943	0.145025177	-6.993242465	-5.651770819	-4.216834611	-6.693205008	-3.717779702	-5.853745532	2.930343141	0.086219982

ARHGAP32	0.195498377	-0.147412857	0.086749089	-0.342911234	-0.108749288	0.234161946	8	0.051474012	0.816496581	0.816496581	0.816496581	0.816496581	0.816496581	0.816496581	15	81	0	P_213849	P_213849	P_213849	chr11	128988033	128988093	-	ARHGAP32	Rho GTPase activating protein 32 [Source:HGNC Symbol;Acc:HGNC:17399]	ENSG00000134909	protein_coding	exon	coding exon	1	2461	11:128988033:128988093	ENSG00000134909	ARHGAP32	7.082604077	0.004334436	3.63682558	-2.742298209	1.613779674	-6.379123789	-2.023045906	4.356077883	9.961963646	0.004589199	0.02081881	0.137766449	8.18E-05	0.070727917	0.001442786	-2.426765682	-3.269977162	-5.784832581	1.630292155	-4.484641636	-1.299952805	14.51872236	0.00057313

PSMC3IP	-0.442087745	-0.087467623	-0.274485483	0.354620122	0.167602262	-0.18701786	8	0.051852415	0.816496581	0.816496581	0.816496581	0.816496581	0.816496581	0.816496581	15	82	0	P_228804	P_228804	P_228804	chr17	42577212	42577272	-	PSMC3IP	PSMC3 interacting protein [Source:HGNC Symbol;Acc:HGNC:17928]	ENSG00000131470	protein_coding	"5'UTR;5'UTR,exon;exon"	multiple Ref	1	2462	17:42577212:42577272	ENSG00000131470	PSMC3IP	9.274977532	0.004365835	-8.194462096	-1.621284754	-5.087815507	6.573177342	3.106646589	-3.466530753	9.961963646	9.77E-06	0.136139759	0.000478012	6.40E-05	0.011173012	0.006090286	3.924274282	-4.958778765	-0.133301895	1.887570699	-2.757578657	-2.780771743	26.75639813	4.41E-05

HECTD2	0.254031565	-0.102656466	0.174677053	-0.356688031	-0.079354512	0.277333519	8	0.079785476	0.816496581	0.816496581	0.816496581	0.816496581	0.816496581	0.816496581	15	83	0	P_209002	P_209002	P_209002	chr10	91460478	91460538	+	HECTD2	HECT domain containing E3 ubiquitin protein ligase 2 [Source:HGNC Symbol;Acc:HGNC:26736]	ENSG00000165338	protein_coding	exon	coding exon	1	2463	10:91460478:91460538	ENSG00000165338	HECTD2	7.640850057	0.007318706	3.63677274	-1.469652946	2.500715787	-5.106425686	-1.136056953	3.970368733	9.961963646	0.0045896	0.172516568	0.031490476	0.000465234	0.282523876	0.002661584	-2.426854816	-5.156022346	-4.402267672	-0.188243446	-5.652543133	-1.932403016	10.79493529	0.001794368

ALMS1	0.006900941	-0.079220452	-0.025893695	-0.086121393	-0.032794636	0.053326758	8	0.055672826	0.816496581	0.816496581	0.816496581	0.816496581	0.816496581	0.816496581	15	85	0	A_23_P312179	A_23_P312179	A_23_P312179	chr2	73600685	73600745	+	ALMS1	Alstrom syndrome 1 [Source:HGNC Symbol;Acc:HGNC:428]	ENSG00000116127	protein_coding	exon	coding exon	1	2465	2:73600685:73600745	ENSG00000116127	ALMS1	5.961464413	0.004695722	0.123339663	-1.415897276	-0.462794778	-1.539236939	-0.586134441	0.953102498	9.961963646	0.904291099	0.1873027	0.653455566	0.154883175	0.570825885	0.363091425	-6.990065264	-5.223071549	-6.931404072	-5.988063762	-6.117136368	-6.596603459	0.97589418	0.442329973

ZNF784	-0.037387587	-0.105218948	0.020839416	-0.067831361	0.058227002	0.126058364	8	0.111615375	0.816496581	0.816496581	0.816496581	0.816496581	0.816496581	0.816496581	15	86	0	P_126855	P_126855	P_126855	chr19	55621595	55621655	-	ZNF784	zinc finger protein 784 [Source:HGNC Symbol;Acc:HGNC:33111]	ENSG00000179922	protein_coding	3'UTR	3'UTR	1	2466	19:55621595:55621655	ENSG00000179922	ZNF784	8.908721932	0.012211131	-0.414376502	-1.1661694	0.230968751	-0.751792899	0.645345252	1.397138151	9.961963646	0.687379751	0.270705572	0.822013556	0.469564781	0.533284186	0.192711452	-6.905465546	-5.511924089	-7.018065802	-6.849029045	-6.079825114	-6.098213793	0.753110598	0.545328239

TEN1	0.175401278	0.11829491	0.160559289	-0.057106368	-0.014841989	0.042264379	8	0.097312298	0.816496581	0.816496581	0.816496581	0.816496581	0.816496581	0.816496581	15	87	0	P_122641	P_122641	P_122641	chr17	76000432	76000492	+	TEN1	TEN1 CST complex subunit [Source:HGNC Symbol;Acc:HGNC:37242]	ENSG00000257949	protein_coding	3'UTR	3'UTR	1	2467	17:76000432:76000492	ENSG00000257949	TEN1	11.7963336	0.009811356	2.168771842	1.46267275	1.985256141	-0.706099092	-0.183515701	0.522583391	9.961963646	0.055385407	0.174377932	0.075321338	0.496307599	0.858074811	0.612687223	-4.899841412	-5.164818075	-5.236120876	-6.88360193	-6.278803588	-6.924209161	1.931595361	0.188718142

ARHGAP28	-0.268383445	0.395541434	-0.362100122	0.663924879	-0.093716677	-0.757641556	8	0.069935026	0.816496581	0.816496581	0.816496581	0.816496581	0.816496581	0.816496581	15	88	0	P_230518	P_230518	P_230518	chr18	6890455	6890515	+	ARHGAP28	Rho GTPase activating protein 28 [Source:HGNC Symbol;Acc:HGNC:25509]	ENSG00000088756	protein_coding	exon	coding exon	1	2468	18:6890455:6890515	ENSG00000088756	ARHGAP28	7.743844473	0.00613435	-4.196788535	6.185194315	-5.662262964	10.38198285	-1.465474429	-11.84745728	9.961963646	0.001853444	0.000105138	0.000211878	1.16E-06	0.173628755	3.41E-07	-1.496647546	1.64459917	0.713169326	6.040050734	-5.265844066	7.283950845	56.37105196	1.49E-06

ATAD5	-0.411011041	0.002021079	-0.295820064	0.41303212	0.115190977	-0.297841143	8	0.053798993	0.816496581	0.816496581	0.816496581	0.816496581	0.816496581	0.816496581	15	89	0	P_228276	P_228276	P_228276	chr17	30855267	30855327	+	ATAD5	"ATPase family, AAA domain containing 5 [Source:HGNC Symbol;Acc:HGNC:25752]"	ENSG00000176208	protein_coding	exon	coding exon	1	2469	17:30855267:30855327	ENSG00000176208	ATAD5	7.692219104	0.00453099	-7.478294468	0.036773282	-5.382409054	7.51506775	2.095885414	-5.419182335	9.961963646	2.16E-05	0.971392228	0.000313104	2.07E-05	0.062612432	0.000297255	3.110132165	-6.181374594	0.306768802	3.065935791	-4.374287029	0.342117996	29.1888473	3.00E-05

DXO	-0.087013273	0.091218161	-0.019994182	0.178231434	0.067019092	-0.111212342	8	0.118944074	0.816496581	0.816496581	0.816496581	0.816496581	0.816496581	0.816496581	15	90	0	P_142213	P_142213	P_142213	chr6	31970279	31970339	-	DXO	decapping exoribonuclease [Source:HGNC Symbol;Acc:HGNC:2992]	ENSG00000204348	protein_coding	exon	non-coding exon	1	2470	6:31970279:31970339	ENSG00000204348	DXO	9.12532279	0.013568053	-0.914897361	0.959109474	-0.210227972	1.874006834	0.704669388	-1.169337446	9.961963646	0.381857084	0.360202511	0.837727196	0.090523532	0.497159301	0.269485267	-6.559922218	-5.719127884	-7.023038601	-5.506141224	-6.039155482	-6.371731408	1.196155764	0.360578818

SPHKAP	0.076551281	0.297627595	0.209361241	0.221076315	0.13280996	-0.088266355	8	0.157945231	0.816496581	0.816496581	0.816496581	0.816496581	0.816496581	0.816496581	15	92	0	A_23_P428485	A_23_P428485	A_23_P428485	chr2	227980594	227980654	-	SPHKAP	"SPHK1 interactor, AKAP domain containing [Source:HGNC Symbol;Acc:HGNC:30619]"	ENSG00000153820	protein_coding	3'UTR	3'UTR	1	2472	2:227980594:227980654	ENSG00000153820	SPHKAP	3.542471555	0.022240241	0.628678118	2.444269454	1.719381176	1.815591335	1.090703058	-0.724888278	9.961963646	0.543704489	0.034680848	0.116404283	0.099604977	0.301081394	0.485198449	-6.786885633	-3.74277348	-5.634851796	-5.5938095	-5.700116183	-6.79168263	2.389571507	0.130022568

CEL	0.103682996	0.117711265	0.143575239	0.014028269	0.039892243	0.025863974	8	0.093660732	0.816496581	0.816496581	0.816496581	0.816496581	0.816496581	0.816496581	15	93	0	A_23_P123794	A_23_P123794	A_23_P123794	chr9	133070502	133070562	+	CEL	carboxyl ester lipase [Source:HGNC Symbol;Acc:HGNC:1848]	ENSG00000170835	protein_coding	3'UTR;exon	multiple Ref	1	2473	9:133070502:133070562	ENSG00000170835	CEL	7.272046847	0.009251345	1.320233408	1.498860478	1.828195887	0.17862707	0.507962479	0.329335409	9.961963646	0.216286238	0.164913178	0.097577068	0.861810965	0.622536864	0.748721763	-6.122441504	-5.118936037	-5.47503765	-7.132191901	-6.16125007	-7.012447962	1.288382418	0.331412979

KIT	-0.019791497	-0.00578886	-0.00781077	0.014002637	0.011980727	-0.00202191	8	0.159294205	0.816496581	0.816496581	0.816496581	0.816496581	0.816496581	0.816496581	15	95	0	P_249012	P_249012	P_249012	chr4	54736549	54736609	+	KIT	v-kit Hardy-Zuckerman 4 feline sarcoma viral oncogene homolog [Source:HGNC Symbol;Acc:HGNC:6342]	ENSG00000157404	protein_coding	exon	coding exon	1	2475	4:54736549:54736609	ENSG00000157404	KIT	3.401862852	0.022583907	-0.161296425	-0.047177957	-0.063656091	0.114118468	0.097640334	-0.016478133	9.961963646	0.875084174	0.963303789	0.950503249	0.911411082	0.924154468	0.987178297	-6.984181953	-6.180914203	-7.044908164	-7.142495886	-6.29161695	-7.07123019	0.009187097	0.998707984

SLC6A5	0.060017562	-0.034682177	0.081363723	-0.094699739	0.021346162	0.1160459	8	0.210540662	0.816496581	0.816496581	0.816496581	0.816496581	0.816496581	0.816496581	15	96	0	P_211263	P_211263	P_211263	chr11	20614760	20614820	+	SLC6A5	"solute carrier family 6 (neurotransmitter transporter), member 5 [Source:HGNC Symbol;Acc:HGNC:11051]"	ENSG00000165970	protein_coding	3'UTR;exon	multiple Ref	1	2476	11:20614760:20614820	ENSG00000165970	SLC6A5	4.555391348	0.037803979	0.378055202	-0.218465681	0.512516303	-0.596520884	0.134461101	0.730981984	9.961963646	0.713320428	0.831476742	0.619460581	0.564136188	0.895715765	0.481629184	-6.920925583	-6.156989561	-6.905544589	-6.958432962	-6.287077865	-6.787082529	0.226929594	0.875532107

TXLNG	-0.124973695	0.054900004	0.008540503	0.179873699	0.133514198	-0.046359501	8	0.066845425	0.816496581	0.816496581	0.816496581	0.816496581	0.816496581	0.816496581	15	97	0	P_314504	P_314504	P_314504	chrX	16835237	16835297	-	TXLNG;RBBP7	taxilin gamma [Source:HGNC Symbol;Acc:HGNC:18578];retinoblastoma binding protein 7 [Source:HGNC Symbol;Acc:HGNC:9890]	ENSG00000086712	protein_coding	3'ext;intron;promoter	multiple non-Ref	0	2477	X:16835237:16835297	ENSG00000086712	TXLNG	8.778405845	0.005794981	-2.010658077	0.883266968	0.137405164	2.893925045	2.148063241	-0.745861804	9.961963646	0.072203716	0.397906137	0.893448015	0.016058931	0.057353133	0.472983336	-5.149588146	-5.786758155	-7.036819774	-3.833250683	-4.294374482	-6.775704872	3.064512385	0.078221277

CADM3	-0.036955365	0.095868656	0.065378539	0.132824021	0.102333904	-0.030490117	8	0.191462394	0.816496581	0.816496581	0.816496581	0.816496581	0.816496581	0.816496581	15	98	0	P_103674	P_103674	P_103674	chr1	159189801	159189861	+	CADM3	cell adhesion molecule 3 [Source:HGNC Symbol;Acc:HGNC:17601]	ENSG00000162706	protein_coding	exon	coding exon	1	2478	1:159189801:159189861	ENSG00000162706	CADM3	4.468699974	0.031644935	-0.254431583	0.660039862	0.450120443	0.914471445	0.704552026	-0.209919419	9.961963646	0.804337047	0.524194696	0.662260188	0.382070105	0.497229257	0.837961547	-6.963149914	-5.957507644	-6.937592457	-6.710585487	-6.039239136	-7.04735849	0.346619034	0.792457625

NCAPH	-0.629670904	-0.045116731	-0.382713186	0.584554173	0.246957718	-0.337596455	8	0.083326535	0.816496581	0.816496581	0.816496581	0.816496581	0.816496581	0.816496581	15	99	0	P_235789	P_235789	P_235789	chr2	96360626	96360686	+	NCAPH	"non-SMC condensin I complex, subunit H [Source:HGNC Symbol;Acc:HGNC:1112]"	ENSG00000121152	protein_coding	exon	coding exon	1	2479	2:96360626:96360686	ENSG00000121152	NCAPH	9.13941626	0.007782542	-8.7417529	-0.626357849	-5.313226451	8.115395051	3.428526449	-4.686868602	9.961963646	5.51E-06	0.545164373	0.000345428	1.06E-05	0.006489913	0.000867384	4.507044162	-5.979399801	0.204550461	3.75995534	-2.236348486	-0.772244185	34.10368516	1.50E-05

AACS	-0.206130186	0.013286302	-0.148415534	0.219416488	0.057714652	-0.161701836	8	0.05408647	0.816496581	0.816496581	0.816496581	0.816496581	0.816496581	0.816496581	15	100	0	P_218048	P_218048	P_218048	chr12	125076551	125076611	+	AACS	acetoacetyl-CoA synthetase [Source:HGNC Symbol;Acc:HGNC:21298]	ENSG00000081760	protein_coding	exon	coding exon	1	2480	12:125076551:125076611	ENSG00000081760	AACS	7.508657917	0.004555897	-3.740247132	0.241080906	-2.693010595	3.981328038	1.047236537	-2.934091501	9.961963646	0.003870474	0.814381753	0.022653587	0.002614968	0.319734048	0.014993374	-2.252727618	-6.151540961	-4.078733026	-1.983604354	-5.744287298	-3.691081985	7.809407487	0.005665052

NCOA7	0.311560979	-0.116319105	0.360883273	-0.427880084	0.049322294	0.477202378	8	0.074991229	0.816496581	0.816496581	0.816496581	0.816496581	0.816496581	0.816496581	15	101	0	P_259054	P_259054	P_259054	chr6	125928711	125928771	+	NCOA7	nuclear receptor coactivator 7 [Source:HGNC Symbol;Acc:HGNC:21081]	ENSG00000111912	protein_coding	3'UTR;exon	multiple Ref	1	2481	6:125928711:125928771	ENSG00000111912	NCOA7	6.472143075	0.006722809	4.653859792	-1.737485888	5.390598523	-6.39134568	0.736738731	7.128084411	9.961963646	0.000911929	0.113058152	0.000309498	8.06E-05	0.478272503	3.25E-05	-0.764254119	-4.800333359	0.318822903	1.646647907	-6.015828439	2.646098684	24.32291526	6.68E-05

SLITRK2	-0.18424795	-0.152140516	0.103129183	0.032107433	0.287377133	0.2552697	8	0.398125894	0.816496581	0.816496581	0.816496581	0.816496581	0.816496581	0.816496581	15	102	0	A_24_P66605	A_24_P66605	A_24_P66605	chrX	145823559	145823619	+	SLITRK2	"SLIT and NTRK-like family, member 2 [Source:HGNC Symbol;Acc:HGNC:13449]"	ENSG00000185985	protein_coding	exon	coding exon	1	2482	X:145823559:145823619	ENSG00000185985	SLITRK2	3.479524777	0.129494221	-0.627080215	-0.517803905	0.350995876	0.10927631	0.978076092	0.868799782	9.961963646	0.544709628	0.615898251	0.732900917	0.915153208	0.351190637	0.405401434	-6.787938585	-6.04268848	-6.980256538	-7.143086694	-5.811598887	-6.674001802	0.4179893	0.744039474

CLEC7A	-0.575434686	0.095315906	-0.580628063	0.670750593	-0.005193376	-0.675943969	8	0.179205968	0.816496581	0.816496581	0.816496581	0.816496581	0.816496581	0.816496581	15	103	0	A_23_P2640	A_23_P2640	A_23_P2640	chr12	10118318	10118378	-	CLEC7A	"C-type lectin domain family 7, member A [Source:HGNC Symbol;Acc:HGNC:14558]"	ENSG00000172243	protein_coding	3'UTR;exon	multiple Ref	1	2483	12:10118318:10118378	ENSG00000172243	CLEC7A	5.637129208	0.027996602	-4.212006192	0.697683329	-4.250020121	4.909689522	-0.038013928	-4.94770345	9.961963646	0.00180935	0.501333963	0.001703965	0.000621104	0.97042757	0.000587121	-1.471839236	-5.931806141	-1.451304567	-0.489996102	-6.295916707	-0.366595771	14.14611582	0.000635419

CNIH1	0.0345761	0.085807517	0.117413329	0.051231417	0.082837229	0.031605812	8	0.060809486	0.816496581	0.816496581	0.816496581	0.816496581	0.816496581	0.816496581	15	105	0	P_114890	P_114890	P_114890	chr14	54427681	54427741	-	CNIH1	cornichon family AMPA receptor auxiliary protein 1 [Source:HGNC Symbol;Acc:HGNC:19431]	ENSG00000100528	protein_coding	3'UTR	3'UTR	1	2485	14:54427681:54427741	ENSG00000100528	CNIH1	10.36834055	0.005176214	0.588593592	1.460712887	1.99874286	0.872119294	1.410149267	0.538029973	9.961963646	0.569238052	0.174903661	0.073650618	0.403673051	0.188946095	0.602368584	-6.812548638	-5.167282964	-5.215198219	-6.748875934	-5.335437688	-6.915506659	1.585614474	0.254027203

KIDINS220	0.142886869	-0.253711479	-0.022279231	-0.396598348	-0.1651661	0.231432247	8	0.071438744	0.816496581	0.816496581	0.816496581	0.816496581	0.816496581	0.816496581	15	106	0	P_233510	P_233510	P_233510	chr2	8813246	8813306	-	KIDINS220	"kinase D-interacting substrate, 220kDa [Source:HGNC Symbol;Acc:HGNC:29508]"	ENSG00000134313	protein_coding	3'UTR;exon	multiple Ref	1	2486	2:8813246:8813306	ENSG00000134313	KIDINS220	8.318022609	0.006305068	2.203905961	-3.913279395	-0.343637812	-6.117185355	-2.547543772	3.569641583	9.961963646	0.052194537	0.00291891	0.738261	0.000114929	0.029065193	0.005129393	-4.84341196	-1.420548354	-6.983014148	1.274787655	-3.664569814	-2.605565931	12.8235535	0.000933266

NAT10	-0.043986468	0.118464449	-0.084963511	0.162450917	-0.040977043	-0.20342796	8	0.140311052	0.816496581	0.816496581	0.816496581	0.816496581	0.816496581	0.816496581	15	109	0	P_211504	P_211504	P_211504	chr11	34134360	34134420	+	NAT10	N-acetyltransferase 10 (GCN5-related) [Source:HGNC Symbol;Acc:HGNC:29830]	ENSG00000135372	protein_coding	exon	coding exon	1	2489	11:34134360:34134420	ENSG00000135372	NAT10	7.437647476	0.018016572	-0.401354963	1.080930044	-0.775250396	1.482285008	-0.373895433	-1.85618044	9.961963646	0.696633074	0.305200803	0.456199905	0.169191682	0.716316754	0.093210195	-6.911165926	-5.601057431	-6.728491588	-6.064518037	-6.222862768	-5.45673822	1.285480202	0.332289666

SMAD2	0.016186435	0.071505253	0.057776294	0.055318818	0.041589859	-0.013728959	8	0.068368319	0.816496581	0.816496581	0.816496581	0.816496581	0.816496581	0.816496581	15	110	0	P_123541	P_123541	P_123541	chr18	47879236	47879296	+	SMAD2;RP11-380M21.4	SMAD family member 2 [Source:HGNC Symbol;Acc:HGNC:6768];	ENSG00000175387	protein_coding;antisense	intron	multiple non-Ref	0	2490	18:47879236:47879296	ENSG00000175387	SMAD2	8.334535519	0.005960343	0.256780006	1.134352271	0.916557417	0.877572266	0.659777412	-0.217794854	9.961963646	0.802573955	0.283204845	0.381027613	0.400844919	0.524356229	0.831985286	-6.962499092	-5.545797982	-6.606802366	-6.744032783	-6.070231024	-7.045526773	0.574273743	0.644833699

EHD2	0.063321923	-0.069934037	0.071721655	-0.13325596	0.008399732	0.141655692	8	0.190688328	0.816496581	0.816496581	0.816496581	0.816496581	0.816496581	0.816496581	15	111	0	P_126222	P_126222	P_126222	chr19	47741142	47741202	+	EHD2	EH-domain containing 2 [Source:HGNC Symbol;Acc:HGNC:3243]	ENSG00000024422	protein_coding	exon	coding exon	1	2491	19:47741142:47741202	ENSG00000024422	EHD2	5.686835157	0.031407384	0.437606581	-0.483301729	0.49565564	-0.920908309	0.058049059	0.978957368	9.961963646	0.671006645	0.639327704	0.63088871	0.3788597	0.954857436	0.350775949	-6.894860341	-6.060431237	-6.914594946	-6.704631401	-6.294893275	-6.571668179	0.413425655	0.747087156

SOX11	0.259225928	-0.016492905	0.229828183	-0.275718832	-0.029397745	0.246321087	8	0.07610845	0.816496581	0.816496581	0.816496581	0.816496581	0.816496581	0.816496581	15	112	0	A_24_P302584	A_24_P302584	A_24_P302584	chr2	5701295	5701355	+	SOX11	SRY (sex determining region Y)-box 11 [Source:HGNC Symbol;Acc:HGNC:11191]	ENSG00000176887	protein_coding	exon	coding exon	1	2492	2:5701295:5701355	ENSG00000176887	SOX11	7.441738908	0.006858374	3.83365898	-0.243911448	3.398899506	-4.077570429	-0.434759474	3.642810954	9.961963646	0.003322108	0.812249105	0.0068202	0.002240609	0.673003888	0.004544035	-2.09631762	-6.150821689	-2.873967908	-1.823894946	-6.197123468	-2.481658787	9.399103313	0.002973312

ITGB1	0.213430275	-0.03256007	0.206248766	-0.245990345	-0.007181508	0.238808837	8	0.068281504	0.816496581	0.816496581	0.816496581	0.816496581	0.816496581	0.816496581	15	113	0	P_105785	P_105785	P_105785	chr10	32953592	32953652	-	ITGB1	"integrin, beta 1 (fibronectin receptor, beta polypeptide, antigen CD29 includes MDF2, MSK12) [Source:HGNC Symbol;Acc:HGNC:6153]"	ENSG00000150093	protein_coding	5'UTR	5'UTR	1	2493	10:32953592:32953652	ENSG00000150093	ITGB1	9.344110657	0.005950816	3.388545962	-0.51694304	3.274528064	-3.905489002	-0.114017898	3.791471104	9.961963646	0.006939677	0.616477518	0.008407524	0.002955996	0.911488781	0.003558968	-2.847612317	-6.043145272	-3.086240282	-2.110132559	-6.289775345	-2.231187087	8.685517574	0.003930981

PDCD5	-0.219095824	-0.092224844	0.037890812	0.12687098	0.256986636	0.130115656	8	0.1165749	0.816496581	0.816496581	0.816496581	0.816496581	0.816496581	0.816496581	15	114	0	P_125352	P_125352	P_125352	chr19	32585324	32585384	+	PDCD5	programmed cell death 5 [Source:HGNC Symbol;Acc:HGNC:8764]	ENSG00000105185	protein_coding	intron;promoter	multiple non-Ref	0	2494	19:32585324:32585384	ENSG00000105185	PDCD5	8.328886582	0.01311996	-2.342682731	-0.986114408	0.405147617	1.356568323	2.747830348	1.391262025	9.961963646	0.041242316	0.347421475	0.693932471	0.204862896	0.020622403	0.194432753	-4.617704112	-5.693937292	-6.95821548	-6.226617428	-3.341123219	-6.105693241	2.991864643	0.082437664

COL12A1	-0.062422017	-0.03343143	0.031540063	0.028990587	0.09396208	0.064971493	8	0.072298848	0.816496581	0.816496581	0.816496581	0.816496581	0.816496581	0.816496581	15	115	0	P_257851	P_257851	P_257851	chr6	75181071	75181131	-	COL12A1	"collagen, type XII, alpha 1 [Source:HGNC Symbol;Acc:HGNC:2188]"	ENSG00000111799	protein_coding	5'UTR;exon	multiple Ref	1	2495	6:75181071:75181131	ENSG00000111799	COL12A1	3.620684886	0.006404349	-0.955313529	-0.511638345	0.482692652	0.443675184	1.438006181	0.994330996	9.961963646	0.362026137	0.620053078	0.63974518	0.6667585	0.181094867	0.343599555	-6.522031951	-6.045944178	-6.921357195	-7.043019767	-5.300611507	-6.556580997	0.776798918	0.533302612

CFAP69	0.330319377	0.028972194	-0.027855714	-0.301347183	-0.358175091	-0.056827908	8	0.075954087	0.816496581	0.816496581	0.816496581	0.816496581	0.816496581	0.816496581	15	117	0	P_261688	P_261688	P_261688	chr7	90286339	90286399	+	CFAP69	cilia and flagella associated protein 69 [Source:HGNC Symbol;Acc:HGNC:26107]	ENSG00000105792	protein_coding	3'UTR;exon	multiple Ref	1	2497	7:90286339:90286399	ENSG00000105792	CFAP69	8.441903381	0.006839524	4.891777941	0.429056093	-0.412521868	-4.462721848	-5.304299809	-0.841577961	9.961963646	0.000637836	0.677012829	0.688694427	0.001222435	0.000349852	0.419767573	-0.394115656	-6.085963666	-6.954977344	-1.195424975	0.563149711	-6.697685188	12.17391518	0.001140133

SMARCD3	0.582788878	-0.068915305	0.335137581	-0.651704183	-0.247651297	0.404052887	8	0.065321533	0.816496581	0.816496581	0.816496581	0.816496581	0.816496581	0.816496581	15	118	0	P_263395	P_263395	P_263395	chr7	151239642	151239702	-	SMARCD3	"SWI/SNF related, matrix associated, actin dependent regulator of chromatin, subfamily d, member 3 [Source:HGNC Symbol;Acc:HGNC:11108]"	ENSG00000082014	protein_coding	3'UTR;exon	multiple Ref	1	2498	7:151239642:151239702	ENSG00000082014	SMARCD3	10.10919112	0.00563324	9.509939983	-1.1245589	5.468769915	-10.63449888	-4.041170069	6.593328814	9.961963646	2.59E-06	0.287141914	0.00027723	9.31E-07	0.002375107	6.24E-05	5.272421144	-5.556081274	0.433393584	6.265144002	-1.268293204	1.9684323	49.08444041	2.83E-06

MBNL2	0.297674456	0.090611645	0.251832459	-0.207062812	-0.045841998	0.161220814	8	0.126552147	0.816496581	0.816496581	0.816496581	0.816496581	0.816496581	0.816496581	15	120	0	P_219649	P_219649	P_219649	chr13	97327245	97327305	-	MBNL2	muscleblind-like splicing regulator 2 [Source:HGNC Symbol;Acc:HGNC:16746]	ENSG00000139793	protein_coding	intron	intron antisense	0	2500	13:97327245:97327305	ENSG00000139793	MBNL2	4.060073079	0.01506796	2.970024051	0.904070731	2.512639036	-2.065953319	-0.457385014	1.608568305	9.961963646	0.014100961	0.387298156	0.030854588	0.065834006	0.657206908	0.138906143	-3.561980426	-5.768671828	-4.382343025	-5.209336169	-6.186604573	-5.815852392	3.836099639	0.046132385

SMG9	-0.183804342	0.110334815	-0.075375792	0.294139157	0.10842855	-0.185710607	8	0.072291547	0.816496581	0.816496581	0.816496581	0.816496581	0.816496581	0.816496581	15	121	0	A_23_P56127	A_23_P56127	A_23_P56127	chr19	43733327	43733387	-	SMG9	SMG9 nonsense mediated mRNA decay factor [Source:HGNC Symbol;Acc:HGNC:25763]	ENSG00000105771	protein_coding	exon	coding exon	1	2501	19:43733327:43733387	ENSG00000105771	SMG9	7.351123761	0.006403501	-2.813148401	1.688688118	-1.153635907	4.501836518	1.659512494	-2.842324025	9.961963646	0.018439484	0.122282613	0.275576307	0.001150799	0.128117247	0.0175411	-3.82927124	-4.867583846	-6.365263036	-1.13262647	-5.009052517	-3.847759005	7.199277817	0.007429004

SYDE2	0.05197178	0.044387792	0.048231951	-0.007583988	-0.003739829	0.003844159	8	0.11130233	0.816496581	0.816496581	0.816496581	0.816496581	0.816496581	0.816496581	15	122	0	A_24_P915300	A_24_P915300	A_24_P915300	chr1	85182956	85183016	-	SYDE2	"synapse defective 1, Rho GTPase, homolog 2 (C. elegans) [Source:HGNC Symbol;Acc:HGNC:25841]"	ENSG00000097096	protein_coding	exon	coding exon	1	2502	1:85182956:85183016	ENSG00000097096	SYDE2	6.032316721	0.012155091	0.577343282	0.493094395	0.535798328	-0.084248887	-0.041544954	0.042703933	9.961963646	0.57652207	0.632633791	0.603853767	0.934527551	0.967682321	0.966781349	-6.819468478	-6.055513385	-6.892576288	-7.145732864	-6.295767312	-7.070382364	0.145699113	0.930089459

AMACR	0.211819267	0.108716686	0.243865997	-0.10310258	0.032046731	0.135149311	8	0.09313103	0.816496581	0.816496581	0.816496581	0.816496581	0.816496581	0.816496581	15	123	0	P_139311	P_139311	P_139311	chr5	33997130	33997190	-	AMACR;RP11-1084J3.3	alpha-methylacyl-CoA racemase [Source:HGNC Symbol;Acc:HGNC:451];	ENSG00000242110	protein_coding;processed_pseudogene	3'UTR;exon	multiple Ref	1	2503	5:33997130:33997190	ENSG00000242110	AMACR	6.579125409	0.009171888	2.708829777	1.390312608	3.118656228	-1.318517168	0.409826452	1.72834362	9.961963646	0.0220477	0.194712087	0.010947307	0.216838681	0.69060704	0.114736756	-4.006228616	-5.254415321	-3.352834601	-6.273755937	-6.208120283	-5.645564814	3.981726915	0.04199299

CCDC150	-0.387129002	-0.031406078	-0.289796847	0.355722924	0.097332155	-0.258390769	8	0.062938385	0.816496581	0.816496581	0.816496581	0.816496581	0.816496581	0.816496581	15	125	0	P_238436	P_238436	P_238436	chr2	196674288	196674348	+	CCDC150	coiled-coil domain containing 150 [Source:HGNC Symbol;Acc:HGNC:26834]	ENSG00000144395	protein_coding	3'UTR;exon	multiple Ref	1	2505	2:196674288:196674348	ENSG00000144395	CCDC150	5.366314994	0.005387776	-6.459465346	-0.524028086	-4.835423545	5.935437259	1.624041801	-4.311395459	9.961963646	7.39E-05	0.611718312	0.000693688	0.00014625	0.135546495	0.001547258	1.841104113	-6.039364446	-0.520234885	1.022473919	-5.057373262	-1.372317136	20.30594615	0.000144556

PRKG1	-0.017051138	-0.161395661	0.025412618	-0.144344524	0.042463756	0.18680828	8	0.085480318	0.816496581	0.816496581	0.816496581	0.816496581	0.816496581	0.816496581	15	130	0	P_208252	P_208252	P_208252	chr10	52133845	52133905	+	PRKG1	"protein kinase, cGMP-dependent, type I [Source:HGNC Symbol;Acc:HGNC:9414]"	ENSG00000185532	protein_coding	exon	coding exon	1	2510	10:52133845:52133905	ENSG00000185532	PRKG1	3.802853108	0.008074511	-0.232402546	-2.199780625	0.346367335	-1.967378079	0.578769881	2.54614796	9.961963646	0.820930241	0.052559818	0.736270905	0.077591103	0.575595607	0.029134735	-6.968967227	-4.122328792	-6.981997847	-5.363335142	-6.121544152	-4.349250891	2.616906098	0.108996309

ZFPM2	0.194907699	0.107970952	0.162897683	-0.086936746	-0.032010015	0.054926731	8	0.149318121	0.816496581	0.816496581	0.816496581	0.816496581	0.816496581	0.816496581	15	132	0	P_265815	P_265815	P_265815	chr8	105798888	105798948	+	ZFPM2	"zinc finger protein, FOG family member 2 [Source:HGNC Symbol;Acc:HGNC:16700]"	ENSG00000169946	protein_coding	exon	coding exon	1	2512	8:105798888:105798948	ENSG00000169946	ZFPM2	3.742753459	0.020111509	1.683264249	0.932460057	1.406818965	-0.750804192	-0.276445284	0.474358907	9.961963646	0.123348839	0.373146476	0.189903859	0.470133552	0.787856658	0.645470538	-5.639163832	-5.743420998	-6.061969586	-6.849797658	-6.256197699	-6.949828715	1.091190865	0.397275548

VGLL2	0.010912293	0.113316033	-0.056114392	0.10240374	-0.067026685	-0.169430425	8	0.110926113	0.816496581	0.816496581	0.816496581	0.816496581	0.816496581	0.816496581	15	133	0	A_32_P106376	A_32_P106376	A_32_P106376	chr6	117273013	117273073	+	VGLL2	vestigial-like family member 2 [Source:HGNC Symbol;Acc:HGNC:20232]	ENSG00000170162	protein_coding	3'UTR	3'UTR	1	2513	6:117273013:117273073	ENSG00000170162	VGLL2	4.624126509	0.012087951	0.121558493	1.262294382	-0.625091438	1.140735889	-0.746649931	-1.887385821	9.961963646	0.905665725	0.235592806	0.545962131	0.280661376	0.472528166	0.088554557	-6.990302938	-5.405406444	-6.837842655	-6.481035505	-6.008431386	-5.40964187	1.236648768	0.347435568

PRKX	0.02560937	-0.06993425	-0.046882903	-0.09554362	-0.072492273	0.023051347	8	0.074201305	0.816496581	0.816496581	0.816496581	0.816496581	0.816496581	0.816496581	15	134	0	P_150416	P_150416	P_150416	chrX	3615814	3615874	-	PRKX	"protein kinase, X-linked [Source:HGNC Symbol;Acc:HGNC:9441]"	ENSG00000183943	protein_coding	exon	coding exon	1	2514	X:3615814:3615874	ENSG00000183943	PRKX	9.389398231	0.006628169	0.385254527	-1.052055808	-0.705282895	-1.437310335	-1.090537422	0.346772913	9.961963646	0.708146773	0.31762373	0.496793713	0.181287531	0.301150852	0.735975373	-6.917970404	-5.630058908	-6.782085349	-6.123593744	-5.700287172	-7.006080851	0.854675127	0.495613058

SLC20A1	-0.272053603	-0.049056128	-0.225084076	0.222997475	0.046969527	-0.176027948	8	0.068926003	0.816496581	0.816496581	0.816496581	0.816496581	0.816496581	0.816496581	15	137	0	P_128705	P_128705	P_128705	chr2	112663115	112663175	+	SLC20A1	"solute carrier family 20 (phosphate transporter), member 1 [Source:HGNC Symbol;Acc:HGNC:10946]"	ENSG00000144136	protein_coding	3'UTR;exon	multiple Ref	1	2517	2:112663115:112663175	ENSG00000144136	SLC20A1	8.913209711	0.00602183	-4.293741043	-0.774238271	-3.552434978	3.519502772	0.741306065	-2.778196707	9.961963646	0.001590712	0.456771419	0.005278095	0.005575306	0.475619895	0.019576982	-1.339071337	-5.875601656	-2.612916287	-2.761997512	-6.012430639	-3.956976532	8.718377117	0.003879421

DIP2B	0.129540845	0.006063286	0.015022552	-0.123477559	-0.114518293	0.008959266	8	0.085307936	0.816496581	0.816496581	0.816496581	0.816496581	0.816496581	0.816496581	15	138	0	P_215422	P_215422	P_215422	chr12	50708473	50708533	+	DIP2B	DIP2 disco-interacting protein 2 homolog B (Drosophila) [Source:HGNC Symbol;Acc:HGNC:29284]	ENSG00000066084	protein_coding	3'UTR;exon	multiple Ref	1	2518	12:50708473:50708533	ENSG00000066084	DIP2B	6.969615795	0.008050868	1.768198405	0.082762253	0.205053877	-1.685436152	-1.563144528	0.122291624	9.961963646	0.10758147	0.935679936	0.841659195	0.122920884	0.149199673	0.905099888	-5.516324474	-6.178477807	-7.024206937	-5.783987113	-5.138940344	-7.063214594	1.41241556	0.29626555

RAF1	-0.226313589	-0.150812241	-0.247028285	0.075501348	-0.020714696	-0.096216044	8	0.109051407	0.816496581	0.816496581	0.816496581	0.816496581	0.816496581	0.816496581	15	139	0	P_243263	P_243263	P_243263	chr3	12608765	12608825	-	RAF1	"Raf-1 proto-oncogene, serine/threonine kinase [Source:HGNC Symbol;Acc:HGNC:9829]"	ENSG00000132155	protein_coding	3'UTR;exon	multiple Ref	1	2519	3:12608765:12608825	ENSG00000132155	RAF1	8.915211243	0.011756777	-2.556301813	-1.703484124	-2.79028253	0.852817689	-0.233980717	-1.086798406	9.961963646	0.028632592	0.119416177	0.019175876	0.413794331	0.819738299	0.302722079	-4.263166921	-4.847298502	-3.913651354	-6.765810492	-6.267648446	-6.461847357	3.197564913	0.071142717

UNC5B-AS1	-0.072403223	-0.623409395	-0.99020337	-0.551006172	-0.917800147	-0.366793975	8	0.384651034	0.816496581	0.816496581	0.816496581	0.816496581	0.816496581	0.816496581	15	140	0	A_32_P52153	A_32_P52153	A_32_P52153	chr10	71217248	71217308	-	UNC5B-AS1	UNC5B antisense RNA 1 [Source:HGNC Symbol;Acc:HGNC:45096]	ENSG00000237512	antisense	exon	non-coding exon	1	2520	10:71217248:71217308	ENSG00000237512	UNC5B-AS1	2.746350479	0.121023755	-0.254899051	-2.194742939	-3.486058875	-1.939843888	-3.231159824	-1.291315936	9.961963646	0.803986001	0.053009225	0.005894933	0.081212824	0.009046615	0.225752023	-6.963020834	-4.130033496	-2.725611219	-5.405773143	-2.555347264	-6.229579824	5.484226924	0.017384084

ATP6V1E2	0.051949319	0.023623041	-0.073764681	-0.028326278	-0.125713999	-0.097387722	8	0.083353912	0.816496581	0.816496581	0.816496581	0.816496581	0.816496581	0.816496581	15	141	0	A_16_P00366052	A_16_P00366052	A_16_P00366052	chr2	46508002	46508062	+	ATP6V1E2	"ATPase, H+ transporting, lysosomal 31kDa, V1 subunit E2 [Source:HGNC Symbol;Acc:HGNC:18125]"	ENSG00000250565	protein_coding	3'ext;intron	multiple non-Ref	0	2521	2:46508002:46508062	ENSG00000250565	ATP6V1E2	9.428072113	0.007786206	0.721045276	0.327882688	-1.023837766	-0.393162589	-1.744883043	-1.351720454	9.961963646	0.487457905	0.749787324	0.330130857	0.702481711	0.111716347	0.206357165	-6.721865345	-6.125729076	-6.503032011	-7.065733223	-4.890458446	-6.15546758	1.116999826	0.387887225

LIFR	0.123050725	-0.008393463	0.076898449	-0.131444189	-0.046152276	0.085291912	8	0.059894782	0.816496581	0.816496581	0.816496581	0.816496581	0.816496581	0.816496581	15	142	0	P_252550	P_252550	P_252550	chr5	38511789	38511849	-	LIFR	leukemia inhibitory factor receptor alpha [Source:HGNC Symbol;Acc:HGNC:6597]	ENSG00000113594	protein_coding	exon	coding exon	1	2522	5:38511789:38511849	ENSG00000113594	LIFR	7.705603177	0.00508755	2.112883146	-0.144122735	1.320410231	-2.257005881	-0.792472914	1.464532966	9.961963646	0.060850468	0.888277416	0.216229386	0.047706174	0.44654635	0.173880194	-4.988939248	-6.171149227	-6.170257462	-4.902848735	-5.973095414	-6.010947419	2.3492459	0.134232987

NECAB3	-0.035491119	0.120690895	0.035919426	0.156182014	0.071410545	-0.084771469	8	0.083041439	0.816496581	0.816496581	0.816496581	0.816496581	0.816496581	0.816496581	15	143	0	P_130987	P_130987	P_130987	chr20	33660192	33660252	-	NECAB3	N-terminal EF-hand calcium binding protein 3 [Source:HGNC Symbol;Acc:HGNC:15851]	ENSG00000125967	protein_coding	3'UTR;exon	multiple Ref	1	2523	20:33660192:33660252	ENSG00000125967	NECAB3	8.868014353	0.007744452	-0.493935195	1.67967318	0.499896005	2.173608375	0.993831199	-1.179777176	9.961963646	0.632060658	0.124059376	0.628004807	0.05493527	0.343831144	0.265494832	-6.866843928	-4.879897253	-6.912345907	-5.037815244	-5.796592359	-6.359975006	1.736552999	0.222764694

SNRPA1	-0.236982433	-0.168820607	-0.354377679	0.068161826	-0.117395246	-0.185557072	8	0.130621891	0.816496581	0.816496581	0.816496581	0.816496581	0.816496581	0.816496581	15	144	0	P_117642	P_117642	P_117642	chr15	101286296	101286356	-	SNRPA1	small nuclear ribonucleoprotein polypeptide A' [Source:HGNC Symbol;Acc:HGNC:11152]	ENSG00000131876	protein_coding	exon	non-coding exon	1	2524	15:101286296:101286356	ENSG00000131876	SNRPA1	5.668463251	0.015908463	-2.301164511	-1.639294457	-3.441104591	0.661870054	-1.13994008	-1.801810134	9.961963646	0.044260451	0.132305908	0.006354731	0.52306907	0.280977475	0.101866543	-4.685668862	-4.934611778	-2.802080569	-6.91519499	-5.648401037	-5.537881325	4.134650057	0.038113292

INTS10	-0.095352669	0.030989787	-0.128045473	0.126342456	-0.032692804	-0.15903526	8	0.040648636	0.816496581	0.816496581	0.816496581	0.816496581	0.816496581	0.816496581	15	145	0	P_263952	P_263952	P_263952	chr8	19823331	19823391	+	INTS10	integrator complex subunit 10 [Source:HGNC Symbol;Acc:HGNC:25548]	ENSG00000104613	protein_coding	3'UTR;exon	multiple Ref	1	2525	8:19823331:19823391	ENSG00000104613	INTS10	10.28071228	0.00353358	-1.964585806	0.638493875	-2.638167568	2.603079681	-0.673581762	-3.276661443	9.961963646	0.077951358	0.537553507	0.024885966	0.02642801	0.515900076	0.00837731	-5.220917994	-5.971633	-4.171438273	-4.327975077	-6.0608731	-3.104793845	4.865574393	0.024548579

EVI5L	-0.016025004	0.140752475	0.07662156	0.156777479	0.092646564	-0.064130915	8	0.067208785	0.816496581	0.816496581	0.816496581	0.816496581	0.816496581	0.816496581	15	146	0	A_23_P101427	A_23_P101427	A_23_P101427	chr19	7864172	7864232	+	EVI5L	ecotropic viral integration site 5-like [Source:HGNC Symbol;Acc:HGNC:30464]	ENSG00000142459	protein_coding	3'UTR	3'UTR	1	2526	19:7864172:7864232	ENSG00000142459	EVI5L	9.002627268	0.005834098	-0.256954903	2.256912953	1.228597877	2.513867855	1.485552779	-1.028315076	9.961963646	0.802442696	0.047713692	0.247455786	0.030789779	0.168340658	0.328122074	-6.962450386	-4.034582851	-6.279996778	-4.478021634	-5.240167937	-6.52255118	2.708827531	0.101649754

MPDZ	0.089871892	-0.017982709	0.114470798	-0.1078546	0.024598907	0.132453507	8	0.05099554	0.816496581	0.816496581	0.816496581	0.816496581	0.816496581	0.816496581	15	147	0	P_266809	P_266809	P_266809	chr9	13136721	13136781	-	MPDZ	multiple PDZ domain protein [Source:HGNC Symbol;Acc:HGNC:7208]	ENSG00000107186	protein_coding	3'UTR;exon	multiple Ref	1	2527	9:13136721:13136781	ENSG00000107186	MPDZ	6.41794562	0.004295063	1.679518218	-0.33605932	2.139220477	-2.015577538	0.459702259	2.475279797	9.961963646	0.12409012	0.743797081	0.058213648	0.071614195	0.655598809	0.032890609	-5.644506009	-6.122899789	-4.99391155	-5.288426477	-6.185498394	-4.467781956	2.985145101	0.082841089

LEF1	0.25714521	-0.131662483	0.148876275	-0.388807693	-0.108268935	0.280538758	8	0.087542716	0.816496581	0.816496581	0.816496581	0.816496581	0.816496581	0.816496581	15	148	0	P_250381	P_250381	P_250381	chr4	108163567	108163627	-	LEF1	lymphoid enhancer-binding factor 1 [Source:HGNC Symbol;Acc:HGNC:6551]	ENSG00000138795	protein_coding	exon	coding exon	1	2528	4:108163567:108163627	ENSG00000138795	LEF1	5.910449185	0.008361075	3.4442369	-1.763504675	1.994068491	-5.207741575	-1.450168409	3.757573166	9.961963646	0.006321522	0.108402476	0.074225678	0.000401778	0.177755694	0.003762042	-2.752901619	-4.76407569	-5.222456615	-0.034989643	-5.285269086	-2.288137922	10.38199042	0.002072679

ATP2B2	-0.102108473	0.019529297	-0.131299441	0.12163777	-0.029190968	-0.150828737	8	0.189249664	0.816496581	0.816496581	0.816496581	0.816496581	0.816496581	0.816496581	15	151	0	P_243218	P_243218	P_243218	chr3	10449482	10449542	-	ATP2B2	"ATPase, Ca++ transporting, plasma membrane 2 [Source:HGNC Symbol;Acc:HGNC:815]"	ENSG00000157087	protein_coding	exon	coding exon	1	2531	3:10449482:10449542	ENSG00000157087	ATP2B2	3.975169027	0.030968431	-0.710636985	0.135916641	-0.913795261	0.846553626	-0.203158276	-1.049711902	9.961963646	0.493610254	0.894594468	0.382408471	0.417116104	0.843100887	0.318648786	-6.729595822	-6.172358202	-6.609347633	-6.77123611	-6.274779408	-6.500652671	0.536386907	0.667887949

THOC2	-0.021974794	-0.001762965	0.008803801	0.020211829	0.030778595	0.010566766	8	0.1692801	0.816496581	0.816496581	0.816496581	0.816496581	0.816496581	0.816496581	15	152	0	P_271349	P_271349	P_271349	chrX	123621166	123621226	-	THOC2	THO complex 2 [Source:HGNC Symbol;Acc:HGNC:19073]	ENSG00000125676	protein_coding	exon	coding exon	1	2532	X:123621166:123621226	ENSG00000125676	THOC2	9.863675671	0.025218816	-0.16947596	-0.013596497	0.067897457	0.155879464	0.237373417	0.081493954	9.961963646	0.868814284	0.989420342	0.947210709	0.879241485	0.817177535	0.9366632	-6.982709626	-6.18198997	-7.044603586	-7.136341355	-6.266802746	-7.067751596	0.020133807	0.99586136

LDAH	0.072662857	0.135852946	0.05951597	0.063190089	-0.013146888	-0.076336977	8	0.050161851	0.816496581	0.816496581	0.816496581	0.816496581	0.816496581	0.816496581	15	153	0	P_127288	P_127288	P_127288	chr2	20686902	20686962	-	C2orf43	chromosome 2 open reading frame 43 [Source:HGNC Symbol;Acc:HGNC:26145]	ENSG00000118961	protein_coding	3'UTR;exon	multiple Ref	1	2533	2:20686902:20686962	ENSG00000118961	LDAH	7.950685372	0.004227339	1.368751377	2.559064075	1.121103249	1.190312698	-0.247648128	-1.437960826	9.961963646	0.20114778	0.02849748	0.288541284	0.261515603	0.809436204	0.18110742	-6.062274305	-3.561595219	-6.400997067	-6.425468575	-6.264168193	-6.045675866	2.204177836	0.150761357

SLC39A12	0.035955133	-0.263530897	-0.037613982	-0.29948603	-0.073569115	0.225916915	8	0.17019112	0.816496581	0.816496581	0.816496581	0.816496581	0.816496581	0.816496581	15	154	0	P_207608	P_207608	P_207608	chr10	18000764	18000824	+	SLC39A12	"solute carrier family 39 (zinc transporter), member 12 [Source:HGNC Symbol;Acc:HGNC:20860]"	ENSG00000148482	protein_coding	exon	coding exon	1	2534	10:18000764:18000824	ENSG00000148482	SLC39A12	4.039777586	0.025467172	0.275940956	-2.022491978	-0.288671948	-2.298432934	-0.564612904	1.73382003	9.961963646	0.788233028	0.070793294	0.778749906	0.044466391	0.584825288	0.113728537	-6.956968733	-4.389870888	-7.001803108	-4.835200319	-6.129871099	-5.637618937	2.142943714	0.158446087

DDX60	-0.086503556	0.356488773	0.337959706	0.442992329	0.424463262	-0.018529067	8	0.235034153	0.816496581	0.816496581	0.816496581	0.816496581	0.816496581	0.816496581	15	156	0	P_312521	P_312521	P_312521	chr4	168321435	168321495	-	DDX60	DEAD (Asp-Glu-Ala-Asp) box polypeptide 60 [Source:HGNC Symbol;Acc:HGNC:25942]	ENSG00000137628	protein_coding	promoter	promoter sense	0	2536	4:168321435:168321495	ENSG00000137628	DDX60	5.573352616	0.046568262	-0.490946962	2.023235667	1.918074798	2.514182629	2.40902176	-0.105160869	9.961963646	0.634098751	0.070705534	0.084187672	0.030773199	0.036832318	0.918335428	-6.868411454	-4.388765572	-5.339419016	-4.477494104	-3.885904436	-7.065340434	3.358180226	0.06358437

MUC20-OT1	0.097033316	0.047979645	0.185863008	-0.049053671	0.088829692	0.137883363	8	0.097701007	0.816496581	0.816496581	0.816496581	0.816496581	0.816496581	0.816496581	15	157	0	P_136925	P_136925	P_136925	chr3	195693935	195693995	+	LINC00969	long intergenic non-protein coding RNA 969 [Source:HGNC Symbol;Acc:HGNC:48729]	ENSG00000242086	lincRNA	intron;promoter	multiple non-Ref	0	2537	3:195693935:195693995	ENSG00000242086	MUC20-OT1	5.820867641	0.00987223	1.196076236	0.591418659	2.29103092	-0.604657577	1.094954684	1.699612261	9.961963646	0.259358972	0.567416969	0.045029142	0.558926147	0.299302709	0.120160349	-6.269318682	-6.000990904	-4.74877842	-6.953275998	-5.695720214	-5.687029884	1.913735909	0.191571565

ITPR1	0.183309085	0.003958989	0.104230355	-0.179350096	-0.07907873	0.100271366	8	0.046674882	0.816496581	0.816496581	0.816496581	0.816496581	0.816496581	0.816496581	16	14	0	P_243077	P_243077	P_243077	chr3	4788079	4788139	+	ITPR1	"inositol 1,4,5-trisphosphate receptor, type 1 [Source:HGNC Symbol;Acc:HGNC:6180]"	ENSG00000150995	protein_coding	exon	coding exon	1	2564	3:4788079:4788139	ENSG00000150995	ITPR1	5.494208594	0.003956174	3.569372906	0.077088961	2.029561188	-3.492283945	-1.539811718	1.952472227	9.961963646	0.005131681	0.940079078	0.069963226	0.005834032	0.154744334	0.0795325	-2.540715843	-6.17895557	-5.167166039	-2.808374687	-5.169707656	-5.310248651	5.874116558	0.014146041

HBD	-0.232424771	-0.455733299	0.348579405	-0.223308529	0.581004175	0.804312704	8	0.312366864	0.816496581	0.816496581	0.816496581	0.816496581	0.816496581	0.816496581	16	15	0	P_210682	P_210682	P_210682	chr11	5234434	5234494	-	HBD	"hemoglobin, delta [Source:HGNC Symbol;Acc:HGNC:4829]"	ENSG00000223609	protein_coding	5'UTR	5'UTR	1	2565	11:5234434:5234494	ENSG00000223609	HBD	4.219322631	0.08056317	-1.002904935	-1.966473598	1.504108207	-0.963568664	2.507013142	3.470581805	9.961963646	0.339644603	0.077707626	0.163578359	0.358068781	0.031153029	0.006049217	-6.475679415	-4.472668097	-5.934650895	-6.664288171	-3.729577522	-2.773876431	4.398949291	0.032367494

UNC13C	0.093734277	-0.145970676	0.188959898	-0.239704953	0.095225621	0.334930574	8	0.095285227	0.816496581	0.816496581	0.816496581	0.816496581	0.816496581	0.816496581	16	16	0	P_223682	P_223682	P_223682	chr15	54322035	54322095	+	UNC13C	unc-13 homolog C (C. elegans) [Source:HGNC Symbol;Acc:HGNC:23149]	ENSG00000137766	protein_coding	exon	coding exon	1	2566	15:54322035:54322095	ENSG00000137766	UNC13C	4.324722852	0.009497837	1.177963187	-1.834420539	2.374668162	-3.012383726	1.196704976	4.209088701	9.961963646	0.26618481	0.096589707	0.039055063	0.013117881	0.259124571	0.001817717	-6.289852501	-4.663917969	-4.611525547	-3.630324335	-5.586638965	-1.538927079	6.435788511	0.010659223

ABCB5	-0.130598446	0.116469497	0.005133414	0.247067942	0.135731859	-0.111336083	8	0.090972484	0.816496581	0.816496581	0.816496581	0.816496581	0.816496581	0.816496581	16	17	0	P_144379	P_144379	P_144379	chr7	20660052	20660112	+	ABCB5	"ATP-binding cassette, sub-family B (MDR/TAP), member 5 [Source:HGNC Symbol;Acc:HGNC:46]"	ENSG00000004846	protein_coding	3'UTR	3'UTR	1	2567	7:20660052:20660112	ENSG00000004846	ABCB5	4.312186165	0.008852757	-1.699982122	1.516067524	0.066820945	3.216049646	1.766803067	-1.449246578	9.961963646	0.120089081	0.160571477	0.9480463	0.00928083	0.10782494	0.178006919	-5.615242878	-5.096877845	-7.044682749	-3.280937422	-4.859510849	-6.030976323	3.45962484	0.059300763

HOXC8	0.469740974	0.224065436	0.71372255	-0.245675539	0.243981576	0.489657115	8	0.261589033	0.816496581	0.816496581	0.816496581	0.816496581	0.816496581	0.816496581	16	19	0	A_14_P113771	A_14_P113771	A_14_P113771	chr12	54011458	54011518	+	HOXC8	homeobox C8 [Source:HGNC Symbol;Acc:HGNC:5129]	ENSG00000037965	protein_coding	3'UTR	3'UTR	1	2569	12:54011458:54011518	ENSG00000037965	HOXC8	4.699365867	0.057158759	2.40637151	1.147834041	3.656231211	-1.258537469	1.249859701	2.50839717	9.961963646	0.036999285	0.2778543	0.004444441	0.236892025	0.239915347	0.031079346	-4.512796115	-5.531531066	-2.437275326	-6.346132653	-5.526804506	-4.412481364	4.98571595	0.022915136

TENM3	0.064481753	0.08026616	0.056196159	0.015784407	-0.008285594	-0.024070001	8	0.078926558	0.816496581	0.816496581	0.816496581	0.816496581	0.816496581	0.816496581	16	21	0	A_32_P105597	A_32_P105597	A_32_P105597	chr4	182518119	182518179	+	TENM3	teneurin transmembrane protein 3 [Source:HGNC Symbol;Acc:HGNC:29944]	ENSG00000218336	protein_coding	intron	intron sense	0	2571	4:182518119:182518179	ENSG00000218336	TENM3	3.62681727	0.007209233	0.930117742	1.157800091	0.810602101	0.227682348	-0.119515641	-0.347197989	9.961963646	0.374299923	0.273950415	0.436530902	0.824498101	0.907242708	0.73566568	-6.545814158	-5.520902815	-6.699707082	-7.121337861	-6.289093417	-7.005921635	0.508227586	0.685453372

MAP4K5	0.101430025	0.150424036	0.117100451	0.048994011	0.015670426	-0.033323585	8	0.060516705	0.816496581	0.816496581	0.816496581	0.816496581	0.816496581	0.816496581	16	22	0	P_220732	P_220732	P_220732	chr14	50468650	50468710	-	MAP4K5	mitogen-activated protein kinase kinase kinase kinase 5 [Source:HGNC Symbol;Acc:HGNC:6867]	ENSG00000012983	protein_coding	3'UTR;exon	multiple Ref	1	2572	14:50468650:50468710	ENSG00000012983	MAP4K5	8.589973804	0.005147688	1.73143448	2.567773814	1.998932347	0.836339333	0.267497866	-0.568841467	9.961963646	0.114166728	0.028075585	0.073627396	0.422571526	0.794542352	0.582060137	-5.569891899	-3.547792182	-5.214903831	-6.780008933	-6.258766612	-6.89743541	2.446942908	0.124296725

TYR	0.091143161	-0.113820894	0.420336418	-0.204964055	0.329193256	0.534157312	8	0.612440829	0.816496581	0.816496581	0.816496581	0.816496581	0.816496581	0.816496581	16	23	0	A_23_P312505	A_23_P312505	A_23_P312505	chr11	89191349	89191409	+	TYR	tyrosinase [Source:HGNC Symbol;Acc:HGNC:12442]	ENSG00000077498	protein_coding	exon	coding exon	1	2573	11:89191349:89191409	ENSG00000077498	TYR	3.226128172	0.303419402	0.202650669	-0.253073079	0.934589664	-0.455723748	0.731938995	1.187662743	9.961963646	0.84348705	0.805357479	0.372099986	0.658360909	0.481070138	0.262511973	-6.975996657	-6.148436787	-6.590027343	-7.037212307	-6.019378916	-6.351042775	0.522088789	0.676761871

MED16	-0.065231429	0.069295587	-0.047809883	0.134527016	0.017421546	-0.11710547	8	0.05904428	0.816496581	0.816496581	0.816496581	0.816496581	0.816496581	0.816496581	16	26	0	A_23_P56170	A_23_P56170	A_23_P56170	chr19	868424	868484	-	MED16	mediator complex subunit 16 [Source:HGNC Symbol;Acc:HGNC:17556]	ENSG00000175221	protein_coding	"3'UTR;3'UTR,exon;exon"	multiple Ref	1	2576	19:868424:868484	ENSG00000175221	MED16	8.443663731	0.005006314	-1.129128739	1.199477623	-0.82756907	2.328606363	0.30155967	-2.027046693	9.961963646	0.28529949	0.258092916	0.427294102	0.042242393	0.769188704	0.070257417	-6.34402703	-5.475714228	-6.685492996	-4.785695794	-6.248543158	-5.194562629	2.170135494	0.154978367

OPRM1	-0.04801673	0.002955347	0.029773459	0.050972077	0.077790189	0.026818112	8	0.11953361	0.816496581	0.816496581	0.816496581	0.816496581	0.816496581	0.816496581	16	27	0	P_143893	P_143893	P_143893	chr6	154246700	154246760	+	OPRM1	"opioid receptor, mu 1 [Source:HGNC Symbol;Acc:HGNC:8156]"	ENSG00000112038	protein_coding	3'UTR;exon	multiple Ref	1	2577	6:154246700:154246760	ENSG00000112038	OPRM1	4.446752122	0.013680955	-0.50278232	0.030945388	0.311757362	0.533727708	0.814539682	0.280811974	9.961963646	0.626045549	0.975924296	0.761651773	0.605233486	0.434375499	0.784600293	-6.862149982	-6.181582621	-6.994301861	-6.996028749	-5.955420173	-7.028470368	0.229689309	0.87362567

IGFBP5	1.180432976	0.142742609	1.363438541	-1.037690367	0.183005564	1.220695931	8	0.122858986	0.816496581	0.816496581	0.816496581	0.816496581	0.816496581	0.816496581	16	28	0	P_129832	P_129832	P_129832	chr2	216676620	216676680	-	IGFBP5	insulin-like growth factor binding protein 5 [Source:HGNC Symbol;Acc:HGNC:5474]	ENSG00000115461	protein_coding	3'UTR	3'UTR	1	2578	2:216676620:216676680	ENSG00000115461	IGFBP5	5.40609812	0.014328254	12.07786917	1.460503556	13.95033233	-10.61736561	1.872463162	12.48982877	9.961963646	2.85E-07	0.174959894	7.30E-08	9.45E-07	0.09075331	2.08E-07	7.454698	-5.167546113	8.775620346	6.250034796	-4.707708196	7.77123921	102.4750266	8.68E-08

SNX15	0.099851083	0.095770955	0.199342212	-0.004080128	0.099491129	0.103571257	8	0.095448267	0.816496581	0.816496581	0.816496581	0.816496581	0.816496581	0.816496581	16	29	0	A_24_P206758	A_24_P206758	A_24_P206758	chr11	65039923	65039983	+	SNX15	sorting nexin 15 [Source:HGNC Symbol;Acc:HGNC:14978]	ENSG00000110025	protein_coding	3'UTR;exon	multiple Ref	1	2579	11:65039923:65039983	ENSG00000110025	SNX15	8.55327189	0.009522809	1.253186963	1.201979072	2.501856299	-0.051207891	1.248669335	1.299877227	9.961963646	0.238752437	0.257164997	0.031429094	0.960172098	0.24033251	0.222914548	-6.203060831	-5.472964465	-4.400362684	-7.148179234	-5.528165073	-6.219220587	2.087665726	0.16577905

LY75	0.029748029	0.061963611	0.163672435	0.032215582	0.133924406	0.101708824	8	0.107414962	0.816496581	0.816496581	0.816496581	0.816496581	0.816496581	0.816496581	16	30	0	P_237123	P_237123	P_237123	chr2	159853629	159853689	-	LY75	lymphocyte antigen 75 [Source:HGNC Symbol;Acc:HGNC:6729]	ENSG00000054219	protein_coding	exon	coding exon	1	2580	2:159853629:159853689	ENSG00000054219	LY75	3.682139617	0.011472306	0.340156253	0.708527945	1.871525748	0.368371692	1.531369495	1.162997803	9.961963646	0.740802349	0.494862748	0.09089311	0.720303379	0.156794601	0.271931626	-6.935587142	-5.924162841	-5.41004232	-7.075908431	-5.18077156	-6.378832022	1.325613479	0.320393167

SF3B2	-0.109248947	0.016598884	0.045319572	0.125847831	0.154568519	0.028720689	8	0.050671653	0.816496581	0.816496581	0.816496581	0.816496581	0.816496581	0.816496581	16	31	0	P_109187	P_109187	P_109187	chr11	66068817	66068877	+	SF3B2	"splicing factor 3b, subunit 2, 145kDa [Source:HGNC Symbol;Acc:HGNC:10769]"	ENSG00000087365	protein_coding	3'UTR	3'UTR	1	2581	11:66068817:66068877	ENSG00000087365	SF3B2	11.57802848	0.00426862	-2.047949023	0.311157849	0.849547533	2.359106872	2.897496556	0.538389684	9.961963646	0.067847078	0.762094148	0.4155262	0.040104639	0.015961155	0.60212937	-5.091351376	-6.131303821	-6.666700377	-4.735466204	-3.097931702	-6.915301149	3.2105634	0.070492765

TSC22D2	0.029918425	-0.102545976	-0.072147284	-0.132464401	-0.102065709	0.030398692	8	0.045434066	0.816496581	0.816496581	0.816496581	0.816496581	0.816496581	0.816496581	16	32	0	P_246732	P_246732	P_246732	chr3	150423060	150423120	+	TSC22D2	"TSC22 domain family, member 2 [Source:HGNC Symbol;Acc:HGNC:29095]"	ENSG00000196428	protein_coding	exon	coding exon	1	2582	3:150423060:150423120	ENSG00000196428	TSC22D2	6.677582386	0.003864393	0.589445598	-2.020336097	-1.421428392	-2.609781694	-2.010873989	0.598907705	9.961963646	0.568688496	0.071048287	0.185732842	0.026126292	0.072177746	0.562605072	-6.812019513	-4.39307421	-6.043205175	-4.316662387	-4.50293647	-6.878894329	2.94382131	0.085374077

RANBP9	0.041400336	-0.102065466	0.031149845	-0.143465802	-0.010250491	0.133215311	8	0.092409037	0.816496581	0.816496581	0.816496581	0.816496581	0.816496581	0.816496581	16	33	0	P_313264	P_313264	P_313264	chr6	13631912	13631972	-	RANBP9;NOL7	"RAN binding protein 9 [Source:HGNC Symbol;Acc:HGNC:13727];nucleolar protein 7, 27kDa [Source:HGNC Symbol;Acc:HGNC:21040]"	ENSG00000010017	protein_coding	intron	multiple non-Ref	0	2583	6:13631912:13631972	ENSG00000010017	RANBP9	6.504409846	0.009064312	0.53257659	-1.312976729	0.400713615	-1.845553319	-0.131862975	1.713690344	9.961963646	0.606001218	0.218630114	0.697090195	0.094846783	0.897717866	0.117474481	-6.845761529	-5.34682802	-6.960135311	-5.549012134	-6.287445225	-5.666759345	1.421384435	0.293891715

FN3K	0.10250128	0.204141976	0.086025221	0.101640696	-0.016476059	-0.118116755	8	0.09293035	0.816496581	0.816496581	0.816496581	0.816496581	0.816496581	0.816496581	16	34	0	P_123070	P_123070	P_123070	chr17	82740792	82740852	+	FN3K	fructosamine 3 kinase [Source:HGNC Symbol;Acc:HGNC:24822]	ENSG00000167363	protein_coding	exon	coding exon	1	2584	17:82740792:82740852	ENSG00000167363	FN3K	6.507219348	0.009141903	1.31297548	2.614927433	1.101927764	1.301951954	-0.211047715	-1.512999669	9.961963646	0.21863052	0.025896965	0.296403053	0.222231375	0.837104667	0.161338183	-6.131312193	-3.472947149	-6.421688777	-6.293984019	-6.273048982	-5.946561499	2.300797153	0.139504996

CXCL8	0.025673665	-0.116079793	0.163077997	-0.141753458	0.137404333	0.27915779	8	0.134011976	0.816496581	0.816496581	0.816496581	0.816496581	0.816496581	0.816496581	16	36	0	A_14_P105298	A_14_P105298	A_14_P105298	chr4	73743427	73743487	+	CXCL8	chemokine (C-X-C motif) ligand 8 [Source:HGNC Symbol;Acc:HGNC:6025]	ENSG00000169429	protein_coding	3'UTR	3'UTR	1	2586	4:73743427:73743487	ENSG00000169429	CXCL8	4.110556428	0.016628909	0.243838131	-1.102479141	1.548849166	-1.346317272	1.305011035	2.651328307	9.961963646	0.812304325	0.296174701	0.152575608	0.208033363	0.221227231	0.024330998	-6.966012305	-5.57901464	-5.874303424	-6.239407922	-5.462760479	-4.172125688	2.369836137	0.132063466

TXNDC11	-0.236430679	-0.008477874	-0.245374749	0.227952805	-0.00894407	-0.236896875	8	0.106958321	0.816496581	0.816496581	0.816496581	0.816496581	0.816496581	0.816496581	16	37	0	P_118280	P_118280	P_118280	chr16	11700607	11700667	+	TXNDC11	thioredoxin domain containing 11 [Source:HGNC Symbol;Acc:HGNC:28030]	ENSG00000153066	protein_coding	intron	intron antisense	0	2587	16:11700607:11700667	ENSG00000153066	TXNDC11	6.386568252	0.011393694	-2.712796298	-0.097274791	-2.815420199	2.615521507	-0.102623901	-2.718145408	9.961963646	0.021898341	0.924437431	0.018367895	0.025870619	0.920297868	0.021698524	-3.999515695	-6.17710159	-3.870876505	-4.306969696	-6.291086662	-4.058988203	4.922519318	0.023757601

ROR1	-0.028332702	-0.095803871	-0.089765314	-0.06747117	-0.061432612	0.006038557	8	0.083874624	0.816496581	0.816496581	0.816496581	0.816496581	0.816496581	0.816496581	16	38	0	P_202072	P_202072	P_202072	chr1	64159132	64159192	+	ROR1	receptor tyrosine kinase-like orphan receptor 1 [Source:HGNC Symbol;Acc:HGNC:10256]	ENSG00000185483	protein_coding	exon	coding exon	1	2588	1:64159132:64159192	ENSG00000185483	ROR1	6.115719758	0.007856134	-0.391497657	-1.323805665	-1.240365654	-0.932308007	-0.848867997	0.08344001	9.961963646	0.703672837	0.21514011	0.24325898	0.373221274	0.415886702	0.935154543	-6.915363939	-5.334104506	-6.266250806	-6.694000538	-5.92708659	-7.067576369	0.84016269	0.502425422

ADGRE1	-0.059182157	0.182074794	-0.032329679	0.241256952	0.026852478	-0.214404473	8	0.155865759	0.816496581	0.816496581	0.816496581	0.816496581	0.816496581	0.816496581	16	39	0	P_232173	P_232173	P_232173	chr19	6903818	6903878	+	EMR1	"egf-like module containing, mucin-like, hormone receptor-like 1 [Source:HGNC Symbol;Acc:HGNC:3336]"	ENSG00000174837	protein_coding	exon	coding exon	1	2589	19:6903818:6903878	ENSG00000174837	ADGRE1	3.742043164	0.021716199	-0.491863408	1.513225154	-0.268692234	2.005088563	0.223171174	-1.781917389	9.961963646	0.633473356	0.161281722	0.793648872	0.072876667	0.827911892	0.105214294	-6.86793168	-5.100532121	-7.007837125	-5.304793136	-6.270262642	-5.567267458	1.641565099	0.241884699

MASTL	-0.33076576	-5.13E-06	-0.159079943	0.330760628	0.171685817	-0.15907481	8	0.061081399	0.816496581	0.816496581	0.816496581	0.816496581	0.816496581	0.816496581	16	40	0	P_314674	P_314674	P_314674	chr10	27181048	27181521	+	MASTL	microtubule associated serine/threonine kinase-like [Source:HGNC Symbol;Acc:HGNC:19042]	ENSG00000120539	protein_coding	"exon,intron;intron"	multiple non-Ref	0	2590	10:27181048:27181521	ENSG00000120539	MASTL	8.057566346	0.00520283	-5.616249019	-8.71E-05	-2.701103562	5.616161874	2.915145457	-2.701016417	9.961963646	0.000225759	0.999932189	0.022341566	0.000225786	0.01548676	0.022344903	0.683022047	-6.18208742	-4.065028349	0.567868493	-3.069213914	-4.088031446	14.36217507	0.000598365

TNRC18	-0.337950149	-0.190240889	-0.226917522	0.14770926	0.111032627	-0.036676634	8	0.192767913	0.816496581	0.816496581	0.816496581	0.816496581	0.816496581	0.816496581	16	41	0	P_260382	P_260382	P_260382	chr7	5359499	5359559	-	TNRC18	trinucleotide repeat containing 18 [Source:HGNC Symbol;Acc:HGNC:11962]	ENSG00000182095	protein_coding	exon	coding exon	1	2591	7:5359499:5359559	ENSG00000182095	TNRC18	7.805628163	0.032047763	-2.312061655	-1.301519367	-1.55243992	1.010542289	0.759621735	-0.250920553	9.961963646	0.043448086	0.222373676	0.151721371	0.336150178	0.465076661	0.806975121	-4.66786456	-5.360211217	-5.869413177	-6.618123704	-5.998617303	-7.037091646	1.851805581	0.201861519

VPS11	0.114578611	0.169058407	0.181094844	0.054479796	0.066516233	0.012036437	8	0.069246867	0.816496581	0.816496581	0.816496581	0.816496581	0.816496581	0.816496581	16	43	0	A_14_P139869	A_14_P139869	A_14_P139869	chr11	119079371	119079431	+	VPS11	vacuolar protein sorting 11 homolog (S. cerevisiae) [Source:HGNC Symbol;Acc:HGNC:14583]	ENSG00000160695	protein_coding	intron	intron sense	0	2593	11:119079371:119079431	ENSG00000160695	VPS11	7.220527606	0.006057434	1.803037865	2.660345651	2.849754048	0.857307786	1.046716183	0.189408398	9.961963646	0.101663167	0.023957899	0.017319451	0.411424441	0.319962539	0.853576189	-5.465026292	-3.400689974	-3.812390944	-6.761900158	-5.744807487	-7.051815047	3.385991601	0.062374457

MRNIP	0.069540125	-0.014432601	0.022885586	-0.083972726	-0.046654539	0.037318187	8	0.037200464	0.816496581	0.816496581	0.816496581	0.816496581	0.816496581	0.816496581	16	44	0	P_255909	P_255909	P_255909	chr5	179837998	179838058	-	C5orf45	chromosome 5 open reading frame 45 [Source:HGNC Symbol;Acc:HGNC:30817]	ENSG00000161010	protein_coding	3'UTR;exon	multiple Ref	1	2594	5:179837998:179838058	ENSG00000161010	MRNIP	8.213545981	0.003318011	1.478571077	-0.30686782	0.486596263	-1.785438896	-0.991974814	0.793464083	9.961963646	0.170163421	0.765262339	0.637071843	0.104614407	0.34469234	0.445994895	-5.920751147	-6.13268771	-6.919338814	-5.638520853	-5.798370695	-6.737938801	1.219751257	0.352854419

NAB1	0.143145587	0.042319025	0.086701764	-0.100826561	-0.056443823	0.044382739	8	0.090391113	0.816496581	0.816496581	0.816496581	0.816496581	0.816496581	0.816496581	16	46	0	P_129480	P_129480	P_129480	chr2	190683759	190683819	+	NAB1	NGFI-A binding protein 1 (EGR1 binding protein 1) [Source:HGNC Symbol;Acc:HGNC:7626]	ENSG00000138386	protein_coding	exon	coding exon	1	2596	2:190683759:190683819	ENSG00000138386	NAB1	6.591935914	0.008768084	1.872281931	0.553514419	1.134021314	-1.318767512	-0.738260617	0.580506895	9.961963646	0.090780322	0.592116277	0.283337201	0.216758025	0.477387587	0.574468654	-5.361602539	-6.023109141	-6.386901864	-6.27344886	-6.014698337	-6.89034748	1.286497822	0.331981973

STK32A	0.067854017	-0.000633497	0.023589205	-0.068487514	-0.044264813	0.024222701	8	0.141553311	0.816496581	0.816496581	0.816496581	0.816496581	0.816496581	0.816496581	16	47	0	P_255274	P_255274	P_255274	chr5	147373234	147373294	+	STK32A	serine/threonine kinase 32A [Source:HGNC Symbol;Acc:HGNC:28317]	ENSG00000169302	protein_coding	exon	coding exon	1	2597	5:147373234:147373294	ENSG00000169302	STK32A	3.985330506	0.01829776	0.614359004	-0.005735761	0.213579693	-0.620094765	-0.400779311	0.219315454	9.961963646	0.552749668	0.995536786	0.835182583	0.549116237	0.697043364	0.83083266	-6.796232938	-6.182070081	-7.022266366	-6.943313955	-6.211955572	-7.045165407	0.169387835	0.914606696

REPS1	-0.185074953	0.041995132	-0.177450662	0.227070085	0.007624291	-0.219445794	8	0.055887149	0.816496581	0.816496581	0.816496581	0.816496581	0.816496581	0.816496581	16	48	0	P_143719	P_143719	P_143719	chr6	138917294	138917354	-	REPS1	RALBP1 associated Eps domain containing 1 [Source:HGNC Symbol;Acc:HGNC:15578]	ENSG00000135597	protein_coding	3'ext;intron;promoter	multiple non-Ref	0	2598	6:138917294:138917354	ENSG00000135597	REPS1	7.407101333	0.004714923	-3.301079403	0.749043897	-3.165089152	4.0501233	0.135990252	-3.914133048	9.961963646	0.008039341	0.47114728	0.010117624	0.002341257	0.894537768	0.002914876	-2.996634201	-5.894673484	-3.273385292	-1.869340952	-6.286858314	-2.025987782	8.869735157	0.003652134

CSNK1G3	-0.045340618	0.186299939	-0.083250395	0.231640557	-0.037909778	-0.269550335	8	0.065172314	0.816496581	0.816496581	0.816496581	0.816496581	0.816496581	0.816496581	16	49	0	P_254541	P_254541	P_254541	chr5	123545746	123545806	+	CSNK1G3	"casein kinase 1, gamma 3 [Source:HGNC Symbol;Acc:HGNC:2456]"	ENSG00000151292	protein_coding	exon	coding exon	1	2599	5:123545746:123545806	ENSG00000151292	CSNK1G3	8.292962488	0.005617602	-0.740896543	3.044267765	-1.360368106	3.785164308	-0.619471563	-4.404635871	9.961963646	0.475857355	0.012424131	0.203698043	0.003595865	0.549510356	0.001337654	-6.706841493	-2.786679456	-6.120764858	-2.312017874	-6.09653694	-1.221596017	7.629854384	0.006126302

SELL	-0.319683489	0.213921618	-0.101568467	0.533605106	0.218115022	-0.315490084	8	0.135409778	0.816496581	0.816496581	0.816496581	0.816496581	0.816496581	0.816496581	16	50	0	P_204784	P_204784	P_204784	chr1	169703254	169703314	-	SELL	selectin L [Source:HGNC Symbol;Acc:HGNC:10720]	ENSG00000188404	protein_coding	exon	coding exon	1	2600	1:169703254:169703314	ENSG00000188404	SELL	5.141423837	0.016931338	-3.008986243	2.013514076	-0.956002202	5.022500319	2.052984042	-2.969516278	9.961963646	0.013194094	0.071860913	0.361694793	0.000525908	0.067278299	0.014113189	-3.495461259	-4.403202036	-6.569753015	-0.316298526	-4.439461894	-3.630511374	8.713409535	0.003887163

ALKBH8	-0.368934492	-0.020710908	-0.378677442	0.348223584	-0.00974295	-0.357966533	8	0.087539007	0.816496581	0.816496581	0.816496581	0.816496581	0.816496581	0.816496581	16	51	0	P_213140	P_213140	P_213140	chr11	107549752	107549812	-	ALKBH8	"alkB, alkylation repair homolog 8 (E. coli) [Source:HGNC Symbol;Acc:HGNC:25189]"	ENSG00000137760	protein_coding	3'UTR;exon	multiple Ref	1	2601	11:107549752:107549812	ENSG00000137760	ALKBH8	6.001854056	0.008360553	-4.941711427	-0.277413294	-5.072213854	4.664298133	-0.130502427	-4.79480056	9.961963646	0.000592343	0.787134413	0.000489013	0.000897586	0.898766599	0.000737167	-0.317442736	-6.1416797	-0.156956995	-0.873945347	-6.287634749	-0.603240864	15.83127395	0.000404945

EBNA1BP2	-0.302525444	-0.002686424	-0.220149078	0.29983902	0.082376366	-0.217462654	8	0.128246002	0.816496581	0.816496581	0.816496581	0.816496581	0.816496581	0.816496581	16	52	0	A_23_P103631	A_23_P103631	A_23_P103631	chr1	43164343	43164403	-	EBNA1BP2	EBNA1 binding protein 2 [Source:HGNC Symbol;Acc:HGNC:15531]	ENSG00000117395	protein_coding	3'UTR;exon	multiple Ref	1	2602	1:43164343:43164403	ENSG00000117395	EBNA1BP2	6.687537332	0.015414552	-2.984297316	-0.02650054	-2.171686107	2.957796777	0.812611209	-2.145185567	9.961963646	0.013761611	0.97938145	0.055113753	0.014398419	0.435430242	0.05763182	-3.537616094	-6.181717216	-4.941968856	-3.723890886	-5.956981751	-5.007760456	4.605612194	0.028584616

HMGCL	0.006332109	0.151761341	0.054680032	0.145429232	0.048347923	-0.097081309	8	0.093796417	0.816496581	0.816496581	0.816496581	0.816496581	0.816496581	0.816496581	16	53	0	A_23_P145	A_23_P145	A_23_P145	chr1	23802333	23802393	-	HMGCL	3-hydroxymethyl-3-methylglutaryl-CoA lyase [Source:HGNC Symbol;Acc:HGNC:5005]	ENSG00000117305	protein_coding	3'UTR	3'UTR	1	2603	1:23802333:23802393	ENSG00000117305	HMGCL	9.275718171	0.009271771	0.080540192	1.930302751	0.69549343	1.849762559	0.614953238	-1.234809321	9.961963646	0.937402688	0.082504354	0.502646992	0.094195402	0.552372606	0.245233315	-6.994821725	-4.525625143	-6.789220004	-5.542690007	-6.099390656	-6.296716719	1.590134936	0.253020634

ZC3H18	-0.128322337	-0.070496897	-0.134214069	0.057825441	-0.005891732	-0.063717173	8	0.085009215	0.816496581	0.816496581	0.816496581	0.816496581	0.816496581	0.816496581	16	54	0	P_119781	P_119781	P_119781	chr16	88631309	88631369	-	ZC3H18	zinc finger CCCH-type containing 18 [Source:HGNC Symbol;Acc:HGNC:25091]	ENSG00000158545	protein_coding	"3'UTR;3'UTR,intron;exon;intron"	multiple non-Ref	0	2604	16:88631309:88631369	ENSG00000158545	ZC3H18	7.904387619	0.008010011	-1.756027578	-0.964715086	-1.836653011	0.791312492	-0.080625433	-0.871937925	9.961963646	0.10972216	0.357521704	0.096237842	0.447192542	0.937336595	0.403767353	-5.534123417	-5.713946055	-5.462409974	-6.817572463	-6.293228914	-6.671229906	1.463425666	0.283047894

PCNX3	-0.003390874	0.004464036	-0.05087297	0.007854911	-0.047482096	-0.055337007	8	0.115465053	0.816496581	0.816496581	0.816496581	0.816496581	0.816496581	0.816496581	16	55	0	A_24_P333123	A_24_P333123	A_24_P333123	chr11	65626904	65626964	+	PCNXL3	pecanex-like 3 (Drosophila) [Source:HGNC Symbol;Acc:HGNC:18760]	ENSG00000197136	protein_coding	exon	coding exon	1	2605	11:65626904:65626964	ENSG00000197136	PCNX3	5.977086202	0.01291315	-0.036546113	0.048112426	-0.548297948	0.084658539	-0.511751835	-0.596410374	9.961963646	0.971568868	0.962577546	0.595559772	0.934210031	0.619976472	0.564207135	-6.99763153	-6.180867272	-6.885389669	-7.145695051	-6.159248833	-6.880468254	0.154840568	0.924172446

NBR1	0.229441167	0.056040695	0.199201094	-0.173400472	-0.030240074	0.143160399	8	0.047345756	0.816496581	0.816496581	0.816496581	0.816496581	0.816496581	0.816496581	16	57	0	P_121649	P_121649	P_121649	chr17	43209949	43210009	+	NBR1	neighbor of BRCA1 gene 1 [Source:HGNC Symbol;Acc:HGNC:6746]	ENSG00000188554	protein_coding	exon	coding exon	1	2607	17:43209949:43210009	ENSG00000188554	NBR1	8.440673316	0.004006828	4.439321999	1.084298397	3.854224626	-3.355023601	-0.585097373	2.769926228	9.961963646	0.001267534	0.303776155	0.003212676	0.007341553	0.571496233	0.019856316	-1.104656836	-5.597634219	-2.104612187	-3.042830582	-6.117760207	-3.971042357	9.168223984	0.003248946

HSPA4L	0.266914019	0.099427402	0.456732264	-0.167486617	0.189818246	0.357304863	8	0.073616334	0.816496581	0.816496581	0.816496581	0.816496581	0.816496581	0.816496581	16	58	0	P_250846	P_250846	P_250846	chr4	127811571	127811631	+	HSPA4L	heat shock 70kDa protein 4-like [Source:HGNC Symbol;Acc:HGNC:17041]	ENSG00000164070	protein_coding	exon	coding exon	1	2608	4:127811571:127811631	ENSG00000164070	HSPA4L	8.138954312	0.006558729	4.036520479	1.50363306	6.907127428	-2.532887419	2.870606948	5.403494367	9.961963646	0.002392888	0.16369883	4.24E-05	0.029803679	0.016712364	0.000303909	-1.759555337	-5.112833331	2.386010589	-4.44612141	-3.141674945	0.319051928	18.35274559	0.00022084

ENPP2	0.086469194	-0.023317639	0.184750616	-0.109786833	0.098281421	0.208068254	8	0.191307251	0.816496581	0.816496581	0.816496581	0.816496581	0.816496581	0.816496581	16	59	0	P_147857	P_147857	P_147857	chr8	119595852	119595912	-	ENPP2	ectonucleotide pyrophosphatase/phosphodiesterase 2 [Source:HGNC Symbol;Acc:HGNC:3357]	ENSG00000136960	protein_coding	exon	coding exon	1	2609	8:119595852:119595912	ENSG00000136960	ENPP2	3.964755513	0.031597247	0.595775203	-0.160659192	1.272936982	-0.756434394	0.67716178	1.433596174	9.961963646	0.564615008	0.875573022	0.231943985	0.466900566	0.513720477	0.182318869	-6.808066286	-6.168498732	-6.227708436	-6.845408734	-6.058417415	-6.051340648	0.847844515	0.498807572

TENM2	0.226715311	-0.13142901	0.006837085	-0.358144321	-0.219878226	0.138266095	8	0.11512997	0.816496581	0.816496581	0.816496581	0.816496581	0.816496581	0.816496581	16	60	0	P_255642	P_255642	P_255642	chr5	167993059	167993119	+	TENM2	teneurin transmembrane protein 2 [Source:HGNC Symbol;Acc:HGNC:29943]	ENSG00000145934	protein_coding	exon	coding exon	1	2610	5:167993059:167993119	ENSG00000145934	TENM2	4.581916053	0.012851099	2.449380965	-1.419929305	0.073866325	-3.86931027	-2.37551464	1.49379563	9.961963646	0.034379289	0.186157204	0.942578902	0.003134805	0.038998747	0.166210449	-4.441543304	-5.218098006	-7.044141719	-2.170692348	-3.9390458	-5.972231681	5.144529955	0.020950633

ABHD16A	0.001969826	0.030596998	0.064552301	0.028627172	0.062582475	0.033955303	8	0.11815887	0.816496581	0.816496581	0.816496581	0.816496581	0.816496581	0.816496581	16	62	0	P_256683	P_256683	P_256683	chr6	31687675	31687735	-	ABHD16A	abhydrolase domain containing 16A [Source:HGNC Symbol;Acc:HGNC:13921]	ENSG00000204427	protein_coding	3'UTR;exon	multiple Ref	1	2612	6:31687675:31687735	ENSG00000204427	ABHD16A	9.245380519	0.013418545	0.020826712	0.323498105	0.682503133	0.302671393	0.661676421	0.359005028	9.961963646	0.983795132	0.753006727	0.510478859	0.768365806	0.523188092	0.72708363	-6.998123628	-6.127218168	-6.798547711	-7.099744967	-6.068954177	-7.001423185	0.204867595	0.890690026

MOCS1	-0.056497395	-0.114252911	-0.136773894	-0.057755516	-0.080276499	-0.022520983	8	0.144102804	0.816496581	0.816496581	0.816496581	0.816496581	0.816496581	0.816496581	16	63	0	P_142576	P_142576	P_142576	chr6	39927503	39927563	-	MOCS1	molybdenum cofactor synthesis 1 [Source:HGNC Symbol;Acc:HGNC:7190]	ENSG00000124615	protein_coding	exon	coding exon	1	2613	6:39927503:39927563	ENSG00000124615	MOCS1	6.917354703	0.018882607	-0.503550544	-1.01831448	-1.219039726	-0.514763936	-0.715489181	-0.200725246	9.961963646	0.625524585	0.332621586	0.250907512	0.617945049	0.490736149	0.844952211	-6.861738655	-5.663159114	-6.291090785	-7.006608509	-6.031389436	-7.049412322	0.598963781	0.630175919

CHIA	0.011684638	-0.11043347	0.122085052	-0.122118109	0.110400414	0.232518523	8	0.343391073	0.816496581	0.816496581	0.816496581	0.816496581	0.816496581	0.816496581	16	64	0	P_102714	P_102714	P_102714	chr1	111319179	111319239	+	CHIA	"chitinase, acidic [Source:HGNC Symbol;Acc:HGNC:17432]"	ENSG00000134216	protein_coding	3'UTR;exon	multiple Ref	1	2614	1:111319179:111319239	ENSG00000134216	CHIA	3.805909629	0.096900809	0.045972402	-0.434492864	0.480335209	-0.480465266	0.434362807	0.914828073	9.961963646	0.964240764	0.673191052	0.641362271	0.641273007	0.673282362	0.381891732	-6.997207141	-6.083536008	-6.922568611	-7.024819822	-6.197303312	-6.632492964	0.279674625	0.838890046

SCMH1	-0.094552772	-0.057572715	-0.155134935	0.036980058	-0.060582163	-0.09756222	8	0.0449315	0.816496581	0.816496581	0.816496581	0.816496581	0.816496581	0.816496581	16	67	0	P_101484	P_101484	P_101484	chr1	41027210	41027270	-	SCMH1	sex comb on midleg homolog 1 (Drosophila) [Source:HGNC Symbol;Acc:HGNC:19003]	ENSG00000010803	protein_coding	3'UTR	3'UTR	1	2617	1:41027210:41027270	ENSG00000010803	SCMH1	8.630816847	0.003827922	-1.871709033	-1.139674358	-3.070956595	0.732034675	-1.199247562	-1.931282237	9.961963646	0.090865761	0.281083083	0.011872218	0.481014268	0.258178393	0.082370886	-5.362465931	-5.540181217	-3.434454029	-6.864217271	-5.583820279	-5.342775237	3.322807887	0.065163711

SHISA3	-0.114803042	0.074458321	0.175686739	0.189261364	0.290489782	0.101228418	8	0.164892707	0.816496581	0.816496581	0.816496581	0.816496581	0.816496581	0.816496581	16	68	0	A_23_P41476	A_23_P41476	A_23_P41476	chr4	42402163	42402223	+	SHISA3	shisa family member 3 [Source:HGNC Symbol;Acc:HGNC:25159]	ENSG00000178343	protein_coding	3'UTR	3'UTR	1	2618	4:42402163:42402223	ENSG00000178343	SHISA3	5.336340566	0.024041419	-0.906815585	0.588137431	1.387728672	1.494953016	2.294544257	0.799591241	9.961963646	0.385913541	0.569532404	0.195474045	0.165913226	0.044761177	0.442595904	-6.567333553	-6.00295955	-6.086294205	-6.047668437	-4.066697223	-6.732927922	1.872195837	0.198404387

IRAG2	0.102595323	-0.214518578	0.042250638	-0.317113901	-0.060344685	0.256769217	8	0.179413149	0.816496581	0.816496581	0.816496581	0.816496581	0.816496581	0.816496581	16	69	0	P_214721	P_214721	P_214721	chr12	25096955	25097015	+	LRMP	lymphoid-restricted membrane protein [Source:HGNC Symbol;Acc:HGNC:6690]	ENSG00000118308	protein_coding	exon	coding exon	1	2619	12:25096955:25097015	ENSG00000118308	IRAG2	3.777814177	0.028056269	0.750167483	-1.568539942	0.308932746	-2.318707425	-0.441234737	1.877472688	9.961963646	0.470500062	0.147942722	0.763736817	0.0429598	0.668465388	0.090009598	-6.699699989	-5.028682707	-6.995250071	-4.801957547	-6.194165352	-5.424643453	2.035750692	0.173028923

CCDC169	0.037564464	0.304002633	0.1826673	0.266438169	0.145102836	-0.121335333	8	0.179913158	0.816496581	0.816496581	0.816496581	0.816496581	0.816496581	0.816496581	16	70	0	P_113602	P_113602	P_113602	chr13	36283642	36283702	-	CCDC169	coiled-coil domain containing 169 [Source:HGNC Symbol;Acc:HGNC:34361]	ENSG00000242715	protein_coding	5'UTR;exon	multiple Ref	1	2620	13:36283642:36283702	ENSG00000242715	CCDC169	4.15028148	0.028200551	0.273964335	2.217145426	1.3322252	1.943181092	1.058260865	-0.884920226	9.961963646	0.78970869	0.051038704	0.212458772	0.080765572	0.314922152	0.397055782	-6.957557371	-4.095729695	-6.155729717	-5.400643685	-5.733218303	-6.659671906	2.074094484	0.167639474

ROS1	0.095407832	0.052461132	0.080848736	-0.0429467	-0.014559096	0.028387604	8	0.084071946	0.816496581	0.816496581	0.816496581	0.816496581	0.816496581	0.816496581	16	71	0	P_258911	P_258911	P_258911	chr6	117425533	117425593	-	ROS1	"ROS proto-oncogene 1 , receptor tyrosine kinase [Source:HGNC Symbol;Acc:HGNC:10261]"	ENSG00000047936	protein_coding	exon	coding exon	1	2621	6:117425533:117425593	ENSG00000047936	ROS1	3.68377235	0.007882747	1.316105953	0.723676519	1.115270104	-0.592429434	-0.200835849	0.391593585	9.961963646	0.217616814	0.485910183	0.290915467	0.566766192	0.84486803	0.703604186	-6.12749031	-5.913308767	-6.407320793	-6.96100152	-6.275276345	-6.988248058	0.674053866	0.587406218

FAM117B	0.491144689	0.011408006	0.313194118	-0.479736683	-0.177950571	0.301786112	8	0.096106543	0.816496581	0.816496581	0.816496581	0.816496581	0.816496581	0.816496581	16	72	0	P_238762	P_238762	P_238762	chr2	202765494	202765554	+	FAM117B	"family with sequence similarity 117, member B [Source:HGNC Symbol;Acc:HGNC:14440]"	ENSG00000138439	protein_coding	exon	coding exon	1	2622	2:202765494:202765554	ENSG00000138439	FAM117B	7.487459783	0.009624071	6.13162617	0.142421633	3.910027518	-5.989204537	-2.221598651	3.767605886	9.961963646	0.000112771	0.889586266	0.002934331	0.000136123	0.050655511	0.003700708	1.402891439	-6.171405642	-2.01149659	1.097602783	-4.180650362	-2.2712718	17.98439404	0.00024022

LAMB1	0.555258927	0.057900753	0.470087991	-0.497358173	-0.085170935	0.412187238	8	0.074057534	0.816496581	0.816496581	0.816496581	0.816496581	0.816496581	0.816496581	16	74	0	P_262420	P_262420	P_262420	chr7	107975815	107975875	-	LAMB1	"laminin, beta 1 [Source:HGNC Symbol;Acc:HGNC:6486]"	ENSG00000091136	protein_coding	exon	coding exon	1	2624	7:107975815:107975875	ENSG00000091136	LAMB1	6.249159986	0.006611051	8.363843843	0.872156821	7.080917322	-7.491687023	-1.282926521	6.208760502	9.961963646	8.16E-06	0.403653541	3.44E-05	2.13E-05	0.228561366	0.000101958	4.108142563	-5.796270777	2.602742284	3.038041209	-5.488640498	1.457224863	36.19565223	1.14E-05

FCHSD2	0.10700032	0.068743272	0.318906417	-0.038257048	0.211906097	0.250163145	8	0.049247436	0.816496581	0.816496581	0.816496581	0.816496581	0.816496581	0.816496581	16	75	0	A_24_P302038	A_24_P302038	A_24_P302038	chr11	72843473	72843533	-	FCHSD2	FCH and double SH3 domains 2 [Source:HGNC Symbol;Acc:HGNC:29114]	ENSG00000137478	protein_coding	3'UTR;exon	multiple Ref	1	2625	11:72843473:72843533	ENSG00000137478	FCHSD2	6.344968252	0.00415434	2.033198048	1.306245493	6.059794052	-0.726952555	4.026596004	4.753548559	9.961963646	0.069539782	0.220823085	0.000123958	0.483987488	0.002431312	0.000784298	-5.114439926	-5.35470054	1.2710087	-6.86806528	-1.290817368	-0.667644958	13.64990581	0.00073148

CPLANE1	-0.01696136	-0.062865254	0.118618741	-0.045903894	0.1355801	0.181483995	8	0.12334783	0.816496581	0.816496581	0.816496581	0.816496581	0.816496581	0.816496581	16	77	0	P_252470	P_252470	P_252470	chr5	37175908	37175968	-	C5orf42	chromosome 5 open reading frame 42 [Source:HGNC Symbol;Acc:HGNC:25801]	ENSG00000197603	protein_coding	exon	coding exon	1	2627	5:37175908:37175968	ENSG00000197603	CPLANE1	4.006952097	0.014424907	-0.172961636	-0.641061638	1.209601794	-0.468100002	1.382563429	1.850663432	9.961963646	0.866145266	0.535951124	0.254353822	0.649786528	0.197004754	0.09405653	-6.982060201	-5.969972141	-6.301982436	-7.031091644	-5.369484432	-5.465025613	1.243261106	0.3453403

LCP2	0.149912615	0.048833645	0.06252002	-0.10107897	-0.087392595	0.013686375	8	0.128262668	0.816496581	0.816496581	0.816496581	0.816496581	0.816496581	0.816496581	16	78	0	P_141119	P_141119	P_141119	chr5	170248242	170248302	-	LCP2	lymphocyte cytosolic protein 2 (SH2 domain containing leukocyte protein of 76kDa) [Source:HGNC Symbol;Acc:HGNC:6529]	ENSG00000043462	protein_coding	3'UTR;exon	multiple Ref	1	2628	5:170248242:170248302	ENSG00000043462	LCP2	3.857741513	0.015417985	1.478665727	0.481671519	0.616667318	-0.996994208	-0.861998408	0.1349958	9.961963646	0.170138597	0.640445385	0.551285781	0.342367452	0.408958678	0.895303824	-5.920626119	-6.061240807	-6.843340951	-6.631623065	-5.915982893	-7.061431923	0.759000156	0.542313506

ZNF451	0.0642021	0.077292053	0.102201695	0.013089953	0.037999595	0.024909642	8	0.05675672	0.816496581	0.816496581	0.816496581	0.816496581	0.816496581	0.816496581	16	79	0	P_257566	P_257566	P_257566	chr6	57152291	57152351	+	ZNF451	zinc finger protein 451 [Source:HGNC Symbol;Acc:HGNC:21091]	ENSG00000112200	protein_coding	exon	coding exon	1	2629	6:57152291:57152351	ENSG00000112200	ZNF451	6.993237143	0.004793584	1.13570281	1.367257484	1.807896505	0.231554674	0.672193694	0.44063902	9.961963646	0.28266524	0.201600256	0.100861979	0.82157081	0.516746656	0.668882344	-6.336836313	-5.282336797	-5.505229673	-7.120370511	-6.061822049	-6.966334404	1.187888058	0.36332955

VWA8	-0.059271341	-0.020198439	-0.040958476	0.039072901	0.018312865	-0.020760037	8	0.045779366	0.816496581	0.816496581	0.816496581	0.816496581	0.816496581	0.816496581	16	80	0	P_218913	P_218913	P_218913	chr13	41670945	41671005	-	VWA8	von Willebrand factor A domain containing 8 [Source:HGNC Symbol;Acc:HGNC:29071]	ENSG00000102763	protein_coding	exon	coding exon	1	2630	13:41670945:41671005	ENSG00000102763	VWA8	7.28919926	0.003889686	-1.163946769	-0.396648831	-0.804326088	0.767297938	0.35962068	-0.407677257	9.961963646	0.271564322	0.699990321	0.439981049	0.460702915	0.726637209	0.692133704	-6.305580055	-6.09982258	-6.704899547	-6.836857814	-6.228355334	-6.981336851	0.507219456	0.686088874

KANSL1L	-0.093398613	-0.092872885	-0.086336006	0.000525728	0.007062607	0.006536879	8	0.066765811	0.816496581	0.816496581	0.816496581	0.816496581	0.816496581	0.816496581	16	81	0	P_311249	P_311249	P_311249	chr2	210060196	210060256	+	KANSL1L;AC007038.7	KAT8 regulatory NSL complex subunit 1-like [Source:HGNC Symbol;Acc:HGNC:26310];	ENSG00000144445	protein_coding;antisense	intron	multiple non-Ref	0	2631	2:210060196:210060256	ENSG00000144445	KANSL1L	5.431969068	0.005786439	-1.50376638	-1.49530188	-1.390054723	0.0084645	0.113711657	0.105247157	9.961963646	0.16366502	0.165823726	0.194788019	0.993413496	0.911725388	0.918268691	-5.887291002	-5.123478674	-6.083342303	-7.149573882	-6.289812389	-7.065330527	1.075595354	0.403068495

ZC4H2	0.102612427	-0.015657459	-0.101642034	-0.118269887	-0.204254461	-0.085984574	8	0.082991072	0.816496581	0.816496581	0.816496581	0.816496581	0.816496581	0.816496581	16	82	0	P_151062	P_151062	P_151062	chrX	64917464	64917524	-	ZC4H2	"zinc finger, C4H2 domain containing [Source:HGNC Symbol;Acc:HGNC:24931]"	ENSG00000126970	protein_coding	3'UTR;exon	multiple Ref	1	2632	X:64917464:64917524	ENSG00000126970	ZC4H2	6.704897332	0.007737737	1.428692025	-0.218001738	-1.415181051	-1.646693763	-2.843873076	-1.197179313	9.961963646	0.183688335	0.831828441	0.187506807	0.13075894	0.017494654	0.258947844	-5.985919676	-6.157095782	-6.051245003	-5.839106284	-3.185148183	-6.340203705	2.720645967	0.100748216

PIKFYVE	0.10280871	0.054282597	0.057928655	-0.048526112	-0.044880054	0.003646058	8	0.047608438	0.816496581	0.816496581	0.816496581	0.816496581	0.816496581	0.816496581	16	84	0	P_238997	P_238997	P_238997	chr2	208317881	208317941	+	PIKFYVE	"phosphoinositide kinase, FYVE finger containing [Source:HGNC Symbol;Acc:HGNC:23785]"	ENSG00000115020	protein_coding	3'UTR;exon	multiple Ref	1	2634	2:208317881:208317941	ENSG00000115020	PIKFYVE	6.693379483	0.004026858	1.9842317	1.047666591	1.118036349	-0.936565109	-0.866195351	0.070369758	9.961963646	0.075449693	0.319545301	0.289787674	0.371131132	0.406761067	0.945291957	-5.190588084	-5.634413236	-6.404325088	-6.690002419	-5.912402968	-7.06867396	1.319531082	0.322165044

RPF1	-0.007726358	-0.044679125	-0.060323449	-0.036952767	-0.05259709	-0.015644324	8	0.073575828	0.816496581	0.816496581	0.816496581	0.816496581	0.816496581	0.816496581	16	86	0	P_102256	P_102256	P_102256	chr1	84499406	84499466	+	RPF1;GNG5	"ribosome production factor 1 homolog (S. cerevisiae) [Source:HGNC Symbol;Acc:HGNC:30350];guanine nucleotide binding protein (G protein), gamma 5 [Source:HGNC Symbol;Acc:HGNC:4408]"	ENSG00000117133	protein_coding	3'ext;intron	multiple non-Ref	0	2636	1:84499406:84499466	ENSG00000117133	RPF1	10.82276837	0.006553941	-0.116887805	-0.675925785	-0.912600108	-0.55903798	-0.795712303	-0.236674323	9.961963646	0.909271891	0.514472353	0.38300705	0.588481621	0.44474572	0.817705018	-6.990909806	-5.946819188	-6.610446931	-6.981346366	-5.970527301	-7.040863797	0.384178942	0.766786269

PPT1	-0.170519555	0.040062938	-0.099599729	0.210582493	0.070919826	-0.139662667	8	0.045091502	0.816496581	0.816496581	0.816496581	0.816496581	0.816496581	0.816496581	16	88	0	P_201295	P_201295	P_201295	chr1	40078559	40078619	-	PPT1	palmitoyl-protein thioesterase 1 [Source:HGNC Symbol;Acc:HGNC:9325]	ENSG00000131238	protein_coding	3'UTR;exon	multiple Ref	1	2638	1:40078559:40078619	ENSG00000131238	PPT1	9.336420928	0.003839489	-3.370412712	0.791865981	-1.96864338	4.162278693	1.401769332	-2.760509361	9.961963646	0.007154163	0.44688425	0.077428371	0.001957686	0.191363965	0.020179249	-2.878481851	-5.861927275	-5.261809675	-1.684165002	-5.345827146	-3.987051785	7.128703909	0.007672986

CCR3	0.171912722	0.114003531	0.168647041	-0.057909191	-0.003265681	0.05464351	8	0.330242596	0.816496581	0.816496581	0.816496581	0.816496581	0.816496581	0.816496581	16	89	0	A_24_P367481	A_24_P367481	A_24_P367481	chr3	46242481	46242538	+	CCR3	chemokine (C-C motif) receptor 3 [Source:HGNC Symbol;Acc:HGNC:1604]	ENSG00000183625	protein_coding	5'UTR;exon	multiple Ref	1	2639	3:46242481:46242538	ENSG00000183625	CCR3	4.143754785	0.089787949	0.702659012	0.465966726	0.689311191	-0.236692286	-0.013347821	0.223344465	9.961963646	0.498358445	0.651260707	0.50636509	0.817691464	0.989613828	0.827780685	-6.73545253	-6.068906232	-6.793679093	-7.119062173	-6.296590495	-7.044195886	0.215348124	0.883509543

SEL1L2	0.176453983	0.291208166	0.220279836	0.114754183	0.043825852	-0.070928331	8	0.17341033	0.816496581	0.816496581	0.816496581	0.816496581	0.816496581	0.816496581	16	90	0	P_240242	P_240242	P_240242	chr20	13877520	13877580	-	SEL1L2	sel-1 suppressor of lin-12-like 2 (C. elegans) [Source:HGNC Symbol;Acc:HGNC:15897]	ENSG00000101251	protein_coding	3'UTR;exon	multiple Ref	1	2640	20:13877520:13877580	ENSG00000101251	SEL1L2	3.733775939	0.026355451	1.331195588	2.196918533	1.66182446	0.865722945	0.330628871	-0.535094073	9.961963646	0.212785166	0.05281469	0.127645924	0.407008022	0.747773484	0.60432285	-6.108978738	-4.12670678	-5.717257699	-6.754523962	-6.238869061	-6.917179148	1.750885101	0.220033645

NMUR2	-0.010700721	-0.012650489	-0.051162963	-0.001949768	-0.040462241	-0.038512473	8	0.131810001	0.816496581	0.816496581	0.816496581	0.816496581	0.816496581	0.816496581	16	91	0	P_255444	P_255444	P_255444	chr5	152398059	152398119	-	NMUR2	neuromedin U receptor 2 [Source:HGNC Symbol;Acc:HGNC:16454]	ENSG00000132911	protein_coding	exon	coding exon	1	2641	5:152398059:152398119	ENSG00000132911	NMUR2	3.92762458	0.016158854	-0.103098749	-0.121884274	-0.492942241	-0.018785524	-0.389843491	-0.371057967	9.961963646	0.919930512	0.905414278	0.632737534	0.985383124	0.704857095	0.718363518	-6.992563081	-6.174261958	-6.916024536	-7.14942008	-6.216480921	-6.996679955	0.093082007	0.962149709

TRAPPC6B	0.049248195	-0.064160416	0.10725003	-0.113408611	0.058001835	0.171410446	8	0.076140309	0.816496581	0.816496581	0.816496581	0.816496581	0.816496581	0.816496581	16	92	0	P_220547	P_220547	P_220547	chr14	39159482	39159542	-	TRAPPC6B	trafficking protein particle complex 6B [Source:HGNC Symbol;Acc:HGNC:23066]	ENSG00000182400	protein_coding	exon	coding exon	1	2642	14:39159482:39159542	ENSG00000182400	TRAPPC6B	6.850113595	0.00686227	0.728118498	-0.948590811	1.5856567	-1.676709309	0.857538203	2.534247511	9.961963646	0.483304353	0.365272214	0.144016744	0.12464859	0.411303078	0.029734373	-6.716554186	-5.728784148	-5.823852568	-5.796465377	-5.919771053	-4.369204746	2.318904095	0.137506723

FAM3D	-0.315860355	0.083877741	-0.112375987	0.399738096	0.203484368	-0.196253728	8	0.184970437	0.816496581	0.816496581	0.816496581	0.816496581	0.816496581	0.816496581	16	93	0	P_135234	P_135234	P_135234	chr3	58657696	58657756	-	FAM3D	"family with sequence similarity 3, member D [Source:HGNC Symbol;Acc:HGNC:18665]"	ENSG00000198643	protein_coding	5'UTR	5'UTR	1	2643	3:58657696:58657756	ENSG00000198643	FAM3D	5.314712479	0.029682442	-2.245385613	0.596269428	-0.798857534	2.841655041	1.446528079	-1.395126962	9.961963646	0.048655303	0.564297629	0.443002017	0.017561196	0.178749565	0.193299131	-4.776401295	-5.99806194	-6.709395092	-3.922611496	-5.289869959	-6.100776122	3.024882205	0.080488839

NEDD4	0.149292851	0.101147538	0.223658799	-0.048145313	0.074365948	0.122511261	8	0.069500999	0.816496581	0.816496581	0.816496581	0.816496581	0.816496581	0.816496581	16	94	0	P_116606	P_116606	P_116606	chr15	55915283	55915343	-	NEDD4	"neural precursor cell expressed, developmentally down-regulated 4, E3 ubiquitin protein ligase [Source:HGNC Symbol;Acc:HGNC:7727]"	ENSG00000069869	protein_coding	exon	coding exon	1	2644	15:55915283:55915343	ENSG00000069869	NEDD4	6.525254563	0.00608575	2.343838051	1.587975894	3.511353692	-0.755862157	1.167515641	1.923377798	9.961963646	0.041161269	0.143491955	0.005651495	0.467228505	0.270186477	0.08345375	-4.615807912	-5.003078024	-2.682633487	-6.845856146	-5.618679056	-5.354867736	4.329775253	0.033765164

TBCD	-0.011288274	-0.016188214	-0.104468401	-0.00489994	-0.093180127	-0.088280187	8	0.050491831	0.816496581	0.816496581	0.816496581	0.816496581	0.816496581	0.816496581	16	95	0	P_123073	P_123073	P_123073	chr17	82770670	82770730	-	TBCD	tubulin folding cofactor D [Source:HGNC Symbol;Acc:HGNC:11581]	ENSG00000141556	protein_coding	intron	intron antisense	0	2645	17:82770670:82770730	ENSG00000141556	TBCD	8.746728315	0.004254011	-0.211969739	-0.303980169	-1.961693978	-0.092010431	-1.74972424	-1.657713809	9.961963646	0.836404607	0.767397437	0.078326114	0.928513721	0.110846047	0.128485011	-6.973897305	-6.133608609	-5.272527985	-7.144985313	-4.883640335	-5.74681129	1.633932381	0.243501297

A2M	0.169285213	0.139085492	0.307306784	-0.03019972	0.138021571	0.168221291	8	0.080757068	0.816496581	0.816496581	0.816496581	0.816496581	0.816496581	0.816496581	16	96	0	P_214312	P_214312	P_214312	chr12	9067857	9067917	-	A2M	alpha-2-macroglobulin [Source:HGNC Symbol;Acc:HGNC:7]	ENSG00000175899	protein_coding	exon	non-coding exon	1	2646	12:9067857:9067917	ENSG00000175899	A2M	3.939666654	0.007443968	2.403047785	1.97435487	4.36230001	-0.428692915	1.959252226	2.387945141	9.961963646	0.03720973	0.076697794	0.00142884	0.67726847	0.07864386	0.038180852	-4.51828928	-4.46107555	-1.269265111	-7.050032547	-4.580030153	-4.612772695	6.40451768	0.010824096

DEFA5	-0.377201394	0.061920372	-0.11564858	0.439121766	0.261552814	-0.177568952	8	0.234339866	0.816496581	0.816496581	0.816496581	0.816496581	0.816496581	0.816496581	16	97	0	P_146479	P_146479	P_146479	chr8	7056525	7056585	-	DEFA5	"defensin, alpha 5, Paneth cell-specific [Source:HGNC Symbol;Acc:HGNC:2764]"	ENSG00000164816	protein_coding	exon	coding exon	1	2647	8:7056525:7056585	ENSG00000164816	DEFA5	4.843955494	0.046306562	-2.14682997	0.352417866	-0.658210288	2.499247836	1.488619682	-1.010628154	9.961963646	0.05747241	0.731866774	0.525321376	0.031569655	0.167545324	0.336111043	-4.934921375	-6.117035279	-6.815560746	-4.502507484	-5.236226786	-6.540377845	2.441652605	0.124812132

ANXA7	-0.061321336	0.039441107	-0.038195162	0.100762444	0.023126174	-0.077636269	8	0.0627706	0.816496581	0.816496581	0.816496581	0.816496581	0.816496581	0.816496581	16	99	0	P_106283	P_106283	P_106283	chr10	73396043	73396103	-	ANXA7	annexin A7 [Source:HGNC Symbol;Acc:HGNC:545]	ENSG00000138279	protein_coding	exon	coding exon	1	2649	10:73396043:73396103	ENSG00000138279	ANXA7	9.637892307	0.005370838	-1.024793168	0.659133988	-0.63831194	1.683927156	0.386481228	-1.297445929	9.961963646	0.329701437	0.524752368	0.537667147	0.123218077	0.707266771	0.223717343	-6.453744074	-5.958110154	-6.829075022	-5.786147349	-6.217847882	-6.222167413	1.093407521	0.396459557

SAV1	0.116583953	0.119871022	0.17154812	0.003287069	0.054964167	0.051677098	8	0.054055126	0.816496581	0.816496581	0.816496581	0.816496581	0.816496581	0.816496581	16	100	0	A_24_P287473	A_24_P287473	A_24_P287473	chr14	50633700	50633760	-	SAV1	salvador family WW domain containing protein 1 [Source:HGNC Symbol;Acc:HGNC:17795]	ENSG00000151748	protein_coding	3'UTR	3'UTR	1	2650	14:50633700:50633760	ENSG00000151748	SAV1	9.572322481	0.004553174	2.116056506	2.175718441	3.113683377	0.059661934	0.997626871	0.937964936	9.961963646	0.060526811	0.054739989	0.011040194	0.953604778	0.342075239	0.370445686	-4.983903108	-4.159080233	-3.36134391	-7.147666816	-5.792947863	-6.610944965	3.464177864	0.059116682

KIAA1109	0.137352211	-0.161057715	-0.087614381	-0.298409926	-0.224966592	0.073443334	8	0.048135438	0.816496581	0.816496581	0.816496581	0.816496581	0.816496581	0.816496581	16	101	0	P_250729	P_250729	P_250729	chr4	122249619	122249679	+	KIAA1109	KIAA1109 [Source:HGNC Symbol;Acc:HGNC:26953]	ENSG00000138688	protein_coding	exon	coding exon	1	2651	4:122249619:122249679	ENSG00000138688	KIAA1109	6.604219773	0.004067378	2.637691706	-3.092928737	-1.682533723	-5.730620443	-4.320225429	1.410395014	9.961963646	0.024906267	0.011436647	0.123493085	0.000192922	0.001525997	0.188875584	-4.126373487	-2.708824906	-5.687785718	0.732518019	-0.842636238	-6.081262438	12.14135299	0.001151879

ESPL1	-0.509972468	-0.133089154	-0.397297142	0.376883314	0.112675325	-0.264207989	8	0.118210586	0.816496581	0.816496581	0.816496581	0.816496581	0.816496581	0.816496581	16	103	0	P_215550	P_215550	P_215550	chr12	53270414	53270474	+	ESPL1	extra spindle pole bodies homolog 1 (S. cerevisiae) [Source:HGNC Symbol;Acc:HGNC:16856]	ENSG00000135476	protein_coding	3'UTR;exon	multiple Ref	1	2653	12:53270414:53270474	ENSG00000135476	ESPL1	5.921386853	0.013428361	-5.389901771	-1.406619985	-4.199035647	3.983281786	1.190866124	-2.792415662	9.961963646	0.000309803	0.189961214	0.001846862	0.002606749	0.261307899	0.019105944	0.35468461	-5.234480279	-1.534472949	-1.980352481	-5.593095103	-3.932782693	12.29063373	0.001099196

ZNF624	0.238616033	0.024986127	0.211208988	-0.213629906	-0.027407045	0.186222861	8	0.158271222	0.816496581	0.816496581	0.816496581	0.816496581	0.816496581	0.816496581	16	106	0	P_120690	P_120690	P_120690	chr17	16622289	16622349	-	ZNF624	zinc finger protein 624 [Source:HGNC Symbol;Acc:HGNC:29254]	ENSG00000197566	protein_coding	exon	coding exon	1	2656	17:16622289:16622349	ENSG00000197566	ZNF624	6.700891492	0.022323023	1.955999537	0.204817973	1.731336649	-1.751181564	-0.224662888	1.526518676	9.961963646	0.079069014	0.841838577	0.114184731	0.110585277	0.82678263	0.157983394	-5.234132556	-6.160020737	-5.617543901	-5.688851914	-6.269909158	-5.92836809	2.052130122	0.170702342

SLC43A2	0.181872844	-0.020000373	0.327030769	-0.201873217	0.145157925	0.347031142	8	0.247330943	0.816496581	0.816496581	0.816496581	0.816496581	0.816496581	0.816496581	16	107	0	A_14_P139378	A_14_P139378	A_14_P139378	chr17	1585689	1585747	+	SLC43A2	"solute carrier family 43 (amino acid system L transporter), member 2 [Source:HGNC Symbol;Acc:HGNC:23087]"	ENSG00000167703	protein_coding	exon;intron;promoter	multiple non-Ref	0	2657	17:1585689:1585747	ENSG00000167703	SLC43A2	8.181233562	0.051331614	0.983152819	-0.108116325	1.767835241	-1.091269144	0.784682422	1.875951566	9.961963646	0.348806689	0.916049997	0.107644789	0.3008441	0.45089634	0.090234823	-6.495142238	-6.175929004	-5.564313616	-6.534662157	-5.979233875	-5.426942076	1.571551528	0.257188022

ARMC10	0.116672126	-0.014271366	0.0839652	-0.130943492	-0.032706926	0.098236566	8	0.1187605	0.816496581	0.816496581	0.816496581	0.816496581	0.816496581	0.816496581	16	108	0	P_145559	P_145559	P_145559	chr7	103097288	103097348	+	ARMC10	armadillo repeat containing 10 [Source:HGNC Symbol;Acc:HGNC:21706]	ENSG00000170632	protein_coding	3'UTR;exon	multiple Ref	1	2658	7:103097288:103097348	ENSG00000170632	ARMC10	7.636878841	0.01353301	1.22833135	-0.150249827	0.883990814	-1.378581177	-0.344340536	1.034240641	9.961963646	0.247551514	0.8835661	0.39753367	0.198191797	0.737748449	0.325477631	-6.232176646	-6.170200546	-6.636397208	-6.198927526	-6.234003992	-6.516522969	0.900253808	0.474830882

CEP120	-0.224322396	0.184259109	-0.208342981	0.408581505	0.015979415	-0.39260209	8	0.041114649	0.816496581	0.816496581	0.816496581	0.816496581	0.816496581	0.816496581	16	109	0	P_254534	P_254534	P_254534	chr5	123389925	123389985	-	CEP120	centrosomal protein 120kDa [Source:HGNC Symbol;Acc:HGNC:26690]	ENSG00000168944	protein_coding	3'UTR;exon	multiple Ref	1	2659	5:123389925:123389985	ENSG00000168944	CEP120	6.94786663	0.003564179	-4.601914227	3.78002655	-4.27410078	8.381940777	0.327813448	-8.054127329	9.961963646	0.000987018	0.003626218	0.001640572	8.00E-06	0.749838125	1.14E-05	-0.846108457	-1.625267513	-1.412131872	4.054911715	-6.239844104	3.734241816	31.49458544	2.14E-05

ALPK1	0.237086103	0.190080487	0.316162732	-0.047005615	0.079076629	0.126082245	8	0.095841752	0.816496581	0.816496581	0.816496581	0.816496581	0.816496581	0.816496581	16	111	0	P_138440	P_138440	P_138440	chr4	112441045	112441105	+	ALPK1	alpha-kinase 1 [Source:HGNC Symbol;Acc:HGNC:20917]	ENSG00000073331	protein_coding	3'UTR;exon	multiple Ref	1	2661	4:112441045:112441105	ENSG00000073331	ALPK1	5.731646001	0.009583255	2.966164322	2.378081016	3.955485391	-0.588083306	0.989321069	1.577404376	9.961963646	0.014194178	0.038828493	0.00272629	0.569567335	0.3459262	0.145897886	-3.568568039	-3.8464918	-1.935871488	-6.96371192	-5.800908167	-5.859003404	5.65201125	0.015892263

NRG3	-0.172731248	0.027128001	0.138585693	0.199859248	0.31131694	0.111457692	8	0.156316017	0.816496581	0.816496581	0.816496581	0.816496581	0.816496581	0.816496581	16	112	0	A_23_P61761	A_23_P61761	A_23_P61761	chr10	82985690	82985749	+	NRG3	neuregulin 3 [Source:HGNC Symbol;Acc:HGNC:7999]	ENSG00000185737	protein_coding	3'UTR	3'UTR	1	2662	10:82985690:82985749	ENSG00000185737	NRG3	3.774082224	0.021829078	-1.431854343	0.224877351	1.148805027	1.656731694	2.58065937	0.923927676	9.961963646	0.182804265	0.826620311	0.277472031	0.128686216	0.027462766	0.377360411	-5.981831207	-6.155498703	-6.370618813	-5.824894431	-3.611324653	-6.624072221	2.279789119	0.141866261

ELMO3	-0.048600477	0.05926315	-0.073941908	0.107863627	-0.025341431	-0.133205058	8	0.100398543	0.816496581	0.816496581	0.816496581	0.816496581	0.816496581	0.816496581	16	114	0	P_119245	P_119245	P_119245	chr16	67203517	67203577	+	ELMO3	engulfment and cell motility 3 [Source:HGNC Symbol;Acc:HGNC:17289]	ENSG00000102890	protein_coding	exon	coding exon	1	2664	16:67203517:67203577	ENSG00000102890	ELMO3	8.905891124	0.010301367	-0.586460456	0.71512659	-0.892254713	1.301587045	-0.305794257	-1.607381303	9.961963646	0.570615242	0.490950563	0.393298575	0.222351407	0.766055879	0.139166903	-6.813870261	-5.919460203	-6.628973517	-6.294427584	-6.247188168	-5.817505058	1.003795666	0.43094435

MCTP1	0.942353284	-0.188463851	0.764249586	-1.130817136	-0.178103699	0.952713437	8	0.078910423	0.816496581	0.816496581	0.816496581	0.816496581	0.816496581	0.816496581	16	116	0	P_253954	P_253954	P_253954	chr5	94923961	94924021	-	MCTP1	"multiple C2 domains, transmembrane 1 [Source:HGNC Symbol;Acc:HGNC:26183]"	ENSG00000175471	protein_coding	exon	coding exon	1	2666	5:94923961:94924021	ENSG00000175471	MCTP1	5.956984719	0.007207188	13.5949137	-2.718884561	11.02549048	-16.31379826	-2.569423223	13.74437504	9.961963646	9.33E-08	0.021671057	6.67E-07	1.63E-08	0.02799639	8.41E-08	8.523110693	-3.307358903	6.621188475	10.23798859	-3.629403191	8.648837942	129.2375411	2.83E-08

LOXL3	-0.208659634	0.046673474	-0.235642755	0.255333108	-0.026983121	-0.282316229	8	0.094193104	0.816496581	0.816496581	0.816496581	0.816496581	0.816496581	0.816496581	16	118	0	P_128215	P_128215	P_128215	chr2	74532842	74532902	-	LOXL3	lysyl oxidase-like 3 [Source:HGNC Symbol;Acc:HGNC:13869]	ENSG00000115318	protein_coding	3'UTR;exon	multiple Ref	1	2668	2:74532842:74532902	ENSG00000115318	LOXL3	6.529389562	0.009331657	-2.645481179	0.591747404	-2.987585388	3.237228583	-0.34210421	-3.579332793	9.961963646	0.024576022	0.567205264	0.013684619	0.008954263	0.739380041	0.005047561	-4.113242324	-6.000793105	-3.57705791	-3.244619767	-6.234810639	-2.589133087	6.613784923	0.009775988

MYOC	-0.474434666	0.174239746	-0.011217551	0.648674412	0.463217115	-0.185457297	8	0.474260103	0.816496581	0.816496581	0.816496581	0.816496581	0.816496581	0.816496581	16	119	0	A_23_P23783	A_23_P23783	A_23_P23783	chr1	171635451	171635511	-	MYOC	"myocilin, trabecular meshwork inducible glucocorticoid response [Source:HGNC Symbol;Acc:HGNC:7610]"	ENSG00000034971	protein_coding	3'UTR	3'UTR	1	2669	1:171635451:171635511	ENSG00000034971	MYOC	3.197276282	0.182831832	-1.358927033	0.499076309	-0.032130521	1.858003342	1.326796512	-0.53120683	9.961963646	0.204139158	0.628561778	0.975002598	0.092932107	0.214184415	0.606915429	-6.074577969	-6.052463165	-7.046556844	-5.530292482	-5.436930126	-6.919380297	1.265256386	0.338471219

PIK3R3	0.460298221	-0.101339483	0.414588023	-0.561637704	-0.045710198	0.515927506	8	0.130708756	0.816496581	0.816496581	0.816496581	0.816496581	0.816496581	0.816496581	16	120	0	P_101704	P_101704	P_101704	chr1	46043255	46043315	-	PIK3R3	"phosphoinositide-3-kinase, regulatory subunit 3 (gamma) [Source:HGNC Symbol;Acc:HGNC:8981]"	ENSG00000117461	protein_coding	3'UTR;exon	multiple Ref	1	2670	1:46043255:46043315	ENSG00000117461	PIK3R3	11.17921321	0.015926693	4.467063454	-0.983470891	4.023458965	-5.450534344	-0.443604488	5.006929856	9.961963646	0.001214255	0.348657725	0.002443592	0.000284421	0.666807918	0.000538065	-1.060296128	-5.69642843	-1.823187282	0.326280614	-6.193072272	-0.275818114	15.34742485	0.000458981

HMHB1	0.44593363	-0.296149057	0.16684743	-0.742082687	-0.2790862	0.462996487	8	0.246995232	0.816496581	0.816496581	0.816496581	0.816496581	0.816496581	0.816496581	16	122	0	A_23_P30204	A_23_P30204	A_23_P30204	chr5	143820643	143820703	+	HMHB1	histocompatibility (minor) HB-1 [Source:HGNC Symbol;Acc:HGNC:29677]	ENSG00000158497	protein_coding	3'UTR	3'UTR	1	2672	5:143820643:143820703	ENSG00000158497	HMHB1	6.89615855	0.051198346	2.41372556	-1.602979679	0.903102792	-4.016705238	-1.510622768	2.50608247	9.961963646	0.036537797	0.140137651	0.387787258	0.00247025	0.161934402	0.031202674	-4.50063511	-4.983188345	-6.619139276	-1.924783155	-5.207804354	-4.416351817	5.651260174	0.015898592

CAPZB	-0.271694638	0.12876323	-0.121360304	0.400457868	0.150334334	-0.250123534	8	0.108458209	0.816496581	0.816496581	0.816496581	0.816496581	0.816496581	0.816496581	16	123	0	P_100637	P_100637	P_100637	chr1	19339069	19339129	-	CAPZB	"capping protein (actin filament) muscle Z-line, beta [Source:HGNC Symbol;Acc:HGNC:1491]"	ENSG00000077549	protein_coding	3'UTR	3'UTR	1	2673	1:19339069:19339129	ENSG00000077549	CAPZB	7.744185982	0.011653161	-3.082512279	1.460883587	-1.376893668	4.543395866	1.705618612	-2.837777255	9.961963646	0.01164107	0.174857817	0.198696636	0.001079536	0.119007713	0.017678146	-3.369863944	-5.167068359	-6.100000518	-1.066121053	-4.945394588	-3.855511055	7.522743261	0.006422902

EPB41	0.152260023	-0.096968096	0.112374134	-0.249228118	-0.039885889	0.209342229	8	0.087262294	0.816496581	0.816496581	0.816496581	0.816496581	0.816496581	0.816496581	16	124	0	P_200854	P_200854	P_200854	chr1	29015707	29015767	+	EPB41	erythrocyte membrane protein band 4.1 [Source:HGNC Symbol;Acc:HGNC:3377]	ENSG00000159023	protein_coding	exon	coding exon	1	2674	1:29015707:29015767	ENSG00000159023	EPB41	7.287913218	0.00832171	2.04420873	-1.301871784	1.508709778	-3.346080514	-0.535498952	2.810581562	9.961963646	0.068272549	0.222257741	0.162415648	0.007452769	0.604053149	0.018520709	-5.09721213	-5.359800777	-5.928494864	-3.0581351	-6.14638751	-3.901853261	4.588707886	0.028873482

PROSER2	-0.261328856	0.189282912	-0.099844005	0.450611769	0.161484852	-0.289126917	8	0.071791507	0.816496581	0.816496581	0.816496581	0.816496581	0.816496581	0.816496581	16	126	0	A_16_P38941797	A_16_P38941797	A_16_P38941797	chr10	11871313	11871373	+	PROSER2	proline and serine rich 2 [Source:HGNC Symbol;Acc:HGNC:23728]	ENSG00000148426	protein_coding	3'UTR	3'UTR	1	2676	10:11871313:11871373	ENSG00000148426	PROSER2	7.916385206	0.006345644	-4.017863353	2.910175659	-1.535075666	6.928039012	2.482787688	-4.445251325	9.961963646	0.002465657	0.015618884	0.155891613	4.13E-05	0.032471062	0.001255942	-1.790348401	-3.001399603	-5.892997437	2.344967333	-3.768341569	-1.156286522	16.8151707	0.000316821

LCP1	-0.917330679	0.068216599	-1.082291461	0.985547278	-0.164960783	-1.150508061	8	0.053599312	0.816496581	0.816496581	0.816496581	0.816496581	0.816496581	0.816496581	16	128	0	P_219052	P_219052	P_219052	chr13	46142292	46142352	-	LCP1	lymphocyte cytosolic protein 1 (L-plastin) [Source:HGNC Symbol;Acc:HGNC:6528]	ENSG00000136167	protein_coding	exon	coding exon	1	2678	13:46142292:46142352	ENSG00000136167	LCP1	6.756110115	0.004513768	-16.72252747	1.243558058	-19.72968866	17.96608553	-3.007161197	-20.97324672	9.961963646	1.28E-08	0.242130471	2.59E-09	6.42E-09	0.013235219	1.43E-09	10.33861606	-5.426647863	11.78689922	11.10095599	-2.919446994	12.32347815	240.3583837	1.37E-09

C1orf229	0.216914714	-0.210921256	0.038390771	-0.42783597	-0.178523943	0.249312026	8	0.159344705	0.816496581	0.816496581	0.816496581	0.816496581	0.816496581	0.816496581	16	129	0	P_105284	P_105284	P_105284	chr1	247111265	247111325	-	C1orf229	chromosome 1 open reading frame 229 [Source:HGNC Symbol;Acc:HGNC:33759]	ENSG00000221953	protein_coding	3'UTR;exon	multiple Ref	1	2679	1:247111265:247111325	ENSG00000221953	C1orf229	8.046436133	0.022596829	1.767302515	-1.718471092	0.312787014	-3.485773607	-1.454515501	2.031258106	9.961963646	0.107737735	0.116574834	0.760892211	0.005897739	0.176575095	0.069765349	-5.517636822	-4.826656884	-6.993954107	-2.819473702	-5.279765315	-5.187975442	4.094429877	0.039090732

LSG1	-0.105497563	-0.032651112	0.005854105	0.072846451	0.111351668	0.038505217	8	0.03859249	0.816496581	0.816496581	0.816496581	0.816496581	0.816496581	0.816496581	16	131	0	P_247786	P_247786	P_247786	chr3	194644623	194644683	-	LSG1	large 60S subunit nuclear export GTPase 1 [Source:HGNC Symbol;Acc:HGNC:25652]	ENSG00000041802	protein_coding	exon	coding exon	1	2681	3:194644623:194644683	ENSG00000041802	LSG1	9.606932074	0.003402738	-2.21500052	-0.68553461	0.122911319	1.52946591	2.337911839	0.808445928	9.961963646	0.051224268	0.508644596	0.904621645	0.157260177	0.041578655	0.43771419	-4.825528983	-5.940242065	-7.038876706	-6.001307922	-3.998468499	-6.725626656	2.308329126	0.138669662

ETF1	-0.055120323	-0.008463316	0.145264314	0.046657007	0.200384636	0.153727629	8	0.118723473	0.816496581	0.816496581	0.816496581	0.816496581	0.816496581	0.816496581	16	132	0	P_255044	P_255044	P_255044	chr5	138510568	138510628	-	ETF1	eukaryotic translation termination factor 1 [Source:HGNC Symbol;Acc:HGNC:3477]	ENSG00000120705	protein_coding	exon	coding exon	1	2682	5:138510568:138510628	ENSG00000120705	ETF1	9.383568269	0.013525949	-0.580461627	-0.089125569	1.529750828	0.491336058	2.110212455	1.618876397	9.961963646	0.574498008	0.930748451	0.157190415	0.633833189	0.061124139	0.136659884	-6.817562938	-6.177901645	-5.900197091	-7.019177162	-4.352413042	-5.801470544	1.66669753	0.236648145

ECT2	-0.283526087	-0.073877456	-0.206884274	0.209648631	0.076641813	-0.133006818	8	0.091439272	0.816496581	0.816496581	0.816496581	0.816496581	0.816496581	0.816496581	16	133	0	P_247265	P_247265	P_247265	chr3	172769062	172769122	+	ECT2	epithelial cell transforming 2 [Source:HGNC Symbol;Acc:HGNC:3155]	ENSG00000114346	protein_coding	exon	coding exon	1	2683	3:172769062:172769122	ENSG00000114346	ECT2	8.735360503	0.008921136	-3.676449306	-0.957960248	-2.68264396	2.718489058	0.993805347	-1.724683712	9.961963646	0.004298668	0.360753911	0.023059647	0.02168575	0.343843127	0.115415071	-2.35998543	-5.720187159	-4.096279105	-4.132498792	-5.796617144	-5.650867589	5.497151948	0.017263374

UBR3	-0.035160389	-0.042243359	-0.004966013	-0.007082969	0.030194376	0.037277345	8	0.054933502	0.816496581	0.816496581	0.816496581	0.816496581	0.816496581	0.816496581	16	134	0	P_237481	P_237481	P_237481	chr2	170055489	170055549	+	UBR3	ubiquitin protein ligase E3 component n-recognin 3 (putative) [Source:HGNC Symbol;Acc:HGNC:30467]	ENSG00000144357	protein_coding	3'UTR;exon	multiple Ref	1	2684	2:170055489:170055549	ENSG00000144357	UBR3	7.249933375	0.004630053	-0.632858053	-0.760345665	-0.08938415	-0.127487612	0.543473903	0.670961515	9.961963646	0.541080214	0.464663043	0.930548117	0.901091193	0.598753596	0.517498856	-6.784119538	-5.88618726	-7.042759202	-7.140732078	-6.141944998	-6.830871798	0.291405117	0.830720345

SCN7A	0.247205283	0.055704586	0.157387416	-0.191500697	-0.089817867	0.10168283	8	0.088124238	0.816496581	0.816496581	0.816496581	0.816496581	0.816496581	0.816496581	16	135	0	P_237374	P_237374	P_237374	chr2	166441398	166441458	-	SCN7A	"sodium channel, voltage-gated, type VII, alpha subunit [Source:HGNC Symbol;Acc:HGNC:10594]"	ENSG00000136546	protein_coding	exon	coding exon	1	2685	2:166441398:166441458	ENSG00000136546	SCN7A	3.648227531	0.00844311	3.294975212	0.742481015	2.097801585	-2.552494197	-1.197173627	1.35532057	9.961963646	0.008122491	0.474939022	0.062411396	0.028819878	0.258949962	0.205246622	-3.007044646	-5.899549555	-5.059761984	-4.413183916	-5.586119755	-6.150976346	4.265709393	0.035124251

NPM3	0.162470866	0.242863033	0.089992901	0.080392167	-0.072477965	-0.152870132	8	0.252572567	0.816496581	0.816496581	0.816496581	0.816496581	0.816496581	0.816496581	16	136	0	P_106920	P_106920	P_106920	chr10	101781743	101781803	-	NPM3	nucleophosmin/nucleoplasmin 3 [Source:HGNC Symbol;Acc:HGNC:7931]	ENSG00000107833	protein_coding	exon	coding exon	1	2686	10:101781743:101781803	ENSG00000107833	NPM3	12.14324493	0.053435862	0.860804868	1.286739503	0.476801345	0.425934635	-0.384003522	-0.809938157	9.961963646	0.409585131	0.227280974	0.643790019	0.679211421	0.709044675	0.436895041	-6.608459311	-5.377354669	-6.924373949	-7.051298333	-6.218847871	-6.724389278	0.601483655	0.628696387

PAK4	0.008647091	-0.107174519	0.004664619	-0.115821609	-0.003982471	0.111839138	8	0.033920203	0.816496581	0.816496581	0.816496581	0.816496581	0.816496581	0.816496581	16	137	0	P_310869	P_310869	P_310869	chr19	39125796	39125856	-	PAK4	p21 protein (Cdc42/Rac)-activated kinase 4 [Source:HGNC Symbol;Acc:HGNC:16059]	ENSG00000130669	protein_coding	"5'UTR;5'UTR,promoter;exon;exon,promoter"	multiple non-Ref	0	2687	19:39125796:39125856	ENSG00000130669	PAK4	8.177772632	0.003130663	0.189276855	-2.345951562	0.10210422	-2.535228418	-0.087172635	2.448055783	9.961963646	0.853676552	0.041013405	0.920699931	0.029684489	0.932261632	0.034457221	-6.978845852	-3.896606968	-7.041430254	-4.442191511	-6.292645006	-4.513114999	2.996283174	0.082173654

NR1H3	-0.182149611	0.047331281	-0.251118802	0.229480893	-0.068969191	-0.298450084	8	0.075138561	0.816496581	0.816496581	0.816496581	0.816496581	0.816496581	0.816496581	16	139	0	P_108451	P_108451	P_108451	chr11	47267962	47268022	+	NR1H3	"nuclear receptor subfamily 1, group H, member 3 [Source:HGNC Symbol;Acc:HGNC:7966]"	ENSG00000025434	protein_coding	exon	coding exon	1	2689	11:47267962:47268022	ENSG00000025434	NR1H3	9.894728588	0.006740572	-2.717224387	0.706066354	-3.746075151	3.42329074	-1.028850764	-4.452141505	9.961963646	0.021732798	0.496327092	0.003833641	0.006547056	0.327882348	0.001242612	-3.992020016	-5.925907092	-2.285904637	-2.926110969	-5.762542186	-1.145228187	9.085655745	0.00335489

ATG2B	0.077365562	-0.027351003	-0.000890725	-0.104716565	-0.078256287	0.026460278	8	0.063853153	0.816496581	0.816496581	0.816496581	0.816496581	0.816496581	0.816496581	16	140	0	P_222125	P_222125	P_222125	chr14	96290814	96290874	-	ATG2B	autophagy related 2B [Source:HGNC Symbol;Acc:HGNC:20187]	ENSG00000066739	protein_coding	exon	coding exon	1	2690	14:96290814:96290874	ENSG00000066739	ATG2B	6.807063827	0.005480918	1.279872356	-0.452472546	-0.014735427	-1.732344903	-1.294607784	0.437737119	9.961963646	0.229591225	0.660622086	0.988534189	0.113999316	0.224657496	0.670915137	-6.17133235	-6.075298648	-7.047002025	-5.716308664	-5.474990235	-6.967700273	1.118610625	0.387309369

UBA6	0.010995537	0.048140421	0.002186938	0.037144884	-0.008808599	-0.045953483	8	0.083930147	0.816496581	0.816496581	0.816496581	0.816496581	0.816496581	0.816496581	16	141	0	P_249183	P_249183	P_249183	chr4	67624993	67625053	-	UBA6	ubiquitin-like modifier activating enzyme 6 [Source:HGNC Symbol;Acc:HGNC:25581]	ENSG00000033178	protein_coding	exon	coding exon	1	2691	4:67624993:67625053	ENSG00000033178	UBA6	7.833563122	0.007863617	0.151862628	0.664881652	0.030204446	0.513019024	-0.121658182	-0.634677206	9.961963646	0.88232675	0.521219968	0.976500558	0.619121444	0.905588781	0.539940389	-6.985790087	-5.954274494	-7.046622404	-7.007563774	-6.288818992	-6.855682485	0.191141094	0.900024557

SLC27A1	0.164390209	0.098070883	0.04894453	-0.066319326	-0.115445679	-0.049126353	8	0.095967877	0.816496581	0.816496581	0.816496581	0.816496581	0.816496581	0.816496581	16	142	0	A_24_P382489	A_24_P382489	A_24_P382489	chr19	17506107	17506167	+	SLC27A1	"solute carrier family 27 (fatty acid transporter), member 1 [Source:HGNC Symbol;Acc:HGNC:10995]"	ENSG00000130304	protein_coding	3'UTR;exon	multiple Ref	1	2692	19:17506107:17506167	ENSG00000130304	SLC27A1	7.814391518	0.009602682	2.054590589	1.225714809	0.611721166	-0.82887578	-1.442869423	-0.613993643	9.961963646	0.06709777	0.248492879	0.554425228	0.426588223	0.179753246	0.552981574	-5.080933991	-5.446664273	-6.846537044	-6.786360745	-5.294486544	-6.869256671	1.540636249	0.264295989

RNF17	0.04527311	0.184673246	0.076681182	0.139400135	0.031408071	-0.107992064	8	0.090083829	0.816496581	0.816496581	0.816496581	0.816496581	0.816496581	0.816496581	16	144	0	P_218327	P_218327	P_218327	chr13	24788099	24788159	+	RNF17	ring finger protein 17 [Source:HGNC Symbol;Acc:HGNC:10060]	ENSG00000132972	protein_coding	exon	coding exon	1	2694	13:24788099:24788159	ENSG00000132972	RNF17	3.521533063	0.008723549	0.593662154	2.421603378	1.005513321	1.827941224	0.411851167	-1.416090058	9.961963646	0.565973078	0.03604971	0.338448134	0.09761766	0.689170128	0.187247795	-6.809390387	-3.778360047	-6.521423608	-5.575388478	-6.207250637	-6.073947597	2.123992711	0.160916543

GLS	0.012490479	0.101005511	0.2220902	0.088515033	0.209599721	0.121084688	8	0.075294389	0.816496581	0.816496581	0.816496581	0.816496581	0.816496581	0.816496581	16	145	0	P_238340	P_238340	P_238340	chr2	190930505	190930565	+	GLS	glutaminase [Source:HGNC Symbol;Acc:HGNC:4331]	ENSG00000115419	protein_coding	exon	coding exon	1	2695	2:190930505:190930565	ENSG00000115419	GLS	9.147231105	0.006759397	0.18606759	1.504654292	3.308423154	1.318586702	3.122355563	1.803768861	9.961963646	0.856125974	0.163439998	0.007940465	0.216816276	0.010878725	0.101542253	-6.979500847	-5.11152594	-3.028335189	-6.27367065	-2.732036668	-5.534978915	4.664271119	0.027608628

CEBPZ	-0.234879578	-0.024979129	-0.105532747	0.209900449	0.129346831	-0.080553618	8	0.105342353	0.816496581	0.816496581	0.816496581	0.816496581	0.816496581	0.816496581	16	146	0	P_234474	P_234474	P_234474	chr2	37210998	37211058	-	CEBPZ	"CCAAT/enhancer binding protein (C/EBP), zeta [Source:HGNC Symbol;Acc:HGNC:24218]"	ENSG00000115816	protein_coding	exon	coding exon	1	2696	2:37210998:37211058	ENSG00000115816	CEBPZ	9.483961307	0.011118189	-2.728185256	-0.290138852	-1.225789348	2.438046404	1.502395908	-0.935650495	9.961963646	0.021328405	0.777659642	0.248466022	0.035051499	0.164012865	0.371579478	-3.973458796	-6.13790407	-6.283263214	-4.604653082	-5.218461459	-6.613120786	3.017740075	0.080905732

RND3	-0.600763651	0.072549949	-0.517200724	0.6733136	0.083562926	-0.589750674	8	0.27424541	0.816496581	0.816496581	0.816496581	0.816496581	0.816496581	0.816496581	16	147	0	A_23_P142849	NM_005168	A_23_P142849	chr2	150468592	150468652	-	RND3	Rho family GTPase 3 [Source:HGNC Symbol;Acc:HGNC:671]	ENSG00000115963	protein_coding	3'UTR	3'UTR	1	2697	2:150468592:150468652	ENSG00000115963	RND3	3.316832041	0.062604853	-2.940663145	0.355122953	-2.531633042	3.295786098	0.409030103	-2.886755994	9.961963646	0.014825984	0.729901053	0.02986774	0.008111395	0.69117255	0.016257028	-3.612081477	-6.116039337	-4.350560847	-3.144256844	-6.208461193	-3.771947003	5.757608283	0.015031704

KLF17	-0.00094185	0.009162545	-0.082928459	0.010104396	-0.081986608	-0.092091004	8	0.20635969	0.816496581	0.816496581	0.816496581	0.816496581	0.816496581	0.816496581	16	148	0	A_16_P15102696	A_16_P15102696	A_16_P15102696	chr1	44115807	44115867	+	KLF17	Kruppel-like factor 17 [Source:HGNC Symbol;Acc:HGNC:18830]	ENSG00000171872	protein_coding	promoter	promoter sense	0	2698	1:44115807:44115867	ENSG00000171872	KLF17	3.663497625	0.036404216	-0.00604577	0.058814705	-0.53232073	0.064860476	-0.52627496	-0.591135436	9.961963646	0.995295558	0.954262772	0.606171931	0.949568192	0.610212966	0.567599394	-6.998340372	-6.180264178	-6.894547927	-7.147312949	-6.151448602	-6.883772621	0.1529124	0.925426994

KMO	0.017675828	0.059139371	-0.010580573	0.041463543	-0.028256401	-0.069719944	8	0.085557453	0.816496581	0.816496581	0.816496581	0.816496581	0.816496581	0.816496581	16	149	0	P_105217	P_105217	P_105217	chr1	241594281	241594341	+	KMO	kynurenine 3-monooxygenase (kynurenine 3-hydroxylase) [Source:HGNC Symbol;Acc:HGNC:6381]	ENSG00000117009	protein_coding	3'UTR	3'UTR	1	2699	1:241594281:241594341	ENSG00000117009	KMO	3.900525904	0.008085106	0.240759011	0.805525849	-0.144115924	0.564766838	-0.384874935	-0.949641773	9.961963646	0.814624383	0.439320102	0.888282655	0.584724503	0.708419174	0.364763373	-6.96682174	-5.8511455	-7.035790116	-6.977934391	-6.218496885	-6.599899063	0.349419332	0.790531307

MIF	-0.039038889	0.066155962	0.039319652	0.105194851	0.078358541	-0.02683631	8	0.060106933	0.816496581	0.816496581	0.816496581	0.816496581	0.816496581	0.816496581	16	150	0	A_23_P91619	A_23_P91619	A_23_P91619	chr22	23895158	23895218	+	MIF;AP000350.10	macrophage migration inhibitory factor (glycosylation-inhibiting factor) [Source:HGNC Symbol;Acc:HGNC:7097];Uncharacterized protein [Source:UniProtKB/TrEMBL;Acc:H7C1H1]	ENSG00000240972	protein_coding	3'UTR;exon	multiple Ref	1	2700	22:23895158:23895218	ENSG00000240972	MIF	17.28693758	0.005107994	-0.668987303	1.133677222	0.673798592	1.802664525	1.342785895	-0.45987863	9.961963646	0.518705405	0.283474862	0.515767908	0.101724972	0.209135033	0.655476487	-6.759509472	-5.54650898	-6.804708455	-5.61302421	-5.417784998	-6.957059559	1.241822384	0.345794992

ACRV1	0.213851133	-0.165798209	0.095332353	-0.379649342	-0.118518781	0.261130562	8	0.12409923	0.816496581	0.816496581	0.816496581	0.816496581	0.816496581	0.816496581	16	151	0	A_23_P202894	A_23_P202894	A_23_P202894	chr11	125672659	125672719	-	ACRV1	acrosomal vesicle protein 1 [Source:HGNC Symbol;Acc:HGNC:127]	ENSG00000134940	protein_coding	exon	coding exon	1	2701	11:125672659:125672719	ENSG00000134940	ACRV1	5.313379791	0.014574221	2.169524287	-1.682026346	0.967148743	-3.851550632	-1.202375543	2.649175089	9.961963646	0.055315144	0.123593358	0.35636237	0.003226689	0.257018171	0.024420944	-4.898636192	-4.876686482	-6.559048009	-2.200465952	-5.580346576	-4.175764337	5.29495047	0.019272816

WWC3	0.142466514	-0.052759579	0.27940665	-0.195226093	0.136940136	0.332166229	8	0.119107617	0.816496581	0.816496581	0.816496581	0.816496581	0.816496581	0.816496581	16	152	0	P_269453	P_269453	P_269453	chrX	10098519	10098579	+	WWC3	WWC family member 3 [Source:HGNC Symbol;Acc:HGNC:29237]	ENSG00000047644	protein_coding	exon	coding exon	1	2702	X:10098519:10098579	ENSG00000047644	WWC3	6.49273948	0.013599317	1.496235237	-0.554100323	2.934430434	-2.05033556	1.438195197	3.488530757	9.961963646	0.165584484	0.591730172	0.014984694	0.067576917	0.181042563	0.005870672	-5.897329941	-6.022777675	-3.667898456	-5.233940445	-5.3003737	-2.74333637	4.9331262	0.023613652

FAM124A	0.160695447	0.102371451	0.207506835	-0.058323996	0.046811388	0.105135384	8	0.06555992	0.816496581	0.816496581	0.816496581	0.816496581	0.816496581	0.816496581	16	153	0	A_24_P150428	A_24_P150428	A_24_P150428	chr13	51280726	51280786	+	FAM124A	family with sequence similarity 124A [Source:HGNC Symbol;Acc:HGNC:26413]	ENSG00000150510	protein_coding	exon	coding exon	1	2703	13:51280726:51280786	ENSG00000150510	FAM124A	5.649638449	0.005658295	2.616413624	1.666793084	3.378588015	-0.94962054	0.762174391	1.711794931	9.961963646	0.025831105	0.126638275	0.007056629	0.364773648	0.463619253	0.1178329	-4.16220995	-4.897428674	-2.90859378	-6.677646333	-5.99666838	-5.669493768	4.241934545	0.035645135

STK26	0.218757916	0.06884791	0.219794379	-0.149910006	0.001036462	0.150946469	8	0.100199933	0.816496581	0.816496581	0.816496581	0.816496581	0.816496581	0.816496581	16	154	0	P_271543	P_271543	P_271543	chrX	132072297	132072357	+	MST4	Serine/threonine-protein kinase MST4 [Source:UniProtKB/Swiss-Prot;Acc:Q9P289]	ENSG00000134602	protein_coding	exon	coding exon	1	2704	X:132072297:132072357	ENSG00000134602	STK26	8.353417077	0.010269373	2.643853885	0.832078753	2.65638031	-1.811775131	0.012526426	1.824301557	9.961963646	0.02464465	0.424861318	0.024121259	0.100226567	0.990252932	0.098199516	-4.115986074	-5.829728496	-4.140685378	-5.599489149	-6.296601799	-5.504459102	3.558239496	0.055461379

SLC22A7	0.140046218	0.049814147	0.256770817	-0.09023207	0.1167246	0.20695667	8	0.251101335	0.816496581	0.816496581	0.816496581	0.816496581	0.816496581	0.816496581	16	155	0	P_142715	P_142715	P_142715	chr6	43304864	43304924	+	SLC22A7	"solute carrier family 22 (organic anion transporter), member 7 [Source:HGNC Symbol;Acc:HGNC:10971]"	ENSG00000137204	protein_coding	3'UTR	3'UTR	1	2705	6:43304864:43304924	ENSG00000137204	SLC22A7	4.150375143	0.052840782	0.74616074	0.265407817	1.368064817	-0.480752923	0.621904077	1.102657	9.961963646	0.472810658	0.796106622	0.201355622	0.641075596	0.547972924	0.29610107	-6.70279616	-6.145088902	-6.111115189	-7.02467206	-6.094992608	-6.444930131	0.722089836	0.56148032

CEP85L	-0.071758888	0.068875561	0.039445524	0.140634449	0.111204412	-0.029430037	8	0.065184589	0.816496581	0.816496581	0.816496581	0.816496581	0.816496581	0.816496581	17	14	0	A_32_P90709	A_32_P90709	A_32_P90709	chr6	118461158	118461218	-	CEP85L	centrosomal protein 85kDa-like [Source:HGNC Symbol;Acc:HGNC:21638]	ENSG00000111860	protein_coding	3'UTR	3'UTR	1	2734	6:118461158:118461218	ENSG00000111860	CEP85L	6.716766945	0.005618887	-1.172454972	1.125344837	0.644492992	2.297799809	1.816947964	-0.480851845	9.961963646	0.268288683	0.286824388	0.533814176	0.044514257	0.099384864	0.641007715	-6.296050128	-5.55525853	-6.824917874	-4.836237011	-4.787994647	-6.946516859	1.97813754	0.181511157

RPGRIP1L	0.269939243	-0.026984934	0.16980801	-0.296924177	-0.100131233	0.196792944	8	0.049216503	0.816496581	0.816496581	0.816496581	0.816496581	0.816496581	0.816496581	17	15	0	A_23_P388489	A_23_P388489	A_23_P388489	chr16	53601784	53601844	-	RPGRIP1L	RPGRIP1-like [Source:HGNC Symbol;Acc:HGNC:29168]	ENSG00000103494	protein_coding	3'UTR	3'UTR	1	2735	16:53601784:53601844	ENSG00000103494	RPGRIP1L	7.255309228	0.004151894	5.130840505	-0.512913168	3.227607095	-5.643753673	-1.90323341	3.740520263	9.961963646	0.00044902	0.619192847	0.009101133	0.000217348	0.086273836	0.00386874	-0.030270541	-6.045274039	-3.166449029	0.607731409	-4.662732165	-2.316825598	14.41196609	0.000590197

COPE	-0.13911846	-0.003879039	-0.022658453	0.135239421	0.116460007	-0.018779414	8	0.107823623	0.816496581	0.816496581	0.816496581	0.816496581	0.816496581	0.816496581	17	16	0	A_24_P399622	A_24_P399622	A_24_P399622	chr19	18912988	18913048	-	COPE	"coatomer protein complex, subunit epsilon [Source:HGNC Symbol;Acc:HGNC:2234]"	ENSG00000105669	protein_coding	exon	coding exon	1	2736	19:18912988:18913048	ENSG00000105669	COPE	11.13276703	0.011542943	-1.585886563	-0.044219259	-0.258295958	1.541667304	1.327590605	-0.214076699	9.961963646	0.143964654	0.965603434	0.801436461	0.154296853	0.213931266	0.834805427	-5.775824216	-6.181056728	-7.010808238	-5.984761482	-5.43598302	-7.046399892	1.128128677	0.383914057

DCC	0.041097968	0.008129037	0.039091996	-0.032968931	-0.002005972	0.030962958	8	0.062504155	0.816496581	0.816496581	0.816496581	0.816496581	0.816496581	0.816496581	17	18	0	P_231450	P_231450	P_231450	chr18	53410574	53410634	+	DCC	DCC netrin 1 receptor [Source:HGNC Symbol;Acc:HGNC:2701]	ENSG00000187323	protein_coding	3'UTR;exon	multiple Ref	1	2738	18:53410574:53410634	ENSG00000187323	DCC	3.717761838	0.005344033	0.688543563	0.136191559	0.654936077	-0.552352004	-0.033607486	0.518744518	9.961963646	0.506827919	0.894382709	0.527341258	0.592882695	0.973853991	0.615265638	-6.745668054	-6.172318842	-6.817810642	-6.985287065	-6.296084546	-6.92633476	0.249483286	0.859905026

MAP3K13	0.177717574	-0.082111128	0.069828136	-0.259828702	-0.107889438	0.151939264	8	0.084753774	0.816496581	0.816496581	0.816496581	0.816496581	0.816496581	0.816496581	17	19	0	P_247607	P_247607	P_247607	chr3	185449945	185450005	+	MAP3K13	mitogen-activated protein kinase kinase kinase 13 [Source:HGNC Symbol;Acc:HGNC:6852]	ENSG00000073803	protein_coding	3'UTR;exon	multiple Ref	1	2739	3:185449945:185450005	ENSG00000073803	MAP3K13	6.013713721	0.007975187	2.437280807	-1.126100652	0.957647418	-3.563381459	-1.479633388	2.083748071	9.961963646	0.035097367	0.28651929	0.360904113	0.005182984	0.169884978	0.063900242	-4.461620686	-5.554466906	-6.568179443	-2.68732838	-5.247760373	-5.105415989	4.559089566	0.029388053

GPATCH8	-0.036209677	-0.133684761	-0.03714073	-0.097475084	-0.000931053	0.096544031	8	0.152392338	0.816496581	0.816496581	0.816496581	0.816496581	0.816496581	0.816496581	17	20	0	P_310470	P_310470	P_310470	chr17	44443289	44443349	+	GPATCH8	G patch domain containing 8 [Source:HGNC Symbol;Acc:HGNC:29066]	ENSG00000186566	protein_coding	intron	intron antisense	0	2740	17:44443289:44443349	ENSG00000186566	GPATCH8	6.359136132	0.02085636	-0.30707964	-1.133726435	-0.314975525	-0.826646795	-0.007895885	0.81875091	9.961963646	0.765105802	0.28345517	0.759278661	0.427792785	0.993855944	0.432078183	-6.94714668	-5.546457157	-6.993211227	-6.788248105	-6.296652097	-6.717040928	0.472100573	0.708505027

RAPGEF4	0.179100853	0.105144133	0.133499637	-0.073956719	-0.045601216	0.028355504	8	0.097110758	0.816496581	0.816496581	0.816496581	0.816496581	0.816496581	0.816496581	17	22	0	A_16_P15931280	A_16_P15931280	A_16_P15931280	chr2	173052290	173052350	+	RAPGEF4	Rap guanine nucleotide exchange factor (GEF) 4 [Source:HGNC Symbol;Acc:HGNC:16626]	ENSG00000091428	protein_coding	3'UTR	3'UTR	1	2742	2:173052290:173052350	ENSG00000091428	RAPGEF4	4.337873297	0.009779889	2.218075463	1.302158081	1.653326966	-0.915917382	-0.564748496	0.351168886	9.961963646	0.050958446	0.222163595	0.12938593	0.381347264	0.584736512	0.732775066	-4.820567289	-5.359467286	-5.729291486	-6.709251065	-6.12979226	-7.004425106	1.771688264	0.216137963

OTUD5	0.062881976	0.079027837	0.057882626	0.016145861	-0.00499935	-0.021145211	8	0.066789397	0.816496581	0.816496581	0.816496581	0.816496581	0.816496581	0.816496581	17	23	0	P_270249	P_270249	P_270249	chrX	48934492	48934552	-	OTUD5	OTU deubiquitinase 5 [Source:HGNC Symbol;Acc:HGNC:25402]	ENSG00000068308	protein_coding	exon	coding exon	1	2743	X:48934492:48934552	ENSG00000068308	OTUD5	8.064330398	0.005788969	1.012211378	1.272111355	0.931736818	0.259899977	-0.080474561	-0.340374537	9.961963646	0.335390068	0.232225384	0.373502355	0.800233416	0.937453577	0.740642917	-6.466399905	-5.394186742	-6.592699554	-7.11279875	-6.293241833	-7.008454416	0.616808608	0.619764355

ITPR2	0.229248103	0.162358155	0.294183283	-0.066889948	0.06493518	0.131825128	8	0.083342992	0.816496581	0.816496581	0.816496581	0.816496581	0.816496581	0.816496581	17	25	0	P_214768	P_214768	P_214768	chr12	26550248	26550308	-	ITPR2	"inositol 1,4,5-trisphosphate receptor, type 2 [Source:HGNC Symbol;Acc:HGNC:6181]"	ENSG00000123104	protein_coding	exon	coding exon	1	2745	12:26550248:26550308	ENSG00000123104	ITPR2	5.801957778	0.007784744	3.182212699	2.253707557	4.083583531	-0.928505142	0.901370832	1.829875974	9.961963646	0.009828149	0.047973738	0.002219173	0.37509551	0.388663507	0.097309657	-3.199529507	-4.039523058	-1.723928762	-6.697559136	-5.881817886	-5.496143724	6.150727853	0.012281472

TNS1	0.046054219	-0.122642148	-0.085853951	-0.168696368	-0.13190817	0.036788197	8	0.135886636	0.816496581	0.816496581	0.816496581	0.816496581	0.816496581	0.816496581	17	26	0	A_23_P351724	A_23_P351724	A_23_P351724	chr2	217802882	217802942	-	TNS1	tensin 1 [Source:HGNC Symbol;Acc:HGNC:11973]	ENSG00000079308	protein_coding	3'UTR	3'UTR	1	2746	2:217802882:217802942	ENSG00000079308	TNS1	4.681478464	0.017035229	0.432156521	-1.150830583	-0.805623137	-1.582987103	-1.237779658	0.345207446	9.961963646	0.674832197	0.27667592	0.439266535	0.144622925	0.244176253	0.737116331	-6.897398476	-5.528342699	-6.703829332	-5.928135972	-5.540568396	-7.00666557	1.052885627	0.41166873

FAM160B2	-0.223898535	-0.144621539	-0.111375925	0.079276996	0.11252261	0.033245614	8	0.063505442	0.816496581	0.816496581	0.816496581	0.816496581	0.816496581	0.816496581	17	27	0	P_146665	P_146665	P_146665	chr8	22096698	22096758	+	FAM160B2	"family with sequence similarity 160, member B2 [Source:HGNC Symbol;Acc:HGNC:16492]"	ENSG00000158863	protein_coding	exon	non-coding exon	1	2747	8:22096698:22096758	ENSG00000158863	FAM160B2	7.124883018	0.005445356	-3.716068882	-2.400299766	-1.848518614	1.315769116	1.867550267	0.551781152	9.961963646	0.004027267	0.037384611	0.094387471	0.2177257	0.091488212	0.593259268	-2.293336769	-3.811743595	-5.444645026	-6.277124071	-4.714858473	-6.907558318	4.751847129	0.026224466

LHCGR	0.025580984	-0.072141233	0.174664824	-0.097722218	0.14908384	0.246806057	8	0.108767608	0.816496581	0.816496581	0.816496581	0.816496581	0.816496581	0.816496581	17	29	0	P_234856	P_234856	P_234856	chr2	48723455	48723515	-	LHCGR	luteinizing hormone/choriogonadotropin receptor [Source:HGNC Symbol;Acc:HGNC:6585]	ENSG00000138039	protein_coding	exon	coding exon	1	2749	2:48723455:48723515	ENSG00000138039	LHCGR	3.795677713	0.011707134	0.289559342	-0.81658969	1.977086998	-1.106149032	1.687527656	2.793676688	9.961963646	0.778090298	0.433256158	0.076350614	0.294658283	0.122510036	0.019064724	-6.952799997	-5.842294958	-5.248764678	-6.518725406	-4.970484282	-3.930636362	2.755913506	0.098113496

MAP3K5	-0.320398374	-0.047858128	-0.21307137	0.272540247	0.107327005	-0.165213242	8	0.070309364	0.816496581	0.816496581	0.816496581	0.816496581	0.816496581	0.816496581	17	30	0	P_259388	P_259388	P_259388	chr6	136567630	136567690	-	MAP3K5	mitogen-activated protein kinase kinase kinase 5 [Source:HGNC Symbol;Acc:HGNC:6857]	ENSG00000197442	protein_coding	exon	coding exon	1	2750	6:136567630:136567690	ENSG00000197442	MAP3K5	7.733420473	0.006176509	-4.993032497	-0.745812729	-3.320467138	4.247219768	1.672565359	-2.574654409	9.961963646	0.00054917	0.473011688	0.007781007	0.001711503	0.125476625	0.027746683	-0.239009547	-5.897078938	-3.007768422	-1.544878175	-4.99112635	-4.30137736	10.66288469	0.001878197

XRCC3	-0.518893658	-0.029746856	-0.427516538	0.489146803	0.09137712	-0.397769683	8	0.112822578	0.816496581	0.816496581	0.816496581	0.816496581	0.816496581	0.816496581	17	33	0	A_23_P48470	A_23_P48470	A_23_P48470	chr14	103698943	103699003	-	XRCC3	X-ray repair complementing defective repair in Chinese hamster cells 3 [Source:HGNC Symbol;Acc:HGNC:12830]	ENSG00000126215	protein_coding	exon	coding exon	1	2753	14:103698943:103699003	ENSG00000126215	XRCC3	7.516576335	0.012428712	-5.700473476	-0.326793667	-4.696620683	5.373679809	1.003852794	-4.369827015	9.961963646	0.000201047	0.750586475	0.00085467	0.000316998	0.339209457	0.001412159	0.803291206	-6.126100759	-0.736845875	0.212838868	-5.78694551	-1.277735949	17.27333018	0.000283736

TFDP2	0.06191117	-0.045330506	0.068811448	-0.107241676	0.006900278	0.114141954	8	0.041199636	0.816496581	0.816496581	0.816496581	0.816496581	0.816496581	0.816496581	17	34	0	P_136195	P_136195	P_136195	chr3	141952512	141952572	-	TFDP2	transcription factor Dp-2 (E2F dimerization partner 2) [Source:HGNC Symbol;Acc:HGNC:11751]	ENSG00000114126	protein_coding	3'UTR;exon	multiple Ref	1	2754	3:141952512:141952572	ENSG00000114126	TFDP2	8.857217752	0.003569797	1.26909138	-0.929211236	1.410537312	-2.198302616	0.141445932	2.339748548	9.961963646	0.233257071	0.374747005	0.188834765	0.052691288	0.89033714	0.041448855	-6.184208659	-5.746343691	-6.057205925	-4.998029671	-6.286054853	-4.69220465	2.465101096	0.122546601

SEC24B	-0.057081226	0.02407528	-0.066403132	0.081156506	-0.009321906	-0.090478413	8	0.053993315	0.816496581	0.816496581	0.816496581	0.816496581	0.816496581	0.816496581	17	35	0	P_250409	P_250409	P_250409	chr4	109526319	109526379	+	SEC24B	SEC24 family member B [Source:HGNC Symbol;Acc:HGNC:10704]	ENSG00000138802	protein_coding	exon	coding exon	1	2755	4:109526319:109526379	ENSG00000138802	SEC24B	6.944702016	0.004547811	-1.036663258	0.437235855	-1.205960216	1.473899112	-0.169296959	-1.643196071	9.961963646	0.324401098	0.671266557	0.2556937	0.171392685	0.868951394	0.131488171	-6.441688509	-6.08230004	-6.306168142	-6.075621862	-6.281463226	-5.767329926	1.270219888	0.336942284

LMTK2	-0.017291577	-0.066775899	0.008309875	-0.049484322	0.025601452	0.075085774	8	0.092545525	0.816496581	0.816496581	0.816496581	0.816496581	0.816496581	0.816496581	17	36	0	P_145281	P_145281	P_145281	chr7	98203741	98203801	+	LMTK2	lemur tyrosine kinase 2 [Source:HGNC Symbol;Acc:HGNC:17880]	ENSG00000164715	protein_coding	intron	intron sense	0	2756	7:98203741:98203801	ENSG00000164715	LMTK2	6.756084076	0.009084584	-0.222191635	-0.85805049	0.106779425	-0.635858855	0.32897106	0.964829915	9.961963646	0.828653649	0.411033338	0.917083714	0.53920075	0.748988958	0.35746694	-6.97148705	-5.808200765	-7.040897525	-6.932901406	-6.239444184	-6.585361598	0.37340947	0.774107775

AP1G2	0.03361024	0.043181688	-0.130167911	0.009571448	-0.16377815	-0.173349599	8	0.112065414	0.816496581	0.816496581	0.816496581	0.816496581	0.816496581	0.816496581	17	37	0	P_114470	P_114470	P_114470	chr14	23565399	23565459	-	AP1G2	"adaptor-related protein complex 1, gamma 2 subunit [Source:HGNC Symbol;Acc:HGNC:556]"	ENSG00000213983	protein_coding	exon	non-coding exon	1	2757	14:23565399:23565459	ENSG00000213983	AP1G2	7.047380612	0.01229197	0.371284216	0.477017697	-1.43793353	0.105733482	-1.809217746	-1.914951227	9.961963646	0.71820023	0.64364126	0.181114974	0.917892569	0.100645113	0.084622783	-6.923655508	-6.063537576	-6.021852383	-7.14350278	-4.79908309	-5.367734225	1.564089552	0.258883448

CSTF2	-0.089859543	-0.007589037	0.077475453	0.082270505	0.167334996	0.08506449	8	0.110778805	0.816496581	0.816496581	0.816496581	0.816496581	0.816496581	0.816496581	17	39	0	P_270899	P_270899	P_270899	chrX	100822274	100822334	+	CSTF2	"cleavage stimulation factor, 3' pre-RNA, subunit 2, 64kDa [Source:HGNC Symbol;Acc:HGNC:2484]"	ENSG00000101811	protein_coding	exon	coding exon	1	2759	X:100822274:100822334	ENSG00000101811	CSTF2	7.739142355	0.012061724	-1.00208634	-0.084630641	0.863982729	0.917455699	1.866069069	0.94861337	9.961963646	0.340020739	0.934231654	0.407918627	0.380579307	0.09171086	0.365261286	-6.476492301	-6.178313052	-6.654126225	-6.707829436	-4.717012536	-6.600876474	1.163601932	0.371544706

LINC00612	0.087939307	0.082805777	0.280589962	-0.00513353	0.192650655	0.197784185	8	0.455783571	0.816496581	0.816496581	0.816496581	0.816496581	0.816496581	0.816496581	17	41	0	A_23_P342398	A_23_P342398	A_23_P342398	chr12	9055617	9055677	-	LINC00612	long intergenic non-protein coding RNA 612 [Source:HGNC Symbol;Acc:HGNC:28621]	ENSG00000214851	processed_transcript	exon	non-coding exon	1	2761	12:9055617:9055677	ENSG00000214851	LINC00612	3.515913675	0.169032158	0.26196544	0.24667299	0.835859133	-0.01529245	0.573893693	0.589186143	9.961963646	0.798685087	0.810169996	0.422829184	0.9881008	0.578765659	0.568855817	-6.96104115	-6.15011196	-6.67845479	-7.149485175	-6.124434006	-6.884986792	0.250810348	0.858982837

RBP1	0.010267366	-0.023604322	-0.038222313	-0.033871688	-0.048489679	-0.014617991	8	0.12244728	0.816496581	0.816496581	0.816496581	0.816496581	0.816496581	0.816496581	17	42	0	P_136165	P_136165	P_136165	chr3	139525808	139525868	-	RBP1	"retinol binding protein 1, cellular [Source:HGNC Symbol;Acc:HGNC:9919]"	ENSG00000114115	protein_coding	3'UTR	3'UTR	1	2762	3:139525808:139525868	ENSG00000114115	RBP1	4.610367244	0.014247151	0.105351485	-0.242199451	-0.392191871	-0.347550936	-0.497543356	-0.14999242	9.961963646	0.918188002	0.813538803	0.70317608	0.735408577	0.629604053	0.883763934	-6.992307114	-6.151257717	-6.963767597	-7.083955821	-6.166679891	-7.059101894	0.102402124	0.956779399

RARG	-0.147367892	0.005580318	0.048707771	0.15294821	0.196075663	0.043127453	8	0.106505335	0.816496581	0.816496581	0.816496581	0.816496581	0.816496581	0.816496581	17	44	0	P_111695	P_111695	P_111695	chr12	53219949	53220009	-	RARG	"retinoic acid receptor, gamma [Source:HGNC Symbol;Acc:HGNC:9866]"	ENSG00000172819	protein_coding	exon	coding exon	1	2764	12:53219949:53220009	ENSG00000172819	RARG	7.201197693	0.011316042	-1.696685034	0.064247663	0.560785292	1.760932696	2.257470326	0.496537629	9.961963646	0.120725742	0.950043956	0.587334327	0.108854778	0.047668613	0.630288325	-5.619970811	-6.179911854	-6.878054502	-5.674577098	-4.124746061	-6.938338935	1.940158976	0.187367456

SPHK2	-0.010630765	-0.083254467	0.039929496	-0.072623702	0.050560261	0.123183963	8	0.106341988	0.816496581	0.816496581	0.816496581	0.816496581	0.816496581	0.816496581	17	45	0	P_126291	P_126291	P_126291	chr19	48628586	48628646	+	SPHK2	sphingosine kinase 2 [Source:HGNC Symbol;Acc:HGNC:18859]	ENSG00000063176	protein_coding	3'UTR	3'UTR	1	2765	19:48628586:48628646	ENSG00000063176	SPHK2	7.821386405	0.011288121	-0.122546054	-0.95971514	0.46028691	-0.837169086	0.582832965	1.420002051	9.961963646	0.904903529	0.359912158	0.655193369	0.42212656	0.572961422	0.186136592	-6.990171587	-5.718569203	-6.932641635	-6.779299723	-6.119118766	-6.068911642	0.700814482	0.572825345

GGNBP1	-0.086692351	0.218611254	0.268373736	0.305303605	0.355066086	0.049762482	8	0.19558159	0.816496581	0.816496581	0.816496581	0.816496581	0.816496581	0.816496581	17	47	0	P_142373	P_142373	P_142373	chr6	33586686	33586746	+	GGNBP1	gametogenetin binding protein 1 (pseudogene) [Source:HGNC Symbol;Acc:HGNC:19427]	ENSG00000204188	unitary_pseudogene	exon	non-coding exon	1	2767	6:33586686:33586746	ENSG00000204188	GGNBP1	4.686626008	0.032925253	-0.585142777	1.475548828	1.81142802	2.060691605	2.396570797	0.335879193	9.961963646	0.571466875	0.170957746	0.100283281	0.066416377	0.037623207	0.743928852	-6.814684416	-5.14857256	-5.499989183	-5.217633789	-3.905671485	-7.010096197	2.650620699	0.106231267

CLCN2	-0.197667873	-0.06100499	-0.126802602	0.136662883	0.070865271	-0.065797612	8	0.108610064	0.816496581	0.816496581	0.816496581	0.816496581	0.816496581	0.816496581	17	48	0	P_247566	P_247566	P_247566	chr3	184357996	184358056	-	CLCN2	"chloride channel, voltage-sensitive 2 [Source:HGNC Symbol;Acc:HGNC:2020]"	ENSG00000114859	protein_coding	exon	coding exon	1	2768	3:184357996:184358056	ENSG00000114859	CLCN2	8.059501011	0.011679632	-2.240098573	-0.691347508	-1.437008069	1.548751065	0.803090504	-0.745660561	9.961963646	0.04909317	0.505138571	0.181371276	0.152599005	0.440662419	0.473099606	-4.784965161	-5.93622238	-6.023053931	-5.975118207	-5.964643798	-6.775860118	1.860098179	0.200447167

CCDC149	-0.246566824	0.058721795	-0.085615967	0.305288619	0.160950857	-0.144337762	8	0.058579791	0.816496581	0.816496581	0.816496581	0.816496581	0.816496581	0.816496581	17	49	0	P_137454	P_137454	P_137454	chr4	24831505	24831565	-	CCDC149	coiled-coil domain containing 149 [Source:HGNC Symbol;Acc:HGNC:25405]	ENSG00000181982	protein_coding	exon	coding exon	1	2769	4:24831505:24831565	ENSG00000181982	CCDC149	7.432586083	0.004962439	-4.286794491	1.020933238	-1.488513544	5.307727729	2.798280947	-2.509446783	9.961963646	0.001608162	0.331438911	0.167572794	0.000348146	0.01891498	0.031023581	-1.350323345	-5.66062098	-5.955425648	0.114812778	-3.259237022	-4.410726033	10.65570955	0.001882885

DGKB	-0.119560214	-0.149753293	-0.187543695	-0.030193078	-0.06798348	-0.037790402	8	0.089280518	0.816496581	0.816496581	0.816496581	0.816496581	0.816496581	0.816496581	17	50	0	P_260542	P_260542	P_260542	chr7	14672929	14672989	-	DGKB	"diacylglycerol kinase, beta 90kDa [Source:HGNC Symbol;Acc:HGNC:2850]"	ENSG00000136267	protein_coding	exon	coding exon	1	2770	7:14672929:14672989	ENSG00000136267	DGKB	4.506901445	0.00860784	-1.578284218	-1.976855423	-2.475716987	-0.398571205	-0.897432769	-0.498861564	9.961963646	0.145696294	0.076379984	0.032866033	0.698618119	0.390661072	0.628707735	-5.786293518	-4.457393558	-4.443974299	-7.063427929	-5.885293305	-6.93710608	2.290203413	0.140689905

EPB41L3	0.04437391	0.10459181	0.005552216	0.060217899	-0.038821695	-0.099039594	8	0.119536181	0.816496581	0.816496581	0.816496581	0.816496581	0.816496581	0.816496581	17	51	0	P_230489	P_230489	P_230489	chr18	5395070	5395130	-	EPB41L3	erythrocyte membrane protein band 4.1-like 3 [Source:HGNC Symbol;Acc:HGNC:3380]	ENSG00000082397	protein_coding	3'UTR;exon	multiple Ref	1	2771	18:5395070:5395130	ENSG00000082397	EPB41L3	3.841500123	0.013681449	0.464630044	1.095159219	0.058136103	0.630529176	-0.40649394	-1.037023116	9.961963646	0.652185212	0.299217345	0.954789829	0.542541442	0.692974883	0.324241418	-6.881824278	-5.586540116	-7.045275171	-6.936448781	-6.209542635	-6.513682679	0.509475985	0.684667037

ACO2	-0.093411401	0.148765426	0.103787845	0.242176827	0.197199246	-0.044977581	8	0.053725573	0.816496581	0.816496581	0.816496581	0.816496581	0.816496581	0.816496581	17	52	0	A_23_P305481	A_23_P305481	A_23_P305481	chr22	41528536	41528596	+	ACO2	"aconitase 2, mitochondrial [Source:HGNC Symbol;Acc:HGNC:118]"	ENSG00000100412	protein_coding	exon	coding exon	1	2772	22:41528536:41528596	ENSG00000100412	ACO2	11.36654873	0.00452465	-1.70079903	2.708663911	1.889729338	4.409462941	3.590528368	-0.818934573	9.961963646	0.119931808	0.022053968	0.088213789	0.001327655	0.004954712	0.431978177	-5.614070682	-3.323668305	-5.38252041	-1.281248012	-1.976613609	-6.716887045	7.801094286	0.005685468

TTC6	0.211399438	-0.294563473	-0.042018853	-0.505962911	-0.253418291	0.25254462	8	0.087419427	0.816496581	0.816496581	0.816496581	0.816496581	0.816496581	0.816496581	17	53	0	P_309839	P_309839	P_309839	chr14	37826034	37826094	+	TTC6	tetratricopeptide repeat domain 6 [Source:HGNC Symbol;Acc:HGNC:19739]	ENSG00000139865	protein_coding	intron;promoter	multiple non-Ref	0	2773	14:37826034:37826094	ENSG00000139865	TTC6	7.140314717	0.008343752	2.83444976	-3.949515525	-0.56338999	-6.783965285	-3.39783975	3.386125535	9.961963646	0.017779126	0.002752706	0.585626294	4.93E-05	0.006832331	0.006967919	-3.793046212	-1.365269117	-6.876504937	2.161276909	-2.285783614	-2.917802564	15.49725545	0.000441371

GSTCD	-0.261857258	-0.088771085	-0.346628161	0.173086174	-0.084770902	-0.257857076	8	0.036734911	0.816496581	0.816496581	0.816496581	0.816496581	0.816496581	0.816496581	17	54	0	A_23_P213038	A_23_P213038	A_23_P213038	chr4	105845874	105845934	+	GSTCD	"glutathione S-transferase, C-terminal domain containing [Source:HGNC Symbol;Acc:HGNC:25806]"	ENSG00000138780	protein_coding	3'UTR	3'UTR	1	2774	4:105845874:105845934	ENSG00000138780	GSTCD	7.917635264	0.003290369	-5.590980185	-1.895373757	-7.40094504	3.695606428	-1.809964855	-5.505571283	9.961963646	0.000233794	0.087398041	2.36E-05	0.004165135	0.100522674	0.000263304	0.646737759	-4.576365728	2.991691925	-2.463112396	-4.798012412	0.468497316	22.8117139	8.80E-05

ZBTB10	-0.757322891	0.112603506	-0.785111195	0.869926396	-0.027788304	-0.897714701	8	0.063048979	0.816496581	0.816496581	0.816496581	0.816496581	0.816496581	0.816496581	17	56	0	P_147475	P_147475	P_147475	chr8	80519714	80519774	+	ZBTB10	zinc finger and BTB domain containing 10 [Source:HGNC Symbol;Acc:HGNC:30953]	ENSG00000205189	protein_coding	3'UTR	3'UTR	1	2776	8:80519714:80519774	ENSG00000205189	ZBTB10	8.119954659	0.005398965	-12.6232577	1.876904938	-13.08644049	14.50016264	-0.463182788	-14.96334543	9.961963646	1.88E-07	0.090093601	1.34E-07	5.05E-08	0.653186892	3.74E-08	7.855284757	-4.60303045	8.195027994	9.160761598	-6.183826848	9.418570073	128.0825489	2.96E-08

CYP26B1	-0.012872073	-0.105347437	0.0559052	-0.092475364	0.068777273	0.161252637	8	0.114909489	0.816496581	0.816496581	0.816496581	0.816496581	0.816496581	0.816496581	17	57	0	A_23_P210100	A_23_P210100	A_23_P210100	chr2	72129349	72129409	-	CYP26B1	"cytochrome P450, family 26, subfamily B, polypeptide 1 [Source:HGNC Symbol;Acc:HGNC:20581]"	ENSG00000003137	protein_coding	3'UTR	3'UTR	1	2777	2:72129349:72129409	ENSG00000003137	CYP26B1	5.341077965	0.012810369	-0.139287871	-1.139957818	0.604946558	-1.000669947	0.744234429	1.744904376	9.961963646	0.891998332	0.280970427	0.558741606	0.340672286	0.473924084	0.111712499	-6.987783662	-5.539881502	-6.850875903	-6.627975233	-6.010242274	-5.621495521	1.047463631	0.41375132

WNT3	-0.007300125	0.11748747	-0.002025123	0.124787595	0.005275001	-0.119512593	8	0.178438176	0.816496581	0.816496581	0.816496581	0.816496581	0.816496581	0.816496581	17	58	0	P_121816	P_121816	P_121816	chr17	46768319	46768379	-	WNT3	"wingless-type MMTV integration site family, member 3 [Source:HGNC Symbol;Acc:HGNC:12782]"	ENSG00000108379	protein_coding	exon	coding exon	1	2778	17:46768319:46768379	ENSG00000108379	WNT3	8.342563594	0.027776087	-0.053646374	0.863379335	-0.014882008	0.917025709	0.038764366	-0.878261343	9.961963646	0.958277478	0.408234694	0.988420142	0.380793856	0.969844096	0.400488517	-6.996790081	-5.803713591	-7.046999654	-6.708227012	-6.295886066	-6.665618484	0.393717517	0.760330909

LRIG3	0.013173039	-0.017385464	-0.018959173	-0.030558503	-0.032132212	-0.001573709	8	0.069727986	0.816496581	0.816496581	0.816496581	0.816496581	0.816496581	0.816496581	17	59	0	P_215889	P_215889	P_215889	chr12	58890006	58890066	-	LRIG3	leucine-rich repeats and immunoglobulin-like domains 3 [Source:HGNC Symbol;Acc:HGNC:30991]	ENSG00000139263	protein_coding	exon	coding exon	1	2779	12:58890006:58890066	ENSG00000139263	LRIG3	6.679821795	0.006111129	0.206381561	-0.272377483	-0.297032731	-0.478759043	-0.503414292	-0.024655248	9.961963646	0.840649795	0.790893999	0.772542642	0.642444544	0.625616968	0.980816833	-6.975167411	-6.143127801	-6.999151834	-7.025694529	-6.163633442	-7.071046448	0.114709223	0.949457901

COL6A4P1	0.037539596	-0.017608129	-0.069684415	-0.055147724	-0.107224011	-0.052076287	8	0.152193768	0.816496581	0.816496581	0.816496581	0.816496581	0.816496581	0.816496581	17	60	0	P_243331	P_243331	P_243331	chr3	15169974	15170034	-	COL6A4P1	"collagen, type VI, alpha 4 pseudogene 1 [Source:HGNC Symbol;Acc:HGNC:33484]"	ENSG00000230524	unitary_pseudogene	exon	non-coding exon	1	2780	3:15169974:15170034	ENSG00000230524	COL6A4P1	4.295779104	0.02080779	0.318729485	-0.149501605	-0.59165469	-0.46823109	-0.910384175	-0.442153086	9.961963646	0.756513759	0.884141181	0.567264966	0.649695994	0.384118631	0.667822847	-6.943207318	-6.170318503	-6.859258268	-7.031025974	-5.873815168	-6.965618318	0.28625559	0.834306175

DENND4C	0.108421196	-0.029462297	0.062455583	-0.137883493	-0.045965613	0.09191788	8	0.054293766	0.816496581	0.816496581	0.816496581	0.816496581	0.816496581	0.816496581	17	61	0	P_266960	P_266960	P_266960	chr9	19305467	19305527	+	DENND4C	DENN/MADD domain containing 4C [Source:HGNC Symbol;Acc:HGNC:26079]	ENSG00000137145	protein_coding	exon	coding exon	1	2781	9:19305467:19305527	ENSG00000137145	DENND4C	7.881563123	0.004573939	1.963426544	-0.5335401	1.131023768	-2.496966644	-0.832402776	1.664563868	9.961963646	0.078101386	0.605358575	0.284538184	0.031693089	0.424686882	0.127089478	-5.222703613	-6.034212155	-6.390183652	-4.506325224	-5.940802803	-5.737094413	2.519572212	0.11746786

NDUFB5	-0.087040863	0.084600618	0.059522913	0.171641481	0.146563776	-0.025077705	8	0.082743929	0.816496581	0.816496581	0.816496581	0.816496581	0.816496581	0.816496581	17	62	0	P_136605	P_136605	P_136605	chr3	179623980	179624040	+	NDUFB5	"NADH dehydrogenase (ubiquinone) 1 beta subcomplex, 5, 16kDa [Source:HGNC Symbol;Acc:HGNC:7700]"	ENSG00000136521	protein_coding	"3'UTR;3'UTR,exon;exon"	multiple Ref	1	2782	3:179623980:179624040	ENSG00000136521	NDUFB5	10.52145574	0.007704843	-1.214470347	1.180421905	0.830515807	2.394892252	2.044986154	-0.349906098	9.961963646	0.252571317	0.265249943	0.4257034	0.037731095	0.068183906	0.73369384	-6.248227334	-5.496522648	-6.682998223	-4.676304652	-4.451556406	-7.004902819	2.266333692	0.143403246

MTR	-0.297186547	-0.07620799	-0.325513018	0.220978557	-0.028326471	-0.249305028	8	0.044471157	0.816496581	0.816496581	0.816496581	0.816496581	0.816496581	0.816496581	17	63	0	P_206915	P_206915	P_206915	chr1	236861217	236861277	+	MTR	5-methyltetrahydrofolate-homocysteine methyltransferase [Source:HGNC Symbol;Acc:HGNC:7468]	ENSG00000116984	protein_coding	exon	coding exon	1	2783	1:236861217:236861277	ENSG00000116984	MTR	8.082449852	0.003794872	-5.908486058	-1.515121901	-6.471656091	4.393364156	-0.563170034	-4.95653419	9.961963646	0.000151629	0.160807463	7.27E-05	0.001361314	0.585770427	0.000579514	1.095914336	-5.09809404	1.825300503	-1.307261044	-6.130708948	-0.353029132	20.54569578	0.000137558

BMPR2	0.305095734	-0.119819889	0.140733933	-0.424915623	-0.164361801	0.260553822	8	0.077248253	0.816496581	0.816496581	0.816496581	0.816496581	0.816496581	0.816496581	17	64	0	P_238758	P_238758	P_238758	chr2	202559886	202559946	+	BMPR2	"bone morphogenetic protein receptor, type II (serine/threonine kinase) [Source:HGNC Symbol;Acc:HGNC:1078]"	ENSG00000204217	protein_coding	3'UTR;exon	multiple Ref	1	2784	2:202559886:202559946	ENSG00000204217	BMPR2	8.238260362	0.006998745	4.466544552	-1.754140799	2.060318491	-6.22068535	-2.40622606	3.81445929	9.961963646	0.00121523	0.110057479	0.066457859	0.000100389	0.037008469	0.003427791	-1.061124969	-4.777155678	-5.118930712	1.41639843	-3.890344898	-2.192623495	14.38481537	0.000594634

PLAAT5	0.14050982	0.056730438	0.302417878	-0.083779382	0.161908058	0.24568744	8	0.273396043	0.816496581	0.816496581	0.816496581	0.816496581	0.816496581	0.816496581	17	65	0	P_212020	P_212020	P_212020	chr11	63468367	63468427	-	HRASLS5	"HRAS-like suppressor family, member 5 [Source:HGNC Symbol;Acc:HGNC:24978]"	ENSG00000168004	protein_coding	3'UTR;5'UTR;exon	multiple Ref	1	2785	11:63468367:63468427	ENSG00000168004	PLAAT5	5.898251429	0.062231313	0.689839128	0.278520575	1.484733845	-0.411318553	0.794894717	1.20621327	9.961963646	0.506046928	0.786308516	0.168553583	0.689547991	0.445199727	0.255600409	-6.744738276	-6.141357789	-5.960440257	-7.057874111	-5.971176331	-6.329854628	0.835646223	0.504565033

SVEP1	0.095779794	0.105750722	0.132088775	0.009970928	0.036308981	0.026338053	8	0.069622601	0.816496581	0.816496581	0.816496581	0.816496581	0.816496581	0.816496581	17	66	0	P_268462	P_268462	P_268462	chr9	110451288	110451348	-	SVEP1	"sushi, von Willebrand factor type A, EGF and pentraxin domain containing 1 [Source:HGNC Symbol;Acc:HGNC:15985]"	ENSG00000165124	protein_coding	exon	coding exon	1	2786	9:110451288:110451348	ENSG00000165124	SVEP1	3.948593308	0.006099336	1.502028819	1.658393966	2.071430089	0.156365147	0.56940127	0.413036123	9.961963646	0.164106146	0.128345833	0.06523299	0.878868583	0.581694599	0.688329783	-5.88960996	-4.908821605	-5.101433918	-7.136258625	-6.127076213	-6.97897438	1.636075073	0.24304623

DIAPH2	0.010487711	0.017243708	0.119691612	0.006755998	0.109203901	0.102447904	8	0.040854093	0.816496581	0.816496581	0.816496581	0.816496581	0.816496581	0.816496581	17	67	0	P_270859	P_270859	P_270859	chrX	96939278	96939338	+	DIAPH2	diaphanous-related formin 2 [Source:HGNC Symbol;Acc:HGNC:2877]	ENSG00000147202	protein_coding	exon	coding exon	1	2787	X:96939278:96939338	ENSG00000147202	DIAPH2	6.953732412	0.003547028	0.215672134	0.354604308	2.461370877	0.138932174	2.245698743	2.106766569	9.961963646	0.83359502	0.730277781	0.033682025	0.892272186	0.048629489	0.061478982	-6.973037291	-6.116230863	-4.467865347	-7.139067639	-4.143120758	-5.068952666	2.621922607	0.108579563

MGAM	0.035522293	0.002861992	-0.016314158	-0.032660302	-0.051836451	-0.01917615	8	0.087221039	0.816496581	0.816496581	0.816496581	0.816496581	0.816496581	0.816496581	17	68	0	P_146137	P_146137	P_146137	chr7	142105831	142105891	+	MGAM	maltase-glucoamylase (alpha-glucosidase) [Source:HGNC Symbol;Acc:HGNC:7043]	ENSG00000257335	protein_coding	exon	coding exon	1	2788	7:142105831:142105891	ENSG00000257335	MGAM	3.691692241	0.008315929	0.477080033	0.03843781	-0.219106319	-0.438642223	-0.696186352	-0.257544129	9.961963646	0.643598402	0.970097995	0.830991161	0.670280808	0.5022313	0.802000534	-6.875566876	-6.181308607	-7.020966609	-7.045401404	-6.045169632	-7.035263722	0.170083636	0.914145048

SRI	0.031779413	0.049405118	-0.061964613	0.017625705	-0.093744026	-0.111369731	8	0.069320274	0.816496581	0.816496581	0.816496581	0.816496581	0.816496581	0.816496581	17	69	0	P_145171	P_145171	P_145171	chr7	88209392	88209452	+	SRI	sorcin [Source:HGNC Symbol;Acc:HGNC:11292]	ENSG00000075142	protein_coding	"3'UTR;exon;exon,intron"	multiple non-Ref	0	2789	7:88209392:88209452	ENSG00000075142	SRI	10.1977961	0.006065602	0.499751998	0.776927705	-0.97443395	0.277175707	-1.474185949	-1.751361656	9.961963646	0.628102639	0.455253809	0.352908265	0.78731166	0.171316994	0.110553091	-6.863766676	-5.873532882	-6.551995946	-7.107761441	-5.254730713	-5.612077701	1.186701412	0.363726257

CABIN1	-0.189917735	-0.025692574	-0.015502324	0.164225161	0.17441541	0.01019025	8	0.066767994	0.816496581	0.816496581	0.816496581	0.816496581	0.816496581	0.816496581	17	71	0	P_132832	P_132832	P_132832	chr22	24011640	24011700	+	CABIN1	calcineurin binding protein 1 [Source:HGNC Symbol;Acc:HGNC:24187]	ENSG00000099991	protein_coding	5'UTR	5'UTR	1	2791	22:24011640:24011700	ENSG00000099991	CABIN1	6.381547633	0.005786673	-3.057712725	-0.413655476	-0.24959046	2.64405725	2.808122266	0.164065016	9.961963646	0.0121429	0.687890728	0.807975175	0.024636063	0.01859887	0.872960916	-3.412233088	-6.0926806	-7.013207163	-4.258727011	-3.243250789	-7.056693465	4.081086682	0.039421579

AARS2	-0.21702674	-0.231437497	-0.009337254	-0.014410756	0.207689486	0.222100242	8	0.201725666	0.816496581	0.816496581	0.816496581	0.816496581	0.816496581	0.816496581	17	73	0	P_142801	P_142801	P_142801	chr6	44312071	44312131	-	AARS2	"alanyl-tRNA synthetase 2, mitochondrial [Source:HGNC Symbol;Acc:HGNC:21022]"	ENSG00000124608	protein_coding	exon	coding exon	1	2793	6:44312071:44312131	ENSG00000124608	AARS2	8.064035636	0.034885578	-1.423101627	-1.51759676	-0.061226842	-0.094495133	1.361874786	1.456369918	9.961963646	0.185260158	0.160190485	0.952389502	0.926589493	0.203237703	0.176073523	-5.993132804	-5.094910071	-7.045073773	-7.144732112	-5.394724507	-6.021659893	1.386118749	0.303351589

LMF1	0.166409901	-0.032951108	0.114654832	-0.199361009	-0.051755069	0.14760594	8	0.062946929	0.816496581	0.816496581	0.816496581	0.816496581	0.816496581	0.816496581	17	74	0	A_24_P18917	A_24_P18917	A_24_P18917	chr16	854169	854229	-	LMF1	lipase maturation factor 1 [Source:HGNC Symbol;Acc:HGNC:14154]	ENSG00000103227	protein_coding	3'UTR	3'UTR	1	2794	16:854169:854229	ENSG00000103227	LMF1	7.509096872	0.00538864	2.776420356	-0.549763725	1.912927103	-3.326184081	-0.863493253	2.462690828	9.961963646	0.019636642	0.594591117	0.084905862	0.007706475	0.408175009	0.033606121	-3.891664052	-6.025223244	-5.347268379	-3.092194725	-5.91470952	-4.488759564	4.924001249	0.023737427

TEAD1	0.044570203	-0.086014103	-0.047719117	-0.130584306	-0.09228932	0.038294986	8	0.08120464	0.816496581	0.816496581	0.816496581	0.816496581	0.816496581	0.816496581	17	75	0	P_107993	P_107993	P_107993	chr11	12853837	12853897	-	TEAD1	TEA domain family member 1 (SV40 transcriptional enhancer factor) [Source:HGNC Symbol;Acc:HGNC:11714]	ENSG00000187079	protein_coding	intron	intron antisense	0	2795	11:12853837:12853897	ENSG00000187079	TEAD1	6.893861929	0.007502181	0.630226231	-1.21624628	-0.674752124	-1.846472511	-1.304978354	0.541494156	9.961963646	0.542731689	0.251923611	0.515186923	0.094704183	0.221237939	0.600066911	-6.785863133	-5.457200784	-6.804037097	-5.547632139	-5.462799005	-6.913522126	1.289562617	0.331057211

PXN-AS1	0.16860895	0.238119133	-0.011579505	0.069510183	-0.180188455	-0.249698639	8	0.13752972	0.816496581	0.816496581	0.816496581	0.816496581	0.816496581	0.816496581	17	76	0	P_112976	P_112976	P_112976	chr12	120209874	120209934	+	PXN-AS1	PXN antisense RNA 1 [Source:HGNC Symbol;Acc:HGNC:44123]	ENSG00000255857	antisense	exon	non-coding exon	1	2796	12:120209874:120209934	ENSG00000255857	PXN-AS1	9.723085155	0.017395998	1.565674879	2.211134968	-0.107525376	0.645460088	-1.673200255	-2.318660344	9.961963646	0.148609018	0.051560336	0.91650691	0.533212797	0.125349441	0.04296324	-5.803591764	-4.104943558	-7.040810325	-6.926441563	-4.990252464	-4.726816767	2.657403498	0.105685029

PPP2CA	0.009906456	-0.040400763	0.01719376	-0.05030722	0.007287304	0.057594523	8	0.067132048	0.816496581	0.816496581	0.816496581	0.816496581	0.816496581	0.816496581	17	77	0	P_140356	P_140356	P_140356	chr5	134196799	134196859	-	PPP2CA	"protein phosphatase 2, catalytic subunit, alpha isozyme [Source:HGNC Symbol;Acc:HGNC:9299]"	ENSG00000113575	protein_coding	3'UTR	3'UTR	1	2797	5:134196799:134196859	ENSG00000113575	PPP2CA	8.96327733	0.005825819	0.15895912	-0.648271144	0.275891285	-0.807230264	0.116932165	0.924162428	9.961963646	0.876877485	0.531467021	0.7882701	0.438382276	0.909237632	0.37724402	-6.984589366	-5.965276288	-7.005712046	-6.804501018	-6.289417854	-6.623854054	0.34016825	0.796901682

KDM7A	0.372538842	0.060692068	0.400748374	-0.311846775	0.028209532	0.340056307	8	0.066332426	0.816496581	0.816496581	0.816496581	0.816496581	0.816496581	0.816496581	17	78	0	P_263200	P_263200	P_263200	chr7	140129492	140129552	-	KDM7A	lysine (K)-specific demethylase 7A [Source:HGNC Symbol;Acc:HGNC:22224]	ENSG00000006459	protein_coding	exon	coding exon	1	2798	7:140129492:140129552	ENSG00000006459	KDM7A	6.522009558	0.005740116	6.02222306	0.981108892	6.478240191	-5.041114168	0.456017131	5.497131299	9.961963646	0.00013028	0.349765052	7.21E-05	0.000511757	0.658157042	0.00026643	1.253274673	-5.698649666	1.83396975	-0.287810896	-6.187255046	0.456198017	22.50609624	9.33E-05

SFXN2	-0.723091664	-0.393256744	-0.95166087	0.32983492	-0.228569206	-0.558404126	8	0.330176875	0.816496581	0.816496581	0.816496581	0.816496581	0.816496581	0.816496581	17	81	0	P_106976	P_106976	P_106976	chr10	102737702	102737762	+	SFXN2	sideroflexin 2 [Source:HGNC Symbol;Acc:HGNC:16086]	ENSG00000156398	protein_coding	3'UTR;exon	multiple Ref	1	2801	10:102737702:102737762	ENSG00000156398	SFXN2	8.459534424	0.089753094	-2.956066985	-1.607670696	-3.890479478	1.348396289	-0.934412493	-2.282808782	9.961963646	0.014441012	0.139103289	0.003028847	0.207387056	0.372186967	0.045662359	-3.585799762	-4.97694787	-2.044080921	-6.236819281	-5.85215505	-4.785448011	5.726880474	0.015276241

REV1	-0.134361927	-0.036004097	-0.130572238	0.09835783	0.003789688	-0.094568142	8	0.046557788	0.816496581	0.816496581	0.816496581	0.816496581	0.816496581	0.816496581	17	83	0	P_235944	P_235944	P_235944	chr2	99442376	99442436	-	REV1	"REV1, polymerase (DNA directed) [Source:HGNC Symbol;Acc:HGNC:14060]"	ENSG00000135945	protein_coding	3'UTR;exon	multiple Ref	1	2803	2:99442376:99442436	ENSG00000135945	REV1	7.024206686	0.003947407	-2.619183279	-0.701845611	-2.545309014	1.917337669	0.073874266	-1.843463403	9.961963646	0.025708813	0.498844125	0.029176612	0.084290165	0.942572742	0.095171744	-4.157548124	-5.928885126	-4.327646413	-5.440262599	-6.293783356	-5.475823195	3.485217458	0.058274808

CELF4	-0.077494064	0.200110735	0.096030701	0.277604799	0.173524765	-0.104080034	8	0.139141775	0.816496581	0.816496581	0.816496581	0.816496581	0.816496581	0.816496581	17	84	0	P_123480	P_123480	P_123480	chr18	37259181	37259241	-	CELF4	"CUGBP, Elav-like family member 4 [Source:HGNC Symbol;Acc:HGNC:14015]"	ENSG00000101489	protein_coding	3'UTR;exon	multiple Ref	1	2804	18:37259181:37259241	ENSG00000101489	CELF4	4.90528195	0.017754168	-0.712301579	1.839356265	0.882684635	2.551657845	1.594986215	-0.95667163	9.961963646	0.492623091	0.095813361	0.398205962	0.028861178	0.14191594	0.361372918	-6.728366298	-4.656877201	-6.637565193	-4.414589963	-5.096515139	-6.593194181	2.439982761	0.124975338

GABPB2	-0.102136481	0.064226122	-0.097236646	0.166362604	0.004899836	-0.161462768	8	0.091976776	0.816496581	0.816496581	0.816496581	0.816496581	0.816496581	0.816496581	17	86	0	P_203969	P_203969	P_203969	chr1	151070680	151070740	+	GABPB2	"GA binding protein transcription factor, beta subunit 2 [Source:HGNC Symbol;Acc:HGNC:28441]"	ENSG00000143458	protein_coding	5'UTR;exon	multiple Ref	1	2806	1:151070680:151070740	ENSG00000143458	GABPB2	7.818899103	0.009000306	-1.318553869	0.829141565	-1.255298338	2.147695435	0.063255532	-2.084439903	9.961963646	0.216826855	0.426444743	0.238016877	0.057388681	0.950814252	0.063826163	-6.124497293	-5.83212718	-6.248670448	-5.079393985	-6.294557481	-5.104322307	2.160553435	0.156190129

OTUD4	-0.003182511	0.127332373	-0.024247877	0.130514884	-0.021065365	-0.151580249	8	0.084510335	0.816496581	0.816496581	0.816496581	0.816496581	0.816496581	0.816496581	17	87	0	A_23_P69637	A_23_P69637	A_23_P69637	chr4	145159246	145159306	-	OTUD4	OTU deubiquitinase 4 [Source:HGNC Symbol;Acc:HGNC:24949]	ENSG00000164164	protein_coding	3'UTR	3'UTR	1	2807	4:145159246:145159306	ENSG00000164164	OTUD4	7.368438145	0.007942097	-0.043736901	1.749914676	-0.333235879	1.793651577	-0.289498979	-2.083150555	9.961963646	0.965978379	0.110811939	0.745863546	0.103227544	0.778135161	0.063964288	-6.997316555	-4.78304751	-6.986815157	-5.626380298	-6.25230069	-5.106360456	1.802612053	0.21049335

MUC6	0.032671252	-0.022079859	0.176841066	-0.054751111	0.144169813	0.198920925	8	0.125634149	0.816496581	0.816496581	0.816496581	0.816496581	0.816496581	0.816496581	17	88	0	P_308869	P_308869	P_308869	chr11	1019284	1019344	-	MUC6	"mucin 6, oligomeric mucus/gel-forming [Source:HGNC Symbol;Acc:HGNC:7517]"	ENSG00000184956	protein_coding	exon	coding exon	1	2808	11:1019284:1019344	ENSG00000184956	MUC6	5.397400407	0.014882048	0.328004696	-0.221671863	1.775404858	-0.549676559	1.447400161	1.997076721	9.961963646	0.74969781	0.829047319	0.106332007	0.594648697	0.178511038	0.07385511	-6.939968218	-6.156249386	-5.553201557	-6.986851479	-5.28876844	-5.241275637	1.61570248	0.247412779

RIMS1	0.232390977	-0.189866731	0.050082255	-0.422257708	-0.182308723	0.239948985	8	0.058317159	0.816496581	0.816496581	0.816496581	0.816496581	0.816496581	0.816496581	17	90	0	P_257725	P_257725	P_257725	chr6	72250400	72250460	+	RIMS1	regulating synaptic membrane exocytosis 1 [Source:HGNC Symbol;Acc:HGNC:17282]	ENSG00000079841	protein_coding	exon	coding exon	1	2810	6:72250400:72250460	ENSG00000079841	RIMS1	6.863470645	0.004937785	4.050408336	-3.309241163	0.872897839	-7.359649499	-3.177510497	4.182139002	9.961963646	0.002340188	0.007929529	0.403268422	2.48E-05	0.009906785	0.001896962	-1.736658502	-2.363858538	-6.646269555	2.879256615	-2.642415256	-1.583024078	18.31168929	0.000222905

CDC23	-0.333919162	0.033325586	-0.205868599	0.367244748	0.128050563	-0.239194185	8	0.041469769	0.816496581	0.816496581	0.816496581	0.816496581	0.816496581	0.816496581	17	91	0	A_24_P913227	A_24_P913227	A_24_P913227	chr5	138206446	138206506	-	CDC23	cell division cycle 23 [Source:HGNC Symbol;Acc:HGNC:1724]	ENSG00000094880	protein_coding	"3'UTR,exon"	multiple Ref	1	2811	5:138206446:138206506	ENSG00000094880	CDC23	9.191506875	0.00358773	-6.827741531	0.681417875	-4.209454701	7.509159406	2.61828683	-4.890872576	9.961963646	4.67E-05	0.51113649	0.001816665	2.09E-05	0.025748332	0.000638695	2.315649586	-5.943070231	-1.517451655	3.058893069	-3.550696811	-0.45416852	25.32757151	5.60E-05

NEO1	0.271294971	-0.110223441	0.042870802	-0.381518412	-0.228424169	0.153094243	8	0.041223836	0.816496581	0.816496581	0.816496581	0.816496581	0.816496581	0.816496581	17	92	0	P_314774	P_314774	P_314774	chr15	73305097	73305157	+	NEO1	neogenin 1 [Source:HGNC Symbol;Acc:HGNC:7754]	ENSG00000067141	protein_coding	3'UTR	3'UTR	1	2812	15:73305097:73305157	ENSG00000067141	NEO1	7.40387923	0.003571399	5.559915767	-2.258917822	0.878593685	-7.818833589	-4.681322081	3.137511508	9.961963646	0.000244095	0.047551737	0.400316703	1.47E-05	0.000874704	0.010602306	0.602003901	-4.031491908	-6.641213773	3.422378509	-0.308876504	-3.342939892	21.61336892	0.000110926

GLG1	0.096927104	-0.124523191	0.087247793	-0.221450295	-0.009679311	0.211770984	8	0.094398615	0.816496581	0.816496581	0.816496581	0.816496581	0.816496581	0.816496581	17	93	0	P_119546	P_119546	P_119546	chr16	74483033	74483093	-	GLG1	golgi glycoprotein 1 [Source:HGNC Symbol;Acc:HGNC:4316]	ENSG00000090863	protein_coding	exon	coding exon	1	2813	16:74483033:74483093	ENSG00000090863	GLG1	9.938901035	0.009362782	1.226841363	-1.57613501	1.104326829	-2.802976374	-0.122514534	2.68046184	9.961963646	0.248087219	0.146189161	0.295410473	0.01876349	0.904927855	0.023146043	-6.233908445	-5.018698814	-6.419115229	-3.988638029	-6.288707947	-4.122847814	3.377573514	0.062737772

PLEKHG3	-0.09581075	-0.063881662	-0.0476604	0.031929088	0.04815035	0.016221262	8	0.091513317	0.816496581	0.816496581	0.816496581	0.816496581	0.816496581	0.816496581	17	95	0	P_115089	P_115089	P_115089	chr14	64729030	64729090	+	PLEKHG3	"pleckstrin homology domain containing, family G (with RhoGef domain) member 3 [Source:HGNC Symbol;Acc:HGNC:20364]"	ENSG00000126822	protein_coding	exon	coding exon	1	2815	14:64729030:64729090	ENSG00000126822	PLEKHG3	7.3467961	0.008932014	-1.241609883	-0.827841373	-0.617630317	0.413768511	0.623979567	0.210211056	9.961963646	0.24281864	0.42714694	0.55067572	0.687810611	0.546663091	0.837740043	-6.216675146	-5.833186653	-6.842715911	-7.05678734	-6.093670526	-7.047291851	0.535521203	0.668422564

NT5C2	-0.119054133	-0.04545398	-0.17485476	0.073600153	-0.055800627	-0.12940078	8	0.063635858	0.816496581	0.816496581	0.816496581	0.816496581	0.816496581	0.816496581	17	96	0	P_209683	P_209683	P_209683	chr10	103093138	103093198	-	NT5C2	"5'-nucleotidase, cytosolic II [Source:HGNC Symbol;Acc:HGNC:8022]"	ENSG00000076685	protein_coding	exon	coding exon	1	2816	10:103093138:103093198	ENSG00000076685	NT5C2	7.643445914	0.005458671	-1.973543201	-0.753484071	-2.898542142	1.22005913	-0.924998941	-2.145058071	9.961963646	0.076801221	0.468592926	0.015932645	0.250537535	0.376829484	0.057644198	-5.207105446	-5.891352911	-3.729176787	-6.391278403	-5.860697053	-5.007964197	3.301599835	0.066132957

ZNF273	-0.045187806	-0.190608768	-0.237152957	-0.145420962	-0.191965151	-0.046544189	8	0.164073856	0.816496581	0.816496581	0.816496581	0.816496581	0.816496581	0.816496581	17	97	0	A_23_P251965	A_23_P251965	A_23_P251965	chr7	64924152	64924212	+	ZNF273	zinc finger protein 273 [Source:HGNC Symbol;Acc:HGNC:13067]	ENSG00000198039	protein_coding	exon	non-coding exon	1	2817	7:64924152:64924212	ENSG00000198039	ZNF273	7.426425827	0.023825097	-0.358549851	-1.512415662	-1.881727955	-1.153865811	-1.523178104	-0.369312293	9.961963646	0.727413755	0.161484497	0.089382314	0.275486343	0.158806648	0.719623897	-6.92865969	-5.101572079	-5.394633098	-6.466494127	-5.191471621	-6.997376374	1.624121441	0.245597484

DYNC1LI1	-0.091775731	0.100043618	-0.031012755	0.191819349	0.060762976	-0.131056373	8	0.054688289	0.816496581	0.816496581	0.816496581	0.816496581	0.816496581	0.816496581	17	98	0	P_134307	P_134307	P_134307	chr3	32526283	32526343	-	DYNC1LI1	"dynein, cytoplasmic 1, light intermediate chain 1 [Source:HGNC Symbol;Acc:HGNC:18745]"	ENSG00000144635	protein_coding	3'UTR;exon	multiple Ref	1	2818	3:32526283:32526343	ENSG00000144635	DYNC1LI1	10.63109805	0.004608467	-1.655751883	1.804915155	-0.559509874	3.460667038	1.09624201	-2.364425029	9.961963646	0.128887231	0.101352912	0.588171655	0.006150244	0.298765752	0.039742874	-5.678244187	-4.705820798	-6.878810805	-2.862297857	-5.694386561	-4.65159108	4.180125488	0.037042997

KIAA1522	0.00878624	0.011836466	0.087793755	0.003050226	0.079007515	0.075957289	8	0.074796422	0.816496581	0.816496581	0.816496581	0.816496581	0.816496581	0.816496581	17	99	0	P_101188	P_101188	P_101188	chr1	32754109	32754169	+	KIAA1522	KIAA1522 [Source:HGNC Symbol;Acc:HGNC:29301]	ENSG00000162522	protein_coding	exon	coding exon	1	2819	1:32754109:32754169	ENSG00000162522	KIAA1522	10.77870207	0.006699376	0.131471465	0.177113021	1.313687499	0.045641556	1.182216034	1.136574478	9.961963646	0.898019628	0.862968813	0.218399611	0.964497912	0.26456942	0.282317395	-6.988936406	-6.165577633	-6.178483357	-7.148473977	-5.602616598	-6.408109841	0.744324677	0.549856242

MFSD6L	0.279742624	0.446664417	0.644709104	0.166921793	0.36496648	0.198044687	8	0.441691269	0.816496581	0.816496581	0.816496581	0.816496581	0.816496581	0.816496581	17	102	0	P_120543	P_120543	P_120543	chr17	8797835	8797895	-	MFSD6L	major facilitator superfamily domain containing 6-like [Source:HGNC Symbol;Acc:HGNC:26656]	ENSG00000185156	protein_coding	exon	coding exon	1	2822	17:8797835:8797895	ENSG00000185156	MFSD6L	4.621202996	0.158875537	0.85955913	1.372456126	1.980983766	0.512896996	1.121424636	0.60852764	9.961963646	0.410239685	0.200029364	0.075857998	0.619203755	0.288410914	0.556457602	-6.609547279	-5.27606796	-5.242736239	-7.007630464	-5.668054252	-6.872774332	1.406289472	0.29789944

GRAMD1C	0.269399145	0.078793352	0.293946986	-0.190605792	0.024547842	0.215153634	8	0.062586321	0.816496581	0.816496581	0.816496581	0.816496581	0.816496581	0.816496581	17	103	0	P_245629	P_245629	P_245629	chr3	113939936	113939996	+	GRAMD1C	GRAM domain containing 1C [Source:HGNC Symbol;Acc:HGNC:25252]	ENSG00000178075	protein_coding	exon	coding exon	1	2823	3:113939936:113939996	ENSG00000178075	GRAMD1C	6.479584303	0.005352287	4.509954449	1.31906295	4.920904707	-3.1908915	0.410950257	3.601841757	9.961963646	0.001136497	0.216662872	0.000610865	0.009684687	0.689809331	0.004862687	-0.991908778	-5.339685836	-0.388174917	-3.324088205	-6.207638102	-2.550990633	11.60314201	0.001368762

PSMA5	-0.15944237	0.080409323	-0.055845629	0.239851693	0.103596741	-0.136254952	8	0.036242249	0.816496581	0.816496581	0.816496581	0.816496581	0.816496581	0.816496581	17	104	0	A_32_P226338	A_32_P226338	A_32_P226338	chr1	109409929	109409989	+	PSMA5	"proteasome (prosome, macropain) subunit, alpha type, 5 [Source:HGNC Symbol;Acc:HGNC:9534]"	ENSG00000143106	protein_coding	exon	exon antisense	0	2824	1:109409929:109409989	ENSG00000143106	PSMA5	11.11055551	0.003261497	-3.419329124	1.72442205	-1.197640166	5.143751174	2.221688958	-2.922062216	9.961963646	0.006590639	0.115463706	0.258776234	0.000440692	0.050647769	0.015304758	-2.795242233	-4.818434581	-6.315696145	-0.131613212	-4.180509961	-3.711640482	9.338718432	0.003042602

IGFBP3	0.459247191	-0.328189527	0.236983409	-0.787436718	-0.222263782	0.565172936	8	0.170078813	0.816496581	0.816496581	0.816496581	0.816496581	0.816496581	0.816496581	17	106	0	P_144801	P_144801	P_144801	chr7	45912438	45912498	-	IGFBP3	insulin-like growth factor binding protein 3 [Source:HGNC Symbol;Acc:HGNC:5472]	ENSG00000146674	protein_coding	3'UTR	3'UTR	1	2826	7:45912438:45912498	ENSG00000146674	IGFBP3	6.420249166	0.025436484	3.52666007	-2.52023947	1.819847658	-6.04689954	-1.706812412	4.340087128	9.961963646	0.005509268	0.030455888	0.098915888	0.000126089	0.118779814	0.001479291	-2.61303382	-3.623033541	-5.487474542	1.177765127	-4.943733956	-1.325821663	13.40255711	0.000785843

GBP5	0.210999259	-0.146220803	0.189131316	-0.357220062	-0.021867943	0.335352119	8	0.189641145	0.816496581	0.816496581	0.816496581	0.816496581	0.816496581	0.816496581	17	107	0	P_202727	P_202727	P_202727	chr1	89266345	89266405	-	GBP5	guanylate binding protein 5 [Source:HGNC Symbol;Acc:HGNC:19895]	ENSG00000154451	protein_coding	exon	coding exon	1	2827	1:89266345:89266405	ENSG00000154451	GBP5	4.238865273	0.031087547	1.465660313	-1.015690904	1.313759418	-2.481351217	-0.151900895	2.329450322	9.961963646	0.173579147	0.333809593	0.218376299	0.032550923	0.882297348	0.042181772	-5.937757668	-5.665696668	-6.178395514	-4.532437739	-6.284427231	-4.709118393	2.752044753	0.098398512

MMRN2	-0.04345592	-0.335388645	-0.144128996	-0.291932726	-0.100673076	0.191259649	8	0.203557171	0.816496581	0.816496581	0.816496581	0.816496581	0.816496581	0.816496581	17	110	0	P_208901	P_208901	P_208901	chr10	86945610	86945670	-	MMRN2	multimerin 2 [Source:HGNC Symbol;Acc:HGNC:19888]	ENSG00000173269	protein_coding	"3'UTR,exon;exon"	multiple Ref	1	2830	10:86945610:86945670	ENSG00000173269	MMRN2	4.822771722	0.035481667	-0.282548213	-2.180680169	-0.937119511	-1.898131957	-0.654571298	1.243560658	9.961963646	0.783306738	0.054283423	0.370859552	0.087001977	0.527566575	0.242129554	-6.954970853	-4.151512271	-6.587651958	-5.469548668	-6.073714284	-6.28646159	1.881867349	0.196788631

PHF7	-0.24055425	-0.010944532	-0.109367583	0.229609718	0.131186667	-0.098423051	8	0.112910968	0.816496581	0.816496581	0.816496581	0.816496581	0.816496581	0.816496581	17	111	0	A_23_P80684	A_23_P80684	A_23_P80684	chr3	52420952	52421012	+	PHF7	PHD finger protein 7 [Source:HGNC Symbol;Acc:HGNC:18458]	ENSG00000010318	protein_coding	exon	coding exon	1	2831	3:52420952:52421012	ENSG00000010318	PHF7	7.32786197	0.012444735	-2.640984389	-0.120157254	-1.200719087	2.520827135	1.440265302	-1.080561833	9.961963646	0.024766133	0.906747368	0.257632057	0.030425273	0.180470579	0.305356848	-4.120823601	-6.174481984	-6.312175935	-4.466355242	-5.297767929	-6.468447482	3.004585016	0.081680314

RSU1	-0.12172672	0.114256743	-0.024759621	0.235983463	0.096967099	-0.139016363	8	0.057003781	0.816496581	0.816496581	0.816496581	0.816496581	0.816496581	0.816496581	17	112	0	A_23_P149813	A_23_P149813	A_23_P149813	chr10	16591526	16591586	-	RSU1	Ras suppressor protein 1 [Source:HGNC Symbol;Acc:HGNC:10464]	ENSG00000148484	protein_coding	3'UTR	3'UTR	1	2832	10:16591526:16591586	ENSG00000148484	RSU1	7.898916467	0.004816154	-2.14823285	2.0164027	-0.436957728	4.16463555	1.711275122	-2.453360428	9.961963646	0.057336747	0.071515759	0.671461579	0.001950374	0.117931368	0.034146305	-4.932682307	-4.398915262	-6.943844609	-1.680288884	-4.93752069	-4.504291071	5.86055758	0.014245841

RARS2	0.051111519	0.219066847	-0.009095396	0.167955327	-0.060206916	-0.228162243	8	0.081431246	0.816496581	0.816496581	0.816496581	0.816496581	0.816496581	0.816496581	17	113	0	P_258174	P_258174	P_258174	chr6	87545615	87545675	-	RARS2	"arginyl-tRNA synthetase 2, mitochondrial [Source:HGNC Symbol;Acc:HGNC:21406]"	ENSG00000146282	protein_coding	exon	coding exon	1	2833	6:87545615:87545675	ENSG00000146282	RARS2	7.661100993	0.007531777	0.721299611	3.091530697	-0.128356695	2.370231085	-0.849656307	-3.219887392	9.961963646	0.487308169	0.011463869	0.900420979	0.039351564	0.415468514	0.00922076	-6.721675177	-2.711060812	-7.038130682	-4.717101095	-5.926424089	-3.201919422	4.466982779	0.031059656

PIEZO2	0.061983143	-0.058075601	0.116367154	-0.120058744	0.054384011	0.174442755	8	0.114471686	0.816496581	0.816496581	0.816496581	0.816496581	0.816496581	0.816496581	17	114	0	P_230637	P_230637	P_230637	chr18	10697784	10697844	-	PIEZO2	piezo-type mechanosensitive ion channel component 2 [Source:HGNC Symbol;Acc:HGNC:26270]	ENSG00000154864	protein_coding	3'UTR;exon	multiple Ref	1	2834	18:10697784:10697844	ENSG00000154864	PIEZO2	4.109042106	0.012729723	0.672836807	-0.630419815	1.263183836	-1.303256622	0.590347029	1.893603651	9.961963646	0.516354319	0.542610115	0.235286068	0.221802657	0.568107379	0.087653103	-6.756813628	-5.97681549	-6.239325288	-6.292397407	-6.114591751	-5.400213527	1.34641569	0.314415457

ANKRD18EP	0.193454136	-0.044604418	-0.037803561	-0.238058553	-0.231257697	0.006800856	8	0.463071341	0.816496581	0.816496581	0.816496581	0.816496581	0.816496581	0.816496581	17	116	0	A_32_P63236	A_32_P63236	A_32_P63236	chr6	39111612	39111672	+	ANKRD18EP	"ankyrin repeat domain 18E, pseudogene [Source:HGNC Symbol;Acc:HGNC:43609]"	ENSG00000217165	processed_pseudogene	exon	non-coding exon	1	2836	6:39111612:39111672	ENSG00000217165	ANKRD18EP	3.455232409	0.174409735	0.567333315	-0.130809155	-0.110864622	-0.69814247	-0.678197937	0.019944533	9.961963646	0.58304554	0.89853015	0.913925481	0.501058936	0.513090675	0.984481436	-6.825520419	-6.173075033	-7.040412549	-6.889422224	-6.057704462	-7.071161218	0.216503704	0.882715419

DCBLD2	0.181813869	-0.057972397	0.267207856	-0.239786266	0.085393988	0.325180253	8	0.196535123	0.816496581	0.816496581	0.816496581	0.816496581	0.816496581	0.816496581	17	119	0	P_245164	P_245164	P_245164	chr3	98881586	98881646	-	DCBLD2	"discoidin, CUB and LCCL domain containing 2 [Source:HGNC Symbol;Acc:HGNC:24627]"	ENSG00000057019	protein_coding	exon	coding exon	1	2839	3:98881586:98881646	ENSG00000057019	DCBLD2	4.094750811	0.033225512	1.221621863	-0.389521153	1.795390866	-1.611143015	0.573769003	2.184912019	9.961963646	0.249971219	0.705087962	0.102936006	0.138342026	0.578846845	0.053896915	-6.239962825	-6.102729612	-5.523745694	-5.889040611	-6.124507604	-4.944067955	2.09439138	0.164865993

CD276	0.067142106	0.003977843	-0.057524745	-0.063164263	-0.124666851	-0.061502588	8	0.097067783	0.816496581	0.816496581	0.816496581	0.816496581	0.816496581	0.816496581	17	120	0	P_117041	P_117041	P_117041	chr15	73714388	73714448	-	CD276	CD276 molecule [Source:HGNC Symbol;Acc:HGNC:19137]	ENSG00000103855	protein_coding	3'ext;3'UTR;exon	multiple non-Ref	0	2840	15:73714388:73714448	ENSG00000103855	CD276	8.540639114	0.009773188	0.831806802	0.049280502	-0.712659717	-0.7825263	-1.544466519	-0.761940219	9.961963646	0.425007758	0.961669801	0.492410863	0.452105145	0.153623961	0.463752827	-6.633431583	-6.180807315	-6.7766495	-6.824689321	-5.163591731	-6.76318076	0.796748688	0.523379997

RPRD2	0.130326577	-0.106248745	0.025355669	-0.236575322	-0.104970908	0.131604414	8	0.069258104	0.816496581	0.816496581	0.816496581	0.816496581	0.816496581	0.816496581	17	121	0	P_203916	P_203916	P_203916	chr1	150364859	150364919	+	RPRD2	regulation of nuclear pre-mRNA domain containing 2 [Source:HGNC Symbol;Acc:HGNC:29039]	ENSG00000163125	protein_coding	exon	coding exon	1	2841	1:150364859:150364919	ENSG00000163125	RPRD2	7.532575093	0.006058684	2.050640282	-1.671784533	0.398962041	-3.722424815	-1.651678241	2.070746574	9.961963646	0.067542496	0.125633201	0.698339277	0.003985419	0.129725989	0.065307712	-5.087131724	-4.890643309	-6.960888071	-2.417796955	-5.019774698	-5.125943111	4.671939782	0.027483995

WDFY3	0.296576781	-0.122146763	0.266977829	-0.418723544	-0.029598952	0.389124592	8	0.065714575	0.816496581	0.816496581	0.816496581	0.816496581	0.816496581	0.816496581	17	122	0	P_138109	P_138109	P_138109	chr4	84684041	84684101	-	WDFY3	WD repeat and FYVE domain containing 3 [Source:HGNC Symbol;Acc:HGNC:20751]	ENSG00000163625	protein_coding	exon	coding exon	1	2842	4:84684041:84684101	ENSG00000163625	WDFY3	6.898015317	0.005674599	4.821866577	-1.985912013	4.340634707	-6.80777859	-0.481231871	6.326546719	9.961963646	0.000707887	0.07523927	0.001478025	4.78E-05	0.640746972	8.75E-05	-0.50205809	-4.444042284	-1.304269261	2.191826514	-6.174965567	1.615973314	22.10633005	0.000100741

SLC6A13	-0.099274827	-0.222064537	0.159550008	-0.12278971	0.258824835	0.381614545	8	0.425817461	0.816496581	0.816496581	0.816496581	0.816496581	0.816496581	0.816496581	17	123	0	A_23_P64980	A_23_P64980	A_23_P64980	chr12	220792	220852	-	SLC6A13	"solute carrier family 6 (neurotransmitter transporter), member 13 [Source:HGNC Symbol;Acc:HGNC:11046]"	ENSG00000010379	protein_coding	3'UTR	3'UTR	1	2843	12:220792:220852	ENSG00000010379	SLC6A13	3.929879115	0.14781694	-0.31624427	-0.707396219	0.508253474	-0.391151949	0.824497745	1.215649693	9.961963646	0.758343792	0.495535654	0.622340057	0.70392027	0.428956315	0.252141044	-6.944059749	-5.924965446	-6.90785991	-7.066582428	-5.947305434	-6.318982829	0.528223666	0.672942872

BEST1	0.224766879	0.083584704	0.27984967	-0.141182175	0.055082791	0.196264966	8	0.171561959	0.816496581	0.816496581	0.816496581	0.816496581	0.816496581	0.816496581	17	125	0	P_108799	P_108799	P_108799	chr11	61955134	61955194	+	BEST1	bestrophin 1 [Source:HGNC Symbol;Acc:HGNC:12703]	ENSG00000167995	protein_coding	5'UTR;exon	multiple Ref	1	2845	11:61955134:61955194	ENSG00000167995	BEST1	4.851658016	0.025843394	1.712391924	0.636792098	2.132041506	-1.075599826	0.419649582	1.495249408	9.961963646	0.117719903	0.538617013	0.058921339	0.307465683	0.683647857	0.165837185	-5.59740284	-5.972730357	-5.005359252	-6.551260958	-6.20386246	-5.970295616	1.908697135	0.192385657

AMMECR1L	-0.005785755	0.007560269	0.043236405	0.013346024	0.04902216	0.035676137	8	0.0714487	0.816496581	0.816496581	0.816496581	0.816496581	0.816496581	0.816496581	17	126	0	P_236454	P_236454	P_236454	chr2	127869453	127869513	-	AMMECR1L	AMMECR1-like [Source:HGNC Symbol;Acc:HGNC:28658]	ENSG00000144233	protein_coding	exon	coding exon	1	2846	2:127869453:127869513	ENSG00000144233	AMMECR1L	6.133167069	0.00630621	-0.089232165	0.11660002	0.666823624	0.205832185	0.75605579	0.550223605	9.961963646	0.930665866	0.909494158	0.520029676	0.841067433	0.467117521	0.594287378	-6.994017059	-6.174925318	-6.809592998	-7.126493455	-6.001330154	-6.908468078	0.230490825	0.873071623

KDM4A-AS1	-0.169009836	0.023488336	-0.176607215	0.192498172	-0.007597379	-0.200095551	8	0.136908959	0.816496581	0.816496581	0.816496581	0.816496581	0.816496581	0.816496581	17	127	0	P_101581	P_101581	P_101581	chr1	43702279	43702339	-	KDM4A-AS1	KDM4A antisense RNA 1 [Source:HGNC Symbol;Acc:HGNC:40528]	ENSG00000236200	antisense	exon	non-coding exon	1	2847	1:43702279:43702339	ENSG00000236200	KDM4A-AS1	6.576047098	0.017259189	-1.575605258	0.218971547	-1.646432323	1.794576805	-0.070827066	-1.86540387	9.961963646	0.146310873	0.831093308	0.130813322	0.103072364	0.94493708	0.091811013	-5.789975615	-6.156873487	-5.739029743	-5.625010786	-6.294017756	-5.442856764	1.991078974	0.179564423

NOC3L	-0.176134513	0.113294716	-0.108965453	0.289429229	0.06716906	-0.222260169	8	0.040897776	0.816496581	0.816496581	0.816496581	0.816496581	0.816496581	0.816496581	17	128	0	P_209245	P_209245	P_209245	chr10	94340432	94340492	-	NOC3L	nucleolar complex associated 3 homolog (S. cerevisiae) [Source:HGNC Symbol;Acc:HGNC:24034]	ENSG00000173145	protein_coding	exon	coding exon	1	2848	10:94340432:94340492	ENSG00000173145	NOC3L	8.785897093	0.003549896	-3.620614658	2.32888208	-2.239889891	5.949496738	1.380724766	-4.568771971	9.961963646	0.004713884	0.042222579	0.049110531	0.000143526	0.19755208	0.001038335	-2.454121357	-3.923164701	-4.83197438	1.042158738	-5.371737886	-0.958983792	13.62103977	0.000737587

CDK8	0.207818615	-0.115531118	0.318282063	-0.323349733	0.110463448	0.433813181	8	0.064580279	0.816496581	0.816496581	0.816496581	0.816496581	0.816496581	0.816496581	17	129	0	P_218423	P_218423	P_218423	chr13	26337582	26337642	+	CDK8	cyclin-dependent kinase 8 [Source:HGNC Symbol;Acc:HGNC:1779]	ENSG00000132964	protein_coding	exon	coding exon	1	2849	13:26337582:26337642	ENSG00000132964	CDK8	8.642029667	0.005555913	3.414698395	-1.898308879	5.229739639	-5.313007274	1.815041245	7.128048519	9.961963646	0.006641964	0.086976629	0.000389263	0.000345536	0.099694357	3.25E-05	-2.803117415	-4.57211746	0.080273832	0.122682895	-4.790731814	2.646054455	20.82423585	0.000129931

CEP68	0.097763862	-0.034825537	0.063900009	-0.1325894	-0.033863854	0.098725546	8	0.041245636	0.816496581	0.816496581	0.816496581	0.816496581	0.816496581	0.816496581	17	131	0	A_32_P164061	A_32_P164061	A_32_P164061	chr2	65088613	65088673	+	CEP68;RAB1A	"centrosomal protein 68kDa [Source:HGNC Symbol;Acc:HGNC:29076];RAB1A, member RAS oncogene family [Source:HGNC Symbol;Acc:HGNC:9758]"	ENSG00000011523	protein_coding	3'ext;exon;intron	multiple non-Ref	0	2851	2:65088613:65088673	ENSG00000011523	CEP68	7.644150136	0.003572842	2.003166518	-0.71356991	1.309301359	-2.716736428	-0.693865159	2.022871269	9.961963646	0.073110275	0.491871748	0.219825265	0.021750979	0.503624639	0.070748522	-5.1612343	-5.920573129	-6.183834318	-4.135477029	-6.0468038	-5.201087987	3.031707002	0.080092845

EMC8	-0.074594759	-0.050272395	0.004451436	0.024322364	0.079046196	0.054723831	8	0.083720838	0.816496581	0.816496581	0.816496581	0.816496581	0.816496581	0.816496581	17	132	0	P_227189	P_227189	P_227189	chr16	85779450	85779510	+	EMC8;RP11-568J23.5	ER membrane protein complex subunit 8 [Source:HGNC Symbol;Acc:HGNC:7864];	ENSG00000131148	protein_coding;antisense	3'UTR;exon;intron;promoter	multiple non-Ref	0	2852	16:85779450:85779510	ENSG00000131148	EMC8	8.638961696	0.007835437	-1.032101349	-0.695574425	0.061590566	0.336526923	1.093691915	0.757164991	9.961963646	0.326430495	0.502598391	0.952107059	0.74345505	0.299830146	0.466482089	-6.446334927	-5.933280146	-7.045049386	-7.088031725	-5.697027242	-6.766925213	0.572593595	0.645841735

SPTLC2	-0.132845012	-0.036351191	-0.180654226	0.09649382	-0.047809214	-0.144303034	8	0.065336839	0.816496581	0.816496581	0.816496581	0.816496581	0.816496581	0.816496581	17	133	0	A_24_P456407	A_24_P456407	A_24_P456407	chr14	77548913	77548973	+	SPTLC2;RN7SL587P	"serine palmitoyltransferase, long chain base subunit 2 [Source:HGNC Symbol;Acc:HGNC:11278];RNA, 7SL, cytoplasmic 587, pseudogene [Source:HGNC Symbol;Acc:HGNC:46603]"	ENSG00000100596	protein_coding;misc_RNA	intron;promoter	multiple non-Ref	0	2853	14:77548913:77548973	ENSG00000100596	SPTLC2	7.912657619	0.005634845	-2.167453932	-0.593093647	-2.947492771	1.574360284	-0.78003884	-2.354399124	9.961963646	0.055508678	0.56633877	0.01465403	0.146597261	0.453502339	0.040427565	-4.901952006	-5.999982034	-3.64558316	-5.940032712	-5.982867378	-4.668106399	3.727932732	0.049523577

COL3A1	0.124757288	-0.098931164	0.086323634	-0.223688451	-0.038433653	0.185254798	8	0.169470922	0.816496581	0.816496581	0.816496581	0.816496581	0.816496581	0.816496581	17	134	0	P_129449	P_129449	P_129449	chr2	189010830	189010890	+	COL3A1	"collagen, type III, alpha 1 [Source:HGNC Symbol;Acc:HGNC:2201]"	ENSG00000168542	protein_coding	exon	coding exon	1	2854	2:189010830:189010890	ENSG00000168542	COL3A1	4.233094761	0.025270726	0.961175504	-0.762201657	0.665068664	-1.723377161	-0.296106841	1.42727032	9.961963646	0.359212767	0.463603701	0.521105273	0.115658103	0.773229228	0.184086983	-6.516423037	-5.884782877	-6.810814679	-5.729324654	-6.250260649	-6.059530785	1.173655785	0.368119228

CCDC194	-0.271131127	-0.44888394	-0.970224707	-0.177752812	-0.69909358	-0.521340768	8	0.685961498	0.816496581	0.816496581	0.816496581	0.816496581	0.816496581	0.816496581	17	136	0	A_32_P75559	A_32_P75559	A_32_P75559	chr19	17391639	17391699	-	CTD-2521M24.5	NA	ENSG00000269720	lincRNA	exon	non-coding exon	1	2856	19:17391639:17391699	ENSG00000269720	CCDC194	3.050993516	0.380078512	-0.538627286	-0.891749836	-1.927441922	-0.35312255	-1.388814636	-1.035692086	9.961963646	0.601971389	0.393556407	0.082895336	0.7313545	0.195153505	0.324832333	-6.842324421	-5.779426306	-5.325111168	-7.08184706	-5.361808092	-6.515042131	1.321088202	0.321710391

OSBP2	-0.126848421	-0.129026659	0.002235119	-0.002178238	0.12908354	0.131261778	8	0.082891632	0.816496581	0.816496581	0.816496581	0.816496581	0.816496581	0.816496581	17	137	0	P_133065	P_133065	P_133065	chr22	30906383	30906443	+	OSBP2	oxysterol binding protein 2 [Source:HGNC Symbol;Acc:HGNC:8504]	ENSG00000184792	protein_coding	3'UTR	3'UTR	1	2857	22:30906383:30906443	ENSG00000184792	OSBP2	6.190039176	0.00772449	-1.767648028	-1.798002035	0.031146657	-0.030354007	1.798794685	1.829148692	9.961963646	0.107677444	0.102499742	0.975767763	0.976384237	0.102367646	0.097425332	-5.517130736	-4.715591697	-7.046590839	-7.149109223	-4.813997936	-5.497229322	2.156788268	0.156669307

GLRA1	-0.202857694	-0.145974453	-0.035543863	0.05688324	0.167313831	0.11043059	8	0.204422097	0.816496581	0.816496581	0.816496581	0.816496581	0.816496581	0.816496581	17	141	0	A_23_P156261	A_23_P156261	A_23_P156261	chr5	151855063	151855123	-	GLRA1	"glycine receptor, alpha 1 [Source:HGNC Symbol;Acc:HGNC:4326]"	ENSG00000145888	protein_coding	3'UTR;exon	multiple Ref	1	2861	5:151855063:151855123	ENSG00000145888	GLRA1	3.629254833	0.035765043	-1.313734906	-0.945351055	-0.230187047	0.368383851	1.083547859	0.715164007	9.961963646	0.218384244	0.36684402	0.822604344	0.720294593	0.304093153	0.490928434	-6.130385623	-5.731740559	-7.018261614	-7.075903598	-5.707484201	-6.798951647	0.74896938	0.547457917

ELOVL2	-0.43230326	0.298108412	-0.508348738	0.730411672	-0.076045478	-0.80645715	8	0.149813761	0.816496581	0.816496581	0.816496581	0.816496581	0.816496581	0.816496581	17	142	0	P_141639	P_141639	P_141639	chr6	10980783	10980843	-	ELOVL2	ELOVL fatty acid elongase 2 [Source:HGNC Symbol;Acc:HGNC:14416]	ENSG00000197977	protein_coding	3'UTR	3'UTR	1	2862	6:10980783:10980843	ENSG00000197977	ELOVL2	3.930417402	0.020230571	-3.722460282	2.566940448	-4.377269756	6.28940073	-0.654809474	-6.944210204	9.961963646	0.003985186	0.028115683	0.001395868	9.18E-05	0.527419452	4.05E-05	-2.28259715	-3.549113191	-1.245113372	1.509591656	-6.073555478	2.417333043	21.30229611	0.00011799

PLD5	-0.241244075	0.015296312	0.013949777	0.256540388	0.255193853	-0.001346535	8	0.152880666	0.816496581	0.816496581	0.816496581	0.816496581	0.816496581	0.816496581	17	143	0	P_207057	P_207057	P_207057	chr1	242107675	242107735	-	PLD5	"phospholipase D family, member 5 [Source:HGNC Symbol;Acc:HGNC:26879]"	ENSG00000180287	protein_coding	3'UTR;exon	multiple Ref	1	2863	1:242107675:242107735	ENSG00000180287	PLD5	3.280080931	0.020976074	-2.040047029	0.129351142	0.117964353	2.16939817	2.158011382	-0.011386789	9.961963646	0.068748929	0.899654188	0.90844051	0.055326915	0.056399631	0.991139662	-5.103728132	-6.173274645	-7.039526499	-5.044582784	-4.279062345	-7.071307692	2.259311732	0.144213095

TRIM26	-0.091356426	0.031586914	-0.01521735	0.12294334	0.076139076	-0.046804264	8	0.079786515	0.816496581	0.816496581	0.816496581	0.816496581	0.816496581	0.816496581	17	145	0	P_142005	P_142005	P_142005	chr6	30185946	30186006	-	TRIM26	tripartite motif containing 26 [Source:HGNC Symbol;Acc:HGNC:12962]	ENSG00000234127	protein_coding	exon	coding exon	1	2865	6:30185946:30186006	ENSG00000234127	TRIM26	8.505880688	0.007318839	-1.307867135	0.452201221	-0.217853005	1.760068356	1.09001413	-0.670054226	9.961963646	0.22029311	0.660810952	0.831941199	0.109007165	0.301370374	0.518053141	-6.137535054	-6.075425335	-7.021264248	-5.675844091	-5.700827246	-6.831507575	1.116234401	0.388162144

HERC6	0.136399473	0.098983511	0.032692448	-0.037415962	-0.103707025	-0.066291063	8	0.066978051	0.816496581	0.816496581	0.816496581	0.816496581	0.816496581	0.816496581	17	147	0	P_249971	P_249971	P_249971	chr4	88440190	88440250	+	HERC6	HECT and RLD domain containing E3 ubiquitin protein ligase family member 6 [Source:HGNC Symbol;Acc:HGNC:26072]	ENSG00000138642	protein_coding	3'UTR;exon	multiple Ref	1	2867	4:88440190:88440250	ENSG00000138642	HERC6	6.351585302	0.005809234	2.191789679	1.590556281	0.525331721	-0.601233399	-1.666457958	-1.06522456	9.961963646	0.053274402	0.142910055	0.610844679	0.561115402	0.126706014	0.311911105	-4.86290722	-4.999664978	-6.898473692	-6.955454127	-4.999523497	-6.484551096	1.980508631	0.181152651

BBS4	-0.329355776	0.005915982	-0.228844382	0.335271758	0.100511393	-0.234760364	8	0.084412655	0.816496581	0.816496581	0.816496581	0.816496581	0.816496581	0.816496581	17	151	0	P_224450	P_224450	P_224450	chr15	72712247	72712307	+	BBS4	Bardet-Biedl syndrome 4 [Source:HGNC Symbol;Acc:HGNC:969]	ENSG00000140463	protein_coding	3'UTR;5'UTR;exon	multiple Ref	1	2871	15:72712247:72712307	ENSG00000140463	BBS4	7.421895586	0.007928846	-4.530080496	0.081370588	-3.147609816	4.611451083	1.382470679	-3.228980404	9.961963646	0.001101845	0.936758847	0.010422127	0.000972756	0.197032333	0.009080019	-0.959902726	-6.178598138	-3.30329194	-0.957706699	-5.369598154	-3.186358902	10.67311979	0.001871534

CD52	0.029682119	-0.194224507	0.161250041	-0.223906626	0.131567922	0.355474549	8	0.198894474	0.816496581	0.816496581	0.816496581	0.816496581	0.816496581	0.816496581	17	153	0	P_100911	P_100911	P_100911	chr1	26318730	26318790	+	CD52	CD52 molecule [Source:HGNC Symbol;Acc:HGNC:1804]	ENSG00000169442	protein_coding	exon	non-coding exon	1	2873	1:26318730:26318790	ENSG00000169442	CD52	4.482791029	0.033974728	0.197225262	-1.290540585	1.071439055	-1.487765846	0.874213794	2.36197964	9.961963646	0.847617129	0.226010464	0.30924257	0.167766419	0.402585128	0.039908822	-6.977175759	-5.37295879	-6.454011785	-6.057239173	-5.905522077	-4.655621061	1.901503207	0.193554917

MREG	0.117646329	0.020034978	0.112962744	-0.097611351	-0.004683585	0.092927766	8	0.074328021	0.816496581	0.816496581	0.816496581	0.816496581	0.816496581	0.816496581	17	154	0	A_23_P108948	A_23_P108948	A_23_P108948	chr2	215943376	215943436	-	MREG	melanoregulin [Source:HGNC Symbol;Acc:HGNC:25478]	ENSG00000118242	protein_coding	3'UTR	3'UTR	1	2874	2:215943376:215943436	ENSG00000118242	MREG	7.192709236	0.006643283	1.767797977	0.30105311	1.697420671	-1.466744867	-0.070377306	1.396367561	9.961963646	0.107651288	0.76956376	0.120583428	0.173289952	0.945286099	0.192936443	-5.516911085	-6.134533359	-5.666474997	-6.085062992	-6.294051518	-6.099195845	1.700519239	0.229805504

RYR3	0.002145521	0.076424727	0.114258157	0.074279207	0.112112636	0.037833429	8	0.056015958	0.816496581	0.816496581	0.816496581	0.816496581	0.816496581	0.816496581	17	156	0	P_222614	P_222614	P_222614	chr15	33649161	33649221	+	RYR3	ryanodine receptor 3 [Source:HGNC Symbol;Acc:HGNC:10485]	ENSG00000198838	protein_coding	exon	coding exon	1	2876	15:33649161:33649221	ENSG00000198838	RYR3	3.848512479	0.004726498	0.038221574	1.361475305	2.035462396	1.323253731	1.997240823	0.673987091	9.961963646	0.970266122	0.203359673	0.069277369	0.215316862	0.073834945	0.515653024	-6.997563181	-5.289290606	-5.157934166	-6.267939049	-4.523375588	-6.828745971	2.032068833	0.173556981

HSD17B14	0.579576998	0.285481192	0.459802365	-0.294095806	-0.119774633	0.174321173	8	0.168127944	0.816496581	0.816496581	0.816496581	0.816496581	0.816496581	0.816496581	17	157	0	A_23_P67360	A_23_P67360	A_23_P67360	chr19	48837519	48838044	-	HSD17B14;PLEKHA4	"hydroxysteroid (17-beta) dehydrogenase 14 [Source:HGNC Symbol;Acc:HGNC:23238];pleckstrin homology domain containing, family A (phosphoinositide binding specific) member 4 [Source:HGNC Symbol;Acc:HGNC:14339]"	ENSG00000087076	protein_coding	"3'UTR,intron;exon,intron;promoter"	multiple non-Ref	0	2877	19:48837519:48838044	ENSG00000087076	HSD17B14	7.360881217	0.024906631	4.497791078	2.215468801	3.56828339	-2.282322277	-0.929507688	1.352814589	9.961963646	0.001157998	0.0511837	0.005140971	0.045700097	0.374600755	0.20601912	-1.011277974	-4.098300669	-2.586049566	-4.861553303	-5.856614753	-6.154103487	7.62902663	0.00612853

KCNK6	0.137913956	0.115112426	0.16394026	-0.02280153	0.026026304	0.048827834	8	0.081641097	0.816496581	0.816496581	0.816496581	0.816496581	0.816496581	0.816496581	18	13	0	P_125666	P_125666	P_125666	chr19	38327610	38327670	+	KCNK6	"potassium channel, subfamily K, member 6 [Source:HGNC Symbol;Acc:HGNC:6281]"	ENSG00000099337	protein_coding	3'UTR	3'UTR	1	2903	19:38327610:38327670	ENSG00000099337	KCNK6	6.515541032	0.007559258	1.942738102	1.621542175	2.309360115	-0.321195927	0.366622013	0.687817939	9.961963646	0.080824808	0.136084269	0.043648118	0.75469911	0.72156801	0.507265658	-5.254492299	-4.958434385	-4.718821074	-7.093486468	-6.225687374	-6.818917244	2.074574423	0.167573267

SNCA	-0.056118223	-0.088460437	-0.164780263	-0.032342214	-0.10866204	-0.076319826	8	0.220212997	0.816496581	0.816496581	0.816496581	0.816496581	0.816496581	0.816496581	18	14	0	A_32_P109653	A_32_P109653	A_32_P109653	chr4	89724175	89724235	-	SNCA	"synuclein, alpha (non A4 component of amyloid precursor) [Source:HGNC Symbol;Acc:HGNC:11138]"	ENSG00000145335	protein_coding	3'UTR	3'UTR	1	2904	4:89724175:89724235	ENSG00000145335	SNCA	4.307958363	0.041149821	-0.338817291	-0.534085439	-0.994871173	-0.195268148	-0.656053882	-0.460785734	9.961963646	0.741780592	0.604995002	0.343349384	0.849108174	0.526651162	0.654847541	-6.936077521	-6.033914118	-6.531980597	-7.128801278	-6.072724899	-6.95661289	0.34511215	0.793494936

MIER3	0.398945268	-0.03078687	0.231458044	-0.429732137	-0.167487224	0.262244914	8	0.093810707	0.816496581	0.816496581	0.816496581	0.816496581	0.816496581	0.816496581	18	16	0	A_23_P18993	A_23_P18993	A_23_P18993	chr5	56923265	56923325	-	MIER3	"mesoderm induction early response 1, family member 3 [Source:HGNC Symbol;Acc:HGNC:26678]"	ENSG00000155545	protein_coding	3'UTR;exon	multiple Ref	1	2906	5:56923265:56923325	ENSG00000155545	MIER3	7.783469981	0.009273924	5.07372796	-0.391542938	2.943649778	-5.465270897	-2.130078181	3.335192716	9.961963646	0.000487934	0.703640432	0.014750537	0.000278594	0.05911631	0.007590518	-0.116447474	-6.10191021	-3.652149002	0.347935553	-4.321996227	-3.004754801	13.34850413	0.00079836

RABGAP1L	0.060043223	-0.046300355	0.075139594	-0.106343578	0.015096371	0.121439949	8	0.076179276	0.816496581	0.816496581	0.816496581	0.816496581	0.816496581	0.816496581	18	17	0	P_308165	P_308165	P_308165	chr1	174982117	174982177	+	RABGAP1L	RAB GTPase activating protein 1-like [Source:HGNC Symbol;Acc:HGNC:24663]	ENSG00000152061	protein_coding	intron	intron sense	0	2907	1:174982117:174982177	ENSG00000152061	RABGAP1L	5.684026546	0.006867036	0.887411326	-0.684298029	1.110528771	-1.571709355	0.223117445	1.7948268	9.961963646	0.395776877	0.509392334	0.292856431	0.147208718	0.827952574	0.103030471	-6.584900295	-5.941093209	-6.412442013	-5.943680643	-6.27027533	-5.548216163	1.371748623	0.30730457

ERCC2	-0.152106516	0.009448983	0.043367511	0.161555499	0.195474027	0.033918528	8	0.11331731	0.816496581	0.816496581	0.816496581	0.816496581	0.816496581	0.816496581	18	18	0	A_23_P130488	A_23_P130488	A_23_P130488	chr19	45353256	45353316	-	ERCC2	excision repair cross-complementation group 2 [Source:HGNC Symbol;Acc:HGNC:3434]	ENSG00000104884	protein_coding	3'UTR;exon	multiple Ref	1	2908	19:45353256:45353316	ENSG00000104884	ERCC2	7.913655279	0.012518557	-1.665007973	0.103431678	0.474715043	1.768439651	2.139723016	0.371283365	9.961963646	0.126999474	0.919672961	0.645225369	0.107539426	0.058164415	0.718200844	-5.665136423	-6.176450835	-6.925433796	-5.663559419	-4.307193361	-6.996589802	1.808658125	0.209409763

TMEM67	0.107176079	0.097574506	0.082502378	-0.009601574	-0.024673701	-0.015072128	8	0.051278603	0.816496581	0.816496581	0.816496581	0.816496581	0.816496581	0.816496581	18	19	0	P_265389	P_265389	P_265389	chr8	93797173	93797233	+	TMEM67	transmembrane protein 67 [Source:HGNC Symbol;Acc:HGNC:28396]	ENSG00000164953	protein_coding	exon	coding exon	1	2909	8:93797173:93797233	ENSG00000164953	TMEM67	5.786139554	0.004318312	1.997498624	1.818548896	1.537641492	-0.178949728	-0.459857133	-0.280907404	9.961963646	0.073803278	0.099125688	0.155269135	0.861564261	0.655491396	0.784529177	-5.170033326	-4.686498504	-5.889522727	-7.13212901	-6.185424271	-7.028441315	1.663977126	0.237208632

ANKRD17	-0.136184248	-0.130298371	-0.184648064	0.005885878	-0.048463816	-0.054349693	8	0.033454534	0.816496581	0.816496581	0.816496581	0.816496581	0.816496581	0.816496581	18	20	0	P_249337	P_249337	P_249337	chr4	73094078	73094138	-	ANKRD17	ankyrin repeat domain 17 [Source:HGNC Symbol;Acc:HGNC:23575]	ENSG00000132466	protein_coding	exon	coding exon	1	2910	4:73094078:73094138	ENSG00000132466	ANKRD17	9.22900246	0.003105467	-2.993015933	-2.863657907	-4.058138912	0.129358027	-1.065122978	-1.194481005	9.961963646	0.013558418	0.016912245	0.002311382	0.89964888	0.31195487	0.259954455	-3.522731082	-3.075884981	-1.765886379	-7.140469796	-5.726282132	-6.343283563	6.03419629	0.013028767

CLTCL1	-0.092149392	-0.080260059	-0.063809919	0.011889333	0.028339473	0.01645014	8	0.094214369	0.816496581	0.816496581	0.816496581	0.816496581	0.816496581	0.816496581	18	21	0	A_14_P108754	A_14_P108754	A_14_P108754	chr22	19177443	19177503	+	CLTCL1;SLC25A1	"clathrin, heavy chain-like 1 [Source:HGNC Symbol;Acc:HGNC:2093];solute carrier family 25 (mitochondrial carrier; citrate transporter), member 1 [Source:HGNC Symbol;Acc:HGNC:10979]"	ENSG00000070371	protein_coding	3'ext;intron	multiple non-Ref	0	2911	22:19177443:19177503	ENSG00000070371	CLTCL1	5.454313419	0.009334875	-1.168110299	-1.017397939	-0.808871573	0.15071236	0.359238726	0.208526365	9.961963646	0.269957435	0.333036255	0.437480432	0.883210635	0.726914158	0.839019802	-6.300923103	-5.664046202	-6.701142366	-7.137205644	-6.228499443	-7.047675536	0.54151723	0.664726832

BGN	-0.275906895	-0.332955774	0.058853696	-0.057048879	0.334760591	0.39180947	8	0.424256855	0.816496581	0.816496581	0.816496581	0.816496581	0.816496581	0.816496581	18	22	0	P_151888	P_151888	P_151888	chrX	153509486	153509546	+	BGN	biglycan [Source:HGNC Symbol;Acc:HGNC:1044]	ENSG00000182492	protein_coding	3'UTR;exon	multiple Ref	1	2912	X:153509486:153509546	ENSG00000182492	BGN	6.863273044	0.146751583	-0.882097896	-1.064488032	0.18816029	-0.182390136	1.070258186	1.252648322	9.961963646	0.398508216	0.312228534	0.85452857	0.858934707	0.309748295	0.238940385	-6.589654138	-5.617647546	-7.027820485	-7.131451403	-5.721068401	-6.275756517	0.782413721	0.530491046

DDX58	0.292704861	0.119412457	0.443586397	-0.173292403	0.150881536	0.324173939	8	0.068961161	0.816496581	0.816496581	0.816496581	0.816496581	0.816496581	0.816496581	18	23	0	P_267119	P_267119	P_267119	chr9	32459373	32459433	-	DDX58	DEAD (Asp-Glu-Ala-Asp) box polypeptide 58 [Source:HGNC Symbol;Acc:HGNC:19102]	ENSG00000107201	protein_coding	exon	coding exon	1	2913	9:32459373:32459433	ENSG00000107201	DDX58	6.694675665	0.006025724	4.618181122	1.884042361	6.998730113	-2.734138761	2.380548991	5.114687752	9.961963646	0.000962822	0.089042846	3.79E-05	0.021111926	0.038665453	0.000459678	-0.820436091	-4.59273942	2.500738557	-4.105893631	-3.93107261	-0.111937133	18.86033246	0.000197119

TYSND1	-0.146990175	0.10262449	-0.005511938	0.249614665	0.141478237	-0.108136428	8	0.133065288	0.816496581	0.816496581	0.816496581	0.816496581	0.816496581	0.816496581	18	24	0	P_106169	P_106169	P_106169	chr10	70139207	70139267	+	TYSND1	trypsin domain containing 1 [Source:HGNC Symbol;Acc:HGNC:28531]	ENSG00000156521	protein_coding	3'UTR;exon	multiple non-Ref	0	2914	10:70139207:70139267	ENSG00000156521	TYSND1	7.248449428	0.016425865	-1.404655456	0.980691734	-0.052672732	2.385347189	1.351982724	-1.033364466	9.961963646	0.190528275	0.349960887	0.959033934	0.03835039	0.206276092	0.325867636	-6.016800641	-5.699041511	-7.045605678	-4.692108206	-5.406702233	-6.517416079	1.920528043	0.190480514

DENND2A	0.041866368	0.102221289	0.161875739	0.060354921	0.120009371	0.05965445	8	0.108338817	0.816496581	0.816496581	0.816496581	0.816496581	0.816496581	0.816496581	18	27	0	A_24_P334726	A_24_P334726	A_24_P334726	chr7	140521991	140522051	-	DENND2A	DENN/MADD domain containing 2A [Source:HGNC Symbol;Acc:HGNC:22212]	ENSG00000146966	protein_coding	3'UTR;exon	multiple Ref	1	2917	7:140521991:140522051	ENSG00000146966	DENND2A	4.481484918	0.011632375	0.475419196	1.160787655	1.838201813	0.685368459	1.362782618	0.677414159	9.961963646	0.644740749	0.272788604	0.095994428	0.508745026	0.202960754	0.513567032	-6.876410757	-5.517703278	-5.460094301	-6.898641307	-5.393622314	-6.826327513	1.289705605	0.331014137

RFC5	-0.437800405	0.01587548	-0.237971104	0.453675885	0.199829301	-0.253846584	8	0.042818561	0.816496581	0.816496581	0.816496581	0.816496581	0.816496581	0.816496581	18	29	0	P_112922	P_112922	P_112922	chr12	118016832	118016892	+	RFC5	"replication factor C (activator 1) 5, 36.5kDa [Source:HGNC Symbol;Acc:HGNC:9973]"	ENSG00000111445	protein_coding	5'UTR;exon	multiple Ref	1	2919	12:118016832:118016892	ENSG00000111445	RFC5	9.263767787	0.003679028	-8.840061111	0.320557521	-4.805109994	9.160618631	4.034951117	-5.125667515	9.961963646	4.99E-06	0.755168658	0.000725865	3.63E-06	0.002398922	0.000452405	4.608321911	-6.128205862	-0.567312712	4.873394445	-1.27790115	-0.095332129	37.96812522	9.21E-06

FKBP5	-0.719836022	0.160479118	-0.613190962	0.88031514	0.10664506	-0.77367008	8	0.071981845	0.816496581	0.816496581	0.816496581	0.816496581	0.816496581	0.816496581	18	30	0	P_256859	P_256859	P_256859	chr6	35619100	35619160	-	FKBP5	FK506 binding protein 5 [Source:HGNC Symbol;Acc:HGNC:3721]	ENSG00000096060	protein_coding	exon	coding exon	1	2920	6:35619100:35619160	ENSG00000096060	FKBP5	7.839746602	0.00636762	-11.04817776	2.463063485	-9.411369449	13.51124124	1.636808306	-11.87443293	9.961963646	6.54E-07	0.033584722	2.84E-06	9.89E-08	0.132829349	3.34E-07	6.64233178	-3.71321559	5.163326686	8.506214849	-5.040048812	7.304971334	90.48962118	1.58E-07

TEK	-0.113184599	0.020609832	0.082280264	0.133794431	0.195464863	0.061670432	8	0.08369606	0.816496581	0.816496581	0.816496581	0.816496581	0.816496581	0.816496581	18	31	0	P_267096	P_267096	P_267096	chr9	27228245	27228305	+	TEK	"TEK tyrosine kinase, endothelial [Source:HGNC Symbol;Acc:HGNC:11724]"	ENSG00000120156	protein_coding	3'UTR;exon	multiple Ref	1	2921	9:27228245:27228305	ENSG00000120156	TEK	4.171806781	0.007832105	-1.566367645	0.28522055	1.138680917	1.851588195	2.705048562	0.853460367	9.961963646	0.148447667	0.781317113	0.28147819	0.093914173	0.022191031	0.413454551	-5.802643546	-6.13938305	-6.381787156	-5.539945796	-3.410459203	-6.68742728	2.55091896	0.114657548

DESI1	-0.134250477	0.05828548	-0.014723846	0.192535957	0.119526632	-0.073009325	8	0.133364175	0.816496581	0.816496581	0.816496581	0.816496581	0.816496581	0.816496581	18	32	0	P_133511	P_133511	P_133511	chr22	41600578	41600638	-	DESI1	desumoylating isopeptidase 1 [Source:HGNC Symbol;Acc:HGNC:24577]	ENSG00000100418	protein_coding	3'UTR	3'UTR	1	2922	22:41600578:41600638	ENSG00000100418	DESI1	8.263031161	0.016489814	-1.280423345	0.555901852	-0.14042971	1.836325197	1.139993635	-0.696331562	9.961963646	0.229405151	0.590543818	0.891119319	0.096289436	0.280956194	0.502144213	-6.170672179	-6.021756428	-7.036361758	-5.562847873	-5.648343836	-6.812776954	1.187464113	0.363471224

C11orf58	0.001749215	-0.146140834	0.072635616	-0.147890049	0.070886401	0.218776449	8	0.148819347	0.816496581	0.816496581	0.816496581	0.816496581	0.816496581	0.816496581	18	33	0	P_108044	P_108044	P_108044	chr11	16738781	16738841	+	C11orf58	chromosome 11 open reading frame 58 [Source:HGNC Symbol;Acc:HGNC:16990]	ENSG00000110696	protein_coding	exon	coding exon	1	2923	11:16738781:16738841	ENSG00000110696	C11orf58	8.324169374	0.019992092	0.015151641	-1.265867046	0.629167289	-1.281018687	0.614015648	1.895034335	9.961963646	0.988210355	0.234362713	0.543396997	0.229204238	0.552967606	0.087446896	-6.998235043	-5.401330424	-6.835157666	-6.319286575	-6.099980407	-5.39804208	1.267946609	0.337641576

SERPINB6	0.043913271	-0.07316258	-0.028768217	-0.117075851	-0.072681488	0.044394363	8	0.084405399	0.816496581	0.816496581	0.816496581	0.816496581	0.816496581	0.816496581	18	34	0	P_256015	P_256015	P_256015	chr6	2953043	2953103	-	SERPINB6	"serpin peptidase inhibitor, clade B (ovalbumin), member 6 [Source:HGNC Symbol;Acc:HGNC:8950]"	ENSG00000124570	protein_coding	exon	coding exon	1	2924	6:2953043:2953103	ENSG00000124570	SERPINB6	10.55859533	0.007927862	0.604036758	-1.006367465	-0.395713182	-1.610404223	-0.99974994	0.610654283	9.961963646	0.559322713	0.338057018	0.700658603	0.138503686	0.341095988	0.555103729	-6.80284736	-5.67467177	-6.962275852	-5.890071613	-5.790904434	-6.871409191	0.91667086	0.467569233

SNX13	0.000113064	0.044936717	0.012583284	0.044823653	0.01247022	-0.032353433	8	0.015753616	0.816496581	0.816496581	0.816496581	0.816496581	0.816496581	0.816496581	18	35	0	P_260617	P_260617	P_260617	chr7	17893331	17893391	-	SNX13	sorting nexin 13 [Source:HGNC Symbol;Acc:HGNC:21335]	ENSG00000071189	protein_coding	exon	coding exon	1	2925	7:17893331:17893391	ENSG00000071189	SNX13	8.114702023	0.002405983	0.002823073	1.122020199	0.314190708	1.119197127	0.311367635	-0.807829492	9.961963646	0.99780325	0.288169447	0.759857154	0.289315441	0.761939338	0.438052877	-6.998355969	-5.558735861	-6.993478213	-6.50461221	-6.24537655	-6.726137231	0.5599656	0.653460961

SMARCA1	0.010370507	0.048627224	-0.048958972	0.038256717	-0.059329479	-0.097586196	8	0.075588596	0.816496581	0.816496581	0.816496581	0.816496581	0.816496581	0.816496581	18	36	0	P_271449	P_271449	P_271449	chrX	129506130	129506190	-	SMARCA1	"SWI/SNF related, matrix associated, actin dependent regulator of chromatin, subfamily a, member 1 [Source:HGNC Symbol;Acc:HGNC:11097]"	ENSG00000102038	protein_coding	exon	coding exon	1	2926	X:129506130:129506190	ENSG00000102038	SMARCA1	4.152439537	0.006795045	0.154081123	0.722485165	-0.727414153	0.568404042	-0.881495276	-1.449899318	9.961963646	0.880622502	0.486610568	0.483716962	0.582345851	0.398818816	0.177828998	-6.985420582	-5.914169843	-6.765624353	-6.975751231	-5.899226798	-6.030123854	0.712863711	0.56637332

SYT16	0.401972807	0.202769081	0.034156214	-0.199203726	-0.367816593	-0.168612868	8	0.267677555	0.816496581	0.816496581	0.816496581	0.816496581	0.816496581	0.816496581	18	37	0	P_115034	P_115034	P_115034	chr14	62100735	62100795	+	SYT16	synaptotagmin XVI [Source:HGNC Symbol;Acc:HGNC:23142]	ENSG00000139973	protein_coding	3'UTR	3'UTR	1	2927	14:62100735:62100795	ENSG00000139973	SYT16	2.83647518	0.059746564	2.014122302	1.015993425	0.1711429	-0.998128877	-1.842979402	-0.844850525	9.961963646	0.071788107	0.333672445	0.867537673	0.341843505	0.095247147	0.418022378	-5.144196646	-5.665404335	-7.031148736	-6.63049818	-4.750488802	-6.694872442	1.704137987	0.229086956

SHC2	0.374263612	0.184442447	0.2752023	-0.189821165	-0.099061312	0.090759853	8	0.103566527	0.816496581	0.816496581	0.816496581	0.816496581	0.816496581	0.816496581	18	38	0	P_231904	P_231904	P_231904	chr19	425096	425156	-	SHC2	SHC (Src homology 2 domain containing) transforming protein 2 [Source:HGNC Symbol;Acc:HGNC:29869]	ENSG00000129946	protein_coding	exon	coding exon	1	2928	19:425096:425156	ENSG00000129946	SHC2	6.378342481	0.010820267	4.406606178	2.171638388	3.240251301	-2.23496779	-1.166354877	1.068612913	9.961963646	0.001333563	0.055118191	0.008908625	0.049521739	0.270634008	0.310453961	-1.157100367	-4.16529931	-3.144829448	-4.938677486	-5.619940958	-6.481009129	7.021803012	0.008061096

SLC22A18AS	0.564501506	0.144635588	0.478563931	-0.419865918	-0.085937575	0.333928343	8	0.376809884	0.816496581	0.816496581	0.816496581	0.816496581	0.816496581	0.816496581	18	42	0	A_24_P372643	A_24_P372643	A_24_P372643	chr11	2888197	2888257	-	SLC22A18AS	"solute carrier family 22 (organic cation transporter), member 18 antisense [Source:HGNC Symbol;Acc:HGNC:10965]"	ENSG00000254827	protein_coding	exon	coding exon	1	2932	11:2888197:2888257	ENSG00000254827	SLC22A18AS	7.088956337	0.116228934	2.027933021	0.519593449	1.719208166	-1.508339571	-0.308724855	1.199614716	9.961963646	0.070153585	0.61469497	0.116436688	0.162508923	0.763890355	0.258041992	-5.122664458	-6.041736615	-5.63510179	-6.029764984	-6.246239581	-6.337419474	1.857940196	0.200814115

TXNIP	0.396894818	-0.084830889	0.231714549	-0.481725706	-0.165180269	0.316545437	8	0.134396057	0.816496581	0.816496581	0.816496581	0.816496581	0.816496581	0.816496581	18	43	0	P_203793	P_203793	P_203793	chr1	145993327	145993387	+	TXNIP	thioredoxin interacting protein [Source:HGNC Symbol;Acc:HGNC:16952]	ENSG00000265972	protein_coding	3'ext;3'UTR	multiple non-Ref	0	2933	1:145993327:145993387	ENSG00000265972	TXNIP	8.703265332	0.016711696	3.760198703	-0.803691515	2.195273677	-4.563890218	-1.564925027	2.998965192	9.961963646	0.003745889	0.440330901	0.052961705	0.001046129	0.14878384	0.013421519	-2.219255131	-5.852602709	-4.90405857	-1.03340834	-5.136581273	-3.580131432	8.646019557	0.00399405

PRKAG2	0.192077735	-0.006988583	0.179252525	-0.199066318	-0.012825211	0.186241108	8	0.085161652	0.816496581	0.816496581	0.816496581	0.816496581	0.816496581	0.816496581	18	44	0	P_263415	P_263415	P_263415	chr7	151595344	151595404	-	PRKAG2	"protein kinase, AMP-activated, gamma 2 non-catalytic subunit [Source:HGNC Symbol;Acc:HGNC:9386]"	ENSG00000106617	protein_coding	3'UTR;exon	multiple Ref	1	2934	7:151595344:151595404	ENSG00000106617	PRKAG2	6.717671864	0.008030842	2.62507733	-0.095511182	2.449798455	-2.720588512	-0.175278875	2.545309637	9.961963646	0.025450483	0.925802776	0.034354773	0.021607871	0.864371907	0.029176581	-4.147624544	-6.177280653	-4.487113527	-4.128930849	-6.280370255	-4.350657158	4.468765254	0.031026247

PGM2	-0.041366284	-0.053243296	-0.19313099	-0.011877012	-0.151764706	-0.139887695	8	0.088557569	0.816496581	0.816496581	0.816496581	0.816496581	0.816496581	0.816496581	18	47	0	A_23_P7172	A_23_P7172	A_23_P7172	chr4	37861926	37861986	+	PGM2	phosphoglucomutase 2 [Source:HGNC Symbol;Acc:HGNC:8906]	ENSG00000169299	protein_coding	3'UTR	3'UTR	1	2937	4:37861926:37861986	ENSG00000169299	PGM2	8.902790732	0.008504593	-0.549370534	-0.707104796	-2.564902255	-0.157734262	-2.015531722	-1.85779746	9.961963646	0.594850874	0.495709021	0.028213992	0.877817557	0.071619665	0.092963476	-6.836131561	-5.925171932	-4.294774211	-7.136024036	-4.495940861	-5.454306959	2.486531396	0.120518258

WDR89	0.024801856	0.063023304	0.082796641	0.038221448	0.057994785	0.019773337	8	0.045258114	0.816496581	0.816496581	0.816496581	0.816496581	0.816496581	0.816496581	18	48	0	A_23_P163047	A_23_P163047	A_23_P163047	chr14	63597159	63597219	-	WDR89	WD repeat domain 89 [Source:HGNC Symbol;Acc:HGNC:20489]	ENSG00000140006	protein_coding	3'UTR	3'UTR	1	2938	14:63597159:63597219	ENSG00000140006	WDR89	9.775309225	0.003851578	0.489452406	1.243733825	1.633950886	0.75428142	1.144498481	0.390217061	9.961963646	0.635119304	0.242068462	0.133433243	0.468135165	0.279170639	0.704589575	-6.86919205	-5.426449653	-5.756600025	-6.847090498	-5.643524925	-6.988827035	1.081219928	0.400968721

SNHG14	-0.0217518	-0.061013041	-0.110925033	-0.03926124	-0.089173233	-0.049911993	8	0.033989695	0.816496581	0.816496581	0.816496581	0.816496581	0.816496581	0.816496581	18	49	0	P_116020	P_116020	P_116020	chr15	25087494	25087554	+	SNHG14	small nucleolar RNA host gene 14 (non-protein coding) [Source:HGNC Symbol;Acc:HGNC:37462]	ENSG00000224078	processed_transcript	exon	non-coding exon	1	2939	15:25087494:25087554	ENSG00000224078	SNHG14	7.753871228	0.003134452	-0.475838963	-1.334711681	-2.426578586	-0.858872718	-1.950739623	-1.091866906	9.961963646	0.644451935	0.211672264	0.035744766	0.410600657	0.079761095	0.300593686	-6.876197737	-5.321218041	-4.525668612	-6.760533132	-4.592663523	-6.456461403	2.271897064	0.142765399

MAN1A2	0.272156445	-0.102671314	0.251746801	-0.37482776	-0.020409644	0.354418116	8	0.045896097	0.816496581	0.816496581	0.816496581	0.816496581	0.816496581	0.816496581	18	50	0	P_203648	P_203648	P_203648	chr1	117402368	117402428	+	MAN1A2	"mannosidase, alpha, class 1A, member 2 [Source:HGNC Symbol;Acc:HGNC:6822]"	ENSG00000198162	protein_coding	exon	coding exon	1	2940	1:117402368:117402428	ENSG00000198162	MAN1A2	9.435468832	0.00389828	5.3386047	-2.013994418	4.938250329	-7.352599118	-0.400354372	6.952244747	9.961963646	0.000333174	0.071803409	0.000595382	2.50E-05	0.697346301	4.01E-05	0.27923473	-4.402489364	-0.361503501	2.870717605	-6.212133678	2.427420973	26.5829824	4.53E-05

NCS1	-0.038821815	0.225764364	0.047417886	0.264586179	0.086239701	-0.178346478	8	0.065584741	0.816496581	0.816496581	0.816496581	0.816496581	0.816496581	0.816496581	18	51	0	A_23_P217049	A_23_P217049	A_23_P217049	chr9	130233512	130233572	+	NCS1	neuronal calcium sensor 1 [Source:HGNC Symbol;Acc:HGNC:3953]	ENSG00000107130	protein_coding	3'UTR	3'UTR	1	2941	9:130233512:130233572	ENSG00000107130	NCS1	8.394156527	0.005660909	-0.631943673	3.675004944	0.771870997	4.306948617	1.403814671	-2.903133947	9.961963646	0.54165366	0.004308915	0.458109956	0.001558084	0.190771407	0.015808047	-6.784726078	-1.788114023	-6.731183847	-1.447448216	-5.34329508	-3.743978333	7.241581286	0.007287208

IGFBP2	0.037099345	0.166880084	0.14527733	0.129780739	0.108177985	-0.021602754	8	0.105199949	0.816496581	0.816496581	0.816496581	0.816496581	0.816496581	0.816496581	18	52	0	P_129829	P_129829	P_129829	chr2	216660563	216660623	-	IGFBP2	"insulin-like growth factor binding protein 2, 36kDa [Source:HGNC Symbol;Acc:HGNC:5471]"	ENSG00000115457	protein_coding	"5'UTR,exon;exon;promoter"	multiple non-Ref	0	2942	2:216660563:216660623	ENSG00000115457	IGFBP2	10.05806577	0.011094112	0.431385556	1.9404563	1.689262758	1.509070744	1.257877202	-0.251193542	9.961963646	0.675374147	0.081130575	0.122170139	0.162324745	0.237120963	0.806769914	-6.897755048	-4.510801041	-5.678165872	-6.028784217	-5.517616429	-7.037017238	1.78795343	0.213147517

CCDC113	0.12899011	0.12936802	0.239065427	0.000377909	0.110075317	0.109697408	8	0.053933652	0.816496581	0.816496581	0.816496581	0.816496581	0.816496581	0.816496581	18	53	0	P_226507	P_226507	P_226507	chr16	58258461	58258521	+	CCDC113	coiled-coil domain containing 113 [Source:HGNC Symbol;Acc:HGNC:25002]	ENSG00000103021	protein_coding	3'UTR;exon	multiple Ref	1	2943	16:58258461:58258521	ENSG00000103021	CCDC113	8.705026278	0.00454264	2.343947477	2.350814668	4.344184253	0.006867191	2.000236776	1.993369586	9.961963646	0.0411536	0.040675142	0.001469846	0.994656391	0.073467719	0.074312023	-4.6156283	-3.889031976	-1.298530302	-7.149587274	-4.518888757	-5.247033506	6.311145471	0.011334874

ARHGAP12	0.395826625	0.029208581	0.135070296	-0.366618044	-0.260756329	0.105861715	8	0.09747445	0.816496581	0.816496581	0.816496581	0.816496581	0.816496581	0.816496581	18	57	0	P_207985	P_207985	P_207985	chr10	31908174	31908234	-	ARHGAP12	Rho GTPase activating protein 12 [Source:HGNC Symbol;Acc:HGNC:16348]	ENSG00000165322	protein_coding	exon	coding exon	1	2947	10:31908174:31908234	ENSG00000165322	ARHGAP12	7.090610169	0.009836721	4.887935503	0.360687363	1.667939586	-4.527248141	-3.219995917	1.307252223	9.961963646	0.000641488	0.725863993	0.126406779	0.001106653	0.009219067	0.220493948	-0.400030396	-6.113967353	-5.70857628	-1.091934647	-2.573455292	-6.210257969	9.896214855	0.002469315

MSX1	0.115064436	0.067467061	0.139515108	-0.047597375	0.024450672	0.072048047	8	0.112178395	0.816496581	0.816496581	0.816496581	0.816496581	0.816496581	0.816496581	18	58	0	P_137233	P_137233	P_137233	chr4	4863492	4863552	+	MSX1	msh homeobox 1 [Source:HGNC Symbol;Acc:HGNC:7391]	ENSG00000163132	protein_coding	3'UTR	3'UTR	1	2948	4:4863492:4863552	ENSG00000163132	MSX1	9.284493329	0.012312316	1.270038445	0.744676327	1.539915867	-0.525362118	0.269877422	0.79523954	9.961963646	0.23293313	0.473668516	0.154719188	0.610824316	0.792762569	0.445008209	-6.18308066	-5.897922738	-5.886439735	-7.000740477	-6.258091537	-6.736490294	0.920021078	0.466101695

FBXW12	0.153248919	0.122762092	0.115822854	-0.030486828	-0.037426065	-0.006939237	8	0.163387774	0.816496581	0.816496581	0.816496581	0.816496581	0.816496581	0.816496581	18	60	0	P_134717	P_134717	P_134717	chr3	48372797	48372857	+	FBXW12	F-box and WD repeat domain containing 12 [Source:HGNC Symbol;Acc:HGNC:20729]	ENSG00000164049	protein_coding	exon	coding exon	1	2950	3:48372797:48372857	ENSG00000164049	FBXW12	4.208241343	0.023644678	1.220608381	0.97778463	0.922514477	-0.242823751	-0.298093904	-0.055270153	9.961963646	0.250338375	0.351327864	0.378061619	0.813068432	0.771755992	0.957016006	-6.241136213	-5.701768435	-6.601291033	-7.117463593	-6.249638342	-7.06970994	0.574643117	0.644612267

RCHY1	0.174847404	0.041377114	0.091125984	-0.13347029	-0.083721421	0.04974887	8	0.265861594	0.816496581	0.816496581	0.816496581	0.816496581	0.816496581	0.816496581	18	62	0	A_23_P350070	A_23_P350070	A_23_P350070	chr4	75482425	75482485	-	RCHY1	"ring finger and CHY zinc finger domain containing 1, E3 ubiquitin protein ligase [Source:HGNC Symbol;Acc:HGNC:17479]"	ENSG00000163743	protein_coding	3'UTR;exon	multiple Ref	1	2952	4:75482425:75482485	ENSG00000163743	RCHY1	8.744566237	0.058968495	0.881850159	0.208687195	0.45959769	-0.673162964	-0.422252468	0.250910495	9.961963646	0.398635883	0.838897608	0.655671336	0.516155413	0.68180901	0.806982682	-6.58987519	-6.159181047	-6.932980618	-6.907305347	-6.202717944	-7.037094386	0.287806947	0.833225778

HIRA	-0.127098549	0.083288428	0.072110272	0.210386977	0.199208821	-0.011178156	8	0.151248661	0.816496581	0.816496581	0.816496581	0.816496581	0.816496581	0.816496581	18	63	0	P_242149	P_242149	P_242149	chr22	19388484	19388544	-	HIRA	histone cell cycle regulator [Source:HGNC Symbol;Acc:HGNC:4916]	ENSG00000100084	protein_coding	exon	coding exon	1	2953	22:19388484:19388544	ENSG00000100084	HIRA	6.505274451	0.020577486	-1.085150727	0.711105668	0.615668036	1.796256395	1.700818763	-0.095437632	9.961963646	0.303416472	0.493332185	0.551919235	0.102791208	0.119928011	0.925859722	-6.391294917	-5.922330447	-6.843988587	-5.622523758	-4.952065191	-7.066404896	1.365116725	0.309148461

RYBP	0.051045063	0.023568162	0.023268988	-0.027476901	-0.027776075	-0.000299174	8	0.055730572	0.816496581	0.816496581	0.816496581	0.816496581	0.816496581	0.816496581	18	64	0	P_244941	P_244941	P_244941	chr3	72379039	72379099	-	RYBP	RING1 and YY1 binding protein [Source:HGNC Symbol;Acc:HGNC:10480]	ENSG00000163602	protein_coding	exon	coding exon	1	2954	3:72379039:72379099	ENSG00000163602	RYBP	9.138666665	0.004700888	0.91182061	0.420999301	0.415655142	-0.490821309	-0.496165468	-0.005344158	9.961963646	0.383397811	0.682694057	0.68647401	0.634184521	0.630541627	0.995841503	-6.562750279	-6.089507117	-6.953584397	-7.019447064	-6.167389965	-7.071362923	0.27809012	0.839993808

TPMT	-0.073271136	0.17335463	-0.028640911	0.246625766	0.044630225	-0.201995541	8	0.067327849	0.816496581	0.816496581	0.816496581	0.816496581	0.816496581	0.816496581	18	66	0	A_24_P397498	A_24_P397498	A_24_P397498	chr6	18129489	18129549	-	TPMT	thiopurine S-methyltransferase [Source:HGNC Symbol;Acc:HGNC:12014]	ENSG00000137364	protein_coding	3'UTR	3'UTR	1	2956	6:18129489:18129549	ENSG00000137364	TPMT	8.365246114	0.005846962	-1.173581972	2.776616821	-0.458740764	3.950198793	0.714841208	-3.235357585	9.961963646	0.267857152	0.019630035	0.656265834	0.002749669	0.491119361	0.008982631	-6.294783855	-3.215140409	-6.933401405	-2.035469607	-6.031857525	-3.17544685	5.979990979	0.013394857

CENPU	-0.398469174	-0.08316524	-0.287540732	0.315303934	0.110928442	-0.204375492	8	0.058544989	0.816496581	0.816496581	0.816496581	0.816496581	0.816496581	0.816496581	18	67	0	P_251833	P_251833	P_251833	chr4	184700819	184700879	-	CENPU	centromere protein U [Source:HGNC Symbol;Acc:HGNC:21348]	ENSG00000151725	protein_coding	exon	coding exon	1	2957	4:184700819:184700879	ENSG00000151725	CENPU	11.50908614	0.004959166	-6.930044664	-1.446382463	-5.000813724	5.483662201	1.92923094	-3.554431261	9.961963646	4.12E-05	0.17878942	0.000542923	0.000271502	0.082650633	0.005260616	2.444229359	-5.185242639	-0.265658043	0.374917221	-4.624479918	-2.631369817	20.25869065	0.000145985

TBCK	0.143062291	-0.048613512	0.065053687	-0.191675803	-0.078008605	0.113667198	8	0.068965253	0.816496581	0.816496581	0.816496581	0.816496581	0.816496581	0.816496581	18	68	0	P_250346	P_250346	P_250346	chr4	106262097	106262157	-	TBCK	TBC1 domain containing kinase [Source:HGNC Symbol;Acc:HGNC:28261]	ENSG00000145348	protein_coding	exon	coding exon	1	2958	4:106262097:106262157	ENSG00000145348	TBCK	6.796318314	0.006026177	2.257095168	-0.766975849	1.026352648	-3.024071016	-1.23074252	1.793328496	9.961963646	0.047698951	0.460885899	0.329001404	0.012859117	0.246686591	0.103281782	-4.757411457	-5.881156451	-6.500486938	-3.610282369	-5.548541879	-5.550430899	3.43531501	0.06029522

SOX2-OT	0.141089773	0.307417448	0.203556364	0.166327675	0.062466591	-0.103861084	8	0.253129122	0.816496581	0.816496581	0.816496581	0.816496581	0.816496581	0.816496581	18	71	0	A_24_P756289	A_24_P756289	A_24_P756289	chr3	181740636	181740696	+	SOX2-OT	SOX2 overlapping transcript [Source:HGNC Symbol;Acc:HGNC:20209]	ENSG00000242808	sense_overlapping	exon	non-coding exon	1	2961	3:181740636:181740696	ENSG00000242808	SOX2-OT	3.67502197	0.053661883	0.745947413	1.62532865	1.076210842	0.879381237	0.330263429	-0.549117808	9.961963646	0.472933881	0.135270379	0.307205403	0.39990976	0.748041357	0.595017867	-6.702960591	-4.953365276	-6.449000087	-6.742420407	-6.238996082	-6.909112504	0.923379366	0.464635474

LINC01098	0.721217177	0.467193465	0.174615139	-0.254023712	-0.546602037	-0.292578326	8	0.313853452	0.816496581	0.816496581	0.816496581	0.816496581	0.816496581	0.816496581	18	73	0	P_251749	P_251749	P_251749	chr4	177960845	177960905	+	LINC01098	long intergenic non-protein coding RNA 1098 [Source:HGNC Symbol;Acc:HGNC:27731]	ENSG00000231171	lincRNA	exon	non-coding exon	1	2963	4:177960845:177960905	ENSG00000231171	LINC01098	3.281251394	0.081310758	3.097688081	2.006634999	0.749986624	-1.091053083	-2.347701457	-1.256648375	9.961963646	0.011344468	0.072689224	0.470604204	0.300934653	0.040891372	0.237547522	-3.343935077	-4.413401094	-6.748365335	-6.534892319	-3.983020974	-6.271026609	3.744333251	0.04899078

TBC1D19	0.105085021	0.097221171	0.054960829	-0.00786385	-0.050124192	-0.042260343	8	0.042290128	0.816496581	0.816496581	0.816496581	0.816496581	0.816496581	0.816496581	18	74	0	P_248457	P_248457	P_248457	chr4	26666345	26666405	+	TBC1D19	"TBC1 domain family, member 19 [Source:HGNC Symbol;Acc:HGNC:25624]"	ENSG00000109680	protein_coding	exon	coding exon	1	2964	4:26666345:26666405	ENSG00000109680	TBC1D19	6.811961533	0.003642911	2.132368094	1.97279614	1.115256164	-0.159571954	-1.01711193	-0.857539976	9.961963646	0.058888966	0.076896531	0.290921159	0.876407213	0.333165733	0.411302144	-4.957972399	-4.463369778	-6.407335875	-7.13570596	-5.774061961	-6.683876791	1.913011551	0.19168835

HS3ST3B1	0.254918664	0.121870974	0.275392378	-0.13304769	0.020473715	0.153521404	8	0.137634505	0.816496581	0.816496581	0.816496581	0.816496581	0.816496581	0.816496581	18	76	0	A_23_P77918	A_23_P77918	A_23_P77918	chr17	14345924	14345984	+	HS3ST3B1	heparan sulfate (glucosamine) 3-O-sulfotransferase 3B1 [Source:HGNC Symbol;Acc:HGNC:5198]	ENSG00000125430	protein_coding	3'UTR	3'UTR	1	2966	17:14345924:14345984	ENSG00000125430	HS3ST3B1	3.844166524	0.017419152	2.365558842	1.130921354	2.555547977	-1.234637488	0.189989135	1.424626623	9.961963646	0.039666161	0.284579287	0.028669575	0.245294579	0.853133135	0.184830246	-4.580109881	-5.549408266	-4.310474421	-6.374294652	-6.277522198	-6.062946547	2.832610637	0.092659434

YDJC	-0.333482618	0.090956448	-0.281023812	0.424439066	0.052458806	-0.37198026	8	0.081846543	0.816496581	0.816496581	0.816496581	0.816496581	0.816496581	0.816496581	18	78	0	A_24_P92367	A_24_P92367	A_24_P92367	chr22	21628234	21628294	-	YDJC	YdjC homolog (bacterial) [Source:HGNC Symbol;Acc:HGNC:27158]	ENSG00000161179	protein_coding	3'UTR;exon	multiple Ref	1	2968	22:21628234:21628294	ENSG00000161179	YDJC	8.570184052	0.007586231	-4.689276033	1.278986874	-3.951624934	5.968262907	0.737651099	-5.230611808	9.961963646	0.000864227	0.22989052	0.002743341	0.000139975	0.477741873	0.000388775	-0.708657561	-5.386292214	-1.942285699	1.068389784	-6.015151195	0.062503554	17.12734132	0.000293807

SOST	-0.203200982	0.067958022	-0.044033947	0.271159005	0.159167035	-0.111991969	8	0.183545191	0.816496581	0.816496581	0.816496581	0.816496581	0.816496581	0.816496581	18	79	0	P_121669	P_121669	P_121669	chr17	43758559	43758619	-	SOST	sclerostin [Source:HGNC Symbol;Acc:HGNC:13771]	ENSG00000167941	protein_coding	exon	coding exon	1	2969	17:43758559:43758619	ENSG00000167941	SOST	3.966737688	0.029260657	-1.454887441	0.486568874	-0.315276215	1.941456316	1.139611227	-0.801845089	9.961963646	0.176474396	0.637090581	0.759057063	0.080996438	0.281108179	0.441349919	-5.95187476	-6.058800918	-6.993108763	-5.403295249	-5.648752239	-6.731076172	1.360627958	0.310403564

PAXX	-0.045402957	0.17418561	0.107100235	0.219588566	0.152503192	-0.067085375	8	0.100671714	0.816496581	0.816496581	0.816496581	0.816496581	0.816496581	0.816496581	18	80	0	A_23_P312646	A_23_P312646	A_23_P312646	chr9	136993656	136993817	+	C9orf142;CLIC3	chromosome 9 open reading frame 142 [Source:HGNC Symbol;Acc:HGNC:27849];chloride intracellular channel 3 [Source:HGNC Symbol;Acc:HGNC:2064]	ENSG00000148362	protein_coding	"3'ext;3'UTR,exon,intron;exon,intron"	multiple non-Ref	0	2970	9:136993656:136993817	ENSG00000148362	PAXX	10.25755758	0.010345476	-0.54670686	2.097406749	1.289617187	2.644113609	1.836324046	-0.807789563	9.961963646	0.59661218	0.06245277	0.22631857	0.024633684	0.096289617	0.438074821	-6.837677751	-4.277772333	-6.207692633	-4.258631642	-4.760101813	-6.726170291	2.896177118	0.088409092

FUBP1	0.033099793	-0.230439463	-0.119764367	-0.263539256	-0.15286416	0.110675096	8	0.067637745	0.816496581	0.816496581	0.816496581	0.816496581	0.816496581	0.816496581	18	81	0	P_102215	P_102215	P_102215	chr1	77949190	77949250	-	FUBP1	far upstream element (FUSE) binding protein 1 [Source:HGNC Symbol;Acc:HGNC:4004]	ENSG00000162613	protein_coding	exon	coding exon	1	2971	1:77949190:77949250	ENSG00000162613	FUBP1	8.980646731	0.00588055	0.528642347	-3.680387302	-1.912776788	-4.209029649	-2.441419135	1.767610514	9.961963646	0.608628939	0.004270859	0.084926919	0.001817887	0.034850135	0.107683989	-6.847976712	-1.779737853	-5.347497439	-1.607397882	-3.834364025	-5.588298622	7.380722229	0.006843148

MDH1B	0.151498751	0.106929506	0.299612071	-0.044569244	0.148113321	0.192682565	8	0.111535654	0.816496581	0.816496581	0.816496581	0.816496581	0.816496581	0.816496581	18	82	0	P_238939	P_238939	P_238939	chr2	206739592	206739652	-	MDH1B	"malate dehydrogenase 1B, NAD (soluble) [Source:HGNC Symbol;Acc:HGNC:17836]"	ENSG00000138400	protein_coding	3'UTR;exon	multiple Ref	1	2972	2:206739592:206739652	ENSG00000138400	MDH1B	6.428882731	0.012196845	1.68008377	1.185821846	3.322623956	-0.494261924	1.642540186	2.13680211	9.961963646	0.12397795	0.263205923	0.007752802	0.631838011	0.131625321	0.058451132	-5.643699903	-5.490651071	-3.004085878	-7.017637904	-5.03224586	-5.021147953	3.796140208	0.04735198

PTPRZ1	-0.039036741	-0.070985443	0.174345784	-0.031948702	0.213382525	0.245331228	8	0.113463736	0.816496581	0.816496581	0.816496581	0.816496581	0.816496581	0.816496581	18	83	0	A_24_P235248	A_24_P235248	A_24_P235248	chr7	122061673	122061733	+	PTPRZ1	"protein tyrosine phosphatase, receptor-type, Z polypeptide 1 [Source:HGNC Symbol;Acc:HGNC:9685]"	ENSG00000106278	protein_coding	3'UTR	3'UTR	1	2973	7:122061673:122061733	ENSG00000106278	PTPRZ1	3.696733442	0.012545224	-0.426854614	-0.776203731	1.906416894	-0.349349117	2.333271509	2.682620625	9.961963646	0.678563106	0.455662014	0.085822332	0.734099226	0.041908357	0.023060569	-6.899838218	-5.874090401	-5.357181513	-7.083278816	-4.005784954	-4.119193019	2.863629438	0.090555895

JAKMIP1	0.431729157	0.06071338	0.470350179	-0.371015777	0.038621022	0.409636799	8	0.07547331	0.816496581	0.816496581	0.816496581	0.816496581	0.816496581	0.816496581	18	84	0	P_137253	P_137253	P_137253	chr4	6053974	6054034	-	JAKMIP1	janus kinase and microtubule interacting protein 1 [Source:HGNC Symbol;Acc:HGNC:26460]	ENSG00000152969	protein_coding	3'UTR;exon	multiple Ref	1	2974	4:6053974:6054034	ENSG00000152969	JAKMIP1	8.447591053	0.00678106	6.421082163	0.902986495	6.995490331	-5.518095668	0.574408168	6.092503836	9.961963646	7.76E-05	0.387846052	3.81E-05	0.000258738	0.578430747	0.000118722	1.790575643	-5.76962321	2.496699621	0.425303348	-6.124130181	1.298629022	26.48008221	4.61E-05

OXCT1	-0.261516084	0.008626445	-0.046919983	0.270142529	0.214596101	-0.055546428	8	0.136248046	0.816496581	0.816496581	0.816496581	0.816496581	0.816496581	0.816496581	18	85	0	P_252654	P_252654	P_252654	chr5	41794676	41794736	-	OXCT1	3-oxoacid CoA transferase 1 [Source:HGNC Symbol;Acc:HGNC:8527]	ENSG00000083720	protein_coding	exon	coding exon	1	2975	5:41794676:41794736	ENSG00000083720	OXCT1	10.18669197	0.017114211	-2.448305433	0.080760509	-0.439263416	2.529065942	2.009042016	-0.520023926	9.961963646	0.034442526	0.937231862	0.669845611	0.029999267	0.072398374	0.614405699	-4.443328864	-6.178650246	-6.942762317	-4.452534998	-4.505686219	-6.925627995	2.816087225	0.093803528

C11orf71	0.104352779	-0.030506214	0.068331165	-0.134858992	-0.036021614	0.098837379	8	0.137488638	0.816496581	0.816496581	0.816496581	0.816496581	0.816496581	0.816496581	18	86	0	A_24_P210082	A_24_P210082	A_24_P210082	chr11	114400004	114400064	-	C11orf71	chromosome 11 open reading frame 71 [Source:HGNC Symbol;Acc:HGNC:25937]	ENSG00000180425	protein_coding	exon	coding exon	1	2976	11:114400004:114400064	ENSG00000180425	C11orf71	7.529464641	0.017386924	0.969255479	-0.283349565	0.634677458	-1.252605045	-0.334578021	0.918027024	9.961963646	0.355360988	0.782709937	0.539940231	0.238955491	0.744880973	0.380294371	-6.508645307	-6.139939146	-6.831502217	-6.353159623	-6.237487651	-6.62954067	0.657715684	0.596479143

GRIN2A	0.038593583	0.225363335	0.034174868	0.186769752	-0.004418715	-0.191188467	8	0.193874246	0.816496581	0.816496581	0.816496581	0.816496581	0.816496581	0.816496581	18	87	0	P_118243	P_118243	P_118243	chr16	9763637	9763697	-	GRIN2A	"glutamate receptor, ionotropic, N-methyl D-aspartate 2A [Source:HGNC Symbol;Acc:HGNC:4585]"	ENSG00000183454	protein_coding	exon	coding exon	1	2977	16:9763637:9763697	ENSG00000183454	GRIN2A	3.911250624	0.032391274	0.262631443	1.533609805	0.232561843	1.270978362	-0.0300696	-1.301047961	9.961963646	0.798186027	0.156248216	0.820809907	0.232611999	0.976605435	0.222528831	-6.960851813	-5.074233217	-7.017664707	-6.331318533	-6.296204358	-6.21780022	0.964005882	0.447278222

MED23	-0.053359119	-0.037000436	-0.07882917	0.016358683	-0.025470051	-0.041828734	8	0.041414518	0.816496581	0.816496581	0.816496581	0.816496581	0.816496581	0.816496581	18	89	0	A_23_P145501	A_23_P145501	A_23_P145501	chr6	131587648	131587707	-	MED23;ARG1	mediator complex subunit 23 [Source:HGNC Symbol;Acc:HGNC:2372];arginase 1 [Source:HGNC Symbol;Acc:HGNC:663]	ENSG00000112282	protein_coding	"3'ext;3'UTR,exon;exon;intron"	multiple non-Ref	0	2979	6:131587648:131587707	ENSG00000112282	MED23	7.227555357	0.003584053	-1.091608923	-0.75694664	-1.612669529	0.334662284	-0.521060605	-0.855722889	9.961963646	0.30070174	0.466607133	0.138008531	0.744819301	0.613709356	0.41225989	-6.384445523	-5.888751355	-5.78638049	-7.088708446	-6.154272924	-6.685460007	0.913507655	0.468959298

CD44	0.165396179	-0.085772699	-0.112576444	-0.251168879	-0.277972623	-0.026803745	8	0.172944151	0.816496581	0.816496581	0.816496581	0.816496581	0.816496581	0.816496581	18	90	0	P_108299	P_108299	P_108299	chr11	35229227	35229287	+	CD44	CD44 molecule (Indian blood group) [Source:HGNC Symbol;Acc:HGNC:1681]	ENSG00000026508	protein_coding	3'UTR;exon	multiple Ref	1	2980	11:35229227:35229287	ENSG00000026508	CD44	3.831940591	0.026225787	1.250854612	-0.648679896	-0.851390671	-1.899534508	-2.102245282	-0.202710774	9.961963646	0.239567133	0.531213448	0.414549469	0.08680122	0.061947549	0.843441322	-6.205810995	-5.96500861	-6.66510504	-5.467414579	-4.364583571	-7.048976507	1.796507863	0.211593937

PPP2R5E	0.180631718	-0.021674515	0.174841615	-0.202306233	-0.005790103	0.19651613	8	0.045698364	0.816496581	0.816496581	0.816496581	0.816496581	0.816496581	0.816496581	18	91	0	P_221127	P_221127	P_221127	chr14	63453688	63453748	-	PPP2R5E	"protein phosphatase 2, regulatory subunit B', epsilon isoform [Source:HGNC Symbol;Acc:HGNC:9313]"	ENSG00000154001	protein_coding	exon	coding exon	1	2981	14:63453688:63453748	ENSG00000154001	PPP2R5E	10.00804208	0.003883735	3.54988949	-0.425961364	3.436098705	-3.975850855	-0.113790785	3.86206007	9.961963646	0.005300471	0.679192581	0.006408179	0.002638158	0.911664252	0.003171981	-2.573688812	-6.087332405	-2.810602444	-1.992722835	-6.289802827	-2.112925773	9.220963189	0.003183374

SPEF2	0.097800083	0.119018031	0.17080623	0.021217948	0.073006147	0.051788199	8	0.084025973	0.816496581	0.816496581	0.816496581	0.816496581	0.816496581	0.816496581	18	92	0	P_139339	P_139339	P_139339	chr5	35773952	35774012	+	SPEF2	sperm flagellar 2 [Source:HGNC Symbol;Acc:HGNC:26293]	ENSG00000152582	protein_coding	exon	coding exon	1	2982	5:35773952:35774012	ENSG00000152582	SPEF2	4.383782568	0.007876541	1.349637306	1.642444151	2.357119266	0.292806845	1.007481959	0.714675115	9.961963646	0.207002061	0.131645413	0.040240669	0.775677978	0.337547191	0.491217618	-6.086156262	-4.93037004	-4.640438986	-7.102928817	-5.78343272	-6.799314765	1.947778524	0.18617511

PINK1-AS	-0.120457421	-0.049932102	-0.075858472	0.070525319	0.044598949	-0.02592637	8	0.083100039	0.816496581	0.816496581	0.816496581	0.816496581	0.816496581	0.816496581	18	93	0	P_100685	P_100685	P_100685	chr1	20650559	20650619	-	PINK1-AS	PINK1 antisense RNA [Source:HGNC Symbol;Acc:HGNC:38872]	ENSG00000117242	antisense	exon	non-coding exon	1	2983	1:20650559:20650619	ENSG00000117242	PINK1-AS	5.644085075	0.007752271	-1.675578301	-0.694561999	-1.055201156	0.981016303	0.620377146	-0.360639157	9.961963646	0.124874096	0.5032061	0.316252111	0.349808511	0.548937709	0.72589893	-5.650117565	-5.933986342	-6.470933625	-6.647351801	-6.095962664	-7.000789038	0.980124981	0.440583076

RNF32	0.187312987	0.03157451	0.139148631	-0.155738477	-0.048164356	0.107574121	8	0.067792051	0.816496581	0.816496581	0.816496581	0.816496581	0.816496581	0.816496581	18	95	0	A_24_P315291	A_24_P315291	A_24_P315291	chr7	156659372	156659432	+	RNF32	ring finger protein 32 [Source:HGNC Symbol;Acc:HGNC:17118]	ENSG00000105982	protein_coding	3'UTR	3'UTR	1	2985	7:156659372:156659432	ENSG00000105982	RNF32	6.731269633	0.005897332	2.987347495	0.503563767	2.219201775	-2.483783728	-0.76814572	1.715638008	9.961963646	0.013690175	0.62551562	0.050861412	0.032415801	0.4602215	0.11710721	-3.532408821	-6.050152065	-4.865458908	-4.52837247	-5.99208673	-5.663947838	3.967553546	0.042375863

PER2	0.373228519	0.20221402	0.334143502	-0.1710145	-0.039085017	0.131929483	8	0.06262172	0.816496581	0.816496581	0.816496581	0.816496581	0.816496581	0.816496581	18	96	0	P_239843	P_239843	P_239843	chr2	238275808	238275868	-	PER2	period circadian clock 2 [Source:HGNC Symbol;Acc:HGNC:8846]	ENSG00000132326	protein_coding	exon	coding exon	1	2986	2:238275808:238275868	ENSG00000132326	PER2	7.028038788	0.005355846	6.246063679	3.384097351	5.591967083	-2.861966328	-0.654096596	2.207869732	9.961963646	9.71E-05	0.006991675	0.000233474	0.016961265	0.527859876	0.051845869	1.557572976	-2.245107982	0.612155023	-3.887904028	-6.074030639	-4.907075938	15.8714841	0.000400808

MPRIP	0.099968436	-0.0833512	0.058507622	-0.183319636	-0.041460813	0.141858823	8	0.10628346	0.816496581	0.816496581	0.816496581	0.816496581	0.816496581	0.816496581	18	97	0	A_32_P105976	A_32_P105976	A_32_P105976	chr17	17192031	17192091	+	MPRIP	myosin phosphatase Rho interacting protein [Source:HGNC Symbol;Acc:HGNC:30321]	ENSG00000133030	protein_coding	3'UTR	3'UTR	1	2987	17:17192031:17192091	ENSG00000133030	MPRIP	7.001804927	0.011278128	1.152895711	-0.961255828	0.674744849	-2.114151539	-0.478150862	1.636000678	9.961963646	0.275866109	0.359174327	0.515191354	0.060720904	0.642862387	0.132999787	-6.317879501	-5.717146728	-6.804042222	-5.132947308	-6.176501078	-5.77746144	1.680537516	0.233820155

SF3A1	-0.099307548	0.003769109	-0.095925567	0.103076657	0.003381981	-0.099694676	8	0.054934465	0.816496581	0.816496581	0.816496581	0.816496581	0.816496581	0.816496581	18	99	0	P_133018	P_133018	P_133018	chr22	30334632	30334692	-	SF3A1	"splicing factor 3a, subunit 1, 120kDa [Source:HGNC Symbol;Acc:HGNC:10765]"	ENSG00000099995	protein_coding	exon	coding exon	1	2989	22:30334632:30334692	ENSG00000099995	SF3A1	9.300105722	0.004630138	-1.787437676	0.067840241	-1.726565363	1.855277917	0.060872313	-1.794405603	9.961963646	0.104275312	0.947255118	0.115065879	0.093348162	0.95266481	0.103101062	-5.488059314	-6.179661807	-5.624458971	-5.534395585	-6.294714762	-5.548838854	2.141036126	0.158692745

RNU1-63P	-0.377039248	-0.185148865	-0.274520316	0.191890382	0.102518931	-0.089371451	8	0.354783531	0.816496581	0.816496581	0.816496581	0.816496581	0.816496581	0.816496581	18	102	0	A_32_P198029	A_32_P198029	A_32_P198029	chr4	67431436	67431496	-	RNU1-63P;RP11-584P21.2	"RNA, U1 small nuclear 63, pseudogene [Source:HGNC Symbol;Acc:HGNC:48405];"	ENSG00000206629	snRNA;lincRNA	intron;promoter	multiple non-Ref	0	2992	4:67431436:67431496	ENSG00000206629	RNU1-63P	3.679488552	0.103288245	-1.43683448	-0.705571834	-1.046151716	0.731262646	0.390682764	-0.340579882	9.961963646	0.181419385	0.496621592	0.320210541	0.481465191	0.704256136	0.740492947	-5.975380533	-5.926256843	-6.480274817	-6.864803375	-6.216137916	-7.008378911	0.74335518	0.550358147

HEBP2	-0.291783216	-0.021005958	-0.404642556	0.270777258	-0.11285934	-0.383636599	8	0.121373754	0.816496581	0.816496581	0.816496581	0.816496581	0.816496581	0.816496581	18	103	0	A_16_P17742090	A_16_P17742090	A_16_P17742090	chr6	138417320	138417380	+	HEBP2	heme binding protein 2 [Source:HGNC Symbol;Acc:HGNC:15716]	ENSG00000051620	protein_coding	3'UTR	3'UTR	1	2993	6:138417320:138417380	ENSG00000051620	HEBP2	9.71810599	0.014036953	-3.016265171	-0.217145931	-4.182931652	2.799119239	-1.166666481	-3.96578572	9.961963646	0.013031361	0.832477301	0.00189458	0.018887844	0.270513812	0.002681335	-3.483030621	-6.157291134	-1.560806797	-3.995217162	-5.619602296	-1.94001776	8.594260119	0.004078544

MTO1	-0.089555619	0.082675589	0.074059072	0.172231208	0.163614691	-0.008616517	8	0.068060692	0.816496581	0.816496581	0.816496581	0.816496581	0.816496581	0.816496581	18	104	0	P_257777	P_257777	P_257777	chr6	73492292	73492352	+	MTO1	mitochondrial tRNA translation optimization 1 [Source:HGNC Symbol;Acc:HGNC:19261]	ENSG00000135297	protein_coding	3'UTR;exon	multiple Ref	1	2994	6:73492292:73492352	ENSG00000135297	MTO1	8.457586216	0.00592664	-1.424735388	1.315281372	1.178201681	2.74001676	2.602937069	-0.13707969	9.961963646	0.184799617	0.217883449	0.266094014	0.020900354	0.026434467	0.893698671	-5.991026734	-5.344126199	-6.337764324	-4.095895474	-3.575444939	-7.061122761	3.241626548	0.068967848

ESR2	0.058056804	0.028280732	0.183034604	-0.029776073	0.1249778	0.154753872	8	0.119372069	0.816496581	0.816496581	0.816496581	0.816496581	0.816496581	0.816496581	18	105	0	P_115072	P_115072	P_115072	chr14	64279980	64280040	-	ESR2	estrogen receptor 2 (ER beta) [Source:HGNC Symbol;Acc:HGNC:3468]	ENSG00000140009	protein_coding	exon	coding exon	1	2995	14:64279980:64280040	ENSG00000140009	ESR2	4.150407597	0.013649963	0.608601506	0.296463027	1.918726616	-0.312138479	1.31012511	1.62226359	9.961963646	0.556410546	0.772965077	0.084097138	0.761370596	0.219556929	0.135928867	-6.799935124	-6.135965829	-5.338424418	-7.096592051	-5.456723516	-5.796733098	1.430899223	0.291396902

UBE2K	-0.111294517	-0.052464702	-0.082221394	0.058829815	0.029073123	-0.029756692	8	0.042301072	0.816496581	0.816496581	0.816496581	0.816496581	0.816496581	0.816496581	18	106	0	P_137567	P_137567	P_137567	chr4	39782633	39782693	+	UBE2K	ubiquitin-conjugating enzyme E2K [Source:HGNC Symbol;Acc:HGNC:4914]	ENSG00000078140	protein_coding	3'UTR	3'UTR	1	2996	4:39782633:39782693	ENSG00000078140	UBE2K	11.02460711	0.003643654	-2.258139763	-1.064496555	-1.668252882	1.193643208	0.589886881	-0.603756327	9.961963646	0.047614526	0.312224859	0.126343586	0.260267634	0.568403977	0.559501898	-4.75571588	-5.617638998	-5.708131024	-6.421671817	-6.114870528	-6.875821061	1.858781354	0.20067099

TBCEL	-0.032516178	0.050054449	-0.069738448	0.082570627	-0.03722227	-0.119792897	8	0.089038889	0.816496581	0.816496581	0.816496581	0.816496581	0.816496581	0.816496581	18	109	0	P_110329	P_110329	P_110329	chr11	121090301	121090361	-	TBCEL	tubulin folding cofactor E-like [Source:HGNC Symbol;Acc:HGNC:28115]	ENSG00000154114	protein_coding	3'ext;3'UTR	multiple non-Ref	0	2999	11:121090301:121090361	ENSG00000154114	TBCEL	6.352110399	0.008573239	-0.43010318	0.6620882	-0.922455536	1.09219138	-0.492352356	-1.584543736	9.961963646	0.676276027	0.522934999	0.378090884	0.300457824	0.633139815	0.144269189	-6.89834679	-5.956142477	-6.601345711	-6.533679333	-6.169345238	-5.849162044	0.903390909	0.473434208

PKN1	-0.180741957	0.031396897	-0.098731972	0.212138854	0.082009985	-0.130128869	8	0.107715757	0.816496581	0.816496581	0.816496581	0.816496581	0.816496581	0.816496581	18	112	0	P_232445	P_232445	P_232445	chr19	14463909	14463969	+	PKN1	protein kinase N1 [Source:HGNC Symbol;Acc:HGNC:9405]	ENSG00000123143	protein_coding	exon	coding exon	1	3002	19:14463909:14463969	ENSG00000123143	PKN1	10.48475473	0.011524272	-2.062043657	0.358200021	-1.126410495	2.420243678	0.935633162	-1.484610517	9.961963646	0.066266265	0.727667516	0.28639429	0.036133493	0.371587978	0.168585669	-5.069227798	-6.114897424	-6.395221293	-4.634251345	-5.851042116	-5.984436407	2.431031527	0.125854496

TMEM184C	0.083967847	-0.10547571	-0.059732201	-0.189443558	-0.143700048	0.045743509	8	0.027956642	0.816496581	0.816496581	0.816496581	0.816496581	0.816496581	0.816496581	18	113	0	P_251097	P_251097	P_251097	chr4	147623901	147623961	+	TMEM184C	transmembrane protein 184C [Source:HGNC Symbol;Acc:HGNC:25587]	ENSG00000164168	protein_coding	exon	coding exon	1	3003	4:147623901:147623961	ENSG00000164168	TMEM184C	7.763172291	0.00283433	1.931672125	-2.42645841	-1.374133446	-4.358130535	-3.305805572	1.052324964	9.961963646	0.082317815	0.035752102	0.19952473	0.001438169	0.007975563	0.317506181	-5.271434297	-3.770743249	-6.103480586	-1.364306561	-2.434427377	-6.4979536	7.089390069	0.007813059

CEP41	0.237717326	0.093272961	0.328538959	-0.144444365	0.090821633	0.235265998	8	0.066872751	0.816496581	0.816496581	0.816496581	0.816496581	0.816496581	0.816496581	18	115	0	P_262899	P_262899	P_262899	chr7	130427954	130428014	-	CEP41	centrosomal protein 41kDa [Source:HGNC Symbol;Acc:HGNC:12370]	ENSG00000106477	protein_coding	"5'UTR,exon;exon"	multiple Ref	1	3005	7:130427954:130428014	ENSG00000106477	CEP41	8.673952277	0.005797916	3.823583	1.500256271	5.284410692	-2.323326728	1.460827693	3.784154421	9.961963646	0.003377139	0.16455722	0.000359929	0.042623559	0.174872827	0.00360181	-2.113150755	-5.117152464	0.161770023	-4.794371564	-5.271754947	-2.243471055	11.10787359	0.001612972

TNNI2	0.03576127	-0.206326607	0.056655419	-0.242087877	0.020894149	0.262982026	8	0.243718821	0.816496581	0.816496581	0.816496581	0.816496581	0.816496581	0.816496581	18	116	0	A_23_P24784	A_23_P24784	A_23_P24784	chr11	1841454	1841514	+	TNNI2	"troponin I type 2 (skeletal, fast) [Source:HGNC Symbol;Acc:HGNC:11946]"	ENSG00000130598	protein_coding	exon	coding exon	1	3006	11:1841454:1841514	ENSG00000130598	TNNI2	5.667463523	0.04990721	0.196054547	-1.131147445	0.310602851	-1.327201992	0.114548305	1.441750297	9.961963646	0.848508973	0.284488554	0.762503756	0.214055121	0.911079003	0.180061221	-6.977426027	-5.549170611	-6.994690447	-6.263079124	-6.28971095	-6.040748478	0.877939403	0.484890173

ARCN1	-0.031789823	0.06789655	0.063861285	0.099686373	0.095651108	-0.004035265	8	0.06806976	0.816496581	0.816496581	0.816496581	0.816496581	0.816496581	0.816496581	18	119	0	P_110223	P_110223	P_110223	chr11	118600934	118600994	+	ARCN1	archain 1 [Source:HGNC Symbol;Acc:HGNC:649]	ENSG00000095139	protein_coding	3'UTR	3'UTR	1	3009	11:118600934:118600994	ENSG00000095139	ARCN1	9.735719725	0.005927631	-0.505700252	1.08007213	1.015880689	1.585772382	1.521580941	-0.064191441	9.961963646	0.624067929	0.305564476	0.333723549	0.143990527	0.159201577	0.950087606	-6.860584474	-5.60192799	-6.511051333	-5.924286691	-5.193553873	-7.069127928	1.214717546	0.354486771

TRPC5	-0.020378681	0.075418828	0.038386372	0.095797509	0.058765053	-0.037032456	8	0.2059582	0.816496581	0.816496581	0.816496581	0.816496581	0.816496581	0.816496581	18	121	0	P_151510	P_151510	P_151510	chrX	111774383	111774443	-	TRPC5	"transient receptor potential cation channel, subfamily C, member 5 [Source:HGNC Symbol;Acc:HGNC:12337]"	ENSG00000072315	protein_coding	3'UTR	3'UTR	1	3011	X:111774383:111774443	ENSG00000072315	TRPC5	4.061437008	0.036271277	-0.131050983	0.485002509	0.246854627	0.616053491	0.37790561	-0.238147881	9.961963646	0.898343739	0.638162648	0.810033299	0.551674843	0.713428093	0.816593295	-6.988996538	-6.059583845	-7.013944291	-6.945944385	-6.22128238	-7.040483726	0.148731328	0.928135606

NLRP1	0.193326787	-0.176973822	-0.005322263	-0.370300608	-0.198649049	0.171651559	8	0.137909307	0.816496581	0.816496581	0.816496581	0.816496581	0.816496581	0.816496581	18	122	0	P_120264	P_120264	P_120264	chr17	5536857	5536917	-	NLRP1	"NLR family, pyrin domain containing 1 [Source:HGNC Symbol;Acc:HGNC:14374]"	ENSG00000091592	protein_coding	exon	coding exon	1	3012	17:5536857:5536917	ENSG00000091592	NLRP1	4.851733351	0.017479959	1.790883981	-1.639398179	-0.049302815	-3.430282159	-1.840186796	1.590095364	9.961963646	0.103693009	0.132284111	0.961652462	0.006470866	0.095683294	0.143013842	-5.482979717	-4.934472166	-7.045793298	-2.914169164	-4.754524441	-5.841491087	3.929808165	0.043415955

TANK	0.068215891	0.00692971	0.006243734	-0.061286181	-0.061972157	-0.000685976	8	0.056198203	0.816496581	0.816496581	0.816496581	0.816496581	0.816496581	0.816496581	18	123	0	P_237167	P_237167	P_237167	chr2	161223931	161223991	+	TANK	TRAF family member-associated NFKB activator [Source:HGNC Symbol;Acc:HGNC:11562]	ENSG00000136560	protein_coding	3'UTR;exon	multiple Ref	1	3013	2:161223931:161223991	ENSG00000136560	TANK	8.083559037	0.004742921	1.213132474	0.12323604	0.111036837	-1.089896433	-1.102095637	-0.012199203	9.961963646	0.253060143	0.904371061	0.913792377	0.301419765	0.296333515	0.990507536	-6.249769438	-6.174087556	-7.040391705	-6.536123842	-5.688306948	-7.071297224	0.650307425	0.600635838

PPP4R4	0.010822151	0.178170112	0.154723135	0.167347961	0.143900984	-0.023446977	8	0.101774656	0.816496581	0.816496581	0.816496581	0.816496581	0.816496581	0.816496581	18	124	0	P_222067	P_222067	P_222067	chr14	94245610	94245670	+	PPP4R4	"protein phosphatase 4, regulatory subunit 4 [Source:HGNC Symbol;Acc:HGNC:23788]"	ENSG00000119698	protein_coding	exon	coding exon	1	3014	14:94245610:94245670	ENSG00000119698	PPP4R4	4.715747511	0.010524787	0.129197041	2.127030982	1.847116206	1.997833941	1.717919165	-0.279914776	9.961963646	0.899773004	0.059420141	0.094604441	0.073762107	0.116678376	0.785269006	-6.989259376	-4.23304448	-5.44674758	-5.316092026	-4.928254639	-7.028743037	2.495621657	0.119669826

MAPK3	-0.022694485	0.053581447	0.095680667	0.076275931	0.118375152	0.04209922	8	0.117997494	0.816496581	0.816496581	0.816496581	0.816496581	0.816496581	0.816496581	18	125	0	A_23_P26511	A_23_P26511	A_23_P26511	chr16	30112201	30112345	-	MAPK3;GDPD3	mitogen-activated protein kinase 3 [Source:HGNC Symbol;Acc:HGNC:6877];glycerophosphodiester phosphodiesterase domain containing 3 [Source:HGNC Symbol;Acc:HGNC:28638]	ENSG00000102882	protein_coding	"3'ext;exon,intron;promoter"	multiple non-Ref	0	3015	16:30112201:30112345	ENSG00000102882	MAPK3	8.65178386	0.01338794	-0.240219951	0.567156848	1.012774925	0.8073768	1.252994876	0.445618077	9.961963646	0.815030748	0.5831609	0.335133715	0.438301709	0.238819448	0.665401035	-6.966962402	-6.015305969	-6.514167632	-6.804379624	-5.523216604	-6.963970647	0.637585749	0.607836045

ACO1	0.138920616	0.047399015	0.11641848	-0.091521601	-0.022502136	0.069019464	8	0.041197579	0.816496581	0.816496581	0.816496581	0.816496581	0.816496581	0.816496581	18	127	0	P_267108	P_267108	P_267108	chr9	32418403	32418463	+	ACO1	"aconitase 1, soluble [Source:HGNC Symbol;Acc:HGNC:117]"	ENSG00000122729	protein_coding	exon	coding exon	1	3017	9:32418403:32418463	ENSG00000122729	ACO1	7.712260952	0.003569661	2.847730339	0.971631262	2.386459592	-1.876099077	-0.461270747	1.414828329	9.961963646	0.01737954	0.354234186	0.038277706	0.090212959	0.65451137	0.187607394	-3.770448197	-5.707518589	-4.592065674	-5.502976483	-6.184746611	-6.075569874	3.413847071	0.061189927

CYP27A1	0.018074624	0.002634662	0.095585025	-0.015439962	0.0775104	0.092950362	8	0.120696984	0.816496581	0.816496581	0.816496581	0.816496581	0.816496581	0.816496581	18	129	0	P_239363	P_239363	P_239363	chr2	218814548	218814608	-	CYP27A1;RP11-459I19.1	"cytochrome P450, family 27, subfamily A, polypeptide 1 [Source:HGNC Symbol;Acc:HGNC:2605];"	ENSG00000135929	protein_coding;lincRNA	3'ext;exon;promoter	multiple non-Ref	0	3019	2:218814548:218814608	ENSG00000135929	CYP27A1	4.384446969	0.013905391	0.187725515	0.027363962	0.992759113	-0.160361553	0.805033599	0.965395151	9.961963646	0.854860387	0.978709852	0.344328302	0.875801371	0.439591202	0.357197461	-6.979163862	-6.181692701	-6.53406484	-7.135568167	-5.963086443	-6.584816886	0.437890506	0.730838023

PGPEP1	0.446014855	0.110495079	0.477667369	-0.335519776	0.031652514	0.367172289	8	0.084683566	0.816496581	0.816496581	0.816496581	0.816496581	0.816496581	0.816496581	18	130	0	A_23_P22854	A_23_P22854	A_23_P22854	chr19	18363391	18363451	+	PGPEP1	pyroglutamyl-peptidase I [Source:HGNC Symbol;Acc:HGNC:13568]	ENSG00000130517	protein_coding	3'UTR;exon	multiple Ref	1	3020	19:18363391:18363451	ENSG00000130517	PGPEP1	7.717310045	0.007965634	6.120470076	1.516276464	6.554823915	-4.604193612	0.434353839	5.03854745	9.961963646	0.000114435	0.160519375	6.55E-05	0.000983589	0.673288659	0.000513684	1.38771277	-5.096609058	1.934370033	-0.969238825	-6.197307377	-0.227561329	21.58319788	0.000111588

FAM83B	-0.256943633	-0.085482715	-0.32224731	0.171460918	-0.065303677	-0.236764595	8	0.426876651	0.816496581	0.816496581	0.816496581	0.816496581	0.816496581	0.816496581	18	131	0	P_257445	P_257445	P_257445	chr6	54927571	54927631	+	FAM83B	"family with sequence similarity 83, member B [Source:HGNC Symbol;Acc:HGNC:21357]"	ENSG00000168143	protein_coding	exon	coding exon	1	3021	6:54927571:54927631	ENSG00000168143	FAM83B	3.197450481	0.148542231	-0.816504379	-0.271643279	-1.024023585	0.5448611	-0.207519206	-0.752380306	9.961963646	0.4333027	0.791442606	0.330047305	0.597834263	0.839785117	0.469227073	-6.64630985	-6.143336748	-6.502844134	-6.989649211	-6.273830982	-6.770656052	0.449184623	0.723413115

SAG	0.073510343	0.062781237	0.115971376	-0.010729106	0.042461033	0.053190139	8	0.209065283	0.816496581	0.816496581	0.816496581	0.816496581	0.816496581	0.816496581	18	132	0	A_23_P5853	A_23_P5853	A_23_P5853	chr2	233346972	233347032	+	SAG	S-antigen; retina and pineal gland (arrestin) [Source:HGNC Symbol;Acc:HGNC:10521]	ENSG00000130561	protein_coding	3'UTR;exon	multiple Ref	1	3022	2:233346972:233347032	ENSG00000130561	SAG	3.356822602	0.037306826	0.466122349	0.398090072	0.735363867	-0.068032277	0.269241519	0.337273795	9.961963646	0.651153112	0.698961448	0.479072821	0.947106066	0.79323807	0.742908867	-6.881082589	-6.099228567	-6.759599856	-7.147082543	-6.258272518	-7.009589145	0.184563127	0.904464299

TBC1D12	-0.086013765	0.063121438	-0.063211929	0.149135202	0.022801835	-0.126333367	8	0.060382418	0.816496581	0.816496581	0.816496581	0.816496581	0.816496581	0.816496581	18	133	0	P_209262	P_209262	P_209262	chr10	94510119	94510179	+	TBC1D12	"TBC1 domain family, member 12 [Source:HGNC Symbol;Acc:HGNC:29082]"	ENSG00000108239	protein_coding	exon	coding exon	1	3023	10:94510119:94510179	ENSG00000108239	TBC1D12	6.74495333	0.00513465	-1.470138195	1.078864956	-1.080411631	2.549003151	0.389726564	-2.159276587	9.961963646	0.17238781	0.306076764	0.30542052	0.02899266	0.704940838	0.056279461	-5.931870044	-5.603152043	-6.444573537	-4.41905233	-6.216528651	-4.985216055	2.634054072	0.107579482

MPV17L	0.296488078	-0.06076417	-0.185366308	-0.357252249	-0.481854387	-0.124602138	8	0.229603848	0.816496581	0.816496581	0.816496581	0.816496581	0.816496581	0.816496581	18	134	0	P_118334	P_118334	P_118334	chr16	15396147	15396207	+	MPV17L;RP11-1021N1.1	MPV17 mitochondrial membrane protein-like [Source:HGNC Symbol;Acc:HGNC:26827];Uncharacterized protein [Source:UniProtKB/TrEMBL;Acc:H3BMD7]	ENSG00000156968	protein_coding	exon	coding exon	1	3024	16:15396147:15396207	ENSG00000156968	MPV17L	5.812889644	0.044542054	1.720551769	-0.352620926	-1.075700349	-2.073172695	-2.796252118	-0.723079423	9.961963646	0.116185244	0.731719144	0.30742285	0.065042855	0.018980817	0.48626113	-5.585634235	-6.116960776	-6.449537086	-5.19793778	-3.262532176	-6.79304146	2.813614323	0.093976186

ACAT1	0.136123954	-0.052922465	0.118813023	-0.189046419	-0.017310931	0.171735488	8	0.090727525	0.816496581	0.816496581	0.816496581	0.816496581	0.816496581	0.816496581	18	138	0	A_24_P203678	A_24_P203678	A_24_P203678	chr11	108135155	108135215	+	ACAT1	acetyl-CoA acetyltransferase 1 [Source:HGNC Symbol;Acc:HGNC:93]	ENSG00000075239	protein_coding	exon	coding exon	1	3028	11:108135155:108135215	ENSG00000075239	ACAT1	11.47467787	0.008817014	1.775494837	-0.690279411	1.549704535	-2.465774248	-0.225790302	2.239983946	9.961963646	0.106316491	0.505781684	0.152371734	0.033429464	0.825929427	0.049102705	-5.505623492	-5.936963284	-5.873139122	-4.558448542	-6.269640455	-4.855113717	2.863167286	0.090586819

VAMP4	0.190227511	0.309765979	0.334936226	0.119538468	0.144708715	0.025170247	8	0.060738141	0.816496581	0.816496581	0.816496581	0.816496581	0.816496581	0.816496581	18	139	0	P_104034	P_104034	P_104034	chr1	171703146	171703206	-	VAMP4	vesicle-associated membrane protein 4 [Source:HGNC Symbol;Acc:HGNC:12645]	ENSG00000117533	protein_coding	3'UTR	3'UTR	1	3029	1:171703146:171703206	ENSG00000117533	VAMP4	7.28289008	0.00516925	3.240449012	5.276738649	5.70550366	2.036289637	2.465054648	0.428765011	9.961963646	0.008905649	0.000363899	0.000199666	0.069181734	0.03347061	0.677217718	-3.100084582	0.521695582	0.774950207	-5.256004334	-3.796670359	-6.971868319	13.55067699	0.000752729

CPA6	0.053949587	-0.106779291	-0.00249033	-0.160728878	-0.056439917	0.104288961	8	0.216515894	0.816496581	0.816496581	0.816496581	0.816496581	0.816496581	0.816496581	18	140	0	P_265028	P_265028	P_265028	chr8	67517963	67518023	-	CPA6	carboxypeptidase A6 [Source:HGNC Symbol;Acc:HGNC:17245]	ENSG00000165078	protein_coding	exon	coding exon	1	3030	8:67517963:67518023	ENSG00000165078	CPA6	3.684605273	0.039853183	0.330980378	-0.655090275	-0.015278159	-0.986070654	-0.346258538	0.639812116	9.961963646	0.74751586	0.527246029	0.988111918	0.347441911	0.73635019	0.536730526	-6.938909784	-5.960790477	-7.04699313	-6.642398815	-6.233308081	-6.852248284	0.340085759	0.796958569

CINP	-0.011787999	0.126390172	0.050309619	0.13817817	0.062097617	-0.076080553	8	0.121119458	0.816496581	0.816496581	0.816496581	0.816496581	0.816496581	0.816496581	18	142	0	A_23_P88134	A_23_P88134	A_23_P88134	chr14	102355799	102355859	-	CINP	cyclin-dependent kinase 2 interacting protein [Source:HGNC Symbol;Acc:HGNC:23789]	ENSG00000100865	protein_coding	exon	coding exon	1	3032	14:102355799:102355859	ENSG00000100865	CINP	9.412095931	0.013987432	-0.122072184	1.308850179	0.520987927	1.430922363	0.643060111	-0.787862252	9.961963646	0.905269248	0.219972351	0.613758161	0.183064433	0.534705928	0.44911747	-6.990234746	-5.35165755	-6.900888899	-6.13188855	-6.081326381	-6.742490359	0.846868479	0.499265764

GRXCR2	-0.193208131	-0.486862976	-0.396514171	-0.293654846	-0.203306041	0.090348805	8	0.308641809	0.816496581	0.816496581	0.816496581	0.816496581	0.816496581	0.816496581	18	143	0	P_140749	P_140749	P_140749	chr5	145866571	145866631	-	GRXCR2	"glutaredoxin, cysteine rich 2 [Source:HGNC Symbol;Acc:HGNC:33862]"	ENSG00000204928	protein_coding	exon	coding exon	1	3033	5:145866571:145866631	ENSG00000204928	GRXCR2	3.488720011	0.078705471	-0.843467918	-2.125445236	-1.731019194	-1.281977318	-0.887551275	0.394426042	9.961963646	0.418759092	0.059578843	0.114243167	0.228881028	0.395705114	0.701578374	-6.623478361	-4.23544414	-5.618004308	-6.318134243	-5.893957223	-6.987050487	1.802028007	0.210598367

ROBO2	0.008019409	0.045319731	0.075175033	0.037300322	0.067155624	0.029855302	8	0.1021685	0.816496581	0.816496581	0.816496581	0.816496581	0.816496581	0.816496581	18	144	0	A_24_P180151	A_24_P180151	A_24_P180151	chr3	77646564	77646624	+	ROBO2	"roundabout, axon guidance receptor, homolog 2 (Drosophila) [Source:HGNC Symbol;Acc:HGNC:10250]"	ENSG00000185008	protein_coding	3'UTR;exon	multiple Ref	1	3034	3:77646564:77646624	ENSG00000185008	ROBO2	3.740849807	0.01058929	0.095445309	0.539385828	0.894717298	0.443940519	0.799271989	0.35533147	9.961963646	0.925853778	0.601467178	0.392042677	0.666573041	0.442772583	0.729749613	-6.993391457	-6.031002437	-6.626749938	-7.042893484	-5.967694681	-7.002838517	0.343790879	0.794404885

INF2	-0.248012172	0.162700242	-0.035040033	0.410712414	0.212972139	-0.197740275	8	0.095461831	0.816496581	0.816496581	0.816496581	0.816496581	0.816496581	0.816496581	18	146	0	A_16_P02990058	A_16_P02990058	A_16_P02990058	chr14	104722473	104722524	+	INF2	"inverted formin, FH2 and WH2 domain containing [Source:HGNC Symbol;Acc:HGNC:23791]"	ENSG00000203485	protein_coding	3'UTR	3'UTR	1	3036	14:104722473:104722524	ENSG00000203485	INF2	6.788761515	0.009524889	-3.112351737	2.041756167	-0.439724013	5.154107903	2.672627724	-2.481480179	9.961963646	0.011065204	0.068552908	0.669523008	0.00043413	0.023458894	0.032543745	-3.318880865	-4.361189406	-6.942545456	-0.11593519	-3.462927478	-4.457440908	8.985728316	0.003488699

RECQL5	-0.216010179	-0.108870839	-0.113978946	0.10713934	0.102031233	-0.005108107	8	0.081600851	0.816496581	0.816496581	0.816496581	0.816496581	0.816496581	0.816496581	18	147	0	P_122600	P_122600	P_122600	chr17	75651190	75651250	-	RECQL5	RecQ protein-like 5 [Source:HGNC Symbol;Acc:HGNC:9950]	ENSG00000108469	protein_coding	3'UTR;exon	multiple Ref	1	3037	17:75651190:75651250	ENSG00000108469	RECQL5	6.251619215	0.007553982	-3.043910409	-1.534154929	-1.606135885	1.50975548	1.437774524	-0.071980956	9.961963646	0.012431696	0.156115519	0.139440963	0.162152429	0.181158988	0.944041703	-3.435811581	-5.073527033	-5.795477961	-6.02786547	-5.300902933	-7.068548727	3.091738809	0.076707229

LINC00307	-0.185129933	-0.181837832	-0.156875038	0.003292101	0.028254895	0.024962794	8	0.356329824	0.816496581	0.816496581	0.816496581	0.816496581	0.816496581	0.816496581	18	148	0	A_24_P212624	A_24_P212624	A_24_P212624	chr21	30209159	30209219	-	LINC00307	long intergenic non-protein coding RNA 307 [Source:HGNC Symbol;Acc:HGNC:16727]	ENSG00000227342	lincRNA	exon	non-coding exon	1	3038	21:30209159:30209219	ENSG00000227342	LINC00307	4.325685525	0.104171275	-0.702503163	-0.690010795	-0.595285747	0.012492368	0.107217415	0.094725048	9.961963646	0.49845148	0.505943498	0.564929423	0.990279432	0.916745033	0.926411465	-6.735566344	-5.937149451	-6.856985585	-7.149527721	-6.290574557	-7.066478856	0.22410253	0.877483016

MMP2	0.106018708	-0.129867012	-0.094309161	-0.23588572	-0.200327869	0.035557851	8	0.22226471	0.816496581	0.816496581	0.816496581	0.816496581	0.816496581	0.816496581	18	149	0	P_226409	P_226409	P_226409	chr16	55482976	55483036	+	MMP2	"matrix metallopeptidase 2 (gelatinase A, 72kDa gelatinase, 72kDa type IV collagenase) [Source:HGNC Symbol;Acc:HGNC:7166]"	ENSG00000087245	protein_coding	"5'UTR,exon;exon"	multiple Ref	1	3039	16:55482976:55483036	ENSG00000087245	MMP2	3.956448625	0.041878863	0.634498638	-0.777225489	-0.564419576	-1.411724127	-1.198918214	0.212805913	9.961963646	0.540052213	0.455085975	0.584951878	0.188494615	0.258300797	0.835769857	-6.783029252	-5.873303432	-6.875890567	-6.156670887	-5.58418564	-7.04669489	0.800148949	0.521707341

GSE1	-0.011089998	-0.075621077	-0.086531676	-0.064531079	-0.075441678	-0.010910599	8	0.06453023	0.816496581	0.816496581	0.816496581	0.816496581	0.816496581	0.816496581	18	150	0	P_227188	P_227188	P_227188	chr16	85676071	85676131	-	GSE1;GINS2	Gse1 coiled-coil protein [Source:HGNC Symbol;Acc:HGNC:28979];GINS complex subunit 2 (Psf2 homolog) [Source:HGNC Symbol;Acc:HGNC:24575]	ENSG00000131149	protein_coding	3'ext;3'UTR;exon	multiple non-Ref	0	3040	16:85676071:85676131	ENSG00000131149	GSE1	7.718304325	0.005550724	-0.182306554	-1.243121768	-1.42247921	-1.060815214	-1.240172656	-0.179357442	9.961963646	0.858998568	0.242284447	0.185435868	0.313815116	0.243327341	0.861252546	-6.980254299	-5.427139775	-6.041850558	-6.566748339	-5.537849497	-7.053832952	1.049593456	0.412931898

SCYL3	0.077443773	-0.041463478	0.054066772	-0.118907251	-0.023377	0.095530251	8	0.062943103	0.816496581	0.816496581	0.816496581	0.816496581	0.816496581	0.816496581	18	153	0	P_104001	P_104001	P_104001	chr1	169852988	169853048	-	SCYL3	SCY1-like 3 (S. cerevisiae) [Source:HGNC Symbol;Acc:HGNC:19285]	ENSG00000000457	protein_coding	3'UTR	3'UTR	1	3043	1:169852988:169853048	ENSG00000000457	SCYL3	6.198477959	0.005388253	1.292135762	-0.691810858	0.902094612	-1.983946621	-0.390041151	1.59390547	9.961963646	0.225479023	0.504859738	0.388297156	0.075485448	0.704715543	0.142157901	-6.156590978	-5.935900646	-6.620057483	-5.337672657	-6.216400203	-5.836217293	1.598450453	0.251180923

FLNC	0.019219668	0.026557268	0.174841879	0.0073376	0.155622211	0.148284611	8	0.107199643	0.816496581	0.816496581	0.816496581	0.816496581	0.816496581	0.816496581	19	13	0	P_262824	P_262824	P_262824	chr7	128842844	128842904	+	FLNC	"filamin C, gamma [Source:HGNC Symbol;Acc:HGNC:3756]"	ENSG00000128591	protein_coding	exon	coding exon	1	3073	7:128842844:128842904	ENSG00000128591	FLNC	5.841942386	0.011435197	0.22012516	0.304163574	2.002485016	0.084038414	1.782359856	1.698321442	9.961963646	0.830219069	0.767261768	0.073193275	0.934690692	0.105138747	0.120409375	-6.971983376	-6.133550373	-5.209382208	-7.14575222	-4.837429495	-5.688883929	1.703180272	0.22927687

STAT2	-0.032658279	0.050651896	-0.038264804	0.083310175	-0.005606525	-0.0889167	8	0.080099217	0.816496581	0.816496581	0.816496581	0.816496581	0.816496581	0.816496581	19	15	0	P_215724	P_215724	P_215724	chr12	56355282	56355342	-	STAT2	"signal transducer and activator of transcription 2, 113kDa [Source:HGNC Symbol;Acc:HGNC:11363]"	ENSG00000170581	protein_coding	3'UTR;exon	multiple Ref	1	3075	12:56355282:56355342	ENSG00000170581	STAT2	6.248289572	0.007358989	-0.466261809	0.723156442	-0.546306093	1.189418251	-0.080044283	-1.269462534	9.961963646	0.651056698	0.486215853	0.596877417	0.261851572	0.93778721	0.233130075	-6.881013159	-5.913684819	-6.886545334	-6.426486884	-6.293278545	-6.255801034	0.678577275	0.584917192

MCPH1	-0.092425861	0.062714975	-0.061146247	0.155140836	0.031279614	-0.123861222	8	0.04581952	0.816496581	0.816496581	0.816496581	0.816496581	0.816496581	0.816496581	19	18	0	A_23_P84350	A_23_P84350	A_23_P84350	chr8	6643183	6643243	+	MCPH1	microcephalin 1 [Source:HGNC Symbol;Acc:HGNC:6954]	ENSG00000147316	protein_coding	3'UTR;exon	multiple Ref	1	3078	8:6643183:6643243	ENSG00000147316	MCPH1	7.817496663	0.003892639	-1.814333194	1.231104138	-1.200309782	3.045437332	0.614023412	-2.43141392	9.961963646	0.099809512	0.246557085	0.257783927	0.012399406	0.552962677	0.035450826	-5.448286462	-5.440640599	-6.312644292	-3.573636183	-6.099975526	-4.540767614	3.635275476	0.052666491

SERPINE3	-0.37220159	-0.030202143	0.088933451	0.341999447	0.461135041	0.119135594	8	0.256185014	0.816496581	0.816496581	0.816496581	0.816496581	0.816496581	0.816496581	19	20	0	P_219210	P_219210	P_219210	chr13	51355083	51355143	+	SERPINE3	"serpin peptidase inhibitor, clade E (nexin, plasminogen activator inhibitor type 1), member 3 [Source:HGNC Symbol;Acc:HGNC:24774]"	ENSG00000253309	protein_coding	exon	coding exon	1	3080	13:51355083:51355143	ENSG00000253309	SERPINE3	3.737160448	0.054911764	-1.945320465	-0.157852219	0.4648128	1.787468246	2.410133265	0.622665019	9.961963646	0.080480068	0.877727017	0.652058774	0.104270134	0.036762513	0.547492488	-5.250532506	-6.168968804	-6.930403559	-5.635523571	-3.884138689	-6.863616433	2.236122797	0.146925823

PBX1	0.357998765	-0.25785618	0.182102265	-0.615854946	-0.175896501	0.439958445	8	0.107104173	0.816496581	0.816496581	0.816496581	0.816496581	0.816496581	0.816496581	19	21	0	P_204622	P_204622	P_204622	chr1	164559946	164560006	+	PBX1	pre-B-cell leukemia homeobox 1 [Source:HGNC Symbol;Acc:HGNC:8632]	ENSG00000185630	protein_coding	exon	coding exon	1	3081	1:164559946:164560006	ENSG00000185630	PBX1	6.577688105	0.011418766	4.103151599	-2.955381695	2.087139035	-7.058533295	-2.016012564	5.042520731	9.961963646	0.002150894	0.014457921	0.063537932	3.53E-05	0.071562284	0.000510704	-1.649896129	-2.929000278	-5.076635304	2.509010516	-4.495218304	-0.221507137	18.2067496	0.000228288

SLC3A2	-0.171218876	-0.030030191	-0.043806009	0.141188685	0.127412867	-0.013775818	8	0.089742692	0.816496581	0.816496581	0.816496581	0.816496581	0.816496581	0.816496581	19	22	0	P_212003	P_212003	P_212003	chr11	62888158	62888218	+	SLC3A2	"solute carrier family 3 (amino acid transporter heavy chain), member 2 [Source:HGNC Symbol;Acc:HGNC:11026]"	ENSG00000168003	protein_coding	exon	coding exon	1	3082	11:62888158:62888218	ENSG00000168003	SLC3A2	8.435803763	0.008674285	-2.251543945	-0.394899772	-0.576053045	1.856644173	1.675490901	-0.181153273	9.961963646	0.048150043	0.701239795	0.577360676	0.093139378	0.124891538	0.859879853	-4.766417996	-6.10054066	-6.868875715	-5.532339061	-4.987098074	-7.053480425	1.974074488	0.182127405

PSME4	-0.121464757	-0.056810215	-0.153553935	0.064654542	-0.032089178	-0.09674372	8	0.08208226	0.816496581	0.816496581	0.816496581	0.816496581	0.816496581	0.816496581	19	23	0	P_234908	P_234908	P_234908	chr2	53895582	53895642	-	PSME4	"proteasome (prosome, macropain) activator subunit 4 [Source:HGNC Symbol;Acc:HGNC:20635]"	ENSG00000068878	protein_coding	3'UTR;exon	multiple Ref	1	3083	2:53895582:53895642	ENSG00000068878	PSME4	8.745793388	0.007617262	-1.704497926	-0.797209789	-2.154800862	0.907288137	-0.450302936	-1.357591073	9.961963646	0.119222014	0.443914956	0.056705676	0.38567552	0.662133025	0.204548815	-5.608759224	-5.857728594	-4.969018914	-6.717188382	-6.18995214	-6.148139622	1.842170233	0.203519432

ANXA3	-0.12511073	-0.06560775	0.103751004	0.05950298	0.228861734	0.169358754	8	0.183166114	0.816496581	0.816496581	0.816496581	0.816496581	0.816496581	0.816496581	19	25	0	A_23_P121716	A_23_P121716	A_23_P121716	chr4	78604340	78604399	+	ANXA3	annexin A3 [Source:HGNC Symbol;Acc:HGNC:541]	ENSG00000138772	protein_coding	exon	coding exon	1	3085	4:78604340:78604399	ENSG00000138772	ANXA3	3.421525028	0.029149023	-0.89748704	-0.470639931	0.744262156	0.426847109	1.641749196	1.214902087	9.961963646	0.390633494	0.648033403	0.473908045	0.678568394	0.131790892	0.252413733	-6.575818986	-6.066650606	-6.752786944	-7.050880466	-5.033323557	-6.319846442	0.989072841	0.436912689

STIP1	-0.108981012	-0.086291753	-0.032111299	0.022689259	0.076869713	0.054180455	8	0.184137331	0.816496581	0.816496581	0.816496581	0.816496581	0.816496581	0.816496581	19	26	0	P_108972	P_108972	P_108972	chr11	64204064	64204124	+	STIP1	stress-induced phosphoprotein 1 [Source:HGNC Symbol;Acc:HGNC:11387]	ENSG00000168439	protein_coding	3'UTR;exon	multiple Ref	1	3086	11:64204064:64204124	ENSG00000168439	STIP1	10.0257795	0.029435498	-0.777966278	-0.615997897	-0.229228074	0.161968381	0.548738204	0.386769823	9.961963646	0.454668636	0.551710088	0.823329265	0.874568748	0.595268742	0.707059807	-6.677812969	-5.985921298	-7.018500935	-7.13528567	-6.13897864	-6.990268294	0.252361453	0.857904682

AP4E1	0.005304322	0.015224326	0.070018955	0.009920004	0.064714633	0.054794629	8	0.093652727	0.816496581	0.816496581	0.816496581	0.816496581	0.816496581	0.816496581	19	28	0	P_223489	P_223489	P_223489	chr15	50912089	50912149	+	AP4E1	"adaptor-related protein complex 4, epsilon 1 subunit [Source:HGNC Symbol;Acc:HGNC:573]"	ENSG00000081014	protein_coding	5'UTR;exon	multiple Ref	1	3088	15:50912089:50912149	ENSG00000081014	AP4E1	5.652389901	0.009250141	0.067546258	0.193869513	0.891634917	0.126323254	0.824088659	0.697765405	9.961963646	0.947483303	0.85017412	0.39361511	0.901989244	0.429178041	0.501284793	-6.995871173	-6.162312316	-6.629532337	-7.140893429	-5.947640482	-6.811736083	0.336519492	0.799419237

ZMYND11	0.310524772	-0.005812917	0.230503938	-0.316337689	-0.080020834	0.236316855	8	0.068974598	0.816496581	0.816496581	0.816496581	0.816496581	0.816496581	0.816496581	19	29	0	A_23_P75083	A_23_P75083	A_23_P75083	chr10	248947	249007	+	ZMYND11	"zinc finger, MYND-type containing 11 [Source:HGNC Symbol;Acc:HGNC:16966]"	ENSG00000015171	protein_coding	exon	coding exon	1	3089	10:248947:249007	ENSG00000015171	ZMYND11	8.157899202	0.006027212	4.898731599	-0.091702575	3.636350546	-4.990434174	-1.262381053	3.728053121	9.961963646	0.000631284	0.928752168	0.004592804	0.000551273	0.235562902	0.003948739	-0.383417074	-6.177656195	-2.470856669	-0.365489189	-5.512436568	-2.337814342	12.9375742	0.000901772

ST6GAL2	-0.024548667	0.013904457	-0.034349315	0.038453124	-0.009800649	-0.048253772	8	0.170680851	0.816496581	0.816496581	0.816496581	0.816496581	0.816496581	0.816496581	19	30	0	A_32_P126157	A_32_P126157	A_32_P126157	chr2	106802664	106802724	-	ST6GAL2	"ST6 beta-galactosamide alpha-2,6-sialyltranferase 2 [Source:HGNC Symbol;Acc:HGNC:10861]"	ENSG00000144057	protein_coding	3'UTR	3'UTR	1	3090	2:106802664:106802724	ENSG00000144057	ST6GAL2	3.545627643	0.025601231	-0.18790707	0.106431268	-0.262925855	0.294338338	-0.075018785	-0.369357122	9.961963646	0.854721821	0.917352942	0.797965444	0.774541217	0.941684853	0.719591519	-6.979126779	-6.176119361	-7.009499354	-7.102441313	-6.293692769	-6.997358529	0.057409041	0.980910268

TVP23C	-0.37580343	0.073142484	-0.199036465	0.448945914	0.176766966	-0.272178949	8	0.214559973	0.816496581	0.816496581	0.816496581	0.816496581	0.816496581	0.816496581	19	31	0	P_120640	P_120640	P_120640	chr17	15503041	15503101	-	TVP23C	trans-golgi network vesicle protein 23 homolog C (S. cerevisiae) [Source:HGNC Symbol;Acc:HGNC:30453]	ENSG00000175106	protein_coding	exon	coding exon	1	3091	17:15503041:15503101	ENSG00000175106	TVP23C	6.677384816	0.039176088	-2.325390202	0.452589846	-1.231594518	2.777980047	1.093795683	-1.684184364	9.961963646	0.042474188	0.660540443	0.246381553	0.019584249	0.299786776	0.123167374	-4.646054323	-6.075243856	-6.276505507	-4.031256306	-5.696919881	-5.709138815	3.146523555	0.073764596

MFAP3	-0.01156321	0.061610741	-0.043801178	0.073173951	-0.032237969	-0.10541192	8	0.063164834	0.816496581	0.816496581	0.816496581	0.816496581	0.816496581	0.816496581	19	32	0	A_16_P17355682	A_16_P17355682	A_16_P17355682	chr5	154152383	154152443	+	MFAP3	microfibrillar-associated protein 3 [Source:HGNC Symbol;Acc:HGNC:7034]	ENSG00000037749	protein_coding	intron	intron sense	0	3092	5:154152383:154152443	ENSG00000037749	MFAP3	6.172759006	0.005410708	-0.192529368	1.025829112	-0.729296923	1.21835848	-0.536767555	-1.755126035	9.961963646	0.851195785	0.329236289	0.48261452	0.251155006	0.603208502	0.109882266	-6.978170686	-5.655861826	-6.764202854	-6.393249972	-6.14568496	-5.606579	1.079041853	0.401780429

EPPIN	-0.267578142	-0.260690708	-0.459664628	0.006887434	-0.192086486	-0.198973919	8	0.223869268	0.816496581	0.816496581	0.816496581	0.816496581	0.816496581	0.816496581	19	33	0	A_24_P134302	A_24_P134302	A_24_P134302	chr20	45541655	45541715	-	EPPIN	epididymal peptidase inhibitor [Source:HGNC Symbol;Acc:HGNC:15932]	ENSG00000101448	protein_coding	3'UTR	3'UTR	1	3093	20:45541655:45541715	ENSG00000101448	EPPIN	3.470467531	0.042453728	-1.590517106	-1.549577361	-2.732302603	0.040939745	-1.141785497	-1.182725241	9.961963646	0.142918873	0.15240203	0.021178455	0.968152819	0.280244895	0.264376528	-5.769432815	-5.053486043	-4.012141712	-7.148696558	-5.64642878	-6.356640874	2.516766892	0.117723295

FGFR1	0.0876282	-0.067697426	0.152679363	-0.155325626	0.065051163	0.220376789	8	0.049868864	0.816496581	0.816496581	0.816496581	0.816496581	0.816496581	0.816496581	19	34	0	P_147024	P_147024	P_147024	chr8	38413363	38413423	-	FGFR1	fibroblast growth factor receptor 1 [Source:HGNC Symbol;Acc:HGNC:3688]	ENSG00000077782	protein_coding	3'UTR;exon	multiple Ref	1	3094	8:38413363:38413423	ENSG00000077782	FGFR1	5.878788138	0.004203803	1.655268051	-1.278782247	2.88406326	-2.934050298	1.228795208	4.162845506	9.961963646	0.128986596	0.22995973	0.016332071	0.014994429	0.247384931	0.001955925	-5.678928221	-5.386527601	-3.753884097	-3.764567033	-5.550742469	-1.61464571	6.690148127	0.009424078

PIGP	0.145013598	-0.121819974	-0.010087634	-0.266833572	-0.155101231	0.11173234	8	0.054828619	0.816496581	0.816496581	0.816496581	0.816496581	0.816496581	0.816496581	19	37	0	A_23_P143484	A_23_P143484	A_23_P143484	chr21	37065559	37065619	-	PIGP	"phosphatidylinositol glycan anchor biosynthesis, class P [Source:HGNC Symbol;Acc:HGNC:3046]"	ENSG00000185808	protein_coding	"3'UTR,exon;exon"	multiple Ref	1	3097	21:37065559:37065619	ENSG00000185808	PIGP	10.9007201	0.004620808	2.61273509	-2.19485155	-0.18175064	-4.80758664	-2.79448573	2.01310091	9.961963646	0.025994424	0.052999497	0.859423347	0.000723177	0.019038325	0.07191041	-4.168400254	-4.129867442	-7.029110847	-0.648742149	-3.265400949	-5.216335391	7.775234391	0.005749544

KIRREL2	-0.020816669	0.046930606	0.111728151	0.067747275	0.13254482	0.064797545	8	0.146819077	0.816496581	0.816496581	0.816496581	0.816496581	0.816496581	0.816496581	19	40	0	P_125535	P_125535	P_125535	chr19	35858803	35858863	+	KIRREL2	kin of IRRE like 2 (Drosophila) [Source:HGNC Symbol;Acc:HGNC:18816]	ENSG00000126259	protein_coding	exon	coding exon	1	3100	19:35858803:35858863	ENSG00000126259	KIRREL2	5.297471211	0.0195172	-0.182493797	0.411427232	0.979488824	0.59392103	1.161982621	0.568061591	9.961963646	0.858855504	0.689470881	0.350526045	0.565806599	0.272324995	0.582569583	-6.98021715	-6.093632836	-6.547077203	-6.960067022	-5.624685705	-6.89790446	0.531269081	0.671053477

NEK8	-0.025010853	0.050207395	0.083459296	0.075218248	0.108470149	0.033251901	8	0.076193035	0.816496581	0.816496581	0.816496581	0.816496581	0.816496581	0.816496581	19	41	0	A_24_P99639	A_24_P99639	A_24_P99639	chr17	28741076	28741135	+	NEK8	NIMA-related kinase 8 [Source:HGNC Symbol;Acc:HGNC:13387]	ENSG00000160602	protein_coding	exon	coding exon	1	3101	17:28741076:28741135	ENSG00000160602	NEK8	7.638140496	0.006868719	-0.369603642	0.741951346	1.233338972	1.111554988	1.602942614	0.491387627	9.961963646	0.719413481	0.475245884	0.245757943	0.292435478	0.140145851	0.633797998	-6.924325651	-5.899941422	-6.274470238	-6.512893783	-5.085836914	-6.941051506	1.042444164	0.415689454

TTC9	0.240440673	-0.093705894	0.126550513	-0.334146568	-0.11389016	0.220256407	8	0.098747485	0.816496581	0.816496581	0.816496581	0.816496581	0.816496581	0.816496581	19	43	0	A_23_P14508	A_23_P14508	A_23_P14508	chr14	70672375	70672435	+	TTC9	tetratricopeptide repeat domain 9 [Source:HGNC Symbol;Acc:HGNC:20267]	ENSG00000133985	protein_coding	3'UTR	3'UTR	1	3103	14:70672375:70672435	ENSG00000133985	TTC9	4.69337743	0.010037321	2.93930496	-1.145522496	1.547036718	-4.084827456	-1.392268242	2.692559214	9.961963646	0.014860423	0.278766003	0.153008391	0.002214765	0.194137079	0.022671118	-3.614398422	-5.533986295	-5.876769181	-1.811892783	-5.357557299	-4.102361436	6.369859894	0.011010406

MAEL	0.24287268	0.085059767	0.17150329	-0.157812912	-0.071369389	0.086443523	8	0.115159139	0.816496581	0.816496581	0.816496581	0.816496581	0.816496581	0.816496581	19	44	0	P_204659	P_204659	P_204659	chr1	166992781	166992841	+	MAEL	maelstrom spermatogenic transposon silencer [Source:HGNC Symbol;Acc:HGNC:25929]	ENSG00000143194	protein_coding	exon	coding exon	1	3104	1:166992781:166992841	ENSG00000143194	MAEL	3.623202632	0.012856494	2.623390988	0.918773685	1.852494018	-1.704617303	-0.770896971	0.933720332	9.961963646	0.025524128	0.379922218	0.093774924	0.119199169	0.458661442	0.372526922	-4.150464163	-5.755675867	-5.438680728	-5.756435273	-5.989965126	-6.614931902	2.588315899	0.111407748

MYO19	-0.417042077	0.016095485	-0.276804061	0.433137562	0.140238016	-0.292899546	8	0.07536164	0.816496581	0.816496581	0.816496581	0.816496581	0.816496581	0.816496581	19	45	0	P_228451	P_228451	P_228451	chr17	36503127	36503187	-	MYO19	myosin XIX [Source:HGNC Symbol;Acc:HGNC:26234]	ENSG00000278259	protein_coding	3'UTR;exon	multiple Ref	1	3105	17:36503127:36503187	ENSG00000278259	MYO19	9.270655933	0.006767533	-6.208837687	0.239626306	-4.121002608	6.448463992	2.087835079	-4.360628914	9.961963646	0.000101948	0.815478329	0.002090533	7.49E-05	0.063463805	0.001432571	1.507459121	-6.151907364	-1.662357048	1.722814002	-4.386554523	-1.292596284	20.09109606	0.000151193

MFSD2A	-0.107140459	0.271259406	0.103489198	0.378399865	0.210629657	-0.167770208	8	0.108707766	0.816496581	0.816496581	0.816496581	0.816496581	0.816496581	0.816496581	19	47	0	P_201290	P_201290	P_201290	chr1	39967814	39967874	+	MFSD2A	major facilitator superfamily domain containing 2A [Source:HGNC Symbol;Acc:HGNC:25897]	ENSG00000168389	protein_coding	exon	coding exon	1	3107	1:39967814:39967874	ENSG00000168389	MFSD2A	6.778037611	0.011696683	-1.213298806	3.071843412	1.171950553	4.285142217	2.385249359	-1.899892859	9.961963646	0.252999328	0.011854314	0.268482005	0.001612342	0.038356789	0.086749995	-6.249577783	-2.742552803	-6.344803375	-1.4829689	-3.92362475	-5.390662279	6.657205873	0.009573996

HIKESHI	0.162494019	0.104289738	0.064900893	-0.058204281	-0.097593126	-0.039388845	8	0.108556854	0.816496581	0.816496581	0.816496581	0.816496581	0.816496581	0.816496581	19	48	0	P_109758	P_109758	P_109758	chr11	86302350	86302410	+	C11orf73	chromosome 11 open reading frame 73 [Source:HGNC Symbol;Acc:HGNC:26938]	ENSG00000149196	protein_coding	5'UTR;exon	multiple Ref	1	3108	11:86302350:86302410	ENSG00000149196	HIKESHI	7.942058339	0.011670353	1.842217977	1.182347701	0.735790723	-0.659870275	-1.106427254	-0.446556978	9.961963646	0.095365884	0.264519533	0.478824255	0.52429907	0.294543566	0.664745494	-5.406739705	-5.494430932	-6.759274712	-6.916579384	-5.683791963	-6.963522054	1.198687388	0.359741141

ZNF200	-0.055153938	0.187053681	0.104823086	0.242207619	0.159977024	-0.082230595	8	0.055876631	0.816496581	0.816496581	0.816496581	0.816496581	0.816496581	0.816496581	19	49	0	A_23_P152356	A_23_P152356	A_23_P152356	chr16	3223378	3223438	-	ZNF200	zinc finger protein 200 [Source:HGNC Symbol;Acc:HGNC:12993]	ENSG00000010539	protein_coding	3'UTR	3'UTR	1	3109	16:3223378:3223438	ENSG00000010539	ZNF200	8.78806043	0.004713979	-0.983848734	3.336706925	1.869858511	4.320555659	2.853707245	-1.466848414	9.961963646	0.348480827	0.007571205	0.091142245	0.001525207	0.017202675	0.173262363	-6.494461909	-2.320241877	-5.412556712	-1.425312975	-3.169158571	-6.007901816	7.426738801	0.006703471

NUDT9	-0.030605211	0.005482061	0.10846152	0.036087272	0.139066731	0.10297946	8	0.066544435	0.816496581	0.816496581	0.816496581	0.816496581	0.816496581	0.816496581	19	50	0	A_23_P94860	A_23_P94860	A_23_P94860	chr4	87458140	87458200	+	NUDT9	nudix (nucleoside diphosphate linked moiety X)-type motif 9 [Source:HGNC Symbol;Acc:HGNC:8056]	ENSG00000170502	protein_coding	3'UTR	3'UTR	1	3110	4:87458140:87458200	ENSG00000170502	NUDT9	9.909753266	0.005762739	-0.493772067	0.088445344	1.749874201	0.582217411	2.243646267	1.661428856	9.961963646	0.632171834	0.931275473	0.110819188	0.573360074	0.048798935	0.127726462	-6.866929733	-6.177965268	-5.59058126	-6.967340581	-4.146321655	-5.741544254	1.907827447	0.192526574

PPM1H	-0.00519456	0.015925081	-0.045699627	0.021119641	-0.040505067	-0.061624708	8	0.08416099	0.816496581	0.816496581	0.816496581	0.816496581	0.816496581	0.816496581	19	53	0	P_215944	P_215944	P_215944	chr12	62689699	62689759	-	PPM1H	"protein phosphatase, Mg2+/Mn2+ dependent, 1H [Source:HGNC Symbol;Acc:HGNC:18583]"	ENSG00000111110	protein_coding	exon	coding exon	1	3113	12:62689699:62689759	ENSG00000111110	PPM1H	6.070169425	0.007894777	-0.071601885	0.219511537	-0.629924276	0.291113422	-0.558322392	-0.849435813	9.961963646	0.94433584	0.830684059	0.542921352	0.776935581	0.588951825	0.415585454	-6.995563372	-6.156749289	-6.834657222	-7.103464956	-6.133509179	-6.690915538	0.261354809	0.85164857

CMTM6	-0.055013169	-0.227976449	-0.172735689	-0.17296328	-0.117722519	0.055240761	8	0.081790585	0.816496581	0.816496581	0.816496581	0.816496581	0.816496581	0.816496581	19	54	0	P_134304	P_134304	P_134304	chr3	32483600	32483660	-	CMTM6	CKLF-like MARVEL transmembrane domain containing 6 [Source:HGNC Symbol;Acc:HGNC:19177]	ENSG00000091317	protein_coding	3'UTR	3'UTR	1	3114	3:32483600:32483660	ENSG00000091317	CMTM6	9.140185601	0.007578878	-0.773944551	-3.20725261	-2.430106223	-2.433308059	-1.656161672	0.777146387	9.961963646	0.45693736	0.009420045	0.035530086	0.03533633	0.128803126	0.455130554	-6.681023124	-2.52622406	-4.519816995	-4.612536167	-5.013641964	-6.751117811	4.343115305	0.03349011

FAM172A	0.399751856	0.100680506	0.393377595	-0.299071349	-0.006374261	0.292697089	8	0.106440536	0.816496581	0.816496581	0.816496581	0.816496581	0.816496581	0.816496581	19	57	0	P_253901	P_253901	P_253901	chr5	93776175	93776235	-	FAM172A	"family with sequence similarity 172, member A [Source:HGNC Symbol;Acc:HGNC:25365]"	ENSG00000113391	protein_coding	exon	coding exon	1	3117	5:93776175:93776235	ENSG00000113391	FAM172A	8.068934178	0.011304961	4.604702659	1.159728937	4.531278173	-3.444973723	-0.073424487	3.371549236	9.961963646	0.000982825	0.273199875	0.001099818	0.006313736	0.942921691	0.007140521	-0.841705215	-5.518837822	-0.998313205	-2.889082768	-6.293818575	-2.942676434	11.05355317	0.001642823

ATXN1	0.114652311	-0.078767174	0.182455532	-0.193419485	0.067803221	0.261222706	8	0.076468785	0.816496581	0.816496581	0.816496581	0.816496581	0.816496581	0.816496581	19	58	0	A_32_P178724	A_32_P178724	A_32_P178724	chr6	16418170	16418230	-	ATXN1	ataxin 1 [Source:HGNC Symbol;Acc:HGNC:10548]	ENSG00000124788	protein_coding	intron	intron sense	0	3118	6:16418170:16418230	ENSG00000124788	ATXN1	5.397713439	0.006902525	1.690146844	-1.161146164	2.689667916	-2.851293008	0.999521072	3.85081408	9.961963646	0.121997278	0.272649448	0.022783732	0.017273896	0.341201452	0.00323056	-5.62933152	-5.517318916	-4.084391783	-3.90614517	-5.791124886	-2.131735729	5.89947559	0.013961623

ELOVL1	-0.49921125	0.057556574	-0.372692947	0.556767825	0.126518303	-0.430249522	8	0.115456262	0.816496581	0.816496581	0.816496581	0.816496581	0.816496581	0.816496581	19	61	0	P_101553	P_101553	P_101553	chr1	43367534	43367594	-	ELOVL1	ELOVL fatty acid elongase 1 [Source:HGNC Symbol;Acc:HGNC:14418]	ENSG00000066322	protein_coding	5'UTR;exon	multiple Ref	1	3121	1:43367534:43367594	ENSG00000066322	ELOVL1	10.38643447	0.01291152	-5.380731336	0.620371563	-4.017058146	6.0011029	1.363673191	-4.637429709	9.961963646	0.000313849	0.548941238	0.00246885	0.000133985	0.202689378	0.000935001	0.341224519	-5.98318035	-1.833777453	1.114173035	-5.392540595	-0.850189973	17.47541361	0.00027047

SCP2	0.089775836	0.044417209	0.098724874	-0.045358626	0.008949038	0.054307665	8	0.044773802	0.816496581	0.816496581	0.816496581	0.816496581	0.816496581	0.816496581	19	62	0	P_201764	P_201764	P_201764	chr1	52978298	52978358	+	SCP2	sterol carrier protein 2 [Source:HGNC Symbol;Acc:HGNC:10606]	ENSG00000116171	protein_coding	exon	coding exon	1	3122	1:52978298:52978358	ENSG00000116171	SCP2	9.05751151	0.003816562	1.779790652	0.880563608	1.957203813	-0.899227044	0.177413161	1.076640205	9.961963646	0.105578107	0.399299345	0.078911354	0.389750041	0.862739258	0.307022604	-5.499312635	-5.789082217	-5.279444518	-6.724545619	-6.279971167	-6.472582375	1.628822106	0.244590576

WDHD1	-0.436654812	-0.008337903	-0.290710594	0.428316908	0.145944218	-0.28237269	8	0.04839647	0.816496581	0.816496581	0.816496581	0.816496581	0.816496581	0.816496581	19	63	0	A_23_P25873	NM_007086	A_23_P25873	chr14	54941246	54941306	-	WDHD1	WD repeat and HMG-box DNA binding protein 1 [Source:HGNC Symbol;Acc:HGNC:23170]	ENSG00000198554	protein_coding	3'UTR	3'UTR	1	3123	14:54941246:54941306	ENSG00000198554	WDHD1	9.837747157	0.004087613	-8.364673509	-0.159723054	-5.568927992	8.204950455	2.795745517	-5.409204938	9.961963646	8.15E-06	0.876291271	0.000241057	9.66E-06	0.018997293	0.000301469	4.109035322	-6.168656419	0.57889228	3.859933882	-3.263354955	0.327452323	34.23385606	1.47E-05

DMXL1	0.047076894	0.039324198	-0.013990898	-0.007752695	-0.061067792	-0.053315097	8	0.048595304	0.816496581	0.816496581	0.816496581	0.816496581	0.816496581	0.816496581	19	65	0	P_254442	P_254442	P_254442	chr5	119116276	119116336	+	DMXL1	Dmx-like 1 [Source:HGNC Symbol;Acc:HGNC:2937]	ENSG00000172869	protein_coding	exon	coding exon	1	3125	5:119116276:119116336	ENSG00000172869	DMXL1	7.262288317	0.0041031	0.900113705	0.751881596	-0.267507015	-0.14823211	-1.167620721	-1.019388611	9.961963646	0.389300396	0.469513778	0.794535507	0.885117071	0.270145993	0.33213611	-6.573437243	-5.892553328	-7.008181681	-7.137610209	-5.618564774	-6.531579468	0.645259111	0.603483671

RHOH	-0.011531906	0.331745293	-0.040834088	0.343277198	-0.029302182	-0.37257938	8	0.110904997	0.816496581	0.816496581	0.816496581	0.816496581	0.816496581	0.816496581	19	67	0	A_23_P58132	NM_004310	A_23_P58132	chr4	40244038	40244098	+	RHOH	ras homolog family member H [Source:HGNC Symbol;Acc:HGNC:686]	ENSG00000168421	protein_coding	exon	coding exon	1	3127	4:40244038:40244098	ENSG00000168421	RHOH	6.451633979	0.012084189	-0.128480717	3.696082367	-0.454945871	3.824563085	-0.326465154	-4.151028239	9.961963646	0.900325343	0.004161873	0.658901587	0.003371744	0.750827607	0.001992991	-6.989359931	-1.755331012	-6.935255811	-2.245766126	-6.24030816	-1.634035297	7.641567605	0.006094872

RAPGEF5	0.037586472	0.041657993	0.006809327	0.004071521	-0.030777146	-0.034848667	8	0.061671696	0.816496581	0.816496581	0.816496581	0.816496581	0.816496581	0.816496581	19	68	0	P_144399	P_144399	P_144399	chr7	22122429	22122489	-	RAPGEF5	Rap guanine nucleotide exchange factor (GEF) 5 [Source:HGNC Symbol;Acc:HGNC:16862]	ENSG00000136237	protein_coding	exon	coding exon	1	3128	7:22122429:22122489	ENSG00000136237	RAPGEF5	6.151826021	0.00526102	0.634661598	0.703410744	0.114978019	0.068749147	-0.519683579	-0.588432725	9.961963646	0.539950163	0.497909845	0.910747036	0.946549672	0.6146344	0.569341847	-6.782920812	-5.92778269	-7.039905877	-7.147028945	-6.155014346	-6.885455077	0.255308751	0.855855292

STXBP5L	0.391661738	-0.097098574	0.507080829	-0.488760313	0.115419091	0.604179403	8	0.067913177	0.816496581	0.816496581	0.816496581	0.816496581	0.816496581	0.816496581	19	69	0	P_245772	P_245772	P_245772	chr3	121413263	121413323	+	STXBP5L	syntaxin binding protein 5-like [Source:HGNC Symbol;Acc:HGNC:30757]	ENSG00000145087	protein_coding	exon	coding exon	1	3129	3:121413263:121413323	ENSG00000145087	STXBP5L	8.245309415	0.005910532	6.239409606	-1.546839321	8.078105893	-7.786248927	1.838696287	9.624945214	9.961963646	9.80E-05	0.153055591	1.11E-05	1.53E-05	0.095916835	2.32E-06	1.548629575	-5.057052736	3.773282752	3.384667341	-4.75667722	5.361909823	43.87079762	4.76E-06

HGSNAT	-0.105800001	-0.055962139	-0.137349873	0.049837862	-0.031549873	-0.081387735	8	0.072771689	0.816496581	0.816496581	0.816496581	0.816496581	0.816496581	0.816496581	19	70	0	A_23_P112061	A_23_P112061	A_23_P112061	chr8	43201753	43201813	+	HGSNAT	heparan-alpha-glucosaminide N-acetyltransferase [Source:HGNC Symbol;Acc:HGNC:26527]	ENSG00000165102	protein_coding	3'UTR	3'UTR	1	3130	8:43201753:43201813	ENSG00000165102	HGSNAT	7.794407678	0.006459435	-1.612256136	-0.852791126	-2.093035679	0.75946501	-0.480779543	-1.240244553	9.961963646	0.138098773	0.413808379	0.062912556	0.465166238	0.641057329	0.243301873	-5.739280787	-5.812606099	-5.067308024	-6.843034182	-6.175191587	-6.290353746	1.675593921	0.234825818

CAVIN1	0.666564501	0.181924487	0.379147202	-0.484640014	-0.287417299	0.197222715	8	0.261858962	0.816496581	0.816496581	0.816496581	0.816496581	0.816496581	0.816496581	19	71	0	P_121530	P_121530	P_121530	chr17	42404200	42404260	-	PTRF	polymerase I and transcript release factor [Source:HGNC Symbol;Acc:HGNC:9688]	ENSG00000177469	protein_coding	3'UTR	3'UTR	1	3131	17:42404200:42404260	ENSG00000177469	CAVIN1	7.187845696	0.057272226	3.411267519	0.931032321	1.940356159	-2.480235198	-1.47091136	1.009323838	9.961963646	0.006680256	0.373849246	0.081144019	0.032613102	0.172182827	0.336705874	-2.808952756	-5.74470646	-5.30533384	-4.534302551	-5.258912991	-6.541682533	4.267071794	0.035094678

CCL15	0.215528188	0.036103301	0.168175617	-0.179424886	-0.047352571	0.132072315	8	0.219928765	0.816496581	0.816496581	0.816496581	0.816496581	0.816496581	0.816496581	19	72	0	A_24_P301501	A_24_P301501	A_24_P301501	chr17	36001629	36001689	-	CCL15	chemokine (C-C motif) ligand 15 [Source:HGNC Symbol;Acc:HGNC:10613]	ENSG00000275718	protein_coding	5'UTR	5'UTR	1	3132	17:36001629:36001689	ENSG00000275718	CCL15	3.84789259	0.041049357	1.302856332	0.218242519	1.01661258	-1.084613813	-0.286243753	0.79837006	9.961963646	0.22193412	0.831645908	0.33339188	0.303643011	0.780555749	0.443271974	-6.143622487	-6.157040682	-6.510315833	-6.541735371	-6.25328904	-6.733929325	0.779047153	0.532175047

MBTPS1	-0.050575946	-0.119334085	-0.073348456	-0.068758139	-0.02277251	0.045985629	8	0.071969212	0.816496581	0.816496581	0.816496581	0.816496581	0.816496581	0.816496581	19	74	0	P_227143	P_227143	P_227143	chr16	84056070	84056130	-	MBTPS1	"membrane-bound transcription factor peptidase, site 1 [Source:HGNC Symbol;Acc:HGNC:15456]"	ENSG00000140943	protein_coding	exon	coding exon	1	3134	16:84056070:84056130	ENSG00000140943	MBTPS1	6.910196123	0.006366159	-0.776338098	-1.831771904	-1.125894926	-1.055433806	-0.349556828	0.705876978	9.961963646	0.455586235	0.097008704	0.28660231	0.316150836	0.733948038	0.496439857	-6.679114362	-4.667692578	-6.395783301	-6.572343232	-6.232102541	-6.805811139	1.160020223	0.372773515

RAD51D	-0.230149893	0.06496303	-0.09042969	0.295112923	0.139720203	-0.15539272	8	0.050943326	0.816496581	0.816496581	0.816496581	0.816496581	0.816496581	0.816496581	19	75	0	A_23_P500873	A_23_P500873	A_23_P500873	chr17	35100607	35100667	-	RAD51D	RAD51 paralog D [Source:HGNC Symbol;Acc:HGNC:9823]	ENSG00000185379	protein_coding	3'UTR	3'UTR	1	3135	17:35100607:35100667	ENSG00000185379	RAD51D	7.61527208	0.004290789	-4.303163233	1.214628076	-1.690783836	5.517791309	2.612379397	-2.905411912	9.961963646	0.001567363	0.252513737	0.121872868	0.000258848	0.02601027	0.015746601	-1.323818844	-5.458995554	-5.675988405	0.424858731	-3.560223713	-3.74008733	11.42720843	0.001450069

WDR7	0.02753149	-0.036790999	-0.016014771	-0.064322489	-0.043546261	0.020776229	8	0.079489527	0.816496581	0.816496581	0.816496581	0.816496581	0.816496581	0.816496581	19	76	0	P_231508	P_231508	P_231508	chr18	56696402	56696462	+	WDR7	WD repeat domain 7 [Source:HGNC Symbol;Acc:HGNC:13490]	ENSG00000091157	protein_coding	exon	coding exon	1	3136	18:56696402:56696462	ENSG00000091157	WDR7	6.877097752	0.007280852	0.395170244	-0.528075609	-0.229866271	-0.923245853	-0.625036515	0.298209338	9.961963646	0.701046516	0.609007953	0.822846811	0.377698605	0.545996744	0.771670436	-6.913811683	-6.037182743	-7.018341777	-6.702460471	-6.092995691	-7.02301349	0.303307372	0.822438682

ENTPD6	-0.172059214	0.095235063	-0.073510665	0.267294277	0.098548548	-0.168745729	8	0.105104846	0.816496581	0.816496581	0.816496581	0.816496581	0.816496581	0.816496581	19	78	0	P_130847	P_130847	P_130847	chr20	25224884	25224944	+	ENTPD6	ectonucleoside triphosphate diphosphohydrolase 6 (putative) [Source:HGNC Symbol;Acc:HGNC:3368]	ENSG00000197586	protein_coding	exon	non-coding exon	1	3138	20:25224884:25224944	ENSG00000197586	ENTPD6	7.317766453	0.01107805	-2.002128256	1.108181347	-0.855390289	3.110309603	1.146737967	-1.963571636	9.961963646	0.07323676	0.29382111	0.412435362	0.011103671	0.278286313	0.078082594	-5.162846916	-5.573125489	-6.661632889	-3.462340312	-5.641123941	-5.293148535	3.46882056	0.058929675

SIPA1L2	-0.469929071	0.144424222	-0.26404339	0.614353293	0.20588568	-0.408467613	8	0.131429825	0.816496581	0.816496581	0.816496581	0.816496581	0.816496581	0.816496581	19	79	0	P_206665	P_206665	P_206665	chr1	232432377	232432437	-	SIPA1L2	signal-induced proliferation-associated 1 like 2 [Source:HGNC Symbol;Acc:HGNC:23800]	ENSG00000116991	protein_coding	exon	coding exon	1	3139	1:232432377:232432437	ENSG00000116991	SIPA1L2	6.451897542	0.016078486	-4.538949603	1.394964277	-2.550341565	5.93391388	1.988608038	-3.945305842	9.961963646	0.001086931	0.193346735	0.028926297	0.000146548	0.074902805	0.002771494	-0.945815462	-5.248744412	-4.319207936	1.020339337	-4.536289146	-1.974071794	13.9639275	0.000668821

ITGAV	-0.182700691	0.212528709	-0.139718088	0.395229399	0.042982603	-0.352246796	8	0.058903052	0.816496581	0.816496581	0.816496581	0.816496581	0.816496581	0.816496581	19	80	0	P_129423	P_129423	P_129423	chr2	186677360	186677420	+	ITGAV	"integrin, alpha V [Source:HGNC Symbol;Acc:HGNC:6150]"	ENSG00000138448	protein_coding	3'UTR	3'UTR	1	3140	2:186677360:186677420	ENSG00000138448	ITGAV	7.601652626	0.004992937	-3.166706076	3.683707767	-2.421699205	6.850413843	0.745006871	-6.105406972	9.961963646	0.01008992	0.004247556	0.036043812	4.54E-05	0.473477405	0.000116723	-3.226017712	-1.774572042	-4.533759009	2.246335689	-6.009663748	1.316325378	19.03692611	0.000189591

EFCAB5	0.05750879	0.073115792	0.124181299	0.015607002	0.066672509	0.051065507	8	0.080681628	0.816496581	0.816496581	0.816496581	0.816496581	0.816496581	0.816496581	19	81	0	P_228231	P_228231	P_228231	chr17	30092096	30092156	+	EFCAB5	EF-hand calcium binding domain 5 [Source:HGNC Symbol;Acc:HGNC:24801]	ENSG00000176927	protein_coding	exon	coding exon	1	3141	17:30092096:30092156	ENSG00000176927	EFCAB5	3.515268014	0.007434188	0.816889104	1.038580258	1.76394513	0.221691154	0.947056026	0.725364871	9.961963646	0.433092834	0.323551146	0.108325191	0.829032707	0.366016226	0.484918698	-6.645988628	-5.643381439	-5.570014668	-7.122802711	-5.840568908	-6.791324099	1.05494591	0.410880351

RNGTT	-0.063842195	0.024118075	0.013348078	0.08796027	0.077190273	-0.010769997	8	0.103383761	0.816496581	0.816496581	0.816496581	0.816496581	0.816496581	0.816496581	19	82	0	P_258196	P_258196	P_258196	chr6	88906364	88906424	-	RNGTT	RNA guanylyltransferase and 5'-phosphatase [Source:HGNC Symbol;Acc:HGNC:10073]	ENSG00000111880	protein_coding	exon	coding exon	1	3142	6:88906364:88906424	ENSG00000111880	RNGTT	5.995600253	0.010789893	-0.752739785	0.284367331	0.157382266	1.037107116	0.910122051	-0.126985065	9.961963646	0.469020481	0.781952178	0.87808775	0.324204153	0.384250272	0.901478782	-6.697704471	-6.139637091	-7.033610814	-6.59122618	-5.874048848	-7.062576441	0.432048277	0.734698122

OPALIN	0.183849362	0.102021358	-0.030613776	-0.081828004	-0.214463138	-0.132635134	8	0.369099967	0.816496581	0.816496581	0.816496581	0.816496581	0.816496581	0.816496581	19	83	0	A_24_P132813	A_24_P132813	A_24_P132813	chr10	96343594	96343654	-	OPALIN	oligodendrocytic myelin paranodal and inner loop protein [Source:HGNC Symbol;Acc:HGNC:20707]	ENSG00000197430	protein_coding	3'UTR	3'UTR	1	3143	10:96343594:96343654	ENSG00000197430	OPALIN	3.826230285	0.111610645	0.673992319	0.374010607	-0.112230197	-0.299981711	-0.786222516	-0.486240804	9.961963646	0.515649839	0.716233725	0.912870114	0.770357226	0.450034201	0.637315049	-6.756001658	-6.108878974	-7.040246384	-7.100623353	-5.978024595	-6.943735487	0.258551698	0.853599309

CCNB3	0.112442479	0.173701892	0.263878111	0.061259413	0.151435632	0.090176219	8	0.11218531	0.816496581	0.816496581	0.816496581	0.816496581	0.816496581	0.816496581	19	84	0	A_23_P171107	A_23_P171107	A_23_P171107	chrX	50351691	50351751	+	CCNB3	cyclin B3 [Source:HGNC Symbol;Acc:HGNC:18709]	ENSG00000147082	protein_coding	"3'UTR;3'UTR,exon"	multiple Ref	1	3144	X:50351691:50351751	ENSG00000147082	CCNB3	5.811799163	0.012313562	1.241035463	1.917159865	2.912441068	0.676124402	1.671405605	0.995281204	9.961963646	0.24302185	0.084314905	0.015558516	0.514351487	0.125709249	0.343159578	-6.217348251	-4.544764459	-3.705450702	-6.905216213	-4.992722196	-6.555642175	2.989884921	0.08255628

ATP12A	0.063557998	-0.123140842	0.072114848	-0.18669884	0.00855685	0.19525569	8	0.097583368	0.816496581	0.816496581	0.816496581	0.816496581	0.816496581	0.816496581	19	85	0	P_218313	P_218313	P_218313	chr13	24691051	24691111	+	ATP12A	"ATPase, H+/K+ transporting, nongastric, alpha polypeptide [Source:HGNC Symbol;Acc:HGNC:13816]"	ENSG00000075673	protein_coding	exon	coding exon	1	3145	13:24691051:24691111	ENSG00000075673	ATP12A	4.786625911	0.009853782	0.784177485	-1.519309583	0.889751758	-2.303487068	0.105574273	2.409061341	9.961963646	0.451179237	0.159764693	0.394577947	0.044086078	0.918015698	0.03682983	-6.672826269	-5.092704639	-6.631228203	-4.826921505	-6.290760312	-4.577835611	2.472611545	0.121831212

OR4D9	0.328000089	0.556486189	-0.057974247	0.2284861	-0.385974336	-0.614460435	8	0.642947601	0.816496581	0.816496581	0.816496581	0.816496581	0.816496581	0.816496581	19	87	0	P_108665	P_108665	P_108665	chr11	59515794	59515854	+	OR4D9	"olfactory receptor, family 4, subfamily D, member 9 [Source:HGNC Symbol;Acc:HGNC:15178]"	ENSG00000172742	protein_coding	exon	coding exon	1	3147	11:59515794:59515854	ENSG00000172742	OR4D9	3.30641164	0.334174663	0.694916832	1.178998521	-0.12282704	0.484081688	-0.817743872	-1.301825561	9.961963646	0.50299306	0.265790831	0.904686684	0.638793293	0.432626803	0.222272944	-6.741078912	-5.498067037	-7.038887999	-7.022956022	-5.952818461	-6.216856316	0.747637218	0.548144735

GYPA	-0.258358775	-0.092086631	0.025794098	0.166272145	0.284152874	0.117880729	8	0.265038966	0.816496581	0.816496581	0.816496581	0.816496581	0.816496581	0.816496581	19	90	0	P_251051	P_251051	P_251051	chr4	144119685	144119745	-	GYPA	glycophorin A (MNS blood group) [Source:HGNC Symbol;Acc:HGNC:4702]	ENSG00000170180	protein_coding	3'UTR;exon	multiple Ref	1	3150	4:144119685:144119745	ENSG00000170180	GYPA	4.466552729	0.058617774	-1.306935592	-0.465830104	0.130482215	0.841105489	1.437417808	0.596312319	9.961963646	0.220597422	0.651355172	0.89878218	0.420019943	0.181257763	0.56427009	-6.138667996	-6.068971852	-7.037830739	-6.775926874	-5.301351627	-6.880529927	0.845220119	0.500040552

MOSMO	-0.044328305	-0.034027802	-0.081204642	0.010300503	-0.036876337	-0.04717684	8	0.06732561	0.816496581	0.816496581	0.816496581	0.816496581	0.816496581	0.816496581	19	92	0	A_23_P339053	A_23_P339053	A_23_P339053	chr16	22087198	22087258	+	C16orf52	chromosome 16 open reading frame 52 [Source:HGNC Symbol;Acc:HGNC:27087]	ENSG00000185716	protein_coding	3'UTR;exon	multiple Ref	1	3152	16:22087198:22087258	ENSG00000185716	MOSMO	7.541581703	0.00584672	-0.710020058	-0.545033742	-1.300679668	0.164986316	-0.59065961	-0.755645926	9.961963646	0.493976425	0.597719901	0.222650112	0.872254596	0.567905947	0.467352462	-6.730050845	-6.027870089	-6.194315754	-7.134747514	-6.11440226	-6.76811198	0.573342981	0.645391961

CLUH	0.071719273	0.00935891	0.023115061	-0.062360363	-0.048604212	0.013756151	8	0.071506559	0.816496581	0.816496581	0.816496581	0.816496581	0.816496581	0.816496581	19	93	0	P_120068	P_120068	P_120068	chr17	2695737	2695797	-	CLUH	clustered mitochondria (cluA/CLU1) homolog [Source:HGNC Symbol;Acc:HGNC:29094]	ENSG00000132361	protein_coding	exon	non-coding exon	1	3153	17:2695737:2695797	ENSG00000132361	CLUH	8.566802646	0.006312853	1.105525286	0.144264033	0.356309869	-0.961261252	-0.749215417	0.212045835	9.961963646	0.294915593	0.888168715	0.729039196	0.359171731	0.471048443	0.836346836	-6.369578076	-6.171127792	-6.97822956	-6.666509145	-6.006502337	-7.046870502	0.483377561	0.701248905

ABCA10	0.024467709	-0.039641994	0.070473535	-0.064109703	0.046005826	0.110115529	8	0.119465496	0.816496581	0.816496581	0.816496581	0.816496581	0.816496581	0.816496581	19	94	0	P_229925	P_229925	P_229925	chr17	69222532	69222592	-	ABCA10	"ATP-binding cassette, sub-family A (ABC1), member 10 [Source:HGNC Symbol;Acc:HGNC:30]"	ENSG00000154263	protein_coding	exon	coding exon	1	3154	17:69222532:69222592	ENSG00000154263	ABCA10	3.951280815	0.013667882	0.256323447	-0.41528908	0.738279957	-0.671612528	0.481956509	1.153569037	9.961963646	0.802916628	0.686733262	0.477376349	0.517101355	0.640249926	0.275602478	-6.96262608	-6.091979329	-6.757375253	-6.908395758	-6.174603057	-6.389338295	0.466215168	0.712313229

FAM238A	0.08719612	0.29280752	0.182325883	0.2056114	0.095129762	-0.110481637	8	0.271345718	0.816496581	0.816496581	0.816496581	0.816496581	0.816496581	0.816496581	19	95	0	P_207808	P_207808	P_207808	chr10	26592900	26592960	+	LINC00264	long intergenic non-protein coding RNA 264 [Source:HGNC Symbol;Acc:HGNC:17776]	ENSG00000233261	lincRNA	exon	non-coding exon	1	3155	10:26592900:26592960	ENSG00000233261	FAM238A	3.685696314	0.061334383	0.431211953	1.448024313	0.901658234	1.016812361	0.470446282	-0.546366079	9.961963646	0.675496209	0.178340492	0.388518005	0.333301389	0.648166986	0.596837713	-6.897835255	-5.183190672	-6.620454648	-6.61182604	-6.180299691	-6.910710835	0.774908118	0.534252755

MYH8	0.076952102	0.169740847	0.208504729	0.092788745	0.131552627	0.038763882	8	0.098512651	0.816496581	0.816496581	0.816496581	0.816496581	0.816496581	0.816496581	19	98	0	P_227792	P_227792	P_227792	chr17	10406291	10406351	-	MYH8	"myosin, heavy chain 8, skeletal muscle, perinatal [Source:HGNC Symbol;Acc:HGNC:7578]"	ENSG00000133020	protein_coding	exon	coding exon	1	3158	17:10406291:10406351	ENSG00000133020	MYH8	3.685931343	0.010000121	0.942461209	2.078879707	2.553635483	1.136418498	1.611174274	0.474755776	9.961963646	0.368250171	0.064423849	0.028763614	0.282379615	0.13833519	0.64519733	-6.534229506	-4.305633394	-4.313682953	-6.485789132	-5.074757262	-6.949627518	2.640625123	0.107042321

SMC1A	-0.23901236	-0.137703002	-0.08167034	0.101309358	0.15734202	0.056032662	8	0.168454019	0.816496581	0.816496581	0.816496581	0.816496581	0.816496581	0.816496581	19	99	0	A_24_P942604	A_24_P942604	A_24_P942604	chrX	53374231	53374291	-	SMC1A	structural maintenance of chromosomes 1A [Source:HGNC Symbol;Acc:HGNC:11111]	ENSG00000072501	protein_coding	3'UTR	3'UTR	1	3159	X:53374231:53374291	ENSG00000072501	SMC1A	9.152963776	0.024994767	-1.851575589	-1.066754528	-0.632681956	0.784821061	1.218893633	0.434072572	9.961963646	0.093916112	0.311252506	0.541190624	0.450818686	0.250960571	0.67348615	-5.392728123	-5.615372486	-6.832829429	-6.822837319	-5.561892634	-6.969412698	1.209441438	0.356206725

TOR2A	-0.057920281	-0.120832503	0.024239156	-0.062912222	0.082159437	0.145071659	8	0.105609935	0.816496581	0.816496581	0.816496581	0.816496581	0.816496581	0.816496581	19	101	0	P_149726	P_149726	P_149726	chr9	127734773	127734833	-	TOR2A	"torsin family 2, member A [Source:HGNC Symbol;Acc:HGNC:11996]"	ENSG00000160404	protein_coding	exon	non-coding exon	1	3161	9:127734773:127734833	ENSG00000160404	TOR2A	8.15734733	0.011163519	-0.671391323	-1.400647461	0.280971693	-0.729256138	0.952363016	1.681619154	9.961963646	0.5172364	0.191689647	0.784481269	0.482638384	0.363448217	0.123673885	-6.757827569	-5.241799097	-7.00417907	-6.866324068	-5.835667279	-5.712804108	1.12636292	0.384541472

C1orf146	0.125060219	0.231954534	0.409140982	0.106894315	0.284080762	0.177186447	8	0.387100939	0.816496581	0.816496581	0.816496581	0.816496581	0.816496581	0.816496581	19	102	0	P_202825	P_202825	P_202825	chr1	92244317	92244377	+	C1orf146	chromosome 1 open reading frame 146 [Source:HGNC Symbol;Acc:HGNC:24032]	ENSG00000203910	protein_coding	exon	coding exon	1	3162	1:92244317:92244377	ENSG00000203910	C1orf146	4.390400427	0.122542106	0.437544417	0.811532332	1.431449208	0.373987915	0.993904791	0.619916877	9.961963646	0.671050224	0.436021059	0.182917322	0.716250083	0.343797037	0.549228719	-6.894889464	-5.846353643	-6.030260477	-7.073659696	-5.796521805	-6.865411866	0.735181218	0.554607643

TSPAN8	-0.078975068	0.217592306	-0.23261944	0.296567374	-0.153644373	-0.450211746	8	0.138850369	0.816496581	0.816496581	0.816496581	0.816496581	0.816496581	0.816496581	19	103	0	A_23_P36531	A_23_P36531	A_23_P36531	chr12	71132734	71132794	-	TSPAN8	tetraspanin 8 [Source:HGNC Symbol;Acc:HGNC:11855]	ENSG00000127324	protein_coding	exon	coding exon	1	3163	12:71132734:71132794	ENSG00000127324	TSPAN8	4.827265916	0.017689113	-0.727248113	2.003715842	-2.142094387	2.730963955	-1.414846274	-4.145810229	9.961963646	0.483814261	0.073043437	0.057932631	0.021227091	0.187602275	0.002009592	-6.717210256	-4.417724973	-4.989324839	-4.111292614	-5.329596619	-1.642602053	5.963338652	0.013509785

TNFSF13B	0.310991315	0.389318763	0.406117424	0.078327447	0.095126109	0.016798662	8	0.2135001	0.816496581	0.816496581	0.816496581	0.816496581	0.816496581	0.816496581	19	104	0	P_114134	P_114134	P_114134	chr13	108306946	108307006	+	TNFSF13B	"tumor necrosis factor (ligand) superfamily, member 13b [Source:HGNC Symbol;Acc:HGNC:11929]"	ENSG00000102524	protein_coding	3'UTR;exon	multiple Ref	1	3164	13:108306946:108307006	ENSG00000102524	TNFSF13B	4.789030475	0.03881175	1.933357981	2.420300825	2.524734053	0.486942844	0.591376072	0.104433228	9.961963646	0.082088707	0.036129968	0.030222516	0.636834755	0.567444398	0.918898227	-5.268856049	-3.780403007	-4.362110581	-7.021472021	-6.113967581	-7.065423658	2.761292749	0.09771882

LINC02153	-0.25315511	0.153381996	0.254321551	0.406537106	0.507476661	0.100939554	8	0.164925844	0.816496581	0.816496581	0.816496581	0.816496581	0.816496581	0.816496581	19	105	0	A_32_P127961	A_32_P127961	A_32_P127961	chr8	20974899	20974959	+	RP11-421P23.1	NA	ENSG00000253199	lincRNA	exon	non-coding exon	1	3165	8:20974899:20974959	ENSG00000253199	LINC02153	3.392434626	0.024050196	-1.999277222	1.211325072	2.008489119	3.210602294	4.007766341	0.797164047	9.961963646	0.073585147	0.253721731	0.072465086	0.009366785	0.002506005	0.443940318	-5.1672733	-5.462653543	-5.200041174	-3.290279814	-1.319959767	-6.73491697	6.084012506	0.01270283

H4C8	-0.018166338	-0.078793	-0.315680705	-0.060626662	-0.297514367	-0.236887705	8	0.082136367	0.816496581	0.816496581	0.816496581	0.816496581	0.816496581	0.816496581	19	106	0	P_141846	P_141846	P_141846	chr6	26281054	26281114	-	HIST1H4H	"histone cluster 1, H4h [Source:HGNC Symbol;Acc:HGNC:4788]"	ENSG00000158406	protein_coding	3'ext	3'extended sense	0	3166	6:26281054:26281114	ENSG00000158406	H4C8	9.880891609	0.007624397	-0.254806362	-1.105173616	-4.427829705	-0.850367253	-4.173023343	-3.32265609	9.961963646	0.804055602	0.295060742	0.001290325	0.415091614	0.001924584	0.007752382	-6.963046446	-5.576234749	-1.163752173	-6.767937051	-1.065812789	-3.026172412	8.344883779	0.004516955

CYP39A1	0.324837041	0.127762393	0.253642082	-0.197074648	-0.071194959	0.125879689	8	0.083856926	0.816496581	0.816496581	0.816496581	0.816496581	0.816496581	0.816496581	19	108	0	P_257209	P_257209	P_257209	chr6	46636399	46636459	-	CYP39A1	"cytochrome P450, family 39, subfamily A, polypeptide 1 [Source:HGNC Symbol;Acc:HGNC:17449]"	ENSG00000146233	protein_coding	exon	coding exon	1	3168	6:46636399:46636459	ENSG00000146233	CYP39A1	6.270085126	0.007853751	4.489237905	1.765672333	3.505325759	-2.723565572	-0.983912146	1.739653426	9.961963646	0.001173375	0.10802261	0.005708546	0.021497917	0.348451146	0.112663461	-1.02490992	-4.761042829	-2.692871962	-4.123870792	-5.806062942	-5.629140398	7.828408503	0.005618717

STX7	-0.00645391	-0.000391994	0.027536556	0.006061915	0.033990465	0.02792855	8	0.099645024	0.816496581	0.816496581	0.816496581	0.816496581	0.816496581	0.816496581	19	109	0	A_32_P182968	A_32_P182968	A_32_P182968	chr6	132473849	132473909	+	STX7	syntaxin 7 [Source:HGNC Symbol;Acc:HGNC:11442]	ENSG00000079950	protein_coding	intron	intron antisense	0	3169	6:132473849:132473909	ENSG00000079950	STX7	7.26353217	0.010180317	-0.078340771	-0.00475822	0.33425244	0.073582552	0.412593212	0.33901066	9.961963646	0.939108221	0.996297441	0.745119284	0.942799059	0.688643834	0.741639288	-6.995012293	-6.182075489	-6.986448726	-7.146652923	-6.20693088	-7.008954782	0.068076732	0.975647234

MPPE1	-0.004938452	0.098633256	0.002931723	0.103571708	0.007870175	-0.095701533	8	0.032788165	0.816496581	0.816496581	0.816496581	0.816496581	0.816496581	0.816496581	19	110	0	P_123219	P_123219	P_123219	chr18	11905750	11905810	-	MPPE1	metallophosphoesterase 1 [Source:HGNC Symbol;Acc:HGNC:15988]	ENSG00000154889	protein_coding	5'UTR;exon	multiple Ref	1	3170	18:11905750:11905810	ENSG00000154889	MPPE1	8.360140924	0.003070019	-0.109160602	2.180210778	0.064803445	2.28937138	0.173964047	-2.115407333	9.961963646	0.915242657	0.054326458	0.949612468	0.045156258	0.865378035	0.060592887	-6.991861747	-4.152228451	-7.044827707	-4.850029675	-6.280613724	-5.055225793	2.419311259	0.127016634

GRIN1	-0.002009371	-0.261063523	0.065585213	-0.259054152	0.067594584	0.326648736	8	0.211074002	0.816496581	0.816496581	0.816496581	0.816496581	0.816496581	0.816496581	19	112	0	P_150270	P_150270	P_150270	chr9	137165362	137165422	+	GRIN1	"glutamate receptor, ionotropic, N-methyl D-aspartate 1 [Source:HGNC Symbol;Acc:HGNC:4584]"	ENSG00000176884	protein_coding	3'UTR;exon	multiple Ref	1	3172	9:137165362:137165422	ENSG00000176884	GRIN1	8.098725274	0.037984557	-0.01262706	-1.640545534	0.412143094	-1.627918474	0.424770154	2.052688628	9.961963646	0.990174631	0.132043202	0.688963059	0.134716209	0.680032431	0.067311545	-6.998273312	-4.932927473	-6.955145044	-5.865556184	-6.201604405	-5.154369568	1.650914977	0.239921175

INTS8	0.305142077	-0.085104809	0.270321601	-0.390246886	-0.034820476	0.35542641	8	0.062807051	0.816496581	0.816496581	0.816496581	0.816496581	0.816496581	0.816496581	19	113	0	P_265458	P_265458	P_265458	chr8	94850031	94850091	+	INTS8	integrator complex subunit 8 [Source:HGNC Symbol;Acc:HGNC:26048]	ENSG00000164941	protein_coding	3'UTR;exon	multiple Ref	1	3173	8:94850031:94850091	ENSG00000164941	INTS8	9.184433441	0.005374514	5.097745343	-1.421772597	4.516029707	-6.51951794	-0.581715636	5.937802304	9.961963646	0.000471148	0.185635521	0.001125917	6.85E-05	0.573685151	0.000145788	-0.080150336	-5.215821243	-1.022611038	1.81694109	-6.119787312	1.084619572	21.08382878	0.000123276

GMPS	-0.201227849	-0.023061652	-0.036629021	0.178166197	0.164598828	-0.01356737	8	0.028861438	0.816496581	0.816496581	0.816496581	0.816496581	0.816496581	0.816496581	19	116	0	A_16_P16463164	A_16_P16463164	A_16_P16463164	chr3	155889683	155889743	+	GMPS	guanine monphosphate synthase [Source:HGNC Symbol;Acc:HGNC:4378]	ENSG00000163655	protein_coding	intron	intron sense	0	3176	3:155889683:155889743	ENSG00000163655	GMPS	10.16792746	0.002875614	-4.595876904	-0.526708972	-0.83657642	4.069167932	3.759300484	-0.309867448	9.961963646	0.000996162	0.609922409	0.422444352	0.002270929	0.003751407	0.763046628	-0.855645662	-6.037921162	-6.677842955	-1.837798785	-1.708638564	-7.019176601	8.814308766	0.003733463

FUT5	-0.210267164	-0.040005445	0.148837474	0.170261719	0.359104639	0.18884292	8	0.415540806	0.816496581	0.816496581	0.816496581	0.816496581	0.816496581	0.816496581	19	117	0	P_124313	P_124313	P_124313	chr19	5865825	5865885	-	FUT5	"fucosyltransferase 5 (alpha (1,3) fucosyltransferase) [Source:HGNC Symbol;Acc:HGNC:4016]"	ENSG00000130383	protein_coding	3'UTR	3'UTR	1	3177	19:5865825:5865885	ENSG00000130383	FUT5	4.492586161	0.140873451	-0.686123841	-0.13054197	0.485672312	0.555581872	1.171796153	0.616214281	9.961963646	0.508288533	0.898736116	0.637704108	0.590754441	0.268541202	0.551572914	-6.747400342	-6.173111779	-6.919817948	-6.98338895	-5.614017447	-6.867819247	0.464197034	0.713622393

PTK7	-0.091283233	-0.001159347	0.098601425	0.090123886	0.189884658	0.099760773	8	0.074161937	0.816496581	0.816496581	0.816496581	0.816496581	0.816496581	0.816496581	19	119	0	A_24_P320545	A_24_P320545	A_24_P320545	chr6	43161119	43161179	+	PTK7	protein tyrosine kinase 7 [Source:HGNC Symbol;Acc:HGNC:9618]	ENSG00000112655	protein_coding	3'UTR;exon	multiple Ref	1	3179	6:43161119:43161179	ENSG00000112655	PTK7	6.276813871	0.006623478	-1.37370534	-0.017446812	1.483835536	1.356258528	2.857540876	1.501282348	9.961963646	0.199653427	0.986424646	0.168787415	0.204958112	0.017090191	0.164295975	-6.056047262	-6.18192696	-5.961630874	-6.227004944	-3.162924658	-5.962248596	2.724660731	0.10044411

ABCC5	0.293310745	-0.076877234	0.195886283	-0.370187979	-0.097424462	0.272763517	8	0.038862017	0.816496581	0.816496581	0.816496581	0.816496581	0.816496581	0.816496581	19	120	0	P_247517	P_247517	P_247517	chr3	183965376	183965436	-	ABCC5	"ATP-binding cassette, sub-family C (CFTR/MRP), member 5 [Source:HGNC Symbol;Acc:HGNC:56]"	ENSG00000114770	protein_coding	3'UTR;exon	multiple Ref	1	3180	3:183965376:183965436	ENSG00000114770	ABCC5	6.604480957	0.003419502	6.143164719	-1.610133681	4.102685375	-7.7532984	-2.040479344	5.712819056	9.961963646	0.000111078	0.138562927	0.002152495	1.58E-05	0.068699297	0.000197676	1.418571856	-4.973667216	-1.692477657	3.346405965	-4.458363744	0.767278406	25.67942069	5.27E-05

CREM	0.150653762	-0.0457656	0.167566179	-0.196419362	0.016912418	0.21333178	8	0.087996114	0.816496581	0.816496581	0.816496581	0.816496581	0.816496581	0.816496581	19	121	0	A_24_P922139	A_24_P922139	A_24_P922139	chr10	35167654	35167714	+	CREM	cAMP responsive element modulator [Source:HGNC Symbol;Acc:HGNC:2352]	ENSG00000095794	protein_coding	5'UTR;exon	multiple Ref	1	3181	10:35167654:35167714	ENSG00000095794	CREM	7.033489468	0.008424989	2.01020772	-0.610660911	2.235873992	-2.620868631	0.225666272	2.846534903	9.961963646	0.072257913	0.555099513	0.049445783	0.02563468	0.826023278	0.017415134	-5.15028877	-5.989241685	-4.838482188	-4.297936729	-6.269670081	-3.840578608	4.072602156	0.03963369

ATP5PB	-0.021112438	0.080408526	0.020687306	0.101520964	0.041799745	-0.05972122	8	0.119950464	0.816496581	0.816496581	0.816496581	0.816496581	0.816496581	0.816496581	19	122	0	P_102722	P_102722	P_102722	chr1	111461556	111461616	+	ATP5F1	"ATP synthase, H+ transporting, mitochondrial Fo complex, subunit B1 [Source:HGNC Symbol;Acc:HGNC:840]"	ENSG00000116459	protein_coding	3'UTR	3'UTR	1	3182	1:111461556:111461616	ENSG00000116459	ATP5PB	7.83961707	0.013761124	-0.22042308	0.839500147	0.215984518	1.059923227	0.436407599	-0.623515628	9.961963646	0.829993337	0.420878199	0.833358077	0.31420136	0.671847404	0.546955725	-6.971912105	-5.82363511	-7.021704839	-6.567677278	-6.196374537	-6.86305922	0.417109453	0.744626474

DEGS2	0.852708553	-0.097956625	0.653582825	-0.950665177	-0.199125727	0.75153945	8	0.113491397	0.816496581	0.816496581	0.816496581	0.816496581	0.816496581	0.816496581	19	123	0	P_115765	P_115765	P_115765	chr14	100146637	100146697	-	DEGS2	"delta(4)-desaturase, sphingolipid 2 [Source:HGNC Symbol;Acc:HGNC:20113]"	ENSG00000168350	protein_coding	3'UTR	3'UTR	1	3183	14:100146637:100146697	ENSG00000168350	DEGS2	9.057417013	0.012550265	9.322229743	-1.07091005	7.145289247	-10.39313979	-2.176940495	8.216199297	9.961963646	3.10E-06	0.309469046	3.19E-05	1.15E-06	0.054627197	9.54E-06	5.090787368	-5.611191396	2.682025206	6.050106267	-4.249862223	3.91426932	53.22805495	1.94E-06

ZNF33A	0.127501134	-0.020649543	0.077262598	-0.148150677	-0.050238536	0.097912141	8	0.055171198	0.816496581	0.816496581	0.816496581	0.816496581	0.816496581	0.816496581	19	124	0	A_24_P212129	A_24_P212129	A_24_P212129	chr10	38017292	38017352	+	ZNF33A	zinc finger protein 33A [Source:HGNC Symbol;Acc:HGNC:13096]	ENSG00000189180	protein_coding	5'UTR;exon	multiple Ref	1	3184	10:38017292:38017352	ENSG00000189180	ZNF33A	9.078051626	0.00465107	2.28972444	-0.370834062	1.387517533	-2.660558502	-0.902206907	1.758351595	9.961963646	0.045129186	0.718525128	0.195536417	0.023949162	0.388240338	0.109310416	-4.704333274	-6.110108436	-6.086561987	-4.230789827	-5.88107838	-5.601862449	3.048318332	0.079138608

STK33	0.17471485	-0.054733461	0.183776172	-0.229448311	0.009061322	0.238509634	8	0.027443712	0.816496581	0.816496581	0.816496581	0.816496581	0.816496581	0.816496581	19	125	0	P_210772	P_210772	P_210772	chr11	8474723	8474783	-	STK33	serine/threonine kinase 33 [Source:HGNC Symbol;Acc:HGNC:14568]	ENSG00000130413	protein_coding	exon	coding exon	1	3185	11:8474723:8474783	ENSG00000130413	STK33	8.273578631	0.00281151	4.035576671	-1.264237584	4.244875774	-5.299814255	0.209299103	5.509113358	9.961963646	0.002396515	0.234923095	0.001717839	0.000352098	0.838432739	0.000262004	-1.761112156	-5.403190478	-1.459682135	0.103008816	-6.273438148	0.473655987	15.73088555	0.0004155

PLCL1	0.138081321	0.065087569	0.153649626	-0.072993752	0.015568305	0.088562057	8	0.139187665	0.816496581	0.816496581	0.816496581	0.816496581	0.816496581	0.816496581	19	126	0	P_238546	P_238546	P_238546	chr2	198101300	198101360	+	PLCL1	phospholipase C-like 1 [Source:HGNC Symbol;Acc:HGNC:9063]	ENSG00000115896	protein_coding	3'UTR;exon	multiple Ref	1	3186	2:198101300:198101360	ENSG00000115896	PLCL1	3.768339618	0.017764425	1.268834521	0.598092154	1.411892273	-0.670742367	0.143057752	0.813800119	9.961963646	0.233344993	0.563127976	0.188446465	0.517632706	0.889096787	0.434779788	-6.184514486	-5.996955612	-6.055467976	-6.909006731	-6.285811458	-6.721177611	0.848954422	0.49828706

ADGRA2	0.085052474	-0.090084161	0.33383885	-0.175136635	0.248786376	0.423923011	8	0.204167772	0.816496581	0.816496581	0.816496581	0.816496581	0.816496581	0.816496581	19	127	0	P_264359	P_264359	P_264359	chr8	37837879	37837939	+	GPR124	G protein-coupled receptor 124 [Source:HGNC Symbol;Acc:HGNC:17849]	ENSG00000020181	protein_coding	exon	coding exon	1	3187	8:37837879:37837939	ENSG00000020181	ADGRA2	6.209667953	0.035681594	0.551455484	-0.584079476	2.164513922	-1.13553496	1.613058438	2.748593398	9.961963646	0.593474158	0.57215461	0.055784624	0.28273226	0.137923681	0.020595459	-6.834916373	-6.005380119	-4.953467774	-6.486760235	-5.072216665	-4.007300269	2.796089223	0.095210607

MEX3C	0.264823387	-0.152653069	0.035336549	-0.417476456	-0.229486838	0.187989618	8	0.118566651	0.816496581	0.816496581	0.816496581	0.816496581	0.816496581	0.816496581	19	128	0	A_23_P50137	A_23_P50137	A_23_P50137	chr18	51174898	51174958	-	MEX3C	mex-3 RNA binding family member C [Source:HGNC Symbol;Acc:HGNC:28040]	ENSG00000176624	protein_coding	3'UTR	3'UTR	1	3188	18:51174898:51174958	ENSG00000176624	MEX3C	9.669528794	0.013496065	2.791890489	-1.60933918	0.372534226	-4.401229669	-2.419356263	1.981873406	9.961963646	0.019123137	0.138737031	0.717298348	0.001344757	0.036188278	0.075745953	-3.865394586	-4.974725788	-6.971857419	-1.294547558	-3.869479908	-5.264860245	6.612556062	0.009781778

ZNF91	0.046471335	-0.200238617	-0.034467091	-0.246709952	-0.080938426	0.165771526	8	0.065919401	0.816496581	0.816496581	0.816496581	0.816496581	0.816496581	0.816496581	19	131	0	A_14_P120848	A_14_P120848	A_14_P120848	chr19	23384395	23384454	+	ZNF91;CDC42EP3P1	zinc finger protein 91 [Source:HGNC Symbol;Acc:HGNC:13166];CDC42 effector protein (Rho GTPase binding) 3 pseudogene 1 [Source:HGNC Symbol;Acc:HGNC:49673]	ENSG00000167232	protein_coding;processed_pseudogene	intron;promoter	multiple non-Ref	0	3191	19:23384395:23384454	ENSG00000167232	ZNF91	8.105335975	0.005696251	0.754112632	-3.249368071	-0.559314021	-4.003480703	-1.313426653	2.690054051	9.961963646	0.468232042	0.008772421	0.588300291	0.002523339	0.21848418	0.02276866	-6.696636964	-2.459104796	-6.8789268	-1.946756077	-5.452817465	-4.106604951	6.071541502	0.012783501

RPRD1A	-0.000846473	-0.262799516	-0.121415628	-0.261953043	-0.120569155	0.141383888	8	0.121465385	0.816496581	0.816496581	0.816496581	0.816496581	0.816496581	0.816496581	19	132	0	P_123458	P_123458	P_123458	chr18	36025946	36026006	+	RPRD1A	regulation of nuclear pre-mRNA domain containing 1A [Source:HGNC Symbol;Acc:HGNC:25560]	ENSG00000141425	protein_coding	3'UTR;exon;intron	multiple non-Ref	0	3192	18:36025946:36026006	ENSG00000141425	RPRD1A	6.818945314	0.014054822	-0.008744725	-2.714923021	-1.254317698	-2.706178296	-1.245572973	1.460605322	9.961963646	0.99319545	0.021818677	0.238358281	0.02214811	0.241420396	0.174932554	-6.998318589	-3.313681107	-6.249829649	-4.153412676	-5.53169979	-6.016106533	3.325402879	0.065046276

SPON1	-0.003143496	0.009891514	-0.058851928	0.01303501	-0.055708432	-0.068743443	8	0.193661922	0.816496581	0.816496581	0.816496581	0.816496581	0.816496581	0.816496581	19	137	0	A_32_P133072	A_32_P133072	A_32_P133072	chr11	14267633	14267693	+	SPON1	"spondin 1, extracellular matrix protein [Source:HGNC Symbol;Acc:HGNC:11252]"	ENSG00000262655	protein_coding	3'UTR	3'UTR	1	3197	11:14267633:14267693	ENSG00000262655	SPON1	3.548401185	0.032325196	-0.021413514	0.06738106	-0.400899711	0.088794574	-0.379486197	-0.468280771	9.961963646	0.983338628	0.947611529	0.696957542	0.931004894	0.712290846	0.649661683	-6.998110103	-6.179694524	-6.960055146	-7.145303023	-6.220654983	-6.952889997	0.089483409	0.964178524

LMOD1	0.149404487	0.115415614	0.409773021	-0.033988873	0.260368534	0.294357408	8	0.216853209	0.816496581	0.816496581	0.816496581	0.816496581	0.816496581	0.816496581	19	138	0	P_205768	P_205768	P_205768	chr1	201946079	201946139	-	LMOD1	leiomodin 1 (smooth muscle) [Source:HGNC Symbol;Acc:HGNC:6647]	ENSG00000163431	protein_coding	exon	coding exon	1	3198	1:201946079:201946139	ENSG00000163431	LMOD1	4.771813319	0.039970575	0.915248595	0.707033506	2.510260498	-0.208215089	1.595011903	1.803226992	9.961963646	0.381681478	0.495751436	0.030980418	0.839256315	0.141910194	0.101631871	-6.559598862	-5.925222432	-4.38631938	-7.125956222	-5.096480713	-5.535782008	2.246338036	0.145723505

AVIL	-0.168595568	-0.146244667	-0.103183311	0.022350901	0.065412257	0.043061356	8	0.077529297	0.816496581	0.816496581	0.816496581	0.816496581	0.816496581	0.816496581	19	139	0	P_215870	P_215870	P_215870	chr12	57808232	57808292	-	AVIL	advillin [Source:HGNC Symbol;Acc:HGNC:14188]	ENSG00000135407	protein_coding	3'UTR;exon	multiple Ref	1	3199	12:57808232:57808292	ENSG00000135407	AVIL	5.453606461	0.007033678	-2.462070892	-2.135671426	-1.50682862	0.326399466	0.955242272	0.628842806	9.961963646	0.03364175	0.058562484	0.1628901	0.750875827	0.362060433	0.543600955	-4.420461925	-4.219958575	-5.931012918	-7.091662954	-5.832998469	-6.859553535	2.384647616	0.130528274

HHATL	0.078436112	0.028994557	0.115685444	-0.049441555	0.037249332	0.086690887	8	0.209708859	0.816496581	0.816496581	0.816496581	0.816496581	0.816496581	0.816496581	19	140	0	P_134521	P_134521	P_134521	chr3	42693107	42693167	-	HHATL	hedgehog acyltransferase-like [Source:HGNC Symbol;Acc:HGNC:13242]	ENSG00000010282	protein_coding	exon	coding exon	1	3200	3:42693107:42693167	ENSG00000010282	HHATL	4.826563844	0.03752326	0.495919751	0.183320834	0.731432186	-0.312598917	0.235512435	0.548111352	9.961963646	0.630708896	0.85822367	0.481366144	0.761030948	0.818581902	0.595683143	-6.865797894	-6.164402078	-6.762586729	-7.096436279	-6.267268124	-6.909697976	0.211357702	0.886248276

FAM9B	0.097975713	-0.219783523	0.106509288	-0.317759237	0.008533575	0.326292811	8	0.206676383	0.816496581	0.816496581	0.816496581	0.816496581	0.816496581	0.816496581	19	142	0	P_150433	P_150433	P_150433	chrX	9025009	9025069	-	FAM9B	"family with sequence similarity 9, member B [Source:HGNC Symbol;Acc:HGNC:18404]"	ENSG00000177138	protein_coding	3'UTR	3'UTR	1	3202	X:9025009:9025069	ENSG00000177138	FAM9B	4.124205946	0.03650926	0.628004199	-1.408767237	0.68270266	-2.036771436	0.054698461	2.091469897	9.961963646	0.544128279	0.189343054	0.510358008	0.069126093	0.957460124	0.063078043	-6.78733002	-5.23184404	-6.79840565	-5.255248524	-6.295094141	-5.093201024	1.89512891	0.194597856

TRPS1	0.483648613	-0.13917018	0.150685186	-0.622818793	-0.332963427	0.289855366	8	0.097581762	0.816496581	0.816496581	0.816496581	0.816496581	0.816496581	0.816496581	19	144	0	P_266003	P_266003	P_266003	chr8	115411976	115412036	+	TRPS1	trichorhinophalangeal syndrome I [Source:HGNC Symbol;Acc:HGNC:12340]	ENSG00000104447	protein_coding	3'ext;3'UTR	multiple non-Ref	0	3204	8:115411976:115412036	ENSG00000104447	TRPS1	6.227996324	0.00985353	5.967324507	-1.717101225	1.859174986	-7.684425732	-4.108149522	3.576276211	9.961963646	0.00014015	0.116831977	0.092753775	1.71E-05	0.002133811	0.005073224	1.177551993	-4.828547513	-5.428643368	3.266018543	-1.165140506	-2.594315279	21.78849548	0.000107172

SWI5	0.025018783	0.029807829	0.197327343	0.004789045	0.17230856	0.167519515	8	0.049337281	0.816496581	0.816496581	0.816496581	0.816496581	0.816496581	0.816496581	19	145	0	A_23_P392222	A_23_P392222	A_23_P392222	chr9	128284549	128284609	+	SWI5	SWI5 recombination repair homolog (yeast) [Source:HGNC Symbol;Acc:HGNC:31412]	ENSG00000175854	protein_coding	exon	coding exon	1	3205	9:128284549:128284609	ENSG00000175854	SWI5	12.5717956	0.004161453	0.474995307	0.565917953	3.746367687	0.090922646	3.27137238	3.180449733	9.961963646	0.645032461	0.583971134	0.003831802	0.929356287	0.008452422	0.009857556	-6.876625688	-6.016021928	-2.285412856	-7.145094043	-2.490170562	-3.269423537	5.9010932	0.013949957

CDKN2B-AS1	-0.023604925	-0.105494429	-0.137467752	-0.081889504	-0.113862827	-0.031973323	8	0.145733596	0.816496581	0.816496581	0.816496581	0.816496581	0.816496581	0.816496581	19	146	0	P_267051	P_267051	P_267051	chr9	22120350	22120410	+	CDKN2B-AS1	CDKN2B antisense RNA 1 [Source:HGNC Symbol;Acc:HGNC:34341]	ENSG00000240498	antisense	exon	non-coding exon	1	3206	9:22120350:22120410	ENSG00000240498	CDKN2B-AS1	4.312768739	0.019262182	-0.208302992	-0.930941556	-1.21309195	-0.722638563	-1.004788958	-0.282150395	9.961963646	0.839189523	0.373893955	0.253074961	0.486520351	0.338780086	0.783603065	-6.974734494	-5.744788125	-6.297961993	-6.87131278	-5.786040347	-7.02806201	0.665508431	0.592135497

SENP6	0.01865085	-0.022821947	-0.072586941	-0.041472797	-0.091237791	-0.049764994	8	0.027170216	0.816496581	0.816496581	0.816496581	0.816496581	0.816496581	0.816496581	19	147	0	P_257870	P_257870	P_257870	chr6	75634751	75634811	+	SENP6	SUMO1/sentrin specific peptidase 6 [Source:HGNC Symbol;Acc:HGNC:20944]	ENSG00000112701	protein_coding	3'UTR;exon	multiple Ref	1	3207	6:75634751:75634811	ENSG00000112701	SENP6	9.070073822	0.002799515	0.431720654	-0.528271142	-1.680206632	-0.959991796	-2.111927285	-1.15193549	9.961963646	0.675138566	0.608877174	0.123953594	0.359779588	0.060948282	0.276242414	-6.897600141	-6.037076946	-5.691107593	-6.667729164	-4.349791364	-6.3911519	1.666220655	0.236746286

ZNF574	0.10827382	-0.039743024	0.069460017	-0.148016844	-0.038813803	0.109203041	8	0.095706785	0.816496581	0.816496581	0.816496581	0.816496581	0.816496581	0.816496581	19	148	0	A_23_P89931	A_23_P89931	A_23_P89931	chr19	42081282	42081342	+	ZNF574	zinc finger protein 574 [Source:HGNC Symbol;Acc:HGNC:26166]	ENSG00000105732	protein_coding	"3'UTR,exon;exon"	multiple Ref	1	3208	19:42081282:42081342	ENSG00000105732	ZNF574	9.644600977	0.009562494	1.356074373	-0.4977611	0.869951285	-1.853835473	-0.486123088	1.367712385	9.961963646	0.20501473	0.629455954	0.404801298	0.093569059	0.637395601	0.201462384	-6.078139188	-6.053136794	-6.648873937	-5.536565995	-6.172508588	-6.135454768	1.397862974	0.300163544

TCEAL3	0.014945371	-0.020200441	0.072348152	-0.035145812	0.057402781	0.092548593	8	0.183600454	0.816496581	0.816496581	0.816496581	0.816496581	0.816496581	0.816496581	19	149	0	A_14_P100325	A_14_P100325	A_14_P100325	chrX	103610199	103610259	+	TCEAL3	transcription elongation factor A (SII)-like 3 [Source:HGNC Symbol;Acc:HGNC:28247]	ENSG00000196507	protein_coding	3'ext;intron	multiple non-Ref	0	3209	X:103610199:103610259	ENSG00000196507	TCEAL3	8.287279235	0.029276951	0.106976753	-0.144591763	0.517857359	-0.251568516	0.410880606	0.662449123	9.961963646	0.916931126	0.887916601	0.615862291	0.80648807	0.68985876	0.522713223	-6.992119015	-6.171077995	-6.902617765	-7.115113807	-6.207668024	-6.836805885	0.16191258	0.91954345

STXBP5	-0.061616854	0.008356156	-0.032373923	0.06997301	0.029242931	-0.040730079	8	0.069778449	0.816496581	0.816496581	0.816496581	0.816496581	0.816496581	0.816496581	19	150	0	P_313290	P_313290	P_313290	chr6	147364241	147364301	+	STXBP5	syntaxin binding protein 5 (tomosyn) [Source:HGNC Symbol;Acc:HGNC:19665]	ENSG00000164506	protein_coding	intron	intron sense	0	3210	6:147364241:147364301	ENSG00000164506	STXBP5	5.788790635	0.006116782	-0.964902969	0.1308551	-0.506966724	1.095758069	0.457936245	-0.637821825	9.961963646	0.357432103	0.898494733	0.623210554	0.298967521	0.656824203	0.537973351	-6.512841698	-6.173068707	-6.908555192	-6.529872198	-6.186341919	-6.853582405	0.503730681	0.688291616

ATP2A2	-0.030496376	-0.043361589	0.071559538	-0.012865213	0.102055914	0.114921128	8	0.119359794	0.816496581	0.816496581	0.816496581	0.816496581	0.816496581	0.816496581	19	151	0	P_112757	P_112757	P_112757	chr12	110346561	110346621	+	ATP2A2	"ATPase, Ca++ transporting, cardiac muscle, slow twitch 2 [Source:HGNC Symbol;Acc:HGNC:812]"	ENSG00000174437	protein_coding	3'UTR;exon	multiple Ref	1	3211	12:110346561:110346621	ENSG00000174437	ATP2A2	8.234593866	0.013647609	-0.319716885	-0.454592778	0.750213487	-0.134875892	1.069930372	1.204806265	9.961963646	0.755787106	0.659147079	0.470473575	0.895396199	0.309888798	0.256119465	-6.942866824	-6.074306165	-6.748189482	-7.139673864	-5.721401817	-6.331470265	0.5810324	0.640792296

ME2	0.421784336	0.083689889	0.445710627	-0.338094447	0.023926291	0.362020737	8	0.066639777	0.816496581	0.816496581	0.816496581	0.816496581	0.816496581	0.816496581	19	152	0	P_231425	P_231425	P_231425	chr18	50918153	50918213	+	ME2	"malic enzyme 2, NAD(+)-dependent, mitochondrial [Source:HGNC Symbol;Acc:HGNC:6984]"	ENSG00000082212	protein_coding	exon	coding exon	1	3212	18:50918153:50918213	ENSG00000082212	ME2	9.834934007	0.005772937	6.798884808	1.349025715	7.184560808	-5.449859092	0.385676	5.835535093	9.961963646	4.84E-05	0.207191717	3.04E-05	0.000284691	0.707844369	0.000167284	2.279128923	-5.304195546	2.730132248	0.325287602	-6.218173552	0.941271561	27.26096649	4.06E-05

RDH12	0.007098877	-0.123531474	0.012997819	-0.130630351	0.005898942	0.136529293	8	0.102650726	0.816496581	0.816496581	0.816496581	0.816496581	0.816496581	0.816496581	19	153	0	A_23_P37088	A_23_P37088	A_23_P37088	chr14	67734221	67734281	+	RDH12	retinol dehydrogenase 12 (all-trans/9-cis/11-cis) [Source:HGNC Symbol;Acc:HGNC:19977]	ENSG00000139988	protein_coding	3'UTR	3'UTR	1	3213	14:67734221:67734281	ENSG00000139988	RDH12	7.483802216	0.010668607	0.08417467	-1.464769843	0.154121153	-1.548944514	0.069946483	1.618890996	9.961963646	0.934585077	0.173816901	0.880591756	0.152552871	0.945620435	0.136656726	-6.994495269	-5.162178293	-7.034164221	-5.974854484	-6.294083656	-5.801450137	1.200219846	0.35923512

MIOS	-0.20481279	0.168496887	-0.12971753	0.373309677	0.075095261	-0.298214416	8	0.060026701	0.816496581	0.816496581	0.816496581	0.816496581	0.816496581	0.816496581	19	156	0	P_260443	P_260443	P_260443	chr7	7583312	7583372	+	MIOS	"missing oocyte, meiosis regulator, homolog (Drosophila) [Source:HGNC Symbol;Acc:HGNC:21905]"	ENSG00000164654	protein_coding	exon	coding exon	1	3216	7:7583312:7583372	ENSG00000164654	MIOS	8.47498899	0.005100254	-3.512422928	2.889625825	-2.224581891	6.402048753	1.287841036	-5.114207717	9.961963646	0.005641437	0.016177433	0.050400363	7.95E-05	0.226912183	0.000459999	-2.637164935	-3.034307612	-4.856760774	1.660954276	-5.482908193	-0.112663499	15.73928336	0.000414605

ERCC6L2	0.140533857	-0.028397994	0.071532211	-0.168931851	-0.069001646	0.099930205	8	0.069000476	0.816496581	0.816496581	0.816496581	0.816496581	0.816496581	0.816496581	19	157	0	P_268053	P_268053	P_268053	chr9	95922334	95922394	+	ERCC6L2	excision repair cross-complementation group 6-like 2 [Source:HGNC Symbol;Acc:HGNC:26922]	ENSG00000182150	protein_coding	exon	coding exon	1	3217	9:95922334:95922394	ENSG00000182150	ERCC6L2	7.166260623	0.006030079	2.216486458	-0.447890422	1.128199141	-2.66437688	-1.088287317	1.576089563	9.961963646	0.051095645	0.663814994	0.285673526	0.023792957	0.302095648	0.1461996	-4.823131575	-6.077428357	-6.393269998	-4.224321169	-5.702608004	-5.860812937	2.858930518	0.090870896

ZC3H12D	-0.004825429	-0.117862312	-0.058772825	-0.113036883	-0.053947396	0.059089487	8	0.1775604	0.816496581	0.816496581	0.816496581	0.816496581	0.816496581	0.816496581	19	158	0	A_32_P65533	A_32_P65533	A_32_P65533	chr6	149450107	149450167	-	ZC3H12D	zinc finger CCCH-type containing 12D [Source:HGNC Symbol;Acc:HGNC:21175]	ENSG00000178199	protein_coding	3'UTR	3'UTR	1	3218	6:149450107:149450167	ENSG00000178199	ZC3H12D	4.304660132	0.027525143	-0.035621872	-0.87007321	-0.433867788	-0.834451338	-0.398245917	0.436205421	9.961963646	0.972287544	0.40473779	0.673629955	0.423585171	0.698850231	0.671989223	-6.997667924	-5.798043374	-6.945286434	-6.781620374	-6.213014698	-6.968417713	0.331953597	0.802573286

ELP6	-0.23247857	-0.057089043	-0.140246947	0.175389527	0.092231623	-0.083157904	8	0.116456646	0.816496581	0.816496581	0.816496581	0.816496581	0.816496581	0.816496581	20	12	0	A_24_P910140	A_24_P910140	A_24_P910140	chr3	47509588	47509648	+	ELP6	elongator acetyltransferase complex subunit 6 [Source:HGNC Symbol;Acc:HGNC:25976]	ENSG00000163832	protein_coding	intron	intron antisense	0	3242	3:47509588:47509648	ENSG00000163832	ELP6	8.432578992	0.01309783	-2.487876834	-0.610940218	-1.500857183	1.876936616	0.987019651	-0.889916964	9.961963646	0.032189685	0.554921838	0.164404181	0.090088912	0.346998877	0.394493413	-4.37751369	-5.989068577	-5.938993047	-5.501709146	-5.803104265	-6.655184514	2.350733738	0.134074823

GPHA2	-0.060080449	0.075273425	0.618994726	0.135353874	0.679075174	0.5437213	8	0.351489505	0.816496581	0.816496581	0.816496581	0.816496581	0.816496581	0.816496581	20	13	0	P_109055	P_109055	P_109055	chr11	64934990	64935050	-	GPHA2	glycoprotein hormone alpha 2 [Source:HGNC Symbol;Acc:HGNC:18054]	ENSG00000149735	protein_coding	exon	coding exon	1	3243	11:64934990:64935050	ENSG00000149735	GPHA2	5.470047954	0.101419953	-0.231055917	0.289484694	2.380514747	0.520540612	2.611570665	2.091030053	9.961963646	0.821947686	0.778145777	0.038667711	0.614058588	0.026046336	0.063124604	-6.969305956	-6.138102214	-4.601879901	-7.0034241	-3.561527716	-5.09389727	2.877951539	0.08960372

ATRN	0.052182774	-0.027450395	0.040127471	-0.079633169	-0.012055303	0.067577866	8	0.040058385	0.816496581	0.816496581	0.816496581	0.816496581	0.816496581	0.816496581	20	15	0	P_240031	P_240031	P_240031	chr20	3591174	3591234	+	ATRN	attractin [Source:HGNC Symbol;Acc:HGNC:885]	ENSG00000088812	protein_coding	exon	coding exon	1	3245	20:3591174:3591234	ENSG00000088812	ATRN	7.115777505	0.003495325	1.081008282	-0.568657093	0.831272948	-1.649665375	-0.249735333	1.399930041	9.961963646	0.305167654	0.582180556	0.425295328	0.130142239	0.807866232	0.191898163	-6.395671454	-6.014437017	-6.682355965	-5.834904136	-6.26361954	-6.094652729	1.154422036	0.37470311

PNLIP	-1.282837852	0.385742378	-0.903587758	1.66858023	0.379250094	-1.289330136	8	0.133700964	0.816496581	0.816496581	0.816496581	0.816496581	0.816496581	0.816496581	20	17	0	P_210084	P_210084	P_210084	chr10	116556034	116556094	+	PNLIP	pancreatic lipase [Source:HGNC Symbol;Acc:HGNC:9155]	ENSG00000175535	protein_coding	exon	coding exon	1	3247	10:116556034:116556094	ENSG00000175535	PNLIP	4.604813879	0.016562045	-12.20844687	3.6710137	-8.599218617	15.87946057	3.609228253	-12.27023232	9.961963646	2.58E-07	0.004337363	6.38E-06	2.11E-08	0.004803564	2.46E-07	7.552389063	-1.794327352	4.33885537	9.993307604	-1.94678143	7.607724362	108.7019125	6.53E-08

ELOVL6	-0.134565288	-0.003886981	0.012652791	0.130678307	0.147218079	0.016539772	8	0.081194306	0.816496581	0.816496581	0.816496581	0.816496581	0.816496581	0.816496581	20	18	0	P_138424	P_138424	P_138424	chr4	110049414	110049474	+	ELOVL6	ELOVL fatty acid elongase 6 [Source:HGNC Symbol;Acc:HGNC:15829]	ENSG00000170522	protein_coding	3'ext;3'UTR	multiple non-Ref	0	3248	4:110049414:110049474	ENSG00000170522	ELOVL6	8.111005647	0.007500833	-1.902934838	-0.054967161	0.178927546	1.847967677	2.081862384	0.233894707	9.961963646	0.086316295	0.957251383	0.861581221	0.094472653	0.064102571	0.819803247	-5.315226368	-6.180494885	-7.029665089	-5.54538671	-4.395644944	-7.041574386	1.910006618	0.192173704

SLC35A2	-0.103061722	0.086744115	-0.045339467	0.189805837	0.057722255	-0.132083582	8	0.121046155	0.816496581	0.816496581	0.816496581	0.816496581	0.816496581	0.816496581	20	19	0	A_24_P310616	A_24_P310616	A_24_P310616	chrX	48903889	48903949	-	SLC35A2	"solute carrier family 35 (UDP-galactose transporter), member A2 [Source:HGNC Symbol;Acc:HGNC:11022]"	ENSG00000102100	protein_coding	3'UTR	3'UTR	1	3249	X:48903889:48903949	ENSG00000102100	SLC35A2	7.790204552	0.013973177	-1.067813595	0.898748284	-0.469758296	1.966561879	0.598055299	-1.36850658	9.961963646	0.310797235	0.389992982	0.648641676	0.077696246	0.563151613	0.201221867	-6.40952364	-5.773332876	-6.927934024	-5.364596915	-6.109891715	-6.134456698	1.377748872	0.305646978

TLE3	0.39238268	-0.091358915	0.287850692	-0.483741595	-0.104531987	0.379209607	8	0.029624809	0.816496581	0.816496581	0.816496581	0.816496581	0.816496581	0.816496581	20	20	0	P_116956	P_116956	P_116956	chr15	70052843	70052903	-	TLE3	transducin-like enhancer of split 3 [Source:HGNC Symbol;Acc:HGNC:11839]	ENSG00000140332	protein_coding	exon	non-coding exon	1	3250	15:70052843:70052903	ENSG00000140332	TLE3	8.130492653	0.002911468	8.906343385	-2.073674268	6.533665331	-10.98001765	-2.372678054	8.607339599	9.961963646	4.67E-06	0.064988226	6.73E-05	6.93E-07	0.039187778	6.33E-06	4.676039771	-4.313445443	1.90671172	6.564898902	-3.943536482	4.33676269	54.43142398	1.75E-06

NXPH3	-0.106659941	-0.145319577	-0.10576353	-0.038659636	0.000896411	0.039556047	8	0.195059224	0.816496581	0.816496581	0.816496581	0.816496581	0.816496581	0.816496581	20	22	0	P_121951	P_121951	P_121951	chr17	49579240	49579300	+	NXPH3	neurexophilin 3 [Source:HGNC Symbol;Acc:HGNC:8077]	ENSG00000182575	protein_coding	exon	coding exon	1	3252	17:49579240:49579300	ENSG00000182575	NXPH3	4.412215682	0.032761384	-0.7217152	-0.983305884	-0.715649631	-0.261590684	0.00606557	0.267656253	9.961963646	0.487063558	0.348734998	0.490641287	0.798965947	0.995280152	0.794423848	-6.72136431	-5.696583744	-6.774431751	-7.112319873	-6.296665688	-7.032382457	0.356670672	0.785551647

C2CD2	0.126089464	0.018688821	0.132005638	-0.107400644	0.005916174	0.113316817	8	0.15776122	0.816496581	0.816496581	0.816496581	0.816496581	0.816496581	0.816496581	20	23	0	P_132292	P_132292	P_132292	chr21	41885211	41885271	-	C2CD2	C2 calcium-dependent domain containing 2 [Source:HGNC Symbol;Acc:HGNC:1266]	ENSG00000157617	protein_coding	3'UTR	3'UTR	1	3253	21:41885211:41885271	ENSG00000157617	C2CD2	6.771135963	0.022193589	1.036598698	0.153643346	1.085236371	-0.882955353	0.048637672	0.931593025	9.961963646	0.324429752	0.880958752	0.303380348	0.398066559	0.962169355	0.373573139	-6.441754379	-6.169658201	-6.439472738	-6.739226379	-6.295427168	-6.616924373	0.654287077	0.598399583

VGLL1	0.202463825	-0.09061769	0.00493436	-0.293081514	-0.197529465	0.095552049	8	0.264879483	0.816496581	0.816496581	0.816496581	0.816496581	0.816496581	0.816496581	20	24	0	A_23_P253123	A_23_P253123	A_23_P253123	chrX	136556572	136556632	+	VGLL1	vestigial-like family member 1 [Source:HGNC Symbol;Acc:HGNC:20985]	ENSG00000102243	protein_coding	3'UTR;exon	multiple Ref	1	3254	X:136556572:136556632	ENSG00000102243	VGLL1	5.08012161	0.058549905	1.02477841	-0.458664909	0.024975452	-1.483443319	-0.999802958	0.483640361	9.961963646	0.329708067	0.656318471	0.980567754	0.168889598	0.34107156	0.639095654	-6.453758993	-6.072387538	-7.046779964	-6.062981156	-5.79085336	-6.94508133	0.782547682	0.530424147

ASNS	-0.008341517	0.169015324	0.532963898	0.177356841	0.541305416	0.363948574	8	0.059295052	0.816496581	0.816496581	0.816496581	0.816496581	0.816496581	0.816496581	20	26	0	P_261944	P_261944	P_261944	chr7	97852358	97852418	-	ASNS	asparagine synthetase (glutamine-hydrolyzing) [Source:HGNC Symbol;Acc:HGNC:753]	ENSG00000070669	protein_coding	3'UTR;exon	multiple Ref	1	3256	7:97852358:97852418	ENSG00000070669	ASNS	11.71533185	0.005030146	-0.144045728	2.918645962	9.203502333	3.06269169	9.347548061	6.284856371	9.961963646	0.888336658	0.015394378	3.48E-06	0.012040408	3.02E-06	9.24E-05	-6.987049528	-2.987834525	4.958586187	-3.544037878	4.793439121	1.560005893	38.24997556	8.91E-06

KIF13A	-0.065464216	-0.053914727	0.012383252	0.011549489	0.077847468	0.066297979	8	0.123716183	0.816496581	0.816496581	0.816496581	0.816496581	0.816496581	0.816496581	20	27	0	P_256281	P_256281	P_256281	chr6	17779616	17779676	-	KIF13A	kinesin family member 13A [Source:HGNC Symbol;Acc:HGNC:14566]	ENSG00000137177	protein_coding	exon	coding exon	1	3257	6:17779616:17779676	ENSG00000137177	KIF13A	6.707735016	0.014497991	-0.665879566	-0.548402126	0.125958199	0.11747744	0.791837765	0.674360325	9.961963646	0.520608115	0.595490898	0.902270834	0.908816523	0.446899963	0.515425597	-6.761675542	-6.02598738	-7.038463251	-7.142071041	-5.973597868	-6.828483125	0.309262633	0.818299773

ZNF28	0.111557007	0.095972645	0.041274744	-0.015584362	-0.070282263	-0.054697901	8	0.102151086	0.816496581	0.816496581	0.816496581	0.816496581	0.816496581	0.816496581	20	30	0	P_126673	P_126673	P_126673	chr19	52799326	52799386	-	ZNF28	zinc finger protein 28 [Source:HGNC Symbol;Acc:HGNC:13073]	ENSG00000198538	protein_coding	3'UTR	3'UTR	1	3260	19:52799326:52799386	ENSG00000198538	ZNF28	7.492806208	0.010586433	1.327906993	1.142400193	0.491309538	-0.1855068	-0.836597455	-0.651090655	9.961963646	0.213830474	0.280001211	0.633851288	0.856554155	0.422433071	0.529719364	-6.113025641	-5.537296733	-6.916881146	-7.130826495	-5.937330481	-6.844615936	0.744671226	0.549676943

NEIL1	0.240758575	-0.095345504	0.156623423	-0.336104079	-0.084135151	0.251968928	8	0.136383737	0.816496581	0.816496581	0.816496581	0.816496581	0.816496581	0.816496581	20	33	0	A_23_P129157	A_23_P129157	A_23_P129157	chr15	75355031	75355091	+	NEIL1	nei endonuclease VIII-like 1 (E. coli) [Source:HGNC Symbol;Acc:HGNC:18448]	ENSG00000140398	protein_coding	"3'UTR,exon;exon"	multiple Ref	1	3263	15:75355031:75355091	ENSG00000140398	NEIL1	7.758866657	0.017143919	2.252020512	-0.89184791	1.465032607	-3.143868423	-0.786987905	2.356880518	9.961963646	0.048111155	0.393506313	0.173746714	0.010488515	0.449606141	0.040257039	-4.765645069	-5.779341193	-5.986446694	-3.40476034	-5.977422837	-4.664020688	4.011909004	0.041191303

KMT5B	0.10903094	-0.091490923	-0.004123259	-0.200521863	-0.1131542	0.087367664	8	0.063208124	0.816496581	0.816496581	0.816496581	0.816496581	0.816496581	0.816496581	20	34	0	P_109394	P_109394	P_109394	chr11	68173803	68173863	-	SUV420H1	suppressor of variegation 4-20 homolog 1 (Drosophila) [Source:HGNC Symbol;Acc:HGNC:24283]	ENSG00000110066	protein_coding	3'UTR;exon	multiple Ref	1	3264	11:68173803:68173863	ENSG00000110066	KMT5B	7.423896744	0.005415101	1.814646762	-1.52272105	-0.068625103	-3.337367812	-1.883271865	1.454095947	9.961963646	0.0997585	0.158919576	0.946645945	0.007562791	0.089155726	0.176688744	-5.447821007	-5.088307554	-7.044549361	-3.07304818	-4.691947028	-6.024637173	3.735910587	0.049263547

HEATR6	0.003593327	-0.103560293	-0.056033686	-0.10715362	-0.059627013	0.047526607	8	0.058139779	0.816496581	0.816496581	0.816496581	0.816496581	0.816496581	0.816496581	20	35	0	P_229442	P_229442	P_229442	chr17	60047308	60047368	-	HEATR6	HEAT repeat containing 6 [Source:HGNC Symbol;Acc:HGNC:24076]	ENSG00000068097	protein_coding	3'UTR;exon	multiple Ref	1	3265	17:60047308:60047368	ENSG00000068097	HEATR6	7.741681911	0.004921196	0.062734592	-1.808021392	-0.978271692	-1.870755984	-1.041006284	0.8297497	9.961963646	0.951218733	0.100841462	0.351098566	0.091008057	0.32247791	0.426116574	-6.996213103	-4.701424624	-6.548263473	-5.511055155	-5.75050207	-6.707772506	1.583633181	0.254469822

CRY1	-0.004236435	0.007336687	0.145442254	0.011573122	0.149678688	0.138105567	8	0.103775049	0.816496581	0.816496581	0.816496581	0.816496581	0.816496581	0.816496581	20	36	0	P_112646	P_112646	P_112646	chr12	106992786	106992846	-	CRY1	cryptochrome circadian clock 1 [Source:HGNC Symbol;Acc:HGNC:2384]	ENSG00000008405	protein_coding	exon	coding exon	1	3266	12:106992786:106992846	ENSG00000008405	CRY1	6.820822608	0.010854988	-0.049800244	0.086244408	1.709706483	0.136044653	1.759506728	1.623462075	9.961963646	0.961265913	0.932980945	0.118228969	0.894495864	0.109106288	0.135671049	-6.997007136	-6.178167794	-5.648810549	-7.139501024	-4.86983384	-5.795055482	1.447169267	0.287186233

ETNK1	-0.043121042	0.035009362	-0.04303342	0.078130404	8.76E-05	-0.078042783	8	0.066153238	0.816496581	0.816496581	0.816496581	0.816496581	0.816496581	0.816496581	20	37	0	P_214701	P_214701	P_214701	chr12	22659094	22659154	+	ETNK1	ethanolamine kinase 1 [Source:HGNC Symbol;Acc:HGNC:24649]	ENSG00000139163	protein_coding	exon	coding exon	1	3267	12:22659094:22659154	ENSG00000139163	ETNK1	9.413214766	0.005721052	-0.698227462	0.566880969	-0.69680867	1.265108431	0.001418791	-1.26368964	9.961963646	0.501008036	0.583341271	0.501858143	0.234623469	0.998895978	0.235111782	-6.738679928	-6.015465527	-6.788266698	-6.33832131	-6.296684186	-6.262674088	0.748638468	0.547628445

SYCP2	-0.277910228	-0.102911861	-0.215221275	0.174998368	0.062688953	-0.112309414	8	0.064742736	0.816496581	0.816496581	0.816496581	0.816496581	0.816496581	0.816496581	20	38	0	P_241248	P_241248	P_241248	chr20	59907363	59907423	-	SYCP2	synaptonemal complex protein 2 [Source:HGNC Symbol;Acc:HGNC:11490]	ENSG00000196074	protein_coding	exon	coding exon	1	3268	20:59907363:59907423	ENSG00000196074	SYCP2	5.683254286	0.005572785	-4.559466404	-1.688398351	-3.530975375	2.871068053	1.028491029	-1.842577024	9.961963646	0.001053246	0.122339365	0.005469844	0.016699186	0.328043319	0.095309877	-0.913267813	-4.867980181	-2.649321231	-3.872344082	-5.762896783	-5.477151047	8.133854273	0.004932679

DHX8	-0.010476329	-0.052932689	0.011283352	-0.04245636	0.021759682	0.064216042	8	0.063006068	0.816496581	0.816496581	0.816496581	0.816496581	0.816496581	0.816496581	20	42	0	A_14_P122239	A_14_P122239	A_14_P122239	chr17	43489448	43489508	+	DHX8	DEAH (Asp-Glu-Ala-His) box polypeptide 8 [Source:HGNC Symbol;Acc:HGNC:2749]	ENSG00000067596	protein_coding	exon	coding exon	1	3272	17:43489448:43489508	ENSG00000067596	DHX8	7.096488195	0.005394621	-0.174692513	-0.882651173	0.1881496	-0.70795866	0.362842113	1.070800774	9.961963646	0.864820569	0.398223196	0.854536728	0.495201165	0.724303046	0.309515845	-6.981732833	-5.787288081	-7.027822674	-6.88223305	-6.227133977	-6.478717343	0.437410833	0.731154463

LATS1	0.061290316	-0.019663311	-0.010766782	-0.080953627	-0.072057099	0.008896529	8	0.032535518	0.816496581	0.816496581	0.816496581	0.816496581	0.816496581	0.816496581	20	43	0	P_143815	P_143815	P_143815	chr6	149660587	149660647	+	LATS1	large tumor suppressor kinase 1 [Source:HGNC Symbol;Acc:HGNC:6514]	ENSG00000131023	protein_coding	3'ext;3'UTR	multiple non-Ref	0	3273	6:149660587:149660647	ENSG00000131023	LATS1	6.942544335	0.003056765	1.357708208	-0.435583309	-0.238506662	-1.793291516	-1.59621487	0.197076646	9.961963646	0.204512869	0.672425694	0.816322678	0.103287992	0.141641308	0.847730331	-6.07610016	-6.083045558	-7.016143904	-5.626913159	-5.094868165	-7.050202079	1.315437567	0.323363725

TMEM175	0.078056926	0.092336189	0.135157047	0.014279263	0.057100121	0.042820858	8	0.054765102	0.816496581	0.816496581	0.816496581	0.816496581	0.816496581	0.816496581	20	44	0	A_23_P132932	A_23_P132932	A_23_P132932	chr4	962052	962464	-	TMEM175;DGKQ	"transmembrane protein 175 [Source:HGNC Symbol;Acc:HGNC:28709];diacylglycerol kinase, theta 110kDa [Source:HGNC Symbol;Acc:HGNC:2856]"	ENSG00000127419	protein_coding	"3'ext;exon,intron;exon,intron,promoter"	multiple non-Ref	0	3274	4:962052:962464	ENSG00000127419	TMEM175	8.583755703	0.004615218	1.407216613	1.664644328	2.436622233	0.257427714	1.02940562	0.771977905	9.961963646	0.189789282	0.127073168	0.03513687	0.802087887	0.327634183	0.458049452	-6.013526734	-4.900346313	-4.509002783	-7.113493481	-5.761995062	-6.755241641	2.068387014	0.168429156

HUS1	-0.341393468	0.069002813	-0.349842833	0.410396281	-0.008449366	-0.418845647	8	0.070193497	0.816496581	0.816496581	0.816496581	0.816496581	0.816496581	0.816496581	20	45	0	P_313820	P_313820	P_313820	chr7	47965445	47965505	+	HUS1	HUS1 checkpoint homolog (S. pombe) [Source:HGNC Symbol;Acc:HGNC:5309]	ENSG00000136273	protein_coding	3'ext;intron	multiple non-Ref	0	3275	7:47965445:47965505	ENSG00000136273	HUS1	7.859444924	0.006163436	-5.325855821	1.076467683	-5.457668839	6.402323504	-0.131813019	-6.534136522	9.961963646	0.000339271	0.307096044	0.000281584	7.94E-05	0.89775637	6.72E-05	0.260423624	-5.605579664	0.417177513	1.661321318	-6.287452219	1.891072927	23.83528884	7.29E-05

KIF15	-0.502317623	-0.14860127	-0.406002371	0.353716354	0.096315252	-0.257401102	8	0.072638155	0.816496581	0.816496581	0.816496581	0.816496581	0.816496581	0.816496581	20	47	0	P_244015	P_244015	P_244015	chr3	44805926	44805986	+	KIF15	kinesin family member 15 [Source:HGNC Symbol;Acc:HGNC:17273]	ENSG00000163808	protein_coding	3'UTR;exon	multiple Ref	1	3277	3:44805926:44805986	ENSG00000163808	KIF15	7.646869804	0.006443842	-7.663931399	-2.267230697	-6.1944359	5.396700702	1.469495499	-3.927205203	9.961963646	1.75E-05	0.04688588	0.000103878	0.00030684	0.172558364	0.002853826	3.327041462	-4.018667624	1.454830057	0.246908153	-5.260719506	-2.004205177	24.82565864	6.11E-05

NBEAL1	0.11006921	-0.04241911	0.080197799	-0.15248832	-0.029871411	0.12261691	8	0.084275349	0.816496581	0.816496581	0.816496581	0.816496581	0.816496581	0.816496581	20	48	0	P_238797	P_238797	P_238797	chr2	203077777	203077837	+	NBEAL1	neurobeachin-like 1 [Source:HGNC Symbol;Acc:HGNC:20681]	ENSG00000144426	protein_coding	exon	coding exon	1	3278	2:203077777:203077837	ENSG00000144426	NBEAL1	6.543403618	0.007910246	1.515711308	-0.584133614	1.104366165	-2.099844922	-0.411345143	1.688499779	9.961963646	0.160660338	0.572119583	0.29539422	0.062197696	0.689529125	0.122319497	-5.871303937	-6.005347928	-6.419072996	-5.155692196	-6.20746837	-5.702965788	1.881300418	0.196882922

TRAM2	-0.037207767	0.104121989	-0.102788892	0.141329756	-0.065581125	-0.206910882	8	0.110741856	0.816496581	0.816496581	0.816496581	0.816496581	0.816496581	0.816496581	20	49	0	P_313536	P_313536	P_313536	chr6	52521255	52521315	+	TRAM2	translocation associated membrane protein 2 [Source:HGNC Symbol;Acc:HGNC:16855]	ENSG00000065308	protein_coding	intron	intron antisense	0	3279	6:52521255:52521315	ENSG00000065308	TRAM2	5.407627905	0.012055151	-0.415042825	1.161453327	-1.146582978	1.576496152	-0.731540153	-2.308036305	9.961963646	0.686907688	0.272530267	0.278347443	0.14610624	0.481303075	0.043746465	-6.905169123	-5.516989532	-6.373076532	-5.937090879	-6.019673014	-4.744219527	1.8639016	0.199802314

ACAD8	-0.261511186	0.184769916	-0.004418989	0.446281103	0.257092197	-0.189188906	8	0.091487747	0.816496581	0.816496581	0.816496581	0.816496581	0.816496581	0.816496581	20	50	0	A_32_P100771	A_32_P100771	A_32_P100771	chr11	134254256	134254316	-	ACAD8;THYN1	"acyl-CoA dehydrogenase family, member 8 [Source:HGNC Symbol;Acc:HGNC:87];thymocyte nuclear protein 1 [Source:HGNC Symbol;Acc:HGNC:29560]"	ENSG00000151498	protein_coding	intron;promoter	multiple non-Ref	0	3280	11:134254256:134254316	ENSG00000151498	ACAD8	5.878449792	0.008928257	-3.389632121	2.394934048	-0.05727766	5.784566169	3.332354462	-2.452211707	9.961963646	0.006927042	0.037728405	0.955456598	0.000179249	0.007626856	0.034213398	-2.845763731	-3.820141736	-7.045329259	0.809468703	-2.391493827	-4.50620228	11.30128834	0.001511835

TTC14	-0.106917474	0.089363114	0.040674504	0.196280589	0.147591979	-0.04868861	8	0.121049397	0.816496581	0.816496581	0.816496581	0.816496581	0.816496581	0.816496581	20	51	0	P_247389	P_247389	P_247389	chr3	180606543	180606603	+	TTC14	tetratricopeptide repeat domain 14 [Source:HGNC Symbol;Acc:HGNC:24697]	ENSG00000163728	protein_coding	3'UTR;exon	multiple Ref	1	3281	3:180606543:180606603	ENSG00000163728	TTC14	7.350116063	0.013973807	-1.107737733	0.925862625	0.42141552	2.033600357	1.529153252	-0.504447105	9.961963646	0.294003691	0.376401824	0.682400047	0.06949309	0.157336762	0.624916864	-6.367200884	-5.749347259	-6.950996982	-5.260221737	-5.183669968	-6.934120552	1.498367081	0.274380247

TOP2B	0.127646218	-0.167547915	-0.093068554	-0.295194133	-0.220714771	0.074479362	8	0.060436127	0.816496581	0.816496581	0.816496581	0.816496581	0.816496581	0.816496581	20	52	0	P_243551	P_243551	P_243551	chr3	25643693	25643753	-	TOP2B	topoisomerase (DNA) II beta 180kDa [Source:HGNC Symbol;Acc:HGNC:11990]	ENSG00000077097	protein_coding	exon	coding exon	1	3282	3:25643693:25643753	ENSG00000077097	TOP2B	8.901427639	0.005139861	2.180609447	-2.862259253	-1.589911333	-5.0428687	-3.77052078	1.272347921	9.961963646	0.054289905	0.016952766	0.1430553	0.000510444	0.003683084	0.232144727	-4.880863765	-3.078124082	-5.817975313	-0.285128197	-1.690929659	-6.252357324	9.456937425	0.002908716

FOXP1	-0.177186539	-0.130761541	-0.226941562	0.046424997	-0.049755023	-0.09618002	8	0.061005483	0.816496581	0.816496581	0.816496581	0.816496581	0.816496581	0.816496581	20	54	0	P_244926	P_244926	P_244926	chr3	70977652	70977712	-	FOXP1	forkhead box P1 [Source:HGNC Symbol;Acc:HGNC:3823]	ENSG00000114861	protein_coding	exon	coding exon	1	3284	3:70977652:70977712	ENSG00000114861	FOXP1	7.032523271	0.005195387	-3.010699422	-2.221860082	-3.856121543	0.788839341	-0.845422121	-1.634261462	9.961963646	0.013155608	0.050633101	0.003202774	0.44857179	0.417718064	0.133367486	-3.492535653	-4.088497034	-2.101441956	-6.819582815	-5.929976311	-5.779906535	5.479743864	0.017426187

AK1	0.286135019	0.18533243	0.394176889	-0.100802589	0.10804187	0.208844459	8	0.06838853	0.816496581	0.816496581	0.816496581	0.816496581	0.816496581	0.816496581	20	55	0	P_149744	P_149744	P_149744	chr9	127867843	127867903	+	AK1	adenylate kinase 1 [Source:HGNC Symbol;Acc:HGNC:361]	ENSG00000106992	protein_coding	"3'ext;3'ext,3'UTR;3'UTR"	multiple non-Ref	0	3285	9:127867843:127867903	ENSG00000106992	AK1	9.699482097	0.005962563	4.538372691	2.939548059	6.252019189	-1.598824633	1.713646498	3.312471131	9.961963646	0.001087895	0.014854253	9.64E-05	0.141059531	0.117482761	0.007886499	-0.946731481	-2.954358844	1.532665625	-5.906195013	-4.934215637	-3.04357642	14.13180049	0.00063797

CRBN	0.16335438	-0.049327133	0.122640048	-0.212681513	-0.040714332	0.171967181	8	0.05479186	0.816496581	0.816496581	0.816496581	0.816496581	0.816496581	0.816496581	20	57	0	A_24_P775694	A_24_P775694	A_24_P775694	chr3	3168187	3168247	-	CRBN	cereblon [Source:HGNC Symbol;Acc:HGNC:30185]	ENSG00000113851	protein_coding	intron	intron sense	0	3287	3:3168187:3168247	ENSG00000113851	CRBN	9.034998366	0.004617572	2.944215215	-0.88904684	2.21040107	-3.833262055	-0.733814145	3.09944791	9.961963646	0.014736297	0.394938787	0.05162438	0.003324258	0.479975939	0.011310576	-3.60602167	-5.78176898	-4.879672099	-2.231157072	-6.017994292	-3.408117253	6.530606491	0.010177363

SCAPER	0.190021407	-0.167067149	0.286694013	-0.357088555	0.096672606	0.453761161	8	0.068985468	0.816496581	0.816496581	0.816496581	0.816496581	0.816496581	0.816496581	20	58	0	P_224606	P_224606	P_224606	chr15	76733263	76733323	-	SCAPER	S-phase cyclin A-associated protein in the ER [Source:HGNC Symbol;Acc:HGNC:13081]	ENSG00000140386	protein_coding	3'UTR;exon	multiple Ref	1	3288	15:76733263:76733323	ENSG00000140386	SCAPER	8.176229075	0.006028416	2.997412683	-2.635330401	4.522334005	-5.632743084	1.524921322	7.157664406	9.961963646	0.013457108	0.02500725	0.001115048	0.000220674	0.158376581	3.14E-05	-3.515224013	-3.440506122	-1.012561661	0.591837099	-5.189197442	2.682493212	20.27771557	0.000145408

ITSN2	0.005279995	-0.030036877	0.048322807	-0.035316872	0.043042812	0.078359684	8	0.036087105	0.816496581	0.816496581	0.816496581	0.816496581	0.816496581	0.816496581	20	59	0	P_233924	P_233924	P_233924	chr2	24220968	24221028	-	ITSN2	intersectin 2 [Source:HGNC Symbol;Acc:HGNC:6184]	ENSG00000198399	protein_coding	exon	coding exon	1	3289	2:24220968:24221028	ENSG00000198399	ITSN2	6.349691384	0.003252485	0.113389144	-0.645049063	1.037743739	-0.758438207	0.924354595	1.682792802	9.961963646	0.911974577	0.53346834	0.323921832	0.465753382	0.377148763	0.123441911	-6.99134886	-5.967380912	-6.488896112	-6.843839651	-5.8612791	-5.711127528	0.961218168	0.44844704

CUL3	0.027303834	-0.115416267	0.086566259	-0.1427201	0.059262425	0.201982526	8	0.067382307	0.816496581	0.816496581	0.816496581	0.816496581	0.816496581	0.816496581	20	60	0	P_239479	P_239479	P_239479	chr2	224514611	224514671	-	CUL3	cullin 3 [Source:HGNC Symbol;Acc:HGNC:2553]	ENSG00000036257	protein_coding	3'UTR;exon	multiple Ref	1	3290	2:224514611:224514671	ENSG00000036257	CUL3	9.188445543	0.005852853	0.437104679	-1.847688885	1.385831647	-2.284793565	0.948726968	3.233520532	9.961963646	0.671358534	0.094515785	0.19603505	0.045508716	0.365206263	0.009010575	-6.89509536	-4.64497088	-6.088699181	-4.857514566	-5.839028043	-3.178590163	3.683593933	0.050998864

HSPA8	-0.055880423	-0.125924188	-0.128118875	-0.070043765	-0.072238451	-0.002194687	8	0.071107256	0.816496581	0.816496581	0.816496581	0.816496581	0.816496581	0.816496581	20	61	0	P_110352	P_110352	P_110352	chr11	123062355	123062415	-	HSPA8	heat shock 70kDa protein 8 [Source:HGNC Symbol;Acc:HGNC:5241]	ENSG00000109971	protein_coding	exon	non-coding exon	1	3291	11:123062355:123062415	ENSG00000109971	HSPA8	9.576561593	0.006267122	-0.864512434	-1.948142484	-1.982095945	-1.083630049	-1.11758351	-0.033953461	9.961963646	0.407641299	0.080104909	0.07571795	0.304058426	0.289972062	0.973584942	-6.605213283	-4.499557546	-5.241014725	-6.542778016	-5.672100524	-7.070748763	1.815957507	0.208110143

SELENOO	-0.097154693	-0.0148268	-0.143924196	0.082327893	-0.046769503	-0.129097396	8	0.071349609	0.816496581	0.816496581	0.816496581	0.816496581	0.816496581	0.816496581	20	63	0	A_24_P341853	A_24_P341853	A_24_P341853	chr22	50217421	50217481	+	SELO	selenoprotein O [Source:EntrezGene;Acc:83642]	ENSG00000073169	protein_coding	3'UTR;exon	multiple Ref	1	3293	22:50217421:50217481	ENSG00000073169	SELENOO	9.612728181	0.006294847	-1.499742911	-0.22887611	-2.221707318	1.270866801	-0.721964408	-1.992831208	9.961963646	0.164688061	0.823595371	0.050646195	0.232650096	0.486916914	0.074378601	-5.892658184	-6.154547299	-4.861409014	-6.331451842	-6.026690892	-5.247869329	2.224217956	0.148341693

NAPSA	0.191034357	0.185362654	0.122930995	-0.005671703	-0.068103361	-0.062431659	8	0.145784829	0.816496581	0.816496581	0.816496581	0.816496581	0.816496581	0.816496581	20	64	0	P_126479	P_126479	P_126479	chr19	50358478	50358538	-	NAPSA	napsin A aspartic peptidase [Source:HGNC Symbol;Acc:HGNC:13395]	ENSG00000131400	protein_coding	3'UTR;exon	multiple Ref	1	3294	19:50358478:50358538	ENSG00000131400	NAPSA	5.207571948	0.019274176	1.685268891	1.635234206	1.084473944	-0.050034686	-0.600794947	-0.550760262	9.961963646	0.122953793	0.13316172	0.303702047	0.961083734	0.561396073	0.593933034	-5.636302331	-4.940073131	-6.44027998	-7.148244173	-6.108207608	-6.908154891	1.226166797	0.350786043

DCAF1	-0.281507611	0.023049042	-0.243694304	0.304556654	0.037813307	-0.266743347	8	0.044617489	0.816496581	0.816496581	0.816496581	0.816496581	0.816496581	0.816496581	20	66	0	P_244404	P_244404	P_244404	chr3	51429278	51429338	-	VPRBP	Vpr (HIV-1) binding protein [Source:HGNC Symbol;Acc:HGNC:30911]	ENSG00000145041	protein_coding	exon	coding exon	1	3296	3:51429278:51429338	ENSG00000145041	DCAF1	6.899399371	0.003805341	-5.589062719	0.457616555	-4.8383159	6.046679274	0.750746819	-5.295932455	9.961963646	0.000234416	0.657046142	0.000690699	0.000126126	0.47016657	0.000354054	0.643980613	-6.072883046	-0.515749691	1.17745998	-6.005348034	0.159943796	20.00486456	0.00015396

MCHR2	-0.429929289	-0.159531415	0.143958686	0.270397873	0.573887975	0.303490102	8	0.094325874	0.816496581	0.816496581	0.816496581	0.816496581	0.816496581	0.816496581	20	67	0	P_258428	P_258428	P_258428	chr6	99934421	99934481	-	MCHR2	melanin-concentrating hormone receptor 2 [Source:HGNC Symbol;Acc:HGNC:20867]	ENSG00000152034	protein_coding	exon	coding exon	1	3297	6:99934421:99934481	ENSG00000152034	MCHR2	3.827119125	0.009351758	-5.444976906	-2.020436605	1.823210797	3.424540301	7.268187702	3.843647402	9.961963646	0.000286652	0.07103638	0.098374512	0.006533371	2.76E-05	0.003268479	0.435264169	-4.392924899	-5.482467645	-2.923976465	2.903266351	-2.143728894	19.39694821	0.000175286

TLR4	0.038168246	0.149810702	0.087342433	0.111642455	0.049174187	-0.062468269	8	0.221439142	0.816496581	0.816496581	0.816496581	0.816496581	0.816496581	0.816496581	20	68	0	P_149514	P_149514	P_149514	chr9	117704261	117704321	+	TLR4	toll-like receptor 4 [Source:HGNC Symbol;Acc:HGNC:11850]	ENSG00000136869	protein_coding	promoter	promoter sense	0	3298	9:117704261:117704321	ENSG00000136869	TLR4	4.046079679	0.041584698	0.229235069	0.899749657	0.524570829	0.670514588	0.295335761	-0.375178828	9.961963646	0.823323977	0.389484969	0.611354514	0.51777185	0.773801173	0.715391769	-6.969760873	-5.77245771	-6.898898128	-6.909166544	-6.250501034	-6.995023314	0.302474149	0.823018064

PCCA	-0.062950284	0.150563667	0.091656581	0.213513952	0.154606865	-0.058907086	8	0.05569231	0.816496581	0.816496581	0.816496581	0.816496581	0.816496581	0.816496581	20	69	0	P_114083	P_114083	P_114083	chr13	100530223	100530283	+	PCCA	"propionyl CoA carboxylase, alpha polypeptide [Source:HGNC Symbol;Acc:HGNC:8653]"	ENSG00000175198	protein_coding	3'UTR	3'UTR	1	3299	13:100530223:100530283	ENSG00000175198	PCCA	8.008934433	0.004697465	-1.124893907	2.69050654	1.637862804	3.815400447	2.762756711	-1.052643736	9.961963646	0.287006534	0.02275101	0.132607106	0.00342253	0.020101705	0.317367005	-6.348642043	-3.352628444	-5.751101385	-2.261161311	-3.316908719	-6.497623968	5.74749506	0.015111667

PTBP1	-0.217829998	-0.121102628	-0.17756168	0.09672737	0.040268318	-0.056459052	8	0.038011516	0.816496581	0.816496581	0.816496581	0.816496581	0.816496581	0.816496581	20	71	0	P_231911	P_231911	P_231911	chr19	805088	805148	+	PTBP1	polypyrimidine tract binding protein 1 [Source:HGNC Symbol;Acc:HGNC:9583]	ENSG00000011304	protein_coding	exon	coding exon	1	3301	19:805088:805148	ENSG00000011304	PTBP1	8.8364639	0.003366998	-4.597713571	-2.556099719	-3.747774654	2.041613852	0.849938917	-1.191674934	9.961963646	0.000993371	0.028642502	0.003822969	0.06856921	0.415318663	0.261004585	-0.852743744	-3.566291444	-2.283047709	-5.24764807	-5.926186453	-6.34648091	8.004850523	0.005209433

CNTN3	-0.184660548	0.11325832	-0.163711418	0.297918868	0.02094913	-0.276969737	8	0.076046005	0.816496581	0.816496581	0.816496581	0.816496581	0.816496581	0.816496581	20	72	0	P_244971	P_244971	P_244971	chr3	74336582	74336642	-	CNTN3	contactin 3 (plasmacytoma associated) [Source:HGNC Symbol;Acc:HGNC:2173]	ENSG00000113805	protein_coding	exon	coding exon	1	3302	3:74336582:74336642	ENSG00000113805	CNTN3	5.341357917	0.006850744	-2.732441496	1.675895235	-2.422455021	4.408336731	0.309986475	-4.098350256	9.961963646	0.021173415	0.124810867	0.03599733	0.00132998	0.762958754	0.00216744	-3.966248562	-4.885047026	-4.532506073	-1.283066718	-6.245828483	-1.720662942	8.744858145	0.003838457

HAT1	-0.373083797	0.042264708	-0.242356926	0.415348504	0.130726871	-0.284621633	8	0.064854787	0.816496581	0.816496581	0.816496581	0.816496581	0.816496581	0.816496581	20	73	0	P_237551	P_237551	P_237551	chr2	171952941	171953001	+	HAT1	histone acetyltransferase 1 [Source:HGNC Symbol;Acc:HGNC:4821]	ENSG00000128708	protein_coding	3'UTR;exon	multiple Ref	1	3303	2:171952941:171953001	ENSG00000128708	HAT1	10.79503758	0.005584446	-6.114514093	0.692681253	-3.972016074	6.807195346	2.142498019	-4.664697327	9.961963646	0.000115334	0.504336209	0.002654521	4.79E-05	0.057893267	0.000897042	1.379601979	-5.935295749	-1.908422891	2.191079177	-4.302930072	-0.80715853	21.05526817	0.000123988

SCN2A	0.127928964	0.176587971	0.046454381	0.048659007	-0.081474582	-0.130133589	8	0.15132847	0.816496581	0.816496581	0.816496581	0.816496581	0.816496581	0.816496581	20	74	0	A_23_P51019	A_23_P51019	A_23_P51019	chr2	165389596	165389656	+	SCN2A	"sodium channel, voltage-gated, type II, alpha subunit [Source:HGNC Symbol;Acc:HGNC:10588]"	ENSG00000136531	protein_coding	exon	coding exon	1	3304	2:165389596:165389656	ENSG00000136531	SCN2A	4.063040913	0.020596878	1.091726394	1.506974984	0.396434653	0.41524859	-0.695291741	-1.110540332	9.961963646	0.300652535	0.162853142	0.700143273	0.68676194	0.502768027	0.292851686	-6.384320642	-5.108552952	-6.961968619	-7.056127774	-6.045800041	-6.436449345	0.91819373	0.466901551

SH2B2	0.058581522	0.175468045	0.051660679	0.116886523	-0.006920843	-0.123807366	8	0.104113284	0.816496581	0.816496581	0.816496581	0.816496581	0.816496581	0.816496581	20	75	0	P_145534	P_145534	P_145534	chr7	102283685	102283745	-	SH2B2;CUX1	SH2B adaptor protein 2 [Source:HGNC Symbol;Acc:HGNC:17381];cut-like homeobox 1 [Source:HGNC Symbol;Acc:HGNC:2557]	ENSG00000160999	protein_coding	3'ext;3'UTR;exon;promoter	multiple non-Ref	0	3305	7:102283685:102283745	ENSG00000160999	SH2B2	7.277024788	0.010911455	0.686854835	2.057322347	0.605709544	1.370467512	-0.081145292	-1.451612804	9.961963646	0.507847015	0.066791857	0.558254535	0.200629047	0.936933524	0.177362669	-6.746877605	-4.337939192	-6.850389483	-6.209169004	-6.293184214	-6.02788488	1.510529058	0.271434482

OAS1	0.083794934	0.309138228	0.395528053	0.225343294	0.311733119	0.086389825	8	0.199905261	0.816496581	0.816496581	0.816496581	0.816496581	0.816496581	0.816496581	20	76	0	A_16_P19685830	A_16_P19685830	A_16_P19685830	chr12	112909125	112909185	+	OAS1;RP1-71H24.1	"2'-5'-oligoadenylate synthetase 1, 40/46kDa [Source:HGNC Symbol;Acc:HGNC:8086];"	ENSG00000089127	protein_coding;antisense	3'ext;intron	multiple non-Ref	0	3306	12:112909125:112909185	ENSG00000089127	OAS1	4.327095572	0.03429844	0.554148562	2.044377817	2.615686781	1.490229255	2.061538219	0.571308964	9.961963646	0.591698389	0.06825326	0.025863294	0.167129222	0.066322344	0.580449855	-6.83334034	-4.357278227	-4.209348847	-6.053962081	-4.426506449	-6.895947382	3.020915937	0.080720039

C12orf4	-0.027365592	0.086053127	0.077782065	0.113418719	0.105147656	-0.008271063	8	0.108729724	0.816496581	0.816496581	0.816496581	0.816496581	0.816496581	0.816496581	20	78	0	P_214100	P_214100	P_214100	chr12	4536016	4536076	-	C12orf4	chromosome 12 open reading frame 4 [Source:HGNC Symbol;Acc:HGNC:1184]	ENSG00000047621	protein_coding	"5'UTR,exon;exon"	multiple Ref	1	3308	12:4536016:4536076	ENSG00000047621	C12orf4	8.596973718	0.011700517	-0.309847461	0.974338256	0.880688984	1.284185717	1.190536445	-0.093649272	9.961963646	0.763061385	0.352953477	0.399234656	0.228137876	0.261431614	0.927244498	-6.946223775	-5.704992635	-6.639346856	-6.315477268	-5.593458938	-7.066589464	0.816038956	0.513961915

ANKH	-0.555524957	0.080947122	-0.495686012	0.63647208	0.059838946	-0.576633134	8	0.066813158	0.816496581	0.816496581	0.816496581	0.816496581	0.816496581	0.816496581	20	79	0	P_252173	P_252173	P_252173	chr5	14712873	14712933	-	ANKH	ANKH inorganic pyrophosphate transport regulator [Source:HGNC Symbol;Acc:HGNC:15492]	ENSG00000154122	protein_coding	exon	coding exon	1	3309	5:14712873:14712933	ENSG00000154122	ANKH	6.096040349	0.005791518	-8.940318772	1.302719285	-7.97730308	10.24303806	0.963015691	-9.280022365	9.961963646	4.51E-06	0.221979145	1.24E-05	1.31E-06	0.358332879	3.23E-06	4.710575694	-5.358813423	3.660365337	5.913953954	-5.825760012	5.02584668	56.20496411	1.51E-06

TRERF1	0.023543088	-0.154127263	-0.106979683	-0.177670352	-0.130522771	0.04714758	8	0.095176024	0.816496581	0.816496581	0.816496581	0.816496581	0.816496581	0.816496581	20	81	0	P_257044	P_257044	P_257044	chr6	42257038	42257098	-	TRERF1	transcriptional regulating factor 1 [Source:HGNC Symbol;Acc:HGNC:18273]	ENSG00000124496	protein_coding	exon	coding exon	1	3311	6:42257038:42257098	ENSG00000124496	TRERF1	7.674137347	0.009481134	0.296127625	-1.938630138	-1.345602544	-2.234757763	-1.641730169	0.593027593	9.961963646	0.773213813	0.081376066	0.208255941	0.049539359	0.131794877	0.566381268	-6.950718808	-4.513469644	-6.139172499	-4.939018394	-5.033349478	-6.882590465	2.28295442	0.141507506

SARS1	-0.128978857	0.114848461	0.161285062	0.243827317	0.290263918	0.046436601	8	0.076300447	0.816496581	0.816496581	0.816496581	0.816496581	0.816496581	0.816496581	20	83	0	P_203306	P_203306	P_203306	chr1	109237313	109237373	+	SARS	seryl-tRNA synthetase [Source:HGNC Symbol;Acc:HGNC:10537]	ENSG00000031698	protein_coding	exon	coding exon	1	3313	1:109237313:109237373	ENSG00000031698	SARS1	9.896341787	0.006881873	-1.904192353	1.695576833	2.381148273	3.599769186	4.285340626	0.68557144	9.961963646	0.0861376	0.120940417	0.038625964	0.004879412	0.001611839	0.508622336	-5.313316351	-4.858151035	-4.600834336	-2.62550108	-0.89524365	-6.820526048	7.327214515	0.007009971

GPR1	-0.270101452	-0.349049038	0.243163073	-0.078947585	0.513264526	0.592212111	8	0.288804027	0.816496581	0.816496581	0.816496581	0.816496581	0.816496581	0.816496581	20	84	0	A_23_P154150	A_23_P154150	A_23_P154150	chr2	206176539	206176599	-	GPR1	G protein-coupled receptor 1 [Source:HGNC Symbol;Acc:HGNC:4463]	ENSG00000183671	protein_coding	exon	coding exon	1	3314	2:206176539:206176599	ENSG00000183671	GPR1	3.166811471	0.069187668	-1.257645387	-1.625240843	1.132215006	-0.367595456	2.389860393	2.757455849	9.961963646	0.237201384	0.135289204	0.284060432	0.720864317	0.038056336	0.020285093	-6.197793405	-4.953482901	-6.388880256	-7.076216633	-3.916315078	-3.992241574	3.159185403	0.073103651

TRAF4	0.133336281	0.128235423	0.168221846	-0.005100858	0.034885564	0.039986423	8	0.077997602	0.816496581	0.816496581	0.816496581	0.816496581	0.816496581	0.816496581	20	85	0	P_228161	P_228161	P_228161	chr17	28748268	28748328	+	TRAF4	TNF receptor-associated factor 4 [Source:HGNC Symbol;Acc:HGNC:12034]	ENSG00000076604	protein_coding	"3'UTR;3'UTR,exon;exon"	multiple Ref	1	3315	17:28748268:28748328	ENSG00000076604	TRAF4	9.940778912	0.007092167	1.939119093	1.864936943	2.446462354	-0.07418215	0.507343261	0.581525411	9.961963646	0.081310268	0.091881375	0.034551158	0.942333885	0.622955761	0.573808415	-5.26003775	-4.620246975	-4.492658382	-7.146604498	-6.161575752	-6.889722246	2.304070448	0.139141248

PRR5-ARHGAP8	-0.481997051	-0.000625442	-0.208561934	0.48137161	0.273435118	-0.207936492	8	0.525868219	0.816496581	0.816496581	0.816496581	0.816496581	0.816496581	0.816496581	20	86	0	P_133653	P_133653	P_133653	chr22	44809741	44809801	+	PRR5-ARHGAP8	PRR5-ARHGAP8 readthrough [Source:HGNC Symbol;Acc:HGNC:34512]	ENSG00000248405	protein_coding	exon	non-coding exon	1	3316	22:44809741:44809801	ENSG00000248405	PRR5-ARHGAP8	4.185329801	0.224281282	-1.246503483	-0.001617469	-0.539366737	1.244886014	0.707136746	-0.537749269	9.961963646	0.241093047	0.998741379	0.601479866	0.241662296	0.495690012	0.602555291	-6.210931627	-6.182086045	-6.890540573	-6.362266708	-6.037393886	-6.915666941	0.697274645	0.574734162

PIH1D2	0.356991978	0.285393403	0.341433397	-0.071598575	-0.015558581	0.056039994	8	0.116061725	0.816496581	0.816496581	0.816496581	0.816496581	0.816496581	0.816496581	20	87	0	P_213347	P_213347	P_213347	chr11	112071634	112071694	-	PIH1D2	PIH1 domain containing 2 [Source:HGNC Symbol;Acc:HGNC:25210]	ENSG00000150773	protein_coding	exon	coding exon	1	3317	11:112071634:112071694	ENSG00000150773	PIH1D2	6.939023653	0.013024089	3.831161116	3.062780607	3.664189771	-0.768380509	-0.166971346	0.601409164	9.961963646	0.003335662	0.012038585	0.004386455	0.460088232	0.870733164	0.561002909	-2.100489715	-2.757053181	-2.42384059	-6.835999765	-6.281877961	-6.877311671	6.410781374	0.010790828

LDHC	0.10011047	-0.0208	-0.150846023	-0.12091047	-0.250956494	-0.130046024	8	0.178086885	0.816496581	0.816496581	0.816496581	0.816496581	0.816496581	0.816496581	20	88	0	P_211196	P_211196	P_211196	chr11	18429850	18429910	+	LDHC	lactate dehydrogenase C [Source:HGNC Symbol;Acc:HGNC:6544]	ENSG00000166796	protein_coding	3'UTR;exon	multiple Ref	1	3318	11:18429850:18429910	ENSG00000166796	LDHC	5.036099325	0.027675509	0.737016691	-0.153130305	-1.110533559	-0.890146996	-1.84755025	-0.957403254	9.961963646	0.478110803	0.881352843	0.292854466	0.39437573	0.09453724	0.361021377	-6.709806606	-6.169740967	-6.412436849	-6.732766034	-4.743877213	-6.592494018	1.153725311	0.374944027

ARAP1	0.050728387	0.006217908	-0.005383719	-0.044510479	-0.056112105	-0.011601626	8	0.082856288	0.816496581	0.816496581	0.816496581	0.816496581	0.816496581	0.816496581	20	89	0	A_23_P105184	A_23_P105184	A_23_P105184	chr11	72685216	72685276	-	ARAP1	"ArfGAP with RhoGAP domain, ankyrin repeat and PH domain 1 [Source:HGNC Symbol;Acc:HGNC:16925]"	ENSG00000186635	protein_coding	3'UTR;exon	multiple Ref	1	3319	11:72685216:72685276	ENSG00000186635	ARAP1	6.918783602	0.007719785	0.707121542	0.08667369	-0.075045623	-0.620447853	-0.782167166	-0.161719313	9.961963646	0.495699057	0.932648273	0.941664034	0.548893012	0.452306695	0.874759789	-6.732183906	-6.178128692	-7.044045869	-6.943083377	-5.981204365	-7.05710983	0.256009866	0.85536764

TCTEX1D4	0.983315525	0.052037533	0.695336978	-0.931277991	-0.287978547	0.643299444	8	0.09749976	0.816496581	0.816496581	0.816496581	0.816496581	0.816496581	0.816496581	20	90	0	P_101645	P_101645	P_101645	chr1	44806002	44806062	-	TCTEX1D4	Tctex1 domain containing 4 [Source:HGNC Symbol;Acc:HGNC:32315]	ENSG00000188396	protein_coding	exon	coding exon	1	3320	1:44806002:44806062	ENSG00000188396	TCTEX1D4	7.558791787	0.009840684	12.14020142	0.642465334	8.584763235	-11.49773609	-3.555438187	7.942297901	9.961963646	2.72E-07	0.535076341	6.48E-06	4.52E-07	0.005251822	1.28E-05	7.501474338	-5.969061629	4.323566358	6.997069727	-2.032681296	3.608276919	71.56909731	4.82E-07

SAMD4A	0.037837494	-0.00589435	0.054760787	-0.043731844	0.016923293	0.060655136	8	0.111571439	0.816496581	0.816496581	0.816496581	0.816496581	0.816496581	0.816496581	20	91	0	A_23_P335661	A_23_P335661	A_23_P335661	chr14	54792876	54792936	+	SAMD4A	sterile alpha motif domain containing 4A [Source:HGNC Symbol;Acc:HGNC:23023]	ENSG00000020577	protein_coding	3'UTR	3'UTR	1	3321	14:54792876:54792936	ENSG00000020577	SAMD4A	4.160245142	0.012203256	0.419498229	-0.065349712	0.607124061	-0.484847941	0.187625832	0.672473773	9.961963646	0.68375485	0.949188383	0.557352173	0.638268486	0.854936468	0.516575769	-6.90317526	-6.179836595	-6.849486191	-7.022559406	-6.277995115	-6.829810348	0.211891821	0.885882021

EXTL2	-0.40454371	0.364712179	-0.334526241	0.769255889	0.070017469	-0.69923842	8	0.097410324	0.816496581	0.816496581	0.816496581	0.816496581	0.816496581	0.816496581	20	92	0	P_102521	P_102521	P_102521	chr1	100875464	100875524	-	EXTL2	exostosin-like glycosyltransferase 2 [Source:HGNC Symbol;Acc:HGNC:3516]	ENSG00000162694	protein_coding	intron	intron sense	0	3322	1:100875464:100875524	ENSG00000162694	EXTL2	7.306773788	0.009826685	-4.99813027	4.50601243	-4.133065699	9.504142699	0.865064571	-8.639078129	9.961963646	0.000545069	0.001143418	0.002050754	2.60E-06	0.407352366	6.12E-06	-0.231239271	-0.539845005	-1.642541507	5.215262558	-5.91336897	4.370321329	38.01307039	9.17E-06

SLC12A8	-0.344365821	0.085416855	-0.1832793	0.429782676	0.161086521	-0.268696154	8	0.11768545	0.816496581	0.816496581	0.816496581	0.816496581	0.816496581	0.816496581	20	93	0	P_245985	P_245985	P_245985	chr3	125135722	125135782	-	SLC12A8	"solute carrier family 12, member 8 [Source:HGNC Symbol;Acc:HGNC:15595]"	ENSG00000221955	protein_coding	3'UTR;exon	multiple Ref	1	3323	3:125135722:125135782	ENSG00000221955	SLC12A8	6.928568503	0.013328881	-3.65316113	0.906133867	-1.944295201	4.559294997	1.708865929	-2.850429068	9.961963646	0.004467022	0.3862571	0.080616773	0.001053523	0.118388717	0.017299455	-2.399219707	-5.766858841	-5.299289708	-1.040738335	-4.940875983	-3.833937405	8.296548027	0.004608346

AGFG1	-0.094791323	0.12076622	0.009909622	0.215557543	0.104700945	-0.110856598	8	0.087486864	0.816496581	0.816496581	0.816496581	0.816496581	0.816496581	0.816496581	20	97	0	P_239550	P_239550	P_239550	chr2	227534961	227535021	+	AGFG1	ArfGAP with FG repeats 1 [Source:HGNC Symbol;Acc:HGNC:5175]	ENSG00000173744	protein_coding	exon	coding exon	1	3327	2:227534961:227535021	ENSG00000173744	AGFG1	7.476035583	0.008353224	-1.270243969	1.61831863	0.132793143	2.888562599	1.403037112	-1.485525487	9.961963646	0.23286288	0.136780596	0.897000997	0.016206876	0.190996491	0.16834775	-6.182835791	-4.962744653	-7.037499061	-3.842424547	-5.344257959	-5.983222777	2.794866991	0.09529741

COL14A1	0.038362556	0.097192088	0.014554452	0.058829532	-0.023808104	-0.082637636	8	0.108758023	0.816496581	0.816496581	0.816496581	0.816496581	0.816496581	0.816496581	20	98	0	P_147868	P_147868	P_147868	chr8	120080599	120080659	+	COL14A1	"collagen, type XIV, alpha 1 [Source:HGNC Symbol;Acc:HGNC:2191]"	ENSG00000187955	protein_coding	intron	intron sense	0	3328	8:120080599:120080659	ENSG00000187955	COL14A1	4.016239067	0.01170546	0.43426909	1.100227001	0.164758277	0.665957911	-0.269510813	-0.935468724	9.961963646	0.673348163	0.297108282	0.872429413	0.520560097	0.793036692	0.371668629	-6.89641827	-5.581334153	-7.032316592	-6.912353151	-6.258195927	-6.613291479	0.46957869	0.710135065

RBM26	-0.13416812	-0.141653142	-0.121864421	-0.007485021	0.0123037	0.019788721	8	0.052429182	0.816496581	0.816496581	0.816496581	0.816496581	0.816496581	0.816496581	20	99	0	P_113976	P_113976	P_113976	chr13	79354569	79354629	-	RBM26	RNA binding motif protein 26 [Source:HGNC Symbol;Acc:HGNC:20327]	ENSG00000139746	protein_coding	"exon,intron;intron"	multiple non-Ref	0	3329	13:79354569:79354629	ENSG00000139746	RBM26	8.063736636	0.004414136	-2.473273424	-2.611253324	-2.246465349	-0.1379799	0.226808074	0.364787975	9.961963646	0.03300363	0.026060501	0.048566347	0.893005424	0.825159402	0.72289456	-4.401830391	-3.478785608	-4.821310657	-7.139211567	-6.269396741	-6.99916643	3.030980423	0.080134893

CA5B	0.013679823	0.022878902	-0.050709972	0.009199079	-0.064389795	-0.073588874	8	0.094807361	0.816496581	0.816496581	0.816496581	0.816496581	0.816496581	0.816496581	20	100	0	P_269571	P_269571	P_269571	chrX	15775248	15775308	+	CA5B	"carbonic anhydrase VB, mitochondrial [Source:HGNC Symbol;Acc:HGNC:1378]"	ENSG00000169239	protein_coding	exon	coding exon	1	3330	X:15775248:15775308	ENSG00000169239	CA5B	5.542086003	0.009424888	0.172579025	0.288630822	-0.639736163	0.116051797	-0.812315188	-0.928366985	9.961963646	0.866438149	0.77878048	0.536777924	0.909917591	0.435592296	0.375163726	-6.982132128	-6.13836019	-6.828120411	-7.142252858	-5.957221168	-6.61993866	0.342923812	0.795002233

RTCB	-0.095756371	-0.035793157	-0.116621711	0.059963214	-0.02086534	-0.080828554	8	0.085607413	0.816496581	0.816496581	0.816496581	0.816496581	0.816496581	0.816496581	20	102	0	A_23_P68892	A_23_P68892	A_23_P68892	chr22	32388041	32388101	-	RTCB	"RNA 2',3'-cyclic phosphate and 5'-OH ligase [Source:HGNC Symbol;Acc:HGNC:26935]"	ENSG00000100220	protein_coding	exon	coding exon	1	3332	22:32388041:32388101	ENSG00000100220	RTCB	10.87859522	0.008091973	-1.303725316	-0.487324705	-1.587807428	0.816400611	-0.284082112	-1.100482723	9.961963646	0.22164881	0.636573582	0.143530018	0.433359317	0.782164508	0.297002163	-6.142567974	-6.058422266	-5.820882766	-6.796866892	-6.253939285	-6.447260895	1.069432054	0.405383115

PTCHD4	-0.053694507	0.411139577	0.289468827	0.464834084	0.343163334	-0.12167075	8	0.324504891	0.816496581	0.816496581	0.816496581	0.816496581	0.816496581	0.816496581	20	103	0	P_257274	P_257274	P_257274	chr6	48008666	48008726	-	PTCHD4	patched domain containing 4 [Source:HGNC Symbol;Acc:HGNC:21345]	ENSG00000244694	protein_coding	exon	coding exon	1	3333	6:48008666:48008726	ENSG00000244694	PTCHD4	3.774612213	0.086771075	-0.223248218	1.709414659	1.203538371	1.932662876	1.426786588	-0.505876288	9.961963646	0.827853558	0.118284408	0.256587926	0.0821831	0.184222791	0.623948722	-6.971231507	-4.839141629	-6.308946671	-5.416797341	-5.314691101	-6.933351565	1.741209906	0.221873032

PDSS1	-0.372436779	0.10943355	-0.100546199	0.481870329	0.271890579	-0.20997975	8	0.047716203	0.816496581	0.816496581	0.816496581	0.816496581	0.816496581	0.816496581	20	104	0	P_207811	P_207811	P_207811	chr10	26723838	26723898	+	PDSS1	"prenyl (decaprenyl) diphosphate synthase, subunit 1 [Source:HGNC Symbol;Acc:HGNC:17759]"	ENSG00000148459	protein_coding	exon	coding exon	1	3334	10:26723838:26723898	ENSG00000148459	PDSS1	9.457953701	0.004035107	-7.180763777	2.109932529	-1.93858004	9.290696306	5.242183738	-4.048512568	9.961963646	3.05E-05	0.061152891	0.08138281	3.19E-06	0.000382368	0.002347309	2.753532357	-4.258886817	-5.30805736	5.004172595	0.4790541	-1.802906357	31.66020975	2.09E-05

FIG4	0.05539744	0.134447074	0.026156381	0.079049634	-0.029241059	-0.108290693	8	0.046906296	0.816496581	0.816496581	0.816496581	0.816496581	0.816496581	0.816496581	20	105	0	P_258676	P_258676	P_258676	chr6	109777000	109777060	+	FIG4	FIG4 phosphoinositide 5-phosphatase [Source:HGNC Symbol;Acc:HGNC:16873]	ENSG00000112367	protein_coding	exon	coding exon	1	3335	6:109777000:109777060	ENSG00000112367	FIG4	7.260767912	0.003973565	1.076329308	2.612202403	0.508198206	1.535873095	-0.568131103	-2.104004197	9.961963646	0.307154958	0.026018159	0.622377434	0.15569792	0.582524166	0.061764859	-6.400598964	-3.477277547	-6.907889808	-5.992629141	-6.127819738	-5.073336553	2.558144061	0.114021073

VAC14	-0.132989291	0.062639486	-0.151104725	0.195628777	-0.018115434	-0.213744211	8	0.044224881	0.816496581	0.816496581	0.816496581	0.816496581	0.816496581	0.816496581	20	106	0	P_119473	P_119473	P_119473	chr16	70689584	70689644	-	VAC14	Vac14 homolog (S. cerevisiae) [Source:HGNC Symbol;Acc:HGNC:25507]	ENSG00000103043	protein_coding	exon	non-coding exon	1	3336	16:70689584:70689644	ENSG00000103043	VAC14	5.502427505	0.00377733	-2.650146143	1.248249314	-3.011141726	3.898395456	-0.360995583	-4.25939104	9.961963646	0.024380339	0.240479848	0.01314569	0.002990194	0.725640631	0.001678994	-4.105375293	-5.421350704	-3.536777304	-2.121996526	-6.227835357	-1.456849756	8.5197654	0.004203944

LRP2BP	0.280502216	-0.006746195	0.074633377	-0.28724841	-0.205868839	0.081379571	8	0.113888039	0.816496581	0.816496581	0.816496581	0.816496581	0.816496581	0.816496581	20	107	0	P_139039	P_139039	P_139039	chr4	185364283	185364343	-	LRP2BP	LRP2 binding protein [Source:HGNC Symbol;Acc:HGNC:25434]	ENSG00000109771	protein_coding	3'UTR	3'UTR	1	3337	4:185364283:185364343	ENSG00000109771	LRP2BP	4.96462972	0.012622691	3.057777986	-0.073540828	0.813584648	-3.131318814	-2.244193338	0.887125476	9.961963646	0.012141551	0.942831429	0.434897624	0.010714372	0.048753715	0.395923483	-3.412121599	-6.179237152	-6.697227085	-3.42629265	-4.145468566	-6.657694106	4.274314552	0.03493796

LINC01698	-0.468035002	0.045743881	-0.109368216	0.513778883	0.358666786	-0.155112097	8	0.331660224	0.816496581	0.816496581	0.816496581	0.816496581	0.816496581	0.816496581	20	108	0	P_308244	P_308244	P_308244	chr1	209364050	209364110	-	RP11-372M18.2	NA	ENSG00000231648	lincRNA	promoter	promoter antisense	0	3338	1:209364050:209364110	ENSG00000231648	LINC01698	4.531159831	0.090541481	-1.905022748	0.18618935	-0.445156749	2.091212099	1.459866	-0.631346099	9.961963646	0.086019788	0.856033013	0.665723242	0.063105329	0.175131259	0.542028614	-5.31205476	-6.163845292	-6.939971098	-5.169388343	-5.272976785	-6.857896671	1.794152892	0.212020316

BAZ1B	-0.021578611	-0.157335256	0.020075825	-0.135756645	0.041654436	0.177411081	8	0.087617383	0.816496581	0.816496581	0.816496581	0.816496581	0.816496581	0.816496581	20	111	0	P_261347	P_261347	P_261347	chr7	73465438	73465498	-	BAZ1B	"bromodomain adjacent to zinc finger domain, 1B [Source:HGNC Symbol;Acc:HGNC:961]"	ENSG00000009954	protein_coding	exon	coding exon	1	3341	7:73465438:73465498	ENSG00000009954	BAZ1B	9.191683829	0.008371578	-0.288845403	-2.106046831	0.26872952	-1.817201427	0.557574923	2.37477635	9.961963646	0.778620961	0.061553345	0.793620985	0.099343789	0.589443192	0.039047861	-6.953023447	-4.264749593	-7.007826262	-5.591411415	-6.133938934	-4.634519633	2.307292335	0.138784298

LDLRAD4	-0.192062236	-0.271079295	-0.308724441	-0.079017058	-0.116662204	-0.037645146	8	0.14485451	0.816496581	0.816496581	0.816496581	0.816496581	0.816496581	0.816496581	20	112	0	P_123249	P_123249	P_123249	chr18	13438250	13438310	+	LDLRAD4	low density lipoprotein receptor class A domain containing 4 [Source:HGNC Symbol;Acc:HGNC:1224]	ENSG00000168675	protein_coding	exon	coding exon	1	3342	18:13438250:13438310	ENSG00000168675	LDLRAD4	4.19512161	0.01905704	-1.703961945	-2.404995438	-2.738980387	-0.701033493	-1.035018442	-0.333984949	9.961963646	0.119324629	0.03708627	0.020937502	0.499329329	0.325131709	0.745315098	-5.609529259	-3.804390788	-4.00081131	-6.887314338	-5.75644718	-7.01078162	2.977293335	0.083315454

PIAS3	0.14153539	-0.009652838	0.062765481	-0.151188228	-0.078769909	0.072418319	8	0.067836227	0.816496581	0.816496581	0.816496581	0.816496581	0.816496581	0.816496581	20	113	0	P_203818	P_203818	P_203818	chr1	145853503	145853563	-	PIAS3	"protein inhibitor of activated STAT, 3 [Source:HGNC Symbol;Acc:HGNC:16861]"	ENSG00000131788	protein_coding	exon	coding exon	1	3343	1:145853503:145853563	ENSG00000131788	PIAS3	8.215679601	0.005902143	2.25634656	-0.153884819	1.000602582	-2.410231379	-1.255743978	1.154487401	9.961963646	0.047759543	0.880773277	0.340703297	0.036756358	0.237861862	0.275243226	-4.758626438	-6.169619149	-6.526306439	-4.650873482	-5.52006522	-6.388317844	2.472489211	0.121842825

MCHR1	-0.206547114	-0.45030566	-0.06179106	-0.243758547	0.144756054	0.388514601	8	0.232541931	0.816496581	0.816496581	0.816496581	0.816496581	0.816496581	0.816496581	20	116	0	A_23_P211543	A_23_P211543	A_23_P211543	chr22	40682185	40682245	+	MCHR1	melanin-concentrating hormone receptor 1 [Source:HGNC Symbol;Acc:HGNC:4479]	ENSG00000128285	protein_coding	3'UTR	3'UTR	1	3346	22:40682185:40682245	ENSG00000128285	MCHR1	4.281871676	0.04563246	-1.184207538	-2.581761361	-0.354269965	-1.397553823	0.829937573	2.227491396	9.961963646	0.263815661	0.027410977	0.730520677	0.19259018	0.426015226	0.050152718	-6.282800161	-3.525610393	-6.979011176	-6.174820103	-5.942836432	-4.875356545	2.632836235	0.107679385

NAXE	-0.014753855	-0.185122644	-0.128205277	-0.170368789	-0.113451421	0.056917367	8	0.214074136	0.816496581	0.816496581	0.816496581	0.816496581	0.816496581	0.816496581	20	119	0	P_103568	P_103568	P_103568	chr1	156586932	156586992	+	APOA1BP;TTC24	apolipoprotein A-I binding protein [Source:HGNC Symbol;Acc:HGNC:18453];tetratricopeptide repeat domain 24 [Source:HGNC Symbol;Acc:HGNC:32348]	ENSG00000163382	protein_coding	3'ext;promoter	multiple non-Ref	0	3349	1:156586932:156586992	ENSG00000163382	NAXE	10.82184983	0.039008855	-0.091489141	-1.14795159	-0.795005128	-1.056462448	-0.703515987	0.352946461	9.961963646	0.928917485	0.277808	0.445138398	0.315703352	0.497847061	0.731482496	-6.993794664	-5.531406111	-6.712545866	-6.571275535	-6.039977061	-7.003749805	0.615635854	0.620443877

UBR1	-0.023008951	-0.019707852	0.008691682	0.003301099	0.031700632	0.028399533	8	0.060216638	0.816496581	0.816496581	0.816496581	0.816496581	0.816496581	0.816496581	20	120	0	P_223038	P_223038	P_223038	chr15	43029943	43030003	-	UBR1	ubiquitin protein ligase E3 component n-recognin 1 [Source:HGNC Symbol;Acc:HGNC:16808]	ENSG00000159459	protein_coding	exon	coding exon	1	3350	15:43029943:43030003	ENSG00000159459	UBR1	7.940272831	0.005118595	-0.393882853	-0.337372396	0.148790118	0.056510457	0.542672971	0.486162514	9.961963646	0.701966681	0.742836772	0.884688092	0.956052529	0.599284733	0.637368622	-6.914357411	-6.122439094	-7.035044055	-7.147866964	-6.142393955	-6.943776106	0.137523951	0.935310176

CASC9	-0.369983036	-0.648468803	-0.109409254	-0.278485767	0.260573782	0.539059549	8	0.496168146	0.816496581	0.816496581	0.816496581	0.816496581	0.816496581	0.816496581	20	122	0	P_314131	P_314131	P_314131	chr8	75225261	75225321	+	CASC9	cancer susceptibility candidate 9 (non-protein coding) [Source:HGNC Symbol;Acc:HGNC:48906]	ENSG00000249395	lincRNA	intron	intron antisense	0	3352	8:75225261:75225321	ENSG00000249395	CASC9	3.510870304	0.199904919	-1.013481208	-1.776327242	-0.299700829	-0.762846034	0.713780378	1.476626412	9.961963646	0.33481264	0.106173045	0.77056529	0.463236275	0.491747138	0.170674162	-6.465128352	-4.746108258	-6.998290106	-6.840375109	-6.032623032	-5.995006453	1.257357546	0.34092033

DRAM1	-0.195065299	0.173491735	-0.000624469	0.368557034	0.19444083	-0.174116204	8	0.099327646	0.816496581	0.816496581	0.816496581	0.816496581	0.816496581	0.816496581	20	124	0	P_112551	P_112551	P_112551	chr12	101923169	101923229	-	DRAM1	DNA-damage regulated autophagy modulator 1 [Source:HGNC Symbol;Acc:HGNC:25645]	ENSG00000136048	protein_coding	3'ext;3'UTR;intron	multiple non-Ref	0	3354	12:101923169:101923229	ENSG00000136048	DRAM1	8.337236728	0.010129605	-2.373719488	2.111194117	-0.007599067	4.484913605	2.366120421	-2.118793184	9.961963646	0.039118273	0.06102341	0.994086903	0.001181231	0.039628218	0.060249013	-4.566674681	-4.256982518	-7.047089067	-1.159771871	-3.953913099	-5.049841237	6.715667167	0.009309872

PTPRO	-0.084604801	-0.062238466	0.014160973	0.022366335	0.098765774	0.076399439	8	0.103662941	0.816496581	0.816496581	0.816496581	0.816496581	0.816496581	0.816496581	20	126	0	P_214485	P_214485	P_214485	chr12	15508630	15508690	+	PTPRO	"protein tyrosine phosphatase, receptor type, O [Source:HGNC Symbol;Acc:HGNC:9678]"	ENSG00000151490	protein_coding	exon	coding exon	1	3356	12:15508630:15508690	ENSG00000151490	PTPRO	3.845707788	0.010836312	-0.995405149	-0.732257375	0.166608807	0.263147774	1.162013956	0.898866182	9.961963646	0.343102218	0.480884245	0.871010992	0.797799188	0.272312846	0.389933146	-6.483106643	-5.907069279	-7.031982642	-7.111876132	-5.624651749	-6.647093823	0.630379061	0.611949748

SLC25A45	-0.021035393	-0.038440856	0.033588825	-0.017405463	0.054624217	0.07202968	8	0.095542568	0.816496581	0.816496581	0.816496581	0.816496581	0.816496581	0.816496581	20	127	0	A_14_P110449	A_14_P110449	A_14_P110449	chr11	65378309	65378368	+	SLC25A45	"solute carrier family 25, member 45 [Source:HGNC Symbol;Acc:HGNC:27442]"	ENSG00000162241	protein_coding	3'ext;exon;intron;promoter	multiple non-Ref	0	3357	11:65378309:65378368	ENSG00000162241	SLC25A45	6.973566145	0.009537273	-0.263805689	-0.482088285	0.421238771	-0.218282596	0.68504446	0.903327056	9.961963646	0.797306356	0.640159558	0.682524892	0.831615528	0.508940901	0.387673898	-6.960516833	-6.061034087	-6.951076884	-7.123618845	-6.052968679	-6.64303523	0.302062939	0.823304024

F13A1	-0.018991188	0.142579648	0.033935481	0.161570835	0.052926669	-0.108644167	8	0.123454367	0.816496581	0.816496581	0.816496581	0.816496581	0.816496581	0.816496581	20	128	0	P_141554	P_141554	P_141554	chr6	6144359	6144419	+	F13A1	"coagulation factor XIII, A1 polypeptide [Source:HGNC Symbol;Acc:HGNC:3531]"	ENSG00000124491	protein_coding	3'UTR	3'UTR antisense	0	3358	6:6144359:6144419	ENSG00000124491	F13A1	4.006331307	0.014446022	-0.193518999	1.452877588	0.345800405	1.646396587	0.539319404	-1.107077183	9.961963646	0.850441307	0.177019131	0.736684086	0.130820757	0.601511322	0.29427572	-6.97796299	-5.17711647	-6.982209582	-5.839526283	-6.144267009	-6.440180754	1.082497973	0.40049326

LIAS	-0.164086598	0.002864417	-0.034034065	0.166951015	0.130052533	-0.036898482	8	0.061755089	0.816496581	0.816496581	0.816496581	0.816496581	0.816496581	0.816496581	20	129	0	P_248614	P_248614	P_248614	chr4	39465136	39465196	+	LIAS	lipoic acid synthetase [Source:HGNC Symbol;Acc:HGNC:16429]	ENSG00000121897	protein_coding	exon	coding exon	1	3359	4:39465136:39465196	ENSG00000121897	LIAS	9.545043929	0.005269286	-2.768489378	0.048328802	-0.574226959	2.816818181	2.19426242	-0.622555761	9.961963646	0.019905252	0.96240939	0.578548699	0.018323981	0.053052285	0.547561456	-3.905124992	-6.180856274	-6.869985687	-3.965019986	-4.223069079	-6.863687955	3.522238936	0.056827757

ADGRG5	-0.084073702	-0.13271921	-0.011435008	-0.048645507	0.072638694	0.121284201	8	0.236679291	0.816496581	0.816496581	0.816496581	0.816496581	0.816496581	0.816496581	20	132	0	A_23_P206293	A_23_P206293	A_23_P206293	chr16	57576147	57576207	+	GPR114	G protein-coupled receptor 114 [Source:HGNC Symbol;Acc:HGNC:19010]	ENSG00000159618	protein_coding	3'UTR;exon	multiple Ref	1	3362	16:57576147:57576207	ENSG00000159618	ADGRG5	5.056617973	0.047191459	-0.47399537	-0.748251702	-0.064468923	-0.274256332	0.409526447	0.683782779	9.961963646	0.64572085	0.471603948	0.949872176	0.789490642	0.690820058	0.509704093	-6.877131974	-5.895264091	-7.044851313	-7.108635099	-6.208248788	-6.821803554	0.249865966	0.859639121

SIRT7	-0.082271024	-0.040533277	0.021041818	0.041737746	0.103312842	0.061575096	8	0.055860963	0.816496581	0.816496581	0.816496581	0.816496581	0.816496581	0.816496581	20	133	0	P_122959	P_122959	P_122959	chr17	81912273	81912333	-	SIRT7	sirtuin 7 [Source:HGNC Symbol;Acc:HGNC:14935]	ENSG00000187531	protein_coding	3'UTR;exon	multiple Ref	1	3363	17:81912273:81912333	ENSG00000187531	SIRT7	9.512712496	0.004712573	-1.467788475	-0.723149837	0.375404817	0.744638637	1.843193292	1.098554655	9.961963646	0.173012068	0.486219736	0.715228941	0.473690309	0.095213818	0.297802991	-5.934960947	-5.913689593	-6.970701259	-6.854570329	-4.750179589	-6.449324723	1.329491335	0.319269213

GPR161	0.255493695	-0.247494044	0.124427784	-0.50298774	-0.131065912	0.371921828	8	0.081424204	0.816496581	0.816496581	0.816496581	0.816496581	0.816496581	0.816496581	20	137	0	A_32_P497742	A_32_P497742	A_32_P497742	chr1	168079874	168079934	-	GPR161	G protein-coupled receptor 161 [Source:HGNC Symbol;Acc:HGNC:23694]	ENSG00000143147	protein_coding	3'UTR	3'UTR	1	3367	1:168079874:168079934	ENSG00000143147	GPR161	6.795959484	0.007530856	3.605816707	-3.49291656	1.75606596	-7.098733267	-1.849750747	5.24898252	9.961963646	0.004830777	0.00582788	0.109715348	3.37E-05	0.094197224	0.000378656	-2.479109545	-2.07324805	-5.581541757	2.559103757	-4.740691534	0.089969818	18.27525953	0.000224756

ATIC	0.122585291	-0.043193778	0.105691254	-0.16577907	-0.016894037	0.148885033	8	0.050925188	0.816496581	0.816496581	0.816496581	0.816496581	0.816496581	0.816496581	20	138	0	P_129791	P_129791	P_129791	chr2	215349670	215349730	+	ATIC	5-aminoimidazole-4-carboxamide ribonucleotide formyltransferase/IMP cyclohydrolase [Source:HGNC Symbol;Acc:HGNC:794]	ENSG00000138363	protein_coding	3'UTR;exon	multiple Ref	1	3368	2:215349670:215349730	ENSG00000138363	ATIC	10.98899597	0.004289305	2.292400579	-0.807743261	1.976474422	-3.100143841	-0.315926157	2.784217684	9.961963646	0.044924492	0.438100268	0.076428327	0.011297202	0.758578155	0.019376112	-4.699969668	-5.849380103	-5.249711889	-3.479782596	-6.24387097	-3.94673343	4.546095003	0.029617263

DHCR24	-0.915955572	0.11596653	-0.798587134	1.031922102	0.117368438	-0.914553664	8	0.085472859	0.816496581	0.816496581	0.816496581	0.816496581	0.816496581	0.816496581	20	140	0	P_101956	P_101956	P_101956	chr1	54850132	54850192	+	DHCR24	24-dehydrocholesterol reductase [Source:HGNC Symbol;Acc:HGNC:2859]	ENSG00000116133	protein_coding	3'ext;3'UTR	multiple non-Ref	0	3370	1:54850132:54850192	ENSG00000116133	DHCR24	8.249857743	0.008073487	-12.48502649	1.580693695	-10.88522394	14.06572018	1.599802545	-12.46591764	9.961963646	2.09E-07	0.145145487	7.51E-07	6.75E-08	0.140842079	2.12E-07	7.755584821	-5.012692883	6.50310546	8.879550673	-5.090054824	7.753585215	105.4442557	7.56E-08

MAGED2	-0.12891098	-0.015989345	-0.120571178	0.112921635	0.008339802	-0.104581833	8	0.069066535	0.816496581	0.816496581	0.816496581	0.816496581	0.816496581	0.816496581	20	142	0	P_270424	P_270424	P_270424	chrX	54815514	54815574	+	MAGED2	"melanoma antigen family D, 2 [Source:HGNC Symbol;Acc:HGNC:16353]"	ENSG00000102316	protein_coding	exon	coding exon	1	3372	X:54815514:54815574	ENSG00000102316	MAGED2	10.31557427	0.006037404	-2.031937906	-0.252029392	-1.900483165	1.779908514	0.131454741	-1.648453773	9.961963646	0.069686226	0.806141699	0.086665676	0.105557914	0.898032518	0.130393369	-5.116409194	-6.148712853	-5.366203288	-5.646680195	-6.287502298	-5.759910778	2.284480205	0.141334954

SEPHS2	0.170880769	0.045081519	0.084751377	-0.125799251	-0.086129392	0.039669858	8	0.145080847	0.816496581	0.816496581	0.816496581	0.816496581	0.816496581	0.816496581	20	143	0	P_118747	P_118747	P_118747	chr16	30444056	30444116	-	SEPHS2	selenophosphate synthetase 2 [Source:HGNC Symbol;Acc:HGNC:19686]	ENSG00000179918	protein_coding	3'UTR	3'UTR	1	3373	16:30444056:30444116	ENSG00000179918	SEPHS2	9.785213525	0.019109739	1.513949699	0.399408032	0.750870459	-1.114541667	-0.76307924	0.351462427	9.961963646	0.161100415	0.698021141	0.470095416	0.291213017	0.463103347	0.732561556	-5.873666625	-6.098683541	-6.747679962	-6.509662448	-5.995976147	-7.004313819	0.827232634	0.508575684

CRISP3	-0.293049453	0.035862568	-0.108513573	0.328912021	0.18453588	-0.144376141	8	0.251725427	0.816496581	0.816496581	0.816496581	0.816496581	0.816496581	0.816496581	20	144	0	A_23_P419760	A_23_P419760	A_23_P419760	chr6	49728464	49728524	-	CRISP3	cysteine-rich secretory protein 3 [Source:HGNC Symbol;Acc:HGNC:16904]	ENSG00000096006	protein_coding	3'UTR	3'UTR	1	3374	6:49728464:49728524	ENSG00000096006	CRISP3	4.430079063	0.053092789	-1.557646044	0.190620343	-0.5767823	1.748266387	0.980863744	-0.767402643	9.961963646	0.150490311	0.852651638	0.576886605	0.111107473	0.349880127	0.46064344	-5.814562738	-6.162968361	-6.868431523	-5.693111402	-5.808957944	-6.758871796	1.233785166	0.348347349

NPSR1-AS1	0.257399779	0.526067729	0.340005277	0.268667951	0.082605499	-0.186062452	8	0.416981632	0.816496581	0.816496581	0.816496581	0.816496581	0.816496581	0.816496581	20	145	0	P_144599	P_144599	P_144599	chr7	34719146	34719206	-	NPSR1-AS1	NPSR1 antisense RNA 1 [Source:HGNC Symbol;Acc:HGNC:22128]	ENSG00000197085	processed_transcript	exon	non-coding exon	1	3375	7:34719146:34719206	ENSG00000197085	NPSR1-AS1	4.170784723	0.141836731	0.8370655	1.710775156	1.105699036	0.873709656	0.268633537	-0.60507612	9.961963646	0.422182092	0.118026147	0.294843899	0.402846801	0.793692775	0.55865888	-6.628957956	-4.837268308	-6.41764126	-6.747466097	-6.258445159	-6.874980533	1.008608383	0.429012352

DNAJC21	0.046859256	0.028646279	0.069036258	-0.018212976	0.022177002	0.040389979	8	0.063698411	0.816496581	0.816496581	0.816496581	0.816496581	0.816496581	0.816496581	20	148	0	P_139331	P_139331	P_139331	chr5	34949555	34949615	+	DNAJC21	"DnaJ (Hsp40) homolog, subfamily C, member 21 [Source:HGNC Symbol;Acc:HGNC:27030]"	ENSG00000168724	protein_coding	exon	coding exon	1	3378	5:34949555:34949615	ENSG00000168724	DNAJC21	7.658492443	0.005465068	0.776324414	0.474587265	1.143734184	-0.301737149	0.36740977	0.669146919	9.961963646	0.455593952	0.645313328	0.279472948	0.769057314	0.720998526	0.518607792	-6.679125289	-6.064728601	-6.376222143	-7.100050942	-6.225384056	-6.832142579	0.468305566	0.710958966

SYK	0.014061068	-0.055619555	0.02565223	-0.069680623	0.011591162	0.081271785	8	0.122842844	0.816496581	0.816496581	0.816496581	0.816496581	0.816496581	0.816496581	20	149	0	P_267889	P_267889	P_267889	chr9	90878893	90878953	+	SYK	spleen tyrosine kinase [Source:HGNC Symbol;Acc:HGNC:11491]	ENSG00000165025	protein_coding	exon	coding exon	1	3379	9:90878893:90878953	ENSG00000165025	SYK	3.814045014	0.014325069	0.143885013	-0.569147423	0.262495815	-0.713032436	0.118610802	0.831643238	9.961963646	0.888460301	0.58186034	0.79828765	0.492190054	0.907941336	0.425095851	-6.987074727	-6.014152549	-7.009621893	-6.878481566	-6.289207852	-6.706166006	0.271274999	0.844741052

TCF7	-0.050160645	0.135201	0.084816388	0.185361646	0.134977033	-0.050384612	8	0.085050709	0.816496581	0.816496581	0.816496581	0.816496581	0.816496581	0.816496581	20	150	0	A_23_P7582	NM_003202	A_23_P7582	chr5	134147545	134147605	+	TCF7	"transcription factor 7 (T-cell specific, HMG-box) [Source:HGNC Symbol;Acc:HGNC:11639]"	ENSG00000081059	protein_coding	3'UTR;exon	multiple Ref	1	3380	5:134147545:134147605	ENSG00000081059	TCF7	5.674053118	0.008015678	-0.686180891	1.849504583	1.160259895	2.535685474	1.846440786	-0.689244688	9.961963646	0.508254066	0.094235204	0.272993557	0.029661273	0.094709102	0.506405177	-6.747359563	-4.642373165	-6.357891494	-4.44142415	-4.745482722	-6.817893012	2.591969513	0.111096106

HSBP1L1	0.021378503	0.078126469	0.020305175	0.056747966	-0.001073328	-0.057821294	8	0.065537909	0.816496581	0.816496581	0.816496581	0.816496581	0.816496581	0.816496581	20	151	0	P_123832	P_123832	P_123832	chr18	79966618	79966678	+	HSBP1L1	heat shock factor binding protein 1-like 1 [Source:HGNC Symbol;Acc:HGNC:37243]	ENSG00000226742	protein_coding	exon	coding exon	1	3381	18:79966618:79966678	ENSG00000226742	HSBP1L1	11.83727818	0.005655978	0.348152141	1.272301316	0.330672828	0.924149175	-0.017479313	-0.941628488	9.961963646	0.734970708	0.232160615	0.747741266	0.377250591	0.986399359	0.368656078	-6.932619431	-5.39396903	-6.987734194	-6.701620295	-6.296522754	-6.607491649	0.598387751	0.630514561

ADAMTSL1	0.023951426	-0.086144247	-0.076964899	-0.110095673	-0.100916326	0.009179347	8	0.096632779	0.816496581	0.816496581	0.816496581	0.816496581	0.816496581	0.816496581	20	153	0	P_266952	P_266952	P_266952	chr9	18909893	18909953	-	ADAMTSL1	ADAMTS-like 1 [Source:HGNC Symbol;Acc:HGNC:14632]	ENSG00000178031	protein_coding	3'ext;3'UTR;exon	multiple non-Ref	0	3383	9:18909893:18909953	ENSG00000178031	ADAMTSL1	5.071288945	0.009705522	0.297760825	-1.070933382	-0.956817003	-1.368694206	-1.254577828	0.114116378	9.961963646	0.772002875	0.309459055	0.361303048	0.201165081	0.23826768	0.911412696	-6.950194202	-5.611167884	-6.568973976	-6.211401887	-5.52140262	-7.064268941	0.935228417	0.459500827

HBS1L	0.046180961	-0.013285349	-0.016057683	-0.059466311	-0.062238644	-0.002772333	8	0.072739106	0.816496581	0.816496581	0.816496581	0.816496581	0.816496581	0.816496581	20	154	0	P_259335	P_259335	P_259335	chr6	135050581	135050641	-	HBS1L	HBS1-like translational GTPase [Source:HGNC Symbol;Acc:HGNC:4834]	ENSG00000112339	protein_coding	exon	coding exon	1	3384	6:135050581:135050641	ENSG00000112339	HBS1L	8.859820929	0.006455627	0.703946039	-0.202511353	-0.244770611	-0.906457391	-0.948716649	-0.042259258	9.961963646	0.497590558	0.843593042	0.811602094	0.38609403	0.36521126	0.967127027	-6.734511755	-6.160513903	-7.014500413	-6.717949162	-5.839037565	-7.070403	0.386662942	0.765102463

MMP8	0.285369586	0.170845945	0.285084801	-0.114523642	-0.000284786	0.114238856	8	0.215256035	0.816496581	0.816496581	0.816496581	0.816496581	0.816496581	0.816496581	20	156	0	P_212956	P_212956	P_212956	chr11	102718426	102718486	-	MMP8	matrix metallopeptidase 8 (neutrophil collagenase) [Source:HGNC Symbol;Acc:HGNC:7175]	ENSG00000118113	protein_coding	3'UTR;exon	multiple Ref	1	3386	11:102718426:102718486	ENSG00000118113	MMP8	3.724622394	0.039416344	1.760415338	1.053930888	1.758658525	-0.70648445	-0.001756813	0.704727637	9.961963646	0.108945967	0.316805506	0.109256143	0.496078188	0.99863295	0.497124584	-5.527714008	-5.628194344	-5.577751848	-6.883318525	-6.296683615	-6.80665442	1.383079449	0.304182842

PTK2B	-0.13349005	0.050724205	0.008533212	0.184214255	0.142023262	-0.042190993	8	0.082716418	0.816496581	0.816496581	0.816496581	0.816496581	0.816496581	0.816496581	20	157	0	A_23_P168836	A_23_P168836	A_23_P168836	chr8	27458695	27458755	+	PTK2B	protein tyrosine kinase 2 beta [Source:HGNC Symbol;Acc:HGNC:9612]	ENSG00000120899	protein_coding	3'UTR	3'UTR	1	3387	8:27458695:27458755	ENSG00000120899	PTK2B	6.817773434	0.007701188	-1.863012064	0.707916485	0.119091103	2.57092855	1.982103167	-0.588825382	9.961963646	0.092171965	0.495226241	0.907570484	0.027924306	0.075717041	0.569088515	-5.375557366	-5.924596623	-7.039380839	-4.382169448	-4.54600462	-6.885211092	2.478544049	0.121269597

TNPO1	-0.117451606	0.002366	-0.061787311	0.119817606	0.055664295	-0.064153311	8	0.052656052	0.816496581	0.816496581	0.816496581	0.816496581	0.816496581	0.816496581	20	158	0	P_313032	P_313032	P_313032	chr5	72877167	72877227	+	TNPO1;MIR4804	transportin 1 [Source:HGNC Symbol;Acc:HGNC:6401];microRNA 4804 [Source:HGNC Symbol;Acc:HGNC:41720]	ENSG00000083312	protein_coding;miRNA	intron;promoter	multiple non-Ref	0	3388	5:72877167:72877227	ENSG00000083312	TNPO1	8.084683789	0.004433281	-2.16043876	0.043520887	-1.136533646	2.203959647	1.023905114	-1.180054533	9.961963646	0.056169293	0.966146293	0.282333681	0.0521898	0.330100573	0.26538946	-4.913178987	-6.181089024	-6.384146154	-4.98889378	-5.767408482	-6.359661594	2.209907824	0.150064827

FATE1	-0.018315139	-0.086347752	0.217036097	-0.068032612	0.235351236	0.303383849	8	0.359695006	0.816496581	0.816496581	0.816496581	0.816496581	0.816496581	0.816496581	21	13	0	P_151854	P_151854	P_151854	chrX	151717319	151717379	+	FATE1	fetal and adult testis expressed 1 [Source:HGNC Symbol;Acc:HGNC:24683]	ENSG00000147378	protein_coding	exon	coding exon	1	3413	X:151717319:151717379	ENSG00000147378	FATE1	4.013482532	0.106106278	-0.068862902	-0.324658011	0.816031757	-0.255795109	0.884894658	1.140689768	9.961963646	0.946461384	0.752154573	0.433560608	0.803313231	0.397068923	0.280679689	-6.995773212	-6.126826148	-6.69518636	-7.113948668	-5.896272627	-6.403583837	0.487615932	0.698535791

PLAGL1	-0.019751859	-0.012910505	-0.097655959	0.006841354	-0.0779041	-0.084745453	8	0.099002004	0.816496581	0.816496581	0.816496581	0.816496581	0.816496581	0.816496581	21	14	0	P_143765	P_143765	P_143765	chr6	143940657	143940717	-	PLAGL1	pleiomorphic adenoma gene-like 1 [Source:HGNC Symbol;Acc:HGNC:9046]	ENSG00000118495	protein_coding	3'UTR	3'UTR	1	3414	6:143940657:143940717	ENSG00000118495	PLAGL1	4.750424846	0.01007774	-0.240975023	-0.157509698	-1.191414278	0.083465325	-0.950439255	-1.033904581	9.961963646	0.81446156	0.877989931	0.261102304	0.93513492	0.364377601	0.325627177	-6.966765287	-6.1690256	-6.322793736	-7.145804679	-5.837446718	-6.516865597	0.580267695	0.641248466

FRMD4A	-0.046903867	-0.296103	0.043213392	-0.249199133	0.090117259	0.339316392	8	0.115286594	0.816496581	0.816496581	0.816496581	0.816496581	0.816496581	0.816496581	21	16	0	P_207466	P_207466	P_207466	chr10	13670406	13670466	-	FRMD4A	FERM domain containing 4A [Source:HGNC Symbol;Acc:HGNC:25491]	ENSG00000151474	protein_coding	exon	coding exon	1	3416	10:13670406:13670466	ENSG00000151474	FRMD4A	4.02963561	0.012880081	-0.506168352	-3.19542881	0.466342183	-2.689260458	0.972510535	3.661770993	9.961963646	0.623750967	0.009610541	0.651001137	0.022799647	0.353817821	0.004403994	-6.860332532	-2.545083801	-6.929642532	-4.182130782	-5.816853125	-2.449614884	5.378485344	0.01841047

INS	0.037525284	-0.098901098	0.013420602	-0.136426382	-0.024104681	0.1123217	8	0.098402925	0.816496581	0.816496581	0.816496581	0.816496581	0.816496581	0.816496581	21	22	0	P_107633	P_107633	P_107633	chr11	2159780	2159840	-	INS	insulin [Source:HGNC Symbol;Acc:HGNC:6081]	ENSG00000254647	protein_coding	3'UTR;exon	multiple Ref	1	3422	11:2159780:2159840	ENSG00000254647	INS	8.368613499	0.00998277	0.459985437	-1.212331004	0.164509927	-1.672316441	-0.29547551	1.376840931	9.961963646	0.655402418	0.253353344	0.872619808	0.12552652	0.773697504	0.19871243	-6.884118114	-5.46154027	-7.032361128	-5.802732979	-6.250457513	-6.123959133	1.081339264	0.400924299

KIAA1210	0.19994928	0.288315247	0.114668009	0.088365968	-0.08528127	-0.173647238	8	0.470274759	0.816496581	0.816496581	0.816496581	0.816496581	0.816496581	0.816496581	21	23	0	A_23_P415791	A_23_P415791	A_23_P415791	chrX	119078784	119078844	-	KIAA1210	KIAA1210 [Source:HGNC Symbol;Acc:HGNC:29218]	ENSG00000250423	protein_coding	3'UTR	3'UTR	1	3423	X:119078784:119078844	ENSG00000250423	KIAA1210	3.796026435	0.179808897	0.5775105	0.832736597	0.331193888	0.255226097	-0.246316612	-0.501542708	9.961963646	0.576413432	0.424507222	0.747359391	0.803740432	0.81043822	0.626886636	-6.819366539	-5.829190256	-6.987547919	-7.114106643	-6.264515827	-6.935676924	0.252335888	0.857922453

DNMT3A	0.200069022	0.072329378	0.209770941	-0.127739644	0.009701919	0.137441563	8	0.095698977	0.816496581	0.816496581	0.816496581	0.816496581	0.816496581	0.816496581	21	24	0	P_127351	P_127351	P_127351	chr2	25282305	25282365	-	DNMT3A	DNA (cytosine-5-)-methyltransferase 3 alpha [Source:HGNC Symbol;Acc:HGNC:2978]	ENSG00000119772	protein_coding	3'UTR	3'UTR	1	3424	2:25282305:25282365	ENSG00000119772	DNMT3A	6.786866395	0.009561294	2.505919712	0.905945419	2.627438924	-1.599974294	0.121519212	1.721493506	9.961963646	0.031211364	0.386352109	0.025347703	0.140803919	0.905696044	0.1160093	-4.347425823	-5.767024585	-4.189537498	-5.904597281	-6.288836939	-5.655484904	3.257005093	0.068227396

EXOC4	-0.087913571	-0.095688924	-0.043667823	-0.007775353	0.044245748	0.052021101	8	0.061828823	0.816496581	0.816496581	0.816496581	0.816496581	0.816496581	0.816496581	21	25	0	P_262942	P_262942	P_262942	chr7	133400565	133400625	+	EXOC4	exocyst complex component 4 [Source:HGNC Symbol;Acc:HGNC:30389]	ENSG00000131558	protein_coding	intron	intron sense	0	3425	7:133400565:133400625	ENSG00000131558	EXOC4	6.610173707	0.005276604	-1.482259753	-1.613355477	-0.736257852	-0.131095724	0.746001901	0.877097624	9.961963646	0.169198273	0.137858906	0.47855233	0.898309251	0.472902405	0.40109054	-5.915874834	-4.969371556	-6.758918698	-7.140222713	-6.008917724	-6.666653777	1.114181777	0.388900439

BEX4	-0.033736138	0.138535505	-0.186227638	0.172271643	-0.1524915	-0.324763143	8	0.118831396	0.816496581	0.816496581	0.816496581	0.816496581	0.816496581	0.816496581	21	26	0	P_151383	P_151383	P_151383	chrX	103216778	103216838	-	BEX4	"brain expressed, X-linked 4 [Source:HGNC Symbol;Acc:HGNC:25475]"	ENSG00000102409	protein_coding	exon	exon antisense	0	3426	X:103216778:103216838	ENSG00000102409	BEX4	6.323645277	0.013546537	-0.354998766	1.457781974	-1.959636939	1.81278074	-1.604638173	-3.417418913	9.961963646	0.729991251	0.175692421	0.078593717	0.100062426	0.139771177	0.006611761	-6.930024817	-5.170965242	-5.275697456	-5.597993083	-5.083557378	-2.86443343	3.938519841	0.043173227

TRHDE	0.017599388	0.138244633	0.17180433	0.120645245	0.154204942	0.033559698	8	0.081986996	0.816496581	0.816496581	0.816496581	0.816496581	0.816496581	0.816496581	21	28	0	A_23_P366983	A_23_P366983	A_23_P366983	chr12	72665040	72665099	+	TRHDE	thyrotropin-releasing hormone degrading enzyme [Source:HGNC Symbol;Acc:HGNC:30748]	ENSG00000072657	protein_coding	3'UTR	3'UTR	0	3428	12:72665040:72665099	ENSG00000072657	TRHDE	3.696219705	0.00760471	0.247173478	1.941567873	2.412894893	1.694394395	2.165721416	0.471327021	9.961963646	0.809793355	0.080981487	0.036589639	0.121169853	0.055671131	0.647559543	-6.965124046	-4.509176164	-4.548346906	-5.771140777	-4.267178929	-6.951360457	2.906052179	0.087769684

EDA	-0.288347937	-0.027313031	-0.299378195	0.261034906	-0.011030258	-0.272065164	8	0.054067954	0.816496581	0.816496581	0.816496581	0.816496581	0.816496581	0.816496581	21	30	0	P_151115	P_151115	P_151115	chrX	69617131	69617191	+	EDA	ectodysplasin A [Source:HGNC Symbol;Acc:HGNC:3157]	ENSG00000158813	protein_coding	exon	coding exon	1	3430	X:69617131:69617191	ENSG00000158813	EDA	7.527816382	0.004554288	-5.233017844	-0.495684422	-5.433198006	4.737333421	-0.200180163	-4.937513584	9.961963646	0.000387434	0.630869113	0.000291442	0.000803693	0.845367108	0.000596031	0.122726451	-6.054197	0.381368166	-0.758806961	-6.275415619	-0.382264147	17.33520997	0.000279593

PHACTR4	-0.050305614	-0.205600855	-0.207283747	-0.155295241	-0.156978133	-0.001682892	8	0.072189032	0.816496581	0.816496581	0.816496581	0.816496581	0.816496581	0.816496581	21	31	0	P_200833	P_200833	P_200833	chr1	28490147	28490207	-	PHACTR4	phosphatase and actin regulator 4 [Source:HGNC Symbol;Acc:HGNC:25793]	ENSG00000204138	protein_coding	intron	intron antisense	0	3431	1:28490147:28490207	ENSG00000204138	PHACTR4	7.116156591	0.006391607	-0.770649771	-3.149673347	-3.175454177	-2.379023576	-2.404804406	-0.02578083	9.961963646	0.458801473	0.010385696	0.009941375	0.038766146	0.037098362	0.979941277	-6.683642047	-2.618126497	-3.25565234	-4.702568833	-3.892602489	-7.071015436	5.340225759	0.01879958

SCAMP1	0.149450423	0.016528491	0.131797184	-0.132921932	-0.017653239	0.115268692	8	0.056996801	0.816496581	0.816496581	0.816496581	0.816496581	0.816496581	0.816496581	21	32	0	P_253574	P_253574	P_253574	chr5	78478145	78478205	-	SCAMP1	secretory carrier membrane protein 1 [Source:HGNC Symbol;Acc:HGNC:10563]	ENSG00000085365	protein_coding	3'ext;3'UTR	multiple non-Ref	0	3432	5:78478145:78478205	ENSG00000085365	SCAMP1	7.842220234	0.004815515	2.637675673	0.291714124	2.326110679	-2.345961549	-0.311564994	2.034396555	9.961963646	0.024906952	0.776489399	0.042422154	0.041012707	0.761793709	0.069400771	-4.126400509	-6.137425118	-4.691382441	-4.757137117	-6.24531181	-5.183062876	3.698766443	0.050488219

NASP	-0.112948771	-0.222695276	-0.155704112	-0.109746505	-0.04275534	0.066991164	8	0.155212165	0.816496581	0.816496581	0.816496581	0.816496581	0.816496581	0.816496581	21	33	0	P_308455	P_308455	P_308455	chr1	45609235	45609295	+	NASP	nuclear autoantigenic sperm protein (histone-binding) [Source:HGNC Symbol;Acc:HGNC:7644]	ENSG00000132780	protein_coding	3'ext;intron;promoter	multiple non-Ref	0	3433	1:45609235:45609295	ENSG00000132780	NASP	7.51268568	0.021552923	-0.942267153	-1.857819623	-1.298950563	-0.915552469	-0.35668341	0.55886906	9.961963646	0.368344734	0.092960099	0.223220244	0.381529597	0.728768038	0.588592598	-6.53441262	-4.630461891	-6.196411946	-6.709588003	-6.229459712	-6.903387765	1.217404618	0.353614356

TANC1	0.168601863	-0.008446634	0.100291046	-0.177048498	-0.068310817	0.10873768	8	0.060925058	0.816496581	0.816496581	0.816496581	0.816496581	0.816496581	0.816496581	21	34	0	P_129065	P_129065	P_129065	chr2	159227750	159227810	+	TANC1	"tetratricopeptide repeat, ankyrin repeat and coiled-coil containing 1 [Source:HGNC Symbol;Acc:HGNC:29364]"	ENSG00000115183	protein_coding	exon	non-coding exon	1	3434	2:159227750:159227810	ENSG00000115183	TANC1	8.623802603	0.005187512	2.867004903	-0.14363152	1.705407728	-3.010636423	-1.161597175	1.849039249	9.961963646	0.016815673	0.888655327	0.119048012	0.013157021	0.272474468	0.094307039	-3.737634316	-6.171223581	-5.65499915	-3.633320468	-5.625103347	-5.467463114	4.16349141	0.037430285

SNTG2	-0.447710464	-0.148307246	0.052310714	0.299403217	0.500021178	0.200617961	8	0.383363889	0.816496581	0.816496581	0.816496581	0.816496581	0.816496581	0.816496581	21	37	0	P_233448	P_233448	P_233448	chr2	1259377	1259437	+	SNTG2	"syntrophin, gamma 2 [Source:HGNC Symbol;Acc:HGNC:13741]"	ENSG00000172554	protein_coding	exon	coding exon	1	3437	2:1259377:1259437	ENSG00000172554	SNTG2	4.534439805	0.120229899	-1.581381427	-0.523843743	0.184769396	1.057537684	1.766150822	0.708613139	9.961963646	0.144988609	0.611841902	0.857117259	0.315236113	0.107938923	0.494812116	-5.782031916	-6.039463434	-7.028508657	-6.57015859	-4.860434532	-6.803798597	1.258187745	0.340661992

QSOX1	0.143631382	0.049076103	0.084188267	-0.094555279	-0.059443114	0.035112165	8	0.123084819	0.816496581	0.816496581	0.816496581	0.816496581	0.816496581	0.816496581	21	38	0	P_205191	P_205191	P_205191	chr1	180178812	180178872	+	QSOX1	quiescin Q6 sulfhydryl oxidase 1 [Source:HGNC Symbol;Acc:HGNC:9756]	ENSG00000116260	protein_coding	"3'UTR,exon;exon"	multiple Ref	1	3438	1:180178812:180178872	ENSG00000116260	QSOX1	9.171335393	0.014372858	1.467315149	0.501353591	0.860053833	-0.965961558	-0.607261316	0.358700242	9.961963646	0.173138054	0.627015003	0.409979665	0.356927573	0.557264658	0.727304675	-5.935583191	-6.051292787	-6.657566663	-6.661980259	-6.104204328	-7.001541152	0.762429278	0.540565802

PTPMT1	0.066008801	0.151609377	0.131756373	0.085600575	0.065747571	-0.019853004	8	0.055650042	0.816496581	0.816496581	0.816496581	0.816496581	0.816496581	0.816496581	21	41	0	P_108490	P_108490	P_108490	chr11	47572230	47572290	+	PTPMT1	"protein tyrosine phosphatase, mitochondrial 1 [Source:HGNC Symbol;Acc:HGNC:26965]"	ENSG00000110536	protein_coding	3'UTR;exon	multiple Ref	1	3441	11:47572230:47572290	ENSG00000110536	PTPMT1	11.7394088	0.004693685	1.18002304	2.710283382	2.355376137	1.530260342	1.175353097	-0.354907245	9.961963646	0.265401423	0.021992846	0.040360339	0.157065729	0.267180098	0.730057727	-6.287529213	-3.321084435	-4.643307745	-6.000233047	-5.610134113	-7.003001046	3.022499651	0.080627628

PRICKLE2	-0.183335306	0.012509946	-0.13892699	0.195845252	0.044408316	-0.151436936	8	0.193601527	0.816496581	0.816496581	0.816496581	0.816496581	0.816496581	0.816496581	21	42	0	P_312302	P_312302	P_312302	chr3	64175634	64175694	+	PRICKLE2	prickle homolog 2 (Drosophila) [Source:HGNC Symbol;Acc:HGNC:20340]	ENSG00000163637	protein_coding	intron	intron antisense	0	3442	3:64175634:64175694	ENSG00000163637	PRICKLE2	3.668066045	0.032306413	-1.249244266	0.085242601	-0.946646607	1.334486868	0.302597659	-1.031889208	9.961963646	0.240130952	0.933757348	0.366214883	0.211743276	0.768420375	0.326525099	-6.207707644	-6.178258291	-6.578658407	-6.254085683	-6.248212736	-6.518918473	0.900205536	0.474852406

CWC27	0.108943205	-0.013360548	0.075907456	-0.122303753	-0.033035749	0.089268004	8	0.053379034	0.816496581	0.816496581	0.816496581	0.816496581	0.816496581	0.816496581	21	43	0	P_253071	P_253071	P_253071	chr5	64977178	64977238	+	CWC27	CWC27 spliceosome-associated protein homolog (S. cerevisiae) [Source:HGNC Symbol;Acc:HGNC:10664]	ENSG00000153015	protein_coding	exon	coding exon	1	3443	5:64977178:64977238	ENSG00000153015	CWC27	9.254594343	0.004494844	1.99016211	-0.244068969	1.386668788	-2.234231078	-0.603493321	1.630737757	9.961963646	0.074709501	0.81213047	0.195787315	0.049583572	0.559669978	0.134115243	-5.181407145	-6.150781417	-6.087638158	-4.939873241	-6.106541895	-5.784855797	2.326410591	0.136688167

COL4A6	-0.263587091	0.217638725	0.012065576	0.481225817	0.275652667	-0.205573149	8	0.131291495	0.816496581	0.816496581	0.816496581	0.816496581	0.816496581	0.816496581	21	45	0	P_271098	P_271098	P_271098	chrX	108176940	108177000	-	COL4A6	"collagen, type IV, alpha 6 [Source:HGNC Symbol;Acc:HGNC:2208]"	ENSG00000197565	protein_coding	exon	coding exon	1	3445	X:108176940:108177000	ENSG00000197565	COL4A6	4.297773301	0.016049302	-2.548248028	2.104038747	0.116644866	4.652286776	2.664892894	-1.987393881	9.961963646	0.029030168	0.061761276	0.909459521	0.000914112	0.023771926	0.075054156	-4.27665905	-4.267777937	-7.03969532	-0.892950128	-3.47543437	-5.256305319	7.271552318	0.007188708

PON1	0.05513057	0.269948414	0.06346955	0.214817844	0.00833898	-0.206478865	8	0.25084778	0.816496581	0.816496581	0.816496581	0.816496581	0.816496581	0.816496581	21	46	0	P_261890	P_261890	P_261890	chr7	95306284	95306344	-	PON1	paraoxonase 1 [Source:HGNC Symbol;Acc:HGNC:9204]	ENSG00000005421	protein_coding	3'UTR;exon	multiple Ref	1	3446	7:95306284:95306344	ENSG00000005421	PON1	3.284829636	0.052738576	0.294017994	1.439667534	0.338490782	1.145649541	0.044472789	-1.101176752	9.961963646	0.774778948	0.180635587	0.742019212	0.278715834	0.965406365	0.296714304	-6.951392255	-5.193619463	-6.984909211	-6.475608552	-6.295633384	-6.446517306	0.800124356	0.521719419

CDH23	0.104444203	0.017972875	0.018395853	-0.086471329	-0.086048351	0.000422978	8	0.082789482	0.816496581	0.816496581	0.816496581	0.816496581	0.816496581	0.816496581	21	47	0	P_208597	P_208597	P_208597	chr10	71702589	71702649	+	CDH23	cadherin-related 23 [Source:HGNC Symbol;Acc:HGNC:13733]	ENSG00000107736	protein_coding	exon	coding exon	1	3447	10:71702589:71702649	ENSG00000107736	CDH23	5.403216367	0.007710899	1.456724667	0.250674799	0.256574244	-1.206049868	-1.200150423	0.005899445	9.961963646	0.175977712	0.807159868	0.802728386	0.255660646	0.257843076	0.995409418	-5.949471953	-6.149069479	-7.011289119	-6.407458646	-5.582818315	-7.071359512	0.857611179	0.494246406

FUBP3	-0.005015639	-0.045780848	-0.073400254	-0.040765209	-0.068384615	-0.027619406	8	0.040601184	0.816496581	0.816496581	0.816496581	0.816496581	0.816496581	0.816496581	21	48	0	P_269185	P_269185	P_269185	chr9	130612967	130613027	+	FUBP3	far upstream element (FUSE) binding protein 3 [Source:HGNC Symbol;Acc:HGNC:4005]	ENSG00000107164	protein_coding	exon	coding exon	1	3448	9:130612967:130613027	ENSG00000107164	FUBP3	7.527509194	0.003530484	-0.103384347	-0.943653015	-1.512955167	-0.840268668	-1.40957082	-0.569302152	9.961963646	0.919709575	0.367669788	0.161349328	0.420467179	0.189112155	0.581759312	-6.992530935	-5.733286746	-5.922804924	-6.776645039	-5.336156151	-6.897158051	1.034541491	0.418760759

C3orf20	0.130150892	0.060853721	0.118493353	-0.069297171	-0.011657539	0.057639632	8	0.116930828	0.816496581	0.816496581	0.816496581	0.816496581	0.816496581	0.816496581	21	49	0	P_243307	P_243307	P_243307	chr3	14726964	14727024	+	C3orf20	chromosome 3 open reading frame 20 [Source:HGNC Symbol;Acc:HGNC:25320]	ENSG00000131379	protein_coding	exon	coding exon	1	3449	3:14726964:14727024	ENSG00000131379	C3orf20	4.458783388	0.013186703	1.388112516	0.649029835	1.263780093	-0.739082681	-0.124332423	0.614750257	9.961963646	0.195360696	0.530996418	0.235080626	0.476910021	0.903525075	0.552501388	-6.037851242	-5.964779324	-6.238616942	-6.858840975	-6.288469645	-6.868767452	0.814142641	0.514880112

GFOD2	0.031898559	0.087938248	0.05277778	0.056039688	0.020879221	-0.035160467	8	0.085811584	0.816496581	0.816496581	0.816496581	0.816496581	0.816496581	0.816496581	21	51	0	A_23_P3574	A_23_P3574	A_23_P3574	chr16	67674749	67674809	-	GFOD2	glucose-fructose oxidoreductase domain containing 2 [Source:HGNC Symbol;Acc:HGNC:28159]	ENSG00000141098	protein_coding	3'UTR;exon	multiple Ref	1	3451	16:67674749:67674809	ENSG00000141098	GFOD2	8.316976505	0.008120079	0.433547393	1.195207513	0.717326092	0.761660121	0.283778699	-0.477881421	9.961963646	0.673854974	0.259683122	0.489650808	0.463912632	0.782390404	0.643047543	-6.896753646	-5.480398482	-6.773184584	-6.84130899	-6.254030167	-6.94803732	0.503344701	0.688535649

ANGPTL6	0.212831342	0.277799971	0.283896198	0.064968629	0.071064856	0.006096227	8	0.125458088	0.816496581	0.816496581	0.816496581	0.816496581	0.816496581	0.816496581	21	54	0	P_124541	P_124541	P_124541	chr19	10092605	10092665	-	ANGPTL6	angiopoietin-like 6 [Source:HGNC Symbol;Acc:HGNC:23140]	ENSG00000130812	protein_coding	exon	coding exon	1	3454	19:10092605:10092665	ENSG00000130812	ANGPTL6	6.239230282	0.014846547	2.139284206	2.792319423	2.853595931	0.653035217	0.714311725	0.061276508	9.961963646	0.058207402	0.019109094	0.017205953	0.52851601	0.491432636	0.952350934	-4.946955747	-3.190034428	-3.805842426	-6.921282131	-6.032239734	-7.069327648	3.576174244	0.054795263

SCAF8	0.018382689	-0.115736282	-0.096626546	-0.13411897	-0.115009235	0.019109735	8	0.036268126	0.816496581	0.816496581	0.816496581	0.816496581	0.816496581	0.816496581	21	55	0	A_23_P111306	A_23_P111306	A_23_P111306	chr6	154833376	154833436	+	SCAF8	SR-related CTD-associated factor 8 [Source:HGNC Symbol;Acc:HGNC:20959]	ENSG00000213079	protein_coding	"3'UTR,exon"	multiple Ref	1	3455	6:154833376:154833436	ENSG00000213079	SCAF8	7.7483092	0.003263003	0.394135812	-2.481454936	-2.071730808	-2.875590747	-2.465866619	0.409724128	9.961963646	0.701785838	0.03254515	0.065200141	0.016570483	0.033424186	0.690679692	-6.914250318	-3.684248588	-5.100959879	-3.864610705	-3.79537411	-6.980438002	4.187070727	0.036882707

ARHGAP31	-0.0621622	0.09757322	0.088238314	0.15973542	0.150400514	-0.009334905	8	0.097315646	0.816496581	0.816496581	0.816496581	0.816496581	0.816496581	0.816496581	21	56	0	P_245675	P_245675	P_245675	chr3	119383166	119383226	+	ARHGAP31	Rho GTPase activating protein 31 [Source:HGNC Symbol;Acc:HGNC:29216]	ENSG00000031081	protein_coding	exon	coding exon	1	3456	3:119383166:119383226	ENSG00000031081	ARHGAP31	3.943651228	0.009811879	-0.768592081	1.206424544	1.091004976	1.975016626	1.859597057	-0.115419568	9.961963646	0.459968164	0.255522541	0.300954817	0.076613567	0.092689611	0.910405945	-6.685272618	-5.468067321	-6.433350611	-5.351515602	-4.726415369	-7.064105743	1.768099898	0.216804214

KCNE3	-0.282843214	0.209069977	0.074291896	0.491913191	0.35713511	-0.134778081	8	0.300198853	0.816496581	0.816496581	0.816496581	0.816496581	0.816496581	0.816496581	21	58	0	A_23_P24948	A_23_P24948	A_23_P24948	chr11	74455440	74455500	-	KCNE3	"potassium voltage-gated channel, Isk-related family, member 3 [Source:HGNC Symbol;Acc:HGNC:6243]"	ENSG00000175538	protein_coding	3'UTR	3'UTR	1	3458	11:74455440:74455500	ENSG00000175538	KCNE3	3.677949523	0.074577437	-1.268491807	0.937634489	0.333183391	2.206126296	1.601675199	-0.604451098	9.961963646	0.233462344	0.370607413	0.745901982	0.051998951	0.140426498	0.559058024	-6.184922466	-5.738748479	-6.986834048	-4.985392616	-5.087539945	-6.8753788	1.73282648	0.223481192

FOXRED1	-0.25461992	-0.082456233	-0.277257587	0.172163688	-0.022637667	-0.194801355	8	0.074550748	0.816496581	0.816496581	0.816496581	0.816496581	0.816496581	0.816496581	21	59	0	P_213824	P_213824	P_213824	chr11	126271457	126271517	+	FOXRED1	FAD-dependent oxidoreductase domain containing 1 [Source:HGNC Symbol;Acc:HGNC:26927]	ENSG00000110074	protein_coding	exon	coding exon	1	3459	11:126271457:126271517	ENSG00000110074	FOXRED1	8.513426639	0.006669912	-3.818369617	-1.236542579	-4.157852005	2.581827038	-0.339482388	-2.921309427	9.961963646	0.003405986	0.244616045	0.001971498	0.027407894	0.741294613	0.015324461	-2.121864056	-5.434542669	-1.601878502	-4.363813072	-6.235749802	-3.712926849	8.118640882	0.004964384

JOSD2	-0.230348744	0.130886465	-0.050435792	0.361235209	0.179912952	-0.181322257	8	0.194715315	0.816496581	0.816496581	0.816496581	0.816496581	0.816496581	0.816496581	21	60	0	P_233208	P_233208	P_233208	chr19	50506172	50506232	-	JOSD2	Josephin domain containing 2 [Source:HGNC Symbol;Acc:HGNC:28853]	ENSG00000161677	protein_coding	exon	coding exon	1	3460	19:50506172:50506232	ENSG00000161677	JOSD2	8.05683485	0.032653736	-1.561223343	0.88710275	-0.341836187	2.448326093	1.219387156	-1.228938937	9.961963646	0.149649513	0.39593514	0.739575677	0.03444131	0.250781368	0.247333332	-5.809678749	-5.783450192	-6.983680658	-4.587538093	-5.561338436	-6.303566113	2.055453034	0.170234808

UCHL5	-0.521891005	0.037604908	-0.403378817	0.559495914	0.118512188	-0.440983725	8	0.037523333	0.816496581	0.816496581	0.816496581	0.816496581	0.816496581	0.816496581	21	61	0	P_205563	P_205563	P_205563	chr1	193029178	193029238	-	UCHL5	ubiquitin carboxyl-terminal hydrolase L5 [Source:HGNC Symbol;Acc:HGNC:19678]	ENSG00000116750	protein_coding	exon	coding exon	1	3461	1:193029178:193029238	ENSG00000116750	UCHL5	10.88753608	0.003337385	-11.06425752	0.797236178	-8.551760934	11.8614937	2.512496589	-9.348997112	9.961963646	6.46E-07	0.443900325	6.70E-06	3.38E-07	0.030862109	3.02E-06	6.655619412	-5.857707799	4.288577172	7.28916998	-3.720793465	5.09395501	71.76623568	4.76E-07

NSF	-0.095967458	0.18343016	0.124923846	0.279397618	0.220891304	-0.058506314	8	0.052038337	0.816496581	0.816496581	0.816496581	0.816496581	0.816496581	0.816496581	21	62	0	P_229008	P_229008	P_229008	chr17	46726555	46726615	+	NSF	N-ethylmaleimide-sensitive factor [Source:HGNC Symbol;Acc:HGNC:8016]	ENSG00000073969	protein_coding	exon	coding exon	1	3462	17:46726555:46726615	ENSG00000073969	NSF	8.85662034	0.004381346	-1.775684584	3.394005764	2.311464226	5.169690348	4.08714881	-1.082541538	9.961963646	0.106283777	0.006876406	0.043492245	0.000424453	0.002206564	0.304518582	-5.505344906	-2.229419899	-4.715377572	-0.092375328	-1.197418054	-6.466355613	10.76959627	0.0018101

FAM118B	-0.031493168	0.115178081	0.003552202	0.146671249	0.03504537	-0.111625879	8	0.096572542	0.816496581	0.816496581	0.816496581	0.816496581	0.816496581	0.816496581	21	64	0	P_110466	P_110466	P_110466	chr11	126256789	126256849	+	FAM118B	"family with sequence similarity 118, member B [Source:HGNC Symbol;Acc:HGNC:26110]"	ENSG00000197798	protein_coding	exon	coding exon	1	3464	11:126256789:126256849	ENSG00000197798	FAM118B	8.131798131	0.009696176	-0.391707366	1.432567926	0.044181758	1.824275292	0.435889124	-1.388386168	9.961963646	0.703522761	0.182605277	0.965632584	0.098203726	0.672211119	0.195279921	-6.915275681	-5.202449085	-7.046054685	-5.580863021	-6.196610433	-6.109346067	1.275538895	0.335312393

TNFSF15	-0.009062104	0.269390952	0.071143049	0.278453056	0.080205153	-0.198247903	8	0.147761869	0.816496581	0.816496581	0.816496581	0.816496581	0.816496581	0.816496581	21	67	0	A_32_P158181	A_32_P158181	A_32_P158181	chr9	114784706	114784766	-	TNFSF15	"tumor necrosis factor (ligand) superfamily, member 15 [Source:HGNC Symbol;Acc:HGNC:11931]"	ENSG00000181634	protein_coding	3'UTR	3'UTR	1	3467	9:114784706:114784766	ENSG00000181634	TNFSF15	6.769535114	0.019740231	-0.078994808	2.34829427	0.620157479	2.427289078	0.699152287	-1.728136791	9.961963646	0.938601016	0.040850112	0.549076584	0.035701426	0.500454378	0.114774992	-6.994956175	-3.892958335	-6.841071348	-4.622544344	-6.043074375	-5.645864646	2.545387402	0.115147664

GRID1	0.052903782	0.056162989	0.395437261	0.003259207	0.342533479	0.339274272	8	0.185724195	0.816496581	0.816496581	0.816496581	0.816496581	0.816496581	0.816496581	21	68	0	P_106486	P_106486	P_106486	chr10	85647317	85647377	-	GRID1	"glutamate receptor, ionotropic, delta 1 [Source:HGNC Symbol;Acc:HGNC:4575]"	ENSG00000182771	protein_coding	exon	coding exon	1	3468	10:85647317:85647377	ENSG00000182771	GRID1	3.787248957	0.029906826	0.374668501	0.397750447	2.80051597	0.023081945	2.425847469	2.402765524	9.961963646	0.71575952	0.699203842	0.018842718	0.982040704	0.035789419	0.037227656	-6.922297026	-6.099368733	-3.89624284	-7.149321714	-3.859155526	-4.588260476	3.300003747	0.066206591

ERRFI1	-0.015644745	-0.033177762	-0.105055263	-0.017533017	-0.089410518	-0.071877501	8	0.085692309	0.816496581	0.816496581	0.816496581	0.816496581	0.816496581	0.816496581	21	69	0	P_100300	P_100300	P_100300	chr1	8005355	8005415	+	ERRFI1	ERBB receptor feedback inhibitor 1 [Source:HGNC Symbol;Acc:HGNC:18185]	ENSG00000116285	protein_coding	intron	intron antisense	0	3469	1:8005355:8005415	ENSG00000116285	ERRFI1	5.276966993	0.008103651	-0.212850039	-0.45139042	-1.429298927	-0.238540381	-1.216448888	-0.977908507	9.961963646	0.835736365	0.66137549	0.183518384	0.816297246	0.251849802	0.351269535	-6.973694161	-6.075803479	-6.033043206	-7.118584597	-5.564635525	-6.572690456	0.797484722	0.523017471

EPX	-0.102203079	0.0761289	-0.159259741	0.178331979	-0.057056661	-0.23538864	8	0.180238796	0.816496581	0.816496581	0.816496581	0.816496581	0.816496581	0.816496581	21	70	0	P_229301	P_229301	P_229301	chr17	58203172	58203232	+	EPX	eosinophil peroxidase [Source:HGNC Symbol;Acc:HGNC:3423]	ENSG00000121053	protein_coding	exon	coding exon	1	3470	17:58203172:58203232	ENSG00000121053	EPX	3.94672409	0.028294732	-0.744143712	0.55429682	-1.159574989	1.298440532	-0.415431277	-1.713871809	9.961963646	0.47397656	0.591600712	0.273259718	0.223388645	0.68663255	0.117440218	-6.704349194	-6.022666436	-6.358655184	-6.298248683	-6.205702799	-5.666497467	1.166916087	0.370411663

EIF3I	-0.163333919	-0.032409418	-0.095379349	0.130924501	0.06795457	-0.062969931	8	0.065433656	0.816496581	0.816496581	0.816496581	0.816496581	0.816496581	0.816496581	21	75	0	P_200920	P_200920	P_200920	chr1	32228729	32228789	+	EIF3I	"eukaryotic translation initiation factor 3, subunit I [Source:HGNC Symbol;Acc:HGNC:3272]"	ENSG00000084623	protein_coding	exon	coding exon	1	3475	1:32228729:32228789	ENSG00000084623	EIF3I	11.32200658	0.005645013	-2.662499564	-0.528304598	-1.554774885	2.134194966	1.107724679	-1.026470287	9.961963646	0.023869628	0.608854799	0.151168145	0.058708194	0.294009065	0.328948642	-4.084531654	-6.03705884	-5.866229502	-5.100984959	-5.682436949	-6.524422223	2.770136332	0.097074032

BANP	0.092984569	-0.143838338	0.00619623	-0.236822907	-0.086788339	0.150034568	8	0.130735386	0.816496581	0.816496581	0.816496581	0.816496581	0.816496581	0.816496581	21	77	0	P_310310	P_310310	P_310310	chr16	87948435	87948495	+	BANP;CTD-3057O21.1	BTG3 associated nuclear protein [Source:HGNC Symbol;Acc:HGNC:13450];	ENSG00000172530	protein_coding;processed_pseudogene	promoter	multiple non-Ref	0	3477	16:87948435:87948495	ENSG00000172530	BANP	7.487631378	0.015932284	0.902230474	-1.39566525	0.060122097	-2.297895724	-0.842108377	1.455787347	9.961963646	0.388228415	0.193141692	0.953247411	0.044507002	0.419484377	0.176230961	-6.571513556	-5.247888768	-7.045146957	-4.836079963	-5.932745674	-6.022422932	1.812384011	0.208745218

ALB	0.39513086	0.215383552	0.692597767	-0.179747307	0.297466907	0.477214215	8	0.13947454	0.816496581	0.816496581	0.816496581	0.816496581	0.816496581	0.816496581	21	78	0	P_249375	P_249375	P_249375	chr4	73416293	73416353	+	ALB	albumin [Source:HGNC Symbol;Acc:HGNC:399]	ENSG00000163631	protein_coding	3'UTR;exon	multiple Ref	1	3478	4:73416293:73416353	ENSG00000163631	ALB	8.025913613	0.017828622	3.62432963	1.975601176	6.352838679	-1.648728455	2.728509048	4.377237503	9.961963646	0.004685003	0.076539238	8.46E-05	0.130336397	0.021316574	0.001395938	-2.447850676	-4.459240637	1.667825622	-5.836229497	-3.372450062	-1.265771414	14.45343331	0.000583496

DUOXA1	0.174792003	-0.102452898	0.129017904	-0.277244901	-0.0457741	0.231470802	8	0.122260892	0.816496581	0.816496581	0.816496581	0.816496581	0.816496581	0.816496581	21	79	0	P_116413	P_116413	P_116413	chr15	45117745	45117805	-	DUOXA1	dual oxidase maturation factor 1 [Source:HGNC Symbol;Acc:HGNC:26507]	ENSG00000140254	protein_coding	3'UTR;exon	multiple Ref	1	3479	15:45117745:45117805	ENSG00000140254	DUOXA1	5.450510518	0.014210523	1.795817348	-1.052603595	1.325533122	-2.848420943	-0.470284226	2.378136717	9.961963646	0.102864634	0.317384528	0.214587686	0.01735901	0.648278785	0.038824806	-5.475699665	-5.629514465	-6.163969412	-3.911052593	-6.180378957	-4.628973375	3.34669595	0.0640921

RNF187	-0.015431919	0.122154477	0.174993264	0.137586396	0.190425183	0.052838787	8	0.080563931	0.816496581	0.816496581	0.816496581	0.816496581	0.816496581	0.816496581	21	82	0	P_105025	P_105025	P_105025	chr1	228493081	228493141	+	RNF187	ring finger protein 187 [Source:HGNC Symbol;Acc:HGNC:27146]	ENSG00000168159	protein_coding	exon	coding exon	1	3482	1:228493081:228493141	ENSG00000168159	RNF187	9.410600651	0.007418947	-0.219429208	1.736936266	2.488260389	1.956365474	2.707689597	0.751324123	9.961963646	0.830746451	0.113158433	0.032168576	0.079021076	0.022090822	0.469834397	-6.972149496	-4.801096322	-4.423059321	-5.380340336	-3.406182044	-6.771476766	3.496662215	0.057822842

LCK	0.014093385	0.165901708	0.087753804	0.151808323	0.073660419	-0.078147904	8	0.170805023	0.816496581	0.816496581	0.816496581	0.816496581	0.816496581	0.816496581	21	84	0	A_23_P103361	A_23_P103361	A_23_P103361	chr1	32285830	32285890	+	LCK	"LCK proto-oncogene, Src family tyrosine kinase [Source:HGNC Symbol;Acc:HGNC:6524]"	ENSG00000182866	protein_coding	3'UTR;exon	multiple Ref	1	3484	1:32285830:32285890	ENSG00000182866	LCK	4.22148839	0.025635282	0.107805738	1.269046197	0.671262716	1.161240459	0.563456978	-0.597783481	9.961963646	0.916290134	0.233272535	0.517314923	0.272612857	0.585582402	0.563325958	-6.992021967	-5.397696488	-6.806489687	-6.458271055	-6.130542484	-6.87960362	0.682667037	0.582675329

TTLL3	-0.107660731	-0.14253703	-0.141867623	-0.0348763	-0.034206892	0.000669408	8	0.069486928	0.816496581	0.816496581	0.816496581	0.816496581	0.816496581	0.816496581	21	85	0	A_16_P00646559	A_16_P00646559	A_16_P00646559	chr3	9835369	9835426	+	TTLL3	"tubulin tyrosine ligase-like family, member 3 [Source:HGNC Symbol;Acc:HGNC:24483]"	ENSG00000214021	protein_coding	3'UTR;exon	multiple Ref	1	3485	3:9835369:9835426	ENSG00000214021	TTLL3	6.720272838	0.006084179	-1.690448557	-2.238063181	-2.227552392	-0.547614624	-0.537103835	0.010510789	9.961963646	0.121938337	0.049262753	0.050147538	0.596011629	0.602984707	0.99182127	-5.628899995	-4.063605686	-4.851955342	-6.988052278	-6.145498466	-7.071318172	2.236167595	0.146920525

SASH1	-0.076160508	0.020383038	-0.075833827	0.096543547	0.000326682	-0.096216865	8	0.07963434	0.816496581	0.816496581	0.816496581	0.816496581	0.816496581	0.816496581	21	87	0	P_313291	P_313291	P_313291	chr6	148508708	148508768	-	SASH1	SAM and SH3 domain containing 1 [Source:HGNC Symbol;Acc:HGNC:19182]	ENSG00000111961	protein_coding	intron	intron antisense	0	3487	6:148508708:148508768	ENSG00000111961	SASH1	5.209386031	0.007299357	-1.091775033	0.292194635	-1.087091991	1.383969669	0.004683042	-1.379286626	9.961963646	0.300632163	0.776132551	0.30259848	0.196587017	0.996355939	0.197981075	-6.384268931	-6.137278514	-6.437506181	-6.192103278	-6.296673592	-6.120870447	1.0461575	0.414254707

EPB41L2	0.083556202	-0.32407968	-0.195239877	-0.407635883	-0.278796079	0.128839803	8	0.091499104	0.816496581	0.816496581	0.816496581	0.816496581	0.816496581	0.816496581	21	88	0	P_259182	P_259182	P_259182	chr6	130904464	130904524	-	EPB41L2	erythrocyte membrane protein band 4.1-like 2 [Source:HGNC Symbol;Acc:HGNC:3379]	ENSG00000079819	protein_coding	exon	coding exon	1	3488	6:130904464:130904524	ENSG00000079819	EPB41L2	6.209900083	0.008929926	1.082930044	-4.200234257	-2.530406161	-5.283164301	-3.613336205	1.669828096	9.961963646	0.304354284	0.001843361	0.029930529	0.000360571	0.004771007	0.126026289	-6.393642731	-0.987722364	-4.352615273	0.078143921	-1.940232418	-5.729611788	11.49566835	0.00141776

SUN5	-0.049301276	-0.069015382	0.230954193	-0.019714107	0.280255468	0.299969575	8	0.252875765	0.816496581	0.816496581	0.816496581	0.816496581	0.816496581	0.816496581	21	89	0	P_130955	P_130955	P_130955	chr20	32989619	32989679	-	SUN5	Sad1 and UNC84 domain containing 5 [Source:HGNC Symbol;Acc:HGNC:16252]	ENSG00000167098	protein_coding	exon	coding exon	1	3489	20:32989619:32989679	ENSG00000167098	SUN5	4.79916734	0.053558931	-0.260908263	-0.36523768	1.222237287	-0.104329417	1.48314555	1.587474967	9.961963646	0.799477463	0.722569202	0.24974848	0.918978525	0.16896721	0.143605161	-6.96134072	-6.112249684	-6.287386527	-7.143663894	-5.243257822	-5.8451138	1.071002965	0.404791794

M1AP	-0.029084926	-0.011468817	0.151754083	0.017616109	0.180839009	0.163222899	8	0.259339094	0.816496581	0.816496581	0.816496581	0.816496581	0.816496581	0.816496581	21	90	0	P_128220	P_128220	P_128220	chr2	74581673	74581733	-	M1AP	meiosis 1 associated protein [Source:HGNC Symbol;Acc:HGNC:25183]	ENSG00000159374	protein_coding	3'UTR;exon	multiple Ref	1	3490	2:74581673:74581733	ENSG00000159374	M1AP	4.644950123	0.056217534	-0.150237275	-0.059241814	0.783880961	0.090995461	0.934118236	0.843122775	9.961963646	0.883575747	0.953931057	0.451345422	0.929299884	0.372331464	0.418943138	-6.986057415	-6.180237606	-6.72156869	-7.145086806	-5.852423156	-6.696358609	0.372043876	0.775038554

LINC00475	0.066094298	0.024080248	0.056734733	-0.042014051	-0.009359565	0.032654486	8	0.20457571	0.816496581	0.816496581	0.816496581	0.816496581	0.816496581	0.816496581	21	91	0	A_24_P773539	A_24_P773539	A_24_P773539	chr9	92148195	92148255	+	LINC00475	long intergenic non-protein coding RNA 475 [Source:HGNC Symbol;Acc:HGNC:23569]	ENSG00000225511	lincRNA	exon	non-coding exon	1	3491	9:92148195:92148255	ENSG00000225511	LINC00475	4.950317984	0.035815497	0.42773436	0.155837184	0.367163211	-0.271897176	-0.060571149	0.211326027	9.961963646	0.677943402	0.879273949	0.721176749	0.791252877	0.952898684	0.836893339	-6.8994354	-6.169301171	-6.973997467	-7.109334449	-6.294734208	-7.047036235	0.077384394	0.970795886

ANK2	0.047597135	0.056581886	0.107614734	0.008984752	0.060017599	0.051032848	8	0.139630663	0.816496581	0.816496581	0.816496581	0.816496581	0.816496581	0.816496581	21	92	0	P_250528	P_250528	P_250528	chr4	113311305	113311365	+	ANK2	"ankyrin 2, neuronal [Source:HGNC Symbol;Acc:HGNC:493]"	ENSG00000145362	protein_coding	exon	coding exon	1	3492	4:113311305:113311365	ENSG00000145362	ANK2	3.717039638	0.017863615	0.436155914	0.518487603	0.98612664	0.08233169	0.549970727	0.467639037	9.961963646	0.672023952	0.615438394	0.347415762	0.936013723	0.594454387	0.650104939	-6.895538918	-6.042325185	-6.540586365	-7.145907393	-6.13828025	-6.953211006	0.326573284	0.806294858

MRPL45	0.000915844	0.042947741	0.093724308	0.042031897	0.092808464	0.050776568	8	0.049923433	0.816496581	0.816496581	0.816496581	0.816496581	0.816496581	0.816496581	21	94	0	P_228546	P_228546	P_228546	chr17	38299408	38299468	+	MRPL45	mitochondrial ribosomal protein L45 [Source:HGNC Symbol;Acc:HGNC:16651]	ENSG00000278845	protein_coding	exon	coding exon	1	3494	17:38299408:38299468	ENSG00000278845	MRPL45	11.80164983	0.004208176	0.017291003	0.81084714	1.769501406	0.793556138	1.752210403	0.958654265	9.961963646	0.986545868	0.436396562	0.107354565	0.445943702	0.110401516	0.360420847	-6.99819717	-5.846901842	-5.561869822	-6.815743875	-4.880135199	-6.591295772	1.401316321	0.299233325

ATAD2	-0.69750974	-0.004320004	-0.533783792	0.693189736	0.163725948	-0.529463788	8	0.076454539	0.816496581	0.816496581	0.816496581	0.816496581	0.816496581	0.816496581	21	95	0	P_147907	P_147907	P_147907	chr8	123320117	123320177	-	ATAD2	"ATPase family, AAA domain containing 2 [Source:HGNC Symbol;Acc:HGNC:30123]"	ENSG00000156802	protein_coding	3'UTR	3'UTR	1	3495	8:123320117:123320177	ENSG00000156802	ATAD2	10.52931953	0.006900776	-10.2836419	-0.063691405	-7.8697702	10.21995049	2.4138717	-7.806078795	9.961963646	1.27E-06	0.950475831	1.39E-05	1.34E-06	0.036528683	1.49E-05	5.986745914	-6.179949356	3.538612532	5.892844257	-3.878198431	3.45287853	56.48327686	1.47E-06

PRR15	0.016538406	0.158226098	0.310668702	0.141687693	0.294130296	0.152442603	8	0.133198747	0.816496581	0.816496581	0.816496581	0.816496581	0.816496581	0.816496581	21	97	0	A_32_P154911	A_32_P154911	A_32_P154911	chr7	29567231	29567291	+	PRR15	proline rich 15 [Source:HGNC Symbol;Acc:HGNC:22310]	ENSG00000176532	protein_coding	exon	coding exon	1	3497	7:29567231:29567291	ENSG00000176532	PRR15	5.906670434	0.016454402	0.15790585	1.510715536	2.966211257	1.352809687	2.808305407	1.455495721	9.961963646	0.877685853	0.161911096	0.014193041	0.206020634	0.018593038	0.176309818	-6.984771026	-5.103755135	-3.613595628	-6.23131492	-3.242953259	-6.022804824	3.823457669	0.046514142

MYO18A	0.125369571	0.033708514	0.208306528	-0.091661057	0.082936957	0.174598014	8	0.131081889	0.816496581	0.816496581	0.816496581	0.816496581	0.816496581	0.816496581	21	98	0	P_228178	P_228178	P_228178	chr17	29098884	29098944	-	MYO18A	myosin XVIIIA [Source:HGNC Symbol;Acc:HGNC:31104]	ENSG00000196535	protein_coding	3'UTR;exon	multiple Ref	1	3498	17:29098884:29098944	ENSG00000196535	MYO18A	6.809574866	0.016005138	1.213690807	0.326328891	2.016595548	-0.887361916	0.802904741	1.690266657	9.961963646	0.252856049	0.750927634	0.071492772	0.395802216	0.44076492	0.121973869	-6.249126026	-6.126259019	-5.1874109	-6.735273209	-5.964792512	-5.700435829	1.655877015	0.23888656

ZNF107	-0.105454215	-0.252490014	-0.209393048	-0.147035799	-0.103938833	0.043096967	8	0.102775023	0.816496581	0.816496581	0.816496581	0.816496581	0.816496581	0.816496581	21	101	0	A_23_P45087	A_23_P45087	A_23_P45087	chr7	64710798	64710858	+	ZNF107	zinc finger protein 107 [Source:HGNC Symbol;Acc:HGNC:12887]	ENSG00000196247	protein_coding	3'UTR	3'UTR	1	3501	7:64710798:64710858	ENSG00000196247	ZNF107	7.877086313	0.010689112	-1.249219492	-2.991017923	-2.480487637	-1.741798431	-1.231268144	0.510530286	9.961963646	0.240139635	0.013604712	0.032599027	0.112274108	0.246498367	0.620801269	-6.207736809	-2.871930392	-4.436022503	-5.702548636	-5.547947455	-6.930833123	3.578346777	0.05471522

PPAT	-0.240895163	0.013276038	-0.028729339	0.254171202	0.212165825	-0.042005377	8	0.062272925	0.816496581	0.816496581	0.816496581	0.816496581	0.816496581	0.816496581	21	105	0	P_137752	P_137752	P_137752	chr4	56393392	56393452	-	PPAT	phosphoribosyl pyrophosphate amidotransferase [Source:HGNC Symbol;Acc:HGNC:9238]	ENSG00000128059	protein_coding	3'UTR;exon	multiple Ref	1	3505	4:56393392:56393452	ENSG00000128059	PPAT	11.45119532	0.005320863	-4.044666086	0.222906686	-0.482369928	4.267572772	3.562296158	-0.705276614	9.961963646	0.002361829	0.828112158	0.639966436	0.001657507	0.005192334	0.496797455	-1.746123151	-6.15596147	-6.921523367	-1.511629914	-2.021714671	-6.806251786	8.006873044	0.005204953

TMEM161A	-0.136657131	-0.055569824	-0.095435432	0.081087308	0.041221699	-0.039865608	8	0.070276358	0.816496581	0.816496581	0.816496581	0.816496581	0.816496581	0.816496581	21	107	0	P_125222	P_125222	P_125222	chr19	19132351	19132411	-	TMEM161A	transmembrane protein 161A [Source:HGNC Symbol;Acc:HGNC:26020]	ENSG00000064545	protein_coding	3'UTR;exon	multiple Ref	1	3507	19:19132351:19132411	ENSG00000064545	TMEM161A	10.95433713	0.006172783	-2.130283631	-0.866251797	-1.487697983	1.264031834	0.642585648	-0.621446186	9.961963646	0.059095879	0.406731567	0.167784003	0.234993931	0.5350014	0.548262139	-4.961290176	-5.801284978	-5.956508354	-6.339603145	-6.081637476	-6.864413635	1.649772328	0.240160153

CNTN1	0.119571597	0.185607845	0.252669633	0.066036248	0.133098036	0.067061788	8	0.10027805	0.816496581	0.816496581	0.816496581	0.816496581	0.816496581	0.816496581	21	109	0	P_215103	P_215103	P_215103	chr12	40933809	40933869	+	CNTN1	contactin 1 [Source:HGNC Symbol;Acc:HGNC:2171]	ENSG00000018236	protein_coding	exon	coding exon	1	3509	12:40933809:40933869	ENSG00000018236	CNTN1	3.634301384	0.010281949	1.444228568	2.241838019	3.051834312	0.797609451	1.607605744	0.809996293	9.961963646	0.179379827	0.048948692	0.012265053	0.443693408	0.139117565	0.436863149	-5.96577616	-4.057799185	-3.467170748	-6.812428845	-5.079564413	-6.72434103	3.383666265	0.062474566

SMC6	0.031855282	-0.144875554	0.050130551	-0.176730835	0.018275269	0.195006104	8	0.075848261	0.816496581	0.816496581	0.816496581	0.816496581	0.816496581	0.816496581	21	112	0	A_23_P209962	NM_024624	A_23_P209962	chr2	17666494	17670459	-	SMC6	structural maintenance of chromosomes 6 [Source:HGNC Symbol;Acc:HGNC:20466]	ENSG00000163029	protein_coding	"exon,intron"	multiple non-Ref	0	3512	2:17666494:17670459	ENSG00000163029	SMC6	9.683249435	0.006826624	0.472197956	-2.147522694	0.743096349	-2.619720651	0.270898392	2.890619043	9.961963646	0.646959128	0.057405383	0.474582688	0.025685153	0.791999318	0.016149978	-6.878039473	-4.201981141	-6.753683709	-4.299876301	-6.257800084	-3.765351133	3.446502916	0.059835109

BEST3	-0.386185717	-0.033810105	-0.241273078	0.352375612	0.144912639	-0.207462973	8	0.219937777	0.816496581	0.816496581	0.816496581	0.816496581	0.816496581	0.816496581	21	113	0	P_216148	P_216148	P_216148	chr12	69697662	69697722	-	BEST3	bestrophin 3 [Source:HGNC Symbol;Acc:HGNC:17105]	ENSG00000127325	protein_coding	exon	coding exon	1	3513	12:69697662:69697722	ENSG00000127325	BEST3	4.114828832	0.04105254	-2.334381437	-0.204372349	-1.458426272	2.130009087	0.875955165	-1.254053922	9.961963646	0.041829264	0.842177458	0.175518767	0.059123183	0.401682175	0.238450182	-4.631321101	-6.160116445	-5.995117442	-5.107669212	-5.904020602	-6.274095694	2.415833224	0.127363926

MOV10L1	-0.027434806	-0.116362017	-0.020110247	-0.08892721	0.00732456	0.09625177	8	0.122453904	0.816496581	0.816496581	0.816496581	0.816496581	0.816496581	0.816496581	21	115	0	P_133765	P_133765	P_133765	chr22	50090125	50090185	+	MOV10L1	Mov10 RISC complex RNA helicase like 1 [Source:HGNC Symbol;Acc:HGNC:7201]	ENSG00000073146	protein_coding	exon	coding exon	1	3515	22:50090125:50090185	ENSG00000073146	MOV10L1	4.32295314	0.014248453	-0.28149045	-1.193913893	-0.206337975	-0.912423443	0.075152475	0.987575918	9.961963646	0.784094724	0.260166415	0.840682927	0.383095587	0.941581145	0.34673938	-6.955293824	-5.481815135	-7.023919674	-6.712472494	-6.293682097	-6.563233967	0.560118874	0.65336803

SCN4A	0.010844623	-0.080038951	-0.1877821	-0.090883574	-0.198626723	-0.107743149	8	0.147770575	0.816496581	0.816496581	0.816496581	0.816496581	0.816496581	0.816496581	21	116	0	A_24_P270999	A_24_P270999	A_24_P270999	chr17	63939160	63939220	-	SCN4A	"sodium channel, voltage-gated, type IV, alpha subunit [Source:HGNC Symbol;Acc:HGNC:10591]"	ENSG00000007314	protein_coding	3'UTR	3'UTR	1	3516	17:63939160:63939220	ENSG00000007314	SCN4A	4.190322826	0.019742297	0.094528169	-0.697666989	-1.636820206	-0.792195159	-1.731348375	-0.939153217	9.961963646	0.926563912	0.501343753	0.132826839	0.446700965	0.114182573	0.369864541	-6.993486447	-5.931817577	-5.752567608	-6.816853627	-4.909468535	-6.609826109	1.280334053	0.33385057

KATNAL2	0.296808161	-0.047820479	0.353299239	-0.344628641	0.056491078	0.401119719	8	0.118536253	0.816496581	0.816496581	0.816496581	0.816496581	0.816496581	0.816496581	21	117	0	A_24_P927976	A_24_P927976	A_24_P927976	chr18	47098884	47098944	+	KATNAL2	katanin p60 subunit A-like 2 [Source:HGNC Symbol;Acc:HGNC:25387]	ENSG00000167216	protein_coding	3'UTR	3'UTR	1	3517	18:47098884:47098944	ENSG00000167216	KATNAL2	5.489316253	0.013490277	3.129759953	-0.504253726	3.725442743	-3.634013679	0.59568279	4.229696469	9.961963646	0.010742773	0.625047918	0.003965707	0.004610578	0.564674365	0.001759477	-3.289137633	-6.049794987	-2.320608043	-2.567398762	-6.111344361	-1.50526518	9.229969739	0.003172335

RESF1	-0.002931321	-0.075871239	-0.063505987	-0.072939918	-0.060574666	0.012365252	8	0.089123225	0.816496581	0.816496581	0.816496581	0.816496581	0.816496581	0.816496581	21	118	0	P_309384	P_309384	P_309384	chr12	31988088	31988148	-	KIAA1551	KIAA1551 [Source:HGNC Symbol;Acc:HGNC:25559]	ENSG00000174718	protein_coding	intron	intron antisense	0	3518	12:31988088:31988148	ENSG00000174718	RESF1	5.903606132	0.008585305	-0.03874638	-1.002870676	-0.839426021	-0.964124296	-0.800679641	0.163444655	9.961963646	0.96985808	0.339660339	0.420917857	0.357803555	0.441993918	0.873436585	-6.997541142	-5.678020698	-6.675407791	-6.663752695	-5.966571488	-7.05680416	0.551537437	0.658588029

STING1	0.161514891	-0.218554198	0.108161643	-0.380069088	-0.053353248	0.32671584	8	0.135713695	0.816496581	0.816496581	0.816496581	0.816496581	0.816496581	0.816496581	21	120	0	A_23_P61371	A_23_P61371	A_23_P61371	chr5	139476011	139476071	-	TMEM173	transmembrane protein 173 [Source:HGNC Symbol;Acc:HGNC:27962]	ENSG00000184584	protein_coding	3'UTR;exon	multiple Ref	1	3520	5:139476011:139476071	ENSG00000184584	STING1	8.563217045	0.016997508	1.51727941	-2.053109671	1.016076185	-3.570389082	-0.501203226	3.069185856	9.961963646	0.160269484	0.067264165	0.333634933	0.005123033	0.627117076	0.011908049	-5.869199346	-4.344237873	-6.510854915	-2.675414701	-6.164784754	-3.459933648	4.994765272	0.022797443

HADHA	0.208230174	-0.090994063	0.160945983	-0.299224237	-0.047284192	0.251940046	8	0.107552342	0.816496581	0.816496581	0.816496581	0.816496581	0.816496581	0.816496581	21	121	0	P_234000	P_234000	P_234000	chr2	26234232	26234292	-	HADHA	"hydroxyacyl-CoA dehydrogenase/3-ketoacyl-CoA thiolase/enoyl-CoA hydratase (trifunctional protein), alpha subunit [Source:HGNC Symbol;Acc:HGNC:4801]"	ENSG00000084754	protein_coding	exon	coding exon	1	3521	2:26234232:26234292	ENSG00000084754	HADHA	8.960160322	0.011496022	2.378567571	-1.039405208	1.83845063	-3.417972779	-0.540116941	2.877855838	9.961963646	0.038796297	0.3231859	0.095955377	0.00660563	0.600981409	0.016506402	-4.558687205	-5.642569776	-5.459722197	-2.935195955	-6.143822554	-3.787140743	5.062361008	0.021940777

PKD1L2	-0.034737875	-0.002777947	0.117151772	0.031959928	0.151889646	0.119929719	8	0.101096433	0.816496581	0.816496581	0.816496581	0.816496581	0.816496581	0.816496581	21	122	0	P_227096	P_227096	P_227096	chr16	81109233	81109293	-	PKD1L2	polycystic kidney disease 1-like 2 [Source:HGNC Symbol;Acc:HGNC:21715]	ENSG00000166473	polymorphic_pseudogene;protein_coding	exon	coding exon	1	3522	16:81109233:81109293	ENSG00000166473	PKD1L2	4.707955455	0.010414294	-0.416901985	-0.033339164	1.40598141	0.38356282	1.822883395	1.439320575	9.961963646	0.685591295	0.974062655	0.190145381	0.709361098	0.098427095	0.180731421	-6.904339619	-6.181501507	-6.063041443	-7.069749678	-4.779465289	-6.04390878	1.28222842	0.333275029

RGS16	0.499999467	-0.024759664	0.423333701	-0.524759131	-0.076665765	0.448093365	8	0.153102088	0.816496581	0.816496581	0.816496581	0.816496581	0.816496581	0.816496581	21	124	0	A_23_P320578	A_23_P320578	A_23_P320578	chr1	182598735	182598795	-	RGS16	regulator of G-protein signaling 16 [Source:HGNC Symbol;Acc:HGNC:9997]	ENSG00000143333	protein_coding	3'UTR	3'UTR	1	3524	1:182598735:182598795	ENSG00000143333	RGS16	6.221084686	0.021030482	4.222703157	-0.209105646	3.575228928	-4.431808803	-0.647474229	3.784334574	9.961963646	0.001779017	0.838579702	0.005082047	0.001282385	0.531961595	0.003600749	-1.454417286	-6.159089302	-2.574280544	-1.245195134	-6.078422049	-2.24316854	10.83975822	0.001766938

IGF1	-0.608998554	-0.021419928	-0.865067315	0.587578626	-0.25606876	-0.843647387	8	0.100907176	0.816496581	0.816496581	0.816496581	0.816496581	0.816496581	0.816496581	21	126	0	P_112570	P_112570	P_112570	chr12	102478009	102478069	-	IGF1	insulin-like growth factor 1 (somatomedin C) [Source:HGNC Symbol;Acc:HGNC:5464]	ENSG00000017427	protein_coding	intron	intron sense	0	3526	12:102478009:102478069	ENSG00000017427	IGF1	6.481511072	0.010383593	-7.31961221	-0.257448176	-10.39732727	7.062164034	-3.077715061	-10.1398791	9.961963646	2.60E-05	0.802072534	1.15E-06	3.52E-05	0.011736466	1.44E-06	2.92133698	-6.147267279	6.080172346	2.51354329	-2.804635074	5.843291425	53.98517596	1.82E-06

TCFL5	0.152709987	0.044675047	0.130056015	-0.10803494	-0.022653972	0.085380968	8	0.061582097	0.816496581	0.816496581	0.816496581	0.816496581	0.816496581	0.816496581	21	127	0	P_131774	P_131774	P_131774	chr20	62860382	62860442	-	TCFL5	transcription factor-like 5 (basic helix-loop-helix) [Source:HGNC Symbol;Acc:HGNC:11646]	ENSG00000101190	protein_coding	intron	intron sense	0	3527	20:62860382:62860442	ENSG00000101190	TCFL5	8.410729196	0.005252152	2.580741125	0.754991426	2.197897556	-1.825749699	-0.382843569	1.44290613	9.961963646	0.02745892	0.467727789	0.052727373	0.097967627	0.709877643	0.179743152	-4.222174213	-5.89022168	-4.899832748	-5.578661859	-6.219313879	-6.039243884	2.937149956	0.085791546

NPR2	-0.021940989	0.113241467	0.190859996	0.135182456	0.212800985	0.077618529	8	0.234402519	0.816496581	0.816496581	0.816496581	0.816496581	0.816496581	0.816496581	21	128	0	P_267315	P_267315	P_267315	chr9	35801091	35801151	+	NPR2	natriuretic peptide receptor 2 [Source:HGNC Symbol;Acc:HGNC:7944]	ENSG00000159899	protein_coding	exon	coding exon	1	3528	9:35801091:35801151	ENSG00000159899	NPR2	4.2018869	0.046330146	-0.124844666	0.64434621	1.085997193	0.769190877	1.210841859	0.441650983	9.961963646	0.903129864	0.533905485	0.303059587	0.459628455	0.253898844	0.668174118	-6.989861756	-5.96783873	-6.43866675	-6.835356764	-5.570911121	-6.965856052	0.643834389	0.604289619

LINC02059	0.491018079	-0.254229226	0.560203438	-0.745247305	0.069185359	0.814432664	8	0.811626192	0.816496581	0.816496581	0.816496581	0.816496581	0.816496581	0.816496581	21	129	0	A_24_P479682	A_24_P479682	A_24_P479682	chr5	86746896	86746956	+	CTC-493L21.1	NA	ENSG00000250544	lincRNA	exon	non-coding exon	1	3529	5:86746896:86746956	ENSG00000250544	LINC02059	2.804537622	0.531208474	0.825107653	-0.427207243	0.941366853	-1.252314897	0.1162592	1.368574097	9.961963646	0.428625884	0.67831468	0.368783677	0.239056787	0.909757395	0.201201431	-6.63909511	-6.086782524	-6.583651865	-6.353502687	-6.289501205	-6.134371832	0.86738068	0.489726857

ASXL3	0.16098384	0.072464164	0.086150126	-0.088519676	-0.074833714	0.013685963	8	0.115703794	0.816496581	0.816496581	0.816496581	0.816496581	0.816496581	0.816496581	21	130	0	P_231156	P_231156	P_231156	chr18	33734319	33734379	+	ASXL3	additional sex combs like transcriptional regulator 3 [Source:HGNC Symbol;Acc:HGNC:29357]	ENSG00000141431	protein_coding	3'UTR;exon	multiple Ref	1	3530	18:33734319:33734379	ENSG00000141431	ASXL3	3.776578413	0.01295747	1.73207941	0.779666367	0.926918254	-0.952413043	-0.805161157	0.147251886	9.961963646	0.114048113	0.453711797	0.37587959	0.363424072	0.43952094	0.885870729	-5.568957316	-5.871419751	-6.597197455	-6.674984908	-5.962984093	-7.059545926	1.007369065	0.429508969

PLEKHA5	-0.104527045	-0.079629385	-0.12697869	0.02489766	-0.022451645	-0.047349305	8	0.07849528	0.816496581	0.816496581	0.816496581	0.816496581	0.816496581	0.816496581	21	131	0	P_214539	P_214539	P_214539	chr12	19265787	19265847	+	PLEKHA5	"pleckstrin homology domain containing, family A member 5 [Source:HGNC Symbol;Acc:HGNC:30036]"	ENSG00000052126	protein_coding	5'UTR;exon	multiple Ref	1	3531	12:19265787:19265847	ENSG00000052126	PLEKHA5	7.432565803	0.007154712	-1.513485393	-1.152983051	-1.838570987	0.360502342	-0.325085594	-0.685587936	9.961963646	0.16121658	0.275831901	0.095936492	0.725998088	0.751840527	0.508612366	-5.874289072	-5.52604857	-5.459542196	-7.079003606	-6.240781041	-6.820514252	1.284337437	0.332635577

CCDC141	-1.67759777	-0.130754266	-1.80331577	1.546843504	-0.125718	-1.672561504	8	0.224574289	0.816496581	0.816496581	0.816496581	0.816496581	0.816496581	0.816496581	21	132	0	P_238077	P_238077	P_238077	chr2	178872213	178872273	-	CCDC141	coiled-coil domain containing 141 [Source:HGNC Symbol;Acc:HGNC:26821]	ENSG00000163492	protein_coding	exon	coding exon	1	3532	2:178872213:178872273	ENSG00000163492	CCDC141	4.614924577	0.042707624	-9.942160399	-0.774905589	-10.68721892	9.167254809	-0.745058522	-9.912313332	9.961963646	1.73E-06	0.456394554	8.90E-07	3.60E-06	0.473447546	1.78E-06	5.678095086	-5.875088929	6.333783519	4.880106129	-6.009625044	5.633470696	66.08521814	7.03E-07

SEPTIN14	-0.240267463	-0.214378053	-0.092205498	0.02588941	0.148061965	0.122172555	8	0.379810764	0.816496581	0.816496581	0.816496581	0.816496581	0.816496581	0.816496581	21	133	0	P_261294	P_261294	P_261294	chr7	55842941	55843001	-	Sep-14	septin 14 [Source:HGNC Symbol;Acc:HGNC:33280]	ENSG00000154997	protein_coding	exon	coding exon	1	3533	7:55842941:55843001	ENSG00000154997	SEPTIN14	3.561487093	0.118052292	-0.856452695	-0.764167811	-0.328673914	0.092284884	0.527778781	0.435493897	9.961963646	0.411875042	0.462483181	0.749206896	0.928301152	0.609206509	0.672488435	-6.612254381	-5.883291832	-6.988446129	-7.144957676	-6.150629134	-6.968750164	0.317035347	0.812904086

RELCH	0.176221742	0.061363175	0.312248287	-0.114858567	0.136026545	0.250885112	8	0.053467359	0.816496581	0.816496581	0.816496581	0.816496581	0.816496581	0.816496581	21	135	0	P_231636	P_231636	P_231636	chr18	62211182	62211242	+	KIAA1468	KIAA1468 [Source:HGNC Symbol;Acc:HGNC:29289]	ENSG00000134444	protein_coding	exon	coding exon	1	3535	18:62211182:62211242	ENSG00000134444	RELCH	8.084938973	0.004502423	3.216488293	1.120031681	5.699313546	-2.096456612	2.482825252	4.579281865	9.961963646	0.009273944	0.288976294	0.000201367	0.06255244	0.032468977	0.001021762	-3.140992597	-5.560811945	0.766122566	-5.161070391	-3.768281517	-0.942289052	12.60196946	0.00099833

TUBGCP5	-0.384727656	-0.115840717	-0.260807651	0.268886939	0.123920005	-0.144966934	8	0.094898616	0.816496581	0.816496581	0.816496581	0.816496581	0.816496581	0.816496581	21	136	0	P_222456	P_222456	P_222456	chr15	23004101	23004161	-	TUBGCP5	"tubulin, gamma complex associated protein 5 [Source:HGNC Symbol;Acc:HGNC:18600]"	ENSG00000275835	protein_coding	exon	coding exon	1	3536	15:23004101:23004161	ENSG00000275835	TUBGCP5	8.305828641	0.00943879	-4.849990294	-1.460322239	-3.287818168	3.389668055	1.562172125	-1.82749593	9.961963646	0.00067877	0.175008615	0.008221112	0.006926625	0.149427209	0.097688676	-0.458552417	-5.167774027	-3.063531879	-2.983572338	-5.140228059	-5.499695575	8.955777973	0.003530045

LIPH	-0.098836864	0.020133523	0.016864235	0.118970387	0.115701099	-0.003269288	8	0.105886008	0.816496581	0.816496581	0.816496581	0.816496581	0.816496581	0.816496581	21	137	0	P_136753	P_136753	P_136753	chr3	185508789	185508849	-	LIPH	"lipase, member H [Source:HGNC Symbol;Acc:HGNC:18483]"	ENSG00000163898	protein_coding	3'UTR;exon	multiple Ref	1	3537	3:185508789:185508849	ENSG00000163898	LIPH	4.59612725	0.011210408	-1.143283337	0.232892059	0.195074974	1.376175396	1.338358311	-0.037817085	9.961963646	0.279651397	0.820560476	0.849255379	0.198911845	0.210523184	0.970580622	-6.32850494	-6.153575114	-7.026378675	-6.201968504	-5.423102139	-7.070597295	0.847646927	0.498900293

OPCML	0.199725506	-0.043808028	0.084760467	-0.243533534	-0.11496504	0.128568494	8	0.186990472	0.816496581	0.816496581	0.816496581	0.816496581	0.816496581	0.816496581	21	138	0	P_110550	P_110550	P_110550	chr11	132436105	132436165	-	OPCML	opioid binding protein/cell adhesion molecule-like [Source:HGNC Symbol;Acc:HGNC:8143]	ENSG00000183715	protein_coding	exon	coding exon	1	3538	11:132436105:132436165	ENSG00000183715	OPCML	4.07614037	0.030285836	1.405592322	-0.308304275	0.596512	-1.713896597	-0.809080322	0.904816275	9.961963646	0.190257669	0.764201005	0.564141891	0.117435538	0.437365819	0.386921727	-6.015603512	-6.132226432	-6.856215148	-5.743045458	-5.95983252	-6.641676516	1.139552146	0.379882052

DCK	0.039515497	0.108019064	0.13992947	0.068503568	0.100413973	0.031910405	8	0.100682182	0.816496581	0.816496581	0.816496581	0.816496581	0.816496581	0.816496581	21	140	0	P_249279	P_249279	P_249279	chr4	70998122	70998182	+	DCK	deoxycytidine kinase [Source:HGNC Symbol;Acc:HGNC:2704]	ENSG00000156136	protein_coding	3'UTR;exon	multiple Ref	1	3540	4:70998122:70998182	ENSG00000156136	DCK	7.977565857	0.010347169	0.475775754	1.300574616	1.684783308	0.824798862	1.209007554	0.384208692	9.961963646	0.644495421	0.222684716	0.12304938	0.428793157	0.25457208	0.708897384	-6.876229825	-5.361311134	-5.684572064	-6.789809458	-5.572959528	-6.991331045	1.174326741	0.367891868

UQCC1	0.146904823	0.008856085	0.054115494	-0.138048739	-0.09278933	0.045259409	8	0.093928005	0.816496581	0.816496581	0.816496581	0.816496581	0.816496581	0.816496581	21	143	0	P_240648	P_240648	P_240648	chr20	35384037	35384097	-	UQCC1	ubiquinol-cytochrome c reductase complex assembly factor 1 [Source:HGNC Symbol;Acc:HGNC:15891]	ENSG00000101019	protein_coding	3'UTR;exon	multiple Ref	1	3543	20:35384037:35384097	ENSG00000101019	UQCC1	6.923017287	0.009291609	1.866535398	0.112523162	0.687577728	-1.754012236	-1.178957671	0.575054566	9.961963646	0.091640709	0.912643723	0.507410619	0.110080361	0.265806367	0.578010106	-5.370257233	-6.175417076	-6.794922906	-5.684712331	-5.60618981	-6.893677073	1.461094476	0.283637159

CCNJ	-0.019825654	0.245509649	-0.003253819	0.265335303	0.016571835	-0.248763467	8	0.287325738	0.816496581	0.816496581	0.816496581	0.816496581	0.816496581	0.816496581	21	144	0	P_209348	P_209348	P_209348	chr10	96057187	96057247	+	CCNJ	cyclin J [Source:HGNC Symbol;Acc:HGNC:23434]	ENSG00000107443	protein_coding	exon	coding exon	1	3544	10:96057187:96057247	ENSG00000107443	CCNJ	3.377024519	0.068503718	-0.092771821	1.148833587	-0.015225862	1.241605408	0.077545959	-1.164059449	9.961963646	0.927924025	0.277460794	0.988152608	0.242820223	0.939724635	0.271520734	-6.993665801	-5.530468231	-7.046994001	-6.366124819	-6.29348782	-6.377644984	0.705214802	0.570460969

CZIB	0.096876694	-0.06716521	-0.014621663	-0.164041904	-0.111498358	0.052543547	8	0.0438299	0.816496581	0.816496581	0.816496581	0.816496581	0.816496581	0.816496581	21	145	0	P_101893	P_101893	P_101893	chr1	53216790	53216850	-	C1orf123	chromosome 1 open reading frame 123 [Source:HGNC Symbol;Acc:HGNC:26059]	ENSG00000162384	protein_coding	exon	coding exon	1	3545	1:53216790:53216850	ENSG00000162384	CZIB	9.455881578	0.0037494	1.937688913	-1.343411677	-0.292456661	-3.28110059	-2.230145574	1.050955016	9.961963646	0.081502866	0.208939455	0.775937983	0.008314798	0.049927831	0.318104826	-5.262227962	-5.310886542	-7.000612159	-3.169418421	-4.167354682	-6.499369308	3.74810016	0.048869371

GLT8D2	0.059366603	0.013391613	-0.061148105	-0.04597499	-0.120514708	-0.074539719	8	0.127965204	0.816496581	0.816496581	0.816496581	0.816496581	0.816496581	0.816496581	21	146	0	P_217144	P_217144	P_217144	chr12	103994334	103994394	-	GLT8D2	glycosyltransferase 8 domain containing 2 [Source:HGNC Symbol;Acc:HGNC:24890]	ENSG00000120820	protein_coding	exon	coding exon	1	3546	12:103994334:103994394	ENSG00000120820	GLT8D2	4.595696552	0.015356777	0.586729319	0.132351387	-0.60433618	-0.454377932	-1.191065499	-0.736687567	9.961963646	0.570441559	0.897341441	0.559131429	0.659296474	0.261233105	0.47830227	-6.813703928	-6.172861465	-6.851264622	-7.037868404	-5.592875035	-6.782744104	0.482465884	0.701833508

ADCY7	0.075132471	-0.067181644	0.220547645	-0.142314116	0.145415173	0.287729289	8	0.107713104	0.816496581	0.816496581	0.816496581	0.816496581	0.816496581	0.816496581	21	147	0	P_118964	P_118964	P_118964	chr16	50315445	50315505	+	ADCY7	adenylate cyclase 7 [Source:HGNC Symbol;Acc:HGNC:238]	ENSG00000121281	protein_coding	exon	coding exon	1	3547	16:50315445:50315505	ENSG00000121281	ADCY7	4.868434201	0.011523813	0.857186258	-0.766475284	2.516227762	-1.623661542	1.659041504	3.282703046	9.961963646	0.411488461	0.461170371	0.030665693	0.135628182	0.128213455	0.00829235	-6.611615879	-5.881537614	-4.376341992	-5.87152892	-5.009697915	-3.094462937	3.969748266	0.042316305

TMEM14EP	-0.227872367	0.330741151	0.499248994	0.558613518	0.727121361	0.168507843	8	0.592257492	0.816496581	0.816496581	0.816496581	0.816496581	0.816496581	0.816496581	21	150	0	A_24_P932416	A_24_P932416	A_24_P932416	chr3	152340633	152340693	-	TMEM14E	transmembrane protein 14E [Source:HGNC Symbol;Acc:HGNC:34386]	ENSG00000221962	protein_coding	exon	coding exon	1	3550	3:152340633:152340693	ENSG00000221962	TMEM14EP	3.158285537	0.283893267	-0.523793068	0.760249803	1.147586106	1.284042871	1.671379174	0.387336303	9.961963646	0.611875878	0.4647178	0.277951976	0.228185885	0.125714555	0.706653631	-6.850685746	-5.886259714	-6.371967464	-6.315649228	-4.99275856	-6.990032305	1.126932776	0.384338866

UROS	0.04961278	0.076032137	0.167529184	0.026419357	0.117916404	0.091497048	8	0.058835529	0.816496581	0.816496581	0.816496581	0.816496581	0.816496581	0.816496581	21	151	0	P_210387	P_210387	P_210387	chr10	125816176	125816236	-	UROS	uroporphyrinogen III synthase [Source:HGNC Symbol;Acc:HGNC:12592]	ENSG00000188690	protein_coding	exon	coding exon	1	3551	10:125816176:125816236	ENSG00000188690	UROS	9.293800192	0.004986553	0.860476407	1.318689654	2.905600345	0.458213247	2.045123938	1.586910691	9.961963646	0.409757646	0.216783107	0.015741529	0.656631927	0.068168207	0.143732779	-6.608746305	-5.340124553	-3.717129115	-7.035993752	-4.451348201	-5.845893452	2.972108709	0.083630446

ATP2B4	0.128970591	-0.021445873	0.036151591	-0.150416464	-0.092818999	0.057597465	8	0.068765577	0.816496581	0.816496581	0.816496581	0.816496581	0.816496581	0.816496581	21	152	0	P_104502	P_104502	P_104502	chr1	203739859	203739919	+	ATP2B4	"ATPase, Ca++ transporting, plasma membrane 4 [Source:HGNC Symbol;Acc:HGNC:817]"	ENSG00000058668	protein_coding	3'UTR	3'UTR	1	3552	1:203739859:203739919	ENSG00000058668	ATP2B4	7.825278035	0.006004092	2.038509149	-0.338973472	0.571412052	-2.377482622	-1.467097098	0.910385524	9.961963646	0.068925763	0.741666462	0.580382632	0.038868126	0.173196119	0.384117954	-5.106134643	-6.121874979	-6.871690245	-4.705116778	-5.263777158	-6.636578537	2.205088935	0.150650352

TSR1	-0.229715287	-0.02381575	-0.16998499	0.205899537	0.059730297	-0.14616924	8	0.029692455	0.816496581	0.816496581	0.816496581	0.816496581	0.816496581	0.816496581	21	154	0	A_24_P941336	A_24_P941336	A_24_P941336	chr17	2323601	2323661	-	TSR1	"TSR1, 20S rRNA accumulation, homolog (S. cerevisiae) [Source:HGNC Symbol;Acc:HGNC:25542]"	ENSG00000167721	protein_coding	3'UTR	3'UTR	1	3554	17:2323601:2323661	ENSG00000167721	TSR1	10.42596517	0.00291469	-5.21121877	-0.540273507	-3.856203828	4.670945263	1.355014942	-3.315930321	9.961963646	0.000399771	0.60087741	0.003202345	0.000888577	0.205340705	0.007840682	0.090212349	-6.030512161	-2.101304445	-0.863436393	-5.40303697	-3.037664981	12.82803226	0.000932005

TRMU	-0.21902084	0.149483089	-0.085712665	0.368503929	0.133308175	-0.235195753	8	0.060156192	0.816496581	0.816496581	0.816496581	0.816496581	0.816496581	0.816496581	21	155	0	P_242899	P_242899	P_242899	chr22	46350403	46350463	+	TRMU	tRNA 5-methylaminomethyl-2-thiouridylate methyltransferase [Source:HGNC Symbol;Acc:HGNC:25481]	ENSG00000100416	protein_coding	3'UTR;exon	multiple Ref	1	3555	22:46350403:46350463	ENSG00000100416	TRMU	8.422839967	0.005112752	-3.751489425	2.560414918	-1.468125842	6.311904343	2.283363583	-4.02854076	9.961963646	0.00379975	0.028431636	0.172922319	8.92E-05	0.045619361	0.002423732	-2.233862331	-3.559454894	-5.982378202	1.539969206	-4.084231398	-1.835940759	14.01130288	0.000659936

TAOK1	0.108534346	-0.148299224	-0.016644911	-0.25683357	-0.125179256	0.131654313	8	0.07176384	0.816496581	0.816496581	0.816496581	0.816496581	0.816496581	0.816496581	21	156	0	P_228183	P_228183	P_228183	chr17	29467156	29467216	+	TAOK1	TAO kinase 1 [Source:HGNC Symbol;Acc:HGNC:29259]	ENSG00000160551	protein_coding	exon	coding exon	1	3556	17:29467156:29467216	ENSG00000160551	TAOK1	9.088405584	0.006342454	1.669106936	-2.280635328	-0.255975522	-3.949742263	-1.925082458	2.024659806	9.961963646	0.126171464	0.045831186	0.803177795	0.002751698	0.083219102	0.070537762	-5.659318706	-3.997960919	-7.011455601	-2.036230978	-4.630599066	-5.198293537	5.243065446	0.01983276

SNRPB	-0.089855085	0.036736235	-0.181283516	0.12659132	-0.09142843	-0.218019751	8	0.135232303	0.816496581	0.816496581	0.816496581	0.816496581	0.816496581	0.816496581	21	157	0	P_130503	P_130503	P_130503	chr20	2461809	2461869	+	SNRPB	small nuclear ribonucleoprotein polypeptides B and B1 [Source:HGNC Symbol;Acc:HGNC:11153]	ENSG00000125835	protein_coding	3'UTR;exon;intron	multiple non-Ref	0	3557	20:2461809:2461869	ENSG00000125835	SNRPB	10.8989805	0.016892765	-0.846716298	0.346170379	-1.708258434	1.192886678	-0.861542135	-2.054428813	9.961963646	0.417029611	0.736414437	0.118504295	0.260550693	0.409198084	0.067115928	-6.620684372	-6.119307075	-5.650896144	-6.422534953	-5.91637119	-5.151634509	1.690136753	0.231881525

TMEM131L	0.080402211	0.00729168	0.051643967	-0.073110531	-0.028758244	0.044352286	8	0.059485317	0.816496581	0.816496581	0.816496581	0.816496581	0.816496581	0.816496581	21	159	0	P_251259	P_251259	P_251259	chr4	153583608	153583668	+	KIAA0922	KIAA0922 [Source:HGNC Symbol;Acc:HGNC:29146]	ENSG00000121210	protein_coding	exon	coding exon	1	3559	4:153583608:153583668	ENSG00000121210	TMEM131L	6.367018363	0.005048295	1.385929904	0.125690046	0.890210815	-1.260239858	-0.495719089	0.764520769	9.961963646	0.19600596	0.902477688	0.394343085	0.23630258	0.63084551	0.462282213	-6.040616076	-6.173766045	-6.630815084	-6.344111731	-6.167619598	-6.761148533	0.85791815	0.494103743

DBR1	-0.045071921	0.027799825	-0.052059946	0.072871746	-0.006988025	-0.079859771	8	0.052092844	0.816496581	0.816496581	0.816496581	0.816496581	0.816496581	0.816496581	22	12	0	P_136126	P_136126	P_136126	chr3	138161366	138161426	-	DBR1	debranching RNA lariats 1 [Source:HGNC Symbol;Acc:HGNC:15594]	ENSG00000138231	protein_coding	3'UTR	3'UTR	1	3582	3:138161366:138161426	ENSG00000138231	DBR1	8.258622937	0.004385904	-0.83353168	0.514112433	-0.962763815	1.347644113	-0.129232135	-1.476876248	9.961963646	0.424079521	0.618384151	0.358453221	0.207620691	0.899745946	0.170608472	-6.63196691	-6.044642178	-6.56327151	-6.237756154	-6.287809933	-5.99467625	0.983334978	0.439262575

C4orf33	0.302953449	0.172299339	0.485309505	-0.13065411	0.182356055	0.313010165	8	0.060819091	0.816496581	0.816496581	0.816496581	0.816496581	0.816496581	0.816496581	22	14	0	P_250909	P_250909	P_250909	chr4	129109612	129109672	+	C4orf33	chromosome 4 open reading frame 33 [Source:HGNC Symbol;Acc:HGNC:27025]	ENSG00000151470	protein_coding	exon	coding exon	1	3584	4:129109612:129109672	ENSG00000151470	C4orf33	8.886347291	0.005177152	5.156749921	2.932809009	8.260740244	-2.223940912	3.103990323	5.327931235	9.961963646	0.000432473	0.015026264	9.10E-06	0.050455079	0.011223572	0.00033827	0.008668605	-2.965151863	3.974921156	-4.956561672	-2.761898223	0.207452078	24.40013136	6.58E-05

SMIM9	0.031381378	0.404571153	0.353007096	0.373189775	0.321625718	-0.051564057	8	0.371077868	0.816496581	0.816496581	0.816496581	0.816496581	0.816496581	0.816496581	22	15	0	P_315141	P_315141	P_315141	chrX	154823613	154823673	-	SMIM9	small integral membrane protein 9 [Source:HGNC Symbol;Acc:HGNC:41915]	ENSG00000203870	protein_coding	3'UTR	3'UTR	1	3585	X:154823613:154823673	ENSG00000203870	SMIM9	3.379349633	0.112786316	0.114443053	1.475408692	1.287362515	1.360965638	1.172919462	-0.188046176	9.961963646	0.911160315	0.170994655	0.22707233	0.203515373	0.268110762	0.854615658	-6.991218003	-5.148749846	-6.210409046	-6.221110384	-5.612792013	-7.052094935	1.185093194	0.364264665

TAOK3	-0.580868939	0.227544337	-0.314591612	0.808413276	0.266277327	-0.542135949	8	0.113982009	0.816496581	0.816496581	0.816496581	0.816496581	0.816496581	0.816496581	22	16	0	P_217712	P_217712	P_217712	chr12	118244903	118244963	-	TAOK3	TAO kinase 3 [Source:HGNC Symbol;Acc:HGNC:18133]	ENSG00000135090	protein_coding	exon	coding exon	1	3586	12:118244903:118244963	ENSG00000135090	TAOK3	10.20475843	0.012639887	-6.327791956	2.478791908	-3.42705581	8.806583864	2.900736146	-5.905847718	9.961963646	8.74E-05	0.032693688	0.006505911	5.16E-06	0.015872987	0.000152167	1.666911736	-3.688445438	-2.826000013	4.508994379	-3.092660702	1.039989628	29.79654979	2.74E-05

FRAS1	0.058388521	0.071998058	0.150986895	0.013609537	0.092598374	0.078988837	8	0.065005619	0.816496581	0.816496581	0.816496581	0.816496581	0.816496581	0.816496581	22	17	0	P_249574	P_249574	P_249574	chr4	78508734	78508794	+	FRAS1	Fraser extracellular matrix complex subunit 1 [Source:HGNC Symbol;Acc:HGNC:19185]	ENSG00000138759	protein_coding	exon	coding exon	1	3587	4:78508734:78508794	ENSG00000138759	FRAS1	4.024855004	0.005600176	0.955591477	1.178326304	2.471064296	0.222734828	1.51547282	1.292737992	9.961963646	0.36189238	0.266046579	0.033128514	0.828242294	0.160719854	0.225278656	-6.521766646	-5.498795918	-4.451726099	-7.122550314	-5.201504887	-6.227862489	2.070867816	0.168085382

AGL	0.139930572	-0.058173455	0.126543487	-0.198104028	-0.013387085	0.184716942	8	0.096933636	0.816496581	0.816496581	0.816496581	0.816496581	0.816496581	0.816496581	22	18	0	P_203070	P_203070	P_203070	chr1	99877767	99877827	+	AGL	"amylo-alpha-1, 6-glucosidase, 4-alpha-glucanotransferase [Source:HGNC Symbol;Acc:HGNC:321]"	ENSG00000162688	protein_coding	exon	coding exon	1	3588	1:99877767:99877827	ENSG00000162688	AGL	7.598190231	0.009752289	1.735421476	-0.721468236	1.569394607	-2.456889713	-0.16602687	2.290862843	9.961963646	0.113435233	0.487208909	0.147744473	0.033940978	0.871456992	0.045042	-5.564111287	-5.914903843	-5.846230191	-4.573266977	-6.282044764	-4.772300375	2.88452135	0.089170903

ATF6B	0.055420341	0.01644702	0.086145421	-0.038973321	0.03072508	0.069698401	8	0.32213564	0.816496581	0.816496581	0.816496581	0.816496581	0.816496581	0.816496581	22	19	0	P_142224	P_142224	P_142224	chr6	32115396	32115456	-	ATF6B	activating transcription factor 6 beta [Source:HGNC Symbol;Acc:HGNC:2349]	ENSG00000213676	protein_coding	3'UTR	3'UTR	1	3589	6:32115396:32115456	ENSG00000213676	ATF6B	5.995818376	0.085540753	0.232074964	0.068872575	0.360737503	-0.163202389	0.128662539	0.291864928	9.961963646	0.821177714	0.946453877	0.725827655	0.87362236	0.900185139	0.776377399	-6.969049806	-6.179587441	-6.976518007	-7.135066817	-6.28788793	-7.025040829	0.052851329	0.983049747

AKT2	-0.002785295	0.001911759	-0.07961274	0.004697054	-0.076827445	-0.081524499	8	0.099776691	0.816496581	0.816496581	0.816496581	0.816496581	0.816496581	0.816496581	22	21	0	P_232903	P_232903	P_232903	chr19	40242006	40242066	-	AKT2	v-akt murine thymoma viral oncogene homolog 2 [Source:HGNC Symbol;Acc:HGNC:392]	ENSG00000105221	protein_coding	3'UTR;exon	multiple Ref	1	3591	19:40242006:40242066	ENSG00000105221	AKT2	7.923672454	0.010201403	-0.03377434	0.023181885	-0.965379875	0.056956225	-0.931605535	-0.98856176	9.961963646	0.973724236	0.981962959	0.357204743	0.955706272	0.37356698	0.346279839	-6.997737879	-6.18180413	-6.56075367	-7.147839313	-5.854709677	-6.562265344	0.463670608	0.713964163

SPTLC3	0.494143972	0.044995503	0.622934958	-0.44914847	0.128790985	0.577939455	8	0.085397884	0.816496581	0.816496581	0.816496581	0.816496581	0.816496581	0.816496581	22	22	0	P_130681	P_130681	P_130681	chr20	13009323	13009383	+	SPTLC3	"serine palmitoyltransferase, long chain base subunit 3 [Source:HGNC Symbol;Acc:HGNC:16253]"	ENSG00000172296	protein_coding	exon	coding exon	1	3592	20:13009323:13009383	ENSG00000172296	SPTLC3	6.948169605	0.008063199	6.739775733	0.613706962	8.49639406	-6.126068771	1.756618328	7.882687098	9.961963646	5.20E-05	0.553163579	7.10E-06	0.000113596	0.109617363	1.37E-05	2.203972699	-5.987349866	4.229614376	1.287000922	-4.873914439	3.540540066	36.7901846	1.06E-05

SHOC1	-0.07450631	-0.078753197	0.020501595	-0.004246888	0.095007905	0.099254792	8	0.10780765	0.816496581	0.816496581	0.816496581	0.816496581	0.816496581	0.816496581	22	23	0	P_268549	P_268549	P_268549	chr9	111705246	111705306	-	C9orf84	chromosome 9 open reading frame 84 [Source:HGNC Symbol;Acc:HGNC:26535]	ENSG00000165181	protein_coding	exon	coding exon	1	3593	9:111705246:111705306	ENSG00000165181	SHOC1	3.480781711	0.011540177	-0.849439499	-0.897857868	0.233736776	-0.04841837	1.083176275	1.131594644	9.961963646	0.4155835	0.390445096	0.819922508	0.962339783	0.304250189	0.284309153	-6.618334947	-5.774110377	-7.017367146	-7.148331175	-5.707865803	-6.413569851	0.673075119	0.587946085

ZNF330	-0.155486016	-0.012720297	-0.171896443	0.142765719	-0.016410427	-0.159176147	8	0.063585208	0.816496581	0.816496581	0.816496581	0.816496581	0.816496581	0.816496581	22	24	0	P_138647	P_138647	P_138647	chr4	141222397	141222457	-	ZNF330	zinc finger protein 330 [Source:HGNC Symbol;Acc:HGNC:15462]	ENSG00000109445	protein_coding	exon	exon antisense	0	3594	4:141222397:141222457	ENSG00000109445	ZNF330	9.241360203	0.005453497	-2.57869182	-0.210962544	-2.850854136	2.367729276	-0.272162316	-2.639891592	9.961963646	0.027555473	0.837169343	0.017286875	0.039519714	0.791054762	0.024812554	-4.225614427	-6.158679985	-3.810515916	-4.721233426	-6.257437775	-4.19144662	4.578461312	0.029050275

BTNL2	-0.324654801	-0.07125099	-0.170038115	0.253403811	0.154616687	-0.098787125	8	0.215712336	0.816496581	0.816496581	0.816496581	0.816496581	0.816496581	0.816496581	22	26	0	A_23_P376686	A_23_P376686	A_23_P376686	chr6	32394888	32394948	-	BTNL2	butyrophilin-like 2 (MHC class II associated) [Source:HGNC Symbol;Acc:HGNC:1142]	ENSG00000204290	protein_coding	3'UTR;exon	multiple Ref	1	3596	6:32394888:32394948	ENSG00000204290	BTNL2	5.758804685	0.039574266	-1.998761726	-0.438662084	-1.046852455	1.560099642	0.951909271	-0.608190371	9.961963646	0.073648306	0.670266891	0.319902689	0.149913177	0.363667271	0.556672486	-5.168073353	-6.08165444	-6.479553784	-5.959613158	-5.836087252	-6.872990427	1.4988851	0.274254037

SFXN5	-0.174459513	0.21443095	0.000118445	0.388890463	0.174577958	-0.214312505	8	0.106566835	0.816496581	0.816496581	0.816496581	0.816496581	0.816496581	0.816496581	22	27	0	P_128116	P_128116	P_128116	chr2	72971615	72971675	-	SFXN5	sideroflexin 5 [Source:HGNC Symbol;Acc:HGNC:16073]	ENSG00000144040	protein_coding	exon	coding exon	1	3597	2:72971615:72971675	ENSG00000144040	SFXN5	7.496053107	0.011326565	-2.00766466	2.467652429	0.001363058	4.475317089	2.009027719	-2.46628937	9.961963646	0.072564671	0.033322308	0.998939346	0.001198865	0.072400098	0.033400041	-5.154243799	-3.705991724	-7.047119588	-1.175181759	-4.505707676	-4.482765677	6.711211073	0.009329695

SCAMP3	-0.123314694	-0.034157017	-0.097301562	0.089157678	0.026013133	-0.063144545	8	0.105841978	0.816496581	0.816496581	0.816496581	0.816496581	0.816496581	0.816496581	22	29	0	A_23_P97274	A_23_P97274	A_23_P97274	chr1	155256333	155256393	-	SCAMP3	secretory carrier membrane protein 3 [Source:HGNC Symbol;Acc:HGNC:10565]	ENSG00000116521	protein_coding	exon	coding exon	1	3599	1:155256333:155256393	ENSG00000116521	SCAMP3	9.962569071	0.011202922	-1.426904166	-0.395239104	-1.125899914	1.031665061	0.301004252	-0.730660809	9.961963646	0.184189773	0.700997313	0.286600297	0.326625079	0.769599937	0.481816893	-5.988228521	-6.100401586	-6.395777865	-6.596782263	-6.248719507	-6.787325845	0.858120273	0.494009831

TMTC2	-0.539676007	-0.058550292	-0.67388356	0.481125715	-0.134207553	-0.615333269	8	0.096589368	0.816496581	0.816496581	0.816496581	0.816496581	0.816496581	0.816496581	22	32	0	P_112335	P_112335	P_112335	chr12	83132418	83132478	+	TMTC2	transmembrane and tetratricopeptide repeat containing 2 [Source:HGNC Symbol;Acc:HGNC:25440]	ENSG00000179104	protein_coding	3'UTR	3'UTR	1	3602	12:83132418:83132478	ENSG00000179104	TMTC2	5.598539594	0.009698786	-6.711507071	-0.728141871	-8.380536136	5.9833652	-1.669029066	-7.652394266	9.961963646	5.39E-05	0.483290665	8.01E-06	0.000137185	0.126187149	1.77E-05	2.167863287	-5.910070038	4.105157367	1.089463308	-4.995990485	3.274924945	35.49222647	1.25E-05

ZNF638	0.051560698	-0.172865661	-0.087473324	-0.224426359	-0.139034022	0.085392337	8	0.112809223	0.816496581	0.816496581	0.816496581	0.816496581	0.816496581	0.816496581	22	34	0	P_311423	P_311423	P_311423	chr2	71334222	71334282	+	ZNF638	zinc finger protein 638 [Source:HGNC Symbol;Acc:HGNC:17894]	ENSG00000075292	protein_coding	intron	intron sense	0	3604	2:71334222:71334282	ENSG00000075292	ZNF638	8.066301516	0.012426292	0.566491808	-1.899256319	-0.961059953	-2.465748127	-1.527551761	0.938196366	9.961963646	0.583595759	0.086841005	0.35926807	0.033430957	0.157729557	0.370332451	-6.82602467	-4.570745529	-6.564908357	-4.558492128	-5.185762822	-6.61072715	2.357544066	0.133353642

CLK1	0.035042926	0.059173383	0.032138722	0.024130457	-0.002904204	-0.027034661	8	0.074007844	0.816496581	0.816496581	0.816496581	0.816496581	0.816496581	0.816496581	22	36	0	P_129576	P_129576	P_129576	chr2	200853243	200853303	-	CLK1	CDC-like kinase 1 [Source:HGNC Symbol;Acc:HGNC:2068]	ENSG00000013441	protein_coding	3'UTR;exon	multiple Ref	1	3606	2:200853243:200853303	ENSG00000013441	CLK1	7.679534873	0.006605143	0.528086273	0.89172495	0.484320803	0.363638677	-0.04376547	-0.407404147	9.961963646	0.60900082	0.393569118	0.638629501	0.723726333	0.965956171	0.692327809	-6.848288559	-5.779447902	-6.920517236	-7.077777821	-6.295666574	-6.981456453	0.26812363	0.846935875

FAM118A	-0.015441072	-0.139252432	-0.069682926	-0.12381136	-0.054241854	0.069569505	8	0.063292673	0.816496581	0.816496581	0.816496581	0.816496581	0.816496581	0.816496581	22	38	0	A_23_P120973	A_23_P120973	A_23_P120973	chr22	45332643	45332703	+	FAM118A	"family with sequence similarity 118, member A [Source:HGNC Symbol;Acc:HGNC:1313]"	ENSG00000100376	protein_coding	exon	coding exon	1	3608	22:45332643:45332703	ENSG00000100376	FAM118A	7.186499257	0.00542369	-0.256788539	-2.315799607	-1.15884291	-2.059011068	-0.902054371	1.156956697	9.961963646	0.802567551	0.043172786	0.273544438	0.06660341	0.388317518	0.274279102	-6.962496716	-3.943486326	-6.359471094	-5.220282193	-5.881213341	-6.385570989	2.193927088	0.152016886

TAF1C	-0.158488672	-0.126944147	-0.097491806	0.031544525	0.060996866	0.029452342	8	0.186539498	0.816496581	0.816496581	0.816496581	0.816496581	0.816496581	0.816496581	22	40	0	P_227165	P_227165	P_227165	chr16	84178793	84178853	+	TAF1C;DNAAF1	"TATA box binding protein (TBP)-associated factor, RNA polymerase I, C, 110kDa [Source:HGNC Symbol;Acc:HGNC:11534];dynein, axonemal, assembly factor 1 [Source:HGNC Symbol;Acc:HGNC:30539]"	ENSG00000103168	protein_coding	"3'ext;3'ext,3'UTR;3'UTR;exon"	multiple non-Ref	0	3610	16:84178793:84178853	ENSG00000103168	TAF1C	8.210547047	0.03015056	-1.117882525	-0.895386667	-0.687647795	0.222495858	0.43023473	0.207738872	9.961963646	0.289850298	0.39170179	0.507368333	0.828423257	0.676183484	0.839618183	-6.356253387	-5.776264798	-6.794872686	-7.122608209	-6.199165563	-7.047853837	0.46699998	0.711804577

TRAFD1	-0.029181691	0.053711593	-0.11191323	0.082893284	-0.082731539	-0.165624823	8	0.094454552	0.816496581	0.816496581	0.816496581	0.816496581	0.816496581	0.816496581	22	41	0	P_217571	P_217571	P_217571	chr12	112153376	112153436	-	TRAFD1	TRAF-type zinc finger domain containing 1 [Source:HGNC Symbol;Acc:HGNC:24808]	ENSG00000135148	protein_coding	3'ext;3'UTR	multiple non-Ref	0	3611	12:112153376:112153436	ENSG00000135148	TRAFD1	7.337080308	0.009371265	-0.369195975	0.679539247	-1.415884865	1.048735222	-1.046688889	-2.095424112	9.961963646	0.71970791	0.512276086	0.187306236	0.319076653	0.319974528	0.062660922	-6.924487765	-5.944355701	-6.050340422	-6.579275826	-5.744834767	-5.086939238	1.531502435	0.266438761

FBXO11	0.099865328	-0.072407828	0.064617847	-0.172273156	-0.035247481	0.137025674	8	0.050993681	0.816496581	0.816496581	0.816496581	0.816496581	0.816496581	0.816496581	22	42	0	P_234823	P_234823	P_234823	chr2	47818988	47819048	-	FBXO11	F-box protein 11 [Source:HGNC Symbol;Acc:HGNC:13590]	ENSG00000138081	protein_coding	exon	coding exon	1	3612	2:47818988:47819048	ENSG00000138081	FBXO11	8.735420186	0.004294911	1.866307778	-1.353175274	1.207594189	-3.219483052	-0.658713589	2.560769463	9.961963646	0.091674944	0.205907785	0.255091798	0.00922707	0.525011292	0.02841438	-5.370599785	-5.299237847	-6.304291178	-3.275049267	-6.070944846	-4.324708753	4.021497787	0.040940433

PDE9A	-0.245759822	-0.075613268	-0.335215683	0.170146554	-0.089455861	-0.259602415	8	0.054781199	0.816496581	0.816496581	0.816496581	0.816496581	0.816496581	0.816496581	22	43	0	P_132329	P_132329	P_132329	chr21	42697430	42697490	+	PDE9A	phosphodiesterase 9A [Source:HGNC Symbol;Acc:HGNC:8795]	ENSG00000160191	protein_coding	exon	coding exon	1	3613	21:42697430:42697490	ENSG00000160191	PDE9A	7.728805837	0.004616634	-4.429898463	-1.362953054	-6.042368622	3.066945409	-1.612470159	-4.679415568	9.961963646	0.001286191	0.202908795	0.000126848	0.011953544	0.138052046	0.000877236	-1.119748583	-5.287515302	1.24703062	-3.536740485	-5.073010079	-0.78397342	15.31583403	0.000462801

STAM2	0.073513976	0.113896074	0.131848359	0.040382099	0.058334384	0.017952285	8	0.063875204	0.816496581	0.816496581	0.816496581	0.816496581	0.816496581	0.816496581	22	44	0	P_236920	P_236920	P_236920	chr2	152144887	152144947	-	STAM2	signal transducing adaptor molecule (SH3 domain and ITAM motif) 2 [Source:HGNC Symbol;Acc:HGNC:11358]	ENSG00000115145	protein_coding	exon	coding exon	1	3614	2:152144887:152144947	ENSG00000115145	STAM2	6.851013395	0.00548318	1.215904007	1.883814497	2.180741104	0.66791049	0.964837097	0.296926607	9.961963646	0.252048338	0.089076215	0.054277839	0.519364212	0.357463515	0.772621327	-6.246573426	-4.593068233	-4.927432292	-6.910990091	-5.824056965	-7.023426838	1.874665306	0.197990371

F2	-0.340149007	0.116286646	-0.265463167	0.456435653	0.07468584	-0.381749813	8	0.083436056	0.816496581	0.816496581	0.816496581	0.816496581	0.816496581	0.816496581	22	45	0	P_108423	P_108423	P_108423	chr11	46739348	46739408	+	F2	coagulation factor II (thrombin) [Source:HGNC Symbol;Acc:HGNC:3535]	ENSG00000180210	protein_coding	exon	coding exon	1	3615	11:46739348:46739408	ENSG00000180210	F2	4.427505422	0.007797209	-4.717862276	1.612894266	-3.68197065	6.330756542	1.035891625	-5.294864917	9.961963646	0.000827657	0.137959493	0.00425973	8.71E-05	0.324743696	0.000354594	-0.663908647	-4.969986791	-2.393842703	1.565363832	-5.755581932	0.15835628	17.93395076	0.00024303

MPDU1	-0.270755392	0.014794505	-0.068087709	0.285549897	0.202667683	-0.082882214	8	0.074112803	0.816496581	0.816496581	0.816496581	0.816496581	0.816496581	0.816496581	22	46	0	A_24_P198844	A_24_P198844	A_24_P198844	chr17	7587692	7587752	+	MPDU1	mannose-P-dolichol utilization defect 1 [Source:HGNC Symbol;Acc:HGNC:7207]	ENSG00000129255	protein_coding	"3'UTR;3'UTR,exon;exon"	multiple Ref	1	3616	17:7587692:7587752	ENSG00000129255	MPDU1	9.154230084	0.006617628	-4.076351062	0.2227383	-1.025092805	4.299089363	3.051258257	-1.247831105	9.961963646	0.002244982	0.828239664	0.329566847	0.001577414	0.012277091	0.240626622	-1.693943956	-6.156000825	-6.501762536	-1.460243568	-2.847680513	-6.281438156	7.844415727	0.005580034

TBC1D9B	-0.072947904	-0.041047648	-0.084182427	0.031900256	-0.011234524	-0.04313478	8	0.124155106	0.816496581	0.816496581	0.816496581	0.816496581	0.816496581	0.816496581	22	49	0	P_255916	P_255916	P_255916	chr5	179871461	179871521	-	TBC1D9B	"TBC1 domain family, member 9B (with GRAM domain) [Source:HGNC Symbol;Acc:HGNC:29097]"	ENSG00000197226	protein_coding	"5'UTR,exon;exon"	multiple Ref	1	3619	5:179871461:179871521	ENSG00000197226	TBC1D9B	7.714819716	0.01458536	-0.739775363	-0.416270203	-0.853706314	0.32350516	-0.113930951	-0.437436111	9.961963646	0.476507853	0.686038509	0.413324571	0.753001543	0.911555957	0.671126154	-6.707699768	-6.091556872	-6.663096493	-7.092680768	-6.289785873	-6.967841455	0.29305819	0.829569523

CARD19	0.000285999	0.129844517	-0.016847352	0.129558517	-0.017133351	-0.146691869	8	0.118722888	0.816496581	0.816496581	0.816496581	0.816496581	0.816496581	0.816496581	22	51	0	P_149100	P_149100	P_149100	chr9	93107756	93107816	+	C9orf89	chromosome 9 open reading frame 89 [Source:HGNC Symbol;Acc:HGNC:28148]	ENSG00000165233	protein_coding	exon	coding exon	1	3621	9:93107756:93107816	ENSG00000165233	CARD19	9.66210189	0.013525837	0.003011818	1.36737352	-0.177416986	1.364361702	-0.180428805	-1.544790506	9.961963646	0.997656379	0.20156508	0.862736332	0.202479784	0.860433561	0.153546245	-6.998355368	-5.282197048	-7.029958108	-6.21684893	-6.279399095	-5.903620164	1.030268552	0.420431587

STPG2	0.073383072	-0.030893959	0.0821089	-0.104277031	0.008725828	0.113002859	8	0.205812091	0.816496581	0.816496581	0.816496581	0.816496581	0.816496581	0.816496581	22	53	0	A_24_P220822	A_24_P220822	A_24_P220822	chr4	97972346	97972406	-	STPG2	sperm-tail PG-rich repeat containing 2 [Source:HGNC Symbol;Acc:HGNC:28712]	ENSG00000163116	protein_coding	exon	coding exon	1	3623	4:97972346:97972406	ENSG00000163116	STPG2	4.037545893	0.036222963	0.472225605	-0.198804959	0.528376966	-0.671030564	0.056151361	0.727181924	9.961963646	0.646940072	0.846414082	0.608806402	0.517456687	0.95633147	0.483853051	-6.878025539	-6.161294749	-6.896769189	-6.908804457	-6.295008506	-6.78995523	0.253988533	0.856773416

EPS15	-0.077606619	0.137000302	-0.079755681	0.214606922	-0.002149062	-0.216755983	8	0.063502529	0.816496581	0.816496581	0.816496581	0.816496581	0.816496581	0.816496581	22	54	0	P_201643	P_201643	P_201643	chr1	51363900	51363960	-	EPS15	epidermal growth factor receptor pathway substrate 15 [Source:HGNC Symbol;Acc:HGNC:3419]	ENSG00000085832	protein_coding	exon	coding exon	1	3624	1:51363900:51363960	ENSG00000085832	EPS15	8.271503435	0.005445059	-1.288080831	2.273871284	-1.323750021	3.561952115	-0.03566919	-3.597621305	9.961963646	0.226831965	0.046360479	0.215157924	0.005195302	0.97225075	0.004896807	-6.161476334	-4.008413808	-6.166159994	-2.689758803	-6.296008587	-2.558139704	5.70226853	0.015475545

COPS5	-0.00443685	-0.039997234	-0.055505008	-0.035560384	-0.051068158	-0.015507774	8	0.071211168	0.816496581	0.816496581	0.816496581	0.816496581	0.816496581	0.816496581	22	55	0	P_147360	P_147360	P_147360	chr8	67043249	67043309	-	COPS5	COP9 signalosome subunit 5 [Source:HGNC Symbol;Acc:HGNC:2240]	ENSG00000121022	protein_coding	3'UTR;exon	multiple Ref	1	3625	8:67043249:67043309	ENSG00000121022	COPS5	10.68021268	0.006278998	-0.068576476	-0.618202009	-0.857892013	-0.549625534	-0.789315537	-0.239690003	9.961963646	0.946683686	0.550313732	0.41111677	0.594682405	0.448306004	0.815430301	-6.995794683	-5.984542232	-6.65945396	-6.986881245	-5.975589664	-7.040083474	0.350902284	0.789511915

ABLIM1	-0.009121755	-0.089233891	0.008165006	-0.080112136	0.017286761	0.097398897	8	0.071556009	0.816496581	0.816496581	0.816496581	0.816496581	0.816496581	0.816496581	22	57	0	P_210024	P_210024	P_210024	chr10	114437861	114437921	-	ABLIM1	actin binding LIM protein 1 [Source:HGNC Symbol;Acc:HGNC:78]	ENSG00000099204	protein_coding	"3'UTR,exon;exon"	multiple Ref	1	3627	10:114437861:114437921	ENSG00000099204	ABLIM1	5.983879102	0.006318534	-0.14054515	-1.374887928	0.125803865	-1.234342778	0.266349016	1.500691793	9.961963646	0.89103046	0.199298089	0.902389887	0.245399682	0.795402076	0.16444629	-6.987592052	-5.273130104	-7.038484437	-6.374639456	-6.25909043	-5.963037204	0.962086019	0.448082825

LTA4H	0.313498398	-0.044637052	0.118887325	-0.358135449	-0.194611073	0.163524377	8	0.043322653	0.816496581	0.816496581	0.816496581	0.816496581	0.816496581	0.816496581	22	58	0	P_216779	P_216779	P_216779	chr12	96013188	96013248	+	LTA4H	leukotriene A4 hydrolase [Source:HGNC Symbol;Acc:HGNC:6710]	ENSG00000111144	protein_coding	3'UTR;exon;promoter	multiple non-Ref	0	3628	12:96013188:96013248	ENSG00000111144	LTA4H	9.505862968	0.003713899	6.300368163	-0.897069523	2.389275109	-7.197437686	-3.911093054	3.286344632	9.961963646	9.05E-05	0.390845689	0.038094345	3.00E-05	0.002929269	0.008241569	1.630327866	-5.77479821	-4.587415335	2.681225765	-1.470295162	-3.088236463	20.6846379	0.000133688

STX5	-0.007455312	0.085955475	0.053267297	0.093410787	0.06072261	-0.032688178	8	0.066108964	0.816496581	0.816496581	0.816496581	0.816496581	0.816496581	0.816496581	22	59	0	P_211997	P_211997	P_211997	chr11	62827154	62827214	-	STX5	syntaxin 5 [Source:HGNC Symbol;Acc:HGNC:11440]	ENSG00000162236	protein_coding	3'UTR;exon	multiple Ref	1	3629	11:62827154:62827214	ENSG00000162236	STX5	7.947351718	0.00571635	-0.120768053	1.392386442	0.862873046	1.513154495	0.983641099	-0.529513396	9.961963646	0.906275855	0.194102371	0.408500026	0.161299413	0.348578029	0.608046652	-6.990407307	-5.251888624	-6.655099314	-6.023300976	-5.806320647	-6.920334462	1.039240932	0.416931423

CERCAM	0.175185288	0.141152549	0.170574544	-0.034032739	-0.004610745	0.029421994	8	0.168250499	0.816496581	0.816496581	0.816496581	0.816496581	0.816496581	0.816496581	22	60	0	P_149808	P_149808	P_149808	chr9	128437288	128437348	+	CERCAM	cerebral endothelial cell adhesion molecule [Source:HGNC Symbol;Acc:HGNC:23723]	ENSG00000167123	protein_coding	3'UTR	3'UTR	1	3630	9:128437288:128437348	ENSG00000167123	CERCAM	5.128177037	0.024939736	1.358617897	1.094683132	1.322860099	-0.263934765	-0.035757798	0.228176967	9.961963646	0.20423389	0.299416071	0.215442993	0.797209678	0.972181848	0.824124035	-6.074964138	-5.587028227	-6.167252514	-7.111650867	-6.296005221	-7.043009975	0.819522917	0.512279305

NUP210L	-0.035378718	-0.022149337	-0.017260398	0.013229382	0.018118321	0.004888939	8	0.101502867	0.816496581	0.816496581	0.816496581	0.816496581	0.816496581	0.816496581	22	63	0	P_204118	P_204118	P_204118	chr1	154094934	154094994	-	NUP210L	nucleoporin 210kDa-like [Source:HGNC Symbol;Acc:HGNC:29915]	ENSG00000143552	protein_coding	exon	coding exon	1	3633	1:154094934:154094994	ENSG00000143552	NUP210L	3.830431635	0.01048042	-0.423251381	-0.26498239	-0.206493833	0.158268991	0.216757547	0.058488557	9.961963646	0.681103893	0.796425143	0.840564451	0.877407131	0.832771811	0.954516082	-6.901479726	-6.145206985	-7.023884686	-7.135931861	-6.271755884	-7.069509992	0.06124182	0.979058779

LEMD3	0.037353573	-0.089169903	-0.075032119	-0.126523476	-0.112385692	0.014137784	8	0.047328212	0.816496581	0.816496581	0.816496581	0.816496581	0.816496581	0.816496581	22	64	0	P_216009	P_216009	P_216009	chr12	65240178	65240238	+	LEMD3	LEM domain containing 3 [Source:HGNC Symbol;Acc:HGNC:28887]	ENSG00000174106	protein_coding	exon	coding exon	1	3634	12:65240178:65240238	ENSG00000174106	LEMD3	8.78618607	0.004005494	0.7228526	-1.72558318	-1.451993979	-2.44843578	-2.174846579	0.273589202	9.961963646	0.486394489	0.11524803	0.177259075	0.034434856	0.054820597	0.789988847	-6.720512691	-4.816828563	-6.003535458	-4.587355377	-4.253096407	-7.030641073	2.734681028	0.099689818

PARN	0.044094688	-0.10648676	-0.015474892	-0.150581448	-0.059569579	0.091011868	8	0.040431924	0.816496581	0.816496581	0.816496581	0.816496581	0.816496581	0.816496581	22	65	0	P_118316	P_118316	P_118316	chr16	14435983	14436043	-	PARN	poly(A)-specific ribonuclease [Source:HGNC Symbol;Acc:HGNC:8609]	ENSG00000140694	protein_coding	3'UTR	3'UTR	1	3635	16:14435983:14436043	ENSG00000140694	PARN	7.899831867	0.00351947	0.910318323	-2.198379303	-0.319473346	-3.108697626	-1.229791669	1.878905956	9.961963646	0.3841517	0.052684459	0.755966309	0.011134132	0.247027383	0.08979786	-6.564128214	-4.124472537	-6.991668527	-3.465106138	-5.549616719	-5.422476782	3.41171505	0.06127964

ZBTB37	-0.01264176	-0.218237206	-0.17410148	-0.205595446	-0.16145972	0.044135726	8	0.10639811	0.816496581	0.816496581	0.816496581	0.816496581	0.816496581	0.816496581	22	66	0	A_32_P6682	A_32_P6682	A_32_P6682	chr1	173903343	173903403	+	ZBTB37	zinc finger and BTB domain containing 37 [Source:HGNC Symbol;Acc:HGNC:28365]	ENSG00000185278	protein_coding	3'UTR	3'UTR	1	3636	1:173903343:173903403	ENSG00000185278	ZBTB37	7.094956353	0.011297709	-0.14566593	-2.514659736	-2.006101478	-2.368993806	-1.860435547	0.508558259	9.961963646	0.887090366	0.030748086	0.07275384	0.039434636	0.092562264	0.622133956	-6.986793927	-3.631850486	-5.203757209	-4.719144904	-4.725198022	-6.931902929	3.283526479	0.066972467

SLC2A2	-0.062779954	0.030504319	0.01704253	0.093284274	0.079822484	-0.013461789	8	0.202348524	0.816496581	0.816496581	0.816496581	0.816496581	0.816496581	0.816496581	22	67	0	A_24_P405705	A_24_P405705	A_24_P405705	chr3	170996763	170996823	-	SLC2A2	"solute carrier family 2 (facilitated glucose transporter), member 2 [Source:HGNC Symbol;Acc:HGNC:11006]"	ENSG00000163581	protein_coding	3'UTR	3'UTR	1	3637	3:170996763:170996823	ENSG00000163581	SLC2A2	3.470966307	0.035087691	-0.410477378	0.199447948	0.111430044	0.609925326	0.521907422	-0.088017904	9.961963646	0.690144945	0.845924521	0.913488481	0.555567591	0.613140844	0.931606662	-6.9071909	-6.161160312	-7.040343994	-6.949902748	-6.153816058	-7.067147886	0.145471871	0.930235518

CA13	0.135888702	0.01071936	-0.304926555	-0.125169342	-0.440815257	-0.315645914	8	0.13045687	0.816496581	0.816496581	0.816496581	0.816496581	0.816496581	0.816496581	22	68	0	P_265238	P_265238	P_265238	chr8	85267904	85267964	+	CA13	carbonic anhydrase XIII [Source:HGNC Symbol;Acc:HGNC:14914]	ENSG00000185015	protein_coding	exon	coding exon	1	3638	8:85267904:85267964	ENSG00000185015	CA13	4.791961557	0.015873865	1.320953834	0.104201301	-2.964145618	-1.216752534	-4.285099452	-3.068346918	9.961963646	0.216054687	0.919077623	0.014243182	0.251739218	0.00161245	0.011925064	-6.121559126	-6.176366689	-3.617126056	-6.395109884	-0.895607966	-3.461370042	6.63320884	0.009685004

PRPF19	0.145609483	0.131736512	0.077891896	-0.013872971	-0.067717587	-0.053844615	8	0.298885022	0.816496581	0.816496581	0.816496581	0.816496581	0.816496581	0.816496581	22	69	0	P_108719	P_108719	P_108719	chr11	60890741	60890801	-	PRPF19	pre-mRNA processing factor 19 [Source:HGNC Symbol;Acc:HGNC:17896]	ENSG00000110107	protein_coding	3'UTR	3'UTR	1	3639	11:60890741:60890801	ENSG00000110107	PRPF19	10.65289425	0.073945357	0.655812718	0.593330037	0.350818472	-0.062482681	-0.304994246	-0.242511566	9.961963646	0.526800003	0.566186696	0.733029975	0.951414334	0.766647405	0.813303634	-6.768628693	-5.999839439	-6.980323695	-7.147478471	-6.247445577	-7.039344537	0.176823648	0.909655703

RETSAT	0.04264786	-0.03248574	-0.057744244	-0.0751336	-0.100392104	-0.025258504	8	0.086548417	0.816496581	0.816496581	0.816496581	0.816496581	0.816496581	0.816496581	22	70	0	P_128278	P_128278	P_128278	chr2	85342125	85342185	-	RETSAT	"retinol saturase (all-trans-retinol 13,14-reductase) [Source:HGNC Symbol;Acc:HGNC:25991]"	ENSG00000042445	protein_coding	3'UTR	3'UTR	1	3640	2:85342125:85342185	ENSG00000042445	RETSAT	7.80679089	0.008222067	0.576039632	-0.438781078	-0.779944724	-1.01482071	-1.355984356	-0.341163646	9.961963646	0.577369397	0.670183517	0.453555258	0.334204333	0.20504241	0.740066669	-6.820262262	-6.081600485	-6.724734497	-6.613829881	-5.401863997	-7.008164019	0.686268591	0.580707803

MAP1S	0.018782595	0.040783604	-0.025853767	0.022001008	-0.044636363	-0.066637371	8	0.147188412	0.816496581	0.816496581	0.816496581	0.816496581	0.816496581	0.816496581	22	71	0	P_125085	P_125085	P_125085	chr19	17734452	17734512	+	MAP1S	microtubule-associated protein 1S [Source:HGNC Symbol;Acc:HGNC:15715]	ENSG00000130479	protein_coding	3'UTR;exon	multiple Ref	1	3641	19:17734452:17734512	ENSG00000130479	MAP1S	11.61541015	0.019604401	0.164295036	0.356742158	-0.226147957	0.192447122	-0.390442993	-0.582890114	9.961963646	0.872784559	0.728725396	0.825658811	0.851258496	0.704427802	0.572924418	-6.983650607	-6.115439639	-7.019262921	-7.129397155	-6.216235982	-6.888882893	0.122440539	0.944740326

MRPL42	-0.09681647	-0.001281968	-0.011713509	0.095534502	0.085102961	-0.010431541	8	0.077879304	0.816496581	0.816496581	0.816496581	0.816496581	0.816496581	0.816496581	22	72	0	P_112409	P_112409	P_112409	chr12	93487600	93487660	+	MRPL42	mitochondrial ribosomal protein L42 [Source:HGNC Symbol;Acc:HGNC:14493]	ENSG00000198015	protein_coding	3'UTR;exon	multiple Ref	1	3642	12:93487600:93487660	ENSG00000198015	MRPL42	12.00420432	0.007077359	-1.409481107	-0.018663246	-0.170528525	1.390817861	1.238952582	-0.151865279	9.961963646	0.189137921	0.985478256	0.86800814	0.194563391	0.24375986	0.882324713	-6.010628745	-6.181903805	-7.031263035	-6.183404484	-5.539236206	-7.058793758	0.918084362	0.466949468

ATP5ME	-0.010274921	0.09933744	0.089826303	0.109612361	0.100101224	-0.009511137	8	0.04512987	0.816496581	0.816496581	0.816496581	0.816496581	0.816496581	0.816496581	22	73	0	P_137051	P_137051	P_137051	chr4	672438	672498	-	ATP5I	"ATP synthase, H+ transporting, mitochondrial Fo complex, subunit E [Source:HGNC Symbol;Acc:HGNC:846]"	ENSG00000169020	protein_coding	3'UTR;exon	multiple Ref	1	3643	4:672438:672498	ENSG00000169020	ATP5ME	16.5323866	0.003842269	-0.203015973	1.962748689	1.774823857	2.165764662	1.97783983	-0.187924832	9.961963646	0.84320914	0.078189237	0.106432248	0.05566707	0.07625521	0.854708266	-6.975916125	-4.478140468	-5.554055323	-5.050419612	-4.552365155	-7.05211977	2.613550056	0.109276165

HLA-DOB	-0.319151447	-0.200661474	-0.219527255	0.118489973	0.099624191	-0.018865781	8	0.293003069	0.816496581	0.816496581	0.816496581	0.816496581	0.816496581	0.816496581	22	74	0	A_24_P911788	A_24_P911788	A_24_P911788	chr6	32816596	32816656	-	HLA-DOB;TAP2	"major histocompatibility complex, class II, DO beta [Source:HGNC Symbol;Acc:HGNC:4937];Uncharacterized protein [Source:UniProtKB/TrEMBL;Acc:E7ENX8]"	ENSG00000241106	protein_coding	intron	intron sense	0	3644	6:32816596:32816656	ENSG00000241106	HLA-DOB	4.402321968	0.071149556	-1.465400544	-0.921347644	-1.007970864	0.5440529	0.45742968	-0.08662322	9.961963646	0.173648476	0.378641282	0.337323721	0.598369789	0.657175894	0.932687383	-5.938098864	-5.753382681	-6.518972722	-6.990116495	-6.186583302	-7.067280846	0.754170758	0.54478435

SILC1	-0.048156131	0.668725281	0.07147072	0.716881412	0.119626851	-0.597254561	8	0.249655923	0.816496581	0.816496581	0.816496581	0.816496581	0.816496581	0.816496581	22	75	0	A_23_P390639	A_23_P390639	A_23_P390639	chr2	5973838	5973898	+	LINC01105	long intergenic non-protein coding RNA 1105 [Source:HGNC Symbol;Acc:HGNC:26403]	ENSG00000232044	processed_transcript	exon	non-coding exon	1	3645	2:5973838:5973898	ENSG00000232044	SILC1	3.259976757	0.052259531	-0.257996887	3.582701451	0.382905	3.840698338	0.640901887	-3.199796452	9.961963646	0.80166083	0.005019435	0.709833519	0.003284218	0.536050734	0.009539717	-6.962159534	-1.932239304	-6.967639793	-2.218674081	-6.08273978	-3.236305479	6.408224101	0.010804396

KNTC1	-0.453232112	-0.157103233	-0.429931344	0.296128879	0.023300768	-0.272828111	8	0.082566235	0.816496581	0.816496581	0.816496581	0.816496581	0.816496581	0.816496581	22	77	0	P_217908	P_217908	P_217908	chr12	122534724	122534784	+	KNTC1	kinetochore associated 1 [Source:HGNC Symbol;Acc:HGNC:17255]	ENSG00000184445	protein_coding	exon	coding exon	1	3647	12:122534724:122534784	ENSG00000184445	KNTC1	8.058509093	0.007681254	-6.333594356	-2.195405234	-6.007982797	4.138189123	0.32561156	-3.812577563	9.961963646	8.68E-05	0.052949932	0.000132766	0.0020341	0.751454287	0.003438337	1.674638744	-4.129020883	1.199587584	-1.723819477	-6.240600983	-2.195778369	18.79940978	0.000199798

MAP3K7	0.073844509	0.106320672	0.103159976	0.032476163	0.029315467	-0.003160696	8	0.069659774	0.816496581	0.816496581	0.816496581	0.816496581	0.816496581	0.816496581	22	78	0	P_143207	P_143207	P_143207	chr6	90544551	90544611	-	MAP3K7	mitogen-activated protein kinase kinase kinase 7 [Source:HGNC Symbol;Acc:HGNC:6859]	ENSG00000135341	protein_coding	exon	coding exon	1	3648	6:90544551:90544611	ENSG00000135341	MAP3K7	6.290665779	0.006103493	1.157642865	1.666763979	1.617214489	0.509121113	0.459571624	-0.049549489	9.961963646	0.274011665	0.126644157	0.137019833	0.621753436	0.655689416	0.961460772	-6.312607015	-4.897468208	-5.780039394	-7.009686602	-6.185560897	-7.070037433	1.201081234	0.358951034

ZBTB11	0.03649079	-0.036351319	0.081267435	-0.072842109	0.044776644	0.117618753	8	0.058984155	0.816496581	0.816496581	0.816496581	0.816496581	0.816496581	0.816496581	22	79	0	P_245277	P_245277	P_245277	chr3	101652495	101652555	-	ZBTB11	zinc finger and BTB domain containing 11 [Source:HGNC Symbol;Acc:HGNC:16740]	ENSG00000066422	protein_coding	exon	coding exon	1	3649	3:101652495:101652555	ENSG00000066422	ZBTB11	7.139625252	0.005000616	0.632000127	-0.629584559	1.407506622	-1.261584686	0.775506496	2.037091182	9.961963646	0.541618246	0.54313478	0.189705757	0.235837786	0.456055368	0.06908919	-6.784688654	-5.977348153	-6.061089243	-6.342513877	-5.986395516	-5.178842556	1.519937083	0.269180461

KIAA0040	-0.072621744	0.161166038	-0.03052861	0.233787782	0.042093134	-0.191694648	8	0.069605998	0.816496581	0.816496581	0.816496581	0.816496581	0.816496581	0.816496581	22	80	0	P_104108	P_104108	P_104108	chr1	175160630	175160690	-	KIAA0040	KIAA0040 [Source:HGNC Symbol;Acc:HGNC:28950]	ENSG00000235750	protein_coding	3'UTR;exon	multiple Ref	1	3650	1:175160630:175160690	ENSG00000235750	KIAA0040	5.912246986	0.006097479	-1.139035198	2.52780751	-0.478825758	3.666842708	0.66020944	-3.006633268	9.961963646	0.281337237	0.030063953	0.642398717	0.004367301	0.52409034	0.013247139	-6.333179093	-3.611069454	-6.923341298	-2.51177807	-6.069940833	-3.567009888	5.139656943	0.021007844

FERMT2	-0.070402355	0.016411735	0.036283108	0.086814089	0.106685462	0.019871373	8	0.082637815	0.816496581	0.816496581	0.816496581	0.816496581	0.816496581	0.816496581	22	82	0	A_23_P88347	A_23_P88347	A_23_P88347	chr14	52858464	52858524	-	FERMT2	fermitin family member 2 [Source:HGNC Symbol;Acc:HGNC:15767]	ENSG00000073712	protein_coding	exon	coding exon	1	3652	14:52858464:52858524	ENSG00000073712	FERMT2	7.6476851	0.00769075	-0.983214923	0.229200606	0.506717326	1.21241553	1.489932249	0.27751672	9.961963646	0.3487776	0.823350032	0.623379345	0.25332241	0.167205936	0.787057258	-6.495081541	-6.154469366	-6.908689759	-6.400123676	-5.234538516	-7.029467619	0.840478668	0.50227608

CCDC120	0.047154539	-0.12341245	-0.037187774	-0.170566989	-0.084342313	0.086224675	8	0.130398193	0.816496581	0.816496581	0.816496581	0.816496581	0.816496581	0.816496581	22	83	0	A_24_P48983	A_24_P48983	A_24_P48983	chrX	49067745	49067805	+	CCDC120	coiled-coil domain containing 120 [Source:HGNC Symbol;Acc:HGNC:28910]	ENSG00000147144	protein_coding	3'UTR;exon	multiple Ref	1	3653	X:49067745:49067805	ENSG00000147144	CCDC120	7.547945235	0.015861573	0.458559824	-1.20013879	-0.361636857	-1.658698614	-0.820196681	0.838501933	9.961963646	0.656391394	0.257847394	0.725175997	0.128283538	0.431291363	0.421412469	-6.884817755	-5.474987838	-6.976167836	-5.822103915	-5.950820847	-6.700320389	0.98474677	0.438683141

ZNF558	0.156336803	0.036076156	0.030595971	-0.120260647	-0.125740832	-0.005480185	8	0.158518501	0.816496581	0.816496581	0.816496581	0.816496581	0.816496581	0.816496581	22	84	0	P_232242	P_232242	P_232242	chr19	8812576	8812636	-	ZNF558	zinc finger protein 558 [Source:HGNC Symbol;Acc:HGNC:26422]	ENSG00000167785	protein_coding	exon	coding exon	1	3654	19:8812576:8812636	ENSG00000167785	ZNF558	7.032874378	0.022385931	1.279732739	0.295310105	0.250450724	-0.984422633	-1.029282015	-0.044859382	9.961963646	0.229638394	0.773820205	0.807328329	0.348212267	0.327689454	0.965105871	-6.171499601	-6.136322237	-7.012973719	-6.644016082	-5.762116965	-7.070279275	0.636361876	0.608532875

SLC37A3	0.084825063	0.092056887	0.137640427	0.007231824	0.052815364	0.04558354	8	0.098989801	0.816496581	0.816496581	0.816496581	0.816496581	0.816496581	0.816496581	22	85	0	P_313699	P_313699	P_313699	chr7	140353069	140353129	-	SLC37A3	"solute carrier family 37, member 3 [Source:HGNC Symbol;Acc:HGNC:20651]"	ENSG00000157800	protein_coding	intron;promoter	multiple non-Ref	0	3655	7:140353069:140353129	ENSG00000157800	SLC37A3	6.900092047	0.0100758	1.034975467	1.123213074	1.679391205	0.088237608	0.644415738	0.556178131	9.961963646	0.325150815	0.287686277	0.124115325	0.93143643	0.533862233	0.590361993	-6.443409486	-5.557489105	-5.692270995	-7.145356902	-6.080436365	-6.904977007	0.98092138	0.440255066

SEPTIN5	0.225129583	0.182671113	0.410469254	-0.04245847	0.185339671	0.227798141	8	0.158138586	0.816496581	0.816496581	0.816496581	0.816496581	0.816496581	0.816496581	22	86	0	A_23_P29124	A_23_P29124	A_23_P29124	chr22	19724659	19724719	+	SEPT5;GP1BB	"septin 5 [Source:HGNC Symbol;Acc:HGNC:9164];glycoprotein Ib (platelet), beta polypeptide [Source:HGNC Symbol;Acc:HGNC:4440]"	ENSG00000184702	protein_coding	3'UTR	3'UTR	1	3656	22:19724659:19724719	ENSG00000184702	SEPTIN5	7.746989057	0.022289321	1.846842156	1.498535676	3.367269246	-0.34830648	1.52042709	1.86873357	9.961963646	0.094646894	0.164996105	0.007192035	0.734858317	0.159487423	0.091310703	-5.399820007	-5.119350929	-2.927897111	-7.083671779	-5.195057346	-5.437837419	3.820019731	0.046618609

PZP	0.130788835	0.116898115	0.222340769	-0.013890721	0.091551934	0.105442655	8	0.448231824	0.816496581	0.816496581	0.816496581	0.816496581	0.816496581	0.816496581	22	87	0	A_23_P139682	A_23_P139682	A_23_P139682	chr12	9151647	9152254	-	PZP	pregnancy-zone protein [Source:HGNC Symbol;Acc:HGNC:9750]	ENSG00000126838	protein_coding	"3'UTR,intron;exon,intron"	multiple non-Ref	0	3657	12:9151647:9152254	ENSG00000126838	PZP	3.765650285	0.163549788	0.396087656	0.354020282	0.673348257	-0.042067375	0.277260601	0.319327975	9.961963646	0.700391105	0.73070209	0.516042433	0.967276193	0.787248325	0.756073285	-6.913421738	-6.116446207	-6.805025223	-7.14864538	-6.255959475	-7.015956941	0.152704521	0.925562048

ERCC3	-0.068375166	-0.024451809	0.021679787	0.043923357	0.090054953	0.046131596	8	0.080340682	0.816496581	0.816496581	0.816496581	0.816496581	0.816496581	0.816496581	22	88	0	P_236406	P_236406	P_236406	chr2	127289347	127289407	-	ERCC3	excision repair cross-complementation group 3 [Source:HGNC Symbol;Acc:HGNC:3435]	ENSG00000163161	protein_coding	3'UTR;exon	multiple Ref	1	3658	2:127289347:127289407	ENSG00000163161	ERCC3	8.259473154	0.0073901	-0.97413437	-0.348362551	0.308869826	0.625771819	1.283004196	0.657232376	9.961963646	0.35304982	0.734817487	0.763783286	0.545533451	0.228535224	0.525924176	-6.503922878	-6.118514439	-6.995271097	-6.939591944	-5.488550017	-6.840408274	0.60588989	0.626116636

INTU	0.128399724	-0.007683471	0.189758932	-0.136083195	0.061359208	0.197442403	8	0.055633862	0.816496581	0.816496581	0.816496581	0.816496581	0.816496581	0.816496581	22	89	0	P_138555	P_138555	P_138555	chr4	127716794	127716854	+	INTU	inturned planar cell polarity protein [Source:HGNC Symbol;Acc:HGNC:29239]	ENSG00000164066	protein_coding	3'UTR	3'UTR	1	3659	4:127716794:127716854	ENSG00000164066	INTU	6.758907263	0.004692239	2.295723819	-0.137376675	3.392796228	-2.433100494	1.097072409	3.530172903	9.961963646	0.044671558	0.893469954	0.006890371	0.035348859	0.298419778	0.005477153	-4.694548794	-6.172148261	-2.884370503	-4.612881402	-5.693525632	-2.672554358	6.06432551	0.012830459

NINL	0.28219581	0.002009252	0.18745313	-0.280186558	-0.094742681	0.185443878	8	0.115216755	0.816496581	0.816496581	0.816496581	0.816496581	0.816496581	0.816496581	22	90	0	P_240503	P_240503	P_240503	chr20	25517752	25517812	-	NINL	ninein-like [Source:HGNC Symbol;Acc:HGNC:29163]	ENSG00000101004	protein_coding	exon	coding exon	1	3660	20:25517752:25517812	ENSG00000101004	NINL	5.222826075	0.012867153	3.046877261	0.02169396	2.023937483	-3.025183301	-1.022939778	2.002243523	9.961963646	0.012369033	0.983120457	0.070622808	0.012834762	0.330534853	0.073222707	-3.430743482	-6.181839329	-5.175953581	-3.608374833	-5.768356134	-5.233243101	4.597895588	0.02871605

OLR1	-0.0146509	-0.069756458	0.120905302	-0.055105557	0.135556203	0.19066176	8	0.233726945	0.816496581	0.816496581	0.816496581	0.816496581	0.816496581	0.816496581	22	91	0	P_214368	P_214368	P_214368	chr12	10172009	10172069	-	OLR1	oxidized low density lipoprotein (lectin-like) receptor 1 [Source:HGNC Symbol;Acc:HGNC:8133]	ENSG00000173391	protein_coding	exon	coding exon	1	3661	12:10172009:10172069	ENSG00000173391	OLR1	3.767026133	0.046076175	-0.083593351	-0.398008033	0.689846975	-0.314414681	0.773440327	1.087855008	9.961963646	0.935035679	0.699019997	0.506042199	0.759692046	0.45722232	0.302277431	-6.994548451	-6.099262436	-6.793294076	-7.095819786	-5.987997877	-6.460726201	0.420297023	0.742501169

VPS13B	0.252012732	-0.1259098	0.126107193	-0.377922532	-0.125905539	0.252016993	8	0.07233101	0.816496581	0.816496581	0.816496581	0.816496581	0.816496581	0.816496581	22	93	0	P_147678	P_147678	P_147678	chr8	99147950	99148010	+	VPS13B	vacuolar protein sorting 13 homolog B (yeast) [Source:HGNC Symbol;Acc:HGNC:2183]	ENSG00000132549	protein_coding	exon	coding exon	1	3663	8:99147950:99148010	ENSG00000132549	VPS13B	6.346114926	0.006408085	3.855706739	-1.926375945	1.929395981	-5.782082684	-1.926310758	3.855771926	9.961963646	0.003204936	0.083041464	0.082628093	0.000179855	0.083050407	0.003204597	-2.059517614	-4.531349401	-5.322122463	0.805935282	-4.628787876	-2.123441797	12.38501634	0.001067377

ANKRD40	-0.091551662	-0.028723595	-0.125036016	0.062828067	-0.033484353	-0.096312421	8	0.072738042	0.816496581	0.816496581	0.816496581	0.816496581	0.816496581	0.816496581	22	96	0	P_229162	P_229162	P_229162	chr17	50700567	50700627	-	ANKRD40	ankyrin repeat domain 40 [Source:HGNC Symbol;Acc:HGNC:28233]	ENSG00000154945	protein_coding	"5'UTR,exon;exon"	multiple Ref	1	3666	17:50700567:50700627	ENSG00000154945	ANKRD40	7.442457598	0.006455503	-1.395554543	-0.437843973	-1.905968446	0.95771057	-0.510413904	-1.468124473	9.961963646	0.193174063	0.670840236	0.085885801	0.360873787	0.62087988	0.172922682	-6.028402056	-6.082025015	-5.357863796	-6.669918188	-6.159957031	-6.006222044	1.517519484	0.269757638

DDX42	-0.014025767	-0.130018112	-0.034918534	-0.115992345	-0.020892768	0.095099578	8	0.057181917	0.816496581	0.816496581	0.816496581	0.816496581	0.816496581	0.816496581	22	98	0	A_23_P152651	A_23_P152651	A_23_P152651	chr17	63818631	63818691	+	DDX42	DEAD (Asp-Glu-Ala-Asp) box helicase 42 [Source:HGNC Symbol;Acc:HGNC:18676]	ENSG00000198231	protein_coding	3'UTR;exon	multiple Ref	1	3668	17:63818631:63818691	ENSG00000198231	DDX42	8.932897284	0.004832489	-0.247108005	-2.290678054	-0.615199828	-2.043570049	-0.368091824	1.675478225	9.961963646	0.809842624	0.045056141	0.552216179	0.068345453	0.720505602	0.124894067	-6.965141598	-3.9824258	-6.844291698	-5.24457563	-6.225120927	-5.721565965	2.13427276	0.159570894

ZFP28	-0.192867027	-0.112553461	-0.320929702	0.080313566	-0.128062675	-0.208376241	8	0.053233836	0.816496581	0.816496581	0.816496581	0.816496581	0.816496581	0.816496581	22	102	0	P_233368	P_233368	P_233368	chr19	56549058	56549118	+	ZFP28	ZFP28 zinc finger protein [Source:HGNC Symbol;Acc:HGNC:17801]	ENSG00000196867	protein_coding	exon	coding exon	1	3672	19:56549058:56549118	ENSG00000196867	ZFP28	5.986163659	0.004482413	-3.528155181	-2.058963016	-5.870831362	1.469192165	-2.342676181	-3.811868345	9.961963646	0.005495575	0.066608765	0.000159503	0.172638911	0.041242776	0.00344232	-2.610500428	-4.335484819	1.008675624	-6.081836719	-3.990952652	-2.196967516	12.22181102	0.001123117

DBNDD1	0.013779265	-0.002402472	0.042135251	-0.016181737	0.028355986	0.044537724	8	0.095353859	0.816496581	0.816496581	0.816496581	0.816496581	0.816496581	0.816496581	22	104	0	P_119915	P_119915	P_119915	chr16	90009979	90010039	-	DBNDD1	dysbindin (dystrobrevin binding protein 1) domain containing 1 [Source:HGNC Symbol;Acc:HGNC:28455]	ENSG00000003249	protein_coding	5'UTR;exon	multiple Ref	1	3674	16:90009979:90010039	ENSG00000003249	DBNDD1	7.456347067	0.009508344	0.173068986	-0.0301753	0.52922309	-0.203244286	0.356154104	0.55939839	9.961963646	0.866063095	0.976523226	0.608240686	0.843035459	0.729152279	0.588244876	-6.982039991	-6.181607432	-6.896293938	-7.127069958	-6.229657787	-6.903074299	0.132003508	0.938793795

COTL1	-0.12085884	0.039766093	-0.09049978	0.160624932	0.030359059	-0.130265873	8	0.046071207	0.816496581	0.816496581	0.816496581	0.816496581	0.816496581	0.816496581	22	108	0	P_119711	P_119711	P_119711	chr16	84566893	84566953	-	COTL1	coactosin-like F-actin binding protein 1 [Source:HGNC Symbol;Acc:HGNC:18304]	ENSG00000103187	protein_coding	exon	coding exon	1	3678	16:84566893:84566953	ENSG00000103187	COTL1	6.803891894	0.003911212	-2.366837029	0.778758602	-1.772300908	3.145595631	0.594536121	-2.55105951	9.961963646	0.039579853	0.454222539	0.106868537	0.010457814	0.565411154	0.028890761	-4.578006377	-5.872120902	-5.557761063	-3.401796884	-6.112044524	-4.341010007	4.350930447	0.033330213

PNPLA1	0.151192373	-0.219744997	0.028399881	-0.37093737	-0.122792493	0.248144877	8	0.383939443	0.816496581	0.816496581	0.816496581	0.816496581	0.816496581	0.816496581	22	109	0	P_142480	P_142480	P_142480	chr6	36291319	36291379	+	PNPLA1	patatin-like phospholipase domain containing 1 [Source:HGNC Symbol;Acc:HGNC:21246]	ENSG00000180316	protein_coding	5'UTR;exon	multiple Ref	1	3679	6:36291319:36291379	ENSG00000180316	PNPLA1	5.154783203	0.120584547	0.533248553	-0.775030505	0.100165075	-1.308279059	-0.433083479	0.87519558	9.961963646	0.605552994	0.456324031	0.9222004	0.220158656	0.674180848	0.40207587	-6.845381628	-5.874992908	-7.041644233	-6.286279137	-6.19788227	-6.668343388	0.593363674	0.633474933

UBE2A	0.031257568	0.080560549	0.113971892	0.049302981	0.082714323	0.033411342	8	0.049847009	0.816496581	0.816496581	0.816496581	0.816496581	0.816496581	0.816496581	22	110	0	A_32_P79192	A_32_P79192	A_32_P79192	chrX	119583649	119583709	-	UBE2A;NKRF	ubiquitin-conjugating enzyme E2A [Source:HGNC Symbol;Acc:HGNC:12472];NFKB repressing factor [Source:HGNC Symbol;Acc:HGNC:19374]	ENSG00000077721	protein_coding	3'ext;3'UTR;exon	multiple non-Ref	0	3680	X:119583649:119583709	ENSG00000077721	UBE2A	9.77658844	0.004202053	0.590568191	1.522079302	2.153340053	0.931511111	1.562771861	0.63126075	9.961963646	0.567964854	0.159078256	0.056845455	0.373613467	0.149286839	0.542082179	-6.811321253	-5.089135165	-4.971355593	-6.694747259	-5.139433908	-6.857953261	1.835138092	0.204739381

PSD	-0.116886785	-0.00049536	-0.180107863	0.116391425	-0.063221078	-0.179612503	8	0.150587656	0.816496581	0.816496581	0.816496581	0.816496581	0.816496581	0.816496581	22	111	0	P_308602	P_308602	P_308602	chr10	102397950	102398010	-	PSD;NFKB2	pleckstrin and Sec7 domain containing [Source:HGNC Symbol;Acc:HGNC:9507];nuclear factor of kappa light polypeptide gene enhancer in B-cells 2 (p49/p100) [Source:HGNC Symbol;Acc:HGNC:7795]	ENSG00000059915	protein_coding	"3'ext;exon,intron;promoter"	multiple non-Ref	0	3681	10:102397950:102398010	ENSG00000059915	PSD	6.250185238	0.020417264	-1.001872069	-0.004245883	-1.543759093	0.997626186	-0.541887023	-1.539513209	9.961963646	0.340119245	0.996696107	0.153793772	0.342075555	0.599806173	0.154816426	-6.476704987	-6.182077921	-5.881223781	-6.630996666	-6.142833907	-5.910786438	1.17432638	0.36789199

RGL4	0.109329995	-0.370213581	-0.020351804	-0.479543576	-0.129681799	0.349861777	8	0.208006305	0.816496581	0.816496581	0.816496581	0.816496581	0.816496581	0.816496581	22	114	0	A_23_P306941	A_23_P306941	A_23_P306941	chr22	23699059	23699119	+	RGL4	ral guanine nucleotide dissociation stimulator-like 4 [Source:HGNC Symbol;Acc:HGNC:31911]	ENSG00000159496	protein_coding	3'UTR;exon	multiple Ref	1	3684	22:23699059:23699119	ENSG00000159496	RGL4	4.128259611	0.036952141	0.69657062	-2.358729676	-0.129666783	-3.055300296	-0.826237403	2.229062893	9.961963646	0.502000863	0.040130419	0.899410829	0.012192879	0.428014272	0.050019448	-6.739881818	-3.87669514	-7.037946389	-3.556717592	-5.945879017	-4.872812135	3.508646553	0.057354025

ARAP2	-0.168370005	0.015745231	0.008087088	0.184115236	0.176457093	-0.007658143	8	0.105242376	0.816496581	0.816496581	0.816496581	0.816496581	0.816496581	0.816496581	22	115	0	P_248491	P_248491	P_248491	chr4	36159330	36159390	-	ARAP2	"ArfGAP with RhoGAP domain, ankyrin repeat and PH domain 2 [Source:HGNC Symbol;Acc:HGNC:16924]"	ENSG00000047365	protein_coding	exon	coding exon	1	3685	4:36159330:36159390	ENSG00000047365	ARAP2	6.30115747	0.011101282	-1.957148535	0.183024024	0.094005065	2.140172559	2.0511536	-0.089018959	9.961963646	0.078918584	0.858450413	0.926968977	0.058120407	0.067484548	0.930831048	-5.232365677	-6.164459199	-7.042296807	-5.091431258	-4.44223149	-7.067051144	2.111376531	0.162586148

RRP8	-0.250183203	0.057091318	-0.175762488	0.307274521	0.074420715	-0.232853806	8	0.091427557	0.816496581	0.816496581	0.816496581	0.816496581	0.816496581	0.816496581	22	116	0	A_23_P356565	A_23_P356565	A_23_P356565	chr11	6599977	6600037	-	RRP8	"ribosomal RNA processing 8, methyltransferase, homolog (yeast) [Source:HGNC Symbol;Acc:HGNC:29030]"	ENSG00000132275	protein_coding	3'UTR;exon	multiple Ref	1	3686	11:6599977:6600037	ENSG00000132275	RRP8	10.22540053	0.008919415	-3.244408922	0.740367776	-2.279311228	3.984776698	0.965097694	-3.019679004	9.961963646	0.008846243	0.476164071	0.045934334	0.002600477	0.357339257	0.012955744	-3.093325052	-5.901111513	-4.76789539	-1.977864573	-5.823813086	-3.544683437	7.032985593	0.008019413

FHL2	-0.341625562	0.20377988	-0.096345815	0.545405441	0.245279746	-0.300125695	8	0.108567748	0.816496581	0.816496581	0.816496581	0.816496581	0.816496581	0.816496581	22	119	0	P_128611	P_128611	P_128611	chr2	105361202	105361262	+	FHL2;AC012360.6	four and a half LIM domains 2 [Source:HGNC Symbol;Acc:HGNC:3703];	ENSG00000115641	protein_coding;antisense	3'ext;3'UTR;promoter	multiple non-Ref	0	3689	2:105361202:105361262	ENSG00000115641	FHL2	5.139545956	0.011672252	-3.872742815	2.310093719	-1.092197442	6.182836534	2.780545373	-3.402291161	9.961963646	0.003117364	0.043593711	0.300455286	0.000105462	0.019498378	0.006781525	-2.031114504	-3.952340397	-6.432081888	1.364786511	-3.288036753	-2.890227394	13.17700841	0.00083969

POMK	-0.166857592	-0.168740246	-0.26177054	-0.001882654	-0.094912948	-0.093030294	8	0.120385929	0.816496581	0.816496581	0.816496581	0.816496581	0.816496581	0.816496581	22	120	0	A_23_P83835	A_23_P83835	A_23_P83835	chr8	43122725	43122785	+	POMK	protein-O-mannose kinase [Source:HGNC Symbol;Acc:HGNC:26267]	ENSG00000185900	protein_coding	exon	coding exon	1	3690	8:43122725:43122785	ENSG00000185900	POMK	6.3515798	0.01384517	-1.736770581	-1.756366562	-2.724690967	-0.019595981	-0.987920386	-0.968324405	9.961963646	0.113188679	0.109662014	0.021456498	0.984752601	0.346578759	0.355803297	-5.562153667	-4.774049767	-4.025052139	-7.149403069	-5.802245247	-6.581989773	2.573193211	0.112708673

GPR78	-0.137381708	-0.455278001	-0.192783681	-0.317896293	-0.055401973	0.26249432	8	0.333522851	0.816496581	0.816496581	0.816496581	0.816496581	0.816496581	0.816496581	22	122	0	P_137330	P_137330	P_137330	chr4	8587432	8587492	+	GPR78	G protein-coupled receptor 78 [Source:HGNC Symbol;Acc:HGNC:4528]	ENSG00000155269	protein_coding	3'UTR;exon	multiple Ref	1	3692	4:8587432:8587492	ENSG00000155269	GPR78	5.898118381	0.091536456	-0.556131502	-1.842999637	-0.780402859	-1.286868135	-0.224271358	1.062596778	9.961963646	0.590392679	0.095243994	0.453297695	0.227237883	0.827078988	0.313044752	-6.83217528	-4.651674324	-6.724366756	-6.3122456	-6.270002163	-6.48729186	1.191732947	0.362047448

SLC2A4RG	-0.139358827	-0.285448175	-0.520992687	-0.146089348	-0.38163386	-0.235544512	8	0.118809249	0.816496581	0.816496581	0.816496581	0.816496581	0.816496581	0.816496581	22	124	0	P_241335	P_241335	P_241335	chr20	63742361	63742421	+	SLC2A4RG	SLC2A4 regulator [Source:HGNC Symbol;Acc:HGNC:15930]	ENSG00000125520	protein_coding	exon	coding exon	1	3694	20:63742361:63742421	ENSG00000125520	SLC2A4RG	11.44717303	0.013542311	-1.466674449	-3.004183904	-5.4831594	-1.537509456	-4.016484951	-2.478975495	9.961963646	0.173308716	0.013302588	0.000271693	0.155301114	0.002471125	0.032683427	-5.936425267	-2.850848369	0.454386626	-5.990409052	-1.306460345	-4.461618951	10.98044968	0.001684049

CLK4	0.057940456	0.033522629	-0.020349197	-0.024417826	-0.078289652	-0.053871826	8	0.060262158	0.816496581	0.816496581	0.816496581	0.816496581	0.816496581	0.816496581	22	125	0	P_141331	P_141331	P_141331	chr5	178603298	178603358	-	CLK4	CDC-like kinase 4 [Source:HGNC Symbol;Acc:HGNC:13659]	ENSG00000113240	protein_coding	3'UTR;exon	multiple Ref	1	3695	5:178603298:178603358	ENSG00000113240	CLK4	7.167678625	0.005122999	0.991437662	0.573616429	-0.348201607	-0.417821233	-1.339639269	-0.921818036	9.961963646	0.344941827	0.578946196	0.734934686	0.684940821	0.210120784	0.378407523	-6.487017316	-6.011549277	-6.981310471	-7.05497592	-5.421565053	-6.626030724	0.708697541	0.568596259

FBN3	0.563902057	0.287691945	0.176936533	-0.276210112	-0.386965523	-0.110755412	8	0.275936887	0.816496581	0.816496581	0.816496581	0.816496581	0.816496581	0.816496581	22	127	0	A_23_P101893	A_23_P101893	A_23_P101893	chr19	8072080	8072140	-	FBN3	fibrillin 3 [Source:HGNC Symbol;Acc:HGNC:18794]	ENSG00000142449	protein_coding	exon	coding exon	1	3697	19:8072080:8072140	ENSG00000142449	FBN3	5.353205499	0.063352192	2.743901302	1.399885483	0.860958705	-1.34401582	-1.882942597	-0.538926778	9.961963646	0.020761703	0.19191112	0.40950435	0.208750785	0.089204005	0.601772287	-3.946827951	-5.242731371	-6.656775485	-6.242270311	-4.69242777	-6.914994057	2.645369505	0.106656449

WDPCP	0.249764497	0.063055997	0.148790795	-0.1867085	-0.100973702	0.085734798	8	0.055563317	0.816496581	0.816496581	0.816496581	0.816496581	0.816496581	0.816496581	22	128	0	P_311402	P_311402	P_311402	chr2	63172947	63173007	-	WDPCP	WD repeat containing planar cell polarity effector [Source:HGNC Symbol;Acc:HGNC:28027]	ENSG00000143951	protein_coding	intron	intron sense	0	3698	2:63172947:63173007	ENSG00000143951	WDPCP	6.539595875	0.00468594	4.468666812	1.128167711	2.662093682	-3.340499102	-1.806573131	1.533925971	9.961963646	0.001211249	0.285686179	0.023886237	0.007523058	0.101079626	0.156171241	-1.057735308	-5.552299841	-4.131031093	-3.067688198	-4.802871354	-5.918357219	7.517342974	0.006438306

SAXO1	0.127104298	0.17753427	0.158670402	0.050429972	0.031566104	-0.018863868	8	0.13401922	0.816496581	0.816496581	0.816496581	0.816496581	0.816496581	0.816496581	22	129	0	A_23_P344578	A_23_P344578	A_23_P344578	chr9	18927916	18927976	-	FAM154A	"family with sequence similarity 154, member A [Source:HGNC Symbol;Acc:HGNC:28566]"	ENSG00000155875	protein_coding	3'UTR	3'UTR	1	3699	9:18927916:18927976	ENSG00000155875	SAXO1	4.082758476	0.016630468	1.207128862	1.686069982	1.506916953	0.47894112	0.29978809	-0.179153029	9.961963646	0.255263093	0.122796245	0.162867795	0.642319477	0.770500649	0.861408825	-6.256674013	-4.871163549	-5.930894724	-7.025601328	-6.249104531	-7.053872858	1.15369068	0.374956006

THRA	0.181026963	0.016204166	0.162334772	-0.164822796	-0.01869219	0.146130606	8	0.067474793	0.816496581	0.816496581	0.816496581	0.816496581	0.816496581	0.816496581	22	130	0	P_121348	P_121348	P_121348	chr17	40089650	40089710	+	THRA	"thyroid hormone receptor, alpha [Source:HGNC Symbol;Acc:HGNC:11796]"	ENSG00000126351	protein_coding	3'UTR	3'UTR	1	3700	17:40089650:40089710	ENSG00000126351	THRA	9.384764607	0.005862869	2.895568223	0.259189394	2.596582308	-2.636378829	-0.298985915	2.337392914	9.961963646	0.016013871	0.800766299	0.026723824	0.024962363	0.771094951	0.0416154	-3.688973867	-6.146796334	-4.241520956	-4.271717005	-6.249357654	-4.696075402	4.61617095	0.028405935

ST3GAL4	-0.025276016	0.025750882	0.062026262	0.051026898	0.087302278	0.03627538	8	0.083932407	0.816496581	0.816496581	0.816496581	0.816496581	0.816496581	0.816496581	22	132	0	P_110481	P_110481	P_110481	chr11	126351628	126351688	-	ST3GAL4	"ST3 beta-galactoside alpha-2,3-sialyltransferase 4 [Source:HGNC Symbol;Acc:HGNC:10864]"	ENSG00000110080	protein_coding	promoter	promoter antisense	0	3702	11:126351628:126351688	ENSG00000110080	ST3GAL4	6.167600676	0.007863921	-0.349087805	0.355646193	0.856646532	0.704733998	1.205734337	0.501000339	9.961963646	0.734289446	0.729521063	0.411772868	0.497120793	0.255776996	0.627254815	-6.932267757	-6.115845837	-6.660539423	-6.884604742	-5.576609128	-6.935966616	0.530606072	0.67146445

TRIP11	0.177858991	-0.054438549	0.12967612	-0.23229754	-0.04818287	0.184114669	8	0.051075118	0.816496581	0.816496581	0.816496581	0.816496581	0.816496581	0.816496581	22	133	0	P_221963	P_221963	P_221963	chr14	92025315	92025375	-	TRIP11	thyroid hormone receptor interactor 11 [Source:HGNC Symbol;Acc:HGNC:12305]	ENSG00000100815	protein_coding	5'UTR;exon	multiple Ref	1	3703	14:92025315:92025375	ENSG00000100815	TRIP11	9.809027144	0.004301586	3.321292539	-1.016571303	2.421538149	-4.337863842	-0.89975439	3.438109452	9.961963646	0.007770201	0.333410579	0.036053725	0.001484443	0.389482569	0.006386654	-2.962170326	-5.664845722	-4.534025984	-1.39718979	-5.883245982	-2.829172285	8.229244272	0.004739321

ANO10	0.011848731	0.094810421	0.142379549	0.08296169	0.130530818	0.047569128	8	0.093113158	0.816496581	0.816496581	0.816496581	0.816496581	0.816496581	0.816496581	22	134	0	P_243987	P_243987	P_243987	chr3	43598531	43598591	-	ANO10	anoctamin 10 [Source:HGNC Symbol;Acc:HGNC:25519]	ENSG00000160746	protein_coding	exon	coding exon	1	3704	3:43598531:43598591	ENSG00000160746	ANO10	8.148295803	0.009169215	0.151548411	1.212650424	1.821072187	1.061102013	1.669523776	0.608421763	9.961963646	0.882568183	0.253236458	0.098718455	0.313691004	0.126087533	0.556525054	-6.98584199	-5.461186625	-5.485652047	-6.566449527	-4.995310354	-6.872842181	1.515536007	0.270232226

PRKCZ	0.160240799	-0.0603728	0.031545655	-0.220613599	-0.128695144	0.091918455	8	0.062555276	0.816496581	0.816496581	0.816496581	0.816496581	0.816496581	0.816496581	22	135	0	P_200040	P_200040	P_200040	chr1	2174773	2174833	+	PRKCZ	"protein kinase C, zeta [Source:HGNC Symbol;Acc:HGNC:9412]"	ENSG00000067606	protein_coding	exon	coding exon	1	3705	1:2174773:2174833	ENSG00000067606	PRKCZ	9.931293484	0.005349167	2.683339713	-1.010983047	0.528253163	-3.69432276	-2.155086549	1.53923621	9.961963646	0.023032168	0.33594933	0.608889198	0.004173946	0.056678378	0.154883351	-4.049334409	-5.670236965	-6.896838666	-2.465282873	-4.283566687	-5.911162173	4.860519489	0.024620301

CWF19L2	-0.22048254	-0.065090857	-0.31698332	0.155391683	-0.09650078	-0.251892463	8	0.061672465	0.816496581	0.816496581	0.816496581	0.816496581	0.816496581	0.816496581	22	136	0	A_24_P922653	A_24_P922653	A_24_P922653	chr11	107454652	107454712	-	CWF19L2	"CWF19-like 2, cell cycle control (S. pombe) [Source:HGNC Symbol;Acc:HGNC:26508]"	ENSG00000152404	protein_coding	intron	intron sense	0	3706	11:107454652:107454712	ENSG00000152404	CWF19L2	8.405903237	0.005261096	-3.722902911	-1.099075422	-5.352342748	2.623827489	-1.629439837	-4.253267326	9.961963646	0.003982289	0.297586524	0.000326735	0.025505045	0.134391613	0.001695268	-2.281853515	-5.582518765	0.262430679	-4.292936898	-5.050057291	-1.466825455	11.8908956	0.001247242

COL8A1	0.290338096	0.040557865	0.208771104	-0.249780231	-0.081566992	0.168213239	8	0.217740473	0.816496581	0.816496581	0.816496581	0.816496581	0.816496581	0.816496581	22	138	0	P_311940	P_311940	P_311940	chr3	99792408	99792468	-	COL8A1	"collagen, type VIII, alpha 1 [Source:HGNC Symbol;Acc:HGNC:2215]"	ENSG00000144810	protein_coding	intron	intron antisense	0	3708	3:99792408:99792468	ENSG00000144810	COL8A1	3.803590232	0.040280233	1.771755002	0.247499729	1.274001768	-1.524255273	-0.497753234	1.026502039	9.961963646	0.106963155	0.809547862	0.231581486	0.15854078	0.629461304	0.328934402	-5.51111119	-6.149897949	-6.226436295	-6.008349425	-6.16657157	-6.524390042	1.408045211	0.297430127

PDS5A	-0.049111929	-0.110110229	-0.11148151	-0.060998299	-0.06236958	-0.001371281	8	0.056896299	0.816496581	0.816496581	0.816496581	0.816496581	0.816496581	0.816496581	22	139	0	P_248658	P_248658	P_248658	chr4	39927960	39928020	-	PDS5A	"PDS5, regulator of cohesion maintenance, homolog A (S. cerevisiae) [Source:HGNC Symbol;Acc:HGNC:29088]"	ENSG00000121892	protein_coding	exon	coding exon	1	3709	4:39927960:39928020	ENSG00000121892	PDS5A	8.887857388	0.004806323	-0.867613189	-1.945211433	-1.969436537	-1.077598244	-1.101823348	-0.024225104	9.961963646	0.4060205	0.080494595	0.077326526	0.306615025	0.296446313	0.981151437	-6.602489325	-4.503847335	-5.260585313	-6.54915453	-5.688590305	-7.071057933	1.798516661	0.211231017

MDN1	-0.112955815	-0.097510262	-0.158877586	0.015445553	-0.045921771	-0.061367324	8	0.060437041	0.816496581	0.816496581	0.816496581	0.816496581	0.816496581	0.816496581	22	140	0	P_258291	P_258291	P_258291	chr6	89749235	89749295	-	MDN1	"MDN1, midasin homolog (yeast) [Source:HGNC Symbol;Acc:HGNC:18302]"	ENSG00000112159	protein_coding	exon	coding exon	1	3710	6:89749235:89749295	ENSG00000112159	MDN1	6.180920269	0.00513995	-1.929633295	-1.665775664	-2.714118604	0.263857631	-0.784485309	-1.048342941	9.961963646	0.082595692	0.126844027	0.021848775	0.797267451	0.451006761	0.319248628	-5.274551015	-4.898810409	-4.042976285	-7.111672975	-5.979388496	-6.502064552	2.60813233	0.109729731

ESM1	-0.015312459	-0.030040776	-0.258132874	-0.014728318	-0.242820415	-0.228092097	8	0.171596214	0.816496581	0.816496581	0.816496581	0.816496581	0.816496581	0.816496581	22	141	0	P_139499	P_139499	P_139499	chr5	54977897	54977957	-	ESM1	endothelial cell-specific molecule 1 [Source:HGNC Symbol;Acc:HGNC:3466]	ENSG00000164283	protein_coding	3'UTR	3'UTR	1	3711	5:54977897:54977957	ENSG00000164283	ESM1	3.600194316	0.025852834	-0.116637038	-0.228824596	-1.966232492	-0.112187557	-1.849595454	-1.737407896	9.961963646	0.909465566	0.823634321	0.077738715	0.912903065	0.094221182	0.113072377	-6.990941718	-6.154559661	-5.265529943	-7.142734545	-4.740916411	-5.632405949	1.730701782	0.223890897

CNDP1	0.005680278	0.025264176	-0.033744568	0.019583898	-0.039424846	-0.059008744	8	0.22917573	0.816496581	0.816496581	0.816496581	0.816496581	0.816496581	0.816496581	22	144	0	P_231851	P_231851	P_231851	chr18	74571188	74571248	+	CNDP1	carnosine dipeptidase 1 (metallopeptidase M20 family) [Source:HGNC Symbol;Acc:HGNC:20675]	ENSG00000150656	protein_coding	3'UTR;exon	multiple Ref	1	3714	18:74571188:74571248	ENSG00000150656	CNDP1	3.842645623	0.044384324	0.033021775	0.146870972	-0.196170949	0.113849197	-0.229192724	-0.343041921	9.961963646	0.974309479	0.886163633	0.848420289	0.911619121	0.823355991	0.738695734	-6.997765307	-6.170728569	-7.026145408	-7.142529412	-6.268821468	-7.00747016	0.040718877	0.98837664

SMO	-0.04400573	-0.076693698	-0.044026636	-0.032687968	-2.09E-05	0.032667062	8	0.142440177	0.816496581	0.816496581	0.816496581	0.816496581	0.816496581	0.816496581	22	146	0	P_145885	P_145885	P_145885	chr7	129212391	129212451	+	SMO	"smoothened, frizzled class receptor [Source:HGNC Symbol;Acc:HGNC:11119]"	ENSG00000128602	protein_coding	exon	coding exon	1	3716	7:129212391:129212451	ENSG00000128602	SMO	8.307897884	0.018500021	-0.396249517	-0.690588267	-0.396437764	-0.29433875	-0.000188247	0.294150504	9.961963646	0.700275496	0.505595667	0.700141051	0.774540911	0.999853517	0.774680608	-6.913352848	-5.936749147	-6.961967293	-7.102441181	-6.296685238	-7.024315416	0.160708098	0.920334807

LAS1L	-0.175970708	-0.008887639	-0.033552007	0.167083069	0.142418701	-0.024664368	8	0.079296148	0.816496581	0.816496581	0.816496581	0.816496581	0.816496581	0.816496581	22	148	0	P_270483	P_270483	P_270483	chrX	65515062	65515122	-	LAS1L	LAS1-like (S. cerevisiae) [Source:HGNC Symbol;Acc:HGNC:25726]	ENSG00000001497	protein_coding	intron	intron sense	0	3718	X:65515062:65515122	ENSG00000001497	LAS1L	7.944074383	0.007256194	-2.530064392	-0.127784326	-0.482402665	2.402280066	2.047661727	-0.354618339	9.961963646	0.029948043	0.900862366	0.63994399	0.037258506	0.067879669	0.730267588	-4.307089627	-6.173486667	-6.921506515	-4.664061445	-4.447512365	-7.003111624	2.789981537	0.095645311

C3orf14	0.116976634	0.015487675	-0.047241728	-0.101488959	-0.164218363	-0.062729404	8	0.320228867	0.816496581	0.816496581	0.816496581	0.816496581	0.816496581	0.816496581	22	149	0	P_135248	P_135248	P_135248	chr3	62333305	62333365	+	C3orf14	chromosome 3 open reading frame 14 [Source:HGNC Symbol;Acc:HGNC:25024]	ENSG00000114405	protein_coding	exon	coding exon	1	3719	3:62333305:62333365	ENSG00000114405	C3orf14	3.50153332	0.084557137	0.492685314	0.065231404	-0.198973974	-0.42745391	-0.691659288	-0.264205378	9.961963646	0.632912734	0.949280228	0.84628539	0.678140927	0.504950939	0.797007001	-6.867500675	-6.179844736	-7.025542915	-7.050602105	-6.048352203	-7.033378018	0.169583082	0.914477189

FAT3	-0.065818991	-0.081150591	-0.011095908	-0.015331601	0.054723083	0.070054683	8	0.114293346	0.816496581	0.816496581	0.816496581	0.816496581	0.816496581	0.816496581	22	150	0	P_109795	P_109795	P_109795	chr11	92896245	92896305	+	FAT3	FAT atypical cadherin 3 [Source:HGNC Symbol;Acc:HGNC:23112]	ENSG00000165323	protein_coding	3'UTR	3'UTR	1	3720	11:92896245:92896305	ENSG00000165323	FAT3	3.767590978	0.01269696	-0.715396758	-0.882038287	-0.120603137	-0.166641529	0.594793621	0.76143515	9.961963646	0.490790797	0.398538932	0.90640316	0.870985915	0.565245652	0.46404101	-6.726073201	-5.787815193	-7.039183202	-7.134448163	-6.111887402	-6.763577795	0.377610253	0.771247879

FRZB	0.037083075	0.074924438	0.282454199	0.037841364	0.245371124	0.207529761	8	0.161847783	0.816496581	0.816496581	0.816496581	0.816496581	0.816496581	0.816496581	22	151	0	P_238167	P_238167	P_238167	chr2	182842477	182842537	-	FRZB	frizzled-related protein [Source:HGNC Symbol;Acc:HGNC:3959]	ENSG00000162998	protein_coding	exon	coding exon	1	3721	2:182842477:182842537	ENSG00000162998	FRZB	3.813423624	0.02324246	0.297906734	0.601905176	2.269094679	0.303998442	1.971187946	1.667189503	9.961963646	0.771894722	0.560685522	0.046737822	0.767383919	0.077102082	0.126558189	-6.950147196	-5.99463112	-4.784535896	-7.099308749	-4.562277958	-5.733363963	2.059557789	0.169659328

KRTAP4-11	0.041468285	0.186350884	0.519973106	0.144882599	0.47850482	0.333622222	8	0.368557929	0.816496581	0.816496581	0.816496581	0.816496581	0.816496581	0.816496581	22	152	0	P_314808	P_314808	P_314808	chr17	41117348	41117408	-	KRTAP4-11	keratin associated protein 4-11 [Source:HGNC Symbol;Acc:HGNC:18911]	ENSG00000212721	protein_coding	3'UTR	3'UTR	1	3722	17:41117348:41117408	ENSG00000212721	KRTAP4-11	3.047130681	0.111289553	0.15224201	0.684147726	1.908970917	0.531905716	1.756728907	1.224823191	9.961963646	0.882035264	0.509483265	0.085461697	0.606448886	0.109597757	0.248814321	-6.985727276	-5.941196568	-5.353294335	-6.997060934	-4.87375828	-6.308353946	1.500769616	0.273795453

RNF25	-0.325461947	0.253430543	0.121252864	0.57889249	0.446714811	-0.132177679	8	0.193663452	0.816496581	0.816496581	0.816496581	0.816496581	0.816496581	0.816496581	22	153	0	P_129891	P_129891	P_129891	chr2	218663873	218663933	-	RNF25	ring finger protein 25 [Source:HGNC Symbol;Acc:HGNC:14662]	ENSG00000163481	protein_coding	3'UTR	3'UTR	1	3723	2:218663873:218663933	ENSG00000163481	RNF25	8.090540347	0.032325672	-2.217032543	1.726357767	0.825969203	3.943390311	3.043001747	-0.900388564	9.961963646	0.051048454	0.115104357	0.428159414	0.002780088	0.012450952	0.389161084	-4.822250388	-4.815756883	-6.686844246	-2.046826531	-2.861116112	-6.645710644	5.699775984	0.015495902

NEURL1B	-0.036795731	-0.088862143	0.248784842	-0.052066411	0.285580573	0.337646984	8	0.159354921	0.816496581	0.816496581	0.816496581	0.816496581	0.816496581	0.816496581	22	154	0	A_32_P198731	A_32_P198731	A_32_P198731	chr5	172691312	172691372	+	NEURL1B	neuralized E3 ubiquitin protein ligase 1B [Source:HGNC Symbol;Acc:HGNC:35422]	ENSG00000214357	protein_coding	3'UTR	3'UTR	1	3724	5:172691312:172691372	ENSG00000214357	NEURL1B	5.79569808	0.022599443	-0.29977416	-0.723958278	2.026845633	-0.424184118	2.326619793	2.750803911	9.961963646	0.770510968	0.485744631	0.070280991	0.680445779	0.042385423	0.020517602	-6.949543591	-5.913104933	-5.171410585	-7.052097566	-4.016266236	-4.003544829	2.980349557	0.083130431

MRPL50	-0.044830243	0.103992641	0.037416166	0.148822883	0.082246408	-0.066576475	8	0.067072045	0.816496581	0.816496581	0.816496581	0.816496581	0.816496581	0.816496581	22	155	0	P_149265	P_149265	P_149265	chr9	101390115	101390175	-	MRPL50	mitochondrial ribosomal protein L50 [Source:HGNC Symbol;Acc:HGNC:16654]	ENSG00000136897	protein_coding	3'UTR	3'UTR	1	3725	9:101390115:101390175	ENSG00000136897	MRPL50	10.42701594	0.005819353	-0.7197462	1.669594087	0.600713752	2.389340287	1.320459952	-1.068880336	9.961963646	0.488223169	0.126073381	0.561448058	0.038090111	0.216213402	0.310339179	-6.722835729	-4.893622345	-6.853564065	-4.68549895	-5.444473682	-6.480729203	2.04358378	0.171911708

ACSL6	0.149530407	0.056352228	0.172341788	-0.093178179	0.022811381	0.11598956	8	0.148599144	0.816496581	0.816496581	0.816496581	0.816496581	0.816496581	0.816496581	22	157	0	P_254717	P_254717	P_254717	chr5	131966470	131966530	-	ACSL6	acyl-CoA synthetase long-chain family member 6 [Source:HGNC Symbol;Acc:HGNC:16496]	ENSG00000164398	protein_coding	exon	coding exon	1	3727	5:131966470:131966530	ENSG00000164398	ACSL6	4.923235069	0.019939498	1.296934487	0.488764454	1.494786331	-0.808170034	0.197851843	1.006021877	9.961963646	0.22388652	0.63558934	0.165956003	0.437865753	0.847139895	0.338215224	-6.150795424	-6.057699443	-5.947085623	-6.803722151	-6.275906526	-6.544979309	0.97661335	0.442032508

RCAN1	0.03342935	0.120688209	0.092006435	0.087258858	0.058577085	-0.028681773	8	0.080689898	0.816496581	0.816496581	0.816496581	0.816496581	0.816496581	0.816496581	22	158	0	P_132151	P_132151	P_132151	chr21	34517873	34517933	-	RCAN1	regulator of calcineurin 1 [Source:HGNC Symbol;Acc:HGNC:3040]	ENSG00000159200	protein_coding	3'UTR;exon	multiple Ref	1	3728	21:34517873:34517933	ENSG00000159200	RCAN1	6.388857462	0.007435259	0.474816176	1.714203633	1.306820006	1.239387456	0.832003829	-0.407383627	9.961963646	0.645155755	0.117377589	0.220635206	0.243605625	0.424901659	0.692342395	-6.87671646	-4.832544064	-6.186855917	-6.368728998	-5.941132261	-6.981465437	1.210999347	0.355697903

AP3B2	0.004210873	0.101121543	-0.015554637	0.09691067	-0.01976551	-0.11667618	8	0.080238974	0.816496581	0.816496581	0.816496581	0.816496581	0.816496581	0.816496581	22	159	0	P_224762	P_224762	P_224762	chr15	82664878	82664938	-	AP3B2	"adaptor-related protein complex 3, beta 2 subunit [Source:HGNC Symbol;Acc:HGNC:567]"	ENSG00000103723	protein_coding	exon	coding exon	1	3729	15:82664878:82664938	ENSG00000103723	AP3B2	4.300884291	0.007376984	0.060045201	1.441948958	-0.221802322	1.381903757	-0.281847523	-1.66375128	9.961963646	0.953307129	0.180006518	0.828948506	0.197200975	0.783828692	0.127254309	-6.996393224	-5.19077619	-7.020320628	-6.194721777	-6.254606378	-5.738248255	1.148197139	0.376861635

FBXW11	-0.03191366	-0.001503701	-0.014114088	0.030409958	0.017799572	-0.012610387	8	0.036713293	0.816496581	0.816496581	0.816496581	0.816496581	0.816496581	0.816496581	23	12	0	P_141139	P_141139	P_141139	chr5	171861561	171861621	-	FBXW11	F-box and WD repeat domain containing 11 [Source:HGNC Symbol;Acc:HGNC:13607]	ENSG00000072803	protein_coding	3'UTR	3'UTR	1	3752	5:171861561:171861621	ENSG00000072803	FBXW11	8.080146679	0.003289094	-0.681528634	-0.032112131	-0.301411848	0.649416503	0.380116786	-0.269299718	9.961963646	0.51106935	0.9750169	0.769298144	0.530756669	0.711837338	0.793194547	-6.750674803	-6.18154384	-6.997733508	-6.923753739	-6.220403968	-7.03190385	0.199185746	0.89456445

CABCOCO1	0.002056915	-0.042933845	0.253652216	-0.044990759	0.251595302	0.296586061	8	0.269315347	0.816496581	0.816496581	0.816496581	0.816496581	0.816496581	0.816496581	23	15	0	P_106046	P_106046	P_106046	chr10	61760943	61761003	+	C10orf107	chromosome 10 open reading frame 107 [Source:HGNC Symbol;Acc:HGNC:28678]	ENSG00000183346	protein_coding	exon	coding exon	1	3755	10:61760943:61761003	ENSG00000183346	CABCOCO1	4.289941513	0.060452836	0.010245981	-0.213863698	1.263502051	-0.224109679	1.25325607	1.477365749	9.961963646	0.992027316	0.834967059	0.235176408	0.827201374	0.238728332	0.170479829	-6.998303031	-6.158033305	-6.238947282	-7.122216034	-5.522917402	-5.994029181	0.907627596	0.471554795

NRF1	-0.066039905	-0.029432235	-0.034059716	0.03660767	0.031980189	-0.004627481	8	0.102909167	0.816496581	0.816496581	0.816496581	0.816496581	0.816496581	0.816496581	23	16	0	P_145895	P_145895	P_145895	chr7	129756578	129756638	-	NRF1	nuclear respiratory factor 1 [Source:HGNC Symbol;Acc:HGNC:7996]	ENSG00000106459	protein_coding	3'ext;3'UTR	multiple non-Ref	0	3756	7:129756578:129756638	ENSG00000106459	NRF1	5.927609796	0.01071127	-0.781504701	-0.348295929	-0.403056726	0.433208772	0.378447976	-0.054760796	9.961963646	0.452678631	0.734865999	0.695420761	0.67409283	0.713037773	0.957411699	-6.674976422	-6.118538599	-6.95912334	-7.047943212	-6.221067381	-7.069740549	0.204734305	0.890781073

ZNF207	-0.07666138	-0.092465205	-0.072484036	-0.015803824	0.004177344	0.019981168	8	0.079893898	0.816496581	0.816496581	0.816496581	0.816496581	0.816496581	0.816496581	23	17	0	P_121070	P_121070	P_121070	chr17	32366697	32366757	+	ZNF207	zinc finger protein 207 [Source:HGNC Symbol;Acc:HGNC:12998]	ENSG00000010244	protein_coding	exon	coding exon	1	3757	17:32366697:32366757	ENSG00000010244	ZNF207	7.995461321	0.007332609	-1.096460516	-1.322497008	-1.036713447	-0.226036492	0.059747069	0.285783561	9.961963646	0.298674684	0.215559393	0.324378824	0.825743147	0.953538661	0.780898147	-6.379279087	-5.335645936	-6.489948703	-7.121744125	-6.294786926	-7.026943883	0.693722204	0.576655872

ST7	0.387888491	-0.307713246	0.209210958	-0.695601736	-0.178677533	0.516924203	8	0.085896672	0.816496581	0.816496581	0.816496581	0.816496581	0.816496581	0.816496581	23	19	0	P_145724	P_145724	P_145724	chr7	116953479	116953539	+	ST7	suppression of tumorigenicity 7 [Source:HGNC Symbol;Acc:HGNC:11351]	ENSG00000004866	protein_coding	5'UTR	5'UTR	1	3759	7:116953479:116953539	ENSG00000004866	ST7	8.018177431	0.008131812	5.268158952	-4.179248233	2.841426353	-9.447407185	-2.426732599	7.020674586	9.961963646	0.000368394	0.001905676	0.017568072	2.75E-06	0.035735367	3.70E-05	0.174995243	-1.01898085	-3.826582823	5.159573002	-3.85774724	2.512998006	32.95428725	1.75E-05

HMGN4	-0.098273361	-0.02016137	-0.095881149	0.078111991	0.002392212	-0.075719779	8	0.179169811	0.816496581	0.816496581	0.816496581	0.816496581	0.816496581	0.816496581	23	22	0	A_24_P397515	A_24_P397515	A_24_P397515	chr6	26545994	26546054	+	HMGN4	high mobility group nucleosomal binding domain 4 [Source:HGNC Symbol;Acc:HGNC:4989]	ENSG00000182952	protein_coding	exon	coding exon	1	3762	6:26545994:26546054	ENSG00000182952	HMGN4	8.463071475	0.027986197	-0.719464712	-0.147602508	-0.701951198	0.571862204	0.017513514	-0.55434869	9.961963646	0.488389087	0.885601135	0.498781062	0.580089143	0.986372751	0.591566541	-6.723045787	-6.170615264	-6.784523655	-6.973663364	-6.296522118	-6.906053346	0.277797838	0.840197412

ATPAF1	0.108152121	0.099909558	0.054056925	-0.008242563	-0.054095197	-0.045852634	8	0.071216847	0.816496581	0.816496581	0.816496581	0.816496581	0.816496581	0.816496581	23	23	0	A_24_P229199	A_24_P229199	A_24_P229199	chr1	46635840	46635900	-	ATPAF1	ATP synthase mitochondrial F1 complex assembly factor 1 [Source:HGNC Symbol;Acc:HGNC:18803]	ENSG00000123472	protein_coding	3'UTR;exon	multiple Ref	1	3763	1:46635840:46635900	ENSG00000123472	ATPAF1	9.17936514	0.006279647	1.671525624	1.544134174	0.835467057	-0.127391449	-0.836058566	-0.708667117	9.961963646	0.125685158	0.153703716	0.423039637	0.901165357	0.422722162	0.494780037	-5.655882079	-5.060572909	-6.678789033	-7.14074546	-5.937777413	-6.803758823	1.182297509	0.365202716

FMO3	-0.006058745	0.034129893	-0.13008033	0.040188638	-0.124021586	-0.164210224	8	0.263122251	0.816496581	0.816496581	0.816496581	0.816496581	0.816496581	0.816496581	23	24	0	P_204861	P_204861	P_204861	chr1	171108098	171108158	+	FMO3	flavin containing monooxygenase 3 [Source:HGNC Symbol;Acc:HGNC:3771]	ENSG00000007933	protein_coding	exon	coding exon	1	3764	1:171108098:171108158	ENSG00000007933	FMO3	3.646523867	0.057804814	-0.030863579	0.173859552	-0.66263635	0.204723131	-0.631772771	-0.836495902	9.961963646	0.975987921	0.865458007	0.5225982	0.841910698	0.541760879	0.422487539	-6.997840538	-6.166177666	-6.812503016	-7.126741403	-6.088670043	-6.702034427	0.268506738	0.846669073

SOX6	0.077893462	0.009564231	-0.027367393	-0.068329231	-0.105260855	-0.036931624	8	0.145321444	0.816496581	0.816496581	0.816496581	0.816496581	0.816496581	0.816496581	23	28	0	P_108041	P_108041	P_108041	chr11	16226533	16226593	-	SOX6	SRY (sex determining region Y)-box 6 [Source:HGNC Symbol;Acc:HGNC:16421]	ENSG00000110693	protein_coding	intron	intron sense	0	3768	11:16226533:16226593	ENSG00000110693	SOX6	4.329424936	0.019165848	0.68910058	0.084611941	-0.242111286	-0.604488638	-0.931211866	-0.326723228	9.961963646	0.506492049	0.934246148	0.813605236	0.559034046	0.373760817	0.750638176	-6.745268499	-6.178314719	-7.015203291	-6.95338373	-5.855067445	-7.013374111	0.313324464	0.81547914

COA1	-0.21503484	-0.037170086	-0.047264515	0.177864754	0.167770324	-0.010094429	8	0.100536759	0.816496581	0.816496581	0.816496581	0.816496581	0.816496581	0.816496581	23	30	0	P_144689	P_144689	P_144689	chr7	43639582	43639642	-	COA1	cytochrome c oxidase assembly factor 1 homolog (S. cerevisiae) [Source:HGNC Symbol;Acc:HGNC:21868]	ENSG00000106603	protein_coding	exon	coding exon	1	3770	7:43639582:43639642	ENSG00000106603	COA1	8.849930294	0.01032367	-2.59201436	-0.448045524	-0.569723041	2.143968836	2.022291319	-0.121677517	9.961963646	0.026933765	0.663706801	0.581484547	0.057750044	0.070816991	0.905573857	-4.203240475	-6.077356608	-6.872709257	-5.085358792	-4.485777068	-7.06329632	2.65748959	0.105678117

ONECUT2	-0.100982122	0.10367163	0.031937318	0.204653752	0.13291944	-0.071734312	8	0.12532879	0.816496581	0.816496581	0.816496581	0.816496581	0.816496581	0.816496581	23	31	0	A_32_P124708	A_32_P124708	A_32_P124708	chr18	57491159	57491219	+	ONECUT2	one cut homeobox 2 [Source:HGNC Symbol;Acc:HGNC:8139]	ENSG00000119547	protein_coding	3'UTR	3'UTR	1	3771	18:57491159:57491219	ENSG00000119547	ONECUT2	7.681470849	0.014820507	-1.015917858	1.042975306	0.321301342	2.058893165	1.337219201	-0.721673964	9.961963646	0.333706699	0.321608821	0.754621587	0.06661655	0.210881565	0.487087826	-6.462684772	-5.639051275	-6.991035423	-5.220467968	-5.42446817	-6.794095148	1.461855117	0.283444735

ZNF7	-0.011136733	-0.023578769	-0.047939569	-0.012442036	-0.036802837	-0.024360801	8	0.104204123	0.816496581	0.816496581	0.816496581	0.816496581	0.816496581	0.816496581	23	32	0	P_313988	P_313988	P_313988	chr8	144844362	144844422	+	ZNF7;COMMD5	zinc finger protein 7 [Source:HGNC Symbol;Acc:HGNC:13139];COMM domain containing 5 [Source:HGNC Symbol;Acc:HGNC:17902]	ENSG00000147789	protein_coding	3'ext;intron	multiple non-Ref	0	3772	8:144844362:144844422	ENSG00000147789	ZNF7	6.181665323	0.010926651	-0.130484789	-0.276263312	-0.561689394	-0.145778523	-0.431204605	-0.285426082	9.961963646	0.898780196	0.787992454	0.586741159	0.887003769	0.675501375	0.781164157	-6.989077205	-6.14201269	-6.877517404	-7.138003849	-6.198729572	-7.027054524	0.116249918	0.948524639

TRAPPC8	-0.058287809	-0.016133888	-0.055729943	0.042153921	0.002557866	-0.039596055	8	0.048371684	0.816496581	0.816496581	0.816496581	0.816496581	0.816496581	0.816496581	23	33	0	P_231114	P_231114	P_231114	chr18	31867407	31867467	-	TRAPPC8	trafficking protein particle complex 8 [Source:HGNC Symbol;Acc:HGNC:29169]	ENSG00000153339	protein_coding	exon	coding exon	1	3773	18:31867407:31867467	ENSG00000153339	TRAPPC8	7.579850443	0.004085687	-1.116839641	-0.30913781	-1.067828951	0.807701831	0.04901069	-0.758691141	9.961963646	0.290275151	0.763585378	0.310790637	0.438123039	0.961879474	0.465608705	-6.357382361	-6.131957797	-6.457791678	-6.804110289	-6.295407799	-6.765730773	0.618925364	0.61853954

PPP1R15B	-0.23744512	0.067759716	-0.045880655	0.305204836	0.191564464	-0.113640371	8	0.093154274	0.816496581	0.816496581	0.816496581	0.816496581	0.816496581	0.816496581	23	34	0	P_205851	P_205851	P_205851	chr1	204405426	204405486	+	PPP1R15B;RP11-739N20.2	"protein phosphatase 1, regulatory subunit 15B [Source:HGNC Symbol;Acc:HGNC:14951];"	ENSG00000158615	protein_coding;antisense	3'UTR;intron	multiple non-Ref	0	3774	1:204405426:204405486	ENSG00000158615	PPP1R15B	9.825642194	0.009175365	-3.035968047	0.866374229	-0.586629046	3.902342276	2.449339001	-1.453003275	9.961963646	0.012601042	0.406667584	0.570506331	0.002971116	0.034381754	0.176985023	-3.449378524	-5.80118131	-6.862382446	-2.115394859	-3.821741922	-6.026066723	5.608432536	0.016264505

GALNT8	0.028179189	0.146384129	-0.073774107	0.11820494	-0.101953296	-0.220158236	8	0.315069153	0.816496581	0.816496581	0.816496581	0.816496581	0.816496581	0.816496581	23	35	0	P_110695	P_110695	P_110695	chr12	4772452	4772512	+	GALNT8	polypeptide N-acetylgalactosaminyltransferase 8 [Source:HGNC Symbol;Acc:HGNC:4130]	ENSG00000130035	protein_coding	exon	coding exon	1	3775	12:4772452:4772512	ENSG00000130035	GALNT8	3.482865452	0.081924759	0.120577572	0.626371566	-0.315676319	0.505793994	-0.436253891	-0.942047885	9.961963646	0.906422895	0.545155736	0.758762234	0.624004448	0.671955222	0.368451604	-6.990432356	-5.9793911	-6.992972272	-7.011486416	-6.196444499	-6.607095603	0.306688511	0.82008832

NABP1	-0.145949902	-0.082893478	-0.284896542	0.063056424	-0.13894664	-0.202003064	8	0.059488221	0.816496581	0.816496581	0.816496581	0.816496581	0.816496581	0.816496581	23	36	0	A_23_P329198	A_23_P329198	A_23_P329198	chr2	191686828	191686888	+	NABP1	nucleic acid binding protein 1 [Source:HGNC Symbol;Acc:HGNC:26232]	ENSG00000173559	protein_coding	3'UTR	3'UTR	1	3776	2:191686828:191686888	ENSG00000173559	NABP1	8.003620988	0.005048572	-2.515736486	-1.428833765	-4.91075784	1.086902721	-2.395021354	-3.481924075	9.961963646	0.030691484	0.183648631	0.00062012	0.302678158	0.037722786	0.005935748	-4.331035783	-5.207081945	-0.403796941	-6.539306583	-3.908129723	-2.754575513	8.587886438	0.004089096

PCSK2	0.027944983	0.263982597	0.048154402	0.236037613	0.020209419	-0.215828195	8	0.152400042	0.816496581	0.816496581	0.816496581	0.816496581	0.816496581	0.816496581	23	38	0	P_240277	P_240277	P_240277	chr20	17260240	17260300	+	PCSK2	proprotein convertase subtilisin/kexin type 2 [Source:HGNC Symbol;Acc:HGNC:8744]	ENSG00000125851	protein_coding	exon	coding exon	1	3778	20:17260240:17260300	ENSG00000125851	PCSK2	3.383021669	0.020858246	0.236979399	2.23862853	0.408359565	2.00164913	0.171380166	-1.830268965	9.961963646	0.817474819	0.049215593	0.691648874	0.073295199	0.867355998	0.097247205	-6.96780142	-4.06273624	-6.95681201	-5.310152116	-6.281086878	-5.495557026	2.103371558	0.163656005

NDRG3	-0.007051815	0.099250831	0.188860903	0.106302645	0.195912718	0.089610072	8	0.062552476	0.816496581	0.816496581	0.816496581	0.816496581	0.816496581	0.816496581	23	40	0	P_240707	P_240707	P_240707	chr20	36666288	36666348	-	NDRG3	NDRG family member 3 [Source:HGNC Symbol;Acc:HGNC:14462]	ENSG00000101079	protein_coding	exon	coding exon	1	3780	20:36666288:36666348	ENSG00000101079	NDRG3	8.981959352	0.005348886	-0.118090474	1.662065463	3.162685714	1.780155938	3.280776189	1.500620251	9.961963646	0.908343118	0.127596883	0.010158948	0.105515537	0.00831935	0.164464508	-6.990755808	-4.903845269	-3.277497354	-5.646315432	-2.474940559	-5.963132727	4.827216098	0.025099135

COP1	0.030891963	-0.044723655	0.000630602	-0.075615617	-0.03026136	0.045354257	8	0.073717235	0.816496581	0.816496581	0.816496581	0.816496581	0.816496581	0.816496581	23	42	0	P_205041	P_205041	P_205041	chr1	176046180	176046240	-	RFWD2	"ring finger and WD repeat domain 2, E3 ubiquitin protein ligase [Source:HGNC Symbol;Acc:HGNC:17440]"	ENSG00000143207	protein_coding	3'UTR;exon	multiple Ref	1	3782	1:176046180:176046240	ENSG00000143207	COP1	6.719240316	0.006570668	0.466752209	-0.675737727	0.009527886	-1.142489936	-0.457224323	0.685265613	9.961963646	0.650717723	0.51458681	0.992586067	0.279965648	0.657318491	0.508807197	-6.88076886	-5.946947071	-7.047071026	-6.479100275	-6.186681084	-6.820744692	0.443082696	0.727418418

LINC01215	0.54216886	0.303083689	0.401666048	-0.239085171	-0.140502812	0.098582359	8	0.340350919	0.816496581	0.816496581	0.816496581	0.816496581	0.816496581	0.816496581	23	43	0	A_32_P203728	A_32_P203728	A_32_P203728	chr3	108138548	108138608	+	LINC01215	long intergenic non-protein coding RNA 1215 [Source:HGNC Symbol;Acc:HGNC:49651]	ENSG00000271856	lincRNA	exon	non-coding exon	1	3783	3:108138548:108138608	ENSG00000271856	LINC01215	3.192562708	0.095231515	2.151740293	1.202867656	1.59411778	-0.948872636	-0.557622513	0.391250123	9.961963646	0.05699891	0.256836019	0.14211034	0.365135713	0.589411901	0.70385	-4.927081951	-5.471986659	-5.812155468	-6.678358026	-6.133911589	-6.988392706	1.663744531	0.237256624

CWC22	0.132123254	0.018208958	0.022411341	-0.113914296	-0.109711913	0.004202384	8	0.049448375	0.816496581	0.816496581	0.816496581	0.816496581	0.816496581	0.816496581	23	44	0	P_238098	P_238098	P_238098	chr2	179952470	179952530	-	CWC22	CWC22 spliceosome-associated protein [Source:HGNC Symbol;Acc:HGNC:29322]	ENSG00000163510	protein_coding	exon	coding exon	1	3784	2:179952470:179952530	ENSG00000163510	CWC22	7.579340127	0.004170266	2.505780379	0.345341548	0.425041753	-2.160438831	-2.080738626	0.079700205	9.961963646	0.031218805	0.737018567	0.679840903	0.056169286	0.064223436	0.938054014	-4.347658355	-6.119605489	-6.949350551	-5.058968702	-4.397354239	-7.067909452	2.597029646	0.110666186

B3GAT2	-0.303220056	-0.035114644	-0.29644405	0.268105413	0.006776007	-0.261329406	8	0.301141588	0.816496581	0.816496581	0.816496581	0.816496581	0.816496581	0.816496581	23	48	0	P_257712	P_257712	P_257712	chr6	70861662	70861722	-	B3GAT2	"beta-1,3-glucuronyltransferase 2 [Source:HGNC Symbol;Acc:HGNC:922]"	ENSG00000112309	protein_coding	exon	coding exon	1	3788	6:70861662:70861722	ENSG00000112309	B3GAT2	4.137916463	0.075032692	-1.355745867	-0.157003246	-1.325449246	1.198742621	0.030296621	-1.168446001	9.961963646	0.205115761	0.878378704	0.214614482	0.258366077	0.976428869	0.269828203	-6.078548962	-6.169109355	-6.164072501	-6.415843156	-6.296197069	-6.372731623	1.070446732	0.405001064

YAF2	-0.158858355	-0.113134292	-0.032953608	0.045724063	0.125904747	0.080180684	8	0.083594873	0.816496581	0.816496581	0.816496581	0.816496581	0.816496581	0.816496581	23	49	0	P_215127	P_215127	P_215127	chr12	42223178	42223238	-	YAF2	YY1 associated factor 2 [Source:HGNC Symbol;Acc:HGNC:17363]	ENSG00000015153	protein_coding	intron	intron sense	0	3789	12:42223178:42223238	ENSG00000015153	YAF2	6.560263787	0.007818512	-2.200359541	-1.567031952	-0.456443006	0.633327589	1.743916535	1.110588946	9.961963646	0.052508409	0.14829309	0.65786116	0.540785884	0.111890839	0.292831734	-4.849121962	-5.030661649	-6.934525988	-6.934589621	-4.891818473	-6.4363969	2.030211332	0.173824099

IKZF2	0.041797643	0.046547323	0.066279321	0.004749681	0.024481679	0.019731998	8	0.101142045	0.816496581	0.816496581	0.816496581	0.816496581	0.816496581	0.816496581	23	50	0	P_129773	P_129773	P_129773	chr2	213007173	213007233	-	IKZF2	IKAROS family zinc finger 2 (Helios) [Source:HGNC Symbol;Acc:HGNC:13177]	ENSG00000030419	protein_coding	3'UTR;exon	multiple Ref	1	3790	2:213007173:213007233	ENSG00000030419	IKZF2	4.663941465	0.010421701	0.50145054	0.558432939	0.795159712	0.056982399	0.293709172	0.236726773	9.961963646	0.626949196	0.588879173	0.445052542	0.955685941	0.775008152	0.81766544	-6.862861639	-6.020316363	-6.712419694	-7.147837682	-6.251006107	-7.040850309	0.223521766	0.877883521

HTR1E	-0.130209365	-0.638038814	-0.305509995	-0.50782945	-0.175300631	0.332528819	8	0.564252721	0.816496581	0.816496581	0.816496581	0.816496581	0.816496581	0.816496581	23	52	0	A_24_P380349	A_24_P380349	A_24_P380349	chr6	87016353	87016413	+	HTR1E	"5-hydroxytryptamine (serotonin) receptor 1E, G protein-coupled [Source:HGNC Symbol;Acc:HGNC:5291]"	ENSG00000168830	protein_coding	exon	coding exon	1	3792	6:87016353:87016413	ENSG00000168830	HTR1E	3.021965285	0.257884094	-0.314033204	-1.538793881	-0.736815537	-1.224760676	-0.422782332	0.801978344	9.961963646	0.759973269	0.154990272	0.478227818	0.248836871	0.681434947	0.441276325	-6.94481265	-5.067511406	-6.758493404	-6.385817332	-6.202484122	-6.730966547	0.888558913	0.480075562

MAMDC2	0.226077588	-0.05366869	0.035853657	-0.279746278	-0.190223931	0.089522348	8	0.186864422	0.816496581	0.816496581	0.816496581	0.816496581	0.816496581	0.816496581	23	53	0	P_267461	P_267461	P_267461	chr9	70140207	70140267	+	MAMDC2	MAM domain containing 2 [Source:HGNC Symbol;Acc:HGNC:23673]	ENSG00000165072	protein_coding	exon	coding exon	1	3793	9:70140207:70140267	ENSG00000165072	MAMDC2	3.927001323	0.030247993	1.592043246	-0.377936073	0.252482228	-1.969979319	-1.339561018	0.630418301	9.961963646	0.142575681	0.713406167	0.805801414	0.077256902	0.210145347	0.542611066	-5.767323916	-6.107345582	-7.012419299	-5.359312335	-5.421658978	-6.858511465	1.468975687	0.281650579

MTFR1L	0.028801306	0.233551732	0.083510299	0.204750426	0.054708993	-0.150041433	8	0.103230612	0.816496581	0.816496581	0.816496581	0.816496581	0.816496581	0.816496581	23	54	0	P_100868	P_100868	P_100868	chr1	25832466	25832526	+	MTFR1L	mitochondrial fission regulator 1-like [Source:HGNC Symbol;Acc:HGNC:28836]	ENSG00000117640	protein_coding	3'UTR;exon	multiple Ref	1	3794	1:25832466:25832526	ENSG00000117640	MTFR1L	9.325024271	0.010764482	0.339986164	2.7569707	0.985800646	2.416984535	0.645814482	-1.771170054	9.961963646	0.740926589	0.020301961	0.347568038	0.036335098	0.53299252	0.107064624	-6.93564954	-3.246538402	-6.540905982	-4.639664005	-6.07951624	-5.583074255	3.014035815	0.081122959

CD3G	-0.110088681	-0.11397745	-0.080884465	-0.00388877	0.029204215	0.033092985	8	0.177902575	0.816496581	0.816496581	0.816496581	0.816496581	0.816496581	0.816496581	23	57	0	P_213503	P_213503	P_213503	chr11	118349910	118349970	+	CD3G	"CD3g molecule, gamma (CD3-TCR complex) [Source:HGNC Symbol;Acc:HGNC:1675]"	ENSG00000160654	protein_coding	3'UTR;exon	multiple Ref	1	3797	11:118349910:118349970	ENSG00000160654	CD3G	4.535878503	0.027622819	-0.811249234	-0.83990578	-0.596041847	-0.028656546	0.215207387	0.243863933	9.961963646	0.436176178	0.420661225	0.564443763	0.977704471	0.833947562	0.812284892	-6.650684336	-5.823300717	-6.856510712	-7.149163997	-6.272110328	-7.038987337	0.30423966	0.821790496

ELF1	0.092352085	0.023255052	0.108630783	-0.069097033	0.016278698	0.085375732	8	0.059713832	0.816496581	0.816496581	0.816496581	0.816496581	0.816496581	0.816496581	23	58	0	P_218892	P_218892	P_218892	chr13	40951360	40951420	-	ELF1	E74-like factor 1 (ets domain transcription factor) [Source:HGNC Symbol;Acc:HGNC:3316]	ENSG00000120690	protein_coding	exon	coding exon	1	3798	13:40951360:40951420	ENSG00000120690	ELF1	8.841968404	0.005070169	1.588477673	0.399992381	1.868475133	-1.188485292	0.27999746	1.468482752	9.961963646	0.143378635	0.697604406	0.091349444	0.262202375	0.785207371	0.172827414	-5.772249143	-6.098441334	-5.414642203	-6.427548427	-6.255154757	-6.005750247	1.636965334	0.242857442

NFIX	0.023090175	-0.213092544	-0.034013791	-0.23618272	-0.057103967	0.179078753	8	0.066867095	0.816496581	0.816496581	0.816496581	0.816496581	0.816496581	0.816496581	23	59	0	P_124814	P_124814	P_124814	chr19	13098592	13098652	+	NFIX	nuclear factor I/X (CCAAT-binding transcription factor) [Source:HGNC Symbol;Acc:HGNC:7788]	ENSG00000008441	protein_coding	3'UTR	3'UTR	1	3799	19:13098592:13098652	ENSG00000008441	NFIX	8.76007844	0.005797308	0.371415198	-3.427683343	-0.54712616	-3.799098541	-0.918541358	2.880557183	9.961963646	0.718105705	0.00649908	0.596334743	0.003514872	0.380037987	0.016430307	-6.923603154	-2.176155057	-6.886070014	-2.288570684	-5.866514933	-3.782529584	5.960131169	0.013532057

ILF3	-0.212226969	-0.062038107	-0.117723714	0.150188862	0.094503255	-0.055685608	8	0.115649019	0.816496581	0.816496581	0.816496581	0.816496581	0.816496581	0.816496581	23	60	0	P_232319	P_232319	P_232319	chr19	10677243	10677303	+	ILF3	"interleukin enhancer binding factor 3, 90kDa [Source:HGNC Symbol;Acc:HGNC:6038]"	ENSG00000129351	protein_coding	3'UTR;exon	multiple Ref	1	3800	19:10677243:10677303	ENSG00000129351	ILF3	10.44127901	0.012947294	-2.284318715	-0.667751177	-1.267126819	1.616567538	1.017191896	-0.599375642	9.961963646	0.045545429	0.519461725	0.233930221	0.137160183	0.333129528	0.562305168	-4.713142933	-5.952348181	-6.234636595	-5.881462121	-5.773983847	-6.878598734	1.87947252	0.197187293

RAB36	0.17994331	0.192429978	0.269705853	0.012486668	0.089762543	0.077275875	8	0.139592841	0.816496581	0.816496581	0.816496581	0.816496581	0.816496581	0.816496581	23	62	0	A_32_P58755	A_32_P58755	A_32_P58755	chr22	23165481	23165541	+	RAB36	"RAB36, member RAS oncogene family [Source:HGNC Symbol;Acc:HGNC:9775]"	ENSG00000100228	protein_coding	3'ext	3'extended sense	0	3802	22:23165481:23165541	ENSG00000100228	RAB36	6.588344414	0.017855134	1.649300451	1.763749094	2.472034025	0.114448643	0.822733574	0.708284931	9.961963646	0.130217831	0.108359582	0.033073637	0.911155997	0.429913054	0.495007192	-5.687355835	-4.763733807	-4.450110722	-7.14245467	-5.948749285	-6.804040377	2.188935272	0.15263272

CCDC173	-0.007280674	0.151541801	0.049572547	0.158822475	0.056853221	-0.101969254	8	0.126822542	0.816496581	0.816496581	0.816496581	0.816496581	0.816496581	0.816496581	23	63	0	P_237447	P_237447	P_237447	chr2	169674575	169674635	-	CCDC173	coiled-coil domain containing 173 [Source:HGNC Symbol;Acc:HGNC:25064]	ENSG00000154479	protein_coding	exon	coding exon	1	3803	2:169674575:169674635	ENSG00000154479	CCDC173	4.32954929	0.015122979	-0.072510105	1.509243821	0.493705759	1.581753926	0.566215865	-1.015538061	9.961963646	0.94363113	0.162281175	0.632217028	0.144903702	0.583776245	0.333878901	-6.995491986	-5.105643695	-6.915623022	-5.929838949	-6.128937937	-6.535454271	1.063447241	0.407644519

GMNC	-0.090569048	0.253654256	-0.198853109	0.344223304	-0.108284061	-0.452507365	8	0.220246302	0.816496581	0.816496581	0.816496581	0.816496581	0.816496581	0.816496581	23	64	0	P_136869	P_136869	P_136869	chr3	190852797	190852857	-	GMNC	geminin coiled-coil domain containing [Source:HGNC Symbol;Acc:HGNC:40049]	ENSG00000205835	protein_coding	3'ext	3'extended sense	0	3804	3:190852797:190852857	ENSG00000205835	GMNC	4.567993809	0.041161601	-0.546738053	1.531234313	-1.200416299	2.077972366	-0.653678247	-2.731650612	9.961963646	0.596591538	0.156827624	0.257744398	0.064521886	0.528118438	0.021202129	-6.837659685	-5.077308809	-6.312522419	-5.190351142	-6.074309264	-4.036071441	2.715296631	0.101155102

PDE1A	0.182845231	0.082861466	0.231346291	-0.099983765	0.04850106	0.148484825	8	0.151422656	0.816496581	0.816496581	0.816496581	0.816496581	0.816496581	0.816496581	23	65	0	P_311172	P_311172	P_311172	chr2	182183573	182183633	+	PDE1A	"phosphodiesterase 1A, calmodulin-dependent [Source:HGNC Symbol;Acc:HGNC:8774]"	ENSG00000115252	protein_coding	intron	intron antisense	0	3805	2:182183573:182183633	ENSG00000115252	PDE1A	3.901296222	0.020619777	1.559506838	0.706734447	1.973177643	-0.852772391	0.413670805	1.266443196	9.961963646	0.150052437	0.495929394	0.076847845	0.413818287	0.687879863	0.234164834	-5.812023091	-5.92543423	-5.254807451	-6.76584985	-6.206465544	-6.259398593	1.554531303	0.261073862

NAALADL2	-0.069058588	-0.385074052	-0.248152499	-0.316015465	-0.179093911	0.136921554	8	0.084043781	0.816496581	0.816496581	0.816496581	0.816496581	0.816496581	0.816496581	23	67	0	P_247289	P_247289	P_247289	chr3	175676681	175676741	+	NAALADL2;RP11-744O11.2	N-acetylated alpha-linked acidic dipeptidase-like 2 [Source:HGNC Symbol;Acc:HGNC:23219];	ENSG00000177694	protein_coding;processed_pseudogene	exon;intron	multiple non-Ref	0	3807	3:175676681:175676741	ENSG00000177694	NAALADL2	5.353147747	0.007878945	-0.952860424	-5.31319619	-3.423972354	-4.360335766	-2.47111193	1.889223836	9.961963646	0.363208195	0.000345443	0.006539588	0.001433227	0.033125817	0.08828719	-6.524370697	0.569433679	-2.831251221	-1.360731596	-3.786998312	-5.406856323	11.59161205	0.00137392

GRPEL1	-0.209365104	0.199774062	0.02911618	0.409139166	0.238481284	-0.170657882	8	0.053245218	0.816496581	0.816496581	0.816496581	0.816496581	0.816496581	0.816496581	23	69	0	A_24_P174563	A_24_P174563	A_24_P174563	chr4	7062410	7062467	-	GRPEL1	"GrpE-like 1, mitochondrial (E. coli) [Source:HGNC Symbol;Acc:HGNC:19696]"	ENSG00000109519	protein_coding	exon	coding exon	1	3809	4:7062410:7062467	ENSG00000109519	GRPEL1	11.11287349	0.004483386	-3.829542073	3.654110259	0.532570292	7.483652332	4.362112365	-3.121539967	9.961963646	0.003344478	0.004460028	0.60600542	2.15E-05	0.001429258	0.010893808	-2.103194259	-1.820660793	-6.894406841	3.028439979	-0.77970561	-3.370288216	18.84643894	0.000197726

TP53AIP1	-0.246544927	-0.278058381	-0.24199502	-0.031513455	0.004549906	0.036063361	8	0.154430512	0.816496581	0.816496581	0.816496581	0.816496581	0.816496581	0.816496581	23	70	0	P_110504	P_110504	P_110504	chr11	128937380	128937440	-	TP53AIP1	tumor protein p53 regulated apoptosis inducing protein 1 [Source:HGNC Symbol;Acc:HGNC:29984]	ENSG00000120471	protein_coding	3'UTR;exon	multiple Ref	1	3810	11:128937380:128937440	ENSG00000120471	TP53AIP1	3.725113344	0.021358557	-2.066120956	-2.330213224	-2.027991367	-0.264092268	0.038129589	0.302221857	9.961963646	0.065815532	0.042127046	0.070146755	0.797091713	0.970337642	0.768698517	-5.062816687	-3.921095356	-5.16962003	-7.111605704	-6.295912024	-7.021709198	2.32900585	0.136406494

SLC26A5	-0.056078219	-0.058910453	-0.032420441	-0.002832234	0.023657778	0.026490012	8	0.098370467	0.816496581	0.816496581	0.816496581	0.816496581	0.816496581	0.816496581	23	71	0	P_262185	P_262185	P_262185	chr7	103421362	103421422	-	SLC26A5	"solute carrier family 26 (anion exchanger), member 5 [Source:HGNC Symbol;Acc:HGNC:9359]"	ENSG00000170615	protein_coding	exon	coding exon	1	3811	7:103421362:103421422	ENSG00000170615	SLC26A5	3.996688881	0.009977641	-0.687584224	-0.722310711	-0.397512338	-0.034726487	0.290071886	0.324798373	9.961963646	0.507406698	0.48671318	0.699373804	0.972983807	0.777709404	0.752051477	-6.746355511	-5.914295826	-6.961508684	-7.148953621	-6.252125608	-7.014051949	0.223893749	0.877627005

SPAG16	0.027058332	-0.100901951	0.033803028	-0.127960283	0.006744696	0.134704979	8	0.133544289	0.816496581	0.816496581	0.816496581	0.816496581	0.816496581	0.816496581	23	72	0	P_239145	P_239145	P_239145	chr2	213340183	213340243	+	SPAG16	sperm associated antigen 16 [Source:HGNC Symbol;Acc:HGNC:23225]	ENSG00000144451	protein_coding	3'UTR;exon	multiple Ref	1	3812	2:213340183:213340243	ENSG00000144451	SPAG16	3.92082452	0.01652842	0.257769174	-0.961234873	0.322022012	-1.219004047	0.064252838	1.283256885	9.961963646	0.801831678	0.359184355	0.754091679	0.250920469	0.950039938	0.228450196	-6.962223196	-5.717166088	-6.990784855	-6.392501803	-6.294489872	-6.239286846	0.705160522	0.570490078

TCF12	0.080086813	-0.086322168	-0.056589084	-0.166408981	-0.136675897	0.029733084	8	0.065071872	0.816496581	0.816496581	0.816496581	0.816496581	0.816496581	0.816496581	23	73	0	P_116628	P_116628	P_116628	chr15	57286450	57286510	+	TCF12	transcription factor 12 [Source:HGNC Symbol;Acc:HGNC:11623]	ENSG00000140262	protein_coding	3'UTR;exon	multiple Ref	1	3813	15:57286450:57286510	ENSG00000140262	TCF12	7.278573458	0.005607097	1.309898411	-1.411883762	-0.925570003	-2.721782173	-2.235468414	0.486313759	9.961963646	0.219630749	0.188448902	0.376546679	0.021563717	0.049479764	0.63726513	-6.135062646	-5.228013143	-6.598452476	-4.126902095	-4.15906664	-6.94369763	2.867974623	0.090265756

EMBP1	0.263339675	-0.480187933	0.015081377	-0.743527608	-0.248258298	0.49526931	8	0.258875624	0.816496581	0.816496581	0.816496581	0.816496581	0.816496581	0.816496581	23	74	0	P_203773	P_203773	P_203773	chr1	121571725	121571785	+	EMBP1	embigin pseudogene 1 [Source:HGNC Symbol;Acc:HGNC:38661]	ENSG00000231752	transcribed_unprocessed_pseudogene	exon	non-coding exon	1	3814	1:121571725:121571785	ENSG00000231752	EMBP1	8.499348491	0.056024659	1.362612333	-2.484661677	0.078036363	-3.84727401	-1.28457597	2.56269804	9.961963646	0.203012678	0.032367169	0.939344299	0.003249233	0.228006758	0.028320693	-6.069969718	-3.679193754	-7.043795993	-2.207640021	-5.486718297	-4.321469563	5.175903278	0.020586723

DNASE1L2	-0.130551939	0.190224015	-0.109767336	0.320775954	0.020784604	-0.29999135	8	0.125597027	0.816496581	0.816496581	0.816496581	0.816496581	0.816496581	0.816496581	23	75	0	P_117967	P_117967	P_117967	chr16	2236894	2236954	+	DNASE1L2	deoxyribonuclease I-like 2 [Source:HGNC Symbol;Acc:HGNC:2958]	ENSG00000167968	protein_coding	exon	coding exon	1	3815	16:2236894:2236954	ENSG00000167968	DNASE1L2	7.418993344	0.014874559	-1.31101275	1.910244381	-1.102292139	3.221257131	0.208720611	-3.01253652	9.961963646	0.219268085	0.085282409	0.296252133	0.009199417	0.83887222	0.013114465	-6.13370516	-4.554812538	-6.421298183	-3.27200687	-6.27356619	-3.556907475	4.346379012	0.033423224

TRIO	-0.075177035	-0.165562061	-0.228975724	-0.090385025	-0.153798688	-0.063413663	8	0.078920995	0.816496581	0.816496581	0.816496581	0.816496581	0.816496581	0.816496581	23	76	0	P_312830	P_312830	P_312830	chr5	14159220	14159280	-	TRIO	trio Rho guanine nucleotide exchange factor [Source:HGNC Symbol;Acc:HGNC:12303]	ENSG00000038382	protein_coding	intron	intron antisense	0	3816	5:14159220:14159280	ENSG00000038382	TRIO	6.646773254	0.007208528	-1.084445007	-2.388268562	-3.303024373	-1.303823554	-2.218579366	-0.914755811	9.961963646	0.303714262	0.038159798	0.008013031	0.221616575	0.050915011	0.381927871	-6.392041449	-3.830568377	-3.037555905	-6.291707609	-4.185343479	-6.632559553	4.208107694	0.036402211

CYP24A1	0.029252543	-0.115837541	0.137473633	-0.145090085	0.10822109	0.253311175	8	0.15493811	0.816496581	0.816496581	0.816496581	0.816496581	0.816496581	0.816496581	23	77	0	A_24_P356930	A_24_P356930	A_24_P356930	chr20	54154302	54154362	-	CYP24A1	"cytochrome P450, family 24, subfamily A, polypeptide 1 [Source:HGNC Symbol;Acc:HGNC:2602]"	ENSG00000019186	protein_coding	3'UTR	3'UTR	1	3817	20:54154302:54154362	ENSG00000019186	CYP24A1	3.746785084	0.021484665	0.244424633	-0.967900403	1.148684469	-1.212325036	0.904259835	2.116584871	9.961963646	0.811862622	0.356004853	0.277519471	0.253355529	0.387202652	0.060473082	-6.965856973	-5.710990497	-6.370752251	-6.40022815	-5.879260119	-5.053353505	1.513899993	0.270624393

CDH1	0.204614782	0.086995691	0.222146519	-0.117619091	0.017531737	0.135150828	8	0.095910735	0.816496581	0.816496581	0.816496581	0.816496581	0.816496581	0.816496581	23	80	0	P_226738	P_226738	P_226738	chr16	68829737	68829797	+	CDH1	"cadherin 1, type 1, E-cadherin (epithelial) [Source:HGNC Symbol;Acc:HGNC:1748]"	ENSG00000039068	protein_coding	3'UTR;exon	multiple Ref	1	3820	16:68829737:68829797	ENSG00000039068	CDH1	9.050773019	0.009593877	2.558500772	1.087793077	2.777717406	-1.470707695	0.219216634	1.689924329	9.961963646	0.028524982	0.30230348	0.019593061	0.172236802	0.830907554	0.122040765	-4.259481645	-5.594073856	-3.935016663	-6.079837065	-6.271188446	-5.700926115	3.418635877	0.060988988

CDC16	-0.024718486	-0.04252659	-0.048754271	-0.017808104	-0.024035785	-0.006227681	8	0.031003656	0.816496581	0.816496581	0.816496581	0.816496581	0.816496581	0.816496581	23	83	0	P_220018	P_220018	P_220018	chr13	114262954	114263014	+	CDC16	cell division cycle 16 [Source:HGNC Symbol;Acc:HGNC:1720]	ENSG00000130177	protein_coding	exon	coding exon	1	3823	13:114262954:114263014	ENSG00000130177	CDC16	9.469913615	0.002978601	-0.554704008	-0.954333116	-1.094087615	-0.399629109	-0.539383607	-0.139754498	9.961963646	0.591332489	0.362498226	0.299664791	0.697863466	0.601468654	0.891639093	-6.833014387	-5.723523528	-6.430068614	-7.062973455	-6.144231252	-7.060719017	0.480940894	0.702812186

MTX2	-0.080948969	-0.006679415	-0.007361053	0.074269554	0.073587916	-0.000681638	8	0.063315677	0.816496581	0.816496581	0.816496581	0.816496581	0.816496581	0.816496581	23	84	0	P_237676	P_237676	P_237676	chr2	176330600	176330660	+	MTX2	metaxin 2 [Source:HGNC Symbol;Acc:HGNC:7506]	ENSG00000128654	protein_coding	3'UTR;exon	multiple Ref	1	3824	2:176330600:176330660	ENSG00000128654	MTX2	9.505967829	0.005426029	-1.345909603	-0.111056244	-0.122389597	1.234853359	1.223520006	-0.011333353	9.961963646	0.208160293	0.913777378	0.905024276	0.245217616	0.249284744	0.99118124	-6.090786949	-6.175589748	-7.03894649	-6.374042051	-5.556691056	-7.071308356	0.810129638	0.516828689

NUP210	-0.228843783	-0.09522296	-0.238241227	0.133620823	-0.009397444	-0.143018267	8	0.046954044	0.816496581	0.816496581	0.816496581	0.816496581	0.816496581	0.816496581	23	85	0	P_243270	P_243270	P_243270	chr3	13317317	13317377	+	NUP210	nucleoporin 210kDa [Source:HGNC Symbol;Acc:HGNC:30052]	ENSG00000132182	protein_coding	3'UTR	3'UTR antisense	0	3825	3:13317317:13317377	ENSG00000132182	NUP210	7.564087755	0.003977164	-4.444245056	-1.849270991	-4.626747471	2.594974066	-0.182502415	-2.77747648	9.961963646	0.001257901	0.094271258	0.000950334	0.026797552	0.85884892	0.019601149	-1.09677717	-4.642707431	-0.846890809	-4.341649887	-6.279000172	-3.958201635	9.843247102	0.002517855

CAPN14	0.00519466	-0.202664984	-0.331736424	-0.207859644	-0.336931083	-0.129071439	8	0.197012367	0.816496581	0.816496581	0.816496581	0.816496581	0.816496581	0.816496581	23	87	0	A_32_P167076	A_32_P167076	A_32_P167076	chr2	31173334	31173394	-	CAPN14	calpain 14 [Source:HGNC Symbol;Acc:HGNC:16664]	ENSG00000214711	protein_coding	3'UTR	3'UTR	1	3827	2:31173334:31173394	ENSG00000214711	CAPN14	4.857895781	0.03337634	0.034824378	-1.358641864	-2.223921385	-1.393466242	-2.258745763	-0.865279521	9.961963646	0.972907685	0.204226544	0.050456747	0.193785547	0.047565615	0.40723992	-6.997698577	-5.292690963	-4.857829001	-6.180032681	-4.122753506	-6.67710101	2.430889336	0.12586852

PARM1	0.195528514	0.227995545	0.206030232	0.032467031	0.010501718	-0.021965313	8	0.160327736	0.816496581	0.816496581	0.816496581	0.816496581	0.816496581	0.816496581	23	89	0	P_137930	P_137930	P_137930	chr4	75046295	75046355	+	PARM1	prostate androgen-regulated mucin-like protein 1 [Source:HGNC Symbol;Acc:HGNC:24536]	ENSG00000169116	protein_coding	3'UTR	3'UTR	1	3829	4:75046295:75046355	ENSG00000169116	PARM1	4.76180747	0.022849187	1.584237742	1.847296538	1.669326192	0.263058796	0.08508845	-0.177970346	9.961963646	0.144338664	0.094576516	0.12612731	0.797865846	0.933876822	0.862313143	-5.77809737	-4.645532055	-5.706605284	-7.111901559	-6.292835819	-7.054102855	1.469512846	0.281515756

GNB2	-0.166492396	0.055288976	-0.116721563	0.221781372	0.049770833	-0.172010539	8	0.05161183	0.816496581	0.816496581	0.816496581	0.816496581	0.816496581	0.816496581	23	90	0	P_145465	P_145465	P_145465	chr7	100679066	100679126	+	GNB2	"guanine nucleotide binding protein (G protein), beta polypeptide 2 [Source:HGNC Symbol;Acc:HGNC:4398]"	ENSG00000172354	protein_coding	3'UTR;exon	multiple Ref	1	3830	7:100679066:100679126	ENSG00000172354	GNB2	13.91768363	0.004345845	-3.093163667	1.027181146	-2.168500825	4.120344812	0.924662842	-3.195681971	9.961963646	0.011432079	0.328629953	0.055410735	0.002092725	0.376996	0.009606421	-3.35166541	-5.654544338	-4.94707736	-1.75323596	-5.861000699	-3.243348291	7.228297401	0.007331382

FCRLB	0.012854992	0.110586585	0.009985979	0.097731593	-0.002869012	-0.100600606	8	0.097537071	0.816496581	0.816496581	0.816496581	0.816496581	0.816496581	0.816496581	23	91	0	P_103853	P_103853	P_103853	chr1	161723521	161723581	+	FCRLB	Fc receptor-like B [Source:HGNC Symbol;Acc:HGNC:26431]	ENSG00000162746	protein_coding	exon	coding exon	1	3831	1:161723521:161723581	ENSG00000162746	FCRLB	8.454994099	0.009846527	0.15866308	1.364917899	0.123252216	1.206254819	-0.035410864	-1.241665682	9.961963646	0.877104676	0.202310601	0.904358578	0.255585094	0.972451624	0.242798908	-6.984640546	-5.285152824	-7.038830948	-6.407222934	-6.296018352	-6.288686677	0.816900706	0.513545204

ANKRD13D	-0.085704903	-0.026069282	-0.003640559	0.059635621	0.082064345	0.022428724	8	0.063099194	0.816496581	0.816496581	0.816496581	0.816496581	0.816496581	0.816496581	23	93	0	A_23_P202667	A_23_P202667	A_23_P202667	chr11	67286548	67286608	+	ANKRD13D;ADRBK1	"ankyrin repeat domain 13 family, member D [Source:HGNC Symbol;Acc:HGNC:27880];adrenergic, beta, receptor kinase 1 [Source:HGNC Symbol;Acc:HGNC:289]"	ENSG00000172932	protein_coding	"3'ext;3'ext,3'UTR;promoter"	multiple non-Ref	0	3833	11:67286548:67286608	ENSG00000172932	ANKRD13D	7.463844058	0.005404052	-1.427879409	-0.434325109	-0.060653226	0.9935543	1.367226183	0.373671883	9.961963646	0.183916103	0.67330883	0.952834946	0.3439595	0.201609746	0.716477923	-5.986969325	-6.083611354	-7.045111938	-6.635026914	-5.388220284	-6.995631189	0.871207497	0.487968148

ALDH1A1	-0.391827196	-0.157283679	-0.380813682	0.234543517	0.011013514	-0.223530003	8	0.19913537	0.816496581	0.816496581	0.816496581	0.816496581	0.816496581	0.816496581	23	95	0	P_148894	P_148894	P_148894	chr9	72900903	72900963	-	ALDH1A1	"aldehyde dehydrogenase 1 family, member A1 [Source:HGNC Symbol;Acc:HGNC:402]"	ENSG00000165092	protein_coding	3'UTR	3'UTR	1	3835	9:72900903:72900963	ENSG00000165092	ALDH1A1	5.344147193	0.034051727	-2.600582484	-1.043901966	-2.527485079	1.556680518	0.073097405	-1.483583113	9.961963646	0.026541315	0.321200422	0.030080549	0.150717957	0.943175457	0.168853172	-4.188839919	-5.638136441	-4.357505823	-5.964291776	-6.293844051	-5.985798604	3.145092952	0.073839718

OXA1L	-0.088133909	-0.019890321	-0.039975946	0.068243588	0.048157964	-0.020085625	8	0.09748092	0.816496581	0.816496581	0.816496581	0.816496581	0.816496581	0.816496581	23	96	0	P_114367	P_114367	P_114367	chr14	22771582	22771642	+	OXA1L	oxidase (cytochrome c) assembly 1-like [Source:HGNC Symbol;Acc:HGNC:8526]	ENSG00000155463	protein_coding	3'UTR;exon	multiple Ref	1	3836	14:22771582:22771642	ENSG00000155463	OXA1L	10.03244897	0.009837734	-1.088281223	-0.24560652	-0.493624662	0.842674703	0.594656561	-0.248018142	9.961963646	0.30209821	0.810972737	0.632272303	0.419182151	0.565333741	0.809157819	-6.387978783	-6.150386982	-6.915665696	-6.774578493	-6.111971042	-7.037877816	0.435595661	0.732352731

NOL4L	0.108077719	0.09221212	0.099682749	-0.015865599	-0.00839497	0.007470629	8	0.100640121	0.816496581	0.816496581	0.816496581	0.816496581	0.816496581	0.816496581	23	98	0	P_130944	P_130944	P_130944	chr20	32485633	32485693	+	NOL4L	nucleolar protein 4-like [Source:HGNC Symbol;Acc:HGNC:16106]	ENSG00000197183	protein_coding	3'UTR;intron;promoter	multiple non-Ref	0	3838	20:32485633:32485693	ENSG00000197183	NOL4L	5.072528003	0.010340369	1.30170864	1.11062034	1.200598023	-0.1910883	-0.101110617	0.089977683	9.961963646	0.222311404	0.29281885	0.25767697	0.852294712	0.921468719	0.930088302	-6.145014454	-5.570599388	-6.312314478	-7.129681103	-6.291250471	-7.06695747	0.737365436	0.553468978

TMEM160	0.018793436	0.247060346	0.199138418	0.22826691	0.180344982	-0.047921928	8	0.085323641	0.816496581	0.816496581	0.816496581	0.816496581	0.816496581	0.816496581	23	100	0	A_23_P107994	A_23_P107994	A_23_P107994	chr19	47046058	47046118	-	TMEM160	transmembrane protein 160 [Source:HGNC Symbol;Acc:HGNC:26042]	ENSG00000130748	protein_coding	exon	coding exon	1	3840	19:47046058:47046118	ENSG00000130748	TMEM160	12.17892105	0.00805302	0.256491172	3.371858021	2.717823732	3.115366849	2.46133256	-0.654034289	9.961963646	0.802790736	0.007136819	0.021710489	0.011008659	0.033684231	0.52789838	-6.962579455	-2.264496847	-4.036695782	-3.453663079	-3.80261127	-6.842603788	5.835534917	0.014432238

PUS10	0.338403133	0.105551471	0.184967284	-0.232851662	-0.153435849	0.079415812	8	0.06267953	0.816496581	0.816496581	0.816496581	0.816496581	0.816496581	0.816496581	23	101	0	P_235109	P_235109	P_235109	chr2	61008789	61008849	-	PUS10	pseudouridylate synthase 10 [Source:HGNC Symbol;Acc:HGNC:26505]	ENSG00000162927	protein_coding	3'UTR;exon	multiple Ref	1	3841	2:61008789:61008849	ENSG00000162927	PUS10	6.121367651	0.005361663	5.660179969	1.765469245	3.093789662	-3.894710724	-2.566390307	1.328320417	9.961963646	0.000212486	0.108058148	0.011419916	0.003008122	0.028142185	0.213698829	0.74588247	-4.761327057	-3.395384972	-2.128161178	-3.634280892	-6.184458414	11.37426989	0.00147566

ZCCHC18	0.378348579	-0.138756934	0.065164949	-0.517105513	-0.31318363	0.203921883	8	0.199641644	0.816496581	0.816496581	0.816496581	0.816496581	0.816496581	0.816496581	23	104	0	A_24_P6825	A_24_P6825	A_24_P6825	chrX	104114901	104114961	+	ZCCHC18	"zinc finger, CCHC domain containing 18 [Source:HGNC Symbol;Acc:HGNC:32459]"	ENSG00000166707	protein_coding	exon	coding exon	1	3844	X:104114901:104114961	ENSG00000166707	ZCCHC18	4.299450002	0.034213857	2.505167226	-0.918754142	0.431478017	-3.423921368	-2.073689209	1.350232159	9.961963646	0.031251572	0.379931955	0.675309141	0.006540146	0.064986599	0.206817736	-4.348681602	-5.755693258	-6.946394793	-2.92503372	-4.40806905	-6.157321867	4.192116105	0.036766782

ZNF771	-0.050063876	0.079792206	-0.011492656	0.129856083	0.03857122	-0.091284862	8	0.07203768	0.816496581	0.816496581	0.816496581	0.816496581	0.816496581	0.816496581	23	105	0	P_310200	P_310200	P_310200	chr16	30413896	30413956	-	ZNF771	zinc finger protein 771 [Source:HGNC Symbol;Acc:HGNC:29653]	ENSG00000179965	protein_coding	3'UTR;intron	multiple non-Ref	0	3845	16:30413896:30413956	ENSG00000179965	ZNF771	8.70219498	0.006374077	-0.768000401	1.224045177	-0.17630206	1.992045578	0.591698342	-1.400347236	9.961963646	0.460303997	0.249095082	0.863589125	0.074475855	0.567236857	0.191776883	-6.685740766	-5.448526539	-7.030172799	-5.32509482	-6.113771899	-6.094120187	1.399753087	0.299654005

TBC1D14	-0.046108811	-0.069257789	-0.122719266	-0.023148978	-0.076610455	-0.053461477	8	0.043493099	0.816496581	0.816496581	0.816496581	0.816496581	0.816496581	0.816496581	23	108	0	A_24_P119617	A_24_P119617	A_24_P119617	chr4	6908280	6908340	-	TBC1D14	"TBC1 domain family, member 14 [Source:HGNC Symbol;Acc:HGNC:29246]"	ENSG00000132405	protein_coding	promoter	promoter antisense	0	3848	4:6908280:6908340	ENSG00000132405	TBC1D14	8.066143762	0.003725782	-0.925168522	-1.38965036	-2.462349364	-0.464481838	-1.537180842	-1.072699004	9.961963646	0.376745487	0.194907132	0.033625741	0.652287756	0.155380729	0.308703673	-6.550423157	-5.255221662	-4.466236867	-7.032897247	-5.173159457	-6.476725937	2.096596797	0.164567871

RFFL	-0.145896371	-0.107633368	-0.039275923	0.038263003	0.106620448	0.068357444	8	0.093109451	0.816496581	0.816496581	0.816496581	0.816496581	0.816496581	0.816496581	23	109	0	A_32_P185741	A_32_P185741	A_32_P185741	chr17	35082457	35082517	+	RFFL;RAD51L3-RFFL	ring finger and FYVE-like domain containing E3 ubiquitin protein ligase [Source:HGNC Symbol;Acc:HGNC:24821];Uncharacterized protein [Source:UniProtKB/TrEMBL;Acc:K7EIL6]	ENSG00000092871	protein_coding	intron	intron antisense	0	3849	17:35082457:35082517	ENSG00000092871	RFFL	7.172850231	0.009168661	-1.866109698	-1.376700939	-0.502364664	0.489408759	1.363745034	0.874336276	9.961963646	0.091704746	0.198754362	0.62632887	0.63514912	0.202667499	0.402521572	-5.370897865	-5.270937557	-6.91102817	-7.020186333	-5.392453311	-6.669105689	1.415637752	0.295410229

RABIF	-0.14045187	-0.111348488	-0.012616293	0.029103381	0.127835577	0.098732195	8	0.130442392	0.816496581	0.816496581	0.816496581	0.816496581	0.816496581	0.816496581	23	110	0	P_104466	P_104466	P_104466	chr1	202878982	202879042	-	RABIF	RAB interacting factor [Source:HGNC Symbol;Acc:HGNC:9797]	ENSG00000183155	protein_coding	3'UTR	3'UTR	1	3850	1:202878982:202879042	ENSG00000183155	RABIF	7.994855799	0.015870831	-1.36544218	-1.082505507	-0.122652826	0.282936673	1.242789353	0.959852681	9.961963646	0.202151236	0.304533822	0.904821129	0.783017417	0.242401817	0.359846245	-6.066425378	-5.599457343	-7.038911318	-7.106010688	-5.534872093	-6.590146687	0.932864991	0.460520201

RNF141	0.07304992	-0.150899026	-0.143127142	-0.223948946	-0.216177062	0.007771884	8	0.050451912	0.816496581	0.816496581	0.816496581	0.816496581	0.816496581	0.816496581	23	111	0	P_210899	P_210899	P_210899	chr11	10530658	10530718	-	RNF141	ring finger protein 141 [Source:HGNC Symbol;Acc:HGNC:21159]	ENSG00000110315	protein_coding	3'UTR;exon	multiple Ref	1	3851	11:10530658:10530718	ENSG00000110315	RNF141	10.00179785	0.004250775	1.37224387	-2.834640541	-2.688645583	-4.206884411	-4.060889453	0.145994958	9.961963646	0.200093299	0.01777332	0.022823685	0.001824066	0.002301223	0.88683731	-6.057885892	-3.12233109	-4.086122265	-1.610914882	-1.237861676	-7.059746851	8.559511572	0.004136471

GLIS1	0.327833783	-0.037273878	0.345198268	-0.365107661	0.017364485	0.382472146	8	0.301517154	0.816496581	0.816496581	0.816496581	0.816496581	0.816496581	0.816496581	23	113	0	A_23_P316612	A_23_P316612	A_23_P316612	chr1	53506256	53506316	-	GLIS1	GLIS family zinc finger 1 [Source:HGNC Symbol;Acc:HGNC:29525]	ENSG00000174332	protein_coding	3'UTR	3'UTR	1	3853	1:53506256:53506316	ENSG00000174332	GLIS1	5.648264526	0.075214454	1.464025636	-0.166456039	1.541571186	-1.630481675	0.077545549	1.708027224	9.961963646	0.174015818	0.87112807	0.154320004	0.134169731	0.939724953	0.118548309	-5.939904108	-6.167501879	-5.884194141	-5.861955506	-6.293487854	-5.674924392	1.688226867	0.232265761

PLEKHG7	-0.322984279	-0.053585349	-0.375829676	0.269398929	-0.052845397	-0.322244327	8	0.062399078	0.816496581	0.816496581	0.816496581	0.816496581	0.816496581	0.816496581	23	114	0	P_216631	P_216631	P_216631	chr12	92756330	92756390	+	PLEKHG7	"pleckstrin homology domain containing, family G (with RhoGef domain) member 7 [Source:HGNC Symbol;Acc:HGNC:33829]"	ENSG00000187510	protein_coding	exon	coding exon	1	3854	12:92756330:92756390	ENSG00000187510	PLEKHG7	4.782166164	0.005333493	-5.416529872	-0.898640166	-6.302760852	4.517889706	-0.88623098	-5.404120686	9.961963646	0.000298369	0.39004786	9.03E-05	0.001122698	0.396382495	0.00030364	0.393699021	-5.773427317	1.600867996	-1.106910577	-5.895108677	0.31997349	20.04539958	0.000152652

SYNJ1	-0.349983487	-0.048751205	-0.396609718	0.301232282	-0.046626231	-0.347858513	8	0.077923685	0.816496581	0.816496581	0.816496581	0.816496581	0.816496581	0.816496581	23	115	0	P_241622	P_241622	P_241622	chr21	32678644	32678704	-	SYNJ1	synaptojanin 1 [Source:HGNC Symbol;Acc:HGNC:11503]	ENSG00000159082	protein_coding	exon	coding exon	1	3855	21:32678644:32678704	ENSG00000159082	SYNJ1	5.830906859	0.007082912	-5.093159336	-0.709455349	-5.771690839	4.383703987	-0.678531503	-5.06223549	9.961963646	0.000474305	0.49431175	0.000182415	0.001381944	0.512888025	0.000496191	-0.087074676	-5.923504279	0.868992768	-1.322886	-6.057474734	-0.19150058	17.50991798	0.00026828

PER3	0.173787791	0.066887975	0.174485381	-0.106899817	0.000697589	0.107597406	8	0.083859894	0.816496581	0.816496581	0.816496581	0.816496581	0.816496581	0.816496581	23	116	0	P_200148	P_200148	P_200148	chr1	7810479	7810539	+	PER3	period circadian clock 3 [Source:HGNC Symbol;Acc:HGNC:8847]	ENSG00000049246	protein_coding	exon	coding exon	1	3856	1:7810479:7810539	ENSG00000049246	PER3	6.817307722	0.00785415	2.401680806	0.924366229	2.411321223	-1.477314577	0.009640417	1.486954994	9.961963646	0.037296623	0.377142996	0.036688051	0.170493273	0.992498506	0.167976619	-4.520547938	-5.750686539	-4.550952911	-6.07110427	-6.296635825	-5.981325711	2.805096685	0.094573769

USP46	0.084655418	0.055268907	0.139084236	-0.029386512	0.054428817	0.083815329	8	0.081101712	0.816496581	0.816496581	0.816496581	0.816496581	0.816496581	0.816496581	23	117	0	P_248975	P_248975	P_248975	chr4	52610540	52610600	-	USP46	ubiquitin specific peptidase 46 [Source:HGNC Symbol;Acc:HGNC:20075]	ENSG00000109189	protein_coding	3'UTR;exon	multiple Ref	1	3857	4:52610540:52610600	ENSG00000109189	USP46	8.189840429	0.007488765	1.198106104	0.782206453	1.96842299	-0.415899651	0.770316887	1.186216537	9.961963646	0.258602823	0.452284643	0.077456692	0.686300869	0.458990085	0.263057016	-6.267002924	-5.869453994	-5.262149843	-7.055836923	-5.99041301	-6.352684284	1.34924409	0.313612416

MYH3	0.017712148	-0.018138855	-0.113101215	-0.035851004	-0.130813363	-0.09496236	8	0.069106758	0.816496581	0.816496581	0.816496581	0.816496581	0.816496581	0.816496581	23	118	0	P_227834	P_227834	P_227834	chr17	10635765	10635825	-	MYH3	"myosin, heavy chain 3, skeletal muscle, embryonic [Source:HGNC Symbol;Acc:HGNC:7573]"	ENSG00000109063	protein_coding	exon	coding exon	1	3858	17:10635765:10635825	ENSG00000109063	MYH3	4.340052488	0.006041867	0.279081655	-0.285805073	-1.782080525	-0.564886728	-2.061162181	-1.496275453	9.961963646	0.785890124	0.78088214	0.105186434	0.584646015	0.066364095	0.165574182	-6.956024861	-6.13920858	-5.543381539	-6.977862639	-4.427076391	-5.968928468	1.690284815	0.231851768

LPCAT4	0.081744658	0.045220531	0.090985889	-0.036524127	0.009241231	0.045765358	8	0.051230534	0.816496581	0.816496581	0.816496581	0.816496581	0.816496581	0.816496581	23	122	0	A_23_P49009	A_23_P49009	A_23_P49009	chr15	34360179	34361429	-	LPCAT4;NUTM1	"lysophosphatidylcholine acyltransferase 4 [Source:HGNC Symbol;Acc:HGNC:30059];NUT midline carcinoma, family member 1 [Source:HGNC Symbol;Acc:HGNC:29919]"	ENSG00000176454	protein_coding	"3'ext;exon;exon,intron"	multiple non-Ref	0	3862	15:34360179:34361429	ENSG00000176454	LPCAT4	7.614763515	0.004314355	1.524217978	0.843185937	1.696530769	-0.681032041	0.172312791	0.853344832	9.961963646	0.158549979	0.418909453	0.120755605	0.511370415	0.866641961	0.41351562	-5.859870728	-5.820591533	-5.66775179	-6.90173574	-6.280916909	-6.687527617	1.189018894	0.362951946

MEIS1	-0.077915891	0.136311984	-0.178916153	0.214227875	-0.101000262	-0.315228136	8	0.106331761	0.816496581	0.816496581	0.816496581	0.816496581	0.816496581	0.816496581	23	124	0	P_128002	P_128002	P_128002	chr2	66572392	66572452	+	MEIS1	Meis homeobox 1 [Source:HGNC Symbol;Acc:HGNC:7000]	ENSG00000143995	protein_coding	3'UTR;exon	multiple Ref	1	3864	2:66572392:66572452	ENSG00000143995	MEIS1	5.571470589	0.011286375	-0.898244241	1.571456771	-2.062613951	2.469701011	-1.16436971	-3.634070722	9.961963646	0.390248867	0.147267096	0.066203045	0.033205815	0.271400745	0.004610143	-6.575132997	-5.024851788	-5.115319225	-4.551895123	-5.622096909	-2.496439535	4.698724222	0.027053923

FAM83F	-0.042813569	0.044303148	-0.121062185	0.087116716	-0.078248617	-0.165365333	8	0.134323547	0.816496581	0.816496581	0.816496581	0.816496581	0.816496581	0.816496581	23	125	0	P_242657	P_242657	P_242657	chr22	40034616	40034676	-	FAM83F	"family with sequence similarity 83, member F [Source:HGNC Symbol;Acc:HGNC:25148]"	ENSG00000133477	protein_coding	3'ext;3'UTR	multiple non-Ref	0	3865	22:40034616:40034676	ENSG00000133477	FAM83F	5.995426591	0.016696048	-0.405807632	0.41992658	-1.147485725	0.825734212	-0.741678093	-1.567412305	9.961963646	0.693462957	0.683452064	0.27799153	0.428286612	0.475404241	0.148204649	-6.909236502	-6.089974029	-6.372078479	-6.789019544	-6.012153049	-5.872732509	0.891074395	0.4789424

CDHR5	0.024081037	0.035359947	0.08652279	0.01127891	0.062441753	0.051162844	8	0.13733685	0.816496581	0.816496581	0.816496581	0.816496581	0.816496581	0.816496581	23	127	0	P_107524	P_107524	P_107524	chr11	626312	626372	-	CDHR5	cadherin-related family member 5 [Source:HGNC Symbol;Acc:HGNC:7521]	ENSG00000099834	protein_coding	promoter	promoter sense	0	3867	11:626312:626372	ENSG00000099834	CDHR5	5.383769853	0.017353425	0.223886656	0.328749142	0.804421268	0.104862486	0.580534612	0.475672126	9.961963646	0.827370204	0.749151719	0.43992859	0.918566209	0.574450681	0.644566718	-6.971076513	-6.125432491	-6.704821065	-7.143602978	-6.12049267	-6.94916235	0.229929226	0.873459844

TMEM147	-0.094642005	0.073559007	0.076053522	0.168201012	0.170695527	0.002494515	8	0.079387442	0.816496581	0.816496581	0.816496581	0.816496581	0.816496581	0.816496581	23	128	0	P_125491	P_125491	P_125491	chr19	35546269	35546329	+	TMEM147	transmembrane protein 147 [Source:HGNC Symbol;Acc:HGNC:30414]	ENSG00000105677	protein_coding	exon	non-coding exon	1	3868	19:35546269:35546329	ENSG00000105677	TMEM147	11.45815709	0.007267827	-1.359650417	1.056766851	1.092603674	2.416417268	2.452254091	0.035836823	9.961963646	0.203917629	0.315571021	0.30028526	0.036370301	0.03421092	0.972120399	-6.073674093	-5.625369303	-6.43164943	-4.640605911	-3.817093908	-7.070676965	2.668849173	0.104770768

PHLPP1	-0.079981657	-0.234649176	-0.083262964	-0.154667519	-0.003281307	0.151386212	8	0.080146697	0.816496581	0.816496581	0.816496581	0.816496581	0.816496581	0.816496581	23	131	0	A_23_P89762	A_23_P89762	A_23_P89762	chr18	62979946	62980006	+	PHLPP1	PH domain and leucine rich repeat protein phosphatase 1 [Source:HGNC Symbol;Acc:HGNC:20610]	ENSG00000081913	protein_coding	3'UTR	3'UTR	1	3871	18:62979946:62980006	ENSG00000081913	PHLPP1	6.317737396	0.007365099	-1.141423286	-3.348693234	-1.188251028	-2.207269948	-0.046827742	2.160442206	9.961963646	0.280388565	0.007420099	0.26229052	0.051898484	0.963575975	0.056168967	-6.330553163	-2.301223288	-6.326389274	-4.983544081	-6.295519049	-4.983348806	3.911712997	0.043925342

RNF168	-0.256133614	-0.093294745	-0.248961982	0.16283887	0.007171632	-0.155667238	8	0.074239684	0.816496581	0.816496581	0.816496581	0.816496581	0.816496581	0.816496581	23	133	0	A_24_P567454	A_24_P567454	A_24_P567454	chr3	196470032	196470092	-	RNF168	"ring finger protein 168, E3 ubiquitin protein ligase [Source:HGNC Symbol;Acc:HGNC:26661]"	ENSG00000163961	protein_coding	3'UTR	3'UTR	1	3873	3:196470032:196470092	ENSG00000163961	RNF168	9.981353701	0.006632744	-3.851816609	-1.40299526	-3.743967388	2.448821349	0.107849221	-2.340972128	9.961963646	0.003225292	0.191008612	0.00384692	0.034412178	0.916256514	0.041362604	-2.066007286	-5.238924497	-2.289448211	-4.586713085	-6.290502371	-4.690193643	7.051781106	0.007949934

ZFYVE16	-0.074699272	-0.096589342	-0.070295046	-0.02189007	0.004404225	0.026294296	8	0.07715948	0.816496581	0.816496581	0.816496581	0.816496581	0.816496581	0.816496581	23	134	0	P_139851	P_139851	P_139851	chr5	80479577	80479637	+	ZFYVE16	"zinc finger, FYVE domain containing 16 [Source:HGNC Symbol;Acc:HGNC:20756]"	ENSG00000039319	protein_coding	3'ext	3'extended sense	0	3874	5:80479577:80479637	ENSG00000039319	ZFYVE16	7.2936441	0.006987738	-1.094444395	-1.415163249	-1.029916592	-0.320718854	0.064527803	0.385246657	9.961963646	0.299515761	0.187511883	0.32740577	0.755049987	0.949826464	0.70815242	-6.381428224	-5.223975991	-6.496871581	-7.093652216	-6.294471046	-6.990901163	0.752777192	0.545499396

NANOS1	0.883473076	-0.188598895	0.757981907	-1.072071972	-0.125491169	0.946580802	8	0.128952019	0.816496581	0.816496581	0.816496581	0.816496581	0.816496581	0.816496581	23	135	0	A_24_P383609	A_24_P383609	A_24_P383609	chr10	119031354	119031414	+	NANOS1	nanos homolog 1 (Drosophila) [Source:HGNC Symbol;Acc:HGNC:23044]	ENSG00000188613	protein_coding	3'UTR;exon	multiple Ref	1	3875	10:119031354:119031414	ENSG00000188613	NANOS1	8.422229362	0.015560375	8.674189752	-1.851717554	7.442081787	-10.52590731	-1.232107965	9.293799341	9.961963646	5.91E-06	0.093894275	2.25E-05	1.02E-06	0.246197872	3.18E-06	4.436852065	-4.639205484	3.040753144	6.16898117	-5.546997329	5.039487311	55.45708799	1.60E-06

SMAD1	0.086586721	-0.014767552	0.13532455	-0.101354273	0.048737829	0.150092102	8	0.088388445	0.816496581	0.816496581	0.816496581	0.816496581	0.816496581	0.816496581	23	140	0	P_251064	P_251064	P_251064	chr4	145539866	145539926	+	SMAD1	SMAD family member 1 [Source:HGNC Symbol;Acc:HGNC:6767]	ENSG00000170365	protein_coding	exon	coding exon	1	3880	4:145539866:145539926	ENSG00000170365	SMAD1	9.027687243	0.008480561	1.151554838	-0.196400156	1.799740632	-1.347954994	0.648185795	1.996140788	9.961963646	0.276391701	0.848245667	0.102210206	0.207524101	0.531519976	0.073970215	-6.319365768	-6.16179373	-5.517312717	-6.23736898	-6.077952159	-5.242729741	1.804237241	0.210201445

S100A7L2	0.075285307	-0.101856109	-0.217965067	-0.177141416	-0.293250374	-0.116108958	8	0.330131435	0.816496581	0.816496581	0.816496581	0.816496581	0.816496581	0.816496581	23	141	0	P_103233	P_103233	P_103233	chr1	153437081	153437141	-	S100A7L2	S100 calcium binding protein A7-like 2 [Source:HGNC Symbol;Acc:HGNC:21655]	ENSG00000197364	protein_coding	exon	coding exon	1	3881	1:153437081:153437141	ENSG00000197364	S100A7L2	3.191122533	0.089728998	0.307814762	-0.416453294	-0.891181403	-0.724268055	-1.198996164	-0.474728109	9.961963646	0.764562628	0.685908893	0.393846836	0.485562659	0.258271822	0.645216375	-6.946902353	-6.09147793	-6.629941026	-6.870088169	-5.584099173	-6.94964155	0.541651727	0.664644124

SERPINA3	0.056863202	0.182277444	0.137622222	0.125414242	0.08075902	-0.044655222	8	0.14407444	0.816496581	0.816496581	0.816496581	0.816496581	0.816496581	0.816496581	23	142	0	A_14_P134662	A_14_P134662	A_14_P134662	chr14	94619594	94619653	+	SERPINA3	"serpin peptidase inhibitor, clade A (alpha-1 antiproteinase, antitrypsin), member 3 [Source:HGNC Symbol;Acc:HGNC:16]"	ENSG00000196136	protein_coding	3'UTR	3'UTR	1	3882	14:94619594:94619653	ENSG00000196136	SERPINA3	4.585394571	0.018876044	0.506899021	1.624886658	1.226813958	1.117987637	0.719914937	-0.3980727	9.961963646	0.623256374	0.135365159	0.248097081	0.289807504	0.488123726	0.698973845	-6.859938827	-4.953957329	-6.282072186	-6.505925833	-6.028182136	-6.985496357	1.054818584	0.410929025

HAS2	0.171644695	0.201038857	0.400440561	0.029394162	0.228795866	0.199401704	8	0.222508624	0.816496581	0.816496581	0.816496581	0.816496581	0.816496581	0.816496581	23	143	0	P_147879	P_147879	P_147879	chr8	121613032	121613092	-	HAS2	hyaluronan synthase 2 [Source:HGNC Symbol;Acc:HGNC:4819]	ENSG00000170961	protein_coding	3'UTR	3'UTR	1	3883	8:121613032:121613092	ENSG00000170961	HAS2	3.711077236	0.041965984	1.026188892	1.201923788	2.394059749	0.175734895	1.367870857	1.192135962	9.961963646	0.329074859	0.257185476	0.037784716	0.864023012	0.201414372	0.26083182	-6.452332348	-5.473025283	-4.579509328	-7.132750601	-5.387435575	-6.345955983	1.92539135	0.189703731

COPA	0.022501658	-0.033347502	0.000764959	-0.055849161	-0.021736699	0.034112462	8	0.026182456	0.816496581	0.816496581	0.816496581	0.816496581	0.816496581	0.816496581	23	144	0	P_204472	P_204472	P_204472	chr1	160305687	160305747	-	COPA	"coatomer protein complex, subunit alpha [Source:HGNC Symbol;Acc:HGNC:2230]"	ENSG00000122218	protein_coding	exon	coding exon	1	3884	1:160305687:160305747	ENSG00000122218	COPA	8.109940346	0.002757195	0.524839405	-0.777813048	0.017842275	-1.302652454	-0.50699713	0.795655324	9.961963646	0.61117453	0.454754941	0.986116972	0.222001103	0.623189978	0.444777351	-6.850103211	-5.872850474	-7.046946753	-6.293132278	-6.161757638	-6.736150666	0.577964131	0.642624299

FAM186B	0.177875092	0.091683104	0.15682893	-0.086191988	-0.021046162	0.065145826	8	0.095009426	0.816496581	0.816496581	0.816496581	0.816496581	0.816496581	0.816496581	23	147	0	P_111481	P_111481	P_111481	chr12	49600261	49600321	-	FAM186B	"family with sequence similarity 186, member B [Source:HGNC Symbol;Acc:HGNC:25296]"	ENSG00000135436	protein_coding	3'UTR;exon	multiple Ref	1	3887	12:49600261:49600321	ENSG00000135436	FAM186B	4.877723889	0.009455689	2.24034121	1.154750979	1.975264279	-1.085590231	-0.265076932	0.820513299	9.961963646	0.049072992	0.275140186	0.076582068	0.303231129	0.796354356	0.431119181	-4.784572283	-5.524161873	-5.251582735	-6.540699775	-6.25944738	-6.715563058	2.029376923	0.173944247

LINC01300	0.370973146	0.442952082	0.400931228	0.071978935	0.029958082	-0.042020854	8	0.529070193	0.816496581	0.816496581	0.816496581	0.816496581	0.816496581	0.816496581	23	149	0	P_148072	P_148072	P_148072	chr8	141343013	141343073	+	LINC01300	long intergenic non-protein coding RNA 1300 [Source:HGNC Symbol;Acc:HGNC:50460]	ENSG00000253595	antisense	exon	non-coding exon	1	3889	8:141343013:141343073	ENSG00000253595	LINC01300	2.952350401	0.226993908	0.95363241	1.138663176	1.030643369	0.185030766	0.077010959	-0.108019807	9.961963646	0.362835904	0.28148525	0.327081094	0.856917659	0.940139577	0.916124621	-6.523635239	-5.541249923	-6.49613308	-7.130922623	-6.293531773	-7.06500786	0.553338284	0.657489726

RAB11FIP3	-0.163276624	-0.127343606	-0.15122786	0.035933018	0.012048764	-0.023884254	8	0.058478135	0.816496581	0.816496581	0.816496581	0.816496581	0.816496581	0.816496581	23	150	0	P_117728	P_117728	P_117728	chr16	520779	520839	+	RAB11FIP3	RAB11 family interacting protein 3 (class II) [Source:HGNC Symbol;Acc:HGNC:17224]	ENSG00000090565	protein_coding	exon	coding exon	1	3890	16:520779:520839	ENSG00000090565	RAB11FIP3	7.318520112	0.004952883	-2.841453761	-2.216122298	-2.63177276	0.625331463	0.209681002	-0.415650461	9.961963646	0.017567247	0.051127138	0.025160165	0.545810879	0.838142641	0.686477325	-3.781129524	-4.097298651	-4.18222782	-6.939881774	-6.273353428	-6.977811025	3.419842707	0.060938473

TMEM62	0.052390657	-0.050043348	-0.009834181	-0.102434005	-0.062224838	0.040209167	8	0.078050537	0.816496581	0.816496581	0.816496581	0.816496581	0.816496581	0.816496581	23	151	0	A_23_P49041	A_23_P49041	A_23_P49041	chr15	43184484	43184544	+	TMEM62	transmembrane protein 62 [Source:HGNC Symbol;Acc:HGNC:26269]	ENSG00000137842	protein_coding	exon	coding exon	1	3891	15:43184484:43184544	ENSG00000137842	TMEM62	7.71087004	0.007098801	0.761564996	-0.727443873	-0.142952366	-1.489008869	-0.904517362	0.584491507	9.961963646	0.463966912	0.483699547	0.889177875	0.16744463	0.387072619	0.571888057	-6.690811897	-5.910577473	-7.035972149	-6.055585999	-5.879031784	-6.887895625	0.751086767	0.546368001

SMOC1	-0.033370427	-0.07646391	0.055411951	-0.043093482	0.088782378	0.13187586	8	0.262104994	0.816496581	0.816496581	0.816496581	0.816496581	0.816496581	0.816496581	23	152	0	A_24_P258473	A_24_P258473	A_24_P258473	chr14	70030484	70030544	+	SMOC1	SPARC related modular calcium binding 1 [Source:HGNC Symbol;Acc:HGNC:20318]	ENSG00000198732	protein_coding	3'UTR	3'UTR	1	3892	14:70030484:70030544	ENSG00000198732	SMOC1	4.397621348	0.057375749	-0.170625214	-0.390965057	0.283324991	-0.220339843	0.453950205	0.674290048	9.961963646	0.867934096	0.704054048	0.782728237	0.830056404	0.659593944	0.515468416	-6.982496958	-6.102144836	-7.003459651	-7.123127763	-6.188234395	-6.828532627	0.161921199	0.919537783

MICU1	0.147001214	0.024818136	0.131238503	-0.122183078	-0.015762711	0.106420368	8	0.115423584	0.816496581	0.816496581	0.816496581	0.816496581	0.816496581	0.816496581	23	154	0	P_106243	P_106243	P_106243	chr10	72368276	72368336	-	MICU1	mitochondrial calcium uptake 1 [Source:HGNC Symbol;Acc:HGNC:1530]	ENSG00000107745	protein_coding	3'UTR;exon	multiple Ref	1	3894	10:72368276:72368336	ENSG00000107745	MICU1	8.899564954	0.012905461	1.584819429	0.267564208	1.414881717	-1.317255221	-0.169937713	1.147317509	9.961963646	0.144206619	0.794492715	0.187592166	0.21724564	0.868460615	0.278057823	-5.777295584	-6.144487494	-6.051629634	-6.275303247	-6.281347956	-6.396268281	1.277585185	0.334687701

MAP3K20	-0.118997576	-0.025231104	-0.108086226	0.093766471	0.01091135	-0.082855122	8	0.056678159	0.816496581	0.816496581	0.816496581	0.816496581	0.816496581	0.816496581	23	155	0	P_237607	P_237607	P_237607	chr2	173187563	173187623	+	ZAK	Mitogen-activated protein kinase kinase kinase MLT [Source:UniProtKB/Swiss-Prot;Acc:Q9NYL2]	ENSG00000091436	protein_coding	exon	coding exon	1	3895	2:173187563:173187623	ENSG00000091436	MAP3K20	7.232037717	0.004786427	-2.106580241	-0.446659065	-1.91341972	1.659921177	0.193160521	-1.466760656	9.961963646	0.061498225	0.664674235	0.084836887	0.128033818	0.850714587	0.173285745	-4.998933595	-6.077997133	-5.346517637	-5.820368495	-6.276878346	-6.008017305	2.207065963	0.150409808

C5orf34	-0.350960858	0.014895482	-0.146952059	0.36585634	0.204008799	-0.161847541	8	0.078145463	0.816496581	0.816496581	0.816496581	0.816496581	0.816496581	0.816496581	23	156	0	P_252707	P_252707	P_252707	chr5	43509144	43509204	-	C5orf34	chromosome 5 open reading frame 34 [Source:HGNC Symbol;Acc:HGNC:24738]	ENSG00000172244	protein_coding	exon	coding exon	1	3896	5:43509144:43509204	ENSG00000172244	C5orf34	7.603909497	0.007110708	-5.09739039	0.216343459	-2.134346309	5.313733849	2.963044081	-2.350689768	9.961963646	0.000471391	0.833085842	0.058693242	0.000345178	0.014269993	0.040683796	-0.080686165	-6.157473621	-5.001685508	0.123765673	-2.991256879	-4.674211772	12.18779458	0.001135171

SYN3	0.212667188	0.029285573	0.198526535	-0.183381615	-0.014140653	0.169240962	8	0.170647472	0.816496581	0.816496581	0.816496581	0.816496581	0.816496581	0.816496581	23	158	0	A_24_P893668	A_24_P893668	A_24_P893668	chr22	32510808	32510868	+	SYN3	synapsin III [Source:HGNC Symbol;Acc:HGNC:11496]	ENSG00000185666	protein_coding	3'ext	3'extended antisense	0	3898	22:32510808:32510868	ENSG00000185666	SYN3	3.962736666	0.025592081	1.628145886	0.224205651	1.519887312	-1.403940236	-0.108258574	1.295681661	9.961963646	0.134667644	0.827128725	0.159621297	0.19073508	0.915940017	0.224301384	-5.717091178	-6.15565689	-5.913492644	-6.166655555	-6.290455375	-6.224303324	1.445457286	0.287626028

PKHD1	-0.027830107	0.020083219	-0.008054997	0.047913326	0.01977511	-0.028138216	8	0.050668044	0.816496581	0.816496581	0.816496581	0.816496581	0.816496581	0.816496581	24	13	0	P_257313	P_257313	P_257313	chr6	51649085	51649145	-	PKHD1	polycystic kidney and hepatic disease 1 (autosomal recessive) [Source:HGNC Symbol;Acc:HGNC:9016]	ENSG00000170927	protein_coding	exon	coding exon	1	3923	6:51649085:51649145	ENSG00000170927	PKHD1	3.689363922	0.004268326	-0.521713049	0.376487145	-0.151001825	0.898200194	0.370711224	-0.52748897	9.961963646	0.613271312	0.714449319	0.882988189	0.390271234	0.718613796	0.609400403	-6.851840505	-6.107913382	-7.034682777	-6.72547881	-6.224105981	-6.921471347	0.276527273	0.841082488

NOTCH4	0.103289354	-0.007442599	0.048451703	-0.110731953	-0.054837651	0.055894302	8	0.146347584	0.816496581	0.816496581	0.816496581	0.816496581	0.816496581	0.816496581	24	14	0	P_313418	P_313418	P_313418	chr6	32196424	32196484	-	NOTCH4;GPSM3	notch 4 [Source:HGNC Symbol;Acc:HGNC:7884];G-protein signaling modulator 3 [Source:HGNC Symbol;Acc:HGNC:13945]	ENSG00000204301	protein_coding	intron;promoter	multiple non-Ref	0	3924	6:32196424:32196484	ENSG00000204301	NOTCH4	5.87656471	0.019406197	0.908094352	-0.065433484	0.42597534	-0.973527835	-0.482119012	0.491408823	9.961963646	0.385269674	0.94912335	0.67918273	0.353336541	0.640138487	0.633783533	-6.566164571	-6.179830822	-6.948924469	-6.654651559	-6.174521692	-6.941040397	0.405341617	0.752503715

TIGAR	0.245791967	0.315095945	0.528116892	0.069303977	0.282324924	0.213020947	8	0.09467024	0.816496581	0.816496581	0.816496581	0.816496581	0.816496581	0.816496581	24	15	0	A_23_P128486	A_23_P128486	A_23_P128486	chr12	4352875	4352935	+	C12orf5	chromosome 12 open reading frame 5 [Source:HGNC Symbol;Acc:HGNC:1185]	ENSG00000078237	protein_coding	3'UTR	3'UTR	1	3925	12:4352875:4352935	ENSG00000078237	TIGAR	8.407423219	0.009404024	3.104248366	3.979528226	6.669892492	0.87527986	3.565644126	2.690364266	9.961963646	0.011218651	0.002622565	5.67E-05	0.402032174	0.005163548	0.022756558	-3.332726235	-1.319615436	2.083719488	-6.746071971	-2.016362459	-4.106079507	15.1130769	0.000488248

ARL6IP4	0.002796413	0.11878794	0.051891438	0.115991527	0.049095025	-0.066896502	8	0.112568589	0.816496581	0.816496581	0.816496581	0.816496581	0.816496581	0.816496581	24	17	0	P_113158	P_113158	P_113158	chr12	122981206	122981266	+	ARL6IP4	ADP-ribosylation factor-like 6 interacting protein 4 [Source:HGNC Symbol;Acc:HGNC:18076]	ENSG00000182196	protein_coding	exon	coding exon	1	3927	12:122981206:122981266	ENSG00000182196	ARL6IP4	12.13889764	0.01238274	0.030777875	1.307403397	0.571127357	1.276625521	0.540349481	-0.73627604	9.961963646	0.976054576	0.220444559	0.580568289	0.230690231	0.600826947	0.478541744	-6.997843421	-5.353348309	-6.871862205	-6.324559537	-6.143692847	-6.783058034	0.750048344	0.546902258

NPAS2	0.039614857	0.149814666	-0.005069037	0.110199809	-0.044683894	-0.154883703	8	0.090754019	0.816496581	0.816496581	0.816496581	0.816496581	0.816496581	0.816496581	24	18	0	P_310975	P_310975	P_310975	chr2	100878521	100878581	-	NPAS2	neuronal PAS domain protein 2 [Source:HGNC Symbol;Acc:HGNC:7895]	ENSG00000170485	protein_coding	intron	intron antisense	0	3928	2:100878521:100878581	ENSG00000170485	NPAS2	4.798457992	0.008820876	0.516592233	1.95363808	-0.066102094	1.437045847	-0.582694328	-2.019740175	9.961963646	0.616713651	0.079379027	0.948604312	0.181360807	0.573051194	0.071118924	-6.854664878	-4.491507124	-7.044734908	-6.123937663	-6.119201783	-5.205977627	1.761987098	0.217944646

NRP1	0.487405993	-0.189849173	0.098806629	-0.677255166	-0.388599364	0.288655801	8	0.102280583	0.816496581	0.816496581	0.816496581	0.816496581	0.816496581	0.816496581	24	19	0	P_208055	P_208055	P_208055	chr10	33194428	33194488	-	NRP1	neuropilin 1 [Source:HGNC Symbol;Acc:HGNC:8004]	ENSG00000099250	protein_coding	3'UTR	3'UTR	1	3929	10:33194428:33194488	ENSG00000099250	NRP1	7.077956554	0.010607693	5.795968726	-2.257583785	1.174955044	-8.053552511	-4.621013682	3.432538829	9.961963646	0.000176495	0.047659442	0.267332145	1.14E-05	0.000958674	0.00644647	0.938407682	-4.033548702	-6.34142371	3.690389135	-0.396620051	-2.838663751	23.01104261	8.48E-05

SLC30A7	-0.252290432	0.123113145	-0.242734352	0.375403576	0.009556079	-0.365847497	8	0.090973526	0.816496581	0.816496581	0.816496581	0.816496581	0.816496581	0.816496581	24	20	0	P_102527	P_102527	P_102527	chr1	100981566	100981626	+	SLC30A7	"solute carrier family 30 (zinc transporter), member 7 [Source:HGNC Symbol;Acc:HGNC:19306]"	ENSG00000162695	protein_coding	3'UTR	3'UTR	1	3930	1:100981566:100981626	ENSG00000162695	SLC30A7	7.125014538	0.00885291	-3.284001844	1.602533204	-3.159612736	4.886535048	0.124389108	-4.76214594	9.961963646	0.008274202	0.140236453	0.010212035	0.000642825	0.90348134	0.000774218	-3.025762185	-4.983781752	-3.282755015	-0.525868601	-6.288462158	-0.65420299	11.65127747	0.001347477

TMEM184B	-0.045550568	0.031771848	0.015666399	0.077322416	0.061216967	-0.016105449	8	0.100558699	0.816496581	0.816496581	0.816496581	0.816496581	0.816496581	0.816496581	24	22	0	A_23_P17870	A_23_P17870	A_23_P17870	chr22	38219365	38219425	-	TMEM184B	transmembrane protein 184B [Source:HGNC Symbol;Acc:HGNC:1310]	ENSG00000198792	protein_coding	3'UTR	3'UTR	1	3932	22:38219365:38219425	ENSG00000198792	TMEM184B	7.288629621	0.010327213	-0.548969044	0.382909845	0.188809241	0.93187889	0.737778285	-0.194100605	9.961963646	0.595116176	0.709830039	0.85403335	0.373432428	0.477667932	0.849997975	-6.836365068	-6.105380382	-7.027687366	-6.694402703	-6.015056726	-7.050835613	0.322337666	0.809228131

TACO1	0.109113699	0.16447588	0.25767186	0.055362182	0.148558161	0.09319598	8	0.071011094	0.816496581	0.816496581	0.816496581	0.816496581	0.816496581	0.816496581	24	23	0	A_23_P73142	A_23_P73142	A_23_P73142	chr17	63607451	63607848	+	TACO1	translational activator of mitochondrially encoded cytochrome c oxidase I [Source:HGNC Symbol;Acc:HGNC:24316]	ENSG00000136463	protein_coding	"exon,intron"	multiple non-Ref	0	3933	17:63607451:63607848	ENSG00000136463	TACO1	11.81436229	0.006256147	1.689551496	2.546797265	3.989873693	0.857245769	2.300322197	1.443076428	9.961963646	0.122113659	0.029102365	0.002579214	0.41145711	0.044323856	0.179696329	-5.630182893	-3.58102319	-1.878802938	-6.761954288	-4.057626715	-6.039022134	5.56144579	0.016677602

ASB16	0.122757182	0.197048168	0.103140513	0.074290986	-0.019616669	-0.093907655	8	0.229572239	0.816496581	0.816496581	0.816496581	0.816496581	0.816496581	0.816496581	24	24	0	P_121702	P_121702	P_121702	chr17	44177170	44177230	+	ASB16	ankyrin repeat and SOCS box containing 16 [Source:HGNC Symbol;Acc:HGNC:19768]	ENSG00000161664	protein_coding	exon	coding exon	1	3934	17:44177170:44177230	ENSG00000161664	ASB16	6.595260284	0.044530398	0.712466173	1.143641067	0.598613665	0.431174894	-0.113852508	-0.545027402	9.961963646	0.492525547	0.279509797	0.562793569	0.675522266	0.911616563	0.5977241	-6.728244583	-5.535981909	-6.854891272	-7.048886829	-6.289795364	-6.911485667	0.444965052	0.726181279

DSN1	-0.488865578	-0.225792205	-0.429143575	0.263073374	0.059722003	-0.20335137	8	0.078378111	0.816496581	0.816496581	0.816496581	0.816496581	0.816496581	0.816496581	24	27	0	P_240713	P_240713	P_240713	chr20	36762460	36762520	-	DSN1	"DSN1, MIS12 kinetochore complex component [Source:HGNC Symbol;Acc:HGNC:16165]"	ENSG00000149636	protein_coding	exon	coding exon	1	3937	20:36762460:36762520	ENSG00000149636	DSN1	8.695408429	0.007139951	-7.085776929	-3.272705764	-6.220146764	3.813071165	0.865630165	-2.947441	9.961963646	3.42E-05	0.008433421	0.000100459	0.003435567	0.407056536	0.014655326	2.637312886	-2.421953506	1.489641109	-2.265076214	-5.91288593	-3.668261216	20.59754987	0.000136098

DOCK10	0.10951994	-0.036210618	0.062828216	-0.145730558	-0.046691724	0.099038834	8	0.082351332	0.816496581	0.816496581	0.816496581	0.816496581	0.816496581	0.816496581	24	29	0	P_239487	P_239487	P_239487	chr2	224789064	224789124	-	DOCK10	dedicator of cytokinesis 10 [Source:HGNC Symbol;Acc:HGNC:23479]	ENSG00000135905	protein_coding	exon	coding exon	1	3939	2:224789064:224789124	ENSG00000135905	DOCK10	3.970434512	0.007652793	1.533306078	-0.506957547	0.879610469	-2.040263625	-0.653695609	1.386568016	9.961963646	0.156322193	0.623216765	0.399791365	0.068724058	0.528107706	0.195817123	-5.847612426	-6.048391198	-6.64030829	-5.249768066	-6.074297704	-6.111652803	1.649052089	0.240310927

ST6GAL1	0.067739721	-0.004115481	-0.00357521	-0.071855202	-0.071314931	0.000540271	8	0.120034147	0.816496581	0.816496581	0.816496581	0.816496581	0.816496581	0.816496581	24	34	0	P_312148	P_312148	P_312148	chr3	187028351	187028411	+	ST6GAL1	"ST6 beta-galactosamide alpha-2,6-sialyltranferase 1 [Source:HGNC Symbol;Acc:HGNC:10860]"	ENSG00000073849	protein_coding	intron	intron sense	0	3944	3:187028351:187028411	ENSG00000073849	ST6GAL1	4.959448103	0.013777251	0.706818229	-0.042942267	-0.037304901	-0.749760496	-0.74412313	0.005637366	9.961963646	0.495879535	0.966596079	0.970978864	0.470734433	0.473988467	0.99561335	-6.732406664	-6.181115393	-7.046360658	-6.850608039	-6.010325589	-7.071361164	0.269786586	0.845777724

SPCS3	-0.392230001	0.020188634	-0.284366488	0.412418635	0.107863513	-0.304555122	8	0.124936407	0.816496581	0.816496581	0.816496581	0.816496581	0.816496581	0.816496581	24	37	0	P_251723	P_251723	P_251723	chr4	176320423	176320483	+	SPCS3	signal peptidase complex subunit 3 homolog (S. cerevisiae) [Source:HGNC Symbol;Acc:HGNC:26212]	ENSG00000129128	protein_coding	exon	non-coding exon	1	3947	4:176320423:176320483	ENSG00000129128	SPCS3	8.011548091	0.014741647	-3.956520632	0.203647723	-2.868474808	4.160168355	1.088045824	-3.07212253	9.961963646	0.002721736	0.842728582	0.016773437	0.001964258	0.302197184	0.011848685	-1.891855609	-6.160271634	-3.780474036	-1.687636234	-5.702856865	-3.454905554	8.642076154	0.004000413

ST7L	0.012964146	0.139152978	0.033769556	0.126188831	0.02080541	-0.105383422	8	0.038656026	0.816496581	0.816496581	0.816496581	0.816496581	0.816496581	0.816496581	24	38	0	P_203438	P_203438	P_203438	chr1	112584000	112584060	-	ST7L	suppression of tumorigenicity 7 like [Source:HGNC Symbol;Acc:HGNC:18441]	ENSG00000007341	protein_coding	exon	coding exon	1	3948	1:112584000:112584060	ENSG00000007341	ST7L	7.877947571	0.003406679	0.27203448	2.919930594	0.708606896	2.647896114	0.436572416	-2.211323697	9.961963646	0.79115028	0.015360616	0.494815826	0.024474527	0.671731801	0.051543878	-6.958128062	-2.985777171	-6.779642275	-4.252230233	-6.196299492	-4.901499212	3.53233716	0.056440488

CENPL	-0.375256365	-0.014596327	-0.247559891	0.360660039	0.127696475	-0.232963564	8	0.072620731	0.816496581	0.816496581	0.816496581	0.816496581	0.816496581	0.816496581	24	40	0	P_204976	P_204976	P_204976	chr1	173807266	173807326	-	CENPL	centromere protein L [Source:HGNC Symbol;Acc:HGNC:17879]	ENSG00000120334	protein_coding	exon	coding exon	1	3950	1:173807266:173807326	ENSG00000120334	CENPL	8.405511548	0.006441809	-5.726242864	-0.222733359	-3.77765226	5.503509505	1.948590605	-3.5549189	9.961963646	0.00019408	0.828243406	0.003640335	0.000264064	0.080045487	0.005256355	0.839882771	-6.156001979	-2.232863099	0.403980389	-4.59584917	-2.630542315	15.63886578	0.000425467

SFSWAP	-0.098365332	-0.071815141	-0.067265742	0.026550191	0.03109959	0.004549399	8	0.031087324	0.816496581	0.816496581	0.816496581	0.816496581	0.816496581	0.816496581	24	41	0	P_309323	P_309323	P_309323	chr12	131714010	131714070	+	SFSWAP	"splicing factor, suppressor of white-apricot family [Source:HGNC Symbol;Acc:HGNC:10790]"	ENSG00000061936	protein_coding	exon	non-coding exon	1	3951	12:131714010:131714070	ENSG00000061936	SFSWAP	7.375325489	0.002982773	-2.205858069	-1.610465857	-1.508444864	0.595392212	0.697413205	0.102020993	9.961963646	0.052022541	0.138490193	0.162482389	0.564861024	0.501495812	0.920764324	-4.840267596	-4.973224546	-5.928849588	-6.95914317	-6.044303909	-7.065695433	1.764350226	0.217502951

POLG	-0.176111234	0.067440848	-0.155114628	0.243552083	0.020996606	-0.222555476	8	0.078004322	0.816496581	0.816496581	0.816496581	0.816496581	0.816496581	0.816496581	24	43	0	A_23_P3355	A_23_P3355	A_23_P3355	chr15	89316481	89316541	-	POLG	"polymerase (DNA directed), gamma [Source:HGNC Symbol;Acc:HGNC:9179]"	ENSG00000140521	protein_coding	3'UTR	3'UTR	1	3953	15:89316481:89316541	ENSG00000140521	POLG	8.853996957	0.007093009	-2.561046302	0.98073888	-2.255709276	3.541785183	0.305337027	-3.236448156	9.961963646	0.028400912	0.34993875	0.047811183	0.005372368	0.766393935	0.008966084	-4.255214767	-5.698997232	-4.806302482	-2.72406414	-6.247335366	-3.173580849	5.953516816	0.013578124

SAFB	-0.121794212	-0.151055487	-0.137515927	-0.029261275	-0.015721715	0.01353956	8	0.107735118	0.816496581	0.816496581	0.816496581	0.816496581	0.816496581	0.816496581	24	44	0	P_124293	P_124293	P_124293	chr19	5668314	5668374	+	SAFB	scaffold attachment factor B [Source:HGNC Symbol;Acc:HGNC:10520]	ENSG00000160633	protein_coding	3'UTR;exon	multiple Ref	1	3954	19:5668314:5668374	ENSG00000160633	SAFB	8.464941005	0.011527622	-1.38932041	-1.723107101	-1.568659799	-0.333786691	-0.179339389	0.154447302	9.961963646	0.195004371	0.115708395	0.147914906	0.745460243	0.861266348	0.880341265	-6.036319879	-4.820252679	-5.847238072	-7.08902495	-6.279606864	-7.058362706	1.254572165	0.341788672

SUCLG2-AS1	0.198378458	-0.451720497	0.071767482	-0.650098955	-0.126610976	0.523487978	8	0.110901573	0.816496581	0.816496581	0.816496581	0.816496581	0.816496581	0.816496581	24	45	0	P_244866	P_244866	P_244866	chr3	67655042	67655102	+	SUCLG2-AS1	SUCLG2 antisense RNA 1 (head to head) [Source:HGNC Symbol;Acc:HGNC:49643]	ENSG00000241316	lincRNA	exon	non-coding exon	1	3955	3:67655042:67655102	ENSG00000241316	SUCLG2-AS1	6.319571209	0.012083579	2.210254812	-5.032892235	0.799605075	-7.243147046	-1.410649737	5.83249731	9.961963646	0.051637153	0.000517957	0.442588249	2.84E-05	0.188802521	0.000167973	-4.833182116	0.196602718	-6.708782149	2.737361997	-5.334815922	0.936990574	19.88762259	0.000157819

PHTF1	0.367501766	0.059860797	0.474044722	-0.307640969	0.106542956	0.414183926	8	0.21245425	0.816496581	0.816496581	0.816496581	0.816496581	0.816496581	0.816496581	24	47	0	P_203485	P_203485	P_203485	chr1	113706593	113706653	-	PHTF1	putative homeodomain transcription factor 1 [Source:HGNC Symbol;Acc:HGNC:8939]	ENSG00000116793	protein_coding	exon	coding exon	1	3957	1:113706593:113706653	ENSG00000116793	PHTF1	4.065669449	0.038454002	2.295272678	0.373867186	2.960698424	-1.921405492	0.665425746	2.586831238	9.961963646	0.044705814	0.716337118	0.014327258	0.083726011	0.520886314	0.027173965	-4.695284834	-6.108934704	-3.623017455	-5.4340424	-6.066423392	-4.280896308	4.16667914	0.037355693

ZGRF1	-0.327663298	0.042090616	-0.216128255	0.369753914	0.111535042	-0.258218872	8	0.043043078	0.816496581	0.816496581	0.816496581	0.816496581	0.816496581	0.816496581	24	50	0	P_250507	P_250507	P_250507	chr4	112617441	112617501	-	ZGRF1	"zinc finger, GRF-type containing 1 [Source:HGNC Symbol;Acc:HGNC:25654]"	ENSG00000138658	protein_coding	3'UTR;exon	multiple Ref	1	3960	4:112617441:112617501	ENSG00000138658	ZGRF1	6.157823376	0.003694508	-6.602297675	0.848110792	-4.354906665	7.450408467	2.24739101	-5.203017457	9.961963646	6.17E-05	0.41628866	0.001445427	2.23E-05	0.048490209	0.000404521	2.027345595	-5.816506882	-1.281203745	2.988630705	-4.140480966	0.021155449	25.15093032	5.77E-05

CA3	0.078242354	0.311099422	0.095530908	0.232857068	0.017288554	-0.215568514	8	0.278496119	0.816496581	0.816496581	0.816496581	0.816496581	0.816496581	0.816496581	24	54	0	A_14_P137899	A_14_P137899	A_14_P137899	chr8	85448262	85448322	+	CA3	"carbonic anhydrase III, muscle specific [Source:HGNC Symbol;Acc:HGNC:1374]"	ENSG00000164879	protein_coding	3'UTR	3'UTR	1	3964	8:85448262:85448322	ENSG00000164879	CA3	3.271092276	0.064491664	0.377342522	1.500351587	0.460720719	1.123009065	0.083378197	-1.039630869	9.961963646	0.713833418	0.164532937	0.654892611	0.287768866	0.93520246	0.323086042	-6.921215179	-5.11703063	-6.932428024	-6.500464877	-6.292988954	-6.511015176	0.825773245	0.509274656

TCERG1	-0.124193028	-0.07160746	-0.079525574	0.052585568	0.044667455	-0.007918114	8	0.054559119	0.816496581	0.816496581	0.816496581	0.816496581	0.816496581	0.816496581	24	55	0	P_312834	P_312834	P_312834	chr5	146498794	146498854	+	TCERG1	transcription elongation regulator 1 [Source:HGNC Symbol;Acc:HGNC:15630]	ENSG00000113649	protein_coding	intron;promoter	multiple non-Ref	0	3965	5:146498794:146498854	ENSG00000113649	TCERG1	8.616469029	0.004597134	-2.243361431	-1.293481738	-1.436510627	0.949879693	0.806850804	-0.143028889	9.961963646	0.048822495	0.225031408	0.181509168	0.364648251	0.438590952	0.889118995	-4.779680798	-5.369551178	-6.023699563	-6.677399619	-5.961627011	-7.060214191	1.723844202	0.225219162

CCKBR	-0.258891682	0.210944852	0.233927855	0.469836534	0.492819537	0.022983003	8	0.357681638	0.816496581	0.816496581	0.816496581	0.816496581	0.816496581	0.816496581	24	56	0	P_107790	P_107790	P_107790	chr11	6269688	6269748	+	CCKBR	cholecystokinin B receptor [Source:HGNC Symbol;Acc:HGNC:1571]	ENSG00000110148	protein_coding	3'UTR;exon	multiple Ref	1	3966	11:6269688:6269748	ENSG00000110148	CCKBR	3.424890069	0.104946392	-0.978768472	0.797500207	0.884389978	1.776268679	1.86315845	0.086889771	9.961963646	0.350864805	0.443753959	0.397328377	0.106183131	0.092149836	0.932480825	-6.499419291	-5.857499702	-6.636039982	-5.652043193	-4.721243076	-7.067255598	1.501696599	0.273570198

PJA2	0.112424413	0.106791019	0.091997946	-0.005633394	-0.020426467	-0.014793073	8	0.059501084	0.816496581	0.816496581	0.816496581	0.816496581	0.816496581	0.816496581	24	58	0	P_254243	P_254243	P_254243	chr5	109368560	109368620	-	PJA2	"praja ring finger 2, E3 ubiquitin protein ligase [Source:HGNC Symbol;Acc:HGNC:17481]"	ENSG00000198961	protein_coding	exon	coding exon	1	3968	5:109368560:109368620	ENSG00000198961	PJA2	8.003878221	0.005049801	1.937622238	1.84053132	1.585574359	-0.097090918	-0.352047879	-0.254956961	9.961963646	0.081511855	0.095629387	0.144035407	0.92457977	0.732135794	0.803942517	-5.262330053	-4.6551997	-5.82396622	-7.144460398	-6.231184666	-7.035983303	1.642391488	0.241710412

PMP2	0.196963593	0.195084605	-0.130860687	-0.001878988	-0.327824281	-0.325945293	8	0.164651045	0.816496581	0.816496581	0.816496581	0.816496581	0.816496581	0.816496581	24	60	0	A_23_P31615	A_23_P31615	A_23_P31615	chr8	81441924	81441984	-	PMP2	peripheral myelin protein 2 [Source:HGNC Symbol;Acc:HGNC:9117]	ENSG00000147588	protein_coding	3'UTR	3'UTR	1	3970	8:81441924:81441984	ENSG00000147588	PMP2	3.697939247	0.023977465	1.557865475	1.543003794	-1.035030604	-0.014861682	-2.592896079	-2.578034398	9.961963646	0.150438617	0.153975257	0.325126302	0.988435957	0.026893114	0.027586518	-5.814263349	-5.062042774	-6.491666161	-7.149492279	-3.591622365	-4.29569416	3.208114367	0.070614681

ACSS3	-0.029460193	0.047750769	0.087869506	0.077210962	0.117329699	0.040118737	8	0.204968365	0.816496581	0.816496581	0.816496581	0.816496581	0.816496581	0.816496581	24	61	0	P_216448	P_216448	P_216448	chr12	81199383	81199443	+	ACSS3	acyl-CoA synthetase short-chain family member 3 [Source:HGNC Symbol;Acc:HGNC:24723]	ENSG00000111058	protein_coding	exon	coding exon	1	3971	12:81199383:81199443	ENSG00000111058	ACSS3	3.829794879	0.035944635	-0.190311127	0.308467175	0.567631871	0.498778302	0.757942997	0.259164696	9.961963646	0.852887506	0.764080678	0.582850397	0.62876433	0.466036721	0.800784823	-6.978632393	-6.132173988	-6.873966985	-7.015244828	-5.999895841	-7.034809335	0.223999997	0.877553731

PRND	-0.024741528	-0.42863448	-0.31597611	-0.403892951	-0.291234582	0.112658369	8	0.472332621	0.816496581	0.816496581	0.816496581	0.816496581	0.816496581	0.816496581	24	62	0	A_24_P927041	A_24_P927041	A_24_P927041	chr20	4728200	4728260	+	PRND	prion protein 2 (dublet) [Source:HGNC Symbol;Acc:HGNC:15748]	ENSG00000171864	protein_coding	exon	coding exon	1	3972	20:4728200:4728260	ENSG00000171864	PRND	3.578068807	0.181366627	-0.071153041	-1.232690489	-0.908701386	-1.161537448	-0.837548345	0.323989103	9.961963646	0.944684127	0.245989614	0.384964294	0.272497635	0.421923284	0.752645961	-6.995598319	-5.438863886	-6.614024512	-6.457939065	-5.936541244	-7.014335763	0.750992036	0.546416718

IREB2	0.064612724	-0.043451528	0.121697324	-0.108064253	0.057084599	0.165148852	8	0.144925171	0.816496581	0.816496581	0.816496581	0.816496581	0.816496581	0.816496581	24	63	0	P_224674	P_224674	P_224674	chr15	78494203	78494263	+	IREB2	iron-responsive element binding protein 2 [Source:HGNC Symbol;Acc:HGNC:6115]	ENSG00000136381	protein_coding	3'UTR;exon	multiple Ref	1	3973	15:78494203:78494263	ENSG00000136381	IREB2	8.709346439	0.019073483	0.572992157	-0.385332533	1.079224142	-0.958324691	0.506231985	1.464556676	9.961963646	0.579352794	0.7080908	0.305924267	0.360578985	0.623707886	0.173873858	-6.8221113	-6.104414123	-6.445826224	-6.669329314	-6.162159291	-6.010916249	0.826851536	0.508758116

SGSM3	-0.361036912	0.177597881	-0.108794552	0.538634794	0.25224236	-0.286392434	8	0.108586482	0.816496581	0.816496581	0.816496581	0.816496581	0.816496581	0.816496581	24	64	0	A_24_P153399	A_24_P153399	A_24_P153399	chr22	40409939	40409999	+	SGSM3	small G protein signaling modulator 3 [Source:HGNC Symbol;Acc:HGNC:25228]	ENSG00000100359	protein_coding	3'UTR;exon	multiple Ref	1	3974	22:40409939:40409999	ENSG00000100359	SGSM3	8.908816903	0.011675519	-4.092221522	2.013007112	-1.233146512	6.105228634	2.85907501	-3.246153624	9.961963646	0.002188755	0.071921651	0.245826681	0.00011675	0.017045386	0.008820199	-1.667850395	-4.403954134	-6.274694887	1.258331608	-3.160429875	-3.156975609	13.05079415	0.000871753

MAGI3	0.228203357	-0.043429389	0.244110315	-0.271632746	0.015906958	0.287539704	8	0.047868002	0.816496581	0.816496581	0.816496581	0.816496581	0.816496581	0.816496581	24	65	0	P_102772	P_102772	P_102772	chr1	113685782	113685842	+	MAGI3	"membrane associated guanylate kinase, WW and PDZ domain containing 3 [Source:HGNC Symbol;Acc:HGNC:29647]"	ENSG00000081026	protein_coding	3'UTR	3'UTR	1	3975	1:113685782:113685842	ENSG00000081026	MAGI3	7.620002698	0.004046759	4.393533723	-0.836133554	4.699785812	-5.229667277	0.306252089	5.535919366	9.961963646	0.001360955	0.422681927	0.000850586	0.000389303	0.765717431	0.000252385	-1.178094124	-5.826405061	-0.731876821	-0.002017205	-6.24704056	0.512637303	16.696643	0.000326122

DNHD1	0.02867227	0.070219066	0.068058005	0.041546796	0.039385735	-0.002161061	8	0.065295685	0.816496581	0.816496581	0.816496581	0.816496581	0.816496581	0.816496581	24	66	0	P_210717	P_210717	P_210717	chr11	6528899	6528959	+	DNHD1	dynein heavy chain domain 1 [Source:HGNC Symbol;Acc:HGNC:26532]	ENSG00000179532	protein_coding	exon	coding exon	1	3976	11:6528899:6528959	ENSG00000179532	DNHD1	4.572984758	0.005630528	0.467986292	1.146109487	1.110836838	0.678123195	0.642850545	-0.035272649	9.961963646	0.649865066	0.278534263	0.292730013	0.51313609	0.534836423	0.972559101	-6.880152996	-5.533363171	-6.412109778	-6.903801447	-6.081463814	-7.070698879	0.606814776	0.625576325

SEMA3C	0.242487865	-0.330524942	0.319861672	-0.573012807	0.077373807	0.650386614	8	0.101841246	0.816496581	0.816496581	0.816496581	0.816496581	0.816496581	0.816496581	24	69	0	A_16_P17997629	A_16_P17997629	A_16_P17997629	chr7	80908035	80908095	+	SEMA3C	"sema domain, immunoglobulin domain (Ig), short basic domain, secreted, (semaphorin) 3C [Source:HGNC Symbol;Acc:HGNC:10725]"	ENSG00000075223	protein_coding	intron	intron antisense	0	3979	7:80908035:80908095	ENSG00000075223	SEMA3C	6.468486632	0.010535676	2.893373092	-3.943834352	3.81659987	-6.837207444	0.923226778	7.760434222	9.961963646	0.016074096	0.002778094	0.003415837	4.62E-05	0.37770807	1.57E-05	-3.692714788	-1.373924495	-2.167562599	2.229476874	-5.862297057	3.400319447	24.38599407	6.60E-05

FSTL5	0.00568233	0.077988599	0.063214698	0.07230627	0.057532368	-0.014773901	8	0.105461834	0.816496581	0.816496581	0.816496581	0.816496581	0.816496581	0.816496581	24	73	0	A_24_P251734	A_24_P251734	A_24_P251734	chr4	161383966	161384026	-	FSTL5	follistatin-like 5 [Source:HGNC Symbol;Acc:HGNC:21386]	ENSG00000168843	protein_coding	3'UTR	3'UTR	1	3983	4:161383966:161384026	ENSG00000168843	FSTL5	3.515040718	0.011138416	0.065941732	0.905034298	0.733587605	0.839092566	0.667645873	-0.171446693	9.961963646	0.948728798	0.386811695	0.480108048	0.421096291	0.519526185	0.867305059	-6.995988	-5.767825524	-6.760951049	-6.777653331	-6.064918664	-7.055344309	0.423467913	0.74039063

MINDY2	-0.04004106	0.071193445	0.059564834	0.111234505	0.099605894	-0.011628611	8	0.080805088	0.816496581	0.816496581	0.816496581	0.816496581	0.816496581	0.816496581	24	74	0	P_223854	P_223854	P_223854	chr15	58772111	58772171	+	FAM63B	"family with sequence similarity 63, member B [Source:HGNC Symbol;Acc:HGNC:26954]"	ENSG00000128923	protein_coding	exon	coding exon	1	3984	15:58772111:58772171	ENSG00000128923	MINDY2	9.543518217	0.007450198	-0.568155586	1.010186888	0.845184753	1.578342474	1.413340339	-0.165002135	9.961963646	0.582508169	0.336312194	0.417844419	0.145682955	0.188032235	0.87224247	-6.825027017	-5.671003108	-6.670464696	-5.93454593	-5.331470739	-7.056525459	1.095589528	0.395658104

MACROH2A2	0.302690401	-0.027025819	0.198148741	-0.32971622	-0.104541659	0.225174561	8	0.072679136	0.816496581	0.816496581	0.816496581	0.816496581	0.816496581	0.816496581	24	75	0	A_16_P18968356	A_16_P18968356	A_16_P18968356	chr10	70112147	70112207	+	H2AFY2	"H2A histone family, member Y2 [Source:HGNC Symbol;Acc:HGNC:14453]"	ENSG00000099284	protein_coding	3'UTR	3'UTR	1	3985	10:70112147:70112207	ENSG00000099284	MACROH2A2	9.768208753	0.006448624	4.616477711	-0.412183842	3.022062297	-5.028661553	-1.594415414	3.434246139	9.961963646	0.000965326	0.688934157	0.012903219	0.000521178	0.142043688	0.006428076	-0.823122727	-6.093310057	-3.518099713	-0.306863612	-5.097280031	-2.835754597	11.70637776	0.001323597

ARHGAP26	-0.369711063	0.150715896	-0.285466081	0.520426959	0.084244982	-0.436181978	8	0.041457604	0.816496581	0.816496581	0.816496581	0.816496581	0.816496581	0.816496581	24	76	0	P_255188	P_255188	P_255188	chr5	142818155	142818215	-	ARHGAP26	Rho GTPase activating protein 26 [Source:HGNC Symbol;Acc:HGNC:17073]	ENSG00000145819	protein_coding	intron	intron antisense	0	3986	5:142818155:142818215	ENSG00000145819	ARHGAP26	6.4740008	0.00358692	-7.560442487	3.082079443	-5.837666509	10.64252193	1.722775978	-8.919745953	9.961963646	1.97E-05	0.011649644	0.000166803	9.25E-07	0.115770085	4.60E-06	3.20663976	-2.726177811	0.96210587	6.27221115	-4.921469245	4.66250183	49.42182542	2.74E-06

ALG14	-0.004160274	0.121510531	0.032860462	0.125670805	0.037020736	-0.08865007	8	0.100583201	0.816496581	0.816496581	0.816496581	0.816496581	0.816496581	0.816496581	24	77	0	P_102444	P_102444	P_102444	chr1	94982871	94982931	-	ALG14	"ALG14, UDP-N-acetylglucosaminyltransferase subunit [Source:HGNC Symbol;Acc:HGNC:28287]"	ENSG00000172339	protein_coding	3'UTR	3'UTR	1	3987	1:94982871:94982931	ENSG00000172339	ALG14	7.46297145	0.010331171	-0.050129435	1.464147328	0.395953807	1.514276763	0.446083241	-1.068193522	9.961963646	0.961010107	0.173983278	0.700486713	0.16101863	0.66507622	0.310634036	-6.996989189	-5.162962151	-6.962173444	-6.021792491	-6.1919229	-6.481448018	0.989346925	0.436800778

MLLT10	-0.115426752	-0.137071861	-0.188734227	-0.021645109	-0.073307474	-0.051662365	8	0.101389954	0.816496581	0.816496581	0.816496581	0.816496581	0.816496581	0.816496581	24	79	0	P_105630	P_105630	P_105630	chr10	21742033	21742093	+	MLLT10	"myeloid/lymphoid or mixed-lineage leukemia (trithorax homolog, Drosophila); translocated to, 10 [Source:HGNC Symbol;Acc:HGNC:16063]"	ENSG00000078403	protein_coding	3'UTR	3'UTR	1	3989	10:21742033:21742093	ENSG00000078403	MLLT10	7.133973978	0.010462023	-1.382115318	-1.641292984	-2.259896083	-0.259177666	-0.877780764	-0.618603098	9.961963646	0.197138028	0.131886471	0.047472905	0.800775095	0.400737057	0.55005985	-6.045441178	-4.931920854	-4.799498479	-7.113002404	-5.90244377	-6.866267596	1.821926274	0.207054365

HIVEP2	0.409804753	-0.008130266	0.300860779	-0.417935019	-0.108943975	0.308991045	8	0.06967469	0.816496581	0.816496581	0.816496581	0.816496581	0.816496581	0.816496581	24	80	0	P_143743	P_143743	P_143743	chr6	142753106	142753166	-	HIVEP2	human immunodeficiency virus type I enhancer binding protein 2 [Source:HGNC Symbol;Acc:HGNC:4921]	ENSG00000010818	protein_coding	exon	coding exon	1	3990	6:142753106:142753166	ENSG00000010818	HIVEP2	7.121858402	0.006105162	6.423533785	-0.12743883	4.715878381	-6.550972615	-1.707655404	4.843317211	9.961963646	7.73E-05	0.901128815	0.000830141	6.58E-05	0.118619123	0.000685561	1.793809124	-6.173533074	-0.706633777	1.858390833	-4.942560945	-0.527797566	22.1344311	0.000100195

NFYC-AS1	0.126748307	-0.333480028	0.007890981	-0.460228335	-0.118857326	0.341371008	8	0.127318494	0.816496581	0.816496581	0.816496581	0.816496581	0.816496581	0.816496581	24	83	0	A_32_P856141	A_32_P856141	A_32_P856141	chr1	40690665	40690725	-	NFYC-AS1	NFYC antisense RNA 1 [Source:HGNC Symbol;Acc:HGNC:49451]	ENSG00000272145	antisense	exon	non-coding exon	1	3993	1:40690665:40690725	ENSG00000272145	NFYC-AS1	8.346335652	0.015224197	1.258115756	-3.310154481	0.078326625	-4.568270237	-1.17978913	3.388481106	9.961963646	0.237038227	0.007917337	0.939119191	0.001039133	0.26549029	0.006940432	-6.197236897	-2.362407351	-7.043771222	-1.026424219	-5.605278707	-2.913783731	7.714818759	0.005902643

SYT7	-0.019417645	0.292901677	0.152667337	0.312319322	0.172084981	-0.14023434	8	0.071596468	0.816496581	0.816496581	0.816496581	0.816496581	0.816496581	0.816496581	24	89	0	P_108767	P_108767	P_108767	chr11	61515703	61515763	-	SYT7	synaptotagmin VII [Source:HGNC Symbol;Acc:HGNC:11514]	ENSG00000011347	protein_coding	3'UTR	3'UTR	1	3999	11:61515703:61515763	ENSG00000011347	SYT7	6.52619661	0.006323185	-0.29907095	4.511277492	2.351385378	4.810348442	2.650456328	-2.159892114	9.961963646	0.771031944	0.001134184	0.040635625	0.000720193	0.024367383	0.056221086	-6.949771323	-0.532260973	-4.649873337	-0.644429153	-3.498765813	-4.984230063	10.13326434	0.002265358

ZNFX1	0.023987448	0.074788713	0.129633131	0.050801265	0.105645683	0.054844418	8	0.061105558	0.816496581	0.816496581	0.816496581	0.816496581	0.816496581	0.816496581	24	91	0	P_241077	P_241077	P_241077	chr20	49251526	49251586	-	ZNFX1	"zinc finger, NFX1-type containing 1 [Source:HGNC Symbol;Acc:HGNC:29271]"	ENSG00000124201	protein_coding	exon	coding exon	1	4001	20:49251526:49251586	ENSG00000124201	ZNFX1	7.642916458	0.005205201	0.407202973	1.269588425	2.200609106	0.862385452	1.793406133	0.931020681	9.961963646	0.692470803	0.233087012	0.052486262	0.408755672	0.103268746	0.373854979	-6.908627638	-5.397076048	-4.895463929	-6.757456814	-4.821692176	-6.617459789	1.907860534	0.192521211

CASC3	0.015220637	-0.067303415	-0.030881102	-0.082524052	-0.046101738	0.036422314	8	0.045656224	0.816496581	0.816496581	0.816496581	0.816496581	0.816496581	0.816496581	24	92	0	P_228626	P_228626	P_228626	chr17	40162839	40162899	+	CASC3	cancer susceptibility candidate 3 [Source:HGNC Symbol;Acc:HGNC:17040]	ENSG00000108349	protein_coding	exon	coding exon	1	4002	17:40162839:40162899	ENSG00000108349	CASC3	8.020937947	0.003880644	0.299244764	-1.323216312	-0.607136756	-1.622461076	-0.906381521	0.716079556	9.961963646	0.770903162	0.21532885	0.557344078	0.135886354	0.386132268	0.490387162	-6.949715083	-5.334798817	-6.849478075	-5.873211614	-5.877377301	-6.798271034	1.029290202	0.420815157

ARRDC1	-0.128137726	0.085868942	-0.158844379	0.214006668	-0.030706653	-0.244713321	8	0.103824604	0.816496581	0.816496581	0.816496581	0.816496581	0.816496581	0.816496581	24	93	0	P_150348	P_150348	P_150348	chr9	137613329	137613389	-	ARRDC1	arrestin domain containing 1 [Source:HGNC Symbol;Acc:HGNC:28633]	ENSG00000197070	protein_coding	3'UTR;exon;intron;promoter	multiple non-Ref	0	4003	9:137613329:137613389	ENSG00000197070	ARRDC1	8.306426074	0.010863249	-1.50571498	1.009024871	-1.866541332	2.51473985	-0.360826352	-2.875566202	9.961963646	0.163171544	0.336842328	0.091639816	0.030743871	0.725763267	0.016571179	-5.884688393	-5.672120444	-5.417556243	-4.476560219	-6.227899463	-3.791048849	3.582046415	0.054579233

NEK3	-0.10338037	0.200341374	-0.090664272	0.303721743	0.012716097	-0.291005646	8	0.074588031	0.816496581	0.816496581	0.816496581	0.816496581	0.816496581	0.816496581	24	94	0	P_113871	P_113871	P_113871	chr13	52133721	52133781	-	NEK3	NIMA-related kinase 3 [Source:HGNC Symbol;Acc:HGNC:7746]	ENSG00000136098	protein_coding	3'UTR;exon	multiple Ref	1	4004	13:52133721:52133781	ENSG00000136098	NEK3	6.095697049	0.006674377	-1.549809596	3.003384336	-1.359178341	4.553193932	0.190631255	-4.362562677	9.961963646	0.15234671	0.01332074	0.204062175	0.001063426	0.852643314	0.001428254	-5.825237921	-2.85212862	-6.122253194	-1.050474655	-6.27739269	-1.289471237	8.844910231	0.003688295

CENPE	-0.326350969	-0.019510702	-0.156108489	0.306840267	0.17024248	-0.136597787	8	0.068392505	0.816496581	0.816496581	0.816496581	0.816496581	0.816496581	0.816496581	24	95	0	A_23_P253524	NM_001813	A_23_P253524	chr4	103106181	103106241	-	CENPE	"centromere protein E, 312kDa [Source:HGNC Symbol;Acc:HGNC:1856]"	ENSG00000138778	protein_coding	"3'UTR,exon"	multiple Ref	1	4005	4:103106181:103106241	ENSG00000138778	CENPE	9.790259388	0.005962999	-5.176046308	-0.309446911	-2.47593801	4.866599397	2.700108298	-2.166491099	9.961963646	0.000420572	0.763357128	0.032853615	0.000662175	0.022379705	0.055598904	0.037605887	-6.131857997	-4.443605972	-0.556814236	-3.418458781	-4.973653115	11.44759	0.001440359

AFG3L2	-0.058011195	0.008058889	-0.074952599	0.066070084	-0.016941405	-0.083011489	8	0.129282029	0.816496581	0.816496581	0.816496581	0.816496581	0.816496581	0.816496581	24	96	0	P_230677	P_230677	P_230677	chr18	12337340	12337400	-	AFG3L2	AFG3-like AAA ATPase 2 [Source:HGNC Symbol;Acc:HGNC:315]	ENSG00000141385	protein_coding	exon	coding exon	1	4006	18:12337340:12337400	ENSG00000141385	AFG3L2	6.830124656	0.015628811	-0.568321997	0.07895104	-0.734292943	0.647273036	-0.165970946	-0.813243982	9.961963646	0.582399449	0.938634958	0.4796968	0.532086501	0.871499855	0.43508397	-6.824927081	-6.178802493	-6.760414853	-6.925211794	-6.282054611	-6.721640926	0.329380633	0.804352367

YWHAEP7	-0.661676633	-0.859454546	-0.132210082	-0.197777912	0.529466551	0.727244463	8	0.57033294	0.816496581	0.816496581	0.816496581	0.816496581	0.816496581	0.816496581	24	98	0	P_121221	P_121221	P_121221	chr17	37843537	37843597	-	YWHAEP7	"tyrosine 3-monooxygenase/tryptophan 5-monooxygenase activation protein, epsilon pseudogene 7 [Source:HGNC Symbol;Acc:HGNC:49437]"	ENSG00000276715	transcribed_unprocessed_pseudogene	exon	non-coding exon	1	4008	17:37843537:37843597	ENSG00000276715	YWHAEP7	2.791836052	0.263423989	-1.578933235	-2.050882982	-0.315487781	-0.471949746	1.263445454	1.735395201	9.961963646	0.145547747	0.067515092	0.758901159	0.647130215	0.235195909	0.11344004	-5.785400915	-4.347565227	-6.993036611	-7.029155758	-5.511210474	-5.635330999	1.938218531	0.187672524

NCKAP1	-0.083805625	-0.017183543	-0.084587209	0.066622082	-0.000781584	-0.067403666	8	0.071573807	0.816496581	0.816496581	0.816496581	0.816496581	0.816496581	0.816496581	24	99	0	P_238183	P_238183	P_238183	chr2	182982820	182982880	-	NCKAP1	NCK-associated protein 1 [Source:HGNC Symbol;Acc:HGNC:7666]	ENSG00000061676	protein_coding	exon	coding exon	1	4009	2:182982820:182982880	ENSG00000061676	NCKAP1	8.579668084	0.00632058	-1.291041929	-0.264715816	-1.303082385	1.026326113	-0.012040456	-1.038366569	9.961963646	0.225843328	0.79662475	0.221859872	0.329013305	0.990631054	0.323645808	-6.157909898	-6.145280882	-6.191399671	-6.602210185	-6.296608149	-6.512309116	0.927763803	0.462728529

CCAR1	-0.057156858	-0.18705597	-0.047097402	-0.129899112	0.010059456	0.139958568	8	0.04955695	0.816496581	0.816496581	0.816496581	0.816496581	0.816496581	0.816496581	24	102	0	P_106120	P_106120	P_106120	chr10	68757161	68757221	+	CCAR1;MIR1254-1	cell division cycle and apoptosis regulator 1 [Source:HGNC Symbol;Acc:HGNC:24236];microRNA 1254-1 [Source:HGNC Symbol;Acc:HGNC:35319]	ENSG00000060339	protein_coding;miRNA	intron;promoter	multiple non-Ref	0	4012	10:68757161:68757221	ENSG00000060339	CCAR1	9.698933429	0.004178899	-1.082886799	-3.543939401	-0.892301587	-2.461052602	0.190585212	2.651637815	9.961963646	0.304372569	0.005353158	0.393274644	0.033700353	0.852678435	0.024318097	-6.393688414	-1.993021842	-6.628931241	-4.566325306	-6.27740199	-4.171602624	4.608773026	0.028530987

TACR2	-0.021211696	-0.040325963	-0.039423452	-0.019114267	-0.018211756	0.000902511	8	0.162581442	0.816496581	0.816496581	0.816496581	0.816496581	0.816496581	0.816496581	24	103	0	P_106154	P_106154	P_106154	chr10	69404250	69404310	-	TACR2	tachykinin receptor 2 [Source:HGNC Symbol;Acc:HGNC:11527]	ENSG00000075073	protein_coding	3'UTR	3'UTR	1	4013	10:69404250:69404310	ENSG00000075073	TACR2	4.893854087	0.023433604	-0.169707676	-0.322634526	-0.315413838	-0.152926849	-0.145706162	0.007220688	9.961963646	0.868636803	0.753641403	0.758955646	0.881509136	0.887059423	0.994381327	-6.982666862	-6.127509146	-6.993061833	-7.136838782	-6.285405567	-7.071350042	0.046174778	0.986051659

HES4	-0.110530066	0.119834931	-0.151231139	0.230364997	-0.040701073	-0.27106607	8	0.046946508	0.816496581	0.816496581	0.816496581	0.816496581	0.816496581	0.816496581	24	107	0	P_100021	P_100021	P_100021	chr1	998964	999024	-	HES4	hes family bHLH transcription factor 4 [Source:HGNC Symbol;Acc:HGNC:24149]	ENSG00000188290	protein_coding	3'UTR;exon	multiple Ref	1	4017	1:998964:999024	ENSG00000188290	HES4	14.3047815	0.003976596	-2.146694958	2.327412364	-2.937183846	4.474107322	-0.790488888	-5.26459621	9.961963646	0.057485482	0.042328302	0.01491437	0.001201108	0.447651546	0.000370278	-4.935136835	-3.925449122	-3.663195091	-1.177125223	-5.974663766	0.113275729	11.16844646	0.001580449

LXN	-0.245148361	0.038310755	-0.12771659	0.283459116	0.117431771	-0.166027345	8	0.128011244	0.816496581	0.816496581	0.816496581	0.816496581	0.816496581	0.816496581	24	108	0	P_246943	P_246943	P_246943	chr3	158669432	158669492	-	LXN	latexin [Source:HGNC Symbol;Acc:HGNC:13347]	ENSG00000079257	protein_coding	exon	coding exon	1	4018	3:158669432:158669492	ENSG00000079257	LXN	4.509336443	0.015366241	-2.422092941	0.378514498	-1.261854044	2.800607439	1.160238897	-1.640368542	9.961963646	0.03601959	0.712989905	0.235744782	0.018839766	0.273001714	0.132080339	-4.486787039	-6.10711832	-6.240904198	-3.99267885	-5.626574246	-5.771314272	3.247074725	0.068704442

WDFY1	-0.040284496	0.112224424	-0.033351655	0.15250892	0.006932841	-0.145576078	8	0.088790087	0.816496581	0.816496581	0.816496581	0.816496581	0.816496581	0.816496581	24	110	0	P_239462	P_239462	P_239462	chr2	223912253	223912313	-	WDFY1	WD repeat and FYVE domain containing 1 [Source:HGNC Symbol;Acc:HGNC:20451]	ENSG00000085449	protein_coding	exon	coding exon	1	4020	2:223912253:223912313	ENSG00000085449	WDFY1	6.996051311	0.008537709	-0.533965132	1.487518412	-0.44207133	2.021483543	0.091893802	-1.929589741	9.961963646	0.6050752	0.167830538	0.667880038	0.070912462	0.928604054	0.082601637	-6.844976006	-5.133391281	-6.941436884	-5.279195308	-6.292195722	-5.345366361	1.751902267	0.219841292

HTRA1	0.600842471	0.060152544	0.494788832	-0.540689927	-0.106053639	0.434636289	8	0.043385738	0.816496581	0.816496581	0.816496581	0.816496581	0.816496581	0.816496581	24	113	0	P_107268	P_107268	P_107268	chr10	122514299	122514359	+	HTRA1	HtrA serine peptidase 1 [Source:HGNC Symbol;Acc:HGNC:9476]	ENSG00000166033	protein_coding	exon	coding exon	1	4023	10:122514299:122514359	ENSG00000166033	HTRA1	6.634492006	0.003718291	12.06797887	1.208169628	9.937881325	-10.85980924	-2.130097544	8.729711697	9.961963646	2.87E-07	0.254880097	1.73E-06	7.67E-07	0.059114384	5.58E-06	7.447251855	-5.46614131	5.663722552	6.461669666	-4.321966532	4.465567824	74.08965194	4.09E-07

CSPG4	0.016389275	-0.113648923	-0.041393336	-0.130038198	-0.057782611	0.072255587	8	0.232737171	0.816496581	0.816496581	0.816496581	0.816496581	0.816496581	0.816496581	24	116	0	P_117183	P_117183	P_117183	chr15	75675729	75675789	-	CSPG4	chondroitin sulfate proteoglycan 4 [Source:HGNC Symbol;Acc:HGNC:2466]	ENSG00000173546	protein_coding	exon	coding exon	1	4026	15:75675729:75675789	ENSG00000173546	CSPG4	4.410752559	0.04570541	0.093890484	-0.651069199	-0.237133142	-0.744959683	-0.331023626	0.413936057	9.961963646	0.927057706	0.529732651	0.817358818	0.473504685	0.747484165	0.687691866	-6.993551955	-5.963440846	-7.01649865	-6.85432268	-6.238731697	-6.978574718	0.22080388	0.87975654

ARHGAP18	0.173246505	0.064674953	0.061548616	-0.108571552	-0.111697889	-0.003126337	8	0.029609013	0.816496581	0.816496581	0.816496581	0.816496581	0.816496581	0.816496581	24	117	0	P_259149	P_259149	P_259149	chr6	129629354	129629414	-	ARHGAP18	Rho GTPase activating protein 18 [Source:HGNC Symbol;Acc:HGNC:21035]	ENSG00000146376	protein_coding	exon	coding exon	1	4027	6:129629354:129629414	ENSG00000146376	ARHGAP18	8.452314259	0.002910717	3.932874947	1.468188358	1.397217274	-2.464686589	-2.535657673	-0.070971084	9.961963646	0.002827764	0.172905692	0.192688363	0.033491674	0.029662685	0.944825323	-1.931088587	-5.157870045	-6.07423175	-4.560263317	-3.683653085	-7.068627552	5.347429205	0.018725574

ZNF263	-0.025801131	-0.109655582	-0.07587211	-0.083854451	-0.050070979	0.033783472	8	0.072286806	0.816496581	0.816496581	0.816496581	0.816496581	0.816496581	0.816496581	24	118	0	P_118072	P_118072	P_118072	chr16	3291392	3291452	+	ZNF263	zinc finger protein 263 [Source:HGNC Symbol;Acc:HGNC:13056]	ENSG00000006194	protein_coding	3'UTR	3'UTR	1	4028	16:3291392:3291452	ENSG00000006194	ZNF263	7.205274053	0.006402951	-0.39490651	-1.678364521	-1.161282036	-1.28345801	-0.766375526	0.517082485	9.961963646	0.701234979	0.124319226	0.272596724	0.228382538	0.461227078	0.616383668	-6.913923621	-4.88168181	-6.356751141	-6.316353149	-5.993448255	-6.92725043	1.137234132	0.380696441

ATG4B	0.00694444	0.069294934	0.025046555	0.062350494	0.018102115	-0.04424838	8	0.0637102	0.816496581	0.816496581	0.816496581	0.816496581	0.816496581	0.816496581	24	122	0	A_23_P9903	A_23_P9903	A_23_P9903	chr2	241672194	241672254	+	ATG4B	"autophagy related 4B, cysteine peptidase [Source:HGNC Symbol;Acc:HGNC:20790]"	ENSG00000168397	protein_coding	"3'UTR;3'UTR,exon;exon"	multiple Ref	1	4032	2:241672194:241672254	ENSG00000168397	ATG4B	8.870456923	0.005466274	0.115036896	1.147893032	0.414904291	1.032856136	0.299867395	-0.732988741	9.961963646	0.910701553	0.277831064	0.687005822	0.326094065	0.770441904	0.48045739	-6.991143738	-5.531468359	-6.953919127	-6.595568243	-6.24907947	-6.785560075	0.532837036	0.670082368

RBM18	-0.037315914	0.25940508	0.062559517	0.296720994	0.099875431	-0.196845563	8	0.146755036	0.816496581	0.816496581	0.816496581	0.816496581	0.816496581	0.816496581	24	123	0	P_149576	P_149576	P_149576	chr9	122243744	122243804	-	RBM18	RNA binding motif protein 18 [Source:HGNC Symbol;Acc:HGNC:28413]	ENSG00000119446	protein_coding	exon	non-coding exon	1	4033	9:122243744:122243804	ENSG00000119446	RBM18	7.838083528	0.019502102	-0.327264579	2.275010442	0.548653689	2.60227502	0.875918268	-1.726356752	9.961963646	0.75024087	0.046270925	0.595324604	0.026464467	0.401701293	0.115104545	-6.940230022	-4.006653992	-6.885182853	-4.329332952	-5.904052442	-5.648444297	2.683861869	0.103585684

RGL1	0.323746762	-0.062647158	0.13730936	-0.38639392	-0.186437403	0.199956518	8	0.059859228	0.816496581	0.816496581	0.816496581	0.816496581	0.816496581	0.816496581	24	125	0	P_104251	P_104251	P_104251	chr1	183926599	183926659	+	RGL1	ral guanine nucleotide dissociation stimulator-like 1 [Source:HGNC Symbol;Acc:HGNC:30281]	ENSG00000143344	protein_coding	3'UTR	3'UTR	1	4035	1:183926599:183926659	ENSG00000143344	RGL1	5.283009291	0.00508413	5.560869724	-1.076065388	2.358508406	-6.636935112	-3.202361318	3.434573794	9.961963646	0.000243771	0.307267348	0.04014555	5.91E-05	0.009498376	0.006424552	0.603379727	-5.605986638	-4.638152385	1.970986979	-2.602069913	-2.83519631	17.29067892	0.000282567

KRT33B	-0.00855387	-0.002273053	0.126733813	0.006280817	0.135287683	0.129006866	8	0.248034883	0.816496581	0.816496581	0.816496581	0.816496581	0.816496581	0.816496581	24	126	0	P_121428	P_121428	P_121428	chr17	41363499	41363559	-	KRT33B	keratin 33B [Source:HGNC Symbol;Acc:HGNC:6451]	ENSG00000131738	protein_coding	3'UTR	3'UTR	1	4036	17:41363499:41363559	ENSG00000131738	KRT33B	5.432590805	0.051611645	-0.046114174	-0.012254099	0.683225836	0.033860075	0.72934001	0.695479935	9.961963646	0.964130573	0.990464823	0.510041209	0.973657563	0.482589309	0.50265509	-6.997200019	-6.182008263	-6.798032973	-7.148986115	-6.021292783	-6.813394259	0.247641653	0.861184411

CLEC14A	0.342901116	0.040487374	0.066933806	-0.302413742	-0.275967309	0.026446432	8	0.202579615	0.816496581	0.816496581	0.816496581	0.816496581	0.816496581	0.816496581	24	127	0	A_24_P215653	A_24_P215653	A_24_P215653	chr14	38254933	38254993	-	CLEC14A	"C-type lectin domain family 14, member A [Source:HGNC Symbol;Acc:HGNC:19832]"	ENSG00000176435	protein_coding	exon	coding exon	1	4037	14:38254933:38254993	ENSG00000176435	CLEC14A	3.705909081	0.035162838	2.239610887	0.264437643	0.437168835	-1.975173244	-1.802442053	0.172731191	9.961963646	0.049133751	0.796833058	0.671313549	0.076593645	0.101761818	0.866321666	-4.78575478	-6.145357915	-6.943745743	-5.35127305	-4.808783513	-7.055103511	2.076140591	0.167357428

WDR13	0.140139689	0.067931074	0.081566959	-0.072208614	-0.05857273	0.013635884	8	0.079165614	0.816496581	0.816496581	0.816496581	0.816496581	0.816496581	0.816496581	24	128	0	A_24_P231120	A_24_P231120	A_24_P231120	chrX	48600039	48600099	+	WDR13	WD repeat domain 13 [Source:HGNC Symbol;Acc:HGNC:14352]	ENSG00000101940	protein_coding	exon	non-coding exon	1	4038	X:48600039:48600099	ENSG00000101940	WDR13	8.059273215	0.007239583	2.017204867	0.977816455	1.1740947	-1.039388412	-0.843110167	0.196278246	9.961963646	0.071420188	0.351312878	0.26766101	0.323193333	0.418949862	0.848338543	-5.139395903	-5.701738618	-6.342392153	-6.588890092	-5.931909447	-7.050372979	1.372237839	0.307169044

LUZP2	0.500001026	-0.317434025	0.359984995	-0.817435052	-0.140016031	0.677419021	8	0.084107684	0.816496581	0.816496581	0.816496581	0.816496581	0.816496581	0.816496581	24	129	0	A_16_P19183242	A_16_P19183242	A_16_P19183242	chr11	24892351	24892411	+	LUZP2	leucine zipper protein 2 [Source:HGNC Symbol;Acc:HGNC:23206]	ENSG00000187398	protein_coding	exon	non-coding exon	1	4039	11:24892351:24892411	ENSG00000187398	LUZP2	6.679227795	0.007887574	6.895167216	-4.377512382	4.964303286	-11.2726796	-1.930863929	9.341815668	9.961963646	4.30E-05	0.00139534	0.000572908	5.43E-07	0.082427861	3.04E-06	2.400549692	-0.726301914	-0.32152345	6.811634473	-4.622069646	5.086885152	51.57021892	2.25E-06

LARS1	0.172730393	-0.106836947	0.158090261	-0.27956734	-0.014640131	0.264927209	8	0.056115399	0.816496581	0.816496581	0.816496581	0.816496581	0.816496581	0.816496581	24	131	0	P_255235	P_255235	P_255235	chr5	146159406	146159466	-	LARS	leucyl-tRNA synthetase [Source:HGNC Symbol;Acc:HGNC:6512]	ENSG00000133706	protein_coding	exon	coding exon	1	4041	5:146159406:146159466	ENSG00000133706	LARS1	9.873936567	0.004735453	3.074210695	-1.901456258	2.813649443	-4.975666954	-0.260561252	4.715105701	9.961963646	0.011806656	0.086526846	0.018423671	0.000563391	0.799737606	0.000831111	-3.384047353	-4.56755877	-3.873890986	-0.388191473	-6.26070089	-0.727874832	11.34005101	0.001492491

SHC1P1	-0.185711066	-0.043145938	-0.199048258	0.142565128	-0.013337192	-0.15590232	8	0.1900851	0.816496581	0.816496581	0.816496581	0.816496581	0.816496581	0.816496581	24	132	0	A_24_P68585	A_24_P68585	A_24_P68585	chrX	64433643	64433703	+	SHC1P1	SHC (Src homology 2 domain containing) transforming protein 1 pseudogene 1 [Source:HGNC Symbol;Acc:HGNC:10841]	ENSG00000230889	processed_pseudogene	exon	non-coding exon	1	4042	X:64433643:64433703	ENSG00000230889	SHC1P1	7.068732234	0.031222928	-1.287201691	-0.299053391	-1.379644518	0.9881483	-0.092442827	-1.080591126	9.961963646	0.227126174	0.771044954	0.197874243	0.346472515	0.928178825	0.305344432	-6.162534077	-6.135160085	-6.096527561	-6.640356775	-6.292141939	-6.468416551	0.967066673	0.445998634

ZNF141	-0.054187963	-0.413862734	-0.291365061	-0.359674771	-0.237177098	0.122497672	8	0.197046075	0.816496581	0.816496581	0.816496581	0.816496581	0.816496581	0.816496581	24	133	0	A_24_P402836	A_24_P402836	A_24_P402836	chr4	373534	373594	+	ZNF141	zinc finger protein 141 [Source:HGNC Symbol;Acc:HGNC:12926]	ENSG00000131127	protein_coding	exon	coding exon	1	4043	4:373534:373594	ENSG00000131127	ZNF141	8.260230016	0.033387007	-0.363211601	-2.774043124	-1.952964543	-2.410831523	-1.589752943	0.82107858	9.961963646	0.724035514	0.019716771	0.079467657	0.036718728	0.14309099	0.430811886	-6.926847504	-3.219254493	-5.285968192	-4.649877636	-5.103521978	-6.715088449	3.442483585	0.059999925

CAND1	-0.023446732	-0.025002845	-0.088992073	-0.001556113	-0.065545341	-0.063989228	8	0.040388116	0.816496581	0.816496581	0.816496581	0.816496581	0.816496581	0.816496581	24	134	0	A_24_P47681	A_24_P47681	A_24_P47681	chr12	67313716	67313776	+	CAND1	cullin-associated and neddylation-dissociated 1 [Source:HGNC Symbol;Acc:HGNC:30688]	ENSG00000111530	protein_coding	3'UTR	3'UTR	1	4044	12:67313716:67313776	ENSG00000111530	CAND1	9.341291881	0.003516626	-0.484244639	-0.516383003	-1.837950591	-0.032138364	-1.353705952	-1.321567588	9.961963646	0.638681671	0.616854509	0.096033872	0.974996498	0.205744068	0.215857585	-6.871894305	-6.043442052	-5.460469979	-7.149048248	-5.404619924	-6.192759191	1.243216703	0.345354324

PKIG	0.373254846	0.219437326	0.554892565	-0.15381752	0.18163772	0.33545524	8	0.236779264	0.816496581	0.816496581	0.816496581	0.816496581	0.816496581	0.816496581	24	136	0	A_23_P91221	A_23_P91221	A_23_P91221	chr20	44618335	44618395	+	PKIG	"protein kinase (cAMP-dependent, catalytic) inhibitor gamma [Source:HGNC Symbol;Acc:HGNC:9019]"	ENSG00000168734	protein_coding	"3'UTR,exon"	multiple Ref	1	4046	20:44618335:44618395	ENSG00000168734	PKIG	8.035200346	0.04722947	2.103509836	1.236657951	3.127144853	-0.866851885	1.023635017	1.890486902	9.961963646	0.061816154	0.244575002	0.010790589	0.40641803	0.33022204	0.088103893	-5.003798187	-5.434413096	-3.338309356	-6.753529693	-5.767673703	-5.404941388	3.517718836	0.057002126

TAPBPL	0.339315068	0.330736124	0.536141656	-0.008578944	0.196826588	0.205405532	8	0.154031307	0.816496581	0.816496581	0.816496581	0.816496581	0.816496581	0.816496581	24	137	0	P_110729	P_110729	P_110729	chr12	6462258	6462318	+	TAPBPL	TAP binding protein-like [Source:HGNC Symbol;Acc:HGNC:30683]	ENSG00000139192	protein_coding	3'UTR;exon	multiple Ref	1	4047	12:6462258:6462318	ENSG00000139192	TAPBPL	5.487501303	0.021259669	2.850168409	2.778107254	4.503466404	-0.072061154	1.653297995	1.72535915	9.961963646	0.017307173	0.019579982	0.001147913	0.943979476	0.129391899	0.115289614	-3.766298579	-3.212757822	-1.042653218	-7.146774034	-5.017560147	-5.649889412	6.973000132	0.008246072

BTC	0.484255613	0.144422925	0.32316324	-0.339832688	-0.161092374	0.178740315	8	0.215658528	0.816496581	0.816496581	0.816496581	0.816496581	0.816496581	0.816496581	24	138	0	P_249413	P_249413	P_249413	chr4	74748089	74748149	-	BTC	betacellulin [Source:HGNC Symbol;Acc:HGNC:1121]	ENSG00000174808	protein_coding	exon	coding exon	1	4048	4:74748089:74748149	ENSG00000174808	BTC	3.872126418	0.039555626	2.982058512	0.889360083	1.990047535	-2.092698429	-0.992010976	1.100687452	9.961963646	0.013814285	0.394778413	0.074723737	0.062948165	0.344675549	0.296917226	-3.541438048	-5.781497802	-5.228694625	-5.167031792	-5.798336079	-6.447041585	3.369818146	0.063074711

DNAAF1	0.273452183	-0.05877082	0.307104067	-0.332223003	0.033651884	0.365874887	8	0.153729556	0.816496581	0.816496581	0.816496581	0.816496581	0.816496581	0.816496581	24	142	0	A_23_P54612	A_23_P54612	A_23_P54612	chr16	84172295	84172355	+	DNAAF1	"dynein, axonemal, assembly factor 1 [Source:HGNC Symbol;Acc:HGNC:30539]"	ENSG00000154099	protein_coding	3'UTR;exon	multiple Ref	1	4052	16:84172295:84172355	ENSG00000154099	DNAAF1	4.126836233	0.021185092	2.300974714	-0.494529498	2.584139887	-2.795504212	0.283165173	3.078669385	9.961963646	0.04427473	0.631655704	0.027299529	0.019005145	0.782847249	0.011717425	-4.685978741	-6.054784801	-4.262450984	-4.001382427	-6.254213646	-3.443695978	4.931675752	0.023633274

DDX60L	0.032530994	0.228059994	0.142491723	0.195529	0.109960729	-0.085568271	8	0.08957168	0.816496581	0.816496581	0.816496581	0.816496581	0.816496581	0.816496581	24	143	0	P_251551	P_251551	P_251551	chr4	168384693	168384753	-	DDX60L	DEAD (Asp-Glu-Ala-Asp) box polypeptide 60-like [Source:HGNC Symbol;Acc:HGNC:26429]	ENSG00000181381	protein_coding	exon	coding exon	1	4053	4:168384693:168384753	ENSG00000181381	DDX60L	4.118571941	0.008649659	0.428394077	3.003275916	1.876444671	2.574881839	1.448050594	-1.126831245	9.961963646	0.677478854	0.013323203	0.090161754	0.027735877	0.178333314	0.286224614	-6.899132804	-2.852302221	-5.402617812	-4.375512631	-5.287946609	-6.418775265	3.786807996	0.04764231

DGKH	0.19524873	-0.281542222	0.06487806	-0.476790951	-0.130370669	0.346420282	8	0.094040498	0.816496581	0.816496581	0.816496581	0.816496581	0.816496581	0.816496581	24	144	0	P_218955	P_218955	P_218955	chr13	42198531	42198591	+	DGKH	"diacylglycerol kinase, eta [Source:HGNC Symbol;Acc:HGNC:2854]"	ENSG00000102780	protein_coding	exon	coding exon	1	4054	13:42198531:42198591	ENSG00000102780	DGKH	7.532424654	0.009308589	2.478517057	-3.573939766	0.823571958	-6.052456823	-1.654945099	4.397511724	9.961963646	0.032709057	0.005092931	0.429458206	0.000125166	0.12905296	0.001352557	-4.393102749	-1.94596556	-6.688864701	1.185461706	-5.015306979	-1.233073369	13.05059154	0.000871806

ALKBH6	-0.013928855	0.15641385	0.067257997	0.170342705	0.081186852	-0.089155853	8	0.114253891	0.816496581	0.816496581	0.816496581	0.816496581	0.816496581	0.816496581	24	145	0	P_125557	P_125557	P_125557	chr19	36013980	36014040	-	ALKBH6	"alkB, alkylation repair homolog 6 (E. coli) [Source:HGNC Symbol;Acc:HGNC:28243]"	ENSG00000239382	protein_coding	5'UTR	5'UTR	1	4055	19:36013980:36014040	ENSG00000239382	ALKBH6	7.356236637	0.012689719	-0.151438066	1.700571251	0.73124609	1.852009317	0.882684156	-0.969325161	9.961963646	0.882652971	0.119975641	0.481474864	0.093849412	0.398206209	0.355327902	-6.985860192	-4.851299429	-6.762727748	-5.539312599	-5.898194722	-6.581022289	1.433043002	0.290838111

FGD6	0.296352659	-0.022680168	0.272503192	-0.319032827	-0.023849467	0.29518336	8	0.062593852	0.816496581	0.816496581	0.816496581	0.816496581	0.816496581	0.816496581	24	147	0	P_216721	P_216721	P_216721	chr12	95107562	95107622	-	FGD6	"FYVE, RhoGEF and PH domain containing 6 [Source:HGNC Symbol;Acc:HGNC:21740]"	ENSG00000180263	protein_coding	3'UTR;exon	multiple Ref	1	4057	12:95107562:95107622	ENSG00000180263	FGD6	5.938312721	0.005353044	4.960826734	-0.379657068	4.561596054	-5.340483802	-0.39923068	4.941253122	9.961963646	0.000575854	0.712167947	0.001049814	0.000332285	0.698147644	0.000592745	-0.288185134	-6.106668416	-0.950094417	0.163577	-6.212603774	-0.376512367	16.44303912	0.000347146

KBTBD2	0.043092127	-0.072553731	0.158346287	-0.115645859	0.11525416	0.230900019	8	0.065337431	0.816496581	0.816496581	0.816496581	0.816496581	0.816496581	0.816496581	24	148	0	P_144582	P_144582	P_144582	chr7	32868431	32868491	-	KBTBD2	kelch repeat and BTB (POZ) domain containing 2 [Source:HGNC Symbol;Acc:HGNC:21751]	ENSG00000170852	protein_coding	3'UTR	3'UTR	1	4058	7:32868431:32868491	ENSG00000170852	KBTBD2	9.065546278	0.005634908	0.703072588	-1.183755426	2.58350981	-1.886828013	1.880437223	3.767265236	9.961963646	0.498111611	0.26398663	0.027329008	0.088635848	0.089572155	0.003702773	-6.735150389	-5.492900323	-4.263510371	-5.486721637	-4.69608446	-2.271844327	4.976427061	0.023036701

ASCC2	-0.120236131	0.070872637	-0.052222174	0.191108767	0.068013957	-0.12309481	8	0.096287143	0.816496581	0.816496581	0.816496581	0.816496581	0.816496581	0.816496581	24	150	0	P_242380	P_242380	P_242380	chr22	29800991	29801051	-	ASCC2	activating signal cointegrator 1 complex subunit 2 [Source:HGNC Symbol;Acc:HGNC:24103]	ENSG00000100325	protein_coding	3'UTR;exon	multiple Ref	1	4060	22:29800991:29801051	ENSG00000100325	ASCC2	7.520479994	0.009651974	-1.498899597	0.883519506	-0.651017249	2.382419102	0.847882348	-1.534536755	9.961963646	0.164903192	0.397776162	0.529764822	0.038542356	0.41640998	0.156022632	-5.893781995	-5.786540741	-6.820489964	-4.696952819	-5.927914191	-5.917530422	2.033459747	0.173357274

FAM193A	0.071042167	-0.080664204	-0.009738871	-0.151706371	-0.080781037	0.070925334	8	0.05116338	0.816496581	0.816496581	0.816496581	0.816496581	0.816496581	0.816496581	24	152	0	A_24_P126465	A_24_P126465	A_24_P126465	chr4	2690815	2690875	+	FAM193A	"family with sequence similarity 193, member A [Source:HGNC Symbol;Acc:HGNC:16822]"	ENSG00000125386	protein_coding	exon	coding exon	1	4062	4:2690815:2690875	ENSG00000125386	FAM193A	7.154573707	0.004308833	1.325506919	-1.505035191	-0.181708146	-2.830542109	-1.507215064	1.323327045	9.961963646	0.214596057	0.163343551	0.859455819	0.017898455	0.162792536	0.215293377	-6.11597474	-5.111038171	-7.029119254	-3.941591187	-5.21222304	-6.190599259	2.681662953	0.103758272

CRYBA1	-0.00850319	0.250122336	0.115378459	0.258625526	0.123881649	-0.134743877	8	0.226685982	0.816496581	0.816496581	0.816496581	0.816496581	0.816496581	0.816496581	24	153	0	P_120978	P_120978	P_120978	chr17	29254244	29254304	+	CRYBA1	"crystallin, beta A1 [Source:HGNC Symbol;Acc:HGNC:2394]"	ENSG00000108255	protein_coding	3'UTR;exon	multiple Ref	1	4063	17:29254244:29254304	ENSG00000108255	CRYBA1	3.897103384	0.043472873	-0.049948026	1.469227115	0.677736995	1.519175141	0.727685021	-0.79149012	9.961963646	0.961151075	0.172629628	0.51337079	0.159798078	0.48355826	0.447093588	-6.996999094	-5.156559686	-6.801929928	-6.01520024	-6.022508325	-6.739545912	1.014054294	0.426837365

SORBS3	-0.004684774	-0.061164745	0.10195093	-0.056479971	0.106635704	0.163115675	8	0.107088171	0.816496581	0.816496581	0.816496581	0.816496581	0.816496581	0.816496581	24	154	0	P_146697	P_146697	P_146697	chr8	22564982	22565042	+	SORBS3	sorbin and SH3 domain containing 3 [Source:HGNC Symbol;Acc:HGNC:30907]	ENSG00000120896	protein_coding	exon	non-coding exon	1	4064	8:22564982:22565042	ENSG00000120896	SORBS3	5.931592083	0.011416014	-0.053700339	-0.701115475	1.168636848	-0.647415136	1.222337187	1.869752323	9.961963646	0.958235552	0.499280335	0.269754755	0.53199828	0.249712338	0.091158134	-6.99678692	-5.929398656	-6.348523281	-6.92511527	-5.558022305	-5.436300851	1.211564504	0.355513518

SUN1	-0.07729311	-0.140373344	-0.145526469	-0.063080235	-0.06823336	-0.005153125	8	0.062553441	0.816496581	0.816496581	0.816496581	0.816496581	0.816496581	0.816496581	24	155	0	A_24_P400355	A_24_P400355	A_24_P400355	chr7	896274	896334	+	SUN1;GET4	Sad1 and UNC84 domain containing 1 [Source:HGNC Symbol;Acc:HGNC:18587];golgi to ER traffic protein 4 homolog (S. cerevisiae) [Source:HGNC Symbol;Acc:HGNC:21690]	ENSG00000164828	protein_coding	3'UTR	3'UTR	1	4065	7:896274:896334	ENSG00000164828	SUN1	8.177379407	0.005348982	-1.294347302	-2.350686368	-2.436980393	-1.056339066	-1.142633091	-0.086294026	9.961963646	0.224743945	0.040684031	0.035115381	0.315757001	0.279908926	0.932942493	-6.153921933	-3.88923187	-4.50840816	-6.571403646	-5.645522115	-7.06731192	2.59480734	0.110854756

PTPA	-0.135047135	-0.030881664	-0.099298135	0.104165471	0.035749	-0.068416471	8	0.192803236	0.816496581	0.816496581	0.816496581	0.816496581	0.816496581	0.816496581	24	156	0	P_149877	P_149877	P_149877	chr9	129137629	129137689	+	PPP2R4	"protein phosphatase 2A activator, regulatory subunit 4 [Source:HGNC Symbol;Acc:HGNC:9308]"	ENSG00000119383	protein_coding	exon	coding exon	1	4066	9:129137629:129137689	ENSG00000119383	PTPA	6.91117477	0.0320587	-0.923757649	-0.21123864	-0.679225159	0.712519009	0.24453249	-0.467986519	9.961963646	0.377444727	0.836959693	0.512466764	0.492494238	0.811781401	0.64986491	-6.551733258	-6.158618818	-6.800876193	-6.878862287	-6.264978725	-6.953037241	0.357631272	0.784892938

PLEKHA2	0.294477355	0.060667288	0.142849382	-0.233810067	-0.151627973	0.082182094	8	0.094055819	0.816496581	0.816496581	0.816496581	0.816496581	0.816496581	0.816496581	24	159	0	P_147039	P_147039	P_147039	chr8	38969723	38969783	+	PLEKHA2	"pleckstrin homology domain containing, family A (phosphoinositide binding specific) member 2 [Source:HGNC Symbol;Acc:HGNC:14336]"	ENSG00000169499	protein_coding	exon	coding exon	1	4069	8:38969723:38969783	ENSG00000169499	PLEKHA2	5.134950231	0.009310904	3.737675721	0.770024068	1.813126405	-2.967651653	-1.924549316	1.043102337	9.961963646	0.003886843	0.459156037	0.100006062	0.014158184	0.083292423	0.321552812	-2.257044136	-5.878830591	-5.497467094	-3.707004767	-4.631385055	-6.507455826	5.241582185	0.019849051

DIO1	0.675683364	-0.013082182	0.545565865	-0.688765546	-0.130117499	0.558648047	8	0.134645389	0.816496581	0.816496581	0.816496581	0.816496581	0.816496581	0.816496581	25	12	0	P_101910	P_101910	P_101910	chr1	53894385	53894445	+	DIO1	"deiodinase, iodothyronine, type I [Source:HGNC Symbol;Acc:HGNC:2883]"	ENSG00000211452	protein_coding	exon	coding exon	1	4092	1:53894385:53894445	ENSG00000211452	DIO1	4.878958093	0.016765565	6.391160956	-0.123741884	5.16040418	-6.51490284	-1.230756777	5.284146063	9.961963646	8.06E-05	0.903980729	0.000430192	6.89E-05	0.246681485	0.000360065	1.751044777	-6.1740218	-0.023703518	1.810848202	-5.548525759	0.142407347	23.22882377	8.14E-05

EGFR	-0.111257486	0.091983021	-0.121091192	0.203240507	-0.009833706	-0.213074213	8	0.084009665	0.816496581	0.816496581	0.816496581	0.816496581	0.816496581	0.816496581	25	13	0	P_261281	P_261281	P_261281	chr7	55142377	55142437	+	EGFR	epidermal growth factor receptor [Source:HGNC Symbol;Acc:HGNC:3236]	ENSG00000146648	protein_coding	exon	coding exon	1	4093	7:55142377:55142437	ENSG00000146648	EGFR	6.688551311	0.007874341	-1.53556346	1.269539436	-1.671287173	2.805102896	-0.135723713	-2.940826609	9.961963646	0.155773104	0.233103769	0.125733026	0.018695284	0.894743079	0.014821844	-5.844560654	-5.397132111	-5.703816279	-3.985010388	-6.286896761	-3.679569175	3.883061777	0.044746516

RP1	0.144072706	0.453540721	-0.211094231	0.309468015	-0.355166937	-0.664634952	8	0.259146254	0.816496581	0.816496581	0.816496581	0.816496581	0.816496581	0.816496581	25	14	0	P_147208	P_147208	P_147208	chr8	54630188	54630248	+	RP1	retinitis pigmentosa 1 (autosomal dominant) [Source:HGNC Symbol;Acc:HGNC:10263]	ENSG00000104237	protein_coding	exon	coding exon	1	4094	8:54630188:54630248	ENSG00000104237	RP1	3.767273555	0.056137241	0.744735081	2.344425216	-1.091180162	1.599690135	-1.835915243	-3.435605378	9.961963646	0.473634542	0.041120138	0.300881391	0.140867059	0.096353992	0.006413471	-6.703894251	-3.898983689	-6.433164291	-5.904992252	-4.760691754	-2.83343864	4.162435284	0.037455037

AIF1L	-0.068045125	0.092717474	0.084523721	0.160762599	0.152568847	-0.008193753	8	0.092496366	0.816496581	0.816496581	0.816496581	0.816496581	0.816496581	0.816496581	25	17	0	P_269199	P_269199	P_269199	chr9	131121893	131121953	-	AIF1L;NUP214	allograft inflammatory factor 1-like [Source:HGNC Symbol;Acc:HGNC:28904];nucleoporin 214kDa [Source:HGNC Symbol;Acc:HGNC:8064]	ENSG00000126878	protein_coding	3'ext;3'UTR;promoter	multiple non-Ref	0	4097	9:131121893:131121953	ENSG00000126878	AIF1L	8.997221778	0.009077279	-0.874711421	1.191871323	1.086541677	2.066582744	1.961253098	-0.105329646	9.961963646	0.40232695	0.26093098	0.302830194	0.065764667	0.078383396	0.918204893	-6.596222172	-5.484049666	-6.438089671	-5.208343019	-4.577057355	-7.065321048	1.915770404	0.191243996

SERTAD2	0.06298319	0.053340098	0.074573594	-0.009643093	0.011590404	0.021233497	8	0.167528509	0.816496581	0.816496581	0.816496581	0.816496581	0.816496581	0.816496581	25	18	0	A_23_P56949	A_23_P56949	A_23_P56949	chr2	64635709	64635769	-	SERTAD2	SERTA domain containing 2 [Source:HGNC Symbol;Acc:HGNC:30784]	ENSG00000179833	protein_coding	3'UTR	3'UTR	1	4098	2:64635709:64635769	ENSG00000179833	SERTAD2	7.578267638	0.024745053	0.490372485	0.415293605	0.580612678	-0.07507888	0.090240193	0.165319073	9.961963646	0.634490935	0.686730057	0.574400062	0.941638235	0.929884943	0.871999515	-6.868711767	-6.091977382	-6.8660898	-7.146531342	-6.292355777	-7.056468423	0.13185926	0.938884342

CCDC88C	-0.151343032	-0.050522691	-0.093815261	0.100820341	0.057527771	-0.043292569	8	0.064852669	0.816496581	0.816496581	0.816496581	0.816496581	0.816496581	0.816496581	25	21	0	A_24_P911663	A_24_P911663	A_24_P911663	chr14	91324902	91325948	-	CCDC88C	coiled-coil domain containing 88C [Source:HGNC Symbol;Acc:HGNC:19967]	ENSG00000015133	protein_coding	"exon,intron"	multiple non-Ref	0	4101	14:91324902:91325948	ENSG00000015133	CCDC88C	6.993266068	0.005584226	-2.480427689	-0.828038668	-1.53757968	1.652389021	0.942848009	-0.709541012	9.961963646	0.032602369	0.427040337	0.155284105	0.129579289	0.368061736	0.494260874	-4.389921597	-5.833025978	-5.889606476	-5.831048859	-5.844439364	-6.803114517	2.220853519	0.148744725

NEK6	-0.074367149	0.071724018	-0.03646326	0.146091167	0.037903889	-0.108187278	8	0.063568545	0.816496581	0.816496581	0.816496581	0.816496581	0.816496581	0.816496581	25	22	0	P_149640	P_149640	P_149640	chr9	124357108	124357168	+	NEK6;PSMB7	"NIMA-related kinase 6 [Source:HGNC Symbol;Acc:HGNC:7749];proteasome (prosome, macropain) subunit, beta type, 7 [Source:HGNC Symbol;Acc:HGNC:9544]"	ENSG00000119408	protein_coding	3'ext;intron	multiple non-Ref	0	4102	9:124357108:124357168	ENSG00000119408	NEK6	7.09850492	0.005451795	-1.233550691	1.189708263	-0.604827271	2.423258954	0.62872342	-1.794535534	9.961963646	0.245682345	0.261742601	0.558817778	0.035947953	0.543676008	0.103079281	-6.226098089	-5.486412927	-6.8509519	-4.629242052	-6.090633457	-5.548646774	2.131783995	0.159895461

PPP1R13L	-0.154022482	0.001013132	-0.048743373	0.155035613	0.105279109	-0.049756505	8	0.111100406	0.816496581	0.816496581	0.816496581	0.816496581	0.816496581	0.816496581	25	25	0	P_310897	P_310897	P_310897	chr19	45397926	45397986	+	PPP1R13L	"protein phosphatase 1, regulatory subunit 13 like [Source:HGNC Symbol;Acc:HGNC:18838]"	ENSG00000104881	protein_coding	intron;promoter	multiple non-Ref	0	4105	19:45397926:45397986	ENSG00000104881	PPP1R13L	5.512212042	0.012119027	-1.713546403	0.011271394	-0.542284676	1.724817797	1.171261727	-0.553556071	9.961963646	0.117501665	0.99122945	0.599542319	0.115390156	0.268746181	0.592088825	-5.595739615	-6.18202045	-6.888866472	-5.727236121	-5.614600151	-6.906518643	1.31395263	0.323799787

MYNN	0.912498531	-0.46109508	0.355485127	-1.373593611	-0.557013404	0.816580207	8	0.050217782	0.816496581	0.816496581	0.816496581	0.816496581	0.816496581	0.816496581	25	26	0	P_136493	P_136493	P_136493	chr3	169786815	169786875	+	MYNN	myoneurin [Source:HGNC Symbol;Acc:HGNC:14955]	ENSG00000085274	protein_coding	3'UTR	3'UTR	1	4106	3:169786815:169786875	ENSG00000085274	MYNN	8.760070372	0.004231847	17.17959021	-8.681027148	6.692710841	-25.86061736	-10.48687937	15.37373799	9.961963646	9.90E-09	5.87E-06	5.51E-05	1.83E-10	1.06E-06	2.88E-08	10.56789665	4.082054881	2.11312271	14.12191285	5.626490727	9.661249288	238.3981533	1.43E-09

SLC39A3	-0.16356892	0.196504373	-0.010887993	0.360073293	0.152680927	-0.207392366	8	0.086673282	0.816496581	0.816496581	0.816496581	0.816496581	0.816496581	0.816496581	25	27	0	A_24_P382467	A_24_P382467	A_24_P382467	chr19	2737118	2737178	-	SLC39A3;AC006538.4	"solute carrier family 39 (zinc transporter), member 3 [Source:HGNC Symbol;Acc:HGNC:17128];Uncharacterized protein [Source:UniProtKB/TrEMBL;Acc:K7EQS6]"	ENSG00000141873	protein_coding	exon	coding exon	1	4107	19:2737118:2737178	ENSG00000141873	SLC39A3	9.290405217	0.008239437	-2.206976241	2.651362381	-0.146907749	4.858338622	2.060068492	-2.79827013	9.961963646	0.051924267	0.024329578	0.886135352	0.000670374	0.066485667	0.01891533	-4.838466077	-3.414993447	-7.035347356	-0.569653463	-4.428733813	-3.922817186	7.933283429	0.005370988

RFTN2	-0.156698395	0.022408738	-0.071544365	0.179107132	0.08515403	-0.093953103	8	0.118661804	0.816496581	0.816496581	0.816496581	0.816496581	0.816496581	0.816496581	25	28	0	P_238532	P_238532	P_238532	chr2	197595990	197596050	-	RFTN2	raftlin family member 2 [Source:HGNC Symbol;Acc:HGNC:26402]	ENSG00000162944	protein_coding	exon	coding exon	1	4108	2:197595990:197596050	ENSG00000162944	RFTN2	3.972434735	0.013514193	-1.650878552	0.23608477	-0.753747719	1.886963322	0.897130834	-0.989832489	9.961963646	0.129891217	0.818149924	0.468441533	0.088616123	0.390814524	0.345688163	-5.68512887	-6.15279029	-6.745443794	-5.486516359	-5.885559238	-6.56101564	1.449085717	0.286694816

ZW10	-0.284870098	0.049821184	-0.155183619	0.334691282	0.129686479	-0.205004802	8	0.068594876	0.816496581	0.816496581	0.816496581	0.816496581	0.816496581	0.816496581	25	30	0	P_213387	P_213387	P_213387	chr11	113743801	113743861	-	ZW10	zw10 kinetochore protein [Source:HGNC Symbol;Acc:HGNC:13194]	ENSG00000086827	protein_coding	exon	coding exon	1	4110	11:113743801:113743861	ENSG00000086827	ZW10	7.85604861	0.005985262	-4.509733875	0.788711351	-2.456687544	5.298445225	2.053046331	-3.245398895	9.961963646	0.001136883	0.448643244	0.033952707	0.000352787	0.067271291	0.008831456	-0.992259851	-5.864394281	-4.475657629	0.100965821	-4.439367629	-3.158266814	11.63603566	0.001354173

STIM2	-0.008594186	-0.141121474	0.081919063	-0.132527288	0.09051325	0.223040538	8	0.138403963	0.816496581	0.816496581	0.816496581	0.816496581	0.816496581	0.816496581	25	32	0	P_248472	P_248472	P_248472	chr4	27017924	27017984	+	STIM2	stromal interaction molecule 2 [Source:HGNC Symbol;Acc:HGNC:19205]	ENSG00000109689	protein_coding	exon	coding exon	1	4112	4:27017924:27017984	ENSG00000109689	STIM2	7.633665288	0.017589721	-0.079363521	-1.303194561	0.756486411	-1.22383104	0.835849931	2.059680971	9.961963646	0.938315093	0.221823035	0.466870764	0.249172402	0.422834123	0.066528794	-6.994924333	-5.358259523	-6.743308239	-6.386898378	-5.93795038	-5.143374065	1.452596591	0.285797028

ATP1A1-AS1	-0.177924782	-0.067295507	-0.165874498	0.110629275	0.012050285	-0.098578991	8	0.151287787	0.816496581	0.816496581	0.816496581	0.816496581	0.816496581	0.816496581	25	35	0	P_102845	P_102845	P_102845	chr1	116398999	116399059	-	ATP1A1-AS1	ATP1A1 antisense RNA 1 [Source:HGNC Symbol;Acc:HGNC:28262]	ENSG00000203865	processed_transcript	exon	non-coding exon	1	4115	1:116398999:116399059	ENSG00000203865	ATP1A1-AS1	5.615505512	0.020586992	-1.518747683	-0.57442754	-1.41588769	0.944320143	0.102859994	-0.84146015	9.961963646	0.159904267	0.578418139	0.187305431	0.3673452	0.920115218	0.419830492	-5.867227514	-6.011074753	-6.050336791	-6.68268002	-6.291060887	-6.697786269	1.041945895	0.415882383

TNR	-0.100161623	0.010012652	0.1224769	0.110174275	0.222638523	0.112464248	8	0.119081909	0.816496581	0.816496581	0.816496581	0.816496581	0.816496581	0.816496581	25	36	0	P_205032	P_205032	P_205032	chr1	175324405	175324465	-	TNR	tenascin R [Source:HGNC Symbol;Acc:HGNC:11953]	ENSG00000116147	protein_coding	exon	coding exon	1	4116	1:175324405:175324465	ENSG00000116147	TNR	4.569835844	0.0135944	-1.052124098	0.105175535	1.286529646	1.157299633	2.338653744	1.181354111	9.961963646	0.317593903	0.918324085	0.22735129	0.274145416	0.041526177	0.264896178	-6.425819123	-6.176259278	-6.211411625	-6.46267022	-3.997298386	-6.358192341	1.829571141	0.205711154

HMGXB3	-0.207863752	0.134917889	-0.080772581	0.342781641	0.127091171	-0.21569047	8	0.055162268	0.816496581	0.816496581	0.816496581	0.816496581	0.816496581	0.816496581	25	37	0	P_255375	P_255375	P_255375	chr5	150032503	150032563	+	HMGXB3	HMG box domain containing 3 [Source:HGNC Symbol;Acc:HGNC:28982]	ENSG00000113716	protein_coding	exon	coding exon	1	4117	5:150032503:150032563	ENSG00000113716	HMGXB3	6.527225892	0.004650279	-3.733231123	2.423124084	-1.450674828	6.156355207	2.282556296	-3.873798911	9.961963646	0.003915305	0.035956232	0.177617811	0.000109176	0.045681941	0.003112019	-2.264506424	-3.775974639	-6.005258871	1.328556783	-4.085496512	-2.09330433	13.33834885	0.000800739

TES	0.254416815	0.003054884	0.238115385	-0.251361932	-0.01630143	0.235060501	8	0.043007365	0.816496581	0.816496581	0.816496581	0.816496581	0.816496581	0.816496581	25	38	0	P_262535	P_262535	P_262535	chr7	116234559	116234619	+	TES	testis derived transcript (3 LIM domains) [Source:HGNC Symbol;Acc:HGNC:14620]	ENSG00000135269	protein_coding	exon	coding exon	1	4118	7:116234559:116234619	ENSG00000135269	TES	9.935763103	0.00369204	5.128120404	0.061575379	4.799542684	-5.066545024	-0.328577719	4.737967305	9.961963646	0.000450797	0.952118852	0.000731945	0.000493077	0.749277454	0.000802925	-0.034364205	-6.180089035	-0.575972854	-0.248969524	-6.239580226	-0.692032177	16.26053273	0.000363288

DUSP11	-0.0425484	0.067239725	-0.075041726	0.109788125	-0.032493326	-0.142281451	8	0.045714944	0.816496581	0.816496581	0.816496581	0.816496581	0.816496581	0.816496581	25	40	0	P_235523	P_235523	P_235523	chr2	73766827	73766887	-	DUSP11	dual specificity phosphatase 11 (RNA/RNP complex 1-interacting) [Source:HGNC Symbol;Acc:HGNC:3066]	ENSG00000144048	protein_coding	exon	coding exon	1	4120	2:73766827:73766887	ENSG00000144048	DUSP11	7.585621614	0.003884952	-0.836057249	1.32123086	-1.474536735	2.157288109	-0.638479487	-2.795767596	9.961963646	0.42272287	0.215965704	0.171224466	0.056468439	0.537562494	0.018996574	-6.629817581	-5.337136299	-5.973928545	-5.064022749	-6.084320988	-3.927077294	2.91612763	0.08712293

HDAC9	0.035653333	-0.036273359	0.052227156	-0.071926693	0.016573823	0.088500515	8	0.100878239	0.816496581	0.816496581	0.816496581	0.816496581	0.816496581	0.816496581	25	41	0	P_260622	P_260622	P_260622	chr7	18593969	18594029	+	HDAC9	histone deacetylase 9 [Source:HGNC Symbol;Acc:HGNC:14065]	ENSG00000048052	protein_coding	exon	coding exon	1	4121	7:18593969:18594029	ENSG00000048052	HDAC9	4.112928491	0.010378903	0.428617633	-0.436071467	0.627864995	-0.8646891	0.199247362	1.063936462	9.961963646	0.677321466	0.672083194	0.54421584	0.407548835	0.846077236	0.31246641	-6.899030162	-6.082825616	-6.83601731	-6.755433504	-6.275612976	-6.485895248	0.447905566	0.724251471

USP45	0.029295578	0.046629587	0.029860518	0.017334009	0.00056494	-0.01676907	8	0.05976255	0.816496581	0.816496581	0.816496581	0.816496581	0.816496581	0.816496581	25	42	0	P_258415	P_258415	P_258415	chr6	99464668	99464728	-	USP45	ubiquitin specific peptidase 45 [Source:HGNC Symbol;Acc:HGNC:20080]	ENSG00000123552	protein_coding	3'UTR;exon	multiple Ref	1	4122	6:99464668:99464728	ENSG00000123552	USP45	6.753764426	0.005074843	0.503658749	0.801670464	0.513371368	0.298011715	0.009712619	-0.288299095	9.961963646	0.625451224	0.441446373	0.618883807	0.771816909	0.992442325	0.779027107	-6.861680671	-5.854204889	-6.905077959	-7.101261822	-6.296635082	-7.02616147	0.221986586	0.878941747

MYL5	0.008821119	-0.077972357	-0.112046165	-0.086793475	-0.120867284	-0.034073809	8	0.086953598	0.816496581	0.816496581	0.816496581	0.816496581	0.816496581	0.816496581	25	43	0	P_247944	P_247944	P_247944	chr4	680527	680587	+	MYL5	"myosin, light chain 5, regulatory [Source:HGNC Symbol;Acc:HGNC:7586]"	ENSG00000215375	protein_coding	3'UTR;exon	multiple Ref	1	4123	4:680527:680587	ENSG00000215375	MYL5	9.418687828	0.008278522	0.118738867	-1.049566341	-1.508225336	-1.168305208	-1.626964203	-0.458658995	9.961963646	0.907842451	0.318712526	0.162537715	0.269882396	0.134920167	0.656322575	-6.990672131	-5.632530355	-5.929143509	-6.450356224	-5.053414218	-6.957658793	1.268791624	0.337381449

GLT8D1	-0.092202332	0.029521468	-0.061961774	0.121723799	0.030240557	-0.091483242	8	0.0452549	0.816496581	0.816496581	0.816496581	0.816496581	0.816496581	0.816496581	25	44	0	P_135098	P_135098	P_135098	chr3	52695714	52695774	-	GLT8D1	glycosyltransferase 8 domain containing 1 [Source:HGNC Symbol;Acc:HGNC:24870]	ENSG00000016864	protein_coding	exon	non-coding exon	1	4124	3:52695714:52695774	ENSG00000016864	GLT8D1	9.066428654	0.003851344	-1.819622725	0.582609274	-1.22282214	2.402232	0.596800585	-1.805431415	9.961963646	0.098952194	0.573106273	0.249536954	0.037261562	0.563956643	0.101267743	-5.4404294	-6.006253244	-6.286708222	-4.664141133	-6.110660623	-5.532514125	2.422037755	0.126745163

ZNF132	-0.00818996	-0.096527196	-0.124321203	-0.088337236	-0.116131243	-0.027794007	8	0.084946203	0.816496581	0.816496581	0.816496581	0.816496581	0.816496581	0.816496581	25	46	0	A_24_P121846	A_24_P121846	A_24_P121846	chr19	58433450	58433510	-	ZNF132	zinc finger protein 132 [Source:HGNC Symbol;Acc:HGNC:12916]	ENSG00000131849	protein_coding	exon	coding exon	1	4126	19:58433450:58433510	ENSG00000131849	ZNF132	6.515412158	0.008001411	-0.112135755	-1.321636517	-1.702188073	-1.209500762	-1.590052319	-0.380551556	9.961963646	0.912943097	0.215835458	0.119664812	0.254390919	0.143023538	0.711524727	-6.991502913	-5.336658914	-5.659628701	-6.403485836	-5.103121501	-6.992836706	1.465453284	0.282536495

PHF12	0.18808957	-0.040344519	0.036608983	-0.228434089	-0.151480588	0.076953501	8	0.043965184	0.816496581	0.816496581	0.816496581	0.816496581	0.816496581	0.816496581	25	49	0	P_228172	P_228172	P_228172	chr17	28921687	28921747	-	PHF12	PHD finger protein 12 [Source:HGNC Symbol;Acc:HGNC:20816]	ENSG00000109118	protein_coding	"3'UTR,exon;exon"	multiple Ref	1	4129	17:28921687:28921747	ENSG00000109118	PHF12	7.145262768	0.003758938	3.757316294	-0.805930475	0.731308637	-4.563246769	-3.026007656	1.537239112	9.961963646	0.003763626	0.439097342	0.481438321	0.001047161	0.012816743	0.155366609	-2.224088777	-5.850823672	-6.762680355	-1.034434561	-2.888772714	-5.913869906	7.94080158	0.005353737

CREB3L2	-0.197049679	0.029421041	-0.266435891	0.226470719	-0.069386213	-0.295856932	8	0.064676814	0.816496581	0.816496581	0.816496581	0.816496581	0.816496581	0.816496581	25	50	0	P_263071	P_263071	P_263071	chr7	137901353	137901413	-	CREB3L2	cAMP responsive element binding protein 3-like 2 [Source:HGNC Symbol;Acc:HGNC:23720]	ENSG00000182158	protein_coding	3'UTR;exon	multiple Ref	1	4130	7:137901353:137901413	ENSG00000182158	CREB3L2	7.309158142	0.005565934	-3.234836631	0.482986119	-4.37390503	3.71782275	-1.139068399	-4.856891149	9.961963646	0.008990546	0.639544014	0.001403208	0.004015674	0.281324031	0.000671821	-3.109665477	-6.060588174	-1.250539451	-2.42556901	-5.649331788	-0.506748965	11.42292721	0.001452119

SOX8	0.119149113	-0.154726047	0.04251071	-0.27387516	-0.076638403	0.197236757	8	0.14877563	0.816496581	0.816496581	0.816496581	0.816496581	0.816496581	0.816496581	25	54	0	P_117816	P_117816	P_117816	chr16	985586	985646	+	SOX8	SRY (sex determining region Y)-box 8 [Source:HGNC Symbol;Acc:HGNC:11203]	ENSG00000005513	protein_coding	3'UTR;exon	multiple Ref	1	4134	16:985586:985646	ENSG00000005513	SOX8	7.918418631	0.019981644	1.032335436	-1.340582212	0.368322609	-2.372917648	-0.664012828	1.70890482	9.961963646	0.326326128	0.209824979	0.720338843	0.039171777	0.521753021	0.118381322	-6.446096904	-5.31425162	-6.973538071	-4.712662279	-6.06737864	-5.673660039	1.998424258	0.178470312

APC2	0.052479966	-0.069474336	0.171152594	-0.121954303	0.118672628	0.24062693	8	0.107952271	0.816496581	0.816496581	0.816496581	0.816496581	0.816496581	0.816496581	25	56	0	A_23_P101905	A_23_P101905	A_23_P101905	chr19	1473181	1473241	+	APC2	adenomatosis polyposis coli 2 [Source:HGNC Symbol;Acc:HGNC:24036]	ENSG00000115266	protein_coding	3'UTR	3'UTR	1	4136	19:1473181:1473241	ENSG00000115266	APC2	6.953043167	0.011565235	0.597670675	-0.791211898	1.949179734	-1.388882573	1.351509059	2.740391632	9.961963646	0.563398321	0.447248588	0.07996743	0.195133468	0.206422531	0.020886933	-6.806874801	-5.862439495	-5.291787243	-6.18586568	-5.407274283	-4.021230875	2.674640996	0.104311683

TUBGCP3	-0.18962026	-0.04544645	-0.185603627	0.14417381	0.004016633	-0.140157177	8	0.0658477	0.816496581	0.816496581	0.816496581	0.816496581	0.816496581	0.816496581	25	57	0	P_309631	P_309631	P_309631	chr13	112490621	112490681	-	TUBGCP3	"tubulin, gamma complex associated protein 3 [Source:HGNC Symbol;Acc:HGNC:18598]"	ENSG00000126216	protein_coding	intron;promoter	multiple non-Ref	0	4137	13:112490621:112490681	ENSG00000126216	TUBGCP3	7.435755105	0.005688664	-3.079110203	-0.737972982	-3.013886929	2.341137221	0.065223274	-2.275913948	9.961963646	0.01170864	0.477554756	0.013084305	0.04135098	0.949286539	0.046200016	-3.375676497	-5.902876779	-3.532082353	-4.765081801	-6.29442307	-4.796692045	4.962333341	0.023222622

PIGM	-0.18054268	0.100618347	-0.143775398	0.281161026	0.036767281	-0.244393745	8	0.111291684	0.816496581	0.816496581	0.816496581	0.816496581	0.816496581	0.816496581	25	60	0	P_103709	P_103709	P_103709	chr1	160030474	160030534	-	PIGM	"phosphatidylinositol glycan anchor biosynthesis, class M [Source:HGNC Symbol;Acc:HGNC:18858]"	ENSG00000143315	protein_coding	exon	coding exon	1	4140	1:160030474:160030534	ENSG00000143315	PIGM	8.810251779	0.012153188	-2.005766649	1.117834985	-1.597294886	3.123601634	0.408471763	-2.715129871	9.961963646	0.07279442	0.289869655	0.14140029	0.010855723	0.691569164	0.021810944	-5.157194272	-5.563102096	-5.807753762	-3.439533788	-6.208699832	-4.064103096	4.18661207	0.036893267

POLR2C	0.134019757	-0.098359987	-0.062781004	-0.232379744	-0.196800761	0.035578983	8	0.126838609	0.816496581	0.816496581	0.816496581	0.816496581	0.816496581	0.816496581	25	61	0	P_119113	P_119113	P_119113	chr16	57471623	57471683	+	POLR2C	"polymerase (RNA) II (DNA directed) polypeptide C, 33kDa [Source:HGNC Symbol;Acc:HGNC:9189]"	ENSG00000102978	protein_coding	3'UTR	3'UTR	1	4141	16:57471623:57471683	ENSG00000102978	POLR2C	9.397767377	0.015126252	1.334592856	-0.979486453	-0.625184534	-2.31407931	-1.959777391	0.354301919	9.961963646	0.211709795	0.350527159	0.545903464	0.043299274	0.078575418	0.730497462	-6.104790801	-5.700172891	-6.837781507	-4.80955357	-4.579250011	-7.003232632	2.075434638	0.167454678

LINC01304	1.897893257	-0.071171299	0.428877947	-1.969064556	-1.46901531	0.500049246	8	1.683814728	0.816496581	0.816496581	0.816496581	0.816496581	0.816496581	0.816496581	25	62	0	A_32_P197744	A_32_P197744	A_32_P197744	chr2	3960458	3960518	-	LINC01304	long intergenic non-protein coding RNA 1304 [Source:HGNC Symbol;Acc:HGNC:50472]	ENSG00000237401	lincRNA	exon	non-coding exon	1	4142	2:3960458:3960518	ENSG00000237401	LINC01304	4.827132777	2.279052605	1.539714634	-0.057739544	0.347938246	-1.597454178	-1.191776388	0.40567779	9.961963646	0.154767777	0.955097839	0.735126481	0.141364773	0.260966559	0.693555312	-5.838941469	-6.180330217	-6.981409381	-5.908098709	-5.592090144	-6.98221068	1.105313551	0.392107888

COL9A1	-0.024357133	-0.06260013	0.051966718	-0.038242997	0.076323851	0.114566848	8	0.069457713	0.816496581	0.816496581	0.816496581	0.816496581	0.816496581	0.816496581	25	63	0	P_143006	P_143006	P_143006	chr6	70216235	70216295	-	COL9A1	"collagen, type IX, alpha 1 [Source:HGNC Symbol;Acc:HGNC:2217]"	ENSG00000112280	protein_coding	3'UTR	3'UTR	1	4143	6:70216235:70216295	ENSG00000112280	COL9A1	4.651398957	0.006080919	-0.3825491	-0.983187265	0.816180656	-0.600638165	1.198729756	1.799367921	9.961963646	0.710089167	0.348790555	0.433479343	0.561496455	0.25837086	0.102272212	-6.919087276	-5.696695383	-6.695062015	-6.955831583	-5.584394673	-5.541497932	1.135766004	0.38121323

MEA1	-0.236901919	0.182073506	-0.116381667	0.418975424	0.120520251	-0.298455173	8	0.106711129	0.816496581	0.816496581	0.816496581	0.816496581	0.816496581	0.816496581	25	64	0	P_142678	P_142678	P_142678	chr6	43012938	43012998	-	MEA1	male-enhanced antigen 1 [Source:HGNC Symbol;Acc:HGNC:6986]	ENSG00000124733	protein_coding	exon	coding exon	1	4144	6:43012938:43012998	ENSG00000124733	MEA1	11.16567081	0.011351279	-2.723276976	2.093003672	-1.337851196	4.816280648	1.385425781	-3.430854867	9.961963646	0.021508551	0.062915935	0.210682667	0.000713827	0.196155254	0.006464666	-3.981771757	-4.284401636	-6.148780347	-0.635168628	-5.365972384	-2.841533261	8.412246113	0.004393185

KIAA0825	0.089253377	-0.017307953	0.10601774	-0.106561329	0.016764364	0.123325693	8	0.050316125	0.816496581	0.816496581	0.816496581	0.816496581	0.816496581	0.816496581	25	67	0	P_253917	P_253917	P_253917	chr5	94469960	94470020	-	KIAA0825	KIAA0825 [Source:HGNC Symbol;Acc:HGNC:28532]	ENSG00000185261	protein_coding	exon	coding exon	1	4147	5:94469960:94470020	ENSG00000185261	KIAA0825	4.495686724	0.004239787	1.678797287	-0.325551199	1.994123937	-2.004348486	0.31532665	2.319675136	9.961963646	0.124233241	0.751498609	0.074218832	0.072966534	0.759019896	0.042889147	-5.645533364	-6.126523337	-5.222370561	-5.305946576	-6.244070199	-4.725153259	2.733101779	0.099808253

DDIT4L	-0.299185788	0.348869866	-0.149784823	0.648055654	0.149400965	-0.498654689	8	0.412979087	0.816496581	0.816496581	0.816496581	0.816496581	0.816496581	0.816496581	25	69	0	A_23_P302672	A_23_P302672	A_23_P302672	chr4	100186078	100186138	-	DDIT4L	DNA-damage-inducible transcript 4-like [Source:HGNC Symbol;Acc:HGNC:30555]	ENSG00000145358	protein_coding	3'UTR	3'UTR	1	4149	4:100186078:100186138	ENSG00000145358	DDIT4L	3.155058078	0.13916902	-0.982234773	1.145348902	-0.491747495	2.127583676	0.490487279	-1.637096397	9.961963646	0.349236907	0.278834565	0.63355244	0.059364922	0.634412558	0.132768599	-6.496039118	-5.534170528	-6.916651635	-5.111540035	-6.170296381	-5.775920246	1.660949923	0.237834123

EFCAB7	0.323612599	0.105909371	0.268741603	-0.217703228	-0.054870996	0.162832232	8	0.060894704	0.816496581	0.816496581	0.816496581	0.816496581	0.816496581	0.816496581	25	71	0	P_102041	P_102041	P_102041	chr1	63572603	63572663	+	EFCAB7	EF-hand calcium binding domain 7 [Source:HGNC Symbol;Acc:HGNC:29379]	ENSG00000203965	protein_coding	3'UTR;exon	multiple Ref	1	4151	1:63572603:63572663	ENSG00000203965	EFCAB7	7.562753951	0.005184543	5.504473987	1.801460699	4.571148244	-3.703013288	-0.933325742	2.769687545	9.961963646	0.000263708	0.101924496	0.001034563	0.004114671	0.372720824	0.019864436	0.521816116	-4.710705532	-0.934927581	-2.450591144	-5.853144868	-3.971448219	12.78241071	0.000944953

FCRL5	-0.155609633	-0.032532422	-0.067214904	0.12307721	0.088394729	-0.034682482	8	0.120475625	0.816496581	0.816496581	0.816496581	0.816496581	0.816496581	0.816496581	25	72	0	P_204350	P_204350	P_204350	chr1	157527660	157527720	-	FCRL5	Fc receptor-like 5 [Source:HGNC Symbol;Acc:HGNC:18508]	ENSG00000143297	protein_coding	exon	coding exon	1	4152	1:157527660:157527720	ENSG00000143297	FCRL5	3.676323518	0.013862519	-1.618680078	-0.338408252	-0.699181819	1.280271826	0.919498259	-0.360773567	9.961963646	0.13670236	0.742079532	0.500436704	0.229456308	0.379561321	0.725801519	-5.730325094	-6.122074424	-6.786542474	-6.320183921	-5.865654979	-7.000736753	0.97303852	0.443513243

SPOUT1	-0.077237218	0.029735843	-0.135760711	0.106973061	-0.058523493	-0.165496554	8	0.068744658	0.816496581	0.816496581	0.816496581	0.816496581	0.816496581	0.816496581	25	73	0	A_23_P9392	A_23_P9392	A_23_P9392	chr9	128823760	128823820	-	C9orf114	chromosome 9 open reading frame 114 [Source:HGNC Symbol;Acc:HGNC:26933]	ENSG00000198917	protein_coding	exon	coding exon	1	4153	9:128823760:128823820	ENSG00000198917	SPOUT1	8.892654749	0.006001782	-1.221046382	0.470095166	-2.146246702	1.691141548	-0.92520032	-2.616341868	9.961963646	0.250179646	0.648409225	0.057528904	0.121803053	0.376729739	0.025834281	-6.240629192	-6.066914656	-4.98269388	-5.775809774	-5.860515075	-4.231186547	2.813253129	0.094001436

DAXX	-0.309764319	0.042282779	-0.091191245	0.352047099	0.218573075	-0.133474024	8	0.132543331	0.816496581	0.816496581	0.816496581	0.816496581	0.816496581	0.816496581	25	74	0	P_142357	P_142357	P_142357	chr6	33320229	33320289	-	DAXX	death-domain associated protein [Source:HGNC Symbol;Acc:HGNC:2681]	ENSG00000204209	protein_coding	exon	non-coding exon	1	4154	6:33320229:33320289	ENSG00000204209	DAXX	8.051172216	0.016314533	-2.970227413	0.405435559	-0.874402629	3.375662973	2.095824784	-1.279838189	9.961963646	0.014096067	0.693727621	0.402487143	0.007091367	0.062618803	0.229602767	-3.561633324	-6.096168739	-6.6449366	-3.007521715	-4.374379484	-6.243391518	4.529462776	0.029913756

DEPDC4	-0.202649742	0.125949489	-0.025172965	0.328599231	0.177476777	-0.151122454	8	0.057784066	0.816496581	0.816496581	0.816496581	0.816496581	0.816496581	0.816496581	25	75	0	A_23_P305137	A_23_P305137	A_23_P305137	chr12	100241720	100241780	-	DEPDC4	DEP domain containing 4 [Source:HGNC Symbol;Acc:HGNC:22952]	ENSG00000166153	protein_coding	3'UTR	3'UTR	1	4155	12:100241720:100241780	ENSG00000166153	DEPDC4	7.417542256	0.004888082	-3.549951825	2.206341904	-0.440971766	5.75629373	3.108980059	-2.647313671	9.961963646	0.005299922	0.051979996	0.668649433	0.00018628	0.011128789	0.024498967	-2.573583278	-4.112285785	-6.941956883	0.769191491	-2.753784157	-4.178909492	11.24558381	0.001540162

UMODL1	0.06216976	0.045471806	0.084686702	-0.016697954	0.022516942	0.039214896	8	0.110836559	0.816496581	0.816496581	0.816496581	0.816496581	0.816496581	0.816496581	25	76	0	P_241925	P_241925	P_241925	chr21	42137560	42137620	+	UMODL1	uromodulin-like 1 [Source:HGNC Symbol;Acc:HGNC:12560]	ENSG00000177398	protein_coding	exon	coding exon	1	4156	21:42137560:42137620	ENSG00000177398	UMODL1	4.907424493	0.012072002	0.693003085	0.50687186	0.943998263	-0.186131226	0.250995178	0.437126404	9.961963646	0.504142715	0.623274756	0.367501783	0.85607739	0.806919024	0.671343301	-6.742460957	-6.048435795	-6.581166057	-7.13070003	-6.263286171	-6.967986619	0.319504664	0.811191651

EPHB3	0.154390797	0.024322798	0.015585358	-0.130068	-0.138805439	-0.00873744	8	0.149290233	0.816496581	0.816496581	0.816496581	0.816496581	0.816496581	0.816496581	25	79	0	A_23_P95060	A_23_P95060	A_23_P95060	chr3	184582036	184582096	+	EPHB3	EPH receptor B3 [Source:HGNC Symbol;Acc:HGNC:3394]	ENSG00000182580	protein_coding	3'UTR	3'UTR	1	4159	3:184582036:184582096	ENSG00000182580	EPHB3	7.777568137	0.020104821	1.333573432	0.210091776	0.134620843	-1.123481656	-1.198952589	-0.075470933	9.961963646	0.212032006	0.837830637	0.895592693	0.287577577	0.25828802	0.941334112	-6.106048262	-6.158872375	-7.03723263	-6.499949945	-5.584147511	-7.068267751	0.757678938	0.542988374

MAFK	-0.109366133	-0.097468979	-0.116339485	0.011897154	-0.006973352	-0.018870506	8	0.160426802	0.816496581	0.816496581	0.816496581	0.816496581	0.816496581	0.816496581	25	80	0	P_144149	P_144149	P_144149	chr7	1537521	1537581	+	MAFK	v-maf avian musculoaponeurotic fibrosarcoma oncogene homolog K [Source:HGNC Symbol;Acc:HGNC:6782]	ENSG00000198517	protein_coding	5'UTR	5'UTR	1	4160	7:1537521:1537581	ENSG00000198517	MAFK	8.810817733	0.022874705	-0.885626775	-0.789285819	-0.942095695	0.096340956	-0.05646892	-0.152809876	9.961963646	0.396692754	0.448322588	0.3684283	0.925160352	0.956084795	0.881598996	-6.58649962	-5.863945675	-6.582963932	-7.144539655	-6.29498949	-7.058636903	0.388445394	0.76389538

EPG5	-0.064001335	-0.041018777	-0.197977602	0.022982559	-0.133976267	-0.156958825	8	0.090110201	0.816496581	0.816496581	0.816496581	0.816496581	0.816496581	0.816496581	25	81	0	P_231311	P_231311	P_231311	chr18	45925737	45925797	-	EPG5	ectopic P-granules autophagy protein 5 homolog (C. elegans) [Source:HGNC Symbol;Acc:HGNC:29331]	ENSG00000152223	protein_coding	exon	coding exon	1	4161	18:45925737:45925797	ENSG00000152223	EPG5	5.522901026	0.008727365	-0.839060154	-0.537757859	-2.595494555	0.301302294	-1.756434401	-2.057736695	9.961963646	0.421113638	0.602549576	0.026773669	0.769379256	0.109649981	0.066745572	-6.627254685	-6.031899615	-4.243351461	-7.100193044	-4.874174167	-5.146432944	2.523320438	0.117127588

NPS	1.667345384	1.85337356	1.195570971	0.186028175	-0.471774413	-0.657802588	8	1.350464443	0.816496581	0.816496581	0.816496581	0.816496581	0.816496581	0.816496581	25	82	0	P_107358	P_107358	P_107358	chr10	127552552	127552612	+	NPS	neuropeptide S [Source:HGNC Symbol;Acc:HGNC:33940]	ENSG00000214285	protein_coding	exon	coding exon	1	4162	10:127552552:127552612	ENSG00000214285	NPS	2.39408573	1.46678076	1.686120462	1.8742434	1.209033652	0.188122938	-0.47708681	-0.665209749	9.961963646	0.122786324	0.090488366	0.254562491	0.854557075	0.643593742	0.521018754	-5.635086198	-4.606863487	-6.302636087	-7.130293829	-6.177029195	-6.834889009	1.42050983	0.294122251

EPB41L1	0.096108533	0.151350094	-0.010278465	0.055241561	-0.106386998	-0.161628559	8	0.082060534	0.816496581	0.816496581	0.816496581	0.816496581	0.816496581	0.816496581	25	83	0	P_131118	P_131118	P_131118	chr20	36229585	36229645	+	EPB41L1	erythrocyte membrane protein band 4.1-like 1 [Source:HGNC Symbol;Acc:HGNC:3378]	ENSG00000088367	protein_coding	3'UTR	3'UTR	1	4163	20:36229585:36229645	ENSG00000088367	EPB41L1	7.559076888	0.007614398	1.34893118	2.124274035	-0.144263388	0.775342855	-1.493194568	-2.268537424	9.961963646	0.207221046	0.059696318	0.888169211	0.45614772	0.166364987	0.046782038	-6.087034114	-4.237216093	-7.035766941	-6.830455765	-5.23033841	-4.808709981	2.388336006	0.130149243

ATM	-0.02306718	-0.191356957	-0.212510067	-0.168289777	-0.189442887	-0.02115311	8	0.049771005	0.816496581	0.816496581	0.816496581	0.816496581	0.816496581	0.816496581	25	85	0	P_213247	P_213247	P_213247	chr11	108354814	108354874	+	ATM	ATM serine/threonine kinase [Source:HGNC Symbol;Acc:HGNC:795]	ENSG00000149311	protein_coding	exon	coding exon	1	4165	11:108354814:108354874	ENSG00000149311	ATM	6.819058072	0.004195973	-0.436137873	-3.618041551	-4.017989544	-3.181903678	-3.581851672	-0.399947993	9.961963646	0.672036608	0.004733997	0.002465157	0.009833297	0.005026515	0.697636058	-6.895547343	-1.876953234	-1.832236178	-3.339505999	-1.990466831	-6.984691753	8.756468617	0.003820661

C3orf84	-0.311799239	0.250090765	0.153468034	0.561890004	0.465267273	-0.096622731	8	0.47504978	0.816496581	0.816496581	0.816496581	0.816496581	0.816496581	0.816496581	25	88	0	P_134839	P_134839	P_134839	chr3	49189850	49189910	-	C3orf84	chromosome 3 open reading frame 84 [Source:HGNC Symbol;Acc:HGNC:44666]	ENSG00000236980	protein_coding	exon	non-coding exon	1	4168	3:49189850:49189910	ENSG00000236980	C3orf84	3.428014033	0.183433841	-0.891622358	0.715160558	0.438857807	1.606782916	1.330480165	-0.276302751	9.961963646	0.393621526	0.490930474	0.670129759	0.139298521	0.213012206	0.787963022	-6.581115473	-5.919435894	-6.942953107	-5.895121238	-5.432533387	-7.029832084	0.987885541	0.437397833

VPS4A	-0.048324881	0.02225017	0.056567077	0.070575051	0.104891957	0.034316907	8	0.148725947	0.816496581	0.816496581	0.816496581	0.816496581	0.816496581	0.816496581	25	89	0	A_23_P329772	A_23_P329772	A_23_P329772	chr16	69324814	69324874	+	VPS4A	vacuolar protein sorting 4 homolog A (S. cerevisiae) [Source:HGNC Symbol;Acc:HGNC:13488]	ENSG00000132612	protein_coding	3'UTR;exon	multiple Ref	1	4169	16:69324814:69324874	ENSG00000132612	VPS4A	9.682142171	0.019969774	-0.418822345	0.192837896	0.490255857	0.611660241	0.909078201	0.297417961	9.961963646	0.684232727	0.850960551	0.63457057	0.554463961	0.384774817	0.772257039	-6.903479051	-6.162521804	-6.917432532	-6.948785838	-5.874978862	-7.023268711	0.290326381	0.831471411

RNF24	0.106804773	0.053303239	0.151919497	-0.053501534	0.045114724	0.098616258	8	0.059094329	0.816496581	0.816496581	0.816496581	0.816496581	0.816496581	0.816496581	25	90	0	A_24_P915904	A_24_P915904	A_24_P915904	chr20	3931157	3931217	-	RNF24	ring finger protein 24 [Source:HGNC Symbol;Acc:HGNC:13779]	ENSG00000101236	protein_coding	3'UTR	3'UTR	1	4170	20:3931157:3931217	ENSG00000101236	RNF24	7.082794292	0.005011063	1.847869851	0.922219534	2.628416789	-0.925650316	0.780546938	1.706197255	9.961963646	0.094487785	0.378208081	0.025305267	0.376506918	0.453216714	0.118897199	-5.398281005	-5.752604682	-4.187888359	-6.700222563	-5.982470727	-5.677559695	2.591812962	0.111109438

DENND6A	-0.037321759	-0.079574948	-0.111208929	-0.042253189	-0.07388717	-0.031633981	8	0.066728287	0.816496581	0.816496581	0.816496581	0.816496581	0.816496581	0.816496581	25	92	0	P_244657	P_244657	P_244657	chr3	57663635	57663695	-	DENND6A	DENN/MADD domain containing 6A [Source:HGNC Symbol;Acc:HGNC:26635]	ENSG00000174839	protein_coding	exon	coding exon	1	4172	3:57663635:57663695	ENSG00000174839	DENND6A	7.406565533	0.005782416	-0.601108758	-1.281643735	-1.79114446	-0.680534976	-1.190035702	-0.509500726	9.961963646	0.561195182	0.228993456	0.103649118	0.511671875	0.26161961	0.621496862	-6.804704657	-5.383233553	-5.530018201	-6.902089299	-5.594011417	-6.93139214	1.225174116	0.351105202

TUT4	0.001765375	-0.199729789	-0.127837114	-0.201495164	-0.129602489	0.071892675	8	0.037858838	0.816496581	0.816496581	0.816496581	0.816496581	0.816496581	0.816496581	25	94	0	P_201727	P_201727	P_201727	chr1	52481435	52481495	-	ZCCHC11	"zinc finger, CCHC domain containing 11 [Source:HGNC Symbol;Acc:HGNC:28981]"	ENSG00000134744	protein_coding	3'UTR;exon	multiple Ref	1	4174	1:52481435:52481495	ENSG00000134744	TUT4	8.118209543	0.003357695	0.037313154	-4.221510088	-2.701978857	-4.258823242	-2.739292011	1.519531231	9.961963646	0.970972448	0.001782374	0.022308078	0.001680496	0.020926325	0.159709665	-6.99760062	-0.956098499	-4.063545763	-1.525916886	-3.354969476	-5.937784085	8.845583454	0.003687309

TEX47	-0.11292724	-0.506154113	-0.194616465	-0.393226873	-0.081689225	0.311537648	8	0.376350047	0.816496581	0.816496581	0.816496581	0.816496581	0.816496581	0.816496581	25	96	0	A_32_P113173	A_32_P113173	A_32_P113173	chr7	88797903	88797963	+	C7orf62;ZNF804B	chromosome 7 open reading frame 62 [Source:HGNC Symbol;Acc:HGNC:22402];zinc finger protein 804B [Source:HGNC Symbol;Acc:HGNC:21958]	ENSG00000164645	protein_coding	intron;promoter	multiple non-Ref	0	4176	7:88797903:88797963	ENSG00000164645	TEX47	3.461506454	0.115950811	-0.40616963	-1.82050344	-0.699984323	-1.41433381	-0.293814692	1.120519118	9.961963646	0.693205501	0.09881011	0.49995659	0.187748495	0.774929833	0.288778353	-6.909078737	-4.683722778	-6.785958209	-6.153315145	-6.250973425	-6.42564698	1.218569149	0.353237007

CEP350	-0.027035254	-0.138776499	-0.052260354	-0.111741246	-0.025225101	0.086516145	8	0.06646631	0.816496581	0.816496581	0.816496581	0.816496581	0.816496581	0.816496581	25	97	0	P_205162	P_205162	P_205162	chr1	180012014	180012074	+	CEP350	centrosomal protein 350kDa [Source:HGNC Symbol;Acc:HGNC:24238]	ENSG00000135837	protein_coding	exon	coding exon	1	4177	1:180012014:180012074	ENSG00000135837	CEP350	7.914037491	0.005754394	-0.436491973	-2.240586643	-0.84375851	-1.80409467	-0.407266537	1.396828133	9.961963646	0.671788222	0.049052589	0.418604178	0.101488404	0.692425621	0.192801942	-6.895381912	-4.05972438	-6.671691659	-5.610901803	-6.209213881	-6.098608929	1.882405769	0.19669913

TBXAS1	-0.257967318	-0.121120904	-0.310692178	0.136846414	-0.05272486	-0.189571274	8	0.099840662	0.816496581	0.816496581	0.816496581	0.816496581	0.816496581	0.816496581	25	98	0	A_14_P100560	A_14_P100560	A_14_P100560	chr7	139957676	139957736	+	TBXAS1	thromboxane A synthase 1 (platelet) [Source:HGNC Symbol;Acc:HGNC:11609]	ENSG00000059377	protein_coding	3'UTR;exon	multiple Ref	1	4178	7:139957676:139957736	ENSG00000059377	TBXAS1	5.740095906	0.010211658	-3.126527008	-1.467968039	-3.765544772	1.658558968	-0.639017764	-2.297576732	9.961963646	0.010801918	0.172964293	0.003713222	0.128312089	0.537226354	0.044531134	-3.294661295	-5.158147896	-2.253190355	-5.822302096	-6.083970106	-4.761329766	5.757908186	0.015029341

GIMAP2	-0.116018067	0.199952228	-0.196801507	0.315970295	-0.08078344	-0.396753735	8	0.120184695	0.816496581	0.816496581	0.816496581	0.816496581	0.816496581	0.816496581	25	99	0	P_146274	P_146274	P_146274	chr7	150693341	150693401	+	GIMAP2	"GTPase, IMAP family member 2 [Source:HGNC Symbol;Acc:HGNC:21789]"	ENSG00000106560	protein_coding	3'UTR	3'UTR	1	4179	7:150693341:150693401	ENSG00000106560	GIMAP2	6.620699838	0.013806293	-1.209296254	2.08417091	-2.051329859	3.293467164	-0.842033606	-4.13550077	9.961963646	0.254466025	0.063854956	0.067464662	0.00814317	0.419524291	0.002042821	-6.254184289	-4.297685429	-5.133057301	-3.148229606	-5.932808054	-1.659536976	6.44540553	0.010609124

BCKDHB	0.009602435	-0.090986068	-0.140569226	-0.100588503	-0.150171661	-0.049583157	8	0.16583037	0.816496581	0.816496581	0.816496581	0.816496581	0.816496581	0.816496581	25	105	0	A_24_P239664	A_24_P239664	A_24_P239664	chr6	80346122	80346182	+	BCKDHB	"branched chain keto acid dehydrogenase E1, beta polypeptide [Source:HGNC Symbol;Acc:HGNC:987]"	ENSG00000083123	protein_coding	3'UTR;exon	multiple Ref	1	4185	6:80346122:80346182	ENSG00000083123	BCKDHB	6.927157192	0.024290452	0.075458681	-0.714994552	-1.104633187	-0.790453233	-1.180091868	-0.389638635	9.961963646	0.941343615	0.491028656	0.295283908	0.447671424	0.265375279	0.705003815	-6.995254034	-5.919554686	-6.418786276	-6.818271558	-5.604946852	-6.989069735	0.651062849	0.600210757

DRP2	-0.043431699	-0.115780585	0.113047624	-0.072348886	0.156479324	0.228828209	8	0.191186037	0.816496581	0.816496581	0.816496581	0.816496581	0.816496581	0.816496581	25	106	0	P_270944	P_270944	P_270944	chrX	101245017	101245077	+	DRP2	dystrophin related protein 2 [Source:HGNC Symbol;Acc:HGNC:3032]	ENSG00000102385	protein_coding	3'UTR;exon	multiple Ref	1	4186	X:101245017:101245077	ENSG00000102385	DRP2	4.963761266	0.031560014	-0.299422103	-0.798201932	0.779360653	-0.498779829	1.078782756	1.577562585	9.961963646	0.770771776	0.443365107	0.453883761	0.628763292	0.306111671	0.145861619	-6.94965767	-5.856946339	-6.725203049	-7.015244016	-5.712370011	-5.858785605	0.872573368	0.487342011

ADGRE2	0.088238189	0.309207187	0.218729118	0.220968999	0.13049093	-0.090478069	8	0.346064273	0.816496581	0.816496581	0.816496581	0.816496581	0.816496581	0.816496581	25	107	0	P_124896	P_124896	P_124896	chr19	14732476	14732536	-	EMR2	"egf-like module containing, mucin-like, hormone receptor-like 2 [Source:HGNC Symbol;Acc:HGNC:3337]"	ENSG00000127507	protein_coding	3'UTR	3'UTR	1	4187	19:14732476:14732536	ENSG00000127507	ADGRE2	4.215497538	0.09838088	0.344545722	1.207368547	0.854076712	0.862822825	0.509530989	-0.353291835	9.961963646	0.737598816	0.25517485	0.413128873	0.408526352	0.62147641	0.731231458	-6.933966304	-5.467025699	-6.66277478	-6.757073024	-6.160423417	-7.003618211	0.572466295	0.645918166

FTCD	0.040247665	-0.026442024	-0.109717854	-0.066689689	-0.149965519	-0.08327583	8	0.109958897	0.816496581	0.816496581	0.816496581	0.816496581	0.816496581	0.816496581	25	108	0	P_132489	P_132489	P_132489	chr21	46136787	46136847	-	FTCD	formimidoyltransferase cyclodeaminase [Source:HGNC Symbol;Acc:HGNC:3974]	ENSG00000160282	protein_coding	"3'UTR;3'UTR,exon"	multiple Ref	1	4188	21:46136787:46136847	ENSG00000160282	FTCD	6.455813379	0.011916383	0.451558567	-0.296666217	-1.23097916	-0.748224784	-1.682537727	-0.934312943	9.961963646	0.661258396	0.772814403	0.246601837	0.47161947	0.123492294	0.372235847	-6.888223501	-6.135902874	-6.277222947	-6.851798618	-4.977379434	-6.614376175	1.011823313	0.427726939

SMARCAD1	0.073780091	-0.124659998	-0.060594966	-0.19844009	-0.134375057	0.064065032	8	0.04248497	0.816496581	0.816496581	0.816496581	0.816496581	0.816496581	0.816496581	25	109	0	P_250064	P_250064	P_250064	chr4	94281530	94281590	+	SMARCAD1	"SWI/SNF-related, matrix-associated actin-dependent regulator of chromatin, subfamily a, containing DEAD/H box 1 [Source:HGNC Symbol;Acc:HGNC:18398]"	ENSG00000163104	protein_coding	3'UTR;exon	multiple Ref	1	4189	4:94281530:94281590	ENSG00000163104	SMARCAD1	9.261493799	0.003656175	1.494415299	-2.524987503	-1.227350666	-4.019402802	-2.721765966	1.297636836	9.961963646	0.166051257	0.030209409	0.247904001	0.002459566	0.021564316	0.22365422	-5.899751145	-3.615528222	-6.281448014	-1.920303346	-3.383378124	-6.221936167	5.893783142	0.014002768

RHO	0.009665044	0.007056045	-0.041641839	-0.002608999	-0.051306882	-0.048697883	8	0.194456205	0.816496581	0.816496581	0.816496581	0.816496581	0.816496581	0.816496581	25	110	0	A_24_P26181	A_24_P26181	A_24_P26181	chr3	129532741	129533636	+	RHO	rhodopsin [Source:HGNC Symbol;Acc:HGNC:10012]	ENSG00000163914	protein_coding	"exon,intron"	multiple non-Ref	0	4190	3:129532741:129533636	ENSG00000163914	RHO	4.728659317	0.032572758	0.065587667	0.047882817	-0.28258445	-0.01770485	-0.348172117	-0.330467267	9.961963646	0.949003656	0.962755988	0.783279747	0.986223889	0.734956161	0.747891938	-6.996013401	-6.180878889	-7.003686676	-7.149441644	-6.232610017	-7.012044421	0.05286573	0.983043098

ZNF32	0.113258451	0.038455294	0.084454615	-0.074803157	-0.028803836	0.045999321	8	0.077935257	0.816496581	0.816496581	0.816496581	0.816496581	0.816496581	0.816496581	25	112	0	P_105860	P_105860	P_105860	chr10	43643912	43643972	-	ZNF32	zinc finger protein 32 [Source:HGNC Symbol;Acc:HGNC:13095]	ENSG00000169740	protein_coding	3'UTR	3'UTR	1	4192	10:43643912:43643972	ENSG00000169740	ZNF32	11.3460866	0.00708436	1.648033087	0.559566159	1.228906081	-1.088466928	-0.419127006	0.669339922	9.961963646	0.130480666	0.588134689	0.247345127	0.302020148	0.684017301	0.518489777	-5.689143424	-6.019669631	-6.279637989	-6.537644468	-6.204091419	-6.832007567	1.057963521	0.409728593

LITAF	-0.169081425	0.103731958	0.139871926	0.272813382	0.30895335	0.036139968	8	0.178953238	0.816496581	0.816496581	0.816496581	0.816496581	0.816496581	0.816496581	25	114	0	P_118273	P_118273	P_118273	chr16	11549296	11549356	-	LITAF	lipopolysaccharide-induced TNF factor [Source:HGNC Symbol;Acc:HGNC:16841]	ENSG00000189067	protein_coding	3'UTR	3'UTR	1	4194	16:11549296:11549356	ENSG00000189067	LITAF	7.825533224	0.027923912	-1.239234221	0.760273888	1.025151505	1.999508108	2.264385726	0.264877618	9.961963646	0.243659963	0.464704043	0.329540485	0.073556876	0.047112737	0.796503593	-6.219457471	-5.886241511	-6.501703129	-5.313486085	-4.113938569	-7.033185079	2.060048165	0.16959073

SLC12A9	0.052306139	0.041647766	-0.00065111	-0.010658373	-0.052957249	-0.042298876	8	0.107439694	0.816496581	0.816496581	0.816496581	0.816496581	0.816496581	0.816496581	25	115	0	P_145480	P_145480	P_145480	chr7	100860510	100860570	+	SLC12A9	"solute carrier family 12, member 9 [Source:HGNC Symbol;Acc:HGNC:17435]"	ENSG00000146828	protein_coding	exon	non-coding exon	1	4195	7:100860510:100860570	ENSG00000146828	SLC12A9	7.262033891	0.011476574	0.597987589	0.476136214	-0.007443786	-0.121851375	-0.605431375	-0.48358	9.961963646	0.56319504	0.644247454	0.99420773	0.905439669	0.558432083	0.639137014	-6.806675247	-6.063970214	-7.047090343	-7.141499403	-6.10534126	-6.945112487	0.199932464	0.894056075

CD5	0.197061701	0.069633429	0.012239106	-0.127428272	-0.184822595	-0.057394323	8	0.173675822	0.816496581	0.816496581	0.816496581	0.816496581	0.816496581	0.816496581	25	117	0	P_108734	P_108734	P_108734	chr11	61126682	61126742	+	CD5	CD5 molecule [Source:HGNC Symbol;Acc:HGNC:1685]	ENSG00000110448	protein_coding	3'UTR	3'UTR	1	4197	11:61126682:61126742	ENSG00000110448	CD5	4.913683044	0.026429451	1.484580601	0.524589187	0.092204315	-0.959991414	-1.392376286	-0.432384872	9.961963646	0.168593453	0.611342211	0.928363554	0.35977977	0.194105353	0.674671714	-5.912802793	-6.039062947	-7.042479767	-6.667729531	-5.357424203	-6.970196692	0.92248782	0.465024248

YTHDC2	-0.12967089	-0.094794824	-0.096292073	0.034876066	0.033378817	-0.001497249	8	0.052982012	0.816496581	0.816496581	0.816496581	0.816496581	0.816496581	0.816496581	25	118	0	P_254361	P_254361	P_254361	chr5	113553299	113553359	+	YTHDC2	YTH domain containing 2 [Source:HGNC Symbol;Acc:HGNC:24721]	ENSG00000047188	protein_coding	3'UTR;exon	multiple Ref	1	4198	5:113553299:113553359	ENSG00000047188	YTHDC2	7.67481691	0.004460933	-2.377799648	-1.738270632	-1.765725964	0.639529016	0.612073684	-0.027455332	9.961963646	0.038847123	0.112915111	0.108013227	0.536907204	0.554201142	0.978638782	-4.55995269	-4.799243796	-5.56740562	-6.930442592	-6.10119927	-7.07096674	2.096281582	0.164610442

SYNPO2	-0.023846665	-0.048678345	-0.079746998	-0.02483168	-0.055900333	-0.031068653	8	0.120458839	0.816496581	0.816496581	0.816496581	0.816496581	0.816496581	0.816496581	25	121	0	P_250605	P_250605	P_250605	chr4	119027094	119027154	+	SYNPO2	synaptopodin 2 [Source:HGNC Symbol;Acc:HGNC:17732]	ENSG00000172403	protein_coding	exon	coding exon	1	4201	4:119027094:119027154	ENSG00000172403	SYNPO2	3.468466338	0.013859272	-0.248086468	-0.506420443	-0.829640159	-0.258333975	-0.58155369	-0.323219716	9.961963646	0.809106415	0.623580303	0.426175673	0.801407941	0.57379009	0.753211305	-6.964878824	-6.048670622	-6.683740372	-7.113239573	-6.119884112	-7.014604948	0.252620579	0.857724538

CXCL12	0.009993843	-0.205943148	0.109234853	-0.215936991	0.09924101	0.315178001	8	0.217811559	0.816496581	0.816496581	0.816496581	0.816496581	0.816496581	0.816496581	25	122	0	P_105865	P_105865	P_105865	chr10	44373039	44373099	-	CXCL12	chemokine (C-X-C motif) ligand 12 [Source:HGNC Symbol;Acc:HGNC:10672]	ENSG00000107562	protein_coding	3'UTR	3'UTR	1	4202	10:44373039:44373099	ENSG00000107562	CXCL12	4.064649296	0.040305097	0.060967467	-1.256356799	0.66638755	-1.317324266	0.605420083	1.922744349	9.961963646	0.952590918	0.237648827	0.520296818	0.217223358	0.558439291	0.083541102	-6.996332344	-5.412162446	-6.809896837	-6.275218616	-6.105348266	-5.355835837	1.304174056	0.326687851

WSCD1	-0.167572659	-0.19663137	0.529618013	-0.029058711	0.697190673	0.726249384	8	0.295332022	0.816496581	0.816496581	0.816496581	0.816496581	0.816496581	0.816496581	25	123	0	P_227579	P_227579	P_227579	chr17	6088030	6088090	+	WSCD1	WSC domain containing 1 [Source:HGNC Symbol;Acc:HGNC:29060]	ENSG00000179314	protein_coding	"5'UTR,exon;exon"	multiple Ref	1	4203	17:6088030:6088090	ENSG00000179314	WSCD1	3.775604498	0.072249905	-0.76353711	-0.895941788	2.413180103	-0.132404678	3.176717213	3.309121891	9.961963646	0.462842429	0.391419244	0.036571831	0.897300371	0.009920114	0.007931122	-6.689261889	-5.775781274	-4.547874552	-7.140034424	-2.64370354	-3.049300336	4.71141496	0.026852958

PDLIM3	0.052435898	0.067418435	0.18669918	0.014982537	0.134263282	0.119280744	8	0.12015179	0.816496581	0.816496581	0.816496581	0.816496581	0.816496581	0.816496581	25	124	0	P_251897	P_251897	P_251897	chr4	185502293	185502353	-	PDLIM3	PDZ and LIM domain 3 [Source:HGNC Symbol;Acc:HGNC:20767]	ENSG00000154553	protein_coding	exon	coding exon	1	4204	4:185502293:185502353	ENSG00000154553	PDLIM3	4.377561591	0.013799943	0.546683163	0.702887239	1.946477551	0.156204076	1.399794388	1.243590313	9.961963646	0.596627861	0.498222223	0.080326046	0.878992248	0.191937612	0.242119091	-6.837691475	-5.928151679	-5.295938728	-7.136286089	-5.348269761	-6.28642675	1.352004786	0.312830823

HOXA13	-0.142216682	-0.088754648	0.25741662	0.053462034	0.399633302	0.346171268	8	0.298126811	0.816496581	0.816496581	0.816496581	0.816496581	0.816496581	0.816496581	25	125	0	A_23_P389281	A_23_P389281	A_23_P389281	chr7	27197054	27197114	-	HOXA13	homeobox A13 [Source:HGNC Symbol;Acc:HGNC:5102]	ENSG00000106031	protein_coding	3'UTR	3'UTR	1	4205	7:27197054:27197114	ENSG00000106031	HOXA13	3.646711447	0.073581846	-0.642112069	-0.400729576	1.162242829	0.241382493	1.804354898	1.562972405	9.961963646	0.535296417	0.697078817	0.272224125	0.814154448	0.101445413	0.149239927	-6.777935565	-6.098135284	-6.355678536	-7.117842987	-4.806046778	-5.878816119	1.28009089	0.333924528

ANGPTL5	0.11802468	-0.10755878	0.27130499	-0.22558346	0.15328031	0.37886377	8	0.123882237	0.816496581	0.816496581	0.816496581	0.816496581	0.816496581	0.816496581	25	128	0	P_212909	P_212909	P_212909	chr11	101900429	101900489	-	ANGPTL5	angiopoietin-like 5 [Source:HGNC Symbol;Acc:HGNC:19705]	ENSG00000187151	protein_coding	exon	coding exon	1	4208	11:101900429:101900489	ENSG00000187151	ANGPTL5	3.606090133	0.014531008	1.199141928	-1.09280739	2.756484398	-2.291949318	1.55734247	3.849291788	9.961963646	0.258217648	0.300200023	0.020318883	0.044958945	0.150561853	0.003238576	-6.265820084	-5.588949743	-3.971094898	-4.84581265	-5.146616767	-2.134282824	5.454295017	0.017667519

MATN1	0.066085023	0.006904994	0.161645475	-0.059180029	0.095560452	0.154740482	8	0.139286258	0.816496581	0.816496581	0.816496581	0.816496581	0.816496581	0.816496581	25	129	0	P_101078	P_101078	P_101078	chr1	30718736	30718796	-	MATN1	"matrilin 1, cartilage matrix protein [Source:HGNC Symbol;Acc:HGNC:6907]"	ENSG00000162510	protein_coding	exon	coding exon	1	4209	1:30718736:30718796	ENSG00000162510	MATN1	4.658435952	0.017786473	0.606881301	0.063410912	1.484445519	-0.54347039	0.877564218	1.421034607	9.961963646	0.557506978	0.95069361	0.168628604	0.598755925	0.400849083	0.185844234	-6.801034976	-6.179968142	-5.960822453	-6.990452885	-5.902630955	-6.067580888	0.943453789	0.455971639

UBE2E2	-0.216251088	0.02448377	-0.224310527	0.240734859	-0.008059439	-0.248794297	8	0.043291837	0.816496581	0.816496581	0.816496581	0.816496581	0.816496581	0.816496581	25	130	0	A_16_P36196044	A_16_P36196044	A_16_P36196044	chr3	23271010	23271070	+	UBE2E2	ubiquitin-conjugating enzyme E2E 2 [Source:HGNC Symbol;Acc:HGNC:12478]	ENSG00000182247	protein_coding	3'UTR	3'UTR	1	4210	3:23271010:23271070	ENSG00000182247	UBE2E2	9.040812189	0.003711755	-4.347246509	0.492191677	-4.509263574	4.839438187	-0.162017064	-5.001455251	9.961963646	0.001462828	0.633249414	0.001137707	0.000689542	0.874531408	0.000542411	-1.25260466	-6.055970652	-1.033402437	-0.599064967	-6.282742456	-0.284188266	14.65587149	0.000552066

CEP97	-0.028190508	-0.110250413	-0.111181893	-0.082059905	-0.082991386	-0.00093148	8	0.070071898	0.816496581	0.816496581	0.816496581	0.816496581	0.816496581	0.816496581	25	131	0	P_245293	P_245293	P_245293	chr3	101757125	101757185	+	CEP97	centrosomal protein 97kDa [Source:HGNC Symbol;Acc:HGNC:26244]	ENSG00000182504	protein_coding	3'UTR;exon	multiple Ref	1	4211	3:101757125:101757185	ENSG00000182504	CEP97	7.7381922	0.006149739	-0.44027112	-1.721858765	-1.736406351	-1.281587645	-1.296135231	-0.014547586	9.961963646	0.669139904	0.115941124	0.113255195	0.229012364	0.224151115	0.988680339	-6.893608308	-4.821978014	-5.610185198	-6.318602728	-5.473198901	-7.071262911	1.58273139	0.254671573

MBTPS2	-0.165670626	0.04800285	-0.15508793	0.213673476	0.010582696	-0.20309078	8	0.127038391	0.816496581	0.816496581	0.816496581	0.816496581	0.816496581	0.816496581	25	133	0	A_23_P114095	A_23_P114095	A_23_P114095	chrX	21882630	21882690	+	MBTPS2	"membrane-bound transcription factor peptidase, site 2 [Source:HGNC Symbol;Acc:HGNC:15455]"	ENSG00000012174	protein_coding	"3'UTR,exon"	multiple Ref	1	4213	X:21882630:21882690	ENSG00000012174	MBTPS2	7.924434815	0.015166982	-1.647561179	0.477378729	-1.54231839	2.124939909	0.105242789	-2.019697119	9.961963646	0.130578655	0.643393057	0.15414011	0.059629502	0.918272069	0.07112403	-5.68980884	-6.063360159	-5.883180006	-5.115757484	-6.290797438	-5.206044842	2.321119403	0.137264555

BPTF	0.105611354	-0.213831854	-0.005652137	-0.319443209	-0.111263491	0.208179718	8	0.051771815	0.816496581	0.816496581	0.816496581	0.816496581	0.816496581	0.816496581	25	134	0	A_23_P114075	A_23_P114075	A_23_P114075	chr17	67886166	67886226	+	BPTF	bromodomain PHD finger transcription factor [Source:HGNC Symbol;Acc:HGNC:3581]	ENSG00000171634	protein_coding	exon	coding exon	1	4214	17:67886166:67886226	ENSG00000171634	BPTF	8.768383725	0.004359128	1.959099267	-3.966598399	-0.104847598	-5.925697666	-2.063946865	3.861750801	9.961963646	0.078663805	0.002677822	0.918577724	0.00014817	0.066055507	0.003173577	-5.229364885	-1.339268832	-7.041120537	1.008820975	-4.422854875	-2.113442892	12.31164242	0.001092016

PRSS33	-0.011233193	0.112581063	0.150028163	0.123814256	0.161261356	0.037447101	8	0.194899828	0.816496581	0.816496581	0.816496581	0.816496581	0.816496581	0.816496581	25	135	0	P_118010	P_118010	P_118010	chr16	2784643	2784703	-	PRSS33	"protease, serine, 33 [Source:HGNC Symbol;Acc:HGNC:30405]"	ENSG00000103355	protein_coding	"3'UTR;exon;exon,exon"	multiple Ref	1	4215	16:2784643:2784703	ENSG00000103355	PRSS33	4.692463089	0.032711467	-0.076067448	0.762361507	1.015940817	0.838428955	1.092008265	0.25357931	9.961963646	0.940871401	0.463512536	0.333696292	0.421451546	0.300534491	0.804977182	-6.995203727	-5.88466178	-6.510990925	-6.778221716	-5.698768079	-7.03636355	0.59647745	0.631638752

ARRB1	-0.164160797	0.172025719	-0.019041805	0.336186516	0.145118992	-0.191067524	8	0.128798704	0.816496581	0.816496581	0.816496581	0.816496581	0.816496581	0.816496581	25	137	0	A_16_P02471013	A_16_P02471013	A_16_P02471013	chr11	75293225	75293285	+	ARRB1	"arrestin, beta 1 [Source:HGNC Symbol;Acc:HGNC:711]"	ENSG00000137486	protein_coding	intron	intron antisense	0	4217	11:75293225:75293285	ENSG00000137486	ARRB1	7.929287587	0.015528641	-1.61342343	1.690722336	-0.18714879	3.304145766	1.426274641	-1.877871125	9.961963646	0.137844091	0.121884874	0.855300585	0.007997902	0.184366613	0.08995069	-5.737654986	-4.864800482	-7.028027067	-3.129936503	-5.31533185	-5.424041213	3.662456735	0.051720534

BAZ1A	-0.018586277	0.072277669	0.037728364	0.090863946	0.056314641	-0.034549305	8	0.041759157	0.816496581	0.816496581	0.816496581	0.816496581	0.816496581	0.816496581	25	138	0	P_220457	P_220457	P_220457	chr14	34807455	34807515	-	BAZ1A	"bromodomain adjacent to zinc finger domain, 1A [Source:HGNC Symbol;Acc:HGNC:960]"	ENSG00000198604	protein_coding	exon	coding exon	1	4218	14:34807455:34807515	ENSG00000198604	BAZ1A	9.609216699	0.003607072	-0.379018675	1.473914676	0.769371648	1.852933352	1.148390323	-0.704543029	9.961963646	0.712627155	0.171388577	0.459525931	0.093707456	0.277635246	0.49723462	-6.920533234	-5.150639269	-6.733168293	-5.537922982	-5.639350146	-6.806789754	1.35942593	0.310740637

PFN1	-0.199750069	-0.073690987	-0.171657946	0.126059082	0.028092122	-0.097966959	8	0.076908934	0.816496581	0.816496581	0.816496581	0.816496581	0.816496581	0.816496581	25	141	0	P_120208	P_120208	P_120208	chr17	4948645	4948705	-	PFN1	profilin 1 [Source:HGNC Symbol;Acc:HGNC:8881]	ENSG00000108518	protein_coding	5'UTR	5'UTR	1	4221	17:4948645:4948705	ENSG00000108518	PFN1	9.374370345	0.006956739	-2.933119239	-1.082074483	-2.520616028	1.851044757	0.412503211	-1.438541545	9.961963646	0.015018301	0.304716188	0.030436267	0.093997805	0.688707657	0.180946757	-3.624949943	-5.599895285	-4.369001494	-5.540762804	-6.206969692	-6.044921315	3.632251031	0.052773014

MEOX2	-0.119650718	-0.038725331	0.058509559	0.080925387	0.178160277	0.09723489	8	0.269557856	0.816496581	0.816496581	0.816496581	0.816496581	0.816496581	0.816496581	25	142	0	P_260561	P_260561	P_260561	chr7	15686253	15686313	-	MEOX2	mesenchyme homeobox 2 [Source:HGNC Symbol;Acc:HGNC:7014]	ENSG00000106511	protein_coding	exon	coding exon	1	4222	7:15686253:15686313	ENSG00000106511	MEOX2	3.79602734	0.06055778	-0.595492044	-0.192732872	0.29119739	0.402759172	0.886689434	0.483930262	9.961963646	0.564796891	0.851040624	0.776873207	0.695632669	0.396147192	0.63889703	-6.808243976	-6.162543068	-7.001010111	-7.061621956	-5.894709015	-6.944931634	0.276529043	0.841081256

AARS1	-0.058862507	-0.129327851	-0.092412002	-0.070465344	-0.033549496	0.036915849	8	0.054161606	0.816496581	0.816496581	0.816496581	0.816496581	0.816496581	0.816496581	25	143	0	P_226805	P_226805	P_226805	chr16	70264957	70265017	-	AARS	alanyl-tRNA synthetase [Source:HGNC Symbol;Acc:HGNC:20]	ENSG00000090861	protein_coding	exon	coding exon	1	4223	16:70264957:70265017	ENSG00000090861	AARS1	9.968752313	0.004562428	-1.067299655	-2.34498289	-1.675621779	-1.277683234	-0.608322124	0.66936111	9.961963646	0.311018103	0.04108111	0.12486542	0.230331737	0.556588537	0.518476822	-6.410060465	-3.898115362	-5.697644913	-6.323291187	-6.103543799	-6.831992743	1.982726034	0.180818129

TRPM7	-0.053212987	-0.090995549	-0.124012481	-0.037782562	-0.070799495	-0.033016932	8	0.061905395	0.816496581	0.816496581	0.816496581	0.816496581	0.816496581	0.816496581	25	145	0	P_223470	P_223470	P_223470	chr15	50628148	50628208	-	TRPM7	"transient receptor potential cation channel, subfamily M, member 7 [Source:HGNC Symbol;Acc:HGNC:17994]"	ENSG00000092439	protein_coding	exon	coding exon	1	4225	15:50628148:50628208	ENSG00000092439	TRPM7	7.720495003	0.005284212	-0.896547167	-1.533118258	-2.089396697	-0.636571091	-1.19284953	-0.556278439	9.961963646	0.391111281	0.156367955	0.063297796	0.538755218	0.260564599	0.590295986	-6.576669786	-5.074869866	-5.073065341	-6.932425265	-5.590904632	-6.904917895	1.609564238	0.248746004

LARP4B	-0.009462039	-0.01981508	0.006372032	-0.010353041	0.015834071	0.026187112	8	0.066407224	0.816496581	0.816496581	0.816496581	0.816496581	0.816496581	0.816496581	25	146	0	P_207181	P_207181	P_207181	chr10	842984	843044	-	LARP4B	"La ribonucleoprotein domain family, member 4B [Source:HGNC Symbol;Acc:HGNC:28987]"	ENSG00000107929	protein_coding	exon	coding exon	1	4226	10:842984:843044	ENSG00000107929	LARP4B	8.253539241	0.00574809	-0.152851114	-0.320095592	0.1029347	-0.167244479	0.255785813	0.423030292	9.961963646	0.881567317	0.755508473	0.920057423	0.870523863	0.80332021	0.681259928	-6.985626107	-6.128360214	-7.041337361	-7.134338378	-6.2620034	-6.974488845	0.068128655	0.975620807

AKAP13	0.070105176	-0.084857962	-0.03983897	-0.154963138	-0.109944146	0.045018992	8	0.056484538	0.816496581	0.816496581	0.816496581	0.816496581	0.816496581	0.816496581	25	147	0	P_224841	P_224841	P_224841	chr15	85718088	85718148	+	AKAP13	A kinase (PRKA) anchor protein 13 [Source:HGNC Symbol;Acc:HGNC:371]	ENSG00000170776	protein_coding	exon	coding exon	1	4227	15:85718088:85718148	ENSG00000170776	AKAP13	7.741092768	0.004768832	1.243339436	-1.504985175	-0.706557853	-2.74832461	-1.949897289	0.798427321	9.961963646	0.242207618	0.163356212	0.496034496	0.020604946	0.079872453	0.443240258	-6.214647086	-5.111102225	-6.781149508	-4.081759475	-4.593912353	-6.733882399	2.717161853	0.101013008

DCAF15	-0.248692715	-0.040374327	-0.118181257	0.208318388	0.130511458	-0.07780693	8	0.122680434	0.816496581	0.816496581	0.816496581	0.816496581	0.816496581	0.816496581	25	148	0	P_232432	P_232432	P_232432	chr19	13959770	13959830	+	DCAF15	DDB1 and CUL4 associated factor 15 [Source:HGNC Symbol;Acc:HGNC:25095]	ENSG00000132017	protein_coding	exon	coding exon	1	4228	19:13959770:13959830	ENSG00000132017	DCAF15	8.57175897	0.014293047	-2.547688212	-0.413607601	-1.21068683	2.134080611	1.337001382	-0.797079229	9.961963646	0.029058006	0.687924662	0.253955688	0.058719494	0.210950152	0.443987347	-4.277596569	-6.092701111	-6.300733462	-5.101167632	-5.42472929	-6.734986381	2.51745952	0.117660168

CCDC186	0.179114347	-0.048387897	0.101375858	-0.227502244	-0.077738489	0.149763755	8	0.100247238	0.816496581	0.816496581	0.816496581	0.816496581	0.816496581	0.816496581	25	149	0	P_209996	P_209996	P_209996	chr10	114161632	114161692	-	CCDC186	coiled-coil domain containing 186 [Source:HGNC Symbol;Acc:HGNC:24349]	ENSG00000165813	protein_coding	3'UTR	3'UTR	1	4229	10:114161632:114161692	ENSG00000165813	CCDC186	9.196874333	0.010276987	2.163929412	-0.584587417	1.22474947	-2.74851683	-0.939179943	1.809336887	9.961963646	0.055839642	0.571826021	0.248840914	0.020598161	0.369851478	0.100625579	-4.907594202	-6.005077986	-6.284471239	-4.081432344	-5.847801508	-5.526719653	3.039074652	0.079667939

MGST2	0.13909338	-0.023446243	0.053082702	-0.162539623	-0.086010677	0.076528945	8	0.061944263	0.816496581	0.816496581	0.816496581	0.816496581	0.816496581	0.816496581	25	150	0	P_312460	P_312460	P_312460	chr4	139741207	139741267	+	MGST2;MAML3	microsomal glutathione S-transferase 2 [Source:HGNC Symbol;Acc:HGNC:7063];mastermind-like 3 (Drosophila) [Source:HGNC Symbol;Acc:HGNC:16272]	ENSG00000085871	protein_coding	3'ext;intron	multiple non-Ref	0	4230	4:139741207:139741267	ENSG00000085871	MGST2	8.148373148	0.005288078	2.342627121	-0.394884396	0.894025137	-2.737511517	-1.448601984	1.288909533	9.961963646	0.041246221	0.701250783	0.392395389	0.020990266	0.178182771	0.226554923	-4.617795379	-6.100546959	-6.627375448	-4.100157104	-5.287249736	-6.232482836	2.949470209	0.085022468

ITGB5	0.071836931	0.086320239	0.067618851	0.014483308	-0.00421808	-0.018701388	8	0.154242726	0.816496581	0.816496581	0.816496581	0.816496581	0.816496581	0.816496581	25	151	0	P_135825	P_135825	P_135825	chr3	124819759	124819819	+	ITGB5	"integrin, beta 5 [Source:HGNC Symbol;Acc:HGNC:6160]"	ENSG00000082781	protein_coding	3'ext;exon;intron	multiple non-Ref	0	4231	3:124819759:124819819	ENSG00000082781	ITGB5	7.463890825	0.021312008	0.602672268	0.724179247	0.567284905	0.121506978	-0.035387363	-0.156894342	9.961963646	0.560194873	0.485614823	0.583077186	0.905705487	0.972469899	0.878462308	-6.80371393	-5.912945028	-6.874175247	-7.141545169	-6.296019237	-7.057947513	0.208348942	0.88830946

ZMYND15	0.279807505	0.0364782	0.330459929	-0.243329305	0.050652424	0.293981729	8	0.120361694	0.816496581	0.816496581	0.816496581	0.816496581	0.816496581	0.816496581	25	153	0	P_227506	P_227506	P_227506	chr17	4745903	4745963	+	ZMYND15	"zinc finger, MYND-type containing 15 [Source:HGNC Symbol;Acc:HGNC:20997]"	ENSG00000141497	protein_coding	exon	coding exon	1	4233	17:4745903:4745963	ENSG00000141497	ZMYND15	9.219444434	0.013840485	2.912925243	0.37975489	3.440240348	-2.533170353	0.527315105	3.060485458	9.961963646	0.015545645	0.712097592	0.006363925	0.029789249	0.609516741	0.012085714	-3.659387164	-6.106629836	-2.80355174	-4.44564648	-6.150882037	-3.474829799	6.087697567	0.012679109

GNS	0.09912346	0.050397043	0.049801097	-0.048726418	-0.049322363	-0.000595945	8	0.044209241	0.816496581	0.816496581	0.816496581	0.816496581	0.816496581	0.816496581	25	154	0	P_215994	P_215994	P_215994	chr12	64739380	64739440	-	GNS	glucosamine (N-acetyl)-6-sulfatase [Source:HGNC Symbol;Acc:HGNC:4422]	ENSG00000135677	protein_coding	exon	coding exon	1	4234	12:64739380:64739440	ENSG00000135677	GNS	7.9731943	0.003776219	1.975574763	1.004435529	0.992558075	-0.971139234	-0.983016689	-0.011877455	9.961963646	0.076542595	0.338942139	0.344421588	0.354467334	0.348870459	0.990757883	-5.203968944	-5.676523179	-6.534263042	-6.656970262	-5.8069141	-7.071301456	1.301088701	0.327605081

MKRN2	-0.110128188	-0.054428404	0.083255545	0.055699785	0.193383733	0.137683948	8	0.078811664	0.816496581	0.816496581	0.816496581	0.816496581	0.816496581	0.816496581	25	155	0	P_134085	P_134085	P_134085	chr3	12582566	12582626	+	MKRN2	makorin ring finger protein 2 [Source:HGNC Symbol;Acc:HGNC:7113]	ENSG00000075975	protein_coding	3'UTR	3'UTR	1	4235	3:12582566:12582626	ENSG00000075975	MKRN2	7.336848274	0.007194679	-1.590151141	-0.785896776	1.202134544	0.804254365	2.792285685	1.98803132	9.961963646	0.143001279	0.450216459	0.257107412	0.440020582	0.019110198	0.07497466	-5.769938347	-5.866588062	-6.310555346	-6.806962154	-3.26897384	-5.255316842	2.831215803	0.092755374

SIM1	-0.110852793	0.050744895	0.086770847	0.161597688	0.197623639	0.036025951	8	0.20086074	0.816496581	0.816496581	0.816496581	0.816496581	0.816496581	0.816496581	25	157	0	P_258433	P_258433	P_258433	chr6	100463293	100463353	-	SIM1	single-minded family bHLH transcription factor 1 [Source:HGNC Symbol;Acc:HGNC:10882]	ENSG00000112246	protein_coding	exon	coding exon	1	4237	6:100463293:100463353	ENSG00000112246	SIM1	4.305349719	0.034605948	-0.729821896	0.334089336	0.57127351	1.063911232	1.301095407	0.237184174	9.961963646	0.482307407	0.745238681	0.580472975	0.312477294	0.222513211	0.817320314	-6.715268175	-6.123587671	-6.871773936	-6.56351924	-5.467371601	-7.040732557	0.641936968	0.605364499

SPIDR	-0.151542865	-0.07693587	-0.069440425	0.074606995	0.08210244	0.007495445	8	0.097258976	0.816496581	0.816496581	0.816496581	0.816496581	0.816496581	0.816496581	25	158	0	P_314088	P_314088	P_314088	chr8	47695986	47696046	+	SPIDR	scaffolding protein involved in DNA repair [Source:HGNC Symbol;Acc:HGNC:28971]	ENSG00000164808	protein_coding	intron;promoter	multiple non-Ref	0	4238	8:47695986:47696046	ENSG00000164808	SPIDR	5.524371664	0.009803024	-1.874567495	-0.951687699	-0.858969928	0.922879796	1.015597567	0.092717771	9.961963646	0.09044021	0.363774273	0.410549522	0.377880264	0.333851916	0.927965885	-5.35815681	-5.725950295	-6.658513437	-6.702800743	-5.775540323	-7.06668422	1.174880705	0.367704268

LAMTOR4	0.103834612	0.144711186	0.203523003	0.040876574	0.099688391	0.058811818	8	0.109461366	0.816496581	0.816496581	0.816496581	0.816496581	0.816496581	0.816496581	25	159	0	P_145399	P_145399	P_145399	chr7	100154138	100154198	+	LAMTOR4	"late endosomal/lysosomal adaptor, MAPK and MTOR activator 4 [Source:HGNC Symbol;Acc:HGNC:33772]"	ENSG00000188186	protein_coding	3'UTR;exon	multiple Ref	1	4239	7:100154138:100154198	ENSG00000188186	LAMTOR4	14.07216151	0.011828715	1.169281253	1.629592228	2.291871929	0.460310975	1.122590677	0.662279701	9.961963646	0.269506874	0.134359137	0.044964856	0.655176683	0.287938298	0.52281732	-6.299611111	-4.947649545	-4.747405435	-7.034962022	-5.666823826	-6.836923287	1.864362807	0.199724282

GSX2	-0.029462917	0.197042518	0.281732382	0.226505435	0.311195299	0.084689864	8	0.391223098	0.816496581	0.816496581	0.816496581	0.816496581	0.816496581	0.816496581	25	160	0	A_23_P92453	A_23_P92453	A_23_P92453	chr4	54101661	54101721	+	GSX2	GS homeobox 2 [Source:HGNC Symbol;Acc:HGNC:24959]	ENSG00000180613	protein_coding	3'UTR;exon	multiple Ref	1	4240	4:54101661:54101721	ENSG00000180613	GSX2	3.390509491	0.125118606	-0.102014151	0.682251704	0.975486913	0.784265854	1.077501064	0.293235209	9.961963646	0.920769618	0.510631171	0.352411059	0.451129719	0.30665635	0.775359966	-6.992684349	-5.942498644	-6.550973069	-6.823285841	-5.713681294	-7.024606587	0.548252892	0.66059512

C5orf64-AS1	-0.05970901	0.597796807	0.432710853	0.657505816	0.492419863	-0.165085953	8	0.359059795	0.816496581	0.816496581	0.816496581	0.816496581	0.816496581	0.816496581	26	10	0	A_32_P336294	A_32_P336294	A_32_P336294	chr5	61733426	61733486	-	CTD-2170G1.2	NA	ENSG00000251575	lincRNA	exon	non-coding exon	1	4260	5:61733426:61733486	ENSG00000251575	C5orf64-AS1	3.562721988	0.105739635	-0.224888161	2.251543374	1.62976323	2.476431535	1.854651391	-0.621780144	9.961963646	0.826612129	0.04815009	0.134322704	0.032825904	0.093444049	0.548051195	-6.970832495	-4.042857408	-5.762477887	-4.540656835	-4.733590485	-6.864195347	2.955867652	0.084626334

CPXM2	-0.179173423	-0.078940243	-0.084492668	0.100233181	0.094680756	-0.005552425	8	0.150874138	0.816496581	0.816496581	0.816496581	0.816496581	0.816496581	0.816496581	26	12	0	P_210330	P_210330	P_210330	chr10	123770937	123770997	-	CPXM2	"carboxypeptidase X (M14 family), member 2 [Source:HGNC Symbol;Acc:HGNC:26977]"	ENSG00000121898	protein_coding	5'UTR;exon	multiple Ref	1	4262	10:123770937:123770997	ENSG00000121898	CPXM2	3.797410917	0.020486619	-1.533147998	-0.675474479	-0.722985371	0.857673519	0.810162627	-0.047510892	9.961963646	0.156360708	0.514747055	0.486316425	0.411231817	0.436771909	0.963045037	-5.847826031	-5.947126029	-6.768955083	-6.761580869	-5.958959838	-7.070145503	0.787290173	0.52806122

AMD1	-0.308480329	-0.098339493	-0.355516045	0.210140836	-0.047035716	-0.257176552	8	0.066890583	0.816496581	0.816496581	0.816496581	0.816496581	0.816496581	0.816496581	26	13	0	P_258712	P_258712	P_258712	chr6	110894215	110894275	-	AMD1	adenosylmethionine decarboxylase 1 [Source:HGNC Symbol;Acc:HGNC:457]	ENSG00000123505	protein_coding	3'ext;3'UTR;exon	multiple non-Ref	0	4263	6:110894215:110894275	ENSG00000123505	AMD1	7.062073598	0.005799831	-4.960956637	-1.58148807	-5.717381366	3.379468567	-0.756424729	-4.135893296	9.961963646	0.000575744	0.144964297	0.000196446	0.007046205	0.466906104	0.002041545	-0.287986485	-5.01164529	0.791873335	-3.001013152	-6.001050002	-1.658891966	14.81654075	0.000528558

UGGT2	0.071142075	0.002461776	0.044503563	-0.068680299	-0.026638512	0.042041787	8	0.058711375	0.816496581	0.816496581	0.816496581	0.816496581	0.816496581	0.816496581	26	15	0	P_219629	P_219629	P_219629	chr13	95902864	95902924	-	UGGT2	UDP-glucose glycoprotein glucosyltransferase 2 [Source:HGNC Symbol;Acc:HGNC:15664]	ENSG00000102595	protein_coding	exon	coding exon	1	4265	13:95902864:95902924	ENSG00000102595	UGGT2	7.349040047	0.004974833	1.235329707	0.042746925	0.772771578	-1.192582782	-0.462558128	0.730024654	9.961963646	0.245047856	0.966747929	0.457600437	0.260664466	0.653619458	0.482188825	-6.224021894	-6.181124216	-6.730467402	-6.422881556	-6.184127733	-6.787807501	0.715698734	0.564865425

MAIP1	-0.063521558	0.139727976	0.106951792	0.203249534	0.17047335	-0.032776183	8	0.098253517	0.816496581	0.816496581	0.816496581	0.816496581	0.816496581	0.816496581	26	16	0	A_23_P131526	A_23_P131526	A_23_P131526	chr2	199961909	199963773	+	C2orf47	chromosome 2 open reading frame 47 [Source:HGNC Symbol;Acc:HGNC:26198]	ENSG00000162972	protein_coding	"exon,intron;intron"	multiple non-Ref	0	4266	2:199961909:199963773	ENSG00000162972	MAIP1	10.23872354	0.009959174	-0.779569966	1.714815208	1.312568646	2.494385173	2.092138611	-0.402246562	9.961963646	0.453766018	0.117262238	0.218762547	0.031833345	0.063007316	0.695997799	-6.676528797	-4.831700831	-6.17984952	-4.510644566	-4.379998708	-6.983700547	2.671993439	0.104521245

PTPN23	-0.118407509	-0.018200658	-0.047966906	0.10020685	0.070440603	-0.029766248	8	0.105581706	0.816496581	0.816496581	0.816496581	0.816496581	0.816496581	0.816496581	26	17	0	A_24_P328675	A_24_P328675	A_24_P328675	chr3	47413243	47413303	+	PTPN23	"protein tyrosine phosphatase, non-receptor type 23 [Source:HGNC Symbol;Acc:HGNC:14406]"	ENSG00000076201	protein_coding	3'UTR	3'UTR	1	4267	3:47413243:47413303	ENSG00000076201	PTPN23	5.973800555	0.011158732	-1.372832201	-0.211020821	-0.556134603	1.161811381	0.816697598	-0.345113782	9.961963646	0.199916125	0.83712509	0.590390638	0.272391393	0.433197291	0.737184617	-6.057145891	-6.158667081	-6.880804489	-6.45763279	-5.953668983	-7.006700473	0.729064652	0.557808463

WDR38	0.096043693	-0.192078604	-0.020666122	-0.288122298	-0.116709816	0.171412482	8	0.196336643	0.816496581	0.816496581	0.816496581	0.816496581	0.816496581	0.816496581	26	18	0	A_32_P449380	A_32_P449380	A_32_P449380	chr9	124856790	124856850	+	WDR38	WD repeat domain 38 [Source:HGNC Symbol;Acc:HGNC:23745]	ENSG00000136918	protein_coding	exon	coding exon	1	4268	9:124856790:124856850	ENSG00000136918	WDR38	9.536505095	0.033162892	0.645934206	-1.291809349	-0.13898836	-1.937743555	-0.784922565	1.152820989	9.961963646	0.532918116	0.225587685	0.892228927	0.081495499	0.450761837	0.275895378	-6.775357333	-5.371489469	-7.036581265	-5.408999445	-5.979045451	-6.39016904	1.300878318	0.327667729

TTC39B	0.229102453	0.111096017	0.341821335	-0.118006436	0.112718882	0.230725318	8	0.095995969	0.816496581	0.816496581	0.816496581	0.816496581	0.816496581	0.816496581	26	19	0	A_32_P58201	A_32_P58201	A_32_P58201	chr9	15164303	15164363	-	TTC39B	tetratricopeptide repeat domain 39B [Source:HGNC Symbol;Acc:HGNC:23704]	ENSG00000155158	protein_coding	3'UTR	3'UTR	1	4269	9:15164303:15164363	ENSG00000155158	TTC39B	6.490903426	0.009607013	2.862735208	1.388193251	4.271206872	-1.474541957	1.408471664	2.883013621	9.961963646	0.016938966	0.195336862	0.001648057	0.171223089	0.189428043	0.016361418	-3.744904862	-5.256994919	-1.416835745	-6.074772089	-5.337520832	-3.778336178	6.805895911	0.008919135

CDC42-IT1	2.746430198	-0.55787743	-0.61586094	-3.304307627	-3.362291137	-0.05798351	8	3.082880275	0.816496581	0.816496581	0.816496581	0.816496581	0.816496581	0.816496581	26	20	0	A_24_P453532	A_24_P453532	A_24_P453532	chr1	22064097	22064157	+	CDC42-IT1	CDC42 intronic transcript 1 (non-protein coding) [Source:HGNC Symbol;Acc:HGNC:41317]	ENSG00000230068	sense_intronic	exon	non-coding exon	1	4270	1:22064097:22064157	ENSG00000230068	CDC42-IT1	5.281263634	7.634557999	1.217369021	-0.247281981	-0.272983464	-1.464651002	-1.490352485	-0.025701483	9.961963646	0.251514823	0.809711707	0.790441291	0.173848653	0.167097402	0.980002997	-6.244881857	-6.149954384	-7.006577065	-6.087820593	-5.233997799	-7.071017667	0.997444889	0.43350806

DDI2	-0.172813432	-0.115316077	-0.215617528	0.057497356	-0.042804095	-0.100301451	8	0.093174197	0.816496581	0.816496581	0.816496581	0.816496581	0.816496581	0.816496581	26	21	0	P_200430	P_200430	P_200430	chr1	15643527	15643587	+	DDI2	DNA-damage inducible 1 homolog 2 (S. cerevisiae) [Source:HGNC Symbol;Acc:HGNC:24578]	ENSG00000197312	protein_coding	"3'UTR;3'UTR,exon;exon"	multiple Ref	1	4271	1:15643527:15643587	ENSG00000197312	DDI2	7.597640651	0.009178346	-2.209229844	-1.474189331	-2.756433168	0.735040513	-0.547203323	-1.282243837	9.961963646	0.051726747	0.171316101	0.020320666	0.479261171	0.596283694	0.228791236	-4.834834303	-5.150292014	-3.971181905	-6.861930032	-6.139846299	-6.240503982	2.855953298	0.09107115

ASCL4	-0.505574222	0.203236885	-0.069232327	0.708811107	0.436341895	-0.272469212	8	0.27062476	0.816496581	0.816496581	0.816496581	0.816496581	0.816496581	0.816496581	26	22	0	P_112657	P_112657	P_112657	chr12	107776567	107776627	+	ASCL4	achaete-scute family bHLH transcription factor 4 [Source:HGNC Symbol;Acc:HGNC:24311]	ENSG00000187855	protein_coding	exon	coding exon	1	4272	12:107776567:107776627	ENSG00000187855	ASCL4	3.349894071	0.061020599	-2.506642031	1.0076505	-0.343254568	3.514292531	2.163387462	-1.350905069	9.961963646	0.031172816	0.337470142	0.738540585	0.005623895	0.055890701	0.206609389	-4.346220314	-5.67344063	-6.983156216	-2.770871705	-4.270777711	-6.156483654	4.378646551	0.032770438

CLEC4A	-0.058643546	0.063205336	0.049153199	0.121848882	0.107796744	-0.014052137	8	0.162115515	0.816496581	0.816496581	0.816496581	0.816496581	0.816496581	0.816496581	26	26	0	P_214272	P_214272	P_214272	chr12	8123900	8123960	+	CLEC4A	"C-type lectin domain family 4, member A [Source:HGNC Symbol;Acc:HGNC:13257]"	ENSG00000111729	protein_coding	exon	coding exon	1	4276	12:8123900:8123960	ENSG00000111729	CLEC4A	4.470531429	0.023312114	-0.470408355	0.507000688	0.394281671	0.977409042	0.864690026	-0.112719016	9.961963646	0.64819315	0.62318757	0.701681571	0.351504758	0.40754835	0.91249238	-6.878939754	-6.048368742	-6.962883847	-6.650873911	-5.913688697	-7.064441878	0.391585768	0.761771121

KMT2C	0.091229847	-0.312207189	-0.135488437	-0.403437036	-0.226718284	0.176718752	8	0.049554601	0.816496581	0.816496581	0.816496581	0.816496581	0.816496581	0.816496581	26	27	0	A_23_P168419	A_23_P168419	A_23_P168419	chr7	152136467	152136527	-	KMT2C	lysine (K)-specific methyltransferase 2C [Source:HGNC Symbol;Acc:HGNC:13726]	ENSG00000055609	protein_coding	3'UTR	3'UTR	1	4277	7:152136467:152136527	ENSG00000055609	KMT2C	6.817920415	0.004178712	1.728468109	-5.9151713	-2.567004674	-7.643639409	-4.295472783	3.348166626	9.961963646	0.114713747	0.000150275	0.028112591	1.79E-05	0.001586393	0.007426672	-5.574188147	1.325202718	-4.291243969	3.218147486	-0.879945726	-2.982596132	22.10881602	0.000100693

USP7	-0.197376944	-0.030681457	-0.13986586	0.166695487	0.057511084	-0.109184403	8	0.022737243	0.816496581	0.816496581	0.816496581	0.816496581	0.816496581	0.816496581	26	28	0	P_225478	P_225478	P_225478	chr16	8917048	8917108	-	USP7	ubiquitin specific peptidase 7 (herpes virus-associated) [Source:HGNC Symbol;Acc:HGNC:12630]	ENSG00000187555	protein_coding	3'UTR;exon	multiple Ref	1	4278	16:8917048:8917108	ENSG00000187555	USP7	10.27295902	0.002621849	-4.72104588	-0.733867719	-3.345442116	3.987178161	1.375603764	-2.611574397	9.961963646	0.000823688	0.4799447	0.007460774	0.002590436	0.199083258	0.026046169	-0.658932019	-5.905891007	-2.965136407	-1.973868424	-5.378003613	-4.239224078	9.771502536	0.00258544

TAFA5	0.134417589	0.100953056	0.202748922	-0.033464533	0.068331333	0.101795866	8	0.16703699	0.816496581	0.816496581	0.816496581	0.816496581	0.816496581	0.816496581	26	30	0	P_133735	P_133735	P_133735	chr22	48576511	48576571	+	FAM19A5	"family with sequence similarity 19 (chemokine (C-C motif)-like), member A5 [Source:HGNC Symbol;Acc:HGNC:21592]"	ENSG00000219438	protein_coding	exon	coding exon	1	4280	22:48576511:48576571	ENSG00000219438	TAFA5	5.388383374	0.024612994	1.049347918	0.788102806	1.582785116	-0.261245113	0.533437198	0.79468231	9.961963646	0.31880819	0.448983086	0.144668881	0.799224961	0.605427193	0.445317727	-6.428682506	-5.864869186	-5.827814029	-7.112418002	-6.147525964	-6.736945218	0.868628339	0.489152745

HINT1	-0.265282709	0.128841828	-0.129156239	0.394124537	0.13612647	-0.257998067	8	0.121316326	0.816496581	0.816496581	0.816496581	0.816496581	0.816496581	0.816496581	26	32	0	P_140241	P_140241	P_140241	chr5	131161137	131161197	-	HINT1	histidine triad nucleotide binding protein 1 [Source:HGNC Symbol;Acc:HGNC:4912]	ENSG00000169567	protein_coding	3'ext;intron	multiple non-Ref	0	4282	5:131161137:131161197	ENSG00000169567	HINT1	10.27949763	0.01402576	-2.743414109	1.332414347	-1.335665827	4.075828457	1.407748283	-2.668080174	9.961963646	0.020779042	0.212398857	0.211371101	0.002246859	0.189636181	0.023642433	-3.947653795	-5.323938559	-6.151482182	-1.826776798	-5.338418581	-4.143801092	6.133072804	0.012391328

PPP1R12B	0.031876456	-0.013382233	-0.139777376	-0.045258689	-0.171653832	-0.126395143	8	0.08158787	0.816496581	0.816496581	0.816496581	0.816496581	0.816496581	0.816496581	26	33	0	P_104457	P_104457	P_104457	chr1	202581089	202581149	+	PPP1R12B	"protein phosphatase 1, regulatory subunit 12B [Source:HGNC Symbol;Acc:HGNC:7619]"	ENSG00000077157	protein_coding	3'UTR	3'UTR	1	4283	1:202581089:202581149	ENSG00000077157	PPP1R12B	5.944849426	0.007552281	0.449238107	-0.188597153	-1.969896642	-0.63783526	-2.419134749	-1.781299489	9.961963646	0.662875166	0.854195189	0.077267504	0.537964956	0.036201966	0.105319877	-6.889341191	-6.163371307	-5.259874971	-6.931578939	-3.869832123	-5.568177573	2.258325194	0.144327302

CFDP1	0.125313232	0.196725615	0.228290513	0.071412383	0.102977282	0.031564899	8	0.061924896	0.816496581	0.816496581	0.816496581	0.816496581	0.816496581	0.816496581	26	41	0	A_23_P89123	A_23_P89123	A_23_P89123	chr16	75395121	75395181	-	CFDP1	craniofacial development protein 1 [Source:HGNC Symbol;Acc:HGNC:1873]	ENSG00000153774	protein_coding	exon	coding exon	1	4291	16:75395121:75395181	ENSG00000153774	CFDP1	11.07279367	0.005286151	2.110924812	3.313879755	3.845596373	1.202954943	1.734671561	0.531716618	9.961963646	0.061051027	0.007867809	0.003258121	0.256803721	0.113572494	0.606575101	-4.992045724	-2.356488759	-2.119036496	-6.411014469	-4.904808026	-6.919092493	5.827553774	0.014492304

ZNF429	0.252823055	-0.067308061	0.060077855	-0.320131116	-0.1927452	0.127385916	8	0.078031319	0.816496581	0.816496581	0.816496581	0.816496581	0.816496581	0.816496581	26	42	0	A_32_P10495	A_32_P10495	A_32_P10495	chr19	21540205	21540265	+	ZNF429	zinc finger protein 429 [Source:HGNC Symbol;Acc:HGNC:20817]	ENSG00000197013	protein_coding	3'ext;intron;promoter	multiple non-Ref	0	4292	19:21540205:21540265	ENSG00000197013	ZNF429	8.909006671	0.007096392	3.67572917	-0.978574532	0.873456438	-4.654303702	-2.802272732	1.852030971	9.961963646	0.004303773	0.35095605	0.402978278	0.000911314	0.018786114	0.093846083	-2.361198026	-5.701028144	-6.645774973	-0.88975752	-3.252753223	-5.462972469	8.029468888	0.00515521

LAMA4	-0.065914884	-0.018276448	0.013724884	0.047638436	0.079639768	0.032001332	8	0.07347489	0.816496581	0.816496581	0.816496581	0.816496581	0.816496581	0.816496581	26	44	0	P_258792	P_258792	P_258792	chr6	112134466	112134526	-	LAMA4	"laminin, alpha 4 [Source:HGNC Symbol;Acc:HGNC:6484]"	ENSG00000112769	protein_coding	exon	coding exon	1	4294	6:112134466:112134526	ENSG00000112769	LAMA4	4.159506417	0.006542022	-0.998097884	-0.276746052	0.207825256	0.721351831	1.205923139	0.484571308	9.961963646	0.341857809	0.787632228	0.839552539	0.487277428	0.255707371	0.638457928	-6.480445197	-6.141873073	-7.023584735	-6.872278044	-5.576398813	-6.944600315	0.554231256	0.656945683

ADO	-0.042960126	-0.060748348	-0.052875243	-0.017788222	-0.009915117	0.007873105	8	0.098260819	0.816496581	0.816496581	0.816496581	0.816496581	0.816496581	0.816496581	26	45	0	A_23_P202345	A_23_P202345	A_23_P202345	chr10	62808043	62808103	+	ADO	2-aminoethanethiol (cysteamine) dioxygenase [Source:HGNC Symbol;Acc:HGNC:23506]	ENSG00000181915	protein_coding	exon	coding exon	1	4295	10:62808043:62808103	ENSG00000181915	ADO	10.3044757	0.009960327	-0.527198763	-0.745492552	-0.64887525	-0.218293789	-0.121676487	0.096617302	9.961963646	0.609594594	0.473196688	0.531092285	0.831607043	0.905574652	0.924946414	-6.848785633	-5.897316791	-6.821948221	-7.123616185	-6.288816627	-7.06628124	0.22108817	0.879560724

EFL1	0.02635817	0.028512178	0.155483065	0.002154007	0.129124895	0.126970887	8	0.10558403	0.816496581	0.816496581	0.816496581	0.816496581	0.816496581	0.816496581	26	46	0	A_23_P152055	A_23_P152055	A_23_P152055	chr15	82130316	82130376	-	EFTUD1	elongation factor Tu GTP binding domain containing 1 [Source:HGNC Symbol;Acc:HGNC:25789]	ENSG00000140598	protein_coding	"3'UTR,exon"	multiple Ref	1	4296	15:82130316:82130376	ENSG00000140598	EFL1	9.20318043	0.011159126	0.305594692	0.330568096	1.802659236	0.024973404	1.497064544	1.47209114	9.961963646	0.766203423	0.747818031	0.101725848	0.980569347	0.165372164	0.171870447	-6.947638474	-6.124807378	-5.51299192	-7.149272009	-5.225348595	-6.00099445	1.310186992	0.324908551

S100G	-0.00663158	0.105529453	-0.05255841	0.112161033	-0.045926831	-0.158087863	8	0.235031913	0.816496581	0.816496581	0.816496581	0.816496581	0.816496581	0.816496581	26	48	0	P_150537	P_150537	P_150537	chrX	16654430	16654490	+	S100G	S100 calcium binding protein G [Source:HGNC Symbol;Acc:HGNC:1436]	ENSG00000169906	protein_coding	exon	coding exon	1	4298	X:16654430:16654490	ENSG00000169906	S100G	3.256853986	0.046567416	-0.037637569	0.598933003	-0.298295555	0.636570572	-0.260657986	-0.897228558	9.961963646	0.970720201	0.562588855	0.771606538	0.538755542	0.799665085	0.390764853	-6.997587353	-5.996444192	-6.998744917	-6.932425612	-6.26067426	-6.648579487	0.287883555	0.83317243

PHBP19	-0.25307439	-0.269952989	-0.266961965	-0.016878599	-0.013887575	0.002991024	8	0.168273376	0.816496581	0.816496581	0.816496581	0.816496581	0.816496581	0.816496581	26	50	0	A_32_P218636	A_32_P218636	A_32_P218636	chr12	51124991	51125051	-	RP11-416A17.6	NA	ENSG00000257246	processed_pseudogene	exon	non-coding exon	1	4300	12:51124991:51125051	ENSG00000257246	PHBP19	10.22719552	0.024945919	-1.962429552	-2.093312257	-2.070118789	-0.130882705	-0.107689236	0.023193469	9.961963646	0.07823063	0.062883368	0.065376414	0.898473454	0.916380213	0.981953948	-5.224238919	-4.283937188	-5.103500672	-7.140253179	-6.29052069	-7.071084656	2.091290491	0.16528623

SLC1A2	-0.042510714	-0.031271226	-0.091199967	0.011239487	-0.048689254	-0.059928741	8	0.13605475	0.816496581	0.816496581	0.816496581	0.816496581	0.816496581	0.816496581	26	52	0	P_211547	P_211547	P_211547	chr11	35286756	35286816	-	SLC1A2	"solute carrier family 1 (glial high affinity glutamate transporter), member 2 [Source:HGNC Symbol;Acc:HGNC:10940]"	ENSG00000110436	protein_coding	exon	coding exon	1	4302	11:35286756:35286816	ENSG00000110436	SLC1A2	4.299623233	0.017071942	-0.39847636	-0.29312245	-0.854867587	0.10535391	-0.456391226	-0.561745137	9.961963646	0.698685792	0.775443673	0.41271123	0.918186126	0.657897131	0.586704597	-6.912402312	-6.136994768	-6.662087455	-7.143546547	-6.187077337	-6.901681217	0.251742128	0.858335202

FAM9A	-0.267857972	-0.142805273	-0.05974701	0.125052699	0.208110961	0.083058263	8	0.247861605	0.816496581	0.816496581	0.816496581	0.816496581	0.816496581	0.816496581	26	55	0	P_269438	P_269438	P_269438	chrX	8793696	8793756	-	FAM9A	"family with sequence similarity 9, member A [Source:HGNC Symbol;Acc:HGNC:18403]"	ENSG00000183304	protein_coding	exon	coding exon	1	4305	X:8793696:8793756	ENSG00000183304	FAM9A	3.699266711	0.05154264	-1.444996769	-0.770382739	-0.322313487	0.67461403	1.122683282	0.448069252	9.961963646	0.179169063	0.458952769	0.753877395	0.515271039	0.28790079	0.66369025	-5.964776485	-5.878556382	-6.990683356	-6.906282744	-5.666726066	-6.962797604	0.783613184	0.529892346

SYNPO2L	-0.138675966	0.000822339	0.203107075	0.139498305	0.34178304	0.202284735	8	0.236410441	0.816496581	0.816496581	0.816496581	0.816496581	0.816496581	0.816496581	26	56	0	P_106296	P_106296	P_106296	chr10	73646323	73646383	-	SYNPO2L	synaptopodin 2-like [Source:HGNC Symbol;Acc:HGNC:23532]	ENSG00000166317	protein_coding	3'UTR	3'UTR	1	4306	10:73646323:73646383	ENSG00000166317	SYNPO2L	5.354578344	0.047089318	-0.782682507	0.004641255	1.146329526	0.787323762	1.929012033	1.14168827	9.961963646	0.452017498	0.996388455	0.278447433	0.449418389	0.082680539	0.280283454	-6.674029721	-6.182076068	-6.373356635	-6.820812041	-4.624802956	-6.402483789	1.261850511	0.339524829

MIS18BP1	-0.334472026	-0.055035591	-0.204941542	0.279436435	0.129530483	-0.149905951	8	0.054418224	0.816496581	0.816496581	0.816496581	0.816496581	0.816496581	0.816496581	26	57	0	A_23_P364107	A_23_P364107	A_23_P364107	chr14	45204009	45204069	-	MIS18BP1	MIS18 binding protein 1 [Source:HGNC Symbol;Acc:HGNC:20190]	ENSG00000129534	protein_coding	3'UTR;exon	multiple Ref	1	4307	14:45204009:45204069	ENSG00000129534	MIS18BP1	7.338722516	0.004584804	-6.049856107	-0.995471611	-3.706937339	5.054384495	2.342918768	-2.711465727	9.961963646	0.000125597	0.343071463	0.004088195	0.000501917	0.041225745	0.02194833	1.291226158	-5.685075802	-2.351764103	-0.267531251	-3.990569862	-4.070317081	14.95353741	0.000509458

TMX4	-0.342913983	0.00847653	-0.37003638	0.351390513	-0.027122398	-0.37851291	8	0.043838751	0.816496581	0.816496581	0.816496581	0.816496581	0.816496581	0.816496581	26	58	0	P_240126	P_240126	P_240126	chr20	7987287	7987347	-	TMX4	thioredoxin-related transmembrane protein 4 [Source:HGNC Symbol;Acc:HGNC:25237]	ENSG00000125827	protein_coding	exon	coding exon	1	4308	20:7987287:7987347	ENSG00000125827	TMX4	9.438038992	0.003750023	-6.858258459	0.169530073	-7.400704737	7.027788532	-0.542446277	-7.57023481	9.961963646	4.50E-05	0.868772836	2.36E-05	3.66E-05	0.599435109	1.95E-05	2.354150402	-6.166959002	2.991404713	2.470559524	-6.142520913	3.178628981	34.80456581	1.37E-05

INPP5F	-0.006817721	0.128672122	0.000235531	0.135489844	0.007053253	-0.128436591	8	0.058882055	0.816496581	0.816496581	0.816496581	0.816496581	0.816496581	0.816496581	26	59	0	P_210177	P_210177	P_210177	chr10	119804237	119804297	+	INPP5F	inositol polyphosphate-5-phosphatase F [Source:HGNC Symbol;Acc:HGNC:17054]	ENSG00000198825	protein_coding	exon	coding exon	1	4309	10:119804237:119804297	ENSG00000198825	INPP5F	6.101851101	0.004990951	-0.118193396	2.230685883	0.004083218	2.348879279	0.122276614	-2.226602665	9.961963646	0.908263643	0.04988217	0.996822683	0.040809434	0.905111472	0.050228238	-6.990742556	-4.07494533	-7.047111497	-4.752329974	-6.288738877	-4.876795228	2.579981788	0.112122486

AP1S1	0.349669472	0.383535721	0.077462992	0.033866248	-0.27220648	-0.306072728	8	0.287801044	0.816496581	0.816496581	0.816496581	0.816496581	0.816496581	0.816496581	26	60	0	P_145501	P_145501	P_145501	chr7	101157419	101157479	+	AP1S1	"adaptor-related protein complex 1, sigma 1 subunit [Source:HGNC Symbol;Acc:HGNC:559]"	ENSG00000106367	protein_coding	exon	coding exon	1	4310	7:101157419:101157479	ENSG00000106367	AP1S1	9.136752494	0.068723241	1.633621837	1.791841662	0.361899582	0.158219824	-1.271722255	-1.429942079	9.961963646	0.133502942	0.103531725	0.724985673	0.877444867	0.232358099	0.183338428	-5.709415114	-4.7242834	-6.976065381	-7.135940349	-5.501651248	-6.056074535	1.616238919	0.247296654

RNF130	0.13277821	-0.057048934	-0.017659942	-0.189827144	-0.150438151	0.039388993	8	0.075094519	0.816496581	0.816496581	0.816496581	0.816496581	0.816496581	0.816496581	26	63	0	A_23_P41734	A_23_P41734	A_23_P41734	chr5	179955561	179955621	-	RNF130	ring finger protein 130 [Source:HGNC Symbol;Acc:HGNC:18280]	ENSG00000113269	protein_coding	3'UTR;exon	multiple Ref	1	4313	5:179955561:179955621	ENSG00000113269	RNF130	9.768740054	0.006735258	1.981505597	-0.851365469	-0.263546809	-2.832871066	-2.245052406	0.58781866	9.961963646	0.075792258	0.414562814	0.797500267	0.017827238	0.048682787	0.56973815	-5.194804473	-5.813796252	-7.009322065	-3.937614179	-4.144128832	-6.885836335	3.021932346	0.080660716

GID8	-0.048659725	0.044691776	-0.157102685	0.093351501	-0.10844296	-0.201794461	8	0.073836084	0.816496581	0.816496581	0.816496581	0.816496581	0.816496581	0.816496581	26	64	0	A_23_P56971	A_23_P56971	A_23_P56971	chr20	62945049	62945109	+	GID8	GID complex subunit 8 [Source:HGNC Symbol;Acc:HGNC:15857]	ENSG00000101193	protein_coding	3'UTR	3'UTR	1	4314	20:62945049:62945109	ENSG00000101193	GID8	8.601589588	0.00658475	-0.734421908	0.674533592	-2.371153004	1.4089555	-1.636731096	-3.045686595	9.961963646	0.479621631	0.515320039	0.039289778	0.189288938	0.132845634	0.012394142	-6.711781843	-5.947765136	-4.617321759	-6.160226512	-5.040153818	-3.500165491	3.426164798	0.060674655

TIAM1	0.207644472	-0.067934209	0.146207043	-0.27557868	-0.061437428	0.214141252	8	0.135466774	0.816496581	0.816496581	0.816496581	0.816496581	0.816496581	0.816496581	26	66	0	P_241549	P_241549	P_241549	chr21	31266009	31266069	-	TIAM1	T-cell lymphoma invasion and metastasis 1 [Source:HGNC Symbol;Acc:HGNC:11805]	ENSG00000156299	protein_coding	exon	coding exon	1	4316	21:31266009:31266069	ENSG00000156299	TIAM1	3.831618832	0.016943736	1.953715923	-0.639189399	1.375654388	-2.592905322	-0.578061535	2.014843787	9.961963646	0.079368789	0.537119199	0.199068073	0.026892688	0.57605552	0.071701834	-5.237642847	-5.971183731	-6.101563578	-4.34513876	-6.121965367	-5.213617739	2.872483771	0.08996583

MPZL1	-0.315035256	-0.041546761	-0.364444821	0.273488495	-0.049409565	-0.32289806	8	0.062739674	0.816496581	0.816496581	0.816496581	0.816496581	0.816496581	0.816496581	26	67	0	P_103941	P_103941	P_103941	chr1	167773625	167773685	+	MPZL1	myelin protein zero-like 1 [Source:HGNC Symbol;Acc:HGNC:7226]	ENSG00000197965	protein_coding	exon	non-coding exon	1	4317	1:167773625:167773685	ENSG00000197965	MPZL1	9.927726813	0.005367721	-5.266351374	-0.694524939	-6.092316493	4.571826435	-0.82596512	-5.397791555	9.961963646	0.000369348	0.503228354	0.000118751	0.001033489	0.428161624	0.000306367	0.172311039	-5.934012175	1.315645469	-1.020755578	-5.946102446	0.310658233	19.34218516	0.000177376

AOPEP	-0.146814804	-0.000282863	-0.133990905	0.146531941	0.012823899	-0.133708042	8	0.073974949	0.816496581	0.816496581	0.816496581	0.816496581	0.816496581	0.816496581	26	68	0	P_268013	P_268013	P_268013	chr9	94800936	94800996	+	C9orf3	chromosome 9 open reading frame 3 [Source:HGNC Symbol;Acc:HGNC:1361]	ENSG00000148120	protein_coding	exon	coding exon	1	4318	9:94800936:94800996	ENSG00000148120	AOPEP	5.527777393	0.006601234	-2.213109612	-0.00426392	-2.019800131	2.208845692	0.19330948	-2.015536211	9.961963646	0.051388397	0.996682072	0.071111814	0.051760366	0.850601027	0.071619129	-4.828578998	-6.18207784	-5.182412161	-4.980996607	-6.276847844	-5.212537776	2.992703162	0.082387485

TMEM245	-0.039535205	0.059203935	-0.004234717	0.09873914	0.035300488	-0.063438652	8	0.069125757	0.816496581	0.816496581	0.816496581	0.816496581	0.816496581	0.816496581	26	69	0	P_268415	P_268415	P_268415	chr9	109106507	109106567	-	TMEM245	transmembrane protein 245 [Source:HGNC Symbol;Acc:HGNC:1363]	ENSG00000106771	protein_coding	"5'UTR,exon;exon"	multiple Ref	1	4319	9:109106507:109106567	ENSG00000106771	TMEM245	6.911412198	0.006043976	-0.622828195	0.932684677	-0.066712718	1.555512872	0.556115477	-0.999397395	9.961963646	0.547389496	0.373035998	0.94813031	0.150993662	0.590403224	0.341258453	-6.790728432	-5.743218613	-7.044690638	-5.965888121	-6.134776484	-6.551566841	0.831657688	0.506462281

DOP1A	0.029396261	-0.058344379	-0.080530517	-0.087740641	-0.109926778	-0.022186138	8	0.075658799	0.816496581	0.816496581	0.816496581	0.816496581	0.816496581	0.816496581	26	71	0	P_258019	P_258019	P_258019	chr6	83110242	83110302	+	DOPEY1	dopey family member 1 [Source:HGNC Symbol;Acc:HGNC:21194]	ENSG00000083097	protein_coding	exon	coding exon	1	4321	6:83110242:83110302	ENSG00000083097	DOP1A	5.81400695	0.006803572	0.436484915	-0.866315652	-1.195742387	-1.302800567	-1.632227302	-0.329426735	9.961963646	0.671793173	0.406698196	0.259483504	0.22195244	0.133798694	0.748654794	-6.895385211	-5.801230911	-6.317862565	-6.292952146	-5.046274095	-7.012415452	1.140133167	0.379678221

UBN2	0.012690389	-0.061013801	-0.020194662	-0.07370419	-0.032885051	0.040819139	8	0.047095959	0.816496581	0.816496581	0.816496581	0.816496581	0.816496581	0.816496581	26	72	0	P_263155	P_263155	P_263155	chr7	139237031	139237091	+	UBN2	ubinuclein 2 [Source:HGNC Symbol;Acc:HGNC:21931]	ENSG00000157741	protein_coding	"5'UTR,exon;exon"	multiple Ref	1	4322	7:139237031:139237091	ENSG00000157741	UBN2	7.692010201	0.003987882	0.24612141	-1.183320905	-0.391661643	-1.429442315	-0.637783053	0.791659262	9.961963646	0.810585147	0.264151029	0.703555481	0.18347825	0.537997578	0.446999375	-6.965405517	-5.493372918	-6.9639911	-6.133806938	-6.084774563	-6.739408342	0.78183665	0.530779326

ELOB	0.04826638	-0.008110693	-0.080951637	-0.056377073	-0.129218016	-0.072840944	8	0.048480079	0.816496581	0.816496581	0.816496581	0.816496581	0.816496581	0.816496581	26	75	0	A_23_P100392	A_23_P100392	A_23_P100392	chr16	2771528	2771588	-	TCEB2	"transcription elongation factor B (SIII), polypeptide 2 (18kDa, elongin B) [Source:HGNC Symbol;Acc:HGNC:11619]"	ENSG00000103363	protein_coding	3'UTR;exon	multiple Ref	1	4325	16:2771528:2771588	ENSG00000103363	ELOB	10.60594481	0.004094118	0.923868593	-0.155247077	-1.549498318	-1.079115671	-2.473366911	-1.394251241	9.961963646	0.377389708	0.879727071	0.152420863	0.305970314	0.032998355	0.193555496	-6.551630299	-6.169397702	-5.873419854	-6.547553029	-3.783396426	-6.101891049	2.084091817	0.166266634

H1-12P	0.098194365	0.105068923	-0.161536132	0.006874558	-0.259730497	-0.266605055	8	0.196817004	0.816496581	0.816496581	0.816496581	0.816496581	0.816496581	0.816496581	26	77	0	P_313385	P_313385	P_313385	chr6	26193446	26193506	+	HIST1H1PS1;HIST1H4D	"histone cluster 1, H1, pseudogene 1 [Source:HGNC Symbol;Acc:HGNC:19163];histone cluster 1, H4d [Source:HGNC Symbol;Acc:HGNC:4782]"	ENSG00000216331	processed_pseudogene;protein_coding	promoter	multiple non-Ref	0	4327	6:26193446:26193506	ENSG00000216331	H1-12P	3.950095349	0.033314553	0.658893459	0.705022395	-1.083922699	0.046128935	-1.742816159	-1.788945094	9.961963646	0.524900501	0.496948925	0.303934803	0.964119101	0.112089802	0.104020244	-6.766511129	-5.926645177	-6.440863347	-7.148449523	-4.893366404	-5.556904882	1.390991029	0.302024358

SETD5	-0.057241996	-0.208775801	-0.08662112	-0.151533805	-0.029379123	0.122154682	8	0.070923247	0.816496581	0.816496581	0.816496581	0.816496581	0.816496581	0.816496581	26	78	0	P_243140	P_243140	P_243140	chr3	9448473	9448533	+	SETD5	SET domain containing 5 [Source:HGNC Symbol;Acc:HGNC:25566]	ENSG00000168137	protein_coding	3'UTR;exon	multiple Ref	1	4328	3:9448473:9448533	ENSG00000168137	SETD5	8.195994303	0.006246134	-0.887063579	-3.235341549	-1.342343822	-2.34827797	-0.455280243	1.892997727	9.961963646	0.395955234	0.008982875	0.209273289	0.040851246	0.65866915	0.087740572	-6.585212163	-2.481448393	-6.14321638	-4.753320806	-6.187604686	-5.401132951	3.726888931	0.049557719

FBXO25	-0.144941152	0.059166939	-0.165954432	0.20410809	-0.02101328	-0.22512137	8	0.061293196	0.816496581	0.816496581	0.816496581	0.816496581	0.816496581	0.816496581	26	79	0	P_146445	P_146445	P_146445	chr8	463051	463111	+	FBXO25	F-box protein 25 [Source:HGNC Symbol;Acc:HGNC:13596]	ENSG00000147364	protein_coding	exon	coding exon	1	4329	8:463051:463111	ENSG00000147364	FBXO25	8.787009879	0.005223644	-2.456125468	1.002623639	-2.812209655	3.458749107	-0.356084187	-3.814833294	9.961963646	0.033985337	0.339773821	0.018469148	0.006169988	0.72920304	0.003425699	-4.430342072	-5.678256933	-3.876341893	-2.865570646	-6.229683929	-2.191996483	6.931504003	0.008407364

PKIA	0.348675008	0.200644452	0.511599993	-0.148030556	0.162924985	0.310955541	8	0.169318802	0.816496581	0.816496581	0.816496581	0.816496581	0.816496581	0.816496581	26	80	0	A_23_P31765	A_23_P31765	A_23_P31765	chr8	78603070	78603130	+	PKIA	"protein kinase (cAMP-dependent, catalytic) inhibitor alpha [Source:HGNC Symbol;Acc:HGNC:9017]"	ENSG00000171033	protein_coding	3'UTR	3'UTR	1	4330	8:78603070:78603130	ENSG00000171033	PKIA	7.803514312	0.025229339	2.688521485	1.547105204	3.94478394	-1.141416281	1.256262455	2.397678736	9.961963646	0.022828539	0.152992018	0.002773833	0.280391344	0.237681614	0.037552161	-4.040576407	-5.056706549	-1.953655872	-6.480285103	-5.519470319	-4.59667824	5.635482071	0.016032241

SEC11A	-0.034357443	-0.017655306	-0.080788947	0.016702137	-0.046431505	-0.063133642	8	0.113371736	0.816496581	0.816496581	0.816496581	0.816496581	0.816496581	0.816496581	26	81	0	P_117387	P_117387	P_117387	chr15	84687624	84687684	-	SEC11A	SEC11 homolog A (S. cerevisiae) [Source:HGNC Symbol;Acc:HGNC:17718]	ENSG00000140612	protein_coding	exon	coding exon	1	4331	15:84687624:84687684	ENSG00000140612	SEC11A	9.791510392	0.012528465	-0.375939131	-0.19318435	-0.883992645	0.182754781	-0.508053514	-0.690808296	9.961963646	0.714844021	0.850696421	0.397532728	0.858656108	0.622475291	0.505463174	-6.921783891	-6.162451574	-6.63639557	-7.131378833	-6.161202157	-6.816768318	0.288137867	0.832995331

RTF2	0.073500085	-0.033447702	0.04182221	-0.106947787	-0.031677875	0.075269912	8	0.066007503	0.816496581	0.816496581	0.816496581	0.816496581	0.816496581	0.816496581	26	83	0	P_241149	P_241149	P_241149	chr20	56513342	56513402	+	RTFDC1	replication termination factor 2 domain containing 1 [Source:HGNC Symbol;Acc:HGNC:15890]	ENSG00000022277	protein_coding	exon	coding exon	1	4333	20:56513342:56513402	ENSG00000022277	RTF2	9.859759336	0.005705585	1.191745131	-0.542327756	0.678113704	-1.734072888	-0.513631427	1.22044146	9.961963646	0.260978274	0.599513737	0.513141857	0.113682181	0.618708441	0.250398887	-6.27424998	-6.029374664	-6.801663387	-5.713796481	-6.158250955	-6.313438197	1.155752905	0.374243389

C22orf46	0.087473785	-0.084760619	0.039029791	-0.172234405	-0.048443994	0.123790411	8	0.054304498	0.816496581	0.816496581	0.816496581	0.816496581	0.816496581	0.816496581	26	84	0	P_133520	P_133520	P_133520	chr22	41693918	41693978	+	C22orf46	chromosome 22 open reading frame 46 [Source:HGNC Symbol;Acc:HGNC:26294]	ENSG00000184208	protein_coding	exon	coding exon	1	4334	22:41693918:41693978	ENSG00000184208	C22orf46	8.845160144	0.004574874	1.58392261	-1.534794231	0.70672795	-3.118716841	-0.87719466	2.241522181	9.961963646	0.144410244	0.155960022	0.495933261	0.010946179	0.401040317	0.048974894	-5.778531668	-5.072698653	-6.781024538	-3.447915143	-5.902950313	-4.852618634	3.480692577	0.058454657

GLCE	0.23049392	0.070124701	0.264607868	-0.160369219	0.034113948	0.194483167	8	0.136856361	0.816496581	0.816496581	0.816496581	0.816496581	0.816496581	0.816496581	26	85	0	P_116935	P_116935	P_116935	chr15	69269184	69269244	+	GLCE	glucuronic acid epimerase [Source:HGNC Symbol;Acc:HGNC:17855]	ENSG00000138604	protein_coding	exon	coding exon	1	4335	15:69269184:69269244	ENSG00000138604	GLCE	6.809511379	0.017247625	2.149514855	0.653961224	2.467650958	-1.495553631	0.318136103	1.813689734	9.961963646	0.057213042	0.527943535	0.033322391	0.165759167	0.756950564	0.099914268	-4.930635701	-5.961536163	-4.457410886	-6.046867313	-6.24313336	-5.520254037	2.794123724	0.095350243

KY	-0.10855468	-0.030573845	-0.006955679	0.077980835	0.101599001	0.023618166	8	0.136778333	0.816496581	0.816496581	0.816496581	0.816496581	0.816496581	0.816496581	26	87	0	P_136093	P_136093	P_136093	chr3	134603312	134603372	-	KY	kyphoscoliosis peptidase [Source:HGNC Symbol;Acc:HGNC:26576]	ENSG00000174611	protein_coding	3'UTR	3'UTR	1	4337	3:134603312:134603372	ENSG00000174611	KY	4.551633868	0.017230479	-1.012850874	-0.285264031	-0.064898777	0.727586843	0.947952096	0.220365254	9.961963646	0.335099178	0.781284754	0.949538457	0.483615779	0.365581709	0.830037151	-6.465759703	-6.139370084	-7.044820957	-6.867586333	-5.839742873	-7.044914472	0.431335158	0.735170183

STOM	0.123900258	0.297482843	0.13714765	0.173582585	0.013247393	-0.160335193	8	0.22378618	0.816496581	0.816496581	0.816496581	0.816496581	0.816496581	0.816496581	26	88	0	P_149553	P_149553	P_149553	chr9	121339473	121339533	-	STOM	stomatin [Source:HGNC Symbol;Acc:HGNC:3383]	ENSG00000148175	protein_coding	3'UTR	3'UTR	1	4338	9:121339473:121339533	ENSG00000148175	STOM	4.553917774	0.042423859	0.736737463	1.768896686	0.815509297	1.032159222	0.078771833	-0.953387389	9.961963646	0.47827324	0.107459816	0.433845831	0.32640469	0.93877393	0.362954035	-6.710019463	-4.756528077	-6.695622506	-6.596278719	-6.293385961	-6.596331717	1.052889694	0.411667172

CERS3	0.083702728	-0.186155645	0.064091167	-0.269858372	-0.019611561	0.250246812	8	0.142548395	0.816496581	0.816496581	0.816496581	0.816496581	0.816496581	0.816496581	26	90	0	P_225142	P_225142	P_225142	chr15	100469400	100469460	-	CERS3	ceramide synthase 3 [Source:HGNC Symbol;Acc:HGNC:23752]	ENSG00000154227	protein_coding	exon	coding exon	1	4340	15:100469400:100469460	ENSG00000154227	CERS3	4.309992829	0.018524788	0.753197061	-1.675117271	0.576722886	-2.428314333	-0.176474175	2.251840157	9.961963646	0.468757769	0.124966124	0.576925221	0.035638976	0.863457464	0.048125869	-6.697349092	-4.886106708	-6.868467732	-4.620840017	-6.280147338	-4.83586824	2.450761783	0.123926226

PGAP2	-0.117585128	-0.104195529	0.01560548	0.013389599	0.133190608	0.119801009	8	0.063554671	0.816496581	0.816496581	0.816496581	0.816496581	0.816496581	0.816496581	26	91	0	P_107698	P_107698	P_107698	chr11	3826101	3826161	-	PGAP2;RHOG	post-GPI attachment to proteins 2 [Source:HGNC Symbol;Acc:HGNC:17893];ras homolog family member G [Source:HGNC Symbol;Acc:HGNC:672]	ENSG00000148985	protein_coding	3'ext;3'UTR	multiple non-Ref	0	4341	11:3826101:3826161	ENSG00000148985	PGAP2	7.14931118	0.005450379	-1.950673949	-1.72854771	0.258886507	0.222126239	2.209560456	1.987434217	9.961963646	0.079769772	0.114699037	0.800993474	0.828703177	0.051697831	0.075049124	-5.24231606	-4.81272565	-7.010642564	-7.122697635	-4.199350606	-5.256242774	2.623570084	0.108443109

AP3S2	-0.13933692	0.071659451	-0.104832293	0.21099637	0.034504627	-0.176491743	8	0.063946119	0.816496581	0.816496581	0.816496581	0.816496581	0.816496581	0.816496581	26	94	0	P_224946	P_224946	P_224946	chr15	89834346	89834406	+	AP3S2	"adaptor-related protein complex 3, sigma 2 subunit [Source:HGNC Symbol;Acc:HGNC:571]"	ENSG00000157823	protein_coding	3'ext;3'UTR	multiple non-Ref	0	4344	15:89834346:89834406	ENSG00000157823	AP3S2	8.781391101	0.005490459	-2.303071927	1.184444649	-1.73275189	3.487516576	0.570320037	-2.917196539	9.961963646	0.044117197	0.263726032	0.11392455	0.005880613	0.581094936	0.015432562	-4.682554225	-5.492150442	-5.615490822	-2.816502005	-6.126537421	-3.719954566	5.117925167	0.021265266

SUPT6H	0.074468101	-0.074856668	0.020546006	-0.149324769	-0.053922095	0.095402674	8	0.043850475	0.816496581	0.816496581	0.816496581	0.816496581	0.816496581	0.816496581	26	96	0	P_228138	P_228138	P_228138	chr17	28675422	28675482	+	SUPT6H	suppressor of Ty 6 homolog (S. cerevisiae) [Source:HGNC Symbol;Acc:HGNC:11470]	ENSG00000109111	protein_coding	exon	coding exon	1	4346	17:28675422:28675482	ENSG00000109111	SUPT6H	7.558522887	0.003750849	1.489193503	-1.496963961	0.41087362	-2.986157464	-1.078319884	1.907837581	9.961963646	0.167396879	0.165397902	0.689863718	0.013718001	0.306308292	0.085621553	-5.906687784	-5.121357812	-6.955706012	-3.675287894	-5.71284371	-5.37857592	3.057861655	0.078596483

BBS2	0.207005272	-0.035377287	0.16315212	-0.242382559	-0.043853152	0.198529407	8	0.030440693	0.816496581	0.816496581	0.816496581	0.816496581	0.816496581	0.816496581	26	98	0	P_226440	P_226440	P_226440	chr16	56498436	56498496	-	BBS2	Bardet-Biedl syndrome 2 [Source:HGNC Symbol;Acc:HGNC:967]	ENSG00000125124	protein_coding	3'UTR;exon	multiple Ref	1	4348	16:56498436:56498496	ENSG00000125124	BBS2	8.146453185	0.002950823	4.667189696	-0.79762465	3.678466189	-5.464814346	-0.988723507	4.476090839	9.961963646	0.000893655	0.443684984	0.004284402	0.000278773	0.346204485	0.001197433	-0.743308588	-5.8574016	-2.399753071	0.347265132	-5.801478783	-1.106838736	14.47118958	0.000580655

ACP3	0.346395804	0.085304452	0.349006542	-0.261091351	0.002610739	0.26370209	8	0.048639131	0.816496581	0.816496581	0.816496581	0.816496581	0.816496581	0.816496581	26	99	0	P_136029	P_136029	P_136029	chr3	132367730	132367790	+	ACPP	"acid phosphatase, prostate [Source:HGNC Symbol;Acc:HGNC:125]"	ENSG00000014257	protein_coding	exon	coding exon	1	4349	3:132367730:132367790	ENSG00000014257	ACP3	6.212132818	0.004106522	6.62035315	1.630347692	6.670249863	-4.990005458	0.049896714	5.039902172	9.961963646	6.03E-05	0.134198247	5.67E-05	0.000551621	0.961190948	0.000512666	2.050689204	-4.946635903	2.084180531	-0.36614784	-6.295361199	-0.225496851	23.54710012	7.68E-05

CCDC81	-0.023997725	-0.142341706	0.020816411	-0.11834398	0.044814136	0.163158117	8	0.155792276	0.816496581	0.816496581	0.816496581	0.816496581	0.816496581	0.816496581	26	100	0	P_212673	P_212673	P_212673	chr11	86400740	86400800	+	CCDC81	coiled-coil domain containing 81 [Source:HGNC Symbol;Acc:HGNC:26281]	ENSG00000149201	protein_coding	exon	coding exon	1	4350	11:86400740:86400800	ENSG00000149201	CCDC81	4.420427564	0.021697808	-0.199529805	-1.18350437	0.173078673	-0.983974565	0.372608478	1.356583043	9.961963646	0.845862201	0.264081606	0.86605568	0.348421931	0.717244788	0.204858372	-6.976678791	-5.493173393	-7.03078593	-6.644455405	-6.223366479	-6.149399437	0.736301923	0.554023117

ATG3	0.375827623	-0.078946217	0.407773726	-0.45477384	0.031946103	0.486719944	8	0.046637335	0.816496581	0.816496581	0.816496581	0.816496581	0.816496581	0.816496581	26	101	0	P_135587	P_135587	P_135587	chr3	112561691	112561751	-	ATG3	autophagy related 3 [Source:HGNC Symbol;Acc:HGNC:20962]	ENSG00000144848	protein_coding	5'UTR;exon	multiple Ref	1	4351	3:112561691:112561751	ENSG00000144848	ATG3	11.35986111	0.003953361	7.320674803	-1.537778355	7.942946877	-8.858453158	0.622272073	9.480725232	9.961963646	2.59E-05	0.155235993	1.28E-05	4.90E-06	0.547740552	2.66E-06	2.922611731	-5.068829231	3.621612286	4.563171085	-6.094758491	5.222762807	47.96516879	3.15E-06

MAT2B	-0.236171077	0.02536309	-0.247705666	0.261534168	-0.011534589	-0.273068756	8	0.065378633	0.816496581	0.816496581	0.816496581	0.816496581	0.816496581	0.816496581	26	102	0	P_255639	P_255639	P_255639	chr5	163516591	163516651	+	MAT2B	"methionine adenosyltransferase II, beta [Source:HGNC Symbol;Acc:HGNC:6905]"	ENSG00000038274	protein_coding	exon	coding exon	1	4352	5:163516591:163516651	ENSG00000038274	MAT2B	9.044296449	0.005639233	-3.851787635	0.413654536	-4.039908835	4.265442171	-0.1881212	-4.453563371	9.961963646	0.003225444	0.687891394	0.002379917	0.001663075	0.854558402	0.00123988	-2.066055626	-6.092681002	-1.795991213	-1.515108091	-6.277896474	-1.142946939	11.56530911	0.001385775

MICAL2	0.051184424	0.022335828	0.06784222	-0.028848596	0.016657796	0.045506392	8	0.0798369	0.816496581	0.816496581	0.816496581	0.816496581	0.816496581	0.816496581	26	103	0	P_210995	P_210995	P_210995	chr11	12260010	12260070	+	MICAL2	"microtubule associated monooxygenase, calponin and LIM domain containing 2 [Source:HGNC Symbol;Acc:HGNC:24693]"	ENSG00000133816	protein_coding	exon	coding exon	1	4353	11:12260010:12260070	ENSG00000133816	MICAL2	6.167692677	0.007325298	0.732437895	0.319620806	0.970807313	-0.412817089	0.238369418	0.651186507	9.961963646	0.480778864	0.755857803	0.354624678	0.688485083	0.816426194	0.52966001	-6.713287929	-6.128518636	-6.555511985	-7.057210119	-6.266552187	-6.844550546	0.372061894	0.77502627

PADI6	-0.291802047	-0.215192389	0.101733634	0.076609659	0.393535682	0.316926023	8	0.235426622	0.816496581	0.816496581	0.816496581	0.816496581	0.816496581	0.816496581	26	106	0	P_200469	P_200469	P_200469	chr1	17382016	17382076	+	PADI6	"peptidyl arginine deiminase, type VI [Source:HGNC Symbol;Acc:HGNC:20449]"	ENSG00000276747	protein_coding	exon	coding exon	1	4356	1:17382016:17382076	ENSG00000276747	PADI6	4.129526619	0.046716539	-1.653478816	-1.219374775	0.576467542	0.434104041	2.229946358	1.795842317	9.961963646	0.129354651	0.250785863	0.577091195	0.673464053	0.049944676	0.102860457	-5.681456836	-5.453725992	-6.868623308	-7.047526497	-4.16766476	-5.546714531	2.156556428	0.156698868

ANKRD2	0.099117394	-0.103169649	-0.003771497	-0.202287043	-0.102888891	0.099398152	8	0.160254782	0.816496581	0.816496581	0.816496581	0.816496581	0.816496581	0.816496581	26	108	0	P_106759	P_106759	P_106759	chr10	97583820	97583880	+	ANKRD2	ankyrin repeat domain 2 (stretch responsive muscle) [Source:HGNC Symbol;Acc:HGNC:495]	ENSG00000165887	protein_coding	3'UTR	3'UTR	1	4358	10:97583820:97583880	ENSG00000165887	ANKRD2	8.051260433	0.022830405	0.803412706	-0.836258937	-0.030570502	-1.639671643	-0.833983208	0.805688435	9.961963646	0.440484672	0.422614656	0.976215858	0.132226656	0.423836759	0.439230584	-6.657161673	-5.82630207	-7.046610256	-5.849019092	-5.939496306	-6.72790795	0.896486747	0.476513704

TAF5L	-0.103101977	0.180651174	-0.001721662	0.283753151	0.101380314	-0.182372837	8	0.106793113	0.816496581	0.816496581	0.816496581	0.816496581	0.816496581	0.816496581	26	110	0	P_105039	P_105039	P_105039	chr1	229593188	229593248	-	TAF5L	"TAF5-like RNA polymerase II, p300/CBP-associated factor (PCAF)-associated factor, 65kDa [Source:HGNC Symbol;Acc:HGNC:17304]"	ENSG00000135801	protein_coding	3'UTR	3'UTR	1	4360	1:229593188:229593248	ENSG00000135801	TAF5L	7.153844834	0.011365335	-1.184462993	2.075368845	-0.019778917	3.259831838	1.164684076	-2.095147762	9.961963646	0.263719099	0.064803984	0.984610281	0.008618727	0.271279213	0.062689988	-6.282511055	-4.310903085	-7.046906965	-3.205869672	-5.621755683	-5.087377004	3.680522731	0.051102974

RXFP2	-0.034113322	0.065819921	0.083956822	0.099933243	0.118070144	0.018136901	8	0.137770651	0.816496581	0.816496581	0.816496581	0.816496581	0.816496581	0.816496581	26	112	0	P_113538	P_113538	P_113538	chr13	31782687	31782747	+	RXFP2	relaxin/insulin-like family peptide receptor 2 [Source:HGNC Symbol;Acc:HGNC:17318]	ENSG00000133105	protein_coding	exon	coding exon	1	4362	13:31782687:31782747	ENSG00000133105	RXFP2	3.63373883	0.017449263	-0.316286823	0.610259348	0.778418372	0.926546171	1.094705195	0.168159024	9.961963646	0.758312445	0.555355014	0.454414062	0.376063605	0.299406859	0.869823128	-6.944045208	-5.989490436	-6.72595831	-6.699387511	-5.69597854	-7.05595249	0.52725562	0.673544345

SNAP23	0.108291796	0.037934168	-0.028128139	-0.070357628	-0.136419935	-0.066062306	8	0.084212815	0.816496581	0.816496581	0.816496581	0.816496581	0.816496581	0.816496581	26	116	0	P_116311	P_116311	P_116311	chr15	42531418	42531478	+	SNAP23	"synaptosomal-associated protein, 23kDa [Source:HGNC Symbol;Acc:HGNC:11131]"	ENSG00000092531	protein_coding	3'UTR;exon	multiple Ref	1	4366	15:42531418:42531478	ENSG00000092531	SNAP23	7.85596161	0.007901785	1.49203356	0.522653177	-0.387546691	-0.969380383	-1.879580251	-0.910199867	9.961963646	0.166663846	0.612640402	0.706502804	0.355301683	0.089698405	0.384211189	-5.90291695	-6.040101947	-6.965715674	-6.658674635	-4.697334738	-6.63674891	1.325084495	0.320546832

EID2B	0.26672	0.013056517	0.080127622	-0.253663482	-0.186592378	0.067071105	8	0.069817564	0.816496581	0.816496581	0.816496581	0.816496581	0.816496581	0.816496581	26	117	0	P_125772	P_125772	P_125772	chr19	39531844	39531904	-	EID2B	EP300 interacting inhibitor of differentiation 2B [Source:HGNC Symbol;Acc:HGNC:26796]	ENSG00000176401	protein_coding	3'UTR	3'UTR	1	4367	19:39531844:39531904	ENSG00000176401	EID2B	8.195466933	0.006121167	4.175265484	0.204388218	1.254326987	-3.970877266	-2.920938497	1.049938769	9.961963646	0.001917751	0.84216539	0.238355046	0.002659402	0.015334178	0.318549461	-1.531782084	-6.16011304	-6.249818672	-2.001005601	-3.059786672	-6.50041855	7.408345331	0.006758888

COA8	0.008424129	-0.003522123	0.005908263	-0.011946251	-0.002515866	0.009430386	8	0.061968849	0.816496581	0.816496581	0.816496581	0.816496581	0.816496581	0.816496581	26	120	0	P_222363	P_222363	P_222363	chr14	103571760	103571820	+	APOPT1;KLC1	"apoptogenic 1, mitochondrial [Source:HGNC Symbol;Acc:HGNC:20492];kinesin light chain 1 [Source:EntrezGene;Acc:3831]"	ENSG00000256053	protein_coding	3'UTR;exon	multiple Ref	1	4370	14:103571760:103571820	ENSG00000256053	COA8	10.0894856	0.005290524	0.141847359	-0.059306288	0.099484651	-0.201153647	-0.042362708	0.15879094	9.961963646	0.890028198	0.953880984	0.922726973	0.844626163	0.967046607	0.877006551	-6.987391787	-6.180233578	-7.041718346	-7.127530405	-6.295730823	-7.057621248	0.016834509	0.996823609

MERTK	0.119768235	0.052959164	0.085760544	-0.066809071	-0.034007691	0.03280138	8	0.066512058	0.816496581	0.816496581	0.816496581	0.816496581	0.816496581	0.816496581	26	121	0	P_128679	P_128679	P_128679	chr2	111906152	111906212	-	MERTK	"MER proto-oncogene, tyrosine kinase [Source:HGNC Symbol;Acc:HGNC:7027]"	ENSG00000153208	protein_coding	intron	intron antisense	0	4371	2:111906152:111906212	ENSG00000153208	MERTK	5.61596813	0.00575928	1.932872425	0.854678269	1.384041361	-1.078194156	-0.548831064	0.529363092	9.961963646	0.082154633	0.412811178	0.19656574	0.306361716	0.595207367	0.608147107	-5.269598733	-5.811028066	-6.090966803	-6.548525814	-6.138926074	-6.920419012	1.354330784	0.312174001

GFM1	-0.373643786	0.071897074	-0.301427529	0.44554086	0.072216257	-0.373324603	8	0.056629365	0.816496581	0.816496581	0.816496581	0.816496581	0.816496581	0.816496581	26	123	0	P_136415	P_136415	P_136415	chr3	158660901	158660961	+	GFM1	"G elongation factor, mitochondrial 1 [Source:HGNC Symbol;Acc:HGNC:13780]"	ENSG00000168827	protein_coding	exon	coding exon	1	4373	3:158660901:158660961	ENSG00000168827	GFM1	9.261019086	0.004781987	-6.617579637	1.273364187	-5.338562428	7.890943824	1.279017209	-6.611926615	9.961963646	6.05E-05	0.23179849	0.000333194	1.36E-05	0.229880261	6.10E-05	2.04710627	-5.392750468	0.242065314	3.505393128	-5.493189285	1.992639748	30.25575307	2.56E-05

ARL6IP5	0.246290334	-0.134780666	0.039139085	-0.381071	-0.20715125	0.17391975	8	0.09793856	0.816496581	0.816496581	0.816496581	0.816496581	0.816496581	0.816496581	26	124	0	P_135327	P_135327	P_135327	chr3	69104772	69104832	+	ARL6IP5	ADP-ribosylation factor-like 6 interacting protein 5 [Source:HGNC Symbol;Acc:HGNC:16937]	ENSG00000144746	protein_coding	3'UTR	3'UTR	1	4374	3:69104772:69104832	ENSG00000144746	ARL6IP5	11.63454077	0.009909552	3.030162964	-1.658235526	0.481536576	-4.68839849	-2.548626388	2.139772103	9.961963646	0.012726303	0.128378242	0.640537945	0.000865376	0.029011368	0.058159608	-3.45929409	-4.909036219	-6.921951973	-0.835871685	-3.662830909	-5.016407367	7.53645194	0.006383996

ZNF420	0.070993075	-0.17905274	0.006926734	-0.250045815	-0.064066341	0.185979475	8	0.169346846	0.816496581	0.816496581	0.816496581	0.816496581	0.816496581	0.816496581	26	126	0	P_232811	P_232811	P_232811	chr19	37091061	37091121	+	ZNF420	zinc finger protein 420 [Source:HGNC Symbol;Acc:HGNC:20649]	ENSG00000197050	protein_coding	exon	coding exon	1	4376	19:37091061:37091121	ENSG00000197050	ZNF420	7.8938265	0.025236966	0.547322175	-1.380409788	0.05340176	-1.927731963	-0.493920415	1.433811549	9.961963646	0.59620507	0.19764597	0.958467523	0.082855618	0.632070731	0.182258927	-6.837321205	-5.26644632	-7.045563458	-5.424356559	-6.16854291	-6.051061382	1.370644659	0.307610648

PDYN	-0.050766603	-0.074681711	-0.01363587	-0.023915108	0.037130733	0.061045841	8	0.144376893	0.816496581	0.816496581	0.816496581	0.816496581	0.816496581	0.816496581	26	128	0	A_23_P40262	A_23_P40262	A_23_P40262	chr20	1980287	1980347	-	PDYN	prodynorphin [Source:HGNC Symbol;Acc:HGNC:8820]	ENSG00000101327	protein_coding	"3'UTR,exon"	multiple Ref	1	4378	20:1980287:1980347	ENSG00000101327	PDYN	4.028789813	0.018946104	-0.451714228	-0.66450756	-0.12133009	-0.212793332	0.330384138	0.54317747	9.961963646	0.661150005	0.521449447	0.905842019	0.835779407	0.747952873	0.598950147	-6.888148326	-5.954525066	-7.039087297	-7.124906845	-6.238954141	-6.91255347	0.184968913	0.904191117

FAM162A	0.041684818	0.114010373	0.006052986	0.072325555	-0.035631832	-0.107957387	8	0.059177161	0.816496581	0.816496581	0.816496581	0.816496581	0.816496581	0.816496581	26	131	0	P_245854	P_245854	P_245854	chr3	122404303	122404363	+	FAM162A	"family with sequence similarity 162, member A [Source:HGNC Symbol;Acc:HGNC:17865]"	ENSG00000114023	protein_coding	exon	coding exon	1	4381	3:122404303:122404363	ENSG00000114023	FAM162A	10.57433183	0.00501893	0.72063935	1.970990045	0.104642888	1.250350694	-0.615996463	-1.866347157	9.961963646	0.487696949	0.077127412	0.91873606	0.23974345	0.551710997	0.09166902	-6.722168732	-4.466027173	-7.041143931	-6.355823588	-6.098733482	-5.441435242	1.640192196	0.242174567

SSX2IP	0.064884722	-0.048878869	0.075476795	-0.113763592	0.010592073	0.124355664	8	0.070564826	0.816496581	0.816496581	0.816496581	0.816496581	0.816496581	0.816496581	26	132	0	P_202530	P_202530	P_202530	chr1	84664472	84664532	-	SSX2IP	"synovial sarcoma, X breakpoint 2 interacting protein [Source:HGNC Symbol;Acc:HGNC:16509]"	ENSG00000117155	protein_coding	exon	coding exon	1	4382	1:84664472:84664532	ENSG00000117155	SSX2IP	9.435457392	0.006205409	1.008794856	-0.759943951	1.173475049	-1.768738807	0.164680193	1.933419	9.961963646	0.336947338	0.464892534	0.26789807	0.107487311	0.872489274	0.082080426	-6.469814636	-5.886490828	-6.34308933	-5.663119854	-6.282280973	-5.339502504	1.644315187	0.241305257

EIF2A	0.073996468	-0.025674985	0.009547104	-0.099671454	-0.064449364	0.035222089	8	0.039893974	0.816496581	0.816496581	0.816496581	0.816496581	0.816496581	0.816496581	26	133	0	P_246742	P_246742	P_246742	chr3	150575700	150575760	+	EIF2A	"eukaryotic translation initiation factor 2A, 65kDa [Source:HGNC Symbol;Acc:HGNC:3254]"	ENSG00000144895	protein_coding	3'UTR;exon	multiple Ref	1	4383	3:150575700:150575760	ENSG00000144895	EIF2A	11.05287193	0.003484769	1.535216553	-0.532683023	0.198075294	-2.067899576	-1.337141259	0.730758317	9.961963646	0.155857376	0.605930212	0.846969721	0.065619825	0.210906105	0.4817599	-5.845029819	-6.034679982	-7.025737004	-5.206264842	-5.424561609	-6.787251985	1.546339669	0.262967958

ATF6	0.090178407	-0.08795674	-0.071298965	-0.178135146	-0.161477371	0.016657775	8	0.074413107	0.816496581	0.816496581	0.816496581	0.816496581	0.816496581	0.816496581	26	134	0	P_204565	P_204565	P_204565	chr1	161791456	161791516	+	ATF6	activating transcription factor 6 [Source:HGNC Symbol;Acc:HGNC:791]	ENSG00000118217	protein_coding	exon	coding exon	1	4384	1:161791456:161791516	ENSG00000118217	ATF6	8.228682148	0.006653446	1.354019324	-1.320661227	-1.070546481	-2.674680551	-2.424565806	0.250114746	9.961963646	0.205647443	0.216148708	0.30962477	0.023376509	0.035867829	0.807580938	-6.080701504	-5.337806486	-6.454947278	-4.20685855	-3.861194516	-7.037310819	2.969762826	0.083773433

SLC13A2	-0.167929099	0.042139595	-0.025317751	0.210068694	0.142611347	-0.067457346	8	0.082191682	0.816496581	0.816496581	0.816496581	0.816496581	0.816496581	0.816496581	26	135	0	A_16_P03228655	A_16_P03228655	A_16_P03228655	chr17	28489877	28489937	+	SLC13A2;RP11-192H23.4	"solute carrier family 13 (sodium-dependent dicarboxylate transporter), member 2 [Source:HGNC Symbol;Acc:HGNC:10917];Uncharacterized protein [Source:UniProtKB/TrEMBL;Acc:E9PMD0]"	ENSG00000007216	protein_coding	intron	multiple non-Ref	0	4385	17:28489877:28489937	ENSG00000007216	SLC13A2	5.068663297	0.007631697	-2.354295834	0.590779519	-0.354944301	2.945075353	1.999351532	-0.945723821	9.961963646	0.040434679	0.567828686	0.730030811	0.014714662	0.073576047	0.36666292	-4.598631929	-6.001375168	-6.978753279	-3.745684228	-4.520214806	-6.603618026	3.263830617	0.067901782

SLC16A6	-0.30532452	0.001046746	-0.233575133	0.306371266	0.071749388	-0.234621878	8	0.141652634	0.816496581	0.816496581	0.816496581	0.816496581	0.816496581	0.816496581	26	137	0	P_122406	P_122406	P_122406	chr17	68268465	68268525	-	SLC16A6	"solute carrier family 16, member 6 [Source:HGNC Symbol;Acc:HGNC:10927]"	ENSG00000108932	protein_coding	3'UTR	3'UTR	1	4387	17:68268465:68268525	ENSG00000108932	SLC16A6	6.508682008	0.018320349	-2.762742682	0.009471525	-2.113515115	2.772214207	0.649227567	-2.12298664	9.961963646	0.020102188	0.992629922	0.060785881	0.019778641	0.530873809	0.059825703	-3.914875804	-6.182040132	-5.034836136	-4.041080695	-6.077263362	-5.043167897	4.118920549	0.038492047

SLC28A3	0.004625717	-0.05365824	0.096405575	-0.058283957	0.091779858	0.150063815	8	0.127058883	0.816496581	0.816496581	0.816496581	0.816496581	0.816496581	0.816496581	26	139	0	P_267760	P_267760	P_267760	chr9	84279268	84279328	-	SLC28A3	"solute carrier family 28 (concentrative nucleoside transporter), member 3 [Source:HGNC Symbol;Acc:HGNC:16484]"	ENSG00000197506	protein_coding	exon	coding exon	1	4389	9:84279268:84279328	ENSG00000197506	SLC28A3	3.922687482	0.015171164	0.045995487	-0.533546902	0.958601997	-0.579542389	0.91260651	1.492148899	9.961963646	0.964222821	0.60535404	0.360445923	0.575094264	0.383003842	0.166634135	-6.997205983	-6.034208439	-6.567265404	-6.968984081	-5.87183172	-5.974423277	0.766822338	0.538334954

ALOX12B	0.389093723	0.00448212	0.259768074	-0.384611603	-0.129325649	0.255285954	8	0.140432702	0.816496581	0.816496581	0.816496581	0.816496581	0.816496581	0.816496581	26	141	0	P_120477	P_120477	P_120477	chr17	8072675	8072735	-	ALOX12B	"arachidonate 12-lipoxygenase, 12R type [Source:HGNC Symbol;Acc:HGNC:430]"	ENSG00000179477	protein_coding	3'UTR	3'UTR	1	4391	17:8072675:8072735	ENSG00000179477	ALOX12B	4.726042134	0.018043998	3.547590351	0.040866053	2.368454332	-3.506724298	-1.179136018	2.327588279	9.961963646	0.005320765	0.968210109	0.03947091	0.005695257	0.265738543	0.042315634	-2.577581444	-6.181207106	-4.621770075	-2.783764932	-5.60599442	-4.712174321	6.216964584	0.011879574

NQO1	0.412194376	0.044484286	0.478976915	-0.367710091	0.066782539	0.434492629	8	0.057898055	0.816496581	0.816496581	0.816496581	0.816496581	0.816496581	0.816496581	26	142	0	P_119424	P_119424	P_119424	chr16	69713027	69713087	-	NQO1	"NAD(P)H dehydrogenase, quinone 1 [Source:HGNC Symbol;Acc:HGNC:2874]"	ENSG00000181019	protein_coding	exon	coding exon	1	4392	16:69713027:69713087	ENSG00000181019	NQO1	11.54402376	0.004898671	7.212877407	0.778418428	8.381486911	-6.434458978	1.168609504	7.603068483	9.961963646	2.94E-05	0.45441403	8.01E-06	7.63E-05	0.269765277	1.87E-05	2.792561731	-5.872383464	4.106184672	1.704179939	-5.617489021	3.217210057	37.24256991	1.01E-05

ARL2	-0.179620134	0.167898479	0.021015856	0.347518613	0.20063599	-0.146882623	8	0.101337174	0.816496581	0.816496581	0.816496581	0.816496581	0.816496581	0.816496581	26	143	0	P_109060	P_109060	P_109060	chr11	65018403	65018463	+	ARL2	ADP-ribosylation factor-like 2 [Source:HGNC Symbol;Acc:HGNC:693]	ENSG00000213465	protein_coding	exon	coding exon	1	4393	11:65018403:65018463	ENSG00000213465	ARL2	11.9706211	0.01045343	-2.151648088	2.011235787	0.251746427	4.162883875	2.403394515	-1.75948936	9.961963646	0.057007766	0.07213425	0.806354356	0.001955806	0.037187722	0.109109354	-4.927229217	-4.406581377	-7.012620618	-1.683169651	-3.894841101	-5.600197682	5.823290885	0.014524508

ADAMTS16	0.075372965	0.154874845	0.196845816	0.07950188	0.121472852	0.041970971	8	0.160842024	0.816496581	0.816496581	0.816496581	0.816496581	0.816496581	0.816496581	26	146	0	A_24_P69084	A_24_P69084	A_24_P69084	chr5	5262684	5262744	+	ADAMTS16	"ADAM metallopeptidase with thrombospondin type 1 motif, 16 [Source:HGNC Symbol;Acc:HGNC:17108]"	ENSG00000145536	protein_coding	exon	coding exon	1	4396	5:5262684:5262744	ENSG00000145536	ADAMTS16	3.748698013	0.02298183	0.608932171	1.251221524	1.590301654	0.642289352	0.981369483	0.339080131	9.961963646	0.556199924	0.23943882	0.142967382	0.535185967	0.349642758	0.741588524	-6.799723374	-5.417987221	-5.817435665	-6.92858473	-5.808478163	-7.008929343	0.992668342	0.435447032

EIF2AK3	0.028068859	-0.015183038	-0.035834151	-0.043251896	-0.06390301	-0.020651114	8	0.092388403	0.816496581	0.816496581	0.816496581	0.816496581	0.816496581	0.816496581	26	147	0	P_235729	P_235729	P_235729	chr2	88570966	88571026	-	EIF2AK3	eukaryotic translation initiation factor 2-alpha kinase 3 [Source:HGNC Symbol;Acc:HGNC:3255]	ENSG00000172071	protein_coding	exon	coding exon	1	4397	2:88570966:88571026	ENSG00000172071	EIF2AK3	5.670439483	0.00906125	0.361140612	-0.195348577	-0.461050717	-0.556489189	-0.822191329	-0.26570214	9.961963646	0.72553554	0.849046885	0.654663867	0.590157315	0.43020741	0.795886282	-6.927655384	-6.162010032	-6.9322654	-6.982853871	-5.949192535	-7.032947774	0.2395713	0.866784814

EVI5	0.196741192	0.070823119	0.199548199	-0.125918074	0.002807007	0.12872508	8	0.054756388	0.816496581	0.816496581	0.816496581	0.816496581	0.816496581	0.816496581	26	148	0	P_202850	P_202850	P_202850	chr1	92695325	92695385	-	EVI5	ecotropic viral integration site 5 [Source:HGNC Symbol;Acc:HGNC:3501]	ENSG00000067208	protein_coding	exon	coding exon	1	4398	1:92695325:92695385	ENSG00000067208	EVI5	8.408936239	0.004614452	3.547160738	1.276910966	3.597769883	-2.270249772	0.050609144	2.320858916	9.961963646	0.005324567	0.230593439	0.004895602	0.0466463	0.960637345	0.042802872	-2.578308851	-5.388678951	-2.536108209	-4.881263577	-6.295323124	-4.723212467	6.283296745	0.011492767

TMEM263	0.219751925	0.031349622	-0.032306288	-0.188402303	-0.252058212	-0.063655909	8	0.113567384	0.816496581	0.816496581	0.816496581	0.816496581	0.816496581	0.816496581	26	149	0	P_112640	P_112640	P_112640	chr12	106970863	106970923	+	TMEM263	transmembrane protein 263 [Source:HGNC Symbol;Acc:HGNC:28281]	ENSG00000151135	protein_coding	intron;promoter	multiple non-Ref	0	4399	12:106970863:106970923	ENSG00000151135	TMEM263	7.545372068	0.01256412	2.401111195	0.342540469	-0.352993447	-2.058570726	-2.754104641	-0.695533915	9.961963646	0.037332889	0.739061646	0.731448342	0.066652499	0.020401897	0.502622699	-4.521489009	-6.12060883	-6.979498065	-5.220975997	-3.33094603	-6.813355151	3.052307759	0.078911445

KATNBL1	0.167159341	-0.020370066	-0.008673634	-0.187529407	-0.175832975	0.011696432	8	0.143522674	0.816496581	0.816496581	0.816496581	0.816496581	0.816496581	0.816496581	26	150	0	A_23_P77286	A_23_P77286	A_23_P77286	chr15	34141248	34141308	-	KATNBL1	katanin p80 subunit B-like 1 [Source:HGNC Symbol;Acc:HGNC:26199]	ENSG00000134152	protein_coding	3'UTR	3'UTR	1	4400	15:34141248:34141308	ENSG00000134152	KATNBL1	5.516854046	0.01874861	1.495174027	-0.182202166	-0.07758222	-1.677376193	-1.572756246	0.104619947	9.961963646	0.165856522	0.859078328	0.939696513	0.124515795	0.146966976	0.918753804	-5.898741977	-6.164616887	-7.043834564	-5.795513087	-5.126186563	-7.065402357	1.262143406	0.339434078

ADAM32	0.104873735	0.196701962	0.176333711	0.091828227	0.071459975	-0.020368252	8	0.259548679	0.816496581	0.816496581	0.816496581	0.816496581	0.816496581	0.816496581	26	151	0	P_264485	P_264485	P_264485	chr8	39164775	39164835	+	ADAM32	ADAM metallopeptidase domain 32 [Source:HGNC Symbol;Acc:HGNC:15479]	ENSG00000197140	protein_coding	exon	coding exon	1	4401	8:39164775:39164835	ENSG00000197140	ADAM32	3.999895876	0.056304867	0.541301708	1.015269533	0.910139593	0.473967825	0.368837885	-0.10512994	9.961963646	0.600194657	0.334000693	0.384241461	0.645739817	0.719966573	0.918359349	-6.840793544	-5.666103733	-6.612706278	-7.028134889	-6.224832561	-7.065343983	0.420645505	0.742269042

PTER	0.18149701	0.034796803	0.094873466	-0.146700207	-0.086623544	0.060076663	8	0.057541659	0.816496581	0.816496581	0.816496581	0.816496581	0.816496581	0.816496581	26	153	0	P_207528	P_207528	P_207528	chr10	16505100	16505160	+	PTER	phosphotriesterase related [Source:HGNC Symbol;Acc:HGNC:9590]	ENSG00000165983	protein_coding	exon	coding exon	1	4403	10:16505100:16505160	ENSG00000165983	PTER	9.246853236	0.004865632	3.186731629	0.610963632	1.665792045	-2.575767998	-1.520939584	1.054828413	9.961963646	0.00975318	0.554906946	0.126840711	0.027693812	0.159360406	0.316414421	-3.191810902	-5.989054063	-5.7116271	-4.374020186	-5.194389649	-6.495362725	3.893983192	0.044431359

CEP128	-0.375337517	-0.066077777	-0.242213068	0.30925974	0.133124449	-0.176135291	8	0.072862535	0.816496581	0.816496581	0.816496581	0.816496581	0.816496581	0.816496581	26	154	0	P_221728	P_221728	P_221728	chr14	80836204	80836264	-	CEP128	centrosomal protein 128kDa [Source:HGNC Symbol;Acc:HGNC:20359]	ENSG00000100629	protein_coding	exon	coding exon	1	4404	14:80836204:80836264	ENSG00000100629	CEP128	7.297253966	0.00647006	-5.714963581	-1.006113361	-3.687984284	4.708850219	2.026979296	-2.681870923	9.961963646	0.000197097	0.338173338	0.004217739	0.000839005	0.070265319	0.023090217	0.823878483	-5.674915447	-2.383702816	-0.803623835	-4.478720368	-4.120462305	13.458108	0.000773223

RANBP17	-0.135854381	-0.071397167	-0.046750845	0.064457214	0.089103536	0.024646322	8	0.10352736	0.816496581	0.816496581	0.816496581	0.816496581	0.816496581	0.816496581	26	155	0	P_255730	P_255730	P_255730	chr5	170896055	170896115	+	RANBP17	RAN binding protein 17 [Source:HGNC Symbol;Acc:HGNC:14428]	ENSG00000204764	protein_coding	exon	coding exon	1	4405	5:170896055:170896115	ENSG00000204764	RANBP17	6.600875301	0.010813754	-1.600040747	-0.840888422	-0.550613501	0.759152325	1.049427246	0.290274921	9.961963646	0.140789156	0.420135922	0.594029926	0.465344986	0.318773444	0.777558536	-5.756253082	-5.822490077	-6.884041221	-6.843279568	-5.742095031	-7.025542197	0.88871138	0.480006799

SRM	-0.310529779	0.091530709	-0.107853671	0.402060488	0.202676107	-0.19938438	8	0.066451173	0.816496581	0.816496581	0.816496581	0.816496581	0.816496581	0.816496581	26	156	0	P_100403	P_100403	P_100403	chr1	11054638	11054698	-	SRM	spermidine synthase [Source:HGNC Symbol;Acc:HGNC:11296]	ENSG00000116649	protein_coding	3'UTR;exon	multiple Ref	1	4406	1:11054638:11054698	ENSG00000116649	SRM	13.14317066	0.005752779	-5.014296918	1.477997226	-1.74157317	6.492294144	3.272723749	-3.219570396	9.961963646	0.000532276	0.170313998	0.112314939	7.09E-05	0.008433165	0.009225706	-0.206621905	-5.145473404	-5.602673785	1.780958224	-2.487981658	-3.202461914	15.59782734	0.000430004

TUBE1	-0.061225283	0.242692216	0.16792347	0.303917499	0.229148752	-0.074768747	8	0.101754501	0.816496581	0.816496581	0.816496581	0.816496581	0.816496581	0.816496581	26	158	0	A_23_P145053	A_23_P145053	A_23_P145053	chr6	112074742	112074802	-	TUBE1	"tubulin, epsilon 1 [Source:HGNC Symbol;Acc:HGNC:20775]"	ENSG00000074935	protein_coding	3'UTR;exon	multiple Ref	1	4408	6:112074742:112074802	ENSG00000074935	TUBE1	6.642822127	0.010521493	-0.731034274	2.897762484	2.005018282	3.628796758	2.736052556	-0.892744202	9.961963646	0.481598628	0.015953899	0.072885197	0.004650518	0.021042806	0.393048716	-6.714351225	-3.021278225	-5.205442443	-2.576244864	-3.360221732	-6.652635942	5.733779758	0.015220924

ETHE1	0.008722918	0.135581067	0.127985756	0.126858149	0.119262838	-0.007595311	8	0.143493478	0.816496581	0.816496581	0.816496581	0.816496581	0.816496581	0.816496581	26	159	0	A_23_P142294	A_23_P142294	A_23_P142294	chr19	43506777	43506837	-	ETHE1	ethylmalonic encephalopathy 1 [Source:HGNC Symbol;Acc:HGNC:23287]	ENSG00000105755	protein_coding	3'UTR	3'UTR	1	4409	19:43506777:43506837	ENSG00000105755	ETHE1	8.440764612	0.01874188	0.078037047	1.212936583	1.144987341	1.134899537	1.066950295	-0.067949242	9.961963646	0.939343769	0.25313178	0.27897741	0.282986089	0.311168311	0.947170514	-6.995038195	-5.460869746	-6.374839157	-6.487458275	-5.724429136	-7.068856776	0.869882989	0.488576118

CFAP206	-0.032300164	-0.037906519	0.019672614	-0.005606355	0.051972778	0.057579133	8	0.19892914	0.816496581	0.816496581	0.816496581	0.816496581	0.816496581	0.816496581	27	12	0	P_258154	P_258154	P_258154	chr6	87426585	87426645	+	C6orf165	chromosome 6 open reading frame 165 [Source:HGNC Symbol;Acc:HGNC:21405]	ENSG00000272514	protein_coding	3'UTR;exon	multiple Ref	1	4432	6:87426585:87426645	ENSG00000272514	CFAP206	3.778382388	0.033985802	-0.214586105	-0.251831917	0.130694989	-0.037245812	0.345281095	0.382526906	9.961963646	0.834418907	0.806290106	0.898618157	0.971024809	0.737062639	0.71010511	-6.973291089	-6.148764961	-7.037800443	-7.148854476	-6.233663199	-6.992025181	0.065580135	0.976909038

TMEM131	-0.016357596	0.039321943	0.018525784	0.055679539	0.034883381	-0.020796159	8	0.095658161	0.816496581	0.816496581	0.816496581	0.816496581	0.816496581	0.816496581	27	13	0	P_235820	P_235820	P_235820	chr2	97757098	97757158	-	TMEM131	transmembrane protein 131 [Source:HGNC Symbol;Acc:HGNC:30366]	ENSG00000075568	protein_coding	exon	coding exon	1	4433	2:97757098:97757158	ENSG00000075568	TMEM131	8.121474869	0.009555021	-0.20495064	0.492679819	0.23211671	0.697630459	0.43706735	-0.26056311	9.961963646	0.841737696	0.632916481	0.821146176	0.501365639	0.671384709	0.799736214	-6.975487228	-6.055723485	-7.017777053	-6.889794716	-6.196073974	-7.034414977	0.180704266	0.907057306

BMPR1B	-0.372611325	0.209795425	-0.160226934	0.58240675	0.212384391	-0.370022359	8	0.084310984	0.816496581	0.816496581	0.816496581	0.816496581	0.816496581	0.816496581	27	14	0	A_16_P16777145	A_16_P16777145	A_16_P16777145	chr4	94856800	94856860	+	BMPR1B	"bone morphogenetic protein receptor, type IB [Source:HGNC Symbol;Acc:HGNC:1077]"	ENSG00000138696	protein_coding	intron	intron sense	0	4434	4:94856800:94856860	ENSG00000138696	BMPR1B	4.390627722	0.00791507	-5.129491243	2.888113485	-2.205737187	8.017604728	2.923754056	-5.093850672	9.961963646	0.000449901	0.016219329	0.052033176	1.18E-05	0.015260571	0.000473828	-0.032301004	-3.036729272	-4.887196644	3.649752639	-3.055204653	-0.143497175	23.04929908	8.42E-05

CNP	-0.08976599	0.109277481	0.061470225	0.19904347	0.151236215	-0.047807256	8	0.106832634	0.816496581	0.816496581	0.816496581	0.816496581	0.816496581	0.816496581	27	15	0	P_121490	P_121490	P_121490	chr17	41971102	41971162	+	CNP	"2',3'-cyclic nucleotide 3' phosphodiesterase [Source:HGNC Symbol;Acc:HGNC:2158]"	ENSG00000173786	protein_coding	exon	non-coding exon	1	4435	17:41971102:41971162	ENSG00000173786	CNP	8.780111757	0.011372115	-1.030948159	1.255034538	0.705975787	2.285982698	1.736923946	-0.549058751	9.961963646	0.326945006	0.238108678	0.496381018	0.045416906	0.113160682	0.595056893	-6.447506867	-5.413663871	-6.781576944	-4.855570731	-4.90164661	-6.909146886	1.946742469	0.186336718

NECTIN3	0.017871163	0.243595708	0.280164576	0.225724544	0.262293413	0.036568868	8	0.104298824	0.816496581	0.816496581	0.816496581	0.816496581	0.816496581	0.816496581	27	16	0	A_23_P401547	A_23_P401547	A_23_P401547	chr3	111133892	111133952	+	PVRL3	poliovirus receptor-related 3 [Source:HGNC Symbol;Acc:HGNC:17664]	ENSG00000177707	protein_coding	3'UTR;exon	multiple Ref	1	4436	3:111133892:111133952	ENSG00000177707	NECTIN3	6.296492846	0.010942508	0.209237741	2.852048001	3.280200733	2.64281026	3.070962993	0.428152732	9.961963646	0.838479353	0.017251592	0.008327431	0.024688764	0.011872089	0.677648783	-6.974522448	-3.094470187	-3.076546948	-4.260837041	-2.815619613	-6.972149726	5.92270827	0.013795187

DIP2C	0.073573747	0.019851609	0.07705563	-0.053722138	0.003481884	0.057204021	8	0.095220585	0.816496581	0.816496581	0.816496581	0.816496581	0.816496581	0.816496581	27	17	0	P_105339	P_105339	P_105339	chr10	329463	329523	-	DIP2C	DIP2 disco-interacting protein 2 homolog C (Drosophila) [Source:HGNC Symbol;Acc:HGNC:29150]	ENSG00000151240	protein_coding	exon	coding exon	1	4437	10:329463:329523	ENSG00000151240	DIP2C	5.915640104	0.009487947	0.925086571	0.249606113	0.96886637	-0.675480458	0.043779799	0.719260257	9.961963646	0.376786077	0.807963404	0.355545787	0.514743415	0.965945033	0.488509622	-6.5504993	-6.149349495	-6.557389289	-6.905671192	-6.295665907	-6.795900423	0.472052709	0.70853594

TBC1D4	-0.124630631	0.112348357	-0.11653242	0.236978988	0.008098211	-0.228880777	8	0.034090706	0.816496581	0.816496581	0.816496581	0.816496581	0.816496581	0.816496581	27	18	0	P_219377	P_219377	P_219377	chr13	75286791	75286851	-	TBC1D4	"TBC1 domain family, member 4 [Source:HGNC Symbol;Acc:HGNC:19165]"	ENSG00000136111	protein_coding	exon	coding exon	1	4438	13:75286791:75286851	ENSG00000136111	TBC1D4	7.260942355	0.003139975	-2.724001459	2.455552762	-2.547002125	5.179554221	0.176999334	-5.002554887	9.961963646	0.021481865	0.034018616	0.029092159	0.000418446	0.863055767	0.000541535	-3.980544855	-3.72503326	-4.324807814	-0.077479582	-6.280048923	-0.282506684	11.97030154	0.001216016

SLFN11	0.179608725	0.06991259	0.294170292	-0.109696135	0.114561567	0.224257702	8	0.266696212	0.816496581	0.816496581	0.816496581	0.816496581	0.816496581	0.816496581	27	19	0	P_310398	P_310398	P_310398	chr17	35360305	35360365	-	SLFN11	schlafen family member 11 [Source:HGNC Symbol;Acc:HGNC:26633]	ENSG00000172716	protein_coding	exon	coding exon	1	4439	17:35360305:35360365	ENSG00000172716	SLFN11	4.282185142	0.059325439	0.903134806	0.351544688	1.479189999	-0.551590118	0.576055194	1.127645311	9.961963646	0.387771074	0.732501727	0.170001147	0.593385316	0.577359278	0.285896549	-6.570690545	-6.117355177	-5.967780751	-6.98573329	-6.123155817	-6.417886858	0.839152399	0.502903226

MAOA	-0.252477326	0.045709665	-0.373570383	0.298186991	-0.121093058	-0.419280049	8	0.064467565	0.816496581	0.816496581	0.816496581	0.816496581	0.816496581	0.816496581	27	20	0	A_23_P83857	A_23_P83857	A_23_P83857	chrX	43744429	43744489	+	MAOA	monoamine oxidase A [Source:HGNC Symbol;Acc:HGNC:6833]	ENSG00000189221	protein_coding	exon	coding exon	1	4440	X:43744429:43744489	ENSG00000189221	MAOA	10.98199186	0.005544232	-4.152860107	0.751853042	-6.144652941	4.904713148	-1.991792834	-6.896505982	9.961963646	0.001987197	0.469530197	0.000110861	0.000625705	0.074507168	4.29E-05	-1.568414879	-5.892574697	1.38716201	-0.497699517	-4.531527723	2.357262761	21.85892239	0.000105705

PLK4	-0.286980285	0.039236993	-0.236187992	0.326217278	0.050792294	-0.275424984	8	0.025085413	0.816496581	0.816496581	0.816496581	0.816496581	0.816496581	0.816496581	27	21	0	P_250861	P_250861	P_250861	chr4	127893544	127893604	+	PLK4	polo-like kinase 4 [Source:HGNC Symbol;Acc:HGNC:11397]	ENSG00000142731	protein_coding	exon	coding exon	1	4441	4:127893544:127893604	ENSG00000142731	PLK4	8.103463863	0.002712028	-6.749173133	0.922771597	-5.554645141	7.671944729	1.194527992	-6.477416738	9.961963646	5.14E-05	0.377933971	0.00024589	1.73E-05	0.2599369	7.22E-05	2.215952746	-5.752111752	0.558232718	3.251390604	-5.589048833	1.816496207	29.91658145	2.69E-05

PIK3C3	0.125869568	0.139337904	0.189787618	0.013468336	0.06391805	0.050449714	8	0.04228648	0.816496581	0.816496581	0.816496581	0.816496581	0.816496581	0.816496581	27	23	0	A_23_P164536	A_23_P164536	A_23_P164536	chr18	42081282	42081342	+	PIK3C3	"phosphatidylinositol 3-kinase, catalytic subunit type 3 [Source:HGNC Symbol;Acc:HGNC:8974]"	ENSG00000078142	protein_coding	3'UTR;exon	multiple Ref	1	4443	18:42081282:42081342	ENSG00000078142	PIK3C3	7.681814386	0.003642663	2.554211589	2.827518163	3.851270339	0.273306574	1.29705875	1.023752175	9.961963646	0.028735254	0.017991352	0.003228161	0.79019994	0.223845406	0.330169347	-4.266669387	-3.133728571	-2.109550232	-7.108917362	-5.472115115	-6.527174076	5.359377571	0.018603585

TMPRSS4	-0.083443921	0.094959289	-0.036905895	0.178403211	0.046538026	-0.131865185	8	0.135361945	0.816496581	0.816496581	0.816496581	0.816496581	0.816496581	0.816496581	27	24	0	P_308945	P_308945	P_308945	chr11	118114109	118114169	-	TMPRSS4	"transmembrane protease, serine 4 [Source:HGNC Symbol;Acc:HGNC:11878]"	ENSG00000137648	protein_coding	intron;promoter	multiple non-Ref	0	4444	11:118114109:118114169	ENSG00000137648	TMPRSS4	4.643173428	0.016920937	-0.78564823	0.894068693	-0.34747949	1.679716922	0.43816874	-1.241548183	9.961963646	0.450355558	0.392373187	0.735460619	0.124050699	0.670612608	0.242840461	-6.671640347	-5.777411773	-6.9815815	-5.792168935	-6.19557124	-6.288824564	1.015371106	0.426313236

MIS18A	-0.355096728	-0.055551473	-0.266227091	0.299545255	0.088869637	-0.210675619	8	0.040818231	0.816496581	0.816496581	0.816496581	0.816496581	0.816496581	0.816496581	27	25	0	P_241568	P_241568	P_241568	chr21	32269729	32269789	-	MIS18A	MIS18 kinetochore protein A [Source:HGNC Symbol;Acc:HGNC:1286]	ENSG00000159055	protein_coding	exon	coding exon	1	4445	21:32269729:32269789	ENSG00000159055	MIS18A	11.21634167	0.003544676	-7.304728075	-1.142754548	-5.476582444	6.161973527	1.828145631	-4.333827896	9.961963646	2.64E-05	0.279860809	0.000274209	0.000108376	0.097585077	0.001493845	2.903466076	-5.53692137	0.444795135	1.336251532	-4.771892221	-1.335956161	24.12533232	6.92E-05

TIMELESS	-0.337555898	-0.032414582	-0.158792722	0.305141316	0.178763176	-0.12637814	8	0.167354976	0.816496581	0.816496581	0.816496581	0.816496581	0.816496581	0.816496581	27	26	0	P_215734	P_215734	P_215734	chr12	56428330	56428390	-	TIMELESS	timeless circadian clock [Source:HGNC Symbol;Acc:HGNC:11813]	ENSG00000111602	protein_coding	exon	coding exon	1	4446	12:56428330:56428390	ENSG00000111602	TIMELESS	8.893185456	0.024698385	-2.630613574	-0.252610724	-1.237490715	2.378002851	1.393122859	-0.984879991	9.961963646	0.025210191	0.805704863	0.244278917	0.038833668	0.193886251	0.347998352	-4.138299918	-6.148559224	-6.269617882	-4.704256637	-5.356504334	-6.565878759	2.846833447	0.091687824

ANKMY1	-0.00976985	0.121834701	-0.092594516	0.131604551	-0.082824666	-0.214429217	8	0.111833286	0.816496581	0.816496581	0.816496581	0.816496581	0.816496581	0.816496581	27	27	0	P_239863	P_239863	P_239863	chr2	240529036	240529096	-	ANKMY1	ankyrin repeat and MYND domain containing 1 [Source:HGNC Symbol;Acc:HGNC:20987]	ENSG00000144504	protein_coding	exon	coding exon	1	4447	2:240529036:240529096	ENSG00000144504	ANKMY1	5.764000706	0.012250233	-0.108108875	1.348169369	-1.024610305	1.456278244	-0.91650143	-2.372779674	9.961963646	0.916055758	0.207457517	0.329783595	0.176098289	0.381055567	0.03918099	-6.991986293	-5.305217388	-6.502250734	-6.098822067	-5.868345682	-4.637814131	1.911646505	0.191908654

HOXC6	-0.066819502	-0.105162275	-0.033560402	-0.038342773	0.0332591	0.071601873	8	0.102187834	0.816496581	0.816496581	0.816496581	0.816496581	0.816496581	0.816496581	27	28	0	P_111756	P_111756	P_111756	chr12	54030578	54030638	+	HOXC6	homeobox C6 [Source:HGNC Symbol;Acc:HGNC:5128]	ENSG00000197757	protein_coding	3'UTR	3'UTR	1	4448	12:54030578:54030638	ENSG00000197757	HOXC6	8.370697863	0.010592463	-0.79515242	-1.251431616	-0.399368957	-0.456279196	0.395783463	0.852062659	9.961963646	0.445056591	0.239365374	0.698049012	0.657974961	0.700608396	0.414193755	-6.663929519	-5.417749254	-6.960713479	-7.036940979	-6.214037938	-6.688640348	0.574781676	0.64452922

SYVN1	-0.086388367	-0.204509356	-0.078057536	-0.118120989	0.008330831	0.12645182	8	0.126353083	0.816496581	0.816496581	0.816496581	0.816496581	0.816496581	0.816496581	27	30	0	P_109088	P_109088	P_109088	chr11	65128847	65128907	-	SYVN1	"synovial apoptosis inhibitor 1, synoviolin [Source:HGNC Symbol;Acc:HGNC:20738]"	ENSG00000162298	protein_coding	exon	non-coding exon	1	4450	11:65128847:65128907	ENSG00000162298	SYVN1	7.512536346	0.015027531	-0.86309197	-2.043219349	-0.779860004	-1.180127379	0.083231966	1.263359345	9.961963646	0.408385279	0.068385516	0.453602899	0.265361791	0.935315814	0.235225581	-6.606458338	-4.359006751	-6.724802481	-6.437030348	-6.293001904	-6.263066645	1.420593285	0.294100245

PFN2	0.199487534	0.043480185	0.192043266	-0.156007349	-0.007444268	0.14856308	8	0.04536558	0.816496581	0.816496581	0.816496581	0.816496581	0.816496581	0.816496581	27	32	0	P_136285	P_136285	P_136285	chr3	149968403	149968463	-	PFN2	profilin 2 [Source:HGNC Symbol;Acc:HGNC:8882]	ENSG00000070087	protein_coding	3'UTR;exon	multiple Ref	1	4452	3:149968403:149968463	ENSG00000070087	PFN2	9.786153757	0.003859399	3.93279719	0.857190158	3.786037156	-3.075607033	-0.146760035	2.928846998	9.961963646	0.00282812	0.411486407	0.003590735	0.011778637	0.886248941	0.015128333	-1.931217696	-5.808922982	-2.218793281	-3.521880663	-6.285241991	-3.700045687	8.09913132	0.005005401

C6orf62	0.197968447	0.022875617	0.175774251	-0.17509283	-0.022194196	0.152898634	8	0.05639929	0.816496581	0.816496581	0.816496581	0.816496581	0.816496581	0.816496581	27	33	0	A_24_P200652	A_24_P200652	A_24_P200652	chr6	24716199	24716259	-	C6orf62	chromosome 6 open reading frame 62 [Source:HGNC Symbol;Acc:HGNC:20998]	ENSG00000112308	protein_coding	exon	coding exon	1	4453	6:24716199:24716259	ENSG00000112308	C6orf62	10.35505245	0.004761104	3.513886843	0.406036074	3.11994581	-3.10785077	-0.393941033	2.713909737	9.961963646	0.005627696	0.693300482	0.010923349	0.011150169	0.701925086	0.021856597	-2.634683108	-6.095916199	-3.350627934	-3.46655917	-6.214799517	-4.06617244	6.57092665	0.009980384

ZNF215	-0.144896001	0.092211632	-0.212125983	0.237107633	-0.067229982	-0.304337615	8	0.073461453	0.816496581	0.816496581	0.816496581	0.816496581	0.816496581	0.816496581	27	34	0	P_210740	P_210740	P_210740	chr11	6943155	6943215	+	ZNF215	zinc finger protein 215 [Source:HGNC Symbol;Acc:HGNC:13007]	ENSG00000149054	protein_coding	exon	coding exon	1	4454	11:6943155:6943215	ENSG00000149054	ZNF215	6.73132662	0.006540436	-2.194313511	1.396458352	-3.212448288	3.590771863	-1.018134777	-4.60890664	9.961963646	0.053047705	0.192909923	0.009337565	0.004952713	0.332702859	0.000976539	-4.85884935	-5.246920326	-3.192372363	-2.640780363	-5.773062427	-0.895310241	8.709532187	0.003893219

BRDT	0.038151841	-0.10815053	0.035159678	-0.146302371	-0.002992164	0.143310207	8	0.10922501	0.816496581	0.816496581	0.816496581	0.816496581	0.816496581	0.816496581	27	35	0	P_202806	P_202806	P_202806	chr1	92004551	92004611	+	BRDT	"bromodomain, testis-specific [Source:HGNC Symbol;Acc:HGNC:1105]"	ENSG00000137948	protein_coding	exon	coding exon	1	4455	1:92004551:92004611	ENSG00000137948	BRDT	4.834170693	0.011787207	0.430383566	-1.220025269	0.396629543	-1.650408834	-0.033754023	1.616654811	9.961963646	0.676078789	0.250549817	0.700004095	0.129988356	0.973740035	0.137141242	-6.898217553	-5.453002679	-6.961885533	-5.833852139	-6.296079296	-5.804574614	1.194928016	0.360985839

GARRE1	-0.026023097	-0.099515569	-0.193345099	-0.073492471	-0.167322001	-0.09382953	8	0.073677748	0.816496581	0.816496581	0.816496581	0.816496581	0.816496581	0.816496581	27	36	0	P_232726	P_232726	P_232726	chr19	34319920	34319980	+	KIAA0355	KIAA0355 [Source:HGNC Symbol;Acc:HGNC:29016]	ENSG00000166398	protein_coding	exon	coding exon	1	4456	19:34319920:34319980	ENSG00000166398	GARRE1	6.691854975	0.006565994	-0.39332757	-1.504133662	-2.922325362	-1.110806092	-2.528997792	-1.4181917	9.961963646	0.702363726	0.163571908	0.015297877	0.292742628	0.030002767	0.186650121	-6.914592264	-5.112192524	-3.688573078	-6.513702967	-3.694338853	-6.07124327	3.433016312	0.060390275

STK4	-0.088185326	-0.127878273	-0.027743694	-0.039692947	0.060441632	0.100134579	8	0.075636316	0.816496581	0.816496581	0.816496581	0.816496581	0.816496581	0.816496581	27	38	0	P_131360	P_131360	P_131360	chr20	44972528	44972588	-	STK4	serine/threonine kinase 4 [Source:HGNC Symbol;Acc:HGNC:11408]	ENSG00000101109	protein_coding	exon;intron	multiple non-Ref	0	4458	20:44972528:44972588	ENSG00000101109	STK4	7.827037431	0.00680084	-1.30966634	-1.899158026	-0.4120298	-0.589491686	0.89763654	1.487128226	9.961963646	0.219706339	0.086855066	0.689043417	0.568658777	0.390557533	0.167931692	-6.135345252	-4.570887875	-6.955195175	-6.962835642	-5.885113788	-5.98109574	1.476099642	0.279868471

TOM1L1	-0.038515596	0.054551454	-0.016624319	0.09306705	0.021891277	-0.071175774	8	0.061218286	0.816496581	0.816496581	0.816496581	0.816496581	0.816496581	0.816496581	27	42	0	A_14_P114876	A_14_P114876	A_14_P114876	chr17	54919383	54919443	+	TOM1L1	target of myb1 (chicken)-like 1 [Source:HGNC Symbol;Acc:HGNC:11983]	ENSG00000141198	protein_coding	3'ext;intron	multiple non-Ref	0	4462	17:54919383:54919443	ENSG00000141198	TOM1L1	9.043565327	0.005216274	-0.65313363	0.925063944	-0.281909229	1.578197575	0.371224401	-1.206973174	9.961963646	0.528455152	0.376797285	0.783782721	0.145716135	0.718243398	0.255320426	-6.770462783	-5.750062306	-7.003893166	-5.934745722	-6.223906319	-6.328981463	0.907849839	0.471456422

NSD2	-0.24009382	-0.159722762	-0.224609972	0.080371058	0.015483848	-0.06488721	8	0.054727629	0.816496581	0.816496581	0.816496581	0.816496581	0.816496581	0.816496581	27	43	0	P_248004	P_248004	P_248004	chr4	1904257	1904317	+	WHSC1	Wolf-Hirschhorn syndrome candidate 1 [Source:HGNC Symbol;Acc:HGNC:12766]	ENSG00000109685	protein_coding	exon	coding exon	1	4463	4:1904257:1904317	ENSG00000109685	NSD2	7.83873522	0.004611923	-4.329976806	-2.880523348	-4.050733039	1.449453459	0.279243768	-1.170209691	9.961963646	0.001502874	0.016431258	0.00233897	0.177950512	0.785769251	0.269150052	-1.280473934	-3.048882696	-1.778111924	-6.10775939	-6.255377141	-6.370752177	7.833805864	0.005605638

CD226	-0.16976319	-0.163009039	-0.050588996	0.006754151	0.119174194	0.112420043	8	0.145638931	0.816496581	0.816496581	0.816496581	0.816496581	0.816496581	0.816496581	27	44	0	P_123757	P_123757	P_123757	chr18	69864246	69864306	-	CD226	CD226 molecule [Source:HGNC Symbol;Acc:HGNC:16961]	ENSG00000150637	protein_coding	3'UTR	3'UTR	1	4464	18:69864246:69864306	ENSG00000150637	CD226	3.523595092	0.019240031	-1.49894698	-1.439310292	-0.446682363	0.059636688	1.052264617	0.992627929	9.961963646	0.164891098	0.180734262	0.664657973	0.953624385	0.317532534	0.344389172	-5.893718861	-5.194064423	-6.939242708	-7.147668463	-5.73925024	-6.558261823	1.102351477	0.393185637

RRP1B	-0.311379542	0.063584754	-0.167244737	0.374964296	0.144134805	-0.230829491	8	0.051752461	0.816496581	0.816496581	0.816496581	0.816496581	0.816496581	0.816496581	27	46	0	P_241974	P_241974	P_241974	chr21	43686842	43686902	+	RRP1B	ribosomal RNA processing 1B [Source:HGNC Symbol;Acc:HGNC:23818]	ENSG00000160208	protein_coding	exon	coding exon	1	4466	21:43686842:43686902	ENSG00000160208	RRP1B	9.175383053	0.004357519	-5.777182283	1.179720138	-3.102976272	6.956902421	2.674206011	-4.28269641	9.961963646	0.000181058	0.265516506	0.011242936	3.99E-05	0.023395528	0.001618551	0.91193121	-5.497284247	-3.379665406	2.381441125	-3.460374926	-1.418926255	19.71456568	0.00016373

ERGIC3	0.084904687	0.042832152	0.057419273	-0.042072535	-0.027485414	0.014587121	8	0.072336277	0.816496581	0.816496581	0.816496581	0.816496581	0.816496581	0.816496581	27	47	0	P_131084	P_131084	P_131084	chr20	35557439	35557499	-	ERGIC3	ERGIC and golgi 3 [Source:HGNC Symbol;Acc:HGNC:15927]	ENSG00000125991	protein_coding	exon	exon antisense	0	4467	20:35557439:35557499	ENSG00000125991	ERGIC3	10.28715876	0.006408696	1.298950022	0.65528567	0.878452871	-0.643664352	-0.420497151	0.223167201	9.961963646	0.223220422	0.527125373	0.400389495	0.534329773	0.68304884	0.8279149	-6.148356668	-5.960661309	-6.641339102	-6.927656531	-6.203490524	-7.04423891	0.588212528	0.63652276

SCGB1A1	0.113091234	-0.121872331	0.159189398	-0.234963564	0.046098165	0.281061729	8	0.161999902	0.816496581	0.816496581	0.816496581	0.816496581	0.816496581	0.816496581	27	48	0	P_108820	P_108820	P_108820	chr11	62422348	62422408	+	SCGB1A1	"secretoglobin, family 1A, member 1 (uteroglobin) [Source:HGNC Symbol;Acc:HGNC:12523]"	ENSG00000149021	protein_coding	exon	coding exon	1	4468	11:62422348:62422408	ENSG00000149021	SCGB1A1	7.359344422	0.023282021	0.907745759	-0.978228707	1.27776041	-1.885974466	0.370014651	2.255989117	9.961963646	0.385445118	0.351118797	0.230305598	0.088760371	0.719116687	0.0477885	-6.566483375	-5.701352309	-6.221939606	-5.488016399	-6.224376567	-4.829125829	2.032817142	0.173449506

TCTE3	0.254107666	-0.111359101	0.096243124	-0.365466767	-0.157864542	0.207602225	8	0.227012254	0.816496581	0.816496581	0.816496581	0.816496581	0.816496581	0.816496581	27	49	0	A_23_P416732	A_23_P416732	A_23_P416732	chr6	169740188	169740248	-	TCTE3	t-complex-associated-testis-expressed 3 [Source:HGNC Symbol;Acc:HGNC:11695]	ENSG00000184786	protein_coding	exon	coding exon	1	4469	6:169740188:169740248	ENSG00000184786	TCTE3	4.993389906	0.043591748	1.490600467	-0.653234634	0.564564019	-2.143835101	-0.926036449	1.217798652	9.961963646	0.167033385	0.528392696	0.584857296	0.057763053	0.376315795	0.251358536	-5.904820281	-5.96201542	-6.875804292	-5.085572779	-5.859759144	-6.316498226	1.650657295	0.239975043

IL4R	0.210534199	0.110120506	0.310985026	-0.100413693	0.100450826	0.20086452	8	0.168559376	0.816496581	0.816496581	0.816496581	0.816496581	0.816496581	0.816496581	27	51	0	A_23_P129556	A_23_P129556	A_23_P129556	chr16	27364677	27364737	+	IL4R	interleukin 4 receptor [Source:HGNC Symbol;Acc:HGNC:6015]	ENSG00000077238	protein_coding	3'UTR	3'UTR	1	4471	16:27364677:27364737	ENSG00000077238	IL4R	6.320729158	0.025023281	1.630032111	0.852592886	2.407758833	-0.777439225	0.777726722	1.555165947	9.961963646	0.134265435	0.413913228	0.03691179	0.454965537	0.454803568	0.151075664	-5.714448783	-5.812771694	-4.556850646	-6.828777784	-5.984669477	-5.889487368	2.134838939	0.159497164

CWC15	0.248371548	-0.012172889	0.160663674	-0.260544437	-0.087707874	0.172836563	8	0.043003423	0.816496581	0.816496581	0.816496581	0.816496581	0.816496581	0.816496581	27	52	0	P_109840	P_109840	P_109840	chr11	94962643	94962703	-	CWC15	CWC15 spliceosome-associated protein [Source:HGNC Symbol;Acc:HGNC:26939]	ENSG00000150316	protein_coding	3'UTR	3'UTR	1	4472	11:94962643:94962703	ENSG00000150316	CWC15	9.525099333	0.003691768	5.006454343	-0.24537034	3.238516471	-5.251824682	-1.767937872	3.483886811	9.961963646	0.000538441	0.811150543	0.008934789	0.000377116	0.107626892	0.005916337	-0.218559313	-6.15044773	-3.147795531	0.031233719	-4.857903377	-2.751236292	13.07625246	0.000865169

DNAJA4	-0.129940994	-0.132960285	-0.091371271	-0.003019291	0.038569723	0.041589014	8	0.184689756	0.816496581	0.816496581	0.816496581	0.816496581	0.816496581	0.816496581	27	56	0	P_310102	P_310102	P_310102	chr15	78266356	78266416	+	DNAJA4;RP11-762H8.3	"DnaJ (Hsp40) homolog, subfamily A, member 4 [Source:HGNC Symbol;Acc:HGNC:14885];"	ENSG00000140403	protein_coding;antisense	3'ext;intron;promoter	multiple non-Ref	0	4476	15:78266356:78266416	ENSG00000140403	DNAJA4	4.19205467	0.02959912	-0.925022753	-0.946516454	-0.650452964	-0.021493701	0.274569789	0.29606349	9.961963646	0.376817688	0.366278052	0.530114337	0.983276247	0.78925659	0.773261379	-6.550558592	-5.730678048	-6.820874553	-7.149360428	-6.256743069	-7.023703984	0.389692282	0.763051571

VAT1L	-0.011173269	0.286052241	0.337506858	0.29722551	0.348680126	0.051454616	8	0.24945101	0.816496581	0.816496581	0.816496581	0.816496581	0.816496581	0.816496581	27	57	0	P_119603	P_119603	P_119603	chr16	77971870	77971930	+	VAT1L	vesicle amine transport 1-like [Source:HGNC Symbol;Acc:HGNC:29315]	ENSG00000171724	protein_coding	exon	coding exon	1	4477	16:77971870:77971930	ENSG00000171724	VAT1L	4.487805405	0.052177399	-0.059907978	1.533733027	1.809618453	1.593641005	1.869526431	0.275885426	9.961963646	0.953413697	0.156218212	0.100579427	0.142217166	0.091191944	0.788274472	-6.996402203	-5.074073602	-5.502675077	-5.913390512	-4.711983377	-7.029957012	1.956929734	0.18475472

MTFMT	-0.027116243	0.028462264	-0.03693857	0.055578507	-0.009822328	-0.065400834	8	0.062271426	0.816496581	0.816496581	0.816496581	0.816496581	0.816496581	0.816496581	27	59	0	P_224155	P_224155	P_224155	chr15	65003091	65003151	-	MTFMT	mitochondrial methionyl-tRNA formyltransferase [Source:HGNC Symbol;Acc:HGNC:29666]	ENSG00000103707	protein_coding	3'UTR;exon	multiple Ref	1	4479	15:65003091:65003151	ENSG00000103707	MTFMT	8.56766071	0.005320713	-0.455292216	0.477892429	-0.6202129	0.933184645	-0.164920685	-1.098105329	9.961963646	0.658660828	0.643039979	0.549041545	0.372790177	0.872304909	0.297989858	-6.886413529	-6.063107498	-6.841035213	-6.69317848	-6.282238931	-6.449805279	0.487367324	0.698694718

BRF1	0.045902141	-0.032013316	-0.08623849	-0.077915457	-0.132140631	-0.054225174	8	0.120208096	0.816496581	0.816496581	0.816496581	0.816496581	0.816496581	0.816496581	27	60	0	A_23_P117416	A_23_P117416	A_23_P117416	chr14	105209493	105209554	-	BRF1	"BRF1, RNA polymerase III transcription initiation factor 90 kDa subunit [Source:HGNC Symbol;Acc:HGNC:11551]"	ENSG00000185024	protein_coding	"3'ext;3'UTR;3'UTR,exon;exon"	multiple non-Ref	0	4480	14:105209493:105209554	ENSG00000185024	BRF1	7.427018687	0.013810811	0.478375564	-0.333631231	-0.898746438	-0.812006794	-1.377122001	-0.565115207	9.961963646	0.642707993	0.745574062	0.389993919	0.435761167	0.198628264	0.584496451	-6.874906658	-6.123747067	-6.623100618	-6.800534071	-5.376147617	-6.899671099	0.670512229	0.589361954

TPGS2	0.091656835	-0.035791898	0.06316943	-0.127448733	-0.028487406	0.098961327	8	0.04906266	0.816496581	0.816496581	0.816496581	0.816496581	0.816496581	0.816496581	27	62	0	A_23_P141715	A_23_P141715	A_23_P141715	chr18	36796126	36796186	-	TPGS2	tubulin polyglutamylase complex subunit 2 [Source:HGNC Symbol;Acc:HGNC:24561]	ENSG00000134779	protein_coding	3'UTR	3'UTR	1	4482	18:36796126:36796186	ENSG00000134779	TPGS2	10.17027502	0.004139752	1.744710221	-0.681307504	1.202445505	-2.426017725	-0.542264716	1.883753009	9.961963646	0.11174753	0.5112034	0.256992269	0.035779016	0.599555562	0.089085222	-5.550616541	-5.943145843	-6.31019913	-4.624657521	-6.14262256	-5.415143728	2.447034547	0.124287819

GABRR1	-0.165169793	-0.399990248	-0.123852575	-0.234820455	0.041317218	0.276137673	8	0.316009026	0.816496581	0.816496581	0.816496581	0.816496581	0.816496581	0.816496581	27	63	0	A_23_P256754	A_23_P256754	A_23_P256754	chr6	89178601	89178661	-	GABRR1	"gamma-aminobutyric acid (GABA) A receptor, rho 1 [Source:HGNC Symbol;Acc:HGNC:4090]"	ENSG00000146276	protein_coding	3'UTR	3'UTR	1	4483	6:89178601:89178661	ENSG00000146276	GABRR1	3.732721319	0.082401078	-0.704708982	-1.706587601	-0.528426053	-1.001878619	0.176282929	1.178161548	9.961963646	0.497135702	0.118822701	0.608773574	0.340116234	0.863603759	0.266109291	-6.733953332	-4.843031822	-6.896741637	-6.626773345	-6.280183105	-6.361799554	1.018531859	0.425057986

CRIP3	0.09174152	0.044368757	0.145929033	-0.047372763	0.054187513	0.101560276	8	0.269136565	0.816496581	0.816496581	0.816496581	0.816496581	0.816496581	0.816496581	27	64	0	A_23_P156562	A_23_P156562	A_23_P156562	chr6	43305625	43305685	-	CRIP3	cysteine-rich protein 3 [Source:HGNC Symbol;Acc:HGNC:17751]	ENSG00000146215	protein_coding	3'UTR	3'UTR	1	4484	6:43305625:43305685	ENSG00000146215	CRIP3	3.863362277	0.06037553	0.457278817	0.221152786	0.727372465	-0.236126032	0.270093648	0.506219679	9.961963646	0.65728065	0.829440512	0.48374139	0.818118784	0.792600905	0.623716217	-6.885444653	-6.156369944	-6.765655791	-7.119207767	-6.258029902	-6.933166492	0.19534127	0.897177727

GAS6	0.111961156	-0.029599474	0.188115011	-0.14156063	0.076153855	0.217714485	8	0.132444037	0.816496581	0.816496581	0.816496581	0.816496581	0.816496581	0.816496581	27	65	0	P_114235	P_114235	P_114235	chr13	113847295	113847355	-	GAS6	growth arrest-specific 6 [Source:HGNC Symbol;Acc:HGNC:4168]	ENSG00000183087	protein_coding	intron	intron sense	0	4485	13:113847295:113847355	ENSG00000183087	GAS6	7.894661037	0.016293403	1.074254308	-0.28400352	1.804941719	-1.358257828	0.730687411	2.088945239	9.961963646	0.30803944	0.782223019	0.101348528	0.204344276	0.481801343	0.063345746	-6.402778782	-6.139745186	-5.509610402	-6.224502996	-6.02030133	-5.097196631	1.872492593	0.198354582

MFN2	-0.174682726	0.05453955	-0.139934767	0.229222277	0.034747959	-0.194474318	8	0.059464159	0.816496581	0.816496581	0.816496581	0.816496581	0.816496581	0.816496581	27	66	0	P_200339	P_200339	P_200339	chr1	12012983	12013043	-	MFN2	mitofusin 2 [Source:HGNC Symbol;Acc:HGNC:16877]	ENSG00000116688	protein_coding	3'UTR	3'UTR antisense	0	4486	1:12012983:12013043	ENSG00000116688	MFN2	7.141229633	0.005046274	-3.011689461	0.940311571	-2.412602967	3.952001031	0.599086494	-3.352914538	9.961963646	0.013133419	0.369298662	0.036607875	0.002741675	0.562490474	0.007367626	-3.490844933	-5.736322642	-4.548830371	-2.032464158	-6.109258661	-2.974488568	7.165727501	0.007543814

CTCF	-0.079183915	-0.265661171	-0.197803026	-0.186477256	-0.118619111	0.067858145	8	0.052449902	0.816496581	0.816496581	0.816496581	0.816496581	0.816496581	0.816496581	27	69	0	P_226681	P_226681	P_226681	chr16	67620703	67620763	+	CTCF	CCCTC-binding factor (zinc finger protein) [Source:HGNC Symbol;Acc:HGNC:13723]	ENSG00000102974	protein_coding	exon	coding exon	1	4489	16:67620703:67620763	ENSG00000102974	CTCF	7.612565453	0.004415881	-1.45939862	-4.896266472	-3.645607375	-3.436867852	-2.186208755	1.250659097	9.961963646	0.175256975	0.000633599	0.004523092	0.006399936	0.053779011	0.23963553	-5.94597134	0.010062364	-2.455216826	-2.902922528	-4.235534758	-6.278104624	9.591573393	0.002764742

IRS4	0.077738919	0.334332905	0.290418061	0.256593987	0.212679142	-0.043914845	8	0.628161547	0.816496581	0.816496581	0.816496581	0.816496581	0.816496581	0.816496581	27	70	0	A_23_P85039	A_23_P85039	A_23_P85039	chrX	108732541	108732601	-	IRS4	insulin receptor substrate 4 [Source:HGNC Symbol;Acc:HGNC:6128]	ENSG00000133124	protein_coding	"3'UTR,exon"	multiple Ref	1	4490	X:108732541:108732601	ENSG00000133124	IRS4	3.496224603	0.319081504	0.168551767	0.724893052	0.629677877	0.556341284	0.46112611	-0.095215174	9.961963646	0.86952224	0.485195646	0.543076148	0.590254632	0.654611612	0.926031964	-6.982879611	-5.912428181	-6.834820177	-6.982941152	-6.184816038	-7.066428044	0.246932118	0.8616772

PLCD1	0.153305209	-0.038604696	0.114873242	-0.191909905	-0.038431967	0.153477938	8	0.085141406	0.816496581	0.816496581	0.816496581	0.816496581	0.816496581	0.816496581	27	71	0	P_243800	P_243800	P_243800	chr3	38008277	38008337	-	PLCD1	"phospholipase C, delta 1 [Source:HGNC Symbol;Acc:HGNC:9060]"	ENSG00000187091	protein_coding	exon	coding exon	1	4491	3:38008277:38008337	ENSG00000187091	PLCD1	7.955070282	0.008028074	2.095544398	-0.527691499	1.570213953	-2.623235897	-0.525330445	2.097905452	9.961963646	0.062648275	0.609264901	0.147554637	0.025530912	0.610845534	0.062400516	-5.016405689	-6.037390467	-5.845106021	-4.293936641	-6.151962162	-5.083007548	3.115647072	0.075406261

ATP7B	0.13611859	0.185684995	0.07764858	0.049566405	-0.05847001	-0.108036415	8	0.176170712	0.816496581	0.816496581	0.816496581	0.816496581	0.816496581	0.816496581	27	72	0	P_113862	P_113862	P_113862	chr13	51934260	51934320	-	ATP7B	"ATPase, Cu++ transporting, beta polypeptide [Source:HGNC Symbol;Acc:HGNC:870]"	ENSG00000123191	protein_coding	3'UTR	3'UTR	1	4492	13:51934260:51934320	ENSG00000123191	ATP7B	4.743209154	0.02713038	1.012127278	1.380684653	0.577365998	0.368557375	-0.43476128	-0.803318655	9.961963646	0.335428337	0.197564035	0.576507311	0.720169222	0.673002621	0.440536551	-6.466484073	-5.266113152	-6.868075611	-7.075834613	-6.197122649	-6.729863024	0.705702668	0.570199406

ZNF319	0.155479173	-0.10338173	0.236404519	-0.258860903	0.080925346	0.339786249	8	0.200041656	0.816496581	0.816496581	0.816496581	0.816496581	0.816496581	0.816496581	27	73	0	A_23_P26314	A_23_P26314	A_23_P26314	chr16	57994746	57994806	-	ZNF319	zinc finger protein 319 [Source:HGNC Symbol;Acc:HGNC:13644]	ENSG00000166188	protein_coding	3'UTR	3'UTR	1	4493	16:57994746:57994806	ENSG00000166188	ZNF319	7.55583553	0.034342247	1.027551257	-0.683242809	1.562381354	-1.710794066	0.534830098	2.245624164	9.961963646	0.328464118	0.510030932	0.149378225	0.118022561	0.604498726	0.048635636	-6.450952869	-5.94181842	-5.855838177	-5.747526731	-6.14675729	-4.845962352	2.036567545	0.172912023

MYL6B	0.276968171	0.034987607	0.214459779	-0.241980564	-0.062508392	0.179472172	8	0.060280643	0.816496581	0.816496581	0.816496581	0.816496581	0.816496581	0.816496581	27	76	0	P_111890	P_111890	P_111890	chr12	56157916	56157976	+	MYL6B	"myosin, light chain 6B, alkali, smooth muscle and non-muscle [Source:HGNC Symbol;Acc:HGNC:29823]"	ENSG00000196465	protein_coding	3'UTR;exon	multiple Ref	1	4496	12:56157916:56157976	ENSG00000196465	MYL6B	12.87231037	0.005124788	4.738463442	0.598579599	3.669049112	-4.139883844	-1.06941433	3.070469513	9.961963646	0.000802325	0.56281541	0.004351437	0.002028623	0.310110076	0.011882063	-0.631729642	-5.99665922	-2.415640007	-1.72102759	-5.721926514	-3.457735814	10.66388717	0.001877543

CYCSP51	-0.274517334	-0.510110698	-0.36069348	-0.235593364	-0.086176146	0.149417218	8	0.431198839	0.816496581	0.816496581	0.816496581	0.816496581	0.816496581	0.816496581	27	77	0	A_24_P392833	A_24_P392833	A_24_P392833	chr1	150903998	150904058	+	CYCSP51	"cytochrome c, somatic pseudogene 51 [Source:HGNC Symbol;Acc:HGNC:24383]"	ENSG00000229357	processed_pseudogene	exon	non-coding exon	1	4497	1:150903998:150904058	ENSG00000229357	CYCSP51	7.508464983	0.151520571	-0.86373318	-1.604997137	-1.134875244	-0.741263957	-0.271142064	0.470121892	9.961963646	0.408049324	0.13969197	0.282995797	0.475644308	0.79181719	0.648390784	-6.605896527	-4.980505782	-6.385965737	-6.857167726	-6.257730364	-6.951966693	0.908999584	0.470947849

UBE2D3	-0.009743976	-0.347100451	-0.172904089	-0.337356475	-0.163160113	0.174196362	8	0.113890328	0.816496581	0.816496581	0.816496581	0.816496581	0.816496581	0.816496581	27	79	0	P_312388	P_312388	P_312388	chr4	102809979	102810039	-	UBE2D3	ubiquitin-conjugating enzyme E2D 3 [Source:HGNC Symbol;Acc:HGNC:12476]	ENSG00000109332	protein_coding	exon	non-coding exon	1	4499	4:102809979:102810039	ENSG00000109332	UBE2D3	5.913038775	0.01262311	-0.106218132	-3.783708147	-1.884810607	-3.677490015	-1.778592475	1.89889754	9.961963646	0.917517764	0.003604441	0.088930428	0.0042913	0.105783581	0.08689234	-6.992207171	-1.619582065	-5.389969358	-2.493756289	-4.842785774	-5.392174821	6.362229421	0.011051939

ANKRD13A	0.157147323	0.049632506	0.036983572	-0.107514816	-0.120163751	-0.012648935	8	0.061875583	0.816496581	0.816496581	0.816496581	0.816496581	0.816496581	0.816496581	27	80	0	P_112743	P_112743	P_112743	chr12	110021346	110021406	+	ANKRD13A	ankyrin repeat domain 13A [Source:HGNC Symbol;Acc:HGNC:21268]	ENSG00000076513	protein_coding	exon	non-coding exon	1	4500	12:110021346:110021406	ENSG00000076513	ANKRD13A	7.50413284	0.005281249	2.64840442	0.836456817	0.623284274	-1.811947603	-2.025120146	-0.213172543	9.961963646	0.024453217	0.422508504	0.547101687	0.100198397	0.070483612	0.835491584	-4.108312811	-5.826139504	-6.839027975	-5.59923259	-4.481519665	-7.04660996	2.588649586	0.111379243

ATOH7	0.289289736	0.231610438	0.149182813	-0.057679297	-0.140106923	-0.082427626	8	0.183798006	0.816496581	0.816496581	0.816496581	0.816496581	0.816496581	0.816496581	27	81	0	A_23_P378514	A_23_P378514	A_23_P378514	chr10	68231224	68231284	-	ATOH7	atonal homolog 7 (Drosophila) [Source:HGNC Symbol;Acc:HGNC:13907]	ENSG00000179774	protein_coding	exon	coding exon	1	4501	10:68231224:68231284	ENSG00000179774	ATOH7	5.430960748	0.029335237	2.068634939	1.65618543	1.066767122	-0.412449509	-1.001867817	-0.589418308	9.961963646	0.065539072	0.128798251	0.311247089	0.688745741	0.3401212	0.568706094	-5.058861239	-4.911812162	-6.458901522	-7.057373208	-5.788862493	-6.884842377	1.613576741	0.24787356

SP100	-0.037914395	0.101414484	0.053040654	0.139328879	0.090955049	-0.04837383	8	0.098518609	0.816496581	0.816496581	0.816496581	0.816496581	0.816496581	0.816496581	27	82	0	A_23_P209712	A_23_P209712	A_23_P209712	chr2	230543407	230543467	+	SP100	SP100 nuclear antigen [Source:HGNC Symbol;Acc:HGNC:11206]	ENSG00000067066	protein_coding	3'UTR;exon	multiple Ref	1	4502	2:230543407:230543467	ENSG00000067066	SP100	5.965114047	0.010001064	-0.464329902	1.242002615	0.649578136	1.706332517	1.113908038	-0.592424479	9.961963646	0.652392888	0.242679785	0.530656469	0.118871379	0.291472031	0.566769381	-6.881973176	-5.428401039	-6.821470187	-5.753963234	-5.675962298	-6.882967653	1.113908841	0.388998725

MRE11	-0.262591135	-0.105577801	-0.276766635	0.157013334	-0.014175499	-0.171188834	8	0.052826913	0.816496581	0.816496581	0.816496581	0.816496581	0.816496581	0.816496581	27	83	0	P_109832	P_109832	P_109832	chr11	94429910	94429970	-	MRE11A	MRE11 meiotic recombination 11 homolog A (S. cerevisiae) [Source:HGNC Symbol;Acc:HGNC:7230]	ENSG00000020922	protein_coding	exon	coding exon	1	4503	11:94429910:94429970	ENSG00000020922	MRE11	8.655949088	0.004447754	-4.822311866	-1.938866227	-5.0826355	2.883445639	-0.260323634	-3.143769273	9.961963646	0.000707416	0.081344289	0.000481635	0.016349333	0.799915756	0.01049028	-0.501368388	-4.513124702	-0.141151907	-3.851177201	-6.260766263	-3.332224881	11.852065	0.001262862

SETD2	0.051756832	-0.056714851	0.10696678	-0.108471683	0.055209948	0.163681631	8	0.074726403	0.816496581	0.816496581	0.816496581	0.816496581	0.816496581	0.816496581	27	84	0	P_134651	P_134651	P_134651	chr3	47017318	47017378	-	SETD2	SET domain containing 2 [Source:HGNC Symbol;Acc:HGNC:18420]	ENSG00000181555	protein_coding	intron	intron sense	0	4504	3:47017318:47017378	ENSG00000181555	SETD2	8.400829855	0.006690969	0.774941312	-0.849176417	1.601585208	-1.624117729	0.826643896	2.450761625	9.961963646	0.456374386	0.415723055	0.140446444	0.135530189	0.427794353	0.034298279	-6.680228879	-5.815620367	-5.801801578	-5.870889294	-5.945545342	-4.508614579	2.202340146	0.150985545

SLC11A2	-0.058145274	0.122920466	0.03223802	0.181065739	0.090383294	-0.090682445	8	0.0755287	0.816496581	0.816496581	0.816496581	0.816496581	0.816496581	0.816496581	27	85	0	P_111550	P_111550	P_111550	chr12	50990838	50990898	-	SLC11A2	"solute carrier family 11 (proton-coupled divalent metal ion transporter), member 2 [Source:HGNC Symbol;Acc:HGNC:10908]"	ENSG00000110911	protein_coding	exon	coding exon	1	4505	12:50990838:50990898	ENSG00000110911	SLC11A2	8.702922248	0.006787776	-0.864363229	1.82728404	0.479236878	2.691647269	1.343600106	-1.348047163	9.961963646	0.407719403	0.097722485	0.642116347	0.022706577	0.208880595	0.207495471	-6.605344147	-4.674082465	-6.923131076	-4.178080771	-5.416805898	-6.160041636	2.530536027	0.116475802

SPIN3	0.261495897	-0.009445976	0.172062244	-0.270941874	-0.089433654	0.18150822	8	0.102103321	0.816496581	0.816496581	0.816496581	0.816496581	0.816496581	0.816496581	27	86	0	A_24_P136725	A_24_P136725	A_24_P136725	chrX	56976642	56976702	-	SPIN3	"spindlin family, member 3 [Source:HGNC Symbol;Acc:HGNC:27272]"	ENSG00000204271	protein_coding	3'UTR	3'UTR	1	4506	X:56976642:56976702	ENSG00000204271	SPIN3	6.972723326	0.010578598	3.113841119	-0.112480807	2.048882966	-3.226321926	-1.064958153	2.161363772	9.961963646	0.011037236	0.912676453	0.067741232	0.009120937	0.312025892	0.056081755	-3.316336119	-6.175422093	-5.136898585	-3.263321481	-5.72644915	-4.981872259	5.020227041	0.022470145

QSER1	0.221261019	-0.001029173	0.272980002	-0.222290192	0.051718983	0.274009175	8	0.114121837	0.816496581	0.816496581	0.816496581	0.816496581	0.816496581	0.816496581	27	87	0	P_108221	P_108221	P_108221	chr11	32913594	32913654	+	QSER1	glutamine and serine rich 1 [Source:HGNC Symbol;Acc:HGNC:26154]	ENSG00000060749	protein_coding	intron	intron sense	0	4507	11:32913594:32913654	ENSG00000060749	QSER1	8.863820614	0.0126655	2.407905067	-0.011200125	2.970744391	-2.419105191	0.562839324	2.981944516	9.961963646	0.03690258	0.991284904	0.014083633	0.036203793	0.585987171	0.013816973	-4.510260931	-6.182021295	-3.605847534	-4.63614231	-6.130900702	-3.609252326	4.94738427	0.023421788

RAPH1	0.01104432	-0.042058376	-0.0142648	-0.053102696	-0.02530912	0.027793576	8	0.071474824	0.816496581	0.816496581	0.816496581	0.816496581	0.816496581	0.816496581	27	88	0	P_238863	P_238863	P_238863	chr2	203461853	203461913	-	RAPH1	Ras association (RalGDS/AF-6) and pleckstrin homology domains 1 [Source:HGNC Symbol;Acc:HGNC:14436]	ENSG00000173166	protein_coding	exon	coding exon	1	4508	2:203461853:203461913	ENSG00000173166	RAPH1	6.959835655	0.006309209	0.170293145	-0.648501041	-0.219949953	-0.818794186	-0.390243098	0.428551088	9.961963646	0.868188401	0.531324394	0.83035183	0.432054618	0.704570931	0.677368313	-6.982558554	-5.965125755	-7.02076531	-6.794861885	-6.216317694	-6.971966683	0.250716828	0.859047833

PHLDB2	0.031830456	0.012424694	-0.005172749	-0.019405763	-0.037003206	-0.017597443	8	0.061332985	0.816496581	0.816496581	0.816496581	0.816496581	0.816496581	0.816496581	27	89	0	P_245467	P_245467	P_245467	chr3	111919153	111919213	+	PHLDB2	"pleckstrin homology-like domain, family B, member 2 [Source:HGNC Symbol;Acc:HGNC:29573]"	ENSG00000144824	protein_coding	exon	coding exon	1	4509	3:111919153:111919213	ENSG00000144824	PHLDB2	4.185646834	0.005227562	0.53918634	0.210465884	-0.087622866	-0.328720457	-0.626809207	-0.29808875	9.961963646	0.601599758	0.837546509	0.931912756	0.749172759	0.544880208	0.771759811	-6.842005002	-6.158789815	-7.042929322	-7.090840342	-6.091861509	-7.023052423	0.155416565	0.923797033

MYT1L-AS1	0.213493607	0.066384204	0.99780731	-0.147109403	0.784313704	0.931423107	8	0.422941164	0.816496581	0.816496581	0.816496581	0.816496581	0.816496581	0.816496581	27	90	0	P_127101	P_127101	P_127101	chr2	2327009	2327069	+	MYT1L-AS1	MYT1L antisense RNA 1 [Source:HGNC Symbol;Acc:HGNC:49274]	ENSG00000225619	antisense	exon	non-coding exon	1	4510	2:2327009:2327069	ENSG00000225619	MYT1L-AS1	3.615343872	0.145856458	0.684648517	0.212886218	3.199848961	-0.471762299	2.515200444	2.986962743	9.961963646	0.509180333	0.835708904	0.009538869	0.647259433	0.030719649	0.013699165	-6.748453812	-6.158252167	-3.213921771	-7.029250369	-3.716460803	-3.600667295	4.37063948	0.032930995

CREBL2	0.180832895	-0.055901695	0.031845391	-0.23673459	-0.148987504	0.087747086	8	0.044316839	0.816496581	0.816496581	0.816496581	0.816496581	0.816496581	0.816496581	27	92	0	A_24_P56194	A_24_P56194	A_24_P56194	chr12	12644549	12644609	+	CREBL2	cAMP responsive element binding protein-like 2 [Source:HGNC Symbol;Acc:HGNC:2350]	ENSG00000111269	protein_coding	3'UTR	3'UTR	1	4512	12:12644549:12644609	ENSG00000111269	CREBL2	10.14046727	0.003783869	3.60043548	-1.113018987	0.634050986	-4.713454467	-2.966384494	1.747069973	9.961963646	0.004874029	0.291835759	0.540332602	0.000833187	0.014188844	0.111322436	-2.488200342	-5.568110907	-6.831919299	-0.796371794	-2.985819664	-5.61833908	8.112051838	0.004978191

MX2	-0.00840039	0.020034464	-0.004778458	0.028434854	0.003621932	-0.024812922	8	0.135435102	0.816496581	0.816496581	0.816496581	0.816496581	0.816496581	0.816496581	27	93	0	A_23_P6263	A_23_P6263	A_23_P6263	chr21	41408573	41408633	+	MX2	MX dynamin-like GTPase 2 [Source:HGNC Symbol;Acc:HGNC:7533]	ENSG00000183486	protein_coding	3'UTR;exon	multiple Ref	1	4513	21:41408573:41408633	ENSG00000183486	MX2	4.054487735	0.016936846	-0.079054904	0.188541563	-0.0449694	0.267596467	0.034085504	-0.233510963	9.961963646	0.938554413	0.85423761	0.965020355	0.79446858	0.973482259	0.820093039	-6.994950995	-6.163382319	-7.046016346	-7.110594035	-6.296067337	-7.041671835	0.028519484	0.99309026

SPATC1L	-0.123196664	0.193910862	-0.095260685	0.317107526	0.027935978	-0.289171547	8	0.064217855	0.816496581	0.816496581	0.816496581	0.816496581	0.816496581	0.816496581	27	94	0	P_242037	P_242037	P_242037	chr21	46189210	46189270	+	SPATC1L;LSS	"spermatogenesis and centriole associated 1-like [Source:HGNC Symbol;Acc:HGNC:1298];lanosterol synthase (2,3-oxidosqualene-lanosterol cyclase) [Source:HGNC Symbol;Acc:HGNC:6708]"	ENSG00000160284	protein_coding	3'ext;3'UTR;exon;intron;promoter	multiple non-Ref	0	4514	21:46189210:46189270	ENSG00000160284	SPATC1L	9.194918072	0.005518427	-2.031126288	3.196981462	-1.570549693	5.22810775	0.460576595	-4.767531156	9.961963646	0.069780701	0.009585302	0.147476911	0.000390177	0.654992524	0.000767973	-5.117677274	-2.542606836	-5.844645272	-0.004360188	-6.185079623	-0.645788433	11.18123093	0.001573685

GOLGA6L2	-0.219841744	0.001316157	0.101052297	0.221157901	0.320894041	0.09973614	8	0.338519532	0.816496581	0.816496581	0.816496581	0.816496581	0.816496581	0.816496581	27	95	0	A_24_P220495	A_24_P220495	A_24_P220495	chr15	23439594	23439654	-	GOLGA6L2	golgin A6 family-like 2 [Source:HGNC Symbol;Acc:HGNC:26695]	ENSG00000174450	protein_coding	3'UTR;exon	multiple Ref	1	4515	15:23439594:23439654	ENSG00000174450	GOLGA6L2	4.421043989	0.094233098	-0.877109988	0.005251115	0.403171742	0.882361103	1.28028173	0.397920627	9.961963646	0.40108414	0.995913903	0.695338857	0.398372607	0.229452964	0.699082378	-6.594094551	-6.182072888	-6.959073518	-6.739758201	-5.49171899	-6.985561444	0.584653368	0.63863607

LRTOMT	-0.073437421	0.16311494	-0.011247443	0.23655236	0.062189978	-0.174362383	8	0.081231348	0.816496581	0.816496581	0.816496581	0.816496581	0.816496581	0.816496581	27	98	0	P_109512	P_109512	P_109512	chr11	72095536	72095596	+	LRTOMT	leucine rich transmembrane and O-methyltransferase domain containing [Source:HGNC Symbol;Acc:HGNC:25033]	ENSG00000184154	protein_coding	3'UTR	3'UTR	1	4518	11:72095536:72095596	ENSG00000184154	LRTOMT	7.864972214	0.007505665	-1.038169964	2.30592292	-0.159002824	3.344092883	0.879167139	-2.464925744	9.961963646	0.32373292	0.043903917	0.876843946	0.007477722	0.40002036	0.033477986	-6.440150273	-3.958808834	-7.033331519	-3.061536982	-5.901244444	-4.485037232	4.075351713	0.039564803

XPNPEP2	-0.145625153	0.011178826	0.077155891	0.156803979	0.222781044	0.065977065	8	0.354739311	0.816496581	0.816496581	0.816496581	0.816496581	0.816496581	0.816496581	27	100	0	A_14_P127021	A_14_P127021	A_14_P127021	chrX	129743892	129743952	+	XPNPEP2	"X-prolyl aminopeptidase (aminopeptidase P) 2, membrane-bound [Source:HGNC Symbol;Acc:HGNC:12823]"	ENSG00000122121	protein_coding	intron	intron sense	0	4520	X:129743892:129743952	ENSG00000122121	XPNPEP2	3.79259131	0.103263048	-0.555021175	0.042605863	0.294064264	0.597627038	0.84908544	0.251458402	9.961963646	0.591123608	0.966857586	0.774744609	0.563426315	0.415771323	0.806570833	-6.832828125	-6.181130562	-7.000101676	-6.957735707	-5.926903903	-7.036944969	0.256278841	0.855180546

FCRL2	0.089748953	0.00323561	0.197160659	-0.086513343	0.107411706	0.193925049	8	0.432860896	0.816496581	0.816496581	0.816496581	0.816496581	0.816496581	0.816496581	27	101	0	A_24_P319647	A_24_P319647	A_24_P319647	chr1	157766886	157766946	-	FCRL2	Fc receptor-like 2 [Source:HGNC Symbol;Acc:HGNC:14875]	ENSG00000132704	protein_coding	exon	coding exon	1	4521	1:157766886:157766946	ENSG00000132704	FCRL2	4.241024988	0.152673851	0.281314872	0.010141903	0.617993008	-0.271172969	0.336678136	0.607851105	9.961963646	0.784225547	0.992108299	0.550446053	0.791794092	0.743344457	0.55688869	-6.955347319	-6.182033201	-6.84248027	-7.109547939	-6.236746505	-6.873207689	0.169587698	0.914474128

SLFN13	0.221963368	-0.037102986	0.0767708	-0.259066354	-0.145192568	0.113873786	8	0.143295297	0.816496581	0.816496581	0.816496581	0.816496581	0.816496581	0.816496581	27	102	0	P_228425	P_228425	P_228425	chr17	35443788	35443848	-	SLFN13	schlafen family member 13 [Source:HGNC Symbol;Acc:HGNC:26481]	ENSG00000154760	protein_coding	3'UTR;exon	multiple Ref	1	4522	17:35443788:35443848	ENSG00000154760	SLFN13	3.381929014	0.018696238	1.988153083	-0.332335993	0.687645458	-2.320489076	-1.300507624	1.019981452	9.961963646	0.074959484	0.746522614	0.507369743	0.042829808	0.222706785	0.33186839	-5.18451865	-6.124196583	-6.79487436	-4.799032178	-5.46806295	-6.530981838	2.10873466	0.162938326

CSNK1A1P1	-0.255304665	-0.550572721	-0.085697031	-0.295268056	0.169607634	0.46487569	8	0.270940445	0.816496581	0.816496581	0.816496581	0.816496581	0.816496581	0.816496581	27	105	0	P_222719	P_222719	P_222719	chr15	36799100	36799160	-	CSNK1A1P1	"casein kinase 1, alpha 1 pseudogene 1 [Source:HGNC Symbol;Acc:HGNC:30446]"	ENSG00000223518	transcribed_processed_pseudogene	exon	non-coding exon	1	4525	15:36799100:36799160	ENSG00000223518	CSNK1A1P1	7.139784692	0.061157893	-1.264381474	-2.726679313	-0.424409553	-1.462297839	0.83997192	2.302269759	9.961963646	0.234873566	0.021383516	0.68028676	0.174478395	0.420625853	0.044177388	-6.189809385	-3.294916944	-6.949638569	-6.090916662	-5.934526181	-4.753655564	2.892979649	0.088617307

UROC1	-0.059834256	-0.158411669	-0.028283542	-0.098577413	0.031550714	0.130128127	8	0.176991335	0.816496581	0.816496581	0.816496581	0.816496581	0.816496581	0.816496581	27	106	0	P_135876	P_135876	P_135876	chr3	126501800	126501860	-	UROC1	urocanate hydratase 1 [Source:HGNC Symbol;Acc:HGNC:26444]	ENSG00000159650	protein_coding	exon	coding exon	1	4526	3:126501800:126501860	ENSG00000159650	UROC1	6.303164958	0.027363116	-0.443009202	-1.172870395	-0.20940963	-0.729861192	0.233599572	0.963460765	9.961963646	0.667224101	0.268129553	0.838348777	0.482284423	0.820026121	0.358120302	-6.892314105	-5.504700286	-7.023225307	-6.865865904	-6.267742702	-6.586679936	0.521876213	0.676894508

AGPAT3	-0.132034804	-0.041047226	-0.02216621	0.090987578	0.109868594	0.018881016	8	0.065225989	0.816496581	0.816496581	0.816496581	0.816496581	0.816496581	0.816496581	27	109	0	A_23_P218743	A_23_P218743	A_23_P218743	chr21	43982364	43982424	+	AGPAT3	1-acylglycerol-3-phosphate O-acyltransferase 3 [Source:HGNC Symbol;Acc:HGNC:326]	ENSG00000160216	protein_coding	"3'UTR,exon;exon"	multiple Ref	1	4529	21:43982364:43982424	ENSG00000160216	AGPAT3	7.917587094	0.005623223	-2.156459985	-0.670404299	-0.362029881	1.486055686	1.794430104	0.308374417	9.961963646	0.056547321	0.517839231	0.724891289	0.168210016	0.103096954	0.764149194	-4.919540834	-5.950560419	-6.976014541	-6.059512192	-4.820230933	-7.019676044	1.792377151	0.21234248

SUCLG1	-0.036401591	0.100910845	0.070203584	0.137312436	0.106605175	-0.030707261	8	0.042461352	0.816496581	0.816496581	0.816496581	0.816496581	0.816496581	0.816496581	27	110	0	P_235610	P_235610	P_235610	chr2	84449648	84449708	-	SUCLG1	"succinate-CoA ligase, alpha subunit [Source:HGNC Symbol;Acc:HGNC:11449]"	ENSG00000163541	protein_coding	exon	coding exon	1	4530	2:84449648:84449708	ENSG00000163541	SUCLG1	8.455712075	0.003654564	-0.737476507	2.044399047	1.422286572	2.781875554	2.159763079	-0.622112475	9.961963646	0.477843386	0.068250838	0.18549028	0.019454002	0.056233319	0.547841324	-6.709455927	-4.357246547	-6.042098939	-4.024617409	-4.276363698	-6.863978015	3.256128373	0.068269354

NDC80	-0.439057079	-0.095183115	-0.293901974	0.343873964	0.145155105	-0.198718859	8	0.084059851	0.816496581	0.816496581	0.816496581	0.816496581	0.816496581	0.816496581	27	111	0	P_230410	P_230410	P_230410	chr18	2616451	2616511	+	NDC80	NDC80 kinetochore complex component [Source:HGNC Symbol;Acc:HGNC:16909]	ENSG00000080986	protein_coding	exon	coding exon	1	4531	18:2616451:2616511	ENSG00000080986	NDC80	10.33469934	0.007881114	-6.057212363	-1.313142112	-4.054658851	4.744070251	2.002553512	-2.741516739	9.961963646	0.000124381	0.218576462	0.002324303	0.000795574	0.073184929	0.020846705	1.30131091	-5.346634243	-1.771630481	-0.748223096	-4.515417297	-4.019320211	14.81445989	0.000528855

FMNL1	0.16134523	-0.009185278	0.147246647	-0.170530508	-0.014098583	0.156431925	8	0.19753216	0.816496581	0.816496581	0.816496581	0.816496581	0.816496581	0.816496581	27	112	0	P_121781	P_121781	P_121781	chr17	45238978	45239038	+	FMNL1	formin-like 1 [Source:HGNC Symbol;Acc:HGNC:1212]	ENSG00000184922	protein_coding	exon	coding exon	1	4532	17:45238978:45239038	ENSG00000184922	FMNL1	5.724282373	0.033541032	1.078980385	-0.061425645	0.984697498	-1.14040603	-0.094282887	1.046123143	9.961963646	0.306027751	0.952235124	0.348083696	0.280792364	0.926753843	0.320223098	-6.397809138	-6.18009874	-6.541986905	-6.481399182	-6.291959355	-6.50435081	0.756898292	0.543387512

NOXRED1	-0.108030635	-0.206136601	-0.241283409	-0.098105966	-0.133252774	-0.035146808	8	0.157449128	0.816496581	0.816496581	0.816496581	0.816496581	0.816496581	0.816496581	27	113	0	P_221670	P_221670	P_221670	chr14	77406801	77406861	-	NOXRED1	NADP-dependent oxidoreductase domain containing 1 [Source:HGNC Symbol;Acc:HGNC:20487]	ENSG00000165555	protein_coding	exon	coding exon	1	4533	14:77406801:77406861	ENSG00000165555	NOXRED1	4.169260845	0.022114589	-0.889719531	-1.6977014	-1.987163754	-0.807981869	-1.097444223	-0.289462354	9.961963646	0.394594439	0.120529159	0.075082875	0.437969141	0.298264967	0.778162381	-6.582827599	-4.85523774	-5.233165153	-6.803878162	-5.693139984	-7.025797379	1.593936276	0.252177724

BLM	-0.459291312	0.037631872	-0.272063695	0.496923184	0.187227617	-0.309695567	8	0.041707621	0.816496581	0.816496581	0.816496581	0.816496581	0.816496581	0.816496581	27	116	0	P_225015	P_225015	P_225015	chr15	90769462	90769522	+	BLM	"Bloom syndrome, RecQ helicase-like [Source:HGNC Symbol;Acc:HGNC:1058]"	ENSG00000197299	protein_coding	3'UTR;exon	multiple Ref	1	4536	15:90769462:90769522	ENSG00000197299	BLM	7.693789521	0.003603618	-9.370537252	0.767771677	-5.550688467	10.13830893	3.819848785	-6.318460144	9.961963646	2.96E-06	0.460433861	0.000247247	1.44E-06	0.003397775	8.85E-05	5.137854811	-5.880550002	0.552504315	5.817833985	-1.613243922	1.605136366	46.08434591	3.79E-06

SV2B	-0.053806919	0.043226767	0.059222969	0.097033686	0.113029888	0.015996202	8	0.1243468	0.816496581	0.816496581	0.816496581	0.816496581	0.816496581	0.816496581	27	117	0	P_225049	P_225049	P_225049	chr15	91281778	91281838	+	SV2B	synaptic vesicle glycoprotein 2B [Source:HGNC Symbol;Acc:HGNC:16874]	ENSG00000185518	protein_coding	exon	coding exon	1	4537	15:91281778:91281838	ENSG00000185518	SV2B	3.757989599	0.014623615	-0.544949659	0.43779522	0.599802732	0.982744879	1.144752391	0.162007511	9.961963646	0.597775598	0.670874409	0.562031521	0.348997811	0.279070264	0.874538735	-6.838693885	-6.082047077	-6.854140339	-6.645660245	-5.643252883	-7.057058996	0.525148345	0.674855132

KLHL36	-0.028552931	0.03025495	-0.054100474	0.058807881	-0.025547543	-0.084355424	8	0.073443238	0.816496581	0.816496581	0.816496581	0.816496581	0.816496581	0.816496581	27	119	0	P_119714	P_119714	P_119714	chr16	84667166	84667226	-	KLHL36	kelch-like family member 36 [Source:HGNC Symbol;Acc:HGNC:17844]	ENSG00000135686	protein_coding	3'ext;exon	multiple non-Ref	0	4539	16:84667166:84667226	ENSG00000135686	KLHL36	8.677634205	0.006538287	-0.432478303	0.458258021	-0.819435367	0.890736325	-0.386957063	-1.277693388	9.961963646	0.674606056	0.656600851	0.431705567	0.394074344	0.706925541	0.230328298	-6.89724947	-6.072579984	-6.692339077	-6.732234653	-6.217655124	-6.245962684	0.60735993	0.625258044

CSGALNACT2	-0.134869366	0.1630473	-0.128849226	0.297916666	0.00602014	-0.291896525	8	0.133341505	0.816496581	0.816496581	0.816496581	0.816496581	0.816496581	0.816496581	27	121	0	P_105843	P_105843	P_105843	chr10	43162252	43162312	+	CSGALNACT2	chondroitin sulfate N-acetylgalactosaminyltransferase 2 [Source:HGNC Symbol;Acc:HGNC:24292]	ENSG00000169826	protein_coding	intron	intron sense	0	4541	10:43162252:43162312	ENSG00000169826	CSGALNACT2	7.29943452	0.016484959	-1.286515463	1.555304057	-1.229089496	2.84181952	0.057425967	-2.784393553	9.961963646	0.227356043	0.151043014	0.247279291	0.017556253	0.955341403	0.019370276	-6.163359358	-5.046014144	-6.27942444	-3.922330529	-6.294931532	-3.946434197	3.569472854	0.055043041

TMTC4	0.09504853	0.105555256	0.049990315	0.010506726	-0.045058215	-0.055564941	8	0.078094957	0.816496581	0.816496581	0.816496581	0.816496581	0.816496581	0.816496581	27	122	0	P_219782	P_219782	P_219782	chr13	100626084	100626144	-	TMTC4	transmembrane and tetratricopeptide repeat containing 4 [Source:HGNC Symbol;Acc:HGNC:25904]	ENSG00000125247	protein_coding	exon	coding exon	1	4542	13:100626084:100626144	ENSG00000125247	TMTC4	6.676715047	0.007104371	1.381109862	1.533778637	0.726388058	0.152668776	-0.654721804	-0.80739058	9.961963646	0.19743734	0.156207107	0.484318449	0.881707394	0.527473604	0.438294133	-6.046711494	-5.074014518	-6.76639767	-7.13688181	-6.073613939	-6.72650056	0.981904819	0.439850379

SCRN3	0.030884024	0.06579679	0.038019671	0.034912766	0.007135646	-0.02777712	8	0.036820127	0.816496581	0.816496581	0.816496581	0.816496581	0.816496581	0.816496581	27	123	0	P_129277	P_129277	P_129277	chr2	174428106	174428166	+	SCRN3	secernin 3 [Source:HGNC Symbol;Acc:HGNC:30382]	ENSG00000144306	protein_coding	3'UTR;exon	multiple Ref	1	4543	2:174428106:174428166	ENSG00000144306	SCRN3	8.917200051	0.003295402	0.658908762	1.403770485	0.811147342	0.744861724	0.15223858	-0.592623144	9.961963646	0.524891076	0.190784192	0.436232017	0.473561319	0.882037899	0.566641521	-6.766500588	-5.237974635	-6.699254308	-6.854398254	-6.284372735	-6.882843448	0.665315017	0.592242947

CPNE4	0.016121896	0.030720477	0.04412591	0.014598581	0.028004014	0.013405434	8	0.08251299	0.816496581	0.816496581	0.816496581	0.816496581	0.816496581	0.816496581	27	124	0	P_312004	P_312004	P_312004	chr3	132036189	132036249	-	CPNE4	copine IV [Source:HGNC Symbol;Acc:HGNC:2317]	ENSG00000196353	protein_coding	intron;promoter	multiple non-Ref	0	4544	3:132036189:132036249	ENSG00000196353	CPNE4	3.884234676	0.007674195	0.225395537	0.429494056	0.616911526	0.204098518	0.391515989	0.18741747	9.961963646	0.82622815	0.676704605	0.551131039	0.842385713	0.703659718	0.855095502	-6.970708461	-6.085769193	-6.843182532	-7.126880462	-6.215796677	-7.052223436	0.140985734	0.933108125

EPB41L4A	0.932153912	0.078638999	1.125739874	-0.853514913	0.193585962	1.047100875	8	0.120391245	0.816496581	0.816496581	0.816496581	0.816496581	0.816496581	0.816496581	27	125	0	P_254321	P_254321	P_254321	chr5	112418950	112419010	-	EPB41L4A	erythrocyte membrane protein band 4.1 like 4A [Source:HGNC Symbol;Acc:HGNC:13278]	ENSG00000129595	protein_coding	exon	coding exon	1	4545	5:112418950:112419010	ENSG00000129595	EPB41L4A	5.309626797	0.013846198	9.702150076	0.818499345	11.71705344	-8.883650731	2.014903368	10.8985541	9.961963646	2.16E-06	0.432215189	3.79E-07	4.77E-06	0.071694714	7.42E-07	5.454950667	-5.840756702	7.181757309	4.589390614	-4.496884979	6.511265242	72.30809434	4.59E-07

CTR9	-0.021120956	-0.072821909	0.027275076	-0.051700953	0.048396033	0.100096985	8	0.060274743	0.816496581	0.816496581	0.816496581	0.816496581	0.816496581	0.816496581	27	126	0	P_210923	P_210923	P_210923	chr11	10770230	10770290	+	CTR9	"CTR9, Paf1/RNA polymerase II complex component [Source:HGNC Symbol;Acc:HGNC:16850]"	ENSG00000198730	protein_coding	exon	coding exon	1	4546	11:10770230:10770290	ENSG00000198730	CTR9	8.806363182	0.005124217	-0.361364474	-1.245930843	0.466657075	-0.884566369	0.82802155	1.712587919	9.961963646	0.725373337	0.241294456	0.650783475	0.397237682	0.427049586	0.117682827	-6.927568274	-5.423970407	-6.929485543	-6.737783068	-5.944413416	-5.668349961	1.050288584	0.412664837

BMPER	0.088235918	-0.008362726	0.203233221	-0.096598644	0.114997303	0.211595947	8	0.154931816	0.816496581	0.816496581	0.816496581	0.816496581	0.816496581	0.816496581	27	128	0	P_260942	P_260942	P_260942	chr7	34085895	34085955	+	BMPER	BMP binding endothelial regulator [Source:HGNC Symbol;Acc:HGNC:24154]	ENSG00000164619	protein_coding	exon	coding exon	1	4548	7:34085895:34085955	ENSG00000164619	BMPER	3.661310373	0.021483099	0.737297191	-0.069878735	1.698211865	-0.807175927	0.960914674	1.7680906	9.961963646	0.47794766	0.94567301	0.120430536	0.438412154	0.35933761	0.107600263	-6.709592705	-6.179513882	-5.665339518	-6.804546027	-5.827721209	-5.587594312	1.35557468	0.311823383

SRD5A2	0.013459678	0.187367208	0.129328022	0.17390753	0.115868344	-0.058039186	8	0.215701626	0.816496581	0.816496581	0.816496581	0.816496581	0.816496581	0.816496581	27	129	0	P_234254	P_234254	P_234254	chr2	31533602	31533662	-	SRD5A2	"steroid-5-alpha-reductase, alpha polypeptide 2 (3-oxo-5 alpha-steroid delta 4-dehydrogenase alpha 2) [Source:HGNC Symbol;Acc:HGNC:11285]"	ENSG00000277893	protein_coding	exon	coding exon	1	4549	2:31533602:31533662	ENSG00000277893	SRD5A2	3.575986391	0.039570556	0.082869407	1.1535944	0.796255033	1.070724993	0.713385626	-0.357339367	9.961963646	0.935596869	0.275592552	0.44444451	0.309548303	0.491980871	0.728291966	-6.994614166	-5.525396398	-6.711525072	-6.556386286	-6.032907629	-7.002066681	0.625788656	0.614583166

WWTR1	-0.683224869	0.225379114	-0.646065577	0.908603983	0.037159293	-0.871444691	8	0.097652459	0.816496581	0.816496581	0.816496581	0.816496581	0.816496581	0.816496581	27	131	0	P_246721	P_246721	P_246721	chr3	149572933	149572993	-	WWTR1	WW domain containing transcription regulator 1 [Source:HGNC Symbol;Acc:HGNC:24042]	ENSG00000018408	protein_coding	exon	coding exon	1	4551	3:149572933:149572993	ENSG00000018408	WWTR1	4.272539811	0.009864614	-8.424987126	2.779196456	-7.966768205	11.20418358	0.458218921	-10.74596466	9.961963646	7.65E-06	0.019543484	1.25E-05	5.74E-07	0.656627989	8.46E-07	4.173732282	-3.211016586	3.648496773	6.754461675	-6.186207109	6.380674654	63.89886457	8.24E-07

SCFD1	0.051953705	-0.080902854	0.007632622	-0.132856559	-0.044321082	0.088535477	8	0.049759603	0.816496581	0.816496581	0.816496581	0.816496581	0.816496581	0.816496581	27	132	0	P_114621	P_114621	P_114621	chr14	30710017	30710077	-	SCFD1	sec1 family domain containing 1 [Source:HGNC Symbol;Acc:HGNC:20726]	ENSG00000092108	protein_coding	intron	intron antisense	0	4552	14:30710017:30710077	ENSG00000092108	SCFD1	10.13374108	0.004195061	0.982410477	-1.529819907	0.144327884	-2.512230384	-0.838082594	1.674147791	9.961963646	0.349154537	0.157173506	0.888119595	0.03087617	0.421637047	0.125159844	-6.495867518	-5.079138708	-7.035756797	-4.480765646	-5.936097479	-5.723461876	2.190460766	0.152444213

AKR1C3	0.07453888	-0.032168523	-0.001000604	-0.106707403	-0.075539484	0.031167919	8	0.156098896	0.816496581	0.816496581	0.816496581	0.816496581	0.816496581	0.816496581	27	135	0	P_207243	P_207243	P_207243	chr10	5102150	5102210	+	AKR1C3	"aldo-keto reductase family 1, member C3 [Source:HGNC Symbol;Acc:HGNC:386]"	ENSG00000196139	protein_coding	exon	coding exon	1	4555	10:5102150:5102210	ENSG00000196139	AKR1C3	4.113654339	0.021774605	0.618661865	-0.266994062	-0.008304864	-0.885655927	-0.626966729	0.258689198	9.961963646	0.550022657	0.794919331	0.993537712	0.396677781	0.544781056	0.801141473	-6.793445031	-6.144646971	-7.047082937	-6.73680566	-6.091760582	-7.03494295	0.283081851	0.836516675

SLC4A8	0.281367317	-0.138965261	0.188934087	-0.420332578	-0.09243323	0.327899348	8	0.092922838	0.816496581	0.816496581	0.816496581	0.816496581	0.816496581	0.816496581	27	137	0	P_111585	P_111585	P_111585	chr12	51505870	51505930	+	SLC4A8	"solute carrier family 4, sodium bicarbonate cotransporter, member 8 [Source:HGNC Symbol;Acc:HGNC:11034]"	ENSG00000050438	protein_coding	exon	coding exon	1	4557	12:51505870:51505930	ENSG00000050438	SLC4A8	4.03711795	0.009140782	3.6043554	-1.78016479	2.420272557	-5.38452019	-1.184082843	4.200437347	9.961963646	0.004842483	0.105514021	0.036131712	0.00031217	0.263862806	0.001842769	-2.481578004	-4.740718458	-4.536123734	0.228891354	-5.600566095	-1.553073709	11.67614459	0.001336636

PGRMC2	0.273664597	0.063867515	0.26764199	-0.209797082	-0.006022607	0.203774475	8	0.092212972	0.816496581	0.816496581	0.816496581	0.816496581	0.816496581	0.816496581	27	139	0	A_23_P155868	A_23_P155868	A_23_P155868	chr4	128271249	128271309	-	PGRMC2	progesterone receptor membrane component 2 [Source:HGNC Symbol;Acc:HGNC:16089]	ENSG00000164040	protein_coding	3'UTR	3'UTR	1	4559	4:128271249:128271309	ENSG00000164040	PGRMC2	9.12321951	0.009035243	3.526097574	0.822916417	3.448497843	-2.703181157	-0.077599731	2.625581426	9.961963646	0.005514428	0.429813829	0.006276635	0.022262161	0.939682931	0.025428509	-2.613986978	-5.837186735	-2.789496866	-4.15850225	-6.293483385	-4.215602173	6.534930136	0.010156019

ADD1	0.001435375	-0.189313085	-0.087908235	-0.190748459	-0.089343609	0.10140485	8	0.040919392	0.816496581	0.816496581	0.816496581	0.816496581	0.816496581	0.816496581	27	140	0	P_248047	P_248047	P_248047	chr4	2894033	2894093	+	ADD1	adducin 1 (alpha) [Source:HGNC Symbol;Acc:HGNC:243]	ENSG00000087274	protein_coding	3'UTR;exon	multiple Ref	1	4560	4:2894033:2894093	ENSG00000087274	ADD1	7.851083955	0.003551316	0.029499611	-3.89073466	-1.806677105	-3.92023427	-1.836176716	2.084057554	9.961963646	0.977048752	0.003027593	0.10106251	0.002886213	0.096312813	0.063867094	-6.997885463	-1.455027244	-5.507037975	-2.085486834	-4.760314431	-5.10492676	6.91430769	0.00847531

LCA5	0.248726301	0.022687512	0.136521293	-0.226038788	-0.112205008	0.113833781	8	0.114459415	0.816496581	0.816496581	0.816496581	0.816496581	0.816496581	0.816496581	27	141	0	P_257950	P_257950	P_257950	chr6	79489083	79489143	-	LCA5	Leber congenital amaurosis 5 [Source:HGNC Symbol;Acc:HGNC:31923]	ENSG00000135338	protein_coding	exon	coding exon	1	4561	6:79489083:79489143	ENSG00000135338	LCA5	5.131992941	0.012727467	2.700202557	0.246298355	1.482091533	-2.453904202	-1.218111024	1.235793178	9.961963646	0.02237609	0.810451961	0.169242185	0.034114589	0.251244955	0.244882776	-4.020824518	-6.150208705	-5.963941065	-4.578243632	-5.562771124	-6.295566442	3.09682952	0.076428003

TP53	0.030098952	-0.194714501	0.117141734	-0.224813453	0.087042783	0.311856236	8	0.080547984	0.816496581	0.816496581	0.816496581	0.816496581	0.816496581	0.816496581	27	142	0	P_120426	P_120426	P_120426	chr17	7674870	7674930	-	TP53	tumor protein p53 [Source:HGNC Symbol;Acc:HGNC:11998]	ENSG00000141510	protein_coding	exon	coding exon	1	4562	17:7674870:7674930	ENSG00000141510	TP53	8.160215171	0.007416884	0.428041876	-2.769065233	1.66589084	-3.197107109	1.237848964	4.434956073	9.961963646	0.677726843	0.019885625	0.12682072	0.009583262	0.244151632	0.001276142	-6.899294406	-3.227210991	-5.711486799	-3.313426471	-5.540489704	-1.172821208	7.008122282	0.008112444

NBN	-0.009884125	-0.003906759	0.023699969	0.005977366	0.033584095	0.027606728	8	0.077968805	0.816496581	0.816496581	0.816496581	0.816496581	0.816496581	0.816496581	27	143	0	P_265323	P_265323	P_265323	chr8	89982721	89982781	-	NBN	nibrin [Source:HGNC Symbol;Acc:HGNC:7652]	ENSG00000104320	protein_coding	5'UTR;exon	multiple Ref	1	4563	8:89982721:89982781	ENSG00000104320	NBN	10.00684711	0.007088561	-0.143782108	-0.056830726	0.344758027	0.086951382	0.488540135	0.401588753	9.961963646	0.888539471	0.955803754	0.737444005	0.932433082	0.63574264	0.696466472	-6.987090847	-6.180385089	-6.982598001	-7.145480033	-6.171285712	-6.983984774	0.091711109	0.962925668

WDR17	0.025658083	0.035989803	0.0608502	0.01033172	0.035192117	0.024860397	8	0.106438515	0.816496581	0.816496581	0.816496581	0.816496581	0.816496581	0.816496581	27	145	0	P_251696	P_251696	P_251696	chr4	176139914	176139974	+	WDR17	WD repeat domain 17 [Source:HGNC Symbol;Acc:HGNC:16661]	ENSG00000150627	protein_coding	exon	coding exon	1	4565	4:176139914:176139974	ENSG00000150627	WDR17	3.585537251	0.011304615	0.295557479	0.414569368	0.700938237	0.119011889	0.405380758	0.286368869	9.961963646	0.7736367	0.687243099	0.499386259	0.907631646	0.693766606	0.780462666	-6.95090128	-6.092288614	-6.78526292	-7.141872873	-6.210015253	-7.026762435	0.168506819	0.915190725

KANSL1	0.093339493	-0.093299482	0.005340205	-0.186638975	-0.087999288	0.098639687	8	0.063826072	0.816496581	0.816496581	0.816496581	0.816496581	0.816496581	0.816496581	27	148	0	P_229003	P_229003	P_229003	chr17	46160243	46160303	+	KANSL1	KAT8 regulatory NSL complex subunit 1 [Source:HGNC Symbol;Acc:HGNC:24565]	ENSG00000120071	protein_coding	intron	intron antisense	0	4568	17:46160243:46160303	ENSG00000120071	KANSL1	6.19361549	0.005478141	1.544523238	-1.543861166	0.088366356	-3.088384404	-1.456156882	1.632227522	9.961963646	0.153610353	0.15376926	0.931336673	0.011525375	0.176131081	0.133798648	-5.832420771	-5.060927967	-7.042857922	-3.49995882	-5.277684469	-5.782764077	3.183257621	0.071866345

RNF212	0.033666611	0.13370482	-0.221662555	0.100038209	-0.255329165	-0.355367375	8	0.241346468	0.816496581	0.816496581	0.816496581	0.816496581	0.816496581	0.816496581	27	149	0	P_137094	P_137094	P_137094	chr4	1072710	1072770	-	RNF212	ring finger protein 212 [Source:HGNC Symbol;Acc:HGNC:27729]	ENSG00000178222	protein_coding	3'UTR	3'UTR	1	4569	4:1072710:1072770	ENSG00000178222	RNF212	3.95565543	0.048983099	0.18630388	0.739894102	-1.226633542	0.553590222	-1.412937422	-1.966527644	9.961963646	0.855945575	0.476438935	0.248162013	0.592066317	0.188147415	0.077700659	-6.979453001	-5.901461075	-6.282281958	-6.984560624	-5.331971945	-5.288587265	1.376144588	0.306089178

MSTN	0.045289535	-0.099590004	-0.262827944	-0.144879539	-0.308117479	-0.16323794	8	0.183230854	0.816496581	0.816496581	0.816496581	0.816496581	0.816496581	0.816496581	27	151	0	P_238316	P_238316	P_238316	chr2	190062226	190062286	-	MSTN	myostatin [Source:HGNC Symbol;Acc:HGNC:4223]	ENSG00000138379	protein_coding	exon	coding exon	1	4571	2:190062226:190062286	ENSG00000138379	MSTN	3.42243869	0.029168072	0.324780263	-0.714179732	-1.884791469	-1.038959995	-2.209571732	-1.170611737	9.961963646	0.752064778	0.49151075	0.088933227	0.323382979	0.051696846	0.268995652	-6.941104572	-5.920137405	-5.389998323	-6.589329113	-4.199333104	-6.370300632	1.916658456	0.191101216

PHLPP2	-0.148296162	-0.061474388	-0.162659028	0.086821774	-0.014362867	-0.101184641	8	0.105135447	0.816496581	0.816496581	0.816496581	0.816496581	0.816496581	0.816496581	27	152	0	A_24_P525401	A_24_P525401	A_24_P525401	chr16	71636049	71636109	-	PHLPP2;MARVELD3	PH domain and leucine rich repeat protein phosphatase 2 [Source:HGNC Symbol;Acc:HGNC:29149];MARVEL domain containing 3 [Source:HGNC Symbol;Acc:HGNC:30525]	ENSG00000040199	protein_coding	3'ext;3'UTR;intron;promoter	multiple non-Ref	0	4572	16:71636049:71636109	ENSG00000040199	PHLPP2	5.52338723	0.011083217	-1.725212617	-0.715166112	-1.89230392	1.010046506	-0.167091302	-1.177137808	9.961963646	0.115316821	0.490927189	0.087840828	0.336376206	0.87064124	0.266499227	-5.578898606	-5.919431919	-5.378617838	-6.618620319	-6.281856708	-6.362954699	1.583733689	0.254447347

DDX4	0.000755992	-0.049649362	0.099800772	-0.050405353	0.09904478	0.149450134	8	0.102504074	0.816496581	0.816496581	0.816496581	0.816496581	0.816496581	0.816496581	27	155	0	P_139530	P_139530	P_139530	chr5	55817032	55817092	+	DDX4	DEAD (Asp-Glu-Ala-Asp) box polypeptide 4 [Source:HGNC Symbol;Acc:HGNC:18700]	ENSG00000152670	protein_coding	3'UTR	3'UTR	1	4575	5:55817032:55817092	ENSG00000152670	DDX4	3.553710094	0.010644446	0.00897431	-0.589383213	1.184726202	-0.598357523	1.175751892	1.774109415	9.961963646	0.993016808	0.568728725	0.263619635	0.562957801	0.267027837	0.106555629	-6.998316369	-6.002213314	-6.330387357	-6.957274594	-5.609698175	-5.578756038	1.106486289	0.39168208

TTC3	0.198760881	-0.243739699	-0.029806889	-0.442500581	-0.228567771	0.21393281	8	0.062764214	0.816496581	0.816496581	0.816496581	0.816496581	0.816496581	0.816496581	27	156	0	P_241792	P_241792	P_241792	chr21	37167560	37167620	+	TTC3	tetratricopeptide repeat domain 3 [Source:HGNC Symbol;Acc:HGNC:12393]	ENSG00000182670	protein_coding	exon	coding exon	1	4576	21:37167560:37167620	ENSG00000182670	TTC3	10.95666756	0.005370194	3.321861747	-4.0735862	-0.498158215	-7.395447947	-3.820019962	3.575427985	9.961963646	0.007762757	0.002254932	0.6291859	2.38E-05	0.003396826	0.005080369	-2.961200016	-1.177332748	-6.913269813	2.922518728	-1.612974823	-2.595753491	18.32431995	0.000222267

BOD1L1	0.075160806	-0.007171596	0.046450577	-0.082332403	-0.02871023	0.053622173	8	0.074119142	0.816496581	0.816496581	0.816496581	0.816496581	0.816496581	0.816496581	27	157	0	P_248224	P_248224	P_248224	chr4	13586411	13586471	-	BOD1L1	biorientation of chromosomes in cell division 1-like 1 [Source:HGNC Symbol;Acc:HGNC:31792]	ENSG00000038219	protein_coding	exon	coding exon	1	4577	4:13586411:13586471	ENSG00000038219	BOD1L1	9.142367389	0.006618382	1.131517142	-0.107965631	0.699295631	-1.239482773	-0.43222151	0.807261263	9.961963646	0.284340238	0.916166509	0.500368597	0.24357183	0.674786521	0.438365232	-6.341418314	-6.175946149	-6.78645965	-6.368617153	-6.198271425	-6.726607575	0.692634287	0.577245607

SLC22A23	-0.002492593	-0.000814128	0.057932963	0.001678464	0.060425555	0.058747091	8	0.101310125	0.816496581	0.816496581	0.816496581	0.816496581	0.816496581	0.816496581	27	160	0	A_24_P252223	A_24_P252223	A_24_P252223	chr6	3270111	3270171	-	SLC22A23	"solute carrier family 22, member 23 [Source:HGNC Symbol;Acc:HGNC:21106]"	ENSG00000137266	protein_coding	3'UTR	3'UTR	1	4580	6:3270111:3270171	ENSG00000137266	SLC22A23	6.615090237	0.010449028	-0.029864758	-0.009754401	0.694118187	0.020110357	0.723982945	0.703872588	9.961963646	0.976764753	0.992409815	0.50347264	0.98435243	0.48573014	0.497634362	-6.997873635	-6.182037265	-6.790215056	-7.149391901	-6.025218438	-6.807280971	0.250463243	0.859224065

CMBL	-0.118663077	0.076671237	0.199842899	0.195334314	0.318505977	0.123171662	8	0.094096056	0.816496581	0.816496581	0.816496581	0.816496581	0.816496581	0.816496581	28	10	0	P_312758	P_312758	P_312758	chr5	10283980	10284040	+	CMBL	carboxymethylenebutenolidase homolog (Pseudomonas) [Source:HGNC Symbol;Acc:HGNC:25090]	ENSG00000164237	protein_coding	intron;promoter	multiple non-Ref	0	4600	5:10283980:10284040	ENSG00000164237	CMBL	9.2155559	0.009316983	-1.505648449	0.972837813	2.535693138	2.478486262	4.041341587	1.562855325	9.961963646	0.163188371	0.353662935	0.029660884	0.032710779	0.002374454	0.149267313	-5.884777289	-5.706393442	-4.343760699	-4.53722455	-1.268028289	-5.878976402	5.760163849	0.015011578

NR3C2	-0.001486471	0.029966868	0.005694242	0.031453339	0.007180714	-0.024272625	8	0.096696243	0.816496581	0.816496581	0.816496581	0.816496581	0.816496581	0.816496581	28	12	0	P_251133	P_251133	P_251133	chr4	148419110	148419170	-	NR3C2	"nuclear receptor subfamily 3, group C, member 2 [Source:HGNC Symbol;Acc:HGNC:7979]"	ENSG00000151623	protein_coding	intron	intron sense	0	4602	4:148419110:148419170	ENSG00000151623	NR3C2	5.770084104	0.009715375	-0.018470232	0.372355005	0.07075413	0.390825237	0.089224362	-0.301600875	9.961963646	0.98562842	0.717427628	0.944993679	0.70415414	0.930671911	0.769158199	-6.998174158	-6.109521036	-7.044387386	-7.066720017	-6.292452662	-7.021912172	0.065943998	0.976726235

RPL13A	-0.279140235	-0.202383645	-0.266273457	0.07675659	0.012866778	-0.063889812	8	0.10910996	0.816496581	0.816496581	0.816496581	0.816496581	0.816496581	0.816496581	28	13	0	P_233150	P_233150	P_233150	chr19	49490717	49490777	+	RPL13A	ribosomal protein L13a [Source:HGNC Symbol;Acc:HGNC:10304]	ENSG00000142541	protein_coding	exon	non-coding exon	1	4603	19:49490717:49490777	ENSG00000142541	RPL13A	9.497295982	0.011767035	-3.151625095	-2.285006941	-3.006353094	0.866618154	0.145272001	-0.721346153	9.961963646	0.010351358	0.045492229	0.01325347	0.406540131	0.887393355	0.487280771	-3.251781287	-3.991200716	-3.54496666	-6.753735634	-6.285472614	-6.794340646	4.24743794	0.035523747

MORC4	-0.290443566	0.188241623	-0.027634336	0.47868519	0.262809231	-0.215875959	8	0.096459401	0.816496581	0.816496581	0.816496581	0.816496581	0.816496581	0.816496581	28	15	0	P_271047	P_271047	P_271047	chrX	106978079	106978139	-	MORC4	MORC family CW-type zinc finger 4 [Source:HGNC Symbol;Acc:HGNC:23485]	ENSG00000133131	protein_coding	exon	coding exon	1	4605	X:106978079:106978139	ENSG00000133131	MORC4	7.289269609	0.009678637	-3.6157657	2.343441835	-0.344023053	5.959207536	3.271742647	-2.687464888	9.961963646	0.00475186	0.041189046	0.737979997	0.000141676	0.008447141	0.022869914	-2.462307671	-3.900514742	-6.982871185	1.055738563	-2.489570805	-4.110990135	12.02045917	0.001196775

OR5AN1	-0.058131416	-0.264053226	-0.137281093	-0.20592181	-0.079149677	0.126772133	8	0.404826065	0.816496581	0.816496581	0.816496581	0.816496581	0.816496581	0.816496581	28	16	0	P_108661	P_108661	P_108661	chr11	59365319	59365379	+	OR5AN1	"olfactory receptor, family 5, subfamily AN, member 1 [Source:HGNC Symbol;Acc:HGNC:15255]"	ENSG00000176495	protein_coding	exon	coding exon	1	4606	11:59365319:59365379	ENSG00000176495	OR5AN1	3.141857372	0.133814587	-0.194627768	-0.884067404	-0.459626043	-0.689439636	-0.264998275	0.424441361	9.961963646	0.849596188	0.397494273	0.655651671	0.506287671	0.796413249	0.680264324	-6.977729035	-5.786068865	-6.932966682	-6.895719896	-6.259469396	-6.973847201	0.292735468	0.82979418

RBMS3-AS2	-0.271237933	0.033087158	-0.089692267	0.304325091	0.181545666	-0.122779425	8	0.45817832	0.816496581	0.816496581	0.816496581	0.816496581	0.816496581	0.816496581	28	17	0	P_134279	P_134279	P_134279	chr3	29642732	29642792	-	RBMS3-AS2	RBMS3 antisense RNA 2 [Source:HGNC Symbol;Acc:HGNC:39988]	ENSG00000203506	antisense	exon	non-coding exon	1	4607	3:29642732:29642792	ENSG00000203506	RBMS3-AS2	4.228624123	0.170789811	-0.803831603	0.098055987	-0.265808982	0.901887591	0.538022621	-0.363864969	9.961963646	0.440253651	0.923832729	0.795806301	0.388401918	0.602373473	0.72356253	-6.656816824	-6.177021228	-7.008672701	-6.722123533	-6.144988366	-6.999528982	0.326946987	0.806036203

ADAM18	-0.099202213	-0.126560529	-0.119062073	-0.027358315	-0.019859859	0.007498456	8	0.193234453	0.816496581	0.816496581	0.816496581	0.816496581	0.816496581	0.816496581	28	18	0	P_264501	P_264501	P_264501	chr8	39645414	39645474	+	ADAM18	ADAM metallopeptidase domain 18 [Source:HGNC Symbol;Acc:HGNC:196]	ENSG00000168619	protein_coding	3'UTR;exon	multiple Ref	1	4608	8:39645414:39645474	ENSG00000168619	ADAM18	3.649829385	0.032192382	-0.677158661	-0.863907723	-0.812722933	-0.186749062	-0.135564272	0.05118479	9.961963646	0.513722373	0.407957907	0.43536909	0.855605716	0.894865899	0.960190048	-6.753770182	-5.803267366	-6.697944422	-7.130574484	-6.286919724	-7.069947463	0.320211957	0.81070132

DISP1	0.055352813	0.049578697	0.300133127	-0.005774116	0.244780315	0.250554431	8	0.135956036	0.816496581	0.816496581	0.816496581	0.816496581	0.816496581	0.816496581	28	19	0	P_104873	P_104873	P_104873	chr1	223004817	223004877	-	DISP1	dispatched homolog 1 (Drosophila) [Source:HGNC Symbol;Acc:HGNC:19711]	ENSG00000154309	protein_coding	exon	exon antisense	0	4609	1:223004817:223004877	ENSG00000154309	DISP1	5.259692262	0.017050379	0.519180418	0.46502223	2.815091682	-0.054158188	2.295911265	2.350069452	9.961963646	0.614972584	0.651913896	0.01837823	0.95787985	0.044657333	0.0407268	-6.853240668	-6.069359501	-3.871435776	-7.148009282	-4.064551786	-4.675232512	3.201250539	0.070957709

ELAPOR2	-0.00928698	-0.037115209	-0.091938109	-0.027828229	-0.082651129	-0.0548229	8	0.056755258	0.816496581	0.816496581	0.816496581	0.816496581	0.816496581	0.816496581	28	21	0	P_261556	P_261556	P_261556	chr7	86912943	86913003	-	KIAA1324L	KIAA1324-like [Source:HGNC Symbol;Acc:HGNC:21945]	ENSG00000164659	protein_coding	3'UTR;exon	multiple Ref	1	4611	7:86912943:86913003	ENSG00000164659	ELAPOR2	7.679547171	0.004793451	-0.164284279	-0.656558474	-1.62636145	-0.492274195	-1.462077171	-0.969802976	9.961963646	0.872792806	0.526339818	0.135049137	0.633193127	0.17453755	0.355101086	-6.983652531	-5.959819043	-5.767246293	-7.018684569	-5.270166714	-6.580560069	1.070605212	0.404941427

CCDC61	0.016772849	0.412356247	0.265949828	0.395583398	0.249176979	-0.146406419	8	0.156734298	0.816496581	0.816496581	0.816496581	0.816496581	0.816496581	0.816496581	28	22	0	P_126140	P_126140	P_126140	chr19	46003438	46003498	+	CCDC61	coiled-coil domain containing 61 [Source:HGNC Symbol;Acc:HGNC:33629]	ENSG00000104983	protein_coding	exon	coding exon	1	4612	19:46003438:46003498	ENSG00000104983	CCDC61	6.709185915	0.021934233	0.138704724	3.410020472	2.199298217	3.271315748	2.060593493	-1.210722255	9.961963646	0.89244731	0.006694231	0.052602694	0.00845323	0.066427283	0.253942698	-6.987871952	-2.204079673	-4.897576247	-3.186186652	-4.427938245	-6.324667616	5.482965319	0.01739592

CROCCP3	0.20352267	-0.150465627	0.116403486	-0.353988298	-0.087119184	0.266869114	8	0.390281922	0.816496581	0.816496581	0.816496581	0.816496581	0.816496581	0.816496581	28	23	0	P_200453	P_200453	P_200453	chr1	16492042	16492102	-	CROCCP3	"ciliary rootlet coiled-coil, rootletin pseudogene 3 [Source:HGNC Symbol;Acc:HGNC:29405]"	ENSG00000080947	transcribed_unprocessed_pseudogene	exon	non-coding exon	1	4613	1:16492042:16492102	ENSG00000080947	CROCCP3	8.45803688	0.124527933	0.706358268	-0.522215239	0.403997083	-1.228573507	-0.302361185	0.926212321	9.961963646	0.496153299	0.612934257	0.694751249	0.247464538	0.768595392	0.376228767	-6.7327443	-6.040336472	-6.958715601	-6.381377192	-6.248288111	-6.621947005	0.565591469	0.650057217

CCDC115	-0.119606238	0.239502097	-0.078396717	0.359108335	0.041209521	-0.317898813	8	0.118480081	0.816496581	0.816496581	0.816496581	0.816496581	0.816496581	0.816496581	28	24	0	A_23_P302094	A_23_P302094	A_23_P302094	chr2	130346092	130346240	+	CCDC115;IMP4	"coiled-coil domain containing 115 [Source:HGNC Symbol;Acc:HGNC:28178];IMP4, U3 small nucleolar ribonucleoprotein [Source:HGNC Symbol;Acc:HGNC:30856]"	ENSG00000136710	protein_coding	"3'ext;exon;exon,intron;promoter"	multiple non-Ref	0	4614	2:130346092:130346240	ENSG00000136710	CCDC115	10.67356728	0.013479586	-1.261714772	2.526484721	-0.826999472	3.788199493	0.434715299	-3.353484194	9.961963646	0.235792866	0.030132096	0.427602042	0.003578058	0.673034898	0.007360574	-6.192973805	-3.613161044	-6.68597434	-2.306909099	-6.197143504	-2.973515878	5.740711062	0.015165591

NCOR1	-0.156440574	-0.082842483	-0.227939645	0.073598091	-0.071499072	-0.145097162	8	0.053605287	0.816496581	0.816496581	0.816496581	0.816496581	0.816496581	0.816496581	28	26	0	P_227912	P_227912	P_227912	chr17	16075533	16075593	-	NCOR1	nuclear receptor corepressor 1 [Source:HGNC Symbol;Acc:HGNC:7672]	ENSG00000141027	protein_coding	exon	coding exon	1	4616	17:16075533:16075593	ENSG00000141027	NCOR1	8.769950082	0.004514283	-2.851679127	-1.510095332	-4.155000924	1.341583795	-1.303321797	-2.644905592	9.961963646	0.017262485	0.162066963	0.001980448	0.20951116	0.221781258	0.024600274	-3.763727164	-5.104551141	-1.606552213	-6.245291417	-5.464750945	-4.182977776	6.361752468	0.011054542

RAVER1	-0.042706952	-0.181729541	-0.05427824	-0.139022589	-0.011571288	0.127451301	8	0.136603072	0.816496581	0.816496581	0.816496581	0.816496581	0.816496581	0.816496581	28	28	0	A_24_P384397	A_24_P384397	A_24_P384397	chr19	10321114	10321174	-	RAVER1	"ribonucleoprotein, PTB-binding 1 [Source:HGNC Symbol;Acc:HGNC:30296]"	ENSG00000161847	protein_coding	exon	coding exon	1	4618	19:10321114:10321174	ENSG00000161847	RAVER1	9.149429658	0.017192002	-0.398915559	-1.697492763	-0.507000232	-1.298577204	-0.108084673	1.190492531	9.961963646	0.698372437	0.12056949	0.623187879	0.223343508	0.91607447	0.261448096	-6.912214229	-4.855523918	-6.908537108	-6.298082847	-6.290475361	-6.347826501	1.068820342	0.405613629

ZC3HC1	-0.044208485	-0.078759614	-0.127786731	-0.034551129	-0.083578245	-0.049027116	8	0.107245406	0.816496581	0.816496581	0.816496581	0.816496581	0.816496581	0.816496581	28	30	0	A_23_P215088	A_23_P215088	A_23_P215088	chr7	130018550	130018610	-	ZC3HC1	"zinc finger, C3HC-type containing 1 [Source:HGNC Symbol;Acc:HGNC:29913]"	ENSG00000091732	protein_coding	3'UTR	3'UTR	1	4620	7:130018550:130018610	ENSG00000091732	ZC3HC1	7.578569445	0.011443078	-0.506150703	-0.90173264	-1.463052697	-0.395581937	-0.956901994	-0.561320057	9.961963646	0.623762916	0.388480343	0.17427617	0.700752366	0.361262203	0.586983436	-6.860342035	-5.770722229	-5.989047952	-7.064705806	-5.831457032	-6.90193396	0.766176697	0.538662245

PRDX2	-0.012001118	-0.077462368	0.020295129	-0.06546125	0.032296247	0.097757497	8	0.103629702	0.816496581	0.816496581	0.816496581	0.816496581	0.816496581	0.816496581	28	31	0	P_124782	P_124782	P_124782	chr19	12799990	12800050	-	PRDX2	peroxiredoxin 2 [Source:HGNC Symbol;Acc:HGNC:9353]	ENSG00000167815	protein_coding	exon	non-coding exon	1	4621	19:12799990:12800050	ENSG00000167815	PRDX2	12.2936926	0.010830779	-0.14123342	-0.911604695	0.238840296	-0.770371274	0.380073716	1.150444991	9.961963646	0.890500699	0.383506099	0.816071051	0.458959265	0.711868309	0.276827332	-6.987486433	-5.762034523	-7.016057429	-6.834419079	-6.220421126	-6.392804978	0.494911049	0.693884178

NIPAL3	-0.12073763	-0.026840295	-0.376632943	0.093897335	-0.255895313	-0.349792648	8	0.077463767	0.816496581	0.816496581	0.816496581	0.816496581	0.816496581	0.816496581	28	32	0	A_32_P35031	A_32_P35031	A_32_P35031	chr1	24472037	24472097	-	NIPAL3;AL031431.1	NIPA-like domain containing 3 [Source:HGNC Symbol;Acc:HGNC:25233];	ENSG00000001461	protein_coding;miRNA	3'ext;3'UTR;promoter	multiple non-Ref	0	4622	1:24472037:24472097	ENSG00000001461	NIPAL3	8.05615887	0.007025521	-1.764204798	-0.392187395	-5.503318608	1.372017403	-3.73911381	-5.111131213	9.961963646	0.108279651	0.703179283	0.000264135	0.200161535	0.003877679	0.000462061	-5.522171869	-6.101648157	0.483746651	-6.207215827	-1.740533995	-0.117319496	12.58996855	0.001002007

TLL1	-0.000490444	0.032266475	0.034097906	0.03275692	0.034588351	0.001831431	8	0.096469858	0.816496581	0.816496581	0.816496581	0.816496581	0.816496581	0.816496581	28	33	0	A_24_P145163	A_24_P145163	A_24_P145163	chr4	166030867	166030927	+	TLL1	tolloid-like 1 [Source:HGNC Symbol;Acc:HGNC:11843]	ENSG00000038295	protein_coding	3'UTR	3'UTR	1	4623	4:166030867:166030927	ENSG00000038295	TLL1	3.73506324	0.009680258	-0.006105088	0.401655483	0.424453273	0.40776057	0.430558361	0.02279779	9.961963646	0.995249402	0.696418923	0.680255923	0.692074496	0.675955842	0.982261753	-6.998339979	-6.097750118	-6.949618665	-7.059441279	-6.199020185	-7.071094597	0.115615651	0.948909266

SLC25A27	-0.287875681	-0.066706217	-0.209902459	0.221169465	0.077973222	-0.143196242	8	0.101878338	0.816496581	0.816496581	0.816496581	0.816496581	0.816496581	0.816496581	28	34	0	P_257216	P_257216	P_257216	chr6	46658986	46659046	+	SLC25A27	"solute carrier family 25, member 27 [Source:HGNC Symbol;Acc:HGNC:21065]"	ENSG00000153291	protein_coding	exon	coding exon	1	4624	6:46658986:46659046	ENSG00000153291	SLC25A27	6.165367142	0.010541744	-3.43395321	-0.795711629	-2.503842005	2.638241581	0.930111205	-1.708130376	9.961963646	0.006431228	0.444746094	0.031322507	0.02488281	0.374303145	0.118528671	-2.770378986	-5.858908206	-4.397045521	-4.268566273	-5.85606706	-5.6747758	4.906258551	0.023980322

TRAPPC13	0.104418633	-0.015537305	0.081338206	-0.119955938	-0.023080427	0.096875511	8	0.103091316	0.816496581	0.816496581	0.816496581	0.816496581	0.816496581	0.816496581	28	36	0	P_253094	P_253094	P_253094	chr5	65635983	65636043	+	TRAPPC13	trafficking protein particle complex 13 [Source:HGNC Symbol;Acc:HGNC:25828]	ENSG00000113597	protein_coding	exon	coding exon	1	4626	5:65635983:65636043	ENSG00000113597	TRAPPC13	7.7133424	0.010741402	1.233937432	-0.183607667	0.961191069	-1.417545099	-0.272746363	1.144798736	9.961963646	0.2455443	0.858004561	0.359205318	0.18683383	0.790618411	0.279051946	-6.225646931	-6.164346789	-6.564782484	-6.149180172	-6.257269795	-6.399052271	0.979098411	0.44100627

IFT122	-0.085442742	-0.144229551	0.016972539	-0.058786809	0.10241528	0.161202089	8	0.072437075	0.816496581	0.816496581	0.816496581	0.816496581	0.816496581	0.816496581	28	40	0	P_135969	P_135969	P_135969	chr3	129466953	129467013	+	IFT122	intraflagellar transport 122 [Source:HGNC Symbol;Acc:HGNC:13556]	ENSG00000163913	protein_coding	3'UTR;exon	multiple Ref	1	4630	3:129466953:129467013	ENSG00000163913	IFT122	6.298148415	0.006420415	-1.305988164	-2.204541691	0.259424432	-0.898553527	1.565412596	2.463966122	9.961963646	0.220907281	0.052138464	0.800590027	0.390091839	0.148670147	0.033532945	-6.139819675	-4.115042185	-7.010491329	-6.725157807	-5.135934993	-4.486635612	2.660325712	0.105450716

GMPR	0.17003995	0.24776384	0.229120856	0.07772389	0.059080906	-0.018642984	8	0.073993072	0.816496581	0.816496581	0.816496581	0.816496581	0.816496581	0.816496581	28	42	0	P_141703	P_141703	P_141703	chr6	16295237	16295297	+	GMPR	guanosine monophosphate reductase [Source:HGNC Symbol;Acc:HGNC:4376]	ENSG00000137198	protein_coding	3'UTR;exon	multiple Ref	1	4632	6:16295237:16295297	ENSG00000137198	GMPR	7.853678914	0.006603387	2.562791161	3.734222327	3.453240864	1.171431166	0.890449703	-0.280981464	9.961963646	0.028316177	0.003908939	0.006227057	0.268681179	0.394220904	0.784473988	-4.252289515	-1.696136085	-2.781425495	-6.446842524	-5.891424353	-7.028418761	5.780214937	0.014854782

AKAP10	-0.176596811	0.028358416	-0.292269183	0.204955227	-0.115672372	-0.320627599	8	0.200292939	0.816496581	0.816496581	0.816496581	0.816496581	0.816496581	0.816496581	28	43	0	P_120814	P_120814	P_120814	chr17	19905883	19905943	-	AKAP10	A kinase (PRKA) anchor protein 10 [Source:HGNC Symbol;Acc:HGNC:368]	ENSG00000108599	protein_coding	3'UTR	3'UTR	1	4633	17:19905883:19905943	ENSG00000108599	AKAP10	6.759521284	0.034423032	-1.165745976	0.187198786	-1.929319232	1.352944763	-0.763573255	-2.116518018	9.961963646	0.270869003	0.855262422	0.082638574	0.205978933	0.462821836	0.060479877	-6.303569168	-6.163647317	-5.322239875	-6.231146259	-5.995597906	-5.053459813	2.001571989	0.178003821

IFT22	0.213700807	0.192663408	0.349030175	-0.021037399	0.135329369	0.156366767	8	0.061437538	0.816496581	0.816496581	0.816496581	0.816496581	0.816496581	0.816496581	28	46	0	A_23_P31584	A_23_P31584	A_23_P31584	chr7	101314848	101314908	-	IFT22	intraflagellar transport 22 [Source:HGNC Symbol;Acc:HGNC:21895]	ENSG00000128581	protein_coding	3'UTR;exon	multiple Ref	1	4636	7:101314848:101314908	ENSG00000128581	IFT22	10.46432595	0.00523787	3.616382981	3.260374544	5.906513901	-0.356008437	2.29013092	2.646139357	9.961963646	0.004747008	0.008610831	0.000152031	0.729258037	0.045098036	0.024548318	-2.461265443	-2.441579673	1.058604256	-7.080741945	-4.073621296	-4.180893491	11.8281116	0.001272614

DPCD	0.15047193	0.08030068	0.145592183	-0.070171251	-0.004879748	0.065291503	8	0.08053637	0.816496581	0.816496581	0.816496581	0.816496581	0.816496581	0.816496581	28	47	0	P_106911	P_106911	P_106911	chr10	101609406	101609466	+	DPCD	deleted in primary ciliary dyskinesia homolog (mouse) [Source:HGNC Symbol;Acc:HGNC:24542]	ENSG00000166171	protein_coding	exon	coding exon	1	4637	10:101609406:101609466	ENSG00000166171	DPCD	10.58442281	0.007415382	2.140101503	1.142084143	2.070698821	-0.99801736	-0.069402682	0.928614678	9.961963646	0.058127361	0.280126482	0.065312936	0.341894973	0.946042461	0.375041431	-4.94565302	-5.537631445	-5.10258654	-6.630608782	-6.294123941	-6.619707537	2.005894141	0.177365593

KSR1	-0.021204989	-0.023920093	0.112703294	-0.002715104	0.133908283	0.136623387	8	0.106661121	0.816496581	0.816496581	0.816496581	0.816496581	0.816496581	0.816496581	28	49	0	A_24_P255483	A_24_P255483	A_24_P255483	chr17	27601919	27603854	+	KSR1	kinase suppressor of ras 1 [Source:HGNC Symbol;Acc:HGNC:6465]	ENSG00000141068	protein_coding	"exon,intron"	multiple non-Ref	0	4639	17:27601919:27603854	ENSG00000141068	KSR1	5.274114383	0.01134271	-0.24385141	-0.275074347	1.296056178	-0.031222938	1.539907587	1.571130525	9.961963646	0.812294324	0.788879893	0.224177299	0.975708437	0.154721187	0.147342529	-6.966008792	-6.142355537	-6.199916387	-7.149079965	-5.169581806	-5.867629781	1.109272066	0.390672619

SLC12A4	-0.074977748	-0.017888728	-0.01046166	0.05708902	0.064516088	0.007427068	8	0.162354157	0.816496581	0.816496581	0.816496581	0.816496581	0.816496581	0.816496581	28	53	0	P_226695	P_226695	P_226695	chr16	67952299	67952359	-	SLC12A4	"solute carrier family 12 (potassium/chloride transporter), member 4 [Source:HGNC Symbol;Acc:HGNC:10913]"	ENSG00000124067	protein_coding	3'UTR;exon	multiple Ref	1	4643	16:67952299:67952359	ENSG00000124067	SLC12A4	5.198408477	0.023374296	-0.600632397	-0.143303178	-0.083806357	0.457329219	0.516826039	0.05949682	9.961963646	0.561500148	0.88890795	0.934870568	0.657245651	0.616556268	0.95373301	-6.80500603	-6.171273141	-7.043286356	-7.036427193	-6.156546989	-7.069445027	0.144686271	0.93074006

VPS13A	0.011725089	-0.075565496	-0.073754514	-0.087290584	-0.085479603	0.001810981	8	0.093727013	0.816496581	0.816496581	0.816496581	0.816496581	0.816496581	0.816496581	28	54	0	P_267660	P_267660	P_267660	chr9	77317638	77317698	+	VPS13A	vacuolar protein sorting 13 homolog A (S. cerevisiae) [Source:HGNC Symbol;Acc:HGNC:1908]	ENSG00000197969	protein_coding	3'UTR;exon	multiple Ref	1	4644	9:77317638:77317698	ENSG00000197969	VPS13A	8.234140274	0.00926132	0.149219412	-0.961684748	-0.938637284	-1.11090416	-1.087856696	0.023047464	9.961963646	0.884358091	0.358969114	0.370116784	0.292702392	0.302276721	0.982067527	-6.986223368	-5.716750384	-6.586224275	-6.513597029	-5.703051735	-7.071088344	0.707702561	0.569128388

ARL13A	-0.031055456	0.194208141	0.063314118	0.225263597	0.094369573	-0.130894023	8	0.133827337	0.816496581	0.816496581	0.816496581	0.816496581	0.816496581	0.816496581	28	55	0	P_151339	P_151339	P_151339	chrX	100988222	100988282	+	ARL13A	ADP-ribosylation factor-like 13A [Source:HGNC Symbol;Acc:HGNC:31709]	ENSG00000174225	protein_coding	exon	coding exon	1	4645	X:100988222:100988282	ENSG00000174225	ARL13A	3.694566318	0.016589195	-0.295305058	1.846717259	0.602051352	2.142022317	0.89735641	-1.244665907	9.961963646	0.773823949	0.094666248	0.560592006	0.057939662	0.390699876	0.241739845	-6.950981955	-4.646360499	-6.85271648	-5.088472903	-5.885360566	-6.285162661	1.80045615	0.210881302

CD1C	-0.25670712	-0.230911798	0.003858989	0.025795322	0.260566109	0.234770786	8	0.114715922	0.816496581	0.816496581	0.816496581	0.816496581	0.816496581	0.816496581	28	57	0	P_204369	P_204369	P_204369	chr1	158291340	158291400	+	CD1C	CD1c molecule [Source:HGNC Symbol;Acc:HGNC:1636]	ENSG00000158481	protein_coding	exon	coding exon	1	4647	1:158291340:158291400	ENSG00000158481	CD1C	4.118037184	0.012774675	-2.78168926	-2.502170052	0.041816165	0.279519208	2.823505425	2.543986217	9.961963646	0.019460211	0.031412229	0.967471481	0.785563895	0.018115376	0.029242763	-3.882718894	-3.6515741	-7.046165763	-7.10705353	-3.218255179	-4.352876971	4.753763075	0.026195122

ZNF396	0.339102488	0.004415313	0.172541952	-0.334687175	-0.166560536	0.168126639	8	0.081018889	0.816496581	0.816496581	0.816496581	0.816496581	0.816496581	0.816496581	28	58	0	P_123444	P_123444	P_123444	chr18	35369337	35369397	-	ZNF396	zinc finger protein 396 [Source:HGNC Symbol;Acc:HGNC:18824]	ENSG00000186496	protein_coding	exon	coding exon	1	4648	18:35369337:35369397	ENSG00000186496	ZNF396	6.300044608	0.007477982	4.802688167	0.062533815	2.443701302	-4.740154353	-2.358986865	2.381167488	9.961963646	0.000728503	0.95137463	0.034714527	0.000800282	0.040112839	0.038624699	-0.531789699	-6.180026352	-4.497246087	-0.754374395	-3.96519308	-4.623969312	10.45754018	0.002018088

ADAMTS17	0.018397018	0.069688887	-0.288695648	0.051291868	-0.307092667	-0.358384535	8	0.123833876	0.816496581	0.816496581	0.816496581	0.816496581	0.816496581	0.816496581	28	60	0	A_14_P129746	A_14_P129746	A_14_P129746	chr15	100332686	100332746	+	ADAMTS17	"ADAM metallopeptidase with thrombospondin type 1 motif, 17 [Source:HGNC Symbol;Acc:HGNC:17109]"	ENSG00000140470	protein_coding	intron	intron antisense	0	4650	15:100332686:100332746	ENSG00000140470	ADAMTS17	3.785029289	0.014521388	0.186977355	0.708280194	-2.934146589	0.521302839	-3.121123944	-3.642426783	9.961963646	0.855431446	0.495010008	0.014991963	0.613546704	0.010901509	0.00454692	-6.979316298	-5.924338642	-3.668383302	-7.003001407	-2.734038984	-2.482308346	5.404380295	0.018152568

WIPF2	-0.008459474	-0.050448745	-0.020811442	-0.041989271	-0.012351968	0.029637303	8	0.092590835	0.816496581	0.816496581	0.816496581	0.816496581	0.816496581	0.816496581	28	61	0	A_24_P838748	A_24_P838748	A_24_P838748	chr17	40283796	40283856	+	WIPF2	"WAS/WASL interacting protein family, member 2 [Source:HGNC Symbol;Acc:HGNC:30923]"	ENSG00000171475	protein_coding	3'UTR	3'UTR	1	4651	17:40283796:40283856	ENSG00000171475	WIPF2	6.933486525	0.009091321	-0.108661453	-0.648011189	-0.267321764	-0.539349737	-0.158660311	0.380689426	9.961963646	0.915628542	0.531628322	0.794674117	0.601491164	0.877106801	0.711425607	-6.991921006	-5.965446443	-7.0082354	-6.992822795	-6.283313475	-6.992780198	0.160697068	0.920342048

CMPK2	-0.094149001	0.047733722	0.182892224	0.141882723	0.277041225	0.135158502	8	0.112389009	0.816496581	0.816496581	0.816496581	0.816496581	0.816496581	0.816496581	28	64	0	P_127122	P_127122	P_127122	chr2	6848335	6848395	-	CMPK2	"cytidine monophosphate (UMP-CMP) kinase 2, mitochondrial [Source:HGNC Symbol;Acc:HGNC:27015]"	ENSG00000134326	protein_coding	3'UTR;exon	multiple Ref	1	4654	2:6848335:6848395	ENSG00000134326	CMPK2	5.915360807	0.012350298	-1.03758248	0.526056277	2.015589798	1.563638757	3.053172278	1.489533521	9.961963646	0.323993328	0.610359394	0.071612732	0.149084144	0.012237141	0.167308971	-6.440750264	-6.038273187	-5.188979064	-5.954763816	-2.844566012	-5.977900884	3.233575345	0.069359301

MARF1	0.240967827	-0.13772132	-0.026890629	-0.378689147	-0.267858456	0.110830691	8	0.055323004	0.816496581	0.816496581	0.816496581	0.816496581	0.816496581	0.816496581	28	66	0	P_118343	P_118343	P_118343	chr16	15596225	15596285	-	KIAA0430	KIAA0430 [Source:HGNC Symbol;Acc:HGNC:29562]	ENSG00000166783	protein_coding	3'UTR;exon	multiple Ref	1	4656	16:15596225:15596285	ENSG00000166783	MARF1	7.402680712	0.004664541	4.32115906	-2.469689566	-0.482216598	-6.790848627	-4.803375658	1.987472969	9.961963646	0.001523767	0.033206465	0.640071571	4.88E-05	0.000727753	0.075044289	-1.294718163	-3.702784058	-6.92160228	2.170115105	-0.133066608	-5.256182685	16.35706777	0.00035464

MYLK4	0.208352485	0.281718086	-0.021189616	0.073365601	-0.229542101	-0.302907702	8	0.179008611	0.816496581	0.816496581	0.816496581	0.816496581	0.816496581	0.816496581	28	67	0	P_141473	P_141473	P_141473	chr6	2667051	2667111	-	MYLK4	"myosin light chain kinase family, member 4 [Source:HGNC Symbol;Acc:HGNC:27972]"	ENSG00000145949	protein_coding	3'UTR	3'UTR	1	4657	6:2667051:2667111	ENSG00000145949	MYLK4	4.17881012	0.02793983	1.526625233	2.064184346	-0.155259022	0.537559113	-1.681884254	-2.219443368	9.961963646	0.157957196	0.066029254	0.879717899	0.602681787	0.123621453	0.050840622	-5.856628124	-4.327669144	-7.033972436	-6.993847335	-4.978281598	-4.888377844	2.443196715	0.124661438

PDCD6	-0.083946957	-0.076447755	-0.058994011	0.007499202	0.024952946	0.017453744	8	0.203851364	0.816496581	0.816496581	0.816496581	0.816496581	0.816496581	0.816496581	28	68	0	P_139092	P_139092	P_139092	chr5	311114	311174	+	PDCD6	programmed cell death 6 [Source:HGNC Symbol;Acc:HGNC:8765]	ENSG00000249915	protein_coding	exon	non-coding exon	1	4658	5:311114:311174	ENSG00000249915	PDCD6	8.860489728	0.035577919	-0.545080091	-0.496386653	-0.383056896	0.048693438	0.162023195	0.113329757	9.961963646	0.5976892	0.630391077	0.709724422	0.962126018	0.874526705	0.912020462	-6.838618566	-6.053838961	-6.967577184	-7.14831657	-6.282741403	-7.064366554	0.1219518	0.945041034

ENG	0.198521788	-0.002721103	0.11474388	-0.201242891	-0.083777908	0.117464983	8	0.227864207	0.816496581	0.816496581	0.816496581	0.816496581	0.816496581	0.816496581	28	70	0	P_149742	P_149742	P_149742	chr9	127824904	127824964	-	ENG	endoglin [Source:HGNC Symbol;Acc:HGNC:3349]	ENSG00000106991	protein_coding	exon	coding exon	1	4660	9:127824904:127824964	ENSG00000106991	ENG	5.549204761	0.043902959	1.160397846	-0.015905365	0.670699938	-1.176303211	-0.489697907	0.686605303	9.961963646	0.272939973	0.987623928	0.517658623	0.266817456	0.634951609	0.507997705	-6.309539605	-6.181954062	-6.806884158	-6.441352007	-6.170697907	-6.819786253	0.648589112	0.601603775

SCN4B	0.138904135	0.116243081	0.111963567	-0.022661054	-0.026940568	-0.004279515	8	0.117746513	0.816496581	0.816496581	0.816496581	0.816496581	0.816496581	0.816496581	28	73	0	A_23_P303833	A_23_P303833	A_23_P303833	chr11	118133581	118133641	-	SCN4B	"sodium channel, voltage-gated, type IV, beta subunit [Source:HGNC Symbol;Acc:HGNC:10592]"	ENSG00000177098	protein_coding	3'UTR;exon	multiple Ref	1	4663	11:118133581:118133641	ENSG00000177098	SCN4B	5.180597598	0.013340426	1.472909141	1.232616273	1.187237233	-0.240292868	-0.285671908	-0.04537904	9.961963646	0.171654145	0.246016139	0.262672245	0.814975777	0.780981228	0.964701955	-5.928220984	-5.438947045	-6.327540098	-7.118128336	-6.253461529	-7.070253663	0.873290803	0.48701346

SLC1A6	0.398286039	0.085419616	0.250611395	-0.312866423	-0.147674644	0.16519178	8	0.102440214	0.816496581	0.816496581	0.816496581	0.816496581	0.816496581	0.816496581	28	74	0	A_32_P503113	A_32_P503113	A_32_P503113	chr19	15021510	15021666	+	SLC1A6;CCDC105	"solute carrier family 1 (high affinity aspartate/glutamate transporter), member 6 [Source:HGNC Symbol;Acc:HGNC:10944];coiled-coil domain containing 105 [Source:HGNC Symbol;Acc:HGNC:26866]"	ENSG00000105143	protein_coding	"exon,intron;intron"	multiple non-Ref	0	4664	19:15021510:15021666	ENSG00000105143	SLC1A6	4.658223866	0.010633936	4.730354497	1.014509732	2.976455672	-3.715844765	-1.753898825	1.96194594	9.961963646	0.000812196	0.334345483	0.013947008	0.004028751	0.11010055	0.078293395	-0.644388787	-5.666837395	-3.596084939	-2.428909997	-4.877753322	-5.295655791	8.83294455	0.003705878

FGFRL1	-0.321317231	0.106059774	-0.19348999	0.427377006	0.127827242	-0.299549764	8	0.070028936	0.816496581	0.816496581	0.816496581	0.816496581	0.816496581	0.816496581	28	75	0	A_24_P46212	A_24_P46212	A_24_P46212	chr4	1024940	1025000	+	FGFRL1	fibroblast growth factor receptor-like 1 [Source:HGNC Symbol;Acc:HGNC:3693]	ENSG00000127418	protein_coding	exon	coding exon	1	4665	4:1024940:1025000	ENSG00000127418	FGFRL1	8.138283581	0.006144905	-5.020212009	1.657061936	-3.023058446	6.677273945	1.997153563	-4.680120382	9.961963646	0.000527676	0.128618528	0.012881329	5.62E-05	0.073845667	0.000876299	-0.197624216	-4.910625553	-3.516395883	2.023481498	-4.52350623	-0.782863892	17.92756695	0.000243388

DNM1P35	0.191079545	-0.016723498	0.095272628	-0.207803043	-0.095806918	0.111996125	8	0.036619378	0.816496581	0.816496581	0.816496581	0.816496581	0.816496581	0.816496581	28	77	0	A_23_P106472	A_23_P106472	A_23_P106472	chr15	75738633	75738693	+	DNM1P35;RP11-24M17.4	DNM1 pseudogene 35 [Source:HGNC Symbol;Acc:HGNC:35182];	ENSG00000246877	antisense;processed_transcript	intron;promoter	multiple non-Ref	0	4667	15:75738633:75738693	ENSG00000246877	DNM1P35	16.24002375	0.003283563	4.084013287	-0.357437459	2.036296852	-4.441450746	-2.047716434	2.393734311	9.961963646	0.002217649	0.728220785	0.069180901	0.001263359	0.067873462	0.037805698	-1.681342505	-6.115181308	-5.156627859	-1.229658431	-4.447429655	-4.603202393	8.433837035	0.004354377

EFHC2	0.671280843	-0.081115989	0.264468876	-0.752396832	-0.406811967	0.345584865	8	0.093448276	0.816496581	0.816496581	0.816496581	0.816496581	0.816496581	0.816496581	28	78	0	P_270154	P_270154	P_270154	chrX	44312567	44312627	-	EFHC2	EF-hand domain (C-terminal) containing 2 [Source:HGNC Symbol;Acc:HGNC:26233]	ENSG00000183690	protein_coding	exon	coding exon	1	4668	X:44312567:44312627	ENSG00000183690	EFHC2	4.905467493	0.009219422	8.562449729	-1.034666171	3.373403963	-9.5971159	-5.189045767	4.408070133	9.961963646	6.63E-06	0.325288345	0.007118316	2.38E-06	0.000412753	0.001330532	4.319699542	-5.647225492	-2.917434133	5.305873919	0.406608582	-1.216065561	37.3713076	9.90E-06

CBFA2T3	-0.00134732	0.003662126	0.227407684	0.005009445	0.228755004	0.223745559	8	0.147203585	0.816496581	0.816496581	0.816496581	0.816496581	0.816496581	0.816496581	28	79	0	P_119826	P_119826	P_119826	chr16	88898077	88898137	-	CBFA2T3	"core-binding factor, runt domain, alpha subunit 2; translocated to, 3 [Source:HGNC Symbol;Acc:HGNC:1537]"	ENSG00000129993	protein_coding	exon	coding exon	1	4669	16:88898077:88898137	ENSG00000129993	CBFA2T3	4.84819022	0.019607989	-0.01178419	0.032030398	1.988997528	0.043814589	2.000781718	1.956967129	9.961963646	0.99083045	0.975080463	0.074854314	0.96591799	0.073401108	0.078942317	-6.99828454	-6.181546603	-5.230322699	-7.148563336	-4.518072346	-5.303328784	1.965340587	0.183460346

C2orf76	-0.249418844	0.002642333	-0.215863883	0.252061177	0.033554961	-0.218506216	8	0.076495141	0.816496581	0.816496581	0.816496581	0.816496581	0.816496581	0.816496581	28	82	0	A_23_P39542	A_23_P39542	A_23_P39542	chr2	119339848	119339908	-	C2orf76	chromosome 2 open reading frame 76 [Source:HGNC Symbol;Acc:HGNC:27017]	ENSG00000186132	protein_coding	exon	coding exon	1	4672	2:119339848:119339908	ENSG00000186132	C2orf76	10.12107879	0.006905763	-3.675945484	0.038942823	-3.181411045	3.714888307	0.494534439	-3.220353868	9.961963646	0.004302239	0.969705346	0.00984151	0.004035091	0.631652338	0.009213486	-2.360833783	-6.181288009	-3.2454616	-2.430525652	-6.168228072	-3.201121122	8.008517743	0.005201313

EEF1AKMT1	0.148873339	0.072598503	0.11392785	-0.076274836	-0.034945489	0.041329347	8	0.058834013	0.816496581	0.816496581	0.816496581	0.816496581	0.816496581	0.816496581	28	85	0	P_218231	P_218231	P_218231	chr13	20731867	20731927	-	N6AMT2	N-6 adenine-specific DNA methyltransferase 2 (putative) [Source:HGNC Symbol;Acc:HGNC:27351]	ENSG00000150456	protein_coding	exon	coding exon	1	4675	13:20731867:20731927	ENSG00000150456	EEF1AKMT1	10.18438566	0.00498641	2.582073328	1.259155335	1.975975448	-1.322917993	-0.60609788	0.716820113	9.961963646	0.027396334	0.236677953	0.076491683	0.215424439	0.558006722	0.489949617	-4.219937538	-5.408980995	-5.250483348	-6.268351843	-6.104927532	-6.797719934	2.464725256	0.122582531

WNK1	-0.028048536	-0.062469005	-0.115371286	-0.034420469	-0.08732275	-0.052902281	8	0.08004156	0.816496581	0.816496581	0.816496581	0.816496581	0.816496581	0.816496581	28	86	0	P_214031	P_214031	P_214031	chr12	896669	896729	+	WNK1	WNK lysine deficient protein kinase 1 [Source:HGNC Symbol;Acc:HGNC:14540]	ENSG00000060237	protein_coding	exon	coding exon	1	4676	12:896669:896729	ENSG00000060237	WNK1	8.119311973	0.007351574	-0.400650467	-0.8923188	-1.647984752	-0.491668332	-1.247334284	-0.755665952	9.961963646	0.69713521	0.393265856	0.130490699	0.633606453	0.240801081	0.467340981	-6.91146931	-5.778932422	-5.736839048	-7.019002795	-5.529689903	-6.768096349	1.006869829	0.429709196

WIPF3	0.114331423	0.174323554	0.123777941	0.059992131	0.009446518	-0.050545613	8	0.217072685	0.816496581	0.816496581	0.816496581	0.816496581	0.816496581	0.816496581	28	88	0	A_32_P3914	A_32_P3914	A_32_P3914	chr7	29916221	29916281	+	WIPF3	"WAS/WASL interacting protein family, member 3 [Source:HGNC Symbol;Acc:HGNC:22004]"	ENSG00000122574	protein_coding	3'UTR	3'UTR	1	4678	7:29916221:29916281	ENSG00000122574	WIPF3	4.384633444	0.040047055	0.699722667	1.066882045	0.757536545	0.367159378	0.057813878	-0.3093455	9.961963646	0.5001131	0.311197662	0.46626936	0.72117952	0.955040103	0.763432011	-6.73759307	-5.615244374	-6.742487545	-7.076389498	-6.294907764	-7.01935147	0.405925636	0.75211165

PPP1R27	0.074066727	0.093657755	0.151985938	0.019591028	0.077919212	0.058328183	8	0.256576586	0.816496581	0.816496581	0.816496581	0.816496581	0.816496581	0.816496581	28	89	0	P_122934	P_122934	P_122934	chr17	81834557	81834617	-	PPP1R27	"protein phosphatase 1, regulatory subunit 27 [Source:HGNC Symbol;Acc:HGNC:16813]"	ENSG00000182676	protein_coding	exon	coding exon	1	4679	17:81834557:81834617	ENSG00000182676	PPP1R27	4.665856162	0.055073004	0.386544411	0.488787385	0.793194429	0.102242974	0.406650018	0.304407044	9.961963646	0.707221458	0.635573669	0.446144878	0.920592578	0.692863908	0.767081681	-6.917435218	-6.057687913	-6.714022157	-7.143899341	-6.20947627	-7.020991693	0.2143281	0.884210139

FOS	0.744805529	-0.629416452	0.11523863	-1.37422198	-0.629566898	0.744655082	8	0.13013365	0.816496581	0.816496581	0.816496581	0.816496581	0.816496581	0.816496581	28	90	0	P_115406	P_115406	P_115406	chr14	75283361	75283421	+	FOS	FBJ murine osteosarcoma viral oncogene homolog [Source:HGNC Symbol;Acc:HGNC:3796]	ENSG00000170345	protein_coding	3'ext	3'extended sense	0	4680	14:75283361:75283421	ENSG00000170345	FOS	7.491654285	0.015806225	7.255618502	-6.131541026	1.122611884	-13.38715953	-6.133006618	7.25415291	9.961963646	2.80E-05	0.000112784	0.287929708	1.08E-07	0.000112567	2.80E-05	2.844302669	1.582084902	-6.399357394	8.420357423	1.624534982	2.800435199	60.15876625	1.10E-06

SH3D19	0.023448705	-0.056860491	0.041051578	-0.080309196	0.017602872	0.097912068	8	0.089093931	0.816496581	0.816496581	0.816496581	0.816496581	0.816496581	0.816496581	28	91	0	A_24_P366656	A_24_P366656	A_24_P366656	chr4	151122149	151122209	-	SH3D19	SH3 domain containing 19 [Source:HGNC Symbol;Acc:HGNC:30418]	ENSG00000109686	protein_coding	exon	coding exon	1	4681	4:151122149:151122209	ENSG00000109686	SH3D19	7.267977235	0.008581113	0.310022128	-0.751769022	0.542754802	-1.061791149	0.232732674	1.294523824	9.961963646	0.762932433	0.469578512	0.599230456	0.313392935	0.820680866	0.224685358	-6.946165261	-5.892637572	-6.888595952	-6.565731261	-6.267956518	-6.225703941	0.635535005	0.609004079

GPN3	-0.165021525	0.040748982	-0.156759751	0.205770506	0.008261773	-0.197508733	8	0.043806527	0.816496581	0.816496581	0.816496581	0.816496581	0.816496581	0.816496581	28	92	0	P_112765	P_112765	P_112765	chr12	110452600	110452660	-	GPN3	GPN-loop GTPase 3 [Source:HGNC Symbol;Acc:HGNC:30186]	ENSG00000111231	protein_coding	3'UTR	3'UTR	1	4682	12:110452600:110452660	ENSG00000111231	GPN3	11.28715481	0.003747755	-3.301418828	0.815223681	-3.136133879	4.116642509	0.165284948	-3.951357561	9.961963646	0.008034743	0.434001808	0.010627132	0.002105109	0.872025673	0.002744527	-2.996055363	-5.843393383	-3.322928077	-1.75934373	-6.282175135	-1.964004497	8.997763812	0.003472249

ABCG1	0.600662858	0.092391606	0.586769124	-0.508271252	-0.013893734	0.494377518	8	0.080051207	0.816496581	0.816496581	0.816496581	0.816496581	0.816496581	0.816496581	28	94	0	A_32_P147063	A_32_P147063	A_32_P147063	chr21	42300491	42300551	+	ABCG1;AP001623.1	"ATP-binding cassette, sub-family G (WHITE), member 1 [Source:HGNC Symbol;Acc:HGNC:73];"	ENSG00000160179	protein_coding;miRNA	3'ext;promoter	multiple non-Ref	0	4684	21:42300491:42300551	ENSG00000160179	ABCG1	6.628390561	0.007352815	8.579255445	1.319627442	8.38081152	-7.259628003	-0.198443926	7.061184078	9.961963646	6.51E-06	0.216481159	8.01E-06	2.78E-05	0.846688997	3.52E-05	4.337404692	-5.339022251	4.105454927	2.757538022	-6.27578223	2.563371683	41.36415694	6.23E-06

DPP8	0.05565266	-0.05999148	0.013172993	-0.11564414	-0.042479667	0.073164473	8	0.040165339	0.816496581	0.816496581	0.816496581	0.816496581	0.816496581	0.816496581	28	95	0	A_23_P205929	A_23_P205929	A_23_P205929	chr15	65446813	65446873	-	DPP8	dipeptidyl-peptidase 8 [Source:HGNC Symbol;Acc:HGNC:16490]	ENSG00000074603	protein_coding	3'UTR	3'UTR	1	4685	15:65446813:65446873	ENSG00000074603	DPP8	9.186473488	0.003502215	1.151755089	-1.241548774	0.272620611	-2.393303863	-0.879134478	1.514169385	9.961963646	0.276313157	0.242840252	0.790712356	0.037833468	0.400037234	0.161045476	-6.319143887	-5.428912277	-7.006684373	-4.678935669	-5.901272718	-5.94499131	1.955965812	0.184903739

SERPINA1	-0.02875599	-0.202595681	-0.052574769	-0.173839692	-0.023818779	0.150020912	8	0.173206168	0.816496581	0.816496581	0.816496581	0.816496581	0.816496581	0.816496581	28	96	0	A_23_P218111	A_23_P218111	A_23_P218111	chr14	94378453	94378513	-	SERPINA1	"serpin peptidase inhibitor, clade A (alpha-1 antiproteinase, antitrypsin), member 1 [Source:HGNC Symbol;Acc:HGNC:8941]"	ENSG00000197249	protein_coding	3'UTR;exon	multiple Ref	1	4686	14:94378453:94378513	ENSG00000197249	SERPINA1	7.747855763	0.026298622	-0.217173811	-1.530063008	-0.397060334	-1.312889196	-0.179886523	1.133002673	9.961963646	0.83245616	0.15711401	0.699696492	0.218658515	0.860848078	0.283744879	-6.972684267	-5.078824267	-6.961701737	-6.280648533	-6.279502673	-6.412027925	0.930941074	0.461351768

GCK	-0.072293307	0.005495272	0.015340178	0.077788579	0.087633485	0.009844906	8	0.200863692	0.816496581	0.816496581	0.816496581	0.816496581	0.816496581	0.816496581	28	97	0	A_23_P250206	A_23_P250206	A_23_P250206	chr7	44145118	44145178	-	GCK	glucokinase (hexokinase 4) [Source:HGNC Symbol;Acc:HGNC:4195]	ENSG00000106633	protein_coding	"3'UTR,exon;exon"	multiple Ref	1	4687	7:44145118:44145178	ENSG00000106633	GCK	4.563615021	0.034606901	-0.475951137	0.03617874	0.100993789	0.512129876	0.576944926	0.064815049	9.961963646	0.644374767	0.971854528	0.921559119	0.619721329	0.576780915	0.949603459	-6.876140781	-6.181397456	-7.041553288	-7.008049362	-6.122628376	-7.069083996	0.139563597	0.934014334

LRP6	0.15997512	-0.157226106	0.06086058	-0.317201226	-0.099114541	0.218086685	8	0.06192401	0.816496581	0.816496581	0.816496581	0.816496581	0.816496581	0.816496581	28	99	0	P_214404	P_214404	P_214404	chr12	12150836	12150896	-	LRP6	low density lipoprotein receptor-related protein 6 [Source:HGNC Symbol;Acc:HGNC:6698]	ENSG00000070018	protein_coding	exon	coding exon	1	4689	12:12150836:12150896	ENSG00000070018	LRP6	6.694963345	0.005286063	2.694833263	-2.648525219	1.025216384	-5.343358482	-1.669616879	3.673741603	9.961963646	0.022582936	0.024448156	0.32951135	0.000330931	0.126068793	0.004317898	-4.029905166	-3.419509724	-6.501637467	0.167849202	-4.995182342	-2.429397804	10.02722642	0.002353992

CSTF3	0.136674213	0.088568294	0.172450602	-0.04810592	0.035776389	0.083882308	8	0.04429492	0.816496581	0.816496581	0.816496581	0.816496581	0.816496581	0.816496581	28	100	0	P_211449	P_211449	P_211449	chr11	33096834	33096894	-	CSTF3	"cleavage stimulation factor, 3' pre-RNA, subunit 3, 77kDa [Source:HGNC Symbol;Acc:HGNC:2485]"	ENSG00000176102	protein_coding	exon	coding exon	1	4690	11:33096834:33096894	ENSG00000176102	CSTF3	8.629827182	0.003782309	2.721784315	1.763784015	3.434249467	-0.9580003	0.712465152	1.670465452	9.961963646	0.021563638	0.108353455	0.006428041	0.360734683	0.492526152	0.12589811	-3.984299422	-4.763684959	-2.813750856	-6.669640408	-6.033570695	-5.728704947	4.421489884	0.031927037

TTC3P1	0.32323344	-0.228574867	0.09170631	-0.551808307	-0.23152713	0.320281177	8	0.126947099	0.816496581	0.816496581	0.816496581	0.816496581	0.816496581	0.816496581	28	101	0	A_32_P165330	A_32_P165330	A_32_P165330	chrX	75745008	75745068	-	TTC3P1	tetratricopeptide repeat domain 3 pseudogene 1 [Source:HGNC Symbol;Acc:HGNC:23318]	ENSG00000215105	processed_pseudogene	exon	non-coding exon	1	4691	X:75745008:75745068	ENSG00000215105	TTC3P1	11.1106283	0.015148362	3.216466801	-2.274527881	0.912561218	-5.490994682	-2.303905584	3.187089098	9.961963646	0.009274281	0.046308841	0.383026539	0.000268728	0.044054728	0.009747275	-3.141029295	-4.007399501	-6.610482681	0.385661054	-4.051998218	-3.258057594	10.32807403	0.002112723

BNC2	0.050600997	0.01028791	-0.007260701	-0.040313087	-0.057861698	-0.017548611	8	0.198114177	0.816496581	0.816496581	0.816496581	0.816496581	0.816496581	0.816496581	28	102	0	P_314277	P_314277	P_314277	chr9	16677205	16677265	+	BNC2	basonuclin 2 [Source:HGNC Symbol;Acc:HGNC:30988]	ENSG00000173068	protein_coding	intron	intron antisense	0	4692	9:16677205:16677265	ENSG00000173068	BNC2	3.674577321	0.033725953	0.33746022	0.068610512	-0.048421927	-0.268849709	-0.385882147	-0.117032439	9.961963646	0.742772559	0.94665727	0.962337019	0.793531092	0.707696479	0.909160189	-6.936572604	-6.179606426	-7.045840299	-7.11022903	-6.21809024	-7.063901205	0.059302212	0.980001516

KRTAP4-5	-0.206387431	-0.116200316	0.19783141	0.090187115	0.404218841	0.314031726	8	0.224758233	0.816496581	0.816496581	0.816496581	0.816496581	0.816496581	0.816496581	28	104	0	A_23_P27197	A_23_P27197	A_23_P27197	chr17	41148993	41149053	-	KRTAP4-5	keratin associated protein 4-5 [Source:HGNC Symbol;Acc:HGNC:18899]	ENSG00000198271	protein_coding	3'UTR	3'UTR	1	4694	17:41148993:41149053	ENSG00000198271	KRTAP4-5	4.713993959	0.042773998	-1.222190638	-0.688118157	1.171523362	0.534072481	2.393714	1.859641519	9.961963646	0.249765358	0.507084521	0.268645816	0.60500364	0.037807008	0.092682854	-6.239303995	-5.938459306	-6.345283379	-6.995833056	-3.910203593	-5.451533091	2.135515041	0.159409172

TMEM182	0.15279517	-0.052180691	-0.106000681	-0.204975861	-0.258795851	-0.05381999	8	0.136471179	0.816496581	0.816496581	0.816496581	0.816496581	0.816496581	0.816496581	28	106	0	A_23_P328000	A_23_P328000	A_23_P328000	chr2	102815562	102815622	+	TMEM182	transmembrane protein 182 [Source:HGNC Symbol;Acc:HGNC:26391]	ENSG00000170417	protein_coding	3'UTR;exon	multiple Ref	1	4696	2:102815562:102815622	ENSG00000170417	TMEM182	5.759099578	0.017163079	1.428425706	-0.487818036	-0.99096128	-1.916243742	-2.419386987	-0.503143245	9.961963646	0.183762955	0.636236247	0.3451632	0.084442483	0.03618638	0.625800765	-5.986263721	-6.058174819	-6.535836138	-5.44193432	-3.869431056	-6.934820305	2.173157882	0.154598435

CLMAT3	0.074141147	0.089335975	-0.022677162	0.015194828	-0.096818309	-0.112013137	8	0.289126193	0.816496581	0.816496581	0.816496581	0.816496581	0.816496581	0.816496581	28	108	0	P_312872	P_312872	P_312872	chr5	151715486	151715546	-	CTB-113P19.1	uncharacterized LOC101927096 [Source:EntrezGene;Acc:101927096]	ENSG00000249035	antisense	intron	intron antisense	0	4698	5:151715486:151715546	ENSG00000249035	CLMAT3	3.66547361	0.069337188	0.344843319	0.415517096	-0.105475409	0.070673778	-0.450318728	-0.520992506	9.961963646	0.737381814	0.686571772	0.918092159	0.945056032	0.662122021	0.613755086	-6.93385568	-6.091881234	-7.041048507	-7.146882273	-6.189944731	-6.925091841	0.130318561	0.939849899

WDR35	0.038555813	0.041994563	-0.047284899	0.00343875	-0.085840712	-0.089279462	8	0.05657339	0.816496581	0.816496581	0.816496581	0.816496581	0.816496581	0.816496581	28	109	0	P_233809	P_233809	P_233809	chr2	19975539	19975599	-	WDR35	WD repeat domain 35 [Source:HGNC Symbol;Acc:HGNC:29250]	ENSG00000118965	protein_coding	exon	coding exon	1	4699	2:19975539:19975599	ENSG00000118965	WDR35	6.996387694	0.004776899	0.683222991	0.744158883	-0.837905553	0.060935892	-1.521128544	-1.582064436	9.961963646	0.510042931	0.473967784	0.421731886	0.952615438	0.159313597	0.144832958	-6.749469764	-5.898306571	-6.676708021	-7.147582829	-5.194143432	-5.852582671	1.090520821	0.397522564

MUCL1	0.603182495	0.422619366	1.082801059	-0.180563129	0.479618564	0.660181693	8	0.119839366	0.816496581	0.816496581	0.816496581	0.816496581	0.816496581	0.816496581	28	111	0	A_14_P200640	A_14_P200640	A_14_P200640	chr12	54858254	54858311	+	MUCL1	mucin-like 1 [Source:HGNC Symbol;Acc:HGNC:30588]	ENSG00000172551	protein_coding	3'UTR	3'UTR	1	4701	12:54858254:54858311	ENSG00000172551	MUCL1	5.79534232	0.01373973	6.302389695	4.415764646	11.31371399	-1.886625049	5.011324299	6.897949348	9.961963646	9.03E-05	0.00131472	5.25E-07	0.088665444	0.000534604	4.29E-05	1.63302823	-0.670525639	6.859106295	-5.487029543	0.160938121	2.359084667	43.91227469	4.74E-06

TAMM41	-0.062143382	0.031811042	-0.048387098	0.093954424	0.013756283	-0.080198141	8	0.092377992	0.816496581	0.816496581	0.816496581	0.816496581	0.816496581	0.816496581	28	112	0	P_134067	P_134067	P_134067	chr3	11790504	11790564	-	TAMM41	"TAM41, mitochondrial translocator assembly and maintenance protein, homolog (S. cerevisiae) [Source:HGNC Symbol;Acc:HGNC:25187]"	ENSG00000144559	protein_coding	3'UTR;exon	multiple Ref	1	4702	3:11790504:11790564	ENSG00000144559	TAMM41	9.185104068	0.009059705	-0.799619698	0.409323332	-0.622612993	1.20894303	0.177006705	-1.031936325	9.961963646	0.442580158	0.690964297	0.547525328	0.254595788	0.86305013	0.326504085	-6.660277002	-6.094527379	-6.839467461	-6.404128491	-6.280047539	-6.518870517	0.625391899	0.614811257

LETMD1	0.013199391	0.005208812	-0.079077785	-0.007990579	-0.092277175	-0.084286596	8	0.036930005	0.816496581	0.816496581	0.816496581	0.816496581	0.816496581	0.816496581	28	113	0	P_111557	P_111557	P_111557	chr12	51059725	51059785	+	LETMD1	LETM1 domain containing 1 [Source:HGNC Symbol;Acc:HGNC:24241]	ENSG00000050426	protein_coding	3'UTR;exon	multiple Ref	1	4703	12:51059725:51059785	ENSG00000050426	LETMD1	6.923916076	0.00330191	0.281330555	0.111020113	-1.685456337	-0.170310441	-1.966786891	-1.79647645	9.961963646	0.784213861	0.913805303	0.122916913	0.868175155	0.077667246	0.102754424	-6.955342542	-6.175593972	-5.683610145	-7.133774033	-4.568828933	-5.545776629	1.676136561	0.234715188

ZNF41	0.044368179	-0.018373963	0.10206439	-0.062742142	0.057696211	0.120438353	8	0.128711096	0.816496581	0.816496581	0.816496581	0.816496581	0.816496581	0.816496581	28	115	0	P_150801	P_150801	P_150801	chrX	47447429	47447489	-	ZNF41	zinc finger protein 41 [Source:HGNC Symbol;Acc:HGNC:13107]	ENSG00000147124	protein_coding	exon	coding exon	1	4705	X:47447429:47447489	ENSG00000147124	ZNF41	5.561281614	0.015510524	0.436318879	-0.180690462	1.003706282	-0.617009341	0.567387404	1.184396745	9.961963646	0.671909635	0.860233568	0.339276691	0.551069065	0.583010184	0.263744138	-6.895462796	-6.164905094	-6.523222642	-6.945323668	-6.128254361	-6.354747698	0.555979025	0.655881938

KAT8	-0.102846076	0.103601183	-0.055291233	0.206447259	0.047554843	-0.158892416	8	0.085385264	0.816496581	0.816496581	0.816496581	0.816496581	0.816496581	0.816496581	28	118	0	P_226128	P_226128	P_226128	chr16	31128079	31128139	+	KAT8	K(lysine) acetyltransferase 8 [Source:HGNC Symbol;Acc:HGNC:17933]	ENSG00000103510	protein_coding	exon	coding exon	1	4708	16:31128079:31128139	ENSG00000103510	KAT8	9.089145051	0.008061468	-1.402898624	1.413198846	-0.754214428	2.81609747	0.648684195	-2.167413275	9.961963646	0.191036603	0.188072676	0.468173614	0.018346607	0.531210782	0.055512485	-6.019044088	-5.226394963	-6.745080355	-3.966250024	-6.077622754	-4.972174129	2.93049523	0.086210382

TSEN15	0.009800823	0.166228154	0.233710598	0.156427332	0.223909776	0.067482444	8	0.119490184	0.816496581	0.816496581	0.816496581	0.816496581	0.816496581	0.816496581	28	119	0	P_104255	P_104255	P_104255	chr1	184054375	184054435	+	TSEN15	TSEN15 tRNA splicing endonuclease subunit [Source:HGNC Symbol;Acc:HGNC:16791]	ENSG00000198860	protein_coding	exon	coding exon	1	4709	1:184054375:184054435	ENSG00000198860	TSEN15	8.151803308	0.013672619	0.102655516	1.741102541	2.447925371	1.638447024	2.345269855	0.706822831	9.961963646	0.920273409	0.112400289	0.034464899	0.132484121	0.041061041	0.495876796	-6.992612793	-4.795309802	-4.490226958	-5.850745353	-3.986859445	-6.805116227	2.953118782	0.084796278

KIAA1328	0.30474737	0.080637855	0.217568976	-0.224109515	-0.087178394	0.136931121	8	0.060142108	0.816496581	0.816496581	0.816496581	0.816496581	0.816496581	0.816496581	28	120	0	P_231249	P_231249	P_231249	chr18	37067485	37067545	+	KIAA1328	KIAA1328 [Source:HGNC Symbol;Acc:HGNC:29248]	ENSG00000150477	protein_coding	3'UTR;exon	multiple Ref	1	4710	18:37067485:37067545	ENSG00000150477	KIAA1328	5.833638393	0.005111391	5.220547416	1.38138598	3.727117164	-3.839161436	-1.493430252	2.345731184	9.961963646	0.000394441	0.197355105	0.003954814	0.003292452	0.166304376	0.041028798	0.104134811	-5.265262867	-2.317790356	-2.221253599	-5.230034755	-4.682369102	10.92094902	0.001718522

NPC2	0.135376574	0.0207586	0.117069505	-0.114617973	-0.018307069	0.096310904	8	0.083562748	0.816496581	0.816496581	0.816496581	0.816496581	0.816496581	0.816496581	28	121	0	A_23_P48717	A_23_P48717	A_23_P48717	chr14	74480050	74480110	-	NPC2	"Niemann-Pick disease, type C2 [Source:HGNC Symbol;Acc:HGNC:14537]"	ENSG00000119655	protein_coding	3'UTR	3'UTR	1	4711	14:74480050:74480110	ENSG00000119655	NPC2	10.4944755	0.007814199	1.875628873	0.28760833	1.621986267	-1.588020544	-0.253642607	1.334377937	9.961963646	0.09028267	0.779540747	0.135988587	0.143481868	0.804929636	0.211777691	-5.356555994	-6.138668126	-5.773370708	-5.921176781	-6.262580237	-6.176987273	1.766374995	0.217125321

MAP1LC3C	-0.149866081	-0.248383633	-0.06159331	-0.098517552	0.088272771	0.186790323	8	0.201094651	0.816496581	0.816496581	0.816496581	0.816496581	0.816496581	0.816496581	28	122	0	P_105224	P_105224	P_105224	chr1	241995507	241995567	-	MAP1LC3C	microtubule-associated protein 1 light chain 3 gamma [Source:HGNC Symbol;Acc:HGNC:13353]	ENSG00000197769	protein_coding	3'UTR	3'UTR	1	4712	1:241995507:241995567	ENSG00000197769	MAP1LC3C	4.216479476	0.034681453	-0.985599188	-1.633503095	-0.40507042	-0.647903907	0.580528767	1.228432675	9.961963646	0.347662166	0.133528102	0.693987395	0.531694898	0.574454471	0.247515118	-6.492748911	-4.942399325	-6.958249079	-6.924783115	-6.120496157	-6.304155683	1.011610021	0.427812091

PRIM1	-0.517042748	-0.005881363	-0.27789448	0.511161385	0.239148268	-0.272013117	8	0.063855851	0.816496581	0.816496581	0.816496581	0.816496581	0.816496581	0.816496581	28	124	0	P_215767	P_215767	P_215767	chr12	56746927	56746987	-	PRIM1	"primase, DNA, polypeptide 1 (49kDa) [Source:HGNC Symbol;Acc:HGNC:9369]"	ENSG00000198056	protein_coding	exon	coding exon	1	4714	12:56746927:56746987	ENSG00000198056	PRIM1	11.21397167	0.005481195	-8.553314948	-0.097293987	-4.597142149	8.456020961	3.956172799	-4.499848163	9.961963646	6.69E-06	0.924422572	0.000994238	7.40E-06	0.002723265	0.001154331	4.31006312	-6.177099623	-0.893716644	4.135506364	-1.400045076	-1.068830829	33.61776764	1.60E-05

KRTAP12-2	0.249896805	0.064601113	-0.08524218	-0.185295692	-0.335138985	-0.149843293	8	0.215773736	0.816496581	0.816496581	0.816496581	0.816496581	0.816496581	0.816496581	28	125	0	P_132434	P_132434	P_132434	chr21	44666594	44666654	-	KRTAP12-2	keratin associated protein 12-2 [Source:HGNC Symbol;Acc:HGNC:20530]	ENSG00000221864	protein_coding	exon	coding exon	1	4715	21:44666594:44666654	ENSG00000221864	KRTAP12-2	4.889393399	0.039595542	1.538095104	0.397614751	-0.524658887	-1.140480353	-2.062753992	-0.922273638	9.961963646	0.155159314	0.699300699	0.6112955	0.280762846	0.066187529	0.378181211	-5.84113483	-6.099424703	-6.898849038	-6.481317246	-4.424663503	-6.625608081	1.534223186	0.265798412

LUZP4	0.098482829	-0.192498514	0.12533947	-0.290981343	0.026856641	0.317837984	8	0.174258217	0.816496581	0.816496581	0.816496581	0.816496581	0.816496581	0.816496581	28	126	0	P_151529	P_151529	P_151529	chrX	115307131	115307191	+	LUZP4	leucine zipper protein 4 [Source:HGNC Symbol;Acc:HGNC:24971]	ENSG00000102021	protein_coding	3'UTR	3'UTR	1	4716	X:115307131:115307191	ENSG00000102021	LUZP4	4.078194222	0.026592179	0.739654977	-1.445759491	0.941361698	-2.185414468	0.201706721	2.387121188	9.961963646	0.476577733	0.178960012	0.368786192	0.053851201	0.844205275	0.038234542	-6.707791855	-5.186020837	-6.58365673	-5.018813523	-6.275090666	-4.614134306	2.3397569	0.135246877

CYP2C9	0.279755216	-0.009490675	0.02319641	-0.289245891	-0.256558805	0.032687086	8	0.084918416	0.816496581	0.816496581	0.816496581	0.816496581	0.816496581	0.816496581	28	127	0	P_106667	P_106667	P_106667	chr10	94988929	94988989	+	CYP2C9	"cytochrome P450, family 2, subfamily C, polypeptide 9 [Source:HGNC Symbol;Acc:HGNC:2623]"	ENSG00000138109	protein_coding	exon	coding exon	1	4717	10:94988929:94988989	ENSG00000138109	CYP2C9	7.702609746	0.007997621	3.831275875	-0.129975756	0.317677179	-3.961251631	-3.513598696	0.447652935	9.961963646	0.003335038	0.89917262	0.757288452	0.002701027	0.005630398	0.663980673	-2.100298025	-6.173189404	-6.992287162	-2.017042854	-2.09967717	-6.962997274	7.172295125	0.007521173

APPBP2	-0.007651886	0.011247348	-0.133870702	0.018899234	-0.126218816	-0.14511805	8	0.064219324	0.816496581	0.816496581	0.816496581	0.816496581	0.816496581	0.816496581	28	128	0	P_229472	P_229472	P_229472	chr17	60451906	60451966	-	APPBP2	amyloid beta precursor protein (cytoplasmic tail) binding protein 2 [Source:HGNC Symbol;Acc:HGNC:622]	ENSG00000062725	protein_coding	3'UTR;exon	multiple Ref	1	4718	17:60451906:60451966	ENSG00000062725	APPBP2	8.13909179	0.005518578	-0.126153851	0.185430912	-2.20707738	0.311584764	-2.080923528	-2.392508292	9.961963646	0.902119913	0.856612101	0.051915387	0.761779122	0.064203534	0.037884845	-6.989682725	-6.163993342	-4.885034971	-7.096779083	-4.397073016	-4.605229681	2.512152283	0.118144896

PDE6C	-0.087924394	-0.139640076	-0.059563599	-0.051715682	0.028360795	0.080076477	8	0.1255081	0.816496581	0.816496581	0.816496581	0.816496581	0.816496581	0.816496581	28	129	0	P_209188	P_209188	P_209188	chr10	93634847	93634907	+	PDE6C	"phosphodiesterase 6C, cGMP-specific, cone, alpha prime [Source:HGNC Symbol;Acc:HGNC:8787]"	ENSG00000095464	protein_coding	exon	coding exon	1	4719	10:93634847:93634907	ENSG00000095464	PDE6C	4.143444938	0.014856626	-0.883476319	-1.403122558	-0.59850318	-0.519646239	0.284973139	0.804619378	9.961963646	0.397798387	0.190971745	0.562864406	0.614659493	0.781501247	0.439819415	-6.588423224	-5.238768546	-6.854960976	-7.003919327	-6.253671842	-6.728790549	0.684357271	0.581751173

RAB11A	-0.10865807	-0.016615956	-0.116806115	0.092042114	-0.008148044	-0.100190158	8	0.079315662	0.816496581	0.816496581	0.816496581	0.816496581	0.816496581	0.816496581	28	130	0	P_116843	P_116843	P_116843	chr15	65887864	65887924	+	RAB11A	"RAB11A, member RAS oncogene family [Source:HGNC Symbol;Acc:HGNC:9760]"	ENSG00000103769	protein_coding	3'UTR;exon	multiple Ref	1	4720	15:65887864:65887924	ENSG00000103769	RAB11A	10.65681774	0.007258679	-1.561992019	-0.238859307	-1.679122574	1.323132712	-0.117130555	-1.440263268	9.961963646	0.149469387	0.816056714	0.124168646	0.215355634	0.909084414	0.180471141	-5.808628416	-6.152099684	-5.692654193	-6.268087852	-6.28939319	-6.042683047	1.525847246	0.267775389

CAP1	0.033278399	0.073397344	0.042921717	0.040118945	0.009643318	-0.030475627	8	0.084469091	0.816496581	0.816496581	0.816496581	0.816496581	0.816496581	0.816496581	28	131	0	P_201292	P_201292	P_201292	chr1	40067637	40067697	+	CAP1	"CAP, adenylate cyclase-associated protein 1 (yeast) [Source:HGNC Symbol;Acc:HGNC:20040]"	ENSG00000131236	protein_coding	exon	coding exon	1	4721	1:40067637:40067697	ENSG00000131236	CAP1	8.289535223	0.0079365	0.457502578	1.009047164	0.59007635	0.551544586	0.132573772	-0.418970814	9.961963646	0.657125278	0.336832151	0.56828184	0.59341536	0.897170055	0.68412774	-6.88533527	-5.672099018	-6.860242114	-6.985759939	-6.287345432	-6.976323283	0.345498111	0.7932292

MTUS1	-0.117256335	-0.20250126	-0.027493944	-0.085244925	0.089762391	0.175007316	8	0.061489965	0.816496581	0.816496581	0.816496581	0.816496581	0.816496581	0.816496581	28	132	0	P_146599	P_146599	P_146599	chr8	17723734	17723794	-	MTUS1	microtubule associated tumor suppressor 1 [Source:HGNC Symbol;Acc:HGNC:29789]	ENSG00000129422	protein_coding	3'UTR;exon	multiple Ref	1	4722	8:17723734:17723794	ENSG00000129422	MTUS1	7.229078679	0.005243046	-1.983307799	-3.425165285	-0.465040581	-1.441857486	1.518267218	2.960124704	9.961963646	0.075565627	0.006526538	0.651901202	0.180031704	0.160023697	0.0143413	-5.192017333	-2.180134518	-6.930290366	-6.117674535	-5.197869802	-3.646573233	4.837992736	0.024942982

CEP250	-0.100728594	0.00770376	-0.04974493	0.108432354	0.050983664	-0.057448691	8	0.113435959	0.816496581	0.816496581	0.816496581	0.816496581	0.816496581	0.816496581	28	133	0	P_240656	P_240656	P_240656	chr20	35479659	35479719	+	CEP250	centrosomal protein 250kDa [Source:HGNC Symbol;Acc:HGNC:1859]	ENSG00000126001	protein_coding	exon	coding exon	1	4723	20:35479659:35479719	ENSG00000126001	CEP250	7.831939696	0.012540162	-1.101658083	0.084255221	-0.544055091	1.185913303	0.557602991	-0.628310312	9.961963646	0.296514792	0.934522642	0.598368337	0.263171413	0.589424737	0.543935758	-6.373724341	-6.178346452	-6.887846589	-6.430471662	-6.133922806	-6.859905195	0.604805121	0.626750876
[truncated: 12,376,528 more chars]
